# (*E*)-Selective Weinreb Amide-Type Horner–Wadsworth–Emmons Reaction: Effect of Reaction Conditions, Substrate Scope, Isolation of a Reactive Magnesium Phosphonoenolate, and Applications

**DOI:** 10.1021/acs.joc.4c01140

**Published:** 2024-10-11

**Authors:** Takatsugu Murata, Hisazumi Tsutsui, Isamu Shiina

**Affiliations:** Department of Applied Chemistry, Faculty of Science, Tokyo University of Science, 1-3 Kagurazaka, Shinjuku-ku, Tokyo 162-8601, Japan

## Abstract

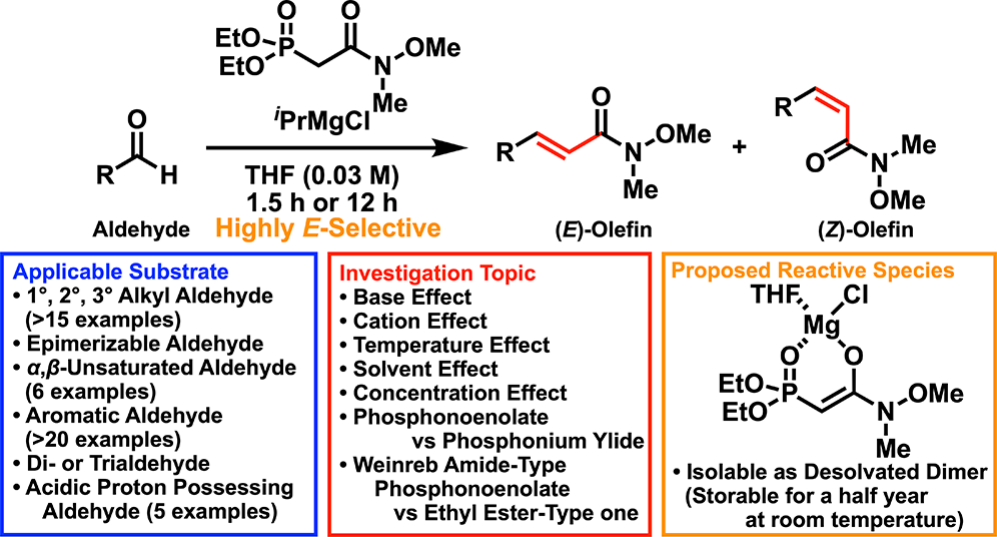

An ^*i*^PrMgCl-deprotonating Weinreb amide-type Horner–Wadsworth–Emmons (HWE) reaction was developed, and the effects of diverse reaction conditions, including the base, cation, solvent, and concentration, were investigated to broaden the substrate scope and achieve high (*E*)-selectivity. The Weinreb amide-type phosphonoenolate generated from ^*i*^PrMgCl was found to be isolable, stable for at least over a half year, and applicable in the HWE reaction keeping high productivity and selectivity compared with the in situ generated phosphonoenolate. The results prompted us to perform an application study including successive elongation, synthesis of a biscyclopropane, and Weinreb ketone syntheses.

## Introduction

**Scheme 1:**
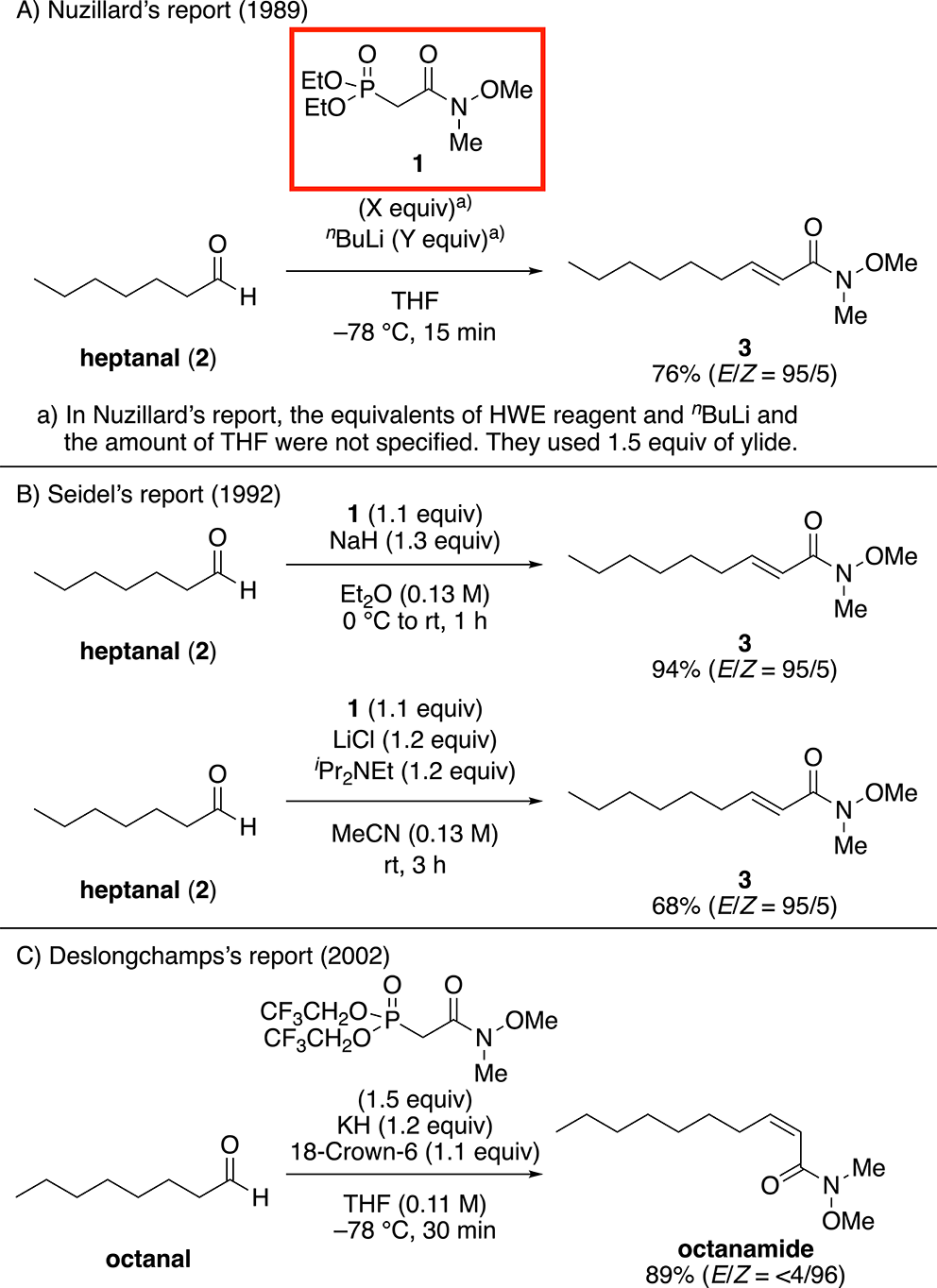
First Report of Weinreb Amide-Type HWE Reagent

The Horner–Wadsworth–Emmons (HWE) reaction is a ubiquitous reaction in organic synthesis. It was discovered by Horner and co-workers in 1958 as a modification of the Wittig reaction^1^ and further developed by Wadsworth, Emmons, and co-workers in 1961.^2^ Subsequently, many researchers have investigated various types of phosphonate-type elongation reagents,^3^ which have been called HWE reagents. The numerous reports on the HWE reaction in organic synthesis demonstrate its important role in this field.^4^ Therefore, the development of highly selective, robust, and scalable synthetic methods with broad substrate scope based on the HWE reaction constitutes an active area of research. Considering that Weinreb amides can be easily transformed not only to aldehydes by reduction, which prevents over-reduction to alcohol, but also to ketones by nucleophilic addition of alkyl lithium and Grignard reagents,^5^ α,β-unsaturated Weinreb amides are attractive building blocks and functional moieties in organic synthesis. A Weinreb amide-type HWE reagent was first reported by Nuzillard in 1989,^6^ and it was further developed by Seidel in 1992^7^ (Scheme 1). After 10 years of Seidel’s report, in 2002, Deslongchamps and co-workers reported the Still–Gennari-type (*Z*)-selective HWE reaction.^3al^

Nuzillard reported an (*E*)-selective HWE reaction using *n*-butyl lithium (^*n*^BuLi) as a phosphonoenolate-generating reagent (Scheme 1A). Meanwhile, Seidel reported two conditions for the (*E*)-selective HWE reaction (Scheme 1B), i.e., using sodium hydride (NaH) as a phosphonoenolate-generating reagent and the Masamune–Roush’s Lewis acid combination conditions. However, despite their efforts, broadening of the substrate scope is still required, and the reason for the selectivity reported by Nuzillard’s remains unclear. In 2008, Davies and co-workers reported that in the case of ethyl ester-type HWE reagents, methyl magnesium bromide (MeMgBr) was very effective for obtaining satisfactory (*E*)-selectivity and reactivity,^8,9^ whereas ^*n*^BuLi gives low (*E*)-selectivity with aliphatic aldehydes. Nevertheless, no report has addressed the effect of different reaction conditions on the HWE reaction using the same substrate. Therefore, with the aim of unveiling the question about the selectivity of the HWE reaction and developing a robust and highly selective Weinreb amide-type HWE reaction with broad substrate scope, we investigated the effect of the reaction conditions on the reactivity and selectivity of the Weinreb amide-type HWE reaction.

## Results and Discussion

To verify Nuzillard’s and Davies’ argument about the reaction selectivity, we first replicated Nuzillard’s reaction conditions, that is, ^*n*^BuLi as the phosphonoenolate-generating reagent, −78 °C as the reaction temperature, and a Weinreb amide-type HWE reagent as an elongation reagent, using heptanal and benzaldehyde as substrates, which were employed in Nuzillard’s and Seidel’s reports (Table 1).

**Table 1:**
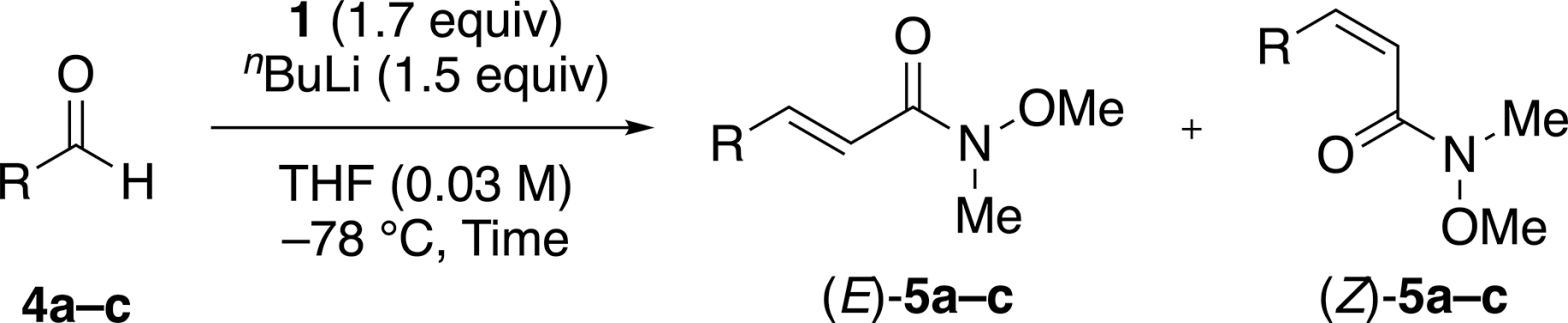

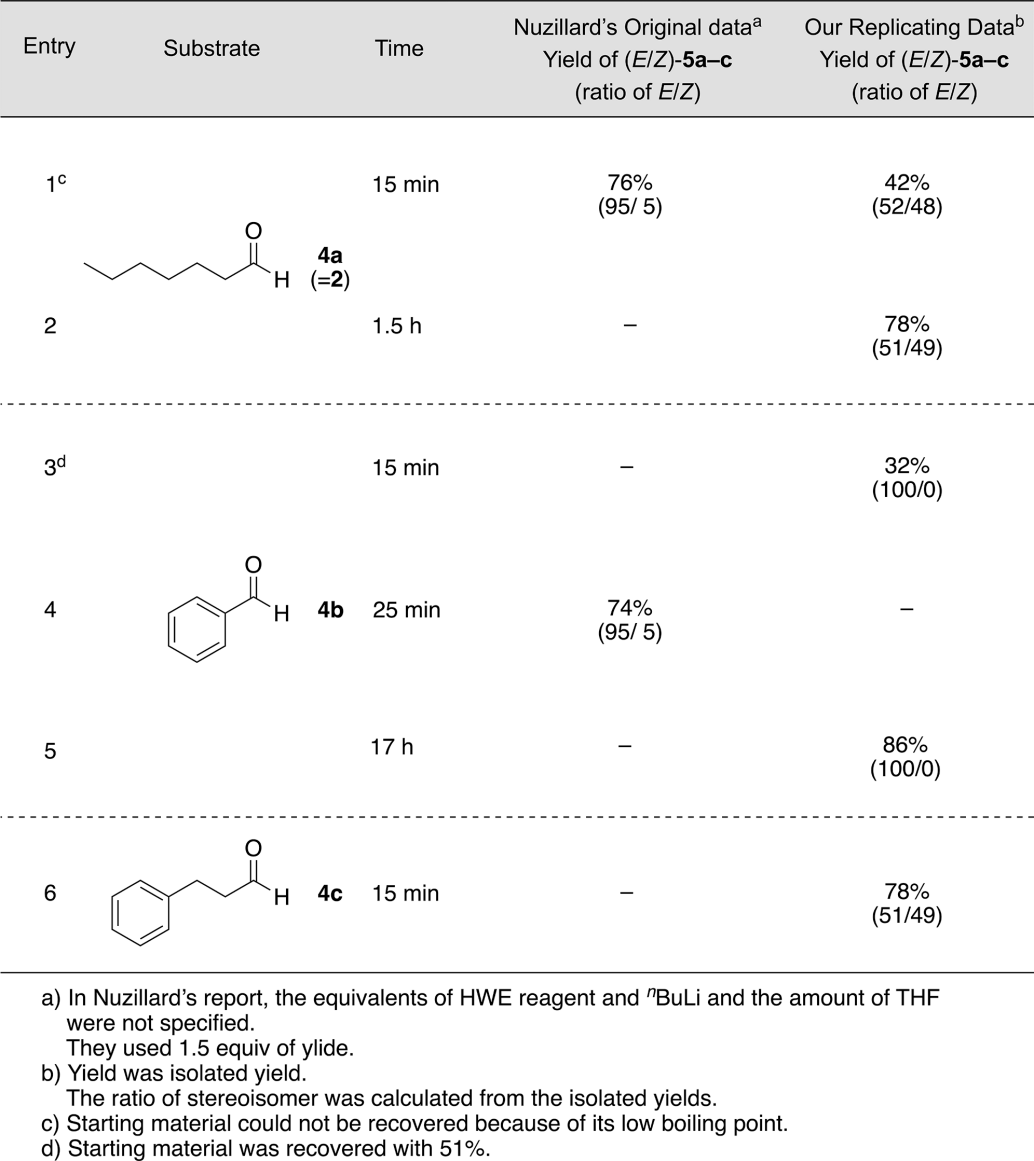
Comparison of Nuzillard’s Original Data with Our Results Employing Nuzillard’s Conditions

Our results revealed that Nuzillard’s conditions gave no selective reaction products in the case of heptanal (Table 1, entry 1), which is consistent with Davies’ report but not with Nuzillard’s original data. In this case, the reaction time did not affect the selectivity (Table 1, entry 2). To confirm this selectivity trend in the case of aliphatic aldehydes, we employed 3-phenypropanal as a typical aliphatic substrate commonly used in this research area. Again, no selective reaction products were obtained (Table 1, entry 6), confirming the selectivity trend described by Davies.^10^ Meanwhile, benzaldehyde as a representative aromatic aldehyde afforded (*E*)-**5b** with high (*E*)-selectivity under Nuzillard’s conditions (Table 1, entries 3 and 5), in consistency with Davies’ and Nuzillard’s reports. According to the results of the HWE reaction of aliphatic aldehydes, the development of an (*E*)-selective HWE reaction suitable for a wide range of aliphatic aldehydes was required. Thus, we tackled the optimization of the Weinreb amide-type HWE reaction conditions.

### Effect of Strong Base Conditions and Cation on the Yield and Selectivity of the Weinreb Amide-Type HWE Reaction

To further investigate the selectivity of the Weinreb amide-type HWE reaction, we evaluated various strong base conditions using 3-phenylpropanal as the substrate (Table 2).

**Table 2:**
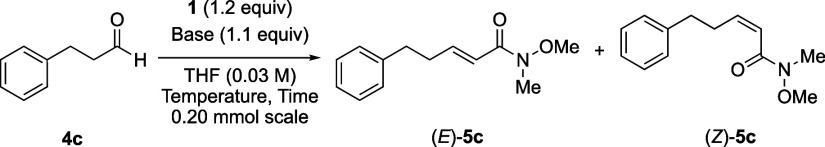
Effect of Strong Base Conditions, Cation, and Temperature on the Weinreb Amide-Type HWE Reaction

				Yield (%)		
Entry	Base	Temperature	Time	(*E*)-**5c**	(*Z*)-**5c**	*E*/*Z*
1	LHMDS	–78 °C	20 min	27	41	40/60
2	LHMDS	0 °C	↑	74	18	80/20
3	LHMDS	rt	↑	73	13	85/15
4	LHMDS	–78 °C to rt	↑	44	47	49/51
5	NaHMDS	0 °C	↑	85	3	97/3
6	NaHMDS	rt	↑	84	4	95/5
7	KHMDS	0 °C	↑	77	10	88/12
8	KHMDS	rt	↑	79	7	91/9
9	^*n*^BuLi	0 °C	↑	72	19	79/21
10	^*n*^BuLi	rt	↑	79	12	87/13
11	NaH	0 °C	30 min	89	3	97/3
12	NaH	rt	↑	90	2	97/3
13	^*t*^BuOK	0 °C	↑	85	10	89/11
14	^*t*^BuOK	rt	20 min	82	8	91/9
15	^*i*^PrMgBr	–78 °C	↑	4	0	100/0
16	^*i*^PrMgBr	0 °C	↑	27	trace	99/1
17	^*i*^PrMgBr	rt	↑	58	1	98/2
18	^*i*^PrMgBr	rt	4 h	73	1	99/1

As a preliminary investigation, we first employed lithium bis(hexamethyldisilazide) (LHMDS) as a strong base for deprotonation of the HWE reagent, which is a commonly used strong base for the HWE reaction.^11^ At –78 °C, the HWE reaction was (*Z*)-selective (Table 2, entry 1), whereas the (*E*)-selective product was favored with increasing reaction temperature (Table 2, entries 1–4). This effect of the reaction temperature is consistent with Heathcock’s pioneering report^9^ and Davies’ report.^8^

**Figure 1:**
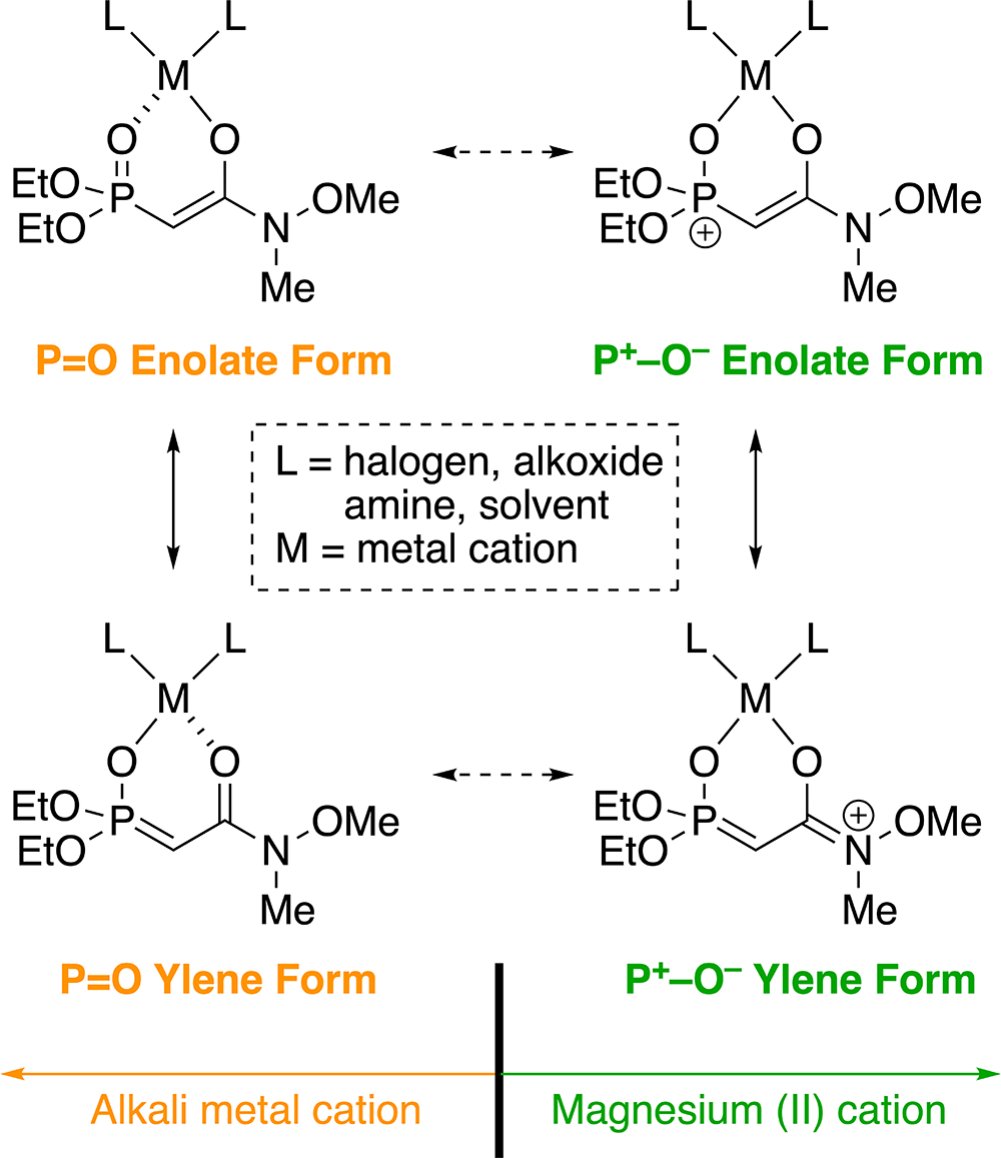
Resonance structures of metal cation–phosphonoenolate.

Next, to confirm the cation effect on the Weinreb amide-type HWE reaction, we examined HMDS bases with other alkali metal cations (Li vs Na vs K), among which the sodium cation was conducive to the (*E*)-selectivity (Table 2, entries 2 and 3 vs 5 and 6 vs 7 and 8). This cation effect was also observed with other conjugated bases, including ^*n*^BuLi, NaH, and potassium *tert*-butoxide (^*t*^BuOK) (Table 2, entries 9–14). As a divalent cation and a congener of sodium ion, we also evaluated magnesium(II) ion (Table 2, entries 15–18).^13a^ As the reaction temperature increased, the yield of the reaction product increased. However, at a difference with alkali metal cations, the selectivity observed in the reactions with magnesium cation was independent of the reaction temperature. Thus, at low temperature, the Weinreb amide-type HWE reaction using isopropylmagnesium bromide (^*i*^PrMgBr) as a base gave a low yield of the elongated product, which increased upon increasing the temperature (Table 2, entries 15–18), but the (*E*)-**5c** product was completely favored regardless of temperature. Therefore, the effect of conjugated base is much less effective on (*E*)-selectivity than that of the corresponding cation and that of the reaction temperature (Table 2, entries 3 and 10 vs 6 and 12 vs 8 and 14). The considerable effect of the magnesium(II) cation on the (*E*)-selectivity can be explained in terms of the resonance structures of the metal cation–phosphonoenolate complex generated in the presence of a strong base, as shown in Figure 1.

Four resonance forms can be drawn for the Weinreb amide-type phosphonoenolate, i.e., P=O enolate form, P^+^–O^–^ enolate form, P=O ylene form, and P^+^–O^–^ ylene form (Figure 1). P=O enolate form has been generally invoked to explain the generation of stable phosphonoenolates from phosphates possessing an electron-withdrawing group to eventually give (*E*)-olefins selectively. P=O enolate form and P=O ylene form would contribute to the stability of phosphonoenolates with monovalent alkali metal cations (M = Li, Na, and K). In contrast, P^+^–O^–^ enolate form and P^+^–O^–^ ylene form would contribute to the stability of the phosphonoenolate of the divalent magnesium cation. In general, the Coulomb attractive force is proportional to the product of two charges and inversely proportional to the square of the distance between two ions. Therefore, the magnesium(II) phosphonoenolate is more stable than the phosphonoenolates, which responded to the strength of electrostatic stabilization involving the six-membered ring. The origin of the (*E*)-selectivity of the HWE reaction is generally explained by a stable phosphonoenolate giving a stable intermediate to afford the corresponding (*E*)-olefin. Our proposal is consistent with this rationale and is supported by our experimental results, according to which the ^*i*^PrMgBr-deprotonating HWE reaction is slower than the LHMDS- and ^*n*^BuLi-deprotonating HWE reactions, whereas the ^*i*^PrMgBr-deprotonating HWE reaction is more (*E*)-selective than the LHMDS- and ^*n*^BuLi-deprotonating HWE reactions (Table 2, entries 1–3, and 9 and 10 vs 15–18).

### Effect of Weak Base Conditions and Cation on the Yield and Selectivity of the Weinreb Amide-Type HWE Reaction

Next, we used Seidel’s conditions to investigate the Weinreb amide-type HWE reaction under weak base conditions. In their pioneering work on a HWE reaction using a weak base, Masamune, Roush, and co-workers reported in 1984 that the combination of a lithium cation and a tertiary amine was effective for the (*E*)-selective HWE reaction and suitable for base-sensitive substrates.^12^ Subsequently, Rathke revealed that the magnesium ion–mediated Masamune–Roush conditions were also effective.^13^ Thus, we investigated the Weinreb amide-type HWE reaction using weak bases under Masamune–Roush’s conditions,^12^ Rathke’s conditions,^13^ and Seidel’s conditions^7^ (Table 3).

**Table 3:**
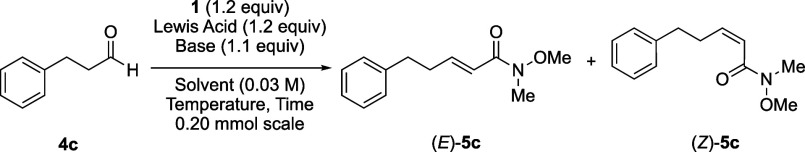
Weinreb Amide-Type HWE Reaction under Weak Base Conditions and Effect of Cation, Solvent, and Temperature

						Yield (%)		
Entry	Lewis Acid	Base	Solvent	Temperature	Time	(*E*)-**5c**	(*Z*)-**5c**	*E*/*Z*
1	LiCl	DBU	THF	rt	1.5 h	78	12	87/13
2^a^	↑	↑	MeCN	↑	↑	83	3	97/3
3	↑	NEt_3_	THF	↑	↑	9	1	93/7
4	↑	↑	MeCN	↑	↑	47	trace	>99/1
5	↑	^*i*^Pr_2_NEt	THF	↑	↑	3	trace	>99/1
6^a^	↑	↑	MeCN	↑	↑	28	trace	>99/1
7	↑	NEt_3_	THF	↑	12 h	47	9	83/17
8	↑	↑	MeCN	↑	↑	69	1	99/1
9	↑	^*i*^Pr_2_NEt	THF	↑	↑	12	1	>99/1
10	↑	↑	MeCN	↑	↑	66	trace	>99/1
11	MgBr_2_	DBU	THF	50 °C	1.5 h	38	2	95/5
12^b^	↑	NEt_3_	↑	↑	↑	28	1	96/4
13	↑	^*i*^Pr_2_NEt	↑	↑	↑	26	2	95/5

a Masamune–Roush conditions. See ref (12).

b Rathke’s conditions. See ref (13a).

Under the mentioned conditions and their modifications, 1,8-diazabicyclo[5.4.0]undec-7-ene (DBU), triethylamine (NEt_3_), and *N*,*N*-diisopropylethylamine (^*i*^Pr_2_NEt) are generally employed as the weak bases. Hence, we selected these three bases as the deprotonating source of the HWE reagent. In contrast to the case of strong base conditions, lithium cation was more effective for the (*E*)-selectivity (Table 3, entries 2, 4–6, and 8–10) than the magnesium cation (Table 3, entries 11–13). In addition, the yield of (*E*)-**5c** obtained under mild reaction conditions was lower than that obtained under strong base conditions. Under our conditions employing magnesium cation as the activating reagent (Table 3, entries 11–13), the (*E*)-selectivity was worse than under strong base conditions. These results might stem from the difference in the conjugated base anion. Deprotonation of the Weinreb amide-type HWE reagent with ^*i*^PrMgBr afforded the magnesium phosphonoenolate accompanied by propane and the newly generated vacant coordination space of the magnesium atom. This vacant coordination space would be simultaneously occupied by tetrahydrofuran (THF) solvent, which is a much weaker coordinating ligand than the bromide anion (Br^–^). Therefore, the difference in the coordination ligands around the magnesium(II) cation could contribute to the corresponding difference in (*E*)-selectivity between strong base conditions and weak base conditions.

### Phosphonoenolate Amount and Effect of Alkyl and Halogen Moieties in the Grignard Reagent

Next, we optimized the amount of ^*i*^PrMgBr and Weinreb amide-type HWE reagent to increase the yield of (*E*)-**5c** while maintaining high (*E*)-selectivity (Table 4). When treated with 1.5 equiv of phosphonoenolate, **4c** was almost consumed and the yield of (*E*)-**5c** was increased within 1.5 h. Further experiments revealed that the optimal amounts of Weinreb amide-type HWE reagent and ^*i*^PrMgBr were 2.0 and 1.8 equiv, respectively.

**Table 4:**
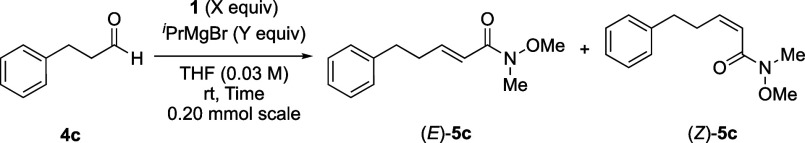
Screening of the Amount of Weinreb Amide-Type HWE Reagent and ^*i*^PrMgBr

				Yield (%)		
Entry	X (equiv)	Y (equiv)	Time (h)	(*E*)-**5c**	(*Z*)-**5c**	rSM (%)
1	1.2	1.1	4	73	1	8
2	1.6	1.5	1.5	79	2	trace
3	2.0	1.8	↑	84	3	0
4	2.4	2.2	↑	76	trace	0

To further improve the reaction outcome, we examined the effect of alkyl and halogen moieties in the Grignard reagent on the yield and (*E*)-selectivity (Table 5). Methylmagnesium bromide, which was employed by Davies,^8^ gave moderate yield and high (*E*)-selectivity (Table 5, entry 1). The alkyl moiety in alkyl magnesium bromides did not exert a remarkable effect on the reaction yield and (*E*)-selectivity (Table 5, entries 2, 6, 8, and 10). Conversely, the halogen anion (Cl^–^, Br^–^, and I^–^) had a considerable effect (Table 5, entries 2–4 and 10 and 11). These results suggest that the halogen ligand on the Grignard reagent was transferred to the corresponding phosphonoenolate via deprotonation with the coordinated magnesium cation, resulting in a Mg(II)–X (X = Cl, Br, and I) structure as the active species for the Weinreb amide-type HWE reaction.

**Table 5:**
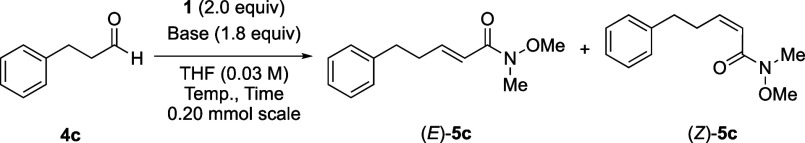
Effect of Alkyl and Halogen Moieties on the Grignard Reagent on the Yield and (*E*)-Selectivity

				Yield (%)		
Entry	Base	Temperature	Time (h)	(*E*)-**5c**	(*Z*)-**5c**	*E*/*Z*
1	MeMgBr	0 °C	5	70	1	99/1
2	MeMgBr	rt	1.5	82	2	98/2
3	MeMgCl	rt	1.5	89	2	98/2
4	MeMgI	rt	1.5	29	trace	>99/1
5	EtMgBr	0 °C	5	63	trace	97/3
6	EtMgBr	rt	1.5	83	1	99/1
7	PhMgBr	0 °C	3.5	64	trace	>99/1
8	PhMgBr	rt	1.5	82	2	98/2
9	^*i*^PrMgBr	0 °C	5	63	1	98/2
10	^*i*^PrMgBr	rt	1.5	84	3	96/4
11	^*i*^PrMgCl	rt	1.5	94	1	99/1
12	^*i*^PrMgCl·LiCl	rt	1.5	90	2	98/2

### Effect of Type and Amount of Solvent on the Yield and Selectivity of the Weinreb Amide-Type HWE Reaction

To investigate the effect of the type and amount of solvent on the Weinreb amide-type HWE reaction, we employed some representative solvents and three concentration conditions using ^*i*^PrMgCl as the deprotonating reagent (Table 6).

**Table 6:**
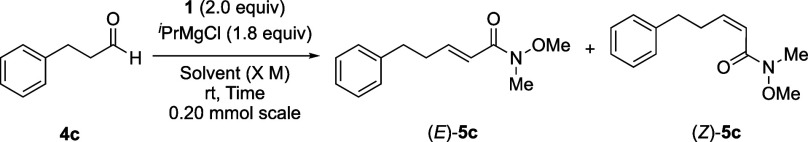
Effect of Type and Amount of Solvent

				Yield (%)		
Entry	Solvent	X (M)	Time (h)	(*E*)-**5c**	(*Z*)-**5c**	*E*/*Z*
1	THF	0.03	1.5	94	1	99/1
2	DME	↑	↑	78	trace	>99/1
3	Et_2_O	↑	↑	23	0	100/0
4	MeCN	↑	↑	15	trace	>99/1
5	toluene	↑	↑	17	3	85/15
6^a^	THF	0.03	1.5	90	2	98/2
7^a^	↑	0.1	2.0	85	1	99/1
8^a^	↑	0.3	5.0	87	1	98/2

a 1.0 mmol of **4c** was used.

**Figure 2:**
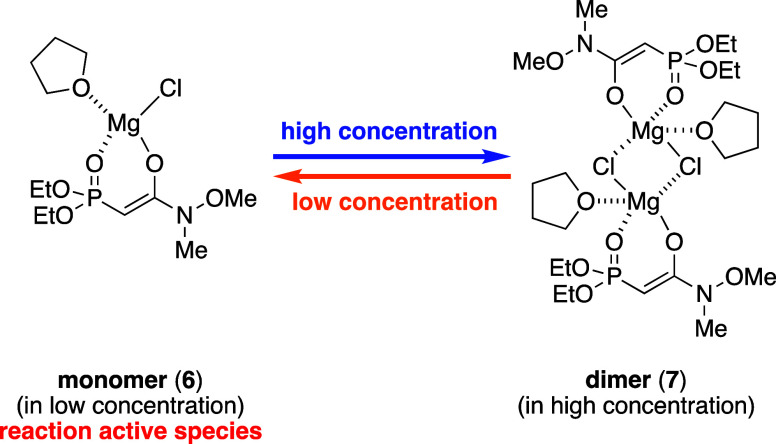
Proposed structure of the active species and its dimer.

Among the three etheric solvents evaluated, i.e., THF, 1,2-dimethoxyethane (DME), and diethyl ether (Et_2_O), THF was the best solvent (Table 6, entries 1–3). Conversely, acetonitrile, which is also an aprotic solvent, gave a low yield (Table 6, entry 4). The low-polar solvent toluene, which is frequently employed in the Wittig reaction, also afforded the product in low yield and with moderate selectivity (Table 6, entry 5). This solvent effect suggests that the reactive species contains solvent molecules. Furthermore, the reaction required more time as the concentration was increased. These solvent and concentration effects indicate that the active species could form a dimer containing solvent molecules under high concentration conditions, as shown in Figure 2.

To the best of our knowledge, no report has described the isolation of a reactive phosphonoenolate species in the HWE reaction. As an example of indirect detection via the phosphorus-31 nuclear magnetic resonance (^31^P NMR) and infrared (IR) spectroscopy, Seyden-Penne and co-workers revealed in 1987 that a lithium phosphonoenolate forms a chelated structure and a dimer in solution under high concentrations conditions.^14^ Accordingly, the reaction concentration could affect the reaction rate owing to the equilibrium of the phosphonoenolate. Our experimental results support this assumption, which is also in accord with our proposed structure for the active species. According to our results, we selected the following as the optimal reaction conditions: 2.0 equiv of Weinreb amide-type HWE reagent and 1.8 equiv of ^*i*^PrMgCl as elongating reagents, 0.03 M of THF as the reaction solvent, and room temperature as the reaction temperature.

### Isolation of a Reactive Magnesium Phosphonoenolate and HWE Reaction of Isolated Magnesium Weinreb Amide-Type Phosphonoenolate

**Scheme 2:**
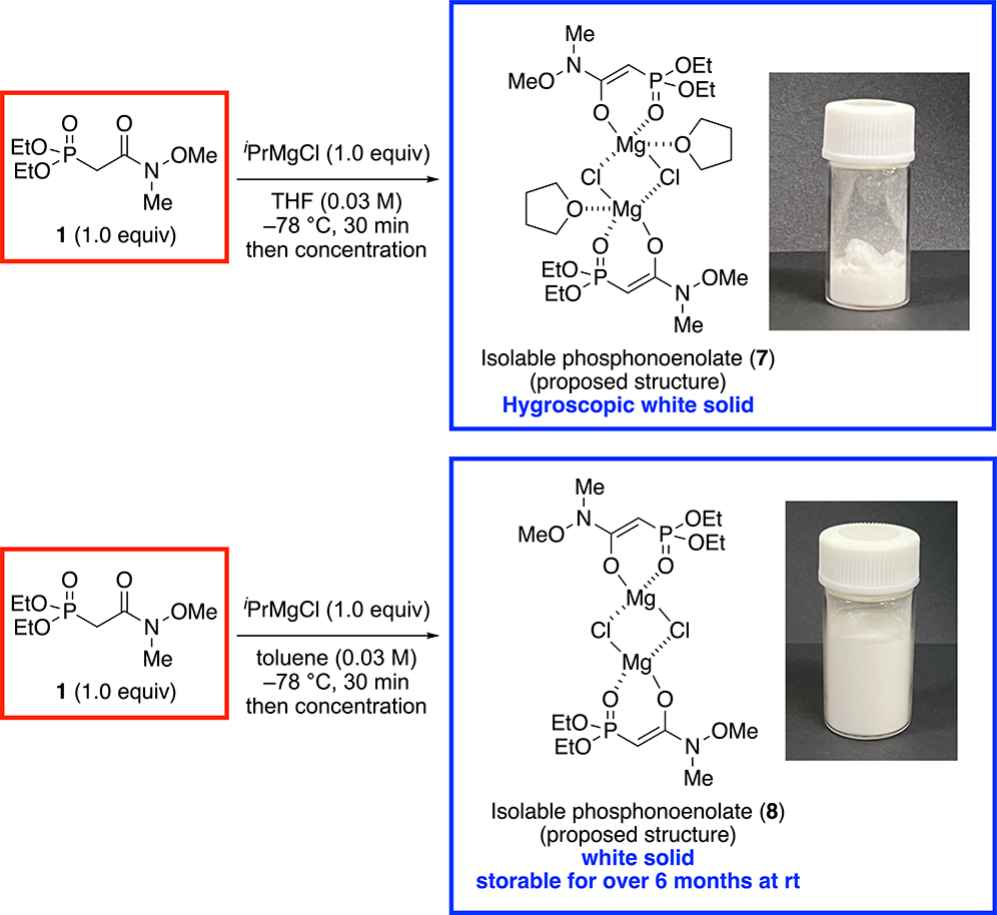
Isolation of Magnesium Phosphonoenolates

According to our results suggesting the high stability of the magnesium phosphonoenolate, we considered that it could be isolated. In fact, the magnesium phosphonoenolate was obtained as a white solid by concentrating a solution of the phosphonoenolate generated using ^*i*^PrMgCl under argon atmosphere (Scheme 2). The absence of sharp signals in the proton (^1^H) NMR spectrum was indicative of the paramagnetic character of the isolated phosphonoenolate or that in the solution. Nevertheless, the presence of THF molecules in the phosphonoenolate solid could be ascertained. The phosphonoenolate could be also isolated from a toluene solution. In this case, the ^1^H NMR spectrum did not reveal the presence of toluene molecules in the phosphonoenolate structure. To get further information on the isolated phosphonoenolate, which the crystallization of both complexes is currently underway, we measured the IR spectrum of the isolated phosphonoenolates. The vibration frequency of the carbonyl group in **1** was 1666 cm^–1^, whereas the corresponding frequencies of isolated phosphonoenolates **7** and **8** were 1636 cm^–1^. On the other hand, the frequency of P=O vibration of **1** was 1257 cm^–1^ shifted to 1248 and 1249 cm^–1^ in **7** and **8** respectively, by the formation of phosphonoenolates. These decreasing shifts showed that the formation of phosphonoenolates weakened P=O bond,^15^ and it supports the proposed structure of the isolated phosphonoenolates and the stabilization of phosphonoenolate.

**Scheme 3:**
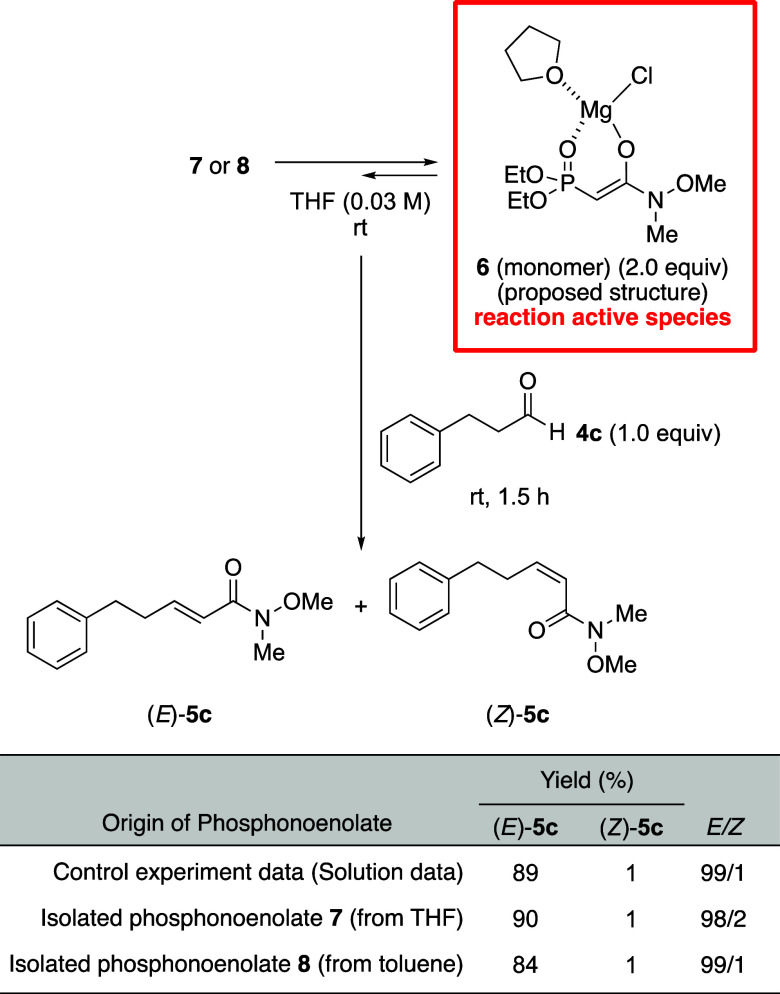
HWE Reaction of the Isolated Magnesium Phosphonoenolate

Using the isolated magnesium phosphonoenolates, we conducted the HWE reaction of 3-phenylpropanal, which afforded the corresponding (*E*)-olefin selectively without significant differences in the yield and selectivity compared with a control experiment (Scheme 3). The desolvated phosphonoenolate **8** isolated from toluene solution was more stable than the solvated phosphonoenolate **7** isolated from THF solution, since **7** became damp under touch with a spatula. In contrast, phosphonoenolate **8** could be stored at least for over a half year at room temperature in argon atmosphere and then used for the HWE reaction without any change in the yield and selectivity. To the best of our knowledge, this is the first report on the isolation of the phosphonoenolate intermediate and the application of the isolated phosphonoenolate to induce the HWE reaction. Similarly, the lithium phosphonoenolate could be isolated and used for the HWE reaction.^16^ In addition, not only the Weinreb amide-type phosphonoenolates but also ethyl ester-type phosphonoenolates were isolable and reactive.^17^ Therefore, this method for the isolation of phosphonoenolates and their application in the HWE reaction could be extended to various HWE reagents and applied to other systems such as solid-phase HWE reactions. Practically, we can use the solid phosphonoenolate as the additional amount of reagent at the time that the reaction is just proceeding, without increasing the volume of solvent.

### Effect of the Phosphonate Ester Group in the Weinreb Amide-Type HWE Reagent

Next, we investigated the effect of the structure of the HWE reagent on the yield and selectivity of the Weinreb Amide-type HWE reaction (Table 7).

**Table 7:**
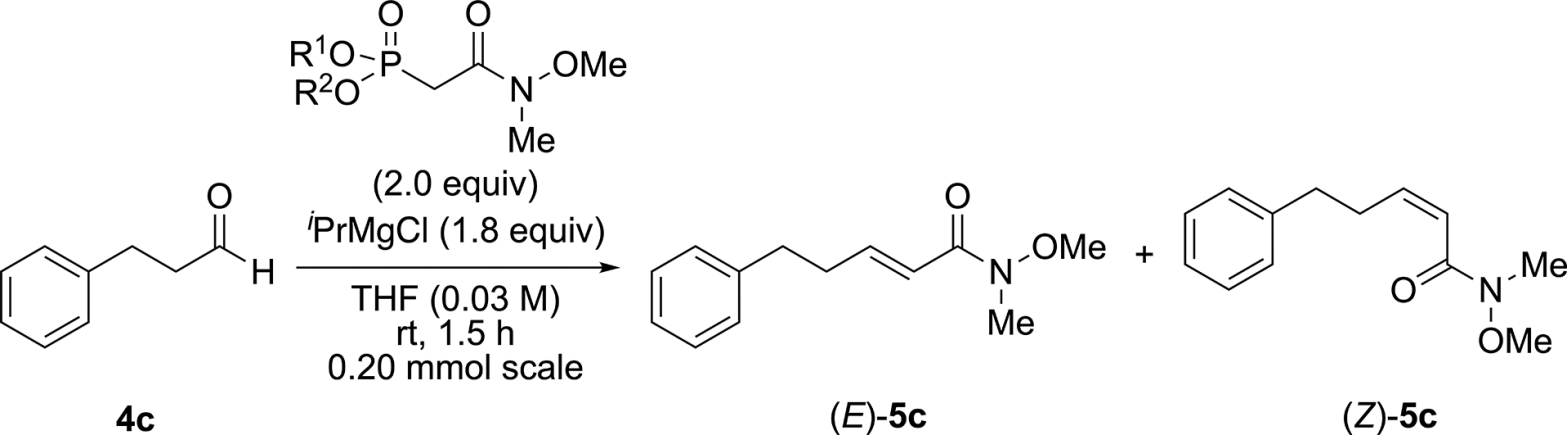
Effect of the Phosphonate Ester Group on the Selectivity of the Weinreb Amide-Type HWE Reaction

			Yield (%)		
Entry	R^1^O	R^2^O	(*E*)-**5c**	(*Z*)-**5c**	*E*/*Z*
1	MeO	MeO	91	3	97/3
2	EtO	EtO	94	1	99/1
3	^*i*^PrO	^*i*^PrO	41	1	98/2
4	PhO	PhO	46	45	50/50
5	CF_3_CH_2_O	EtO	83	5	95/5
6	CF_3_CH_2_O	CF_3_CH_2_O	17	70	19/81

Although dimethyl phosphonate has similar reactivity to diethyl phosphonate, the selectivity induced by the former was lower than that by the latter (Table 7, entry 1 vs 2). Diisopropyl phosphonate gave lower yield than diethyl phosphonate (Table 7, entry 2 vs 3). This can be attributed to the bulkiness of the phosphonate group increasing the activation energy. We also employed electron-withdrawing alkyl phosphonates. Representative (*Z*)-selective HWE reagents such as the Still–Gennari reagent^3w^ and the Ando reagent^3ag^ have a bis(2,2,2-trifluoroethyl) phosphonate ester moiety and diphenyl phosphonate ester moiety, respectively. It has long been known that the structure of the phosphonate ester moiety affects the selectivity of the HWE reaction; however, the effect of subjecting (*Z*)-selective HWE reagents to highly (*E*)-selective conditions is unknown. The Weinreb amide-type Ando reagent^3aj^ gave no selective reaction product under our optimized conditions (Table 7, entry 4), and the Weinreb amide-type Still–Gennari reagent^3al^ afforded selectively the (*Z*)-olefin. These results suggest that the Ando reagent is more affected by the cation effect than the Still–Gennari reagent. When ethyl (2,2,2-trifluoroethyl) phosphonate was subjected to the optimized reaction conditions, the (*E*)-olefin was selectively obtained, indicating that a (*Z*)-selective HWE reagent should possess two strong electron-withdrawing ester moieties. Consequently, the diethyl phosphonate moiety resulted in the most suitable Weinreb amide-type to obtain (*E*)-olefins under our optimized conditions.

### Comparison among Wittig Reagents and HWE Reagents and among Ethyl Ester-Type Reagents and Weinreb Amide-Type Reagents

To compare Wittig reagents with HWE reagents and ethyl ester-type reagents with Weinreb amide-type reagents, we performed comparison experiments using each elongation reagent. Under the optimized conditions, the yield of the Weinreb amide-type HWE reaction was higher than that of the ethyl ester-type HWE reaction, and the selectivity was almost the same (Table 8, entry 1 vs 4). The difference in the yield might stem from the effect of the amide moiety, and the fact that the same selectivity was observed might be attributed to the effect of the magnesium cation and temperature. In the case of Weinreb amide-type reagents, the phosphonoenolate generated from the HWE reagent gave (*E*)-**5c** in higher yield than the Wittig reagent and the phosphonium salt (Table 8, entry 1 vs 2 and 3) because the Wittig reagent is more stable and less reactive than the phosphonoenolate generated from the HWE reagent. In general, the (*E*)-selectivity of the HWE reaction was higher than that of the Wittig reaction using Weinreb amide-type and ethyl ester-type reagents (Table 8, entries 1 vs 2 and 3 and 4 vs 5 and 6).

**Table 8:**
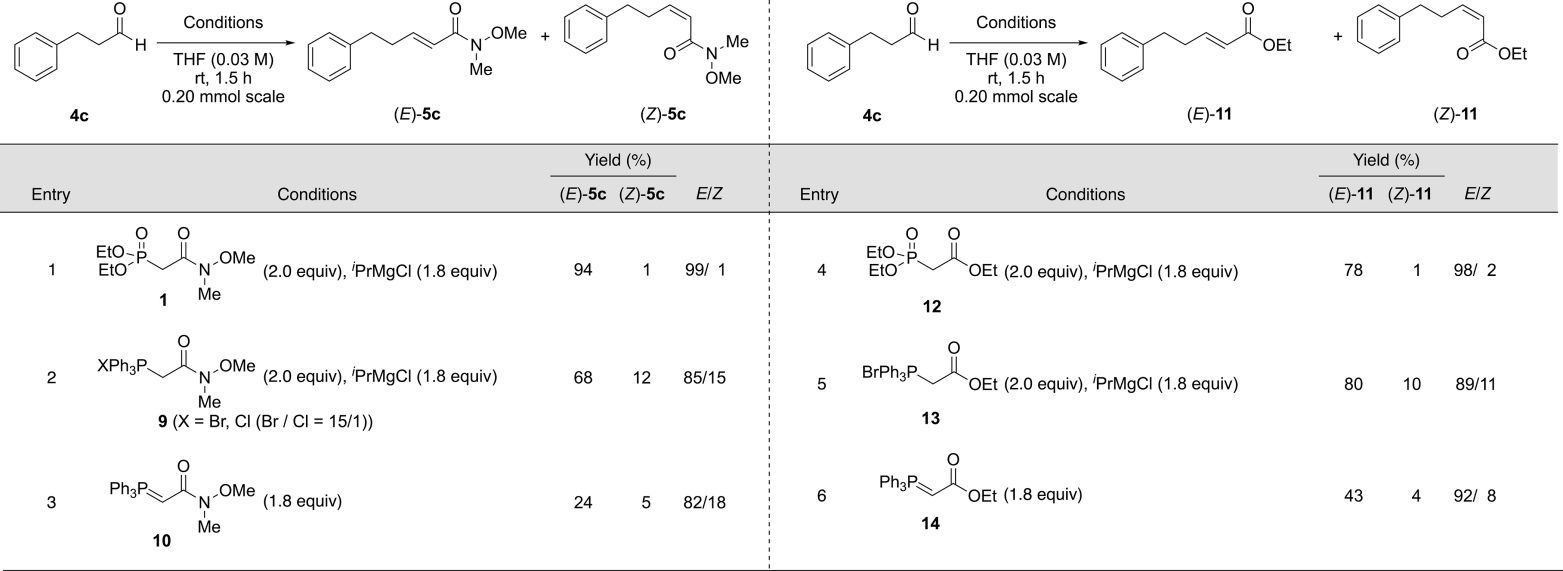
Comparison among Wittig Reagents and HWE Reagents and among Ethyl Ester-Type Reagents and Weinreb Amide-Type Reagents

### Substrate Scope of Saturated Aliphatic Aldehydes for the ^*i*^PrMgCl-Deprotonating Weinreb Amide-Type HWE Reaction

Using our optimized conditions, we investigated that the substrate scope of saturated aliphatic aldehydes for the ^*i*^PrMgCl-deprotonating Weinreb amide-type HWE reaction (Table 9).

**Table 9:**
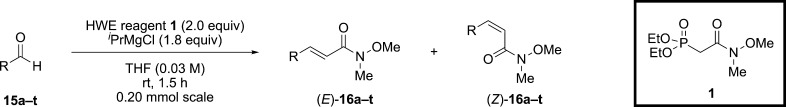

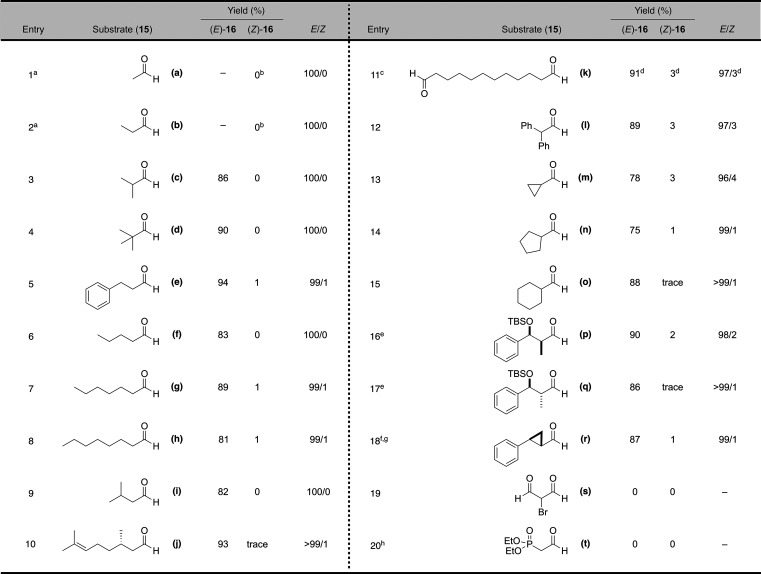
Substrate Scope of Saturated Aliphatic Aldehydes in the Weinreb Amide-Type HWE Reaction

a Reaction (*E*)-products were highly volatile so that we could not get the accurate yield. Only (*E*)-selectivity was calculated from ^1^H NMR spectra of crude product.

b (*Z*)-product was not detected in TLC monitoring. See also note a.

c HWE reagent (3.0 equiv), ^*i*^PrMgCl (2.7 equiv) were used and stirred for 12 h.

d Yield of (*E*,*E*)-**16k**. (*E*,*Z*)-**16k** was also given in 3% yield. The ratio of *E*/*Z* is the ratio of (*E*,*E*)-**16k**/(*E*,*Z*)-**16k**. (*Z*,*Z*)-**16k** was not detected.

e Reaction time was 20 min.

f Reaction time was 12 h. In case that reaction time is 1.5 h, (*E*)-**16r** was 46% yield.

g Purity: 91%.

h Purity: 92%.

α-Primary aldehydes including middle-chain aliphatic aldehydes, β-branched aldehydes, a chiral aldehyde, and a dialdehyde gave (*E*)-olefins selectively (Table 9, entries 1, 2, and 6–11). Particularly, the dialdehyde enabled the bidirectional elongation to give the double-elongated (*E*,*E*)-olefin selectively. In this case, (*Z*,*Z*)-olefin was not detected and the result indicated that each elongation was highly (*E*)-selective. Not only α-primary aldehydes but also α-secondary and α-tertiary aldehydes gave the (*E*)-Weinreb amides in excellent yield with complete (*E*)-selectivity (Table 9, entries 3 and 4). Generally, in the case of aldehydes possessing an α-acidic proton, epimerization of the aldehydes or the elongation products and regioisomerization of products could occur. However, regioisomerization of the reaction product was not detected under the optimized conditions (Table 9, entry 12). Even employing α-epimerizable aldehydes, the corresponding (*E*)-olefins were obtained in good yield with good selectivity without epimerization (Table 9, entries 16 and 17). Other α-secondary aldehydes such as α-cycloalkyl aldehydes were also applicable, affording good yield and high selectivity without regioisomerization (Table 9, entries 13–15). Meanwhile, an α-carbocycle-possessing aldehyde also afforded the corresponding (*E*)-olefin in good yield with high selectivity without undergoing ring opening (Table 9, entry 18). Conversely, aldehydes containing a highly acidic proton did not give any elongation product (Table 9, entries 19 and 20), which was the only limitation in the substrate scope of this HWE reaction.

### Substrate Scope of α,β-Unsaturated Aliphatic Aldehydes for the ^*i*^PrMgCl-Deprotonating Weinreb Amide-Type HWE Reaction

Next, we expanded the substrate scope of the Weinreb amide-type HWE reaction to representative unsaturated aliphatic aldehydes (Table 10). Our optimized conditions were applicable to α,β-unsaturated aliphatic aldehydes, although longer reaction times (12 h) were required owing to their lower reactivity compared with saturated aliphatic aldehydes. In addition, an α-methyl substituent slowed down the reaction (Table 10, entries 1 vs 2), whereas an electron-withdrawing α-bromo substituent did not (Table 10, entry 5).^18^ Furthermore, not only α,β-unsaturated aliphatic aldehydes but also a propargyl aldehyde was applicable to this reaction (Table 10, entry 3). An α,β,γ,δ-unsaturated aliphatic aldehyde gave the elongated α,β,γ,δ,ε,ζ-unsaturated aliphatic olefin in good yield with high selectivity. This elongation result prompted us to evaluate the successive elongation process using the ^*i*^PrMgCl-deprotonating Weinreb amide-type HWE reaction.

**Table 10:**
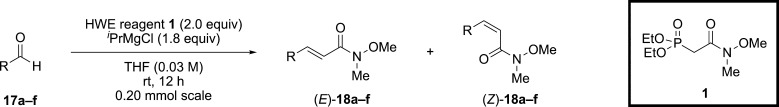

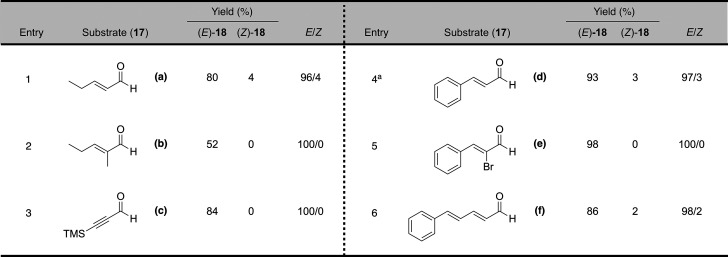
Substrate Scope of Unsaturated Aliphatic Aldehydes in the ^*i*^PrMgCl-Deprotonating Weinreb Amide-Type HWE Reaction

a THF (0.2 M), 21 h, 20 mmol scale.

### Substrate Scope of Aromatic Aldehydes for the ^*i*^PrMgCl-Deprotonating Weinreb Amide-Type HWE Reaction

We also expanded the substrate scope of the Weinreb amide-type HWE reaction to various aromatic aldehydes (Table 11). Similar to α,β-unsaturated aliphatic aldehydes, aromatic aldehydes needed longer reaction times than saturated aliphatic aldehydes. In the case of *ortho*-, *meta*-, and *para*-tolualdehydes, no substituent effect was observed, and their reactions gave the corresponding (*E*)-olefins in good yield (Table 11, entries 2–4). To examine the steric effect of substituents on the aromatic ring, 2,6-dimethylbenzaldehyde, which could suppress the rotation of the aromatic ring because of the two directional substituents, was subjected to the reaction conditions to afford the elongation product with a good yield and complete (*E*)-selectivity, albeit with higher reaction temperature and longer reaction time (Table 11, entry 5). Electron-withdrawing and electron-donating groups did not affect the reaction yield and selectivity (Table 11, entries 6, 7, and 9–13). The aldehyde possessing an acetyl group also gave the (*E*)-olefin stereoselectively while the acetyl group remained intact (Table 11, entry 14), indicating that the acyclic ketone moiety does not need protection under these reaction conditions. Aldehydes containing an acidic proton, i.e., those with a carboxyl group, a phenolic hydroxy group, a boronic acid group, an NH-free pyrrolyl group, and an NH-free indolyl group, required high temperature but gave the (*E*)-olefin in good yields and with complete (*E*)-selectivity in most cases (entries 15–17, 24, and 26). In addition, heterocyclic aldehydes such as pyridinyl, furyl, thiophenyl, and pyrrolyl aldehydes were applicable to this HWE reaction. However, only 2-pyridynecarbardehyde gave the corresponding olefin in low yield, which could be due to the directing pyridinyl nitrogen being closer to the reaction site than that of 3-pyridynecarbaldehyde and 4-pyridinecarbardehyde. Meanwhile, polycyclic aromatic aldehydes were also suitable substrates (Table 11, entries 27 and 28). 9-Anthracenecarbaldehyde needed higher reaction temperature and longer reaction time due to the steric hindrance of the two bidirectionally extended rings (Table 11, entry 28). As multireactive-site substrates, aromatic dialdehydes including *ortho*-, *meta*-, and *para*-phtalaldehydes were also applicable without producing the double-elongated (*Z*,*Z*)-olefins and (*E*,*Z*)-olefins (Table 11, entries 29–31). The monoelongated intermediates, in which only one of two formyl groups reacted and the other remained intact, also gave the (*E*)-olefins in moderate yields. Furthermore, a trialdehyde was also subjected to the reaction conditions to afford the triple-elongated product in a moderate yield. Even though the probability of obtaining an (*E*,*E*,*Z*)-trielongated olefin is three times higher than that of producing a (*Z*)-olefin from a monoaldehyde such as benzaldehyde, the trielongated compounds were only obtained as (*E*,*E*,*E*)-olefins. This result confirmed that our reaction conditions provided very high (*E*)-selectivity in the case of aromatic aldehydes.

**Table 11:**
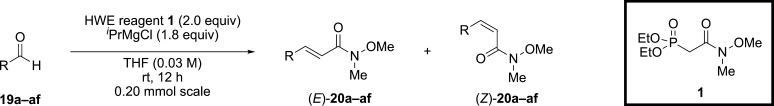

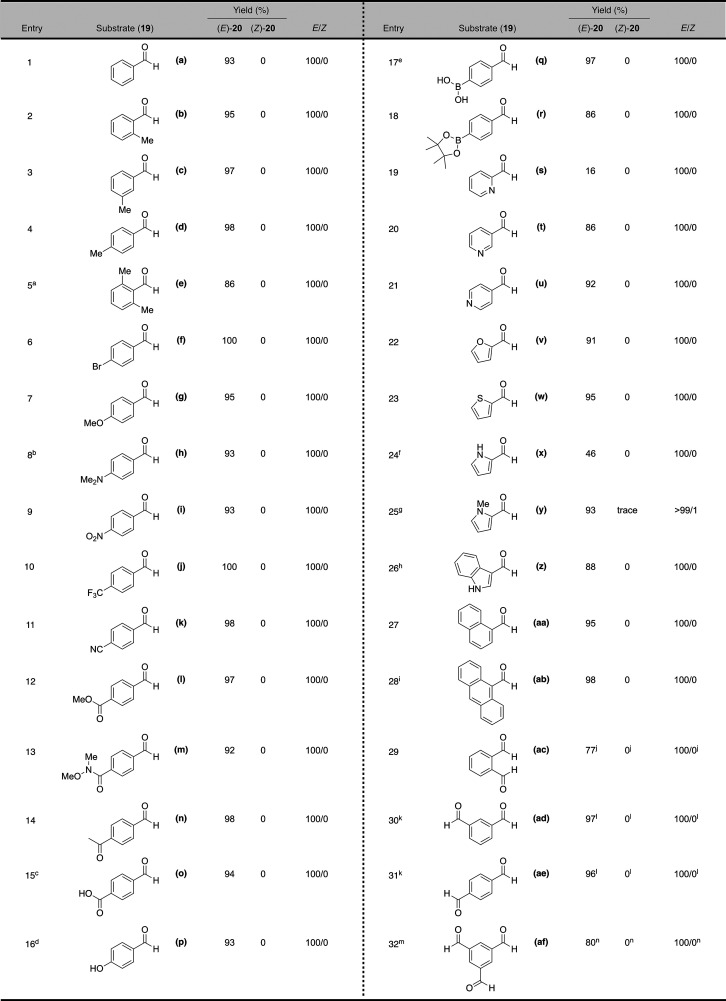
Substrate Scope of Aromatic Aldehydes in the ^*i*^PrMgCl-Deprotonating Weinreb Amide-Type HWE Reaction

a Reflux, 6 h.

b Reflux, 6 h.

c **1** (3.0 equiv), ^*i*^PrMgCl (2.7 equiv), reflux, 6 h.

d **1** (3.0 equiv), ^*i*^PrMgCl (2.7 equiv), reflux, 24 h.

e **1** (3.0 equiv), ^*i*^PrMgCl (2.7 equiv), reflux, 4 h.

f **1** (3.0 equiv), ^*i*^PrMgCl (2.7 equiv), reflux, 24 h.

g Reflux, 6 h.

h **1** (3.0 equiv), ^*i*^PrMgCl (2.7 equiv), reflux, 24 h.

i Reflux, 6 h.

j Yield of (*E*,*E*)-**20ac**. (*E*)-**20ac** that one of two formyl groups was reacted also given in a 23% yield. (*Z*)-**20** is (*Z*,*Z*)-**20ac** and (*E*,*Z*)-**20ac**, and they were not detected. The ratio of *E*/*Z* is the ratio of (*E*,*E*)-**20ac**/other geometric isomers.

k **1** (4.0 equiv), ^*i*^PrMgCl (3.6 equiv), reflux, 6 h.

l Yield of (*E*,*E*)-**20ad**, **ae** and (*Z*,*Z*)-**20ad**, **ae**. The ratio of *E*/*Z* is the ratio of (*E*,*E*)-**20ad**, **ae**/(*E*,*Z*)-**20ad**, **ae**. Other geometric isomers and unilateral elongation products were not detected.

m **1** (6.0 equiv), ^*i*^PrMgCl (5.4 equiv), reflux 4 h.

n Yield of (*E*,*E*,*E*)-**20af**. The ratio of *E*/*Z* is the ratio of (*E*,*E*,*E*)-**20af**/other geometric isomers.

Other geometric isomers, unilateral elongation products, and other intermediates were not detected.

### Application of the ^*i*^PrMgCl-Deprotonating Weinreb Amide-Type HWE Reaction to Successive Elongation

**Scheme 4:**
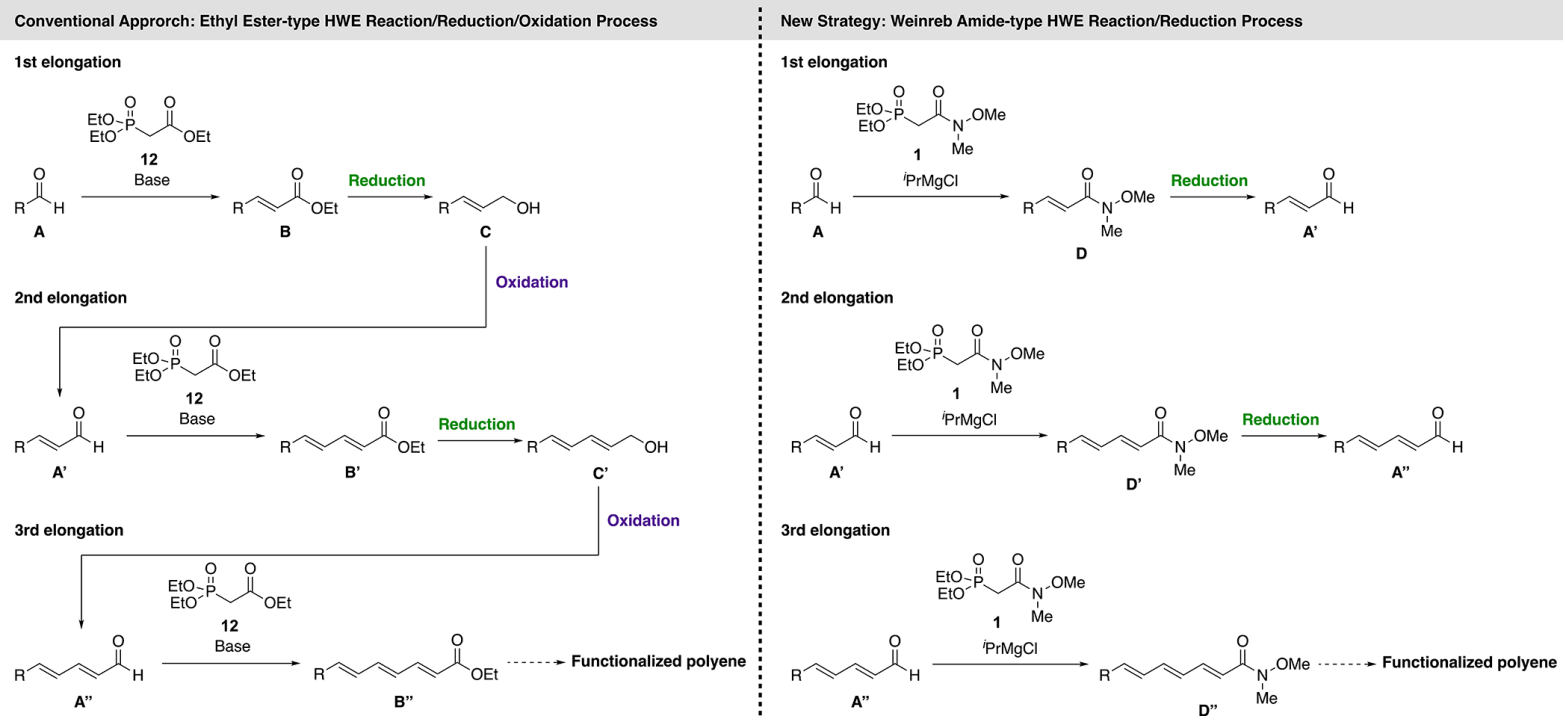
Comparison of the Successive Elongation Strategy between Ethyl Ester-Type HWE Reagent and Weinreb Amide-Type HWE Reagent

The polyene structure is present in many natural products,^19^ pigments,^20^ and bioimaging and biosensing probes;^21^ therefore, the development of a synthetic methodology to polyene compounds is highly desired. Typically, ester-type HWE reagents are used for successive elongation (Scheme 4). The produced ester (**B**) requires reduction to the corresponding aldehyde for the subsequent elongation. However, the ester group is sometimes excessively reduced to the corresponding hydroxy group (from **B** to **C**). Therefore, successive elongation has been commonly performed using a redox process involving the HWE reaction, reduction to alcohol, and oxidation to aldehyde.^22^ To improve the synthetic efficiency, we addressed the successive elongation by taking advantage of the selective reduction of the Weinreb amide (**D**) to the corresponding aldehyde (**A**′) and the following our HWE reaction (from **A**′ to **D**′), which would eliminate a step in each elongation process.

**Scheme 5:**
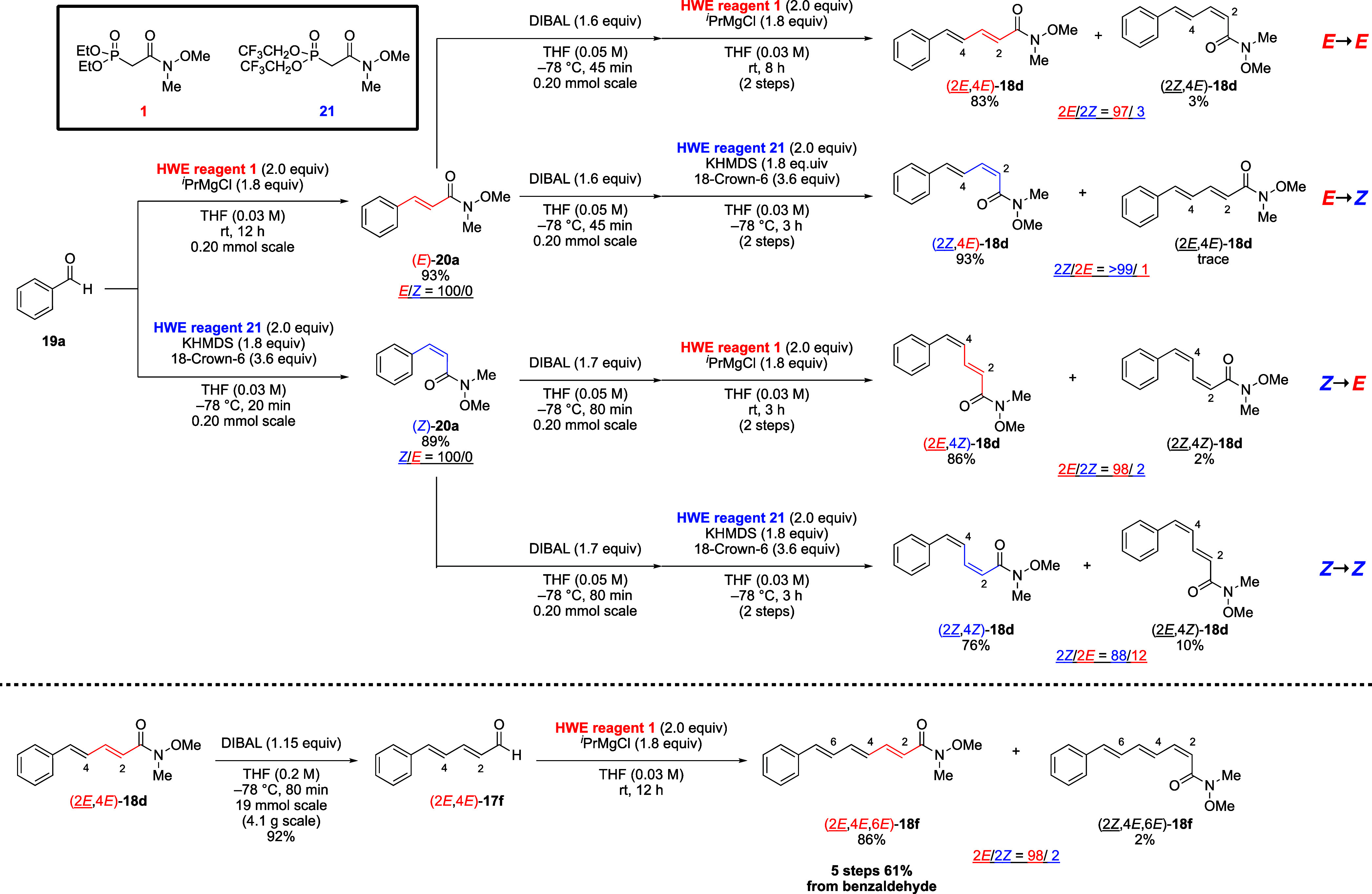
Selective Synthesis of (2*E*,4*E*)-, (2*Z*,4*E*)-, (2*E*,4*Z*)-, (2*Z*,4*Z*)-Olefins and (2*E*,4*E*,6*E*)-Olefin via Successive Elongation

The successive elongation of benzaldehyde was performed (Scheme 5). In this process, the Still–Gennari reagent was employed as a (*Z*)-selective Weinreb amide-type HWE reagent. The first processes were completely (*E*)- and (*Z*)-selective and afforded good yield. The second processes were highly selective and also gave the olefins in good yields. The minor stereoisomers were easily separable via silica gel chromatography by virtue of the structure of the α,β-unsaturated Weinreb amide. In this two processes, all stereoisomers of α,β,γ,δ-unsaturated Weinreb amide were synthesized selectively in high yields. As a representative substrate, (2*E*,4*E*)-**18d** was subjected to a third process to afford (2*E*,4*E*,6*E*)-**18f**. In successive elongation processes, all-trans-triene was obtained after five steps in 61% yield, enabling further elongation.

**Scheme 6:**
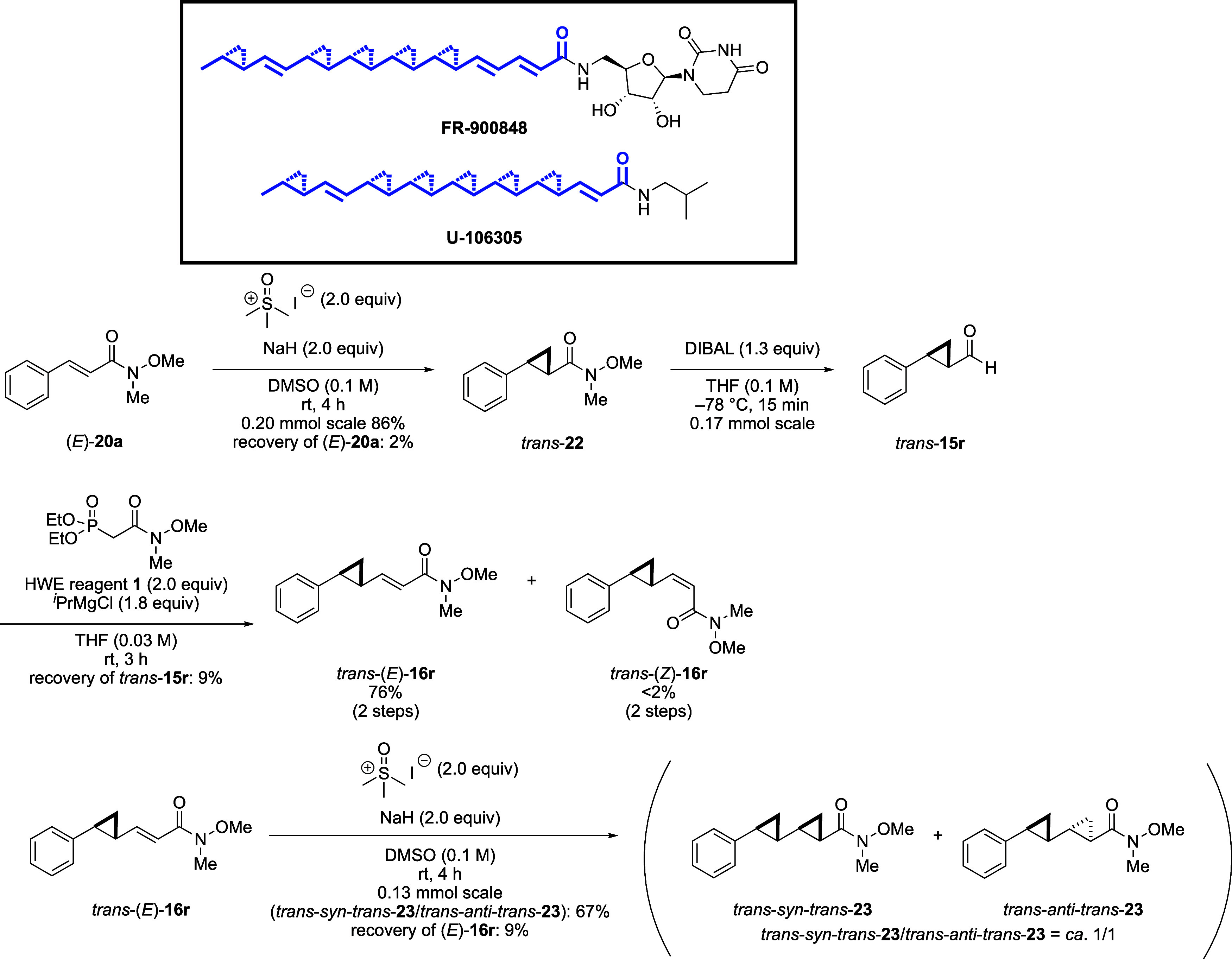
Synthesis of Biscyclopropane as a Model of Polycyclopropane Natural Products

As a second application of successive elongation, we conducted the synthesis of a polycyclopropane (Scheme 6).^23^ Some natural products, such as Jawsamycin (FR-900848)^24^ and U106305,^25^ contain a polycyclopropane structure. As a model substrate, we subjected the elongated unsaturated Weinreb amide (*E*)-**20a** to the cyclopropanation reaction. The reaction of (*E*)-**20a** with a sulfoxonium ylide gave cyclopropane *trans*-**22**, which was reduced to aldehyde *trans*-**15r**. This aldehyde was elongated again under our optimized conditions to give *trans*-(*E*)-**16r**. The obtained *trans*-(*E*)-**16r** was subjected to a second cyclopropanation reaction to afford a mixture of *trans*-*syn*-*trans*-**23** and *trans-anti*-*trans*-**23**, in which two cyclopropane rings are directly linked.

### Application of the HWE Reaction to a Ketone and Weinreb Ketone Syntheses

**Scheme 7:**
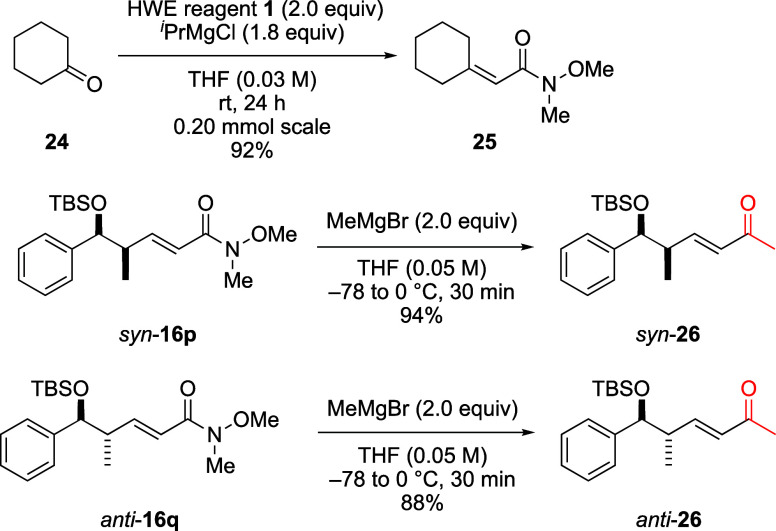
HWE Reaction of a Ketone and Weinreb Ketone Syntheses

Using our synthetic method to obtain α,β-unsaturated Weinreb amides from aldehydes via the HWE reaction, we applied the HWE reaction to a ketone and performed the Weinreb ketone syntheses of elongated products (Scheme 7).^5b^

The representative cyclic ketone cyclohexanone (**24**) was subjected to our optimized conditions to afford elongated Weinreb amide **25** in high yield, demonstrating that our conditions are applicable to cyclic ketones. Next, the Weinreb ketone syntheses were performed. Using MeMgBr, elongated *syn*-**16p** and *anti*-**16q** were converted to methyl ketones *syn*-**26** and *anti*-**26**, respectively, in high yields without epimerization or regioisomerization.

## Conclusion

We have developed an ^*i*^PrMgCl-deprotonating (*E*)-selective Weinreb amide-type HWE reaction. Using Nuzillard’s conditions, we observed low (*E*)-selectivity at low reaction temperatures, in contrast to Nuzillard’s results and in consistency with Davies’s argument. The magnesium cation proved to be conducive to the (*E*)-selectivity and reactivity under Weinreb amide-type HWE reaction conditions. The phosphonoenolate generated using ^*i*^PrMgCl could be isolated as a THF-desolvated dimer, which was prepared in toluene solvent. The desolvated dimer was very stable at room temperature for at least half a year in argon atmosphere. The isolated phosphonoenolate could be also used for the HWE reaction to give the corresponding (*E*)-olefin selectively and in high yield. This is the first example of an isolated phosphonoenolate mediating the HWE reaction. In addition, our optimized conditions were applicable to various aliphatic saturated aldehydes, aliphatic α,β-unsaturated aldehydes, and aromatic aldehydes, demonstrating their robustness and scalability. Our methodology was successfully applied to the successive elongation process, HWE reaction of a cyclic ketone, and Weinreb ketone syntheses, enabling the synthesis of polyene compounds and their derivatives. The crystallization of the phosphonoenolate, DFT calculation study of the reactive species, and experimental mechanistic study of this reaction are currently in progress and will be reported in the near future.

## Experimental Section

### General Methods

Melting points are recorded on a Yanaco MP-S3. ^1^H, ^13^C, ^31^P and ^19^F NMR spectra were recorded on a JEOL JNM-ECA500II or a Bruker Biospin AVANCE 400 M with chloroform (in chloroform-*d*), with benzene (in benzene-*d*_6_), with acetone (in acetone-*d*_6_) or with dimethyl sulfoxide (in dimethyl sulfoxide-*d*_6_) as internal standard. ^31^P NMR spectra were recorded with triphenyl phosphine as the internal standard and ^19^F NMR was recorded with trifluoromethylbenzene or fluorobenzene as the internal standard. Structural assignments were made with additional information from gHMQC and gHMBC experiments. IR spectra were recorded on a Horiba FT-300 (FT-IR) or Jasco FT/IR-4600 (ATR-IR). Absorbance frequencies are recorded in reciprocal centimeters (cm^–1^). High resolution mass spectra (HRMS) were obtained from a Bruker Daltonics micro TOF focus. Ionization was achieved by ESI, modes of ionization, calculated, and found mass are given. Optical rotations were determined using a Jasco P-1020. All reactions were carried out under argon atmosphere in dried glassware.

### Experimental Procedures and Physical Data of Compounds

#### General Procedure of ^i^PrMgCl-Deprotonating Weinreb Amide-Type Horner–Wadsworth–Emmons Reaction

To a solution of phosphate **1** (2.0 equiv: 95.7 mg, 0.400 mmol) in THF (4.7 mL), a 2.0 M solution of isopropylmagnesium chloride in THF (1.8 equiv: 0.18 mL, 0.360 mmol) was added at −78 °C. After the reaction mixture was stirred at −78 °C for 30 min, a solution of an aldehyde (1.0 equiv: 0.200 mmol) in THF (2.0 mL) was added at temperature (room temperature or reflux), and the reaction mixture was stirred for Time. To the reaction mixture, saturated aqueous ammonium chloride was added at 0 °C, and the mixture was extracted with ethyl acetate, and the organic layer was dried over sodium sulfate. After filtration of the mixture and concentration of the solvent, the crude mixture was purified by thin layer chromatography on silica to afford alkene.

#### Substrate Scope of Saturated Aliphatic Aldehyde

##### (2*E*)-*N*-Methoxy-*N*-methylbut-2-enamide [(*E*)-**16a**]

*R*_*f*_ = 0.55 (silica gel, hexane/ethyl acetate = 2/1); FT-IR (neat) ν_max_: 2970, 2939, 1666, 1635, 1450, 1412, 1381, 1180, 1011, 964 cm^–1^; ^1^H NMR (500 MHz, CDCl_3_, δ): 6.99 (dq, *J* = 15.5, 7.0 Hz, 1H, H-3), 6.49–6.37 (m, 1H, H-2), 3.71 (s, 3H, OMe), 3.24 (s, 3H, NMe), 1.92 (dd, *J* = 7.0, 1.0 Hz, 3H, H-4); ^13^C{^1^H} NMR (125 MHz, CDCl_3_): δ166.9 (C-1), 142.8 (C-3), 120.1 (C-2), 61.6 (OMe), 32.2 (NMe), 18.2 (C-4); HRMS calcd for C_6_H_11_NO_2_Na [M + Na]^+^ 152.0682; found, 152.0685.

##### (2*E*)-*N*-Methoxy-*N*-methylpent-2-enamide ((*E*)-**16b**)

*R*_*f*_ = 0.36 (silica gel, hexane/ethyl acetate = 2/1); FT-IR (neat) ν_max_: 2970, 2939, 1666, 1635, 1458, 1412, 1381, 1180, 995 cm^–1^; ^1^H NMR (500 MHz, CDCl_3_, δ): 7.03 (dt, *J* = 15.5, 6.5 Hz, 1H, H-3), 6.39 (dt, *J* = 15.5, 1.5 Hz, 1H, H-2), 3.71 (s, 3H, OMe), 3.24 (s, 3H, NMe), 2.32–2.22 (m, 2H, H-4), 1.09 (t, *J* = 7.5 Hz, 3H, H-5); ^13^C{^1^H} NMR (125 MHz, CDCl_3_): δ 167.1 (C-1), 149.2 (C-3), 117.7 (C-2), 61.6 (OMe), 32.3 (NMe), 25.5 (C-4), 12.5 (C-5); HRMS calcd for C_7_H_13_NO_2_Na [M + Na]^+^ 166.0838; found, 166.0835.

##### (2*E*)-*N*-Methoxy-*N*,4-dimethylpent-2-enamide ((*E*)-**16c**)

*R*_*f*_ = 0.44 (silica gel, hexane/ethyl acetate = 2/1); FT-IR (neat) ν_max_: 2962, 2870, 1666, 1635, 1466, 1412, 1381, 1180, 1003, 957 cm^–1^; ^1^H NMR (500 MHz, CDCl_3_, δ): 6.96 (dd, *J* = 15.5, 6.5 Hz, 1H, H-3), 6.34 (dd, *J* = 15.5, 1.5 Hz, 1H, H-2), 3.71 (s, 3H, OMe), 3.24 (s, 3H, NMe), 2.50 (qqdd, *J* = 7.0, 7.0, 6.5, 1.5 Hz, 1H, H-4), 1.08 (d, *J* = 7.0 Hz, 6H, 4-Me, H-5); ^13^C{^1^H} NMR (125 MHz, CDCl_3_): δ 167.2 (C-1), 154.0 (C-3), 115.8 (C-2), 61.5 (OMe), 32.3 (NMe), 31.1 (C-4), 21.4 (4-Me, C-5); HRMS calcd for C_8_H_15_NO_2_Na [M + Na]^+^ 180.0995; found, 180.0991.

##### (2*E*)-*N*-Methoxy-*N*,4,4-trimethylpent-2-enamide ((*E*)-**16d**)

*R*_*f*_ = 0.52 (silica gel, hexane/ethyl acetate = 2/1); FT-IR (neat) ν_max_: 2962, 2908, 2870, 1666, 1628, 1466, 1412, 1381, 1180, 1003 cm^–1^; ^1^H NMR (500 MHz, CDCl_3_, δ): 7.00 (d, *J* = 16.0 Hz, 1H, H-3), 6.30 (d, *J* = 16.0 Hz, 1H, H-2), 3.71 (s, 3H, OMe), 3.25 (s, 3H, NMe), 1.10 (s, 9H, 4-Me, H-5); ^13^C{^1^H} NMR (125 MHz, CDCl_3_): δ 167.4 (C-1), 157.7 (C-3), 113.7 (C-2), 61.6 (OMe), 33.7 (C-4), 32.4 (NMe), 28.8 (4-Me, C-5); HRMS calcd for C_18_H_34_N_2_O_4_Na [2 M + Na]^+^ 365.2411; found, 365.2403.

##### (2*E*)-*N*-Methoxy-*N*-methyl-5-phenylpent-2-enamide ((*E*)-**5c**, (*E*)-**16e**)

*R*_*f*_ = 0.37 (silica gel, hexane/ethyl acetate = 2/1); FT-IR (neat) ν_max_: 2931, 1658, 1635, 1450, 1419, 1381, 987, 702 cm^–1^; ^1^H NMR (500 MHz, CDCl_3_, δ): 7.33–7.24 (m, 2H, Ar), 7.23–7.15 (m, 3H, Ar), 7.01 (dt, *J* = 15.5, 7.0 Hz, 1H, H-3), 6.39 (d, *J* = 15.5 Hz, 1H, H-2), 3.63 (s, 3H, OMe), 3.23 (s, 3H, NMe), 2.80 (t, *J* = 7.5 Hz, 2H, H-5), 2.61–2.57 (m, 2H, H-4); ^13^C{^1^H} NMR (125 MHz, CDCl_3_): δ 166.8 (C-1), 146.5 (C-3), 141.0 (Ar), 128.384 (Ar), 126.0 (Ar), 119.3 (C-2), 61.6 (OMe), 34.5 (C-5), 34.2 (C-4), 32.3 (NMe); HRMS calcd for C_26_H_34_N_2_O_4_Na [2 M + Na]^+^ 461.2411; found, 461.2391.

#### (2*Z*)-*N*-Methoxy-*N*-methyl-5-phenylpent-2-enamide ((*Z*)-**5c**, (*Z*)-**16e**)

*R*_*f*_ = 0.56 (silica gel, hexane/ethyl acetate = 2/1); FT-IR (neat) ν_max_: 2931, 1658, 1450, 1435, 1350, 1003, 702 cm^–1^; ^1^H NMR (500 MHz, CDCl_3_, δ): 7.31–7.25 (m, 2H, Ar), 7.24–7.20 (m, 2H, Ar), 7.20–7.15 (m, 1H, Ar), 6.26 (brd, *J* = 11.5 Hz, 1H, H-2), 6.13 (dt, *J* = 11.5, 7.5 Hz, 1H, H-3), 3.61 (s, 3H, OMe), 3.20 (s, 3H, NMe), 2.97 (brdt, *J* = 7.5, 7.5 Hz, 2H, H-4), 2.78 (t, *J* = 7.5 Hz, 2H, H-5); ^13^C{^1^H} NMR (125 MHz, CDCl_3_): δ 167.2 (C-1), 146.1 (C-3), 141.3 (Ar), 128.5 (Ar), 128.2 (Ar), 125.8 (Ar), 118.6 (C-2), 61.4 (OMe), 35.2 (C-5), 31.8 (NMe), 30.4 (C-4); HRMS calcd for C_26_H_34_N_2_O_4_Na [2 M + Na]^+^ 461.2411; found, 461.2394.

##### (2*E*)-*N*-Methoxy-*N*-methylhept-2-enamide ((*E*)-**16f**)

*R*_*f*_ = 0.44 (silica gel, hexane/ethyl acetate = 2/1); FT-IR (neat) ν_max_: 2954, 2931, 2870, 1666, 1635, 1466, 1412, 1381, 995 cm^–1^; ^1^H NMR (500 MHz, CDCl_3_, δ): 6.98 (dt, *J* = 15.5, 7.0 Hz, 1H, H-3), 6.39 (dt, *J* = 15.5, 1.0 Hz, 1H, H-2), 3.70 (s, 3H, OMe), 3.24 (s, 3H, NMe), 2.24 (tdd, *J* = 7.5, 7.0, 1.0 Hz, 2H, H-4), 1.51–1.41 (m, 2H, H-5), 1.36 (tq, *J* = 7.5, 7.0 Hz, 2H, H-6), 0.91 (t, *J* = 7.0 Hz, 3H, H-7); ^13^C{^1^H} NMR (125 MHz, CDCl_3_): δ 167.1 (C-1), 147.9 (C-3), 118.6 (C-2), 61.6 (OMe), 32.3 (NMe), 32.2 (C-4), 30.4 (C-5), 22.2 (C-6), 13.8 (C-7); HRMS calcd for C_9_H_17_NO_2_Na [M + Na]^+^ 194.1151; found, 194.1156.

##### (2*E*)-*N*-Methoxy-*N*-methylnon-2-enamide ((*E*)-**5a**, (*E*)-**16g**)

*R*_*f*_ = 0.43 (silica gel, hexane/ethyl acetate = 2/1); FT-IR (neat) ν_max_: 2931, 2854, 1666, 1635, 1466, 1412, 1381, 995 cm^–1^; ^1^H NMR (500 MHz, CDCl_3_, δ): 6.98 (dt, *J* = 15.5, 7.0 Hz, 1H, H-3), 6.39 (dt, *J* = 15.5, 1.0 Hz, 1H, H-2), 3.70 (s, 3H, OMe), 3.24 (s, 3H, NMe), 2.23 (dtd, *J* = 7.0, 6.5, 1.0 Hz, 2H, H-4), 1.47 (tt, *J* = 7.5, 6.5 Hz, 2H, H-5), 1.38–1.16 (m, 6H, H-6, H-7, H-8), 0.88 (t, *J* = 7.0 Hz, 3H, H-9); ^13^C{^1^H} NMR (125 MHz, CDCl_3_): δ 167.1 (C-1), 148.0 (C-3), 118.6 (C-2), 61.6 (OMe), 32.5 (C-4), 32.3 (NMe), 31.6 (C-7), 28.8 (C-6), 28.2 (C-5), 22.5 (C-8), 14.0 (C-9); HRMS calcd for C_11_H_21_NO_2_Na [M + Na]^+^ 222.1465; found, 222.1473.

##### (2*Z*)-*N*-Methoxy-*N*-methylnon-2-enamide ((*Z*)-**5a**, (*Z*)-**16g**)

*R*_*f*_ = 0.62 (silica gel, hexane/ethyl acetate = 2/1); FT-IR (neat) ν_max_: 2954, 2924, 2854, 1658, 1458, 1435, 1342, 1003 cm^–1^; ^1^H NMR (500 MHz, CDCl_3_, δ): 6.23 (brd, *J* = 11.5 Hz, 1H, H-2), 6.12 (dt, *J* = 11.5, 7.5 Hz, 1H, H-3), 3.68 (s, 3H, OMe), 3.21 (s, 3H, NMe), 2.62 (dt, *J* = 7.5, 7.0 Hz, 2H, H-4), 1.43 (tt, *J* = 7.5, 7.0 Hz, 2H, H-5), 1.38–1.22 (m, 6H, H-6, H-7, H-8), 0.88 (t, *J* = 7.0 Hz, 3H, H-9); ^13^C{^1^H} NMR (125 MHz, CDCl_3_): δ 167.6 (C-1), 147.8 (C-3), 117.9 (C-2), 61.4 (OMe), 32.0 (NMe), 31.7 (C-7), 29.3 (C-5), 29.1 (C-4), 29.0 (C-6), 22.6 (C-8), 14.0 (C-9); HRMS calcd for C_11_H_21_NO_2_Na [M + Na]^+^ 222.1465; found, 222.1464.

##### (2*E*)-*N*-Methoxy-*N*-methyldec-2-enamide ((*E*)-**16h**)

*R*_*f*_ = 0.53 (silica gel, hexane/ethyl acetate = 2/1); FT-IR (neat) ν_max_: 2931, 2854, 1666, 1635, 1466, 1412, 1381, 1180, 987 cm^–1^; ^1^H NMR (500 MHz, CDCl_3_, δ): 6.98 (dt, *J* = 15.5, 7.0 Hz, 1H, H-3), 6.39 (dt, *J* = 15.5, 1.0 Hz, 1H, H-2), 3.70 (s, 3H, OMe), 3.24 (s, 3H, NMe), 2.23 (dtd, *J* = 7.0, 6.5, 1.0 Hz, 2H, H-4), 1.47 (tt, *J* = 7.5, 6.5 Hz, 2H, H-5), 1.37–1.17 (m, 8H, H-6, H-7, H-8, H-9), 0.88 (t, *J* = 7.0 Hz, 3H, H-10); ^13^C{^1^H} NMR (125 MHz, CDCl_3_): δ 167.0 (C-1), 148.0 (C-3), 118.5 (C-2), 61.6 (OMe), 32.4 (C-4), 32.3 (NMe), 31.7 (C-8), 29.1 (C-7), 29.0 (C-6), 28.2 (C-5), 22.6 (C-9), 14.0 (C-10); HRMS calcd for C_24_H_46_N_2_O_4_Na [2 M + Na]^+^ 449.3350; found, 449.3346.

##### (2*Z*)-*N*-Methoxy-*N*-methyldec-2-enamide ((*Z*)-**16h**)

*R*_*f*_ = 0.71 (silica gel, hexane/ethyl acetate = 2/1); FT-IR (neat) ν_max_: 2924, 2854, 1658, 1458, 1435, 1342, 1180, 1003 cm^–1^; ^1^H NMR (500 MHz, CDCl_3_, δ): 6.23 (brd, *J* = 11.5 Hz, 1H, H-2), 6.12 (dt, *J* = 11.5, 7.0 Hz, 1H, H-3), 3.68 (s, 3H, OMe), 3.22 (s, 3H, NMe), 2.62 (dt, *J* = 7.0, 7.0 Hz, 2H, H-4), 1.43 (tt, *J* = 7.5, 7.0 Hz, 2H, H-5), 1.38–1.19 (m, 8H, H-6, H-7, H-8, H-9), 0.87 (t, *J* = 7.0 Hz, 3H, H-10); ^13^C{^1^H} NMR (125 MHz, CDCl_3_): δ 167.6 (C-1), 147.8 (C-3), 117.9 (C-2), 61.4 (OMe), 31.9 (NMe), 31.8 (C-8), 29.308 (C-5, C-7), 29.13 (C-4), 29.09 (C-6), 22.6 (C-9), 14.1 (C-10); HRMS calcd for C_24_H_46_N_2_O_4_Na [2 M + Na]^+^ 449.3350; found, 449.3353.

##### (2*E*)-*N*-Methoxy-*N*,5-dimethylhex-2-enamide ((*E*)-**16i**)

*R*_*f*_ = 0.42 (silica gel, hexane/ethyl acetate = 2/1); FT-IR (neat) ν_max_: 2954, 1666, 1635, 1466, 1412, 1381, 995 cm^–1^; ^1^H NMR (500 MHz, CDCl_3_, δ): 6.96 (dt, *J* = 15.5, 7.5 Hz, 1H, H-3), 6.38 (d, *J* = 15.5 Hz, 1H, H-2), 3.70 (s, 3H, OMe), 3.24 (s, 3H, NMe), 2.17–2.08 (m, 2H, H-4), 1.77 (tqq, *J* = 7.5, 7.0, 7.0 Hz, 1H, H-5), 0.93 (d, *J* = 7.0 Hz, 6H, 5-Me, H-6); ^13^C{^1^H} NMR (125 MHz, CDCl_3_): δ 166.9 (C-1), 146.7 (C-3), 119.6 (C-2), 61.6 (OMe), 41.7 (C-4), 32.3 (NMe), 27.9 (C-5), 22.3 (5-Me, C-6); HRMS calcd for C_18_H_34_N_2_O_4_Na [2 M + Na]^+^ 365.2411; found, 365.2416.

##### (5*S*,2*E*)-*N*-Methoxy-*N*,5,9-trimethyldeca-2,8-dienamide ((*E*)-**16j**)

*R*_*f*_ = 0.26 (silica gel, hexane/ethyl acetate = 2/1); [α]_D_^21^ + 4.5 (*c* 1.00, CHCl_3_); FT-IR (neat) ν_max_: 2962, 2916, 1666, 1635, 1450, 1419, 1381, 995 cm^–1^; ^1^H NMR (500 MHz, CDCl_3_, δ): δ 6.96 (dt, *J* = 15.5, 7.5 Hz, 1H, H-3), 6.39 (d, *J* = 15.5 Hz, 1H, H-2), 5.12–5.04 (m, 1H, H-8), 3.70 (s, 3H, OMe), 3.25 (s, 3H, NMe), 2.30–2.20 (m, 1H, H-4), 2.14–1.89 (m, 3H, H-4, H-7), 1.69–1.58 (m, 1H, H-5), 1.68 (d, *J* = 1.0 Hz, 3H, H-10), 1.60 (s, 3H, 9-Me), 1.43–1.30 (m, 1H, H-6), 1.25–1.12 (m, 1H, H-6), 0.91 (d, *J* = 7.0 Hz, 3H, 5-Me); ^13^C{^1^H} NMR (125 MHz, CDCl_3_): δ 166.9 (C-1), 146.7 (C-3), 131.3 (C-9), 124.4 (C-8), 119.7 (C-2), 61.6 (OMe), 39.9 (C-4), 36.6 (C-6), 32.3 (NMe), 32.2 (C-5), 25.7 (C-10), 25.5 (C-7), 19.5 (5-Me), 17.6 (9-Me); HRMS calcd for C_28_H_50_N_2_O_4_Na [2 M + Na]^+^ 501.3663; found, 501.3671.

##### (5*S*,2*Z*)-*N*-Methoxy-*N*,5,9-trimethyldeca-2,8-dienamide ((*Z*)-**16j**)

*R*_*f*_ = 0.47 (silica gel, hexane/ethyl acetate = 2/1); [α]_D_^23^ + 12.9 (*c* 1.00, CHCl_3_); FT-IR (neat) ν_max_: 2962, 2916, 1658, 1442, 1342, 1003 cm^–1^; ^1^H NMR (500 MHz, CDCl_3_, δ): 6.28 (brd, *J* = 11.5 Hz, 1H, H-2), 6.13 (dt, *J* = 11.5, 7.0 Hz, 1H, H-3), 5.12–5.05 (m, 1H, H-8), 3.68 (s, 3H, OMe), 3.21 (s, 3H, NMe), 2.68–2.47 (m, 2H, H-4), 2.08–1.90 (m, 2H, H-7), 1.68 (s, 3H, H-10), 1.64–1.54 (m, 1H, H-5), 1.60 (s, 3H, 9-Me), 1.43–1.33 (m, 1H, H-6), 1.26–1.14 (m, 1H, H-6), 0.92 (d, *J* = 7.0 Hz, 3H, 5-Me); ^13^C{^1^H} NMR (125 MHz, CDCl_3_): δ 167.6 (C-1), 146.7 (C-3), 131.1 (C-9), 124.7 (C-8), 118.7 (C-2), 61.4 (OMe), 36.7 (C-6), 36.0 (C-4), 32.9 (C-5), 31.9 (NMe), 25.7 (C-10), 25.5 (C-7), 19.5 (5-Me), 17.6 (9-Me); HRMS calcd for C_28_H_50_N_2_O_4_Na [2 M + Na]^+^ 501.3663; found, 501.3645.

##### (2*E*,14*E*)-*N*^1^,*N*^16^-Dimethoxy-*N*^1^,*N*^16^-dimethylhexadeca-2,14-dienediamide ((*E*,*E*)-**16k**)

*R*_*f*_ = 0.20 (silica gel, hexane/ethyl acetate = 1/1); FT-IR (neat) ν_max_: 2924, 2854, 1666, 1635, 1466, 1442, 1381, 995 cm^–1^; ^1^H NMR (500 MHz, CDCl_3_, δ): 6.98 (dt, *J* = 15.5, 7.5 Hz, 2H, H-3, H-14), 6.39 (d, *J* = 15.5 Hz, 2H, H-2, H-15), 3.70 (s, 6H, OMe), 3.24 (s, 6H, NMe), 2.23 (dt, *J* = 7.5, 7.0 Hz, 4H, H-4, H-13), 1.46 (tt, *J* = 7.5, 7.0 Hz, 4H, H-5, H-12), 1.37–1.18 (m, 12H, H-6, H-7, H-8, H-9, H-10, H-11); ^13^C{^1^H} NMR (125 MHz, CDCl_3_): δ 167.1 (C-1, C-16), 147.9 (C-3, C-14), 118.5 (C-2, C-15), 61.6 (OMe), 32.5 (C-4, C-13), 32.3 (NMe), 29.5 (C-6 or C-7 or C-8 or C-9 or C-10 or C-11), 29.3 (C-6 or C-7 or C-8 or C-9 or C-10 or C-11), 29.1 (C-6 or C-7 or C-8 or C-9 or C-10 or C-11), 28.3 (C-5, C-12); HRMS calcd for C_20_H_36_N_2_O_4_Na [M + Na]^+^ 391.2567; found, 391.2565.

##### (2*E*,14*Z*)-*N*^1^,*N*^16^-Dimethoxy-*N*^1^,*N*^16^-dimethylhexadeca-2,14-dienediamide ((*E*,*Z*)-**16k**)

*R*_*f*_ = 0.30 (silica gel, hexane/ethyl acetate = 1/1); ATR-IR ν_max_: 2927, 2854, 1664, 1634, 1464, 1441, 1000 cm^–1^; ^1^H NMR (500 MHz, CDCl_3_, δ): 6.98 (dt, *J* = 15.5, 7.0 Hz, 1H, H-3), 6.39 (d, *J* = 15.5 Hz, 1H, H-2), 6.23 (brd, *J* = 12.0 Hz, 1H, H-15), 6.12 (dt, *J* = 12.0, 7.0 Hz, 1H, H-14), 3.70 (s, 3H, 1NOMe), 3.68 (s, 3H, 16NOMe), 3.24 (s, 3H, 1NMe), 3.21 (s, 3H, 16NMe), 2.61 (td, *J* = 7.5, 7.0 Hz, 2H, H-13), 2.23 (td, *J* = 7.5, 7.0 Hz, 2H, H-4), 1.51–1.38 (m, 4H, H-5, H-12), 1.38–1.19 (m, 12H, H-6, H-7, H-8, H-9, H-10, H-11); ^13^C{^1^H} NMR (125 MHz, CDCl_3_): δ 167.5 (C-16), 167.1 (C-1), 148.0 (C-3), 147.8 (C-14), 118.6 (C-2), 117.9 (C-15), 61.6 (1NOMe), 61.4 (16NOMe), 32.5 (C-4), 32.3 (1NMe), 32.0 (16NMe), 29.52 (C-6 or C-7 or C-8 or C-9 or C-10 or C-11), 29.49 (C-6 or C-7 or C-8 or C-9 or C-10 or C-11), 29.41 (C-6 or C-7 or C-8 or C-9 or C-10 or C-11), 29.37 (C-6 or C-7 or C-8 or C-9 or C-10 or C-11), 29.33 (C-6 or C-7 or C-8 or C-9 or C-10 or C-11), 29.28 (C-6 or C-7 or C-8 or C-9 or C-10 or C-11), 29.2 (C-13), 29.1 (C-12), 28.3 (C-5); HRMS calcd for C_20_H_36_N_2_O_4_Na [M + Na]^+^ 391.2567; found, 391.2575.

##### (2*E*)-*N*-Methoxy-*N*-methyl-4,4-diphenylbut-2-enamide ((*E*)-**16l**)

*R*_*f*_ = 0.42 (silica gel, hexane/ethyl acetate = 2/1); mp: 83.9 °C; ATR-IR ν_max_: 1656, 1615, 1494, 1452, 1422, 1387, 995, 707 cm^–1^; ^1^H NMR (500 MHz, CDCl_3_, δ): 7.43 (dd, *J* = 15.5, 7.5 Hz, 1H, H-3), 7.35–7.26 (m, 4H, Ar), 7.27–7.16 (m, 6H, Ar), 6.34 (d, *J* = 15.5 Hz, 1H, H-2), 4.91 (d, *J* = 7.5 Hz, 1H, H-4), 3.61 (s, 3H, OMe), 3.23 (s, 3H, NMe); ^13^C{^1^H} NMR (125 MHz, CDCl_3_): δ 166.6 (C-1), 148.3 (C-3), 142.1 (Ar), 128.545 (Ar), 126.7 (Ar), 120.3 (C-2), 61.7 (OMe), 53.6 (C-4), 32.4 (NMe); HRMS calcd for C_18_H_19_NO_2_Na [M + Na]^+^ 304.1308; found, 304.1308.

##### (2*Z*)-*N*-Methoxy-*N*-methyl-4,4-diphenylbut-2-enamide ((*Z*)-**16l**)

*R*_*f*_ = 0.61 (silica gel, hexane/ethyl acetate = 2/1); ATR-IR ν_max_: 1653, 1494, 1449, 1393, 1353, 1000, 704 cm^–1^; ^1^H NMR (500 MHz, CDCl_3_, δ): 7.34–7.21 (m, 8H, Ar), 7.25–7.16 (m, 2H, Ar), 6.57 (dd, *J* = 11.0, 10.5 Hz, 1H, H-3), 6.42 (brd, *J* = 11.0 Hz, 1H, H-2), 6.23 (brd, *J* = 10.5 Hz, 1H, H-4), 3.65 (s, 3H, OMe), 3.22 (s, 3H, NMe); ^13^C{^1^H} NMR (125 MHz, CDCl_3_): δ 166.9 (C-1), 147.3 (C-3), 143.4 (Ar), 128.5 (Ar), 128.4 (Ar), 126.4 (Ar), 117.6 (C-2), 61.6 (OMe), 48.0 (C-4), 32.0 (NMe); HRMS calcd for C_18_H_19_NO_2_Na [M + Na]^+^ 304.1308; found, 304.1311.

##### (2*E*)-*N*-Methoxy-*N*-methyl-3-cyclopropylprop-2-enamide ((*E*)-**16m**)

*R*_*f*_ = 0.31 (silica gel, hexane/ethyl acetate = 2/1); FT-IR (neat) ν_max_: 3008, 2962, 2939, 1658, 1628, 1466, 1427, 1389, 1180, 1011, 980, 957, 941 cm^–1^; ^1^H NMR (500 MHz, CDCl_3_, δ): 6.50 (d, *J* = 15.5 Hz, 1H, H-2), 6.42 (dd, *J* = 15.5, 10.0 Hz, 1H, H-3), 3.71 (s, 3H, OMe), 3.23 (s, 3H, NMe), 1.67–1.53 (m, 1H, H-1′), 0.98–0.85 (m, 2H, H-2′, H-3′), 0.72–0.57 (m, 2H, H-2′, H-3′); ^13^C{^1^H} NMR (125 MHz, CDCl_3_): δ 167.1 (C-1), 152.6 (C-3), 115.6 (C-2), 61.6 (OMe), 32.3 (NMe), 14.7 (C-1′), 8.4 (C-2′, C-3′); HRMS calcd for C_8_H_13_NO_2_Na [M + Na]^+^ 178.0838; found, 178.0835.

##### (2*Z*)-*N*-Methoxy-*N*-methyl-3-cyclopropylprop-2-enamide ((*Z*)-**16m**)

*R*_*f*_ = 0.46 (silica gel, hexane/ethyl acetate = 2/1); ATR-IR ν_max_: 3005, 2937, 1654, 1623, 1463, 1445, 1353, 1179, 1002, 948, 926, 815 cm^−1^; ^1^H NMR (500 MHz, CDCl_3_, δ): 6.19 (d, *J* = 11.0 Hz, 1H, H-2), 5.38 (dd, *J* = 11.5, 11.0 Hz, 1H, H-3), 3.69 (s, 3H, OMe), 3.24 (s, 3H, NMe), 3.01–2.85 (m, 1H, H-1′), 1.02–0.90 (m, 2H, H-2′, H-3′), 0.58–0.46 (m, 2H, H-2′, H-3′); ^13^C{^1^H} NMR (125 MHz, CDCl_3_): δ 168.0 (C-1), 153.0 (C-3), 115.1 (C-2), 61.5 (OMe), 32.1 (NMe), 11.8 (C-1′), 8.8 (C-2′, C-3′); HRMS calcd for C_8_H_13_NO_2_Na [M + Na]^+^ 178.0838; found, 178.0831.

##### (2*E*)-*N*-Methoxy-*N*-methyl-3-cyclopentylprop-2-enamide ((*E*)-**16n**)

*R*_*f*_ = 0.38 (silica gel, hexane/ethyl acetate = 2/1); FT-IR (neat) ν_max_: 2954, 2870, 1658, 1628, 1450, 1419, 1381, 995 cm^–1^; ^1^H NMR (500 MHz, CDCl_3_, δ): 6.96 (dd, *J* = 15.0, 8.0 Hz, 1H, H-3), 6.37 (d, *J* = 15.0 Hz, 1H, H-2), 3.70 (s, 3H, OMe), 3.24 (s, 3H, NMe), 2.64 (ddddd, *J* = 8.5, 8.0, 8.0, 8.0, 8.0 Hz, 1H, H-1′), 1.92–1.78 (m, 2H, H-2′, H-5′), 1.77–1.52 (m, 4H, H-3′, H-4′), 1.52–1.35 (m, 2H, H-2′, H-5′); ^13^C{^1^H} NMR (125 MHz, CDCl_3_): δ 167.3 (C-1), 152.1 (C-3), 116.7 (C-2), 61.6 (OMe), 43.1 (C-1′), 32.6 (C-2′, C-5′), 32.4 (NMe), 25.3 (C-3′, C-4′); HRMS calcd for C_10_H_17_NO_2_Na [M + Na]^+^ 206.1151; found, 206.1150.

##### (2*Z*)-*N*-Methoxy-*N*-methyl-3-cyclopentylprop-2-enamide ((*Z*)-**16n**)

*R*_*f*_ = 0.58 (silica gel, hexane/ethyl acetate = 2/1); ATR-IR ν_max_: 2954, 2868, 1660, 1633, 1433, 1347, 1001 cm^–1^; ^1^H NMR (500 MHz, CDCl_3_, δ): 6.16 (brd, *J* = 11.0 Hz, 1H, H-2), 6.01 (dd, *J* = 11.0, 10.5 Hz, 1H, H-3), 3.72–3.52 (m, 1H, H-1′), 3.68 (s, 3H, OMe), 3.21 (s, 3H, NMe), 1.98–1.86 (m, 2H, H-2′, H-5′), 1.77–1.53 (m, 4H, H-3′, H-4′), 1.35–1.18 (m, 2H, H-2′, H-5′); ^13^C{^1^H} NMR (125 MHz, CDCl_3_): δ 167.7 (C-1), 152.3 (C-3), 116.5 (C-2), 61.4 (OMe), 39.3 (C-1′), 33.6 (C-2′, C-5′), 32.0 (NMe), 25.6 (C-3′, C-4′); HRMS calcd for C_10_H_17_NO_2_Na [M + Na]^+^ 206.1151; found, 206.1148.

##### (2*E*)-*N*-Methoxy-*N*-methyl-3-cyclohexylprop-2-enamide ((*E*)-**16o**)

*R*_*f*_ = 0.44 (silica gel, hexane/ethyl acetate = 2/1); FT-IR (neat) ν_max_: 2924, 2854, 1666, 1635, 1450, 1412, 1381, 1180, 1003, 987, 964 cm^–1^; ^1^H NMR (500 MHz, CDCl_3_, δ): 6.93 (dd, *J* = 15.5, 6.5 Hz, 1H, H-3), 6.34 (d, *J* = 15.5 Hz, 1H, H-2), 3.70 (s, 3H, OMe), 3.24 (s, 3H, NMe), 2.23–2.12 (m, 1H, H-1′), 1.83–1.70 (m, 4H, H-2′, H-3′, H-5′, H-6′), 1.72–1.63 (m, 1H, H-4′), 1.37–1.23 (m, 2H, H-2′, H-6′), 1.25–1.10 (m, 3H, H-3′, H-4′, H-5′); ^13^C{^1^H} NMR (125 MHz, CDCl_3_): δ 167.3 (C-1), 152.9 (C-3), 116.1 (C-2), 61.6 (OMe), 40.7 (C-1′), 32.3 (NMe), 31.9 (C-2′, C-6′), 25.9 (C-4′), 25.7 (C-3′, C-5′); HRMS calcd for C_22_H_38_N_2_O_4_Na [2 M + Na]^+^ 417.2724; found, 417.2742.

##### (2*Z*)-*N*-Methoxy-*N*-methyl-3-cyclohexylprop-2-enamide ((*Z*)-**16o**)

*R*_*f*_ = 0.70 (silica gel, hexane/ethyl acetate = 2/1); FT-IR (neat) ν_max_: 2924, 2854, 1658, 1442, 1342, 1003 cm^–1^; ^1^H NMR (500 MHz, CDCl_3_, δ): 6.14 (brd, *J* = 11.0 Hz, 1H, H-2), 5.93 (dd, *J* = 11.0, 10.5 Hz, 1H, H-3), 3.68 (s, 3H, OMe), 3.35–3.15 (m, 1H, H-1′), 3.22 (s, 3H, NMe), 1.80–1.60 (m, 5H, H-2′, H-3′, H-4′, H-5′, H-6′), 1.42–1.28 (m, 2H, H-2′, H-6′), 1.24–1.00 (m, 3H, H-4′, H-3′, H-5′); ^13^C{^1^H} NMR (125 MHz, CDCl_3_): δ 167.5 (C-1), 153.0 (C-3), 115.9 (C-2), 61.4 (OMe), 37.3 (C-1′), 32.5 (C-2′, C-6′), 32.0 (NMe), 26.0 (C-4′), 25.5 (C-3′, C-5′); HRMS calcd for C_11_H_19_NO_2_Na [M + Na]^+^ 220.1308; found, 220.1312.

##### (4*RS*,5*SR*,2*E*)-5-((*tert*-Butyldimethylsilyl)oxy)-*N*-methoxy-*N*,4-dimethyl-5-phenylpent-2-enamide ((*E*)-**16p**)

*R*_*f*_ = 0.53 (silica gel, hexane/ethyl acetate = 2/1); FT-IR (neat) ν_max_: 2954, 2931, 2893, 2854, 1666, 1635, 1466, 1412, 1381, 1257, 1088, 1065, 841, 779, 702 cm^–1^; ^1^H NMR (500 MHz, CDCl_3_, δ): 7.30–7.22 (m, 4H, Ar), 7.22–7.17 (m, 1H, Ar), 6.90 (dd, *J* = 15.5, 8.0 Hz, 1H, H-3), 6.21 (d, *J* = 15.5 Hz, 1H, H-2), 4.58 (d, *J* = 6.0 Hz, 1H, H-5), 3.54 (s, 3H, OMe), 3.20 (s, 3H, NMe), 2.62 (dqd, *J* = 8.0, 7.0, 6.0 Hz, 1H, H-4), 1.06 (d, *J* = 7.0 Hz, 3H, 4-Me), 0.88 (s, 9H, TBS), 0.03 (s, 3H, TBS), −0.21 (s, 3H, TBS); ^13^C{^1^H} NMR (125 MHz, CDCl_3_): δ 166.7 (C-1), 149.6 (C-3), 143.1 (Ar), 127.7 (Ar), 127.0 (Ar), 126.6 (Ar), 118.7 (C-2), 78.0 (C-5), 61.5 (OMe), 45.7 (C-4), 32.2 (NMe), 25.8 (TBS), 18.1 (TBS), 14.6 (4-Me), −4.7 (TBS), −5.1 (TBS); HRMS calcd for C_20_H_33_NO_3_SiNa [M + Na]^+^ 386.2122; found, 386.2103.

##### (4*RS*,5*SR*,2*Z*)-5-((*tert*-Butyldimethylsilyl)oxy)-*N*-methoxy-*N*,4-dimethyl-5-phenylpent-2-enamide ((*Z*)-**16p**)

*R*_*f*_ = 0.75 (silica gel, hexane/ethyl acetate = 2/1); FT-IR (neat) ν_max_: 2954, 2931, 2893, 2854, 1658, 1458, 1350, 1257, 1095, 1026, 1003, 856, 841, 779, 702 cm^–1^; ^1^H NMR (500 MHz, CDCl_3_, δ): 7.38–7.32 (m, 2H, Ar), 7.31–7.23 (m, 2H, Ar), 7.21–7.14 (m, 1H, Ar), 6.20 (brd, *J* = 11.0 Hz, 1H, H-2), 6.05 (dd, *J* = 11.0, 11.0 Hz, 1H, H-3), 4.76 (d, *J* = 4.0 Hz, 1H, H-5), 3.76–3.59 (m, 1H, H-4), 3.55 (s, 3H, OMe), 3.19 (s, 3H, NMe), 0.94 (d, *J* = 6.0 Hz, 3H, 4-Me), 0.91 (s, 9H, TBS), 0.00 (s, 3H, TBS), −0.22 (s, 3H, TBS); ^13^C{^1^H} NMR (125 MHz, CDCl_3_): δ 167.2 (C-1), 150.6 (C-3), 143.8 (Ar), 127.6 (Ar), 126.6 (Ar), 126.5 (Ar), 117.2 (C-2), 77.8 (C-5), 61.4 (OMe), 41.2 (C-4), 32.0 (NMe), 25.8 (TBS), 18.2 (TBS), 14.0 (4-Me), −4.6 (TBS), −5.1 (TBS); HRMS calcd for C_20_H_33_NO_3_SiNa [M + Na]^+^ 386.2122; found, 386.2105.

##### (4*SR*,5*SR*,2*E*)-5-((*tert*-Butyldimethylsilyl)oxy)-*N*-methoxy-*N*,4-dimethyl-5-phenylpent-2-enamide ((*E*)-**16q**)

*R*_*f*_ = 0.68 (silica gel, hexane/ethyl acetate = 2/1); FT-IR (neat) ν_max_: 2954, 2931, 2893, 2854, 1666, 1635, 1466, 1412, 1381, 1257, 1088, 1065, 841, 779, 702 cm^–1^; ^1^H NMR (500 MHz, CDCl_3_, δ): 7.32–7.17 (m, 5H, Ar), 6.99 (dd, *J* = 15.5, 8.5 Hz, 1H, H-3), 6.26 (d, *J* = 15.5 Hz, 1H, H-2), 4.53 (d, *J* = 5.5 Hz, 1H, H-5), 3.58 (s, 3H, OMe), 3.22 (s, 3H, NMe), 2.66–2.56 (m, 1H, H-4), 0.96 (d, *J* = 7.0 Hz, 3H, 4-Me), 0.86 (s, 9H, TBS), 0.02 (s, 3H, TBS), −0.22 (s, 3H, TBS); ^13^C{^1^H} NMR (125 MHz, CDCl_3_): δ 166.7 (C-1), 149.4 (C-3), 143.1 (Ar), 127.7 (Ar), 127.0 (Ar), 126.6 (Ar), 118.8 (C-2), 78.6 (C-5), 61.5 (OMe), 45.8 (C-4), 32.2 (NMe), 25.7 (TBS), 18.1 (TBS), 16.2 (4-Me), −4.7 (TBS), −5.1 (TBS); HRMS calcd for C_20_H_33_NO_3_SiNa [M + Na]^+^ 386.2122; found, 386.2122.

##### (4*SR*,5*SR*,2*Z*)-5-((*tert*-Butyldimethylsilyl)oxy)-*N*-methoxy-*N*,4-dimethyl-5-phenylpent-2-enamide ((*Z*)-**16q**)

*R*_*f*_ = 0.78 (silica gel, hexane/ethyl acetate = 2/1); FT-IR (neat) ν_max_: 2954, 2931, 2893, 2854, 1658, 1458, 1257, 1095, 1065, 1003, 856, 841, 779, 702 cm^–1^; ^1^H NMR (500 MHz, CDCl_3_, δ): 7.30–7.21 (m, 4H, Ar), 7.20–7.13 (m, 1H, Ar), 6.18 (brd, *J* = 11.0 Hz, 1H, H-2), 6.10 (dd, *J* = 11.0, 11.0 Hz, 1H, H-3), 4.61 (d, *J* = 5.5 Hz, 1H, H-5), 3.81–3.59 (m, 1H, H-4), 3.44 (s, 3H, OMe), 3.11 (s, 3H, NMe), 1.05 (d, *J* = 7.0 Hz, 3H, 4-Me), 0.90 (s, 9H, TBS), 0.06 (s, 3H, TBS), −0.21 (s, 3H, TBS); ^13^C{^1^H} NMR (125 MHz, CDCl_3_): δ 167.1 (C-1), 148.4 (C-3), 143.7 (Ar), 127.6 (Ar), 126.7 (Ar), 126.5 (Ar), 118.2 (C-2), 78.3 (C-5), 61.3 (OMe), 41.5 (C-4), 31.9 (NMe), 25.8 (TBS), 18.2 (TBS), 17.3 (4-Me), −4.6 (TBS), −5.1 (TBS); HRMS calcd for C_20_H_33_NO_3_SiNa [M + Na]^+^ 386.2122; found, 386.2132.

##### (2*E*)-*N*-Methoxy-*N*-methyl-3-((1*RS*,2*SR*)-2-phenylcyclopropyl)prop-2-enamide ((*E*)-**16r**)

*R*_*f*_ = 0.22 (silica gel, hexane/ethyl acetate = 2/1); FT-IR (neat) ν_max_: 3001, 2939, 1658, 1628, 1496, 1458, 1419, 1373, 1180, 1088, 1003, 964, 926, 748, 702 cm^–1^; ^1^H NMR (500 MHz, CDCl_3_, δ): 7.33–7.23 (m, 2H, Ar), 7.22–7.15 (m, 1H, Ar), 7.13–7.04 (m, 2H, Ar), 6.59 (dd, *J* = 15.0, 10.0 Hz, 1H, H-3), 6.50 (d, *J* = 15.0 Hz, 1H, H-2), 3.70 (s, 3H, OMe), 3.24 (s, 3H, NMe), 2.18 (ddd, *J* = 9.0, 6.0, 4.5 Hz, 1H, H-5), 1.93–1.82 (m, 1H, H-4), 1.44 (ddd, *J* = 8.0, 6.0, 5.5 Hz, 1H, CH_2_), 1.32 (ddd, *J* = 9.0, 6.0, 5.5 Hz, 1H, CH_2_); ^13^C{^1^H} NMR (125 MHz, CDCl_3_): δ 166.9 (C-1), 150.1 (C-3), 141.1 (Ar), 128.4 (Ar), 126.0 (Ar), 125.8 (Ar), 116.4 (C-2), 61.7 (OMe), 32.4 (NMe), 27.4 (C-4), 26.6 (C-5), 17.8 (CH_2_); HRMS calcd for C_14_H_17_NO_2_Na [M + Na]^+^ 254.1151; found, 254.1147.

##### (2*Z*)-*N*-Methoxy-*N*-methyl-3-((1*RS*,2*SR*)-2-phenylcyclopropyl)prop-2-enamide ((*Z*)-**16r**)

*R*_*f*_ = 0.44 (silica gel, hexane/ethyl acetate = 2/1); ATR-IR ν_max_: 1654, 1621, 1497, 1459, 1350, 1179, 1001, 751, 699 cm^–1^; ^1^H NMR (500 MHz, CDCl_3_, δ): 7.27–7.21 (m, 2H, Ar), 7.17–7.08 (m, 3H, Ar), 6.25 (brd, *J* = 11.0 Hz, 1H, H-2), 5.56 (dd, *J* = 11.0, 11.0 Hz, 1H, H-3), 3.69 (s, 3H, OMe), 3.41–3.27 (m, 1H, H-4), 3.21 (s, 3H, NMe), 2.05 (ddd, *J* = 9.0, 5.5, 4.5 Hz, 1H, H-5), 1.40 (ddd, *J* = 7.5, 5.5, 5.0 Hz, 1H, CH_2_), 1.17 (ddd, *J* = 9.0, 5.5, 5.0 Hz, 1H, CH_2_); ^13^C{^1^H} NMR (125 MHz, CDCl_3_): δ 167.7 (C-1), 150.6 (C-3), 141.1 (Ar), 128.3 (Ar), 126.0 (Ar), 125.8 (Ar), 115.5 (C-2), 61.5 (OMe), 32.0 (NMe), 27.1 (C-5), 23.7 (C-4), 18.6 (CH_2_); HRMS calcd for C_14_H_17_NO_2_Na [M + Na]^+^ 254.1151; found, 254.1141.

#### Substrate Scope of Unsaturated Aliphatic Aldehyde

##### (2*E*,4*E*)-*N*-Methoxy-*N*-methylhepta-2,4-dienamide ((*E*)-**18a**)

*R*_*f*_ = 0.39 (silica gel, hexane/ethyl acetate = 2/1); FT-IR (neat) ν_max_: 2970, 2939, 1658, 1628, 1612, 1381, 1003 cm^–1^; ^1^H NMR (500 MHz, CDCl_3_, δ): 7.32 (dd, *J* = 15.5, 10.0 Hz, 1H, H-3), 6.39 (d, *J* = 15.5 Hz, 1H, H-2), 6.23 (dd, *J* = 15.0, 10.0 Hz, 1H, H-4), 6.17 (dt, *J* = 15.0, 6.0 Hz, 1H, H-5), 3.71 (s, 3H, OMe), 3.25 (s, 3H, NMe), 2.20 (qd, *J* = 7.0, 6.0 Hz, 2H, H-6), 1.05 (t, *J* = 7.0 Hz, 3H, H-7); ^13^C{^1^H} NMR (125 MHz, CDCl_3_): δ 167.4 (C-1), 145.2 (C-5), 143.9 (C-3), 127.8 (C-4), 116.9 (C-2), 61.6 (OMe), 32.4 (NMe), 25.9 (C-6), 12.9 (C-7); HRMS calcd for C_9_H_15_NO_2_Na [M + Na]^+^ 192.0995; found, 192.0990.

##### (2*Z*,4*E*)-*N*-Methoxy-*N*-methylhepta-2,4-dienamide ((*Z*)-**18a**)

*R*_*f*_ = 0.58 (silica gel, hexane/ethyl acetate = 2/1); ATR-IR ν_max_: 2967, 2937, 1653, 1595, 1463, 1437, 1350, 1180, 1001 cm^–1^; ^1^H NMR (500 MHz, CDCl_3_, δ): 7.41 (dd, *J* = 15.0, 11.5 Hz, 1H, H-4), 6.51 (dd, *J* = 11.5, 11.5 Hz, 1H, H-3), 6.09 (d, *J* = 11.5 Hz, 1H, H-2), 6.05 (dt, *J* = 15.0, 7.0 Hz, 1H, H-5), 3.69 (s, 3H, OMe), 3.23 (s, 3H, NMe), 2.21 (qd, *J* = 7.5, 7.0 Hz, 2H, H-6), 1.05 (t, *J* = 7.5 Hz, 3H, H-7); ^13^C{^1^H} NMR (125 MHz, CDCl_3_): δ 167.5 (C-1), 145.9 (C-5), 143.4 (C-3), 126.3 (C-4), 113.7 (C-2), 61.6 (OMe), 32.1 (NMe), 26.0 (C-6), 13.1 (C-7); HRMS calcd for C_9_H_15_NO_2_Na [M + Na]^+^ 192.0995; found, 192.0993.

##### (2*E*,4*E*)-*N*-Methoxy-*N*,4-dimethylhepta-2,4-dienamide ((*E*)-**18b**)

*R*_*f*_ = 0.44 (silica gel, hexane/ethyl acetate = 2/1); ATR-IR ν_max_: 2967, 2936, 1659, 1609, 1463, 1416, 1379, 1004 cm^–1^; ^1^H NMR (500 MHz, CDCl_3_, δ): 7.36 (d, *J* = 15.5 Hz, 1H, H-3), 6.36 (d, *J* = 15.5 Hz, 1H, H-2), 5.90 (t, *J* = 7.5 Hz, 1H, H-5), 3.72 (s, 3H, OMe), 3.27 (s, 3H, NMe), 2.21 (dt, *J* = 7.5, 7.5 Hz, 2H, H-6), 1.81 (s, 3H, 4-Me), 1.03 (t, *J* = 7.5 Hz, 3H, H-7); ^13^C{^1^H} NMR (125 MHz, CDCl_3_): δ 167.7 (C-1), 148.5 (C-3), 143.1 (C-5), 132.4 (C-4), 112.9 (C-2), 61.6 (OMe), 32.4 (NMe), 22.0 (C-6), 13.5 (C-7), 12.2 (4-Me); HRMS calcd for C_10_H_17_NO_2_Na [M + Na]^+^ 206.1151; found, 206.1160.

##### (2*E*)-*N*-Methoxy-*N*-methyl-5-(trimethylsilyl)pent-2-en-4-ynamide ((*E*)-**18c**)

*R*_*f*_ = 0.55 (silica gel, hexane/ethyl acetate = 2/1); FT-IR (neat) ν_max_: 2962, 1658, 1604, 1381, 1250, 1057, 995, 849, 764 cm^–1^; ^1^H NMR (500 MHz, CDCl_3_, δ): 6.86 (d, *J* = 15.5 Hz, 1H, H-3), 6.78 (d, *J* = 15.5 Hz, 1H, H-2), 3.72 (s, 3H, OMe), 3.26 (s, 3H, NMe), 0.22 (s, 9H, TMS); ^13^C{^1^H} NMR (125 MHz, CDCl_3_): δ 165.6 (C-1), 128.7 (C-3), 123.5 (C-2), 103.4 (C-4), 102.3 (C-5), 62.0 (OMe), 32.3 (NMe), −0.4 (TMS); HRMS calcd for C_20_H_34_N_2_O_4_Si_2_Na [2 M + Na]^+^ 445.1949; found, 445.1969.

##### (2*E*,4*E*)-*N*-Methoxy-*N*-methyl-5-phenylpenta-2,4-dienamide ((*E*)-**18d**)

*R*_*f*_ = 0.38 (silica gel, hexane/ethyl acetate = 2/1); mp: 64.0 °C; FT-IR (KBr) ν_max_: 3433, 1643, 1604, 1381, 1018, 995, 694 cm^–1^; ^1^H NMR (500 MHz, C_6_D_6_, δ): 7.84 (dd, *J* = 15.0, 11.0 Hz, 1H, H-3), 7.22–7.12 (m, 2H, Ar), 7.12–6.97 (m, 3H, Ar), 6.72 (dd, *J* = 15.0, 11.0 Hz, 1H, H-4), 6.65 (d, *J* = 15.0 Hz, H-5), 6.51 (d, *J* = 15.0 Hz, 1H, H-2), 3.12 (s, 3H, OMe), 3.01 (s, 3H, NMe); ^13^C{^1^H} NMR (125 MHz, C_6_D_6_): δ 167.1 (C-1), 143.5 (C-3), 139.6 (C-5), 136.8 (Ar), 128.8 (Ar), 128.7 (Ar), 127.4 (Ar), 127.2 (C-4), 120.1 (C-2), 61.1 (OMe), 32.2 (NMe); HRMS calcd for C_13_H_15_NO_2_Na [M + Na]^+^ 240.0995; found, 240.1003.

##### (2*Z*,4*E*)-*N*-Methoxy-*N*-methyl-5-phenylpenta-2,4-dienamide ((*Z*)-**18d**, (2*Z*,4*E*)-**18d**)

*R*_*f*_ = 0.54 (silica gel, hexane/ethyl acetate = 2/1); mp: 61.7 °C; FT-IR (KBr) ν_max_: 3433, 1635, 1612, 1581, 1458, 1350, 995, 810, 756, 694 cm^–1^; ^1^H NMR (500 MHz, C_6_D_6_, δ): 8.87 (dd, *J* = 15.5, 11.5 Hz, 1H, H-4), 7.43–7.35 (m, 2H, Ar), 7.06–6.93 (m, 3H, Ar), 6.56 (d, *J* = 15.5 Hz, 1H, H-5), 6.51 (dd, *J* = 11.5, 11.5 Hz, 1H, H-3), 6.26 (d, *J* = 11.5 Hz, 1H, H-2), 3.06 (s, 3H, OMe), 2.95 (s, 3H, NMe); ^13^C{^1^H} NMR (125 MHz, C_6_D_6_): δ 167.4 (C-1), 143.0 (C-3), 140.5 (C-5), 137.1 (Ar), 128.9 (Ar), 128.7 (Ar), 127.7 (Ar), 126.3 (C-4), 116.3 (C-2), 61.0 (OMe), 31.9 (NMe); HRMS calcd for C_13_H_15_NO_2_Na [M + Na]^+^ 240.0995; found, 240.0990.

##### (2*E*,4*Z*)-4-Bromo-*N*-methoxy-*N*-methyl-5-phenylpenta-2,4-dienamide ((*E*)-**18e**)

*R*_*f*_ = 0.43 (silica gel, hexane/ethyl acetate = 2/1); mp: 75.8 °C; ATR-IR ν_max_: 2967, 1646, 1606, 1592, 1464, 1446, 1414, 1380, 1146, 1002, 968, 853, 755, 694, 522 cm^–1^; ^1^H NMR (500 MHz, CDCl_3_, δ): 7.82–7.73 (m, 2H, Ar), 7.51 (d, *J* = 14.5 Hz, 1H, H-3), 7.46–7.33 (m, 3H, Ar), 7.29 (s, 1H, H-5), 6.94 (d, *J* = 14.5 Hz, 1H, H-2), 3.78 (s, 3H, OMe), 3.31 (s, 3H, NMe); ^13^C{^1^H} NMR (125 MHz, CDCl_3_): δ 166.3 (C-1), 143.7 (C-3), 138.4 (C-5), 134.9 (Ar), 129.9 (Ar), 129.2 (Ar), 128.3 (Ar), 121.1 (C-4), 120.9 (C-2), 62.0 (OMe), 32.5 (NMe); HRMS calcd for C_13_H_14_BrNO_2_Na [M + Na]^+^ 318.0100; found, 318.0113.

##### (2*E*,4*E*,6*E*)-*N*-Methoxy-*N*-methyl-7-phenylhepta-2,4,6-trienamide ((*E*)-**18f**)

*R*_*f*_ = 0.27 (silica gel, hexane/ethyl acetate = 2/1); mp: 79.9 °C; FT-IR (KBr) ν_max_: 3433, 2931, 1635, 1589, 1458, 1419, 1381, 1173, 1018, 995, 949, 748, 687 cm^–1^; ^1^H NMR (500 MHz, C_6_D_6_, δ): 7.84 (dd, *J* = 15.0, 11.5 Hz, 1H, H-3), 7.35–6.95 (m, 5H, Ar), 6.64 (dd, *J* = 15.5, 11.5 Hz, 1H, H-6), 6.63 (d, *J* = 15.0 Hz, 1H, H-2), 6.35 (dd, *J* = 15.0, 11.5 Hz, 1H, H-5), 6.34 (d, *J* = 15.5 Hz, 1H, H-7), 6.25 (dd, *J* = 15.0, 11.5 Hz, 1H, H-4), 3.10 (s, 3H, OMe), 3.00 (s, 3H, NMe); ^13^C{^1^H} NMR (125 MHz, C_6_D_6_): δ 167.2 (C-1), 143.4 (C-3), 140.3 (C-5), 137.3 (Ar), 136.1 (C-7), 131.3 (C-4), 128.9 (Ar), 128.5 (C-6), 128.3 (Ar), 127.0 (Ar), 119.7 (C-2), 61.1 (OMe), 32.3 (NMe); HRMS calcd for C_30_H_34_N_2_O_4_Na [2 M + Na]^+^ 509.2411; found, 509.2429.

##### (2*Z*,4*E*,6*E*)-*N*-Methoxy-*N*-methyl-7-phenylhepta-2,4,6-trienamide ((*Z*)-**18f**)

*R*_*f*_ = 0.47 (silica gel, hexane/ethyl acetate = 2/1); FT-IR (neat) ν_max_: 2970, 2931, 1643, 1597, 1566, 1435, 1350, 1180, 1095, 1003, 802, 756, 694 cm^–1^; ^1^H NMR (500 MHz, C_6_D_6_, δ): 8.42 (dd, *J* = 15.0, 11.5 Hz, 1H, H-4), 7.15–7.05 (m, 4H, Ar), 7.05–6.98 (m, 1H, Ar), 6.75 (dd, *J* = 15.5, 11.0 Hz, 1H, H-6), 6.49 (dd, *J* = 11.5, 11.5 Hz, 1H, H-3), 6,43 (d, *J* = 15.5 Hz, 1H, H-7), 6.38 (dd, *J* = 15.0, 11.0 Hz, 1H, H-5), 6.26 (d, *J* = 11.5 Hz, 1H, H-2 Hz), 3.06 (s, 3H, OMe), 2.96 (s, 3H, NMe); ^13^C{^1^H} NMR (125 MHz, C_6_D_6_): δ 167.4 (C-1), 142.8 (C-3), 141.0 (C-5), 137.3 (Ar), 135.9 (C-7), 130.7 (C-4), 129.3 (C-6), 128.8 (Ar), 128.1 (Ar), 127.2 (Ar). 115.8 (C-2), 61.0 (OMe), 32.0 (NMe); HRMS calcd for C_30_H_34_N_2_O_4_Na [2 M + Na]^+^ 509.2411; found, 509.2435.

#### Substrate Scope of Aromatic Aldehyde

##### (2*E*)-*N*-Methoxy-*N*-methyl-3-phenylprop-2-enamide ((*E*)-**5b**, (*E*)-**20a**)

*R*_*f*_ = 0.36 (silica gel, hexane/ethyl acetate = 2/1); mp: 36.6 °C; FT-IR (KBr) ν_max_: 2939, 1651, 1612, 1574, 1381, 1173, 995, 756, 532 cm^–1^; ^1^H NMR (500 MHz, CDCl_3_, δ): 7.74 (d, *J* = 15.5 Hz, 1H, H-3), 7.63–7.54 (m, 2H, Ar), 7.44–7.33 (m, 3H, Ar), 7.05 (d, *J* = 15.5 Hz, 1H, H-2), 3.78 (s, 3H, OMe), 3.32 (s, 3H, NMe); ^13^C{^1^H} NMR (125 MHz, CDCl_3_): δ 166.9 (C-1), 143.4 (C-3), 135.1 (Ar), 129.8 (Ar), 128.8 (Ar), 128.0 (Ar), 115.7 (C-2), 61.9 (OMe), 32.5 (NMe); HRMS calcd for C_11_H_13_NO_2_Na [M + Na]^+^ 214.0838; found, 214.0840.

##### (2*E*)-*N*-Methoxy-*N*-methyl-3-(2-methylphenyl)-prop-2-enamide ((*E*)-**20b**)

*R*_*f*_ = 0.32 (silica gel, hexane/ethyl acetate = 2/1); FT-IR (neat) ν_max_: 2962, 2939, 1658, 1620, 1481, 1458, 1412, 1381, 1003, 764 cm^–1^; ^1^H NMR (500 MHz, CDCl_3_, δ): 8.02 (d, *J* = 15.5 Hz, 1H, H-3), 7.65–7.58 (m, 1H, Ar), 7.32–7.17 (m, 3H, Ar), 6.95 (d, *J* = 15.5 Hz, 1H, H-2), 3.77 (s, 3H, OMe), 3.32 (s, 3H, NMe), 2.45 (s, 3H, ArMe); ^13^C{^1^H} NMR (125 MHz, CDCl_3_): δ 166.9 (C-1), 141.1 (C-3), 137.6 (Ar), 134.2 (Ar), 130.7 (Ar), 129.5 (Ar), 126.3 (Ar), 126.1 (Ar), 116.9 (C-2), 61.8 (OMe), 32.4 (NMe), 19.8 (ArMe); HRMS calcd for C_24_H_30_N_2_O_4_Na [2 M + Na]^+^ 433.2098; found, 433.2100.

##### (2*E*)-*N*-Methoxy-*N*-methyl-3-(3-methylphenyl)-prop-2-enamide ((*E*)-**20c**)

*R*_*f*_ = 0.52 (silica gel, hexane/ethyl acetate = 1/1); ATR-IR ν_max_: 2965, 2936, 1660, 1622, 1485, 1462, 1423, 1381, 1177, 1100, 997, 787 cm^–1^; ^1^H NMR (500 MHz, CDCl_3_, δ): 7.71 (d, *J* = 16.0 Hz, 1H, H-3), 7.41–7.35 (m, 2H, Ar), 7.31–7.23 (m, 1H, Ar), 7.22–7.15 (m, 1H, Ar), 7.02 (d, *J* = 16.0 Hz, 1H, H-2), 3.77 (s, 3H, OMe), 3.31 (s, 3H, NMe), 2.38 (s, 3H, ArMe); ^13^C{^1^H} NMR (125 MHz, CDCl_3_): δ 167.0 (C-1), 143.6 (C-3), 138.4 (Ar), 135.1 (Ar), 130.6 (Ar), 128.64 (Ar), 128.61 (Ar), 125.2 (Ar), 115.5 (C-2), 61.9 (OMe), 32.5 (NMe), 21.3 (ArMe); HRMS calcd for C_12_H_15_NO_2_Na [M + Na]^+^ 228.0995; found, 228.0997.

##### (2*E*)-*N*-Methoxy-*N*-methyl-3-(4-methylphenyl)-prop-2-enamide ((*E*)-**20d**)

*R*_*f*_ = 0.31 (silica gel, hexane/ethyl acetate = 2/1); mp: 50.2 °C; FT-IR (KBr) ν_max_: 3464, 3433, 2970, 2939, 1651, 1612, 1381, 1180, 987, 810, 494 cm^–1^; ^1^H NMR (500 MHz, CDCl_3_, δ): 7.71 (d, *J* = 15.5 Hz, 1H, H-3), 7.51–7.44 (m, 2H, Ar), 7.23–7.15 (m, 2H, Ar), 7.00 (d, *J* = 15.5 Hz, 1H, H-2), 3.77 (s, 3H, OMe), 3.31 (s, 3H, NMe), 2.38 (s, 3H, ArMe); ^13^C{^1^H} NMR (125 MHz, CDCl_3_): δ 167.1 (C-1), 143.4 (C-3), 140.1 (Ar), 132.4 (Ar), 129.5 (Ar), 128.0 (Ar), 114.6 (C-2), 61.8 (OMe), 32.5 (NMe), 21.4 (ArMe); HRMS calcd for C_24_H_30_N_2_O_4_Na [2 M + Na]^+^ 433.2098; found, 433.2094.

##### (2*E*)-*N*-Methoxy-*N*-methyl-3-(2,6-dimethylphenyl)-prop-2-enamide ((*E*)-**20e**)

*R*_*f*_ = 0.60 (silica gel, hexane/ethyl acetate = 1/1); FT-IR (neat) ν_max_: 2962, 2939, 1658, 1628, 1466, 1412, 1381, 1180, 995, 771 cm^–1^; ^1^H NMR (500 MHz, CDCl_3_, δ): 7.85 (d, *J* = 16.0 Hz, 1H, H-3), 7.17–7.04 (m, 3H, Ar), 6.66 (d, *J* = 16.0 Hz, 1H, H-2), 3.72 (s, 3H, OMe), 3.32 (s, 3H, NMe), 2.36 (s, 6H, ArMe); ^13^C{^1^H} NMR (125 MHz, CDCl_3_): δ 166.7 (C-1), 141.8 (C-3), 136.5 (Ar), 135.0 (Ar), 128.0 (Ar), 127.9 (Ar), 121.7 (C-2), 61.9 (OMe), 32.4 (NMe), 21.0 (ArMe); HRMS calcd for C_26_H_34_N_2_O_4_Na [2 M + Na]^+^ 461.2411; found, 461.2431.

##### (2*E*)-*N*-Methoxy-*N*-methyl-3-(4-bromophenyl)-prop-2-enamide ((*E*)-**20f**)

*R*_*f*_ = 0.45 (silica gel, hexane/ethyl acetate = 1/1); ATR-IR ν_max_: 1661, 1623, 1489, 1417, 1382, 1072, 1010, 818 cm^–1^; ^1^H NMR (500 MHz, CDCl_3_, δ): 7.66 (d, *J* = 15.5 Hz, 1H, H-3), 7.55–7.48 (m, 2H, Ar), 7.47–7.39 (m, 2H, Ar), 7.02 (d, *J* = 15.5 Hz, 1H, H-2), 3.77 (s, 3H, OMe), 3.31 (s, 3H, NMe); ^13^C{^1^H} NMR (125 MHz, CDCl_3_): δ 166.6 (C-1), 142.0 (C-3), 134.0 (Ar), 132.0 (Ar), 129.4 (Ar), 123.9 (Ar), 116.4 (C-2), 61.9 (OMe), 32.5 (NMe); HRMS calcd for C_11_H_12_BrNO_2_Na [M + Na]^+^ 291.9944; found, 291.9932.

##### (2*E*)-*N*-Methoxy-*N*-methyl-3-(4-methoxyphenyl)-prop-2-enamide ((*E*)-**20g**)

*R*_*f*_ = 0.29 (silica gel, hexane/ethyl acetate = 2/1); FT-IR (neat) ν_max_: 2962, 2939, 1651, 1604, 1512, 1381, 1250, 1173, 1034, 1003, 825 cm^–1^; ^1^H NMR (500 MHz, CDCl_3_, δ): 7.70 (d, *J* = 15.5 Hz, 1H, H-3), 7.57–7.50 (m, 2H, Ar), 6.95–6.88 (m, 2H, Ar), 6.92 (d, *J* = 15.5 Hz, 1H, H-2), 3.84 (s, 3H, ArOMe), 3.77 (s, 3H, NOMe), 3.31 (s, 3H, NMe); ^13^C{^1^H} NMR (125 MHz, CDCl_3_): δ 167.3 (C-1), 160.9 (Ar), 143.0 (C-3), 129.6 (Ar), 127.8 (Ar), 114.1 (C-2), 113.2 (Ar), 61.8 (OMe), 55.3 (ArOMe), 32.5 (NMe); HRMS calcd for C_24_H_30_N_2_O_6_Na [2 M + Na]^+^ 465.1996; found, 465.1985.

##### (2*E*)-*N*-Methoxy-*N*-methyl-3-(4-dimethylaminophenyl)-prop-2-enamide ((*E*)-**20h**)

*R*_*f*_ = 0.34 (silica gel, hexane/ethyl acetate = 1/1); FT-IR (neat) ν_max_: 2931, 2900, 1651, 1597, 1527, 1435, 1412, 1365, 1180, 1003, 818 cm^–1^; ^1^H NMR (500 MHz, CDCl_3_, δ): 7.68 (d, *J* = 16.0 Hz, 1H, H-3), 7.52–7.44 (m, 2H, Ar), 6.83 (d, *J* = 16.0 Hz, 1H, H-2), 6.71–6.64 (m, 2H, Ar), 3.76 (s, 3H, OMe), 3.30 (s, 3H, NMe), 3.02 (s, 6H, NMe_2_); ^13^C{^1^H} NMR (125 MHz, CDCl_3_): δ 168.0 (C-1), 151.4 (Ar), 143.9 (C-3), 129.6 (Ar), 123.0 (Ar), 111.7 (C-2), 110.2 (Ar), 61.7 (OMe), 40.1 (NMe_2_), 32.5 (NMe); HRMS calcd for C_26_H_36_N_4_O_4_Na [2 M + Na]^+^ 491.2629; found, 491.2642.

##### (2*E*)-*N*-Methoxy-*N*-methyl-3-(4-nitrophenyl)-prop-2-enamide ((*E*)-**20i**)

*R*_*f*_ = 0.34 (silica gel, hexane/ethyl acetate = 1/1); mp: 154.0 °C; FT-IR (KBr) ν_max_: 3471, 3024, 2939, 1651, 1620, 1589, 1520, 1342, 1173, 995, 849, 748 cm^–1^; ^1^H NMR (500 MHz, CDCl_3_, δ): 8.28–8.23 (m, 2H, Ar), 7.76 (d, *J* = 16.0 Hz, 1H, H-3), 7.74–7.69 (m, 2H, Ar), 7.17 (d, *J* = 16.0 Hz, 1H, H-2), 3.80 (s, 3H, OMe), 3.34 (s, 3H, NMe); ^13^C{^1^H} NMR (125 MHz, CDCl_3_): δ 165.8 (C-1), 148.2 (Ar), 141.3 (Ar), 140.5 (C-3), 128.6 (Ar), 124.1 (Ar), 120.0 (C-2), 62.1 (OMe), 32.5 (NMe); HRMS calcd for C_11_H_12_N_2_O_4_Na [M + Na]^+^ 259.0689; found, 259.0683.

##### (2*E*)-*N*-Methoxy-*N*-methyl-3-(4-trifluoromethylphenyl)-prop-2-enamide ((*E*)-**20j**)

*R*_*f*_ = 0.33 (silica gel, hexane/ethyl acetate = 2/1); mp: 55.8 °C; FT-IR (KBr) ν_max_: 3464, 2939, 1658, 1620, 1327, 1165, 1126, 1065, 995, 833 cm^–1^; ^1^H NMR (500 MHz, CDCl_3_, δ): 7.74 (d, *J* = 16.0 Hz, 1H, H-3), 7.67 (d, *J* = 8.5 Hz, 2H, Ar), 7.64 (d, *J* = 8.5 Hz, 2H, Ar), 7.11 (d, *J* = 16.0 Hz, 1H, H-2), 3.78 (s, 3H, OMe), 3.33 (s, 3H, NMe); ^13^C{^1^H} NMR (125 MHz, CDCl_3_): δ 166.2 (C-1), 141.6 (C-3), 138.6 (Ar), 131.3 (q, ^2^*J*_CF_ = 32.3 Hz, Ar), 128.1 (Ar), 125.7 (q, ^3^*J*_CF_ = 3.6 Hz, Ar), 123.9 (q, ^1^*J*_CF_ = 270.6 Hz, CF_3_), 118.3 (C-2), 62.0 (OMe), 32.5 (NMe); ^19^F{^1^H, ^13^C} NMR (470 MHz, CDCl_3_): −63.2 (CF_3_); HRMS calcd for C_12_H_12_F_3_NO_2_Na [M + Na]^+^ 282.0712; found, 282.0721.

##### (2*E*)-*N*-Methoxy-*N*-methyl-3-(4-cyanophenyl)-prop-2-enamide ((*E*)-**20k**)

*R*_*f*_ = 0.33 (silica gel, hexane/ethyl acetate = 1/1); mp: 151.6 °C; ATR-IR ν_max_: 2978, 2224, 1655, 1618, 1470, 1412, 1383, 1008, 831, 548 cm^–1^; ^1^H NMR (500 MHz, CDCl_3_, δ): 7.72–7.61 (m, 4H, Ar), 7.71 (d, *J* = 15.5 Hz, 1H, H-3), 7.12 (d, *J* = 15.5 Hz, 1H, H-2), 3.78 (s, 3H, OMe), 3.33 (s, 3H, NMe); ^13^C{^1^H} NMR (125 MHz, CDCl_3_): δ 165.9 (C-1), 141.0 (C-3), 139.5 (Ar), 132.5 (Ar), 128.4 (Ar), 119.3 (C-2), 118.5 (CN), 112.9 (Ar), 62.0 (OMe), 32.5 (NMe); HRMS calcd for C_12_H_12_N_2_O_2_Na [M + Na]^+^ 239.0791; found, 239.0790.

##### Methyl (2*E*)-4-(3-(methoxy(methyl)amino)-3-oxoprop-1-en-1-yl)benzoate ((*E*)-20l)

*R*_*f*_ = 0.18 (silica gel, hexane/ethyl acetate = 2/1); mp: 88.8 °C; FT-IR (KBr) ν_max_: 3417, 2947, 1720, 1651, 1612, 1566, 1442, 1412, 1381, 1281, 1173, 1095, 995, 771 cm^–1^; ^1^H NMR (500 MHz, CDCl_3_, δ): 8.12–8.00 (m, 2H, Ar), 7.75 (d, *J* = 16.0 Hz, 1H, H-3), 7.68–7.58 (m, 2H, Ar), 7.12 (d, *J* = 16.0 Hz, 1H, H-2), 3.93 (s, 3H, COOMe), 3.79 (s, 3H, NOMe), 3.32 (s, 3H, NMe); ^13^C{^1^H} NMR (125 MHz, CDCl_3_): δ 166.5 (COOMe), 166.3 (C-1), 142.0 (C-3), 139.4 (Ar), 130.9 (Ar), 130.0 (Ar), 127.8 (Ar), 118.1 (C-2), 62.0 (NOMe), 52.2 (COOMe), 32.5 (NMe); HRMS calcd for C_13_H_15_NO_4_Na [M + Na]^+^ 272.0893; found, 272.0883.

##### (2*E*)-3-(4-Methoxy(methyl)carbamoylphenyl)-*N*-methoxy-*N*-methylprop-2-enamide ((*E*)-**20m**)

*R*_*f*_ = 0.28 (silica gel, hexane/ethyl acetate = 1/2); mp: 79.5 °C; ATR-IR ν_max_: 1652, 1622, 1556, 1455, 1424, 1385, 1208, 1186, 1005, 976, 851 cm^–1^; ^1^H NMR (500 MHz, CDCl_3_, δ): 7.75–7.68 (m, 2H, Ar), 7.74 (d, *J* = 15.5 Hz, 1H, H-3), 7.64–7.57 (m, 2H, Ar), 7.09 (d, *J* = 15.5 Hz, 1H, H-2), 3.78 (s, 3H, 1NOMe), 3.56 (s, 3H, 4′NOMe), 3.37 (s, 3H, 4′NMe), 3.32 (s, 3H, 1NMe); ^13^C{^1^H} NMR (125 MHz, CDCl_3_): δ 169.1 (C-4′), 166.5 (C-1), 142.3 (C-3), 137.2 (Ar), 135.0 (Ar), 128.7 (Ar), 127.5 (Ar), 117.3 (C-2), 61.9 (1NOMe), 61.1 (4′NOMe), 33.5 (4′NMe), 32.5 (1NMe); HRMS calcd for C_14_H_18_N_2_O_4_Na [M + Na]^+^ 301.1159; found, 301.1151.

##### (2*E*)-*N*-Methoxy-*N*-methyl-3-(4-acetylphenyl)-prop-2-enamide ((*E*)-**20n**)

*R*_*f*_ = 0.17 (silica gel, hexane/ethyl acetate = 2/1); FT-IR (neat) ν_max_: 2970, 2939, 1682, 1658, 1620, 1419, 1381, 1365, 1265, 1180, 995, 957, 825 cm^–1^; ^1^H NMR (500 MHz, CDCl_3_, δ): 8.01–7.94 (m, 2H, Ar), 7.75 (d, *J* = 15.5 Hz, 1H, H-3), 7.68–7.62 (m, 2H, Ar), 7.12 (d, *J* = 15.5 Hz, 1H, H-2), 3.79 (s, 3H, OMe), 3.33 (s, 3H, NMe), 2.62 (s, 3H, COCH_3_); ^13^C{^1^H} NMR (125 MHz, CDCl_3_): δ 197.3 (COCH_3_), 166.3 (C-1), 141.9 (C-3), 139.5 (Ar), 137.6 (Ar), 128.8 (Ar), 128.1 (Ar), 118.3 (C-2), 62.0 (OMe), 32.5 (NMe), 26.6 (COCH_3_); HRMS calcd for C_13_H_15_NO_3_Na [M + Na]^+^ 256.0944; found, 256.0932.

##### (2*E*)-4-(3-(Methoxy(methyl)amino)-3-oxoprop-1-en-1-yl)benzoic Acid ((*E*)-**20o**)

*R*_*f*_ = 0.58 (silica gel, chloroform/methanol/acetic acid = 90/10/1); mp: 172.7 °C; ATR-IR ν_max_: 1686, 1655, 1616, 1420, 1388, 1315, 1295, 771 cm^–1^; ^1^H NMR (500 MHz, dimethyl sulfoxide-*d*_6_, δ): 7.96 (d, *J* = 8.0 Hz, 2H, Ar), 7.81 (d, *J* = 8.0 Hz, 2H, Ar), 7.61 (d, *J* = 15.0 Hz, 1H, H-3), 7.20 (d, *J* = 15.0 Hz, 1H, H-2), 3.75 (s, 3H, OMe), 3.22 (s, 3H, NMe); ^13^C{^1^H} NMR (125 MHz, dimethyl sulfoxide-*d*_6_, δ): 167.3 (COOH), 165.5 (C-1), 141.2 (C-3), 138.4 (Ar), 132.8 (Ar), 129.7 (Ar), 128.1 (Ar), 118.5 (C-2), 61.9 (OMe), 32.2 (NMe); HRMS calcd for C_12_H_12_NO_4_Na [M + Na]^+^ 234.0761; found, 234.0756.

##### (2*E*)-*N*-Methoxy-*N*-methyl-3-(4-hydroxyphenyl)-prop-2-enamide ((*E*)-**20p**)

*R*_*f*_ = 0.32 (silica gel, hexane/ethyl acetate = 1/1); mp: 147.5 °C; ATR-IR ν_max_: 3100, 3008, 1645, 1576, 1515, 1282, 830 cm^–1^; ^1^H NMR (500 MHz, CDCl_3_, δ): 7.68 (d, *J* = 16.0 Hz, 1H, H-3), 7.64 (s, 1H, OH), 7.47–7.40 (m, 2H, Ar), 6.92–6.87 (m, 2H, Ar), 6.89 (d, *J* = 16.0 Hz, 1H, H-2), 3.77 (s, 3H, OMe), 3.33 (s, 3H, NMe); ^13^C{^1^H} NMR (125 MHz, CDCl_3_): δ 167.6 (C-1), 158.1 (Ar), 143.8 (C-3), 129.9 (Ar), 127.4 (Ar), 115.9 (Ar), 112.6 (C-2), 61.9 (OMe), 32.6 (NMe); HRMS calcd for C_11_H_13_NO_3_Na [M + Na]^+^ 230.0788; found, 230.0777.

##### (2*E*)-*N*-Methoxy-*N*-methyl-3-(4-boronophenyl)-prop-2-enamide ((*E*)-**20q**)

*R*_*f*_ = 0.55 (silica gel, chloroform/methanol/acetic acid = 90/10/1); mp: 247.1 °C; ATR-IR ν_max_: 3369, 1647, 1606, 1554, 1463, 1409, 1375, 1349, 1047, 829 cm^–1^; ^1^H NMR (500 MHz, acetone-*d*_6_, δ): 7.92 (d, *J* = 8.0 Hz, 2H, Ar), 7.66 (d, *J* = 8.0 Hz, 2H, Ar), 7.64 (d, *J* = 15.0 Hz, 1H, H-3), 7.31–7.25 (m, 2H, B(OH)_2_), 7.20 (d, *J* = 15.0 Hz, 1H, H-2), 3.81 (s, 3H, OMe), 3.24 (s, 3H, NMe); ^13^C{^1^H} NMR (125 MHz, acetone-*d*_6_, δ): 167.1 (C-1), 143.2 (C-3), 137.8 (Ar), 135.4 (Ar), 127.902 (Ar), 117.8 (C-2), 62.3 (OMe), 32.5 (NMe); HRMS calcd for C_11_H_14_BNO_4_Na [M + Na]^+^ 258.0910; found, 258.0908.

##### (2*E*)-*N*-Methoxy-*N*-methyl-3-(4-(4,4,5,5-tetramethyl-1,3,2-dioxaborolan-2-yl)phenyl)-prop-2-enamide ((*E*)-**20r**)

*R*_*f*_ = 0.56 (silica gel, hexane/ethyl acetate = 1/1); mp: 108.5 °C; ATR-IR ν_max_: 2989, 1653, 1623, 1421, 1399, 1383, 1362, 1330, 1147, 1089, 998, 829, 646 cm^–1^; ^1^H NMR (500 MHz, CDCl_3_, δ): 7.86–7.78 (m, 2H, Ar), 7.74 (d, *J* = 16.0 Hz, 1H, H-3), 7.59–7.53 (m, 2H, Ar), 7.08 (d, *J* = 16.0 Hz, 1H, H-2), 3.78 (s, 3H, OMe), 3.32 (s, 3H, NMe), 1.35 (s, 12H, Bpin); ^13^C{^1^H} NMR (125 MHz, CDCl_3_): δ 166.8 (C-1), 143.3 (C-3), 137.6 (Ar), 135.14 (Ar), 135.13 (Ar), 127.2 (Ar), 116.6 (C-2), 83.9 (Bpin), 61,9 (OMe), 32.5 (NMe), 24.8 (Bpin); HRMS calcd for C_17_H_24_BNO_4_Na [M + Na]^+^ 340.1694; found, 340.1684.

##### (2*E*)-*N*-Methoxy-*N*-methyl-3-(pyridin-2-yl)-prop-2-enamide ((*E*)-**20s**)

*R*_*f*_ = 0.53 (silica gel, diethyl ether/methanol = 12/1); ATR-IR ν_max_: 2936, 1661, 1623, 1586, 1471, 1435, 1416, 1384, 993, 783 cm^–1^; ^1^H NMR (500 MHz, CDCl_3_, δ): 8.69–8.63 (m, 1H, Py), 7.75–7.67 (m, 1H, Py), 7.72 (d, *J* = 15.0 Hz, 1H, H-3), 7.56 (d, *J* = 15.0 Hz, 1H, H-2), 7.44–7.38 (m, 1H, Py), 7.28–7.22 (m, 1H, Py), 3.80 (s, 3H, OMe), 3.33 (s, 3H, NMe); ^13^C{^1^H} NMR (125 MHz, CDCl_3_): δ 166.6 (C-1), 153.4 (Py), 150.0 (Py), 141.9 (C-3), 136.7 (Py), 124.8 (Py), 123.9 (Py), 120.0 (C-2), 62.0 (OMe), 32.5 (NMe); HRMS calcd for C_10_H_12_N_2_O_2_Na [M + Na]^+^ 215.0791; found, 215.0788.

##### (2*E*)-*N*-Methoxy-*N*-methyl-3-(pyridin-3-yl)-prop-2-enamide ((*E*)-**20t**)

*R*_*f*_ = 0.71 (silica gel, chloroform/methanol = 9/1); mp: 66.9 °C; FT-IR (KBr) ν_max_: 3464, 3433, 1643, 1612, 1381, 995, 810 cm^–1^; ^1^H NMR (500 MHz, CDCl_3_, δ): 8.83–8.78 (m, 1H, Py), 8.62–8.57 (m, 1H, Py), 7.91–7.85 (m, 1H, Py), 7.72 (d, *J* = 16.0 Hz, 1H, H-3), 7.37–7.31 (m, 1H, Py), 7.12 (d, *J* = 16.0 Hz, 1H, H-2), 3.79 (s, 3H, OMe), 3.33 (s, 3H, NMe); ^13^C{^1^H} NMR (125 MHz, CDCl_3_): δ 166.1 (C-1), 150.5 (Py), 149.5 (Py), 139.7 (C-3), 134.4 (Py), 130.9 (Py), 123.6 (Py), 117.9 (C-2), 62.0 (OMe), 32.5 (NMe); HRMS calcd for C_20_H_24_N_4_O_4_Na [2 M + Na]^+^ 407.1690; found, 407.1685.

##### (2*E*)-*N*-Methoxy-*N*-methyl-3-(pyridin-4-yl)-prop-2-enamide ((*E*)-**20u**)

*R*_*f*_ = 0.70 (silica gel, chloroform/methanol = 9/1); mp: 97.6 °C; FT-IR (KBr) ν_max_: 3433, 2962, 2939, 1651, 1620, 1597, 1550, 1450, 1419, 1381, 1203, 1180, 995, 957, 825, 548, 501 cm^–1^; ^1^H NMR (500 MHz, CDCl_3_, δ): 8.65 (dd, *J* = 6.5, 0.5 Hz, 2H, Py), 7.65 (d, *J* = 16.0 Hz, 1H, H-3), 7.42 (dd, *J* = 6.5, 0.5 Hz, 2H, Py), 7.20 (d, *J* = 16.0 Hz, 1H, H-2), 3.79 (s, 3H, OMe), 3.33 (s, 3H, NMe); ^13^C{^1^H} NMR (125 MHz, CDCl_3_): δ 165.8 (C-1), 150.5 (Py), 142.3 (Py), 140.5 (C-3), 121.8 (Py), 120.3 (C-2), 62.0 (OMe), 32.5 (NMe); HRMS calcd for C_20_H_24_N_4_O_4_Na [2 M + Na]^+^ 407.1690; found, 407.1702.

##### (2*E*)-*N*-Methoxy-*N*-methyl-3-(furan-2-yl)-prop-2-enamide ((*E*)-**20v**)

*R*_*f*_ = 0.45 (silica gel, hexane/ethyl acetate = 1/1); FT-IR (neat) ν_max_: 2939, 1658, 1620, 1481, 1412, 1381, 1003, 980, 748 cm^–1^; ^1^H NMR (500 MHz, CDCl_3_, δ): 7.48 (d, *J* = 15.5 Hz, 1H, H-3), 7.47 (d, *J* = 2.0 Hz, 1H, furan), 6.91 (d, *J* = 15.5 Hz, 1H, H-2), 6.59 (d, *J* = 3.5 Hz, 1H, furan), 6.46 (dd, *J* = 3.5, 2.0 Hz, 1H, furan), 3.76 (s, 3H, OMe), 3.30 (s, 3H, NMe); ^13^C{^1^H} NMR (125 MHz, CDCl_3_): δ 167.0 (C-1), 151.7 (furan), 144.1 (furan), 129.9 (C-3), 114.3 (furan), 113.6 (C-2), 112.2 (furan), 61.9 (OMe), 32.5 (NMe); HRMS calcd for C_9_H_11_NO_3_Na [M + Na]^+^ 204.0631; found, 204.0623.

##### (2*E*)-*N*-Methoxy-*N*-methyl-3-(thiophen-2-yl)-prop-2-enamide ((*E*)-**20w**)

*R*_*f*_ = 0.48 (silica gel, hexane/ethyl acetate = 1/1); FT-IR (neat) ν_max_: 2939, 1651, 1612, 1412, 1381, 1350, 1196, 1003, 972, 825, 710 cm^–1^; ^1^H NMR (500 MHz, CDCl_3_, δ): 7.83 (d, *J* = 15.5 Hz, 1H, H-3), 7.37–7.32 (m, 1H, thiophene), 7.28–7.23 (m, 1H, thiophene), 7.08–7.03 (m, 1H, thiophene), 6.82 (d, *J* = 15.5 Hz, 1H, H-2), 3.76 (s, 3H, OMe), 3.30 (s, 3H, NMe); ^13^C{^1^H} NMR (125 MHz, CDCl_3_): δ 166.7 (C-1), 140.4 (thiophene), 135.9 (C-3), 130.6 (thiophene), 128.0 (thiophene), 127.5 (thiophene), 114.7 (C-2), 61.9 (OMe), 32.5 (NMe); HRMS calcd for C_9_H_11_NO_2_SNa [M + Na]^+^ 220.0403; found, 220.0393.

##### (2*E*)-*N*-Methoxy-*N*-methyl-3-(1*H*-pyrrol-2-yl)-prop-2-enamide ((*E*)-**20x**)

*R*_*f*_ = 0.27 (silica gel, hexane/ethyl acetate = 1/1); FT-IR (neat) ν_max_: 3394, 3255, 1643, 1589, 1550, 1404, 1381, 1034, 1003, 741 cm^–1^; ^1^H NMR (500 MHz, CDCl_3_, δ): 9.10–8.80 (m, 1H, NH), 7.64 (d, *J* = 15.5 Hz, 1H, H-3), 6.95–6.87 (m, 1H, pyrrole), 6.64 (d, *J* = 15.5 Hz, 1H, H-2), 6.62–6.56 (m, 1H, pyrrole), 6.32–6.25 (m, 1H, pyrrole), 3.74 (s, 3H, OMe), 3.30 (s, 3H, NMe); ^13^C{^1^H} NMR (125 MHz, CDCl_3_): δ 167.7 (C-1), 133.3 (C-3), 129.2 (pyrrole), 121.6 (pyrrole), 113.1 (pyrrole), 110.8 (pyrrole), 109.2 (C-2), 61.8 (OMe), 32.6 (NMe); HRMS calcd for C_9_H_12_N_2_O_2_Na [M + Na]^+^ 203.0791; found, 203.0789.

##### (2*E*)-*N*-Methoxy-*N*-methyl-3-(1-methyl-1*H*-pyrrol-2-yl)-prop-2-enamide ((*E*)-**20y**)

*R*_*f*_ = 0.19 (silica gel, hexane/ethyl acetate = 1/1); FT-IR (neat) ν_max_: 3471, 2939, 1643, 1604, 1481, 1404, 1373, 1180, 1095, 1057, 1003, 972, 733 cm^–1^; ^1^H NMR (500 MHz, CDCl_3_, δ): 7.65 (d, *J* = 15.5 Hz, 1H, H-3), 6.76–6.66 (m, 2H, pyrrole), 6.75 (d, *J* = 15.5 Hz, 1H, H-2), 6.21–6.14 (m, 1H, pyrrole), 3.74 (s, 3H, OMe), 3.72 (s, 3H, NMe), 3.29 (s, 3H, CONMe); ^13^C{^1^H} NMR (125 MHz, CDCl_3_): δ 167.8 (C-1), 131.1 (C-3), 130.1 (pyrrole), 126.2 (pyrrole), 110.8 (pyrrole), 110.7 (C-2), 109.0 (pyrrole), 61.7 (OMe), 34.3 (NMe), 32.5 (CONMe); HRMS calcd for C_10_H_14_N_2_O_2_Na [M + Na]^+^ 217.0947; found, 217.0947.

##### (2*E*)-*N*-Methoxy-*N*-methyl-3-(1*H*-indol-3-yl)-prop-2-enamide ((*E*)-**20z**)

*R*_*f*_ = 0.18 (silica gel, hexane/ethyl acetate = 1/1); mp: 152.8 °C; ATR-IR ν_max_: 3157, 2970, 2927, 1634, 1570, 1417, 1386, 1246, 745 cm^–1^; ^1^H NMR (500 MHz, CDCl_3_, δ): 9.08–8.91 (m, 1H, NH), 7.97 (d, *J* = 16.0 Hz, 1H, H-3), 7.96–7.89 (m, 1H, indole), 7.52–7.46 (m, 1H, indole), 7.46–7.39 (m, 1H, indole), 7.31–7.20 (m, 2H, indole), 7.07 (d, *J* = 16.0 Hz, 1H, H-2), 3.82 (s, 3H, OMe), 3.34 (s, 3H, NMe); ^13^C{^1^H} NMR (125 MHz, CDCl_3_): δ 168.5 (C-1), 137.2 (indole), 137.1 (C-3), 128.9 (indole), 125.4 (indole), 123.1 (indole), 121.2 (indole), 120.4 (indole), 114.0 (indole), 111.9 (indole), 111.0 (C-2), 61.7 (OMe), 32.6 (NMe); HRMS calcd for C_13_H_14_N_2_O_2_Na [M + Na]^+^ 253.0947; found, 253.0937.

##### (2*E*)-*N*-Methoxy-*N*-methyl-3-(naphthalen-1-yl)-prop-2-enamide ((*E*)-**20aa**)

*R*_*f*_ = 0.53 (silica gel, hexane/ethyl acetate = 1/1); mp: 68.7 °C; FT-IR (KBr) ν_max_: 3464, 3433, 3402, 1643, 1612, 1419, 1381, 1173, 995, 795, 771 cm^–1^; ^1^H NMR (500 MHz, CDCl_3_, δ): 8.57 (d, *J* = 15.5 Hz, 1H, H-3), 8.30–8.20 (m, 1H, naphthalene), 7.94–7.84 (m, 2H, naphthalene), 7.82–7.74 (m, 1H, naphthalene), 7.63–7.44 (m, 3H, naphthalene), 7.01 (d, *J* = 15.5 Hz, 1H, H-2), 3.79 (s, 3H, OMe), 3.36 (s, 3H, NMe); ^13^C{^1^H} NMR (125 MHz, CDCl_3_): δ 166.8 (C-1), 140.5 (C-3), 133.6 (naphthalene), 132.7 (naphthalene), 131.5 (naphthalene), 130.0 (naphthalene), 128.6 (naphthalene), 126.7 (naphthalene), 126.1 (naphthalene), 125.3 (naphthalene), 124.7 (naphthalene), 123.7 (naphthalene), 118.7 (C-2), 61.9 (OMe), 32.5 (NMe); HRMS calcd for C_15_H_15_NO_2_Na [M + Na]^+^ 264.0995; found, 264.1007.

##### (2*E*)-*N*-Methoxy-*N*-methyl-3-(anthracen-9-yl)-prop-2-enamide ((*E*)-**20ab**)

*R*_*f*_ = 0.56 (silica gel, hexane/ethyl acetate = 1/1); FT-IR (neat) ν_max_: 3055, 2970, 2939, 1658, 1628, 1442, 1419, 1381, 1180, 995, 887, 779, 741, 702 cm^–1^; ^1^H NMR (500 MHz, CDCl_3_, δ): 8.66 (d, *J* = 16.0 Hz, 1H, H-3), 8.47–8.41 (m, 1H, anthracene), 8.33–8.24 (m, 2H, anthracene), 8.06–7.95 (m, 2H, anthracene), 7.54–7.44 (m, 4H, anthracene), 7.00 (d, *J* = 16.0 Hz, 1H, H-2), 3.72 (s, 3H, OMe), 3.40 (s, 3H, NMe); ^13^C{^1^H} NMR (125 MHz, CDCl_3_): δ 166.4 (C-1), 140.6 (C-3), 131.3 (anthracene), 130.5 (anthracene), 129.4 (anthracene), 128.7 (anthracene), 127.7 (anthracene), 126.0 (anthracene), 125.5 (anthracene), 125.3 (anthracene), 125.1 (C-2), 62.0 (OMe), 32.6 (NMe); HRMS calcd for C_19_H_17_NO_2_Na [M + Na]^+^ 314.1151; found, 314.1155.

##### (2*E*)-*N*-Methoxy-*N*-methyl-3-(2-formylphenyl)-prop-2-enamide ((*E*)-**20ac**)

*R*_*f*_ = 0.45 (silica gel, hexane/ethyl acetate = 1/2); FT-IR (neat) ν_max_: 3479, 3456, 2939, 1697, 1651, 1620, 1481, 1412, 1381, 1188, 995, 764 cm^–1^; ^1^H NMR (500 MHz, CDCl_3_, δ): 10.38 (s, 1H CHO), 8.47 (d, *J* = 16.0 Hz, 1H, H-3), 7.95–7.87 (m, 1H, Ar), 7.70–7.58 (m, 2H, Ar), 7.57–7.50 (m, 1H, Ar), 6.95 (d, *J* = 16.0 Hz, 1H, H-2), 3.77 (s, 3H, OMe), 3.33 (s, 3H, NMe); ^13^C{^1^H} NMR (125 MHz, CDCl_3_): δ 191.3 (CHO), 166.0 (C-1), 139.3 (C-3), 137.9 (Ar), 134.0 (Ar), 133.8 (Ar), 130.8 (Ar), 129.5 (Ar), 128.1 (Ar), 121.4 (C-2), 62.0 (OMe), 32.5 (NMe); HRMS calcd for C_12_H_13_NO_3_Na [M + Na]^+^ 242.0788; found, 242.0781.

##### (2*E*,2′*E*)-3,3′-(1,2-Phenylene)bis(*N*-methoxy-*N*-methyl-prop-2-enamide) ((*E*,*E*)-**20ac**)

*R*_*f*_ = 0.18 (silica gel, hexane/ethyl acetate = 1/2); FT-IR (neat) ν_max_: 3471, 2970, 2939, 1651, 1612, 1473, 1419, 1381, 1180, 1103, 995, 980, 764 cm^–1^; ^1^H NMR (500 MHz, CDCl_3_, δ): 8.05 (d, *J* = 16.0 Hz, 2H, H-3, H-3′), 7.63–7.54 (m, 2H, Ar), 7.43–7.34 (m, 2H, Ar), 6.91 (d, *J* = 16.0 Hz, 2H, H-2, H-2′), 3.77 (s, 6H, OMe), 3.31 (s, 6H, NMe); ^13^C{^1^H} NMR (125 MHz, CDCl_3_): δ 166.4 (C-1, C-1′), 140.9 (C-3, C-3′), 135.3 (Ar), 129.3 (Ar), 128.2 (Ar), 119.9 (C-2, C-2′), 62.0 (OMe), 32.5 (NMe); HRMS calcd for C_16_H_20_N_2_O_4_Na [M + Na]^+^ 327.1315; found, 327.1312.

##### (2*E*,2′*E*)-3,3′-(1,3-Phenylene)bis(*N*-methoxy-*N*-methyl-prop-2-enamide) ((*E*,*E*)-**20ad**)

*R*_*f*_ = 0.23 (silica gel, hexane/ethyl acetate = 1/2); mp: 86.5 °C; ATR-IR ν_max_: 2938, 1662, 1647, 1623, 1465, 1441, 1413, 1374, 1179, 1096, 994, 794 cm^–1^; ^1^H NMR (500 MHz, CDCl_3_, δ): 7.75 (d, *J* = 16.0 Hz, 2H, H-3, H-3′), 7.74–7.69 (m, 1H, Ar), 7.63–7.54 (m, 2H, Ar), 7.45–7.38 (m, 1H, Ar), 7.07 (d, *J* = 16.0 Hz, 2H, H-2, H-2′), 3.79 (s, 6H, OMe), 3.33 (s, 6H, NMe); ^13^C{^1^H} NMR (125 MHz, CDCl_3_): δ 166.7 (C-1, C-1′), 142.7 (C-3, C-3′), 135.8 (Ar), 129.2 (Ar), 129.0 (Ar), 127.8 (Ar), 116.6 (C-2, C-2′), 61.9 (OMe), 32.5 (NMe); HRMS calcd for C_16_H_20_N_2_O_4_Na [M + Na]^+^ 327.1315; found, 327.1314.

##### (2*E*,2′*E*)-3,3′-(1,4-Phenylene)bis(*N*-methoxy-*N*-methyl-prop-2-enamide) ((*E*,*E*)-**20ae**)

*R*_*f*_ = 0.22 (silica gel, hexane/ethyl acetate = 1/2); mp: 183.6 °C; FT-IR (KBr) ν_max_: 3433, 2978, 2939, 1643, 1604, 1458, 1427, 1381, 1196, 1180, 995, 825 cm^–1^; ^1^H NMR (500 MHz, CDCl_3_, δ): 7.73 (d, *J* = 16.0 Hz, 2H, H-3, H-3′), 7.59 (s, 4H, Ar), 7.07 (d, *J* = 16.0 Hz, 2H, H-2, H-2′), 3.78 (s, 6H, OMe), 3.32 (s, 6H, NMe); ^13^C{^1^H} NMR (125 MHz, CDCl_3_): δ 166.7 (C-1, C-1′), 142.4 (C-3, C-3′), 136.5 (Ar), 128.4 (Ar), 116.6 (C-2, C-2′), 61.9 (OMe), 32.5 (NMe); HRMS calcd for C_16_H_20_N_2_O_4_Na [M + Na]^+^ 327.1315; found, 327.1308.

##### (2*E*,2′*E*,2″*E*)-3,3′,3″-(Benzene-1,3,5-triyl)tris(*N*-methoxy-*N*-methyl-prop-2-enamide) ((*E*,*E*,*E*)-**20af**)

*R*_*f*_ = 0.18 (silica gel, ethyl acetate); mp: 213.6 °C; ATR-IR ν_max_: 2964, 1654, 1615, 1472, 1448, 1416, 1386, 1181, 1000 cm^–1^; ^1^H NMR (500 MHz, CDCl_3_, δ): 7.76 (d, *J* = 16.0 Hz, 3H, H-3, H-3′, H-3″), 7.72 (s, 3H, Ar), 7.00 (d, *J* = 16.0 Hz, 3H, H-2, H-2′, H-2″), 3.81 (s, 9H, OMe), 3.34 (s, 9H, NMe); ^13^C{^1^H} NMR (125 MHz, CDCl_3_): δ 166.4 (C-1, C-1′, C-1″), 142.1 (C-3, C-3′, C-3″), 136.4 (Ar), 128.3 (Ar), 117.4 (C-2, C-2′, C-2″), 62.0 (OMe), 32.5 (NMe); HRMS calcd for C_21_H_27_N_3_O_6_Na [M + Na]^+^ 440.1792; found, 440.1794.

#### Gram-Scale Reaction of **17d**

To a solution of phosphate **1** (9.57 g, 40.0 mmol) in THF (80 mL), a 2.0 M solution of isopropylmagnesium chloride in THF (18.0 mL, 36.0 mmol) was added at −78 °C. After the reaction mixture was stirred at −78 °C for 30 min, a solution of *trans*-cinnamaldehyde **17d** (2.64 g, 20.0 mmol) in THF (20 mL) was added at room temperature, and the reaction mixture was stirred for 21 h. To the reaction mixture, saturated aqueous ammonium chloride was added at 0 °C, and the mixture was extracted with ethyl acetate. The organic layer was dried over sodium sulfate. After filtration of the mixture and concentration of the solvent, the crude mixture was purified by flash column chromatography (eluant: hexane/ethyl acetate = 4/1 to 1/1) to afford alkene (*E*)-**18d** (4.03 g, 93%) as white solid and alkene (*Z*)-**18d** (131 mg, 3.0%) as white solid.

#### Reduction in the Successive Elongation Process

To a solution of Weinreb amide in THF, a 1.0 M solution of diisobutylaluminum hydride in hexane was added at −78 °C, and the reaction mixture was stirred at −78 °C for Time. Since Weinreb amide remained, a 1.0 M solution of diisobutylaluminum hydride in hexane was added to the reaction mixture. After the reaction mixture stirred at −78 °C for Time, methanol and saturated aqueous Rochelle salt were added, and the mixture was stirred at room temperature. The mixture was extracted with ethyl acetate, and the organic layer was dried over sodium sulfate. After filtration of the mixture and concentration of the solvent, α,β-unsaturated aldehyde was obtained as a colorless oil and used in the next HWE reaction without further purification.

#### *E*-Selective HWE Reaction in the Successive Elongation Process

To a solution of phosphate **1** (2.0 equiv: 95.7 mg, 0.400 mmol) in THF (4.7 mL), a 2.0 M solution of isopropylmagnesium chloride in THF (1.8 equiv: 0.18 mL, 0.360 mmol) was added at −78 °C. After the reaction mixture was stirred at −78 °C for 30 min, a solution of α,β-unsaturated aldehyde in THF (2.0 mL) was added at room temperature, and the reaction mixture was stirred for Time. To the reaction mixture, saturated aqueous ammonium chloride was added at 0 °C, and the mixture was extracted with ethyl acetate. The organic layer was dried over sodium sulfate. After filtration of the mixture and concentration of the solvent, the crude mixture was purified by thin layer chromatography on silica (eluant; hexane/ethyl acetate = 1/1) to afford diene.

#### *Z*-Selective HWE Reaction in the Successive Elongation Process

To a solution of phosphate **21** (2.0 equiv: 139 mg, 0.400 mmol) and 1,4,7,10,13,16-hexaoxacyclooctadecane (3.6 equiv: 190 mg, 0.720 mmol) in THF (4.7 mL), a 1.0 M solution of potassium bis(trimethylsilyl)amide in THF (1.8 equiv: 0.36 mL, 0.360 mmol) was added at −78 °C. After the reaction mixture was stirred at −78 °C for 30 min, a solution of α,β-unsaturated aldehyde in THF (2.0 mL) was added at −78 °C, and the reaction mixture was stirred for Time. To the reaction mixture, saturated aqueous ammonium chloride was added at −78 °C, and the mixture was extracted with ethyl acetate. The organic layer was dried over sodium sulfate. After filtration of the mixture and concentration of the solvent, the crude mixture was purified by thin layer chromatography on silica (eluant; hexane/ethyl acetate = 1/1) to afford diene.

#### (2*Z*)-*N*-Methoxy-*N*-methyl-3-phenylprop-2-enamide ((*Z*)-**20a**)

*R*_*f*_ = 0.36 (silica gel, hexane/ethyl acetate = 2/1); FT-IR (neat) ν_max_: 2970, 2931, 1651, 1496, 1427, 1358, 1180, 1103, 1003, 787, 702 cm^–1^; ^1^H NMR (500 MHz, CDCl_3_, δ): 7.65–7.40 (m, 2H, Ar), 7.40–7.23 (m, 3H, Ar), 6.77 (d, *J* = 12.5 Hz, 1H, H-3), 6.28 (brd, *J* = 12.5 Hz, 1H, H-2), 3.65 (br s, 3H, OMe), 3.26 (br s, 3H, NMe); ^13^C{^1^H} NMR (125 MHz, CDCl_3_): δ 168.1 (C-1), 137.7 (C-3), 135.2 (Ar), 129.1 (Ar), 128.6 (Ar), 128.2 (Ar), 120.6 (C-2), 61.7 (OMe), 32.2 (NMe); HRMS calcd for C_11_H_13_NO_2_Na [M + Na]^+^ 214.0838; found, 214.0842.

##### (2*E*,4*Z*)-*N*-Methoxy-*N*-methyl-5-phenylpenta-2,4-dienamide ((2*E*,4*Z*)-**18d**)

*R*_*f*_ = 0.30 (silica gel, hexane/ethyl acetate = 2/1); FT-IR (neat) ν_max_: 2962, 2939, 1651, 1612, 1450, 1419, 1381, 1180, 1003, 864, 810, 702 cm^–1^; ^1^H NMR (500 MHz, C_6_D_6_, δ): 8.36 (ddd, *J* = 15.0, 11.5, 1.0 Hz, 1H, H-3), 7.29–7.23 (m, 2H, Ar), 7.05–6.93 (m, 3H, Ar), 6.69 (d, *J* = 15.0 Hz, 1H, H-2), 6.50 (dd, *J* = 11.5, 1.0 Hz, 1H, H-5), 6.24 (dd, *J* = 11.5, 11.5 Hz, 1H, H-4), 3.06 (s, 3H, OMe), 2.94 (s, 3H, NMe); ^13^C{^1^H} NMR (125 MHz, C_6_D_6_): δ 167.0 (C-1), 139.1 (C-3), 137.0 (C-5), 136.8 (Ar), 129.5 (Ar), 128.7 (Ar), 128.2 (Ar), 128.1 (C-4), 122.6 (C-2), 61.1 (OMe), 32.2 (NMe); HRMS calcd for C_13_H_15_NO_2_Na [M + Na]^+^ 240.0995; found, 240.0987.

##### (2*Z*,4*Z*)-*N*-Methoxy-*N*-methyl-5-phenylpenta-2,4-dienamide ((2*Z*,4*Z*)-**18d**)

*R*_*f*_ = 0.45 (silica gel, hexane/ethyl acetate = 2/1); FT-IR (neat) ν_max_: 2962, 2931, 1643, 1450, 1435, 1381, 1095, 802, 702 cm^–1^; ^1^H NMR (500 MHz, C_6_D_6_, δ): 8.19 (dd, *J* = 11.5, 11.5 Hz, 1H, H-4), 7.22–7.12 (m, 2H, Ar), 7.12–7.06 (m, 2H, Ar), 7.06–6.99 (m, 1H, Ar), 7.00 (dd, *J* = 11.5, 11.5 Hz, 1H, H-3), 6.63 (d, *J* = 11.5 Hz, 1H, H-5), 6.21 (d, *J* = 11.5 Hz, 1H, H-2), 3.03 (s, 3H, OMe), 2.93 (s, 3H, NMe); ^13^C{^1^H} NMR (125 MHz, C_6_D_6_): δ 167.2 (C-1), 138.1 (C-3), 137.1 (C-5), 136.7 (Ar), 129.8 (Ar), 128.4 (Ar), 127.7 (Ar), 127.2 (C-4), 118.4 (C-2), 60.9 (OMe), 31.9 (NMe); HRMS calcd for C_13_H_15_NO_2_Na [M + Na]^+^ 240.0995; found, 240.0995.

#### Synthesis of a Successive Biscyclopropane

To a solution of trimethylsulfoxonium iodide (2.0 equiv) in dimethyl sulfoxide, sodium hydride (55% dispersion in mineral oil) (2.0 equiv) was added at 0 °C. After the mixture was stirred at 0 °C for 30 min, a solution of alkene (1.0 equiv) in dimethyl sulfoxide was added to the mixture at 0 °C. The reaction mixture was stirred at room temperature for Time. To the reaction mixture, saturated aqueous ammonium chloride was added at 0 °C, and the mixture was extracted with ethyl acetate. The organic layer was dried over sodium sulfate. After filtration of the mixture and concentration of the solvent, the crude mixture was purified by thin layer chromatography on silica (eluant; hexane/ethyl acetate = 1/1) to afford cyclopropane.

##### (1*SR*,2*SR*)-*N*-Methoxy-*N*-methyl-2-phenylcyclopropane-1-carboxamide (*trans*-**22**)

*R*_*f*_ = 0.39 (silica gel, hexane/ethyl acetate = 2/1); ATR-IR ν_max_: 2936, 1653, 1462, 1441, 1422, 1369, 995, 750, 699 cm^–1^; ^1^H NMR (500 MHz, CDCl_3_, δ): 7.32–7.24 (m, 2H, Ar), 7.23–7.17 (m, 1H, Ar), 7.16–7.11 (m, 2H, Ar), 3.69 (s, 3H, OMe), 3.24 (s, 3H, NMe), 2.51 (ddd, *J* = 9.5, 6.5, 4.0 Hz, 1H, H-3), 2.47–2.35 (m, 1H, H-2), 1.63 (ddd, *J* = 9.5, 5.5, 4.0 Hz, 1H, CH_2_), 1.31 (ddd, *J* = 8.5, 6.5, 4.0 Hz, 1H, CH_2_); ^13^C{^1^H} NMR (125 MHz, CDCl_3_): δ 173.0 (C-1), 140.7 (Ar), 128.4 (Ar), 126.21 (Ar), 126.16 (Ar), 61.6 (OMe), 32.5 (NMe), 25.8 (C-3), 21.5 (C-2), 16.4 (CH_2_); HRMS calcd for C_12_H_15_NO_2_Na [M + Na]^+^ 228.0995; found, 228.0986.

#### (1*RS*,1′*RS*,2*SR***,**2′*SR*)-*N***-**Methoxy-*N*-methyl-2′-phenyl-[1,1′-bi(cyclopropane)]-2-carboxamide (*trans*-*syn*-*trans*-**23**). (1*SR***,**1′*RS*,2*RS***,**2′*SR*)-*N***-**Methoxy-*N*-methyl-2′-phenyl-[1,1′-bi(cyclopropane)]-2-carboxamide (*trans*-*anti*-*trans*-**23**)

##### Mixture of *trans*-*syn*-*trans*-**23** and *trans*-*anti*-*trans*-**23**

*R*_*f*_ = 0.33 (silica gel, hexane/ethyl acetate = 2/1); ATR-IR ν_max_: 3003, 1654, 1498, 1464, 1422, 1390, 700 cm^–1^.

##### *trans*-*syn*-trans-**23** or *trans*-*anti*-*trans*-**23**

^1^H NMR (500 MHz, CDCl_3_, δ): 7.29–7.20 (m, 2H, Ar), 7.17–7.11 (m, 1H, Ar), 7.07–7.00 (m, 2H, Ar), 3.76 (s, 3H, OMe), 3.209 (s, 3H, NMe), 2.15–1.95 (m, 1H, H-2), 1.82–1.71 (m, 1H, H-2′), 1.58–1.44 (m, 1H, H-1), 1.24–1.05 (m, 2H, H-3, H-1′), 0.97–0.73 (m, 3H, H-3, H-3′); ^13^C{^1^H} NMR (125 MHz, CDCl_3_): δ 173.92 (CO), 142.9 (Ar), 128.28 (Ar), 125.7 (Ar), 125.48 (Ar), 61.6 (OMe), 32.6 (NMe), 24.30 (C-1′), 24.2 (C-1), 22.5 (C-2′), 16.9 (C-2), 14.7 (C-3′), 13.73 (C-3).

##### *trans*-*syn*-trans-**23** or *trans*-*anti*-*trans*-**23**

^1^H NMR (500 MHz, CDCl_3_, δ): 7.29–7.20 (m, 2H, Ar), 7.17–7.11 (m, 1H, Ar), 7.07–7.00 (m, 2H, Ar), 3.75 (s, 3H, OMe), 3.209 (s, 3H, NMe), 2.15–1.95 (m, 1H, H-2), 1.82–1.71 (m, 1H, H-2′), 1.58–1.44 (m, 1H, H-1), 1.24–1.05 (m, 2H, H-3, H-1′), 0.97–0.73 (m, 3H, H-3, H-3′); ^13^C{^1^H} NMR (125 MHz, CDCl_3_): δ 173.87 (CO), 142.8 (Ar), 128.27 (Ar), 125.6 (Ar), 125.46 (Ar), 61.5 (OMe), 32.5 (NMe), 24.29 (C-1′), 24.1 (C-1), 21.8 (C-2′), 16.5 (C-2), 13.74 (C-3′), 12.9 (C-3).

##### Mixture of *trans*-*syn*-*trans*-**23** and *trans*-*anti*-*trans*-**23**

HRMS calcd for C_15_H_19_NO_2_Na [M + Na]^+^ 268.1308; found, 268.1295.

#### HWE Reaction of Cyclohexanone (**24**)

To a solution of phosphate **1** (2.0 equiv: 95.7 mg, 0.400 mmol) in THF (4.7 mL), a 2.0 M solution of isopropylmagnesium chloride in THF (1.8 equiv: 0.18 mL, 0.360 mmol) was added at −78 °C. After the reaction mixture was stirred at −78 °C for 30 min, a solution of cyclohexanone **24** (1.0 equiv: 19.5 mg, 0.199 mmol) in THF (2.0 mL) was added at room temperature, and the reaction mixture was stirred for 24 h. To the reaction mixture, saturated aqueous ammonium chloride was added at 0 °C, and the mixture was extracted with ethyl acetate. The organic layer was dried over sodium sulfate. After filtration of the mixture and concentration of the solvent, the crude mixture was purified by thin layer chromatography on silica (eluant; hexane/ethyl acetate = 3/1) to afford alkene **25** (33.5 mg, 92%) as a colorless oil.

##### 2-Cyclohexylidene-*N*-methoxy-*N*-methylacetamide (**25**)

*R*_*f*_ = 0.52 (silica gel, hexane/ethyl acetate = 2/1); FT-IR (neat) ν_max_: 2931, 2854, 1651, 1450, 1412, 1389, 1342, 1180, 1011, 987 cm^–1^; ^1^H NMR (500 MHz, CDCl_3_, δ): 6.02 (s, 1H, H-2), 3.68 (s, 3H, OMe), 3.20 (s, 3H, NMe), 2.76 (t, *J* = 5.5 Hz, 2H, CH_2_(CH_2_)_3_CH_2_ or CH_2_(CH_2_)_3_CH_2_), 2.22 (t, *J* = 5.5 Hz, 2H, CH_2_(CH_2_)_3_CH_2_ or CH_2_(CH_2_)_3_CH_2_), 1.72–1.53 (m, 6H, CH_2_CH_2_CH_2_CH_2_CH_2_); ^13^C{^1^H} NMR (125 MHz, CDCl_3_): δ 168.1 (C-1), 159.9 (C-3), 111.5 (C-2), 61.3 (OMe), 38.2 (CH_2_(CH_2_)_3_CH_2_ or CH_2_(CH_2_)_3_CH_2_), 32.1 (NMe), 30.0 (CH_2_(CH_2_)_3_CH_2_ or CH_2_(CH_2_)_3_CH_2_), 28.7 (CH_2_CH_2_(CH_2_)_3_ or (CH_2_)_3_CH_2_CH_2_), 27.8 (CH_2_CH_2_(CH_2_)_3_ or (CH_2_)_3_CH_2_CH_2_), 26.3 ((CH_2_)_2_CH_2_(CH_2_)_2_); HRMS calcd for C_20_H_34_N_2_O_4_Na [2 M + Na]^+^ 389.2411; found, 389.2420.

#### Weinreb Ketone Synthesis

To a solution of Weinreb amide *syn*-**16p** (or *anti*-**16q**) (1.0 equiv: 0.100 mmol) in THF (2.0 mL), a 1.04 M solution of methylmagnesium bromide in THF (0.19 mL, 0.200 mmol) was added at −78 °C. After the rection mixture was stirred at −78 °C for 10 min, the reaction mixture was stirred at 0 °C for 30 min. To the reaction mixture, saturated aqueous ammonium chloride was added at 0 °C, and the mixture was extracted with ethyl acetate. The organic layer was dried over sodium sulfate. After filtration of the mixture and concentration of the solvent, the crude mixture was purified by thin layer chromatography on silica (eluant; hexane/ethyl acetate = 6/1) to afford ketone.

##### (5*RS*,6*SR*,3*E*)-6-((*tert*-Butyldimethylsilyl)oxy)-5-methyl-6-phenylhex-3-en-2-one (*syn*-**26**)

*R*_*f*_ = 0.96 (silica gel, hexane/ethyl acetate = 2/1); FT-IR (neat) ν_max_: 2954, 2931, 2885, 2854, 1674, 1365, 1257, 1088, 1065, 864, 841, 779, 702 cm^–1^; ^1^H NMR (500 MHz, CDCl_3_, δ): 7.34–7.19 (m, 5H, Ar), 6.73 (dd, *J* = 16.0, 7.5 Hz, 1H, H-3), 5.96 (dd, *J* = 16.0, 1.0 Hz, 1H, H-2), 4.61 (dd, *J* = 6.5, 0.5 Hz, 1H, H-5), 2.60 (dqdd, *J* = 7.5, 7.0, 6.5, 1.0 Hz, 1H, H-4), 2.19 (s, 3H, COCH_3_), 1.02 (d, *J* = 7.0 Hz, 3H, 4-Me), 0.90 (s, 9H, TBS), 0.02 (s, 3H, TBS), −0.20 (s, 3H, TBS); ^13^C{^1^H} NMR (125 MHz, CDCl_3_): δ 198.7 (C-1), 150.6 (C-3), 142.7 (Ar), 131.0 (C-2), 127.9 (Ar), 127.3 (Ar), 126.4 (Ar), 77.8 (C-5), 45.4 (C-4), 26.6 (COCH_3_), 25.8 (TBS), 18.2 (TBS), 13.9 (4-Me), −4.6 (TBS), −5.2 (TBS); HRMS calcd for C_19_H_30_O_2_SiNa [M + Na]^+^ 341.1907; found, 341.1905.

##### (5*SR*,6*SR*,3*E*)-6-((*tert*-Butyldimethylsilyl)oxy)-5-methyl-6-phenylhex-3-en-2-one (*anti*-**26**)

*R*_*f*_ = 0.94 (silica gel, hexane/ethyl acetate = 2/1); FT-IR (neat) ν_max_: 2954, 2931, 2893, 2854, 1674, 1365, 1257, 1088, 1065, 841, 779, 702 cm^–1^; ^1^H NMR (500 MHz, CDCl_3_, δ): 7.34–7.21 (m, 5H, Ar), 6.86 (dd, *J* = 16.0, 8.0 Hz, 1H, H-3), 5.97 (dd, *J* = 16.0, 1.0 Hz, 1H, H-2), 4.48 (d, *J* = 6.0 Hz, 1H, H-5), 2.59 (dqdd, *J* = 8.0, 7.0, 6.0, 1.0 Hz, 1H, H-4), 2.23 (s, 3H, COC*H*_*3*_), 0.94 (d, *J* = 7.0 Hz, 3H, 4-Me), 0.87 (s, 9H, TBS), 0.01 (s, 3H, TBS), −0.24 (s, 3H, TBS); ^13^C{^1^H} NMR (125 MHz, CDCl_3_): δ 198.8 (C-1), 150.6 (C-3), 143.0 (Ar), 131.3 (C-2), 128.0 (Ar), 127.4 (Ar), 126.6 (Ar), 78.8 (C-5), 45.6 (C-4), 26.0 (CO*C*H_3_), 25.7 (TBS), 18.1 (TBS), 16.0 (4-Me), −4.6 (TBS), −5.1 (TBS); HRMS calcd for C_19_H_30_O_2_SiNa [M + Na]^+^ 341.1907; found, 341.1908.

## Data Availability

The data underlying this study are available in the published article and its Supporting Information.

## Supporting Information Available

Experimental procedures, compounds data, NMR spectra (PDF)
