# Supplementary material for: (E)-Selective Weinreb Amide-Type Horner–Wadsworth–Emmons Reaction: Effect of Reaction Conditions, Substrate Scope, Isolation of a Reactive Magnesium Phosphonoenolate, and Applications
Source: J Org Chem. 2024 Oct 11;89(21):15414–35. doi: 10.1021/acs.joc.4c01140 (PMC11536377; doi:10.1021/acs.joc.4c01140)
Supplement: Supplementary file 1 — jo4c01140_si_001.pdf [file jo4c01140_si_001.pdf]

(E)-Selective Weinreb Amide-Type Horner–Wadsworth–Emmons Reaction: Effect of Reaction Conditions, Substrate Scope, Isolation of a Reactive Magnesium Phosphonoenolate, and Applications

Takatsugu Murata,‡* Hisazumi Tsutsui,‡ Isamu Shiina*

Department of Applied Chemistry, Faculty of Science, Tokyo University of Science, 1-3 Kagurazaka, Shinjuku-ku, Tokyo 162-8601, Japan

‡: The authors contribute equally.

Supporting Information

S1	General Information
S1–6	Reagents
S6–10	Preparation of HWE Reagents
S10–19	Preparation of Substrates
S19–73	General Procedures
S73–74	References
S75–368	^{1}H NMR, ^{13}C NMR, ^{31}P NMR, ^{19}F NMR, and 2D NMR Spectroscopic Data of Compounds

General Information. Melting points are recorded on a Yanaco MP-S3. ^{1}H, ^{13}C, ^{31}P and ^{19}F NMR spectra were recorded on a JEOL JNM-ECA500II or a Bruker Biospin AVANCE 400M with chloroform (in chloroform-*d*), with benzene (in benzene-d_6), with acetone (in acetone-d_6) or with dimethyl sulfoxide (in dimethyl sulfoxide-d_6) as the internal standard. ^{31}P NMR spectra were recorded with triphenyl phosphine as the internal standard and ^{19}F NMR were recorded with trifluoromethylbenzene as the internal standard. Structural assignments were made with additional information from gHMQC and gHMBC experiments. Infrared spectra were recorded on a Horiba FT-300 (FT-IR) or Jasco FT/IR-4600 (ATR-IR). Absorbance frequencies are recorded in reciprocal centimeters (cm^{-1}). High resolution mass spectra (HRMS) were obtained from a Bruker Daltonics micro TOF focus. Optical rotations were determined using a Jasco P-1020. All reactions were carried out under argon atmosphere in dried glassware.

Reagents.
[Solvent]
Dichloromethane was purchased from Kokusan Chemical Co., Ltd. and distilled from phosphorus pentoxide, then calcium hydride and dried over Molecular Sieves 4A.
Dimethyl sulfoxide was purchased from FUJIFILM Wako Pure Chemical Corp. and distilled from calcium hydride under negative pressure with rotary pump and dried over Molecular Sieves 4A.
Methanol was purchased from Kokusan Chemical Co., Ltd. and distilled from magnesium/iodine and dried over Molecular Sieves 3A.
Toluene was purchased from Kokusan Chemical Co., Ltd. and distilled from phosphorus pentoxide and dried over Molecular Sieves 4A.

Acetonitrile was purchased from Kanto Chemical Co., Inc. and distilled from phosphorus pentoxide, then calcium hydride and dried over Molecular Sieves 3A.
N,*N*-Dimethylformamide was purchased from Kanto Chemical Co., Inc. and dried over phosphorus pentoxide, then distilled from calcium hydride and dried over Molecular Sieves 4A.
Tetrahydrofuran (Tetrahydrofuran, Super Dehydrated, Stabilizer free) was purchased from Kanto Chemical Co., Inc. and used as received.
Diethyl ether (Diethyl ether, Infinity pure) was purchased from FUJIFILM Wako Pure Chemical Corp. and used as received.
1,2-Dimethoxyethane (1,2-Dimethoxyethane, Anhydrous, Inhibitor free) was purchased from Sigma-Aldrich Co., LLC and used as received.
Ethyl acetate (for HPLC) was purchased from Kokusan Chemical Co., Ltd. and used as received.
Hexane was purchased from Kokusan Chemical Co., Ltd. and used as received.
Acetic acid was purchased from Kokusan Chemical Co., Ltd. and used as received.
28–30% Aqueous ammonia was purchased from Kanto Chemical Co., Inc. and used as received.

[Desiccant]
Phosphorus pentoxide was purchased from Kokusan Chemical Co., Ltd. and used as received.
Calcium hydride was purchased from Junsei Chemical Co., Ltd. and used as received.
Magnesium was purchased from Nacalai Tesque, Inc. and used as received.
Iodine was purchased from Kanto Chemical Co., Inc. and used as received.
Potassium hydroxide was purchased from Kokusan Chemical Co., Ltd. and used as received.
Molecular Sieves 3A and Molecular Sieves 4A were purchased from Kokusan Chemical Co., Ltd. and dried by heating under negative pressure with rotary pump before use.

[Silica gel]
Flash column chromatography was performed on CHROMATOREX® PSQ 60B (60 μm) or Silica gel 60 (35–70 μm). CHROMATOREX® PSQ 60B was purchased from Fuji Silysia Chemical Ltd. and used as received. Silica gel 60 was purchased from Merck KGaA and used as received.
Open column chromatography was performed on Silica gel 60 (63–200 μm). Silica gel 60 was purchased from Merck KGaA and used as received.
Thin layer chromatography was performed on Wakogel B5F and Wakogel B5F was purchased from FUJIFILM Wako Pure Chemical Corp.

[Internal standard]
1,1,2,2-Tetrachloroethane was purchased from Tokyo Kasei Kogyo Co., Ltd. and used as received.
Trifluoromethylbenzene was purchased from Tokyo Kasei Kogyo Co., Ltd., distilled and dried over Molecular Sieves 4A.
Triphenylphosphine was purchased from Tokyo Kasei Kogyo Co., Ltd. and used as received.

[HWE reagents and reagents for HWE reagent's synthesis]
Bromoacetyl bromide was purchased from Tokyo Kasei Kogyo Co., Ltd. and used as received.
Chloroacetyl chloride was purchased from Tokyo Kasei Kogyo Co., Ltd. and used as received.
N,*O*-Dimethylhydroxyamine hydrochlroride was purchased from Tokyo Kasei Kogyo Co., Ltd. and used as received.
Triethylamine was purchased from Kokusan Chemical Co., Ltd. and distilled over potassium hydroxide.
Trimethyl phosphite was purchased from Tokyo Kasei Kogyo Co., Ltd. and used as received.
Triethyl phosphite was purchased from Tokyo Kasei Kogyo Co., Ltd. and used as received.
Triisopropyl phosphite was purchased from Sigma-Aldrich Co., LLC and used as received.
Triethyl phosphonoacetate was purchased from Tokyo Kasei Kogyo Co., Ltd. and used as received.
Chlorotrimethylsilane was purchased from Tokyo Kasei Kogyo Co., Ltd. and used as received.
Oxalyl chloride was purchased from Tokyo Kasei Kogyo Co., Ltd. and used as received.
2,2,2-Trifluoroethanol was purchased from Tokyo Kasei Kogyo Co., Ltd. and used as received.

4-Dimethylaminopyridine was purchased from Tokyo Kasei Kogyo Co., Ltd. and recrystallized with toluene.
Ammonium chloride was purchased from Kokusan Chemical Co., Ltd. and used as received.
Bis(2,2,2-trifluoroethyl) phosphite was purchased from Tokyo Kasei Kogyo Co., Ltd. and used as received.
Diphenyl phosphite was purchased from Tokyo Kasei Kogyo Co., Ltd. and used as received.
Diethyl 2,2-diethoxyethylphosphonate was purchased from Tokyo Kasei Kogyo Co., Ltd. and used as received.
12 M Aqueous Hydrochloride was purchased from Kokusan Chemical Co., Ltd. and used as received.

[Wittig reagents and reagents for Wittig reagent's synthesis]
Ethyl 2-(triphenyl-λ^5-phosphaneylidene)acetate was purchased from Tokyo Kasei Kogyo Co., Ltd. and used as received.
N-Methoxy-*N*-methyl-2-(triphenyl-λ^5-phosphaneylidene)acetamide was purchased from Sigma-Aldrich Co., LLC and used as received.
Triphenylphosphine was purchased from Tokyo Kasei Kogyo Co., Ltd. and used as received.
Ethyl bromoacetate was purchased from Tokyo Kasei Kogyo Co., Ltd. and used as received.

[Base]
A 1.0 M solution of lithium bis(trimethylsilyl)amide in tetrahydrofuran was purchased from Sigma-Aldrich Co., LLC and used as received.
A 1.0 M solution of sodium bis(trimethylsilyl)amide in tetrahydrofuran was purchased from Sigma-Aldrich Co., LLC and used as received.
A 1.0 M solution of potassium bis(trimethylsilyl)amide in tetrahydrofuran was purchased from Sigma-Aldrich Co., LLC and used as received.
A 1.6 M solution of *n*-butyllithium in hexane was purchased from Kanto Chemical Co., Inc. and used as received.
Sodium hydride (55% dispersion in mineral oil) was purchased from Kanto Chemical Co., Inc. and used as received.
Potassium *tert*-butoxide was purchased from Tokyo Kasei Kogyo Co., Ltd. and used as received.
A 0.70 M solution of isopropylmagnesium bromide in tetrahydrofuran was purchased from Kanto Chemical Co., Inc. and used as received.
A 1.0 M solution of methylmagnesium chloride in tetrahydrofuran was purchased from Sigma-Aldrich Co., LLC and used as received.
A 1.0 M solution of methylmagnesium bromide in tetrahydrofuran was purchased from Kanto Chemical Co., Inc. and used as received.
A 1.0 M solution of methylmagnesium iodide in diethyl ether was purchased from FUJIFILM Wako Pure Chemical Corp. and used as received.
A 0.90 M solution of ethylmagnesium bromide in tetrahydrofuran was purchased from FUJIFILM Wako Pure Chemical Corp. and used as received.
A 2.0 M solution of isopropylmagnesium chloride in tetrahydrofuran was purchased from Sigma-Aldrich Co., LLC and used as received.
A 1.3 M solution of isopropylmagnesium chloride-lithium chloride complex in tetrahydrofuran was purchased from Sigma-Aldrich Co., LLC and used as received.
A 1.0 M solution of phenylmagnesium bromide in tetrahydrofuran was purchased from Sigma-Aldrich Co., LLC and used as received.
A 1.2 M solution of methyl lithium in diethyl ether was purchased from Kanto Chemical Co., Inc. and used as received.
A 1.6 M solution of phenyl lithium in dibutyl ether was purchased from Tokyo Kasei Kogyo Co., Ltd. and used as received.
1,8-Diazabicyclo[5.4.0]-7-undecene was purchased from Tokyo Kasei Kogyo Co., Ltd. and used as received.

N,*N*-Diisopropylethylamine was purchased from Tokyo Kasei Kogyo Co., Ltd. and distilled from ninhydrin, then from potassium hydroxide.
Lithium hydroxide was purchased from Merck KGaA and used as received.
Sodium hydroxide was purchased from FUJIFILM Wako Pure Chemical Corp. and used as received.
Benzyltrimethylammonium hydroxide (40% in MeOH) was purchased from Tokyo Kasei Kogyo Co., Ltd. and used as received.

[Additive]
Lithium chloride anhydrous was purchased from Tokyo Kasei Kogyo Co., Ltd. and dried at 140 °C for 4 h under negative pressure with rotary pump before use.[1]
Magnesium bromide anhydrous was purchased from Strem Chemicals, Inc. and dried at 150 °C for 1.5 h under negative pressure with rotary pump before use.[2]
Magnesium chloride anhydrous was purchased from Sigma-Aldrich Co., LLC and dried at 200 °C for 1.5 h under negative pressure with rotary pump before use.[3]
1,4,7,10,13,16-Hexaoxacyclooctadecane was purchased from Tokyo Kasei Kogyo Co., Ltd. and recrystallized with acetonitrile.[4]

[Reagents for substrate synthesis]
trans-Cinnamaldehyde was purchased from Tokyo Kasei Kogyo Co., Ltd. and used as received.
A 1.0 M solution of diisobutylaluminum hydride in hexane was purchased from Kanto Chemical Co., Inc. and used as received.
Rochelle salt was purchased from Kokusan Chemical Co., Ltd. and used as received.
Benzaldehyde was purchased from Kanto Chemical Co., Inc. and washed with 10% aqueous sodium carbonate, saturated aqueous sodium sulfite and water, then dried over sodium sulfate and distilled.[5]
A 1.3 M solution of lithium bis(trimethylsilyl)amide in tetrahydrofuran was purchased from Tokyo Kasei Kogyo Co., Ltd. and used as received.
Trimethylsulfoxonium iodide was purchased from Tokyo Kasei Kogyo Co., Ltd. and used as received.
Ethyl acrylate was purchased from Tokyo Kasei Kogyo Co., Ltd. and used as received.
1,4-Diazabicyclo[2.2.2]octane was purchased from Sigma-Aldrich Co., LLC and used as received.
Palladium 10% on carbon (M) dry was purchased from Kawaken Fine Chemicals Co., Ltd. and used as received.
tert-Butyldimethylchlorosilane was purchased from Kanto Chemical Co., Inc. and used as received.
Trifluoromethanesulfonic acid was purchased from Tokyo Kasei Kogyo Co., Ltd. and used as received.
tert-Butyldimethylsilyl trifluoromethanesulfonate was prepared from *tert*-butyldimethylchlorosilane and trifluoromethanesulfonic acid and distilled.[6]
2,6-Lutidine was purchased from Kokusan Chemical Co., Ltd. and used as received.
Sodium hydrogen carbonate was purchased from Kokusan Chemical Co., Ltd. and used as received.
Benzene-1,4-dicarbaldehyde was purchased from Tokyo Kasei Kogyo Co., Ltd. and used as received.
Tetrapropylammonium perruthenate was purchased from Tokyo Kasei Kogyo Co., Ltd. and used as received.
4-Methylmorpholine *N*-oxide was purchased from Tokyo Kasei Kogyo Co., Ltd. and used as received.
Benzene-1,4-dicarbonyl dichloride was purchased from Tokyo Kasei Kogyo Co., Ltd. and used as received.
Potassium carbonate anhydrous was purchased from Kokusan Chemical Co., Ltd. and used as received.
Sodium *tert*-butoxide was purchased from Tokyo Kasei Kogyo Co., Ltd. and used as received.
1,12-Dodecanediol was purchased from Tokyo Kasei Kogyo Co., Ltd. and used as received.
Sulfur trioxide-pyridine complex was purchased from Sigma-Aldrich Co., LLC and used as received.

[Substrate scope]
Acetaldehyde was purchased from Kanto Chemical Co., Inc. and distilled before use.
Propanal was purchased from Tokyo Kasei Kogyo Co., Ltd. and distilled before use.
2-Methylpropanal was purchased from Tokyo Kasei Kogyo Co., Ltd. and used as received.

2,2-Dimethylpropanal was purchased from Tokyo Kasei Kogyo Co., Ltd. and washed with 10% aqueous sodium carbonate, saturated aqueous sodium sulfite and water, then dried over sodium sulfate and distilled.[5]
2,2-Diphenylacetaldehyde was purchased from Tokyo Kasei Kogyo Co., Ltd. and used as received.
3-Methylbutanal was purchased from Sigma-Aldrich Co., LLC and used as received.
3-Phenylpropanal was purchased from Tokyo Kasei Kogyo Co., Ltd. and washed with 10% aqueous sodium carbonate, saturated aqueous sodium sulfite and water, then dried over sodium sulfate and distilled.[5]
Pentanal was purchased from Tokyo Kasei Kogyo Co., Ltd. and washed with 10% aqueous sodium carbonate, saturated aqueous sodium sulfite and water, then dried over sodium sulfate and distilled.[5]
Heptanal was purchased from Tokyo Kasei Kogyo Co., Ltd. and washed with 10% aqueous sodium carbonate, saturated aqueous sodium sulfite and water, then dried over sodium sulfate and distilled.[5]
Octanal was purchased from Tokyo Kasei Kogyo Co., Ltd. and washed with 10% aqueous sodium carbonate, saturated aqueous sodium sulfite and water, then dried over sodium sulfate and distilled.[5]
Cyclopropanecarbaldehyde was purchased from Tokyo Kasei Kogyo Co., Ltd. and used as received.
Cyclopentanecarbaldehyde was purchased from Sigma-Aldrich Co., LLC and used as received.
Cyclohexanecarbaldehyde was purchased from Tokyo Kasei Kogyo Co., Ltd. and used as received.
(2*E*)-Pent-2-enal was purchased from Tokyo Kasei Kogyo Co., Ltd. and used as received.
(2*E*)-2-Methylpent-2-enal was purchased from Tokyo Kasei Kogyo Co., Ltd. and used as received.
(2*Z*)-2-Bromo-3-phenylprop-2-enal was purchased from Tokyo Kasei Kogyo Co., Ltd. and used as received.
3-(Trimethylsilyl)-prop-2-ynal was purchased from Tokyo Kasei Kogyo Co., Ltd. and used as received.
(3*S*)-3,7-Dimethyloct-6-enal was purchased from Tokyo Kasei Kogyo Co., Ltd. and used as received.
2-Bromopropane-1,3-dial was purchased from Kanto Chemical Co., Inc. and used as received.
2-Methylbenzaldehyde was purchased from Tokyo Kasei Kogyo Co., Ltd. and used as received.
3-Methylbenzaldehyde was purchased from Tokyo Kasei Kogyo Co., Ltd. and used as received.
4-Methylbenzaldehyde was purchased from Tokyo Kasei Kogyo Co., Ltd. and used as received.
2,6-Dimethylbenzaldehyde was purchased from Tokyo Kasei Kogyo Co., Ltd. and distilled before use.
4-Bromobenzaldehyde was purchased from Tokyo Kasei Kogyo Co., Ltd. and used as received.
4-Methoxybenzaldehyde was purchased from FUJIFILM Wako Pure Chemical Corp. and used as received.
4-Dimethylaminobenzaldehyde was purchased from Tokyo Kasei Kogyo Co., Ltd. and used as received.
Methyl 4-formylbenzoate was purchased from Kanto Chemical Co., Inc. and used as received.
4-(Trifluoromethyl)benzaldehyde was purchased from Tokyo Kasei Kogyo Co., Ltd. and used as received.
4-Cyanobenzaldehyde was purchased from Tokyo Kasei Kogyo Co., Ltd. and used as received.
4-Nitrobenzaldehyde was purchased from Tokyo Kasei Kogyo Co., Ltd. and used as received.
4-Formylbenzoic acid was purchased from Tokyo Kasei Kogyo Co., Ltd. and used as received.
4-Hydroxybenzaldehyde was purchased from Tokyo Kasei Kogyo Co., Ltd. and used as received.
4-Boronobenzaldehyde was purchased from Tokyo Kasei Kogyo Co., Ltd. and used as received.
Potassium dihydrogenphosphate was purchased from Kokusan Chemical Co., Ltd. and used as received.
Disodium hydrogenphosphate was purchased from Kokusan Chemical Co., Ltd. and used as received.
Buffer solution pH7 was prepared from potassium dihydrogenphosphate (9.10 g), disodium hydrogenphosphate (40.8 g) and water (1 L).
4-(4,4,5,5-Tetramethyl-1,3,2-dioxaborolan-2-yl)benzaldehyde was purchased from Tokyo Kasei Kogyo Co., Ltd. and used as received.
2-Pyridinecarbaldehyde was purchased from Tokyo Kasei Kogyo Co., Ltd. and used as received.
3-Pyridinecarbaldehyde was purchased from Tokyo Kasei Kogyo Co., Ltd. and used as received.
4-Pyridinecarbaldehyde was purchased from Tokyo Kasei Kogyo Co., Ltd. and used as received.
Furan-2-carbaldehyde was purchased from Tokyo Kasei Kogyo Co., Ltd. and used as received.
Thiophene-2-carbaldehyde was purchased from Tokyo Kasei Kogyo Co., Ltd. and used as received.

1H-Pyrrole-2-carbaldehyde was purchased from Tokyo Kasei Kogyo Co., Ltd. and used as received.
1-Methyl-1H-pyrrole-2-carbaldehyde was purchased from Tokyo Kasei Kogyo Co., Ltd. and used as received.
1H-Indole-3-carbaldehyde was purchased from Tokyo Kasei Kogyo Co., Ltd. and used as received.
1-Naphthaldehyde was purchased from Tokyo Kasei Kogyo Co., Ltd. and used as received.
Anthracene-9-carbaldehyde was purchased from Sigma-Aldrich Co., LLC and used as received.
Benzene-1,2-dicarbaldehyde was purchased from Tokyo Kasei Kogyo Co., Ltd. and used as received.
Benzene-1,3-dicarbaldehyde was purchased from Tokyo Kasei Kogyo Co., Ltd. and used as received.
Benzene-1,3,5-tricarbaldehyde was purchased from Tokyo Kasei Kogyo Co., Ltd. and used as received.

[Reagents for applications]
Cyclohexanone was purchased from Tokyo Kasei Kogyo Co., Ltd. and used as received.

Preparation of HWE Reagents.

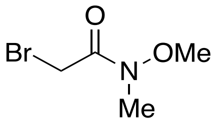

2-Bromo-*N*-methoxy-*N*-methylacetamide (S1).
Ref. 7, which is modification method of ref. 8.
To a suspension of *N*,*O*-dimethylhydroxylamine hydrochloride (28.8 g, 295 mmol) and triethylamine (40.9 mL, 295 mmol) in dichloromethane (600 mL), a solution of bromoacetyl bromide (56.7 g, 280 mmol) in dichloromethane (100 mL) was added dropwise at 0 °C with stirring. After the reaction mixture was stirred for 20 min at room temperature, water was added at 0 °C. The mixture was extracted with dichloromethane, and the organic layer was washed with brine and dried over sodium sulfate. After filtration of the mixture and concentration of the solvent, the crude mixture was purified by open column chromatography (eluant; hexane/ethyl acetate = 1/1) to afford almost pure Weinreb amide **S1**. Almost pure Weinreb amide **S1** was distilled under negative pressure by diaphragm pump (bp = 95–98 °C, P = 9.0 mmHg) to afford Weinreb amide **S1** (**S1**/2-chloro-*N*-methoxy-*N*-methylacetamide = 17/1) (36.7 g, 73%) as colorless oil.
R_f = 0.76 (silica gel, chloroform/methanol = 20/1); FT-IR (neat) ν_{max}: 2947, 1736, 1666, 1435, 1389, 1180 cm^{-1}; ^{1}H NMR (500 MHz, $CDCl_3$): δ 4.01 (s, 2H, H-2), 3.79 (s, 3H, OMe), 3.24 (s, 3H, NMe); $^{13}C\{^1H\}$ NMR (125 MHz, $CDCl_3$): 167.6 (C-1), 61.6 (OMe), 32.5 (C-2), 25.1 (NMe); HRMS calcd for $C_4H_8BrNO_2Na$ $[M + Na]^+$ 203.9631, found 203.9637.

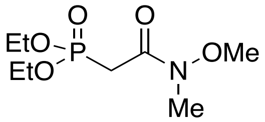

2-Diethoxyphosphoryl-*N*-methoxy-*N*-methylacetamide (1).
Ref. 9
To a 100 mL two-necked flask under argon equipped with a Dean–Stark apparatus, Weinreb amide **S1** (**S1**/2-chloro-*N*-methoxy-*N*-methylacetamide = 17/1) (22.1 g, 121 mmol) and triethyl phosphite (22.8 mL, 133 mmol) were added successively. The reaction mixture was stirred at 80 °C for 1.5 h. The resulting produced byproduct of bromoethane was distilled off in the Dean–Stark apparatus. The reaction mixture was cooled to room temperature. After concentration of mixture, the crude mixture was purified by open column chromatography (eluant; hexane/ethyl acetate = 4/1 to chloroform/methanol = 9/1), and purified by open column chromatography (eluant; hexane/ethyl acetate = 1/3 to chloroform/methanol = 9/1) to afford almost pure phosphate **1** (27.2 g, 94%) as slightly yellow oil. Almost pure product **1** was distilled under negative pressure by rotary pump (bp = 115 °C, P = 1.3 mmHg) to afford phosphate **1** (21.7 g, 75%) as colorless oil.

R_f = 0.42 (silica gel, chloroform/methanol = 20/1); FT-IR (neat) ν_{max}: 2985, 1666, 1381, 1257, 1049, 1026, 964 cm^{-1}; ^{1}H NMR (500 MHz, CDCl$_3$): δ 4.25–4.13 (m, 4H, P(OCH_2CH$_3$)$_2$), 3.78 (s, 3H, OMe), 3.22 (s, 3H, NMe), 3.18 (d, $^2J_{HP}$ = 22.5 Hz, 2H, H-2), 1.35 (t, J = 7.0 Hz, 6H, P(OCH$_2$CH_3)$_2$); ^{13}C{^{1}H} NMR (125 MHz, CDCl$_3$): 165.7 (d, $^2J_{CP}$ = 5.9 Hz, C-1), 62.1 (d, $^2J_{CP}$ = 6.0 Hz, P(OCH$_2$CH$_3$)$_2$), 61.1 (OMe), 31.8 (NMe), 31.0 (d, $^1J_{CP}$ = 134.8 Hz, C-2), 16.0 (d, $^3J_{CP}$ = 5.9 Hz, P(OCH$_2$$CH_3$)$_2$); ^{31}P{^{1}H, ^{13}C} NMR (202 MHz, CDCl$_3$): 20.6 (P(OCH$_2$CH$_3$)$_2$); HRMS calcd for $C_{16}H_{36}N_2O_{10}P_2Na$ [2M + Na]$^+$ 501.1737, found 501.1736.

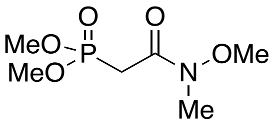

2-Dimethoxyphosphoryl-*N*-methoxy-*N*-methylacetamide (S2).

To Weinreb amide **S1** (**S1**/2-chloro-*N*-methoxy-*N*-methylacetamide = 17/1) (7.07 g, 38.8 mmol), trimethyl phosphite (4.77 mL, 40.4 mmol) was added. The reaction mixture was stirred at 60 °C for 2 h, and stirred at 70 °C for 30 min. Since Weinreb amide **S1** remained from ^{1}H NMR spectra of the reaction mixture, trimethyl phosphite (2.27 mL, 19.2 mmol) was added to the reaction mixture. After the reaction mixture was stirred at 80 °C for 2 h, the crude mixture was purified by open column chromatography (eluant; hexane/ethyl acetate = 4/1 to chloroform/methanol = 9/1), and purified by open column chromatography (eluant; hexane/ethyl acetate = 1/3 to chloroform/methanol = 9/1) to afford the mixture of product and impurity (9.75 g). The mixture was distilled under negative pressure by rotary pump (bp = 104 °C, P = 1.1 mmHg) to afford phosphate **S2** (4.79 g, 58%) as colorless oil.

R_f = 0.49 (silica gel, chloroform/methanol = 20/1); FT-IR (neat) ν_{max}: 2954, 1658, 1466, 1381, 1265, 1180, 1034, 872, 810 cm^{-1}; ^{1}H NMR (500 MHz, CDCl$_3$): δ 3.81 (d, $^3J_{HP}$ = 11.0 Hz, 6H, P(OCH_3)$_2$), 3.76 (s, 3H, NOMe), 3.21 (s, 3H, NMe), 3.18 (d, $^2J_{HP}$ = 22.0 Hz, 2H, H-2); ^{13}C{^{1}H} NMR (125 MHz, CDCl$_3$): 165.6 (d, $^2J_{CP}$ = 6.0 Hz, C-1), 61.3 (NOMe), 52.9 (d, $^2J_{CP}$ = 6.0 Hz, P(OCH$_3$)$_2$), 31.9 (NMe), 30.4 (d, $^1J_{CP}$ = 135.9 Hz, C-2); ^{31}P{^{1}H, ^{13}C} NMR (202 MHz, CDCl$_3$): 23.3 (P(OCH$_3$)$_2$); HRMS calcd for $C_6H_{14}NO_5PNa$ [M + Na]$^+$ 234.0502, found 234.0502.

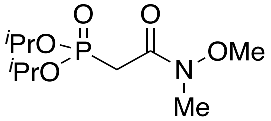

2-Diisopropoxyphosphoryl-*N*-methoxy-*N*-methylacetamide (S3).

To Weinreb amide **S1** (**S1**/2-chloro-*N*-methoxy-*N*-methylacetamide = 17/1) (7.04 g, 38.7 mmol), triisopropyl phosphite (9.24 mL, 40.4 mmol) was added. The reaction mixture was stirred at 90 °C for 2 h. Since Weinreb amide **S1** remained from ^{1}H NMR spectra of the reaction mixture, triisopropyl phosphite (0.88 mL, 3.85 mmol) was added to the reaction mixture. After the reaction mixture was stirred at 90 °C for 20 min, the crude mixture was purified by open column chromatography (eluant; hexane/ethyl acetate = 4/1 to chloroform/methanol = 9/1), and purified by open column chromatography (eluant; hexane/ethyl acetate = 1/3 to chloroform/methanol = 9/1) to afford almost pure phosphate **S3** (9.89 g, 95%). Almost pure phosphate **S3** was distilled under negative pressure by rotary pump (bp = 97 °C, P = 1.1 mmHg) to afford phosphate **S3** (8.15 g, 78%) as colorless oil.

R_f = 0.48 (silica gel, chloroform/methanol = 20/1); FT-IR (neat) ν_{max}: 2978, 1666, 1381, 1257, 987 cm^{-1}; ^{1}H NMR (500 MHz, CDCl$_3$): δ 4.84–4.71 (m, 2H, P(OCH(CH$_3$)$_2$)$_2$), 3.78 (s, 3H, OMe), 3.21 (s, 3H, NMe), 3.13 (d, $^2J_{HP}$ = 22.5 Hz, 2H, H-2), 1.353 (d, J = 6.0 Hz, 6H, P(OCH(CH_3)$_2$)$_2$), 1.345 (d, J = 6.0 Hz, 6H, P(OCH(CH_3)$_2$)$_2$); ^{13}C{^{1}H} NMR (125 MHz, CDCl$_3$): 166.2 (d, $^2J_{CP}$ = 5.9 Hz, C-1), 71.1 (d, $^2J_{CP}$ = 5.9 Hz, P(OCH(CH$_3$)$_2$)$_2$), 61.3 (OMe), 32.3 (d, $^1J_{CP}$ = 135.9 Hz, C-2), 32.0 (NMe), 23.9 (d, $^3J_{CP}$ = 3.6 Hz, P(OCH(CH$_3$)$_2$)$_2$), 23.7 (d, $^3J_{CP}$ = 4.8 Hz, P(OCH(CH$_3$)$_2$)$_2$); ^{31}P{^{1}H, ^{13}C} NMR (202 MHz, CDCl$_3$): 18.4 (P(OCH(CH$_3$)$_2$)$_2$); HRMS calcd for $C_{10}H_{22}NO_5PNa$ [M + Na]$^+$ 290.1128, found 290.1130.

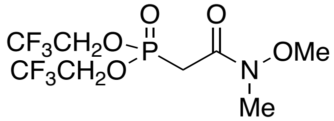

2-Bis(2,2,2-trifluoroethoxy)phosphoryl-*N*-methoxy-*N*-methylacetamide (21).

Ref. 10, 11

To Weinreb amide **1** (20.0 g, 83.6 mmol), chlorotrimethylsilane (53.0 mL, 420 mmol) was added. The reaction mixture was stirred at 70 °C for 124 h. Since Weinreb amide **1** remained from ^{1}H NMR spectra of the reaction mixture, chlorotrimethylsilane (10.6 mL, 83.9 mmol) was added to the reaction mixture. After the reaction mixture was stirred at 70 °C for 13 h, remaining solvent was concentrated. The crude bis(trimethylsilyloxy) phosphate was obtained, and was used in the next step without further purification.

To a solution of above crude bis(trimethylsilyloxy) phosphate in dichloromethane (168 mL), one drop of *N*,*N*-dimethylformamide was added. After oxalyl chloride (18.3 mL, 209 mmol) was added to the mixture at 0 °C, the reaction mixture was stirred at room temperature for 1 h. The crude dichlorophosphoryl Weinreb amide was obtained by concentrating the remaining solvent, and was used in the next step without further purification.

To a solution of above crude dichlorophosphoryl Weinreb amide in dichloromethane (84 mL), a mixture of dichloromethane (84 mL), 2,2,2-trifluoroethanol (23.9 mL, 334 mmol) and triethylamine (69.6 mL, 502 mmol) was added dropwise at 0 °C over 1 h. After 4-dimethylaminopyridine (204 mg, 1.67 mmol) was added to the mixture, the reaction mixture was stirred at room temperature for 19 h. To the reaction mixture, saturated aqueous ammonium chloride was added at 0 °C, and the mixture was extracted with dichloromethane. The organic layer was washed with brine and dried over sodium sulfate. After filtration of the mixture and concentration of the solvent, the crude mixture was purified by open column chromatography (eluant: ethyl acetate) to afford crude phosphate. The crude phosphate was purified by flash column chromatography (eluant: hexane/ethyl acetate = 1/1 to ethyl acetate) to afford almost pure bis-2,2,2-trifluoroethyl phosphate **21** (7.75 g, 27%) as pale yellow oil and almost pure ethoxy(2,2,2-trifluoroethyl) phosphate **S4** (7.34 g, 30%) as slightly pale yellow oil. Almost pure bis-2,2,2-trifluoroethyl phosphate **21** was distilled under negative pressure by rotary pump with Kugelrohr (bp = 120–125 °C, P = 1.9 mmHg) to afford bis-2,2,2-trifluoroethyl phosphate **21** (6.66 g, 23%) as slightly pale yellow oil. Almost pure ethoxy(2,2,2-trifluoroethyl) phosphate **S4** was distilled under negative pressure by rotary pump with Kugelrohr (bp = 130–135 °C, P = 1.8 mmHg) to afford ethoxy(2,2,2-trifluoroethyl) phosphate **S4** (6.40 g, 26%) as colorless oil.

R_f= 0.64 (silica gel, ethyl acetate); ATR-IR ν_{max}: 2977, 1667, 1230, 1267, 1179, 1110, 1076, 964 cm^{-1}; ^{1}H NMR (500 MHz, CDCl$_3$): δ 4.55–4.43 (m, 4H, P(OCH_2CF$_3$)$_2$), 3.75 (s, 3H, OMe), 3.35 (d, $^2J_{HP}$ = 21.5 Hz, 2H, H-2), 3.22 (s, 3H, NMe); ^{13}C{^{1}H} NMR (125 MHz, CDCl$_3$): 164.9 (d, $^2J_{CP}$ = 3.6 Hz, C-1), 122.5 (qd, $^1J_{CF}$ = 275.4 Hz, $^3J_{CP}$ = 8.4 Hz, P(OCH$_2$$CF_3$)$_2$), 62.4 (qd, $^2J_{CF}$ = 37.6 Hz, $^2J_{CP}$ = 6.0 Hz, P(OCH$_2$CF$_3$)$_2$), 61.4 (OMe), 31.9 (NMe), 31.5 (d, $^1J_{CP}$ = 146.6 Hz, C-2); ^{31}P{^{1}H, ^{13}C} NMR (202 MHz, CDCl$_3$): 24.5 (P(OCH$_2$CF$_3$)$_2$); ^{19}F{^{1}H, ^{13}C} NMR (470 MHz, CDCl$_3$): –76.3 (P(OCH$_2$CF_3)$_2$); HRMS calcd for $C_8H_{12}F_6NO_5PNa$ [M + Na]$^+$ 370.0255, found 370.0273.

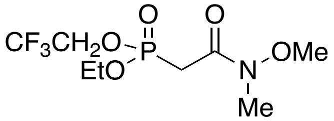

2-[Ethoxy(2,2,2-trifluoroethoxy)phosphoryl]-*N*-methoxy-*N*-methylacetamide (S4).

R_f= 0.33 (silica gel, ethyl acetate); ATR-IR ν_{max}: 2984, 1666, 1293, 1264, 1176, 1102, 1039 cm^{-1}; ^{1}H NMR (500 MHz, CDCl$_3$): δ 4.53–4.31 (m, 2H, POCH_2CF$_3$), 4.24–4.05 (m, 2H, POCH_2CH$_3$), 3.67 (s, 3H, OMe), 3.26 (dd, $^2J_{HP}$ = 21.0 Hz, J = 15.5 Hz, 1H, H-2), 3.14 (s, 3H, NMe), 3.08 (dd, $^2J_{HP}$ = 22.5 Hz, J = 15.5 Hz, 1H, H-2), 1.28 (t, J = 7.0 Hz, 3H, POCH$_2$CH_3); ^{13}C{^{1}H} NMR (125 MHz, CDCl$_3$): 165.4 (d, $^2J_{CP}$ = 4.8 Hz, C-1), 122.8 (qd, $^1J_{CF}$ = 275.4 Hz, $^3J_{CP}$ = 8.4 Hz, POCH$_2$$CF_3$), 62.7 (qd, $^2J_{CF}$ =

37.0 Hz, $^2J_{CP}$ = 4.8 Hz, PO*C*H$_2$CF$_3$), 62.6 (d, $^2J_{CP}$ = 5.9 Hz, PO*C*H$_2$CH$_3$), 61.3 (OMe), 31.9 (NMe), 31.3 (d, $^1J_{CP}$ = 141.9 Hz, C-2), 16.0 (d, $^3J_{CP}$ = 7.1 Hz, POCH$_2$*C*H$_3$); ^{31}P{^{1}H, ^{13}C} NMR (202 MHz, CDCl$_3$): 22.3 (*P*(OCH$_2$CH$_3$)OCH$_2$CF$_3$); ^{19}F{^{1}H, ^{13}C} NMR (470 MHz, CDCl$_3$): –76.3 (POCH$_2$C*F$_3$*); HRMS calcd for $C_8H_{15}F_3NO_5PNa$ [M + Na]$^+$ 316.0538, found 316.0527.

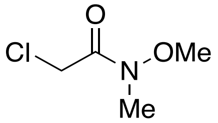

2-Chloro-*N*-methoxy-*N*-methylacetamide (S5).

To a suspension of *N,O*-dimethylhydroxylamine hydrochloride (47.5 g, 487 mmol) and triethylamine (67.5 mL, 487 mmol) in dichloromethane (420 mL), a solution of chloroacetyl chloride (50.4 g, 446 mmol) in dichloromethane (22 mL) was added dropwise at 0 °C with stirring. After the reaction mixture was stirred at room temperature for 1 h, the reaction mixture was diluted with water at 0 °C. The mixture was extracted with dichloromethane, and the organic layer was dried over sodium sulfate. After filtration of the mixture and concentration of the solvent, the crude mixture was purified by flash column chromatography (eluant: hexane/ethyl acetate = 3/1 to 2/1) to afford Weinreb amide **S5** (57.4 g, 94%) as colorless solid.

Weinreb amide **S5** is solid at room temperature, but melting point was 25–30 °C to make immeasurable accurately.

R_f= 0.61 (silica gel, hexane/ethyl acetate = 1/1); ATR-IR ν_{max}: 2948, 1684, 1471, 998, 774 cm^{-1}; ^{1}H NMR (500 MHz, CDCl$_3$): δ 4.24 (s, 2H, H-2), 3.75 (s, 3H, OMe), 3.24 (s, 3H, NMe); ^{13}C{^{1}H} NMR (125 MHz, CDCl$_3$): 167.5 (C-1), 61.6 (OMe), 40.8 (C-2), 32.6 (NMe); HRMS calcd for $C_4H_8ClNO_2Na$ [M + Na]$^+$ 160.0136, found 160.0129.

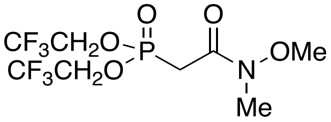

2-Bis(2,2,2-trifluoroethoxy)phosphoryl-*N*-methoxy-*N*-methylacetamide (21).

Ref. 12

To a solution of bis(2,2,2-trifluoroethyl) phosphite (23.2 g, 94.5 mmol) in tetrahydrofuran (100 mL), sodium hydride (55% dispersion in mineral oil) (4.28 g, 98.1 mmol) was added at 0 °C. After the mixture was stirred at 0 °C for 30 min, a solution of Weinreb amide **S5** (10.0 g, 72.6 mmol) in tetrahydrofuran (45 mL) was added dropwise to the mixture, and the reaction mixture was stirred at room temperature for 12 h. To the reaction mixture, saturated aqueous ammonium chloride was added at 0 °C, and the mixture was extracted with dichloromethane. The organic layer was washed with brine and dried over sodium sulfate. After filtration of the mixture and concentration of the solvent, the crude mixture was purified by flash column chromatography (eluant: hexane/ethyl acetate = 2/1 to 1/2) to afford almost pure bis-2,2,2-trifluoroethyl phosphate **21** (22.9 g, 91%) as pale yellow oil. Almost pure product **S4** was distilled under negative pressure by rotary pump with Kugelrohr (bp = 100 °C, *P* = 0.50 mmHg) to afford bis-2,2,2-trifluoroethyl Weinreb amide **21** (20.0 g, 79%) as colorless oil.

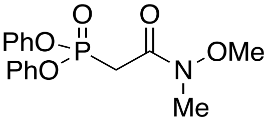

2-Diphenoxyphosphoryl-*N*-methoxy-*N*-methylacetamide (S6).

Ref. 13, which is modification method of ref. 14.

To a suspension of sodium hydride (55% dispersion in mineral oil) (1.75 g, 40.0 mmol) in tetrahydrofuran (14 mL), a solution of diphenyl phosphite (9.20 g, 39.3 mmol) in tetrahydrofuran (4 mL) was added at 0 °C. After the mixture was stirred at 0 °C for 30 min, a solution of Weinreb amide **S5** (5.05 g, 36.7 mmol) in tetrahydrofuran (18 mL) was added dropwise to the mixture, and the reaction

mixture was stirred at room temperature for 14 h. To the reaction mixture, saturated aqueous ammonium chloride was added at 0 °C, and the mixture was extracted with dichloromethane. The organic layer was washed with brine and dried over sodium sulfate. After filtration of the mixture and concentration of the solvent, the crude mixture was purified by flash column chromatography (eluant: hexane/ethyl acetate = 1/1 to chloroform/methanol = 9/1) to afford phosphate **S6** (8.48 g, 69%) as slightly pale yellow oil.
R_f = 0.48 (silica gel, chloroform/methanol = 20/1); ATR-IR ν_{max}: 2941, 1668, 1591, 1491, 1282, 1215, 1191, 1163, 946, 768, 691 cm^{-1}; ^{1}H NMR (500 MHz, $CDCl_3$): δ 7.36–7.29 (m, 4H, OPh), 7.29–7.21 (m, 4H, OPh), 7.21–7.15 (m, 2H, OPh), 3.76 (s, 3H, OMe), 3.48 (d, $^2J_{HP}$ = 22.5 Hz, 2H, H-2), 3.25 (s, 3H, NMe); ^{13}C{^{1}H} NMR (125 MHz, $CDCl_3$): 164.8 (d, $^2J_{CP}$ = 5.9 Hz, C-1), 150.0 (d, $^2J_{CP}$ = 8.3 Hz, OPh), 129.7 (OPh), 125.3 (OPh), 120.7 (d, $^3J_{CP}$ = 3.6 Hz, OPh), 61.4 (OMe), 32.1 (NMe), 31.1 (d, $^1J_{CP}$ = 138.3 Hz, C-2); ^{31}P{^{1}H, ^{13}C} NMR (202 MHz, $CDCl_3$): 14.0–13.4 (m, *P*(OPh)$_2$); HRMS calcd for $C_{16}H_{18}NO_5PNa$ [M + Na]$^+$ 358.0815, found 358.0818.

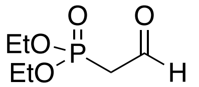

2-Diethoxyphosphorylacetaldehyde (15t).
Ref. 15
To diethyl 2,2-diethoxyethylphosphonate (47.3 g, 186 mmol), 2 M aqueous hydrochloride (558 mL, 1120 mmol) was added at room temperature. The reaction mixture was stirred at room temperature for 17 h. To the reaction mixture, sodium chloride was added until saturated. The mixture was extracted with dichloromethane, and the organic layer was dried over sodium sulfate. After filtration of the mixture and concentration of the solvent, the crude mixture was distilled under negative pressure by rotary pump (bp = 90 °C, *P* = 1.8 mmHg) to afford aldehyde **15t** (29.5 g, 88%) as colorless oil.
R_f = 0.28 (silica gel, chloroform/methanol = 20/1); FT-IR (neat) ν_{max}: 2985, 1728, 1257, 1026, 964 cm^{-1}; ^{1}H NMR (500 MHz, $CDCl_3$): δ 9.68 (t, *J* = 3.5 Hz, 1H, H-1), 4.25–4.10 (m, 4H, P(OCH_2CH$_3$)$_2$), 3.09 (dd, $^2J_{HP}$ = 22.0 Hz, *J* = 3.5 Hz, 2H, H-2), 1.36 (t, *J* = 7.0 Hz, 6H, P(OCH$_2$CH_3)$_2$); ^{13}C{^{1}H} NMR (125 MHz, $CDCl_3$): 193.1 (d, $^2J_{CP}$ = 7.3 Hz, C-1), 62.8 (d, $^2J_{CP}$ = 6.0 Hz, P(O*C*H$_2$CH$_3$)$_2$), 43.3 (d, $^1J_{CP}$ = 127.5 Hz, C-2), 16.4 (d, $^3J_{CP}$ = 6.0 Hz, P(OCH$_2$*C*H$_3$)$_2$); ^{31}P{^{1}H, ^{13}C} NMR (202 MHz, $CDCl_3$): 18.4 (*P*(OCH$_2$CH$_3$)$_2$); HRMS calcd for $C_6H_{13}O_4PNa$ [M + Na]$^+$ 203.0444, found 203.0451.

Preparation of substrates.

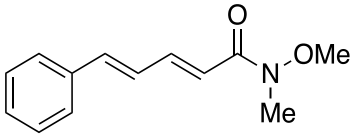

(2*E*,4*E*)-*N*-Methoxy-*N*-methyl-5-phenylpenta-2,4-dienamide ((*E*)-18d, (2*E*,4*E*)-18d).
To a solution of phosphate **1** (9.57 g, 40.0 mmol) in tetrahydrofuran (80 mL), a 2.0 M solution of isopropylmagnesium chloride in tetrahydrofuran (18.0 mL, 36.0 mmol) was added at –78 °C. After the mixture was stirred at –78 °C for 30 min, a solution of *trans*-cinnamaldehyde (2.64 g, 20.0 mmol) in tetrahydrofuran (20 mL) was added at room temperature, and the reaction mixture was stirred for 21 h. To the reaction mixture, saturated aqueous ammonium chloride was added at 0 °C, and the mixture was extracted with ethyl acetate. The organic layer was dried over sodium sulfate. After filtration of the mixture and concentration of the solvent, the crude mixture was purified by open column chromatography (eluant: hexane/ethyl acetate = 1/2) to afford crude diene **18d**. The crude diene was purified by flash column chromatography (eluant: hexane/ethyl acetate = 4/1 to 1/1) to afford (2*E*,4*E*)-diene **18d** (4.03 g, 93%) as white solid and (2*Z*,4*E*)-diene **18d** (131 mg, 3.0%) as white solid.
R_f = 0.38 (silica gel, hexane/ethyl acetate = 2/1); mp: 64.0 °C; FT-IR (KBr) ν_{max}: 3433, 1643, 1604, 1381, 1018, 995, 694 cm^{-1}; ^{1}H NMR (500 MHz, C_6D_6): δ 7.84 (dd, *J* = 15.0, 11.0 Hz, 1H, H-3),

7.22–7.12 (m, 2H, Ar), 7.12–6.97 (m, 3H, Ar), 6.72 (dd, *J* = 15.0, 11.0 Hz, 1H, H-4), 6.65 (d, *J* = 15.0 Hz, H-5), 6.51 (d, *J* = 15.0 Hz, 1H, H-2), 3.12 (s, 3H, OMe), 3.01 (s, 3H, NMe); $^{13}C\{^1H\}$ NMR (125 MHz, C_6D_6): 167.1 (C-1), 143.5 (C-3), 139.6 (C-5), 136.8 (Ar), 128.8 (Ar), 128.7 (Ar), 127.4 (Ar), 127.2 (C-4), 120.1 (C-2), 61.1 (OMe), 32.2 (NMe); HRMS calcd for $C_{13}H_{15}NO_2Na$ $[M + Na]^+$ 240.0995, found 240.1003.

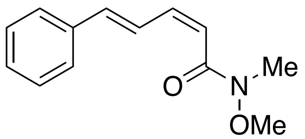

(2*Z*,4*E*)-*N*-Methoxy-*N*-methyl-5-phenylpenta-2,4-dienamide ((*Z*)-18d, (2*Z*,4*E*)-18d).
R_f = 0.54 (silica gel, hexane/ethyl acetate = 2/1); mp: 61.7 °C; FT-IR (KBr) ν_{max}: 3433, 1635, 1612, 1581, 1458, 1350, 995, 810, 756, 694 cm^{-1}; ^1H NMR (500 MHz, C_6D_6): δ 8.87 (dd, *J* = 15.5, 11.5 Hz, 1H, H-4), 7.43–7.35 (m, 2H, Ar), 7.06–6.93 (m, 3H, Ar), 6.56 (d, *J* = 15.5 Hz, 1H, H-5), 6.51 (dd, *J* = 11.5, 11.5 Hz, 1H, H-3), 6.26 (d, *J* = 11.5 Hz, 1H, H-2), 3.06 (s, 3H, OMe), 2.95 (s, 3H, NMe); $^{13}C\{^1H\}$ NMR (125 MHz, C_6D_6): 167.4 (C-1), 143.0 (C-3), 140.5 (C-5), 137.1 (Ar), 128.9 (Ar), 128.7 (Ar), 127.7 (Ar), 126.3 (C-4), 116.3 (C-2), 61.0 (OMe), 31.9 (NMe); HRMS calcd for $C_{13}H_{15}NO_2Na$ $[M + Na]^+$ 240.0995, found 240.0990.

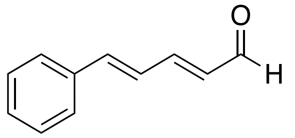

(2*E*,4*E*)-5-Phenylpenta-2,4-dienal (17f, (2*E*,4*E*)-17f).
To a solution of Weinreb amide (*E*)-**18d** (4.07 g, 18.7 mmol) in tetrahydrofuran (94 mL), a 1.0 M solution of diisobutylaluminum hydride in hexane (20.9 mL, 21.5 mmol) was added at –78 °C. After the reaction mixture was stirred at –78 °C for 1.5 h, saturated aqueous Rochelle salt was added, and the mixture was stirred at room temperature. The mixture was extracted with ethyl acetate, and the organic layer was washed with brine and dried over sodium sulfate. After filtration of the mixture and concentration of the solvent, the crude mixture was purified by flash column chromatography (eluant: hexane/ethyl acetate = 10/1 to 6/1) to afford aldehyde **17f** (2.73 g, 92%) as yellow solid.
R_f = 0.68 (silica gel, hexane/ethyl acetate = 2/1); mp: 32.8 °C; FT-IR (KBr) ν_{max}: 3379, 1674, 1620, 1157, 1111, 748 cm^{-1}; ^1H NMR (500 MHz, C_6D_6): δ 9.46 (dd, *J* = 7.5, 1.0 Hz, 1H, H-1), 7.13–6.98 (m, 5H, Ar), 6.49 (ddd, *J* = 15.0, 9.5, 1.0 Hz, 1H, H-3), 6.46–6.32 (m, 2H, H-4, H-5), 6.03 (dd, *J* = 15.0, 7.5 Hz, 1H, H-2); $^{13}C\{^1H\}$ NMR (125 MHz, C_6D_6): 192.3 (C-1), 150.9 (C-3), 141.5 (C-5), 136.1 (Ar), 132.0 (C-2), 129.5 (Ar), 129.0 (Ar), 127.7 (Ar), 126.5 (C-4); HRMS calcd for $C_{11}H_{11}O$ $[M + H]^+$ 159.0804, found 159.0809.

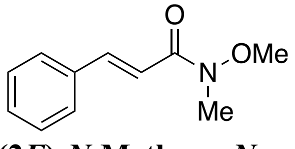

(2*E*)-*N*-Methoxy-*N*-methyl-3-phenylprop-2-enamide ((*E*)-5b, (*E*)-20a).
To a solution of phosphate **1** (13.5 g, 56.5 mmol) in tetrahydrofuran (80 mL), a 1.3 M solution of lithium bis(trimethylsilyl)amide in tetrahydrofuran (39.9 mL, 51.8 mmol) was added at –78 °C. After the reaction mixture was stirred at –78 °C for 30 min, a solution of benzaldehyde (5.00 g, 47.1 mmol) in tetrahydrofuran (14 mL) was added at room temperature, and the reaction mixture was stirred for 1 h. To the reaction mixture, saturated aqueous ammonium chloride was added at 0 °C, and the mixture was extracted with ethyl acetate. The organic layer was dried over sodium sulfate. After filtration of the mixture and concentration of the solvent, the crude mixture was purified by flash column chromatography (eluant: hexane/ethyl acetate = 3/1 to 2/1) to afford alkene (*E*)-**5b** (8.71 g, 97%) as

white solid.
R_f= 0.36 (silica gel, hexane/ethyl acetate = 2/1); mp: 36.6 °C; FT-IR (KBr) ν_{max}: 2939, 1651, 1612, 1574, 1381, 1173, 995, 756, 532 cm^{-1}; ^{1}H NMR (500 MHz, $CDCl_3$): δ 7.74 (d, J = 15.5 Hz, 1H, H-3), 7.63–7.54 (m, 2H, Ar), 7.44–7.33 (m, 3H, Ar), 7.05 (d, J = 15.5 Hz, 1H, H-2), 3.78 (s, 3H, OMe), 3.32 (s, 3H, NMe); ^{13}C{^{1}H} NMR (125 MHz, $CDCl_3$): 166.9 (C-1), 143.4 (C-3), 135.1 (Ar), 129.8 (Ar), 128.8 (Ar), 128.0 (Ar), 115.7 (C-2), 61.9 (OMe), 32.5 (NMe); HRMS calcd for $C_{11}H_{13}NO_2Na$ [M + Na]$^+$ 214.0838, found 214.0840.

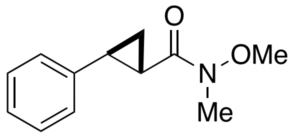

(1*SR*,2*SR*)-*N*-Methoxy-*N*-methyl-2-phenylcyclopropane-1-carboxamide (*trans*-22).
Ref. 16

To a solution of trimethylsulfoxonium iodide (20.0 g, 91.1 mmol) in dimethyl sulfoxide (76 mL), sodium hydride (55% dispersion in mineral oil) (3.98 g, 91.1 mmol) was added at 0 °C. After the mixture was stirred at 0 °C for 30 min, a solution of alkene (*E*)-**5b** (8.71 g, 45.5 mmol) in dimethyl sulfoxide (15 mL) was added to the mixture at 0 °C. The reaction mixture was stirred at room temperature for 4 h. To the reaction mixture, saturated aqueous ammonium chloride was added at 0 °C, and the mixture was extracted with ethyl acetate. The organic layer was washed with brine and dried over sodium sulfate. After filtration of the mixture and concentration of the solvent, the crude mixture was purified by flash column chromatography (eluant: hexane/ethyl acetate = 4/1 to 2/1) to afford cyclopropane *trans*-**22** (8.21 g, 88%) as white solid.

Weinreb amide *trans*-**22** is solid at room temperature, but melting point was 25–30 °C to make immeasurable accurately.

R_f= 0.39 (silica gel, hexane/ethyl acetate = 2/1); ATR-IR ν_{max}: 2936, 1653, 1462, 1441, 1422, 1369, 995, 750, 699 cm^{-1}; ^{1}H NMR (500 MHz, $CDCl_3$): δ 7.32–7.24 (m, 2H, Ar), 7.23–7.17 (m, 1H, Ar), 7.16–7.11 (m, 2H, Ar), 3.69 (s, 3H, OMe), 3.24 (s, 3H, NMe), 2.51 (ddd, J = 9.5, 6.5, 4.0 Hz, 1H, H-3), 2.47–2.35 (m, 1H, H-2), 1.63 (ddd, J = 9.5, 5.5, 4.0 Hz, 1H, CH_2), 1.31 (ddd, J = 8.5, 6.5, 4.0 Hz, 1H, CH_2); ^{13}C{^{1}H} NMR (125 MHz, $CDCl_3$): 173.0 (C-1), 140.7 (Ar), 128.4 (Ar), 126.21 (Ar), 126.16 (Ar), 61.6 (OMe), 32.5 (NMe), 25.8 (C-3), 21.5 (C-2), 16.4 (CH_2); HRMS calcd for $C_{12}H_{15}NO_2Na$ [M + Na]$^+$ 228.0995, found 228.0986.

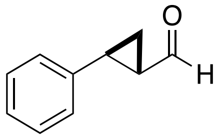

(1*SR*,2*SR*)-2-Phenylcyclopropane-1-carbaldehyde (15r, *trans*-15r).

To a solution of Weinreb amide *trans*-**22** (267 mg, 1.30 mmol) in tetrahydrofuran (4.3 mL), a 1.0 M solution of diisobutylaluminum hydride in hexane (1.7 mL, 1.70 mmol) was added at –78 °C. After the reaction mixture was stirred at –78 °C for 15 min, methanol and saturated aqueous Roshelle salt were added, and mixture was stirred at room temperature. The mixture was extracted with ethyl acetate, and the organic layer was dried over sodium sulfate. After filtration of the mixture and concentration of the solvent, almost pure aldehyde **15r** (purity: 90.7%, calculated from ^{1}H NMR spectra using 1,1,2,2-tetrachloroethane as the internal standard) was obtained and used in the next HWE reaction without further purification.

R_f= 0.64 (silica gel, hexane/ethyl acetate = 2/1)

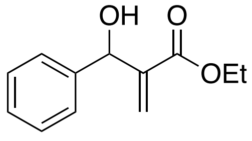

Ethyl 3-hydroxy-2-methylene-3-phenylpropanoate (S7).
Ref. 17

The mixture of benzaldehyde (5.10 g, 48.1 mmol), ethyl acrylate (12.8 mL, 118 mmol) and 1,4-diazabicyclo[2.2.2]octane (5.30 g, 47.1 mmol) was stirred at room temperature for 125 h. The crude product was concentrated and purified by flash column chromatography (eluant: hexane/ethyl acetate = 5/1 to 3/1) to afford *α,β*-unsaturated ester **S7** (8.15 g, 84%) as colorless oil.

R_f= 0.65 (silica gel, hexane/ethyl acetate = 2/1); FT-IR (neat) ν_{max}: 3448, 1712, 1273, 1149, 1041, 702 cm^{-1}; ^{1}H NMR (500 MHz, $CDCl_3$): δ 7.41–7.32 (m, 4H, Ar), 7.31–7.25 (m, 1H, Ar), 6.34 (dd, *J* = 1.0, 1.0 Hz, 1H, C=C*H₂*), 5.81 (dd, *J* = 1.0, 1.0 Hz, 1H, C=C*H₂*), 5.59–5.54 (m, 1H, H-3), 4.18 (q, *J* = 7.0 Hz, 2H, OEt), 3.03 (d, *J* = 5.5 Hz, 1H, 3-OH), 1.25 (t, *J* = 7.0Hz, 3H, OEt); ^{13}C{^{1}H} NMR (125 MHz, $CDCl_3$): 166.3 (C-1), 142.1 (C-2), 141.3 (Ar), 128.4 (Ar), 127.7 (Ar), 126.5 (Ar), 125.8 (C=*C*H₂), 73.3 (C-3), 60.9 (OEt), 14.0 (OEt); HRMS calcd for $C_{12}H_{14}O_3Na$ $[M + Na]^+$ 229.0835, found 229.0833.

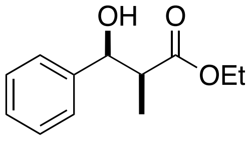

Ethyl (2*SR*,3*SR*)-3-hydroxy-2-methyl-3-phenylpropanoate (*syn*-S8).

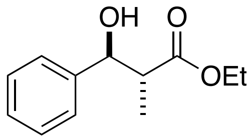

Ethyl (2*RS*,3*SR*)-3-hydroxy-2-methyl-3-phenylpropanoate (*anti*-S8).
Ref. 18, 19
Bach 1

α,β-Unsaturated **S7** (1.90 g, 9.21 mmol) was dissolved in ethyl acetate (36.8 mL) and placed under an argon atmosphere. After addition of magnesium bromide (2.54 g, 13.8 mmol), the mixture was stirred at room temperature for 15 min. To the reaction mixture, palladium 10% on carbon (735 mg, 0.691 mmol) was added, and the argon was replaced with hydrogen atmosphere (1 bar), and the slurry was stirred at room temperature for 3.5 h. After the catalyst was removed by filtration through a bed of celite and washed with ethyl acetate, the combined solution was concentrated, and the crude mixture-1 was obtained.

Bach 2

α,β-Unsaturated **S7** (1.89 g, 9.16 mmol) was dissolved in ethyl acetate (36.6 mL) and placed under an argon atmosphere. After addition of magnesium bromide (2.52 g, 13.7 mmol), the mixture was stirred at room temperature for 15 min. To the reaction mixture, palladium 10% on carbon (731 mg, 0.687 mmol) was added, and the argon was replaced with hydrogen atmosphere (1 bar), and the slurry was stirred at room temperature for 3.5 h. After the catalyst was removed by filtration through a bed of celite and washed with ethyl acetate, the combined solution was concentrated, and the crude mixture-2 was obtained.

Bach 3

α,β-Unsaturated **S7** (1.89 g, 9.16 mmol) was dissolved in ethyl acetate (36.6 mL) and placed under an argon atmosphere. After addition of magnesium bromide (2.52 g, 13.7 mmol), the mixture was stirred at room temperature for 15 min. To the reaction mixture, palladium 10% on carbon (731 mg, 0.687 mmol) was added, and the argon was replaced with hydrogen atmosphere (1 bar), and the slurry was stirred at room temperature for 3.5 h. After the catalyst was removed by filtration through a bed of celite and washed with ethyl acetate, the combined solution was concentrated, and the crude mixture-3 was obtained.

Bach 4

α,β-Unsaturated **S7** (1.76 g, 8.53 mmol) was dissolved in ethyl acetate (34.1 mL) and placed under an argon atmosphere. After addition of magnesium bromide (2.36 g, 12.8 mmol), the mixture was stirred at room temperature for 15 min. To the reaction mixture, palladium 10% on carbon (681 mg, 0.640 mmol) was added, and the argon was replaced with hydrogen atmosphere (1 bar), and the slurry was stirred at room temperature for 3.5 h. After the catalyst was removed by filtration through a bed of celite and washed with ethyl acetate, the combined solution was concentrated, and the crude mixture-4 was obtained.

Bach 1–4

After the crude mixture-1, the crude mixture-2, the crude mixture-3 and the crude mixture-4 were mixed, the crude mixture was dissolved in dichloromethane and water added. The mixture was extracted with dichloromethane, and the organic layer was dried over sodium sulfate. After filtration of the mixture and concentration of the solvent, the crude mixture was purified by open column chromatography (eluant: hexane/ethyl acetate = 2/1) to afford the mixture of *syn*-**S8**, *anti*-**S8** and *α,β*-unsaturated **S7**. The mixture of *syn*-**S8**, *anti*-**S8** and *α,β*-unsaturated **S7** was purified by flash column chromatography (eluant: hexane/ethyl acetate/methanol = 70/10/1 to 60/10/1) to afford *syn*-**S8** (3.12 g, 42%) as colorless oil, *anti*-**S8** (2.07 g, 28%) as colorless oil and *α,β*-unsaturated **S7** (580 mg, 7.8%).

Ethyl (2*SR*,3*SR*)-3-hydroxy-2-methyl-3-phenylpropanoate (*syn*-S8).

R_f= 0.43 (silica gel, hexane/ethyl acetate/methanol = 50/10/1); FT-IR (neat) ν_{max}: 3456, 1720, 1188, 1034, 702 cm^{-1}; ^{1}H NMR (500 MHz, $CDCl_3$): *δ* 7.39–7.31 (m, 4H, Ar), 7.30–7.24 (m, 1H, Ar), 5.10 (dd, *J* = 4.0, 3.0 Hz, 1H, H-3), 4.13 (q, *J* = 7.0 Hz, 2H, OEt), 2.95 (d, *J* = 3.0 Hz, 1H, 3-OH), 2.78 (qd, *J* = 7.0, 4.0 Hz, 1H, H-2), 1.22 (t, *J* = 7.0 Hz, 3H, OEt), 1.13 (d, *J* = 7.0 Hz, 3H, 2-Me); ^{13}C{^{1}H} NMR (125 MHz, $CDCl_3$): 175.8 (C-1), 141.4 (Ar), 128.2 (Ar), 127.4 (Ar), 126.0 (Ar), 73.6 (C-3), 60.7 (OEt), 46.4 (C-2), 14.0 (OEt), 10.8 (2-Me); HRMS calcd for $C_{24}H_{32}O_6Na$ [2M + Na]$^+$ 439.2091, found 439.2076.

Ethyl (2*RS*,3*SR*)-3-hydroxy-2-methyl-3-phenylpropanoate (*anti*-S8).

R_f= 0.40 (silica gel, hexane/ethyl acetate/methanol = 50/10/1); FT-IR (neat) ν_{max}: 3456, 1728, 1180, 1026, 702 cm^{-1}; ^{1}H NMR (500 MHz, $CDCl_3$): *δ* 7.39–7.27 (m, 5H, Ar), 4.76 (dd, *J* = 8.5, 4.5 Hz, 1H, H-3), 4.19 (q, *J* = 7.5 Hz, 2H, OEt), 2.98 (d, *J* = 4.5 Hz, 1H, 3-OH), 2.81 (dq, *J* = 8.5, 7.5 Hz, 1H, H-2), 1.26 (t, *J* = 7.5 Hz, 3H, OEt), 1.03 (d, *J* = 7.5 Hz, 3H, 2-Me); ^{13}C{^{1}H} NMR (125 MHz, $CDCl_3$): 175.8 (C-1), 141.6 (Ar), 128.4 (Ar), 128.0 (Ar), 126.6 (Ar), 76.3 (C-3), 60.7 (OEt), 47.1 (C-2), 14.5 (2-Me), 14.1 (OEt); HRMS calcd for $C_{24}H_{32}O_6Na$ [2M + Na]$^+$ 439.2091, found 439.2071.

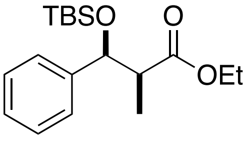

Ethyl (2*SR*,3*SR*)-3-((*tert*-butyldimethylsilyl)oxy)-2-methyl-3-phenylpropanoate (*syn*-S9).

To a solution of alcohol *syn*-**S8** (3.22 g, 15.4 mmol) in dichloromethane (77 mL), 2,6-lutidine (5.3 mL, 46.4 mmol) and *tert*-butyldimethylsilyl trifluoromethanesulfonate (7.1 mL, 30.9 mmol) were added at 0 °C. After the reaction mixture was stirred at 0 °C for 30 min, saturated aqueous ammonium chloride was added. The mixture was extracted with dichloromethane, and the organic layer was dried over sodium sulfate. After filtration of the mixture and concentration of the solvent, the crude mixture was purified by flash column chromatography (eluant: hexane/ethyl acetate = 12/1) to afford TBS ether *syn*-**S9** (5.03 g, quant.) as colorless oil.

R_f= 0.76 (silica gel, hexane/ethyl acetate = 4/1); FT-IR (neat) ν_{max}: 2954, 2939, 1736, 1257, 1088, 1065, 841, 779 cm^{-1}; ^{1}H NMR (400 MHz, $CDCl_3$): *δ* 7.33–7.19 (m, 5H, Ar), 4.96 (d, *J* = 6.0 Hz, 1H, H-3), 4.07–3.90 (m, 2H, OEt), 2.65 (qd, *J* = 6.8, 6.0 Hz, 1H, H-2), 1.17 (d, *J* = 6.8 Hz, 3H, 2-Me), 1.10 (t, *J* = 6.8 Hz, 3H, OEt), 0.87 (s, 9H, TBS), 0.02 (s, 3H, TBS), –0.22 (s, 3H, TBS); ^{13}C{^{1}H} NMR (100 MHz, $CDCl_3$): 174.3 (C-1), 143.2 (Ar), 127.9 (Ar), 127.3 (Ar), 126.5 (Ar), 76.1 (C-3), 60.2 (OEt), 49.2 (C-2), 25.7 (TBS), 18.1 (TBS), 14.0 (OEt), 11.8 (2-Me), –4.6 (TBS), –5.3 (TBS); HRMS calcd for

$C_{18}H_{30}O_3SiNa$ [M + Na]$^+$ 345.1856, found 345.1864.

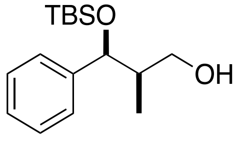

(2*RS*,3*SR*)-3-((*tert*-Butyldimethylsilyl)oxy)-2-methyl-3-phenylpropan-1-ol (*syn*-S10).

To a solution of ester *syn*-**S9** (4.92 g, 15.2 mmol) in dichloromethane (51 mL), a 1.0 M solution of diisobutylaluminium hydride in hexane (37.4 mL, 38.1 mmol) was added at 0 °C. After the reaction mixture was stirred at 0 °C for 45 min, saturated aqueous Rochelle salt was added, and the mixture was stirred at room temperature. The mixture was extracted with dichloromethane, and the organic layer was washed with brine and dried over sodium sulfate. After filtration of the mixture and concentration of the solvent, the crude mixture was purified by flash column chromatography (eluant: hexane/ethyl acetate = 20/1) to afford alcohol *syn*-**S10** (4.19 g, 98%) as colorless oil.

R_f= 0.59 (silica gel, hexane/ethyl acetate = 4/1); FT-IR (neat) ν_{max}: 3402, 2954, 2931, 2885, 2862, 1466, 1381, 1257, 1095, 1034, 841 cm^{-1}; ^{1}H NMR (400 MHz, $CDCl_3$): δ 7.35–7.21 (m, 5H, Ar), 4.83 (d, J = 4.4 Hz, 1H, H-3), 3.58 (dd, J = 10.8, 8.0 Hz, 1H, H-1), 3.44 (dd, J = 10.8, 4.8 Hz, 1H, H-1), 2.22 (brs, 1H, 1-OH), 2.12–2.00 (m, 1H, H-2), 0.91 (s, 9H, TBS), 0.78 (d, J = 7.2 Hz, 3H, 2-Me), 0.05 (s, 3H, TBS), –0.18 (s, 3H, TBS); $^{13}C\{^1H\}$ NMR (100 MHz, $CDCl_3$): 142.3 (Ar), 127.8 (Ar), 127.1 (Ar), 126.7 (Ar), 77.5 (C-3), 65.5 (C-1), 42.9 (C-2), 25.8 (TBS), 18.1 (TBS), 11.9 (2-Me), –4.7 (TBS), –5.3 (TBS); HRMS calcd for $C_{16}H_{28}O_2SiNa$ [M + Na]$^+$ 303.1751, found 303.1751.

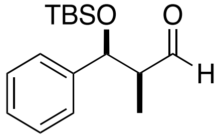

(2*SR*,3*SR*)-3-((*tert*-Butyldimethylsilyl)oxy)-2-methyl-3-phenylpropanal (15p).

To a mixture of dichloromethane (60 mL) and dimethyl sulfoxide (2.79 mL, 36.1 mmol), oxalyl chloride (1.89 mL, 21.7 mmol) was added dropwise at –78 °C. After the mixture was stirred at –78 °C for 30 min, a solution of alcohol *syn*-**S10** (4.05 g, 14.4 mmol) in dichloromethane (12 mL) and triethylamine (6.04 mL, 43.3 mmol) were added at –78 °C. The reaction mixture was stirred at room temperature for 15 min. To the reaction mixture, saturated aqueous sodium hydrogen carbonate was added at 0 °C, and the mixture was extracted with dichloromethane. The organic layer was washed with water and brine. The combined organic layer was dried over sodium sulfate, filtered, and concentrated. The crude mixture was purified by flash column chromatography (eluant: hexane/ethyl acetate = 25/1) to afford aldehyde **15p** (3.72 g, 93%) as colorless oil.

R_f= 0.74 (silica gel, hexane/ethyl acetate = 4/1); FT-IR (neat) ν_{max}: 3448, 2954, 2931, 1728, 1257, 1034, 841, 779, 702 cm^{-1}; ^{1}H NMR (500 MHz, $CDCl_3$): δ 9.73 (d, J = 1.0 Hz, 1H, H-1), 7.32–7.18 (m, 5H, Ar), 5.12 (d, J = 4.5 Hz, 1H, H-3), 2.55 (qdd, J = 6.5, 4.5, 1.0 Hz, 1H, H-2), 1.00 (d, J = 6.5 Hz, 3H, 2-Me), 0.85 (s, 9H, TBS), 0.00 (s, 3H, TBS), –0.21 (s, 3H, TBS); $^{13}C\{^1H\}$ NMR (125 MHz, $CDCl_3$): 204.3 (C-1), 142.2 (Ar), 128.1 (Ar), 127.4 (Ar), 126.2 (Ar), 74.2 (C-3), 54.7 (C-2), 25.7 (TBS), 18.1 (TBS), 7.9 (2-Me), –4.6 (TBS), –5.3 (TBS); HRMS calcd for $C_{16}H_{26}O_2SiNa$ [M + Na]$^+$ 301.1594, found 301.1609.

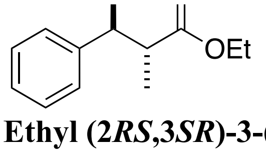

Ethyl (2*RS*,3*SR*)-3-((*tert*-butyldimethylsilyl)oxy)-2-methyl-3-phenylpropanoate (*anti*-S9).

To a solution of alcohol *anti*-**S8** (1.85 g, 8.88 mmol) in dichloromethane (44 mL), 2,6-lutidine (3.1 mL, 26.7 mmol) and *tert*-butyldimethylsilyl trifluoromethanesulfonate (4.1 mL, 17.8 mmol) were added at 0 °C. After the reaction mixture was stirred at 0 °C for 25 min, saturated aqueous ammonium chloride

was added. The mixture was extracted with dichloromethane, and the organic layer was dried over sodium sulfate. After filtration of the mixture and concentration of the solvent, the crude mixture was purified by flash column chromatography (eluant: hexane/ethyl acetate = 12/1) to afford TBS ether *anti*-**S9** (2.81 g, 98%) as colorless oil.

R_f = 0.74 (silica gel, hexane/ethyl acetate = 4/1); FT-IR (neat) ν_{max}: 2954, 2939, 1736, 1257, 1173, 1080, 856, 841, 779 cm^{-1}; ^{1}H NMR (400 MHz, $CDCl_3$): δ 7.34–7.22 (m, 5H, Ar), 4.71 (d, J = 9.2 Hz, 1H, H-3), 4.24–4.08 (m, 2H, OEt), 2.73 (dq, J = 9.2, 7.2 Hz, 1H, H-2), 1.29 (t, J = 7.2 Hz, 3H, OEt), 0.84 (d, J = 7.2 Hz, 3H, 2-Me), 0.80 (s, 9H, TBS), –0.02 (s, 3H, TBS), –0.30 (s, 3H, TBS); ^{13}C{^{1}H} NMR (100 MHz, $CDCl_3$): 175.4 (C-1), 142.3 (Ar), 128.1 (Ar), 127.7 (Ar), 127.1 (Ar), 77.7 (C-3), 60.3 (OEt), 49.4 (C-2), 25.6 (TBS), 18.0 (TBS), 14.2 (2-Me), 13.9 (OEt), –4.7 (TBS), –5.4 (TBS); HRMS calcd for $C_{18}H_{30}O_3SiNa$ [M + Na]$^+$ 345.1856, found 345.1860.

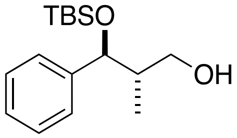

(2*SR*,3*SR*)-3-((*tert*-Butyldimethylsilyl)oxy)-2-methyl-3-phenylpropan-1-ol (*anti*-S10).

To a solution of ester *anti*-**S9** (2.70 g, 8.37 mmol) in dichloromethane (42 mL), a 1.0 M solution of diisobutylaluminium hydride in hexane (20.5 mL, 20.9 mmol) was added at 0 °C. After the reaction mixture was stirred at 0 °C for 45 min, saturated aqueous Rochelle salt was added, and the mixture was stirred at room temperature. The mixture was extracted with dichloromethane, and the organic layer was washed with brine and dried over sodium sulfate. After filtration of the mixture and concentration of the solvent, the crude mixture was purified by flash column chromatography (eluant: hexane/ethyl acetate = 20/1) to afford alcohol *anti*-**S10** (2.20 g, 94%) as colorless oil.

R_f = 0.65 (silica gel, hexane/ethyl acetate = 4/1); FT-IR (neat) ν_{max}: 3433, 2954, 2931, 2893, 2862, 1466, 1257, 1080, 1065, 1034, 841, 779, 702 cm^{-1}; ^{1}H NMR (400 MHz, $CDCl_3$): δ 7.36–7.21 (m, 5H, Ar), 4.56 (d, J = 6.8 Hz, 1H, H-3), 3.68 (dd, J = 10.8, 3.6 Hz, 1H, H-1), 3.60 (dd, J = 10.8, 6.4 Hz, 1H, H-1), 2.64 (brs, 1H, 1-OH), 1.93 (qddd, J = 7.2, 6.8, 6.4, 3.6 Hz, 1H, H-2), 0.89 (s, 9H, TBS), 0.85 (d, J = 7.2 Hz, 3H, 2-Me), 0.04 (s, 3H, TBS), –0.25 (s, 3H, TBS); ^{13}C{^{1}H} NMR (100 MHz, $CDCl_3$): 143.6 (Ar), 128.1 (Ar), 127.4 (Ar), 126.7 (Ar), 81.0 (C-3), 66.3 (C-1), 43.1 (C-2), 25.8 (TBS), 18.0 (TBS), 14.3 (2-Me), –4.6 (TBS), –5.2 (TBS); HRMS calcd for $C_{16}H_{28}O_2SiNa$ [M + Na]$^+$ 303.1751, found 303.1751.

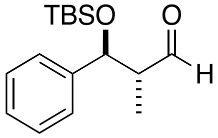

(2*RS*,3*SR*)-3-((*tert*-Butyldimethylsilyl)oxy)-2-methyl-3-phenylpropanal (15q).

To a mixture of dichloromethane (42 mL) and dimethyl sulfoxide (1.45 mL, 18.7 mmol), oxalyl chloride (0.98 mL, 11.2 mmol) was added dropwise at –78 °C. After the mixture was stirred at –78 °C for 30 min, a solution of alcohol *anti*-**S10** (2.10 g, 7.48 mmol) in dichloromethane (8 mL) and triethylamine (3.14 mL, 22.5 mmol) were added at –78 °C. The reaction mixture was stirred at room temperature for 15 min. To the reaction mixture, saturated aqueous sodium hydrogen carbonate was added at 0 °C, and the mixture was extracted with dichloromethane. The organic layer was washed with water and brine. The combined organic layer was dried over sodium sulfate, filtered, and concentrated. The crude mixture was purified by flash column chromatography (eluant: hexane/ethyl acetate = 25/1) to afford aldehyde **15q** (2.03 g, 98%) as colorless oil.

R_f = 0.76 (silica gel, hexane/ethyl acetate = 4/1); FT-IR (neat) ν_{max}: 3440, 2954, 2931, 1728, 1458, 1389, 1257, 1080, 841, 779, 702 cm^{-1}; ^{1}H NMR (500 MHz, $CDCl_3$): δ 9.77 (d, J = 2.5 Hz, 1H, H-1), 7.33–7.19 (m, 5H, Ar), 4.72 (d, J = 7.0 Hz, 1H, H-3), 2.65 (dqd, J = 7.0, 7.0, 2.5 Hz, 1H, H-2), 0.84 (d, J = 7.0 Hz, 3H, 2-Me), 0.81 (s, 9H, TBS), –0.03 (s, 3H, TBS), –0.30 (s, 3H, TBS); ^{13}C{^{1}H} NMR (125 MHz, $CDCl_3$): 204.5 (C-1), 142.2 (Ar), 128.3 (Ar), 127.8 (Ar), 126.6 (Ar), 76.8 (C-3), 54.5 (C-2), 25.7

(TBS), 18.0 (TBS), 11.1 (2-Me), –4.6 (TBS), –5.3 (TBS); HRMS calcd for $C_{16}H_{26}O_2SiNa$ $[M + Na]^+$ 301.1594, found 301.1597.

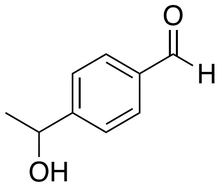

4-(1-Hydroxyethyl)benzaldehyde (S11).

To a solution of benzene-1,4-dicarbaldehyde (10.0 g, 74.5 mmol) in tetrahydrofuran (373 mL), a 1.0 M solution of methylmagnesium bromide in tetrahydrofuran (71.7 mL, 74.5 mmol) was added at –90 °C. The reaction mixture was stirred for 1 h while the reaction temperature was gradually raised from –90 °C to room temperature. To the reaction mixture, saturated aqueous ammonium chloride was added at 0 °C, and the mixture was extracted with ethyl acetate. The organic layer was dried over sodium sulfate. After filtration of the mixture and concentration of the solvent, the crude mixture was purified by flash column chromatography (eluant: hexane/ethyl acetate = 6/1 to 3/1) to afford alcohol **S11** (3.40 g, 30%) as colorless oil.

R_f= 0.42 (silica gel, hexane/ethyl acetate = 1/1); FT-IR (neat) ν_{max}: 3410, 1697, 1604, 1211, 1088, 833 cm^{-1}; ^{1}H NMR (500 MHz, $CDCl_3$): δ 10.00 (s, 1H, H-1), 7.91–7.84 (m, 2H, Ar), 7.58–7.51 (m, 2H, Ar), 5.04–4.95 (m, 1H, C*H*(OH)CH_3), 2.04–1.88 (m, 1H, OH), 1.52 (d, *J* = 6.5 Hz, 3H, CH(OH)C*H*$_3$); $^{13}C\{^1H\}$ NMR (125 MHz, $CDCl_3$): 192.0 (C-1), 152.7 (Ar), 135.6 (Ar), 130.0 (Ar), 125.9 (Ar), 69.9 (*C*H(OH)CH_3), 25.4 (CH(OH)*C*H_3); HRMS calcd for $C_9H_{10}O_2Na$ $[M + Na]^+$ 173.0573, found 173.0567.

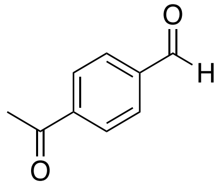

4-Acetylbenzaldehyde (19n).

To a solution of alcohol **S11** (3.35 g, 22.3 mmol) in dichloromethane (45 mL), 4-methylmorpholine *N*-oxide (7.86 g, 66.9 mmol) and tetrapropylammonium perruthenate (784 mg, 2.23 mmol) were added at 0 °C. After the reaction mixture was stirred at 0 °C for 30 min, the mixture was purified by open column chromatography (eluant: hexane/ethyl acetate = 1/2) to afford the crude ketone **19n** (2.00 g). The crude ketone **19n** was purified by flash column chromatography (eluant: hexane/ethyl acetate = 4/1 to 3/1) to afford ketone **19n** (1.63 g, 49%) as white solid.

Weinreb amide **19n** is solid at room temperature, but melting point was 25–30 °C to make immeasurable accurately.

R_f= 0.61 (silica gel, hexane/ethyl acetate = 1/1); FT-IR (neat) ν_{max}: 1689, 1265, 1203, 833, 710 cm^{-1}; ^{1}H NMR (500 MHz, $CDCl_3$): δ 10.12 (s, 1H, H-1), 8.14–8.08 (m, 2H, Ar), 8.02–7.96 (m, 2H, Ar), 2.67 (s, 3H, COC*H*$_3$); $^{13}C\{^1H\}$ NMR (125 MHz, $CDCl_3$): 197.3 (*C*OCH_3), 191.5 (C-1), 141.2 (Ar), 139.0 (Ar), 129.8 (Ar), 128.8 (Ar), 26.9 (CO*C*H_3); HRMS calcd for $C_9H_8O_2Na$ $[M + Na]^+$ 171.0417, found 171.0419.

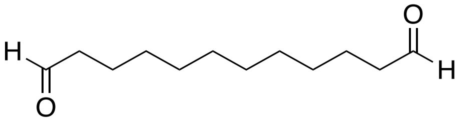

1,12-Dodecanedial (15k).

To a solution of 1,12-dodecanediol (3.01 g, 14.9 mmol) in dichloromethane (118 mL), dimethyl sulfoxide (29.6 mL) and triethylamine (16.5 mL, 119 mmol) were added. After sulfur trioxide-pyridine complex (9.44 g, 59.3 mmol) was added to the mixture at 0 °C, the reaction mixture was stirred at room temperature for 40 min. To the reaction mixture, saturated aqueous ammonium chloride was added at

0 °C, and the mixture was extracted with dichloromethane. The organic layer was washed with brine and dried over sodium sulfate. After filtration of the mixture and concentration of the solvent, the crude mixture was purified by flash column chromatography (eluant: hexane/ethyl acetate = 7/1 to 5/1) to afford dialdehyde **15k** (2.31 g, 79%) as white solid.

R_f = 0.79 (silica gel, hexane/ethyl acetate = 1/1); mp: 33.3 °C; ATR-IR ν_{max}: 2926, 2916, 2851, 1711, 1471, 1411, 1393 cm^{-1}; ^{1}H NMR (500 MHz, $CDCl_3$): δ 9.75 (t, J = 2.0 Hz, 2H, H-1, H-12), 2.40 (td, J = 7.0, 2.0 Hz, 4H, H-2, H-11), 1.61 (tt, J = 7.5, 7.0 Hz, 4H, H-3, H-10), 1.40–1.17 (m, 12H, H-4, H-5, H-6, H-7, H-8, H-9); ^{13}C{^{1}H} NMR (125 MHz, $CDCl_3$): 202.9 (C-1, C-12), 43.8 (C-2, C-11), 29.26 (C-4 or C-5 or C-6 or C-7 or C-8 or C-9), 29.25 (C-4 or C-5 or C-6 or C-7 or C-8 or C-9), 29.1 (C-4 or C-5 or C-6 or C-7 or C-8 or C-9), 22.0 (C-3, C-10); HRMS calcd for $C_{12}H_{22}O_2Na$ [M + Na]$^+$ 221.1512, found 221.1514.

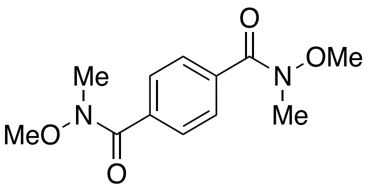

N^1,N^4-Dimethoxy-N^1,N^4-dimethylterephthalamide (S12).

Ref. 20

To a suspension of potassium carbonate anhydrous (16.3 g, 118 mmol) in a mixed solvent of diethyl ether (33 mL) and water (33 mL), *N*,*O*-dimethylhydroxylamine hydrochloride (11.5 g, 118 mmol) was added at 0 °C. After the mixture was stirred vigorously at room temperature for 10 min, benzene-1,4-dicarbonyl dichloride (10.0 g, 49.2 mmol) was added little by little at 0 °C. The reaction mixture was stirred at room temperature for 1 h. The mixture was diluted with water and filtered, and the white solid was washed with water to afford terephthalamide **S12** (12.2 g, 99%).

R_f = 0.13 (silica gel, hexane/ethyl acetate = 1/1); mp: 167.2 °C; ATR-IR ν_{max}: 1633, 1461, 1423, 1386, 1219, 968, 717 cm^{-1}; ^{1}H NMR (500 MHz, $CDCl_3$): δ 7.71 (s, 4H, Ar), 3.54 (s, 6H, OMe), 3.37 (s, 6H, NMe); ^{13}C{^{1}H} NMR (125 MHz, $CDCl_3$): 169.1 (C-1), 136.0 (Ar), 127.8 (Ar), 61.2 (OMe), 33.5 (NMe); HRMS calcd for $C_{12}H_{16}N_2O_4Na$ [M + Na]$^+$ 275.1002, found 275.0991.

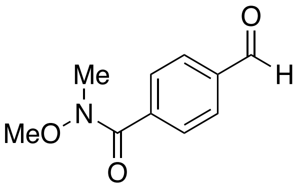

4-Formyl-*N*-methoxy-*N*-methylbenzamide (19m).

Ref. 21

<u>Preparation of sodium diisobutyl-*tert*-butoxyaluminum hydride</u>

To a suspension of sodium *tert*-butoxide (9.08 g, 94.5 mmol) in tetrahydrofuran (44 mL), a 1.0 M solution of diisobutylaluminium hydride in hexane (87.4 mL, 90.0 mmol) was added at 0 °C over 25 min. The mixture was stirred at room temperature for 2 h. A 0.69 M solution of sodium diisobutyl-*tert*-butoxyaluminum hydride in a mixed solvent of hexane and tetrahydrofuran was obtained and used in the next step without further purification.

<u>Reduction with sodium diisobutyl-*tert*-butoxyaluminum hydride</u>

To a solution of terephthalamide **S12** (10.1 g, 40.0 mmol) in dichloromethane (198 mL), a 0.69 M solution of sodium diisobutyl-*tert*-butoxyaluminum hydride in a mixed solvent of hexane and tetrahydrofuran (97.5 mL, 67.3 mmol) was added at –78 °C over 10 min. After the reaction mixture was stirred at –78 °C for 4 min, methanol and saturated aqueous Rochelle salt were added, and the mixture was stirred at room temperature. The mixture was extracted with dichloromethane, and the organic layer was washed with brine and dried over sodium sulfate. After filtration of the mixture and concentration of the solvent, the crude mixture was purified by flash column chromatography (eluant: hexane/ethyl

acetate = 3/1 to 1/1) to afford almost pure aldehyde **19m** (6.11 g) as colorless oil. Almost pure aldehyde **19m** was used in the next HWE reaction.
R_f = 0.37 (silica gel, hexane/ethyl acetate = 1/1)

General Procedures.

Optimized Reaction Conditions

To a solution of phosphate **1** (95.7 mg, 0.400 mmol) in tetrahydrofuran (4.7 mL), a 2.0 M solution of isopropylmagnesium chloride in tetrahydrofuran (0.18 mL, 0.360 mmol) was added at –78 °C. After the reaction mixture was stirred at –78 °C for 30 min, a solution of 3-phenylpropanal (**4c**, **15e**) (26.5 mg, 0.197 mmol) in tetrahydrofuran (2.0 mL) was added at room temperature, and the reaction mixture was stirred for 1.5 h. To the reaction mixture, saturated aqueous ammonium chloride was added at 0 °C, and the mixture was extracted with ethyl acetate. The organic layer was dried over sodium sulfate. After filtration of the mixture and concentration of the solvent, the crude mixture was purified by thin layer chromatography on silica (eluant; hexane/ethyl acetate = 1/1) to afford alkene (*E*)-**5c** (40.8 mg, 94%) as colorless oil and alkene (*Z*)-**5c** (0.4 mg, 0.9%) as colorless oil.

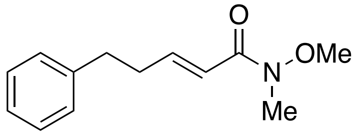

(2*E*)-*N*-methoxy-*N*-methyl-5-phenylpent-2-enamide ((*E*)-5c, (*E*)-16e).
R_f = 0.37 (silica gel, hexane/ethyl acetate = 2/1); FT-IR (neat) ν_{max}: 2931, 1658, 1635, 1450, 1419, 1381, 987, 702 cm^{-1}; ^{1}H NMR (500 MHz, CDCl$_3$): δ 7.33–7.24 (m, 2H, Ar), 7.23–7.15 (m, 3H, Ar), 7.01 (dt, *J* = 15.5, 7.0 Hz, 1H, H-3), 6.39 (d, *J* = 15.5 Hz, 1H, H-2), 3.63 (s, 3H, OMe), 3.23 (s, 3H, NMe), 2.80 (t, *J* = 7.5 Hz, 2H, H-5), 2.61–2.57 (m, 2H, H-4); ^{13}C{^{1}H} NMR (125 MHz, CDCl$_3$): 166.8 (C-1), 146.5 (C-3), 141.0 (Ar), 128.384 (Ar), 126.0 (Ar), 119.3 (C-2), 61.6 (OMe), 34.5 (C-5), 34.2 (C-4), 32.3 (NMe); HRMS calcd for $C_{26}H_{34}N_2O_4Na$ [2M + Na]$^+$ 461.2411, found 461.2391.

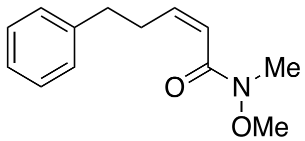

(2*Z*)-*N*-methoxy-*N*-methyl-5-phenylpent-2-enamide ((*Z*)-5c, (*Z*)-16e).
R_f = 0.56 (silica gel, hexane/ethyl acetate = 2/1); FT-IR (neat) ν_{max}: 2931, 1658, 1450, 1435, 1350, 1003, 702 cm^{-1}; ^{1}H NMR (500 MHz, CDCl$_3$): δ 7.31–7.25 (m, 2H, Ar), 7.24–7.20 (m, 2H, Ar), 7.20–7.15 (m, 1H, Ar), 6.26 (brd, *J* = 11.5 Hz, 1H, H-2), 6.13 (dt, *J* = 11.5, 7.5 Hz, 1H, H-3), 3.61 (s, 3H, OMe), 3.20 (s, 3H, NMe), 2.97 (brdt, *J* = 7.5, 7.5 Hz, 2H, H-4), 2.78 (t, *J* = 7.5 Hz, 2H, H-5); ^{13}C{^{1}H} NMR (125 MHz, CDCl$_3$): 167.2 (C-1), 146.1 (C-3), 141.3 (Ar), 128.5 (Ar), 128.2 (Ar), 125.8 (Ar), 118.6 (C-2), 61.4 (OMe), 35.2 (C-5), 31.8 (NMe), 30.4 (C-4); HRMS calcd for $C_{26}H_{34}N_2O_4Na$ [2M + Na]$^+$ 461.2411, found 461.2394.

Procedure of Our Experimental Data under Nuzillard's Reaction Conditions

Table S1. Comparison of Nuzillard's Data with Our Experimental Data.

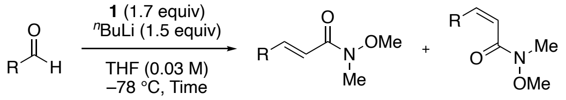

4a–e (*E*)-**5a–e** (*Z*)-**5a–e**

Entry	Substrate	Time	Nuzillard's Original data[a] Yield of (*E*/*Z*)-**5a–e** (ratio of *E*/*Z*)	Our Replicating Data[b] Yield of (*E*/*Z*)-**5a–e** (ratio of *E*/*Z*)
1[c]	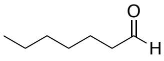 **4a** (=**2**)	15 min	76% (95/ 5)	42% (52/48)
2		1.5 h	–	78% (51/49)
3[d]		15 min	–	32% (100/0)
4	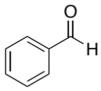 **4b**	25 min	74% (95/ 5)	–
5		17 h	–	86% (100/0)
6	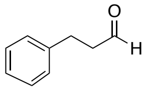 **4c**	15 min	–	78% (51/49)
7[e]	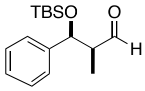 **4d**	2 h	–	91% (38/62)
8	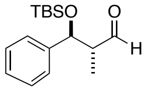 **4e**	6 h	–	86% (44/56)

a) In Nuzillard's report, the equivalents of HWE reagent and ^{n}BuLi and the amount of THF were not specified.
They used 1.5 equiv of ylide.
b) Yield was isolated yield.
The ratio of stereoisomer was calculated from the isolated yields.
c) Starting material could not be recovered because of its low boiling point.
d) Starting material was recovered with 51%.
e) An epimerized reaction product was obtained.

To a solution of phosphate **1** (81.3 mg, 0.340 mmol) in tetrahydrofuran (4.7 mL), a 1.6 M solution of *n*-butyllithium in hexane (0.19 mL, 0.300 mmol) was added at –78 °C. After the reaction mixture was stirred at –78 °C for 30 min, a solution of aldehyde (0.200 mmol) in tetrahydrofuran (2.0 mL) was added at –78 °C, and the reaction mixture was stirred for *Time*. To the reaction mixture, saturated aqueous ammonium chloride was added, and the mixture was extracted with ethyl acetate. The organic layer was dried over sodium sulfate. After filtration of the mixture and concentration of the solvent, the crude mixture was purified by thin layer chromatography on silica to afford alkene **5**.

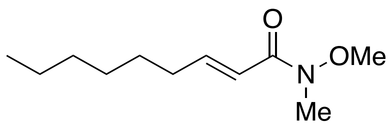

(2*E*)-*N*-methoxy-*N*-methylnon-2-enamide ((*E*)-5a, (*E*)-16g).

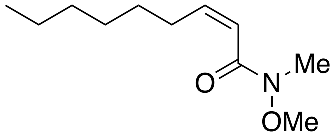

(2*Z*)-*N*-methoxy-*N*-methylnon-2-enamide ((*Z*)-5a, (*Z*)-16g).

Heptanal (**2**, **4a**, **15g**) (23.0 mg, 0.201 mmol) was employed. Purification by thin layer chromatography on silica (eluant; hexane/ethyl acetate = 3/2) to afford alkene (*E*)-**5a** (15.9 mg, 40%) as colorless oil and alkene (*Z*)-**5a** (15.5 mg, 39%) as colorless oil.

(*E*)-**5a**

R_f = 0.43 (silica gel, hexane/ethyl acetate = 2/1); FT-IR (neat) ν_{max}: 2931, 2854, 1666, 1635, 1466, 1412, 1381, 995 cm^{-1}; ^{1}H NMR (500 MHz, $CDCl_3$): δ 6.98 (dt, J = 15.5, 7.0 Hz, 1H, H-3), 6.39 (dt, J = 15.5, 1.0 Hz, 1H, H-2), 3.70 (s, 3H, OMe), 3.24 (s, 3H, NMe), 2.23 (dtd, J = 7.0, 6.5, 1.0 Hz, 2H, H-4), 1.47 (tt, J = 7.5, 6.5 Hz, 2H, H-5), 1.38–1.16 (m, 6H, H-6, H-7, H-8), 0.88 (t, J = 7.0 Hz, 3H, H-9); ^{13}C{^{1}H} NMR (125 MHz, $CDCl_3$): 167.1 (C-1), 148.0 (C-3), 118.6 (C-2), 61.6 (OMe), 32.5 (C-4), 32.3 (NMe), 31.6 (C-7), 28.8 (C-6), 28.2 (C-5), 22.5 (C-8), 14.0 (C-9); HRMS calcd for $C_{11}H_{21}NO_2Na$ $[M + Na]^+$ 222.1465, found 222.1473.

(*Z*)-**5a**

R_f = 0.62 (silica gel, hexane/ethyl acetate = 2/1); FT-IR (neat) ν_{max}: 2954, 2924, 2854, 1658, 1458, 1435, 1342, 1003 cm^{-1}; ^{1}H NMR (500 MHz, $CDCl_3$): δ 6.23 (brd, J = 11.5 Hz, 1H, H-2), 6.12 (dt, J = 11.5, 7.5 Hz, 1H, H-3), 3.68 (s, 3H, OMe), 3.21 (s, 3H, NMe), 2.62 (dt, J = 7.5, 7.0 Hz, 2H, H-4), 1.43 (tt, J = 7.5, 7.0 Hz, 2H, H-5), 1.38–1.22 (m, 6H, H-6, H-7, H-8), 0.88 (t, J = 7.0 Hz, 3H, H-9); ^{13}C{^{1}H} NMR (125 MHz, $CDCl_3$): 167.6 (C-1), 147.8 (C-3), 117.9 (C-2), 61.4 (OMe), 32.0 (NMe), 31.7 (C-7), 29.3 (C-5), 29.1 (C-4), 29.0 (C-6), 22.6 (C-8), 14.0 (C-9); HRMS calcd for $C_{11}H_{21}NO_2Na$ $[M + Na]^+$ 222.1465, found 222.1464.

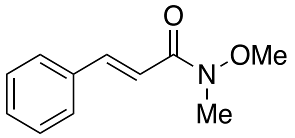

(2*E*)-*N*-Methoxy-*N*-methyl-3-phenylprop-2-enamide ((*E*)-5b, (*E*)-20a).

Benzaldehyde (**4b**, **19a**) (21.3 mg, 0.201 mmol) was employed. Purification by thin layer chromatography on silica (eluant; hexane/ethyl acetate = 1/1) to afford alkene (*E*)-**5b** (32.9 mg, 86%) as white solid.

3-Phenylpropanal (**4c**, **15e**) (27.0 mg, 0.201 mmol) was employed. Purification by thin layer chromatography on silica (eluant; hexane/ethyl acetate = 1/1) to afford alkene (*E*)-**5c** (14.8 mg, 34%) as colorless oil and alkene (*Z*)-**5c** (18.8 mg, 43%) as colorless oil.

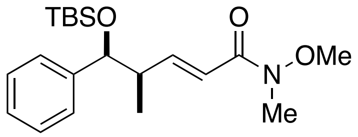

(4*RS*,5*SR*,2*E*)-5-((*tert*-Butyldimethylsilyl)oxy)-*N*-methoxy-*N*,4-dimethyl-5-phenylpent-2-enamide ((*E*)-5d, (*E*)-16p).

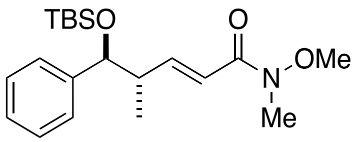

(4*SR*,5*SR*,2*E*)-5-((*tert*-Butyldimethylsilyl)oxy)-*N*-methoxy-*N*,4-dimethyl-5-phenylpent-2-enamide ((*E*)-5e, (*E*)-16q).

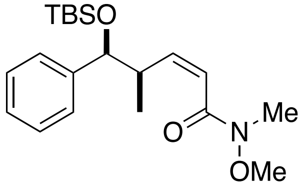

(4*RS*,5*SR*,2*Z*)-5-((*tert*-Butyldimethylsilyl)oxy)-*N*-methoxy-*N*,4-dimethyl-5-phenylpent-2-enamide ((*Z*)-5d, (*Z*)-16p).

Aldehyde **15p** (55.4 mg, 0.199 mmol) was employed. Purification by thin layer chromatography on silica (eluant; hexane/ethyl acetate = 3/1) to afford alkene (*E*)-**16p** (23.5 mg, 33%) as colorless oil, (*E*)-**16q** (1.2 mg, 1.7%) as colorless oil and alkene (*Z*)-**16p** (40.6 mg, 56%) as colorless oil.

(*E*)-**16p**

R_f= 0.53 (silica gel, hexane/ethyl acetate = 2/1); FT-IR (neat) ν_{max}: 2954, 2931, 2893, 2854, 1666, 1635, 1466, 1412, 1381, 1257, 1088, 1065, 841, 779, 702 cm^{-1}; ^{1}H NMR (500 MHz, $CDCl_3$): δ 7.30–7.22 (m, 4H, Ar), 7.22–7.17 (m, 1H, Ar), 6.90 (dd, *J* = 15.5, 8.0 Hz, 1H, H-3), 6.21 (d, *J* = 15.5 Hz, 1H, H-2), 4.58 (d, *J* = 6.0 Hz, 1H, H-5), 3.54 (s, 3H, OMe), 3.20 (s, 3H, NMe), 2.62 (dqd, *J* = 8.0, 7.0, 6.0 Hz, 1H, H-4), 1.06 (d, *J* = 7.0 Hz, 3H, 4-Me), 0.88 (s, 9H, TBS), 0.03 (s, 3H, TBS), –0.21 (s, 3H, TBS); ^{13}C{^{1}H} NMR (125 MHz, $CDCl_3$): 166.7 (C-1), 149.6 (C-3), 143.1 (Ar), 127.7 (Ar), 127.0 (Ar), 126.6 (Ar), 118.7 (C-2), 78.0 (C-5), 61.5 (OMe), 45.7 (C-4), 32.2 (NMe), 25.8 (TBS), 18.1 (TBS), 14.6 (4-Me), –4.7 (TBS), –5.1 (TBS); HRMS calcd for $C_{20}H_{33}NO_3SiNa$ [M + Na]$^+$ 386.2122, found 386.2103.

(*Z*)-**16p**

R_f= 0.75 (silica gel, hexane/ethyl acetate = 2/1); FT-IR (neat) ν_{max}: 2954, 2931, 2893, 2854, 1658, 1458, 1350, 1257, 1095, 1026, 1003, 856, 841, 779, 702 cm^{-1}; ^{1}H NMR (500 MHz, $CDCl_3$): δ 7.38–7.32 (m, 2H, Ar), 7.31–7.23 (m, 2H, Ar), 7.21–7.14 (m, 1H, Ar), 6.20 (brd, *J* = 11.0 Hz, 1H, H-2), 6.05 (dd, *J* = 11.0, 11.0 Hz, 1H, H-3), 4.76 (d, *J* = 4.0 Hz, 1H, H-5), 3.76–3.59 (m, 1H, H-4), 3.55 (s, 3H, OMe), 3.19 (s, 3H, NMe), 0.94 (d, *J* = 6.0 Hz, 3H, 4-Me), 0.91 (s, 9H, TBS), 0.00 (s, 3H, TBS), –0.22 (s, 3H, TBS); ^{13}C{^{1}H} NMR (125 MHz, $CDCl_3$): 167.2 (C-1), 150.6 (C-3), 143.8 (Ar), 127.6 (Ar), 126.6 (Ar), 126.5 (Ar), 117.2 (C-2), 77.8 (C-5), 61.4 (OMe), 41.2 (C-4), 32.0 (NMe), 25.8 (TBS), 18.2 (TBS), 14.0 (4-Me), –4.6 (TBS), –5.1 (TBS); HRMS calcd for $C_{20}H_{33}NO_3SiNa$ [M + Na]$^+$ 386.2122, found 386.2105.

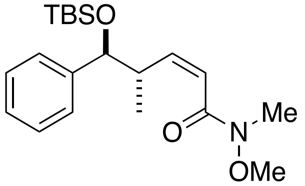

(4*SR*,5*SR*,2*Z*)-5-((*tert*-Butyldimethylsilyl)oxy)-*N*-methoxy-*N*,4-dimethyl-5-phenylpent-2-enamide ((*Z*)-5e, (*Z*)-16q).

Aldehyde **15q** (56.0 mg, 0.201 mmol) was employed. Purification by thin layer chromatography on silica (eluant; hexane/ethyl acetate = 3/1) to afford alkene (*E*)-**16q** (27.5 mg, 38%) as colorless oil and alkene (*Z*)-**16q** (35.3 mg, 48%) as colorless oil.

(*E*)-**16q**

R_f= 0.68 (silica gel, hexane/ethyl acetate = 2/1); FT-IR (neat) ν_{max}: 2954, 2931, 2893, 2854, 1666, 1635, 1466, 1412, 1381, 1257, 1088, 1065, 841, 779, 702 cm^{-1}; ^{1}H NMR (500 MHz, $CDCl_3$): δ 7.32–7.17 (m, 5H, Ar), 6.99 (dd, *J* = 15.5, 8.5 Hz, 1H, H-3), 6.26 (d, *J* = 15.5 Hz, 1H, H-2), 4.53 (d, *J* = 5.5 Hz, 1H,

H-5), 3.58 (s, 3H, OMe), 3.22 (s, 3H, NMe), 2.66–2.56 (m, 1H, H-4), 0.96 (d, *J* = 7.0 Hz, 3H, 4-Me), 0.86 (s, 9H, TBS), 0.02 (s, 3H, TBS), –0.22 (s, 3H, TBS); $^{13}C\{^{1}H\}$ NMR (125 MHz, $CDCl_3$): 166.7 (C-1), 149.4 (C-3), 143.1 (Ar), 127.7 (Ar), 127.0 (Ar), 126.6 (Ar), 118.8 (C-2), 78.6 (C-5), 61.5 (OMe), 45.8 (C-4), 32.2 (NMe), 25.7 (TBS), 18.1 (TBS), 16.2 (4-Me), –4.7 (TBS), –5.1 (TBS); HRMS calcd for $C_{20}H_{33}NO_3SiNa$ $[M + Na]^+$ 386.2122, found 386.2122.

(*Z*)-16q

R_f = 0.78 (silica gel, hexane/ethyl acetate = 2/1); FT-IR (neat) ν_{max}: 2954, 2931, 2893, 2854, 1658, 1458, 1257, 1095, 1065, 1003, 856, 841, 779, 702 cm^{-1}; ^{1}H NMR (500 MHz, $CDCl_3$): δ 7.30–7.21 (m, 4H, Ar), 7.20–7.13 (m, 1H, Ar), 6.18 (brd, *J* = 11.0 Hz, 1H, H-2), 6.10 (dd, *J* = 11.0, 11.0 Hz, 1H, H-3), 4.61 (d, *J* = 5.5 Hz, 1H, H-5), 3.81–3.59 (m, 1H, H-4), 3.44 (s, 3H, OMe), 3.11 (s, 3H, NMe), 1.05 (d, *J* = 7.0 Hz, 3H, 4-Me), 0.90 (s, 9H, TBS), 0.06 (s, 3H, TBS), –0.21 (s, 3H, TBS); $^{13}C\{^{1}H\}$ NMR (125 MHz, $CDCl_3$): 167.1 (C-1), 148.4 (C-3), 143.7 (Ar), 127.6 (Ar), 126.7 (Ar), 126.5 (Ar), 118.2 (C-2), 78.3 (C-5), 61.3 (OMe), 41.5 (C-4), 31.9 (NMe), 25.8 (TBS), 18.2 (TBS), 17.3 (4-Me), –4.6 (TBS), –5.1 (TBS); HRMS calcd for $C_{20}H_{33}NO_3SiNa$ $[M + Na]^+$ 386.2122, found 386.2132.

Procedure with Strong Base

Effect of Strong Base Conditions and Cation on the Yield and Selectivity of the Weinreb Amide-Type HWE Reaction

Table S2. Effect of Strong Base Conditions, Cation, and Temperature on the Weinreb Amide-Type HWE Reaction.

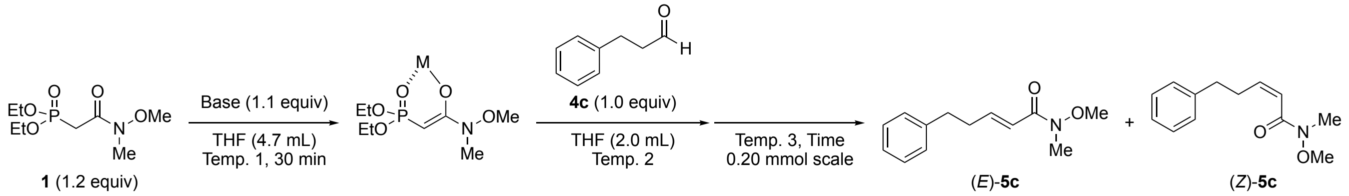

Entry	Base	Temp. 1	Temp. 2	Temp. 3	Time	Yield (*E*)-**5c**	Yield (*Z*)-**5c**	*E*/*Z*
1	LHMDS	–78 °C	–78 °C	–78 °C	20 min	27	41	40/60
2	↑	↑	0 °C	0 °C	↑	74	18	80/20
3	↑	↑	rt	rt	↑	73	13	85/15
4	↑	↑	–78 °C	rt	↑	44	47	49/51
5	NaHMDS	↑	0 °C	0 °C	↑	85	2.5	97/ 3
6	↑	↑	rt	rt	↑	84	4.4	95/ 5
7	KHMDS	↑	0 °C	0 °C	↑	77	10	88/12
8	↑	↑	rt	rt	↑	79	7.4	91/ 9
9	^{n}BuLi	↑	0 °C	0 °C	20 min	72	19	79/21
10	↑	↑	rt	rt	↑	79	12	87/13
11	NaH	0 °C	0 °C	0 °C	30 min	89	3.0	97/ 3
12	↑	↑	rt	rt	↑	90	3.2	97/ 3
13	^{t}BuOK	0 °C	0 °C	0 °C	↑	85	10	89/11
14	↑	rt	rt	rt	20 min	82	7.9	91/ 9
15	^{i}PrMgBr	–78 °C	–78 °C	–78 °C	↑	4.1	0	100/0
16	↑	↑	0 °C	0 °C	↑	27	trace	99/ 1
17	↑	↑	rt	rt	↑	58	1.1	98/ 2
18	↑	↑	rt	rt	4 h	73	1.1	99/ 1

To a solution of phosphate **1** (57.4 mg, 0.240 mmol) in tetrahydrofuran (4.7 mL), *Base* (0.220 mmol) was added at *Temp.* 1. After the reaction mixture was stirred at *Temp. 1* for 30 min, a solution of

3-phenylpropanal **4c** in tetrahydrofuran (2.0 mL) was added at *Temp. 2*, and the reaction mixture was stirred at *Temp. 3* for *Time*. To the reaction mixture, saturated aqueous ammonium chloride was added at 0 °C, and the mixture was extracted with ethyl acetate. The organic layer was dried over sodium sulfate. After filtration of the mixture and concentration of the solvent, the crude mixture was purified by thin layer chromatography on silica (eluant; hexane/ethyl acetate = 1/1) to afford alkene (*E*)-**5c** and alkene (*Z*)-**5c**.
Entry 1: 3-phenylpropanal **4c** (26.5 mg, 0.197 mmol), (*E*)-**5c** (11.5 mg, 27%), (*Z*)-**5c** (17.6 mg, 41%)
Entry 2: 3-phenylpropanal **4c** (26.6 mg, 0.198 mmol), (*E*)-**5c** (31.9 mg, 74%), (*Z*)-**5c** (7.6 mg, 18%)
Entry 3: 3-phenylpropanal **4c** (26.7 mg, 0.199 mmol), (*E*)-**5c** (31.7 mg, 73%), (*Z*)-**5c** (5.7 mg, 13%)
Entry 4: 3-phenylpropanal **4c** (26.2 mg, 0.195 mmol), (*E*)-**5c** (18.9 mg, 44%), (*Z*)-**5c** (20.0 mg, 47%)
Entry 5: 3-phenylpropanal **4c** (27.0 mg, 0.201 mmol), (*E*)-**5c** (37.5 mg, 85%), (*Z*)-**5c** (1.1 mg, 2.5%)
Entry 6: 3-phenylpropanal **4c** (26.5 mg, 0.197 mmol), (*E*)-**5c** (36.4 mg, 84%), (*Z*)-**5c** (1.9 mg, 4.4%)
Entry 7: 3-phenylpropanal **4c** (26.5 mg, 0.197 mmol), (*E*)-**5c** (33.3 mg, 77%), (*Z*)-**5c** (4.5 mg, 10%)
Entry 8: 3-phenylpropanal **4c** (26.5 mg, 0.197 mmol), (*E*)-**5c** (34.0 mg, 79%), (*Z*)-**5c** (3.2 mg, 7.4%)
Entry 9: 3-phenylpropanal **4c** (26.8 mg, 0.200 mmol), (*E*)-**5c** (31.3 mg, 72%), (*Z*)-**5c** (8.4 mg, 19%)
Entry 10: 3-phenylpropanal **4c** (26.8 mg, 0.200 mmol), (*E*)-**5c** (34.5 mg, 79%), (*Z*)-**5c** (5.3 mg, 12%)
Entry 11: 3-phenylpropanal **4c** (26.4 mg, 0.197 mmol), (*E*)-**5c** (38.2 mg, 89%), (*Z*)-**5c** (1.3 mg, 3.0%)
Entry 12: 3-phenylpropanal **4c** (26.6 mg, 0.198 mmol), (*E*)-**5c** (38.9 mg, 90%), (*Z*)-**5c** (1.4 mg, 3.2%)
Entry 13: 3-phenylpropanal **4c** (26.6 mg, 0.198 mmol), (*E*)-**5c** (36.7 mg, 85%), (*Z*)-**5c** (4.5 mg, 10%)
Entry 14: 3-phenylpropanal **4c** (26.4 mg, 0.197 mmol), (*E*)-**5c** (35.3 mg, 82%), (*Z*)-**5c** (3.4 mg, 7.9%)
Entry 15: 3-phenylpropanal **4c** (26.6 mg, 0.198 mmol), (*E*)-**5c** (1.8 mg, 4.1%), (*Z*)-**5c** (0 mg, 0%)
Entry 16: 3-phenylpropanal **4c** (26.9 mg, 0.200 mmol), (*E*)-**5c** (12.0 mg, 27%), (*Z*)-**5c** (0.1 mg, trace)
Entry 17: 3-phenylpropanal **4c** (27.0 mg, 0.201 mmol), (*E*)-**5c** (25.5 mg, 58%), (*Z*)-**5c** (0.5 mg, 1.1%)
Entry 18: 3-phenylpropanal **4c** (26.4 mg, 0.197 mmol), (*E*)-**5c** (31.6 mg, 73%), (*Z*)-**5c** (0.5 mg, 1.1%)

Procedure with Weak Base

Reaction Conditions of Weak Base and Cation Effect to Reactivity and Selectivity.

Table S3. Weinreb Amide-Type HWE reaction under Weak Base Conditions and Effect of Cation and Temperature.

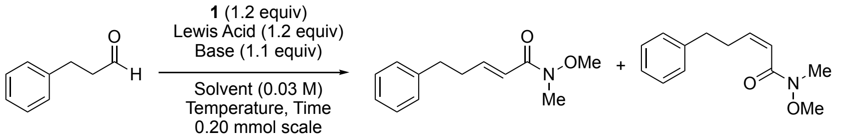

4c (*E*)-**5c** (*Z*)-**5c**

Entry	Lewis Acid	Base	Solvent	Temperature	Time	Yield (%) (*E*)-**5c**	Yield (%) (*Z*)-**5c**	*E*/*Z*	rSM (%)
1	LiCl	DBU	THF	rt	1.5 h	78	12	87/13	0
2	↑	↑	MeCN	↑	↑	83	2.5	97/ 3	0
3	↑	NEt_3	THF	↑	↑	8.5	0.6	93/ 7	66
4	↑	↑	MeCN	↑	↑	47	trace	>99/ 1	31
5	↑	$^iPr_2NEt$	THF	↑	↑	3.2	trace	>99/ 1	69
6	↑	↑	MeCN	↑	↑	28	trace	>99/ 1	46
7	↑	NEt_3	THF	↑	12 h	47	9.4	83/17	27
8	↑	↑	MeCN	↑	↑	69	0.6	99/ 1	6.8
9	↑	$^iPr_2NEt$	THF	↑	↑	12	0.6	>99/ 1	53
10	↑	↑	MeCN	↑	↑	66	trace	>99/ 1	3.9
11	$MgBr_2$	DBU	THF	50 °C	1.5 h	38	1.8	95/ 5	42
12	↑	NEt_3	↑	↑	↑	28	0.6	96/ 4	45
13	↑	$^iPr_2NEt$	↑	↑	↑	26	2.4	95/ 5	49

To a solution of phosphate **1** (57.4 mg, 0.240 mmol) in *Solvent* (4.7 mL), *Lewis Acid* (0.240 mmol, LiCl: 10.2 mg; $MgBr_2$: 44.2 mg) and *Base* (0.220 mmol) were added at room temperature. After a solution of 3-phenylpropanal **4c** in *Solvent* (2.0 mL) was added at *Temperature*, the reaction mixture was stirred at *Temperature* for *Time*. To the reaction mixture, saturated aqueous ammonium chloride was added at 0 °C, and the mixture was extracted with ethyl acetate. The organic layer was dried over sodium sulfate. After filtration of the mixture and concentration of the solvent, the crude mixture was purified by thin layer chromatography on silica (eluant; hexane/ethyl acetate = 1/1) to afford alkene (*E*)-**5c** and alkene (*Z*)-**5c**.

Entry 1: 3-phenylpropanal **4c** (26.4 mg, 0.197 mmol), (*E*)-**5c** (33.7 mg, 78%), (*Z*)-**5c** (5.0 mg, 12%), recovery of starting material (**4c**) (0 mg, 0%)

Entry 2: 3-phenylpropanal **4c** (26.5 mg, 0.197 mmol), (*E*)-**5c** (37.0 mg, 83%), (*Z*)-**5c** (1.1 mg, 2.5%), recovery of starting material (**4c**) (0 mg, 0%)

Entry 3: 3-phenylpropanal **4c** (26.7 mg, 0.199 mmol), (*E*)-**5c** (3.7 mg, 8.5%), (*Z*)-**5c** (0.3 mg, 0.6%), recovery of starting material (**4c**) (17.7 mg, 66%)

Entry 4: 3-phenylpropanal **4c** (26.6 mg, 0.198 mmol), (*E*)-**5c** (20.2 mg, 47%), (*Z*)-**5c** (trace), recovery of starting material (**4c**) (8.1 mg, 31%)

Entry 5: 3-phenylpropanal **4c** (26.5 mg, 0.197 mmol), (*E*)-**5c** (1.4 mg, 3.2%), (*Z*)-**5c** (trace), recovery of starting material (**4c**) (18.2 mg, 69%)

Entry 6: 3-phenylpropanal **4c** (26.9 mg, 0.200 mmol), (*E*)-**5c** (12.4 mg, 28%), (*Z*)-**5c** (trace), recovery of starting material (**4c**) (12.3 mg, 46%)

Entry 7: 3-phenylpropanal **4c** (26.7 mg, 0.199 mmol), (*E*)-**5c** (20.6 mg, 47%), (*Z*)-**5c** (4.1 mg, 9.4%), recovery of starting material (**4c**) (7.1 mg, 27%)

Entry 8: 3-phenylpropanal **4c** (26.4 mg, 0.197 mmol), (*E*)-**5c** (29.9 mg, 69%), (*Z*)-**5c** (0.3 mg, 0.6%), recovery of starting material (**4c**) (1.8 mg, 6.8%)

Entry 9: 3-phenylpropanal **4c** (26.9 mg, 0.200 mmol), (*E*)-**5c** (5.3 mg, 12%), (*Z*)-**5c** (0.3 mg, 0.6%), recovery of starting material (**4c**) (14.2 mg, 53%)
Entry 10: 3-phenylpropanal **4c** (26.9 mg, 0.200 mmol), (*E*)-**5c** (29.1 mg, 66%), (*Z*)-**5c** (trace), recovery of starting material (**4c**) (1.1 mg, 3.9%)
Entry 11: 3-phenylpropanal **4c** (26.7 mg, 0.199 mmol), (*E*)-**5c** (16.5 mg, 38%), (*Z*)-**5c** (0.8 mg, 1.8%), recovery of starting material (**4c**) (11.2 mg, 42%)
Entry 12: 3-phenylpropanal **4c** (26.5 mg, 0.197 mmol), (*E*)-**5c** (12.3 mg, 28%), (*Z*)-**5c** (0.3 mg, 0.6%), recovery of starting material (**4c**) (11.9 mg, 45%)
Entry 13: 3-phenylpropanal **4c** (26.6 mg, 0.198 mmol), (*E*)-**5c** (11.3 mg, 26%), (*Z*)-**5c** (1.0 mg, 2.4%), recovery of starting material (**4c**) (12.9 mg, 49%)

Subsequently, we investigated the high *E*-selective reaction conditions (Table S3. Entries 8,10,11) under which the total yields of alkene (*E*)-**5c**, alkene (*Z*)-**5c** and 3-phenylpropanal **4c** was high.

Table S4. Additional Investigation of Weinreb Amide-Type HWE Reaction with Lewis Acid

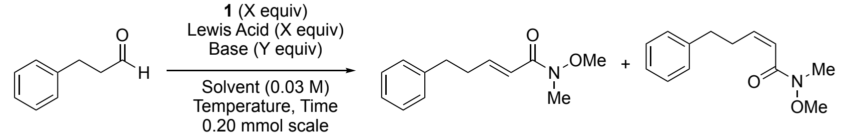

4c (*E*)-**5c** (*Z*)-**5c**

Entry	Lewis Acid	X (equiv)	Base	Y (equiv)	Solvent	Temperature	Time	Yield (%) (*E*)-**5c**	(*Z*)-**5c**	*E*/*Z*	rSM (%)
1	–	2.0	^{i}Pr$_2$NEt	1.8	MeCN	rt	12 h	0	0	–	–
2	↑	↑	↑	↑	↑	50 °C	↑	0	0	–	–
3	LiCl	1.6	NEt$_3$	1.5	↑	rt	↑	74	1.5	98/ 2	0.7
4	↑	2.0	↑	1.8	↑	↑	↑	84	3.0	97/ 3	0
5	↑	1.6	^{i}Pr$_2$NEt	1.5	↑	↑	↑	74	0.8	99/ 1	2.6
6	↑	2.0	↑	1.8	↑	↑	↑	87	1.8	98/ 2	0
7	↑	↑	↑	↑	↑	50 °C	8 h	87	2.1	98/ 2	0
8	–	2.4	DBU	2.2	THF	rt	3 h	trace	0	>99/ 1	70
9	↑	↑	↑	↑	↑	50 °C	↑	<3.2	trace	>99/ 1	72
10	MgBr$_2$	↑	↑	↑	MeCN	rt	13 h	0	0	–	–
11	↑	↑	↑	↑	THF	↑	3 h	71	0	100/ 0	0
12	↑	↑	↑	↑	↑	50 °C	30 min	75	0.8	99/ 1	trace
13	↑	2.0	^{i}PrMgBr	1.8	↑	rt	1.5 h	76	0.9	99/ 1	0
14	↑	↑	↑	↑	↑	50 °C	20 min	84	1.1	99/ 1	0
15	MgCl$_2$[a]	↑	^{i}PrMgCl	↑	↑	rt	1.5 h	89	2.3	97/ 3	0
16	MgBr$_2$	↑	↑	↑	↑	↑	↑	79	1.6	98/ 2	0

a) MgCl$_2$ was hardly dissolved in tetrahydrofuran.

Procedure with Grignard Reagent as a Base
Equivalent of Phosphonoenolate and Effect of Alkyl and Halogen Moieties in Grignard Reagent.

Table S5. Screening of the Amount of Weinreb Amide-Type HWE Reagent and ^{i}PrMgBr.

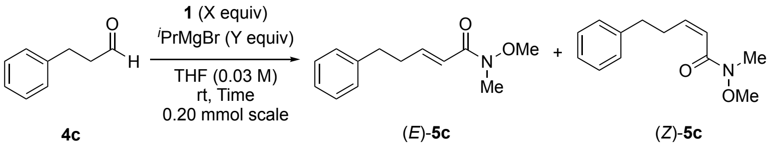

Entry	X (equiv)	Y (equiv)	Time (h)	Yield (%) (*E*)-**5c**	(*Z*)-**5c**	rSM (%)	*E*/*Z*
1	1.2	1.1	4	73	1.1	6.8	99/ 1
2	1.6	1.5	1.5	79	2.3	trace	97/ 3
3	2.0	1.8	↑	84	3.2	0	96/ 4
4	2.4	2.2	↑	76	trace	0	>99/ 1

To a solution of phosphate **1** (X equiv) in tetrahydrofuran (4.7 mL), a 0.70 M solution of isopropylmagnesium bromide in tetrahydrofuran (Y equiv) was added at –78 °C. After the reaction mixture was stirred at –78 °C for 30 min, a solution of 3-phenylpropanal **4c** in tetrahydrofuran (2.0 mL) was added at room temperature, and the reaction mixture was stirred at room temperature for *Time*. To the reaction mixture, saturated aqueous ammonium chloride was added at 0 °C, and the mixture was extracted with ethyl acetate. The organic layer was dried over sodium sulfate. After filtration of the mixture and concentration of the solvent, the crude mixture was purified by thin layer chromatography on silica (eluant; hexane/ethyl acetate = 1/1) to afford alkene (*E*)-**5c** and alkene (*Z*)-**5c**.
Entry 1: 3-phenylpropanal **4c** (26.4 mg, 0.197 mmol), (*E*)-**5c** (31.6 mg, 73%), (*Z*)-**5c** (0.5 mg, 1.1%), recovery of starting material (**4c**) (1.8 mg, 6.8%)
Entry 2: 3-phenylpropanal **4c** (26.4 mg, 0.197 mmol), (*E*)-**5c** (34.2 mg, 79%), (*Z*)-**5c** (1.0 mg, 2.3%), recovery of starting material (**4c**) (trace)
Entry 3: 3-phenylpropanal **4c** (26.8 mg, 0.200 mmol), (*E*)-**5c** (36.9 mg, 84%), (*Z*)-**5c** (1.4 mg, 3.2%), recovery of starting material (**4c**) (0 mg, 0%)
Entry 4: 3-phenylpropanal **4c** (26.6 mg, 0.198 mmol), (*E*)-**5c** (33.0 mg, 76%), (*Z*)-**5c** (trace), recovery of starting material (**4c**) (0 mg, 0%)

Table S6. Effect of Alkyl and Halogen Moieties on the Grignard Reagent on the Yield and (*E*)-Selectivity.

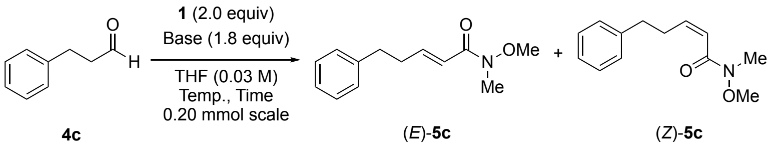

Entry	Base	Temp.	Time	Yield (%) (*E*)-**5c**	Yield (%) (*Z*)-**5c**	*E*/*Z*
1	MeMgBr	0 °C	5 h	70	0.8	99/1
2	↑	rt	1.5 h	82	1.5	98/2
3	MeMgCl	↑	↑	89	1.6	98/2
4	MeMgI	↑	↑	29	trace	>99/1
5	EtMgBr	0 °C	5 h	63	trace	>99/1
6	↑	rt	1.5 h	83	0.7	99/1
7	PhMgBr	0 °C	3.5 h	64	trace	>99/1
8	↑	rt	1.5 h	82	1.6	98/2
9	^{i}PrMgBr	0 °C	5 h	63	1.2	98/2
10	↑	rt	1.5 h	84	3.2	96/4
11	↑	50 °C	0.5 h	83	1.1	99/1
12	^{i}PrMgCl	rt	1.5 h	94	0.9	99/1
13	↑	50 °C	0.5 h	87	0.7	99/1
14	^{i}PrMgCl•LiCl	rt	1.5 h	90	2.3	98/2

To a solution of phosphate **1** (95.7 mg, 0.400 mmol) in tetrahydrofuran (4.7 mL), *Base* (0.360 mmol) was added at –78 °C. After the reaction mixture was stirred at –78 °C for 30 min, a solution of 3-phenylpropanal **4c** in tetrahydrofuran (2.0 mL) was added at *Temp.*, and the reaction mixture was stirred at *Temp.* for *Time*. To the reaction mixture, saturated aqueous ammonium chloride was added at 0 °C, and the mixture was extracted with ethyl acetate. The organic layer was dried over sodium sulfate. After filtration of the mixture and concentration of the solvent, the crude mixture was purified by thin layer chromatography on silica (eluant; hexane/ethyl acetate = 1/1) to afford alkene (*E*)-**5c** and alkene (*Z*)-**5c**.

Entry 1: 3-phenylpropanal **4c** (26.8 mg, 0.200 mmol), (*E*)-**5c** (30.6 mg, 70%), (*Z*)-**5c** (0.4 mg, 0.8%)
Entry 2: 3-phenylpropanal **4c** (26.9 mg, 0.200 mmol), (*E*)-**5c** (35.8 mg, 82%), (*Z*)-**5c** (0.7 mg, 1.5%)
Entry 3: 3-phenylpropanal **4c** (27.0 mg, 0.201 mmol), (*E*)-**5c** (39.4 mg, 89%), (*Z*)-**5c** (0.7 mg, 1.6%)
Entry 4: 3-phenylpropanal **4c** (26.6 mg, 0.198 mmol), (*E*)-**5c** (12.6 mg, 29%), (*Z*)-**5c** (trace)
Entry 5: 3-phenylpropanal **4c** (26.4 mg, 0.197 mmol), (*E*)-**5c** (27.0 mg, 63%), (*Z*)-**5c** (trace)
Entry 6: 3-phenylpropanal **4c** (27.1 mg, 0.202 mmol), (*E*)-**5c** (36.6 mg, 83%), (*Z*)-**5c** (0.3 mg, 0.7%)
Entry 7: 3-phenylpropanal **4c** (26.8 mg, 0.200 mmol), (*E*)-**5c** (28.2 mg, 64%), (*Z*)-**5c** (trace)
Entry 8: 3-phenylpropanal **4c** (27.2 mg, 0.203 mmol), (*E*)-**5c** (36.2 mg, 82%), (*Z*)-**5c** (0.7 mg, 1.6%)
Entry 9: 3-phenylpropanal **4c** (26.5 mg, 0.197 mmol), (*E*)-**5c** (27.3 mg, 63%), (*Z*)-**5c** (0.5 mg, 1.2%)
Entry 10: 3-phenylpropanal **4c** (26.8 mg, 0.200 mmol), (*E*)-**5c** (36.9 mg, 84%), (*Z*)-**5c** (1.4 mg, 3.2%)
Entry 11: 3-phenylpropanal **4c** (27.0 mg, 0.201 mmol), (*E*)-**5c** (36.5 mg, 83%), (*Z*)-**5c** (0.5 mg, 1.1%)
Entry 12: 3-phenylpropanal **4c** (26.5 mg, 0.197 mmol), (*E*)-**5c** (40.8 mg, 94%), (*Z*)-**5c** (0.4 mg, 0.9%)
Entry 13: 3-phenylpropanal **4c** (26.6 mg, 0.198 mmol), (*E*)-**5c** (37.8 mg, 87%), (*Z*)-**5c** (0.3 mg, 0.7%)
Entry 14: 3-phenylpropanal **4c** (26.6 mg, 0.198 mmol), (*E*)-**5c** (39.1 mg, 90%), (*Z*)-**5c** (1.0 mg, 2.3%)

In addition, we also investigated the effect of alkyl moieties on organolithium reagents for the yield and (*E*)-selectivity to reveal that alkyl moieties on organolithium reagents don't affect them as the same as the Grignard reagents.

Table S7. Effect of Alkyl Moieties on Organolithium Reagent for the Yield and (*E*)-Selectivity.

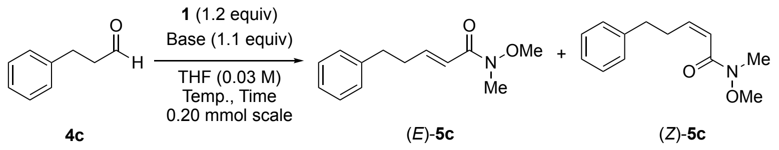

Entry	Base	Temp.	Time	Yield (%) (*E*)-**5c**	(*Z*)-**5c**	*E*/*Z*
1	MeLi	0 °C	30 min	77	20	79/21
2	↑	rt	↑	82	13	86/14
3	^{n}BuLi	0 °C	20 min	72	19	79/21
4	↑	rt	↑	79	12	87/13
5	PhLi	0 °C	30 min	76	18	81/19
6	↑	rt	↑	81	13	86/14

Procedure with Hydroxide as a Base

Table S8. Weinreb Amide-Type HWE Reaction under Metal Hydroxide Conditions and Effect of Cation.

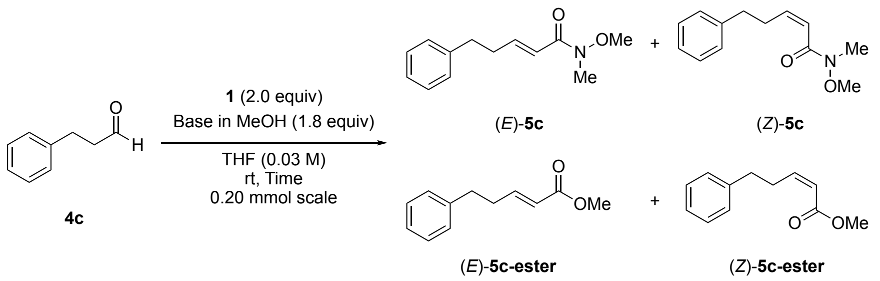

Entry	Base	Time	Yield (%) (*E*)-**5c**	(*Z*)-**5c**	(*E*)-**5c-ester**	(*Z*)-**5c-ester**	*E*/*Z*
1	LiOH	20 min	66	13	10	0.5	85/15
2	NaOH	↑	83	5.0	8.8	0.8	94/ 6
3	KOH	↑	73	14	4.5	0.5	84/16
4	Triron B	1 h	62	15	0	0	80/20

Procedure for Investigation of Solvent and Concentration Effect
Solvent and Concentration Effect in Weinreb Amide-Type HWE Reaction

Table S9. Effect of Type and Amount of Solvent.

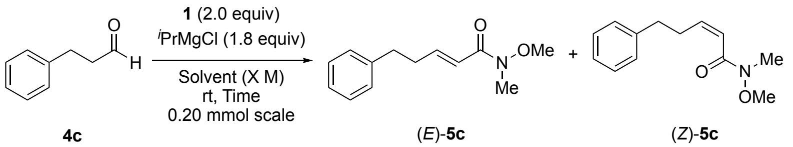

Entry	Solvent	X (M)	Time (h)	Yield (%) (E)-5c	(Z)-5c	E/Z
1	THF	0.03	1.5	94	0.9	99/ 1
2	DME	↑	↑	78	trace	>99/ 1
3	Et_2O	↑	↑	23	0	100/ 0
4	MeCN	↑	↑	15	trace	>99/ 1
5	toluene	↑	↑	17	2.8	85/15
6[a]	THF	0.03	1.5	90	1.5	98/ 2
7[a]	↑	0.1	2.0	85	1.3	99/ 1
8[a]	↑	0.3	5.0	87	1.4	98/ 2

a) 1.0 mmol of **4c** was used.

Entries 1–5

To a solution of phosphate **1** (95.7 mg, 0.400 mmol) in *Solvent* (4.7 mL), a 2.0 M solution of isopropylmagnesium chloride in tetrahydrofuran (0.18 mL, 0.360 mmol) was added at –78 °C. After the reaction mixture was stirred at –78 °C for 30 min, a solution of 3-phenylpropanal **4c** in *Solvent* (2.0 mL) was added at room temperature, and the reaction mixture was stirred at room temperature for 1.5 h. To the reaction mixture, saturated aqueous ammonium chloride was added at 0 °C, and the mixture was extracted with ethyl acetate. The organic layer was dried over sodium sulfate. After filtration of the mixture and concentration of the solvent, the crude mixture was purified by thin layer chromatography on silica (eluant; hexane/ethyl acetate = 1/1) to afford alkene (*E*)-**5c** and alkene (*Z*)-**5c**.

Entry 1: 3-phenylpropanal **4c** (26.5 mg, 0.197 mmol), (*E*)-**5c** (40.8 mg, 94%), (*Z*)-**5c** (0.4 mg, 0.9%)
Entry 2: 3-phenylpropanal **4c** (26.8 mg, 0.200 mmol), (*E*)-**5c** (34.3 mg, 78%), (*Z*)-**5c** (trace)
Entry 3: 3-phenylpropanal **4c** (27.0 mg, 0.201 mmol), (*E*)-**5c** (10.0 mg, 23%), (*Z*)-**5c** (0 mg, 0%)
Entry 4: 3-phenylpropanal **4c** (26.6 mg, 0.198 mmol), (*E*)-**5c** (6.3 mg, 15%), (*Z*)-**5c** (trace)
Entry 5: 3-phenylpropanal **4c** (26.4 mg, 0.197 mmol), (*E*)-**5c** (7.2 mg, 17%), (*Z*)-**5c** (1.2 mg, 2.8%)

Entries 6–8

To a solution of phosphate **1** (478 mg, 2.00 mmol) in tetrahydrofuran (Entry 6: 25 mL, Entry 7: 6.5 mL, Entry 8: 2.0 mL), a 2.0 M solution of isopropylmagnesium chloride in tetrahydrofuran (0.90 mL, 1.80 mmol) was added at –78 °C. After the reaction mixture was stirred at –78 °C for 30 min, a solution of 3-phenylpropanal **4c** in tetrahydrofuran (Entry 6: 8.0 mL, Entry 7: 3.5 mL, Entry 8: 1.3 mL) was added at room temperature, and the reaction mixture was stirred at room temperature for *Time*. To the reaction mixture, saturated aqueous ammonium chloride was added at 0 °C, and the mixture was extracted with ethyl acetate. The organic layer was dried over sodium sulfate. After filtration of the mixture and concentration of the solvent, the crude mixture was purified by thin layer chromatography on silica (eluant; hexane/ethyl acetate = 1/1) to afford alkene (*E*)-**5c** and alkene (*Z*)-**5c**.

Entry 6: 3-phenylpropanal **4c** (133.9 mg, 0.998 mmol), (*E*)-**5c** (198 mg, 90%), (*Z*)-**5c** (3.3 mg, 1.5%)
Entry 7: 3-phenylpropanal **4c** (134.0 mg, 0.998 mmol), (*E*)-**5c** (186 mg, 85%), (*Z*)-**5c** (2.8 mg, 1.3%)
Entry 8: 3-phenylpropanal **4c** (134.0 mg, 0.998 mmol), (*E*)-**5c** (191 mg, 87%), (*Z*)-**5c** (3.0 mg, 1.4%)

Procedure for Isolation of Phosphonoenolate

Isolation of a Reactive Magnesium Phosphonoenolate and HWE Reaction of Isolated Magnesium Weinreb Amide-Type Phosphonoenolate.

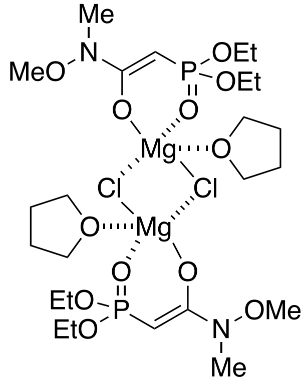

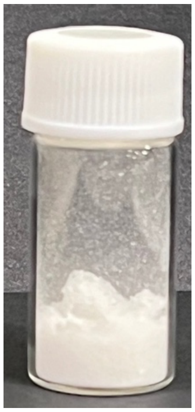

Magnesium phosphonoenolate (7)

To a solution of phosphate **1** (240 mg, 1.00 mmol) in tetrahydrofuran (7.1 mL), a 2.0 M solution of isopropylmagnesium chloride in tetrahydrofuran (0.50 mL, 1.00 mmol) was added at –78 °C. After the reaction mixture was stirred at –78 °C for 30 min and at room temperature for 15 min, the solvent was concentrated and dried under negative pressure with rotary pump to afford phosphonoenolate **7** (361 mg, 97%) as white solid.

FT-IR (KBr) ν_{max}: 3422, 2983, 2933, 1636, 1248, 1050, 1023 cm^{-1}

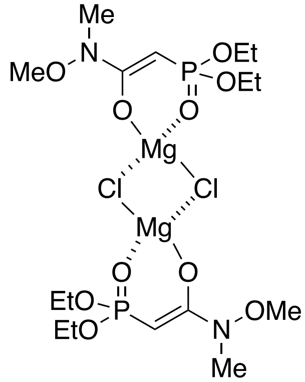

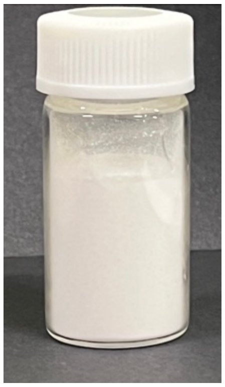

Magnesium phosphonoenolate (8)

To a solution of phosphate **1** (5.00 g, 20.9 mmol) in toluene (31.4 mL), a 2.0 M solution of isopropylmagnesium chloride in tetrahydrofuran (10.5 mL, 20.9 mmol) was added at –78 °C. After the reaction mixture was stirred at –78 °C for 30 min and at room temperature for 15 min, the solvent was concentrated and dried under negative pressure with rotary pump to afford phosphonoenolate **8** (6.64 g,[a] 97%) as white solid.

a) The purity of magnesium phosphonoenolate **8** was 91% because of containing little amount of toluene and tetrahydrofuran.

FT-IR (KBr) ν_{max}: 3398, 2985, 2941, 1636, 1249, 1049, 1023 cm^{-1}

In addition, lithium phosphonoenolate and ethyl ester-type phosphonoenolate were isolated with the same preparation's method.

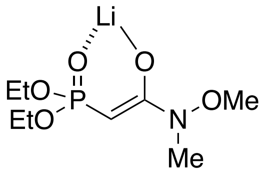

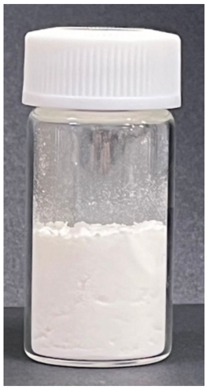

Lithium phosphonoenolate

To a solution of phosphate **1** (3.00 g, 12.5 mmol) in toluene (18.8 mL), a 1.6 M solution of *n*-butyllithium in hexane (8.0 mL, 12.5 mmol) was added at –78 °C. After the reaction mixture was stirred at –78 °C for 30 min and at room temperature for 15 min, the solvent was concentrated and dried under negative pressure with rotary pump to afford lithium phosphonoenolate (3.10 g, quant.) as a white solid.

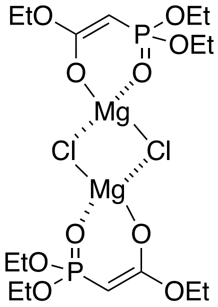

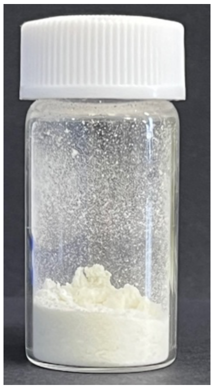

Magnesium ethyl ester-type phosphonoenolate

To a solution of phosphate **12** (3.00 g, 13.4 mmol) in toluene (20.1 mL), a 2.0 M solution of isopropylmagnesium chloride in tetrahydrofuran (6.7 mL, 13.4 mmol) was added at –78 °C. After the reaction mixture was stirred at –78 °C for 30 min and at room temperature for 15 min, the solvent was concentrated and dried under negative pressure with rotary pump to afford magnesium ethyl ester-type phosphonoenolate (4.24 g,[a] 96%) as a white solid.

a) The purity of magnesium ethyl ester-type phosphonoenolate was 86% because of containing little amount of toluene and tetrahydrofuran.

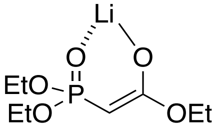

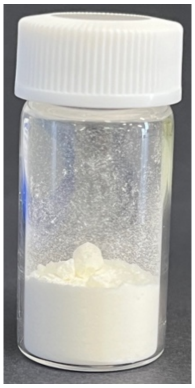

Lithium ethyl ester-type phosphonoenolate

To a solution of phosphate **12** (3.00 g, 13.4 mmol) in toluene (20.1 mL), a 1.6 M solution of *n*-butyllithium in hexane (8.6 mL, 13.4 mmol) was added at –78 °C. After the reaction mixture was stirred at –78 °C for 30 min and at room temperature for 15 min, the solvent was concentrated and dried under negative pressure with rotary pump to afford lithium ethyl ester-type phosphonoenolate (3.09 g,

quant.) as a white solid.

Table S10. HWE Reaction of Isolable Magnesium Phosphonoenolate.

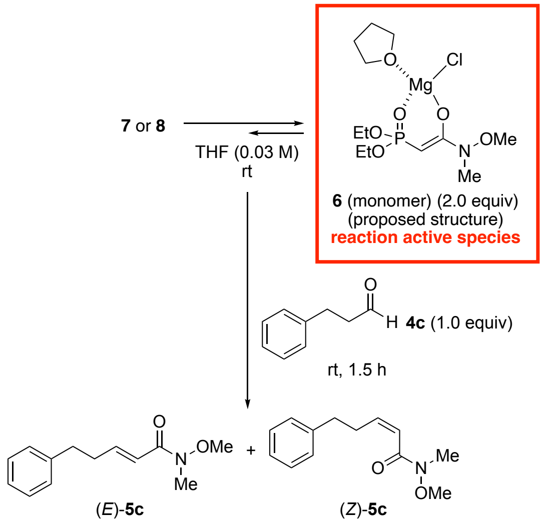

Origin of Phosphonoenolate	Yield (%) (*E*)-**5c**	Yield (%) (*Z*)-**5c**	*E/Z*
Control experiment data (Solution data)	89	0.5	99/1
Isolated phosphonoenolate **7** (from THF)	90	1.4	98/2
Isolated phosphonoenolate **8** (from toluene)	84	0.9	99/1

Entry 1 (Control experiment data)

To a solution of phosphate **1** (95.7 mg, 0.400 mmol) in tetrahydrofuran (4.7 mL), a 2.0 M solution of isopropylmagnesium chloride in tetrahydrofuran (0.20 mL, 0.400 mmol) was added at –78 °C. After the reaction mixture was stirred at –78 °C for 30 min, a solution of 3-phenylpropanal **4c** (27.0 mg, 0.201 mmol) in tetrahydrofuran (2.0 mL) was added at room temperature, and the reaction mixture was stirred for 1.5 h. To the reaction mixture, saturated aqueous ammonium chloride was added at 0 °C, and the mixture was extracted with ethyl acetate. The organic layer was dried over sodium sulfate. After filtration of the mixture and concentration of the solvent, the crude mixture was purified by thin layer chromatography on silica (eluant; hexane/ethyl acetate = 1/1) to afford alkene (*E*)-**5c** (39.4 mg, 89%) and alkene (*Z*)-**5c** (0.2 mg, 0.5%).

Entries 2,3

To a solution of phosphate **1** (95.7 mg, 0.400 mmol) in *solvent* (tetrahydrofuran or toluene) (4.7 mL), a 2.0 M solution of isopropylmagnesium chloride in tetrahydrofuran (0.20 mL, 0.400 mmol) was added at –78 °C. After the reaction mixture was stirred at –78 °C for 30 min and at room temperature for 15 min, the solvent was concentrated and dried under negative pressure with rotary pump to afford phosphonoenolate as white solid, and the phosphonoenolate was used in the next step without further purification.

To a solution of phosphonoenolate in tetrahydrofuran (4.7 mL), a solution of 3-phenylpropanal **4c** in tetrahydrofuran (2.0 mL) was added at room temperature. After the reaction mixture was stirred at room temperature for 1.5 h, saturated aqueous ammonium chloride was added at 0 °C. The mixture was extracted with ethyl acetate, and the organic layer was dried over sodium sulfate. After filtration of the mixture and concentration of the solvent, the crude mixture was purified by thin layer chromatography on silica (eluant; hexane/ethyl acetate = 1/1) to afford alkene (*E*)-**5c** and alkene (*Z*)-**5c**.

Entry 2: 3-phenylpropanal **4c** (26.6 mg, 0.198 mmol), (*E*)-**5c** (39.3 mg, 90%), (*Z*)-**5c** (0.6 mg, 1.4%)

Entry 3: 3-phenylpropanal **4c** (27.0 mg, 0.201 mmol), (*E*)-**5c** (37.2 mg, 84%), (*Z*)-**5c** (0.4 mg, 0.9%)

The Stability of Magnesium Phosphonoenolate (**8**)

To a solution of magnesium phosphonoenolate **8**[a] (137 mg,[b] 0.400 mmol) in tetrahydrofuran (4.7 mL), a solution of 3-phenylpropanal **4c** (26.8 mg, 0.200 mmol) in tetrahydrofuran (2.0 mL) was added at room temperature. After the reaction mixture was stirred at room temperature for 1.5 h, saturated aqueous ammonium chloride was added at 0 °C. The mixture was extracted with ethyl acetate, and the organic layer was dried over sodium sulfate. After filtration of the mixture and concentration of the solvent, the crude mixture was purified by thin layer chromatography on silica (eluant; hexane/ethyl acetate = 1/1) to afford alkene (*E*)-**5c** (38.4 mg, 88%) as colorless oil and alkene (*Z*)-**5c** (0.3 mg, 0.7%) as colorless oil.

a) Magnesium phosphonoenolate **8** was stored at room temperature under argon atmosphere for six months after preparation.
b) The purity of magnesium phosphonoenolate **8** was 87% because of containing little amount of toluene and tetrahydrofuran. Therefore, magnesium phosphonoenolate **8** was added based on the purity of it.

Lithium Phosphonoenolate and Ethyl Ester-Type Phosphonoenolate

Table S11. HWE Reaction of Isolable Lithium Phosphonoenolate and Ethyl Ester-Type Phosphonoenolate

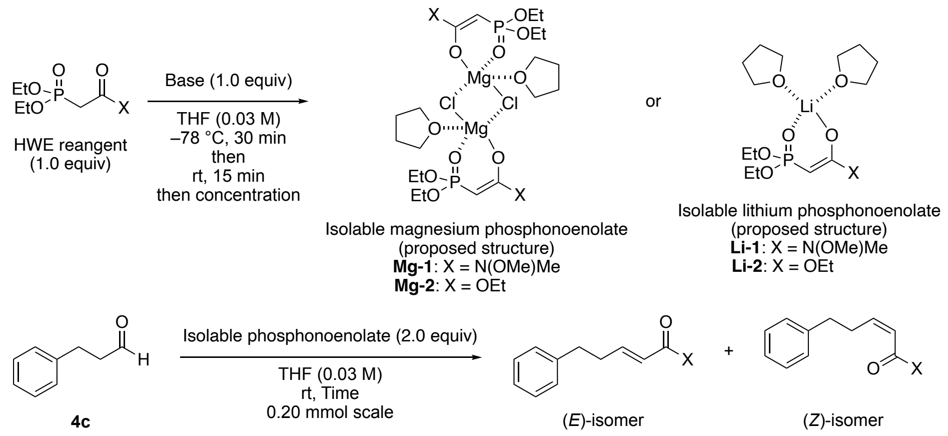

Entry	X	Base	Phosphonoenolate	Time	Yield (%) (*E*)-isomer	Yield (%) (*Z*)-isomer	*E*/*Z*
1	N(OMe)Me	^{i}PrMgCl	**Mg-1**	1.5 h	90	1.4	98/ 2
2	↑	^{n}BuLi	**Li-1**	20 min	80	12	87/13
3	OEt	^{i}PrMgCl	**Mg-2**	1.5 h	72	1.2	96/ 4
4	↑	^{n}BuLi	**Li-1**	20 min	80	8.1	91/ 9

To a solution of HWE reagent (0.400 mmol) in tetrahydrofuran (4.7 mL), *Base* (0.400 mmol) was added at –78 °C. After the reaction mixture was stirred at –78 °C for 30 min and at room temperature for 15 min, the solvent was concentrated and dried under negative pressure with rotary pump to afford phosphonoenolate as white solid, and the phosphonoenolate was used in the next step without further purification.

To a solution of phosphonoenolate in tetrahydrofuran (4.7 mL), a solution of 3-phenylpropanal **4c** in tetrahydrofuran (2.0 mL) was added at room temperature. After the reaction mixture was stirred at room temperature for *Time*, saturated aqueous ammonium chloride was added at 0 °C. The mixture was extracted with ethyl acetate, and the organic layer was dried over sodium sulfate. After filtration of the mixture and concentration of the solvent, the crude mixture was purified by thin layer chromatography on silica (eluant; hexane/ethyl acetate = 1/1 (Entries 1,2) or 7/1 (Entries 3,4)) to afford alkene (*E*)-isomer and alkene (*Z*)-isomer.

Entry 2: 3-phenylpropanal **4c** (26.8 mg, 0.200 mmol), (*E*)-**5c** (35.0 mg, 80%), (*Z*)-**5c** (5.3 mg, 12%)

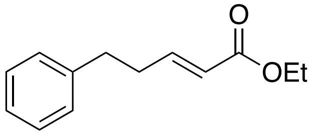

Ethyl (2*E*)-5-phenylpent-2-enoate ((*E*)-11).
R_f= 0.57 (silica gel, hexane/ethyl acetate = 7/1); FT-IR (neat) ν_{max}: 2985, 2931, 1720, 1658, 1311, 1265, 1196, 1149, 1041, 702 cm^{-1}; ^{1}H NMR (500 MHz, $CDCl_3$): δ 7.33–7.26 (m, 2H, Ar), 7.24–7.15 (m, 3H, Ar), 7.00 (dt, J = 15.5, 7.0 Hz, 1H, H-3), 5.85 (dt, J = 15.5, 1.5 Hz, 1H, H-2), 4.18 (q, J = 7.0 Hz, 2H, OEt), 2.78 (t, J = 7.5 Hz, 2H, H-5), 2.57–2.48 (m, 2H, H-4), 1.28 (t, J = 7.0 Hz, 3H, OEt); ^{13}C{^{1}H} NMR (125 MHz, $CDCl_3$): 166.5 (C-1), 148.0 (C-3), 140.7 (Ar), 128.4 (Ar), 128.3 (Ar), 126.1 (Ar), 121.8 (C-2), 60.1 (OEt), 34.3 (C-5), 33.8 (C-4), 14.2 (OEt); HRMS calcd for $C_{13}H_{16}O_2Na$ [M + Na]$^+$ 227.1043, found 227.1044.

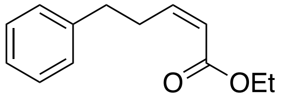

Ethyl (2*Z*)-5-phenylpent-2-enoate ((*Z*)-11).
R_f= 0.70 (silica gel, hexane/ethyl acetate = 7/1); FT-IR (neat) ν_{max}: 2985, 2931, 1720, 1643, 1450, 1412, 1389, 1188, 1165, 1034, 825, 702 cm^{-1}; ^{1}H NMR (500 MHz, $CDCl_3$): δ 7.32–7.26 (m, 2H, Ar), 7.25–7.14 (m, 3H, Ar), 6.24 (dt, J = 11.5, 7.5 Hz, 1H, H-3), 5.78 (dt, J = 11.5, 1.5 Hz, 1H, H-2), 4.16 (q, J = 7.0 Hz, 2H, OEt), 2.99 (dtd, J = 7.5, 7.5, 1.5 Hz, 2H, H-4), 2.77 (t, J = 7.5 Hz, 2H, H-5), 1.28 (t, J = 7.0 Hz, 3H, OEt); ^{13}C{^{1}H} NMR (125 MHz, $CDCl_3$): 166.3 (C-1), 148.9 (C-3), 141.1 (Ar), 128.4 (Ar), 128.3 (Ar), 126.0 (Ar), 120.3 (C-2), 59.8 (OEt), 35.0 (C-5), 30.4 (C-4), 14.3 (OEt); HRMS calcd for $C_{13}H_{16}O_2Na$ [M + Na]$^+$ 227.1043, found 227.1047.
Entry 3: 3-phenylpropanal **4c** (26.8 mg, 0.200 mmol), (*E*)-**11** (29.2 mg, 72%), (*Z*)-**11** (0.5 mg, 1.2%)
Entry 4: 3-phenylpropanal **4c** (26.9 mg, 0.200 mmol), (*E*)-**11** (32.9 mg, 80%), (*Z*)-**11** (3.3 mg, 8.1%)

Procedure for Investigating the Effect of Phosphonate Ester Group
Effect of Phosphonate Ester Group in Weinreb Amide-Type Horner–Wadsworth–Emmons Reagent.

Table S12. Effect of Phosphonate Ester Group on the Selectivity of Weinreb Amide-Type HWE Reaction with 3-Phenylpropanal.

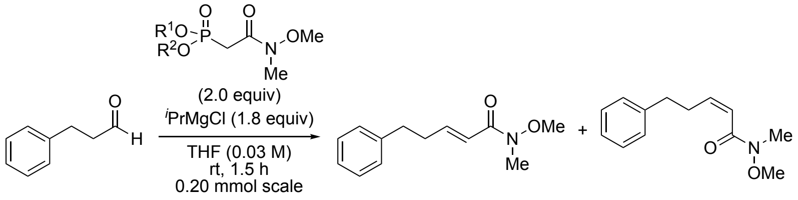

4c (*E*)-**5c** (*Z*)-**5c**

Entry	R^1O	R^2O	Yield (%) (*E*)-**5c**	(*Z*)-**5c**	*E*/*Z*
1	MeO	MeO	91	3.0	97/ 3
2	EtO	EtO	94	0.9	99/ 1
3	^{i}PrO	^{i}PrO	41	0.9	98/ 2
4	PhO	PhO	46	45	50/50
5	CF_3CH_2O	OEt	83	4.8	95/ 5
6	CF_3CH_2O	CF_3CH_2O	17	70	19/81

To a solution of phosphate (0.400 mmol) in tetrahydrofuran (4.7 mL), a 2.0 M solution of isopropylmagnesium chloride in tetrahydrofuran (0.18 mL, 0.360 mmol) was added at –78 °C. After the

reaction mixture was stirred at –78 °C for 30 min, a solution of 3-phenylpropanal **4c** in tetrahydrofuran (2.0 mL) was added at room temperature, and the reaction mixture was stirred at room temperature for 1.5 h. To the reaction mixture, saturated aqueous ammonium chloride was added at 0 °C, and the mixture was extracted with ethyl acetate. The organic layer was dried over sodium sulfate. After filtration of the mixture and concentration of the solvent, the crude mixture was purified by thin layer chromatography on silica (eluant; hexane/ethyl acetate = 1/1) to afford alkene (*E*)-**5c** and alkene (*Z*)-**5c**.
Entry 1: 3-phenylpropanal **4c** (26.7 mg, 0.199 mmol), (*E*)-**5c** (39.5 mg, 91%), (*Z*)-**5c** (1.3 mg, 3.0%)
Entry 3: 3-phenylpropanal **4c** (26.7 mg, 0.199 mmol), (*E*)-**5c** (17.8 mg, 41%), (*Z*)-**5c** (0.4 mg, 0.9%)
Entry 4: 3-phenylpropanal **4c** (27.0 mg, 0.201 mmol), (*E*)-**5c** (20.3 mg, 46%), (*Z*)-**5c** (19.9 mg, 45%)
Entry 5: 3-phenylpropanal **4c** (26.9 mg, 0.200 mmol), (*E*)-**5c** (36.3 mg, 83%), (*Z*)-**5c** (2.1 mg, 4.8%)
Entry 6: 3-phenylpropanal **4c** (26.5 mg, 0.197 mmol), (*E*)-**5c** (7.3 mg, 17%), (*Z*)-**5c** (30.4 mg, 70%)

Table S13. Effect of Phosphonate Ester Group on the Selectivity of Weinreb Amide-Type HWE Reaction with Benzaldehyde.

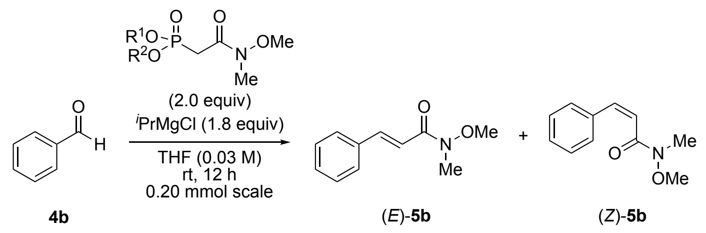

Entry	R^1O	R^2O	Yield (%) (*E*)-**5b**	Yield (%) (*Z*)-**5b**	*E*/*Z*
1	MeO	MeO	98	0	100/ 0
2	EtO	EtO	93	0	100/ 0
3	^{i}PrO	^{i}PrO	98	0	100/ 0
4	PhO	PhO	87	0	100/ 0
5	CF_3CH_2O	OEt	86	0	100/ 0
6	CF_3CH_2O	CF_3CH_2O	58	0	100/ 0

In addition, the effect of HWE reagents with the structure of aldehyde and acetal instead of Weinreb amide was investigated on reactivity and selectivity.

Table S14. Effect of the Structure of Aldehyde and Acetal on the Selectivity of HWE Reaction.

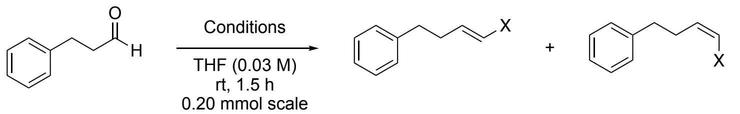

4c (*E*)-isomer (*Z*)-isomer

Entry	Conditions		X	Yield (%) (*E*)-isomer	Yield (%) (*Z*)-isomer	*E*/*Z*
1	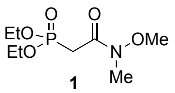 **1**	(2.0 equiv), ^{i}PrMgCl (1.8 equiv)	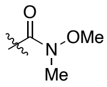	94	0.9	99/1
2	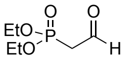	(1.2 equiv), LHMDS (1.1 equiv)	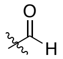	49	2.8	95/5
3	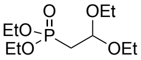	(1.2 equiv), LHMDS (1.1 equiv)	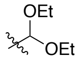	0	0	–
4	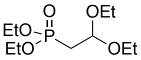	(1.2 equiv), ^{n}BuLi (1.1 equiv)	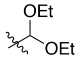	0	0[a]	–

a) *Z*-isomer (X = CHO) was given in a 5.9% yield.

Procedure for Comparison among Elongation Reagents

Comparison among Wittig Reagents and HWE Reagents, and among Ethyl Ester-Type Reagents and Weinreb Amide-Type Reagents.

Table S15. Comparison among Wittig Reagents and HWE Reagents and among Ethyl Ester-Type Reagents and Weinreb Amide-Type Reagents.

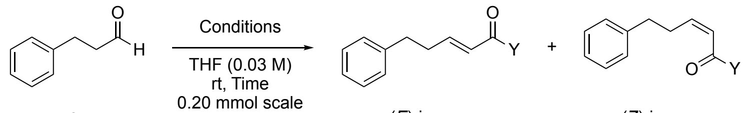

4c (*E*)-isomer (*Z*)-isomer

Entry		Conditions	Y	Time	Yield (%) (*E*)-isomer	(*Z*)-isomer	rSM	*E*/*Z*
1	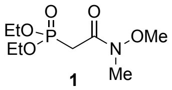 **1**	(1.2 equiv), ^{n}BuLi (1.1 equiv)	N(OMe)Me	20 min	79	12	0	87/13
2		(2.0 equiv), ^{i}PrMgCl (1.8 equiv)	↑	1.5 h	94	0.9	0	99/ 1
3	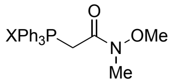 **9** (X = Br, Cl (Br / Cl = 15/1))	(1.2 equiv), ^{n}BuLi (1.1 equiv)	↑	20 min	48	6.8	29	88/12
4		(2.0 equiv), ^{i}PrMgCl (1.8 equiv)	↑	1.5 h	68	12	11	85/15
5	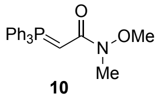 **10**	(1.8 equiv)	↑	1.5 h	24	5.2	49	82/18
6	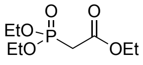 **12**	(1.2 equiv), ^{n}BuLi (1.1 equiv)	OEt	20 min	75	8.5	0	90/10
7		(2.0 equiv), ^{i}PrMgCl (1.8 equiv)	↑	1.5 h	78	1.2	0	98/ 2
8	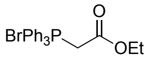 **13**	(1.2 equiv), ^{n}BuLi (1.1 equiv)	↑	20 min	74	11	0	87/13
9		(2.0 equiv), ^{i}PrMgCl (1.8 equiv)	↑	1.5 h	80	10	0	89/11
10	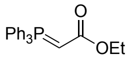 **14**	(1.8 equiv)	↑	1.5 h	43	3.9	21	92/ 8

Entries 1,3,6,8

To a solution of elongation reagent (0.240 mmol) in tetrahydrofuran (4.7 mL), a 1.6 M solution of *n*-butyllithium in hexane in tetrahydrofuran (0.14 mL, 0.220 mmol) was added at –78 °C. After the reaction mixture was stirred at –78 °C for 30 min, a solution of 3-phenylpropanal **4c** in tetrahydrofuran (2.0 mL) was added at room temperature, and the reaction mixture was stirred at room temperature for 20 min. To the reaction mixture, saturated aqueous ammonium chloride was added at 0 °C, and the mixture was extracted with ethyl acetate. The organic layer was dried over sodium sulfate. After filtration of the mixture and concentration of the solvent, the crude mixture was purified by thin layer chromatography on silica (eluant; hexane/ethyl acetate = 1/1 (Entries 1,3) or 7/1 (Entries 6,8)) to afford alkene (*E*)-isomer and alkene (*Z*)-isomer.

Entries 2,4,7,9

To a solution of elongation reagent (0.400 mmol) in tetrahydrofuran (4.7 mL), a 2.0 M solution of isopropylmagnesium chloride in tetrahydrofuran (0.18 mL, 0.360 mmol) was added at –78 °C. After the reaction mixture was stirred at –78 °C for 30 min, a solution of 3-phenylpropanal **4c** in tetrahydrofuran (2.0 mL) was added at room temperature, and the reaction mixture was stirred at room temperature for 1.5 h. To the reaction mixture, saturated aqueous ammonium chloride was added at 0 °C, and the mixture was extracted with ethyl acetate. The organic layer was dried over sodium sulfate. After

filtration of the mixture and concentration of the solvent, the crude mixture was purified by thin layer chromatography on silica (eluant; hexane/ethyl acetate = 1/1 (Entries 2,4) or 7/1 (Entries 7,9)) to afford alkene (*E*)-isomer and alkene (*Z*)-isomer.

Entries 5,10

To a solution of Wittig reagent (0.400 mmol) in tetrahydrofuran (4.7 mL), a solution of 3-phenylpropanal **4c** in tetrahydrofuran (2.0 mL) was added at room temperature. After the reaction mixture was stirred at room temperature for 1.5 h, the mixture was filtered through a short pad of silica (eluant; hexane/ethyl acetate = 1/1) to afford the crude mixture. The crude mixture was purified by thin layer chromatography on silica (eluant; hexane/ethyl acetate = 2/1 (Entry 5) or 7/1 (Entry 10)) to afford alkene (*E*)-isomer and alkene (*Z*)-isomer.

Entry 3: 3-phenylpropanal **4c** (26.9 mg, 0.200 mmol), (*E*)-**5c** (21.0 mg, 48%), (*Z*)-**5c** (3.0 mg, 6.8%), recovery of starting material (**4c**) (7.8 mg, 29%)

Entry 4: 3-phenylpropanal **4c** (26.5 mg, 0.197 mmol), (*E*)-**5c** (29.3 mg, 68%), (*Z*)-**5c** (5.3 mg, 12%), recovery of starting material (**4c**) (2.9 mg, 11%)

Entry 5: 3-phenylpropanal **4c** (27.2 mg, 0.203 mmol), (*E*)-**5c** (10.7 mg, 24%), (*Z*)-**5c** (2.3 mg, 5.2%), recovery of starting material (**4c**) (13.3 mg, 49%)

Entry 6: 3-phenylpropanal **4c** (27.0 mg, 0.201 mmol), (*E*)-**11** (30.8 mg, 75%), (*Z*)-**11** (3.5 mg, 8.5%), recovery of starting material (**4c**) (0 mg, 0%)

Entry 7: 3-phenylpropanal **4c** (26.8 mg, 0.200 mmol), (*E*)-**11** (32.0 mg, 78%), (*Z*)-**11** (0.5 mg, 1.2%), recovery of starting material (**4c**) (0 mg, 0%)

Entry 8: 3-phenylpropanal **4c** (26.5 mg, 0.197 mmol), (*E*)-**11** (30.0 mg, 74%), (*Z*)-**11** (4.3 mg, 11%), recovery of starting material (**4c**) (0 mg, 0%)

Entry 9: 3-phenylpropanal **4c** (26.9 mg, 0.200 mmol), (*E*)-**11** (32.7 mg, 80%), (*Z*)-**11** (4.1 mg, 10%), recovery of starting material (**4c**) (0 mg, 0%)

Entry 10: 3-phenylpropanal **4c** (27.2 mg, 0.203 mmol), (*E*)-**11** (17.9 mg, 43%), (*Z*)-**11** (1.6 mg, 3.9%), recovery of starting material (**4c**) (5.8 mg, 21%)

Procedure for Substrate Scope

Substrate Scope of Saturated Aliphatic Aldehydes for the ^{i}PrMgCl-deprotonating Weinreb Amide-Type Horner–Wadsworth–Emmons Reaction

Table S16. Substrate Scope of Saturated Aliphatic Aldehyde in Weinreb Amide-Type HWE Reaction.

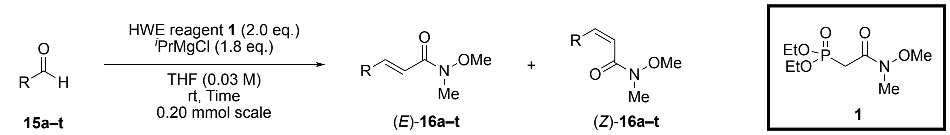

Entry	Substrate (15)	Time	Yield (%) (E)-16	Yield (%) (Z)-16	E/Z
1[a]	(a)	1.5 h	–	0[b]	100/0
2[a]	(a)	12 h	–	0[b]	100/0
3[a]	(b)	1.5 h	–	0[b]	100/0
4[a]	(b)	12 h	–	0[b]	100/0
5	(c)	1.5 h	86	0	100/0
6	(c)	12 h	80	0	100/0
7	(d)	1.5 h	90	0	100/0
8	(d)	12 h	78	0	100/0
9	(e)	1.5 h	94	0.9	99/1
10	(f)	1.5 h	83	0	100/0
11	(f)	12 h	81	0	100/0
12	(g)	1.5 h	89	0.7	99/1
13	(g)	12 h	86	trace	>99/1
14	(h)	1.5 h	81	0.7	99/1
15	(i)	1.5 h	82	0	100/0
16	(i)	12 h	85	0	100/0
17	(j)	1.5 h	93	trace	>99/1
18	(j)	12 h	87	1.0	99/1
19	(k)	12 h	75[c]	1.5[c]	98/2[c]
20[d]	(k)	12 h	91[e]	2.8[e]	97/3[e]
21	(l)	1.5 h	89	2.7	97/3
22	(l)	12 h	91	1.3	98/2
23	(m)	1.5 h	78	2.9	96/4
24	(m)	12 h	83	1.6	98/2
25	(n)	1.5 h	75	0.8	99/1
26	(n)	12 h	79	0.8	99/1
27	(o)	1.5 h	88	trace	>99/1
28	(o)	12 h	88	trace	>99/1
29	(p)	20 min	90	1.8	98/2
30	(q)	20 min	86	trace	>99/1
31[f]	(r)	1.5 h	46	1.1	98/2
32[f]	(r)	12 h	87	1.1	99/1
33	(s)	1.5 h	0	0	–
34	(s)	12 h	0	0	–
35[g]	(s)	12 h	0	0	–
36[h]	(t)	1.5 h	0	0	–
37[h]	(t)	12 h	0	0	–
38[g,h]	(t)	12 h	0	0	–

a) Reaction (*E*)-products were highly vilatile so that we cannot get the accurate yield. Only (*E*)-selectivity was calculated from ^{1}H NMR spetra of crude product.
b) (*Z*)-product was not detected in TLC monitoring. See also note a).
c) The yield of (*E*)-**16** is that of (*E*,*E*)-**16k** that two formyl groups were reacted. (*E*)-**16k** that one of two formyl groups was reacted was also given in 17% yield. (*E*,*Z*)-**16k** was also given in 1.5% yield. The ratio of *E*/*Z* is the ratio of (*E*,*E*)-**16k**/(*E*,*Z*)-**16k**. (*Z*,*Z*)-**16k** was not detected.
d) HWE reagent (3.0 equiv), ^{i}PrMgCl (2.7 eq.) were used.
e) The yield of (*E*)-**16** is that of (*E*,*E*)-**16k** that two formyl groups were reacted. (*E*,*Z*)-**16k** was also given in 2.8% yield. The ratio of *E*/*Z* is the ratio of (*E*,*E*)-**16k**/(*E*,*Z*)-**16k**. (*Z*,*Z*)-**16k** was not detected.
f) Purity: 91%
g) HWE reagent (3.0 equiv), ^{i}PrMgCl (2.7 eq.) were used and the reaction mixture was stirred unfer reflux.
h) Purity: 92%

To a solution of phosphate **1** (2.0 equiv: 95.7 mg, 0.400 mmol, 3.0 equiv: 144 mg, 0.600 mmol) in tetrahydrofuran (4.7 mL), a 2.0 M solution of isopropylmagnesium chloride in tetrahydrofuran (1.8 equiv: 0.18 mL, 0.360 mmol, 2.7 equiv: 0.27 mL, 0.540 mmol) was added at –78 °C. After the reaction mixture was stirred at –78 °C for 30 min, a solution of *Substrate* **15a–t** (0.200 mmol) in tetrahydrofuran (2.0 mL) was added at *Temperature* (room temperature or reflux), and the reaction mixture was stirred for *Time*. To the reaction mixture, saturated aqueous ammonium chloride was added at 0 °C, and the mixture was extracted with ethyl acetate. The organic layer was dried over sodium sulfate. After filtration of the mixture and concentration of the solvent, the crude mixture was purified by thin layer chromatography on silica to afford alkene **16a–t**.

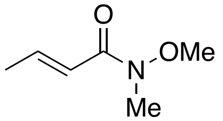

(2*E*)-*N*-Methoxy-*N*-methylbut-2-enamide ((*E*)-16a).

Acetaldehyde **15a** (8.8 mg, 0.200 mmol) was employed. The reaction time was 1.5 h. Purification by thin layer chromatography on silica (eluant; hexane/ethyl acetate = 1/1) to afford alkene (*E*)-**16a** as colorless oil.

R_f = 0.55 (silica gel, hexane/ethyl acetate = 2/1); FT-IR (neat) ν_{max}: 2970, 2939, 1666, 1635, 1450, 1412, 1381, 1180, 1011, 964 cm^{-1}; ^{1}H NMR (500 MHz, $CDCl_3$): δ 6.99 (dq, *J* = 15.5, 7.0 Hz, 1H, H-3), 6.49–6.37 (m, 1H, H-2), 3.71 (s, 3H, OMe), 3.24 (s, 3H, NMe), 1.92 (dd, *J* = 7.0, 1.0 Hz, 3H, H-4); ^{13}C{^{1}H} NMR (125 MHz, $CDCl_3$): 166.9 (C-1), 142.8 (C-3), 120.1 (C-2), 61.6 (OMe), 32.2 (NMe), 18.2 (C-4); HRMS calcd for $C_6H_{11}NO_2Na$ [M + Na]$^+$ 152.0682, found 152.0685.

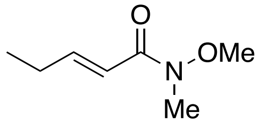

(2*E*)-*N*-Methoxy-*N*-methylpent-2-enamide ((*E*)-16b).

Propanal **15b** (11.6 mg, 0.200 mmol) was employed. The reaction time was 1.5 h. Purification by thin layer chromatography on silica (eluant; hexane/ethyl acetate = 1/1) to afford alkene (*E*)-**16b** as colorless oil.

R_f = 0.36 (silica gel, hexane/ethyl acetate = 2/1); FT-IR (neat) ν_{max}: 2970, 2939, 1666, 1635, 1458, 1412, 1381, 1180, 995 cm^{-1}; ^{1}H NMR (500 MHz, $CDCl_3$): δ 7.03 (dt, *J* = 15.5, 6.5 Hz, 1H, H-3), 6.39 (dt, *J* = 15.5, 1.5 Hz, 1H, H-2), 3.71 (s, 3H, OMe), 3.24 (s, 3H, NMe), 2.32–2.22 (m, 2H, H-4), 1.09 (t, *J* = 7.5 Hz, 3H, H-5); ^{13}C{^{1}H} NMR (125 MHz, $CDCl_3$): 167.1 (C-1), 149.2 (C-3), 117.7 (C-2), 61.6 (OMe), 32.3 (NMe), 25.5 (C-4), 12.5 (C-5); HRMS calcd for $C_7H_{13}NO_2Na$ [M + Na]$^+$ 166.0838, found 166.0835.

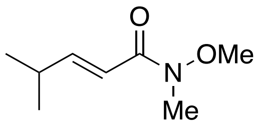

(2*E*)-*N*-Methoxy-*N*,4-dimethylpent-2-enamide ((*E*)-16c).

2-Methylpropanal **15c** (14.3 mg, 0.198 mmol) was employed. The reaction time was 1.5 h. Purification by thin layer chromatography on silica (eluant; hexane/ethyl acetate = 1/1) to afford alkene (*E*)-**16c** (26.8 mg, 86%) as colorless oil.

R_f = 0.44 (silica gel, hexane/ethyl acetate = 2/1); FT-IR (neat) ν_{max}: 2962, 2870, 1666, 1635, 1466, 1412, 1381, 1180, 1003, 957 cm^{-1}; ^{1}H NMR (500 MHz, $CDCl_3$): δ 6.96 (dd, *J* = 15.5, 6.5 Hz, 1H, H-3), 6.34 (dd, *J* = 15.5, 1.5 Hz, 1H, H-2), 3.71 (s, 3H, OMe), 3.24 (s, 3H, NMe), 2.50 (qqdd, *J* = 7.0, 7.0, 6.5, 1.5 Hz, 1H, H-4), 1.08 (d, *J* = 7.0 Hz, 6H, 4-Me, H-5); ^{13}C{^{1}H} NMR (125 MHz, $CDCl_3$): 167.2 (C-1), 154.0 (C-3), 115.8 (C-2), 61.5 (OMe), 32.3 (NMe), 31.1 (C-4), 21.4 (4-Me, C-5); HRMS calcd for $C_8H_{15}NO_2Na$ [M + Na]$^+$ 180.0995, found 180.0991.

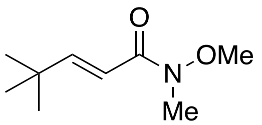

(2*E*)-*N*-Methoxy-*N*,4,4-trimethylpent-2-enamide ((*E*)-16d).

2,2-Dimethylpropanal **15d** (17.4 mg, 0.202 mmol) was employed. The reaction time was 1.5 h. Purification by thin layer chromatography on silica (eluant; hexane/ethyl acetate = 2/1) to afford alkene (*E*)-**16d** (31.0 mg, 90%) as colorless oil.

R_f = 0.52 (silica gel, hexane/ethyl acetate = 2/1); FT-IR (neat) ν_{max}: 2962, 2908, 2870, 1666, 1628, 1466,

1412, 1381, 1180, 1003 cm^{-1}; ^{1}H NMR (500 MHz, $CDCl_3$): δ 7.00 (d, J = 16.0 Hz, 1H, H-3), 6.30 (d, J = 16.0 Hz, 1H, H-2), 3.71 (s, 3H, OMe), 3.25 (s, 3H, NMe), 1.10 (s, 9H, 4-Me, H-5); ^{13}C{^{1}H} NMR (125 MHz, $CDCl_3$): 167.4 (C-1), 157.7 (C-3), 113.7 (C-2), 61.6 (OMe), 33.7 (C-4), 32.4 (NMe), 28.8 (4-Me, C-5); HRMS calcd for $C_{18}H_{34}N_2O_4Na$ $[2M + Na]^+$ 365.2411, found 365.2403.

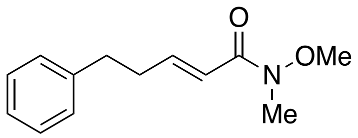

(2*E*)-*N*-Methoxy-*N*-methyl-5-phenylpent-2-enamide ((*E*)-5c, (*E*)-16e).

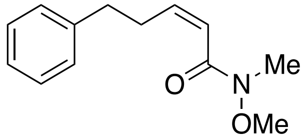

(2*Z*)-*N*-Methoxy-*N*-methyl-5-phenylpent-2-enamide ((*Z*)-5c, (*Z*)-16e).

3-Phenylpropanal **15e** (26.5 mg, 0.197 mmol) was employed. The reaction time was 1.5 h. Purification by thin layer chromatography on silica (eluant; hexane/ethyl acetate = 1/1) to afford alkene (*E*)-**16e** (40.8 mg, 94%) as colorless oil and alkene (*Z*)-**16e** (0.4 mg, 0.9%) as colorless oil.

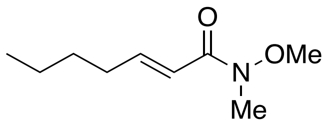

(2*E*)-*N*-Methoxy-*N*-methylhept-2-enamide ((*E*)-16f).

Pentanal **15f** (17.4 mg, 0.202 mmol) was employed. The reaction time was 1.5 h. Purification by thin layer chromatography on silica (eluant; hexane/ethyl acetate = 3/2) to afford alkene (*E*)-**16f** (28.8 mg, 83%) as colorless oil.

R_f= 0.44 (silica gel, hexane/ethyl acetate = 2/1); FT-IR (neat) ν_{max}: 2954, 2931, 2870, 1666, 1635, 1466, 1412, 1381, 995 cm^{-1}; ^{1}H NMR (500 MHz, $CDCl_3$): δ 6.98 (dt, J = 15.5, 7.0 Hz, 1H, H-3), 6.39 (dt, J = 15.5, 1.0 Hz, 1H, H-2), 3.70 (s, 3H, OMe), 3.24 (s, 3H, NMe), 2.24 (tdd, J = 7.5, 7.0, 1.0 Hz, 2H, H-4), 1.51–1.41 (m, 2H, H-5), 1.36 (tq, J = 7.5, 7.0 Hz, 2H, H-6), 0.91 (t, J = 7.0 Hz, 3H, H-7); ^{13}C{^{1}H} NMR (125 MHz, $CDCl_3$): 167.1 (C-1), 147.9 (C-3), 118.6 (C-2), 61.6 (OMe), 32.3 (NMe), 32.2 (C-4), 30.4 (C-5), 22.2 (C-6), 13.8 (C-7); HRMS calcd for $C_9H_{17}NO_2Na$ $[M + Na]^+$ 194.1151, found 194.1156.

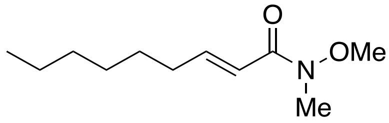

(2*E*)-*N*-Methoxy-*N*-methylnon-2-enamide ((*E*)-5a, (*E*)-16g).

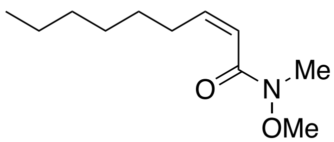

(2*Z*)-*N*-Methoxy-*N*-methylnon-2-enamide ((*Z*)-5a, (*Z*)-16g).

Heptanal **15g** (23.0 mg, 0.201 mmol) was employed. The reaction time was 1.5 h. Purification by thin layer chromatography on silica (eluant; hexane/ethyl acetate = 3/2) to afford alkene (*E*)-**16g** (35.6 mg, 89%) as colorless oil and alkene (*Z*)-**16g** (0.3 mg, 0.7%) as colorless oil.

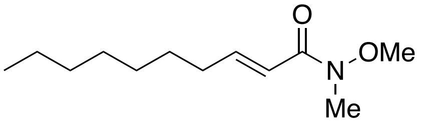

(2*E*)-*N*-Methoxy-*N*-methyldec-2-enamide ((*E*)-16h).

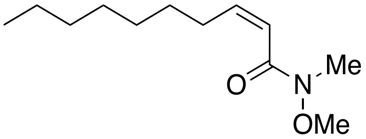

(2*Z*)-*N*-Methoxy-*N*-methyldec-2-enamide ((*Z*)-16h).

Octanal **15h** (25.6 mg, 0.200 mmol) was employed. The reaction time was 1.5 h. Purification by thin layer chromatography on silica (eluant; hexane/ethyl acetate = 2/1) to afford alkene (*E*)-**16h** (34.3 mg, 81%) as colorless oil and alkene (*Z*)-**16h** (0.3 mg, 0.7%) as colorless oil.

(*E*)-**16h**

R_f = 0.53 (silica gel, hexane/ethyl acetate = 2/1); FT-IR (neat) $\nu_{\max}$: 2931, 2854, 1666, 1635, 1466, 1412, 1381, 1180, 987 cm^{-1}; ^{1}H NMR (500 MHz, $CDCl_3$): δ 6.98 (dt, J = 15.5, 7.0 Hz, 1H, H-3), 6.39 (dt, J = 15.5, 1.0 Hz, 1H, H-2), 3.70 (s, 3H, OMe), 3.24 (s, 3H, NMe), 2.23 (dtd, J = 7.0, 6.5, 1.0 Hz, 2H, H-4), 1.47 (tt, J = 7.5, 6.5 Hz, 2H, H-5), 1.37–1.17 (m, 8H, H-6, H-7, H-8, H-9), 0.88 (t, J = 7.0 Hz, 3H, H-10); ^{13}C{^{1}H} NMR (125 MHz, $CDCl_3$): 167.0 (C-1), 148.0 (C-3), 118.5 (C-2), 61.6 (OMe), 32.4 (C-4), 32.3 (NMe), 31.7 (C-8), 29.1 (C-7), 29.0 (C-6), 28.2 (C-5), 22.6 (C-9), 14.0 (C-10); HRMS calcd for $C_{24}H_{46}N_2O_4Na$ [2M + Na]$^+$ 449.3350, found 449.3346.

(*Z*)-**16h**

R_f = 0.71 (silica gel, hexane/ethyl acetate = 2/1); FT-IR (neat) $\nu_{\max}$: 2924, 2854, 1658, 1458, 1435, 1342, 1180, 1003 cm^{-1}; ^{1}H NMR (500 MHz, $CDCl_3$): δ 6.23 (brd, J = 11.5 Hz, 1H, H-2), 6.12 (dt, J = 11.5, 7.0 Hz, 1H, H-3), 3.68 (s, 3H, OMe), 3.22 (s, 3H, NMe), 2.62 (dt, J = 7.0, 7.0 Hz, 2H, H-4), 1.43 (tt, J = 7.5, 7.0 Hz, 2H, H-5), 1.38–1.19 (m, 8H, H-6, H-7, H-8, H-9), 0.87 (t, J = 7.0 Hz, 3H, H-10); ^{13}C{^{1}H} NMR (125 MHz, $CDCl_3$): 167.6 (C-1), 147.8 (C-3), 117.9 (C-2), 61.4 (OMe), 31.9 (NMe), 31.8 (C-8), 29.308 (C-5, C-7), 29.13 (C-4), 29.09 (C-6), 22.6 (C-9), 14.1 (C-10); HRMS calcd for $C_{24}H_{46}N_2O_4Na$ [2M + Na]$^+$ 449.3350, found 449.3353.

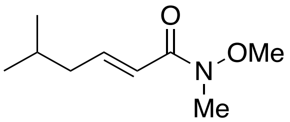

(2*E*)-*N*-Methoxy-*N*,5-dimethylhex-2-enamide ((*E*)-16i).

3-Methylbutanal **15i** (17.0 mg, 0.197 mmol) was employed. The reaction time was 1.5 h. Purification by thin layer chromatography on silica (eluant; hexane/ethyl acetate = 2/1) to afford alkene (*E*)-**16i** (27.6 mg, 82%) as colorless oil.

R_f = 0.42 (silica gel, hexane/ethyl acetate = 2/1); FT-IR (neat) $\nu_{\max}$: 2954, 1666, 1635, 1466, 1412, 1381, 995 cm^{-1}; ^{1}H NMR (500 MHz, $CDCl_3$): δ 6.96 (dt, J = 15.5, 7.5 Hz, 1H, H-3), 6.38 (d, J = 15.5 Hz, 1H, H-2), 3.70 (s, 3H, OMe), 3.24 (s, 3H, NMe), 2.17–2.08 (m, 2H, H-4), 1.77 (tqq, J = 7.5, 7.0, 7.0 Hz, 1H, H-5), 0.93 (d, J =7.0 Hz, 6H, 5-Me, H-6); ^{13}C{^{1}H} NMR (125 MHz, $CDCl_3$): 166.9 (C-1), 146.7 (C-3), 119.6 (C-2), 61.6 (OMe), 41.7 (C-4), 32.3 (NMe), 27.9 (C-5), 22.3 (5-Me, C-6); HRMS calcd for $C_{18}H_{34}N_2O_4Na$ [2M + Na]$^+$ 365.2411, found 365.2416.

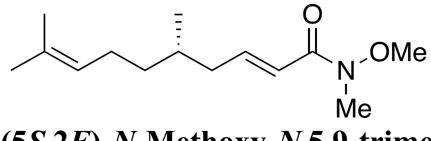

(5*S*,2*E*)-*N*-Methoxy-*N*,5,9-trimethyldeca-2,8-dienamide ((*E*)-16j).

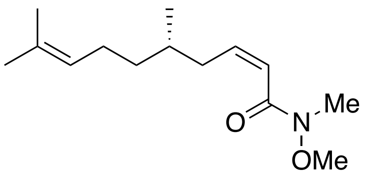

(5*S*,2*Z*)-*N*-Methoxy-*N*,5,9-trimethyldeca-2,8-dienamide ((*Z*)-16j).

(3*S*)-3,7-Dimethyloct-6-enal **15j** (31.0 mg, 0.201 mmol) was employed. The reaction time was 1.5 h. Purification by thin layer chromatography on silica (eluant; hexane/ethyl acetate = 2/1) to afford alkene (*E*)-**16j** (44.8 mg, 93%) as colorless oil and alkene (*Z*)-**16j** (0.1 mg, trace) as colorless oil.

(*E*)-**16j**

R_f= 0.26 (silica gel, hexane/ethyl acetate = 2/1); $[\alpha]_D^{21}$ +4.5 (*c* 1.00, $CHCl_3$); FT-IR (neat) ν_{max}: 2962, 2916, 1666, 1635, 1450, 1419, 1381, 995 cm^{-1}; ^1H NMR (500 MHz, $CDCl_3$): δ 6.96 (dt, J = 15.5, 7.5 Hz, 1H, H-3), 6.39 (d, J = 15.5 Hz, 1H, H-2), 5.12–5.04 (m, 1H, H-8), 3.70 (s, 3H, OMe), 3.25 (s, 3H, NMe), 2.30–2.20 (m, 1H, H-4), 2.14–1.89 (m, 3H, H-4, H-7), 1.69–1.58 (m, 1H, H-5), 1.68 (d, J = 1.0 Hz, 3H, H-10), 1.60 (s, 3H, 9-Me), 1.43–1.30 (m, 1H, H-6), 1.25–1.12 (m, 1H, H-6), 0.91 (d, J = 7.0 Hz, 3H, 5-Me); $^{13}C\{^1H\}$ NMR (125 MHz, $CDCl_3$): 166.9 (C-1), 146.7 (C-3), 131.3 (C-9), 124.4 (C-8), 119.7 (C-2), 61.6 (OMe), 39.9 (C-4), 36.6 (C-6), 32.3 (NMe), 32.2 (C-5), 25.7 (C-10), 25.5 (C-7), 19.5 (5-Me), 17.6 (9-Me); HRMS calcd for $C_{28}H_{50}N_2O_4Na$ $[2M + Na]^+$ 501.3663, found 501.3671.

(*Z*)-**16j**

R_f= 0.47 (silica gel, hexane/ethyl acetate = 2/1); $[\alpha]_D^{23}$ +12.9 (*c* 1.00, $CHCl_3$); FT-IR (neat) ν_{max}: 2962, 2916, 1658, 1442, 1342, 1003 cm^{-1}; ^1H NMR (500 MHz, $CDCl_3$): δ 6.28 (brd, J = 11.5 Hz, 1H, H-2), 6.13 (dt, J = 11.5, 7.0 Hz, 1H, H-3), 5.12–5.05 (m, 1H, H-8), 3.68 (s, 3H, OMe), 3.21 (s, 3H, NMe), 2.68–2.47 (m, 2H, H-4), 2.08–1.90 (m, 2H, H-7), 1.68 (s, 3H, H-10), 1.64–1.54 (m, 1H, H-5), 1.60 (s, 3H, 9-Me), 1.43–1.33 (m, 1H, H-6), 1.26–1.14 (m, 1H, H-6), 0.92 (d, J = 7.0 Hz, 3H, 5-Me); $^{13}C\{^1H\}$ NMR (125 MHz, $CDCl_3$): 167.6 (C-1), 146.7 (C-3), 131.1 (C-9), 124.7 (C-8), 118.7 (C-2), 61.4 (OMe), 36.7 (C-6), 36.0 (C-4), 32.9 (C-5), 31.9 (NMe), 25.7 (C-10), 25.5 (C-7), 19.5 (5-Me), 17.6 (9-Me); HRMS calcd for $C_{28}H_{50}N_2O_4Na$ $[2M + Na]^+$ 501.3663, found 501.3645.

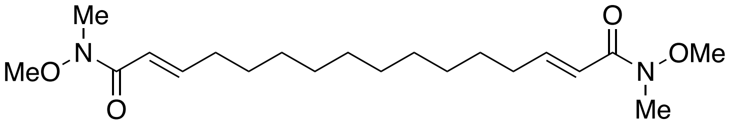

(2*E*,14*E*)-*N*¹,*N*¹⁶-Dimethoxy-*N*¹,*N*¹⁶-dimethylhexadeca-2,14-dienediamide ((*E*,*E*)-16k).

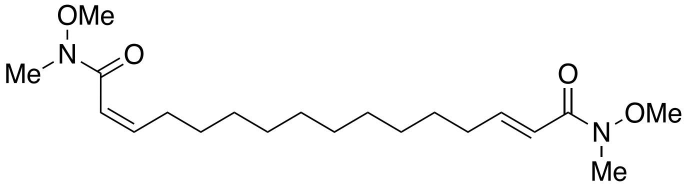

(2*E*,14*Z*)-*N*¹,*N*¹⁶-Dimethoxy-*N*¹,*N*¹⁶-dimethylhexadeca-2,14-dienediamide ((*E*,*Z*)-16k).

1,12-Dodecanedial **15k** (39.8 mg, 0.201 mmol) was employed. Phosphate **1** (144 mg, 0.600 mmol) and a 2.0 M solution of isopropylmagnesium chloride in tetrahydrofuran (0.27 mL, 0.540 mmol) were used. The reaction time was 12 h. Purification by thin layer chromatography on silica (eluant; hexane/ethyl acetate = 1/2) to afford diene (*E*,*E*)-**16k** (67.5 mg, 91%) as colorless oil and diene (*E*,*Z*)-**16k** (2.1 mg, 2.8) as colorless oil.

(*E*,*E*)-**16k**

R_f= 0.20 (silica gel, hexane/ethyl acetate = 1/1); FT-IR (neat) ν_{max}: 2924, 2854, 1666, 1635, 1466, 1442, 1381, 995 cm^{-1}; ^1H NMR (500 MHz, $CDCl_3$): δ 6.98 (dt, J = 15.5, 7.5 Hz, 2H, H-3, H-14), 6.39 (d, J = 15.5 Hz, 2H, H-2, H-15), 3.70 (s, 6H, OMe), 3.24 (s, 6H, NMe), 2.23 (dt, J = 7.5, 7.0 Hz, 4H, H-4, H-13), 1.46 (tt, J = 7.5, 7.0 Hz, 4H, H-5, H-12), 1.37–1.18 (m, 12H, H-6, H-7, H-8, H-9, H-10, H-11); $^{13}C\{^1H\}$ NMR (125 MHz, $CDCl_3$): 167.1 (C-1, C-16), 147.9 (C-3, C-14), 118.5 (C-2, C-15), 61.6 (OMe), 32.5 (C-4, C-13), 32.3 (NMe), 29.5 (C-6 or C-7 or C-8 or C-9 or C-10 or C-11), 29.3 (C-6 or

C-7 or C-8 or C-9 or C-10 or C-11), 29.1 (C-6 or C-7 or C-8 or C-9 or C-10 or C-11), 28.3 (C-5, C-12); HRMS calcd for $C_{20}H_{36}N_2O_4Na$ [M + Na]$^+$ 391.2567, found 391.2565.

(*E*,*Z*)-**16k**

R_f= 0.30 (silica gel, hexane/ethyl acetate = 1/1); ATR-IR ν_{max}: 2927, 2854, 1664, 1634, 1464, 1441, 1000 cm^{-1}; ^{1}H NMR (500 MHz, $CDCl_3$): δ 6.98 (dt, J = 15.5, 7.0 Hz, 1H, H-3), 6.39 (d, J = 15.5 Hz, 1H, H-2), 6.23 (brd, J = 12.0 Hz, 1H, H-15), 6.12 (dt, J = 12.0, 7.0 Hz, 1H, H-14), 3.70 (s, 3H, 1NOMe), 3.68 (s, 3H, 16NOMe), 3.24 (s, 3H, 1NMe), 3.21 (s, 3H, 16NMe), 2.61 (td, J = 7.5, 7.0 Hz, 2H, H-13), 2.23 (td, J = 7.5, 7.0 Hz, 2H, H-4), 1.51–1.38 (m, 4H, H-5, H-12), 1.38–1.19 (m, 12H, H-6, H-7, H-8, H-9, H-10, H-11); ^{13}C{^{1}H} NMR (125 MHz, $CDCl_3$): 167.5 (C-16), 167.1 (C-1), 148.0 (C-3), 147.8 (C-14), 118.6 (C-2), 117.9 (C-15), 61.6 (1NOMe), 61.4 (16NOMe), 32.5 (C-4), 32.3 (1NMe), 32.0 (16NMe), 29.52 (C-6 or C-7 or C-8 or C-9 or C-10 or C-11), 29.49 (C-6 or C-7 or C-8 or C-9 or C-10 or C-11), 29.41 (C-6 or C-7 or C-8 or C-9 or C-10 or C-11), 29.37 (C-6 or C-7 or C-8 or C-9 or C-10 or C-11), 29.33 (C-6 or C-7 or C-8 or C-9 or C-10 or C-11), 29.28 (C-6 or C-7 or C-8 or C-9 or C-10 or C-11), 29.2 (C-13), 29.1 (C-12), 28.3 (C-5); HRMS calcd for $C_{20}H_{36}N_2O_4Na$ [M + Na]$^+$ 391.2567, found 391.2575.

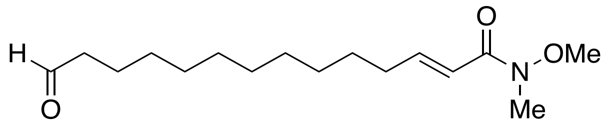

(2*E*)-*N*-Methoxy-*N*-methyl-14-oxotetradec-2-enamide ((*E*)-16k).

R_f= 0.50 (silica gel, hexane/ethyl acetate = 1/1); FT-IR (neat) ν_{max}: 3448, 2924, 2854, 1720, 1666, 1635, 1466, 1419, 1381, 1180, 995 cm^{-1}; ^{1}H NMR (500 MHz, $CDCl_3$): δ 9.77 (t, J = 2.0 Hz, 1H, H-14), 6.98 (dt, J = 15.5, 7.5 Hz, 1H, H-3), 6.39 (d, J = 15.5 Hz, 1H, H-2), 3.70 (s, 3H, OMe), 3.24 (s, 3H, NMe), 2.42 (td, J = 7.5, 2.0 Hz, 2H, H-13), 2.23 (dt, J = 7.5, 7.0 Hz, H-4), 1.63 (tt, J = 7.5, 7.5 Hz, 2H, H-12), 1.46 (tt, J = 7.5, 7.0 Hz, 2H, H-5), 1.39–1.20 (m, 12H, H-6, H-7, H-8, H-9, H-10, H-11); ^{13}C{^{1}H} NMR (125 MHz, $CDCl_3$): 202.9 (C-14), 167.1 (C-1), 148.0 (C-3), 118.6 (C-2), 61.6 (OMe), 43.9 (C-13), 32.5 (C-4), 32.3 (NMe), 29.41 (C-6 or C-7 or C-8 or C-9 or C-10 or C-11), 29.40 (C-6 or C-7 or C-8 or C-9 or C-10 or C-11), 29.33 (C-6 or C-7 or C-8 or C-9 or C-10 or C-11), 29.28 (C-6 or C-7 or C-8 or C-9 or C-10 or C-11), 29.12 (C-6 or C-7 or C-8 or C-9 or C-10 or C-11), 29.10 (C-6 or C-7 or C-8 or C-9 or C-10 or C-11), 28.3 (C-5), 22.0 (C-12); HRMS calcd for $C_{16}H_{29}NO_3Na$ [M + Na]$^+$ 306.2040, found 306.2052.

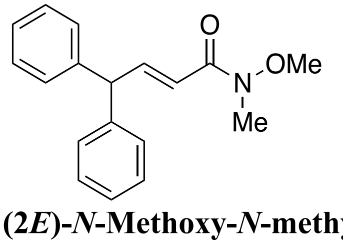

(2*E*)-*N*-Methoxy-*N*-methyl-4,4-diphenylbut-2-enamide ((*E*)-16l).

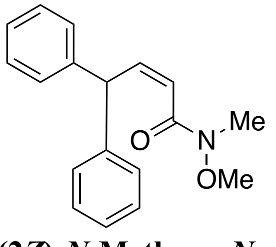

(2*Z*)-*N*-Methoxy-*N*-methyl-4,4-diphenylbut-2-enamide ((*Z*)-16l).

2,2-Diphenylacetaldehyde **15l** (39.3 mg, 0.200 mmol) was employed. The reaction time was 1.5 h. Purification by thin layer chromatography on silica (eluant; hexane/ethyl acetate = 2/1) to afford alkene (*E*)-**16l** (50.2 mg, 89%) as white solid and alkene (*Z*)-**16l** (1.5 mg, 2.7%) as colorless oil.

(E)-**16l**

R_f= 0.42 (silica gel, hexane/ethyl acetate = 2/1); mp: 83.9 °C; ATR-IR ν_{max}: 1656, 1615, 1494, 1452, 1422, 1387, 995, 707 cm^{-1}; ^{1}H NMR (500 MHz, $CDCl_3$): δ 7.43 (dd, J = 15.5, 7.5 Hz, 1H, H-3), 7.35–7.26 (m, 4H, Ar), 7.27–7.16 (m, 6H, Ar), 6.34 (d, J = 15.5 Hz, 1H, H-2), 4.91 (d, J = 7.5 Hz, 1H, H-4), 3.61 (s, 3H, OMe), 3.23 (s, 3H, NMe); ^{13}C{^{1}H} NMR (125 MHz, $CDCl_3$): 166.6 (C-1), 148.3 (C-3), 142.1 (Ar), 128.545 (Ar), 126.7 (Ar), 120.3 (C-2), 61.7 (OMe), 53.6 (C-4), 32.4 (NMe); HRMS calcd for $C_{18}H_{19}NO_2Na$ $[M + Na]^+$ 304.1308, found 304.1308.

(Z)-**16l**

R_f= 0.61 (silica gel, hexane/ethyl acetate = 2/1); ATR-IR ν_{max}: 1653, 1494, 1449, 1393, 1353, 1000, 704 cm^{-1}; ^{1}H NMR (500 MHz, $CDCl_3$): δ 7.34–7.21 (m, 8H, Ar), 7.25–7.16 (m, 2H, Ar), 6.57 (dd, J = 11.0, 10.5 Hz, 1H, H-3), 6.42 (brd, J = 11.0 Hz, 1H, H-2), 6.23 (brd, J = 10.5 Hz, 1H, H-4), 3.65 (s, 3H, OMe), 3.22 (s, 3H, NMe); ^{13}C{^{1}H} NMR (125 MHz, $CDCl_3$): 166.9 (C-1), 147.3 (C-3), 143.4 (Ar), 128.5 (Ar), 128.4 (Ar), 126.4 (Ar), 117.6 (C-2), 61.6 (OMe), 48.0 (C-4), 32.0 (NMe); HRMS calcd for $C_{18}H_{19}NO_2Na$ $[M + Na]^+$ 304.1308, found 304.1311.

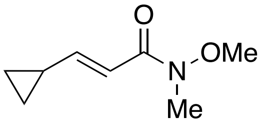

(2*E*)-*N*-Methoxy-*N*-methyl-3-cyclopropylprop-2-enamide ((*E*)-16m).

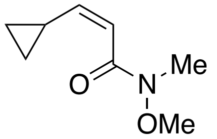

(2*Z*)-*N*-Methoxy-*N*-methyl-3-cyclopropylprop-2-enamide ((*Z*)-16m).

Cyclopropanecarbaldehyde **15m** (14.0 mg, 0.200 mmol) was employed. The reaction time was 1.5 h. Purification by thin layer chromatography on silica (eluant; hexane/ethyl acetate = 1/1) to afford alkene (E)-**16m** (24.1 mg, 78%) as colorless oil and alkene (Z)-**16m** (0.9 mg, 2.9%) as colorless oil.

(E)-**16m**

R_f= 0.31 (silica gel, hexane/ethyl acetate = 2/1); FT-IR (neat) ν_{max}: 3008, 2962, 2939, 1658, 1628, 1466, 1427, 1389, 1180, 1011, 980, 957, 941 cm^{-1}; ^{1}H NMR (500 MHz, $CDCl_3$): δ 6.50 (d, J = 15.5 Hz, 1H, H-2), 6.42 (dd, J = 15.5, 10.0 Hz, 1H, H-3), 3.71 (s, 3H, OMe), 3.23 (s, 3H, NMe), 1.67–1.53 (m, 1H, H-1'), 0.98–0.85 (m, 2H, H-2', H-3'), 0.72–0.57 (m, 2H, H-2', H-3'); ^{13}C{^{1}H} NMR (125 MHz, $CDCl_3$): 167.1 (C-1), 152.6 (C-3), 115.6 (C-2), 61.6 (OMe), 32.3 (NMe), 14.7 (C-1'), 8.4 (C-2', C-3'); HRMS calcd for $C_8H_{13}NO_2Na$ $[M + Na]^+$ 178.0838, found 178.0835.

(Z)-**16m**

R_f= 0.46 (silica gel, hexane/ethyl acetate = 2/1); ATR-IR ν_{max}: 3005, 2937, 1654, 1623, 1463, 1445, 1353, 1179, 1002, 948, 926, 815; ^{1}H NMR (500 MHz, $CDCl_3$): δ 6.19 (d, J = 11.0 Hz, 1H, H-2), 5.38 (dd, J = 11.5, 11.0 Hz, 1H, H-3), 3.69 (s, 3H, OMe), 3.24 (s, 3H, NMe), 3.01–2.85 (m, 1H, H-1'), 1.02–0.90 (m, 2H, H-2', H-3'), 0.58–0.46 (m, 2H, H-2', H-3'); ^{13}C{^{1}H} NMR (125 MHz, $CDCl_3$): 168.0 (C-1), 153.0 (C-3), 115.1 (C-2), 61.5 (OMe), 32.1 (NMe), 11.8 (C-1'), 8.8 (C-2', C-3'); HRMS calcd for $C_8H_{13}NO_2Na$ $[M + Na]^+$ 178.0838, found 178.0831.

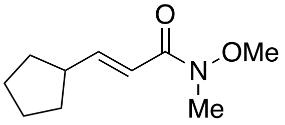

(2*E*)-*N*-Methoxy-*N*-methyl-3-cyclopentylprop-2-enamide ((*E*)-16n).

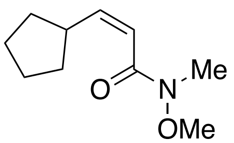

(2*Z*)-*N*-Methoxy-*N*-methyl-3-cyclopentylprop-2-enamide ((*Z*)-16n).

Cyclopentanecarbaldehyde **15n** (19.8 mg, 0.202 mmol) was employed. The reaction time was 1.5 h. Purification by thin layer chromatography on silica (eluant; hexane/ethyl acetate = 2/1) to afford alkene (*E*)-**16n** (27.8 mg, 75%) as colorless oil and alkene (*Z*)-**16n** (0.3 mg, 0.8%) as colorless oil.

(*E*)-**16n**

R_f= 0.38 (silica gel, hexane/ethyl acetate = 2/1); FT-IR (neat) ν_{max}: 2954, 2870, 1658, 1628, 1450, 1419, 1381, 995 cm^{-1}; ^{1}H NMR (500 MHz, $CDCl_3$): δ 6.96 (dd, J = 15.0, 8.0 Hz, 1H, H-3), 6.37 (d, J = 15.0 Hz, 1H, H-2), 3.70 (s, 3H, OMe), 3.24 (s, 3H, NMe), 2.64 (ddddd, J = 8.5, 8.0, 8.0, 8.0, 8.0 Hz, 1H, H-1'), 1.92–1.78 (m, 2H, H-2', H-5'), 1.77–1.52 (m, 4H, H-3', H-4'), 1.52–1.35 (m, 2H, H-2', H-5'); ^{13}C{^{1}H} NMR (125 MHz, $CDCl_3$): 167.3 (C-1), 152.1 (C-3), 116.7 (C-2), 61.6 (OMe), 43.1 (C-1'), 32.6 (C-2', C-5'), 32.4 (NMe), 25.3 (C-3', C-4'); HRMS calcd for $C_{10}H_{17}NO_2Na$ $[M + Na]^+$ 206.1151, found 206.1150.

(*Z*)-**16n**

R_f= 0.58 (silica gel, hexane/ethyl acetate = 2/1); ATR-IR ν_{max}: 2954, 2868, 1660, 1633, 1433, 1347, 1001 cm^{-1}; ^{1}H NMR (500 MHz, $CDCl_3$): δ 6.16 (brd, J = 11.0 Hz, 1H, H-2), 6.01 (dd, J = 11.0, 10.5 Hz, 1H, H-3), 3.72–3.52 (m, 1H, H-1'), 3.68 (s, 3H, OMe), 3.21 (s, 3H, NMe), 1.98–1.86 (m, 2H, H-2', H-5'), 1.77–1.53 (m, 4H, H-3', H-4'), 1.35–1.18 (m, 2H, H-2', H-5'); ^{13}C{^{1}H} NMR (125 MHz, $CDCl_3$): 167.7 (C-1), 152.3 (C-3), 116.5 (C-2), 61.4 (OMe), 39.3 (C-1'), 33.6 (C-2', C-5'), 32.0 (NMe), 25.6 (C-3', C-4'); HRMS calcd for $C_{10}H_{17}NO_2Na$ $[M + Na]^+$ 206.1151, found 206.1148.

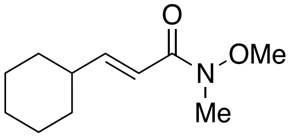

(2*E*)-*N*-Methoxy-*N*-methyl-3-cyclohexylprop-2-enamide ((*E*)-16o).

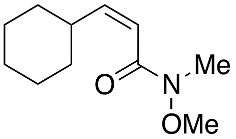

(2*Z*)-*N*-Methoxy-*N*-methyl-3-cyclohexylprop-2-enamide ((*Z*)-16o).

Cyclohexanecarbaldehyde **15o** (22.1 mg, 0.197 mmol) was employed. The reaction time was 1.5 h. Purification by thin layer chromatography on silica (eluant; hexane/ethyl acetate = 2/1) to afford alkene (*E*)-**16o** (34.3 mg, 88%) as colorless oil and alkene (*Z*)-**16o** (trace) as colorless oil.

(*E*)-**16o**

R_f= 0.44 (silica gel, hexane/ethyl acetate = 2/1); FT-IR (neat) ν_{max}: 2924, 2854, 1666, 1635, 1450, 1412, 1381, 1180, 1003, 987, 964 cm^{-1}; ^{1}H NMR (500 MHz, $CDCl_3$): δ 6.93 (dd, J = 15.5, 6.5 Hz, 1H, H-3), 6.34 (d, J = 15.5 Hz, 1H, H-2), 3.70 (s, 3H, OMe), 3.24 (s, 3H, NMe), 2.23–2.12 (m, 1H, H-1'), 1.83–1.70 (m, 4H, H-2', H-3', H-5', H-6'), 1.72–1.63 (m, 1H, H-4'), 1.37–1.23 (m, 2H, H-2', H-6'), 1.25–1.10 (m, 3H, H-3', H-4', H-5'); ^{13}C{^{1}H} NMR (125 MHz, $CDCl_3$): 167.3 (C-1), 152.9 (C-3), 116.1 (C-2), 61.6 (OMe), 40.7 (C-1'), 32.3 (NMe), 31.9 (C-2', C-6'), 25.9 (C-4'), 25.7 (C-3', C-5'); HRMS calcd for $C_{22}H_{38}N_2O_4Na$ $[2M + Na]^+$ 417.2724, found 417.2742.

(*Z*)-**16o**

R_f= 0.70 (silica gel, hexane/ethyl acetate = 2/1); FT-IR (neat) ν_{max}: 2924, 2854, 1658, 1442, 1342, 1003 cm^{-1}; ^{1}H NMR (500 MHz, $CDCl_3$): δ 6.14 (brd, J = 11.0 Hz, 1H, H-2), 5.93 (dd, J = 11.0, 10.5 Hz, 1H, H-3), 3.68 (s, 3H, OMe), 3.35–3.15 (m, 1H, H-1'), 3.22 (s, 3H, NMe), 1.80–1.60 (m, 5H, H-2', H-3', H-4', H-5', H-6'), 1.42–1.28 (m, 2H, H-2', H-6'), 1.24–1.00 (m, 3H, H-4', H-3', H-5'); ^{13}C{^{1}H} NMR (125 MHz, $CDCl_3$): 167.5 (C-1), 153.0 (C-3), 115.9 (C-2), 61.4 (OMe), 37.3 (C-1'), 32.5 (C-2', C-6'),

32.0 (NMe), 26.0 (C-4'), 25.5 (C-3', C-5'); HRMS calcd for $C_{11}H_{19}NO_2Na$ $[M + Na]^+$ 220.1308, found 220.1312.

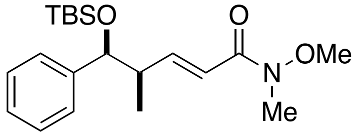

(4*RS*,5*SR*,2*E*)-5-((*tert*-Butyldimethylsilyl)oxy)-*N*-methoxy-*N*,4-dimethyl-5-phenylpent-2-enamide ((*E*)-16p).

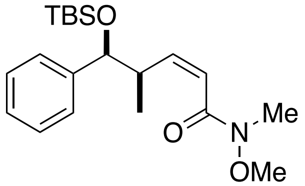

(4*RS*,5*SR*,2*Z*)-5-((*tert*-Butyldimethylsilyl)oxy)-*N*-methoxy-*N*,4-dimethyl-5-phenylpent-2-enamide ((*Z*)-16p).

Aldehyde **15p** (56.1 mg, 0.201 mmol) was employed. The reaction time was 20 min. Purification by thin layer chromatography on silica (eluant; hexane/ethyl acetate = 3/1) to afford alkene (*E*)-**16p** (66.2 mg, 90%) as colorless oil and alkene (*Z*)-**16p** (1.3 mg, 1.8%) as colorless oil.

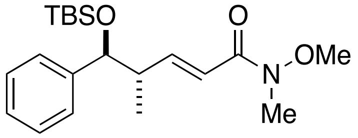

(4*SR*,5*SR*,2*E*)-5-((*tert*-Butyldimethylsilyl)oxy)-*N*-methoxy-*N*,4-dimethyl-5-phenylpent-2-enamide ((*E*)-16q).

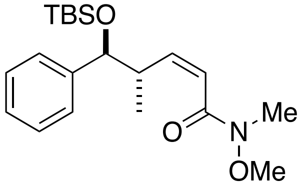

(4*SR*,5*SR*,2*Z*)-5-((*tert*-Butyldimethylsilyl)oxy)-*N*-methoxy-*N*,4-dimethyl-5-phenylpent-2-enamide ((*Z*)-16q).

Aldehyde **15q** (55.6 mg, 0.200 mmol) was employed. The reaction time was 20 min. Purification by thin layer chromatography on silica (eluant; hexane/ethyl acetate = 3/1) to afford alkene (*E*)-**16q** (62.6 mg, 86%) as colorless oil and alkene (*Z*)-**16q** (trace) as colorless oil.

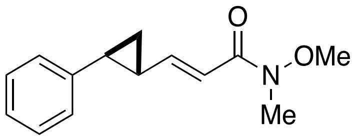

(2*E*)-*N*-Methoxy-*N*-methyl-3-((1*RS*,2*SR*)-2-phenylcyclopropyl)prop-2-enamide ((*E*)-16r).

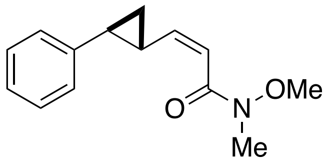

(2*Z*)-*N*-Methoxy-*N*-methyl-3-((1*RS*,2*SR*)-2-phenylcyclopropyl)prop-2-enamide ((*Z*)-16r).

Aldehyde **15r** (28.9 mg (26.2 mg), 0.198 mmol (0.179 mmol[a])) (purity: 90.7%) was employed. The reaction time was 12 h. Purification by thin layer chromatography on silica (eluant; hexane/ethyl acetate = 1/1) to afford alkene (*E*)-**16r** (39.6 mg, 87% (96%[a])) as colorless oil and alkene (*Z*)-**16r** (0.5 mg, 1.1% (1.2%[a])) as colorless oil.

a) Values are based on the purity.

(*E*)-**16r**

R_f = 0.22 (silica gel, hexane/ethyl acetate = 2/1); FT-IR (neat) ν_{max}: 3001, 2939, 1658, 1628, 1496, 1458, 1419, 1373, 1180, 1088, 1003, 964, 926, 748, 702 cm^{-1}; ^{1}H NMR (500 MHz, $CDCl_3$): δ 7.33–7.23 (m, 2H, Ar), 7.22–7.15 (m, 1H, Ar), 7.13–7.04 (m, 2H, Ar), 6.59 (dd, J = 15.0, 10.0 Hz, 1H, H-3), 6.50 (d, J = 15.0 Hz, 1H, H-2), 3.70 (s, 3H, OMe), 3.24 (s, 3H, NMe), 2.18 (ddd, J = 9.0, 6.0, 4.5 Hz, 1H, H-5), 1.93–1.82 (m, 1H, H-4), 1.44 (ddd, J = 8.0, 6.0, 5.5 Hz, 1H, CH_2), 1.32 (ddd, J = 9.0, 6.0, 5.5 Hz, 1H, CH_2); ^{13}C{^{1}H} NMR (125 MHz, $CDCl_3$): 166.9 (C-1), 150.1 (C-3), 141.1 (Ar), 128.4 (Ar), 126.0 (Ar), 125.8 (Ar), 116.4 (C-2), 61.7 (OMe), 32.4 (NMe), 27.4 (C-4), 26.6 (C-5), 17.8 (CH_2); HRMS calcd for $C_{14}H_{17}NO_2Na$ [M + Na]$^+$ 254.1151, found 254.1147.

(*Z*)-**16r**

R_f = 0.44 (silica gel, hexane/ethyl acetate = 2/1); ATR-IR ν_{max}: 1654, 1621, 1497, 1459, 1350, 1179, 1001, 751, 699 cm^{-1}; ^{1}H NMR (500 MHz, $CDCl_3$): δ 7.27–7.21 (m, 2H, Ar), 7.17–7.08 (m, 3H, Ar), 6.25 (brd, J = 11.0 Hz, 1H, H-2), 5.56 (dd, J = 11.0, 11.0 Hz, 1H, H-3), 3.69 (s, 3H, OMe), 3.41–3.27 (m, 1H, H-4), 3.21 (s, 3H, NMe), 2.05 (ddd, J = 9.0, 5.5, 4.5 Hz, 1H, H-5), 1.40 (ddd, J = 7.5, 5.5, 5.0 Hz, 1H, CH_2), 1.17 (ddd, J = 9.0, 5.5, 5.0 Hz, 1H, CH_2); ^{13}C{^{1}H} NMR (125 MHz, $CDCl_3$): 167.7 (C-1), 150.6 (C-3), 141.1 (Ar), 128.3 (Ar), 126.0 (Ar), 125.8 (Ar), 115.5 (C-2), 61.5 (OMe), 32.0 (NMe), 27.1 (C-5), 23.7 (C-4), 18.6 (CH_2); HRMS calcd for $C_{14}H_{17}NO_2Na$ [M + Na]$^+$ 254.1151, found 254.1141.

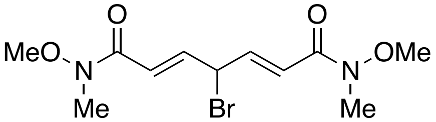

(2*E*,5*E*)-4-Bromo-*N*¹,*N*⁷-dimethoxy-*N*¹,*N*⁷-dimethylhepta-2,5-dienediamide ((*E*,*E*)-16s).

2-Bromopropane-1,3-dial **15s** (56.1 mg, 0.201 mmol) was employed. The reaction time was 1.5 h. Reaction product was not detected from ^{1}H NMR spectra of the crude mixture.

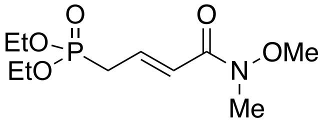

(2*E*)-4-Diethoxyphosphoryl-*N*-methoxy-*N*-methylbut-2-enamide ((*E*)-16t).

Aldehyde **15t** (35.8 mg, 0.199 mmol) was employed. The reaction time was 1.5 h. Reaction product was not detected from ^{1}H NMR spectra of the crude mixture.

Substrate Scope of *α,β*-Unsaturated Aliphatic Aldehyde for *ⁱ*PrMgCl-deprotonating Weinreb Amide-Type Horner–Wadsworth–Emmons Reaction.

Table S17. Substrate Scope of Unsaturated Aliphatic Aldehyde for *ⁱ*PrMgCl-deprotonating Weinreb Amide-Type HWE Reaction.

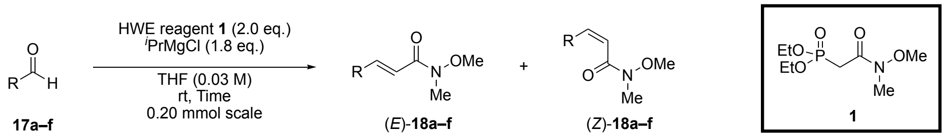

Entry	Substrate (17)	Time	Yield (%) (*E*)-18	Yield (%) (*Z*)-18	*E*/*Z*
1	(a)	1.5 h	56	0.6	99/1
2	(a)	12 h	80	3.6	96/4
3	(b)	1.5 h	22	0	100/0
4	(b)	12 h	52	0	100/0
5	(c)	1.5 h	76	0	100/0
6	(c)	12 h	84	0	100/0
7	(d)	8 h	95	3.7	96/4
8[a]	(d)	21 h	93	3.0	97/3
9	(e)	1.5 h	92	0	100/0
10	(e)	12 h	98	0	100/0
11	(f)	1.5 h	53	2.7	95/5
12	(f)	12 h	86	2.0	98/2

a) THF (0.2 M), 20 mmol scale.

To a solution of phosphate **1** (95.7 mg, 0.400 mmol) in tetrahydrofuran (4.7 mL), a 2.0 M solution of isopropylmagnesium chloride in tetrahydrofuran (0.18 mL, 0.360 mmol) was added at –78 °C. After the reaction mixture was stirred at –78 °C for 30 min, a solution of *Substrate* **17a–f** (0.200 mmol) in tetrahydrofuran (2.0 mL) was added at room temperature, and the reaction mixture was stirred for *Time*. To the reaction mixture, saturated aqueous ammonium chloride was added at 0 °C, and the mixture was extracted with ethyl acetate. The organic layer was dried over sodium sulfate. After filtration of the mixture and concentration of the solvent, the crude mixture was purified by thin layer chromatography on silica to afford alkene **18a–f**.

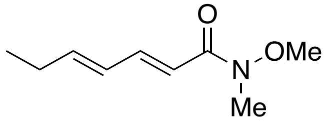

(2*E*,4*E*)-*N*-Methoxy-*N*-methylhepta-2,4-dienamide ((*E*)-18a).

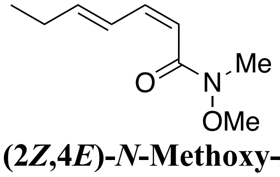

(2*Z*,4*E*)-*N*-Methoxy-*N*-methylhepta-2,4-dienamide ((*Z*)-18a).

(2*E*)-Pent-2-enal **17a** (16.8 mg, 0.200 mmol) was employed. The reaction time was 12 h. Purification by thin layer chromatography on silica (eluant; hexane/ethyl acetate = 2/1) to afford alkene (*E*)-**18a** (26.9 mg, 80%) as colorless oil and alkene (*Z*)-**18a** (1.2 mg, 3.6%) as colorless oil.

(*E*)-**18a**

R_f = 0.39 (silica gel, hexane/ethyl acetate = 2/1); FT-IR (neat) ν_{max}: 2970, 2939, 1658, 1628, 1612, 1381, 1003 cm^{-1}; ^{1}H NMR (500 MHz, $CDCl_3$): δ 7.32 (dd, J = 15.5, 10.0 Hz, 1H, H-3), 6.39 (d, J = 15.5 Hz, 1H, H-2), 6.23 (dd, J = 15.0, 10.0 Hz, 1H, H-4), 6.17 (dt, J = 15.0, 6.0 Hz, 1H, H-5), 3.71 (s, 3H, OMe), 3.25 (s, 3H, NMe), 2.20 (qd, J = 7.0, 6.0 Hz, 2H, H-6), 1.05 (t, J = 7.0 Hz, 3H, H-7); ^{13}C{^{1}H} NMR (125 MHz, $CDCl_3$): 167.4 (C-1), 145.2 (C-5), 143.9 (C-3), 127.8 (C-4), 116.9 (C-2), 61.6 (OMe), 32.4 (NMe), 25.9 (C-6), 12.9 (C-7); HRMS calcd for $C_9H_{15}NO_2Na$ [M + Na]$^+$ 192.0995, found 192.0990.

(*Z*)-**18a**

R_f= 0.58 (silica gel, hexane/ethyl acetate = 2/1); ATR-IR ν_{max}: 2967, 2937, 1653, 1595, 1463, 1437, 1350, 1180, 1001 cm^{-1}; ^{1}H NMR (500 MHz, $CDCl_3$): δ 7.41 (dd, *J* = 15.0, 11.5 Hz, 1H, H-4), 6.51 (dd, *J* = 11.5, 11.5 Hz, 1H, H-3), 6.09 (d, *J* = 11.5 Hz, 1H, H-2), 6.05 (dt, *J* = 15.0, 7.0 Hz, 1H, H-5), 3.69 (s, 3H, OMe), 3.23 (s, 3H, NMe), 2.21 (qd, *J* = 7.5, 7.0 Hz, 2H, H-6), 1.05 (t, *J* = 7.5 Hz, 3H, H-7); ^{13}C{^{1}H} NMR (125 MHz, $CDCl_3$): 167.5 (C-1), 145.9 (C-5), 143.4 (C-3), 126.3 (C-4), 113.7 (C-2), 61.6 (OMe), 32.1 (NMe), 26.0 (C-6), 13.1 (C-7); HRMS calcd for $C_9H_{15}NO_2Na$ $[M + Na]^+$ 192.0995, found 192.0993.

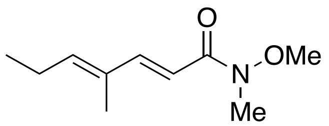

(2*E*,4*E*)-*N*-Methoxy-*N*,4-dimethylhepta-2,4-dienamide ((*E*)-18b).

(2*E*)-2-Methylpent-2-enal **17b** (19.6 mg, 0.200 mmol) was employed. The reaction time was 12 h. Purification by thin layer chromatography on silica (eluant; hexane/ethyl acetate = 2/1) to afford alkene (*E*)-**18b** (19.1 mg, 52%) as colorless oil.

R_f= 0.44 (silica gel, hexane/ethyl acetate = 2/1); ATR-IR ν_{max}: 2967, 2936, 1659, 1609, 1463, 1416, 1379, 1004 cm^{-1}; ^{1}H NMR (500 MHz, $CDCl_3$): δ 7.36 (d, *J* = 15.5 Hz, 1H, H-3), 6.36 (d, *J* = 15.5 Hz, 1H, H-2), 5.90 (t, *J* = 7.5 Hz, 1H, H-5), 3.72 (s, 3H, OMe), 3.27 (s, 3H, NMe), 2.21 (dt, *J* = 7.5, 7.5 Hz, 2H, H-6), 1.81 (s, 3H, 4-Me), 1.03 (t, *J* = 7.5 Hz, 3H, H-7); ^{13}C{^{1}H} NMR (125 MHz, $CDCl_3$): 167.7 (C-1), 148.5 (C-3), 143.1 (C-5), 132.4 (C-4), 112.9 (C-2), 61.6 (OMe), 32.4 (NMe), 22.0 (C-6), 13.5 (C-7), 12.2 (4-Me); HRMS calcd for $C_{10}H_{17}NO_2Na$ $[M + Na]^+$ 206.1151, found 206.1160.

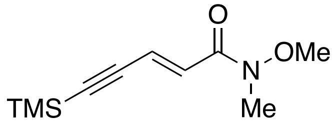

(2*E*)-*N*-Methoxy-*N*-methyl-5-(trimethylsilyl)pent-2-en-4-ynamide ((*E*)-18c).

3-(Trimethylsilyl)-prop-2-ynal **17c** (24.8 mg, 0.196 mmol) was employed. The reaction time was 12 h. Purification by thin layer chromatography on silica (eluant; hexane/ethyl acetate = 2/1) to afford alkene (*E*)-**18c** (34.7 mg, 84%) as colorless oil.

R_f= 0.55 (silica gel, hexane/ethyl acetate = 2/1); FT-IR (neat) ν_{max}: 2962, 1658, 1604, 1381, 1250, 1057, 995, 849, 764 cm^{-1}; ^{1}H NMR (500 MHz, $CDCl_3$): δ 6.86 (d, *J* = 15.5 Hz, 1H, H-3), 6.78 (d, *J* = 15.5Hz, 1H, H-2), 3.72 (s, 3H, OMe), 3.26 (s, 3H, NMe), 0.22 (s, 9H, TMS); ^{13}C{^{1}H} NMR (125 MHz, $CDCl_3$): 165.6 (C-1), 128.7 (C-3), 123.5 (C-2), 103.4 (C-4), 102.3 (C-5), 62.0 (OMe), 32.3 (NMe), –0.4 (TMS); HRMS calcd for $C_{20}H_{34}N_2O_4Si_2Na$ $[2M + Na]^+$ 445.1949, found 445.1969.

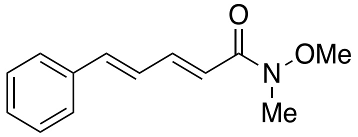

(2*E*,4*E*)-*N*-Methoxy-*N*-methyl-5-phenylpenta-2,4-dienamide ((*E*)-18d).

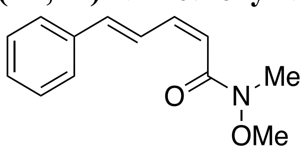

(2*Z*,4*E*)-*N*-Methoxy-*N*-methyl-5-phenylpenta-2,4-dienamide ((*Z*)-18d).

trans-cinnamaldehyde **17d** (26.4 mg, 0.200 mmol) was employed. The reaction time was 8 h. Purification by thin layer chromatography on silica (eluant; hexane/ethyl acetate = 1/1) to afford alkene (*E*)-**18d** (41.1 mg, 95%) as white solid and alkene (*Z*)-**18d** (1.6 mg, 3.7%) as colorless oil.

Large-scale Reaction

To a solution of phosphate **1** (9.57 g, 40.0 mmol) in tetrahydrofuran (80 mL), a 2.0 M solution of isopropylmagnesium chloride in tetrahydrofuran (18.0 mL, 36.0 mmol) was added at –78 °C. After the reaction mixture was stirred at –78 °C for 30 min, a solution of *trans*-cinnamaldehyde **17d** (2.64 g, 20.0 mmol) in tetrahydrofuran (20 mL) was added at room temperature, and the reaction mixture was stirred for 21 h. To the reaction mixture, saturated aqueous ammonium chloride was added at 0 °C, and the mixture was extracted with ethyl acetate. The organic layer was dried over sodium sulfate. After filtration of the mixture and concentration of the solvent, the crude mixture was purified by flash column chromatography (eluant: hexane/ethyl acetate = 4/1 to 1/1) to afford alkene (*E*)-**18d** (4.03 g, 93%) as white solid and alkene (*Z*)-**18d** (131 mg, 3.0%) as white solid.

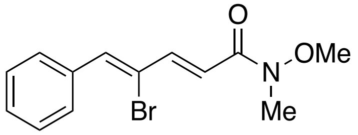

(2*E*,4*Z*)-4-Bromo-*N*-methoxy-*N*-methyl-5-phenylpenta-2,4-dienamide ((*E*)-18e).

(2*Z*)-2-Bromo-3-phenylprop-2-enal **17e** (42.1 mg, 0.199 mmol) was employed. The reaction time was 12 h. Purification by thin layer chromatography on silica (eluant; hexane/ethyl acetate = 2/1) to afford alkene (*E*)-**18e** (58.0 mg, 98%) as white solid.

R_f= 0.43 (silica gel, hexane/ethyl acetate = 2/1); mp: 75.8 °C; ATR-IR ν_{max}: 2967, 1646, 1606, 1592, 1464, 1446, 1414, 1380, 1146, 1002, 968, 853, 755, 694, 522 cm^{-1}; ^{1}H NMR (500 MHz, $CDCl_3$): δ 7.82–7.73 (m, 2H, Ar), 7.51 (d, J = 14.5 Hz, 1H, H-3), 7.46–7.33 (m, 3H, Ar), 7.29 (s, 1H, H-5), 6.94 (d, J = 14.5 Hz, 1H, H-2), 3.78 (s, 3H, OMe), 3.31 (s, 3H, NMe); ^{13}C{^{1}H} NMR (125 MHz, $CDCl_3$): 166.3 (C-1), 143.7 (C-3), 138.4 (C-5), 134.9 (Ar), 129.9 (Ar), 129.2 (Ar), 128.3 (Ar), 121.1 (C-4), 120.9 (C-2), 62.0 (OMe), 32.5 (NMe); HRMS calcd for $C_{13}H_{14}BrNO_2Na$ $[M + Na]^+$ 318.0100, found 318.0113.

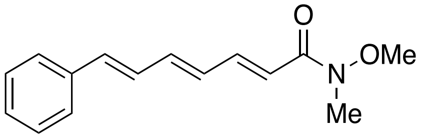

(2*E*,4*E*,6*E*)-*N*-Methoxy-*N*-methyl-7-phenylhepta-2,4,6-trienamide ((*E*)-18f).

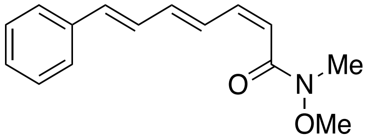

(2*Z*,4*E*,6*E*)-*N*-Methoxy-*N*-methyl-7-phenylhepta-2,4,6-trienamide ((*Z*)-18f).

Aldehyde **17f** (31.8 mg, 0.201 mmol) was employed. The reaction time was 12 h. Purification by thin layer chromatography on silica (eluant; hexane/ethyl acetate = 1/1) to afford alkene (*E*)-**18f** (42.0 mg, 86%) as yellow solid and alkene (*Z*)-**18f** (1.0 mg, 2.0%) as yellow oil.

(*E*)-**18f**

R_f= 0.27 (silica gel, hexane/ethyl acetate = 2/1); mp: 79.9 °C; FT-IR (KBr) ν_{max}: 3433, 2931, 1635, 1589, 1458, 1419, 1381, 1173, 1018, 995, 949, 748, 687 cm^{-1}; ^{1}H NMR (500 MHz, C_6D_6): δ 7.84 (dd, J = 15.0, 11.5 Hz, 1H, H-3), 7.35–6.95 (m, 5H, Ar), 6.64 (dd, J = 15.5, 11.5 Hz, 1H, H-6), 6.63 (d, J = 15.0 Hz, 1H, H-2), 6.35 (dd, J = 15.0, 11.5 Hz, 1H, H-5), 6.34 (d, J = 15.5 Hz, 1H, H-7), 6.25 (dd, J = 15.0, 11.5 Hz, 1H, H-4), 3.10 (s, 3H, OMe), 3.00 (s, 3H, NMe); ^{13}C{^{1}H} NMR (125 MHz, C_6D_6): 167.2 (C-1), 143.4 (C-3), 140.3 (C-5), 137.3 (Ar), 136.1 (C-7), 131.3 (C-4), 128.9 (Ar), 128.5 (C-6), 128.3 (Ar), 127.0 (Ar), 119.7 (C-2), 61.1 (OMe), 32.3 (NMe); HRMS calcd for $C_{30}H_{34}N_2O_4Na$ $[2M + Na]^+$ 509.2411, found 509.2429.

(*Z*)-**18f**

R_f= 0.47 (silica gel, hexane/ethyl acetate = 2/1); FT-IR (neat) ν_{max}: 2970, 2931, 1643, 1597, 1566, 1435, 1350, 1180, 1095, 1003, 802, 756, 694 cm^{-1}; ^{1}H NMR (500 MHz, C_6D_6): δ 8.42 (dd, J = 15.0, 11.5 Hz,

1H, H-4), 7.15–7.05 (m, 4H, Ar), 7.05–6.98 (m, 1H, Ar), 6.75 (dd, J = 15.5, 11.0 Hz, 1H, H-6), 6.49 (dd, J = 11.5, 11.5 Hz, 1H, H-3), 6,43 (d, J = 15.5 Hz, 1H, H-7), 6.38 (dd, J = 15.0, 11.0 Hz, 1H, H-5), 6.26 (d, J = 11.5 Hz, 1H, H-2), 3.06 (s, 3H, OMe), 2.96 (s, 3H, NMe); $^{13}C\{^{1}H\}$ NMR (125 MHz, C_6D_6): 167.4 (C-1), 142.8 (C-3), 141.0 (C-5), 137.3 (Ar), 135.9 (C-7), 130.7 (C-4), 129.3 (C-6), 128.8 (Ar), 128.1 (Ar), 127.2 (Ar). 115.8 (C-2), 61.0 (OMe), 32.0 (NMe); HRMS calcd for $C_{30}H_{34}N_2O_4Na$ [2M + Na]$^+$ 509.2411, found 509.2435.

Substrate Scope of Aromatic Aldehyde for ^{i}PrMgCl-deprotonating Weinreb Amide-Type Horner–Wadsworth–Emmons Reaction.

Table S18. Substrate Scope of Aromatic Aldehydes in the ^{i}PrMgCl-deprotonating Weinreb Amide-Type HWE Reaction.

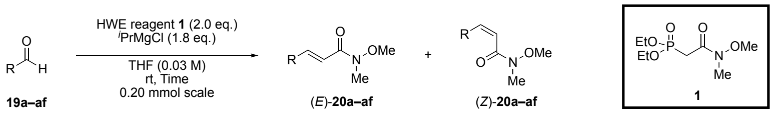

Entry	Substrate (19)		Time	Yield (%) (E)-20	Yield (%) (Z)-20	E/Z
1	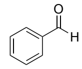	(a)	2 h	87	0	100/0
2		(a)	12 h	93	0	100/0
3	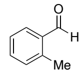	(b)	2 h	92	0	100/0
4		(b)	12 h	95	0	100/0
5	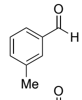	(c)	2 h	95	0	100/0
6		(c)	12 h	97	0	100/0
7	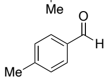	(d)	2 h	90	0	100/0
8		(d)	12 h	98	0	100/0
9	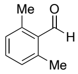	(e)	2 h	9.5	0	100/0
10		(e)	12 h	30	0	100/0
11		(e)	48 h	84	0	100/0
12[a]		(e)	6 h	86	0	100/0
13	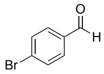	(f)	2 h	97	0	100/0
14		(f)	12 h	100	0	100/0
15	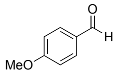	(g)	2 h	74	0	100/0
16		(g)	12 h	95	0	100/0
17	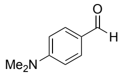	(h)	60 h	74	10	88/12
18[a]		(h)	6 h	93	0	100/0
19	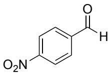	(i)	2 h	87	0	100/0
20		(i)	12 h	93	0	100/0
21	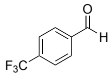	(j)	2 h	94	0	100/0
22		(j)	12 h	100	0	100/0
23	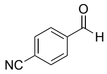	(k)	2 h	91	0	100/0
24		(k)	12 h	98	0	100/0
25	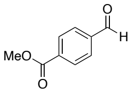	(l)	2 h	96	0	100/0
26		(l)	12 h	97	0	100/0
27	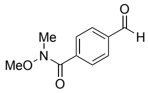	(m)	12 h	92	0	100/0
28	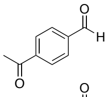	(n)	2 h	96	0	100/0
29		(n)	12 h	98	0	100/0
30	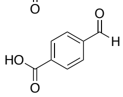	(o)	12 h	0	0	–
31[b]		(o)	60 h	0	0	–
32[c]		(o)	6 h	94	0	100/0
33	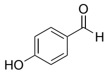	(p)	12 h	trace	0	100/0
34[b]		(p)	60 h	27	0	100/0
35[d]		(p)	12 h	27	0	100/0
36[e]		(p)	12 h	43	0	100/0
37[e]		(p)	60 h	89	0	100/0
38[c]		(p)	24 h	93	0	100/0
39	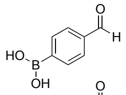	(q)	2 h	0	0	100/0
40		(q)	12 h	0	0	100/0
41[c,f]		(q)	4 h	97[f]	0	100/0
42	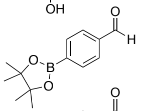	(r)	2 h	87	0	100/0
43		(r)	12 h	86	0	100/0
44	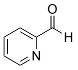	(s)	2 h	12	0	100/0
45		(s)	12 h	16	0	100/0
46[c]		(s)	12 h	7.3	0	100/0
47	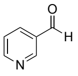	(t)	2 h	64	0	100/0
48		(t)	12 h	86	0	100/0
49	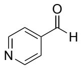	(u)	2 h	90	0	100/0
50		(u)	12 h	92	0	100/0
51	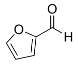	(v)	2 h	93	0	100/0
52		(v)	12 h	91	0	100/0
53	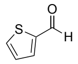	(w)	2 h	89	0	100/0
54		(w)	12 h	95	0	100/0
55	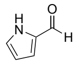	(x)	12 h	6.9	0	100/0
56		(x)	60 h	23	0	100/0
57[g]		(x)	60 h	28	0	100/0
58[b]		(x)	60 h	44	0	100/0
59[c]		(x)	24 h	46	0	100/0
60	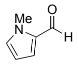	(y)	2 h	11	0	100/0
61		(y)	12 h	38	0	100/0
62		(y)	60 h	66	0.8	99/1
63[a]		(y)	6 h	93	trace	>99/1
64	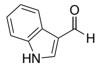	(z)	2 h	2.8	0	100/0
65		(z)	12 h	8.9	0	100/0
66[b]		(z)	60 h	42	0	100/0
67[c]		(z)	24 h	88	0	100/0
68	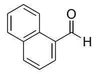	(aa)	2 h	100	0	100/0
69		(aa)	12 h	95	0	100/0
70	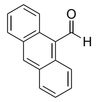	(ab)	2 h	29	0	100/0
71		(ab)	12 h	68	0	100/0
72		(ab)	60 h	88	0	100/0
73[a]		(ab)	6 h	98	0	100/0
74	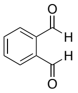	(ac)	12 h	77[h]	0[h]	100/0
75[b]		(ac)	12 h	29[h]	0[h]	100/0
76[i]		(ac)	2 h	53[h]	0[h]	100/0
77	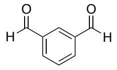	(ad)	12 h	63[j]	0[j]	100/0
78[b]		(ad)	12 h	45[j]	0[j]	100/0
79[i]		(ad)	6 h	97[j]	0[j]	100/0
80	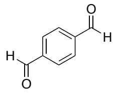	(ae)	12 h	57[k]	0[k]	100/0
81[b]		(ae)	12 h	37[k]	0[k]	100/0
82[i]		(ae)	2 h	96[k]	0[k]	100/0
83	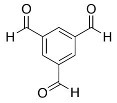	(af)	12 h	9.1[l]	0[l]	100/0
84[b]		(af)	24 h	55[l]	0[l]	100/0
85[m]		(af)	24 h	14[l]	0[l]	100/0
86[n]		(af)	4 h	80[l]	0[l]	100/0

a) reflux.
b) **1** (4.0 equiv), ^{i}PrMgCl (3.6 eq.).
c) **1** (3.0 equiv), ^{i}PrMgCl (2.7 eq.), reflux.
d) 50 °C.
e) **1** (3.0 equiv), ^{i}PrMgCl (2.7 eq.), 50 °C.
f) 0.80 mmol scale.
The yield of (*E*)-**20q** was calculated from ^{1}H NMR spectra of the crude mixture.
Isolated yield of (*E*)-**20q** was 61%.
g) **1** (3.0 equiv), ^{i}PrMgCl (2.7 eq.).
h) The yield of (*E*)-**20** is that of (*E*,*E*)-**20ac** that two formyl groups were reacted.
(*E*)-**20ac** that one of two formyl groups was reacted was also given in a 23% yield (Entry 74).
(*E*)-**20ac** that one of two formyl groups was reacted was not detected (Entry 75, 76).
(*Z*)-**20** is (*Z*,*Z*)-**20ac** and (*E*,*Z*)-**20ac**, and they were not detected.
i) **1** (4.0 equiv), ^{i}PrMgCl (3.6 eq.), reflux.
j) The yield of (*E*)-**20** is that of (*E*,*E*)-**20ad** that two formyl groups were reacted.
(*E*)-**20ad** that one of two formyl groups was reacted was also given in a 37% yield (Entry 77).
(*E*)-**20ad** that one of two formyl groups was reacted was also given in a 4.5% yield (Entry 78).
(*E*)-**20ad** that one of two formyl groups was reacted was not detected (Entry 79).
(*Z*)-**20** is (*Z*,*Z*)-**20ad** and (*E*,*Z*)-**20ad**, and they were not detected.
k) The yield of (*E*)-**20** is that of (*E*,*E*)-**20ae** that two formyl groups were reacted.
(*E*)-**20ae** that one of two formyl groups was reacted was also given in a 38% yield (Entry 80).
(*E*)-**20ae** that one of two formyl groups was reacted was also given in a 2.3% yield (Entry 81).
(*E*)-**20ae** that one of two formyl groups was reacted was not detected (Entry 82).
(*Z*)-**20** is (*Z*,*Z*)-**20ae** and (*E*,*Z*)-**20ae**, and they were not detected.
l) The yield of (*E*)-**20** is that of (*E*,*E*,*E*)-**20af** that three formyl groups were reacted.
(*E*)-**20af** that one of three formyl groups was reacted also was also given in a 32% yield (Entry 83).
(*E*)-**20af** that one of three formyl groups was reacted also was also given in a 24% yield (Entry 84).
(*E*)-**20af** that one of three formyl groups was reacted also was also given in a 69% yield (Entry 85).
(*E*)-**20af** that one of three formyl groups was reacted also was not detected (Entry 86).
(*E*,*E*)-**20af** that two of three formyl grops were reacted was also given in a 59% yield (Entry 83).
(*E*,*E*)-**20af** that two of three formyl grops were reacted was also given in a 5.5% yield (Entry 84).
(*E*,*E*)-**20af** that two of three formyl grops were reacted was not detected (Entry 85, 86).
(*Z*)-**20** is (*Z*,*Z*,*Z*)-**20af**, (*E*,*Z*,*Z*)-**20af** and (*E*,*E*,*Z*)-**20af**, and they were not detected.
m) **1** (6.0 equiv), ^{i}PrMgCl (5.4 eq.).
n) **1** (6.0 equiv), ^{i}PrMgCl (5.4 eq.), reflux.

To a solution of phosphate **1** (2.0 equiv: 95.7 mg, 0.400 mmol, 3.0 equiv: 144 mg, 0.600 mmol, 4.0 equiv: 191 mg, 0.800 mmol, 6.0 equiv: 287 mg, 1.20 mmol) in tetrahydrofuran (4.7 mL), a 2.0 M solution of isopropylmagnesium chloride in tetrahydrofuran (1.8 equiv: 0.18 mL, 0.360 mmol, 2.7 equiv: 0.27 mL, 0.540 mmol, 3.6 equiv: 0.36 mL, 0.720 mmol, 5.4 equiv: 0.54 mL, 1.08 mmol) was added at –78 °C. After the reaction mixture was stirred at –78 °C for 30 min, a solution of *Substrate* **19a–af** (0.200 mmol) in tetrahydrofuran (2.0 mL) was added at *Temperature* (room temperature, 50 °C or reflux), and the reaction mixture was stirred for *Time*. To the reaction mixture, saturated aqueous ammonium chloride was added at 0 °C, and the mixture was extracted with ethyl acetate. The organic layer was dried over sodium sulfate. After filtration of the mixture and concentration of the solvent, the crude mixture was purified by thin layer chromatography on silica to afford alkene **20a–af**.

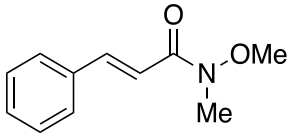

(2*E*)-*N*-Methoxy-*N*-methyl-3-phenylprop-2-enamide ((*E*)-5b, (*E*)-20a).

Benzaldehyde **19a** (21.0 mg, 0.198 mmol) was employed. The reaction time was 12 h. Purification by thin layer chromatography on silica (eluant; hexane/ethyl acetate = 1/1) to afford alkene (*E*)-**20a** (35.2 mg, 93%) as white solid.

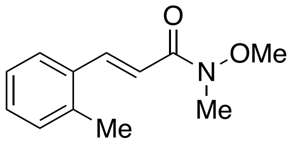

(2*E*)-*N*-Methoxy-*N*-methyl-3-(2-methylphenyl)-prop-2-enamide ((*E*)-20b).

2-Methylbenzaldehyde **19b** (24.2 mg, 0.201 mmol) was employed. The reaction time was 12 h. Purification by thin layer chromatography on silica (eluant; hexane/ethyl acetate = 2/1) to afford alkene (*E*)-**20b** (39.2 mg, 95%) as colorless oil.

R_f= 0.32 (silica gel, hexane/ethyl acetate = 2/1); FT-IR (neat) ν_{max}: 2962, 2939, 1658, 1620, 1481, 1458, 1412, 1381, 1003, 764 cm^{-1}; ^{1}H NMR (500 MHz, $CDCl_3$): δ 8.02 (d, J = 15.5 Hz, 1H, H-3), 7.65–7.58 (m, 1H, Ar), 7.32–7.17 (m, 3H, Ar), 6.95 (d, J = 15.5 Hz, 1H, H-2), 3.77 (s, 3H, OMe), 3.32 (s, 3H, NMe), 2.45 (s, 3H, ArMe); ^{13}C{^{1}H} NMR (125 MHz, $CDCl_3$): 166.9 (C-1), 141.1 (C-3), 137.6 (Ar), 134.2 (Ar), 130.7 (Ar), 129.5 (Ar), 126.3 (Ar), 126.1 (Ar), 116.9 (C-2), 61.8 (OMe), 32.4 (NMe), 19.8 (ArMe); HRMS calcd for $C_{24}H_{30}N_2O_4Na$ [2M + Na]$^+$ 433.2098, found 433.2100.

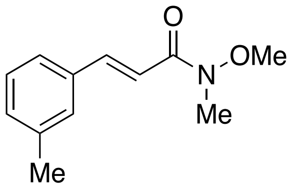

(2*E*)-*N*-Methoxy-*N*-methyl-3-(3-methylphenyl)-prop-2-enamide ((*E*)-20c).

3-Methylbenzaldehyde **19c** (23.9 mg, 0.199 mmol) was employed. The reaction time was 12 h. Purification by thin layer chromatography on silica (eluant; hexane/ethyl acetate = 1/1) to afford alkene (*E*)-**20c** (39.6 mg, 97%) as colorless oil.

R_f= 0.52 (silica gel, hexane/ethyl acetate = 1/1); ATR-IR ν_{max}: 2965, 2936, 1660, 1622, 1485, 1462, 1423, 1381, 1177, 1100, 997, 787 cm^{-1}; ^{1}H NMR (500 MHz, $CDCl_3$): δ 7.71 (d, J = 16.0 Hz, 1H, H-3), 7.41–7.35 (m, 2H, Ar), 7.31–7.23 (m, 1H, Ar), 7.22–7.15 (m, 1H, Ar), 7.02 (d, J = 16.0 Hz, 1H, H-2), 3.77 (s, 3H, OMe), 3.31 (s, 3H, NMe), 2.38 (s, 3H, ArMe); ^{13}C{^{1}H} NMR (125 MHz, $CDCl_3$): 167.0 (C-1), 143.6 (C-3), 138.4 (Ar), 135.1 (Ar), 130.6 (Ar), 128.64 (Ar), 128.61 (Ar), 125.2 (Ar), 115.5 (C-2), 61.9 (OMe), 32.5 (NMe), 21.3 (ArMe); HRMS calcd for $C_{12}H_{15}NO_2Na$ [M + Na]$^+$ 228.0995, found 228.0997.

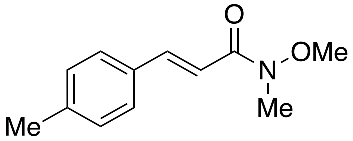

(2*E*)-*N*-Methoxy-*N*-methyl-3-(4-methylphenyl)-prop-2-enamide ((*E*)-20d).
4-Methylbenzaldehyde **19d** (23.9 mg, 0.199 mmol) was employed. The reaction time was 12 h. Purification by thin layer chromatography on silica (eluant; hexane/ethyl acetate = 2/1) to afford alkene (*E*)-**20d** (39.9 mg, 98%) as white solid.
R_f = 0.31 (silica gel, hexane/ethyl acetate = 2/1); mp: 50.2 °C; FT-IR (KBr) ν_{max}: 3464, 3433, 2970, 2939, 1651, 1612, 1381, 1180, 987, 810, 494 cm^{-1}; ^{1}H NMR (500 MHz, $CDCl_3$): δ 7.71 (d, J = 15.5 Hz, 1H, H-3), 7.51–7.44 (m, 2H, Ar), 7.23–7.15 (m, 2H, Ar), 7.00 (d, J = 15.5 Hz, 1H, H-2), 3.77 (s, 3H, OMe), 3.31 (s, 3H, NMe), 2.38 (s, 3H, ArMe); ^{13}C{^{1}H} NMR (125 MHz, $CDCl_3$): 167.1 (C-1), 143.4 (C-3), 140.1 (Ar), 132.4 (Ar), 129.5 (Ar), 128.0 (Ar), 114.6 (C-2), 61.8 (OMe), 32.5 (NMe), 21.4 (ArMe); HRMS calcd for $C_{24}H_{30}N_2O_4Na$ [2M + Na]$^+$ 433.2098, found 433.2094.

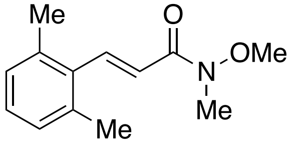

(2*E*)-*N*-Methoxy-*N*-methyl-3-(2,6-dimethylphenyl)-prop-2-enamide ((*E*)-20e).
2,6-Dimethylbenzaldehyde **19e** (26.8 mg, 0.200 mmol) was employed. The reaction temperature was reflux and the reaction time was 6 h. Purification by thin layer chromatography on silica (eluant; hexane/ethyl acetate = 1/1) to afford alkene (*E*)-**20e** (37.6 mg, 86%) as colorless oil.
R_f = 0.60 (silica gel, hexane/ethyl acetate = 1/1); FT-IR (neat) ν_{max}: 2962, 2939, 1658, 1628, 1466, 1412, 1381, 1180, 995, 771 cm^{-1}; ^{1}H NMR (500 MHz, $CDCl_3$): δ 7.85 (d, J = 16.0 Hz, 1H, H-3), 7.17–7.04 (m, 3H, Ar), 6.66 (d, J = 16.0 Hz, 1H, H-2), 3.72 (s, 3H, OMe), 3.32 (s, 3H, NMe), 2.36 (s, 6H, ArMe); ^{13}C{^{1}H} NMR (125 MHz, $CDCl_3$): 166.7 (C-1), 141.8 (C-3), 136.5 (Ar), 135.0 (Ar), 128.0 (Ar), 127.9 (Ar), 121.7 (C-2), 61.9 (OMe), 32.4 (NMe), 21.0 (ArMe); HRMS calcd for $C_{26}H_{34}N_2O_4Na$ [2M + Na]$^+$ 461.2411, found 461.2431.

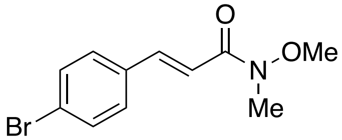

(2*E*)-*N*-Methoxy-*N*-methyl-3-(4-bromophenyl)-prop-2-enamide ((*E*)-20f).
4-Bromobenzaldehyde **19f** (37.1 mg, 0.201 mmol) was employed. The reaction time was 12 h. Purification by thin layer chromatography on silica (eluant; hexane/ethyl acetate = 1/1) to afford alkene (*E*)-**20f** (54.1 mg, 100%) as colorless oil.
R_f = 0.45 (silica gel, hexane/ethyl acetate = 1/1); ATR-IR ν_{max}: 1661, 1623, 1489, 1417, 1382, 1072, 1010, 818 cm^{-1}; ^{1}H NMR (500 MHz, $CDCl_3$): δ 7.66 (d, J = 15.5 Hz, 1H, H-3), 7.55–7.48 (m, 2H, Ar), 7.47–7.39 (m, 2H, Ar), 7.02 (d, J = 15.5 Hz, 1H, H-2), 3.77 (s, 3H, OMe), 3.31 (s, 3H, NMe); ^{13}C{^{1}H} NMR (125 MHz, $CDCl_3$): 166.6 (C-1), 142.0 (C-3), 134.0 (Ar), 132.0 (Ar), 129.4 (Ar), 123.9 (Ar), 116.4 (C-2), 61.9 (OMe), 32.5 (NMe); HRMS calcd for $C_{11}H_{12}BrNO_2Na$ [M + Na]$^+$ 291.9944, found 291.9932.

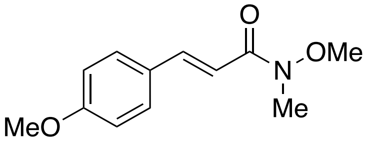

(2*E*)-*N*-Methoxy-*N*-methyl-3-(4-methoxyphenyl)-prop-2-enamide ((*E*)-20g).
4-Methoxybenzaldehyde **19g** (27.5 mg, 0.202 mmol) was employed. The reaction time was 12 h.

Purification by thin layer chromatography on silica (eluant; hexane/ethyl acetate = 1/1) to afford alkene (*E*)-**20g** (42.6 mg, 95%) as colorless oil.

R_f= 0.29 (silica gel, hexane/ethyl acetate = 2/1); FT-IR (neat) ν_{max}: 2962, 2939, 1651, 1604, 1512, 1381, 1250, 1173, 1034, 1003, 825 cm^{-1}; ^{1}H NMR (500 MHz, $CDCl_3$): δ 7.70 (d, J = 15.5 Hz, 1H, H-3), 7.57–7.50 (m, 2H, Ar), 6.95–6.88 (m, 2H, Ar), 6.92 (d, J = 15.5 Hz, 1H, H-2), 3.84 (s, 3H, ArOMe), 3.77 (s, 3H, NOMe), 3.31 (s, 3H, NMe); $^{13}C\{^1H\}$ NMR (125 MHz, $CDCl_3$): 167.3 (C-1), 160.9 (Ar), 143.0 (C-3), 129.6 (Ar), 127.8 (Ar), 114.1 (C-2), 113.2 (Ar), 61.8 (OMe), 55.3 (ArOMe), 32.5 (NMe); HRMS calcd for $C_{24}H_{30}N_2O_6Na$ [2M + Na]$^+$ 465.1996, found 465.1985.

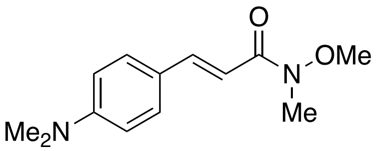

(2*E*)-*N*-Methoxy-*N*-methyl-3-(4-dimethylaminophenyl)-prop-2-enamide ((*E*)-20h).

4-Dimethylaminobenzaldehyde **19h** (29.9 mg, 0.200 mmol) was employed. The reaction temperature was reflux and the reaction time was 6 h. Purification by thin layer chromatography on silica (eluant; hexane/ethyl acetate = 1/2) to afford alkene (*E*)-**20h** (43.9 mg, 93%) as yellow oil.

R_f= 0.34 (silica gel, hexane/ethyl acetate = 1/1); FT-IR (neat) ν_{max}: 2931, 2900, 1651, 1597, 1527, 1435, 1412, 1365, 1180, 1003, 818 cm^{-1}; ^{1}H NMR (500 MHz, $CDCl_3$): δ 7.68 (d, J = 16.0 Hz, 1H, H-3), 7.52–7.44 (m, 2H, Ar), 6.83 (d, J = 16.0 Hz, 1H, H-2), 6.71–6.64 (m, 2H, Ar), 3.76 (s, 3H, OMe), 3.30 (s, 3H, NMe), 3.02 (s, 6H, NMe_2); $^{13}C\{^1H\}$ NMR (125 MHz, $CDCl_3$): 168.0 (C-1), 151.4 (Ar), 143.9 (C-3), 129.6 (Ar), 123.0 (Ar), 111.7 (C-2), 110.2 (Ar), 61.7 (OMe), 40.1 (NMe_2), 32.5 (NMe); HRMS calcd for $C_{26}H_{36}N_4O_4Na$ [2M + Na]$^+$ 491.2629, found 491.2642.

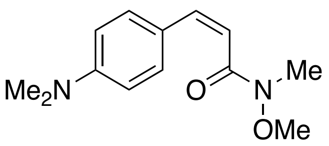

(2*Z*)-*N*-Methoxy-*N*-methyl-3-(4-dimethylaminophenyl)-prop-2-enamide ((*Z*)-20h).

yellow solid

R_f= 0.41 (silica gel, hexane/ethyl acetate = 1/1); mp: 45.5 °C; ATR-IR ν_{max}: 2969, 1647, 1620, 1585, 1530, 1447, 1365, 1336, 1215, 1187, 1093, 992, 830 cm^{-1}; ^{1}H NMR (500 MHz, $CDCl_3$): δ 7.68–7.45 (m, 2H, Ar), 6.73–6.58 (m, 3H, H-3, Ar), 6.13–5.93 (m, 1H, H-2), 3.69 (s, 3H, OMe), 3.25 (s, 3H, NMe), 2.98 (s, 6H, NMe_2); $^{13}C\{^1H\}$ NMR (125 MHz, $CDCl_3$): 167.6 (C-1), 150.6 (Ar), 139.2 (C-3), 131.3 (Ar), 123.1 (Ar), 114.8 (C-2), 111.4 (Ar), 61.4 (OMe), 40.1 (NMe_2), 32.5 (NMe); HRMS calcd for $C_{13}H_{18}N_2O_2Na$ [M + Na]$^+$ 257.1260, found 257.1261.

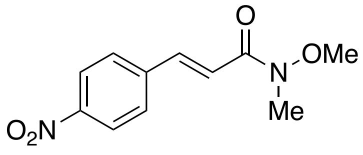

(2*E*)-*N*-Methoxy-*N*-methyl-3-(4-nitrophenyl)-prop-2-enamide ((*E*)-20i).

4-Nitrobenzaldehyde **19i** (30.4 mg, 0.201 mmol) was employed. The reaction time was 12 h. The crude mixture was purified by thin layer chromatography on silica (eluant; hexane/ethyl acetate = 2/3) to afford the mixture of alkene (*E*)-**20i** and phosphate **1**. The mixture was purified by thin layer chromatography on silica (eluant; chloroform/methanol = 40/1) to afford alkene (*E*)-**20i** (44.2 mg, 93%) as yellow solid.

R_f= 0.34 (silica gel, hexane/ethyl acetate = 1/1); mp: 154.0 °C; FT-IR (KBr) ν_{max}: 3471, 3024, 2939, 1651, 1620, 1589, 1520, 1342, 1173, 995, 849, 748 cm^{-1}; ^{1}H NMR (500 MHz, $CDCl_3$): δ 8.28–8.23 (m, 2H, Ar), 7.76 (d, J = 16.0 Hz, 1H, H-3), 7.74–7.69 (m, 2H, Ar), 7.17 (d, J = 16.0 Hz, 1H, H-2), 3.80 (s,

3H, OMe), 3.34 (s, 3H, NMe); $^{13}C\{^1H\}$ NMR (125 MHz, $CDCl_3$): 165.8 (C-1), 148.2 (Ar), 141.3 (Ar), 140.5 (C-3), 128.6 (Ar), 124.1 (Ar), 120.0 (C-2), 62.1 (OMe), 32.5 (NMe); HRMS calcd for $C_{11}H_{12}N_2O_4Na$ $[M + Na]^+$ 259.0689, found 259.0683.

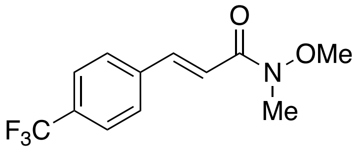

(2*E*)-*N*-Methoxy-*N*-methyl-3-(4-trifluoromethylphenyl)-prop-2-enamide ((*E*)-20j).

4-(Trifluoromethyl)benzaldehyde **19j** (34.5 mg, 0.198 mmol) was employed. The reaction time was 12 h. Purification by thin layer chromatography on silica (eluant; hexane/ethyl acetate = 1/1) to afford alkene (*E*)-**20j** (51.5 mg, 100%) as white solid.

R_f = 0.33 (silica gel, hexane/ethyl acetate = 2/1); mp: 55.8 °C; FT-IR (KBr) ν_{max}: 3464, 2939, 1658, 1620, 1327, 1165, 1126, 1065, 995, 833 cm^{-1}; ^1H NMR (500 MHz, $CDCl_3$): δ 7.74 (d, J = 16.0 Hz, 1H, H-3), 7.67 (d, J = 8.5 Hz, 2H, Ar), 7.64 (d, J = 8.5 Hz, 2H, Ar), 7.11 (d, J = 16.0 Hz, 1H, H-2), 3.78 (s, 3H, OMe), 3.33 (s, 3H, NMe); $^{13}C\{^1H\}$ NMR (125 MHz, $CDCl_3$): 166.2 (C-1), 141.6 (C-3), 138.6 (Ar), 131.3 (q, $^2J_{CF}$ = 32.3 Hz, Ar), 128.1 (Ar), 125.7 (q, $^3J_{CF}$ = 3.6 Hz, Ar), 123.9 (q, $^1J_{CF}$ = 270.6 Hz, CF_3), 118.3 (C-2), 62.0 (OMe), 32.5 (NMe); $^{19}F\{^1H, ^{13}C\}$ NMR (470 MHz, $CDCl_3$): –63.2 (CF_3); HRMS calcd for $C_{12}H_{12}F_3NO_2Na$ $[M + Na]^+$ 282.0712, found 282.0721.

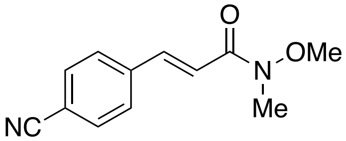

(2*E*)-*N*-Methoxy-*N*-methyl-3-(4-cyanophenyl)-prop-2-enamide ((*E*)-20k).

4-Cyanobenzaldehyde **19k** (26.3 mg, 0.201 mmol) was employed. The reaction time was 12 h. Purification by thin layer chromatography on silica (eluant; hexane/ethyl acetate = 1/2) to afford alkene (*E*)-**20k** (42.4 mg, 98%) as white solid.

R_f = 0.33 (silica gel, hexane/ethyl acetate = 1/1); mp: 151.6 °C; ATR-IR ν_{max}: 2978, 2224, 1655, 1618, 1470, 1412, 1383, 1008, 831, 548 cm^{-1}; ^1H NMR (500 MHz, $CDCl_3$): δ 7.72–7.61 (m, 4H, Ar), 7.71 (d, J = 15.5 Hz, 1H, H-3), 7.12 (d, J = 15.5 Hz, 1H, H-2), 3.78 (s, 3H, OMe), 3.33 (s, 3H, NMe); $^{13}C\{^1H\}$ NMR (125 MHz, $CDCl_3$): 165.9 (C-1), 141.0 (C-3), 139.5 (Ar), 132.5 (Ar), 128.4 (Ar), 119.3 (C-2), 118.5 (CN), 112.9 (Ar), 62.0 (OMe), 32.5 (NMe); HRMS calcd for $C_{12}H_{12}N_2O_2Na$ $[M + Na]^+$ 239.0791, found 239.0790.

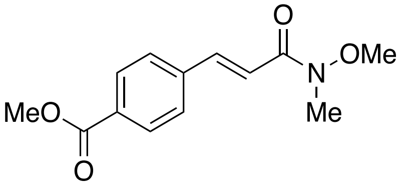

Methyl (2*E*)-4-(3-(methoxy(methyl)amino)-3-oxoprop-1-en-1-yl)benzoate ((*E*)-20l).

Methyl-4-formylbenzoate **19l** (33.0 mg, 0.201 mmol) was employed. The reaction time was 12 h. Purification by thin layer chromatography on silica (eluant; hexane/ethyl acetate = 1/1) to afford alkene (*E*)-**20l** (48.4 mg, 97%) as white solid.

R_f = 0.18 (silica gel, hexane/ethyl acetate = 2/1); mp: 88.8 °C; FT-IR (KBr) ν_{max}: 3417, 2947, 1720, 1651, 1612, 1566, 1442, 1412, 1381, 1281, 1173, 1095, 995, 771 cm^{-1}; ^1H NMR (500 MHz, $CDCl_3$): δ 8.12–8.00 (m, 2H, Ar), 7.75 (d, J = 16.0 Hz, 1H, H-3), 7.68–7.58 (m, 2H, Ar), 7.12 (d, J = 16.0 Hz, 1H, H-2), 3.93 (s, 3H, COOMe), 3.79 (s, 3H, NOMe), 3.32 (s, 3H, NMe); $^{13}C\{^1H\}$ NMR (125 MHz, $CDCl_3$): 166.5 (*C*OOMe), 166.3 (C-1), 142.0 (C-3), 139.4 (Ar), 130.9 (Ar), 130.0 (Ar), 127.8 (Ar), 118.1 (C-2), 62.0 (NOMe), 52.2 (COO*Me*), 32.5 (NMe); HRMS calcd for $C_{13}H_{15}NO_4Na$ $[M + Na]^+$

272.0893, found 272.0883.

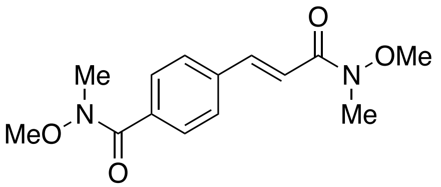

(2*E*)-3-(4-Methoxy(methyl)carbamoylphenyl)-*N*-methoxy-*N*-methylprop-2-enamide ((*E*)-20m).
4-Formyl-*N*-methoxy-*N*-methylbenzamide **19m** (38.5 mg, 0.199 mmol) was employed. The reaction time was 12 h. Purification by thin layer chromatography on silica (eluant; hexane/ethyl acetate = 1/3) to afford alkene (*E*)-**20m** (51.1 mg, 92%) as white solid.
R_f= 0.28 (silica gel, hexane/ethyl acetate = 1/2); mp: 79.5 °C; ATR-IR ν_{max}: 1652, 1622, 1556, 1455, 1424, 1385, 1208, 1186, 1005, 976, 851 cm^{-1}; ^{1}H NMR (500 MHz, $CDCl_3$): δ 7.75–7.68 (m, 2H, Ar), 7.74 (d, J =15.5 Hz, 1H, H-3), 7.64–7.57 (m, 2H, Ar), 7.09 (d, J = 15.5 Hz, 1H, H-2), 3.78 (s, 3H, 1NOMe), 3.56 (s, 3H, 4'NOMe), 3.37 (s, 3H, 4'NMe), 3.32 (s, 3H, 1NMe); $^{13}C\{^1H\}$ NMR (125 MHz, $CDCl_3$): 169.1 (C-4'), 166.5 (C-1), 142.3 (C-3), 137.2 (Ar), 135.0 (Ar), 128.7 (Ar), 127.5 (Ar), 117.3 (C-2), 61.9 (1NOMe), 61.1 (4'NOMe), 33.5 (4'NMe), 32.5 (1NMe); HRMS calcd for $C_{14}H_{18}N_2O_4Na$ $[M + Na]^+$ 301.1159, found 301.1151.

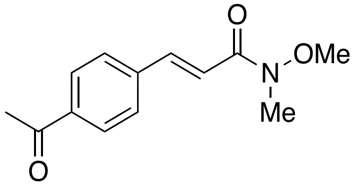

(2*E*)-*N*-Methoxy-*N*-methyl-3-(4-acetylphenyl)-prop-2-enamide ((*E*)-20n).
4-Acetylbenzaldehyde **19n** (29.5 mg, 0.199 mmol) was employed. The reaction time was 12 h. Purification by thin layer chromatography on silica (eluant; hexane/ethyl acetate = 1/2) to afford alkene (*E*)-**20n** (45.6 mg, 98%) as colorless oil.
R_f= 0.17 (silica gel, hexane/ethyl acetate = 2/1); FT-IR (neat) ν_{max}: 2970, 2939, 1682, 1658, 1620, 1419, 1381, 1365, 1265, 1180, 995, 957, 825 cm^{-1}; ^{1}H NMR (500 MHz, $CDCl_3$): 8.01–7.94 (m, 2H, Ar), 7.75 (d, J = 15.5 Hz, 1H, H-3), 7.68–7.62 (m, 2H, Ar), 7.12 (d, J = 15.5 Hz, 1H, H-2), 3.79 (s, 3H, OMe), 3.33 (s, 3H, NMe), 2.62 (s, 3H, $COCH_3$); $^{13}C\{^1H\}$ NMR (125 MHz, $CDCl_3$): 197.3 ($COCH_3$), 166.3 (C-1), 141.9 (C-3), 139.5 (Ar), 137.6 (Ar), 128.8 (Ar), 128.1 (Ar), 118.3 (C-2), 62.0 (OMe), 32.5 (NMe), 26.6 ($COCH_3$); HRMS calcd for $C_{13}H_{15}NO_3Na$ $[M + Na]^+$ 256.0944, found 256.0932.

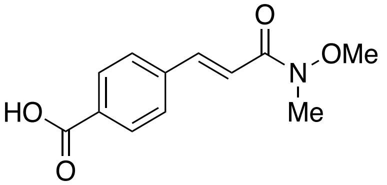

(2*E*)-4-(3-(Methoxy(methyl)amino)-3-oxoprop-1-en-1-yl)benzoic acid ((*E*)-20o).
To a solution of phosphate **1** (144 mg, 0.600 mmol) in tetrahydrofuran (4.7 mL), a 2.0 M solution of isopropylmagnesium chloride in tetrahydrofuran (0.27 mL, 0.540 mmol) was added at –78 °C. After the reaction mixture was stirred at –78 °C for 30 min, a solution of 4-formylbenzoic acid **19o** (30.1 mg, 0.200 mmol) in tetrahydrofuran (2.0 mL) was added under reflux, and the reaction mixture was stirred for 6 h. To the reaction mixture, saturated aqueous ammonium chloride and 1 M aqueous hydrochloride were added at 0 °C, and the mixture was extracted with ethyl acetate. The organic layer was dried over sodium sulfate. After filtration of the mixture and concentration of the solvent, 10% aqueous potassium carbonate was added to the crude mixture and was washed with diethyl ether for removing phosphate **1**. The mixture was acidified with 6 M aqueous hydrochloride and was extracted with ethyl acetate, and the organic layer was dried over sodium sulfate. After filtration of the mixture and concentration of the

solvent, the crude mixture was purified by thin layer chromatography on silica (eluant; chloroform/methanol = 9/1) to afford alkene (*E*)-**20o** (44.3 mg, 94%) as white solid.
R_f= 0.58 (silica gel, chloroform/methanol/acetic acid = 90/10/1); mp: 172.7 °C; ATR-IR ν_{max}: 1686, 1655, 1616, 1420, 1388, 1315, 1295, 771 cm^{-1}; ^{1}H NMR (500 MHz, dimethyl sulfoxide-d_6): δ 7.96 (d, J = 8.0 Hz, 2H, Ar), 7.81 (d, J = 8.0 Hz, 2H, Ar), 7.61 (d, J = 15.0 Hz, 1H, H-3), 7.20 (d, J = 15.0 Hz, 1H, H-2), 3.75 (s, 3H, OMe), 3.22 (s, 3H, NMe); ^{13}C{^{1}H} NMR (125 MHz, dimethyl sulfoxide-d_6): 167.3 (COOH), 165.5 (C-1), 141.2 (C-3), 138.4 (Ar), 132.8 (Ar), 129.7 (Ar), 128.1 (Ar), 118.5 (C-2), 61.9 (OMe), 32.2 (NMe); HRMS calcd for $C_{12}H_{12}NO_4$ [M – H]$^-$ 234.0761, found 234.0756.

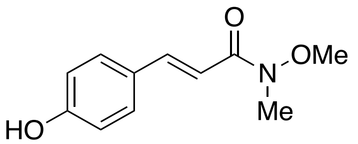

(2*E*)-*N*-Methoxy-*N*-methyl-3-(4-hydroxyphenyl)-prop-2-enamide ((*E*)-20p).

4-Hydroxybenzaldehyde **19p** (24.5 mg, 0.201 mmol) was employed. Phosphate **1** (144 mg, 0.600 mmol) and a 2.0 M solution of isopropylmagnesium chloride in tetrahydrofuran (0.27 mL, 0.540 mmol) were used. The reaction temperature was reflux and the reaction time was 24 h. Purification by thin layer chromatography on silica (eluant; hexane/ethyl acetate = 1/2) to afford alkene (*E*)-**20p** (38.8 mg, 93%) as white solid.
R_f= 0.32 (silica gel, hexane/ethyl acetate = 1/1); mp: 147.5 °C; ATR-IR ν_{max}: 3100, 3008, 1645, 1576, 1515, 1282, 830 cm^{-1}; ^{1}H NMR (500 MHz, $CDCl_3$): δ 7.68 (d, J = 16.0 Hz, 1H, H-3), 7.64 (s, 1H, OH), 7.47–7.40 (m, 2H, Ar), 6.92–6.87 (m, 2H, Ar), 6.89 (d, J = 16.0 Hz, 1H, H-2), 3.77 (s, 3H, OMe), 3.33 (s, 3H, NMe); ^{13}C{^{1}H} NMR (125 MHz, $CDCl_3$): 167.6 (C-1), 158.1 (Ar), 143.8 (C-3), 129.9 (Ar), 127.4 (Ar), 115.9 (Ar), 112.6 (C-2), 61.9 (OMe), 32.6 (NMe); HRMS calcd for $C_{11}H_{13}NO_3Na$ [M + Na]$^+$ 230.0788, found 230.0777.

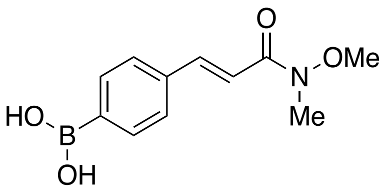

(2*E*)-*N*-Methoxy-*N*-methyl-3-(4-boronophenyl)-prop-2-enamide ((*E*)-20q).

To a solution of phosphate **1** (574 mg, 2.40 mmol) in tetrahydrofuran (9.3 mL), a 2.0 M solution of isopropylmagnesium chloride in tetrahydrofuran (1.08 mL, 2.16 mmol) was added at –78 °C. After the reaction mixture was stirred at –78 °C for 30 min, a solution of 4-boronobenzaldehyde **19q** (120 mg, 0.800 mmol) in tetrahydrofuran (4.0 mL) was added under reflux, and the reaction mixture was stirred for 4 h. To the reaction mixture, saturated aqueous ammonium chloride was added at 0 °C, and the mixture was extracted with ethyl acetate. The organic layer was dried over sodium sulfate. The mixture was filtered and concentrated to afford the crude mixture. From ^{1}H NMR spectra of the crude mixture, the yield of alkene (*E*)-**20q** was 97%. To the crude mixture, 28–30% aqueous ammonia and 10% aqueous potassium carbonate were added, and the mixture was washed with diethyl ether for removing phosphate **1**. To the mixture, buffer solution pH7 was added, and the mixture was neutralized with 6 M aqueous hydrochloride. The mixture was extracted with ethyl acetate, and the organic layer was dried over sodium sulfate. After filtration of the mixture and concentration of the solvent, the crude mixture was recrystallized with ethyl acetate (*ca.* 7 mL) to afford alkene (*E*)-**20q** (104 mg, 56%) as white solid and filtrate (159 mg) with the filtration of the mixture. The filtrate was washed with ethyl acetate to afford alkene (*E*)-**20q** (4.5 mg, 2.4%) as white solid and filtrate (147 mg) with the filtration of the mixture. The filtrate was washed with ethyl acetate to afford alkene (*E*)-**20q** (5.0 mg, 2.7%) as white solid and filtrate (136 mg) with the filtration of the mixture.
Total isolated yield: 114 mg, 61%
R_f= 0.55 (silica gel, chloroform/methanol/acetic acid = 90/10/1); mp: 247.1 °C; ATR-IR ν_{max}: 3369,

1647, 1606, 1554, 1463, 1409, 1375, 1349, 1047, 829 cm^{-1}; ^{1}H NMR (500 MHz, acetone-d_6): δ 7.92 (d, J = 8.0 Hz, 2H, Ar), 7.66 (d, J = 8.0 Hz, 2H, Ar), 7.64 (d, J = 15.0 Hz, 1H, H-3), 7.31–7.25 (m, 2H, $B(OH)_2$), 7.20 (d, J = 15.0 Hz, 1H, H-2), 3.81 (s, 3H, OMe), 3.24 (s, 3H, NMe); $^{13}C\{^1H\}$ NMR (125 MHz, acetone-d_6): 167.1 (C-1), 143.2 (C-3), 137.8 (Ar), 135.4 (Ar), 127.902 (Ar), 117.8 (C-2), 62.3 (OMe), 32.5 (NMe); HRMS calcd for $C_{11}H_{14}BNO_4Na$ $[M + Na]^+$ 258.0910, found 258.0908.

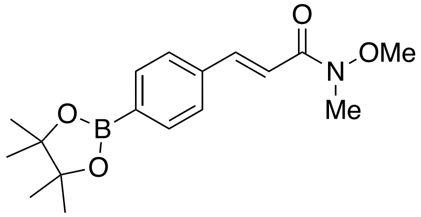

(2*E*)-*N*-Methoxy-*N*-methyl-3-(4-(4,4,5,5-tetramethyl-1,3,2-dioxaborolan-2-yl)phenyl)-prop-2-enamide ((*E*)-20r).

4-(4,4,5,5-Tetramethyl-1,3,2-dioxaborolan-2-yl)benzaldehyde **19r** (46.2 mg, 0.199 mmol) was employed. The reaction time was 12 h. Purification by thin layer chromatography on silica (eluant; hexane/ethyl acetate = 1/1) to afford alkene (*E*)-**20r** (54.2 mg, 86%) as white solid.

R_f= 0.56 (silica gel, hexane/ethyl acetate = 1/1); mp: 108.5 °C; ATR-IR ν_{max}: 2989, 1653, 1623, 1421, 1399, 1383, 1362, 1330, 1147, 1089, 998, 829, 646 cm^{-1}; ^{1}H NMR (500 MHz, $CDCl_3$): δ 7.86–7.78 (m, 2H, Ar), 7.74 (d, J = 16.0 Hz, 1H, H-3), 7.59–7.53 (m, 2H, Ar), 7.08 (d, J = 16.0 Hz, 1H, H-2), 3.78 (s, 3H, OMe), 3.32 (s, 3H, NMe), 1.35 (s, 12H, Bpin); $^{13}C\{^1H\}$ NMR (125 MHz, $CDCl_3$): 166.8 (C-1), 143.3 (C-3), 137.6 (Ar), 135.14 (Ar), 135.13 (Ar), 127.2 (Ar), 116.6 (C-2), 83.9 (Bpin), 61,9 (OMe), 32.5 (NMe), 24.8 (Bpin); HRMS calcd for $C_{17}H_{24}BNO_4Na$ $[M + Na]^+$ 340.1694, found 340.1684.

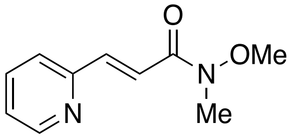

(2*E*)-*N*-Methoxy-*N*-methyl-3-(pyridin-2-yl)-prop-2-enamide ((*E*)-20s).

2-Pyridinecarbaldehyde **19s** (21.4 mg, 0.200 mmol) was employed. The reaction time was 12 h. Purification by thin layer chromatography on silica (eluant; diethyl ether/methanol = 12/1) to afford alkene (*E*)-**20s** (6.2 mg, 16%) as colorless oil.

R_f= 0.53 (silica gel, diethyl ether/methanol = 12/1); ATR-IR ν_{max}: 2936, 1661, 1623, 1586, 1471, 1435, 1416, 1384, 993, 783 cm^{-1}; ^{1}H NMR (500 MHz, $CDCl_3$): δ 8.69–8.63 (m, 1H, Py), 7.75–7.67 (m, 1H, Py), 7.72 (d, J = 15.0 Hz, 1H, H-3), 7.56 (d, J = 15.0 Hz, 1H, H-2), 7.44–7.38 (m, 1H, Py), 7.28–7.22 (m, 1H, Py), 3.80 (s, 3H, OMe), 3.33 (s, 3H, NMe); $^{13}C\{^1H\}$ NMR (125 MHz, $CDCl_3$): 166.6 (C-1), 153.4 (Py), 150.0 (Py), 141.9 (C-3), 136.7 (Py), 124.8 (Py), 123.9 (Py), 120.0 (C-2), 62.0 (OMe), 32.5 (NMe); HRMS calcd for $C_{10}H_{12}N_2O_2Na$ $[M + Na]^+$ 215.0791, found 215.0788.

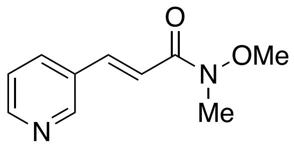

(2*E*)-*N*-Methoxy-*N*-methyl-3-(pyridin-3-yl)-prop-2-enamide ((*E*)-20t).

3-Pyridinecarbaldehyde **19t** (21.6 mg, 0.202 mmol) was employed. The reaction time was 12 h. The crude mixture was purified by thin layer chromatography on silica (eluant; ethyl acetate/methanol = 15/1×2) to afford the mixture of alkene (*E*)-**20t** and phosphate **1**. The mixture was purified by thin layer chromatography on silica (eluant; ethyl acetate/methanol = 40/1×3) to afford alkene (*E*)-**20t** (33.4 mg, 86%) as white solid.

R_f= 0.71 (silica gel, chloroform/methanol = 9/1); mp: 66.9 °C; FT-IR (KBr) ν_{max}: 3464, 3433, 1643, 1612, 1381, 995, 810 cm^{-1}; ^{1}H NMR (500 MHz, $CDCl_3$): δ 8.83–8.78 (m, 1H, Py), 8.62–8.57 (m, 1H,

Py), 7.91–7.85 (m, 1H, Py), 7.72 (d, J = 16.0 Hz, 1H, H-3), 7.37–7.31 (m, 1H, Py), 7.12 (d, J = 16.0 Hz, 1H, H-2), 3.79 (s, 3H, OMe), 3.33 (s, 3H, NMe); $^{13}C\{^1H\}$ NMR (125 MHz, $CDCl_3$): 166.1 (C-1), 150.5 (Py), 149.5 (Py), 139.7 (C-3), 134.4 (Py), 130.9 (Py), 123.6 (Py), 117.9 (C-2), 62.0 (OMe), 32.5 (NMe); HRMS calcd for $C_{20}H_{24}N_4O_4Na$ $[2M + Na]^+$ 407.1690, found 407.1685.

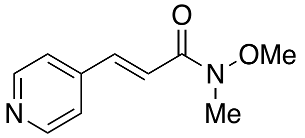

(2*E*)-*N*-Methoxy-*N*-methyl-3-(pyridin-4-yl)-prop-2-enamide ((*E*)-20u).

4-Pyridinecarbaldehyde **19u** (21.2 mg, 0.198 mmol) was employed. The reaction time was 12 h. The crude mixture was purified by thin layer chromatography on silica (eluant; ethyl acetate/methanol = 15/1 × 2) to afford the mixture of alkene (*E*)-**20u** and phosphate **1**. The mixture was purified by thin layer chromatography on silica (eluant; ethyl acetate/methanol = 40/1 × 3) to afford alkene (*E*)-**20u** (35.1 mg, 92%) as white solid.

R_f = 0.70 (silica gel, chloroform/methanol = 9/1); mp: 97.6 °C; FT-IR (KBr) ν_{max}: 3433, 2962, 2939, 1651, 1620, 1597, 1550, 1450, 1419, 1381, 1203, 1180, 995, 957, 825, 548, 501 cm^{-1}; ^1H NMR (500 MHz, $CDCl_3$): δ 8.65 (dd, J = 6.5, 0.5 Hz, 2H, Py), 7.65 (d, J = 16.0 Hz, 1H, H-3), 7.42 (dd, J = 6.5, 0.5 Hz, 2H, Py), 7.20 (d, J = 16.0 Hz, 1H, H-2), 3.79 (s, 3H, OMe), 3.33 (s, 3H, NMe); $^{13}C\{^1H\}$ NMR (125 MHz, $CDCl_3$): 165.8 (C-1), 150.5 (Py), 142.3 (Py), 140.5 (C-3), 121.8 (Py), 120.3 (C-2), 62.0 (OMe), 32.5 (NMe); HRMS calcd for $C_{20}H_{24}N_4O_4Na$ $[2M + Na]^+$ 407.1690, found 407.1702.

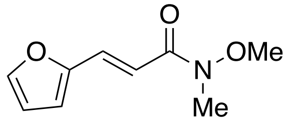

(2*E*)-*N*-Methoxy-*N*-methyl-3-(furan-2-yl)-prop-2-enamide ((*E*)-20v).

Furan-2-carbaldehyde **19v** (19.3 mg, 0.201 mmol) was employed. The reaction time was 12 h. Purification by thin layer chromatography on silica (eluant; hexane/ethyl acetate = 1/1) to afford alkene (*E*)-**20v** (33.1 mg, 91%) as colorless oil.

R_f = 0.45 (silica gel, hexane/ethyl acetate = 1/1); FT-IR (neat) ν_{max}: 2939, 1658, 1620, 1481, 1412, 1381, 1003, 980, 748 cm^{-1}; ^1H NMR (500 MHz, $CDCl_3$): δ 7.48 (d, J = 15.5 Hz, 1H, H-3), 7.47 (d, J = 2.0 Hz, 1H, furan), 6.91 (d, J = 15.5 Hz, 1H, H-2), 6.59 (d, J = 3.5 Hz, 1H, furan), 6.46 (dd, J = 3.5, 2.0 Hz, 1H, furan), 3.76 (s, 3H, OMe), 3.30 (s, 3H, NMe); $^{13}C\{^1H\}$ NMR (125 MHz, $CDCl_3$): 167.0 (C-1), 151.7 (furan), 144.1 (furan), 129.9 (C-3), 114.3 (furan), 113.6 (C-2), 112.2 (furan), 61.9 (OMe), 32.5 (NMe); HRMS calcd for $C_9H_{11}NO_3Na$ $[M + Na]^+$ 204.0631, found 204.0623.

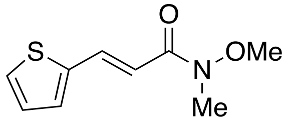

(2*E*)-*N*-Methoxy-*N*-methyl-3-(thiophen-2-yl)-prop-2-enamide ((*E*)-20w).

Thiophene-2-carbaldehyde **19w** (22.5 mg, 0.201 mmol) was employed. The reaction time was 12 h. Purification by thin layer chromatography on silica (eluant; hexane/ethyl acetate = 1/1) to afford alkene (*E*)-**20w** (37.5 mg, 95%) as colorless oil.

R_f = 0.48 (silica gel, hexane/ethyl acetate = 1/1); FT-IR (neat) ν_{max}: 2939, 1651, 1612, 1412, 1381, 1350, 1196, 1003, 972, 825, 710 cm^{-1}; ^1H NMR (500 MHz, $CDCl_3$): δ 7.83 (d, J = 15.5 Hz, 1H, H-3), 7.37–7.32 (m, 1H, thiophene), 7.28–7.23 (m, 1H, thiophene), 7.08–7.03 (m, 1H, thiophene), 6.82 (d, J = 15.5 Hz, 1H, H-2), 3.76 (s, 3H, OMe), 3.30 (s, 3H, NMe); $^{13}C\{^1H\}$ NMR (125 MHz, $CDCl_3$): 166.7 (C-1), 140.4 (thiophene), 135.9 (C-3), 130.6 (thiophene), 128.0 (thiophene), 127.5 (thiophene), 114.7 (C-2), 61.9 (OMe), 32.5 (NMe); HRMS calcd for $C_9H_{11}NO_2SNa$ $[M + Na]^+$ 220.0403, found 220.0393.

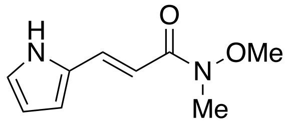

(2*E*)-*N*-Methoxy-*N*-methyl-3-(1*H*-pyrrol-2-yl)-prop-2-enamide ((*E*)-20x).

1*H*-Pyrrole-2-carbaldehyde **19x** (18.8 mg, 0.198 mmol) was employed. Phosphate **1** (144 mg, 0.600 mmol) and a 2.0 M solution of isopropylmagnesium chloride in tetrahydrofuran (0.27 mL, 0.540 mmol) were used. The reaction temperature was reflux and the reaction time was 24 h. Purification by thin layer chromatography on silica (eluant; hexane/ethyl acetate = 2/3) to afford alkene (*E*)-**20x** (16.5 mg, 46%) as colorless oil.

R_f= 0.27 (silica gel, hexane/ethyl acetate = 1/1); FT-IR (neat) ν_{max}: 3394, 3255, 1643, 1589, 1550, 1404, 1381, 1034, 1003, 741 cm^{-1}; ^{1}H NMR (500 MHz, $CDCl_3$): δ 9.10–8.80 (m, 1H, NH), 7.64 (d, J = 15.5 Hz, 1H, H-3), 6.95–6.87 (m, 1H, pyrrole), 6.64 (d, J = 15.5 Hz, 1H, H-2), 6.62–6.56 (m, 1H, pyrrole), 6.32–6.25 (m, 1H, pyrrole), 3.74 (s, 3H, OMe), 3.30 (s, 3H, NMe); ^{13}C{^{1}H} NMR (125 MHz, $CDCl_3$): 167.7 (C-1), 133.3 (C-3), 129.2 (pyrrole), 121.6 (pyrrole), 113.1 (pyrrole), 110.8 (pyrrole), 109.2 (C-2), 61.8 (OMe), 32.6 (NMe); HRMS calcd for $C_9H_{12}N_2O_2Na$ [M + Na]$^+$ 203.0791, found 203.0789.

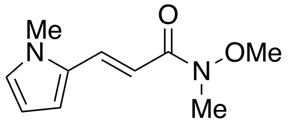

(2*E*)-*N*-Methoxy-*N*-methyl-3-(1-methyl-1*H*-pyrrol-2-yl)-prop-2-enamide ((*E*)-20y).

1-Methyl-1*H*-pyrrole-2-carbaldehyde **19y** (21.7 mg, 0.199 mmol) was employed. The reaction temperature was reflux and the reaction time was 6 h. Purification by thin layer chromatography on silica (eluant; hexane/ethyl acetate = 1/2) to afford alkene (*E*)-**20y** (35.8 mg, 93%) as colorless oil.

R_f= 0.19 (silica gel, hexane/ethyl acetate = 1/1); FT-IR (neat) ν_{max}: 3471, 2939, 1643, 1604, 1481, 1404, 1373, 1180, 1095, 1057, 1003, 972, 733 cm^{-1}; ^{1}H NMR (500 MHz, $CDCl_3$): δ 7.65 (d, J = 15.5 Hz, 1H, H-3), 6.76–6.66 (m, 2H, pyrrole), 6.75 (d, J = 15.5 Hz, 1H, H-2), 6.21–6.14 (m, 1H, pyrrole), 3.74 (s, 3H, OMe), 3.72 (s, 3H, NMe), 3.29 (s, 3H, CONMe); ^{13}C{^{1}H} NMR (125 MHz, $CDCl_3$): 167.8 (C-1), 131.1 (C-3), 130.1 (pyrrole), 126.2 (pyrrole), 110.8 (pyrrole), 110.7 (C-2), 109.0 (pyrrole), 61.7 (OMe), 34.3 (NMe), 32.5 (CONMe); HRMS calcd for $C_{10}H_{14}N_2O_2Na$ [M + Na]$^+$ 217.0947, found 217.0947.

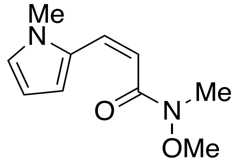

(2*Z*)-*N*-Methoxy-*N*-methyl-3-(1-methyl-1*H*-pyrrol-2-yl)-prop-2-enamide ((*Z*)-20y).

R_f= 0.48 (silica gel, hexane/ethyl acetate = 1/1); ATR-IR ν_{max}: 2964, 2934, 1653, 1600, 1484, 1464, 1400, 1364, 1340, 1307, 1093, 1062, 1001, 737 cm^{-1}; ^{1}H NMR (500 MHz, $CDCl_3$): δ 7.35–7.14 (m, 1H, pyrrole), 6.74–6.63 (m, 1H, pyrrole), 6.64 (d, J = 11.5 Hz, 1H, H-3), 6.20–6.11 (m, 1H, pyrrole), 6.05 (d, J = 11.5 Hz, 1H, H-2), 3.70 (s, 3H, OMe), 3.66 (s, 3H, NMe), 3.27 (s, 3H, CONMe); ^{13}C{^{1}H} NMR (125 MHz, $CDCl_3$): 167.7 (C-1), 128.3 (pyrrole), 126.3 (C-3), 125.3 (pyrrole), 115.1 (pyrrole), 112.4 (C-2), 108.6 (pyrrole), 61.5 (OMe), 34.1 (NMe), 32.7 (CONMe); HRMS calcd for $C_{10}H_{14}N_2O_2Na$ [M + Na]$^+$ 217.0947, found 217.0956.

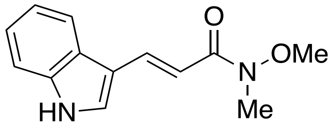

(2*E*)-*N*-Methoxy-*N*-methyl-3-(1*H*-indol-3-yl)-prop-2-enamide ((*E*)-20z).

1*H*-Indole-3-carbaldehyde **19z** (28.9 mg, 0.199 mmol) was employed. Phosphate **1** (144 mg, 0.600 mmol) and a 2.0 M solution of isopropylmagnesium chloride in tetrahydrofuran (0.27 mL, 0.540 mmol)

were used. The reaction temperature was reflux and the reaction time was 24 h. Purification by thin layer chromatography on silica (eluant; hexane/ethyl acetate = 1/3) to afford alkene (*E*)-**20z** (40.4 mg, 88%) as white solid.
R_f= 0.18 (silica gel, hexane/ethyl acetate = 1/1); mp: 152.8 °C; ATR-IR ν_{max}: 3157, 2970, 2927, 1634, 1570, 1417, 1386, 1246, 745 cm^{-1}; ^{1}H NMR (500 MHz, $CDCl_3$): δ 9.08–8.91 (m, 1H, NH), 7.97 (d, J = 16.0 Hz, 1H, H-3), 7.96–7.89 (m, 1H, indole), 7.52–7.46 (m, 1H, indole), 7.46–7.39 (m, 1H, indole), 7.31–7.20 (m, 2H, indole), 7.07 (d, J = 16.0 Hz, 1H, H-2), 3.82 (s, 3H, OMe), 3.34 (s, 3H, NMe); ^{13}C{^{1}H} NMR (125 MHz, $CDCl_3$): 168.5 (C-1), 137.2 (indole), 137.1 (C-3), 128.9 (indole), 125.4 (indole), 123.1 (indole), 121.2 (indole), 120.4 (indole), 114.0 (indole), 111.9 (indole), 111.0 (C-2), 61.7 (OMe), 32.6 (NMe); HRMS calcd for $C_{13}H_{14}N_2O_2Na$ [M + Na]$^+$ 253.0947, found 253.0937.

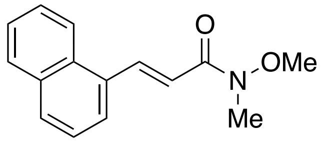

(2*E*)-*N*-Methoxy-*N*-methyl-3-(naphthalen-1-yl)-prop-2-enamide ((*E*)-20aa).

1-Naphthaldehyde **19aa** (31.1 mg, 0.199 mmol) was employed. The reaction time was 12 h. Purification by thin layer chromatography on silica (eluant; hexane/ethyl acetate = 1/1) to afford alkene (*E*)-**20aa** (45.5 mg, 95%) as white solid.
R_f= 0.53 (silica gel, hexane/ethyl acetate = 1/1); mp: 68.7 °C; FT-IR (KBr) ν_{max}: 3464, 3433, 3402, 1643, 1612, 1419, 1381, 1173, 995, 795, 771 cm^{-1}; ^{1}H NMR (500 MHz, $CDCl_3$): δ 8.57 (d, J = 15.5 Hz, 1H, H-3), 8.30–8.20 (m, 1H, naphthalene), 7.94–7.84 (m, 2H, naphthalene), 7.82–7.74 (m, 1H, naphthalene), 7.63–7.44 (m, 3H, naphthalene), 7.01 (d, J = 15.5 Hz, 1H, H-2), 3.79 (s, 3H, OMe), 3.36 (s, 3H, NMe); ^{13}C{^{1}H} NMR (125 MHz, $CDCl_3$): 166.8 (C-1), 140.5 (C-3), 133.6 (naphthalene), 132.7 (naphthalene), 131.5 (naphthalene), 130.0 (naphthalene), 128.6 (naphthalene), 126.7 (naphthalene), 126.1 (naphthalene), 125.3 (naphthalene), 124.7 (naphthalene), 123.7 (naphthalene), 118.7 (C-2), 61.9 (OMe), 32.5 (NMe); HRMS calcd for $C_{15}H_{15}NO_2Na$ [M + Na]$^+$ 264.0995, found 264.1007.

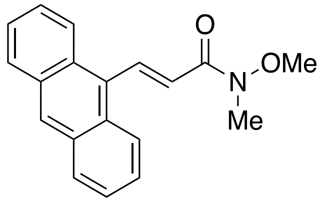

(2*E*)-*N*-Methoxy-*N*-methyl-3-(anthracen-9-yl)-prop-2-enamide ((*E*)-20ab).

Anthracene-9-carbaldehyde **19ab** (41.1 mg, 0.199 mmol) was employed. The reaction temperature was reflux and the reaction time was 6 h. Purification by thin layer chromatography on silica (eluant; hexane/ethyl acetate = 1/1) to afford alkene (*E*)-**20ab** (56.9 mg, 98%) as yellow oil.
R_f= 0.56 (silica gel, hexane/ethyl acetate = 1/1); FT-IR (neat) ν_{max}: 3055, 2970, 2939, 1658, 1628, 1442, 1419, 1381, 1180, 995, 887, 779, 741, 702 cm^{-1}; ^{1}H NMR (500 MHz, $CDCl_3$): δ 8.66 (d, J = 16.0 Hz, 1H, H-3), 8.47–8.41 (m, 1H, anthracene), 8.33–8.24 (m, 2H, anthracene), 8.06–7.95 (m, 2H, anthracene), 7.54–7.44 (m, 4H, anthracene), 7.00 (d, J = 16.0 Hz, 1H, H-2), 3.72 (s, 3H, OMe), 3.40 (s, 3H, NMe); ^{13}C{^{1}H} NMR (125 MHz, $CDCl_3$): 166.4 (C-1), 140.6 (C-3), 131.3 (anthracene), 130.5 (anthracene), 129.4 (anthracene), 128.7 (anthracene), 127.7 (anthracene), 126.0 (anthracene), 125.5 (anthracene), 125.3 (anthracene), 125.1 (C-2), 62.0 (OMe), 32.6 (NMe); HRMS calcd for $C_{19}H_{17}NO_2Na$ [M + Na]$^+$ 314.1151, found 314.1155.

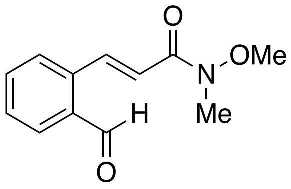

(2*E*)-*N*-Methoxy-*N*-methyl-3-(2-formylphenyl)-prop-2-enamide ((*E*)-20ac).

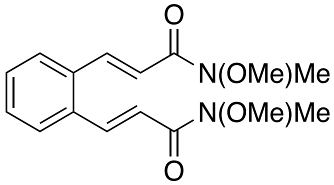

(2*E*,2'*E*)-3,3'-(1,2-Phenylene)bis(*N*-methoxy-*N*-methyl-prop-2-enamide) ((*E*,*E*)-20ac).

Benzene-1,2-dicarbaldehyde **19ac** (27.0 mg, 0.201 mmol) was employed. The reaction time was 12 h. Purification by thin layer chromatography on silica (eluant; hexane/ethyl acetate = 1/3) to afford alkene (*E*)-**20ac** (10.3 mg, 23%) as colorless oil and alkene (*E*,*E*)-**20ac** (47.3 mg, 77%) as black oil.

(*E*)-**20ac**

R_f= 0.45 (silica gel, hexane/ethyl acetate = 1/2); FT-IR (neat) ν_{max}: 3479, 3456, 2939, 1697, 1651, 1620, 1481, 1412, 1381, 1188, 995, 764 cm^{-1}; ^{1}H NMR (500 MHz, $CDCl_3$): δ 10.38 (s, 1H CHO), 8.47 (d, J = 16.0 Hz, 1H, H-3), 7.95–7.87 (m, 1H, Ar), 7.70–7.58 (m, 2H, Ar), 7.57–7.50 (m, 1H, Ar), 6.95 (d, J = 16.0 Hz, 1H, H-2), 3.77 (s, 3H, OMe), 3.33 (s, 3H, NMe); ^{13}C{^{1}H} NMR (125 MHz, $CDCl_3$): 191.3 (CHO), 166.0 (C-1), 139.3 (C-3), 137.9 (Ar), 134.0 (Ar), 133.8 (Ar), 130.8 (Ar), 129.5 (Ar), 128.1 (Ar), 121.4 (C-2), 62.0 (OMe), 32.5 (NMe); HRMS calcd for $C_{12}H_{13}NO_3Na$ [M + Na]$^+$ 242.0788, found 242.0781.

(*E*,*E*)-**20ac**

R_f= 0.18 (silica gel, hexane/ethyl acetate = 1/2); FT-IR (neat) ν_{max}: 3471, 2970, 2939, 1651, 1612, 1473, 1419, 1381, 1180, 1103, 995, 980, 764 cm^{-1}; ^{1}H NMR (500 MHz, $CDCl_3$): δ 8.05 (d, J = 16.0 Hz, 2H, H-3, H-3'), 7.63–7.54 (m, 2H, Ar), 7.43–7.34 (m, 2H, Ar), 6.91 (d, J = 16.0 Hz, 2H, H-2, H-2'), 3.77 (s, 6H, OMe), 3.31 (s, 6H, NMe); ^{13}C{^{1}H} NMR (125 MHz, $CDCl_3$): 166.4 (C-1, C-1'), 140.9 (C-3, C-3'), 135.3 (Ar), 129.3 (Ar), 128.2 (Ar), 119.9 (C-2, C-2'), 62.0 (OMe), 32.5 (NMe); HRMS calcd for $C_{16}H_{20}N_2O_4Na$ [M + Na]$^+$ 327.1315, found 327.1312.

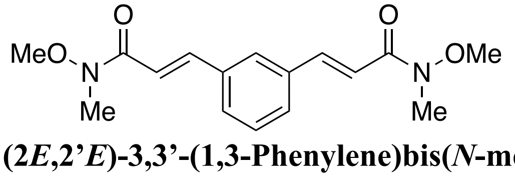

(2*E*,2'*E*)-3,3'-(1,3-Phenylene)bis(*N*-methoxy-*N*-methyl-prop-2-enamide) ((*E*,*E*)-20ad).

Benzene-1,3-dicarbaldehyde **19ad** (26.9 mg, 0.201 mmol) was employed. Phosphate **1** (191 mg, 0.800 mmol) and a 2.0 M solution of isopropylmagnesium chloride in tetrahydrofuran (0.36 mL, 0.720 mmol) were used. The reaction temperature was reflux and the reaction time was 6 h. Purification by thin layer chromatography on silica (eluant; ethyl acetate) to afford alkene (*E*,*E*)-**20ad** (59.0 mg, 97 %) as white solid.

R_f= 0.23 (silica gel, hexane/ethyl acetate = 1/2); mp: 86.5 °C; ATR-IR ν_{max}: 2938, 1662, 1647, 1623, 1465, 1441, 1413, 1374, 1179, 1096, 994, 794 cm^{-1}; ^{1}H NMR (500 MHz, $CDCl_3$): δ 7.75 (d, J = 16.0 Hz, 2H, H-3, H-3'), 7.74–7.69 (m, 1H, Ar), 7.63–7.54 (m, 2H, Ar), 7.45–7.38 (m, 1H, Ar), 7.07 (d, J = 16.0 Hz, 2H, H-2, H-2'), 3.79 (s, 6H, OMe), 3.33 (s, 6H, NMe); ^{13}C{^{1}H} NMR (125 MHz, $CDCl_3$): 166.7 (C-1, C-1'), 142.7 (C-3, C-3'), 135.8 (Ar), 129.2 (Ar), 129.0 (Ar), 127.8 (Ar), 116.6 (C-2, C-2'), 61.9 (OMe), 32.5 (NMe); HRMS calcd for $C_{16}H_{20}N_2O_4Na$ [M + Na]$^+$ 327.1315, found 327.1314.

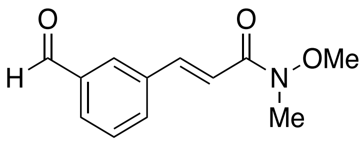

(2*E*)-*N*-Methoxy-*N*-methyl-3-(3-formylphenyl)-prop-2-enamide ((*E*)-20ad).
colorless oil
R_f= 0.42 (silica gel, hexane/ethyl acetate = 1/2); FT-IR (neat) ν_{max}: 3471, 1697, 1658, 1620, 1442, 1419, 1381, 1149, 995, 795 cm^{-1}; ^{1}H NMR (500 MHz, $CDCl_3$): δ 10.07 (s, 1H CHO), 8.13–8.07 (m, 1H, Ar), 7.93–7.84 (m, 1H Ar), 7.84–7.77 (m, 1H, Ar), 7.78 (d, J = 16.0 Hz, 1H, H-3), 7.62–7.53 (m, 1H, Ar), 7.14 (d, J = 16.0 Hz, 1H, H-2), 3.80 (s, 3H, OMe), 3.33 (s, 3H, NMe); ^{13}C{^{1}H} NMR (125 MHz, $CDCl_3$): 191.9 (CHO), 166.3 (C-1), 141.7 (C-3), 136.8 (Ar), 136.2 (Ar), 134.0 (Ar), 130.8 (Ar), 129.6 (Ar), 128.2 (Ar), 117.6 (C-2), 62.0 (OMe), 32.5 (NMe); HRMS calcd for $C_{12}H_{13}NO_3Na$ [M + Na]$^+$ 242.0788, found 242.0779.

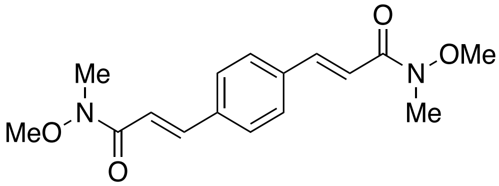

(2*E*,2'*E*)-3,3'-(1,4-Phenylene)bis(*N*-methoxy-*N*-methyl-prop-2-enamide) ((*E*,*E*)-20ae).
Benzene-1,4-dicarbaldehyde **19ae** (26.6 mg, 0.198 mmol) was employed. Phosphate **1** (191 mg, 0.800 mmol) and a 2.0 M solution of isopropylmagnesium chloride in tetrahydrofuran (0.36 mL, 0.720 mmol) were used. The reaction temperature was reflux and the reaction time was 2 h. Purification by thin layer chromatography on silica (eluant; ethyl acetate×2) to afford alkene (*E*,*E*)-**20ae** (58.2 mg, 96 %) as white solid.
R_f= 0.22 (silica gel, hexane/ethyl acetate = 1/2); mp: 183.6 °C; FT-IR (KBr) ν_{max}: 3433, 2978, 2939, 1643, 1604, 1458, 1427, 1381, 1196, 1180, 995, 825 cm^{-1}; ^{1}H NMR (500 MHz, $CDCl_3$): δ 7.73 (d, J = 16.0 Hz, 2H, H-3, H-3'), 7.59 (s, 4H, Ar), 7.07 (d, J = 16.0 Hz, 2H, H-2, H-2'), 3.78 (s, 6H, OMe), 3.32 (s, 6H, NMe); ^{13}C{^{1}H} NMR (125 MHz, $CDCl_3$): 166.7 (C-1, C-1'), 142.4 (C-3, C-3'), 136.5 (Ar), 128.4 (Ar), 116.6 (C-2, C-2'), 61.9 (OMe), 32.5 (NMe); HRMS calcd for $C_{16}H_{20}N_2O_4Na$ [M + Na]$^+$ 327.1315, found 327.1308.

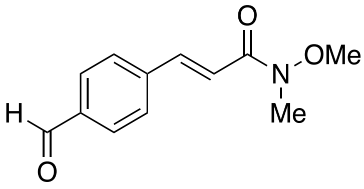

(2*E*)-*N*-Methoxy-*N*-methyl-3-(4-formylphenyl)-prop-2-enamide ((*E*)-20ae).
white solid
R_f= 0.45 (silica gel, hexane/ethyl acetate = 1/2); mp: 91.4 °C; FT-IR (KBr) ν_{max}: 3425, 3394, 2723, 1705, 1651, 1612, 1597, 1419, 1389, 1196, 1165, 995, 825, 764, 540 cm^{-1}; ^{1}H NMR (500 MHz, $CDCl_3$): δ 10.03 (s, 1H, CHO), 7.95–7.87 (m, 2H, Ar), 7.76 (d, J = 16.0 Hz, 1H, H-3), 7.75–7.69 (m, 2H, Ar), 7.15 (d, J = 16.0 Hz, 1H, H-2), 3.79 (s, 3H, OMe), 3.33 (s, 3H, NMe); ^{13}C{^{1}H} NMR (125 MHz, $CDCl_3$): 191.5 (CHO), 166.1 (C-1), 141.7 (C-3), 140.9 (Ar), 136.8 (Ar), 130.1 (Ar), 128.5 (Ar), 118.9 (C-2), 62.0 (OMe), 32.5 (NMe); HRMS calcd for $C_{12}H_{13}NO_3Na$ [M + Na]$^+$ 242.0788, found 242.0793.

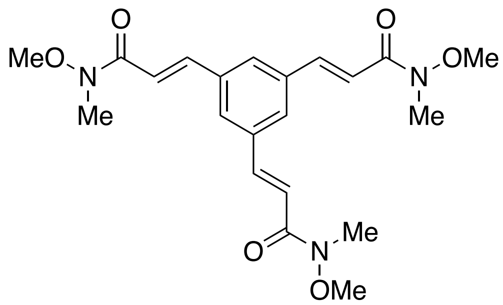

(2*E*,2'*E*,2''*E*)-3,3',3''-(Benzene-1,3,5-triyl)tris(*N*-methoxy-*N*-methyl-prop-2-enamide) ((*E*,*E*,*E*)-20af).

Benzene-1,3,5-tricarbaldehyde **19af** (32.3 mg, 0.199 mmol) was employed. Phosphate **1** (287 mg, 1.20 mmol) and a 2.0 M solution of isopropylmagnesium chloride in tetrahydrofuran (0.54 mL, 1.08 mmol) were used. The reaction temperature was reflux and the reaction time was 4 h. Purification by thin layer chromatography on silica (eluant; chloroform/methanol = 20/1) to afford the mixture of alkene (*E*,*E*,*E*)-**20af** and phosphate **1**. The mixture was purified by flash column chromatography (eluant: ethyl acetate to ethyl acetate/methanol = 15/1) to alkene (*E*,*E*,*E*)-**20af** (66.6 mg, 80 %) as white solid.

R_f= 0.18 (silica gel, ethyl acetate); mp: 213.6 °C; ATR-IR ν_{max}: 2964, 1654, 1615, 1472, 1448, 1416, 1386, 1181, 1000 cm^{-1}; ^{1}H NMR (500 MHz, $CDCl_3$): δ 7.76 (d, J = 16.0 Hz, 3H, H-3, H-3', H-3''), 7.72 (s, 3H, Ar), 7.00 (d, J = 16.0 Hz, 3H, H-2, H-2', H-2''), 3.81 (s, 9H, OMe), 3.34 (s, 9H, NMe); $^{13}C\{^1H\}$ NMR (125 MHz, $CDCl_3$): 166.4 (C-1, C-1', C-1''), 142.1 (C-3, C-3', C-3''), 136.4 (Ar), 128.3 (Ar), 117.4 (C-2, C-2', C-2''), 62.0 (OMe), 32.5 (NMe); HRMS calcd for $C_{21}H_{27}N_3O_6Na$ $[M + Na]^+$ 440.1792, found 440.1794.

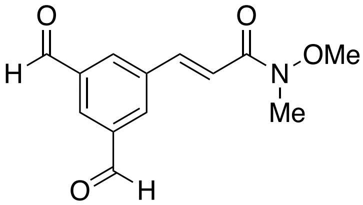

(2*E*)-*N*-Methoxy-*N*-methyl-3-(3,5-diformylphenyl)-prop-2-enamide ((*E*,*E*)-20af).

white solid

R_f= 0.60 (silica gel, hexane/ethyl acetate = 1/2); mp: 141.2 °C; ATR-IR ν_{max}: 1697, 1659, 1624, 1595, 1463, 1381, 1134, 994 cm^{-1}; ^{1}H NMR (500 MHz, $CDCl_3$): δ 10.15 (s, 2H, CHO), 8.38–8.34 (m, 1H, Ar), 8.33–8.27 (m, 2H, Ar), 7.83 (d, J = 16.5 Hz, 1H, H-3), 7.22 (d, J = 16.5 Hz, 1H, H-2), 3.81 (s, 3H, OMe), 3.34 (s, 3H, NMe); $^{13}C\{^1H\}$ NMR (125 MHz, $CDCl_3$): 190.6 (CHO), 165.8 (C-1), 140.2 (C-3), 137.5 (Ar), 137.4 (Ar), 133.2 (Ar), 131.5 (Ar), 119.4 (C-2), 62.1 (OMe), 32.5 (NMe); HRMS calcd for $C_{13}H_{13}NO_4Na$ $[M + Na]^+$ 270.0737, found 270.0745.

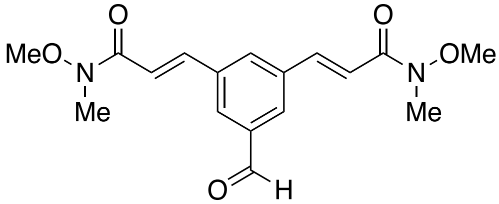

(2*E*,2'*E*)-3,3'-(5-Formyl-1,3-phenylene)bis(*N*-methoxy-*N*-methyl-prop-2-enamide) ((*E*)-20af).

white solid

R_f= 0.33 (silica gel, hexane/ethyl acetate = 1/2); mp: 136.2 °C; ATR-IR ν_{max}: 1702, 1661, 1623, 1471, 1428, 1386, 982 cm^{-1}; ^{1}H NMR (500 MHz, $CDCl_3$): δ 10.10 (s, 1H, CHO), 8.09–8.04 (m, 2H, Ar), 7.93–7.89 (m, 1H, Ar), 7.79 (d, J = 16.0 Hz, 2H, H-3, H-3'), 7.16 (d, J = 16.0 Hz, 2H, H-2, H-2'), 3.81 (s, 6H, OMe), 3.34 (s, 6H, NMe); $^{13}C\{^1H\}$ NMR (125 MHz, $CDCl_3$): 191.4 (CHO), 166.1 (C-1, C-1'), 141.1 (C-3, C-3'), 137.3 (Ar), 136.9 (Ar), 133.5 (Ar), 129.0 (Ar), 118.4 (C-2, C-2'), 62.1 (OMe), 32.5

(NMe); HRMS calcd for $C_{17}H_{20}N_2O_5Na$ $[M + Na]^+$ 355.1264, found 355.1270.

Application of ^{i}PrMgCl-deprotonating Weinreb Amide-Type Horner–Wadsworth–Emmons Reaction to Successive Elongation.

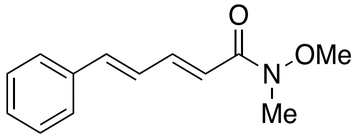

(2*E*,4*E*)-*N*-Methoxy-*N*-methyl-5-phenylpenta-2,4-dienamide ((2*E*,4*E*)-18d).
Reduction

To a solution of Weinreb amide (*E*)-**20a** (38.2 mg, 0.200 mmol) in tetrahydrofuran (4.0 mL), a 1.0 M solution of diisobutylaluminum hydride in hexane (0.28 mL, 0.280 mmol) was added at –78 °C, and the reaction mixture was stirred at –78 °C for 30 min. Since Weinreb amide (*E*)-**20a** remained, a 1.0 M solution of diisobutylaluminum hydride in hexane (0.04 mL, 0.040 mmol) was added to the reaction mixture. After the reaction mixture stirred at –78 °C for 15 min, methanol and saturated aqueous Rochelle salt were added, and the mixture was stirred at room temperature. The mixture was extracted with ethyl acetate, and the organic layer was dried over sodium sulfate. After filtration of the mixture and concentration of the solvent, *trans*-cinnamaldehyde **17d** (26.7 mg) was obtained as colorless oil and used in the next HWE reaction without further purification.

E-selective HWE reaction

To a solution of phosphate **1** (95.7 mg, 0.400 mmol) in tetrahydrofuran (4.7 mL), a 2.0 M solution of isopropylmagnesium chloride in tetrahydrofuran (0.18 mL, 0.360 mmol) was added at –78 °C. After the reaction mixture was stirred at –78 °C for 30 min, a solution of *trans*-cinnamaldehyde **17d** (26.7 mg) in tetrahydrofuran (2.0 mL) was added at room temperature, and the reaction mixture was stirred for 8 h. To the reaction mixture, saturated aqueous ammonium chloride was added at 0 °C, and the mixture was extracted with ethyl acetate. The organic layer was dried over sodium sulfate. After filtration of the mixture and concentration of the solvent, the crude mixture was purified by thin layer chromatography on silica (eluant; hexane/ethyl acetate = 1/1) to afford diene (2*E*,4*E*)-**18d** (36.1 mg, 83% over 2 steps) as white solid and alkene diene (2*Z*,4*E*)-**18d** (1.1 mg, 2.5% over 2 steps).

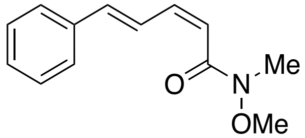

(2*Z*,4*E*)-*N*-Methoxy-*N*-methyl-5-phenylpenta-2,4-dienamide ((2*Z*,4*E*)-18d).
Reduction

To a solution of Weinreb amide (*E*)-**20a** (38.2 mg, 0.200 mmol) in tetrahydrofuran (4.0 mL), a 1.0 M solution of diisobutylaluminum hydride in hexane (0.28 mL, 0.280 mmol) was added at –78 °C, and the reaction mixture was stirred at –78 °C for 30 min. Since Weinreb amide (*E*)-**20a** remained, a 1.0 M solution of diisobutylaluminum hydride in hexane (0.04 mL, 0.040 mmol) was added to the reaction mixture. After the reaction mixture stirred at –78 °C for 15 min, methanol and saturated aqueous Rochelle salt were added, and the mixture was stirred at room temperature. The mixture was extracted with ethyl acetate, and the organic layer was dried over sodium sulfate. After filtration of the mixture and concentration of the solvent, *trans*-cinnamaldehyde **17d** (26.8 mg) was obtained as colorless oil and used in the next HWE reaction without further purification.

Z-selective HWE reaction
Ref. 4a

To a solution of phosphate **21** (139 mg, 0.400 mmol) and 1,4,7,10,13,16-hexaoxacyclooctadecane (190 mg, 0.720 mmol) in tetrahydrofuran (4.7 mL), a 1.0 M solution of potassium bis(trimethylsilyl)amide in tetrahydrofuran (0.36 mL, 0.360 mmol) was added at –78 °C. After the reaction mixture was stirred at –78 °C for 30 min, a solution of *trans*-cinnamaldehyde **17d** (26.8 mg) in tetrahydrofuran (2.0 mL) was

added at –78 °C, and the reaction mixture was stirred for 3 h. To the reaction mixture, saturated aqueous ammonium chloride was added at –78 °C, and the mixture was extracted with ethyl acetate. The organic layer was dried over sodium sulfate. After filtration of the mixture and concentration of the solvent, the crude mixture was purified by thin layer chromatography on silica (eluant; hexane/ethyl acetate = 1/1) to afford diene (2*Z*,4*E*)-**18d** (40.2 mg, 93% over 2 steps) as white solid and diene (2*E*,4*E*)-**18d** (trace over 2 steps).

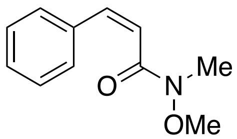

(2*Z*)-*N*-Methoxy-*N*-methyl-3-phenylprop-2-enamide ((*Z*)-20a).
Ref. 4a

To a solution of phosphate **21** (139 mg, 0.400 mmol) and 1,4,7,10,13,16-hexaoxacyclooctadecane (190 mg, 0.720 mmol) in tetrahydrofuran (4.7 mL), a 1.0 M solution of potassium bis(trimethylsilyl)amide in tetrahydrofuran (0.36 mL, 0.360 mmol) was added at –78 °C. After the reaction mixture was stirred at –78 °C for 30 min, a solution of benzaldehyde **19a** (21.3 mg, 0.201 mmol) in tetrahydrofuran (2.0 mL) was added at –78 °C, and the reaction mixture was stirred for 20 min. To the reaction mixture, saturated aqueous ammonium chloride was added at –78 °C, and the mixture was extracted with ethyl acetate. The organic layer was dried over sodium sulfate. After filtration of the mixture and concentration of the solvent, the crude mixture was purified by thin layer chromatography on silica (eluant; hexane/ethyl acetate = 1/1) to afford alkene (*Z*)-**20a** (34.1 mg, 89%) as colorless oil.

R_f= 0.36 (silica gel, hexane/ethyl acetate = 2/1); FT-IR (neat) ν_{max}: 2970, 2931, 1651, 1496, 1427, 1358, 1180, 1103, 1003, 787, 702 cm^{-1}; ^1H NMR (500 MHz, $CDCl_3$): δ 7.65–7.40 (m, 2H, Ar), 7.40–7.23 (m, 3H, Ar), 6.77 (d, J = 12.5 Hz, 1H, H-3), 6.28 (brd, J = 12.5 Hz, 1H, H-2), 3.65 (brs, 3H, OMe), 3.26 (brs, 3H, NMe); $^{13}C\{^1H\}$ NMR (125 MHz, $CDCl_3$): 168.1 (C-1), 137.7 (C-3), 135.2 (Ar), 129.1 (Ar), 128.6 (Ar), 128.2 (Ar), 120.6 (C-2), 61.7 (OMe), 32.2 (NMe); HRMS calcd for $C_{11}H_{13}NO_2Na$ $[M + Na]^+$ 214.0838, found 214.0842.

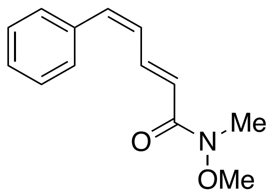

(2*E*,4*Z*)-*N*-Methoxy-*N*-methyl-5-phenylpenta-2,4-dienamide ((2*E*,4*Z*)-18d).
Reduction

To a solution of Weinreb amide (*Z*)-**20a** (37.9 mg, 0.198 mmol) in tetrahydrofuran (4.0 mL), a 1.0 M solution of diisobutylaluminum hydride in hexane (0.28 mL, 0.280 mmol) was added at –78 °C, and the reaction mixture was stirred at –78 °C for 50 min. Since Weinreb amide (*Z*)-**20a** remained, a 1.0 M solution of diisobutylaluminum hydride in hexane (0.06 mL, 0.060 mmol) was added to the reaction mixture. After the reaction mixture stirred at –78 °C for 30 min, methanol and saturated aqueous Rochelle salt were added, and the mixture was stirred at room temperature. The mixture was extracted with ethyl acetate, and the organic layer was dried over sodium sulfate. After filtration of the mixture and concentration of the solvent, (2*Z*)-3-phenylprop-2-enal (26.4 mg) was obtained as yellow oil and used in the next HWE reaction without further purification.

E-selective HWE reaction

To a solution of phosphate **1** (94.7 mg, 0.396 mmol) in tetrahydrofuran (4.6 mL), a 2.0 M solution of isopropylmagnesium chloride in tetrahydrofuran (0.18 mL, 0.356 mmol) was added at –78 °C. After the reaction mixture was stirred at –78 °C for 30 min, a solution of (2*Z*)-3-phenylprop-2-enal (26.4 mg) in tetrahydrofuran (2.0 mL) was added at room temperature, and the reaction mixture was stirred for 3 h. To the reaction mixture, saturated aqueous ammonium chloride was added at 0 °C, and the mixture was

extracted with ethyl acetate. The organic layer was dried over sodium sulfate. After filtration of the mixture and concentration of the solvent, the crude mixture was purified by thin layer chromatography on silica (eluant; hexane/ethyl acetate = 1/1) to afford diene (2*E*,4*Z*)-**18d** (37.1 mg, 86% over 2 steps) as pale yellow oil and diene (2*Z*,4*Z*)-**18d** (0.8 mg, 1.9% over 2 steps) as pale yellow oil.

(2*E*,4*Z*)-**18d**

R_f= 0.30 (silica gel, hexane/ethyl acetate = 2/1); FT-IR (neat) ν_{max}: 2962, 2939, 1651, 1612, 1450, 1419, 1381, 1180, 1003, 864, 810, 702 cm^{-1}; ^{1}H NMR (500 MHz, C_6D_6): δ 8.36 (ddd, J = 15.0, 11.5, 1.0 Hz, 1H, H-3), 7.29–7.23 (m, 2H, Ar), 7.05–6.93 (m, 3H, Ar), 6.69 (d, J = 15.0 Hz, 1H, H-2), 6.50 (dd, J = 11.5, 1.0 Hz, 1H, H-5), 6.24 (dd, J = 11.5, 11.5 Hz, 1H, H-4), 3.06 (s, 3H, OMe), 2.94 (s, 3H, NMe); ^{13}C{^{1}H} NMR (125 MHz, C_6D_6): 167.0 (C-1), 139.1 (C-3), 137.0 (C-5), 136.8 (Ar), 129.5 (Ar), 128.7 (Ar), 128.2 (Ar), 128.1 (C-4), 122.6 (C-2), 61.1 (OMe), 32.2 (NMe); HRMS calcd for $C_{13}H_{15}NO_2Na$ [M + Na]$^+$ 240.0995, found 240.0987.

(2*Z*,4*Z*)-**18d**

R_f= 0.45 (silica gel, hexane/ethyl acetate = 2/1); FT-IR (neat) ν_{max}: 2962, 2931, 1643, 1450, 1435, 1381, 1095, 802. 702 cm^{-1}; ^{1}H NMR (500 MHz, C_6D_6): δ 8.19 (dd, J = 11.5, 11.5 Hz, 1H, H-4), 7.22–7.12 (m, 2H, Ar), 7.12–7.06 (m, 2H, Ar), 7.06–6.99 (m, 1H, Ar), 7.00 (dd, J = 11.5, 11.5 Hz, 1H, H-3), 6.63 (d, J = 11.5 Hz, 1H, H-5), 6.21 (d, J = 11.5 Hz, 1H, H-2), 3.03 (s, 3H, OMe), 2.93 (s, 3H, NMe); ^{13}C{^{1}H} NMR (125 MHz, C_6D_6): 167.2 (C-1), 138.1 (C-3), 137.1 (C-5), 136.7 (Ar), 129.8 (Ar), 128.4 (Ar), 127.7 (Ar), 127.2 (C-4), 118.4 (C-2), 60.9 (OMe), 31.9 (NMe); HRMS calcd for $C_{13}H_{15}NO_2Na$ [M + Na]$^+$ 240.0995, found 240.0995.

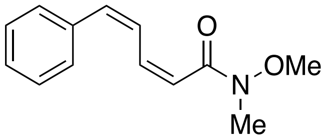

(2*Z*,4*Z*)-*N*-Methoxy-*N*-methyl-5-phenylpenta-2,4-dienamide ((2*Z*,4*Z*)-18d).

Reduction

To a solution of Weinreb amide (*Z*)-**20a** (38.0 mg, 0.199 mmol) in tetrahydrofuran (4.0 mL), a 1.0 M solution of diisobutylaluminum hydride in hexane (0.28 mL, 0.280 mmol) was added at –78 °C, and the reaction mixture was stirred at –78 °C for 50 min. Since Weinreb amide (*Z*)-**20a** remained, a 1.0 M solution of diisobutylaluminum hydride in hexane (0.06 mL, 0.060 mmol) was added to the reaction mixture. After the reaction mixture stirred at –78 °C for 30 min, methanol and saturated aqueous Rochelle salt were added, and the mixture was stirred at room temperature. The mixture was extracted with ethyl acetate, and the organic layer was dried over sodium sulfate. After filtration of the mixture and concentration of the solvent, (2*Z*)-3-phenylprop-2-enal (25.4 mg) was obtained as yellow oil and used in the next HWE reaction without further purification.

Z-selective HWE reaction

Ref. 4a

To a solution of phosphate **21** (137 mg, 0.396 mmol) and 1,4,7,10,13,16-hexaoxacyclooctadecane (188 mg, 0.713 mmol) in tetrahydrofuran (4.6 mL), a 1.0 M solution of potassium bis(trimethylsilyl)amide in tetrahydrofuran (0.36 mL, 0.356 mmol) was added at –78 °C. After the reaction mixture was stirred at –78 °C for 30 min, a solution of (2*Z*)-3-phenylprop-2-enal (25.4 mg) in tetrahydrofuran (2.0 mL) was added at –78 °C, and the reaction mixture was stirred for 3 h. To the reaction mixture, saturated aqueous ammonium chloride was added at –78 °C, and the mixture was extracted with ethyl acetate. The organic layer was dried over sodium sulfate. After filtration of the mixture and concentration of the solvent, the crude mixture was purified by thin layer chromatography on silica (eluant; hexane/ethyl acetate = 1/1) to afford diene (2*Z*,4*Z*)-**18d** (32.7 mg, 76% over 2 steps) as pale yellow oil and diene (2*E*,4*Z*)-**18d** (4.3 mg, 10% over 2 steps) as pale yellow oil.

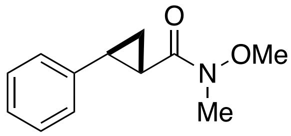

(1*SR*,2*SR*)-*N*-Methoxy-*N*-methyl-2-phenylcyclopropane-1-carboxamide (*trans*-22).

To a solution of trimethylsulfoxonium iodide (90.6 mg, 0.400 mmol) in dimethyl sulfoxide (1.0 mL), sodium hydride (55% dispersion in mineral oil) (17.5 mg, 0.400 mmol) was added at 0 °C. After the mixture was stirred at 0 °C for 30 min, a solution of alkene (*E*)-**20a** (38.5 mg, 201 mmol) in dimethyl sulfoxide (1.0 mL) was added to the mixture at 0 °C. The reaction mixture was stirred at room temperature for 4 h. To the reaction mixture, saturated aqueous ammonium chloride was added at 0 °C, and the mixture was extracted with ethyl acetate. The organic layer was dried over sodium sulfate. After filtration of the mixture and concentration of the solvent, the crude mixture was purified by thin layer chromatography on silica (eluant; hexane/ethyl acetate = 1/1) to afford cyclopropane *trans*-**22** (35.3 mg, 86%) as white solid and alkene (*E*)-**20a** (0.7 mg, 1.8%).

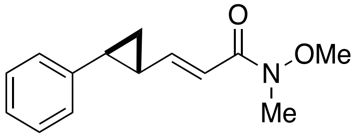

(2*E*)-*N*-Methoxy-*N*-methyl-3-((1*RS*,2*SR*)-2-phenylcyclopropyl)prop-2-enamide (*trans*-(*E*)-16r).

Reduction

To a solution of Weinreb amide *trans*-**22** (34.3 mg, 0.167 mmol) in tetrahydrofuran (1.7 mL), a 1.0 M solution of diisobutylaluminum hydride in hexane (0.22 mL, 0.217 mmol) was added at –78 °C. After the reaction mixture was stirred at –78 °C for 15 min, methanol and saturated aqueous Rochelle salt were added, and the mixture was stirred at room temperature. The mixture was extracted with ethyl acetate, and the organic layer was dried over sodium sulfate. After filtration of the mixture and concentration of the solvent, aldehyde **15r** (25.1 mg) was obtained as colorless oil and used in the next HWE reaction without further purification.

HWE reaction

To a solution of phosphate **1** (79.9 mg, 0.334 mmol) in tetrahydrofuran (4.0 mL), a 2.0 M solution of isopropylmagnesium chloride in tetrahydrofuran (0.15 mL, 0.300 mmol) was added at –78 °C. After the reaction mixture was stirred at –78 °C for 30 min, a solution of aldehyde **15r** (25.1 mg) in tetrahydrofuran (1.6 mL) was added at room temperature, and the reaction mixture was stirred for 3 h. To the reaction mixture, saturated aqueous ammonium chloride was added at 0 °C, and the mixture was extracted with ethyl acetate. The organic layer was dried over sodium sulfate. After filtration of the mixture and concentration of the solvent, the crude mixture was purified by thin layer chromatography on silica (eluant; hexane/ethyl acetate = 1/1) to afford alkene (*E*)-**16r** (29.2 mg, 76% over 2 steps) as colorless oil, alkene (*Z*)-**16r** (<0.7 mg, <1.8% over 2 steps) as colorless oil and aldehyde **15r** (2.2 mg, 9.0% over 2 steps).

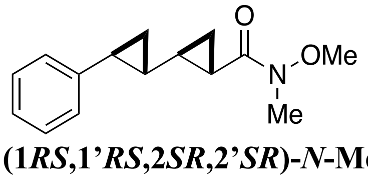

(1*RS*,1’*RS*,2*SR*,2’*SR*)-*N*-Methoxy-*N*-methyl-2’-phenyl-[1,1’-bi(cyclopropane)]-2-carboxamide (*trans-syn-trans*-23).

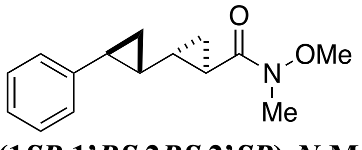

(1*SR*,1’*RS*,2*RS*,2’*SR*)-*N*-Methoxy-*N*-methyl-2’-phenyl-[1,1’-bi(cyclopropane)]-2-carboxamide (*trans-anti-trans*-23).

To a solution of trimethylsulfoxonium iodide (55.9 mg, 0.254 mmol) in dimethyl sulfoxide (1.5 mL), sodium hydride (55% dispersion in mineral oil) (11.1 mg, 0.254 mmol) was added at 0 °C. After the mixture was stirred at 0 °C for 30 min, a solution of alkene (*E*)-**16r** (29.4 mg, 0.127 mmol) in dimethyl sulfoxide (1.0 mL) was added to the mixture at 0 °C. The reaction mixture was stirred at room temperature for 4 h. To the reaction mixture, saturated aqueous ammonium chloride was added at 0 °C, and the mixture was extracted with ethyl acetate. The organic layer was dried over sodium sulfate. After filtration of the mixture and concentration of the solvent, the crude mixture was purified by thin layer chromatography on silica (eluant; hexane/ethyl acetate = 1/1) to afford cyclopropane **23** (20.9 mg, 67%) as colorless oil and alkene (*E*)-**16r** (2.5 mg, 8.5%).

Cyclopropane **23** was the mixture of stereoisomers (*trans*-*syn*-*trans*-**23** and *trans*-*anti*-*trans*-**23**), and the ratio between stereoisomers was 1:1. Therefore, the below described physical property data of cyclopropane **23** was equal mixture of stereoisomers.

The mixture of *trans*-*syn*-*trans*-**23** and *trans*-*anti*-*trans*-**23**

R_f = 0.33 (silica gel, hexane/ethyl acetate = 2/1); ATR-IR ν_{max}: 3003, 1654, 1498, 1464, 1422, 1390, 700 cm^{-1};

trans-*syn*-*trans*-**23** or *trans*-*anti*-*trans*-**23**

^{1}H NMR (500 MHz, $CDCl_3$): δ 7.29–7.20 (m, 2H, Ar), 7.17–7.11 (m, 1H, Ar), 7.07–7.00 (m, 2H, Ar), 3.76 (s, 3H, OMe), 3.209 (s, 3H, NMe), 2.15–1.95 (m, 1H, H-2), 1.82–1.71 (m, 1H, H-2'), 1.58–1.44 (m, 1H, H-1), 1.24–1.05 (m, 2H, H-3, H-1'), 0.97–0.73 (m, 3H, H-3, H-3'); ^{13}C{^{1}H} NMR (125 MHz, $CDCl_3$): 173.92 (CO), 142.9 (Ar), 128.28 (Ar), 125.7 (Ar), 125.48 (Ar), 61.6 (OMe), 32.6 (NMe), 24.30 (C-1'), 24.2 (C-1), 22.5 (C-2'), 16.9 (C-2), 14.7 (C-3'), 13.73 (C-3);

trans-*syn*-*trans*-**23** or *trans*-*anti*-*trans*-**23**

^{1}H NMR (500 MHz, $CDCl_3$): δ 7.29–7.20 (m, 2H, Ar), 7.17–7.11 (m, 1H, Ar), 7.07–7.00 (m, 2H, Ar), 3.75 (s, 3H, OMe), 3.209 (s, 3H, NMe), 2.15–1.95 (m, 1H, H-2), 1.82–1.71 (m, 1H, H-2'), 1.58–1.44 (m, 1H, H-1), 1.24–1.05 (m, 2H, H-3, H-1'), 0.97–0.73 (m, 3H, H-3, H-3'); ^{13}C{^{1}H} NMR (125 MHz, $CDCl_3$): 173.87 (CO), 142.8 (Ar), 128.27 (Ar), 125.6 (Ar), 125.46 (Ar), 61.5 (OMe), 32.5 (NMe), 24.29 (C-1'), 24.1 (C-1), 21.8 (C-2'), 16.5 (C-2), 13.74 (C-3'), 12.9 (C-3);

The mixture of *trans*-*syn*-*trans*-**23** and *trans*-*anti*-*trans*-**23**

HRMS calcd for $C_{15}H_{19}NO_2Na$ $[M + Na]^+$ 268.1308, found 268.1295.

And then, we compared the *E*-selective HWE reagent with the *Z*-selective HWE reagent under *E*-selective HWE reaction conditions and *Z*-selective reaction conditions respectively.

Table S19. Comparison between *E*-Selective HWE Reagent and *Z*-Selective HWE Reagent

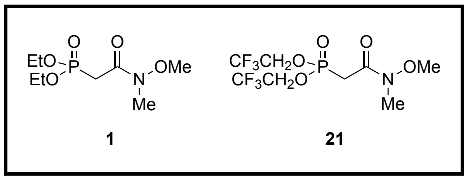

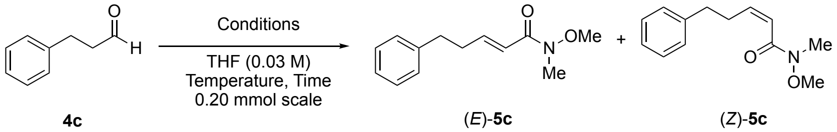

Entry	Conditions	Temperature	Time	Yield (%) (*E*)-**5c**	(*Z*)-**5c**	*E*/*Z*
1	HWE reagent **1** (2.0 equiv)<br>^{i}PrMgCl (1.8 equiv)	rt	1.5 h	94	0.9	99/ 1
2	HWE reagent **1** (1.2 equiv)<br>KHMDS (1.1 equiv)<br>18-Crown-6 (5.5 equiv)	–78 °C	20 min	36	48	43/57
3	HWE reagent **21** (2.0 equiv)<br>^{i}PrMgCl (1.8 equiv)	rt	1.5 h	17	70	19/81
4	HWE reagent **21** (1.2 equiv)<br>KHMDS (1.1 equiv)<br>18-Crown-6 (5.5 equiv)	–78 °C	20 min	6.0	61	9/91

Application of the HWE Reaction to a Ketone and Weinreb Ketone Syntheses

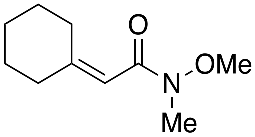

2-Cyclohexylidene-*N*-methoxy-*N*-methylacetamide (25).

To a solution of phosphate **1** (95.7 mg, 0.400 mmol) in tetrahydrofuran (4.7 mL), a 2.0 M solution of isopropylmagnesium chloride in tetrahydrofuran (0.18 mL, 0.360 mmol) was added at –78 °C. After the reaction mixture was stirred at –78 °C for 30 min, a solution of cyclohexanone **24** (19.5 mg, 0.199 mmol) in tetrahydrofuran (2.0 mL) was added at room temperature, and the reaction mixture was stirred for 24 h. To the reaction mixture, saturated aqueous ammonium chloride was added at 0 °C, and the mixture was extracted with ethyl acetate. The organic layer was dried over sodium sulfate. After filtration of the mixture and concentration of the solvent, the crude mixture was purified by thin layer chromatography on silica (eluant; hexane/ethyl acetate = 3/1) to afford alkene **25** (33.5 mg, 92%) as colorless oil.

R_f= 0.52 (silica gel, hexane/ethyl acetate = 2/1); FT-IR (neat) ν_{max}: 2931, 2854, 1651, 1450, 1412, 1389, 1342, 1180, 1011, 987 cm^{-1}; ^{1}H NMR (500 MHz, $CDCl_3$): δ 6.02 (s, 1H, H-2), 3.68 (s, 3H, OMe), 3.20 (s, 3H, NMe), 2.76 (t, J = 5.5 Hz, 2H, CH_2(CH_2)$_3$$CH_2$ or CH_2(CH_2)$_3$CH_2), 2.22 (t, J = 5.5 Hz, 2H, CH_2(CH_2)$_3$$CH_2$ or CH_2(CH_2)$_3$CH_2), 1.72–1.53 (m, 6H, CH_2CH_2CH_2C$H_2$$CH_2$); ^{13}C{^{1}H} NMR (125 MHz, $CDCl_3$): 168.1 (C-1), 159.9 (C-3), 111.5 (C-2), 61.3 (OMe), 38.2 (*C*H_2(CH_2)$_3$$CH_2$ or CH_2(CH_2)$_3$*C*H_2), 32.1 (NMe), 30.0 (*C*H_2(CH_2)$_3$$CH_2$ or CH_2(CH_2)$_3$*C*H_2), 28.7 (CH_2*C*H_2(CH_2)$_3$ or (CH_2)$_3$*C*$H_2$$CH_2$), 27.8 ($CH_2$*C*$H_2$($CH_2$)$_3$ or (CH_2)$_3$*C*$H_2$$CH_2$), 26.3 (($CH_2$)$_2$*C*$H_2$($CH_2$)$_2$); HRMS calcd for $C_{20}H_{34}N_2O_4Na$ [2M + Na]$^+$ 389.2411, found 389.2420.

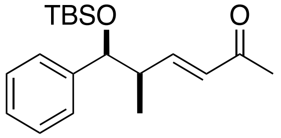

(5*RS*,6*SR*,3*E*)-6-((*tert*-Butyldimethylsilyl)oxy)-5-methyl-6-phenylhex-3-en-2-one (*syn*-26).

To a solution of Weinreb amide *syn*-**16p** ((*E*)-**16p**) (36.4 mg, 0.100 mmol) in tetrahydrofuran (2.0 mL), a 1.04 M solution of methylmagnesium bromide in tetrahydrofuran (0.19 mL, 0.200 mmol) was added at –78 °C. After the rection mixture was stirred at –78 °C for 10 min, the reaction mixture was stirred at 0 °C for 30 min. To the reaction mixture, saturated aqueous ammonium chloride was added at 0 °C, and the mixture was extracted with ethyl acetate. The organic layer was dried over sodium sulfate. After filtration of the mixture and concentration of the solvent, the crude mixture was purified by thin layer chromatography on silica (eluant; hexane/ethyl acetate = 6/1) to afford ketone *syn*-**26** (30.0 mg, 94%) as colorless oil.

R_f= 0.96 (silica gel, hexane/ethyl acetate = 2/1); FT-IR (neat) ν_{max}: 2954, 2931, 2885, 2854, 1674, 1365, 1257, 1088, 1065, 864, 841, 779, 702 cm^{-1}; ^1H NMR (500 MHz, $CDCl_3$): δ 7.34–7.19 (m, 5H, Ar), 6.73 (dd, J = 16.0, 7.5 Hz, 1H, H-3), 5.96 (dd, J = 16.0, 1.0 Hz, 1H, H-2), 4.61 (dd, J = 6.5, 0.5 Hz, 1H, H-5), 2.60 (dqdd, J = 7.5, 7.0, 6.5, 1.0 Hz, 1H, H-4), 2.19 (s, 3H, COCH_3), 1.02 (d, J = 7.0 Hz, 3H, 4-Me), 0.90 (s, 9H, TBS), 0.02 (s, 3H, TBS), –0.20 (s, 3H, TBS); $^{13}C\{^1H\}$ NMR (125 MHz, $CDCl_3$): 198.7 (C-1), 150.6 (C-3), 142.7 (Ar), 131.0 (C-2), 127.9 (Ar), 127.3 (Ar), 126.4 (Ar), 77.8 (C-5), 45.4 (C-4), 26.6 (CO*C*H_3), 25.8 (TBS), 18.2 (TBS), 13.9 (4-Me), –4.6 (TBS), –5.2 (TBS); HRMS calcd for $C_{19}H_{30}O_2SiNa$ $[M + Na]^+$ 341.1907, found 341.1905.

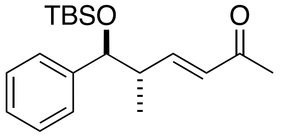

(5*SR*,6*SR*,3*E*)-6-((*tert*-Butyldimethylsilyl)oxy)-5-methyl-6-phenylhex-3-en-2-one (*anti*-26).

To a solution of Weinreb amide *anti*-**16q** ((*E*)-**16q**) (36.5 mg, 0.100 mmol) in tetrahydrofuran (2.0 mL), a 1.04 M solution of methylmagnesium bromide in tetrahydrofuran (0.19 mL, 0.200 mmol) was added at –78 °C. After the rection mixture was stirred at –78 °C for 10 min, the reaction mixture was stirred at 0 °C for 30 min. To the reaction mixture, saturated aqueous ammonium chloride was added at 0 °C, and the mixture was extracted with ethyl acetate. The organic layer was dried over sodium sulfate. After filtration of the mixture and concentration of the solvent, the crude mixture was purified by thin layer chromatography on silica (eluant; hexane/ethyl acetate = 6/1) to afford ketone *anti*-**26** (28.1 mg, 88%) as colorless oil.

R_f= 0.94 (silica gel, hexane/ethyl acetate = 2/1); FT-IR (neat) ν_{max}: 2954, 2931, 2893, 2854, 1674, 1365, 1257, 1088, 1065, 841, 779, 702 cm^{-1}; ^1H NMR (500 MHz, $CDCl_3$): δ 7.34–7.21 (m, 5H, Ar), 6.86 (dd, J = 16.0, 8.0 Hz, 1H, H-3), 5.97 (dd, J = 16.0, 1.0 Hz, 1H, H-2), 4.48 (d, J = 6.0 Hz, 1H, H-5), 2.59 (dqdd, J = 8.0, 7.0, 6.0, 1.0 Hz, 1H, H-4), 2.23 (s, 3H, COCH_3), 0.94 (d, J = 7.0 Hz, 3H, 4-Me), 0.87 (s, 9H, TBS), 0.01 (s, 3H, TBS), –0.24 (s, 3H, TBS); $^{13}C\{^1H\}$ NMR (125 MHz, $CDCl_3$): 198.8 (C-1), 150.6 (C-3), 143.0 (Ar), 131.3 (C-2), 128.0 (Ar), 127.4 (Ar), 126.6 (Ar), 78.8 (C-5), 45.6 (C-4), 26.0 (CO*C*H_3), 25.7 (TBS), 18.1 (TBS), 16.0 (4-Me), –4.6 (TBS), –5.1 (TBS); HRMS calcd for $C_{19}H_{30}O_2SiNa$ $[M + Na]^+$ 341.1907, found 341.1908.

References.

(1) Krasovskiy, A.; Knochel, P. Convenient Titration Method for Organometallic Zinc, Magnesium, and Lanthanide Reagents. *Synthesis* **2006**, *5*, 890–891.

(2) a) Ashby, E. C.; Arnott, R. C. Concerning the purity of magnesium and beryllium alkyls and halides prepared by different methods. *J. Organometal. Chem.* **1968**, *14*, 1–11. b) Rathke, M. W.;

Nowak, M. The Horner–Wadsworth–Emmons Modification of the Wittig Reaction Using Triethylamine and Lithium or Magnesium Salts. *J. Org. Chem.* **1985**, *50*, 2624–2626.
(3) Brauer, G. *Handbook of Preparative Inorganic Chemistry*; Academic Press Inc., 1963.
(4) a) Still, W. C.; Gennari, C. Direct synthesis of Z-unsaturated esters. A useful modification of the horner–emmons olefination. *Tetrahedron Lett.* **1983**, *24*, 4405–4408. b) Gokel, G. W.; Cram, D. J. Preparation and Purification of 18-Crown-6. *J. Org. Chem.* **1974**, *39*, 2445–2446.
(5) Perrin, D. D.; Armarego, W. L. F. Purification of Laboratory Chemicals. *Pergamon Press* **1988**, *3*, 91.
(6) Corey, E. J.; Cho, H.; Rücker, C.; Hua, D. H. Studies with trialkylsilyltriflates: new syntheses and applications. *Tetrahedron Lett.* **1981**, *22*, 3455–3458.
(7) Roiser, L.; Waser, M. Enantioselective Spirocyclopropanation of *para*-Quinone Methides Using Ammonium Ylides. *Org. Lett.* **2017**, *19*, 2338–2341.
(8) Fritz, J. E.; Kaldor, S. W.; Liang, S. X.; Singh, U.; Xu, Y.-C. New Serotonin 5-HT_{1F} Agonists. WO 9815545, 1998.
(9) Nuzillard, J.-M.; Boumendjel, A.; Massiot, G. A new synthesis of α,β-unsaturated aldehydes. *Tetrahedron Lett.* **1989**, *30*, 3779–3780.
(10) Messik, F.; Oberthür, M. An Expedient Access to Still–Gennari Phosphonates. *Synthesis* **2013**, *45*, 167–170.
(11) Fortin, S.; Dupont, F.; Deslongchamps, P. A New Bis(2,2,2-trifluoroethyl)phosphonate for the Synthesis of *Z*-Unsaturated *N*-Methoxy-*N*-methylamides. *J. Org. Chem.* **2002**, *67*, 5437–5439.
(12) Kojima, S.; Hidaka, T.; Yamakawa, A. Application of Organocerium Reagents for the Efficient Conversion of *Z*-α,β-Unsaturated Weinreb Amides to *Z*-α,β-Unsaturated Ketones. *Chem. Lett.* **2005**, *34*, 470–471.
(13) Kojima, S.; Hidaka, T.; Ohba, Y. Synthesis of *β*-Monosubstituted *α,β*-Unsaturated Amides with *Z*-Selectivity Using Diphenylphosphonoacetamides. *Heteroatom Chem.* **2004**, *15*, 515–523.
(14) Ando, K. Preparation of *Z-α,β*-Unsaturated Amides by Using Horner–Wadsworth–Emmons Reagents, (Diphenylphosphono)acetamides. *Synthesis* **2001**, *8*, 1272–1274.
(15) Friese, A.; Hell-Momeni, K.; Zündorf, I.; Winckler, T.; Dingermann, T.; Dannhardt, G. Synthesis and Biological Evaluation of Cycloalkylidene Carboxylic Acids as Novel Effectors of Ras/Raf Interaction. *J. Med. Chem.* **2002**, *45*, 1535–1542.
(16) Maeta, N.; Kamiya, H.; Okada, Y. Radical-Cation Vinylcyclopropane Rearrangements by TiO_2 Photocatalysis. *J. Org. Chem.* **2020**, *85*, 6551–6566.
(17) Dahiya, A.; Ali, W.; Alam, T.; Patel, B. K. A cascade synthesis of *S*-allyl benzoylcarbamothioates *via* Mumm-type rearrangement. *Org. Biomol. Chem.* **2018**, *16*, 7787–7791.
(18) Bouzide, A. Magnesium Bromide Mediated Highly Diastereoselective Heterogeneous Hydrogenation of Olefins. *Org. Lett.* **2002**, *4*, 1347–1350.
(19) Brown, J. M.; Cutting, I. Effective Kinetic Resolution in the Asymmetric Hydrogenation of α-(Hydroxyalkyl)acrylate Esters. *J. Chem. Soc. Chem. Commun.* **1985**, *9*, 578–579.
(20) Liu, C.; Achtenhagen, M.; Szostak, M. Chemoselective Ketone Synthesis by the Addition of Organometallics to *N*-Acylazetidines. *Org. Lett.* **2016**, *18*, 2375–2378.
(21) Song, J. I.; An, D. K. New Method for Synthesis of Aldehydes from Esters by Sodium Diisobutyl-*t*-butoxyaluminum Hydride. *Chem. Lett.* **2007**, *36*, 886–887.

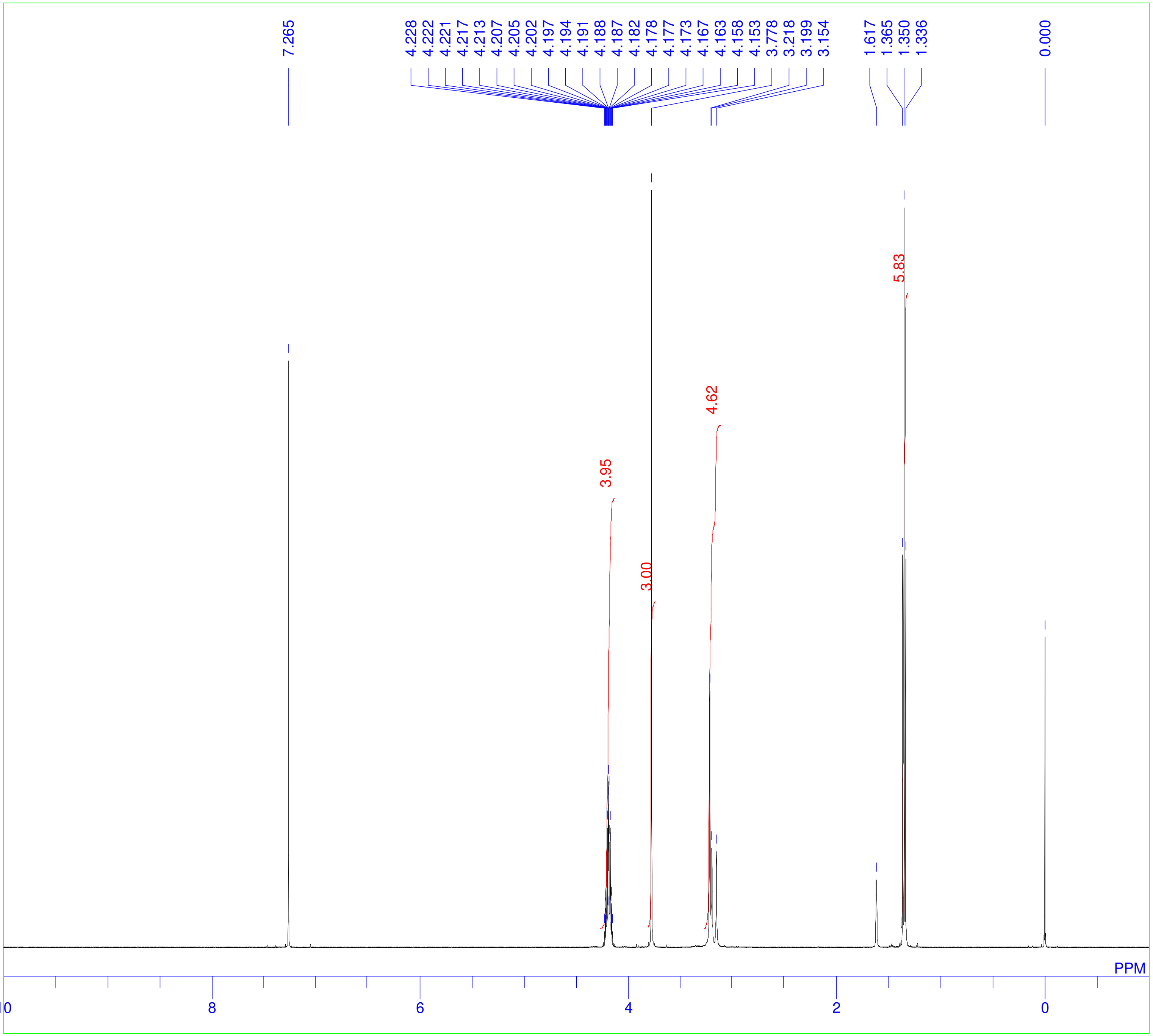

DFILE	1_1H.als
COMNT	
DATIM	2022-02-19 18:47:17
OBNUC	1H
EXMOD	proton.jxp
OBFRQ	500.16 MHz
OBSET	2.41 KHz
OBFIN	6.01 Hz
POINT	13107
FREQU	7507.51 Hz
SCANS	8
ACQTM	1.7459 sec
PD	5.0000 sec
PW1	3.84 usec
IRNUC	1H
CTEMP	19.2 c
SLVNT	CDCL3
EXREF	0.00 ppm
BF	0.30 Hz
RGAIN	44

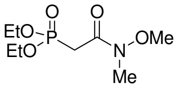

1

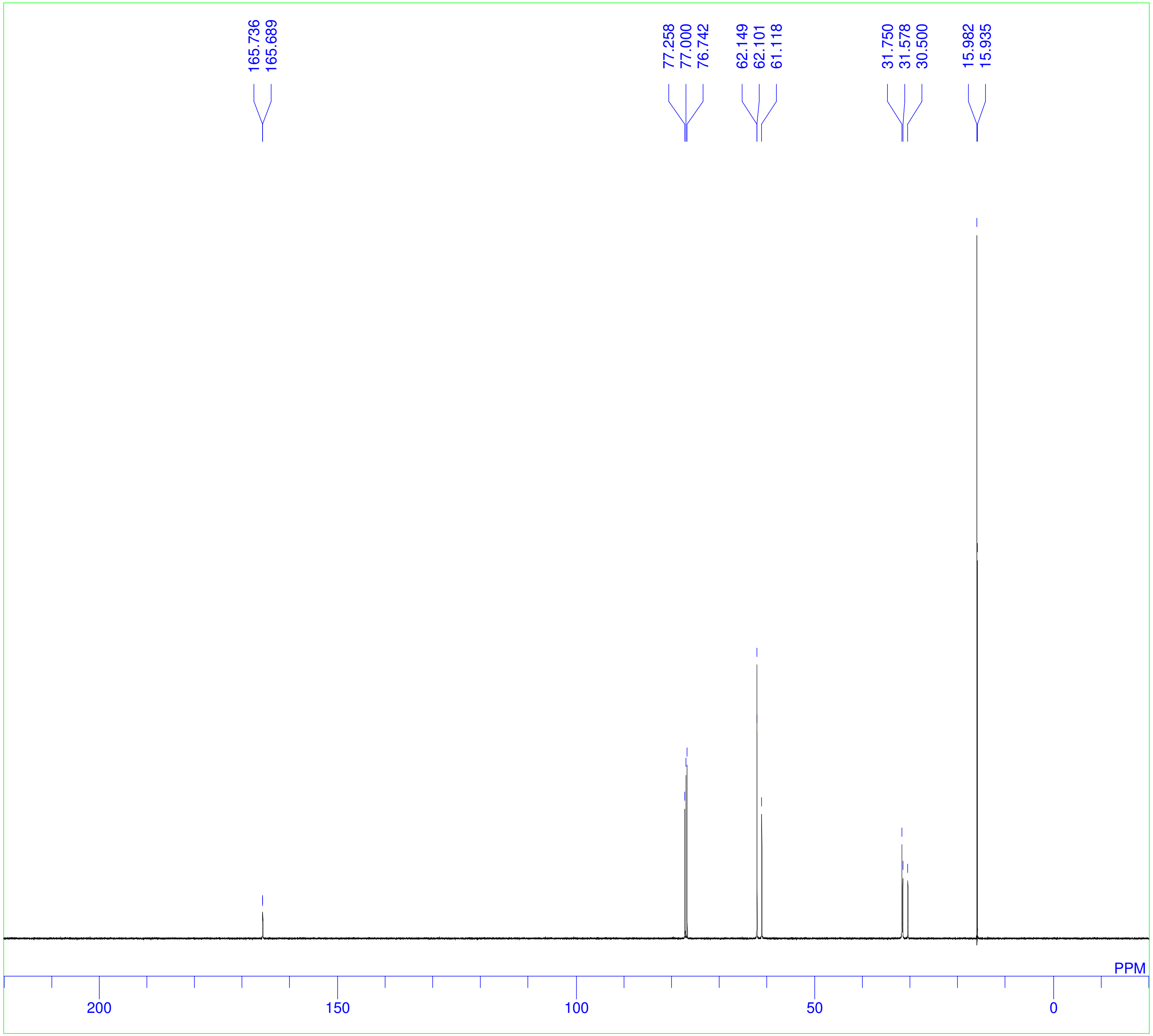

DFILE 1_13C.als
COMNT
DATIM 2022-12-21 19:01:50
OBNUC 13C
EXMOD carbon.jxp
OBFRQ 125.77 MHz
OBSET 7.87 KHz
OBFIN 4.21 Hz
POINT 26214
FREQU 31446.54 Hz
SCANS 1024
ACQTM 0.8336 sec
PD 2.0000 sec
PW1 3.87 usec
IRNUC 1H
CTEMP 23.2 c
SLVNT CDCL3
EXREF 77.00 ppm
BF 0.12 Hz
RGAIN 26

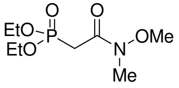

1

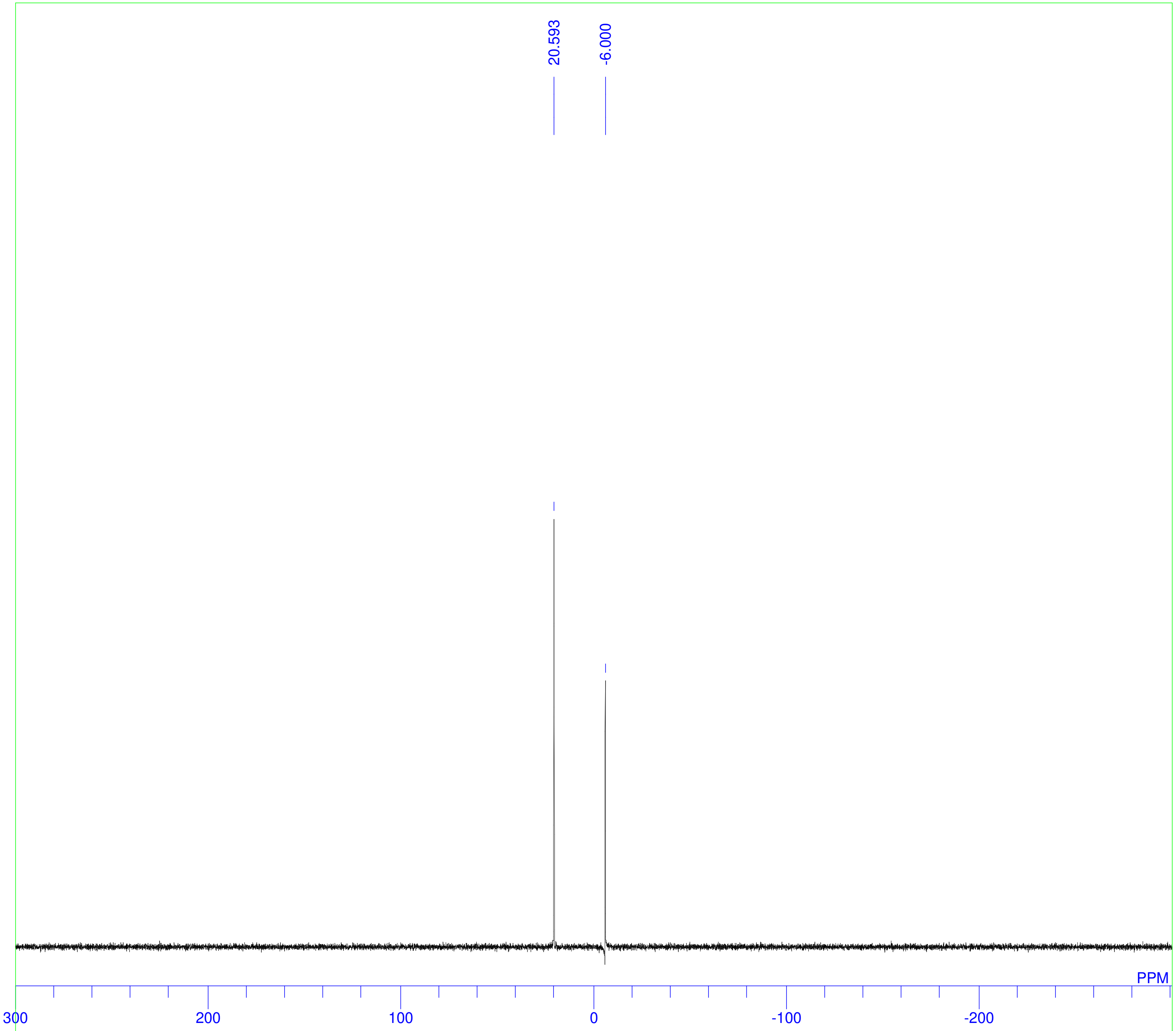

DFILE	1_31P.als
COMNT	
DATIM	2023-04-03 14:04:23
OBNUC	31P
EXMOD	single_pulse.jxp
OBFRQ	202.46 MHz
OBSET	8.31 KHz
OBFIN	0.75 Hz
POINT	13107
FREQU	142857.14 Hz
SCANS	64
ACQTM	0.0918 sec
PD	5.0000 sec
PW1	6.45 usec
IRNUC	31P
CTEMP	23.4 c
SLVNT	CDCL3
EXREF	-6.00 ppm
BF	0.25 Hz
RGAIN	50

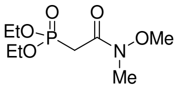

1

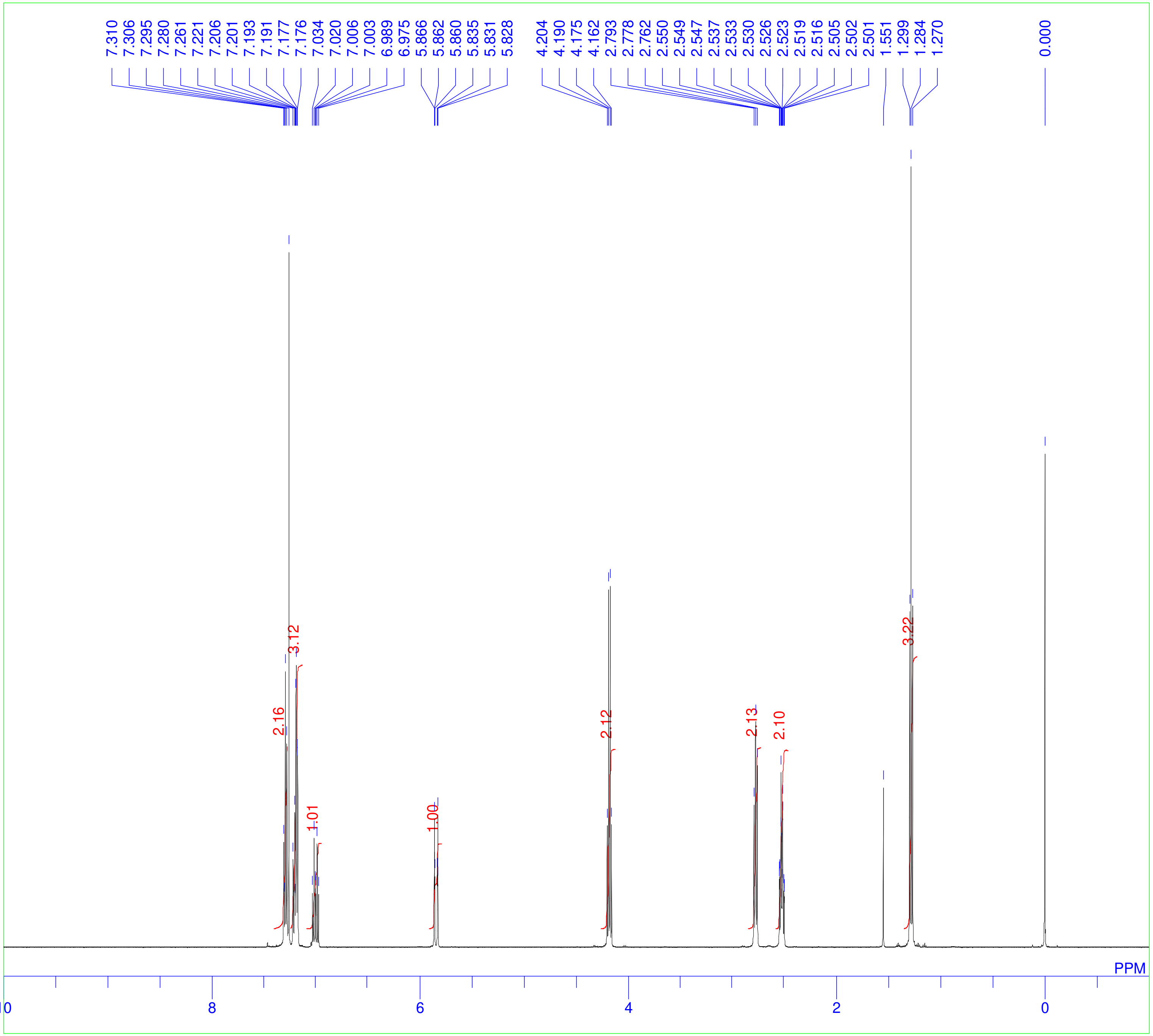

DFILE	(E)-11_1H.als
COMNT	
DATIM	2023-01-17 17:36:32
OBNUC	1H
EXMOD	proton.jxp
OBFRQ	500.16 MHz
OBSET	2.41 KHz
OBFIN	6.01 Hz
POINT	13107
FREQU	7507.51 Hz
SCANS	8
ACQTM	1.7459 sec
PD	5.0000 sec
PW1	3.84 usec
IRNUC	1H
CTEMP	21.2 c
SLVNT	CDCL3
EXREF	0.00 ppm
BF	0.30 Hz
RGAIN	44

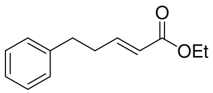

(*E*)-11

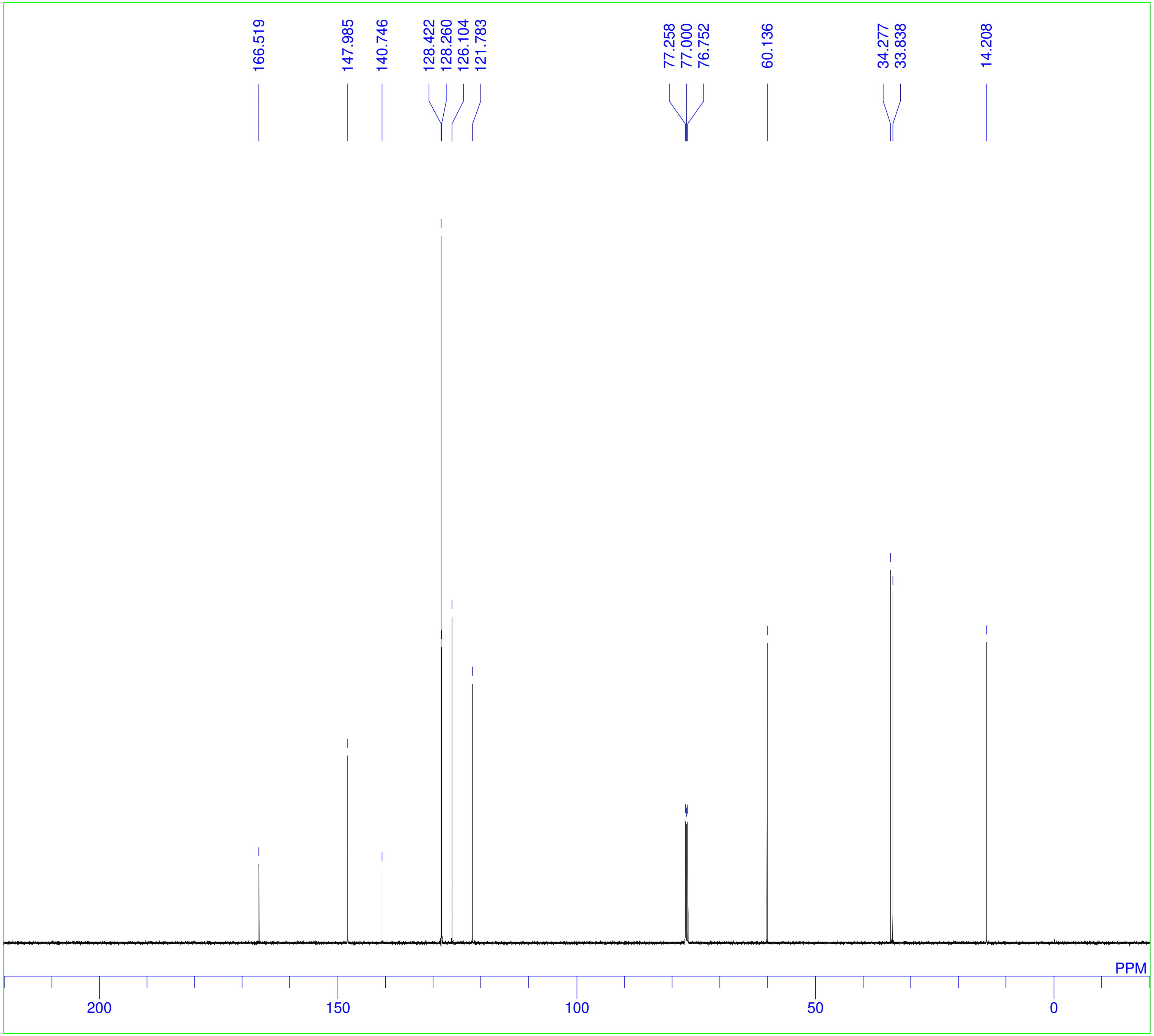

DFILE	(E)-11_13C.als
COMNT	
DATIM	2023-01-17 17:43:32
OBNUC	13C
EXMOD	carbon.jxp
OBFRQ	125.77 MHz
OBSET	7.87 KHz
OBFIN	4.21 Hz
POINT	26214
FREQU	31446.54 Hz
SCANS	1024
ACQTM	0.8336 sec
PD	2.0000 sec
PW1	3.87 usec
IRNUC	1H
CTEMP	21.4 c
SLVNT	CDCL3
EXREF	77.00 ppm
BF	0.30 Hz
RGAIN	24

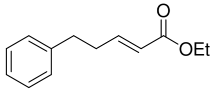

(*E*)-11

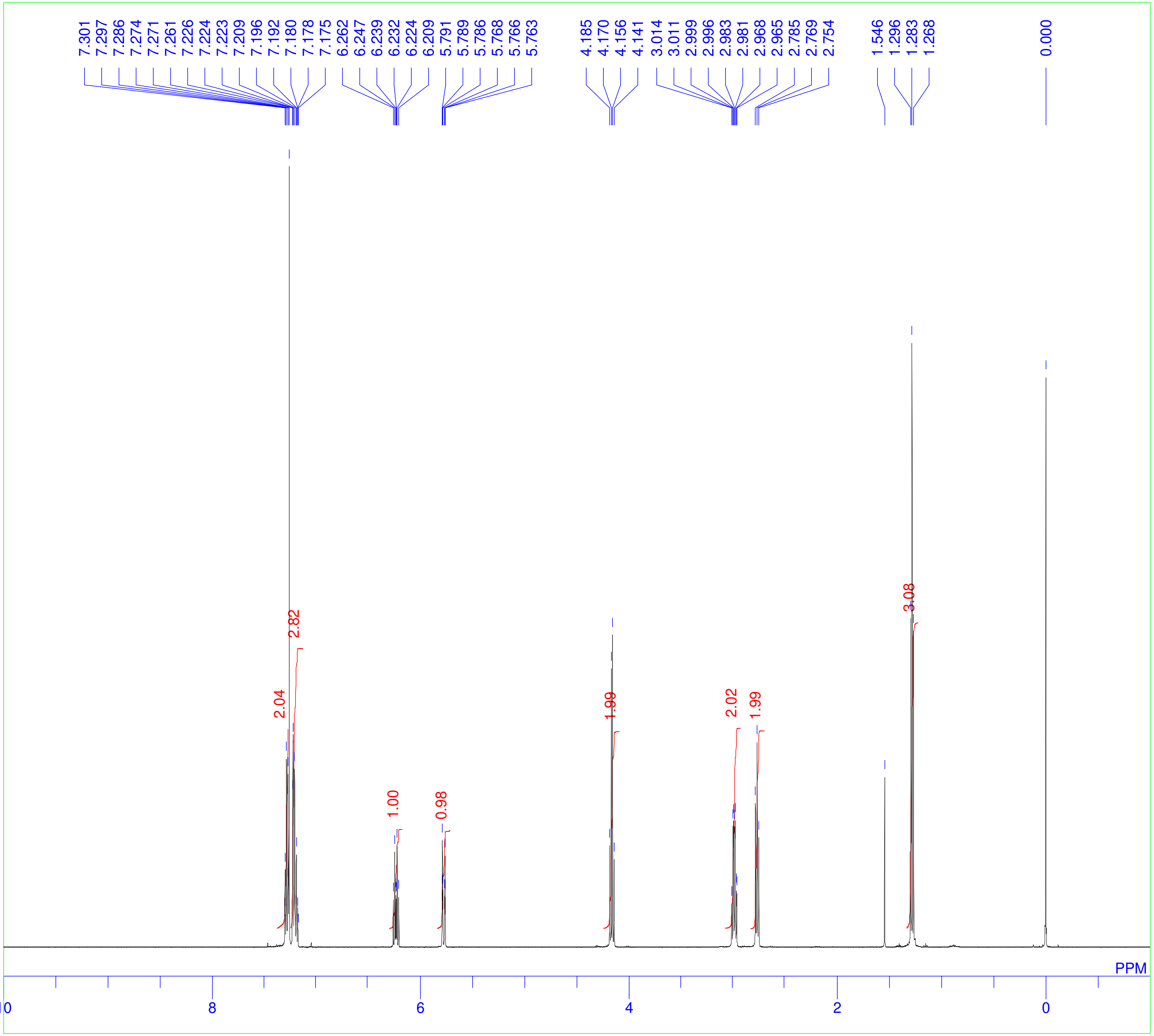

DFILE	(Z)-11_1H.als
COMNT	
DATIM	2023-01-17 17:29:27
OBNUC	1H
EXMOD	proton.jxp
OBFRQ	500.16 MHz
OBSET	2.41 KHz
OBFIN	6.01 Hz
POINT	13107
FREQU	7507.51 Hz
SCANS	8
ACQTM	1.7459 sec
PD	5.0000 sec
PW1	3.84 usec
IRNUC	1H
CTEMP	21.2 c
SLVNT	CDCL3
EXREF	0.00 ppm
BF	0.30 Hz
RGAIN	44

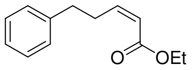

(*Z*)-11

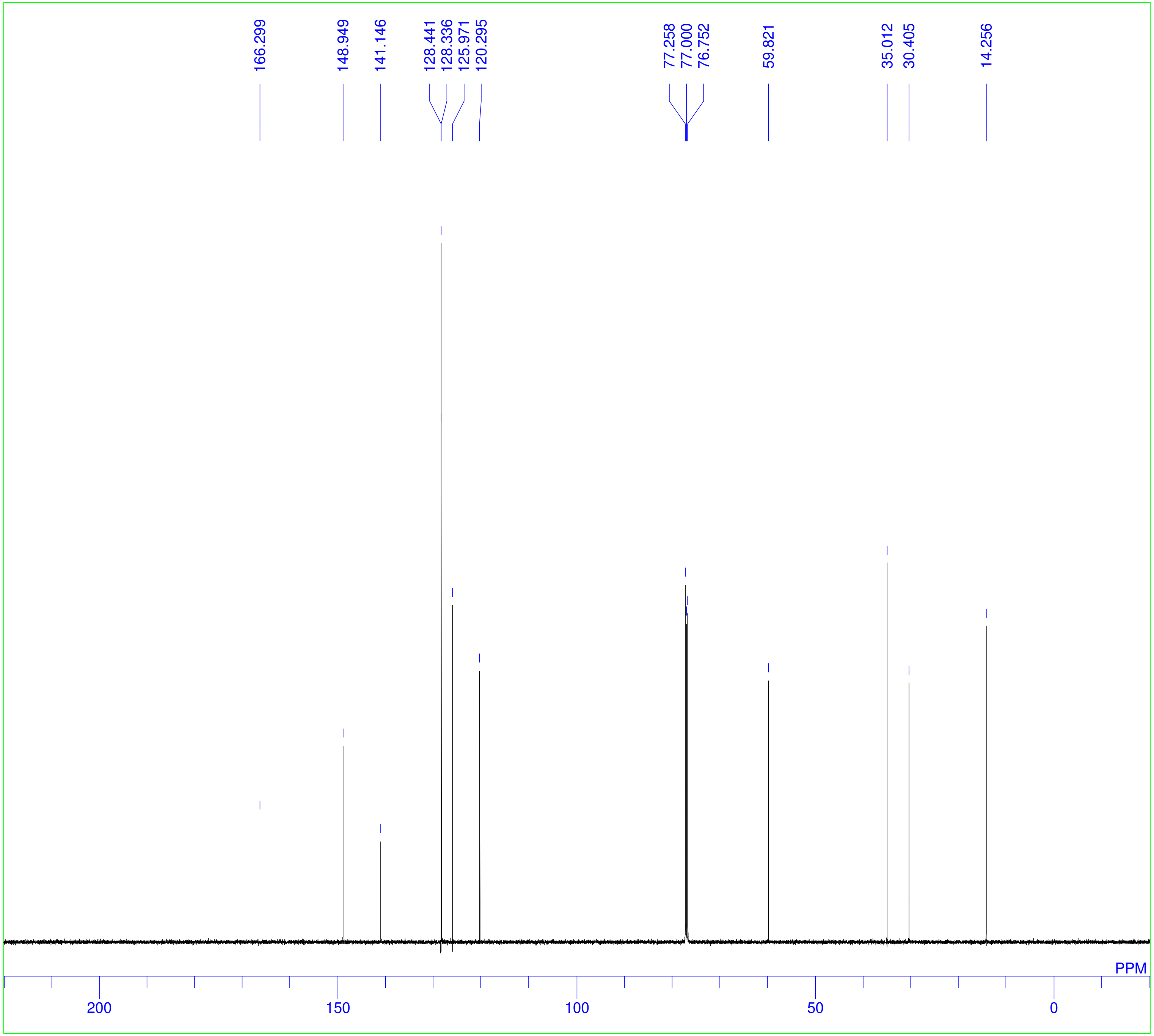

DFILE (Z)-11_13C.als
COMNT
DATIM 2023-01-17 18:37:30
OBNUC 13C
EXMOD carbon.jxp
OBFRQ 125.77 MHz
OBSET 7.87 KHz
OBFIN 4.21 Hz
POINT 26214
FREQU 31446.54 Hz
SCANS 1024
ACQTM 0.8336 sec
PD 2.0000 sec
PW1 3.87 usec
IRNUC 1H
CTEMP 21.3 c
SLVNT CDCL3
EXREF 77.00 ppm
BF 0.30 Hz
RGAIN 24

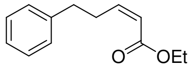

(*Z*)-11

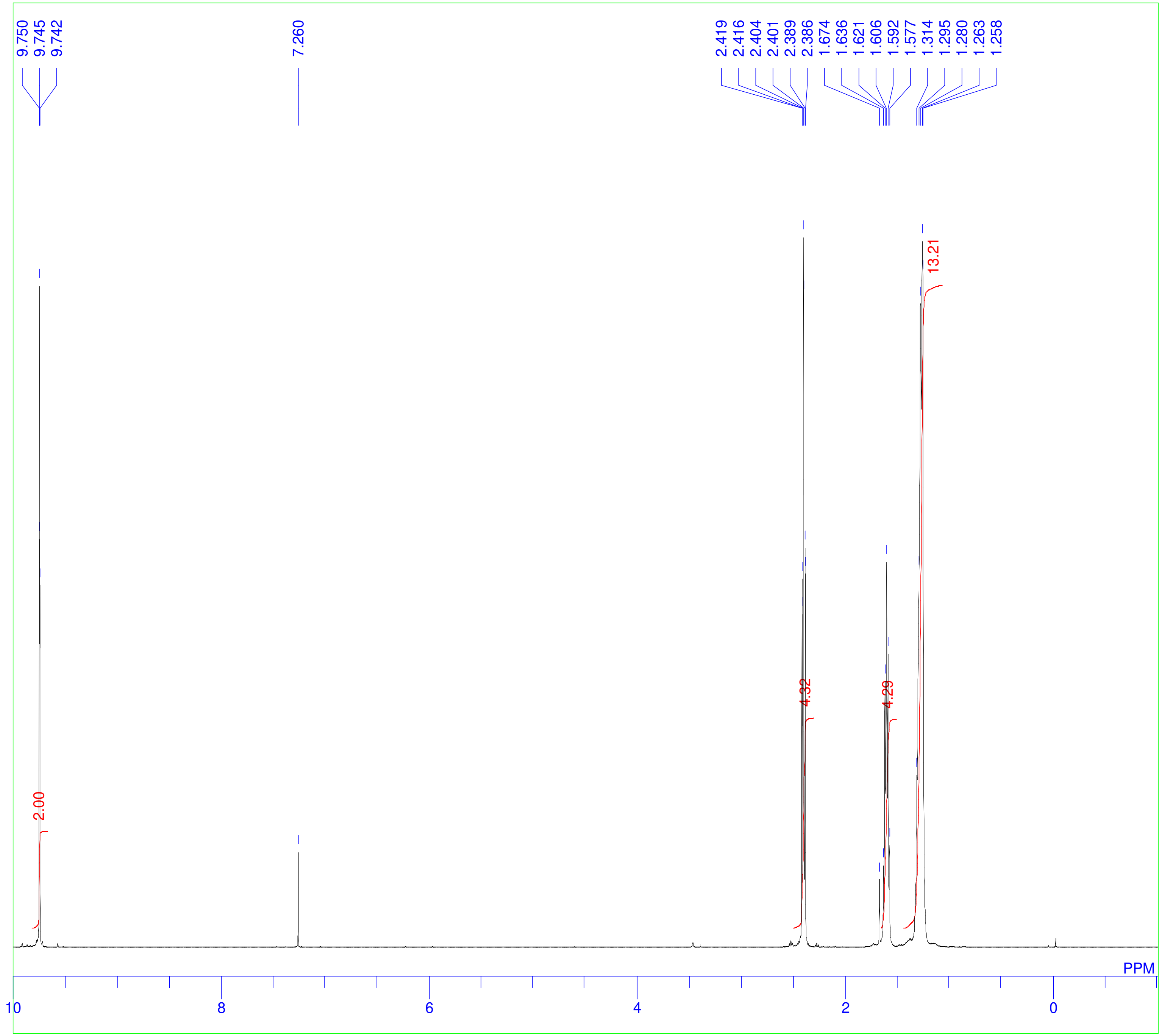

DFILE	15k_1H.als
COMNT	
DATIM	2023-05-22 18:48:15
OBNUC	1H
EXMOD	proton.jxp
OBFRQ	500.16 MHz
OBSET	2.41 KHz
OBFIN	6.01 Hz
POINT	13107
FREQU	7507.51 Hz
SCANS	8
ACQTM	1.7459 sec
PD	5.0000 sec
PW1	3.84 usec
IRNUC	1H
CTEMP	23.6 c
SLVNT	CDCL3
EXREF	7.26 ppm
BF	0.30 Hz
RGAIN	26

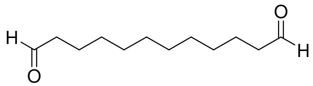

15k

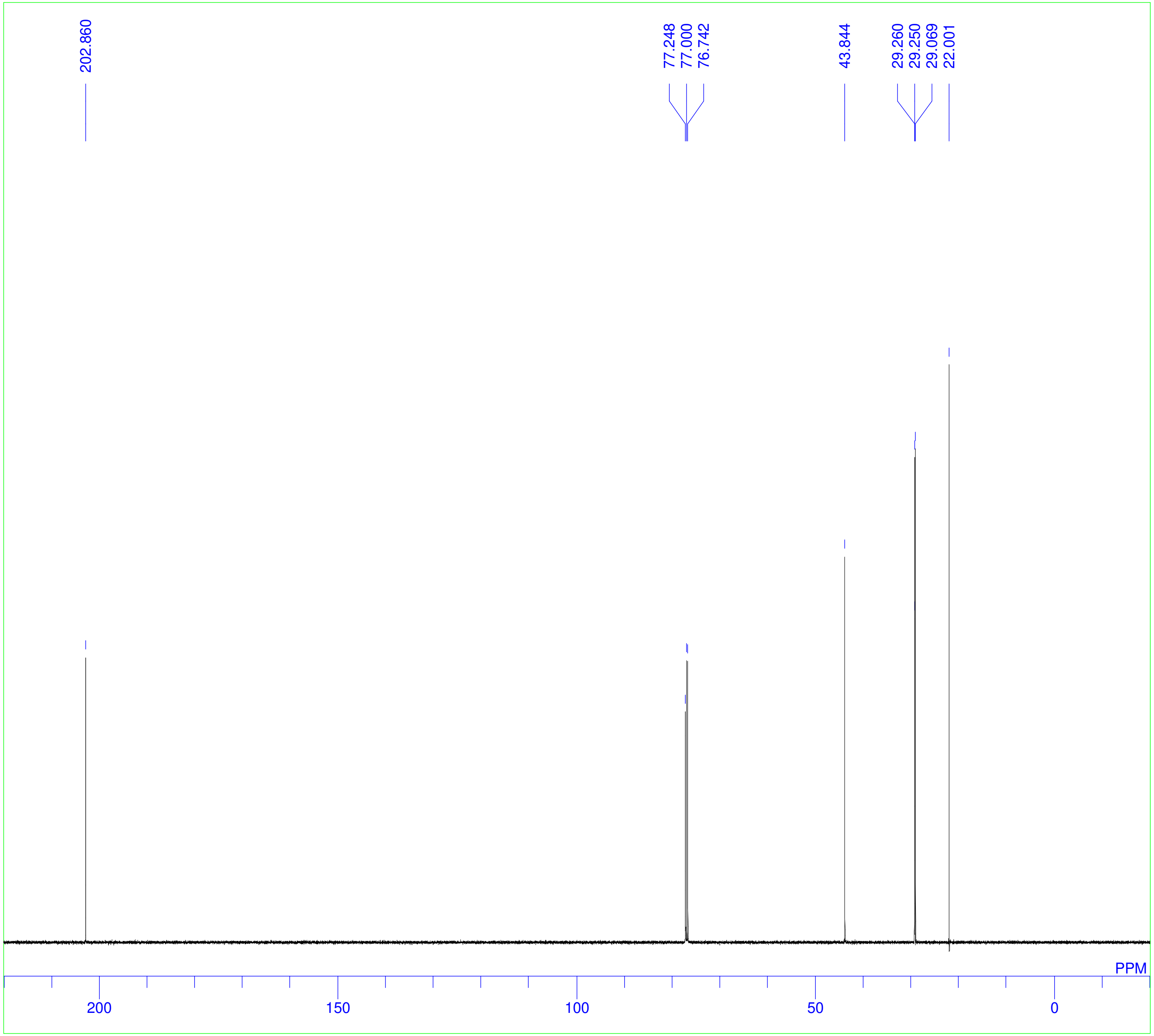

DFILE 15k_13C.als
COMNT
DATIM 2023-05-22 17:02:44
OBNUC 13C
EXMOD carbon.jxp
OBFRQ 125.77 MHz
OBSET 7.87 KHz
OBFIN 4.21 Hz
POINT 26214
FREQU 31446.54 Hz
SCANS 1024
ACQTM 0.8336 sec
PD 2.0000 sec
PW1 3.87 usec
IRNUC 1H
CTEMP 24.0 c
SLVNT CDCL3
EXREF 77.00 ppm
BF 0.30 Hz
RGAIN 30

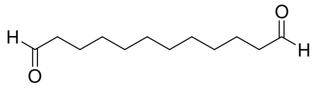

15k

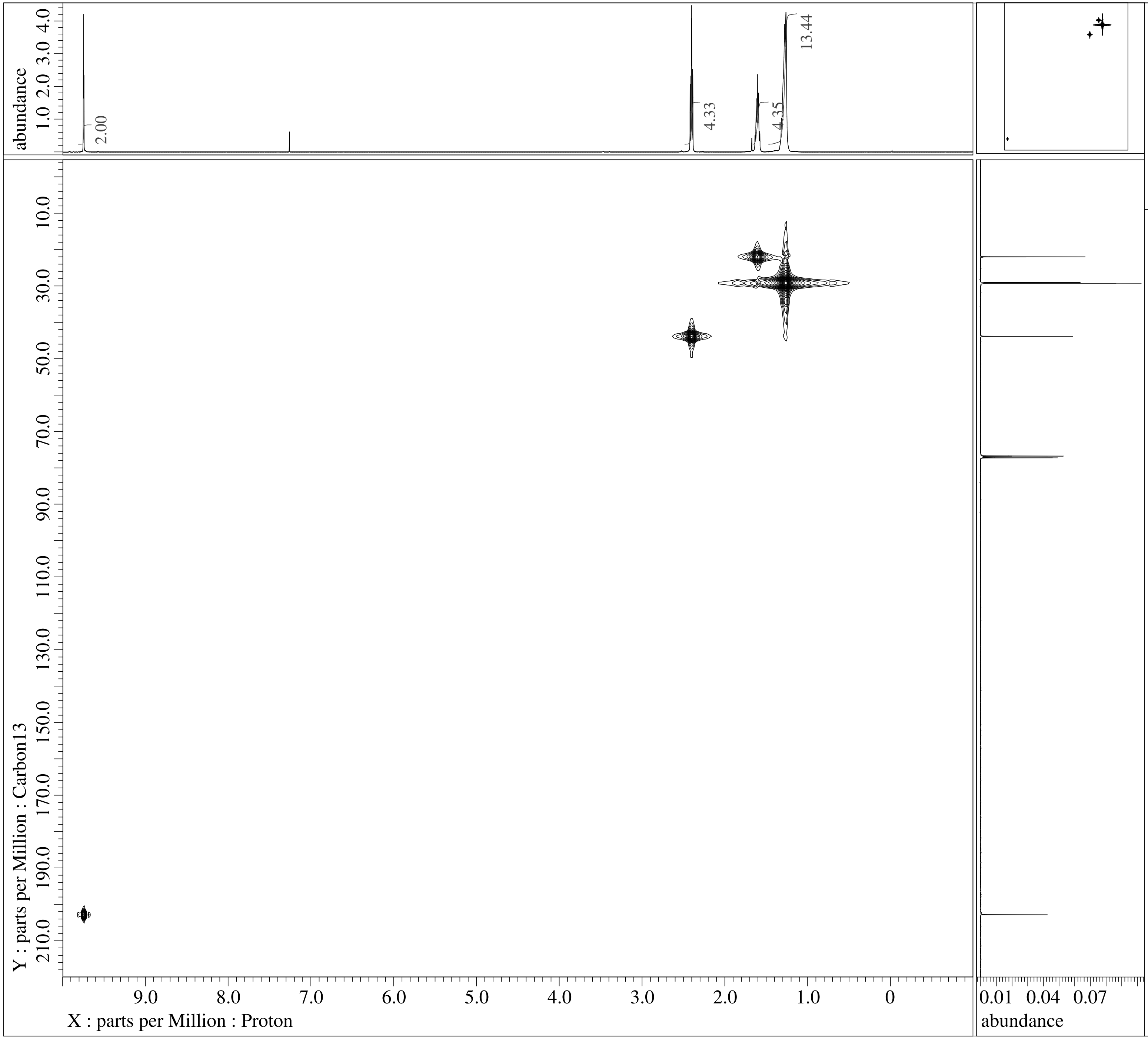


```
---- PROCESSING PARAMETERS ----
sinbell4( -60, 160 )
zerofill( 1 )
fft( 1, TRUE, TRUE )
ppm
[transpose]
sinbell4( -60, 160 )
zerofill( 2 )
fft( 1, TRUE, TRUE )
ppm
abs
```

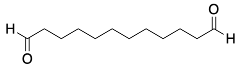

15k

```
Filename            = HT-1438-TM_13C_HMQC-1-5
Author              = delta
Experiment          = hmqc.jxp
Sample_Id           = HT-1438-TM_13C
Solvent             = CHLOROFORM-D
Creation_Time       = 22-MAY-2023 17:51:36
Revision_Time       = 10-APR-2024 18:45:30
Current_Time        = 10-APR-2024 18:47:04

Comment             = gradient enhanced HMQC
Data_Format         = 2D REAL REAL
Dim_Size            = 819, 512
Dim_Title           = Proton Carbon13
Dim_Units           = [ppm] [ppm]
Dimensions          = X Y
Site                = JNM-ECA500II
Spectrometer        = DELTA2_NMR

Field_Strength      = 11.7473579[T] (500[MHz]
X_Acq_Duration      = 0.10911744[s]
X_Domain            = 1H
X_Freq              = 500.15991521[MHz]
X_Offset            = 5.0[ppm]
X_Points            = 1024
X_Prescans          = 4
X_Resolution        = 9.16443788[Hz]
X_Sweep             = 9.38438438[kHz]
X_Sweep_Clipped     = 7.50750751[kHz]
Y_Domain            = 13C
Y_Freq              = 125.76529768[MHz]
Y_Offset            = 110.00570336[ppm]
Y_Points            = 256
Y_Prescans          = 0
Y_Resolution        = 113.02806713[Hz]
Y_Sweep             = 28.93518519[kHz]
Tri_Domain          = Proton
Tri_Freq            = 500.15991521[MHz]
Tri_Offset          = 5.0[ppm]
Clipped             = FALSE
Scans               = 8
Total_Scans         = 2048

Relaxation_Delay    = 1.5[s]
Recvr_Gain          = 50
Temp_Get            = 23.5[dC]
X_Acq_Time          = 0.10911744[s]
X_Atn               = 3.2[dB]
X_Gamma             = 42576375
X_Pulse             = 7.68[us]
```

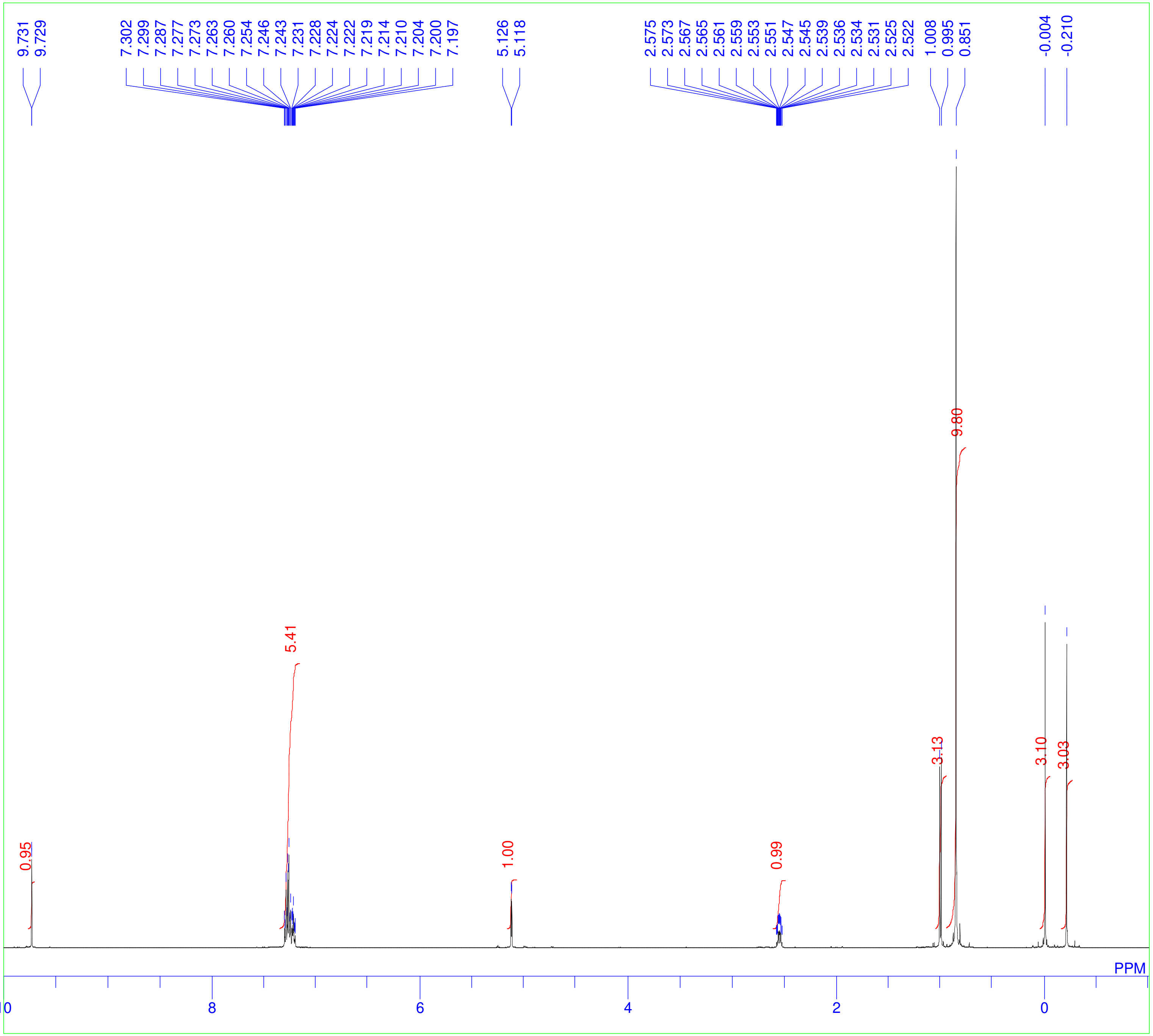

DFILE	15p_1H.als
COMNT	
DATIM	2023-01-05 10:37:24
OBNUC	1H
EXMOD	proton.jxp
OBFRQ	500.16 MHz
OBSET	2.41 KHz
OBFIN	6.01 Hz
POINT	13107
FREQU	7507.51 Hz
SCANS	8
ACQTM	1.7459 sec
PD	5.0000 sec
PW1	3.84 usec
IRNUC	1H
CTEMP	20.5 c
SLVNT	CDCL3
EXREF	7.26 ppm
BF	0.30 Hz
RGAIN	22

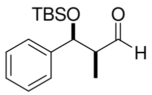

15p

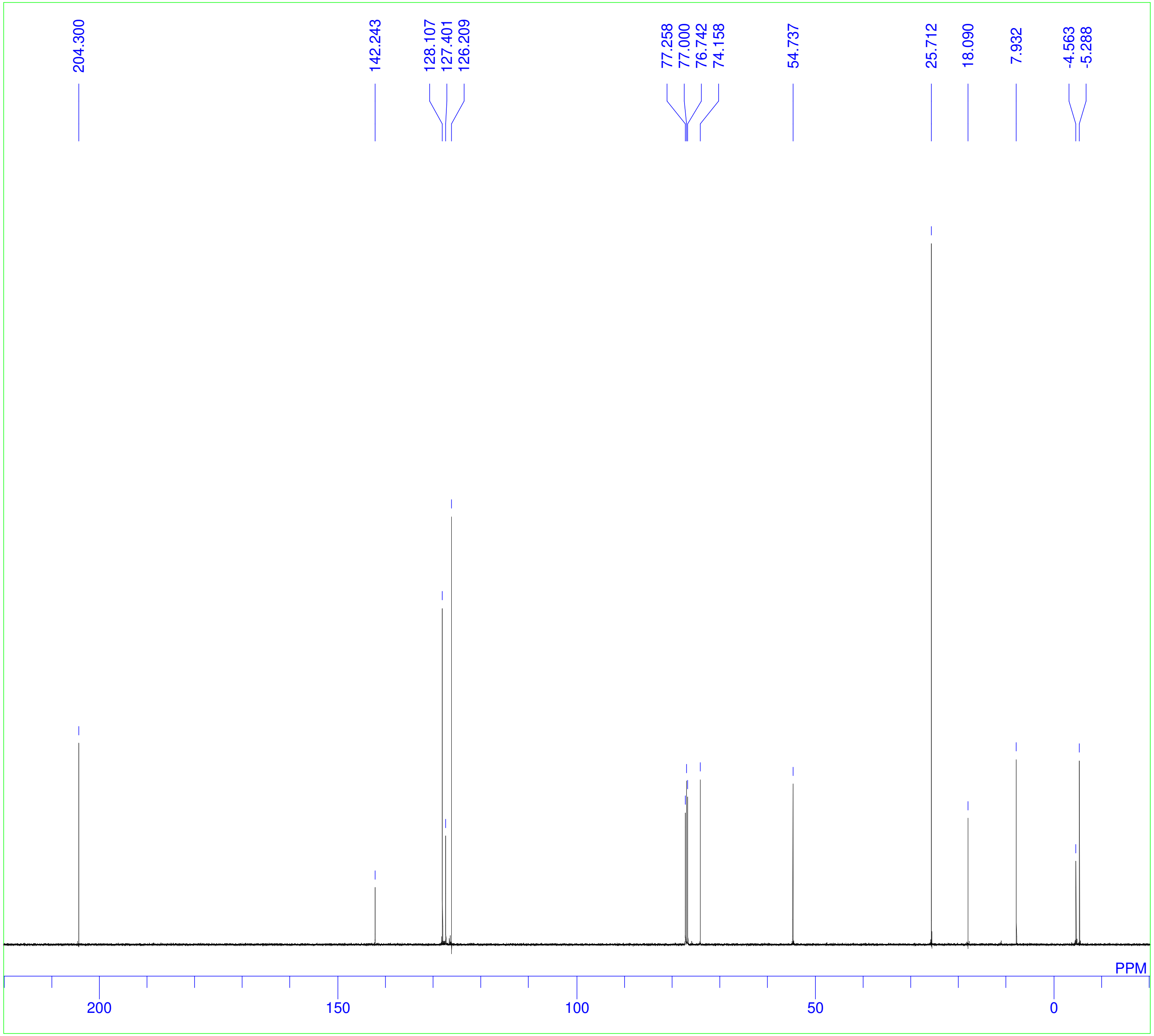

DFILE	15p_13C.als
COMNT	
DATIM	2023-01-05 10:39:50
OBNUC	13C
EXMOD	carbon.jxp
OBFRQ	125.77 MHz
OBSET	7.87 KHz
OBFIN	4.21 Hz
POINT	26214
FREQU	31446.54 Hz
SCANS	1024
ACQTM	0.8336 sec
PD	2.0000 sec
PW1	3.87 usec
IRNUC	1H
CTEMP	20.6 c
SLVNT	CDCL3
EXREF	77.00 ppm
BF	0.30 Hz
RGAIN	24

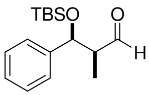

15p

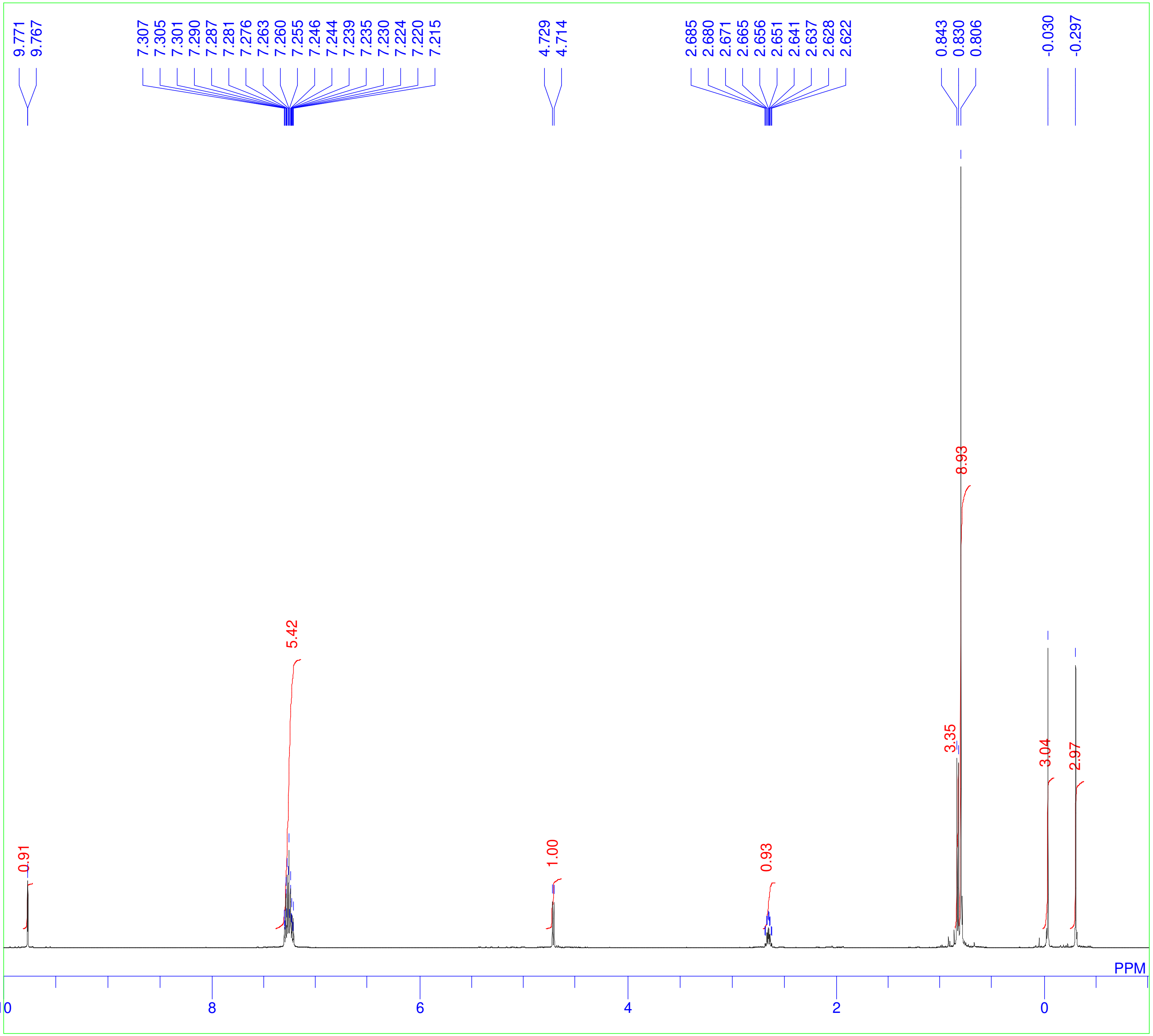

DFILE 15q_1H.als
COMNT
DATIM 2023-01-05 11:34:55
OBNUC 1H
EXMOD proton.jxp
OBFRQ 500.16 MHz
OBSET 2.41 KHz
OBFIN 6.01 Hz
POINT 13107
FREQU 7507.51 Hz
SCANS 8
ACQTM 1.7459 sec
PD 5.0000 sec
PW1 3.84 usec
IRNUC 1H
CTEMP 20.6 c
SLVNT CDCL3
EXREF 7.26 ppm
BF 0.30 Hz
RGAIN 24

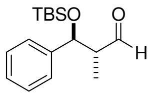

15q

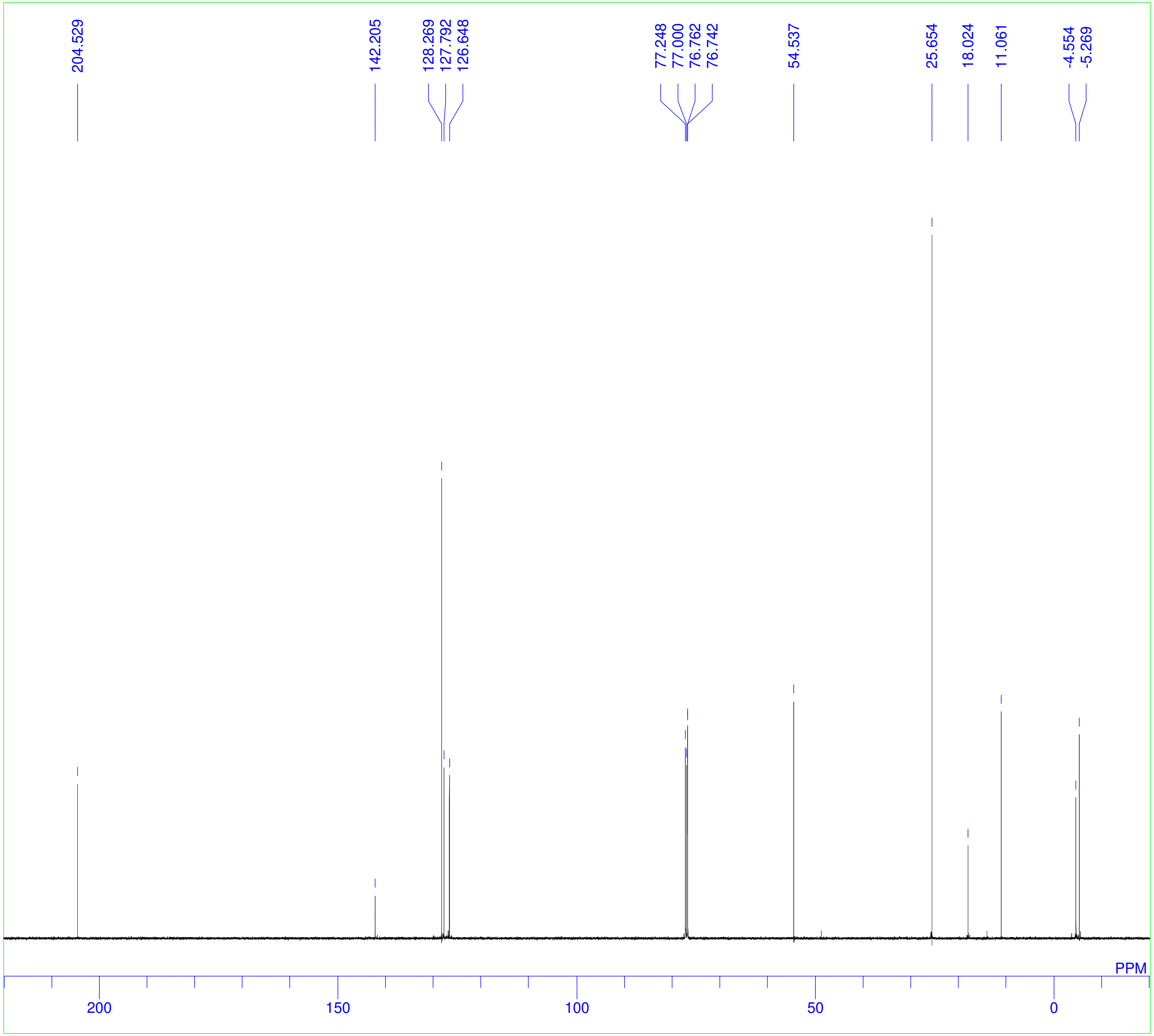

DFILE 15q_13C.als
COMNT
DATIM 2023-01-05 11:36:44
OBNUC 13C
EXMOD carbon.jxp
OBFRQ 125.77 MHz
OBSET 7.87 KHz
OBFIN 4.21 Hz
POINT 26214
FREQU 31446.54 Hz
SCANS 1024
ACQTM 0.8336 sec
PD 2.0000 sec
PW1 3.87 usec
IRNUC 1H
CTEMP 20.7 c
SLVNT CDCL3
EXREF 77.00 ppm
BF 0.30 Hz
RGAIN 24

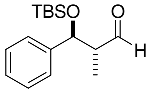

15q

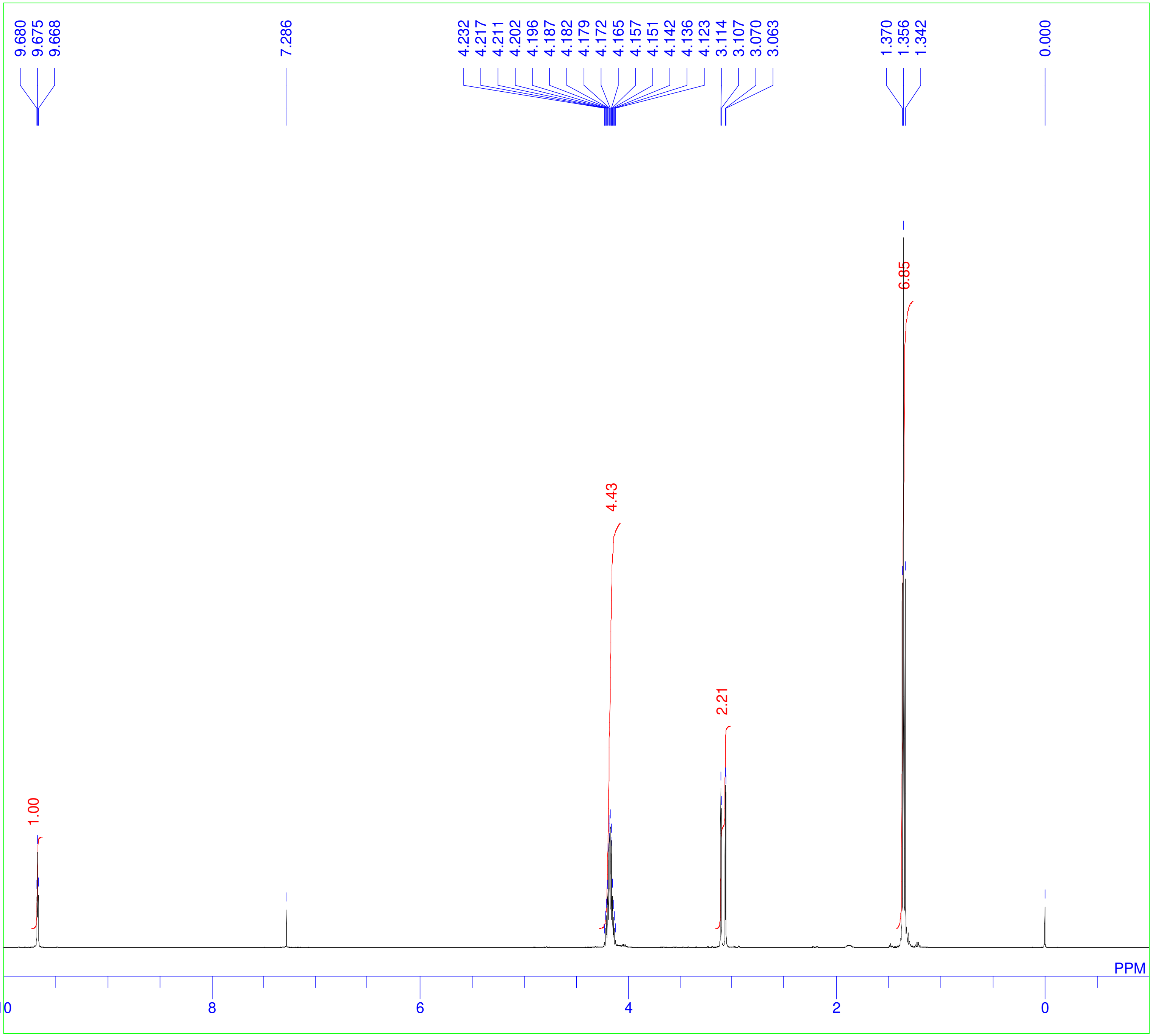

DFILE	15t_1H.als
COMNT	
DATIM	2024-01-11 21:42:43
OBNUC	1H
EXMOD	proton.jxp
OBFRQ	500.16 MHz
OBSET	2.41 KHz
OBFIN	6.01 Hz
POINT	13107
FREQU	7507.51 Hz
SCANS	8
ACQTM	1.7459 sec
PD	5.0000 sec
PW1	3.80 usec
IRNUC	1H
CTEMP	21.3 c
SLVNT	CDCL3
EXREF	0.00 ppm
BF	0.30 Hz
RGAIN	30

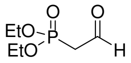

15t

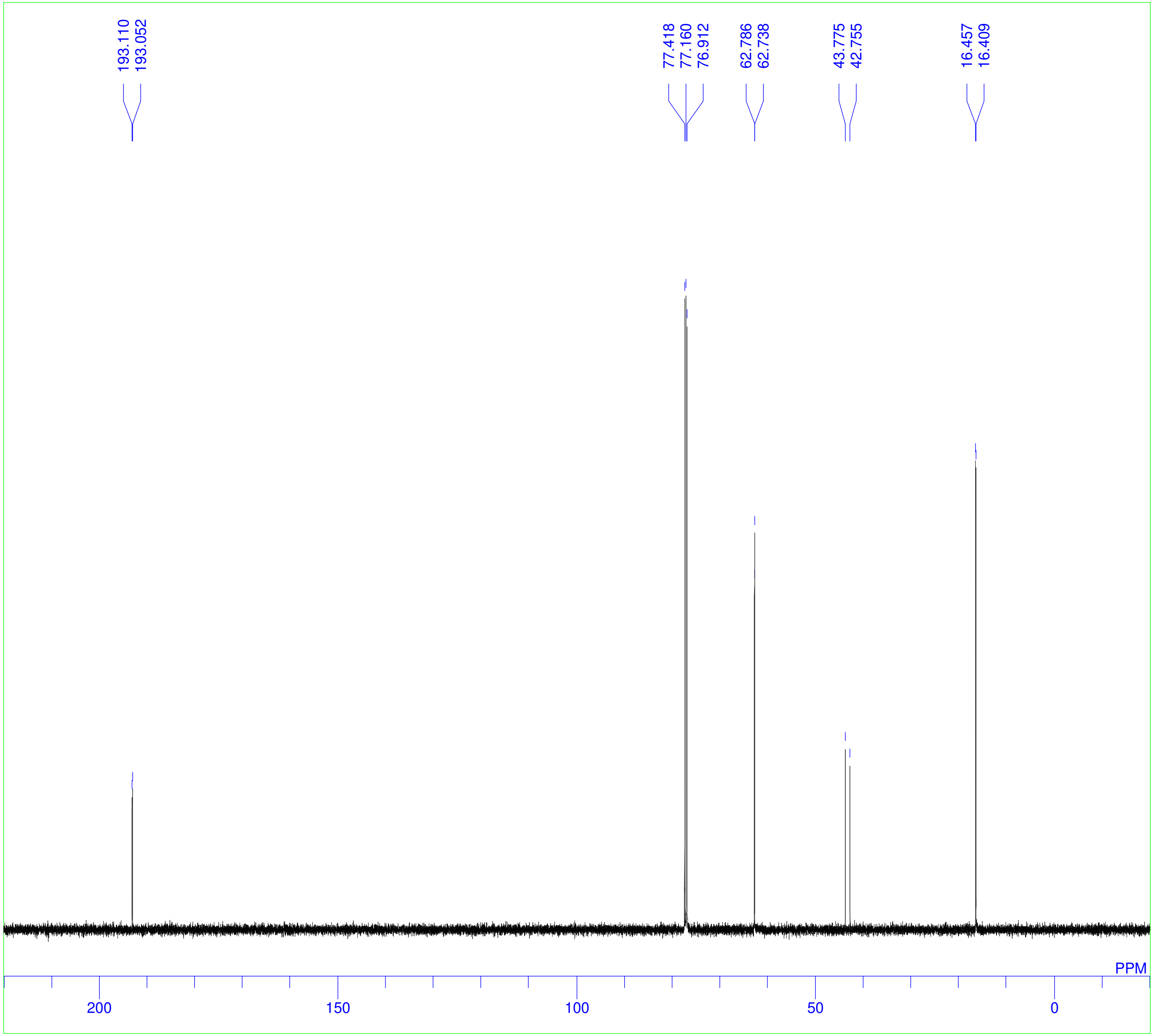

DFILE	15t_13C.als
COMNT	
DATIM	2023-05-16 19:06:21
OBNUC	13C
EXMOD	carbon.jxp
OBFRQ	125.77 MHz
OBSET	7.87 KHz
OBFIN	4.21 Hz
POINT	26214
FREQU	31446.54 Hz
SCANS	1024
ACQTM	0.8336 sec
PD	2.0000 sec
PW1	3.87 usec
IRNUC	1H
CTEMP	23.9 c
SLVNT	CDCL3
EXREF	77.16 ppm
BF	0.12 Hz
RGAIN	30

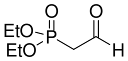

15t

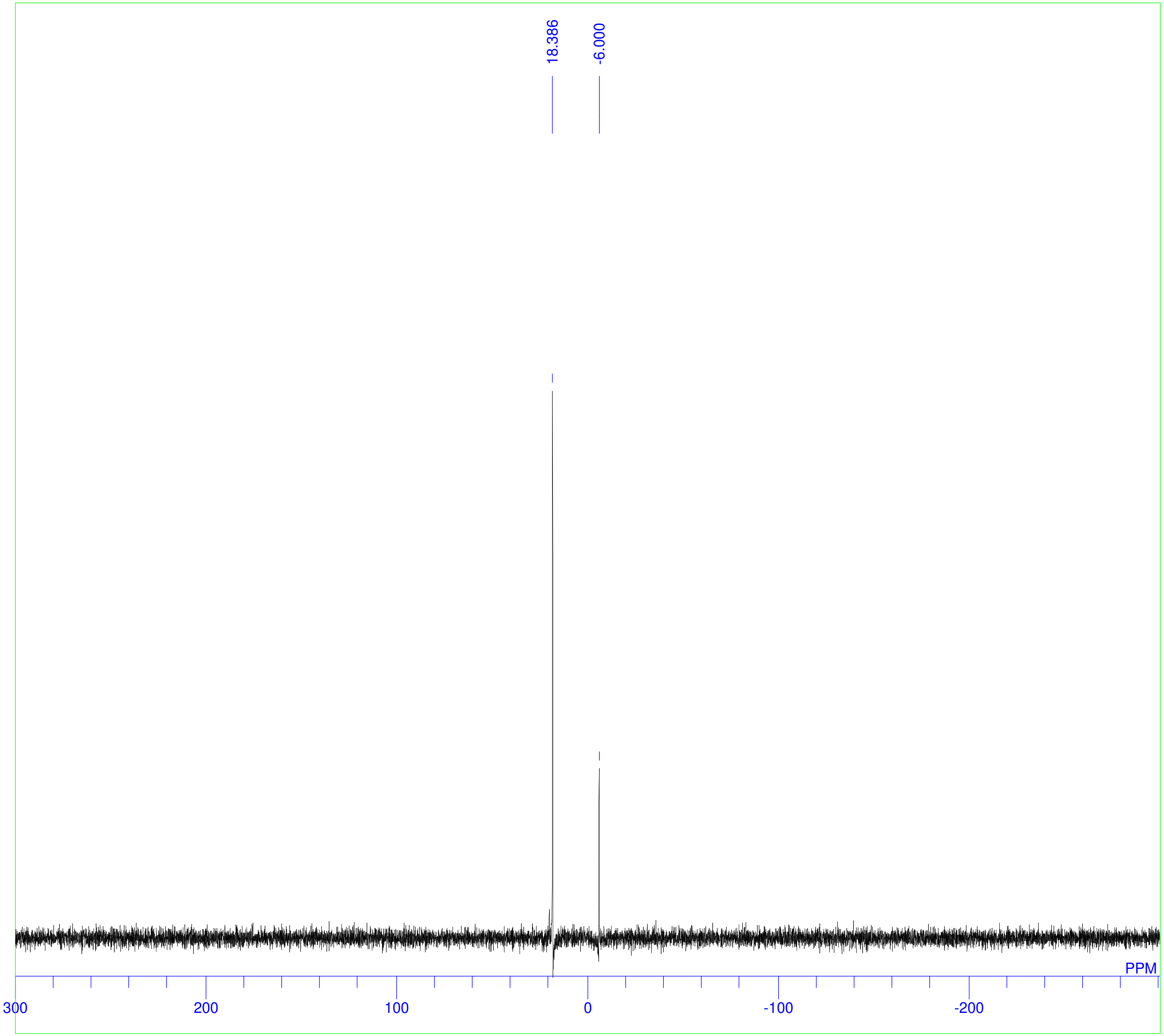

DFILE	15t_31P.als
COMNT	
DATIM	2023-05-16 20:13:53
OBNUC	31P
EXMOD	single_pulse.jxp
OBFRQ	202.46 MHz
OBSET	8.31 KHz
OBFIN	0.75 Hz
POINT	13107
FREQU	142857.14 Hz
SCANS	32
ACQTM	0.0918 sec
PD	5.0000 sec
PW1	6.45 usec
IRNUC	31P
CTEMP	23.7 c
SLVNT	CDCL3
EXREF	-6.00 ppm
BF	0.12 Hz
RGAIN	50

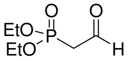

15t

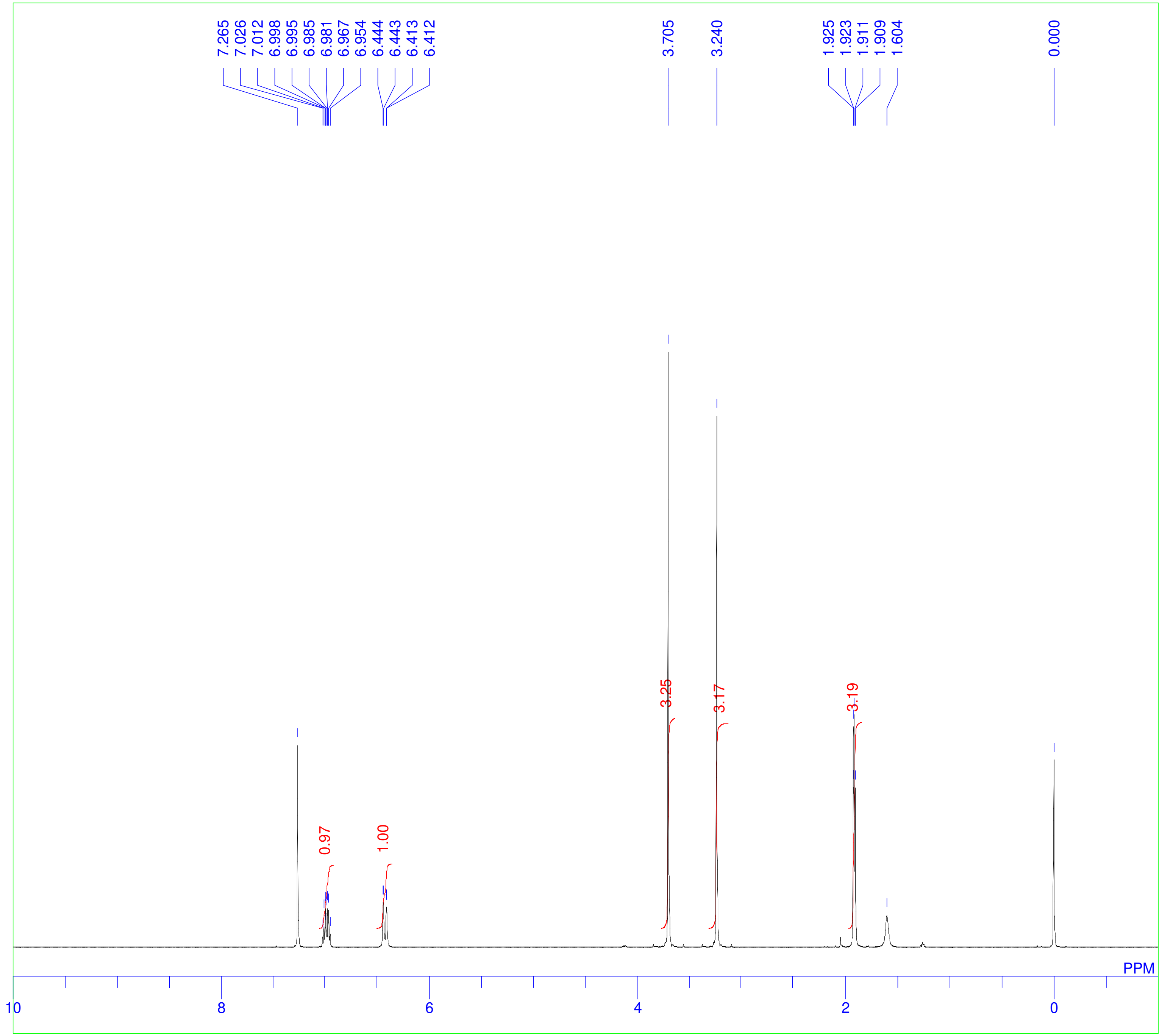

DFILE (E)-16a_1H.als
COMNT
DATIM 2022-01-15 08:05:12
OBNUC 1H
EXMOD proton.jxp
OBFRQ 500.16 MHz
OBSET 2.41 KHz
OBFIN 6.01 Hz
POINT 13107
FREQU 7507.51 Hz
SCANS 8
ACQTM 1.7459 sec
PD 5.0000 sec
PW1 3.84 usec
IRNUC 1H
CTEMP 18.4 c
SLVNT CDCL3
EXREF 0.00 ppm
BF 0.30 Hz
RGAIN 44

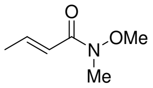

(*E*)-16a

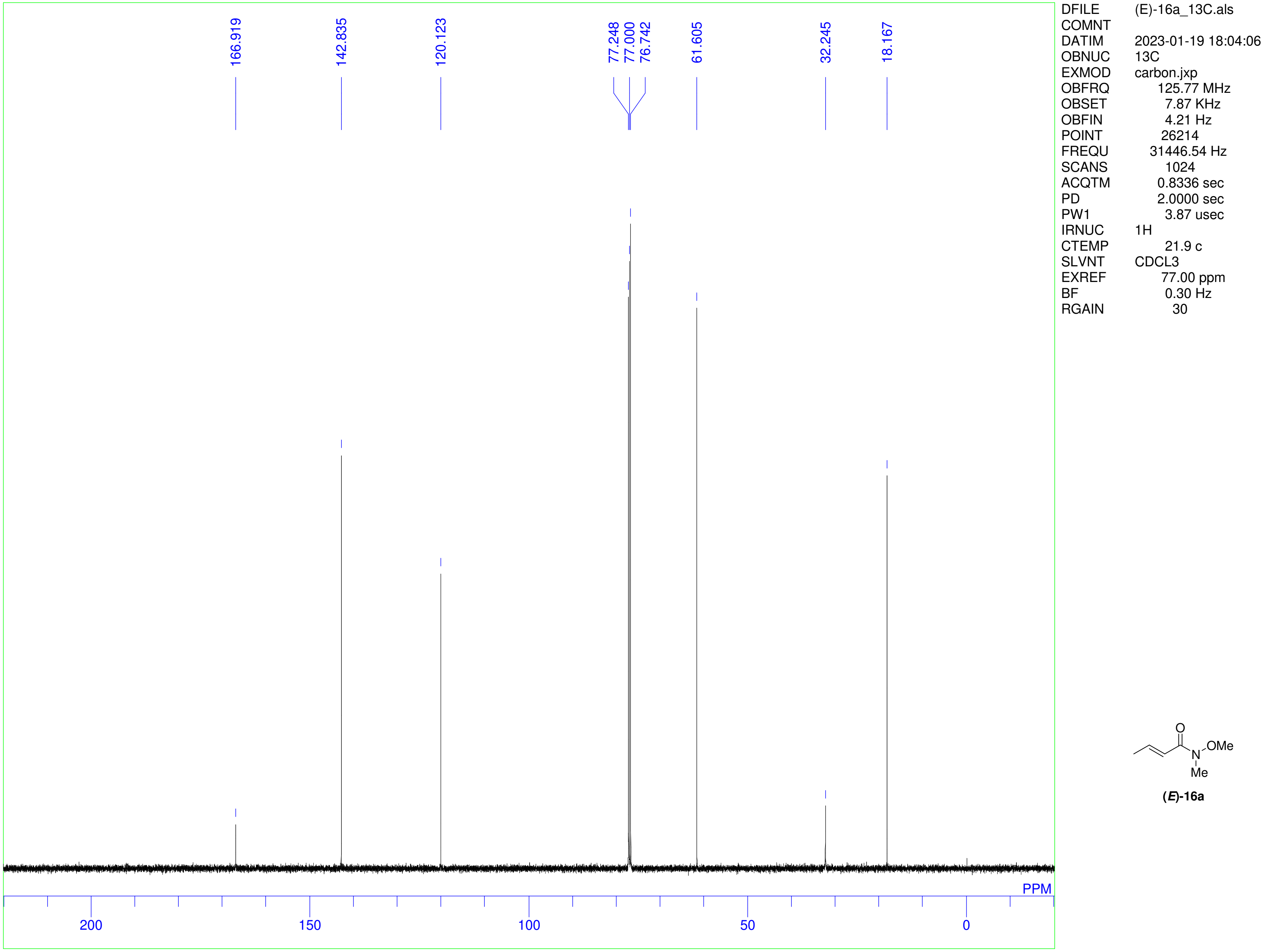
166.919
142.835
120.123
77.248
77.000
76.742
61.605
32.245
18.167
PPM
200
150
100
50
0
DFILE (E)-16a_13C.als
COMNT
DATIM 2023-01-19 18:04:06
OBNUC 13C
EXMOD carbon.jxp
OBFRQ 125.77 MHz
OBSET 7.87 KHz
OBFIN 4.21 Hz
POINT 26214
FREQU 31446.54 Hz
SCANS 1024
ACQTM 0.8336 sec
PD 2.0000 sec
PW1 3.87 usec
IRNUC 1H
CTEMP 21.9 c
SLVNT CDCL3
EXREF 77.00 ppm
BF 0.30 Hz
RGAIN 30
O
N
OMe
Me

(*E*)-16a

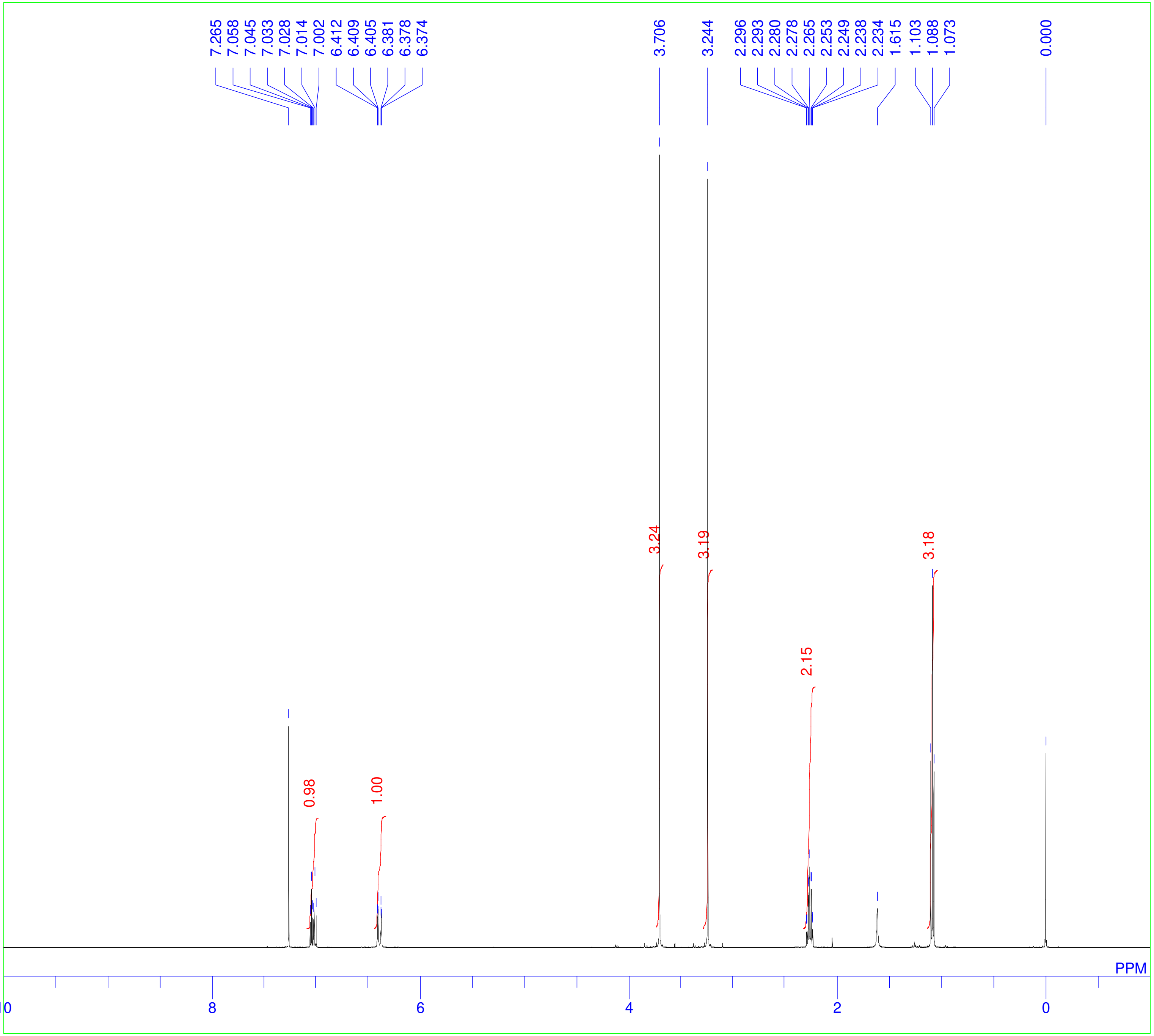

DFILE	(E)-16b_1H.als
COMNT	
DATIM	2022-01-26 20:58:06
OBNUC	1H
EXMOD	proton.jxp
OBFRQ	500.16 MHz
OBSET	2.41 KHz
OBFIN	6.01 Hz
POINT	13107
FREQU	7507.51 Hz
SCANS	8
ACQTM	1.7459 sec
PD	5.0000 sec
PW1	3.84 usec
IRNUC	1H
CTEMP	19.9 c
SLVNT	CDCL3
EXREF	0.00 ppm
BF	0.30 Hz
RGAIN	42

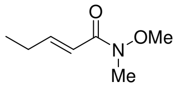

(*E*)-16b

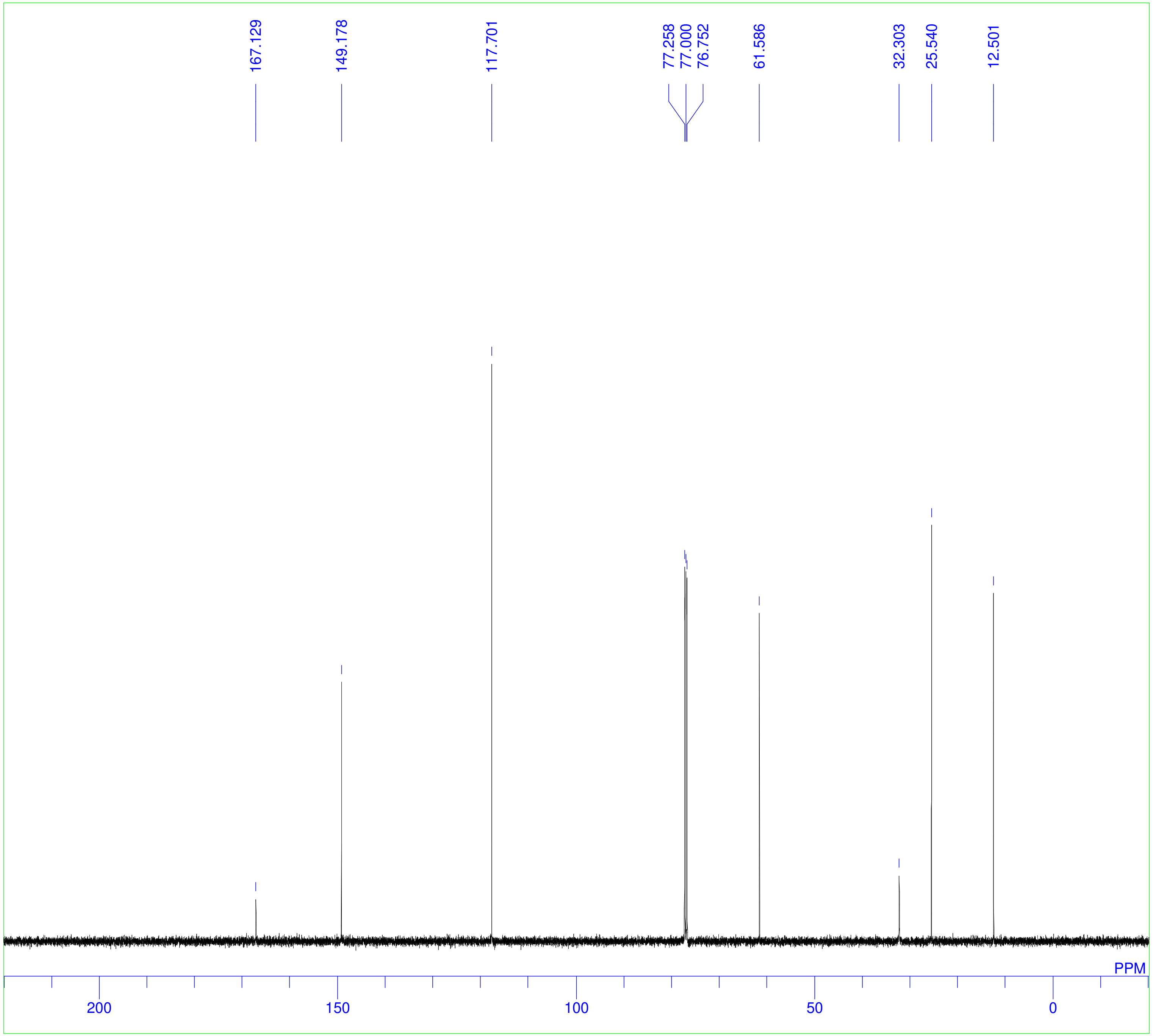

DFILE	(E)-16b_13C.als
COMNT	
DATIM	2023-01-19 18:59:06
OBNUC	13C
EXMOD	carbon.jxp
OBFRQ	125.77 MHz
OBSET	7.87 KHz
OBFIN	4.21 Hz
POINT	26214
FREQU	31446.54 Hz
SCANS	1024
ACQTM	0.8336 sec
PD	2.0000 sec
PW1	3.87 usec
IRNUC	1H
CTEMP	21.8 c
SLVNT	CDCL3
EXREF	77.00 ppm
BF	0.30 Hz
RGAIN	24

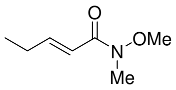

(*E*)-16b

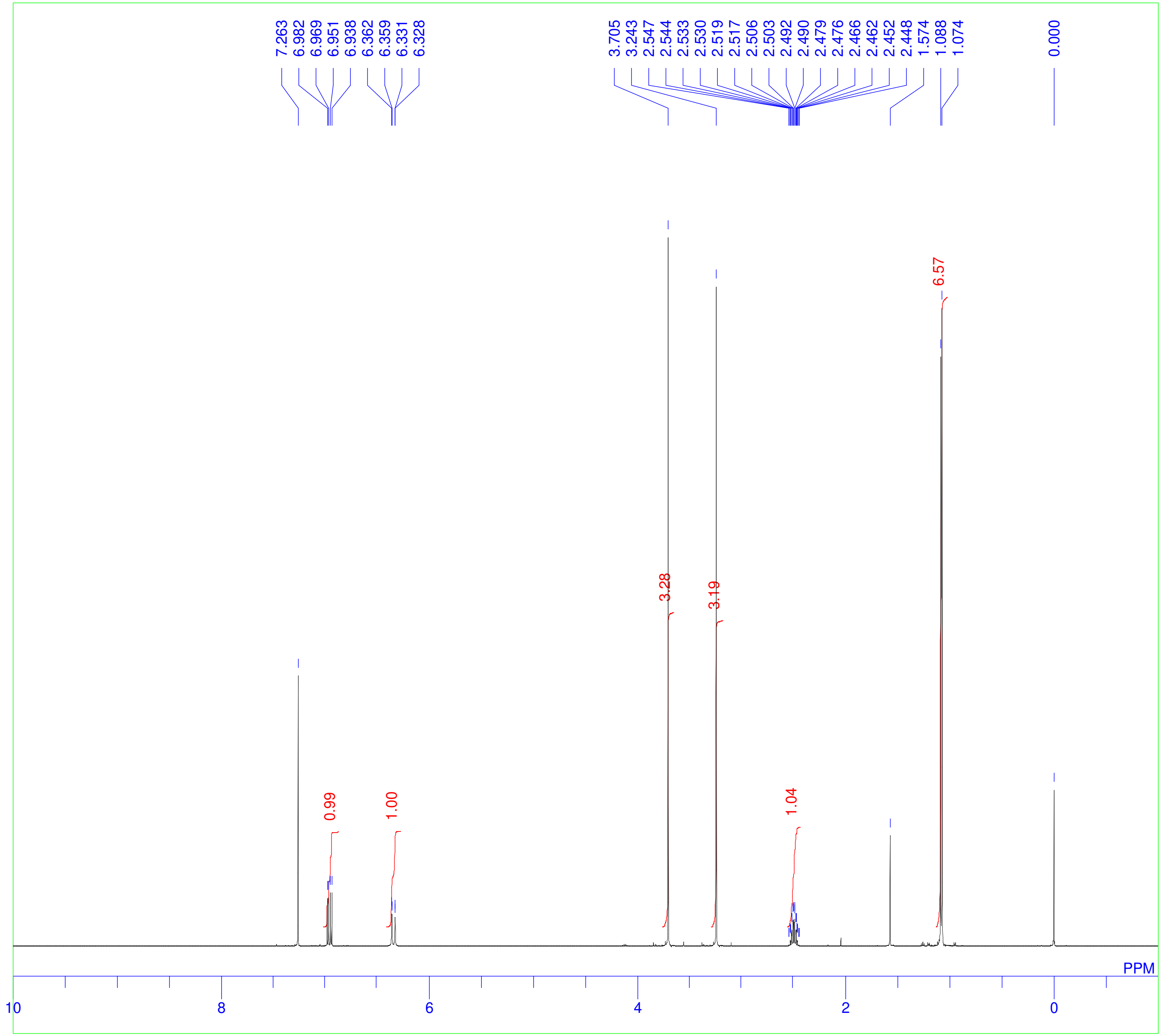

DFILE	(E)-16c_1H.als
COMNT	
DATIM	2023-01-18 20:18:33
OBNUC	1H
EXMOD	proton.jxp
OBFRQ	500.16 MHz
OBSET	2.41 KHz
OBFIN	6.01 Hz
POINT	13107
FREQU	7507.51 Hz
SCANS	8
ACQTM	1.7459 sec
PD	5.0000 sec
PW1	3.84 usec
IRNUC	1H
CTEMP	22.3 c
SLVNT	CDCL3
EXREF	0.00 ppm
BF	0.12 Hz
RGAIN	44

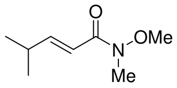

(*E*)-16c

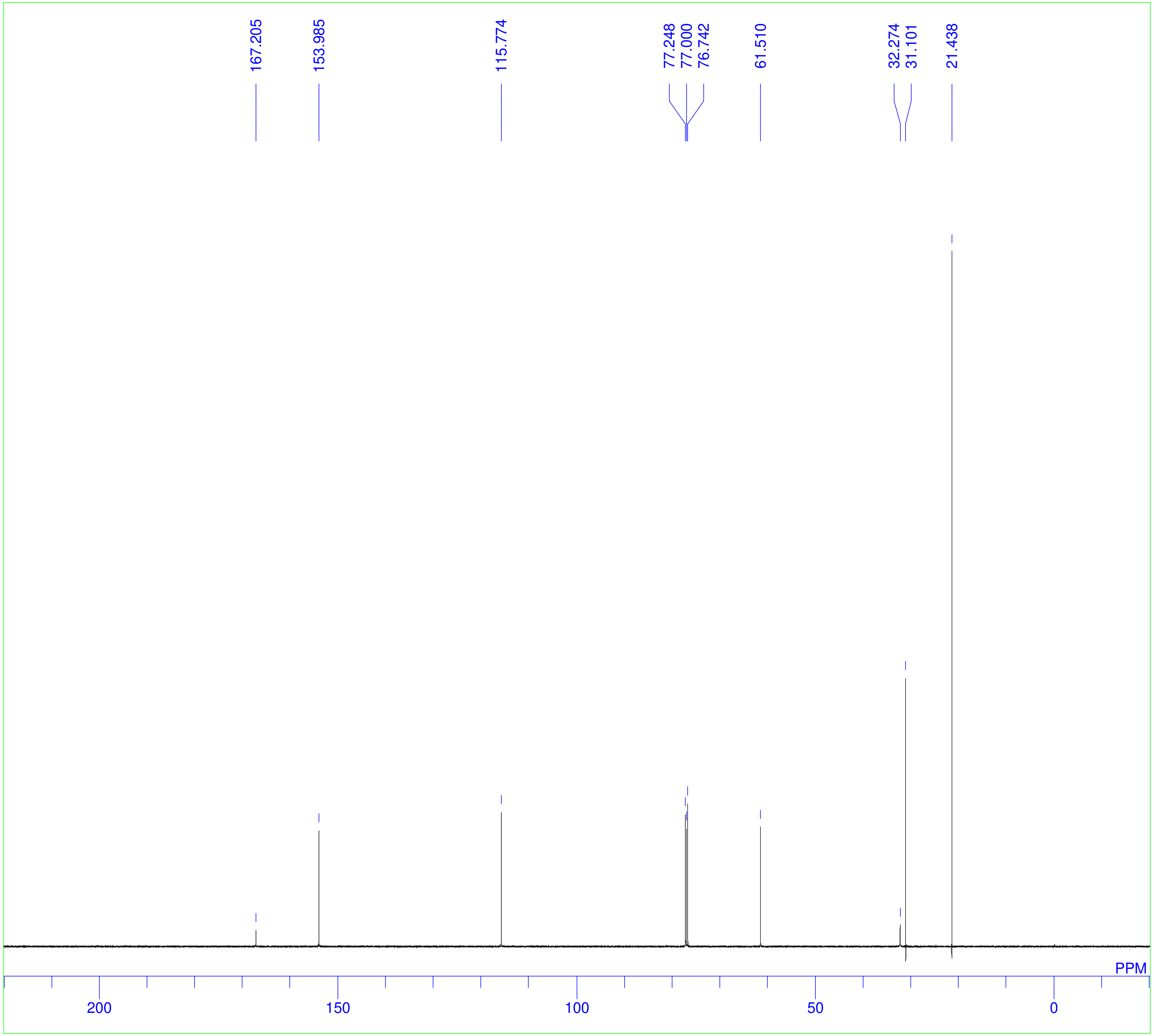

DFILE	(E)-16c_13C.als
COMNT	
DATIM	2023-01-19 19:52:28
OBNUC	13C
EXMOD	carbon.jxp
OBFRQ	125.77 MHz
OBSET	7.87 KHz
OBFIN	4.21 Hz
POINT	26214
FREQU	31446.54 Hz
SCANS	1024
ACQTM	0.8336 sec
PD	2.0000 sec
PW1	3.87 usec
IRNUC	1H
CTEMP	21.9 c
SLVNT	CDCL3
EXREF	77.00 ppm
BF	0.12 Hz
RGAIN	26

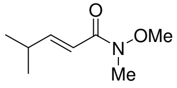

(*E*)-16c

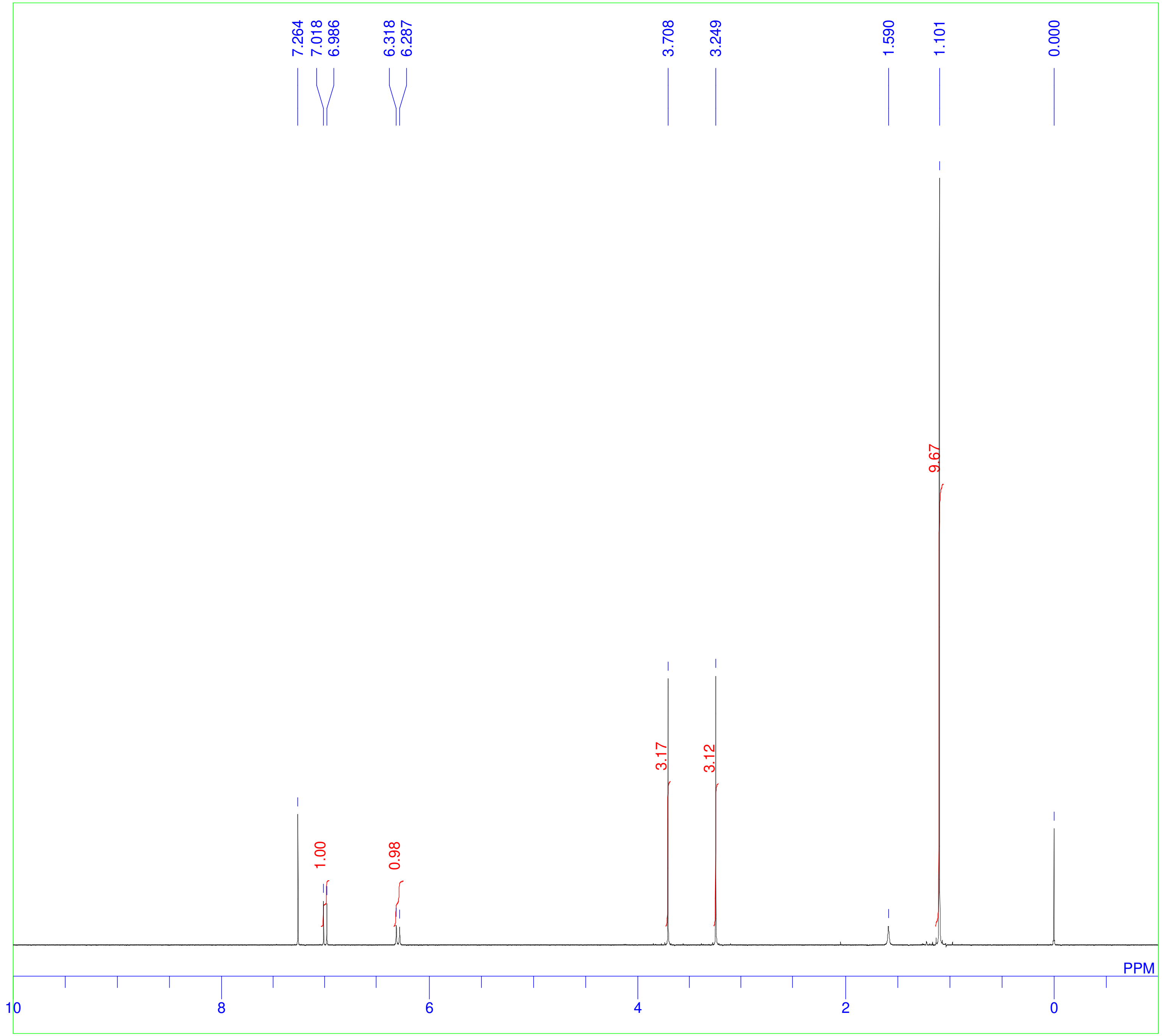

DFILE	(E)-16d_1H.als
COMNT	
DATIM	2022-01-13 15:24:51
OBNUC	1H
EXMOD	proton.jxp
OBFRQ	500.16 MHz
OBSET	2.41 KHz
OBFIN	6.01 Hz
POINT	13107
FREQU	7507.51 Hz
SCANS	8
ACQTM	1.7459 sec
PD	5.0000 sec
PW1	3.84 usec
IRNUC	1H
CTEMP	19.8 c
SLVNT	CDCL3
EXREF	0.00 ppm
BF	0.30 Hz
RGAIN	48

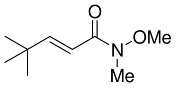

(*E*)-16d

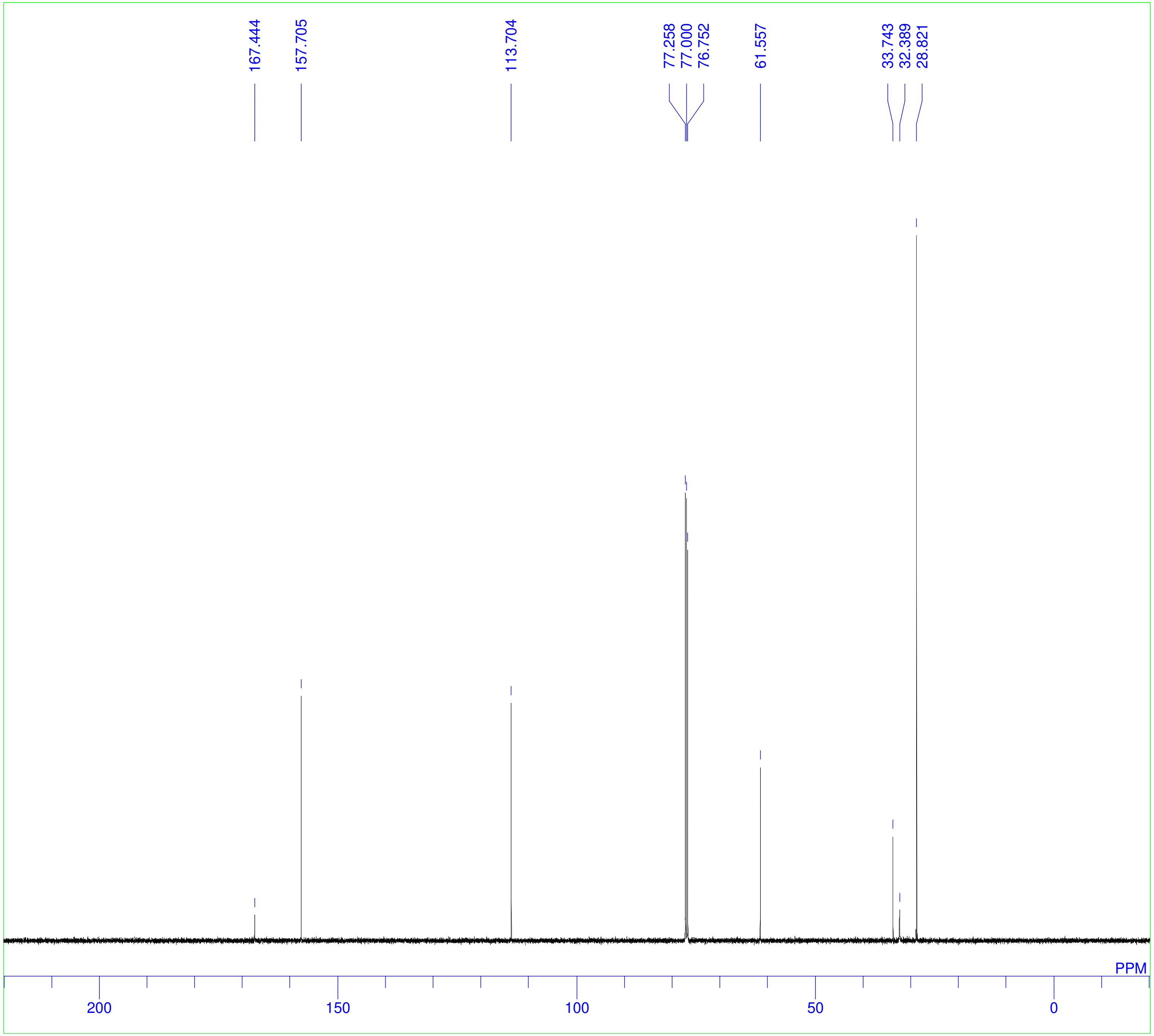

DFILE	(E)-16d_13C.als
COMNT	
DATIM	2023-01-06 09:43:15
OBNUC	13C
EXMOD	carbon.jxp
OBFRQ	125.77 MHz
OBSET	7.87 KHz
OBFIN	4.21 Hz
POINT	26214
FREQU	31446.54 Hz
SCANS	1024
ACQTM	0.8336 sec
PD	2.0000 sec
PW1	3.87 usec
IRNUC	1H
CTEMP	20.2 c
SLVNT	CDCL3
EXREF	77.00 ppm
BF	0.30 Hz
RGAIN	24

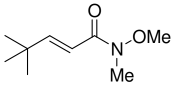

(*E*)-16d

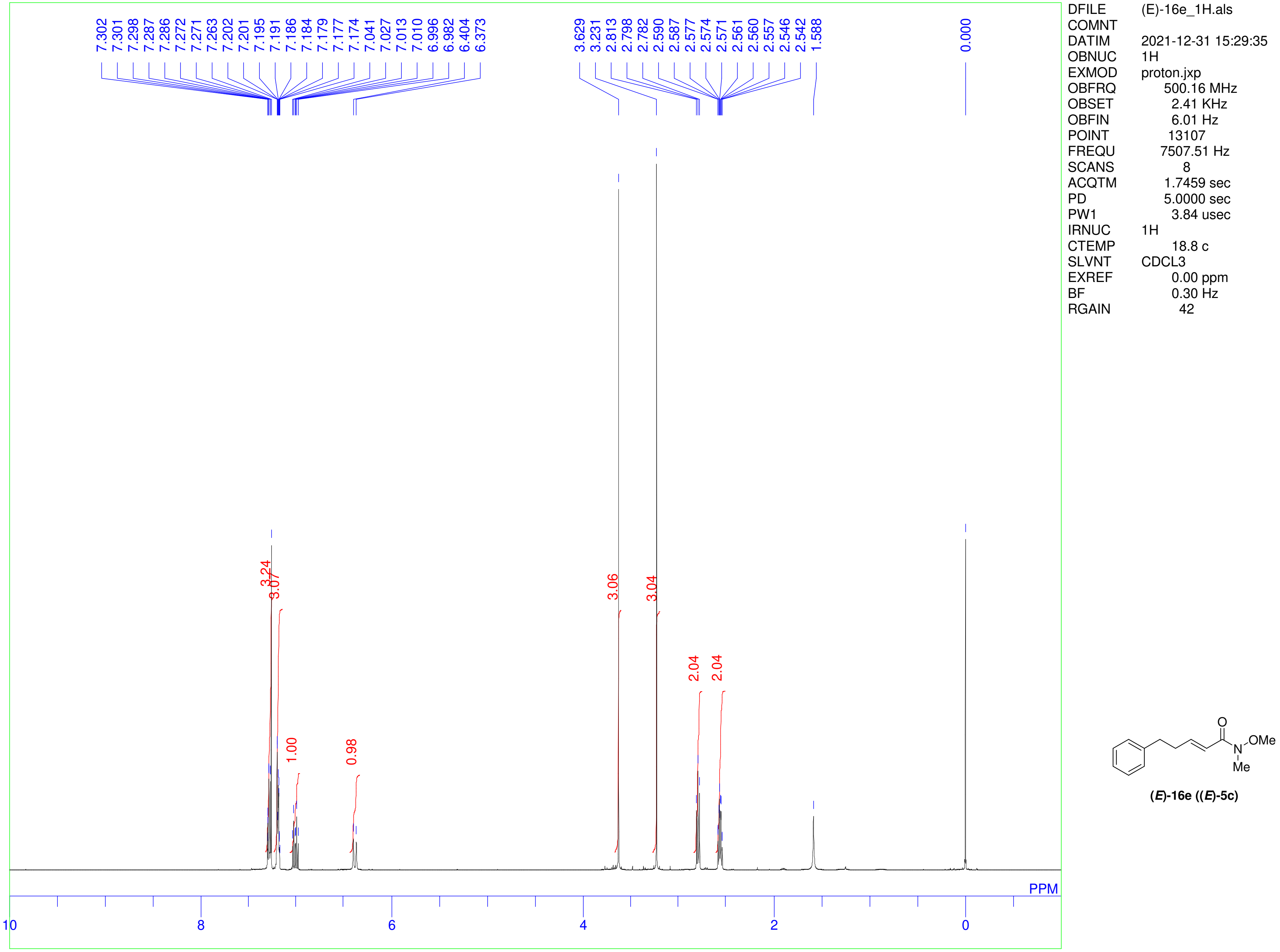
7.302
7.301
7.298
7.287
7.286
7.272
7.271
7.263
7.202
7.201
7.195
7.191
7.186
7.184
7.179
7.177
7.174
7.041
7.027
7.013
7.010
6.996
6.982
6.404
6.373
3.629
3.231
2.813
2.798
2.782
2.590
2.587
2.577
2.574
2.571
2.561
2.560
2.557
2.546
2.542
1.588
0.000
3.24
3.07
1.00
0.98
3.06
3.04
2.04
2.04
PPM
10
8
6
4
2
0
DFILE (E)-16e_1H.als
COMNT
DATIM 2021-12-31 15:29:35
OBNUC 1H
EXMOD proton.jxp
OBFRQ 500.16 MHz
OBSET 2.41 KHz
OBFIN 6.01 Hz
POINT 13107
FREQU 7507.51 Hz
SCANS 8
ACQTM 1.7459 sec
PD 5.0000 sec
PW1 3.84 usec
IRNUC 1H
CTEMP 18.8 c
SLVNT CDCL3
EXREF 0.00 ppm
BF 0.30 Hz
RGAIN 42
O
N
OMe
Me

(*E*)-16e ((*E*)-5c)

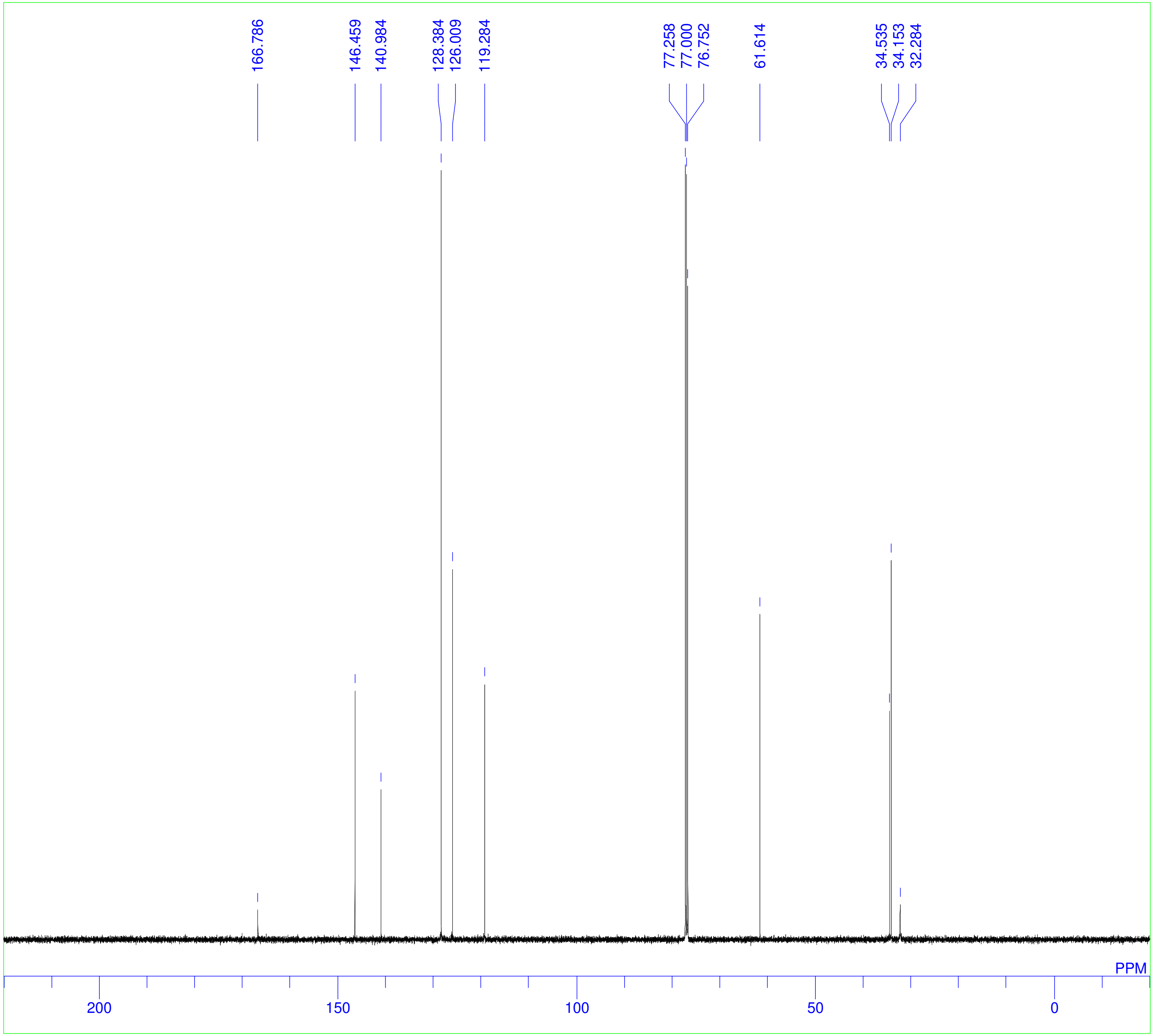

DFILE	(E)-16e_13C.als
COMNT	
DATIM	2022-02-03 18:38:40
OBNUC	13C
EXMOD	carbon.jxp
OBFRQ	125.77 MHz
OBSET	7.87 KHz
OBFIN	4.21 Hz
POINT	26214
FREQU	31446.54 Hz
SCANS	1792
ACQTM	0.8336 sec
PD	2.0000 sec
PW1	3.87 usec
IRNUC	1H
CTEMP	20.2 c
SLVNT	CDCL3
EXREF	77.00 ppm
BF	0.30 Hz
RGAIN	30

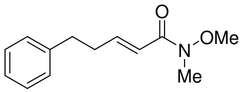

(*E*)-16e ((*E*)-5c)

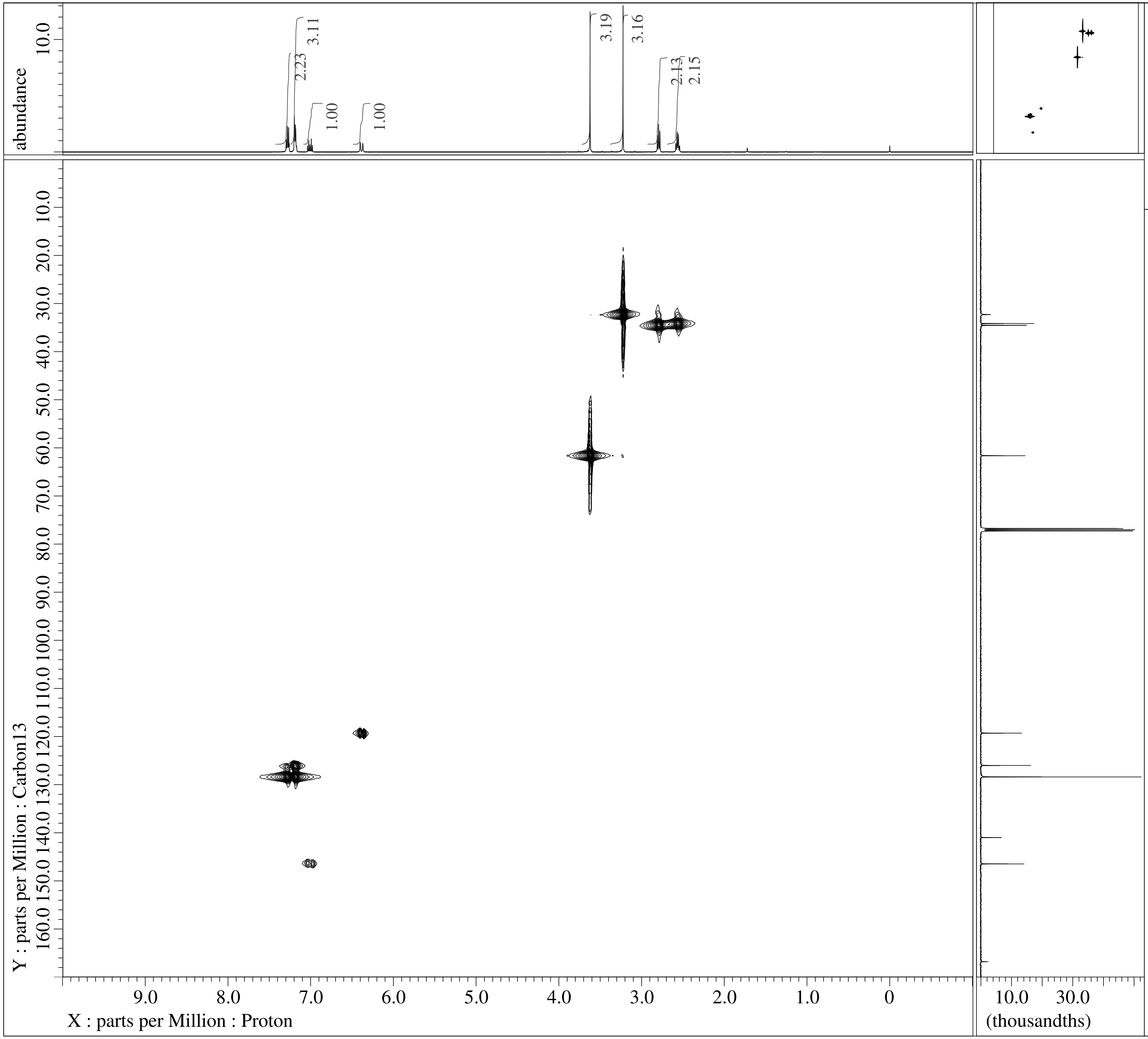


```
---- PROCESSING PARAMETERS ----
sinbell4( -60, 160 )
zerofill( 1 )
fft( 1, TRUE, TRUE )
ppm
[transpose]
sinbell4( -60, 160 )
zerofill( 2 )
fft( 1, TRUE, TRUE )
ppm
abs
```

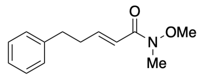

(*E*)-16e ((*E*)-5c)

```
Filename            = HT-1706-PTLC 3_HMQC,HMB
Author              = delta
Experiment          = hmqc.jxp
Sample_Id           = HT-1706-PTLC 3_HMQC,HMB
Solvent             = CHLOROFORM-D
Creation_Time       = 30-DEC-2023 14:22:49
Revision_Time       = 10-APR-2024 19:14:58
Current_Time        = 10-APR-2024 19:15:34

Comment             = gradient enhanced HMQC
Data_Format         = 2D REAL REAL
Dim_Size            = 819, 512
Dim_Title           = Proton Carbon13
Dim_Units           = [ppm] [ppm]
Dimensions          = X Y
Site                = JNM-ECA500II
Spectrometer        = DELTA2_NMR

Field_Strength      = 11.7473579[T] (500[MHz]
X_Acq_Duration      = 0.12812288[s]
X_Domain            = 1H
X_Freq              = 500.15991521[MHz]
X_Offset            = 4.89636044[ppm]
X_Points            = 1024
X_Prescans          = 4
X_Resolution        = 7.80500719[Hz]
X_Sweep             = 7.99232737[kHz]
X_Sweep_Clipped     = 6.39386189[kHz]
Y_Domain            = 13C
Y_Freq              = 125.76529768[MHz]
Y_Offset            = 85[ppm]
Y_Points            = 256
Y_Prescans          = 0
Y_Resolution        = 83.60980308[Hz]
Y_Sweep             = 21.40410959[kHz]
Tri_Domain          = Proton
Tri_Freq            = 500.15991521[MHz]
Tri_Offset          = 5.0[ppm]
Clipped             = FALSE
Scans               = 8
Total_Scans         = 2048

Relaxation_Delay    = 1.5[s]
Recvr_Gain          = 50
Temp_Get            = 22.9[dC]
X_Acq_Time          = 0.12812288[s]
X_Atn               = 3.2[dB]
X_Gamma             = 42576375
X_Pulse             = 7.59[us]
```

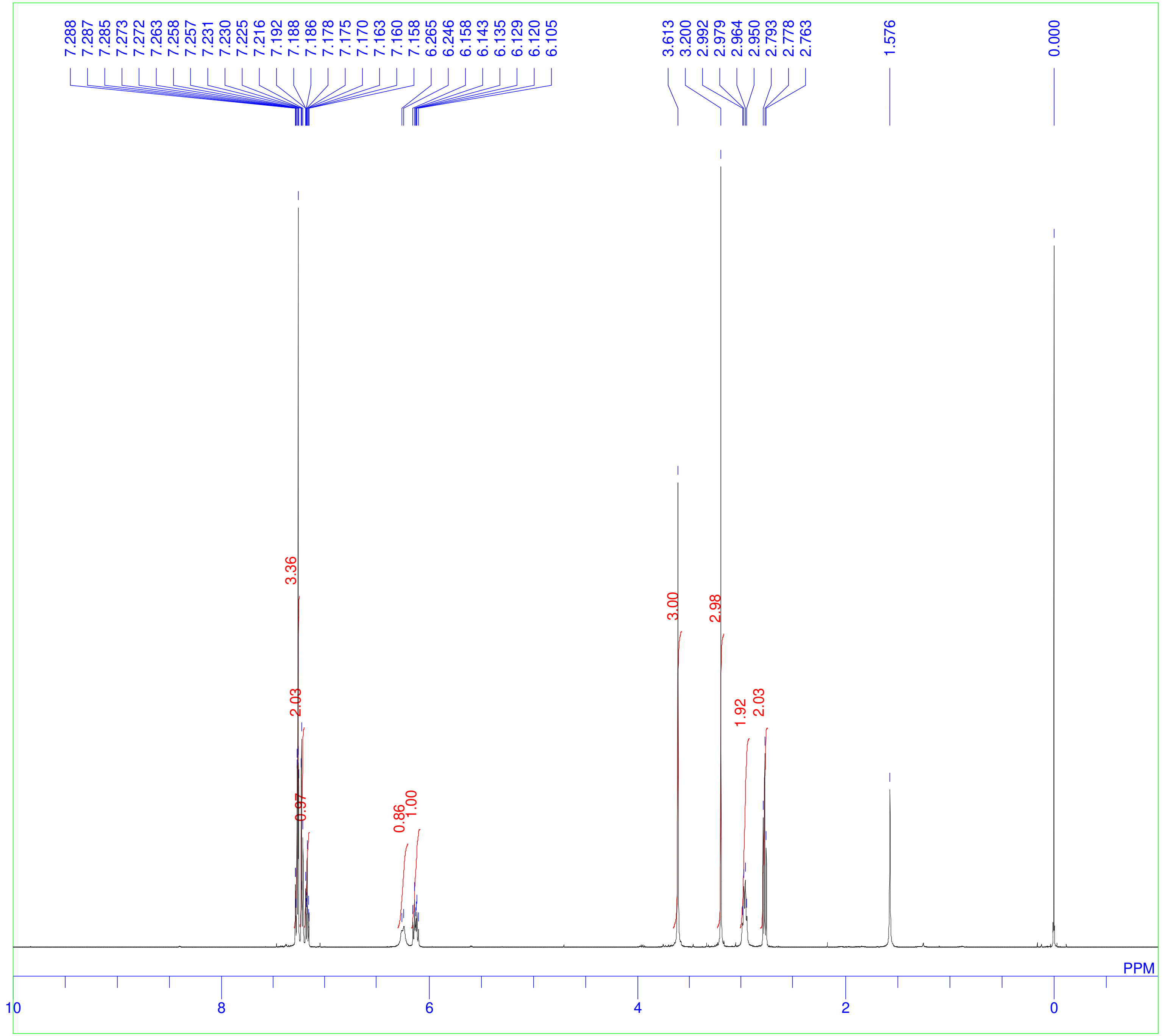

DFILE	(Z)-16e_1H.als
COMNT	
DATIM	2021-12-31 15:22:14
OBNUC	1H
EXMOD	proton.jxp
OBFRQ	500.16 MHz
OBSET	2.41 KHz
OBFIN	6.01 Hz
POINT	13107
FREQU	7507.51 Hz
SCANS	8
ACQTM	1.7459 sec
PD	5.0000 sec
PW1	3.84 usec
IRNUC	1H
CTEMP	18.8 c
SLVNT	CDCL3
EXREF	0.00 ppm
BF	0.30 Hz
RGAIN	44

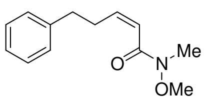

(*Z*)-16e ((*Z*)-5c)

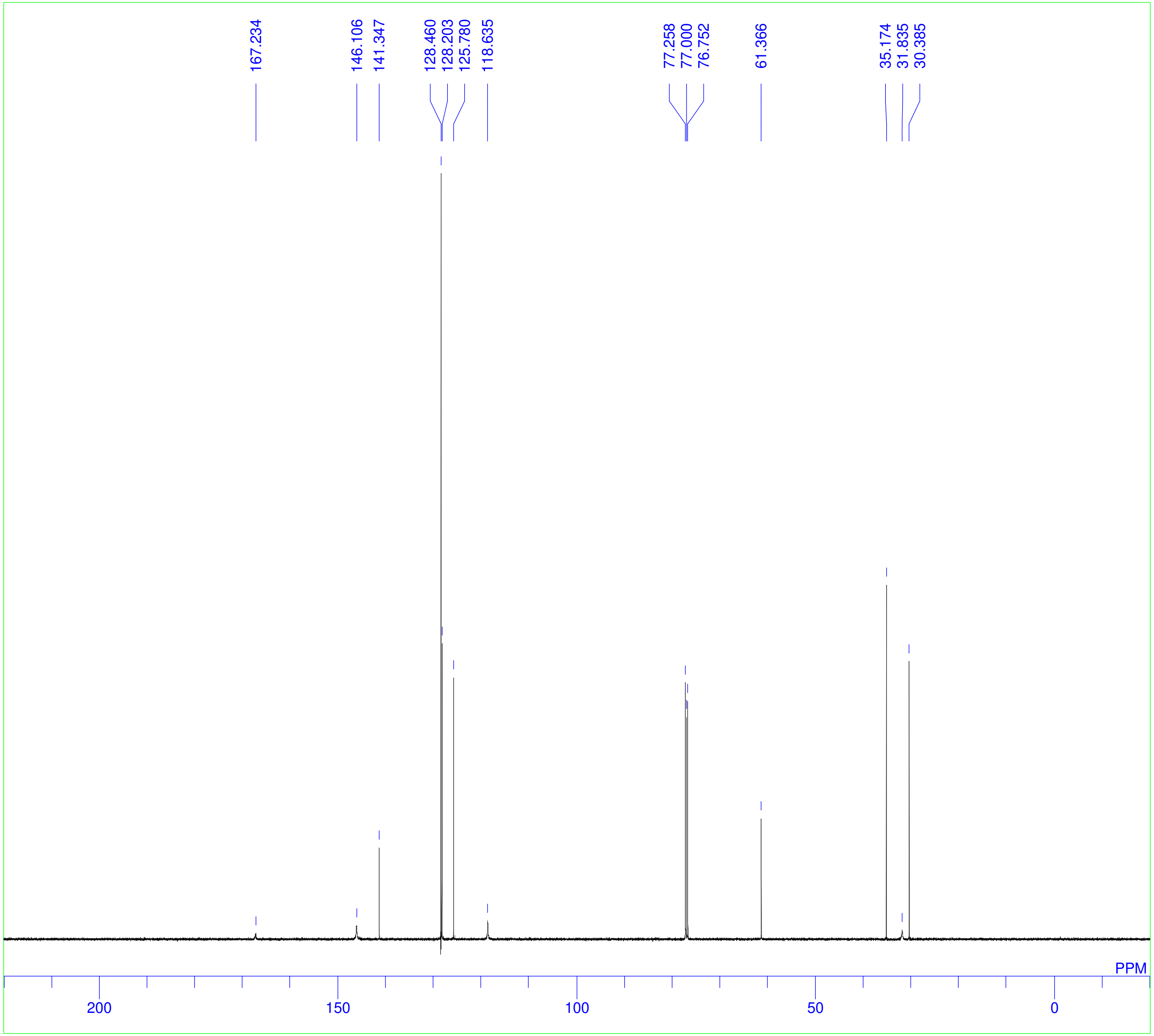

DFILE	(Z)-16e_13C.als
COMNT	
DATIM	2023-01-04 11:47:05
OBNUC	13C
EXMOD	carbon.jxp
OBFRQ	125.77 MHz
OBSET	7.87 KHz
OBFIN	4.21 Hz
POINT	26214
FREQU	31446.54 Hz
SCANS	1024
ACQTM	0.8336 sec
PD	2.0000 sec
PW1	3.87 usec
IRNUC	1H
CTEMP	20.9 c
SLVNT	CDCL3
EXREF	77.00 ppm
BF	0.30 Hz
RGAIN	26

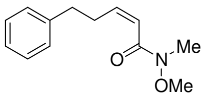

(*Z*)-16e ((*Z*)-5c)

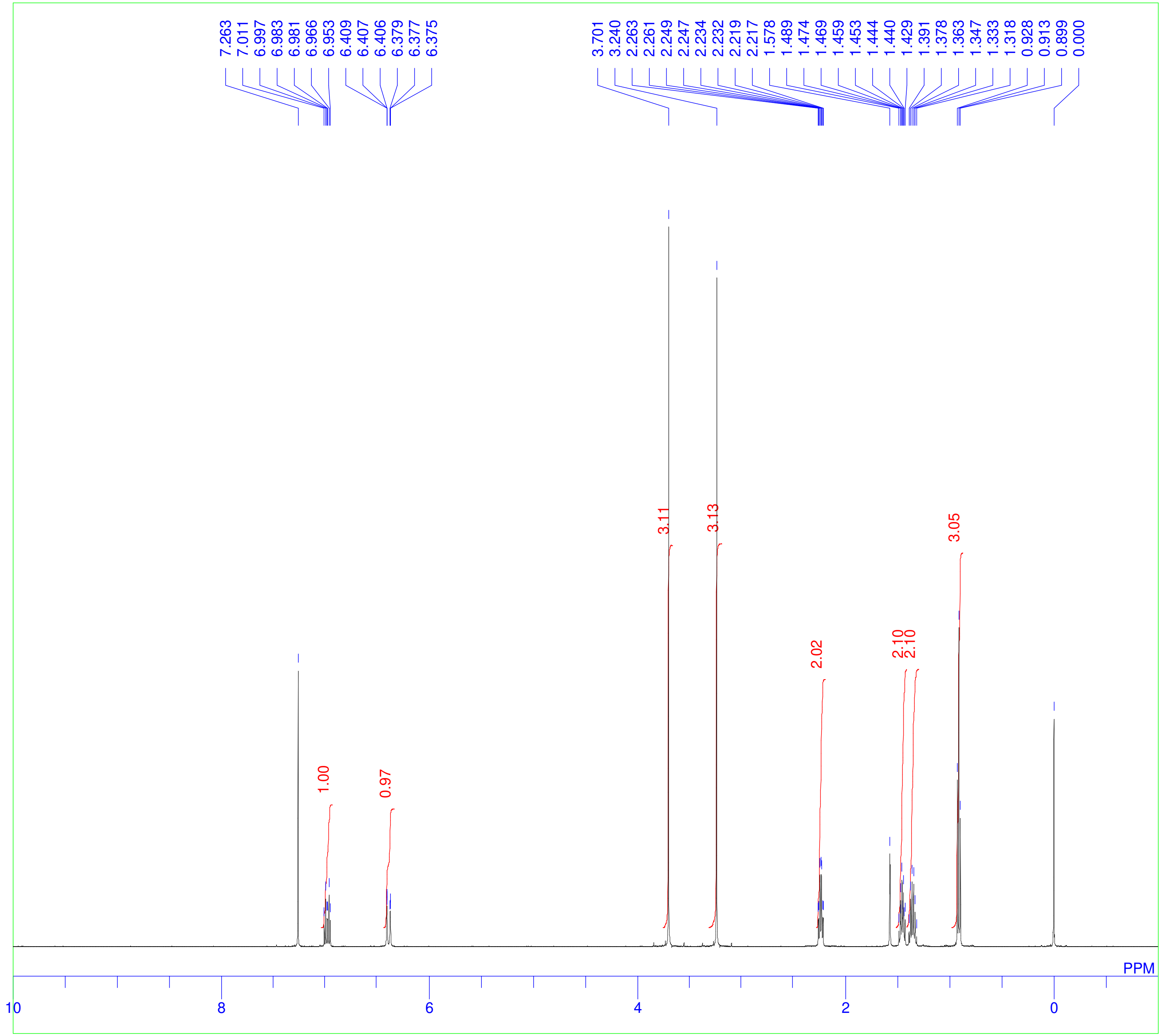

DFILE	(E)-16f_1H.als
COMNT	
DATIM	2023-05-04 10:22:28
OBNUC	1H
EXMOD	proton.jxp
OBFRQ	500.16 MHz
OBSET	2.41 KHz
OBFIN	6.01 Hz
POINT	13107
FREQU	7507.51 Hz
SCANS	8
ACQTM	1.7459 sec
PD	5.0000 sec
PW1	3.84 usec
IRNUC	1H
CTEMP	24.1 c
SLVNT	CDCL3
EXREF	0.00 ppm
BF	0.30 Hz
RGAIN	46

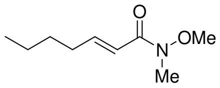

(*E*)-16f

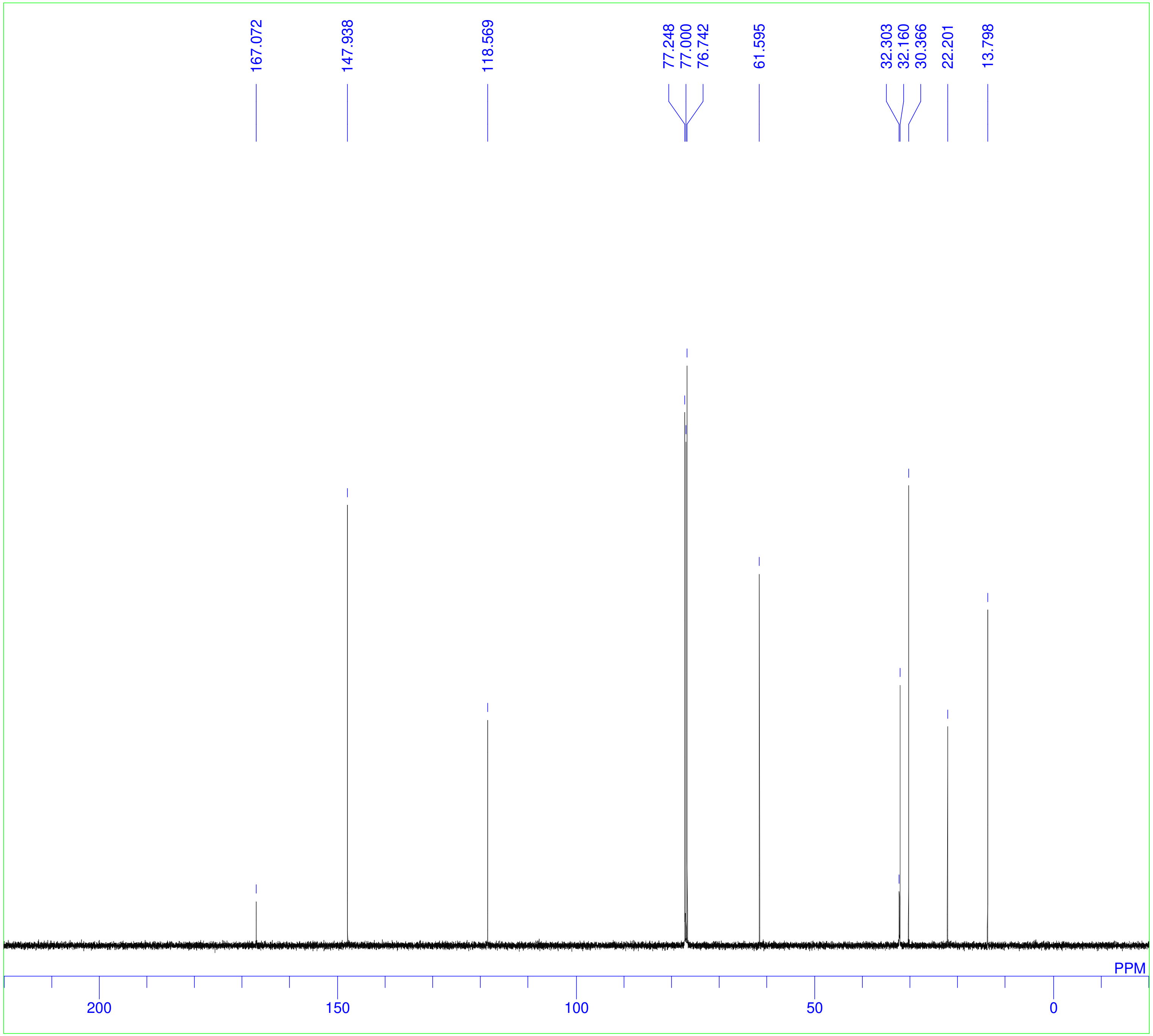

DFILE	(E)-16f_13C.als
COMNT	
DATIM	2023-05-05 10:31:54
OBNUC	13C
EXMOD	carbon.jxp
OBFRQ	125.77 MHz
OBSET	7.87 KHz
OBFIN	4.21 Hz
POINT	26214
FREQU	31446.54 Hz
SCANS	1024
ACQTM	0.8336 sec
PD	2.0000 sec
PW1	3.87 usec
IRNUC	1H
CTEMP	24.0 c
SLVNT	CDCL3
EXREF	77.00 ppm
BF	0.30 Hz
RGAIN	30

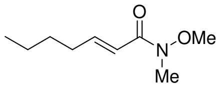

(*E*)-16f

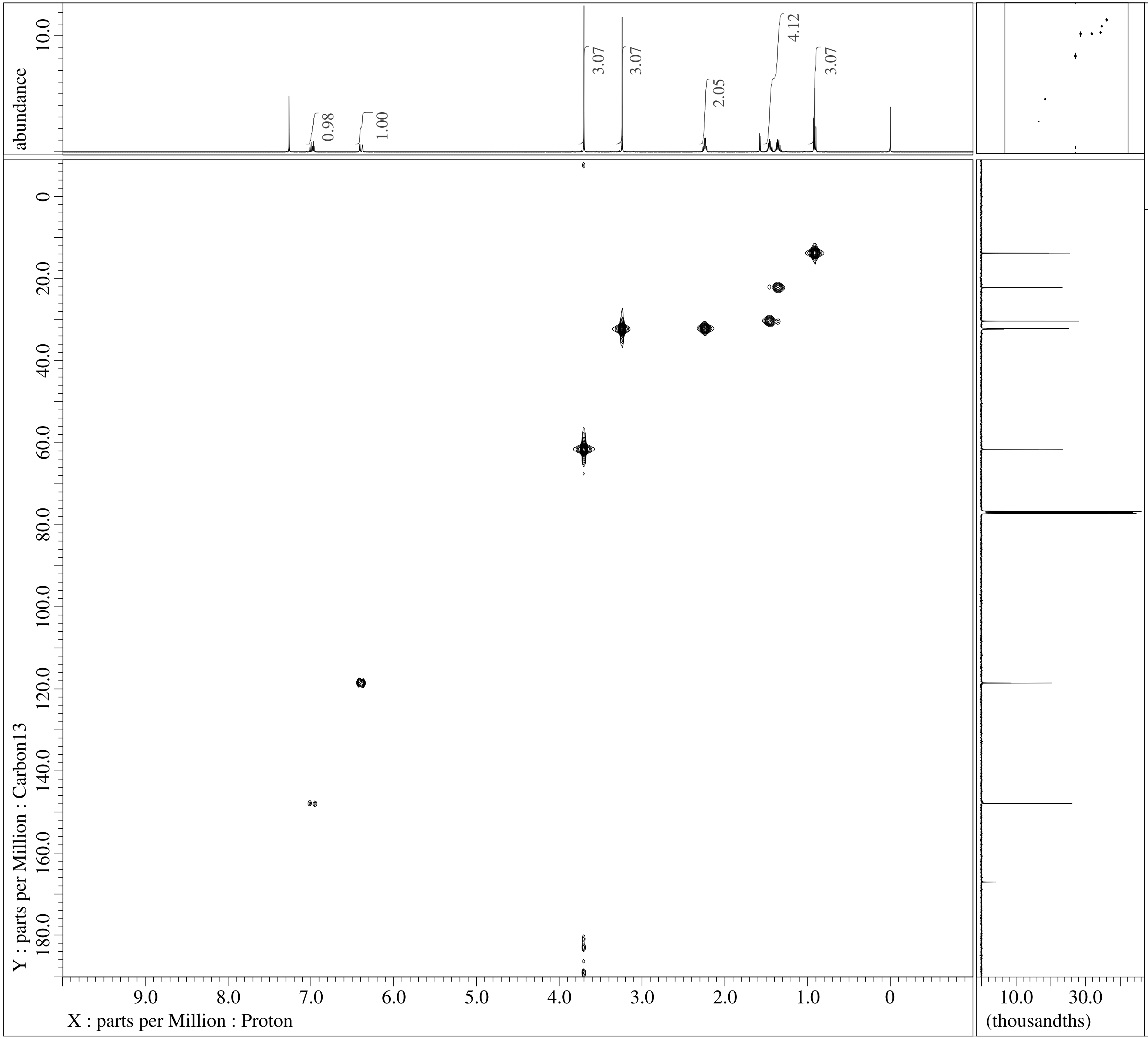


```
---- PROCESSING PARAMETERS ----
sinbell4( -60, 160 )
zerofill( 1 )
fft( 1, TRUE, TRUE )
ppm
[transpose]
sinbell4( -60, 160 )
zerofill( 2 )
fft( 1, TRUE, TRUE )
ppm
abs
```

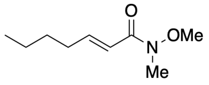

(*E*)-16f

```
Filename             = HT-1402-PTLC 2_13C_HMQC
Author               = delta
Experiment           = hmqc.jxp
Sample_Id            = HT-1402-PTLC 2_13C
Solvent              = CHLOROFORM-D
Creation_Time        =  5-MAY-2023 11:20:45
Revision_Time        = 10-APR-2024 19:19:04
Current_Time         = 10-APR-2024 19:20:18

Comment              = gradient enhanced HMQC
Data_Format          = 2D REAL REAL
Dim_Size             = 819, 512
Dim_Title            = Proton Carbon13
Dim_Units            = [ppm] [ppm]
Dimensions           = X Y
Site                 = JNM-ECA500II
Spectrometer         = DELTA2_NMR

Field_Strength       = 11.7473579[T] (500[MHz]
X_Acq_Duration       = 0.10911744[s]
X_Domain             = 1H
X_Freq               = 500.15991521[MHz]
X_Offset             = 5.0[ppm]
X_Points             = 1024
X_Prescans           = 4
X_Resolution         = 9.16443788[Hz]
X_Sweep              = 9.38438438[kHz]
X_Sweep_Clipped      = 7.50750751[kHz]
Y_Domain             = 13C
Y_Freq               = 125.76529768[MHz]
Y_Offset             = 90.54275035[ppm]
Y_Points             = 256
Y_Prescans           = 0
Y_Resolution         = 98.04844378[Hz]
Y_Sweep              = 25.10040161[kHz]
Tri_Domain           = Proton
Tri_Freq             = 500.15991521[MHz]
Tri_Offset           = 5.0[ppm]
Clipped              = FALSE
Scans                = 8
Total_Scans          = 2048

Relaxation_Delay     = 1.5[s]
Recvr_Gain           = 50
Temp_Get             = 23.9[dC]
X_Acq_Time           = 0.10911744[s]
X_Atn                = 3.2[dB]
X_Gamma              = 42576375
X_Pulse              = 7.68[us]
```

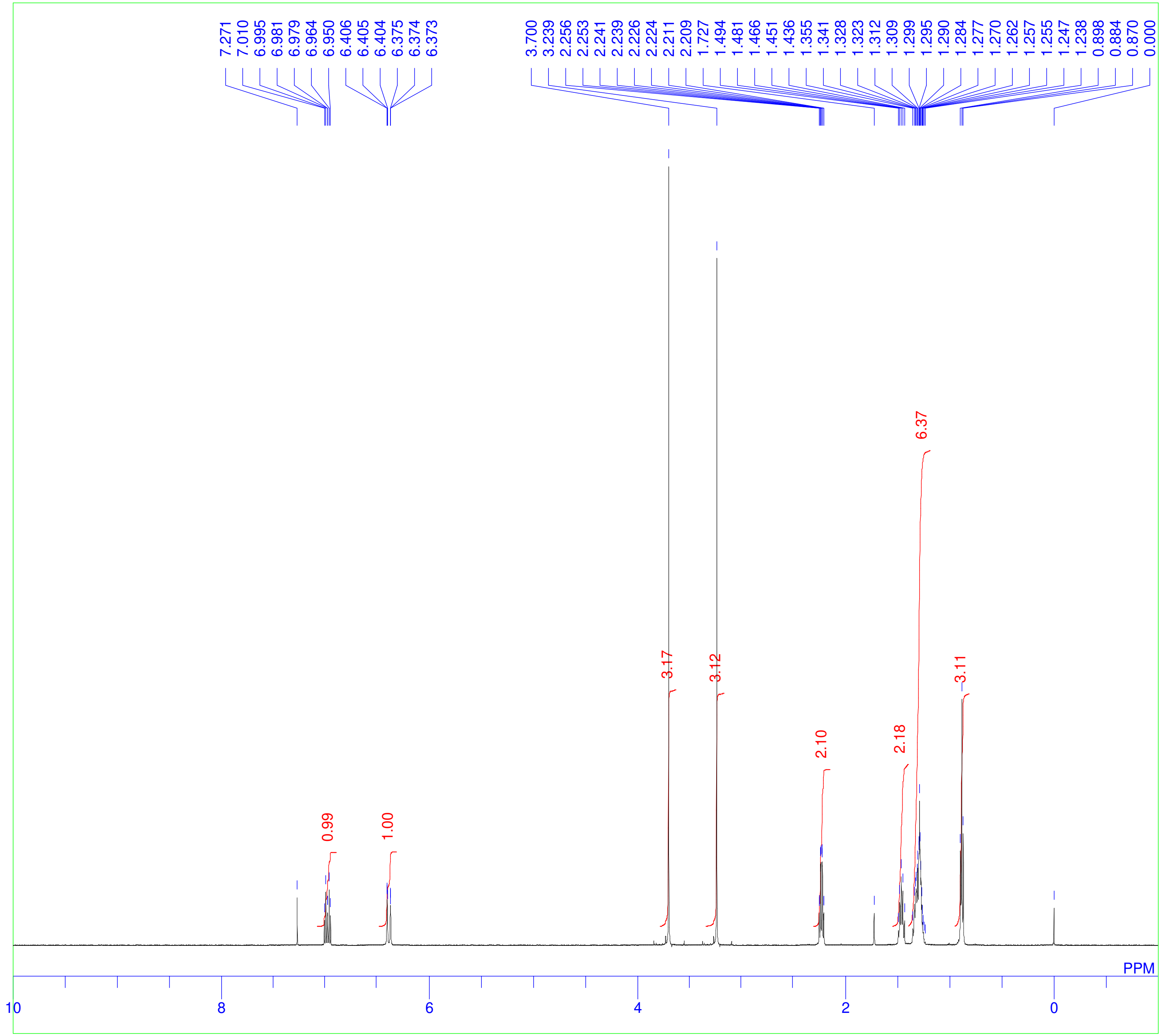

DFILE	(E)-16g_1H.als
COMNT	
DATIM	2023-05-04 10:41:46
OBNUC	1H
EXMOD	proton.jxp
OBFRQ	500.16 MHz
OBSET	2.41 KHz
OBFIN	6.01 Hz
POINT	13107
FREQU	7507.51 Hz
SCANS	8
ACQTM	1.7459 sec
PD	5.0000 sec
PW1	3.84 usec
IRNUC	1H
CTEMP	24.0 c
SLVNT	CDCL3
EXREF	0.00 ppm
BF	0.30 Hz
RGAIN	30

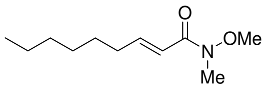

(*E*)-16g ((*E*)-5a)

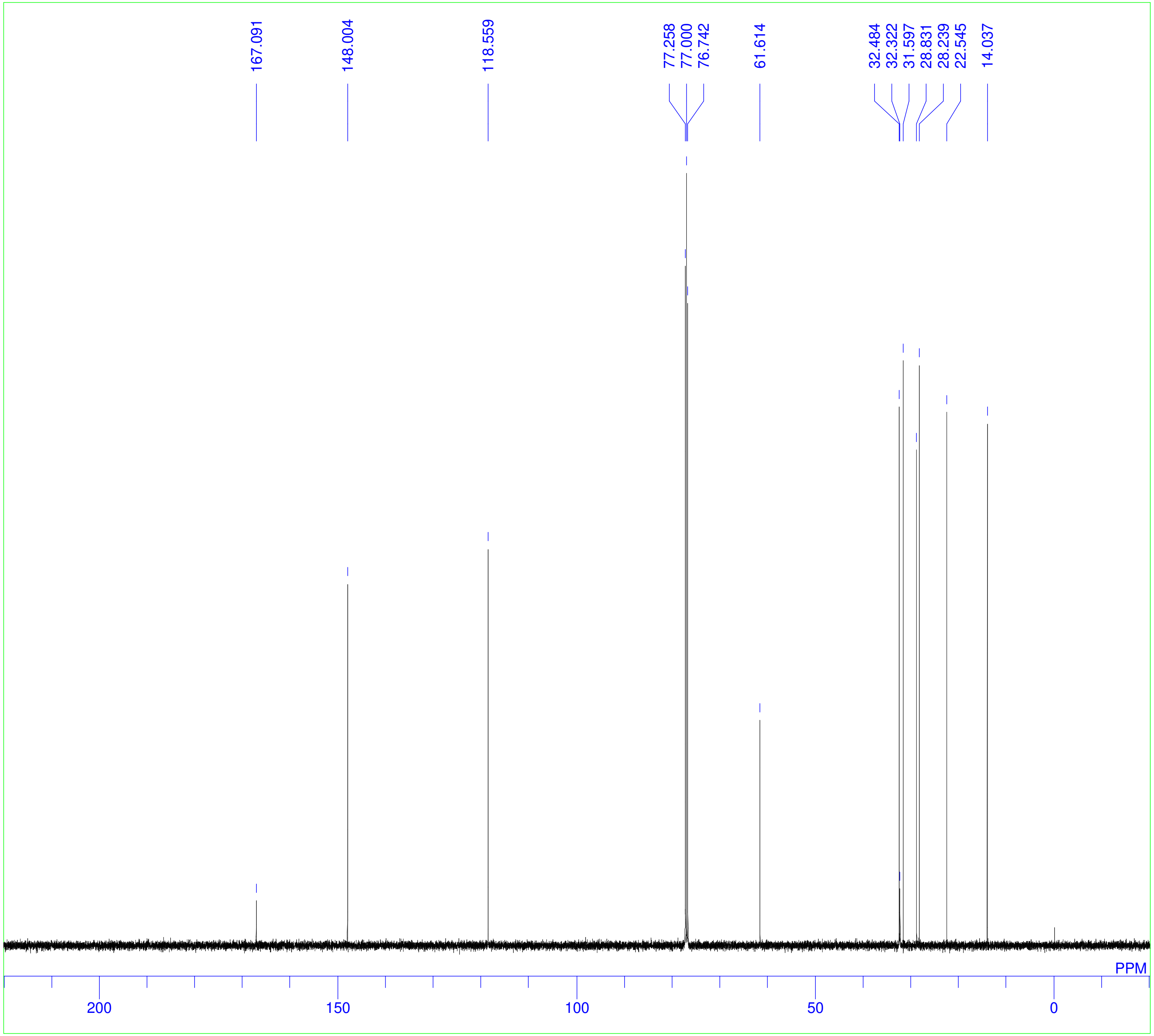

DFILE	(E)-16g_13C.als
COMNT	
DATIM	2023-05-04 10:44:02
OBNUC	13C
EXMOD	carbon.jxp
OBFRQ	125.77 MHz
OBSET	7.87 KHz
OBFIN	4.21 Hz
POINT	26214
FREQU	31446.54 Hz
SCANS	1024
ACQTM	0.8336 sec
PD	2.0000 sec
PW1	3.87 usec
IRNUC	1H
CTEMP	23.8 c
SLVNT	CDCL3
EXREF	77.00 ppm
BF	0.30 Hz
RGAIN	30

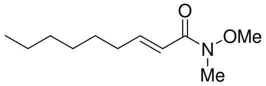

(*E*)-16g ((*E*)-5a)

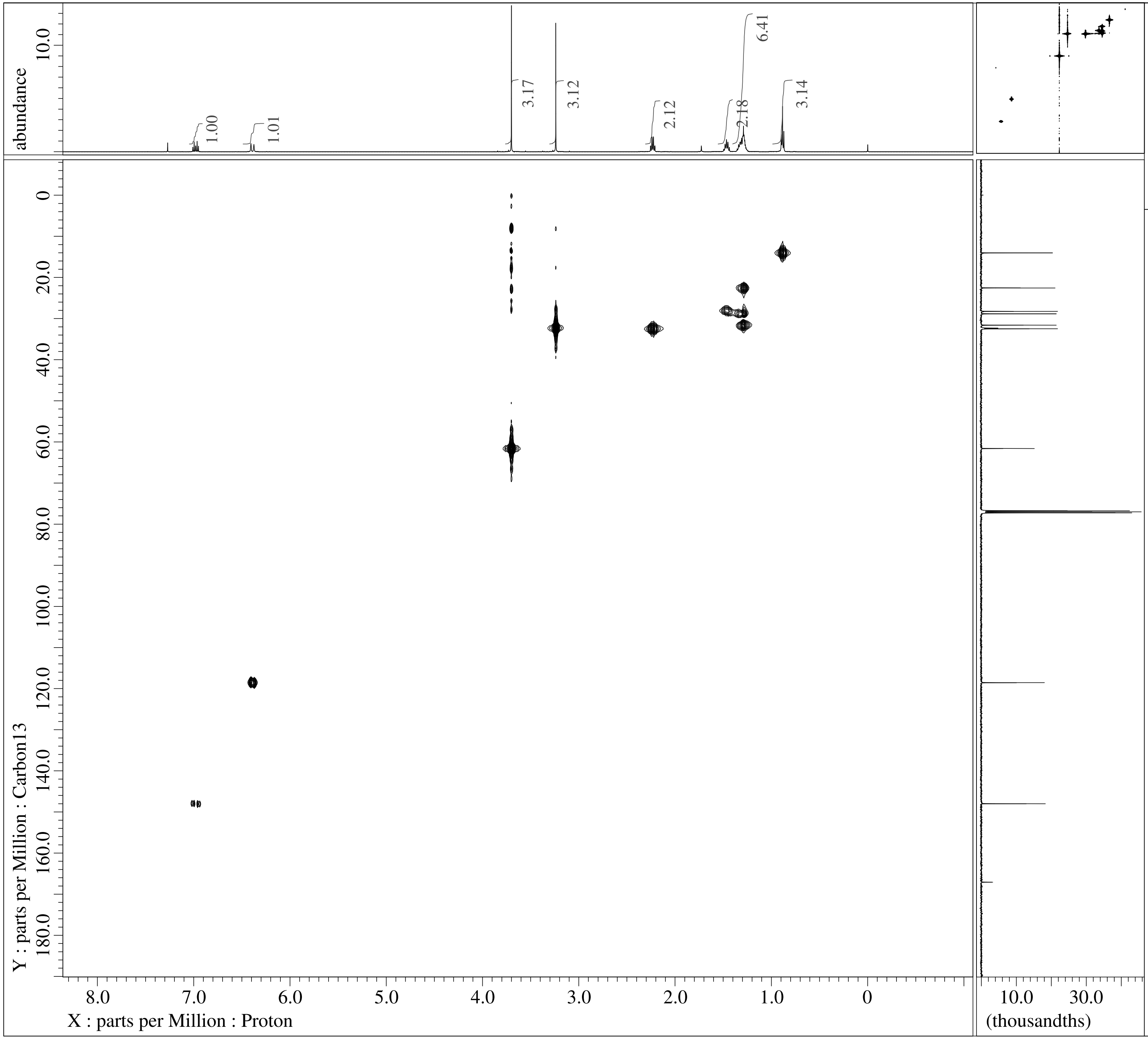


```
---- PROCESSING PARAMETERS ----
sinbell4( -60, 160 )
zerofill( 1 )
fft( 1, TRUE, TRUE )
ppm
[transpose]
sinbell4( -60, 160 )
zerofill( 2 )
fft( 1, TRUE, TRUE )
ppm
abs
```

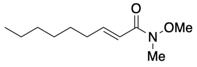

(*E*)-16g ((*E*)-5a)

```
Filename           = HT-1399-PTLC 2_HMQC-1-5
Author             = delta
Experiment         = hmqc.jxp
Sample_Id          = HT-1399-PTLC 2
Solvent            = CHLOROFORM-D
Creation_Time      =  4-MAY-2023 12:38:55
Revision_Time      = 10-APR-2024 19:22:14
Current_Time       = 10-APR-2024 19:23:07

Comment            = gradient enhanced HMQC
Data_Format        = 2D REAL REAL
Dim_Size           = 819, 512
Dim_Title          = Proton Carbon13
Dim_Units          = [ppm] [ppm]
Dimensions         = X Y
Site               = JNM-ECA500II
Spectrometer       = DELTA2_NMR

Field_Strength     = 11.7473579[T] (500[MHz]
X_Acq_Duration     = 0.17301504[s]
X_Domain           = 1H
X_Freq             = 500.15991521[MHz]
X_Offset           = 3.60916861[ppm]
X_Points           = 1024
X_Prescans         = 4
X_Resolution       = 5.77984434[Hz]
X_Sweep            = 5.91856061[kHz]
X_Sweep_Clipped    = 4.73484848[kHz]
Y_Domain           = 13C
Y_Freq             = 125.76529768[MHz]
Y_Offset           = 90.66197942[ppm]
Y_Points           = 256
Y_Prescans         = 0
Y_Resolution       = 97.85195391[Hz]
Y_Sweep            = 25.0501002[kHz]
Tri_Domain         = Proton
Tri_Freq           = 500.15991521[MHz]
Tri_Offset         = 5.0[ppm]
Clipped            = FALSE
Scans              = 8
Total_Scans        = 2048

Relaxation_Delay   = 1.5[s]
Recvr_Gain         = 50
Temp_Get           = 24.1[dC]
X_Acq_Time         = 0.17301504[s]
X_Atn              = 3.2[dB]
X_Gamma            = 42576375
X_Pulse            = 7.68[us]
```

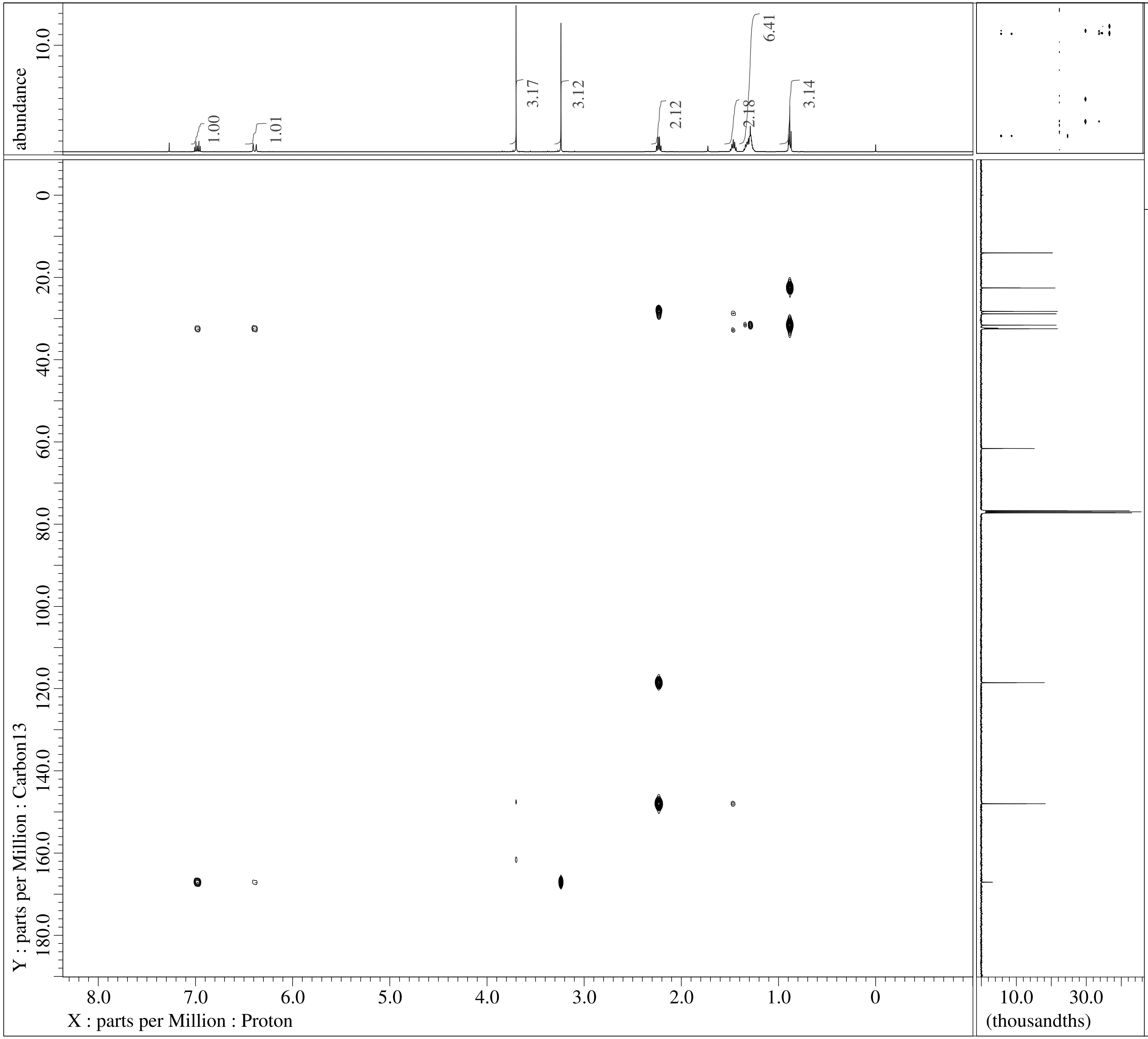


```
---- PROCESSING PARAMETERS ----
gauss( 5.0[Hz], 0.0[s] )
sinbell_auto
zerofill( 1 )
fft( 1, TRUE, TRUE )
ppm
[transpose]
sinbell4( -60, 160 )
trapezoid( 0[%], 5[%], 80[%], 100[%] )
zerofill( 2 )
fft( 1, TRUE, TRUE )

Filename            = HT-1399-PTLC 2_HMBC-1-5
Author              = delta
Experiment          = hmbc.jxp
Sample_Id           = HT-1399-PTLC 2
Solvent             = CHLOROFORM-D
Creation_Time       =  4-MAY-2023 11:32:54
Revision_Time       = 10-APR-2024 19:24:25
Current_Time        = 10-APR-2024 19:25:31

Comment             = gradient enhanced HMBC
Data_Format         = 2D REAL REAL
Dim_Size            = 1638, 512
Dim_Title           = Proton Carbon13
Dim_Units           = [ppm] [ppm]
Dimensions          = X Y
Site                = JNM-ECA500II
Spectrometer        = DELTA2_NMR

Field_Strength      = 11.7473579[T] (500[MHz]
X_Acq_Duration      = 0.34603008[s]
X_Domain            = 1H
X_Freq              = 500.15991521[MHz]
X_Offset            = 3.60916861[ppm]
X_Points            = 2048
X_Prescans          = 4
X_Resolution        = 2.88992217[Hz]
X_Sweep             = 5.91856061[kHz]
X_Sweep_Clipped     = 4.73484848[kHz]
Y_Domain            = 13C
Y_Freq              = 125.76529768[MHz]
Y_Offset            = 90.66197942[ppm]
Y_Points            = 256
Y_Prescans          = 0
Y_Resolution        = 97.85195391[Hz]
Y_Sweep             = 25.0501002[kHz]
Tri_Domain          = Proton
Tri_Freq            = 500.15991521[MHz]
Tri_Offset          = 5.0[ppm]
Clipped             = FALSE
Scans               = 8
Total_Scans         = 2048

Relaxation_Delay    = 1.5[s]
Recvr_Gain          = 50
Temp_Get            = 23.9[dC]
X_Acq_Time          = 0.34603008[s]
X_Atn               = 3.2[dB]
X_Gamma             = 42576375
X_Pulse             = 7.68[us]
```

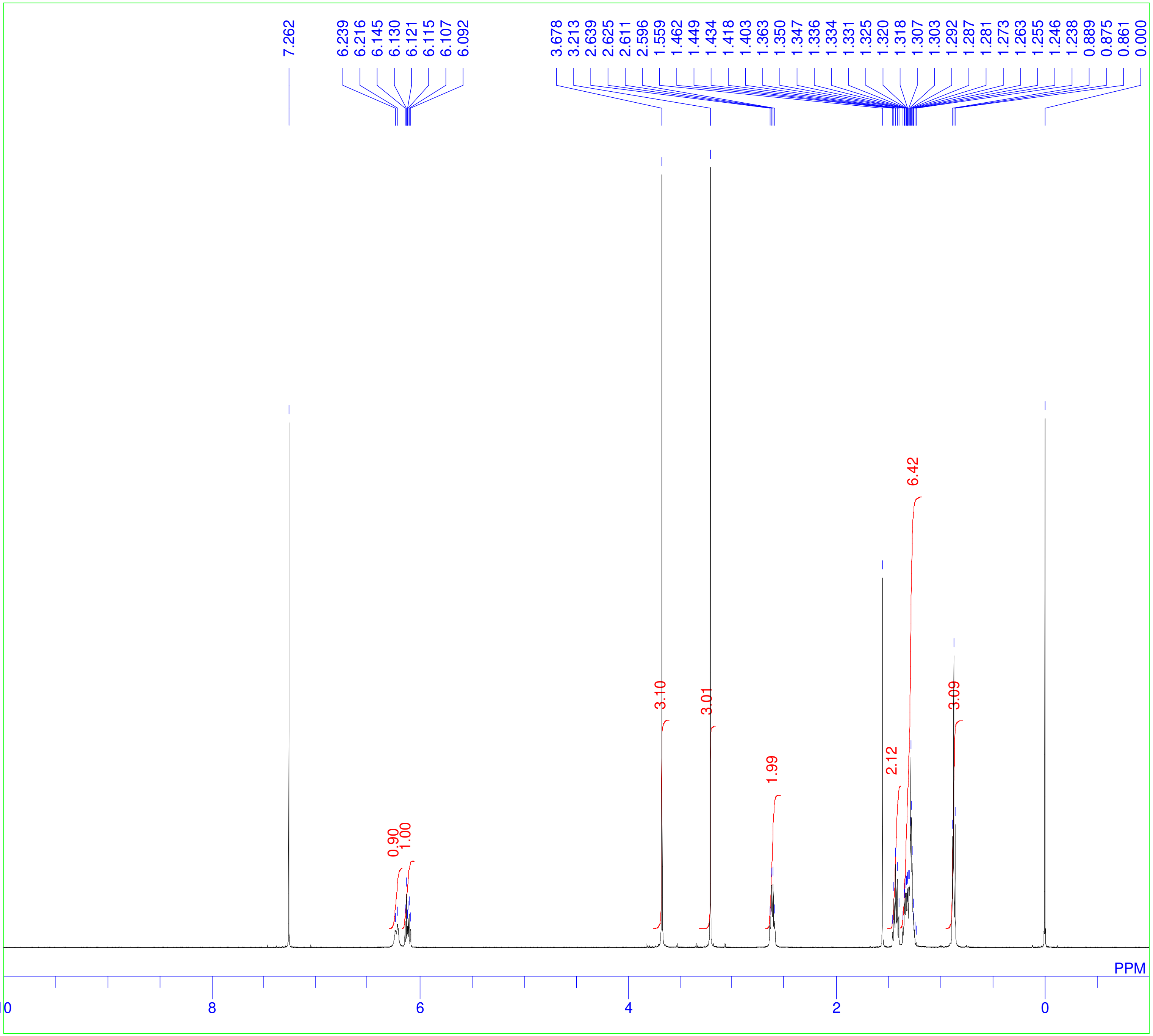

DFILE	(Z)-16g_1H.als
COMNT	
DATIM	2023-05-04 09:44:50
OBNUC	1H
EXMOD	proton.jxp
OBFRQ	500.16 MHz
OBSET	2.41 KHz
OBFIN	6.01 Hz
POINT	13107
FREQU	7507.51 Hz
SCANS	8
ACQTM	1.7459 sec
PD	5.0000 sec
PW1	3.84 usec
IRNUC	1H
CTEMP	23.8 c
SLVNT	CDCL3
EXREF	0.00 ppm
BF	0.30 Hz
RGAIN	44

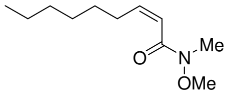

(*Z*)-16g ((*Z*)-5a)

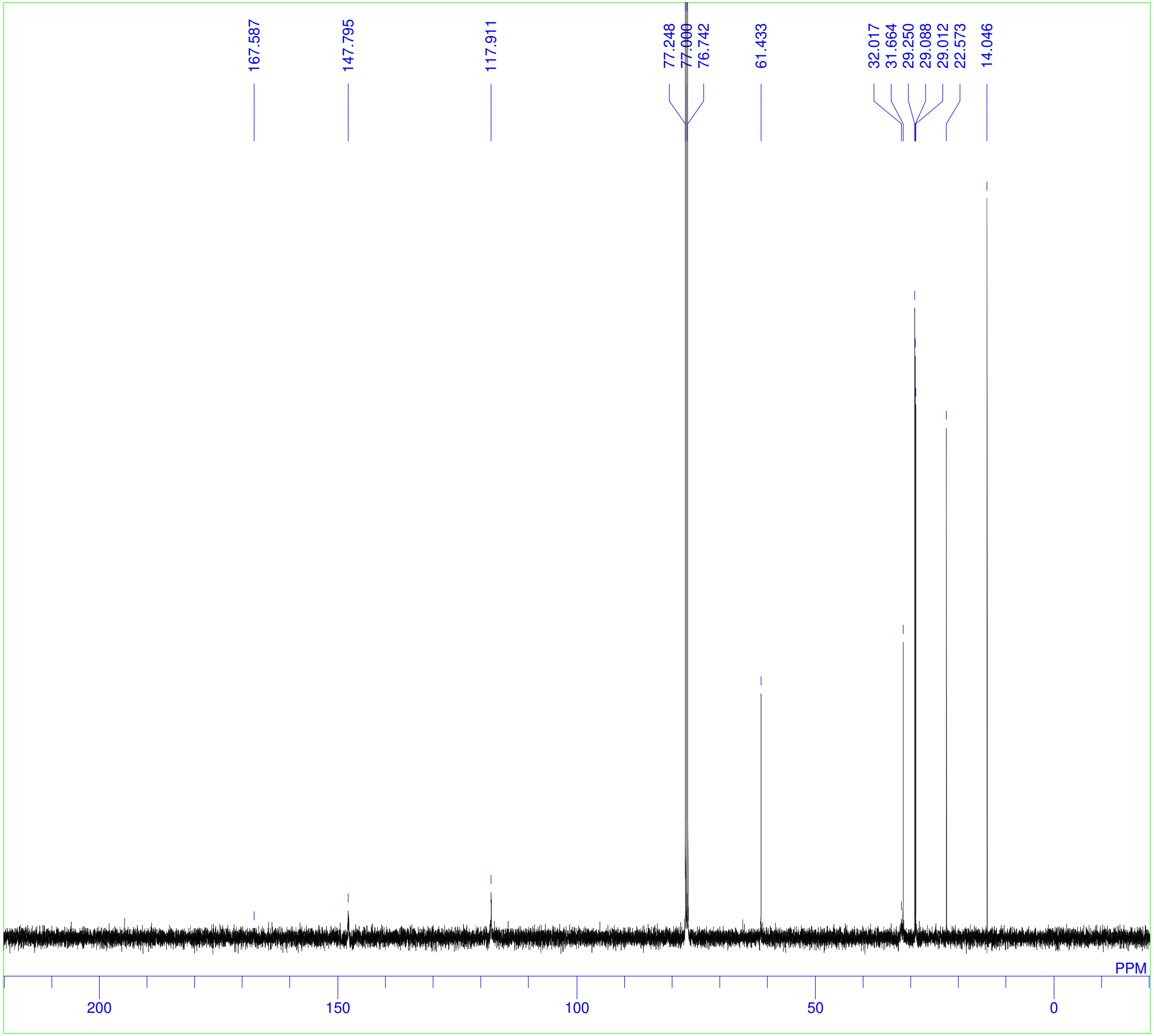

DFILE (Z)-16g_13C.als
COMNT
DATIM 2023-05-06 08:24:34
OBNUC 13C
EXMOD carbon.jxp
OBFRQ 125.77 MHz
OBSET 7.87 KHz
OBFIN 4.21 Hz
POINT 26214
FREQU 31446.54 Hz
SCANS 1024
ACQTM 0.8336 sec
PD 2.0000 sec
PW1 3.87 usec
IRNUC 1H
CTEMP 24.1 c
SLVNT CDCL3
EXREF 77.00 ppm
BF 0.30 Hz
RGAIN 30

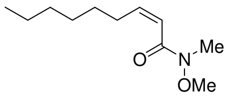

(*Z*)-16g ((*Z*)-5a)

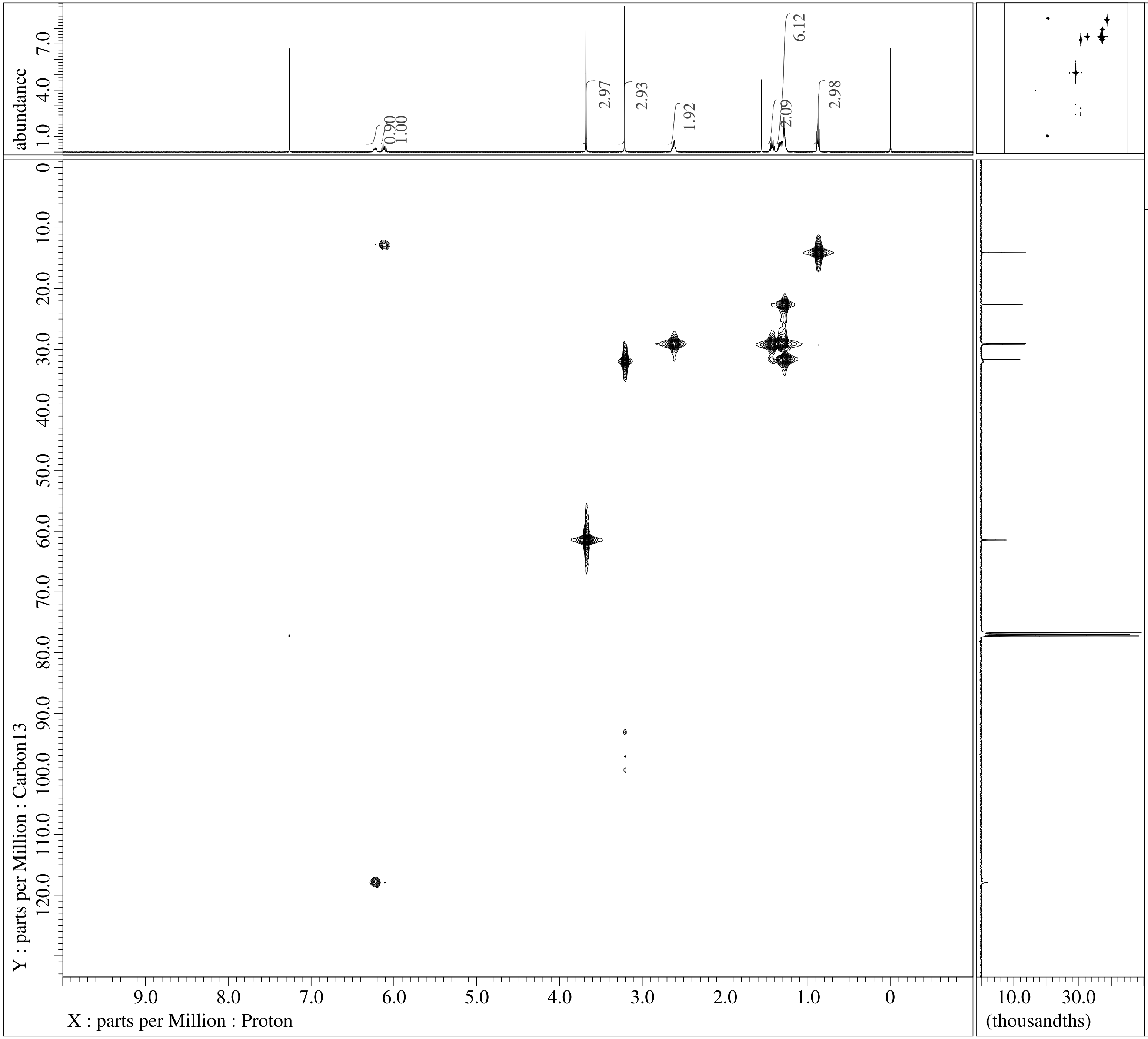


```
---- PROCESSING PARAMETERS ----
sinbell4( -60, 160 )
zerofill( 1 )
fft( 1, TRUE, TRUE )
ppm
[transpose]
sinbell4( -60, 160 )
zerofill( 2 )
fft( 1, TRUE, TRUE )
ppm
abs
```

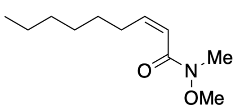

(*Z*)-16g ((*Z*)-5a)

```
Filename            = HT-1398-PTLC 1_13C_HMQC
Author              = delta
Experiment          = hmqc.jxp
Sample_Id           = HT-1398-PTLC 1_13C
Solvent             = CHLOROFORM-D
Creation_Time       =  6-MAY-2023 09:13:25
Revision_Time       = 10-APR-2024 19:27:43
Current_Time        = 10-APR-2024 19:30:34

Comment             = gradient enhanced HMQC
Data_Format         = 2D REAL REAL
Dim_Size            = 819, 512
Dim_Title           = Proton Carbon13
Dim_Units           = [ppm] [ppm]
Dimensions          = X Y
Site                = JNM-ECA500II
Spectrometer        = DELTA2_NMR

Field_Strength      = 11.7473579[T] (500[MHz]
X_Acq_Duration      = 0.10911744[s]
X_Domain            = 1H
X_Freq              = 500.15991521[MHz]
X_Offset            = 5.0[ppm]
X_Points            = 1024
X_Prescans          = 4
X_Resolution        = 9.16443788[Hz]
X_Sweep             = 9.38438438[kHz]
X_Sweep_Clipped     = 7.50750751[kHz]
Y_Domain            = 13C
Y_Freq              = 125.76529768[MHz]
Y_Offset            = 66.07694566[ppm]
Y_Points            = 256
Y_Prescans          = 0
Y_Resolution        = 66.34256114[Hz]
Y_Sweep             = 16.98369565[kHz]
Tri_Domain          = Proton
Tri_Freq            = 500.15991521[MHz]
Tri_Offset          = 5.0[ppm]
Clipped             = FALSE
Scans               = 8
Total_Scans         = 2048

Relaxation_Delay    = 1.5[s]
Recvr_Gain          = 50
Temp_Get            = 24[dC]
X_Acq_Time          = 0.10911744[s]
X_Atn               = 3.2[dB]
X_Gamma             = 42576375
X_Pulse             = 7.68[us]
```

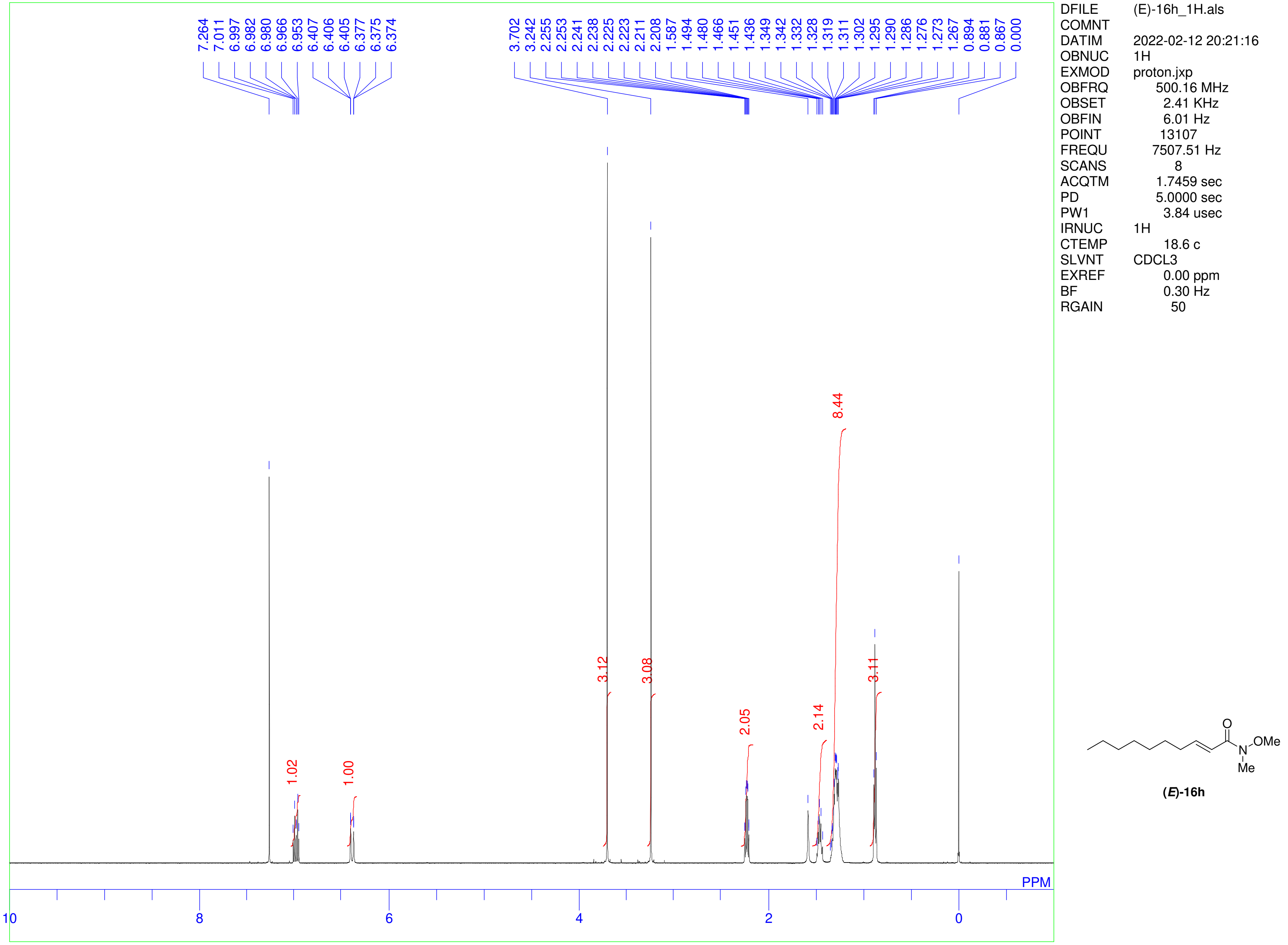

DFILE (E)-16h_1H.als
COMNT
DATIM 2022-02-12 20:21:16
OBNUC 1H
EXMOD proton.jxp
OBFRQ 500.16 MHz
OBSET 2.41 KHz
OBFIN 6.01 Hz
POINT 13107
FREQU 7507.51 Hz
SCANS 8
ACQTM 1.7459 sec
PD 5.0000 sec
PW1 3.84 usec
IRNUC 1H
CTEMP 18.6 c
SLVNT CDCL3
EXREF 0.00 ppm
BF 0.30 Hz
RGAIN 50

(*E*)-16h

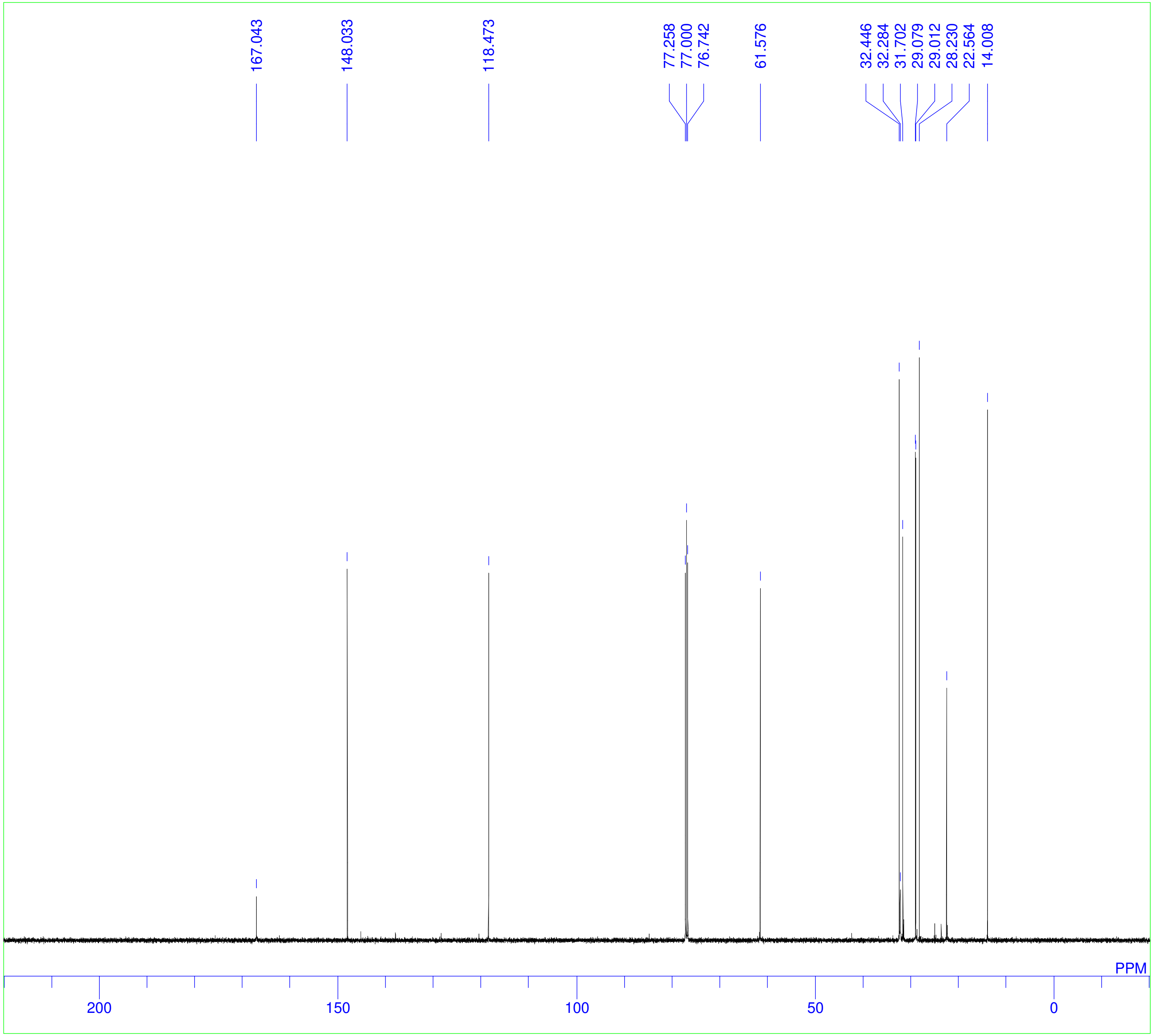

DFILE	(E)-16h_13C.als
COMNT	
DATIM	2023-01-04 15:06:24
OBNUC	13C
EXMOD	carbon.jxp
OBFRQ	125.77 MHz
OBSET	7.87 KHz
OBFIN	4.21 Hz
POINT	26214
FREQU	31446.54 Hz
SCANS	1024
ACQTM	0.8336 sec
PD	2.0000 sec
PW1	3.87 usec
IRNUC	1H
CTEMP	21.3 c
SLVNT	CDCL3
EXREF	77.00 ppm
BF	0.30 Hz
RGAIN	26

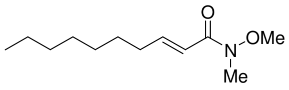

(*E*)-16h

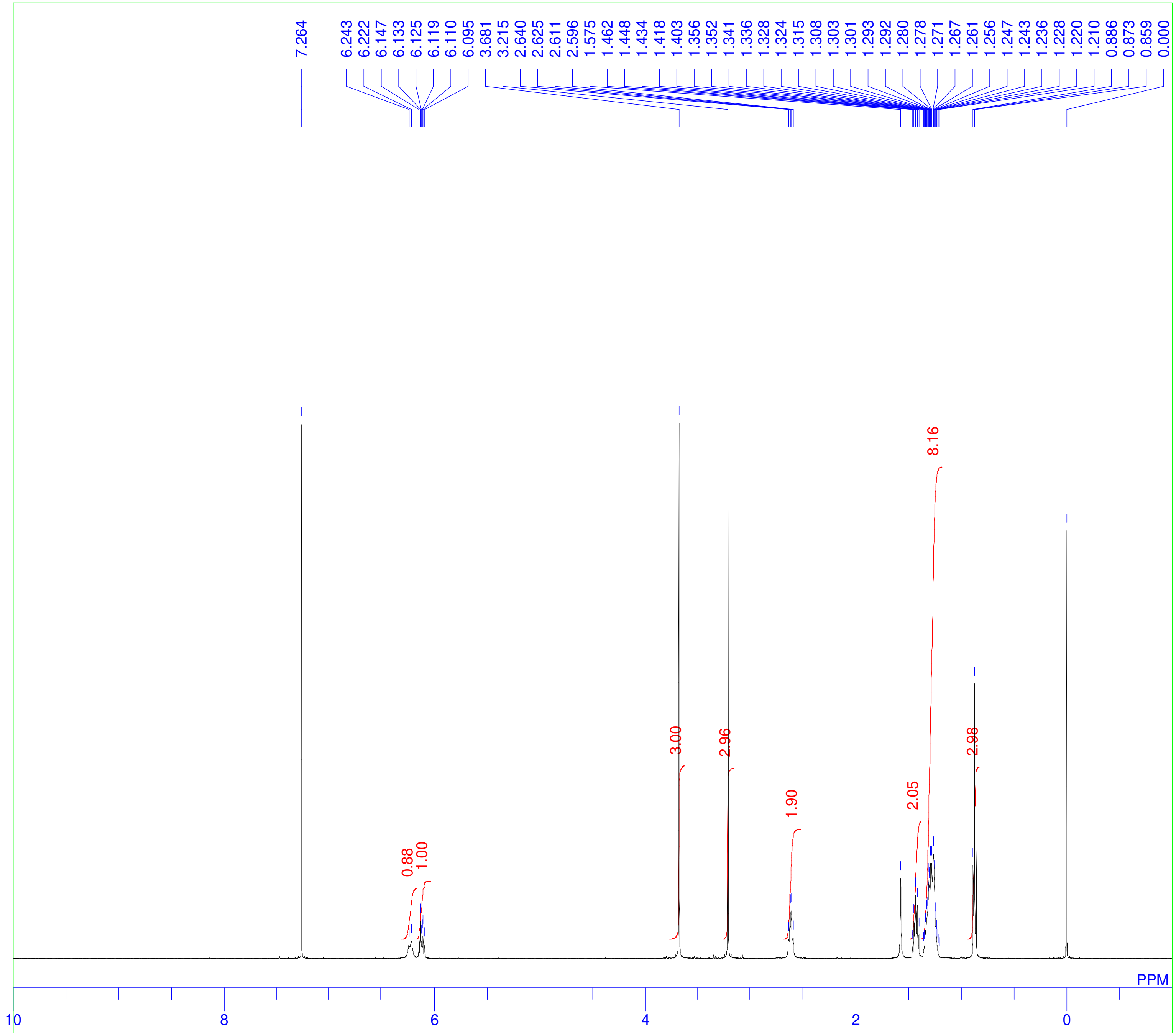

DFILE	(Z)-16h_1H.als
COMNT	
DATIM	2022-02-12 20:15:43
OBNUC	1H
EXMOD	proton.jxp
OBFRQ	500.16 MHz
OBSET	2.41 KHz
OBFIN	6.01 Hz
POINT	13107
FREQU	7507.51 Hz
SCANS	8
ACQTM	1.7459 sec
PD	5.0000 sec
PW1	3.84 usec
IRNUC	1H
CTEMP	18.6 c
SLVNT	CDCL3
EXREF	0.00 ppm
BF	0.30 Hz
RGAIN	50

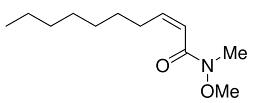

(*Z*)-16h

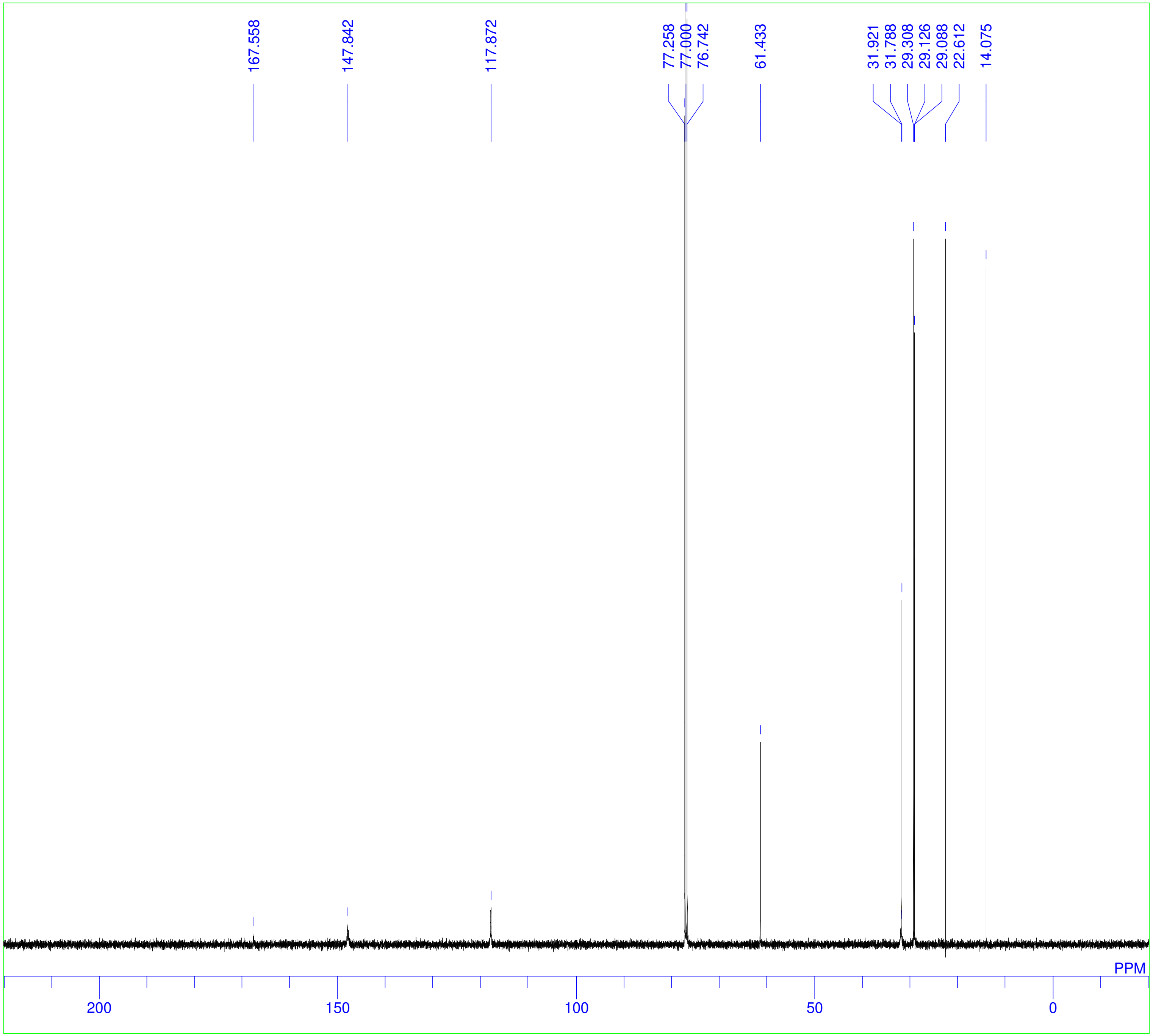
167.558
147.842
117.872
77.258
77.000
76.742
61.433
31.921
31.788
29.308
29.126
29.088
22.612
14.075
PPM
200
150
100
50
0
DFILE (Z)-16h_13C.als
COMNT
DATIM 2022-02-28 18:56:07
OBNUC 13C
EXMOD carbon.jxp
OBFRQ 125.77 MHz
OBSET 7.87 KHz
OBFIN 4.21 Hz
POINT 26214
FREQU 31446.54 Hz
SCANS 1700
ACQTM 0.8336 sec
PD 2.0000 sec
PW1 3.87 usec
IRNUC 1H
CTEMP 21.1 c
SLVNT CDCL3
EXREF 77.00 ppm
BF 0.30 Hz
RGAIN 26

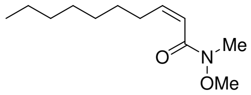
O
N
Me
OMe

(*Z*)-16h

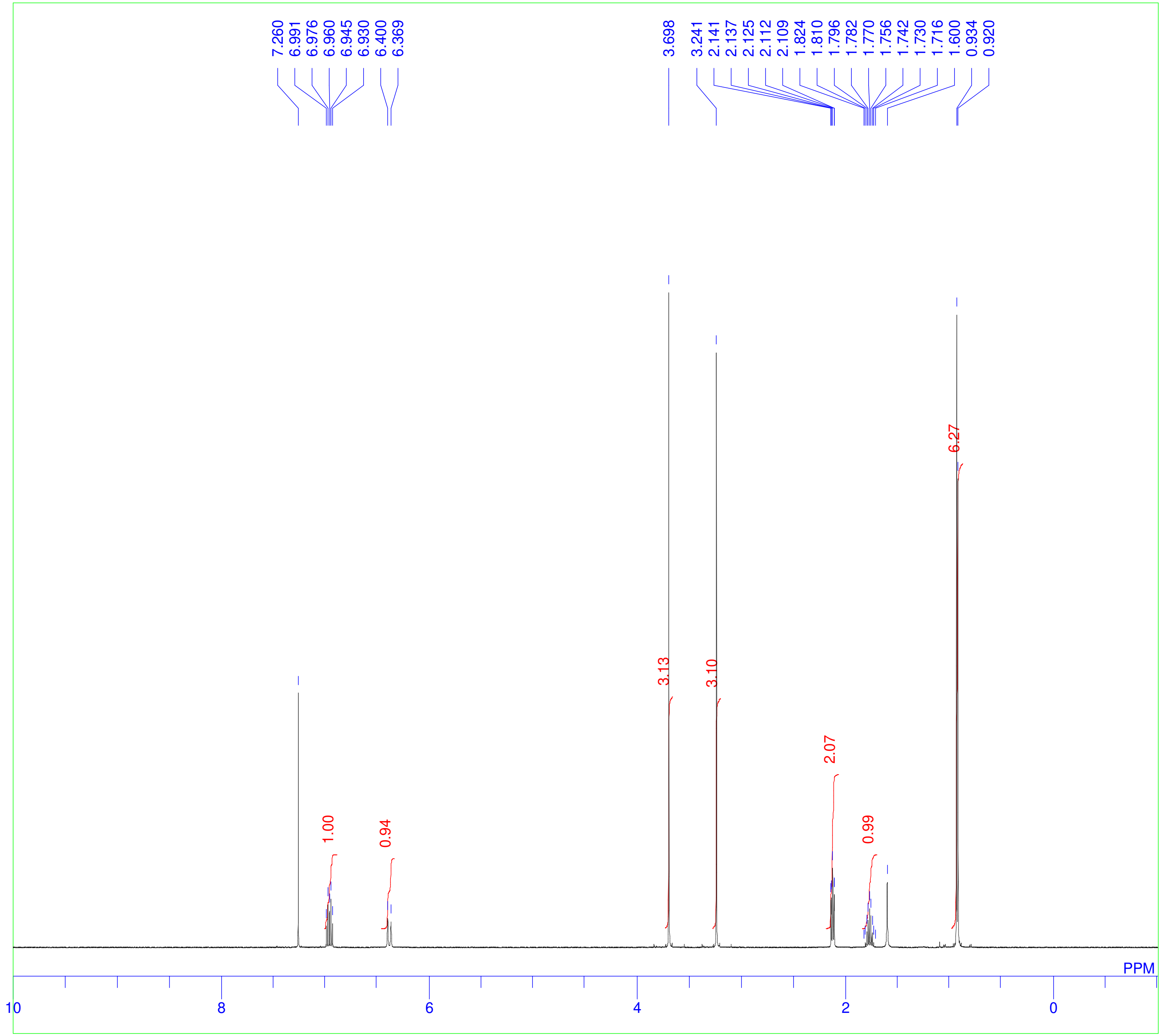

DFILE	(E)-16i_1H.als
COMNT	
DATIM	2022-01-12 15:42:00
OBNUC	1H
EXMOD	proton.jxp
OBFRQ	500.16 MHz
OBSET	2.41 KHz
OBFIN	6.01 Hz
POINT	13107
FREQU	7507.51 Hz
SCANS	8
ACQTM	1.7459 sec
PD	5.0000 sec
PW1	3.84 usec
IRNUC	1H
CTEMP	19.5 c
SLVNT	CDCL3
EXREF	7.26 ppm
BF	0.30 Hz
RGAIN	42

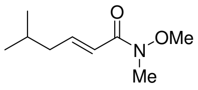

(*E*)-16i

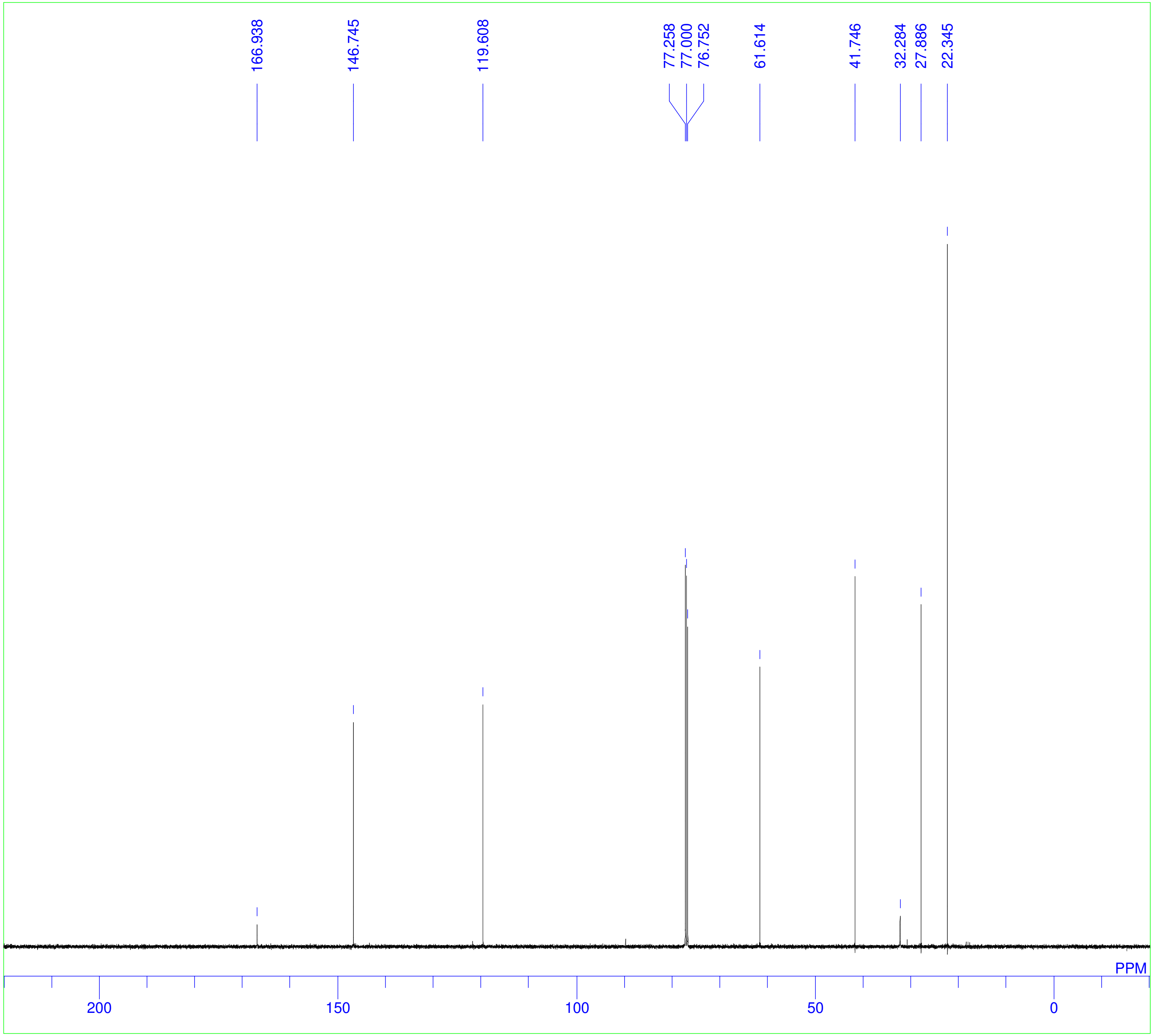

DFILE	(E)-16i_13C.als
COMNT	
DATIM	2023-01-09 15:53:39
OBNUC	13C
EXMOD	carbon.jxp
OBFRQ	125.77 MHz
OBSET	7.87 KHz
OBFIN	4.21 Hz
POINT	26214
FREQU	31446.54 Hz
SCANS	1024
ACQTM	0.8336 sec
PD	2.0000 sec
PW1	3.87 usec
IRNUC	1H
CTEMP	22.2 c
SLVNT	CDCL3
EXREF	77.00 ppm
BF	0.30 Hz
RGAIN	28

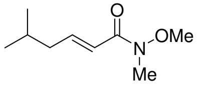

(*E*)-16i

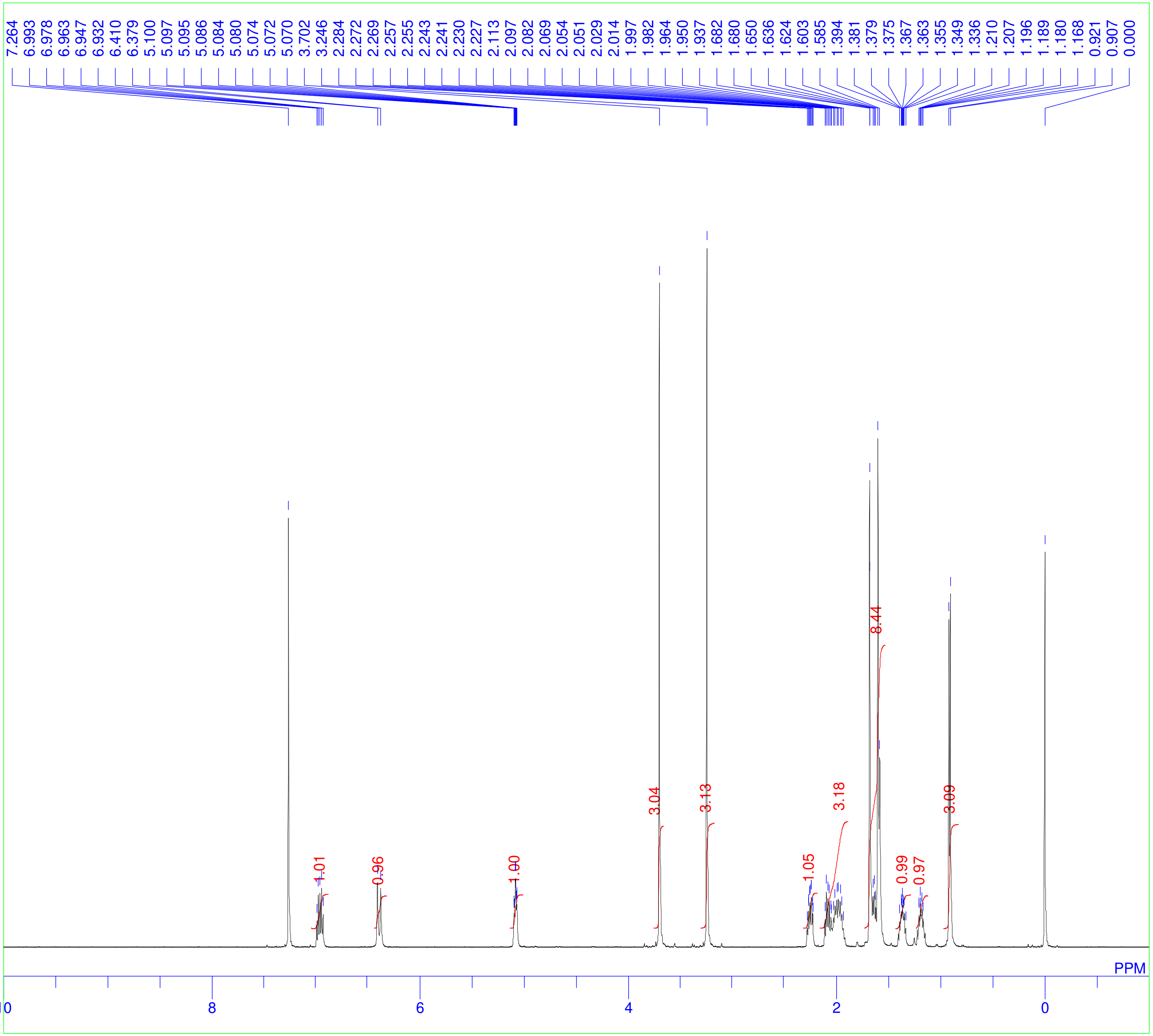

DFILE (E)-16j_1H.als
COMNT
DATIM 2022-01-15 08:19:35
OBNUC 1H
EXMOD proton.jxp
OBFRQ 500.16 MHz
OBSET 2.41 KHz
OBFIN 6.01 Hz
POINT 13107
FREQU 7507.51 Hz
SCANS 8
ACQTM 1.7459 sec
PD 5.0000 sec
PW1 3.84 usec
IRNUC 1H
CTEMP 18.5 c
SLVNT CDCL3
EXREF 0.00 ppm
BF 0.30 Hz
RGAIN 42

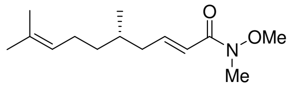

(*E*)-16j

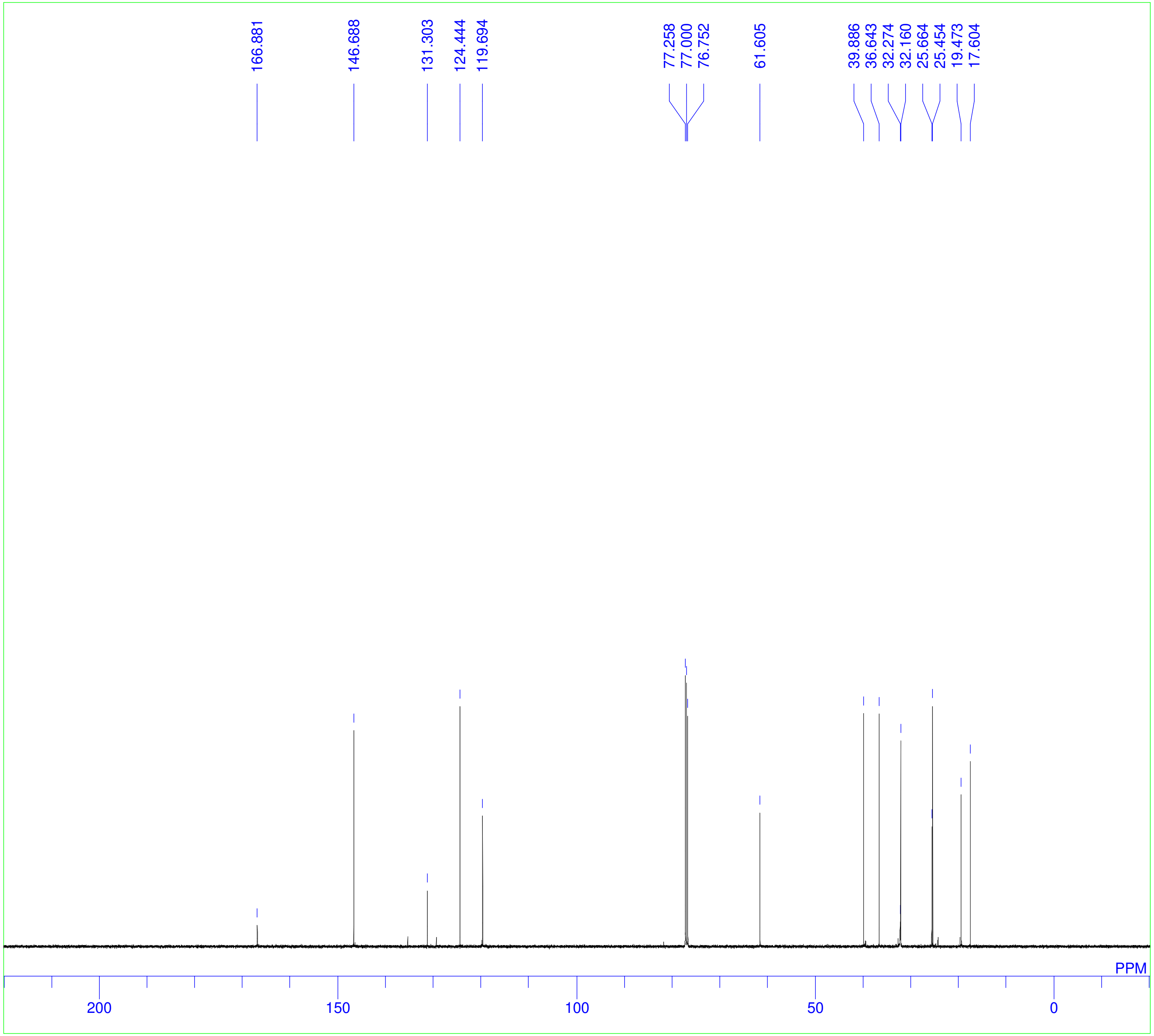

DFILE	(E)-16j_13C.als
COMNT	
DATIM	2023-01-06 14:18:58
OBNUC	13C
EXMOD	carbon.jxp
OBFRQ	125.77 MHz
OBSET	7.87 KHz
OBFIN	4.21 Hz
POINT	26214
FREQU	31446.54 Hz
SCANS	1024
ACQTM	0.8336 sec
PD	2.0000 sec
PW1	3.87 usec
IRNUC	1H
CTEMP	20.9 c
SLVNT	CDCL3
EXREF	77.00 ppm
BF	0.30 Hz
RGAIN	26

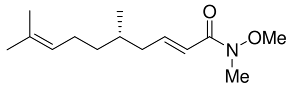

(*E*)-16j

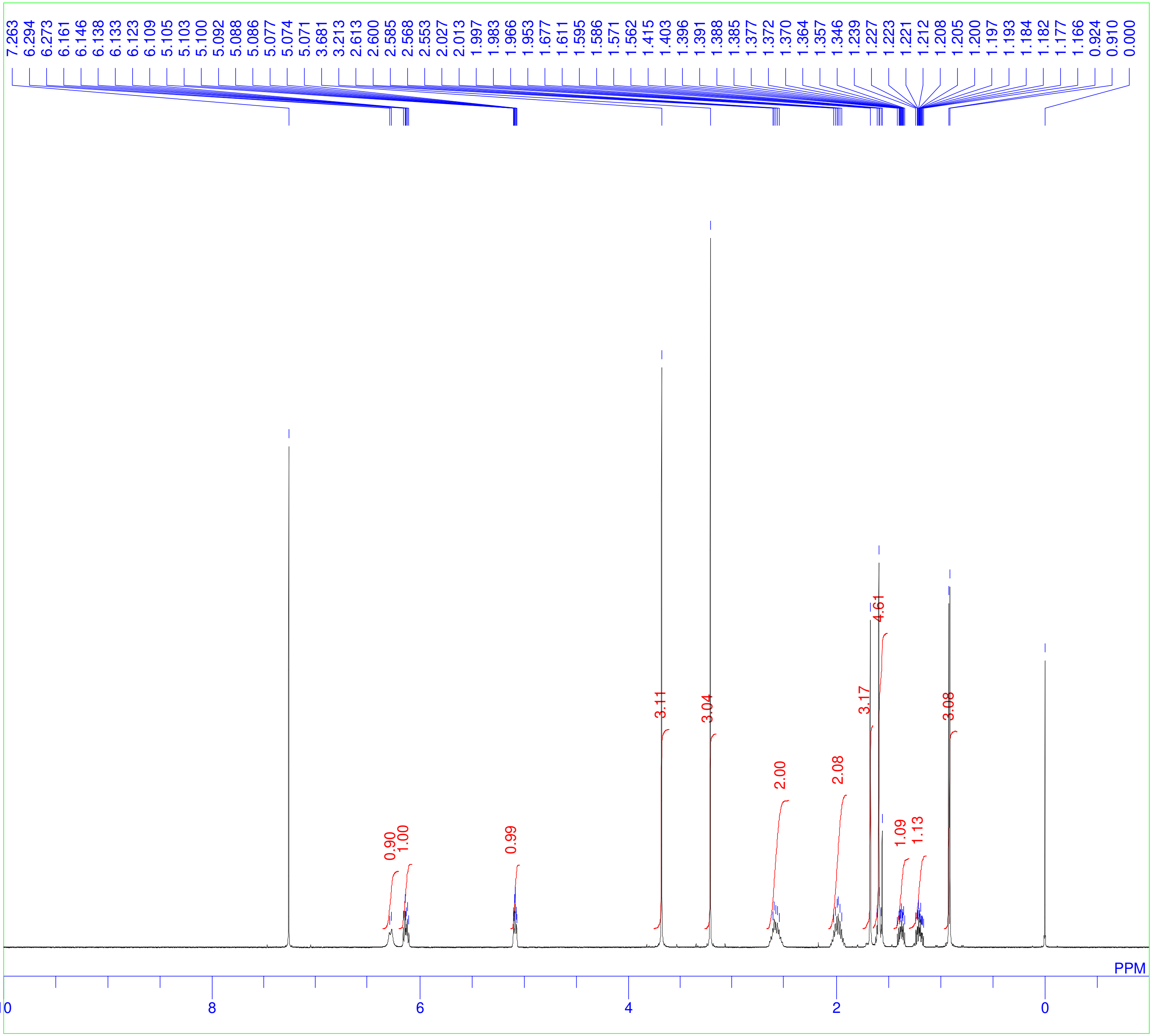

DFILE	(Z)-16j_1H.als
COMNT	
DATIM	2023-01-27 19:59:14
OBNUC	1H
EXMOD	proton.jxp
OBFRQ	500.16 MHz
OBSET	2.41 KHz
OBFIN	6.01 Hz
POINT	13107
FREQU	7507.51 Hz
SCANS	8
ACQTM	1.7459 sec
PD	5.0000 sec
PW1	3.84 usec
IRNUC	1H
CTEMP	19.7 c
SLVNT	CDCL3
EXREF	0.00 ppm
BF	0.30 Hz
RGAIN	44

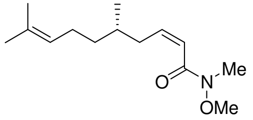

(*Z*)-16j

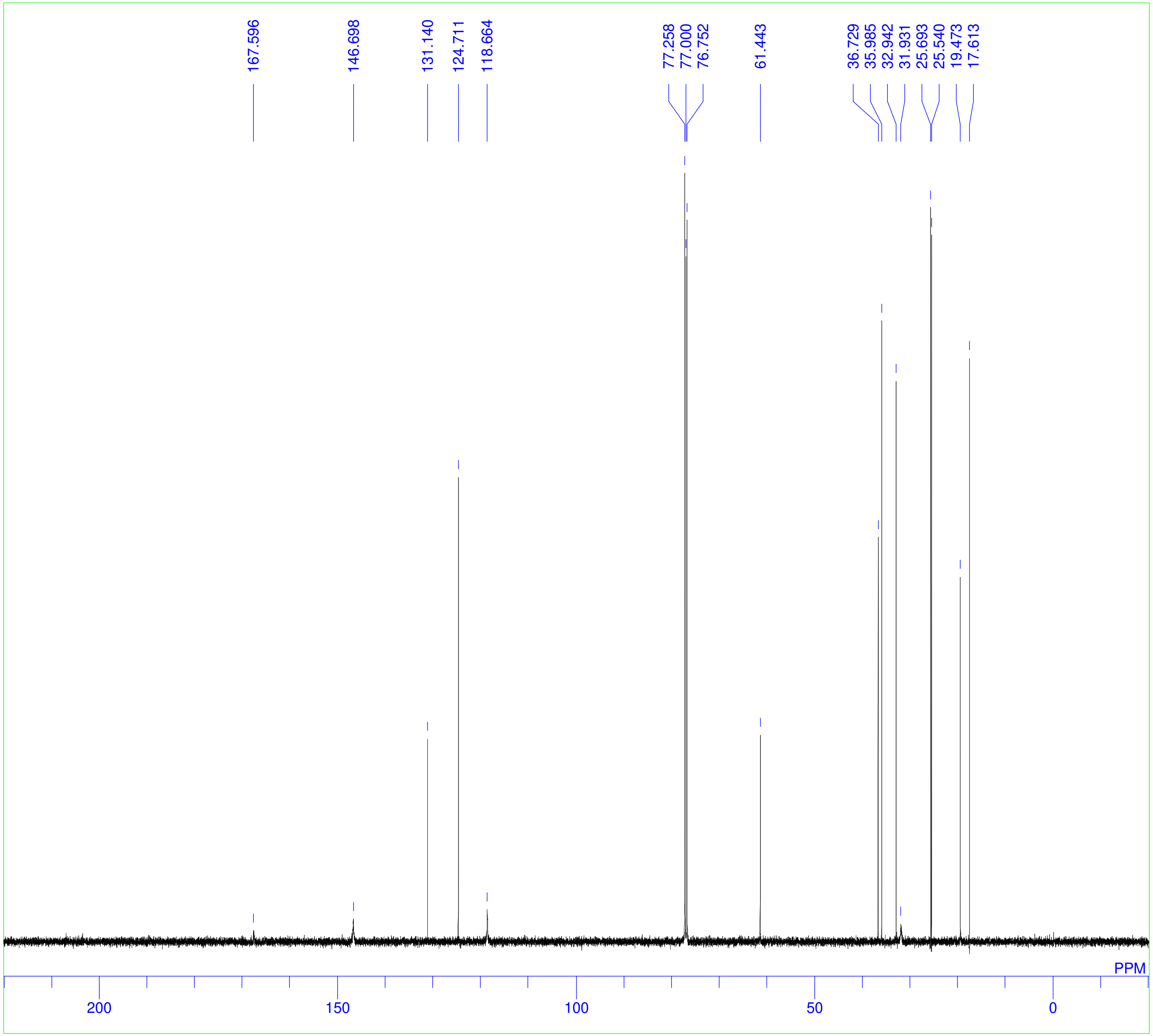

DFILE	(Z)-16j_13C.als
COMNT	
DATIM	2023-01-27 20:12:23
OBNUC	13C
EXMOD	carbon.jxp
OBFRQ	125.77 MHz
OBSET	7.87 KHz
OBFIN	4.21 Hz
POINT	26214
FREQU	31446.54 Hz
SCANS	1024
ACQTM	0.8336 sec
PD	2.0000 sec
PW1	3.87 usec
IRNUC	1H
CTEMP	19.9 c
SLVNT	CDCL3
EXREF	77.00 ppm
BF	0.30 Hz
RGAIN	28

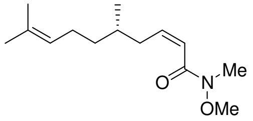

(*Z*)-16j

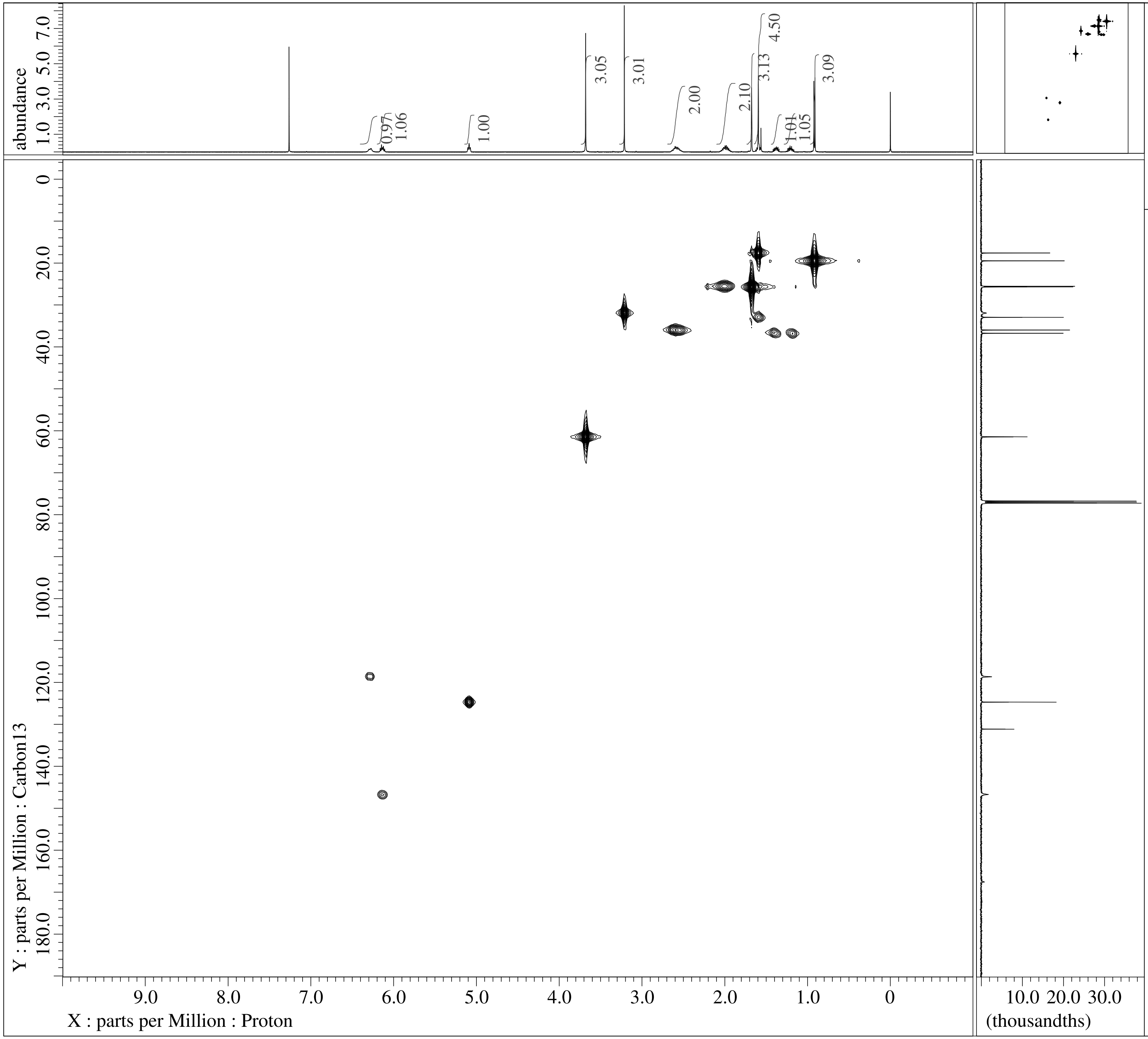

---- PROCESSING PARAMETERS ----
sinbell4(-60, 160)
zerofill(1)
fft(1, TRUE, TRUE)
ppm
[transpose]
sinbell4(-60, 160)
zerofill(2)
fft(1, TRUE, TRUE)
ppm
abs

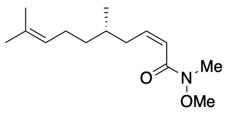

(Z)-16j

Filename	= HT-1259-PTLC 1_HMQC_HMQ
Author	= delta
Experiment	= hmqc.jxp
Sample_Id	= HT-1259-PTLC 1_HMQC
Solvent	= CHLOROFORM-D
Creation_Time	= 29-JAN-2023 17:32:55
Revision_Time	= 10-APR-2024 19:35:41
Current_Time	= 10-APR-2024 19:36:15
Comment	= gradient enhanced HMQC
Data_Format	= 2D REAL REAL
Dim_Size	= 819, 512
Dim_Title	= Proton Carbon13
Dim_Units	= [ppm] [ppm]
Dimensions	= X Y
Site	= JNM-ECA500II
Spectrometer	= DELTA2_NMR
Field_Strength	= 11.7473579[T] (500[MHz]
X_Acq_Duration	= 0.10911744[s]
X_Domain	= 1H
X_Freq	= 500.15991521[MHz]
X_Offset	= 5.0[ppm]
X_Points	= 1024
X_Prescans	= 4
X_Resolution	= 9.16443788[Hz]
X_Sweep	= 9.38438438[kHz]
X_Sweep_Clipped	= 7.50750751[kHz]
Y_Domain	= 13C
Y_Freq	= 125.76529768[MHz]
Y_Offset	= 92.73179603[ppm]
Y_Points	= 256
Y_Prescans	= 0
Y_Resolution	= 95.92951866[Hz]
Y_Sweep	= 24.55795678[kHz]
Tri_Domain	= Proton
Tri_Freq	= 500.15991521[MHz]
Tri_Offset	= 5.0[ppm]
Clipped	= FALSE
Scans	= 8
Total_Scans	= 2048
Relaxation_Delay	= 1.5[s]
Recvr_Gain	= 50
Temp_Get	= 18.8[dC]
X_Acq_Time	= 0.10911744[s]
X_Atn	= 3.2[dB]
X_Gamma	= 42576375
X_Pulse	= 7.68[us]

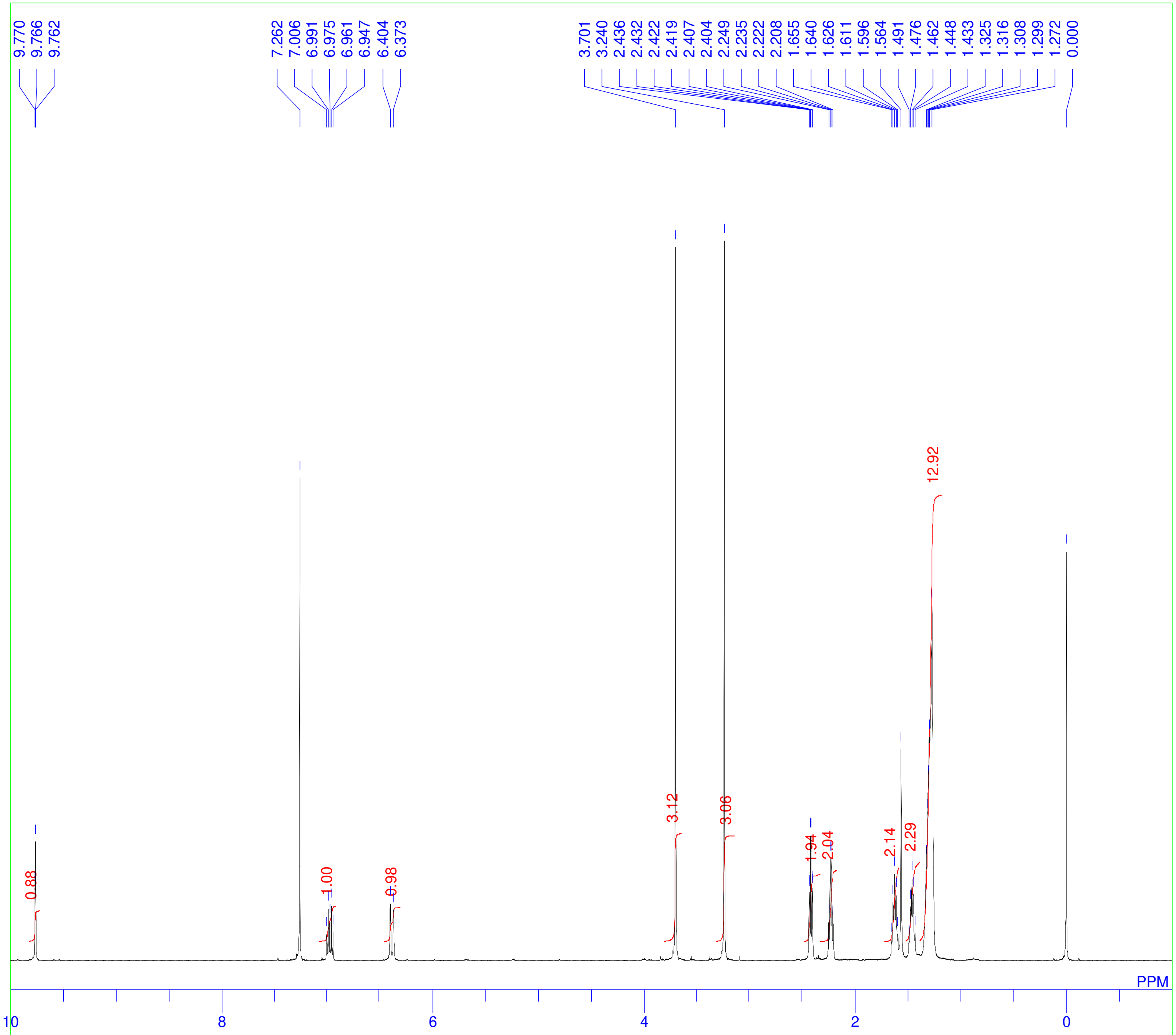

DFILE	(E)-16k_1H.als
COMNT	
DATIM	2023-05-19 18:25:26
OBNUC	1H
EXMOD	proton.jxp
OBFRQ	500.16 MHz
OBSET	2.41 KHz
OBFIN	6.01 Hz
POINT	13107
FREQU	7507.51 Hz
SCANS	8
ACQTM	1.7459 sec
PD	5.0000 sec
PW1	3.84 usec
IRNUC	1H
CTEMP	23.6 c
SLVNT	CDCL3
EXREF	0.00 ppm
BF	1.00 Hz
RGAIN	44

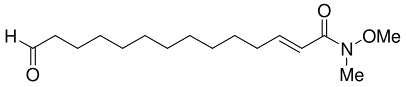

(*E*)-16k

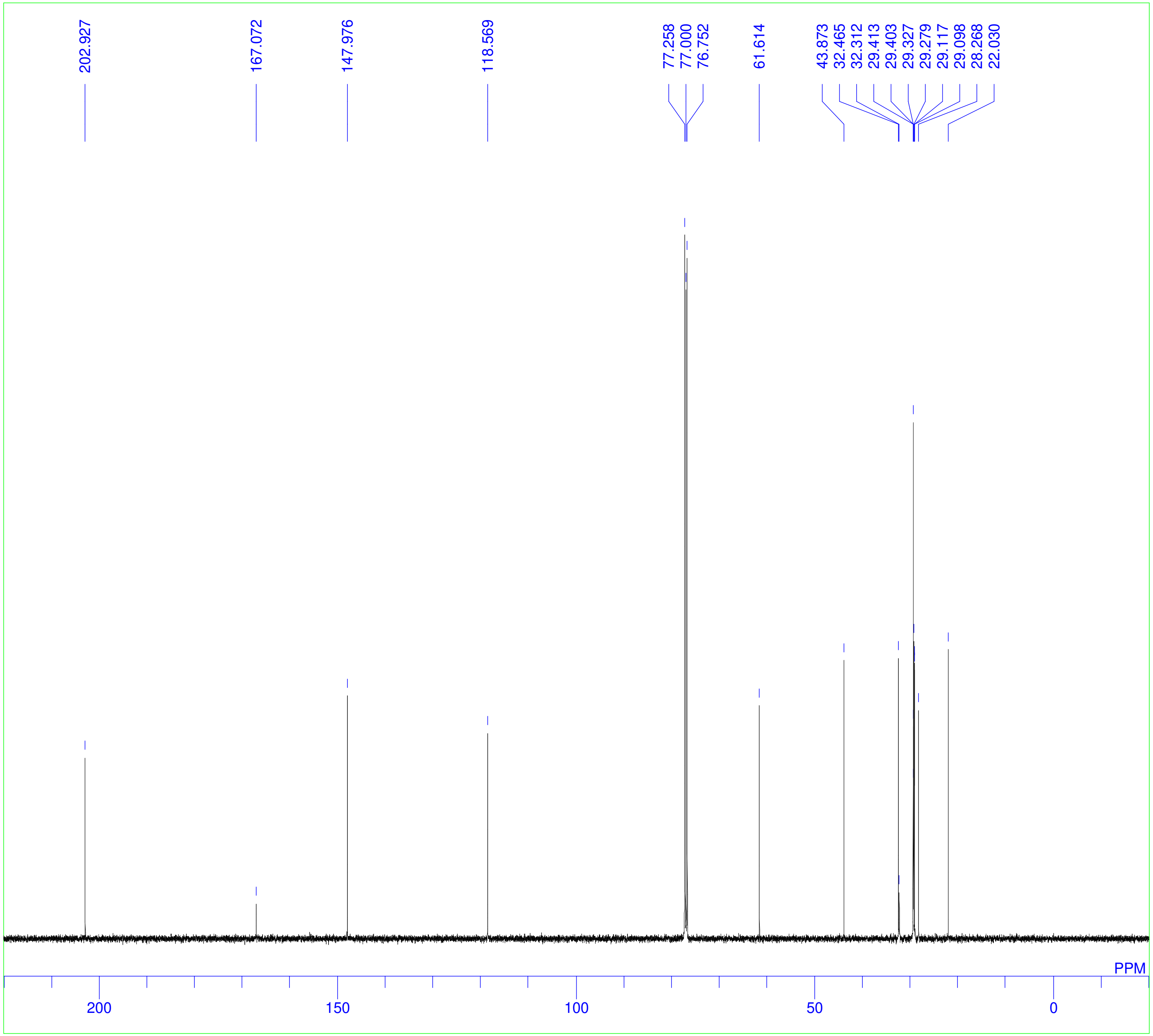

DFILE (E)-16k_13C.als
COMNT
DATIM 2023-05-21 13:02:10
OBNUC 13C
EXMOD carbon.jxp
OBFRQ 125.77 MHz
OBSET 7.87 KHz
OBFIN 4.21 Hz
POINT 26214
FREQU 31446.54 Hz
SCANS 1024
ACQTM 0.8336 sec
PD 2.0000 sec
PW1 3.87 usec
IRNUC 1H
CTEMP 23.9 c
SLVNT CDCL3
EXREF 77.00 ppm
BF 1.00 Hz
RGAIN 30

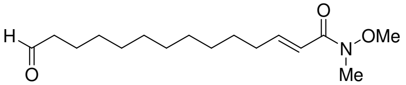

(*E*)-16k

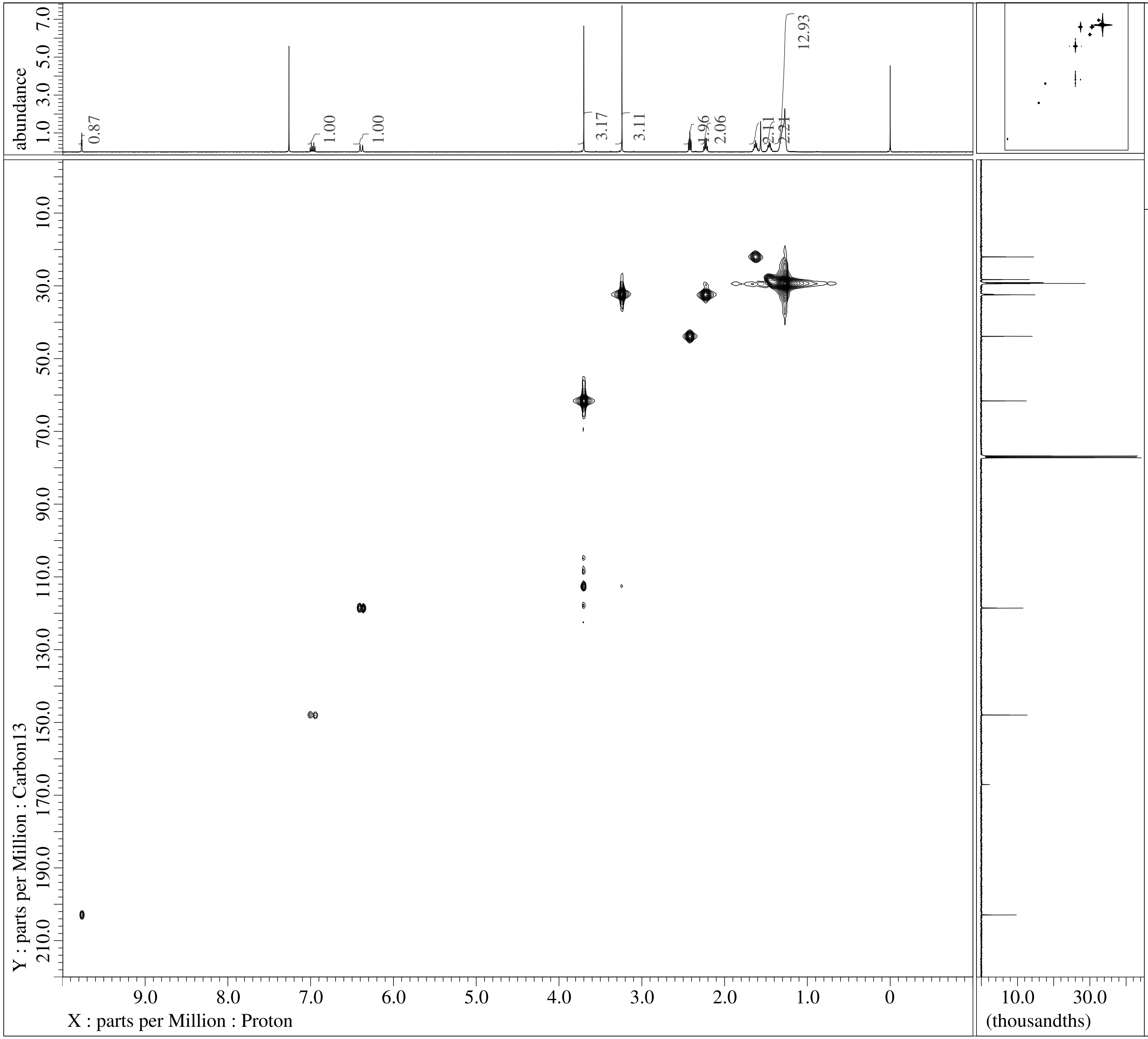


```
---- PROCESSING PARAMETERS ----
sinbell4( -60, 160 )
zerofill( 1 )
fft( 1, TRUE, TRUE )
ppm
[transpose]
sinbell4( -60, 160 )
zerofill( 2 )
fft( 1, TRUE, TRUE )
ppm
abs
```

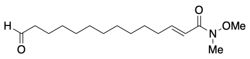

(*E*)-16k

```
Filename            = HT-1441-PTLC 1_13C_HMQC
Author              = delta
Experiment          = hmqc.jxp
Sample_Id           = HT-1441-PTLC 1_13C
Solvent             = CHLOROFORM-D
Creation_Time       = 21-MAY-2023 14:52:39
Revision_Time       = 10-APR-2024 19:39:16
Current_Time        = 10-APR-2024 19:40:07

Comment             = gradient enhanced HMQC
Data_Format         = 2D REAL REAL
Dim_Size            = 819, 512
Dim_Title           = Proton Carbon13
Dim_Units           = [ppm] [ppm]
Dimensions          = X Y
Site                = JNM-ECA500II
Spectrometer        = DELTA2_NMR

Field_Strength      = 11.7473579[T] (500[MHz]
X_Acq_Duration      = 0.10911744[s]
X_Domain            = 1H
X_Freq              = 500.15991521[MHz]
X_Offset            = 5.0[ppm]
X_Points            = 1024
X_Prescans          = 4
X_Resolution        = 9.16443788[Hz]
X_Sweep             = 9.38438438[kHz]
X_Sweep_Clipped     = 7.50750751[kHz]
Y_Domain            = 13C
Y_Freq              = 125.76529768[MHz]
Y_Offset            = 110.01047252[ppm]
Y_Points            = 256
Y_Prescans          = 0
Y_Resolution        = 113.02806713[Hz]
Y_Sweep             = 28.93518519[kHz]
Tri_Domain          = Proton
Tri_Freq            = 500.15991521[MHz]
Tri_Offset          = 5.0[ppm]
Clipped             = FALSE
Scans               = 8
Total_Scans         = 2048

Relaxation_Delay    = 1.5[s]
Recvr_Gain          = 50
Temp_Get            = 23.5[dC]
X_Acq_Time          = 0.10911744[s]
X_Atn               = 3.2[dB]
X_Gamma             = 42576375
X_Pulse             = 7.68[us]
```

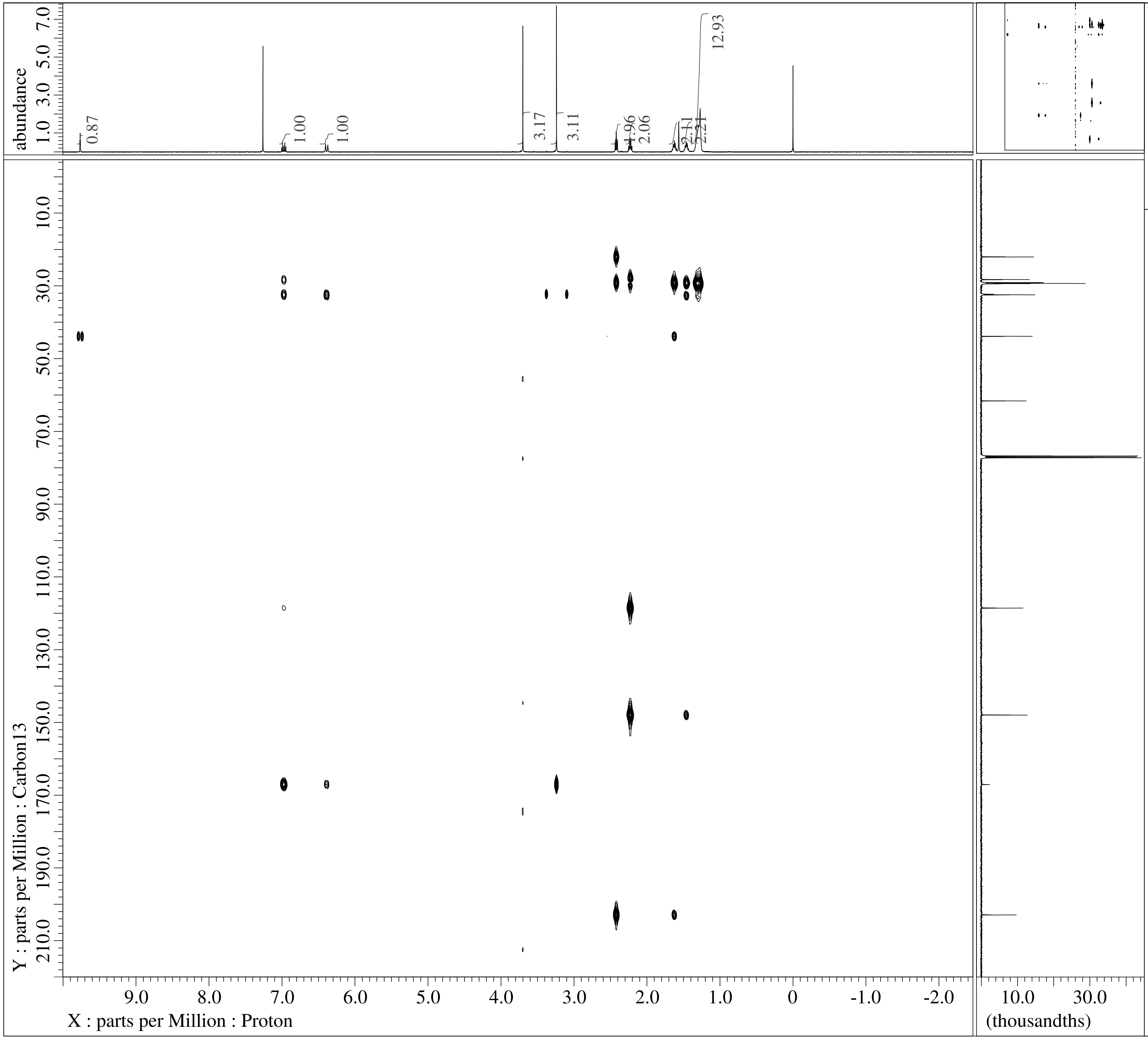


```
---- PROCESSING PARAMETERS ----
gauss( 5.0[Hz], 0.0[s] )
sinbell_auto
zerofill( 1 )
fft( 1, TRUE, TRUE )
ppm
[transpose]
sinbell4( -60, 160 )
trapezoid( 0[%], 5[%], 80[%], 100[%] )
zerofill( 2 )
fft( 1, TRUE, TRUE )
```

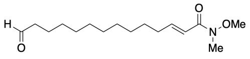

(*E*)-16k

```
Filename            = HT-1441-PTLC 1_13C_HMBC
Author              = delta
Experiment          = hmbc.jxp
Sample_Id           = HT-1441-PTLC 1_13C
Solvent             = CHLOROFORM-D
Creation_Time       = 21-MAY-2023 13:51:02
Revision_Time       = 10-APR-2024 19:45:11
Current_Time        = 10-APR-2024 19:46:01

Comment             = gradient enhanced HMBC
Data_Format         = 2D REAL REAL
Dim_Size            = 1638, 512
Dim_Title           = Proton Carbon13
Dim_Units           = [ppm] [ppm]
Dimensions          = X Y
Site                = JNM-ECA500II
Spectrometer        = DELTA2_NMR

Field_Strength      = 11.7473579[T] (500[MHz]
X_Acq_Duration      = 0.21823488[s]
X_Domain            = 1H
X_Freq              = 500.15991521[MHz]
X_Offset            = 5.0[ppm]
X_Points            = 2048
X_Prescans          = 4
X_Resolution        = 4.58221894[Hz]
X_Sweep             = 9.38438438[kHz]
X_Sweep_Clipped     = 7.50750751[kHz]
Y_Domain            = 13C
Y_Freq              = 125.76529768[MHz]
Y_Offset            = 110.01047252[ppm]
Y_Points            = 256
Y_Prescans          = 0
Y_Resolution        = 113.02806713[Hz]
Y_Sweep             = 28.93518519[kHz]
Tri_Domain          = Proton
Tri_Freq            = 500.15991521[MHz]
Tri_Offset          = 5.0[ppm]
Clipped             = FALSE
Scans               = 8
Total_Scans         = 2048

Relaxation_Delay    = 1.5[s]
Recvr_Gain          = 50
Temp_Get            = 23.6[dC]
X_Acq_Time          = 0.21823488[s]
X_Atn               = 3.2[dB]
X_Gamma             = 42576375
X_Pulse             = 7.68[us]
```

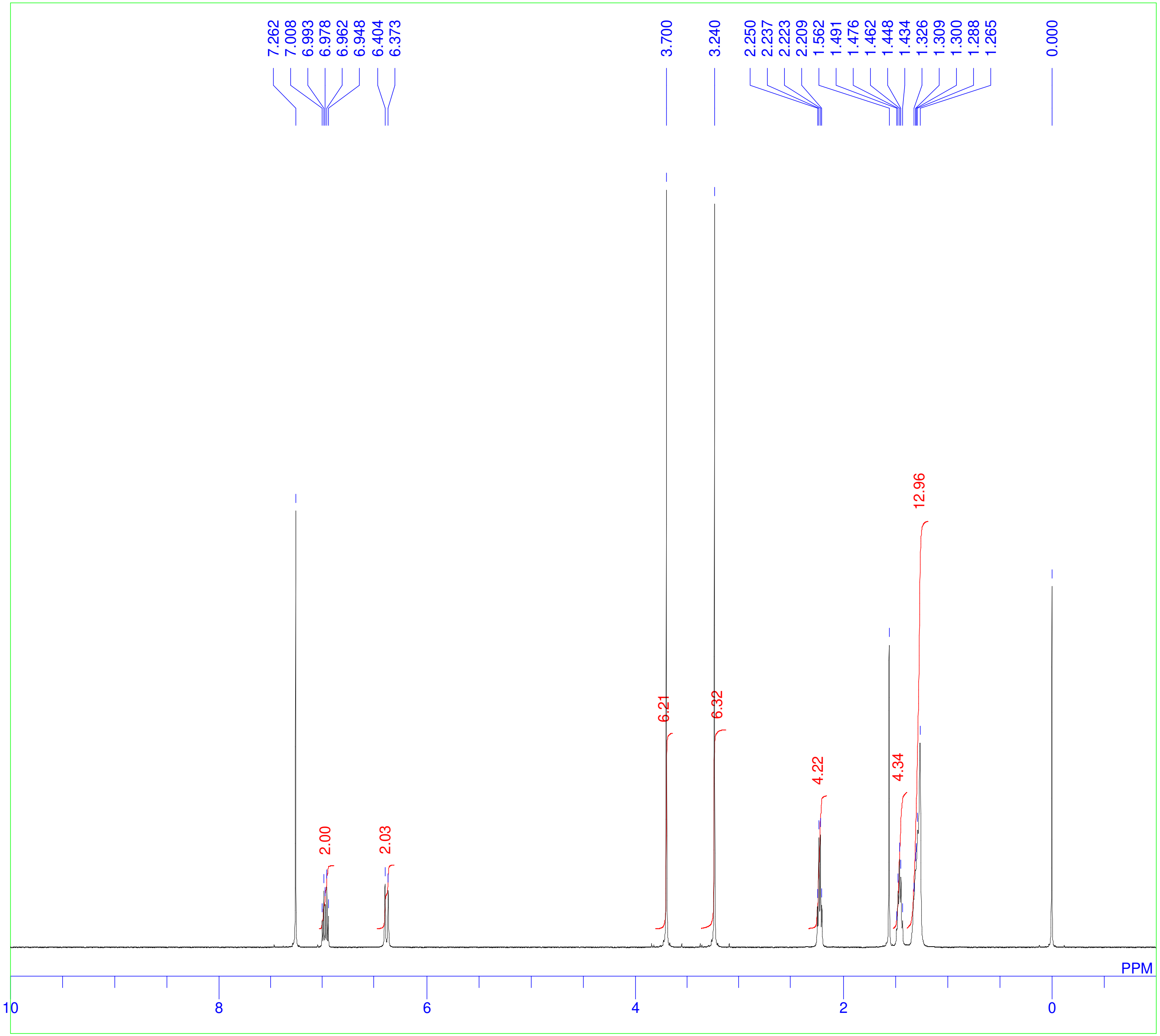

DFILE	(E,E)-16k_1H.als
COMNT	
DATIM	2023-05-19 18:38:57
OBNUC	1H
EXMOD	proton.jxp
OBFRQ	500.16 MHz
OBSET	2.41 KHz
OBFIN	6.01 Hz
POINT	13107
FREQU	7507.51 Hz
SCANS	8
ACQTM	1.7459 sec
PD	5.0000 sec
PW1	3.84 usec
IRNUC	1H
CTEMP	23.8 c
SLVNT	CDCL3
EXREF	0.00 ppm
BF	1.00 Hz
RGAIN	44

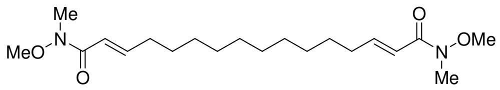

(*E*,*E*)-16k

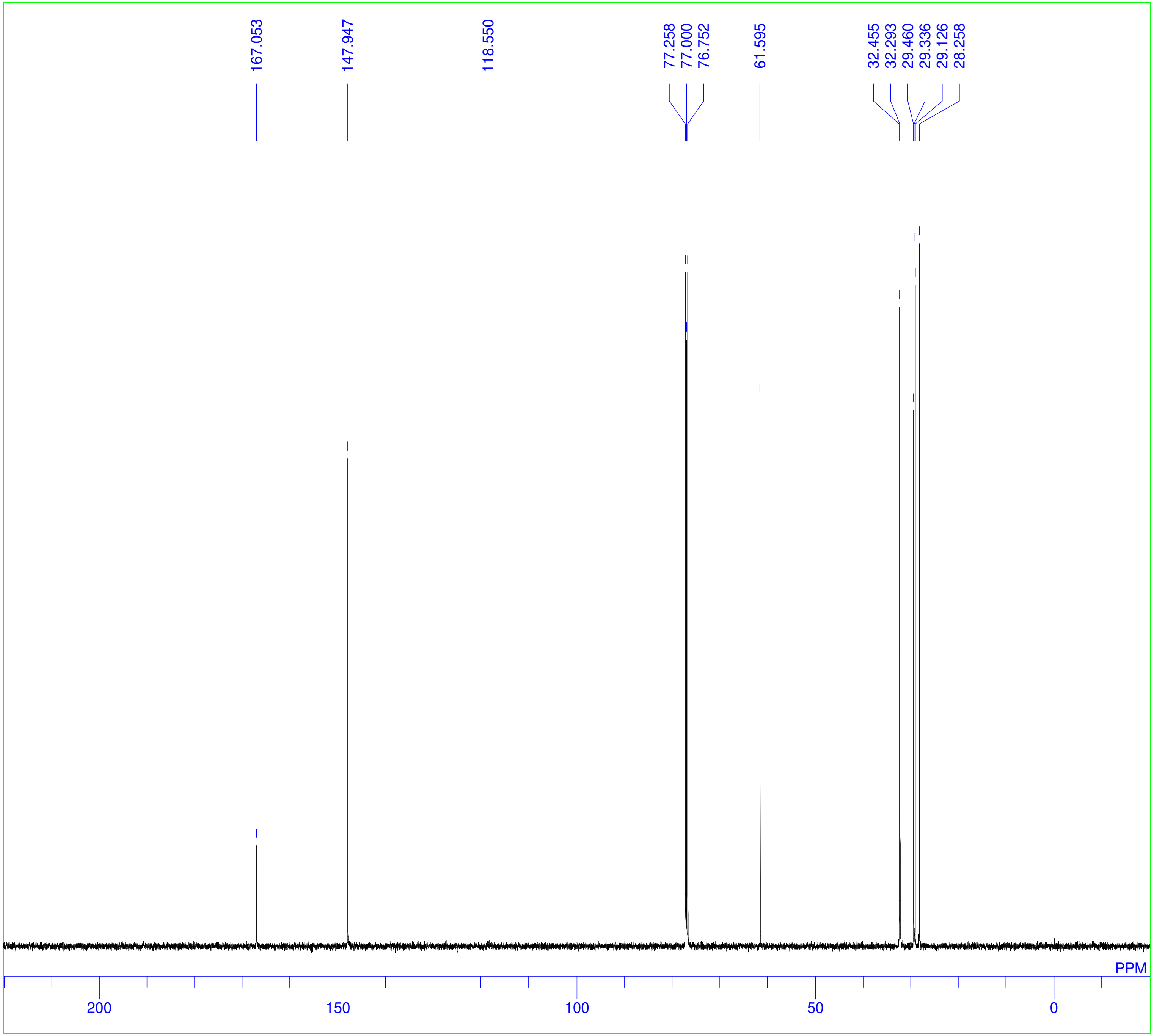

DFILE	(E,E)-16k_13C.als
COMNT	
DATIM	2023-05-21 16:23:08
OBNUC	13C
EXMOD	carbon.jxp
OBFRQ	125.77 MHz
OBSET	7.87 KHz
OBFIN	4.21 Hz
POINT	26214
FREQU	31446.54 Hz
SCANS	1024
ACQTM	0.8336 sec
PD	2.0000 sec
PW1	3.87 usec
IRNUC	1H
CTEMP	23.9 c
SLVNT	CDCL3
EXREF	77.00 ppm
BF	1.00 Hz
RGAIN	34

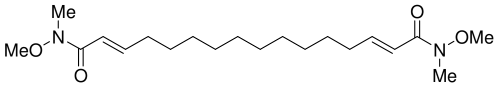

(*E*,*E*)-16k

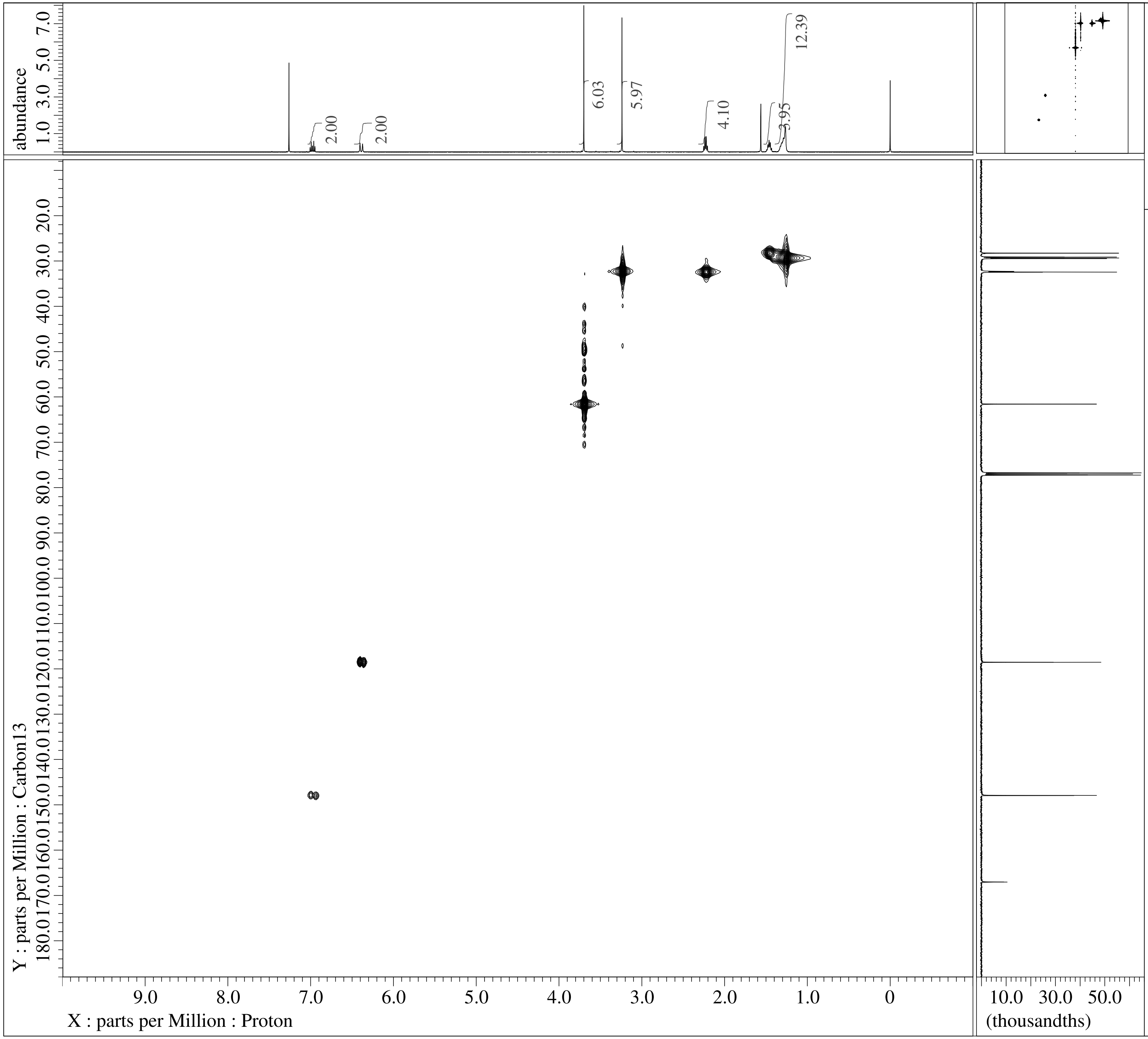


```
---- PROCESSING PARAMETERS ----
sinbell4( -60, 160 )
zerofill( 1 )
fft( 1, TRUE, TRUE )
ppm
[transpose]
sinbell4( -60, 160 )
zerofill( 2 )
fft( 1, TRUE, TRUE )
ppm
abs
```

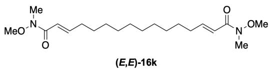


```
Filename            = HT-1441-PTLC 3_13C_HMQC
Author              = delta
Experiment          = hmqc.jxp
Sample_Id           = HT-1441-PTLC 3_13C
Solvent             = CHLOROFORM-D
Creation_Time       = 21-MAY-2023 17:11:59
Revision_Time       = 10-APR-2024 19:48:11
Current_Time        = 10-APR-2024 19:49:04

Comment             = gradient enhanced HMQC
Data_Format         = 2D REAL REAL
Dim_Size            = 819, 512
Dim_Title           = Proton Carbon13
Dim_Units           = [ppm] [ppm]
Dimensions          = X Y
Site                = JNM-ECA500II
Spectrometer        = DELTA2_NMR

Field_Strength      = 11.7473579[T] (500[MHz]
X_Acq_Duration      = 0.10911744[s]
X_Domain            = 1H
X_Freq              = 500.15991521[MHz]
X_Offset            = 5.0[ppm]
X_Points            = 1024
X_Prescans          = 4
X_Resolution        = 9.16443788[Hz]
X_Sweep             = 9.38438438[kHz]
X_Sweep_Clipped     = 7.50750751[kHz]
Y_Domain            = 13C
Y_Freq              = 125.76529768[MHz]
Y_Offset            = 97.77280102[ppm]
Y_Points            = 256
Y_Prescans          = 0
Y_Resolution        = 88.77840909[Hz]
Y_Sweep             = 22.72727273[kHz]
Tri_Domain          = Proton
Tri_Freq            = 500.15991521[MHz]
Tri_Offset          = 5.0[ppm]
Clipped             = FALSE
Scans               = 8
Total_Scans         = 2048

Relaxation_Delay    = 1.5[s]
Recvr_Gain          = 50
Temp_Get            = 23.5[dC]
X_Acq_Time          = 0.10911744[s]
X_Atn               = 3.2[dB]
X_Gamma             = 42576375
X_Pulse             = 7.68[us]
```

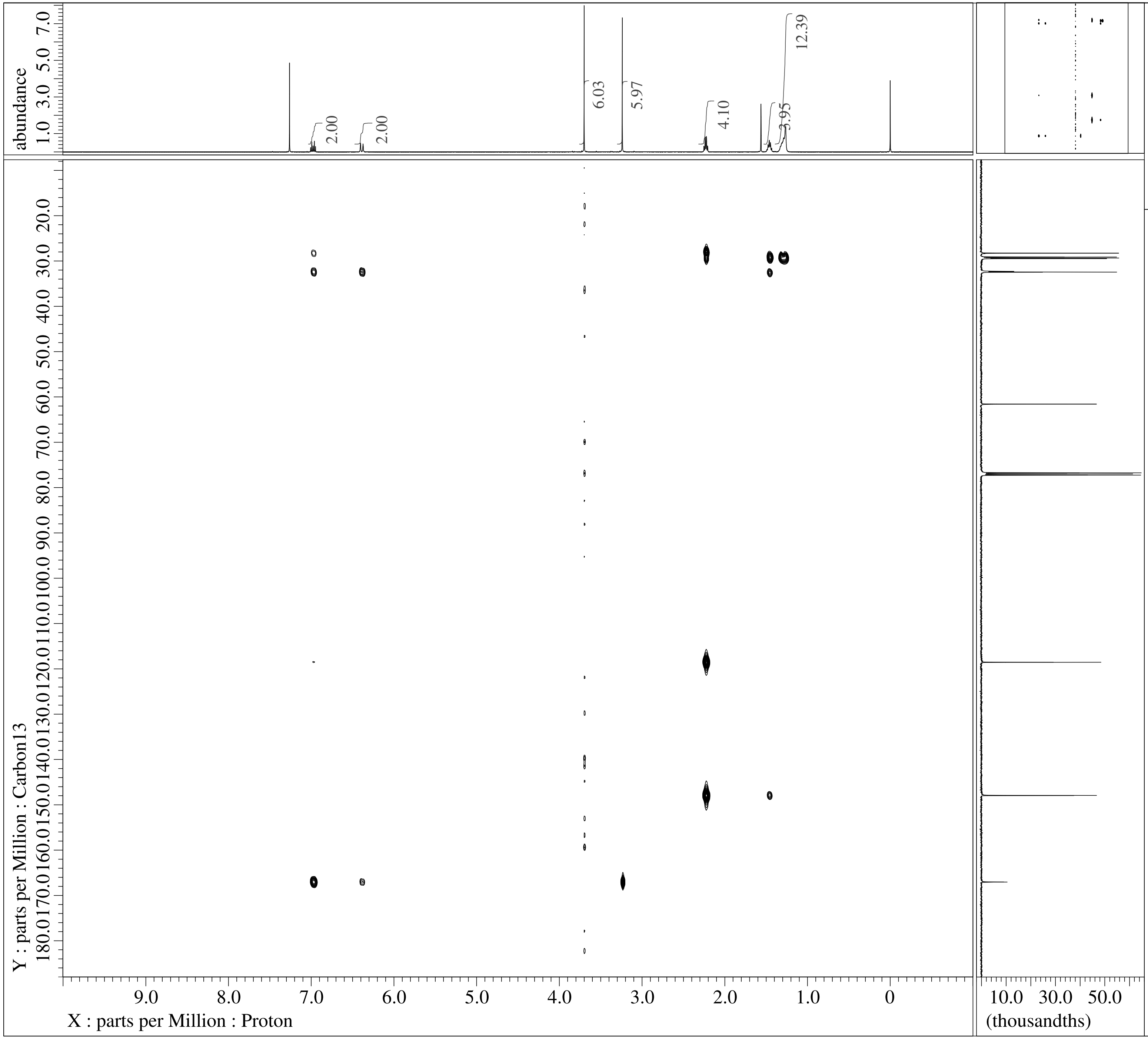

---- PROCESSING PARAMETERS ----
gauss(5.0[Hz], 0.0[s])
sinbell_auto
zerofill(1)
fft(1, TRUE, TRUE)
ppm
[transpose]
sinbell4(-60, 160)
trapezoid(0[%], 5[%], 80[%], 100[%])
zerofill(2)
fft(1, TRUE, TRUE)

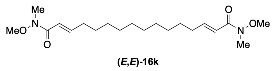

Filename	= HT-1441-PTLC 3_13C_HMBC
Author	= delta
Experiment	= hmbc.jxp
Sample_Id	= HT-1441-PTLC 3_13C
Solvent	= CHLOROFORM-D
Creation_Time	= 21-MAY-2023 18:07:58
Revision_Time	= 10-APR-2024 19:50:26
Current_Time	= 10-APR-2024 19:51:11
Comment	= gradient enhanced HMBC
Data_Format	= 2D REAL REAL
Dim_Size	= 1638, 512
Dim_Title	= Proton Carbon13
Dim_Units	= [ppm] [ppm]
Dimensions	= X Y
Site	= JNM-ECA500II
Spectrometer	= DELTA2_NMR
Field_Strength	= 11.7473579[T] (500[MHz]
X_Acq_Duration	= 0.21823488[s]
X_Domain	= 1H
X_Freq	= 500.15991521[MHz]
X_Offset	= 5.0[ppm]
X_Points	= 2048
X_Prescans	= 4
X_Resolution	= 4.58221894[Hz]
X_Sweep	= 9.38438438[kHz]
X_Sweep_Clipped	= 7.50750751[kHz]
Y_Domain	= 13C
Y_Freq	= 125.76529768[MHz]
Y_Offset	= 97.77280102[ppm]
Y_Points	= 256
Y_Prescans	= 0
Y_Resolution	= 88.77840909[Hz]
Y_Sweep	= 22.72727273[kHz]
Tri_Domain	= Proton
Tri_Freq	= 500.15991521[MHz]
Tri_Offset	= 5.0[ppm]
Clipped	= FALSE
Scans	= 8
Total_Scans	= 2048
Relaxation_Delay	= 1.5[s]
Recvr_Gain	= 50
Temp_Get	= 23.7[dC]
X_Acq_Time	= 0.21823488[s]
X_Atn	= 3.2[dB]
X_Gamma	= 42576375
X_Pulse	= 7.68[us]

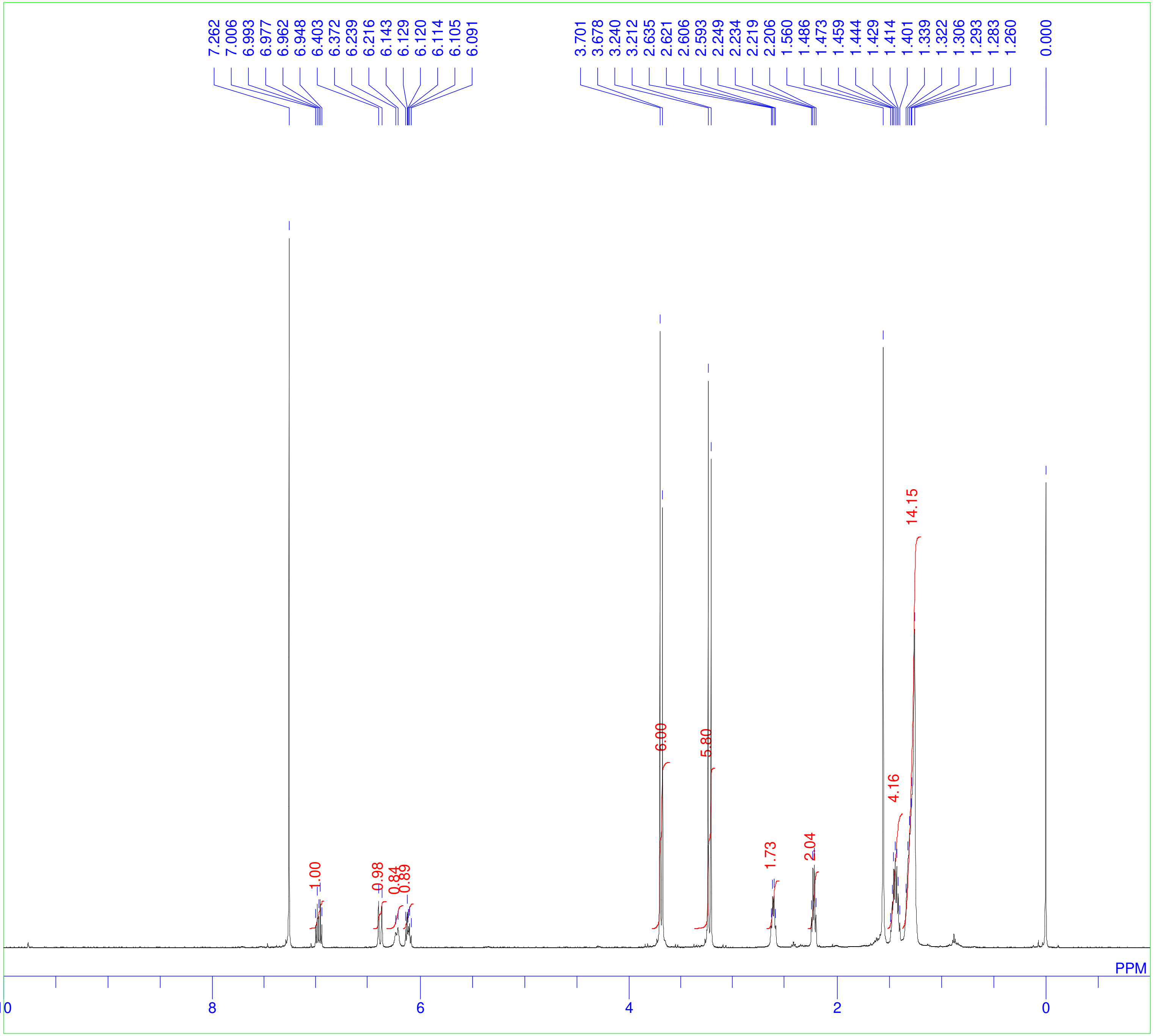

DFILE (E,Z)-16k_1H.als
COMNT
DATIM 2023-05-19 18:32:32
OBNUC 1H
EXMOD proton.jxp
OBFRQ 500.16 MHz
OBSET 2.41 KHz
OBFIN 6.01 Hz
POINT 13107
FREQU 7507.51 Hz
SCANS 8
ACQTM 1.7459 sec
PD 5.0000 sec
PW1 3.84 usec
IRNUC 1H
CTEMP 23.8 c
SLVNT CDCL3
EXREF 0.00 ppm
BF 1.00 Hz
RGAIN 44

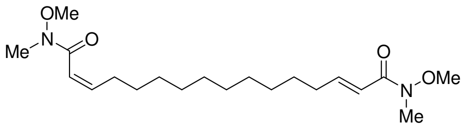

(*E*,*Z*)-16k

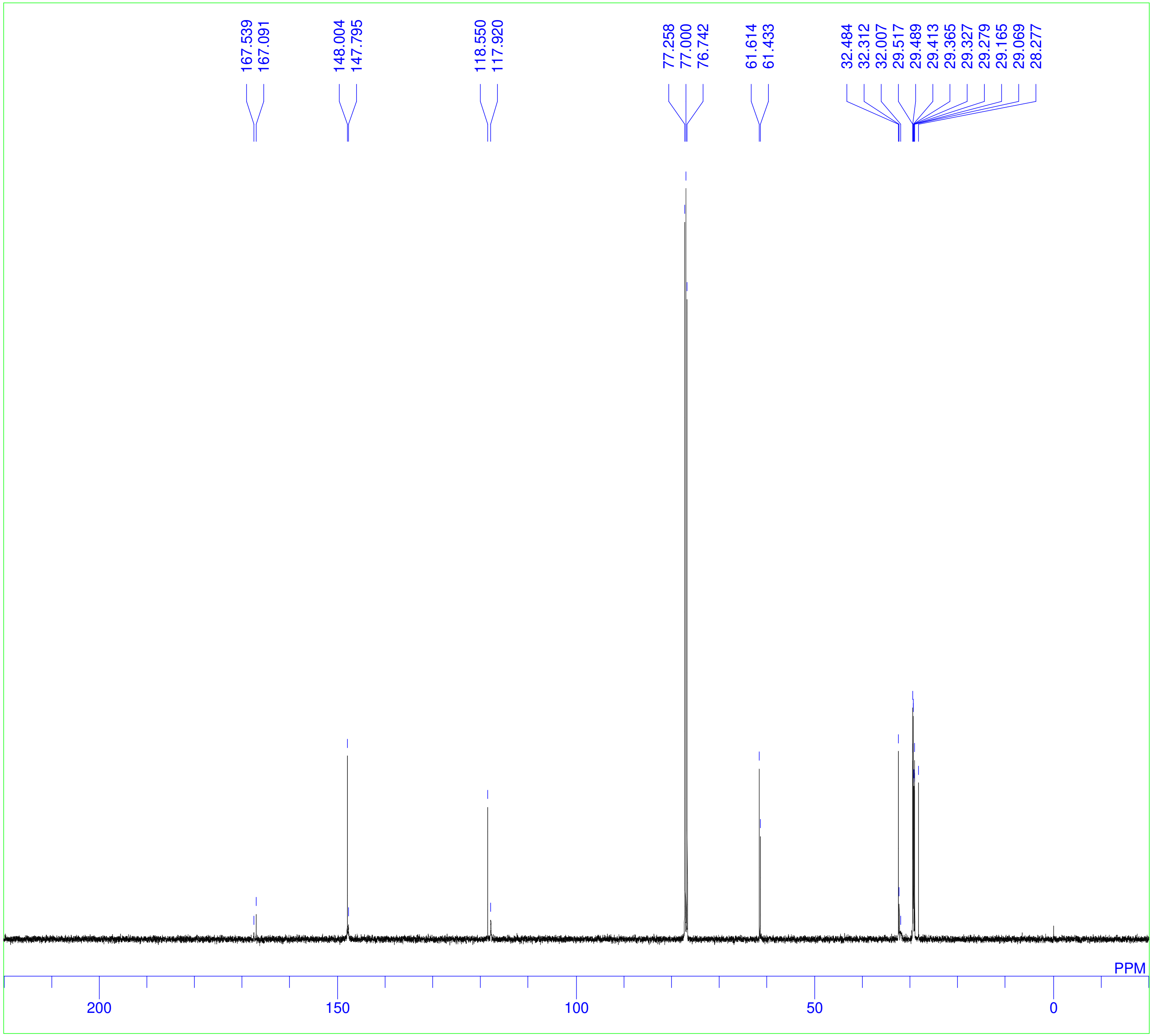

DFILE	(E,Z)-16k_13C.als
COMNT	
DATIM	2023-11-10 15:11:29
OBNUC	13C
EXMOD	carbon.jxp
OBFRQ	125.77 MHz
OBSET	7.87 KHz
OBFIN	4.21 Hz
POINT	26214
FREQU	31446.54 Hz
SCANS	1024
ACQTM	0.8336 sec
PD	2.0000 sec
PW1	4.30 usec
IRNUC	1H
CTEMP	23.7 c
SLVNT	CDCL3
EXREF	77.00 ppm
BF	1.00 Hz
RGAIN	34

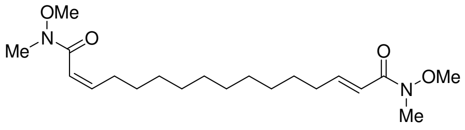

(*E*,*Z*)-16k

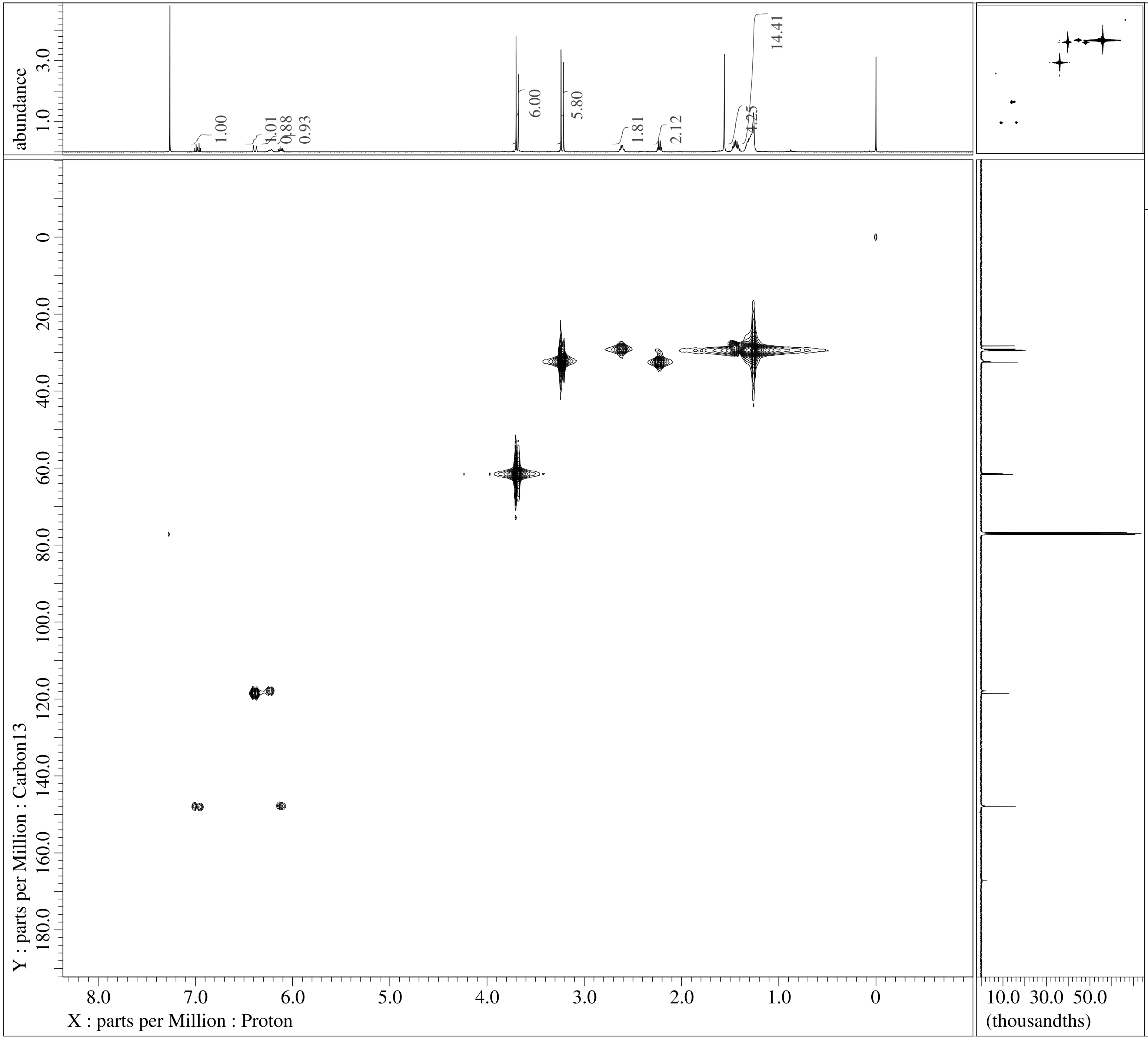


```
---- PROCESSING PARAMETERS ----
sinbell4( -60, 160 )
zerofill( 1 )
fft( 1, TRUE, TRUE )
ppm
[transpose]
sinbell4( -60, 160 )
zerofill( 2 )
fft( 1, TRUE, TRUE )
ppm
abs
```

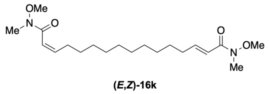


```
Filename            = HT-1610-PTLC 1_HMQC-1-6
Author              = delta
Experiment          = hmqc.jxp
Sample_Id           = HT-1610-PTLC 1
Solvent             = CHLOROFORM-D
Creation_Time       = 10-NOV-2023 16:00:22
Revision_Time       = 10-APR-2024 19:53:16
Current_Time        = 10-APR-2024 19:54:03

Comment             = gradient enhanced HMQC
Data_Format         = 2D REAL REAL
Dim_Size            = 819, 512
Dim_Title           = Proton Carbon13
Dim_Units           = [ppm] [ppm]
Dimensions          = X Y
Site                = JNM-ECA500II
Spectrometer        = DELTA2_NMR

Field_Strength      = 11.7473579[T] (500[MHz]
X_Acq_Duration      = 0.17301504[s]
X_Domain            = 1H
X_Freq              = 500.15991521[MHz]
X_Offset            = 3.60859601[ppm]
X_Points            = 1024
X_Prescans          = 4
X_Resolution        = 5.77984434[Hz]
X_Sweep             = 5.91856061[kHz]
X_Sweep_Clipped     = 4.73484848[kHz]
Y_Domain            = 13C
Y_Freq              = 125.76529768[MHz]
Y_Offset            = 83.62746443[ppm]
Y_Points            = 256
Y_Prescans          = 0
Y_Resolution        = 106.84491247[Hz]
Y_Sweep             = 27.35229759[kHz]
Tri_Domain          = Proton
Tri_Freq            = 500.15991521[MHz]
Tri_Offset          = 5.0[ppm]
Clipped             = FALSE
Scans               = 8
Total_Scans         = 2048

Relaxation_Delay    = 1.5[s]
Recvr_Gain          = 50
Temp_Get            = 23.9[dC]
X_Acq_Time          = 0.17301504[s]
X_Atn               = 3.2[dB]
X_Gamma             = 42576375
X_Pulse             = 7.59[us]
```

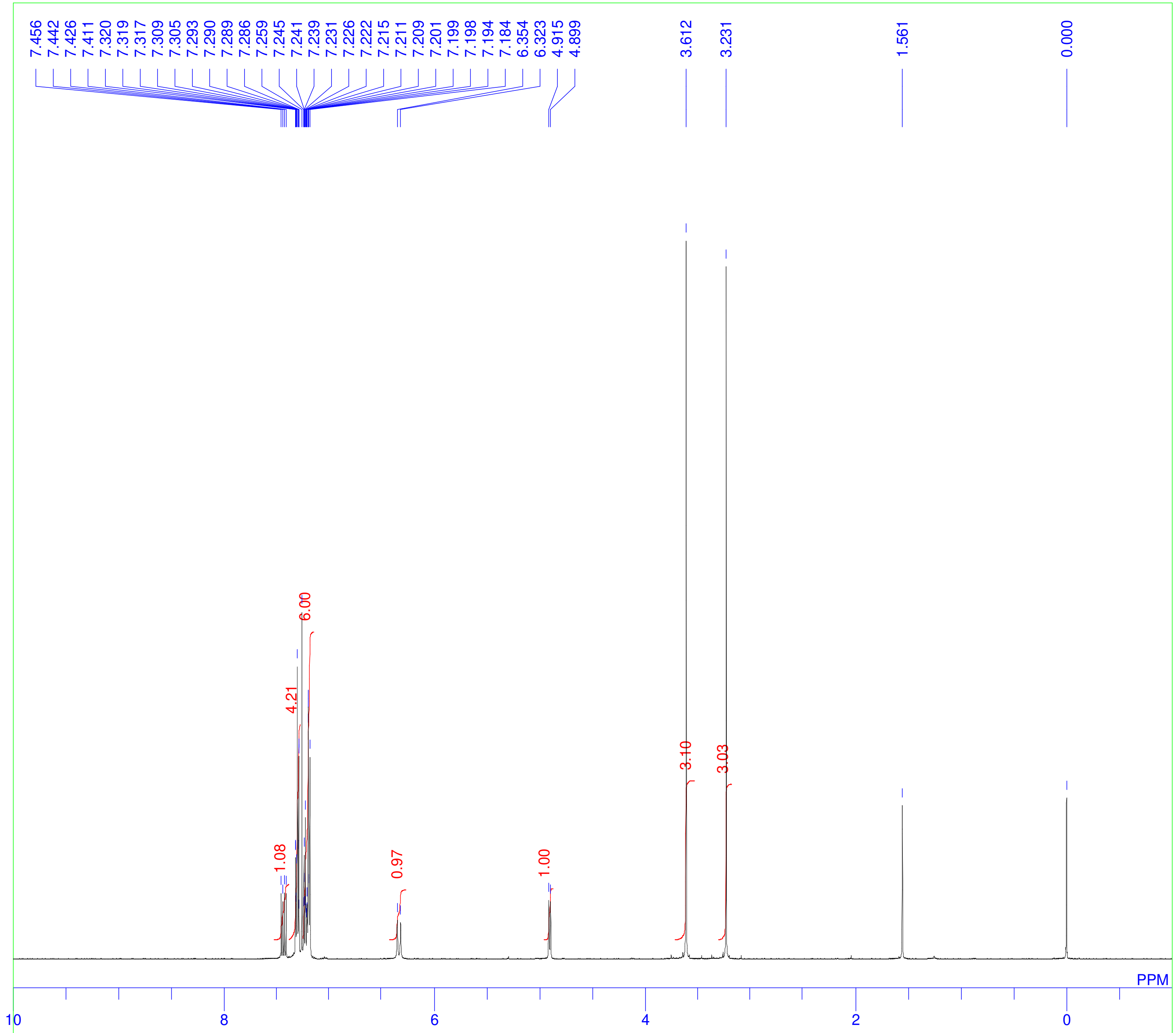

DFILE	(E)-16l_1H.als
COMNT	
DATIM	2023-09-13 20:47:56
OBNUC	1H
EXMOD	proton.jxp
OBFRQ	500.16 MHz
OBSET	2.41 KHz
OBFIN	6.01 Hz
POINT	13107
FREQU	7507.51 Hz
SCANS	8
ACQTM	1.7459 sec
PD	5.0000 sec
PW1	3.84 usec
IRNUC	1H
CTEMP	24.2 c
SLVNT	CDCL3
EXREF	0.00 ppm
BF	0.30 Hz
RGAIN	46

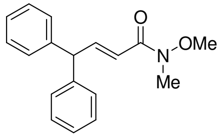

(*E*)-16l

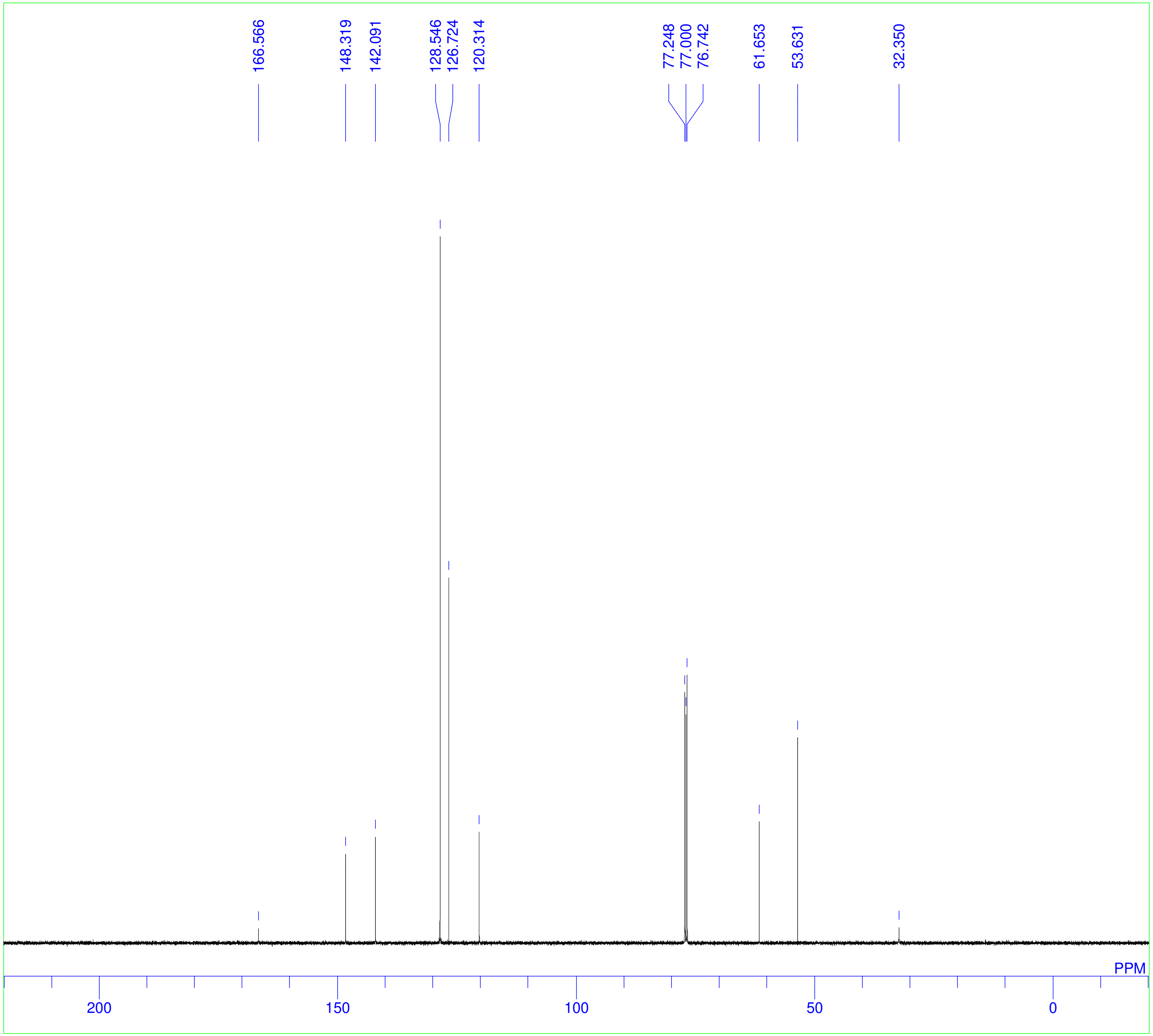

DFILE (E)-16l_13C.als
COMNT
DATIM 2023-09-13 21:28:53
OBNUC 13C
EXMOD carbon.jxp
OBFRQ 125.77 MHz
OBSET 7.87 KHz
OBFIN 4.21 Hz
POINT 26214
FREQU 31446.54 Hz
SCANS 1024
ACQTM 0.8336 sec
PD 2.0000 sec
PW1 3.87 usec
IRNUC 1H
CTEMP 24.2 c
SLVNT CDCL3
EXREF 77.00 ppm
BF 0.30 Hz
RGAIN 30

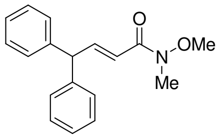

(*E*)-16l

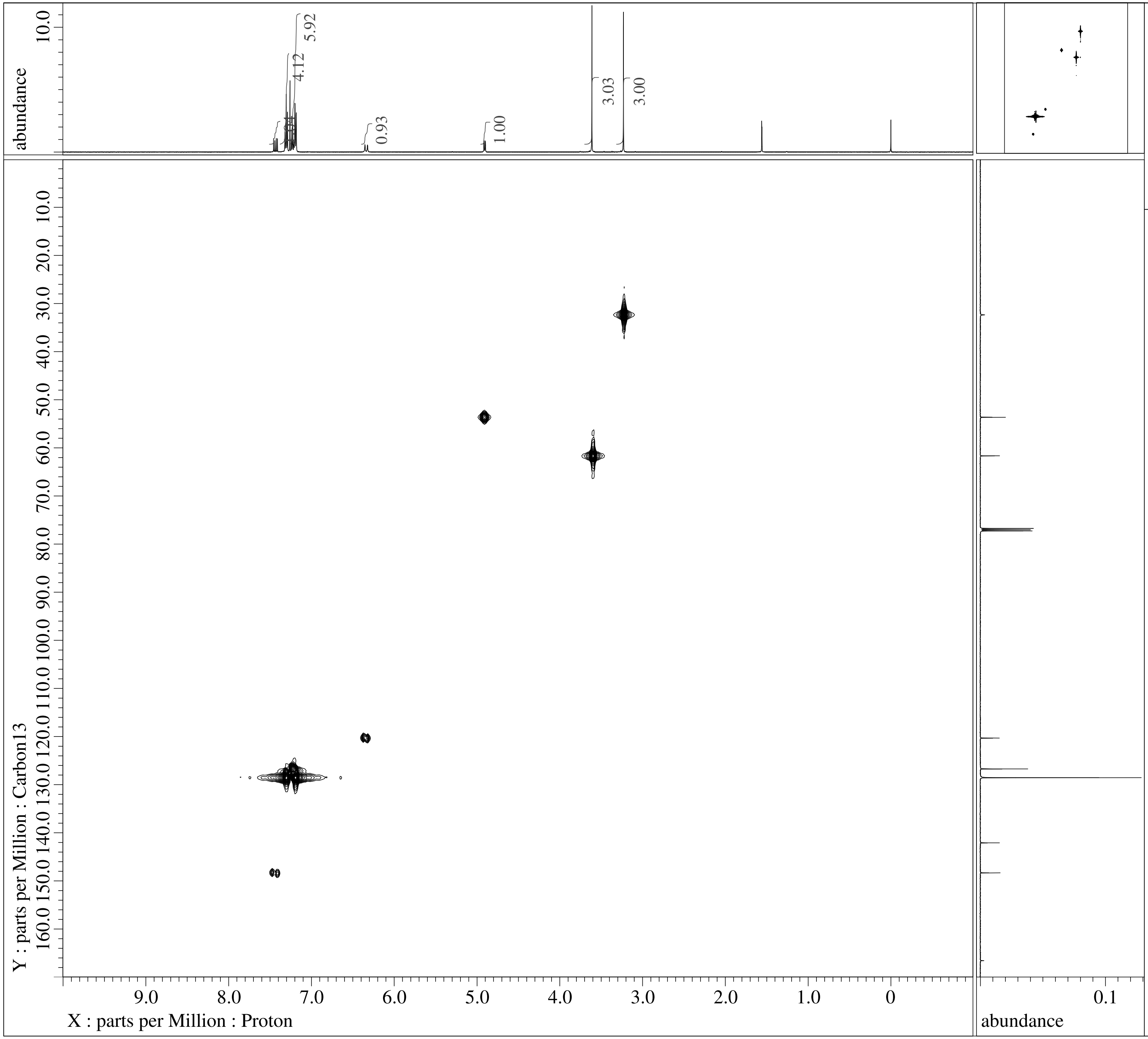


```
---- PROCESSING PARAMETERS ----
sinbell4( -60, 160 )
zerofill( 1 )
fft( 1, TRUE, TRUE )
ppm
[transpose]
sinbell4( -60, 160 )
zerofill( 2 )
fft( 1, TRUE, TRUE )
ppm
abs
```

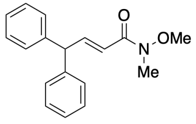

(*E*)-16l

```
Filename           = HT-1543,1544-TM_HMQC_HM
Author             = delta
Experiment         = hmqc.jxp
Sample_Id          = HT-1543,1544-TM_HMQC
Solvent            = CHLOROFORM-D
Creation_Time      = 16-JAN-2024 10:19:34
Revision_Time      = 10-APR-2024 19:57:55
Current_Time       = 10-APR-2024 19:58:45

Comment            = gradient enhanced HMQC
Data_Format        = 2D REAL REAL
Dim_Size           = 819, 512
Dim_Title          = Proton Carbon13
Dim_Units          = [ppm] [ppm]
Dimensions         = X Y
Site               = JNM-ECA500II
Spectrometer       = DELTA2_NMR

Field_Strength     = 11.7473579[T] (500[MHz]
X_Acq_Duration     = 0.10911744[s]
X_Domain           = 1H
X_Freq             = 500.15991521[MHz]
X_Offset           = 5.0[ppm]
X_Points           = 1024
X_Prescans         = 4
X_Resolution       = 9.16443788[Hz]
X_Sweep            = 9.38438438[kHz]
X_Sweep_Clipped    = 7.50750751[kHz]
Y_Domain           = 13C
Y_Freq             = 125.76529768[MHz]
Y_Offset           = 85[ppm]
Y_Points           = 256
Y_Prescans         = 0
Y_Resolution       = 83.60980308[Hz]
Y_Sweep            = 21.40410959[kHz]
Tri_Domain         = Proton
Tri_Freq           = 500.15991521[MHz]
Tri_Offset         = 5.0[ppm]
Clipped            = FALSE
Scans              = 4
Total_Scans        = 1024

Relaxation_Delay   = 1.5[s]
Recvr_Gain         = 50
Temp_Get           = 20[dC]
X_Acq_Time         = 0.10911744[s]
X_Atn              = 3.2[dB]
X_Gamma            = 42576375
X_Pulse            = 7.59[us]
```

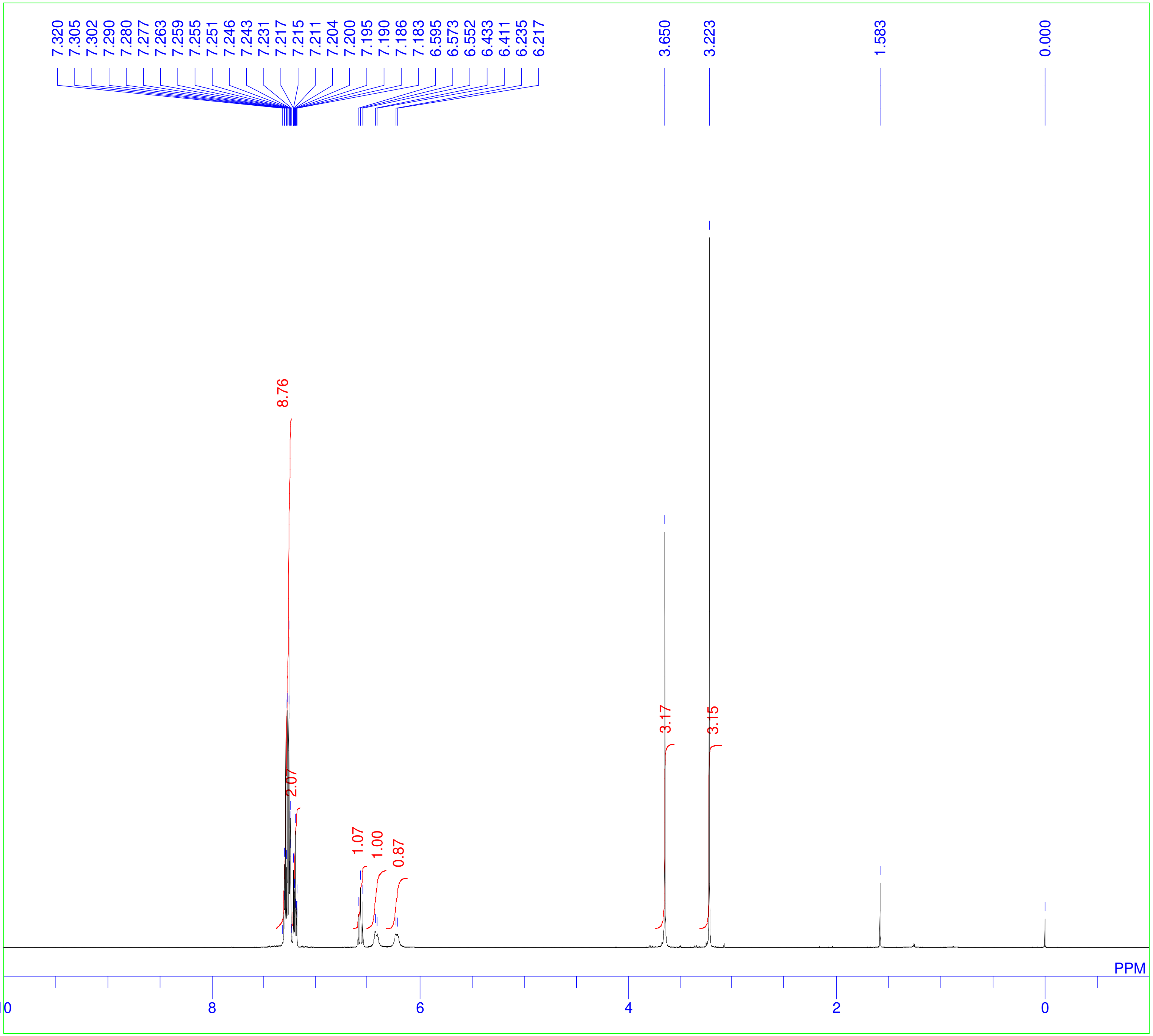

DFILE (Z)-16l_1H.als
COMNT
DATIM 2023-12-01 20:21:17
OBNUC 1H
EXMOD proton.jxp
OBFRQ 500.16 MHz
OBSET 2.41 KHz
OBFIN 6.01 Hz
POINT 13107
FREQU 7507.51 Hz
SCANS 8
ACQTM 1.7459 sec
PD 5.0000 sec
PW1 3.80 usec
IRNUC 1H
CTEMP 23.8 c
SLVNT CDCL3
EXREF 0.00 ppm
BF 0.30 Hz
RGAIN 34

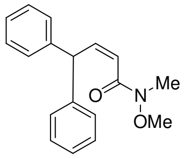

(*Z*)-16l

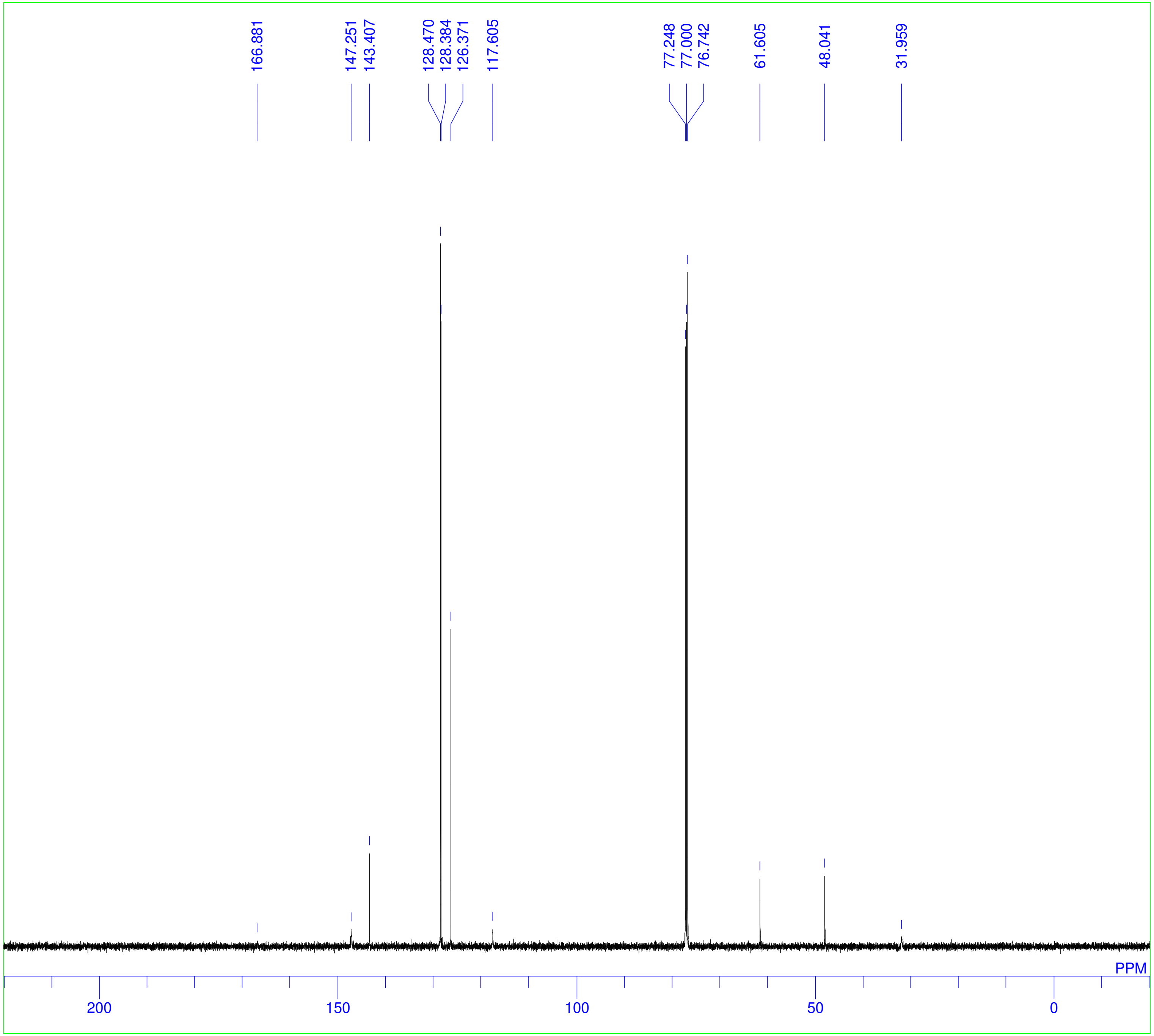

DFILE (Z)-16l_13C.als
COMNT
DATIM 2023-12-01 20:22:54
OBNUC 13C
EXMOD carbon.jxp
OBFRQ 125.77 MHz
OBSET 7.87 KHz
OBFIN 4.21 Hz
POINT 26214
FREQU 31446.54 Hz
SCANS 1024
ACQTM 0.8336 sec
PD 2.0000 sec
PW1 4.30 usec
IRNUC 1H
CTEMP 23.7 c
SLVNT CDCL3
EXREF 77.00 ppm
BF 0.30 Hz
RGAIN 30

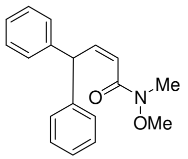

(*Z*)-16l

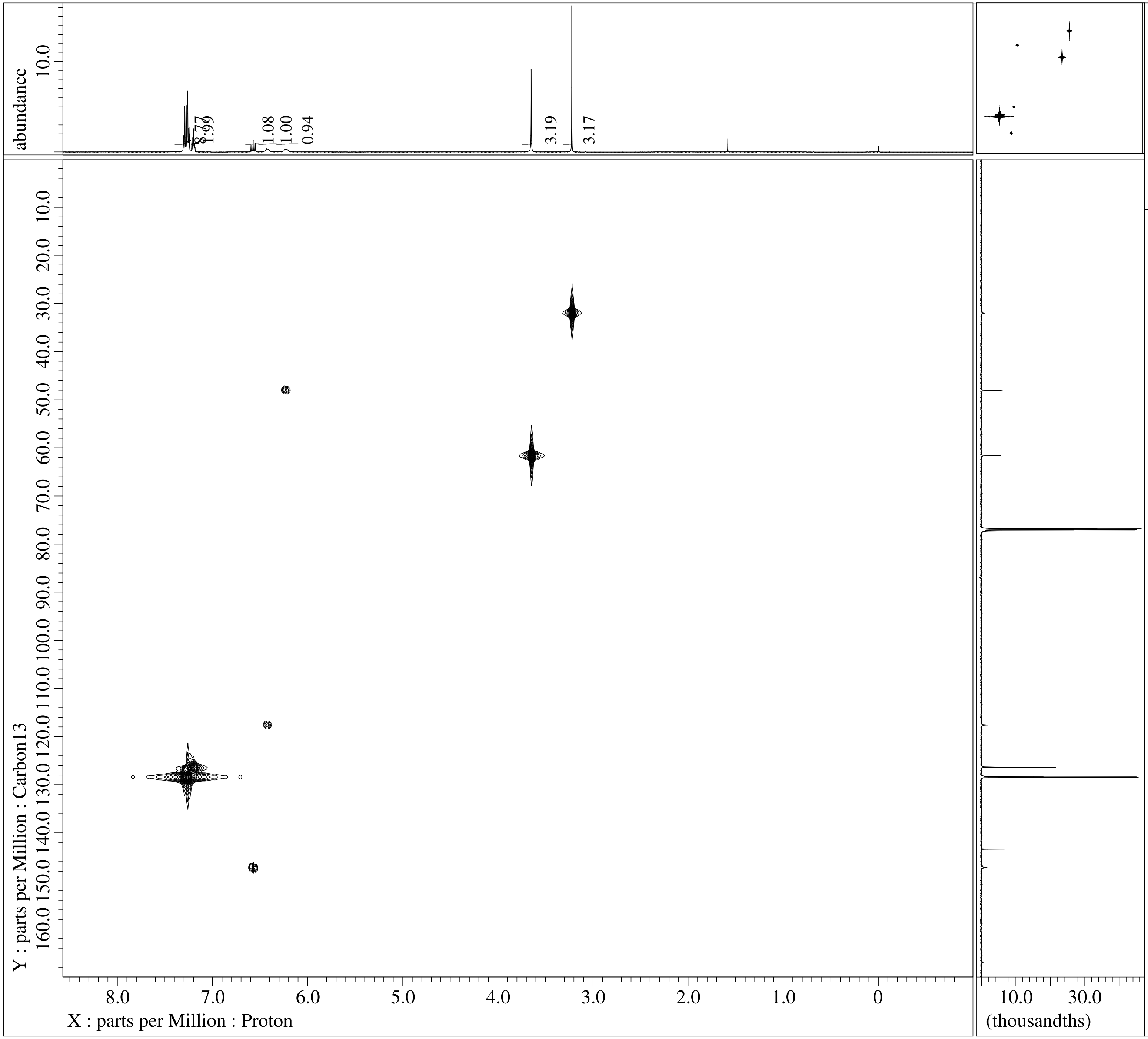


```
---- PROCESSING PARAMETERS ----
sinbell4( -60, 160 )
zerofill( 1 )
fft( 1, TRUE, TRUE )
ppm
[transpose]
sinbell4( -60, 160 )
zerofill( 2 )
fft( 1, TRUE, TRUE )
ppm
abs
```

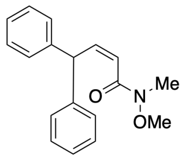

(*Z*)-16l

```
Filename             = HT-1631-PTLC 1_HMQC_HMQ
Author               = delta
Experiment           = hmqc.jxp
Sample_Id            = HT-1631-PTLC 1_HMQC
Solvent              = CHLOROFORM-D
Creation_Time        = 16-JAN-2024 18:03:06
Revision_Time        = 10-APR-2024 20:03:09
Current_Time         = 10-APR-2024 20:03:37

Comment              = gradient enhanced HMQC
Data_Format          = 2D REAL REAL
Dim_Size             = 819, 512
Dim_Title            = Proton Carbon13
Dim_Units            = [ppm] [ppm]
Dimensions           = X Y
Site                 = JNM-ECA500II
Spectrometer         = DELTA2_NMR

Field_Strength       = 11.7473579[T] (500[MHz]
X_Acq_Duration       = 0.16883712[s]
X_Domain             = 1H
X_Freq               = 500.15991521[MHz]
X_Offset             = 3.72941339[ppm]
X_Points             = 1024
X_Prescans           = 4
X_Resolution         = 5.92286815[Hz]
X_Sweep              = 6.06501698[kHz]
X_Sweep_Clipped      = 4.85201359[kHz]
Y_Domain             = 13C
Y_Freq               = 125.76529768[MHz]
Y_Offset             = 85[ppm]
Y_Points             = 256
Y_Prescans           = 0
Y_Resolution         = 83.60980308[Hz]
Y_Sweep              = 21.40410959[kHz]
Tri_Domain           = Proton
Tri_Freq             = 500.15991521[MHz]
Tri_Offset           = 5.0[ppm]
Clipped              = FALSE
Scans                = 16
Total_Scans          = 4096

Relaxation_Delay     = 1.5[s]
Recvr_Gain           = 50
Temp_Get             = 20.5[dC]
X_Acq_Time           = 0.16883712[s]
X_Atn                = 3.2[dB]
X_Gamma              = 42576375
X_Pulse              = 7.59[us]
```

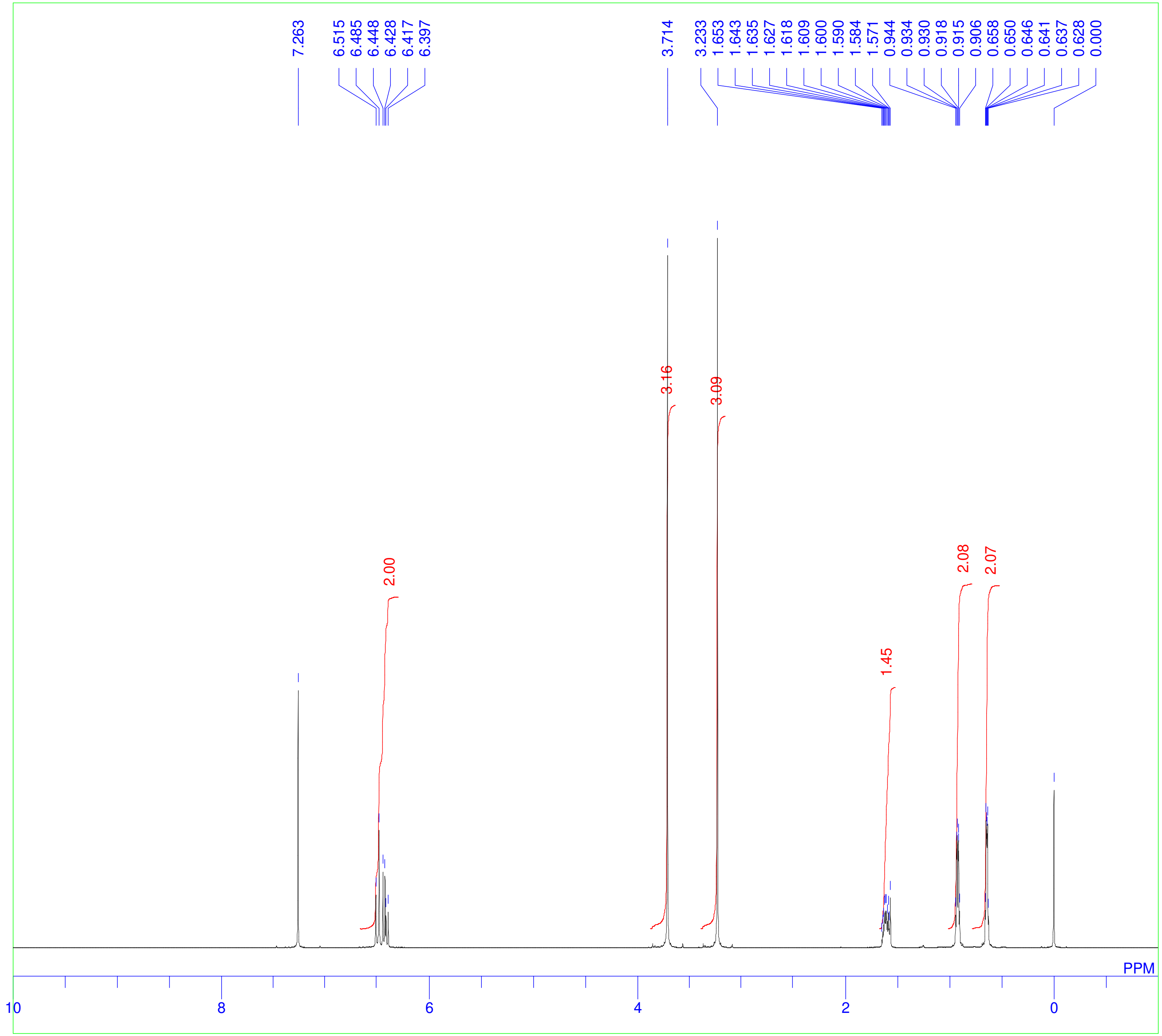

DFILE	(E)-16m_1H.als
COMNT	
DATIM	2023-04-18 14:12:13
OBNUC	1H
EXMOD	proton.jxp
OBFRQ	500.16 MHz
OBSET	2.41 KHz
OBFIN	6.01 Hz
POINT	13107
FREQU	7507.51 Hz
SCANS	8
ACQTM	1.7459 sec
PD	5.0000 sec
PW1	3.84 usec
IRNUC	1H
CTEMP	23.8 c
SLVNT	CDCL3
EXREF	0.00 ppm
BF	1.00 Hz
RGAIN	44

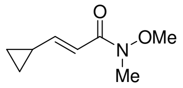

(*E*)-16m

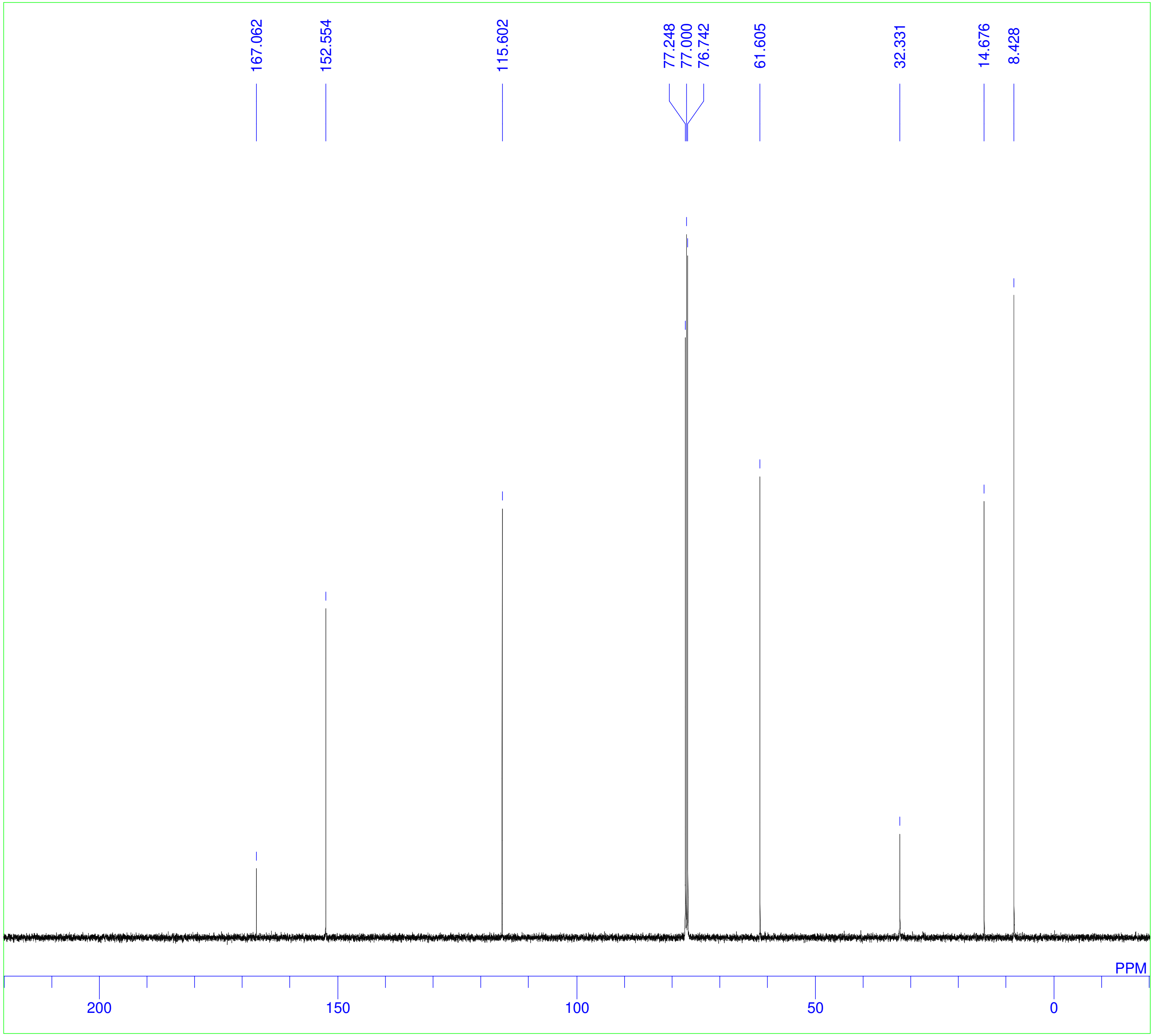

DFILE	(E)-16m_13C.als
COMNT	
DATIM	2023-04-18 20:00:36
OBNUC	13C
EXMOD	carbon.jxp
OBFRQ	125.77 MHz
OBSET	7.87 KHz
OBFIN	4.21 Hz
POINT	26214
FREQU	31446.54 Hz
SCANS	1024
ACQTM	0.8336 sec
PD	2.0000 sec
PW1	3.87 usec
IRNUC	1H
CTEMP	24.2 c
SLVNT	CDCL3
EXREF	77.00 ppm
BF	1.00 Hz
RGAIN	30

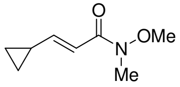

(*E*)-16m

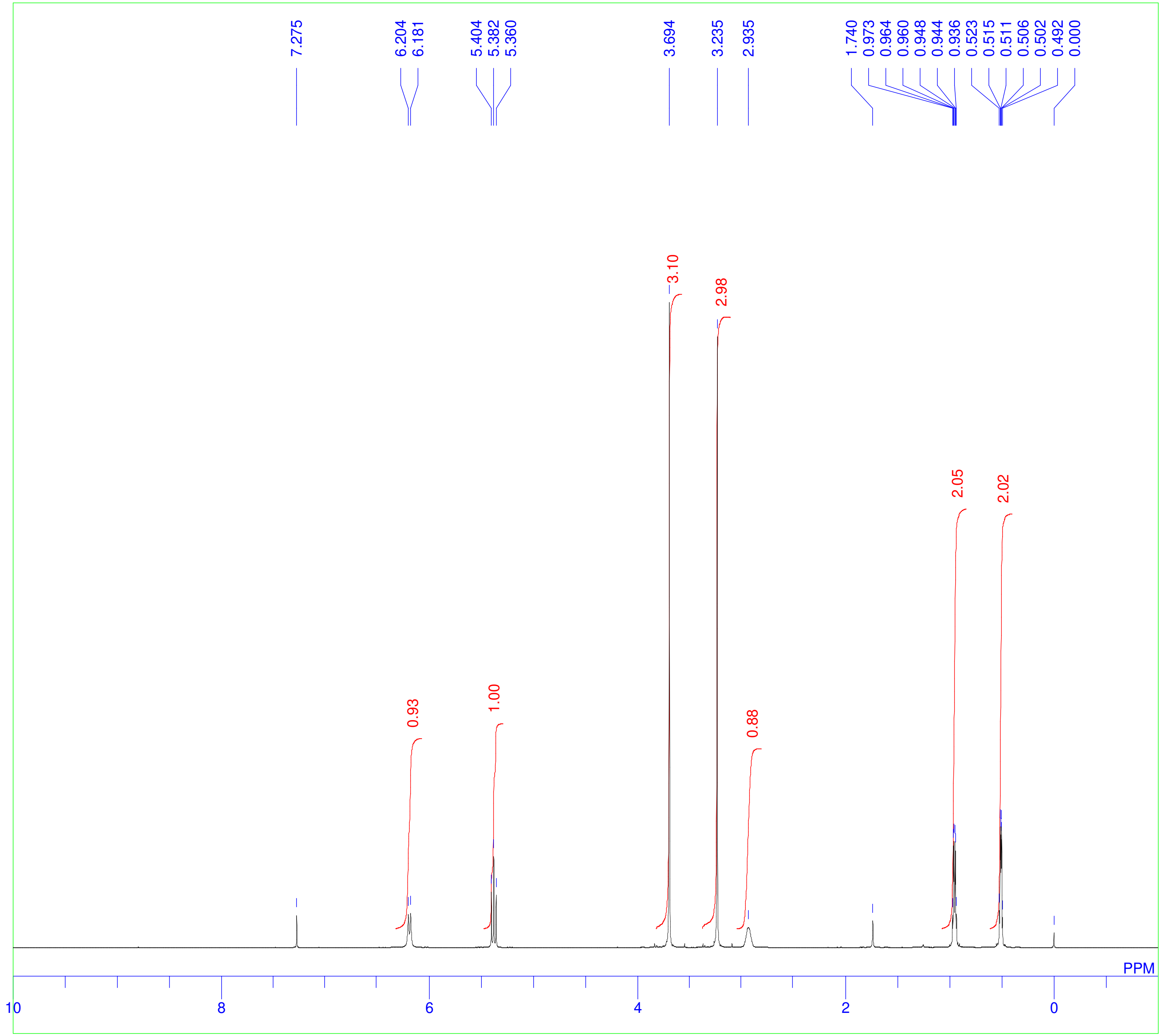

DFILE (Z)-16m_1H.als
COMNT
DATIM 2023-11-13 18:58:32
OBNUC 1H
EXMOD proton.jxp
OBFRQ 500.16 MHz
OBSET 2.41 KHz
OBFIN 6.01 Hz
POINT 13107
FREQU 7507.51 Hz
SCANS 8
ACQTM 1.7459 sec
PD 5.0000 sec
PW1 3.80 usec
IRNUC 1H
CTEMP 23.7 c
SLVNT CDCL3
EXREF 0.00 ppm
BF 1.00 Hz
RGAIN 30

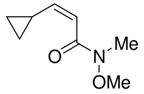

(*Z*)-16m

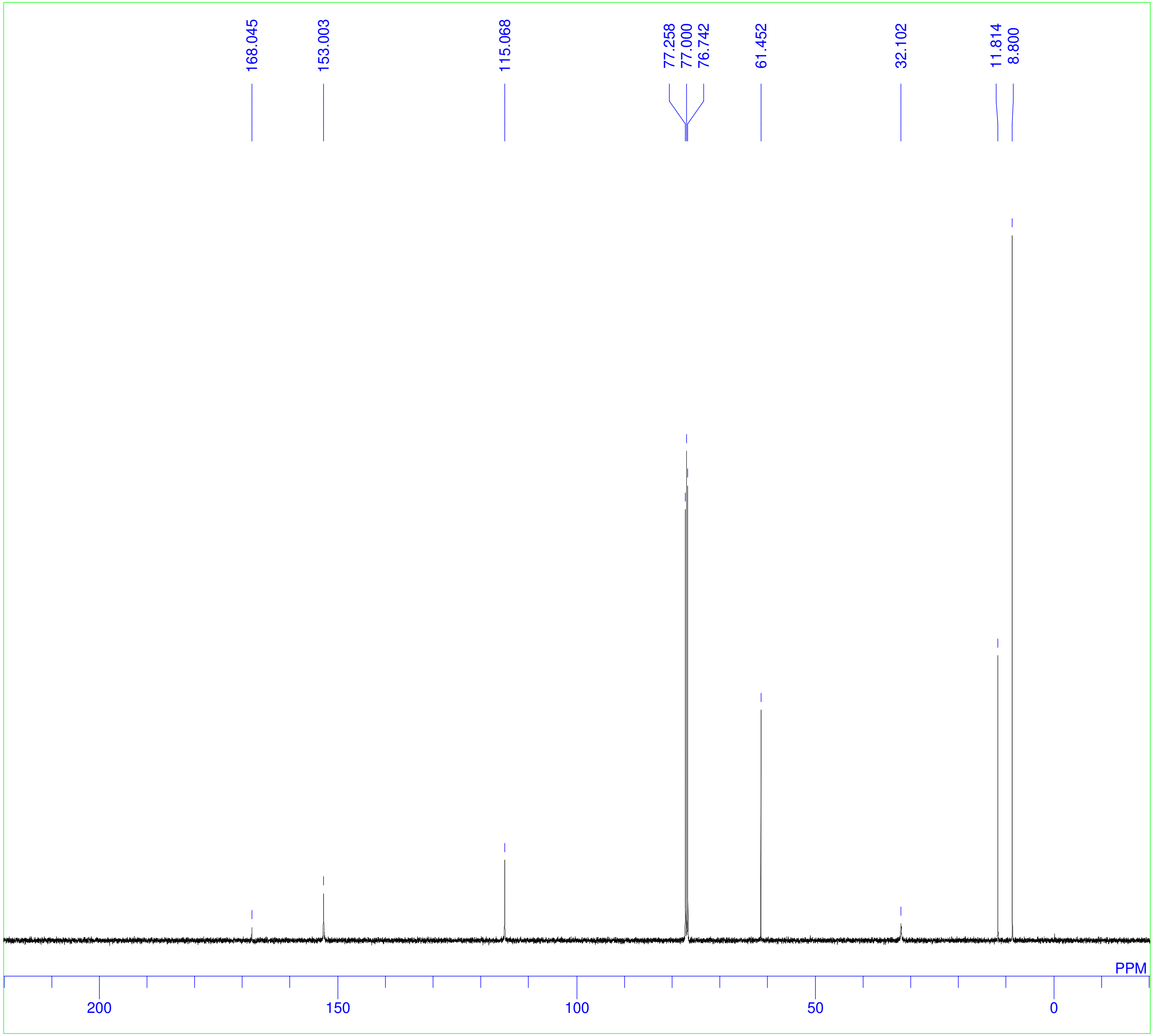

DFILE	(Z)-16m_13C.als
COMNT	
DATIM	2023-11-13 19:00:08
OBNUC	13C
EXMOD	carbon.jxp
OBFRQ	125.77 MHz
OBSET	7.87 KHz
OBFIN	4.21 Hz
POINT	26214
FREQU	31446.54 Hz
SCANS	1024
ACQTM	0.8336 sec
PD	2.0000 sec
PW1	4.30 usec
IRNUC	1H
CTEMP	23.9 c
SLVNT	CDCL3
EXREF	77.00 ppm
BF	1.00 Hz
RGAIN	30

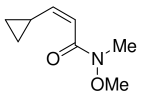

(*Z*)-16m

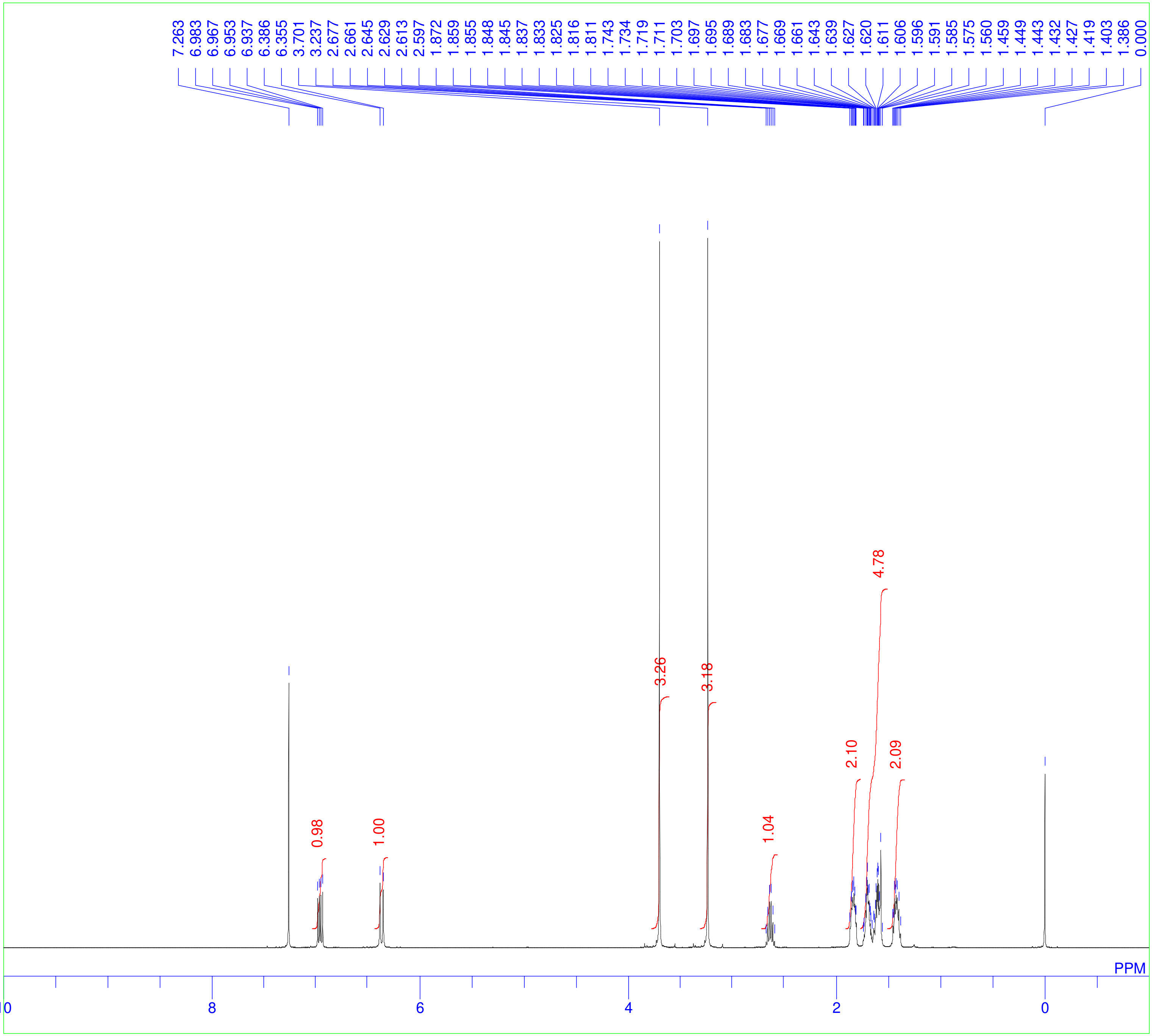

DFILE	(E)-16n_1H.als
COMNT	
DATIM	2023-04-18 14:26:09
OBNUC	1H
EXMOD	proton.jxp
OBFRQ	500.16 MHz
OBSET	2.41 KHz
OBFIN	6.01 Hz
POINT	13107
FREQU	7507.51 Hz
SCANS	8
ACQTM	1.7459 sec
PD	5.0000 sec
PW1	3.84 usec
IRNUC	1H
CTEMP	23.7 c
SLVNT	CDCL3
EXREF	0.00 ppm
BF	1.00 Hz
RGAIN	44

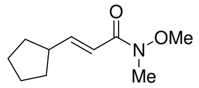

(*E*)-16n

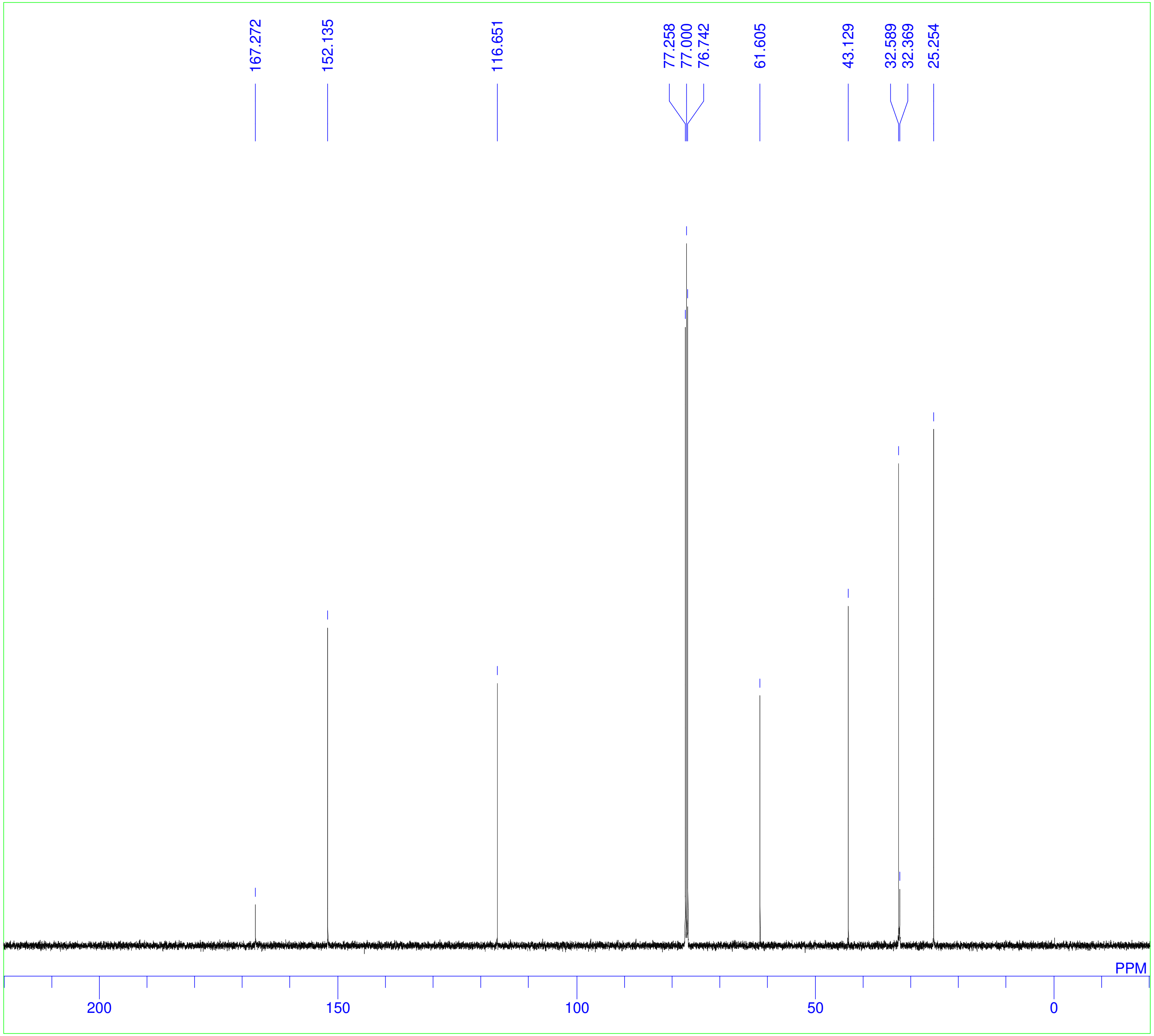

DFILE (E)-16n_13C.als
COMNT
DATIM 2023-04-18 20:54:01
OBNUC 13C
EXMOD carbon.jxp
OBFRQ 125.77 MHz
OBSET 7.87 KHz
OBFIN 4.21 Hz
POINT 26214
FREQU 31446.54 Hz
SCANS 1024
ACQTM 0.8336 sec
PD 2.0000 sec
PW1 3.87 usec
IRNUC 1H
CTEMP 23.9 c
SLVNT CDCL3
EXREF 77.00 ppm
BF 1.00 Hz
RGAIN 30

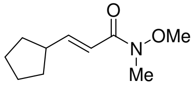

(*E*)-16n

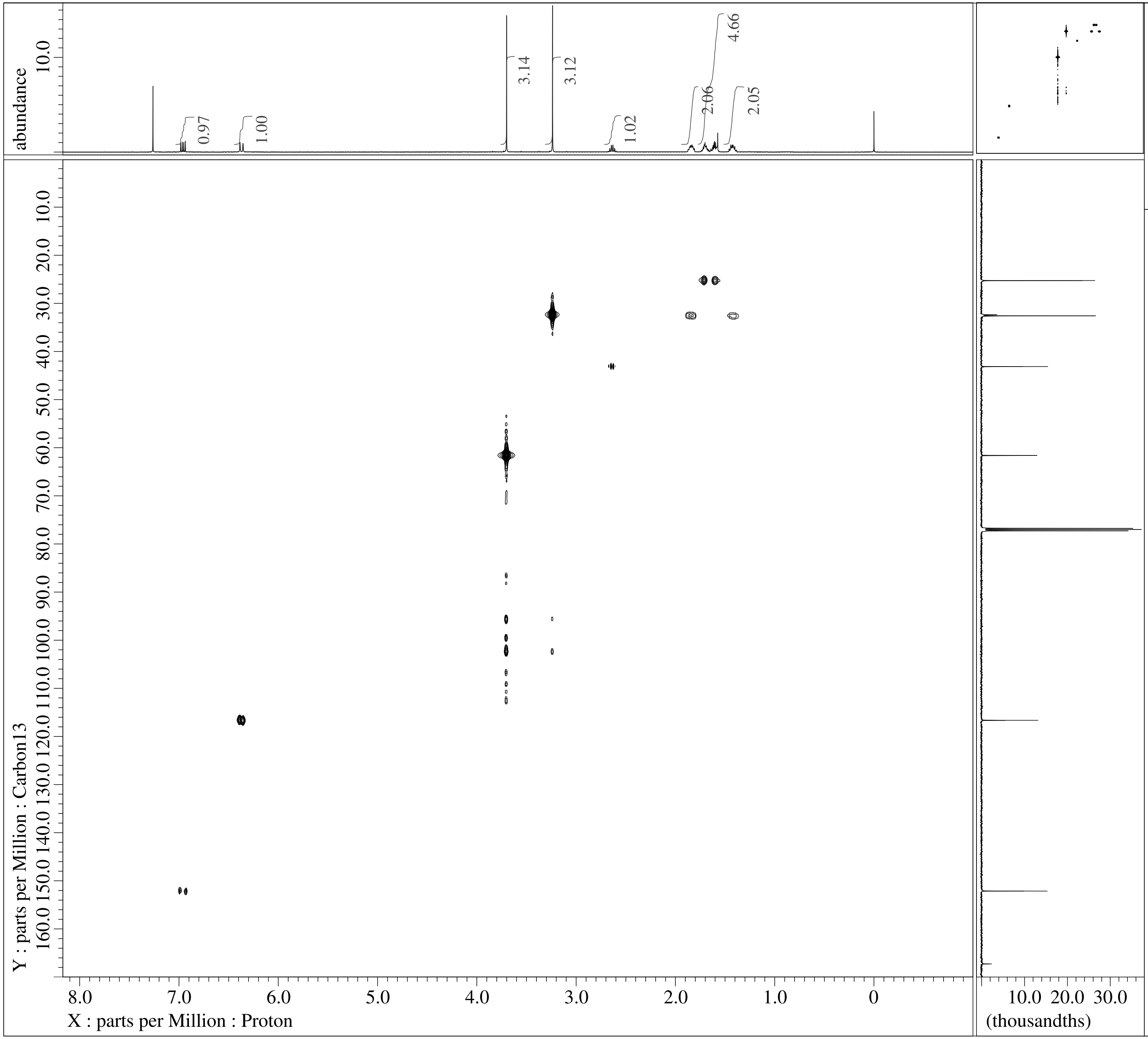


```
---- PROCESSING PARAMETERS ----
sinbell4( -60, 160 )
zerofill( 1 )
fft( 1, TRUE, TRUE )
ppm
[transpose]
sinbell4( -60, 160 )
zerofill( 2 )
fft( 1, TRUE, TRUE )
ppm
abs
```

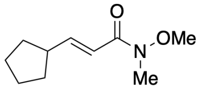

(*E*)-16n

```
Filename            = HT-1358-PTLC 2_HMQC_HMQ
Author              = delta
Experiment          = hmqc.jxp
Sample_Id           = HT-1358-PTLC 2_HMQC
Solvent             = CHLOROFORM-D
Creation_Time       =  4-MAY-2023 16:05:22
Revision_Time       = 10-APR-2024 21:36:16
Current_Time        = 10-APR-2024 21:36:49

Comment             = gradient enhanced HMQC
Data_Format         = 2D REAL REAL
Dim_Size            = 819, 512
Dim_Title           = Proton Carbon13
Dim_Units           = [ppm] [ppm]
Dimensions          = X Y
Site                = JNM-ECA500II
Spectrometer        = DELTA2_NMR

Field_Strength      = 11.7473579[T] (500[MHz]
X_Acq_Duration      = 0.17711104[s]
X_Domain            = 1H
X_Freq              = 500.15991521[MHz]
X_Offset            = 3.52270688[ppm]
X_Points            = 1024
X_Prescans          = 4
X_Resolution        = 5.64617542[Hz]
X_Sweep             = 5.78168363[kHz]
X_Sweep_Clipped     = 4.6253469[kHz]
Y_Domain            = 13C
Y_Freq              = 125.76529768[MHz]
Y_Offset            = 85[ppm]
Y_Points            = 256
Y_Prescans          = 0
Y_Resolution        = 83.60980308[Hz]
Y_Sweep             = 21.40410959[kHz]
Tri_Domain          = Proton
Tri_Freq            = 500.15991521[MHz]
Tri_Offset          = 5.0[ppm]
Clipped             = FALSE
Scans               = 8
Total_Scans         = 2048

Relaxation_Delay    = 1.5[s]
Recvr_Gain          = 50
Temp_Get            = 23.8[dC]
X_Acq_Time          = 0.17711104[s]
X_Atn               = 3.2[dB]
X_Gamma             = 42576375
X_Pulse             = 7.68[us]
```

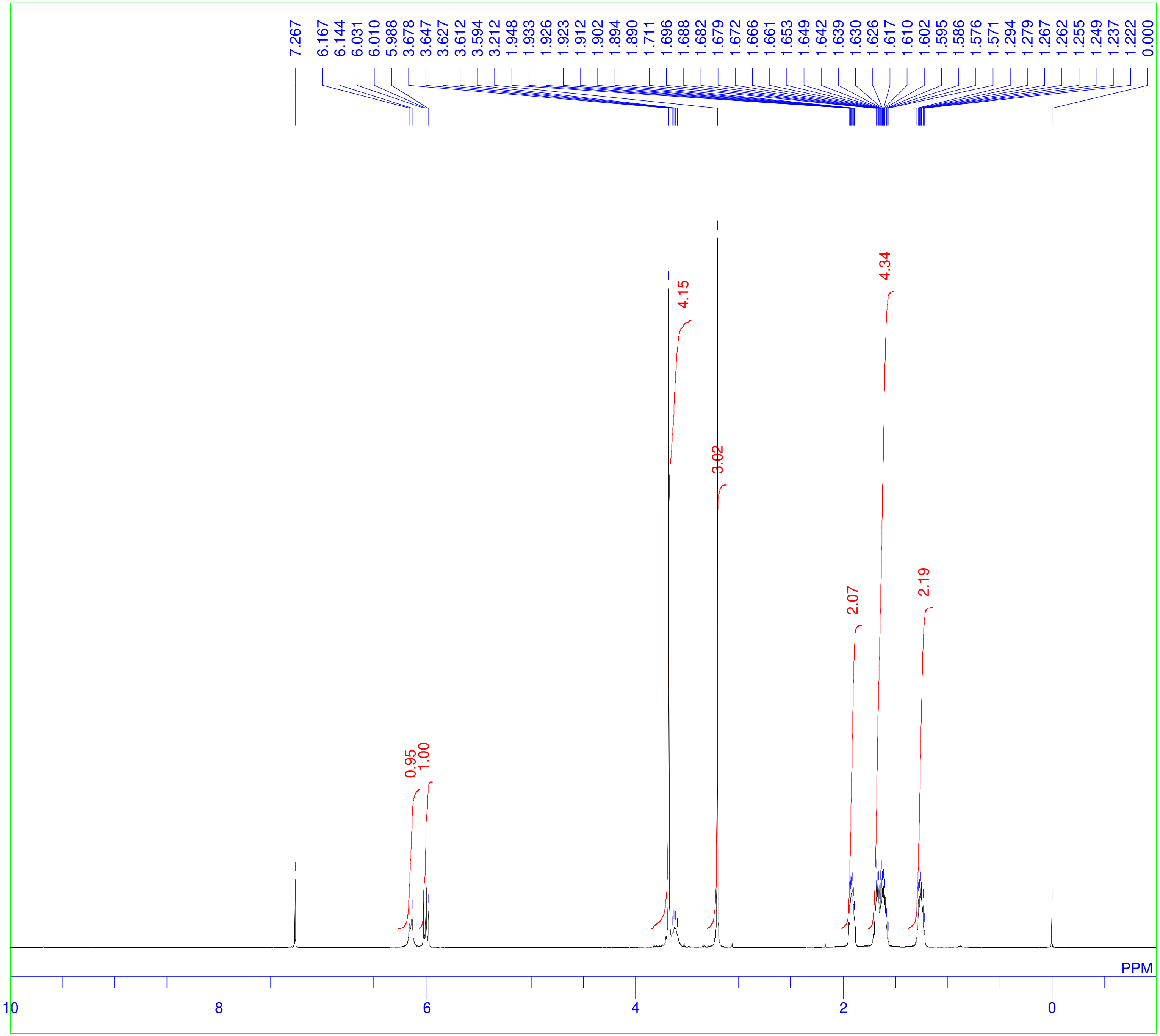

DFILE (Z)-16n_1H.als
COMNT
DATIM 2023-11-10 17:03:31
OBNUC 1H
EXMOD proton.jxp
OBFRQ 500.16 MHz
OBSET 2.41 KHz
OBFIN 6.01 Hz
POINT 13107
FREQU 7507.51 Hz
SCANS 8
ACQTM 1.7459 sec
PD 5.0000 sec
PW1 3.80 usec
IRNUC 1H
CTEMP 24.2 c
SLVNT CDCL3
EXREF 0.00 ppm
BF 1.00 Hz
RGAIN 34

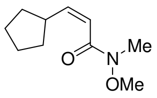

(*Z*)-16n

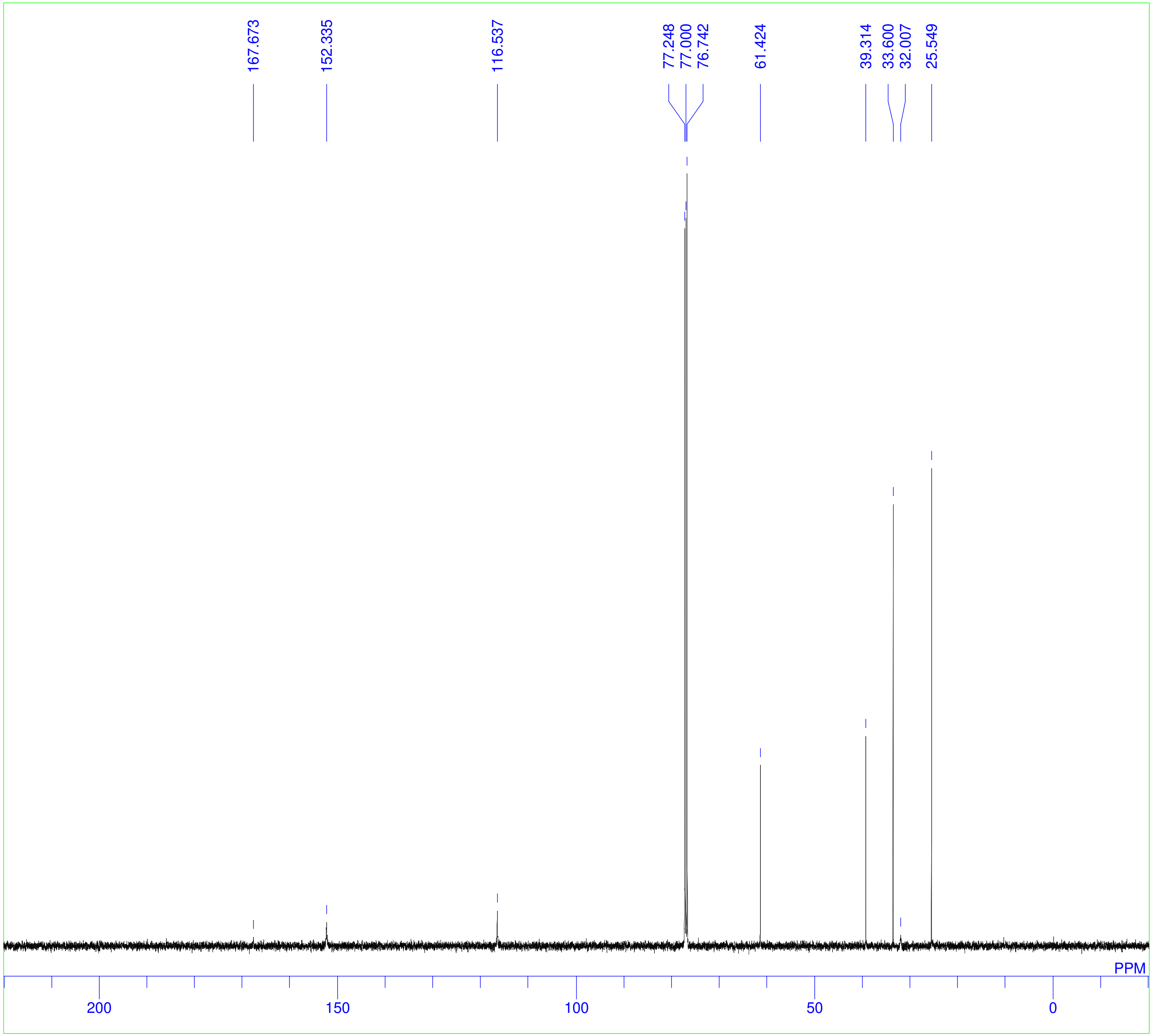

DFILE	(Z)-16n_13C.als
COMNT	
DATIM	2023-11-10 17:05:26
OBNUC	13C
EXMOD	carbon.jxp
OBFRQ	125.77 MHz
OBSET	7.87 KHz
OBFIN	4.21 Hz
POINT	26214
FREQU	31446.54 Hz
SCANS	1024
ACQTM	0.8336 sec
PD	2.0000 sec
PW1	4.30 usec
IRNUC	1H
CTEMP	23.9 c
SLVNT	CDCL3
EXREF	77.00 ppm
BF	1.00 Hz
RGAIN	36

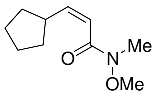

(*Z*)-16n

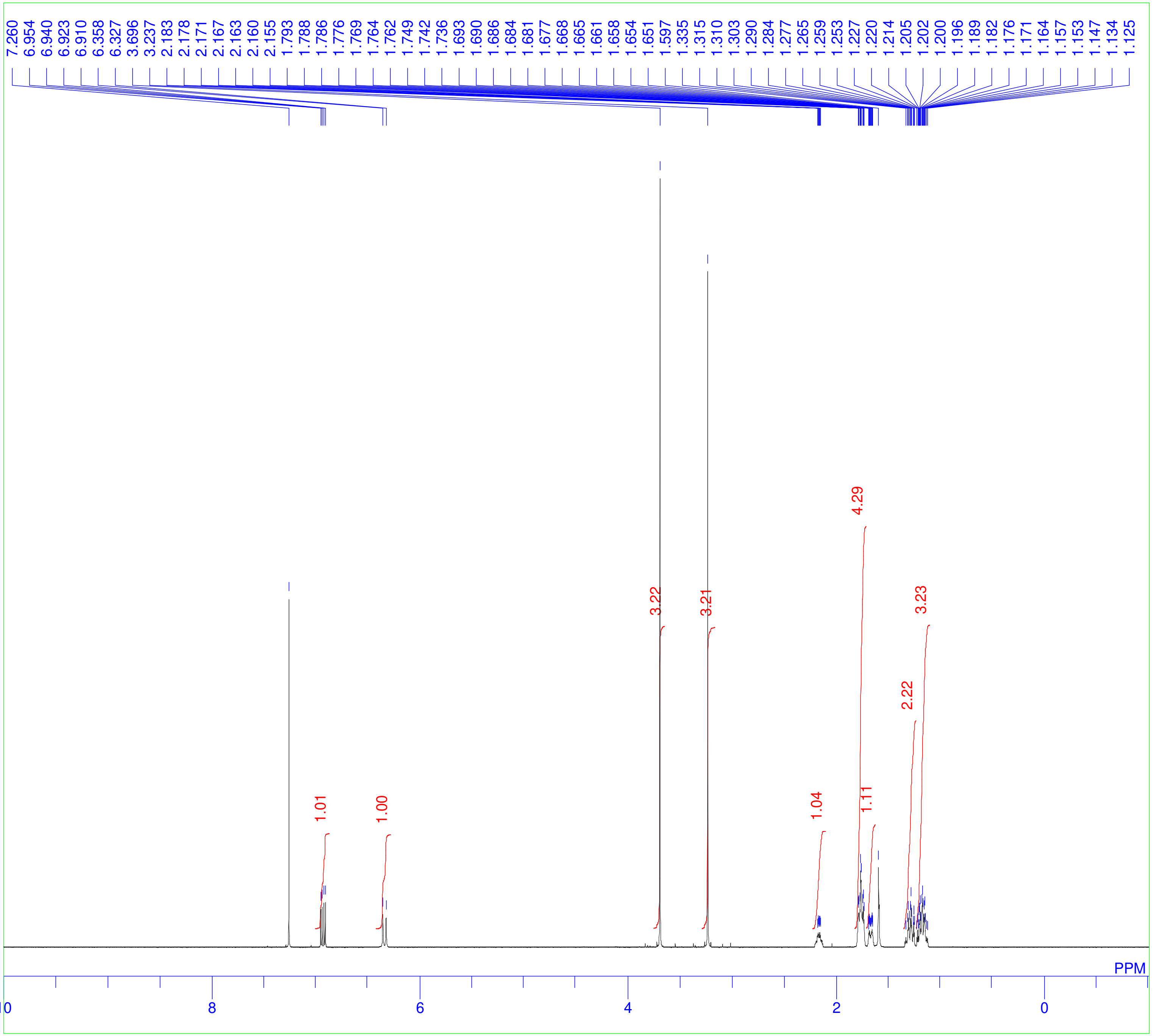

DFILE	(E)-16o_1H.als
COMNT	
DATIM	2022-01-12 16:00:24
OBNUC	1H
EXMOD	proton.jxp
OBFRQ	500.16 MHz
OBSET	2.41 KHz
OBFIN	6.01 Hz
POINT	13107
FREQU	7507.51 Hz
SCANS	8
ACQTM	1.7459 sec
PD	5.0000 sec
PW1	3.84 usec
IRNUC	1H
CTEMP	19.5 c
SLVNT	CDCL3
EXREF	7.26 ppm
BF	0.30 Hz
RGAIN	42

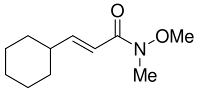

(*E*)-16o

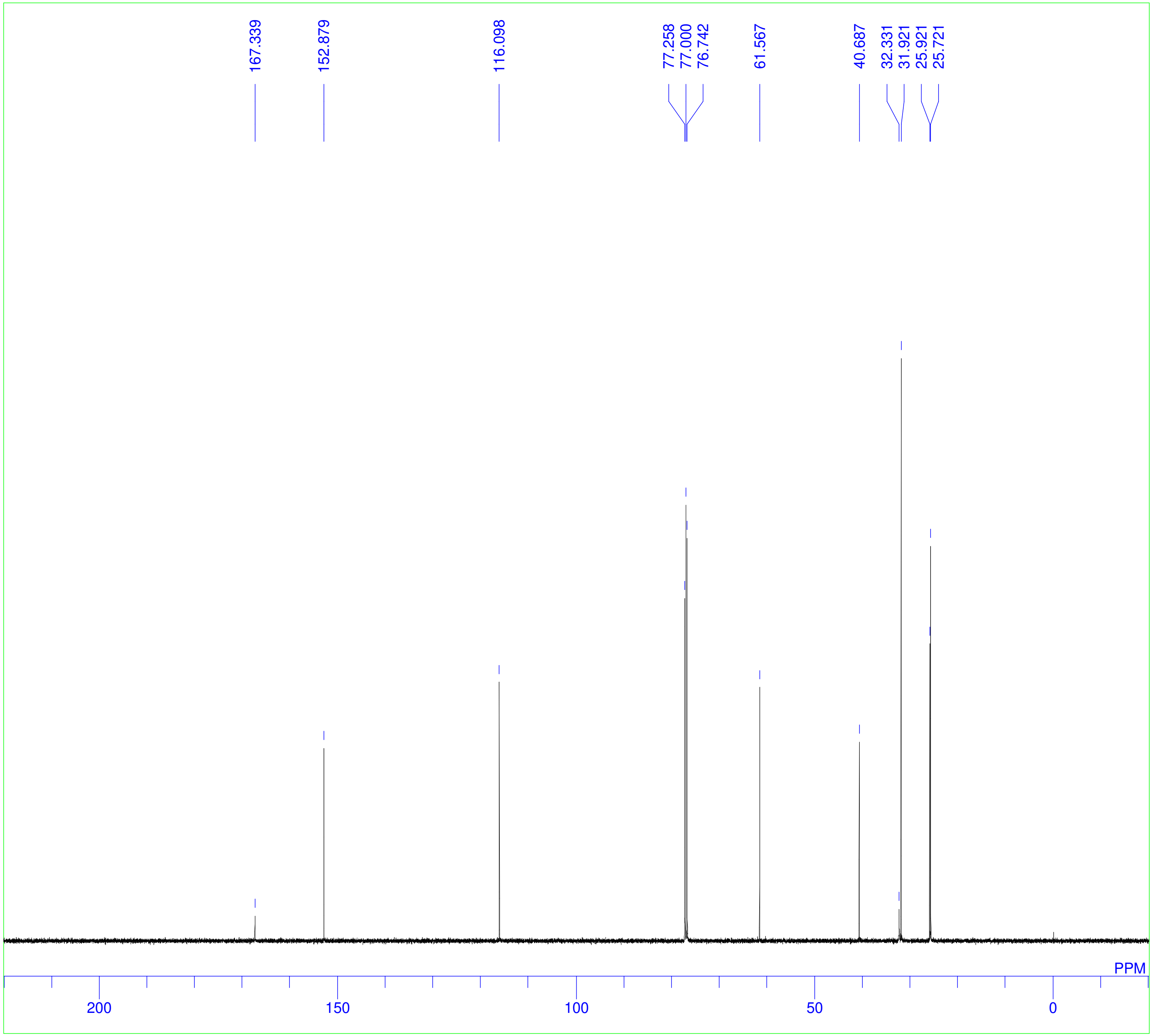

DFILE	(E)-16o_13C.als
COMNT	
DATIM	2023-01-23 20:45:20
OBNUC	13C
EXMOD	carbon.jxp
OBFRQ	125.77 MHz
OBSET	7.87 KHz
OBFIN	4.21 Hz
POINT	26214
FREQU	31446.54 Hz
SCANS	1024
ACQTM	0.8336 sec
PD	2.0000 sec
PW1	3.87 usec
IRNUC	1H
CTEMP	20.7 c
SLVNT	CDCL3
EXREF	77.00 ppm
BF	0.30 Hz
RGAIN	30

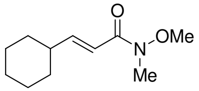

(*E*)-16o

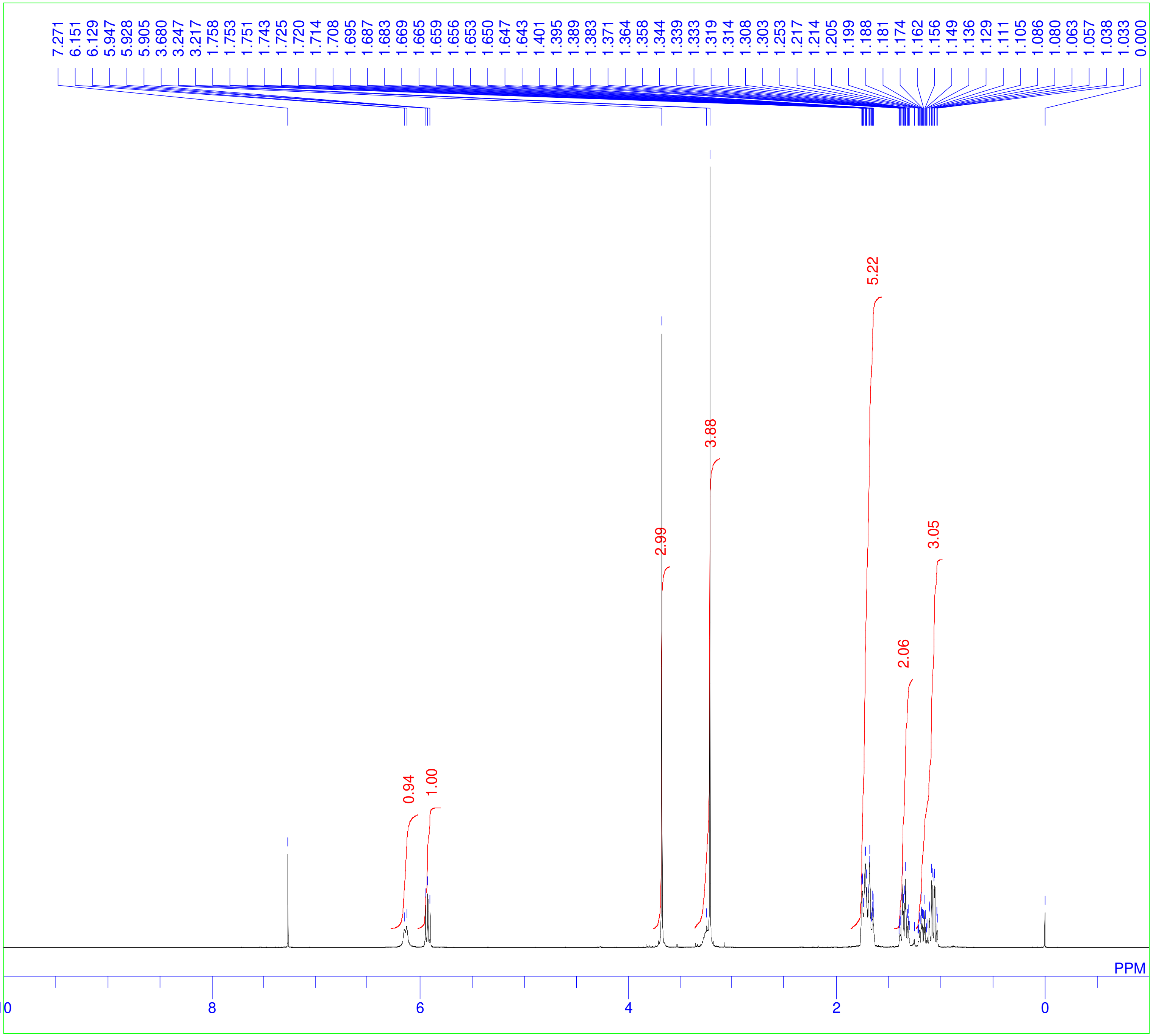

DFILE (Z)-16o_1H.als
COMNT
DATIM 2023-01-25 19:40:07
OBNUC 1H
EXMOD proton.jxp
OBFRQ 500.16 MHz
OBSET 2.41 KHz
OBFIN 6.01 Hz
POINT 13107
FREQU 7507.51 Hz
SCANS 8
ACQTM 1.7459 sec
PD 5.0000 sec
PW1 3.84 usec
IRNUC 1H
CTEMP 18.7 c
SLVNT CDCL3
EXREF 0.00 ppm
BF 0.30 Hz
RGAIN 30

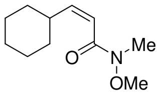

(*Z*)-16o

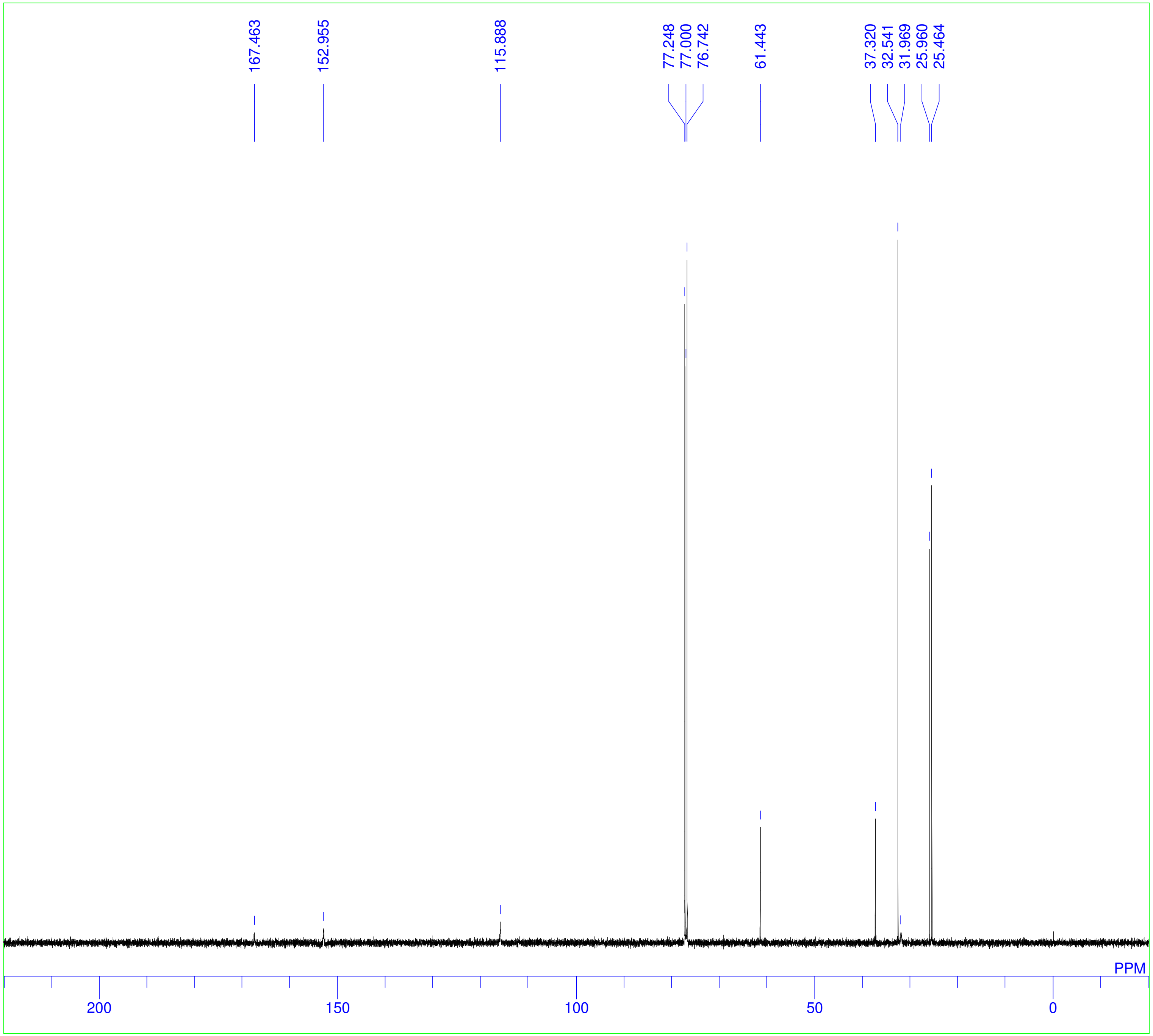

DFILE	(Z)-16o_13C.als
COMNT	
DATIM	2023-01-25 19:42:29
OBNUC	13C
EXMOD	carbon.jxp
OBFRQ	125.77 MHz
OBSET	7.87 KHz
OBFIN	4.21 Hz
POINT	26214
FREQU	31446.54 Hz
SCANS	1024
ACQTM	0.8336 sec
PD	2.0000 sec
PW1	3.87 usec
IRNUC	1H
CTEMP	18.6 c
SLVNT	CDCL3
EXREF	77.00 ppm
BF	0.30 Hz
RGAIN	24

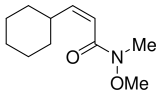

(*Z*)-16o

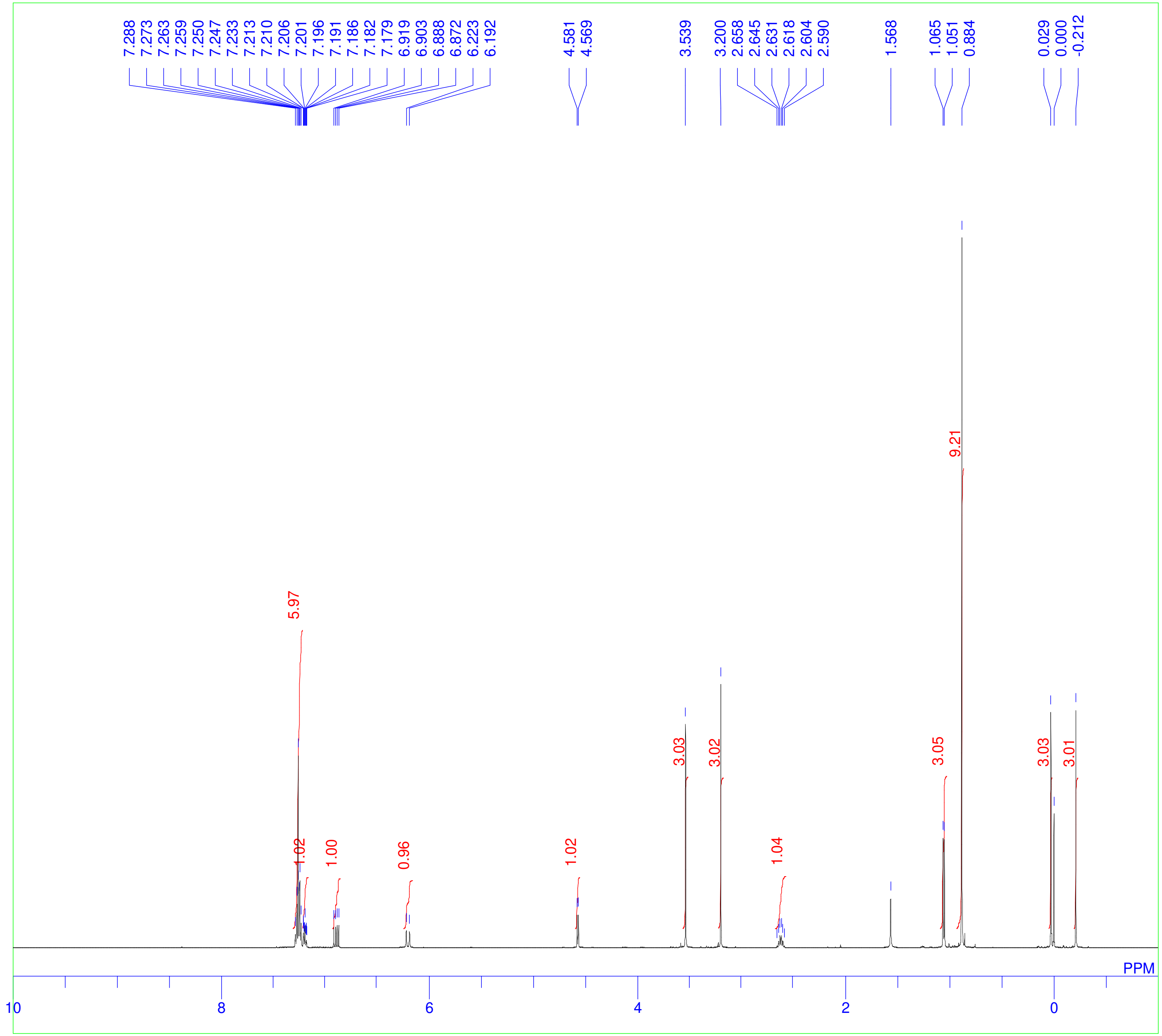

DFILE (E)-16p_1H.als
COMNT
DATIM 2021-12-19 21:00:26
OBNUC 1H
EXMOD proton.jxp
OBFRQ 500.16 MHz
OBSET 2.41 KHz
OBFIN 6.01 Hz
POINT 13107
FREQU 7507.51 Hz
SCANS 8
ACQTM 1.7459 sec
PD 5.0000 sec
PW1 3.84 usec
IRNUC 1H
CTEMP 20.5 c
SLVNT CDCL3
EXREF 0.00 ppm
BF 0.30 Hz
RGAIN 44

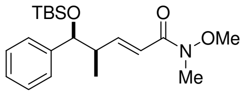

(*E*)-16p

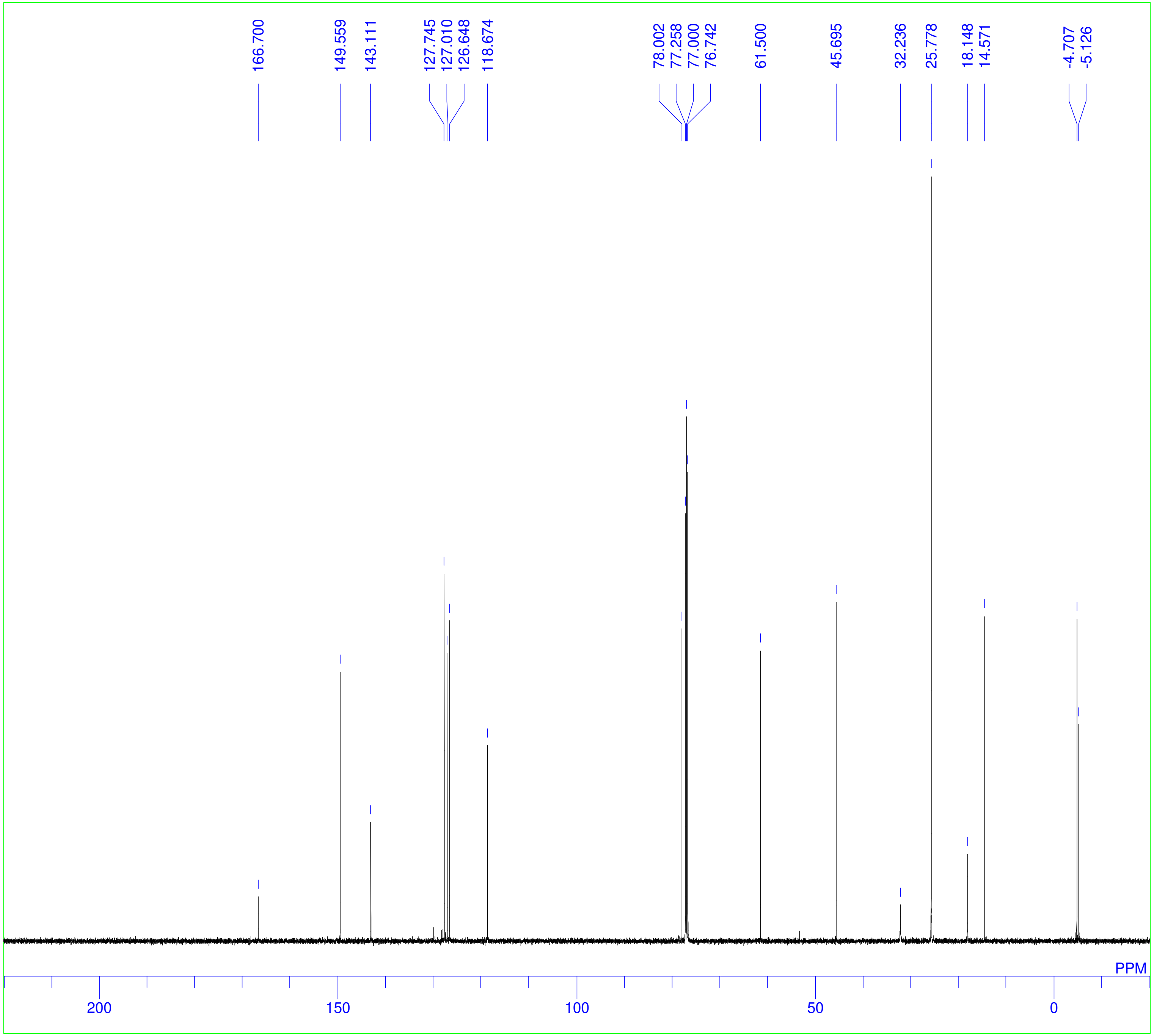

DFILE	(E)-16p_13C.als
COMNT	
DATIM	2022-02-28 20:25:04
OBNUC	13C
EXMOD	carbon.jxp
OBFRQ	125.77 MHz
OBSET	7.87 KHz
OBFIN	4.21 Hz
POINT	26214
FREQU	31446.54 Hz
SCANS	1024
ACQTM	0.8336 sec
PD	2.0000 sec
PW1	3.87 usec
IRNUC	1H
CTEMP	21.1 c
SLVNT	CDCL3
EXREF	77.00 ppm
BF	0.30 Hz
RGAIN	24

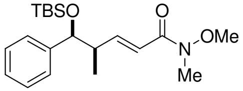

(*E*)-16p

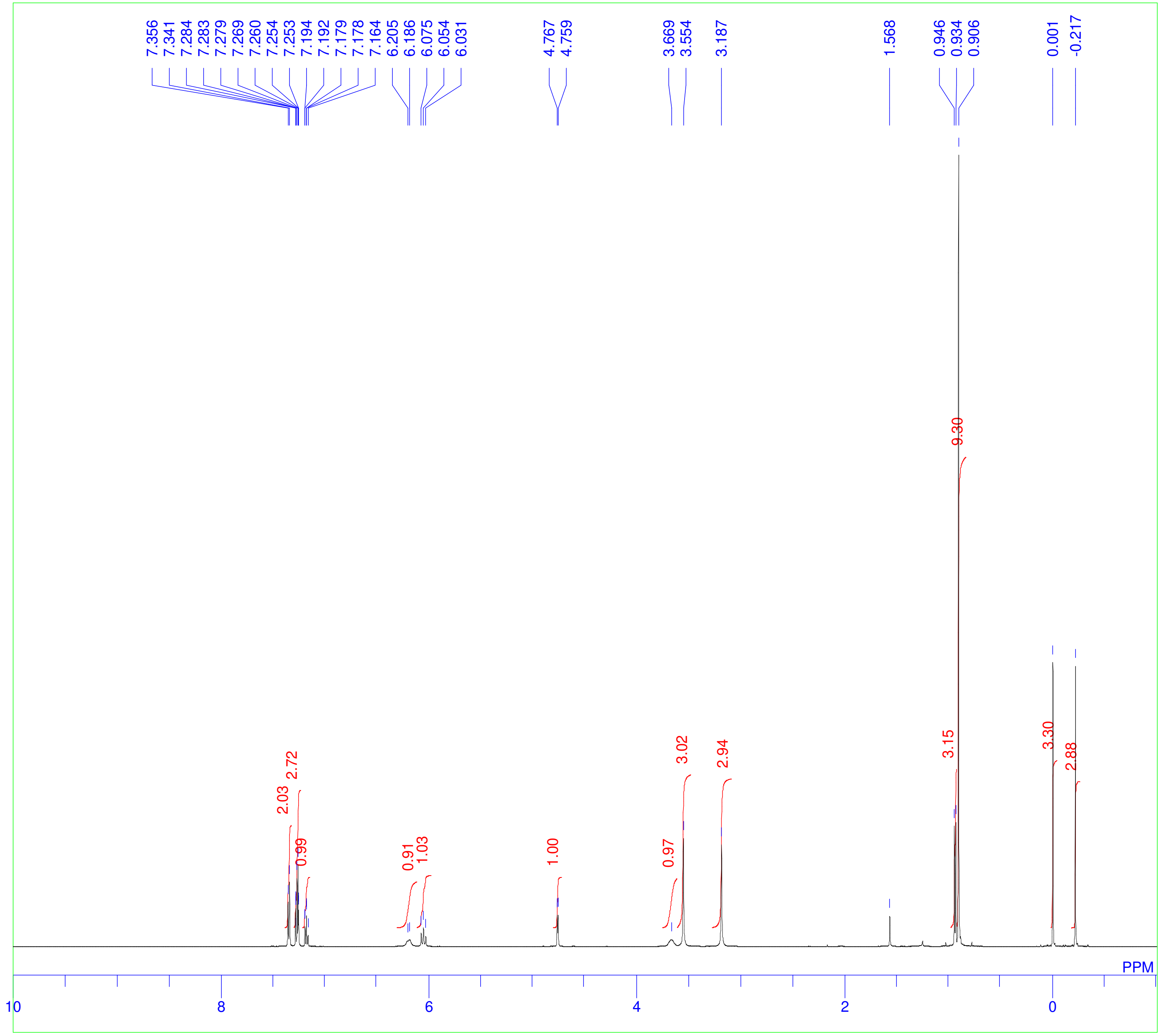

DFILE (Z)-16p_1H.als
COMNT
DATIM 2021-12-04 11:03:50
OBNUC 1H
EXMOD proton.jxp
OBFRQ 500.16 MHz
OBSET 2.41 KHz
OBFIN 6.01 Hz
POINT 13107
FREQU 7507.51 Hz
SCANS 8
ACQTM 1.7459 sec
PD 5.0000 sec
PW1 3.84 usec
IRNUC 1H
CTEMP 22.4 c
SLVNT CDCL3
EXREF 7.26 ppm
BF 0.30 Hz
RGAIN 38

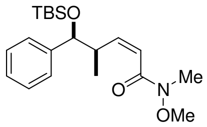

(Z)-16p

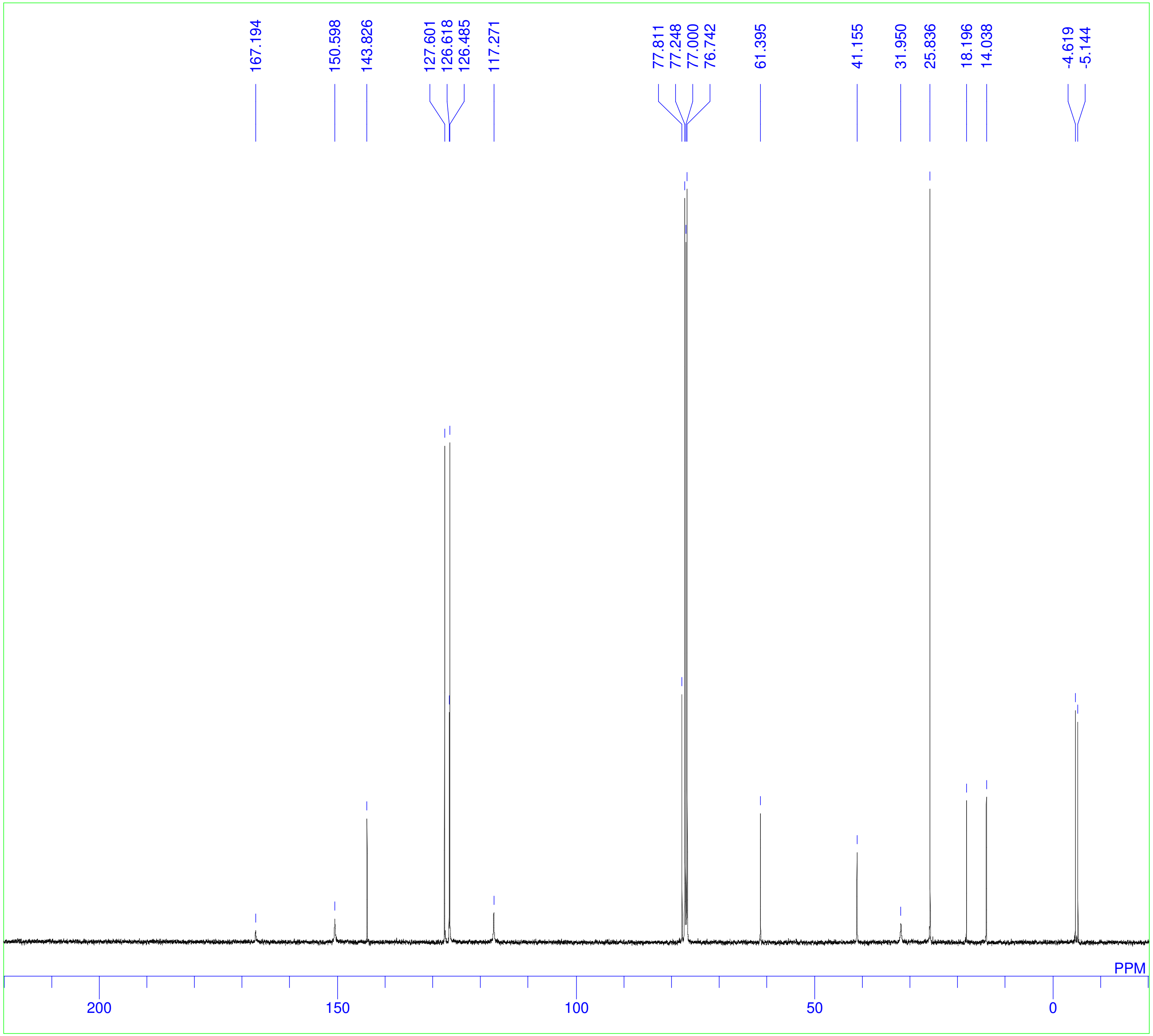

DFILE	(Z)-16p_13C.als
COMNT	
DATIM	2022-03-01 08:30:50
OBNUC	13C
EXMOD	carbon.jxp
OBFRQ	125.77 MHz
OBSET	7.87 KHz
OBFIN	4.21 Hz
POINT	32768
FREQU	39308.18 Hz
SCANS	2048
ACQTM	0.8336 sec
PD	2.0000 sec
PW1	3.87 usec
IRNUC	1H
CTEMP	20.6 c
SLVNT	CDCL3
EXREF	77.00 ppm
BF	0.30 Hz
RGAIN	26

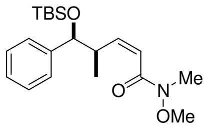

(*Z*)-16p

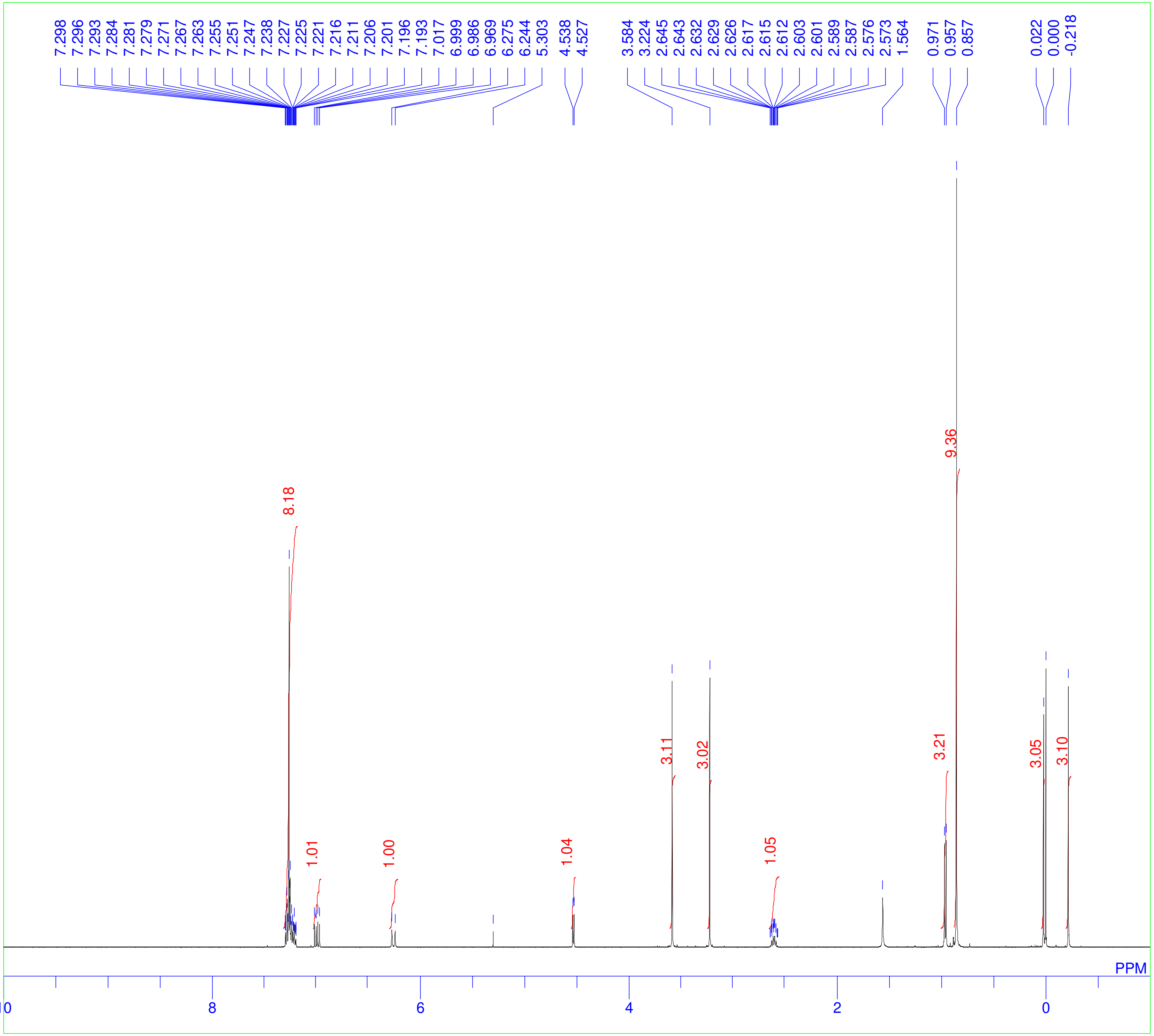

DFILE (E)-16q_1H.als
COMNT
DATIM 2022-02-19 19:21:06
OBNUC 1H
EXMOD proton.jxp
OBFRQ 500.16 MHz
OBSET 2.41 KHz
OBFIN 6.01 Hz
POINT 13107
FREQU 7507.51 Hz
SCANS 8
ACQTM 1.7459 sec
PD 5.0000 sec
PW1 3.84 usec
IRNUC 1H
CTEMP 19.2 c
SLVNT CDCL3
EXREF 0.00 ppm
BF 0.30 Hz
RGAIN 44

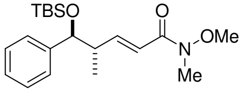

(*E*)-16q

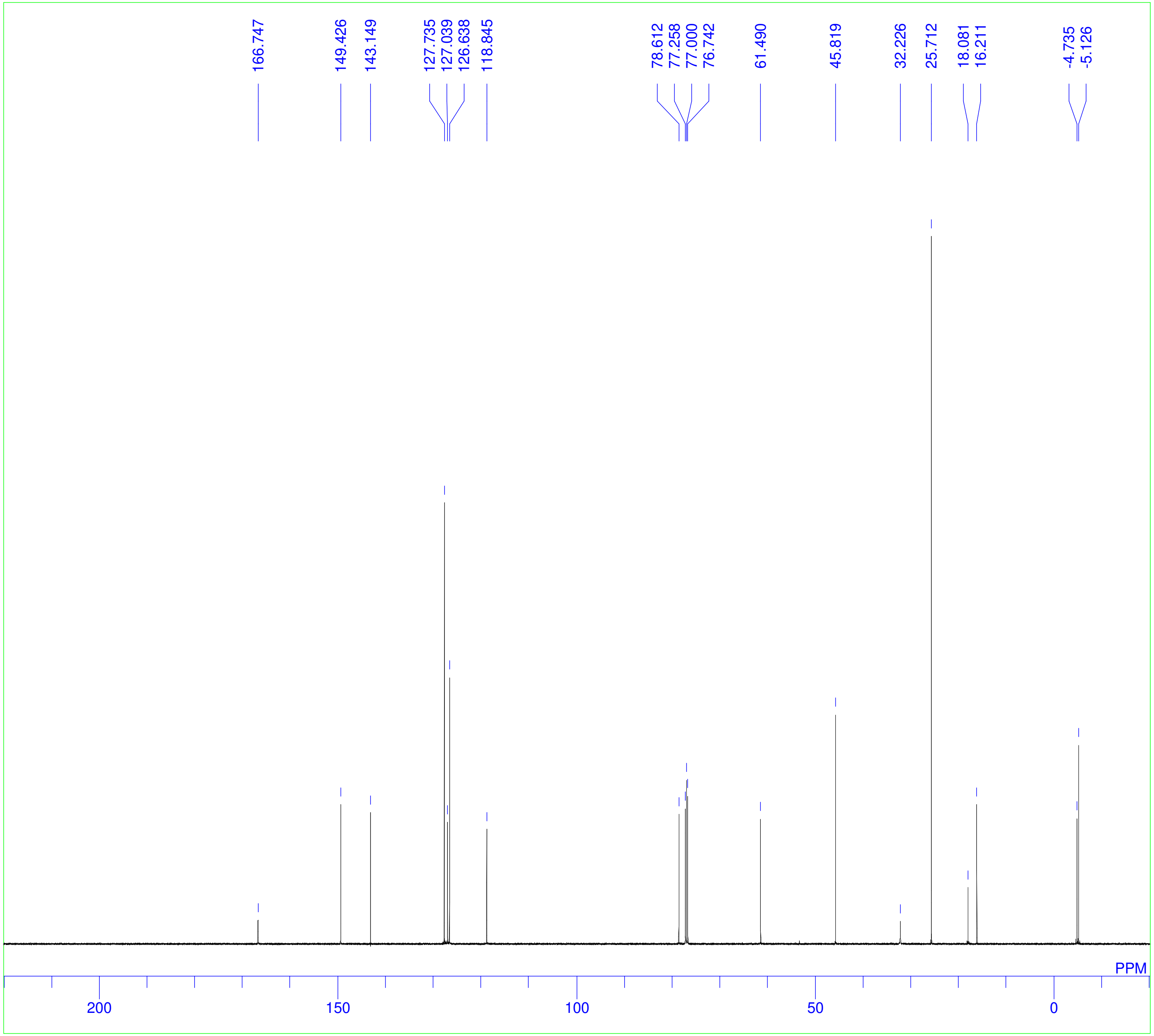

DFILE	(E)-16q_13C.als
COMNT	
DATIM	2022-03-01 14:06:33
OBNUC	13C
EXMOD	carbon.jxp
OBFRQ	125.77 MHz
OBSET	7.87 KHz
OBFIN	4.21 Hz
POINT	26214
FREQU	31446.54 Hz
SCANS	1024
ACQTM	0.8336 sec
PD	2.0000 sec
PW1	3.87 usec
IRNUC	1H
CTEMP	21.6 c
SLVNT	CDCL3
EXREF	77.00 ppm
BF	0.30 Hz
RGAIN	26

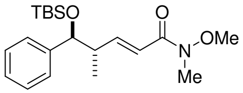

(*E*)-16q

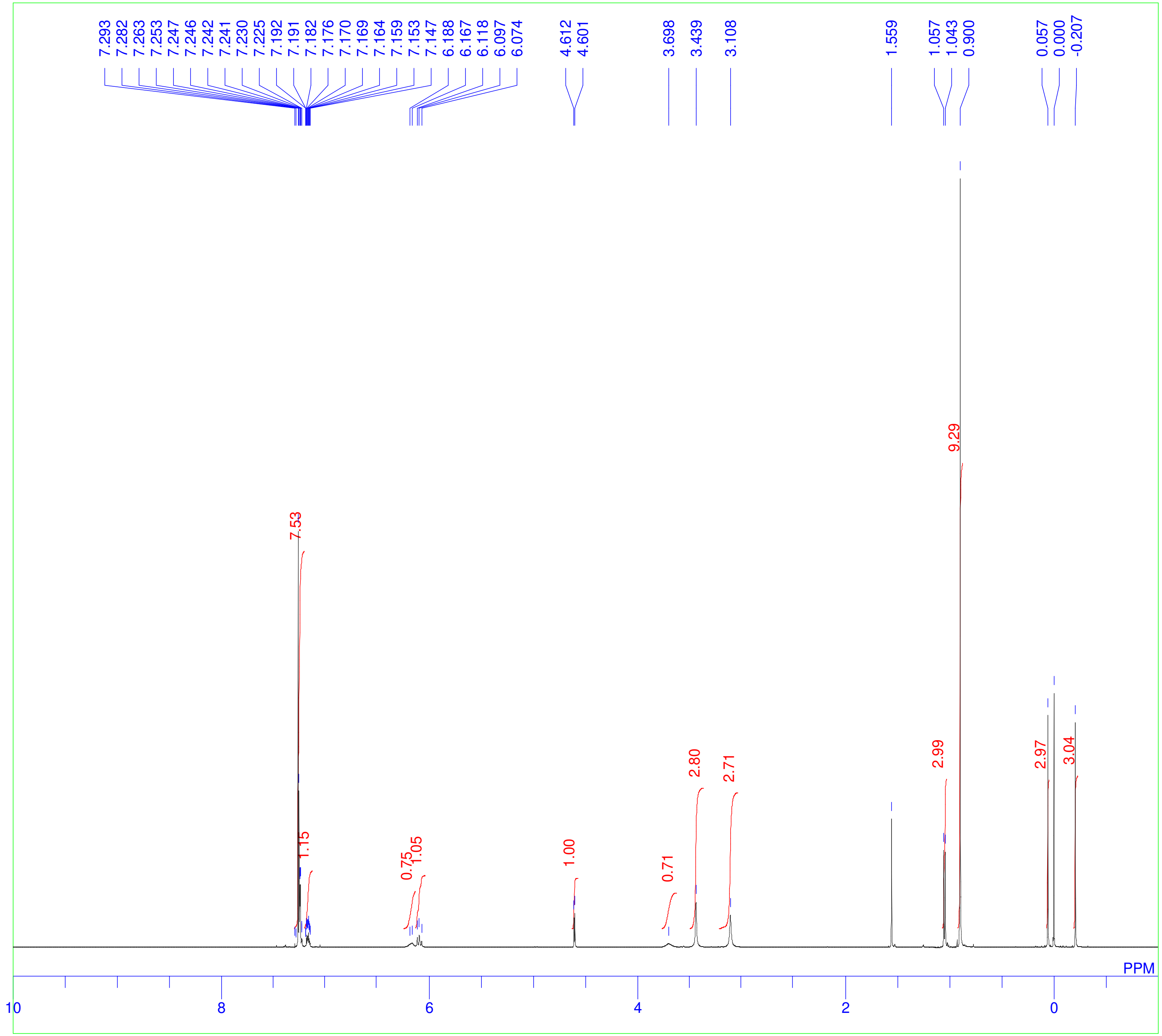

DFILE	(Z)-16q_1H.als
COMNT	
DATIM	2022-02-19 19:28:09
OBNUC	1H
EXMOD	proton.jxp
OBFRQ	500.16 MHz
OBSET	2.41 KHz
OBFIN	6.01 Hz
POINT	13107
FREQU	7507.51 Hz
SCANS	8
ACQTM	1.7459 sec
PD	5.0000 sec
PW1	3.84 usec
IRNUC	1H
CTEMP	19.2 c
SLVNT	CDCL3
EXREF	0.00 ppm
BF	0.30 Hz
RGAIN	44

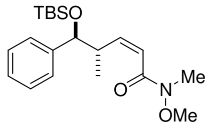

(*Z*)-16q

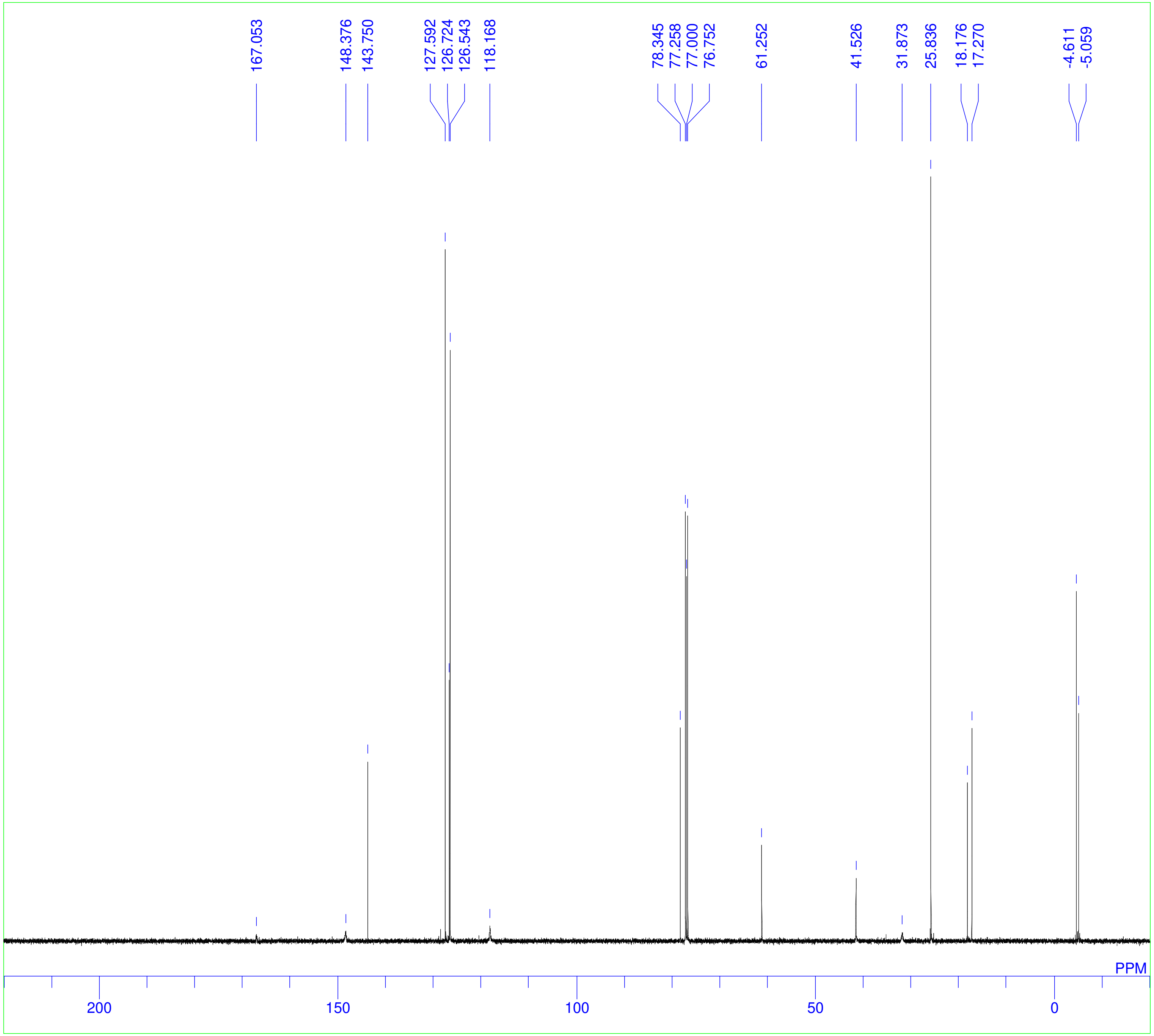
167.053
148.376
143.750
127.592
126.724
126.543
118.168
78.345
77.258
77.000
76.752
61.252
41.526
31.873
25.836
18.176
17.270
-4.611
-5.059
PPM
200
150
100
50
0
DFILE (Z)-16q_13C.als
COMNT
DATIM 2022-03-01 15:01:58
OBNUC 13C
EXMOD carbon.jxp
OBFRQ 125.77 MHz
OBSET 7.87 KHz
OBFIN 4.21 Hz
POINT 26214
FREQU 31446.54 Hz
SCANS 1024
ACQTM 0.8336 sec
PD 2.0000 sec
PW1 3.87 usec
IRNUC 1H
CTEMP 21.6 c
SLVNT CDCL3
EXREF 77.00 ppm
BF 0.30 Hz
RGAIN 26

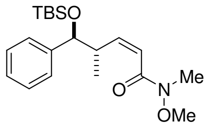
TBSO
O
N
Me
OMe

(*Z*)-16q

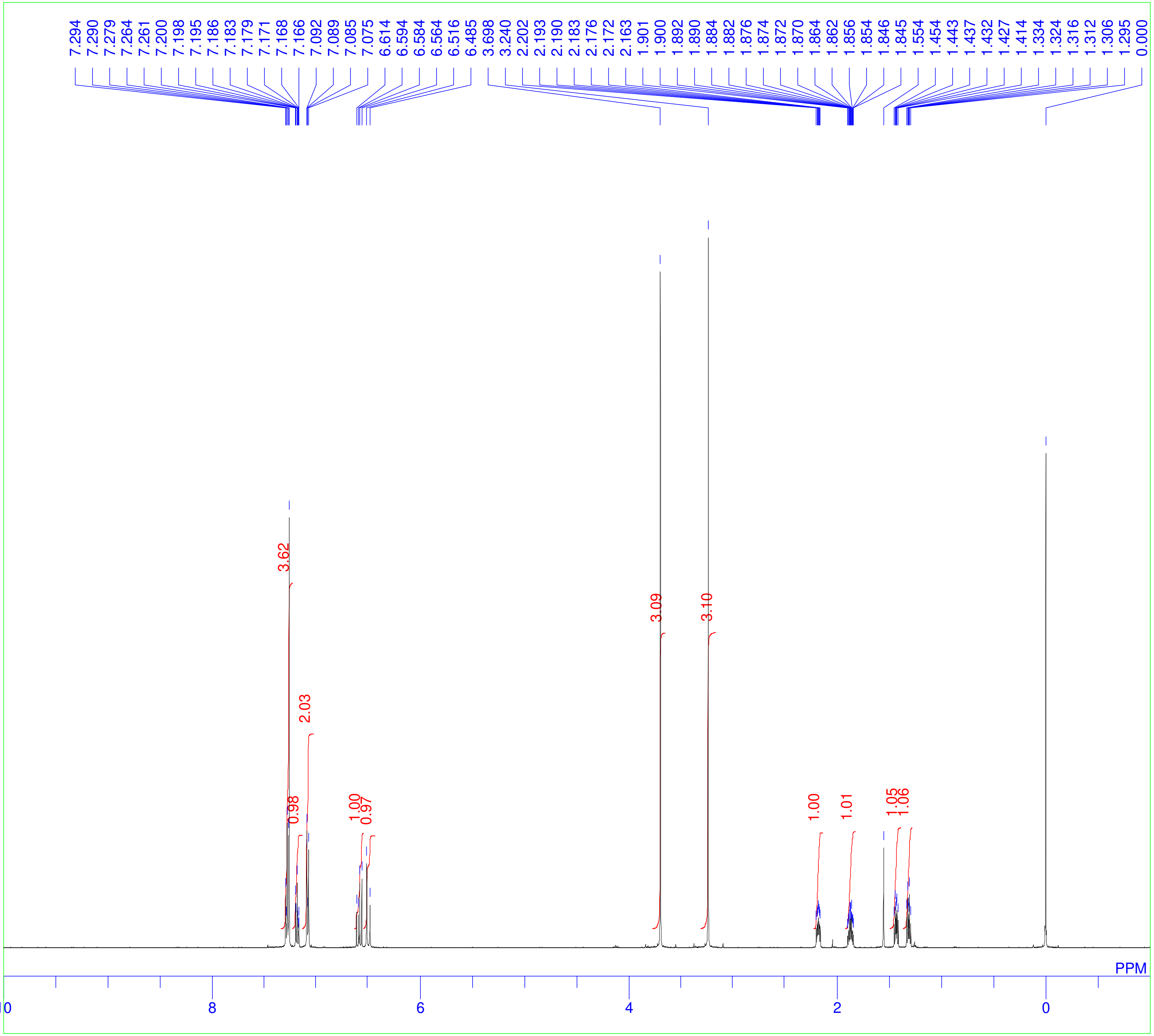

DFILE (E)-16r_1H.als
COMNT
DATIM 2023-04-24 13:27:50
OBNUC 1H
EXMOD proton.jxp
OBFRQ 500.16 MHz
OBSET 2.41 KHz
OBFIN 6.01 Hz
POINT 13107
FREQU 7507.51 Hz
SCANS 8
ACQTM 1.7459 sec
PD 5.0000 sec
PW1 3.84 usec
IRNUC 1H
CTEMP 23.9 c
SLVNT CDCL3
EXREF 0.00 ppm
BF 0.30 Hz
RGAIN 46

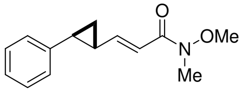

(*E*)-16r

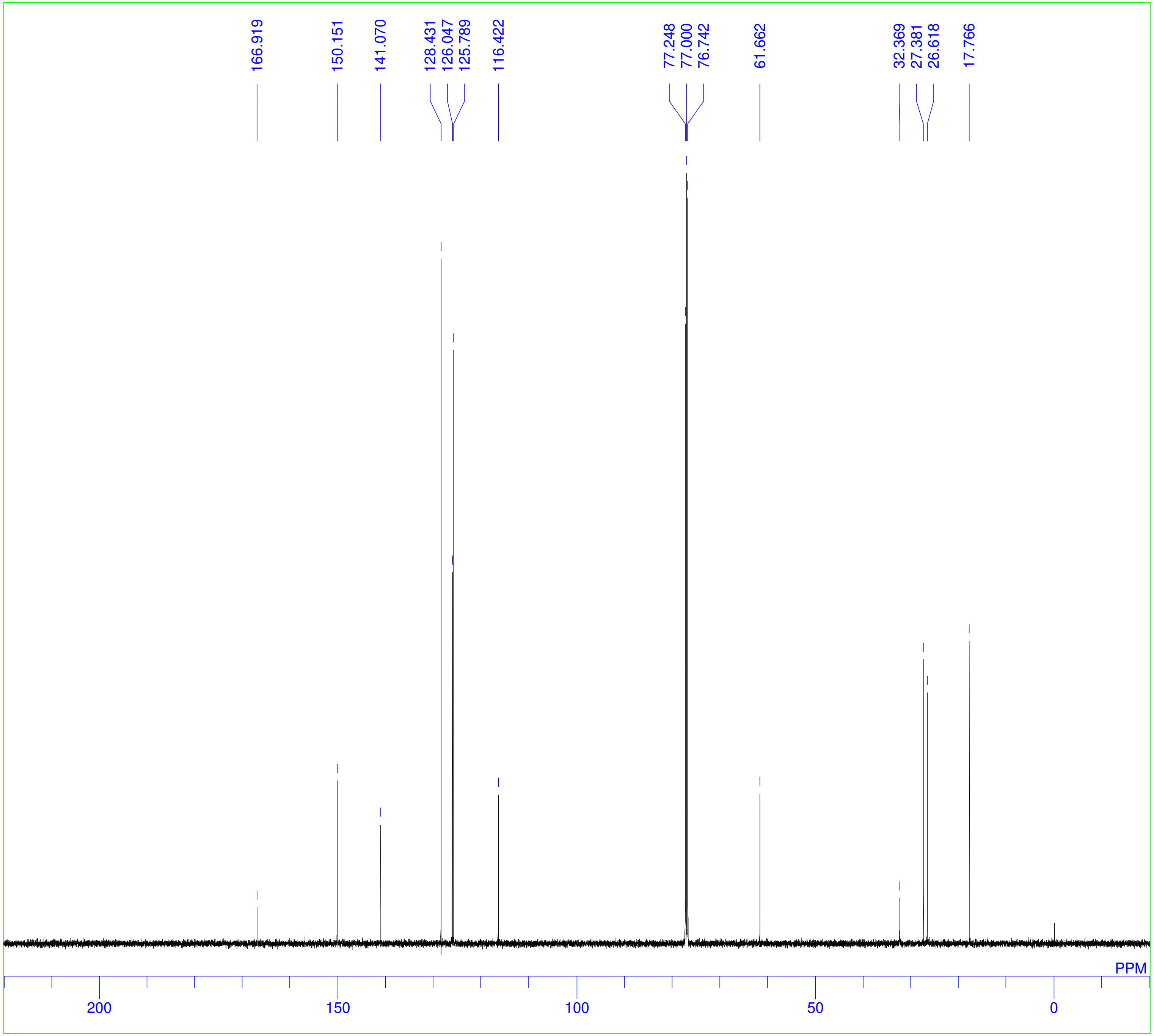

DFILE (E)-16r_13C.als
COMNT
DATIM 2023-04-24 22:12:16
OBNUC 13C
EXMOD carbon.jxp
OBFRQ 125.77 MHz
OBSET 7.87 KHz
OBFIN 4.21 Hz
POINT 26214
FREQU 31446.54 Hz
SCANS 2048
ACQTM 0.8336 sec
PD 2.0000 sec
PW1 3.87 usec
IRNUC 1H
CTEMP 23.9 c
SLVNT CDCL3
EXREF 77.00 ppm
BF 0.30 Hz
RGAIN 30

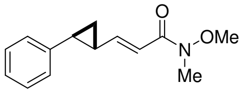

(*E*)-16r

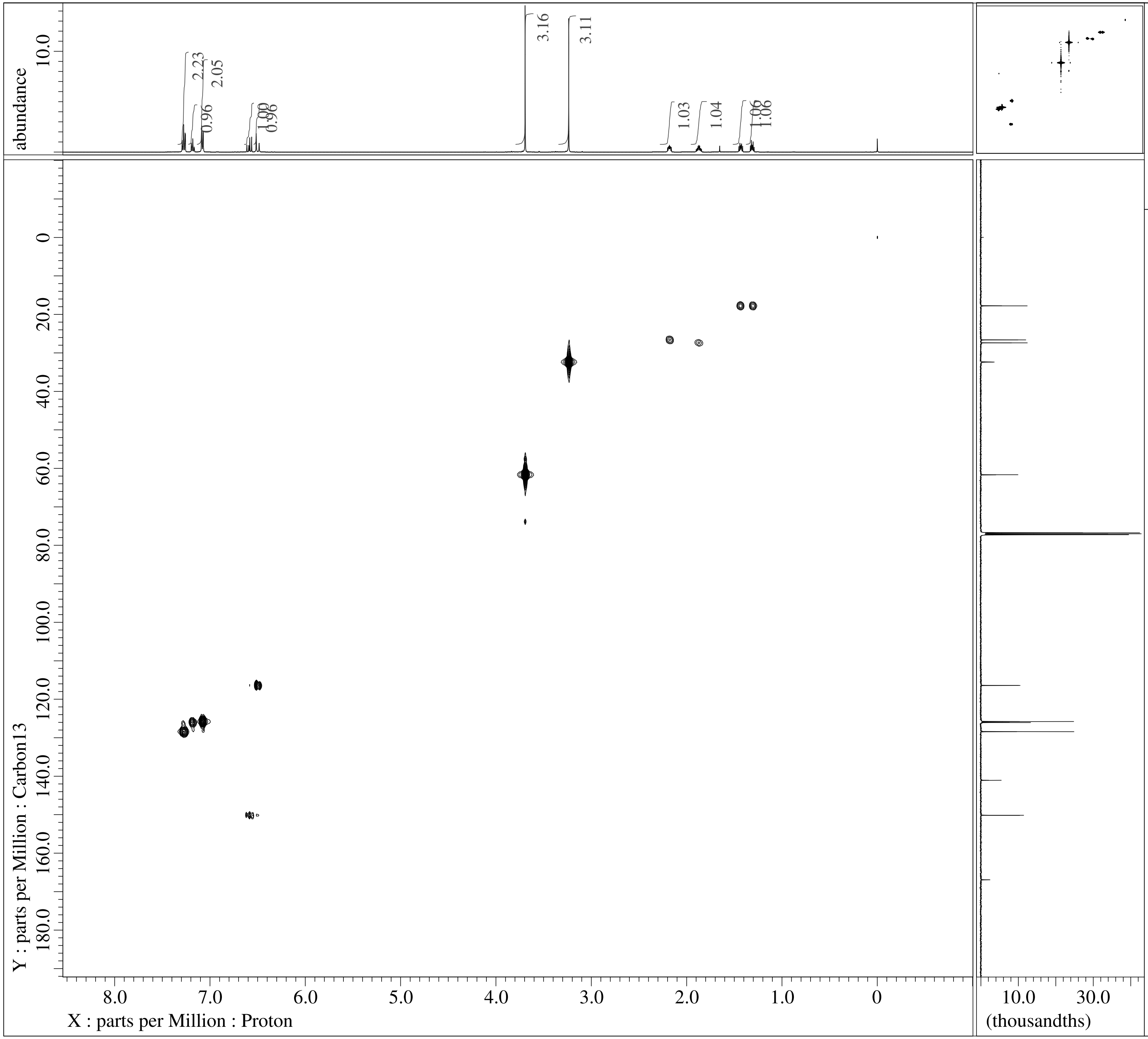

---- PROCESSING PARAMETERS ----
sinbell4(-60, 160)
zerofill(1)
fft(1, TRUE, TRUE)
ppm
[transpose]
sinbell4(-60, 160)
zerofill(2)
fft(1, TRUE, TRUE)
ppm
abs

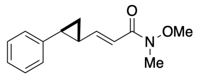

(*E*)-16r

Filename	= HT-1375-PTLC 3_13C_HMQC
Author	= delta
Experiment	= hmqc.jxp
Sample_Id	= HT-1375-PTLC 2_13C
Solvent	= CHLOROFORM-D
Creation_Time	= 25-APR-2023 04:10:49
Revision_Time	= 10-APR-2024 21:42:20
Current_Time	= 10-APR-2024 21:43:26
Comment	= gradient enhanced HMQC
Data_Format	= 2D REAL REAL
Dim_Size	= 819, 512
Dim_Title	= Proton Carbon13
Dim_Units	= [ppm] [ppm]
Dimensions	= X Y
Site	= JNM-ECA500II
Spectrometer	= DELTA2_NMR
Field_Strength	= 11.7473579[T] (500[MHz]
X_Acq_Duration	= 0.16941056[s]
X_Domain	= 1H
X_Freq	= 500.15991521[MHz]
X_Offset	= 3.70593703[ppm]
X_Points	= 1024
X_Prescans	= 4
X_Resolution	= 5.90281975[Hz]
X_Sweep	= 6.04448743[kHz]
X_Sweep_Clipped	= 4.83558994[kHz]
Y_Domain	= 13C
Y_Freq	= 125.76529768[MHz]
Y_Offset	= 83.55115782[ppm]
Y_Points	= 256
Y_Prescans	= 0
Y_Resolution	= 106.84491247[Hz]
Y_Sweep	= 27.35229759[kHz]
Tri_Domain	= Proton
Tri_Freq	= 500.15991521[MHz]
Tri_Offset	= 5.0[ppm]
Clipped	= FALSE
Scans	= 16
Total_Scans	= 4096
Relaxation_Delay	= 1.5[s]
Recvr_Gain	= 50
Temp_Get	= 23.6[dC]
X_Acq_Time	= 0.16941056[s]
X_Atn	= 3.2[dB]
X_Gamma	= 42576375
X_Pulse	= 7.68[us]

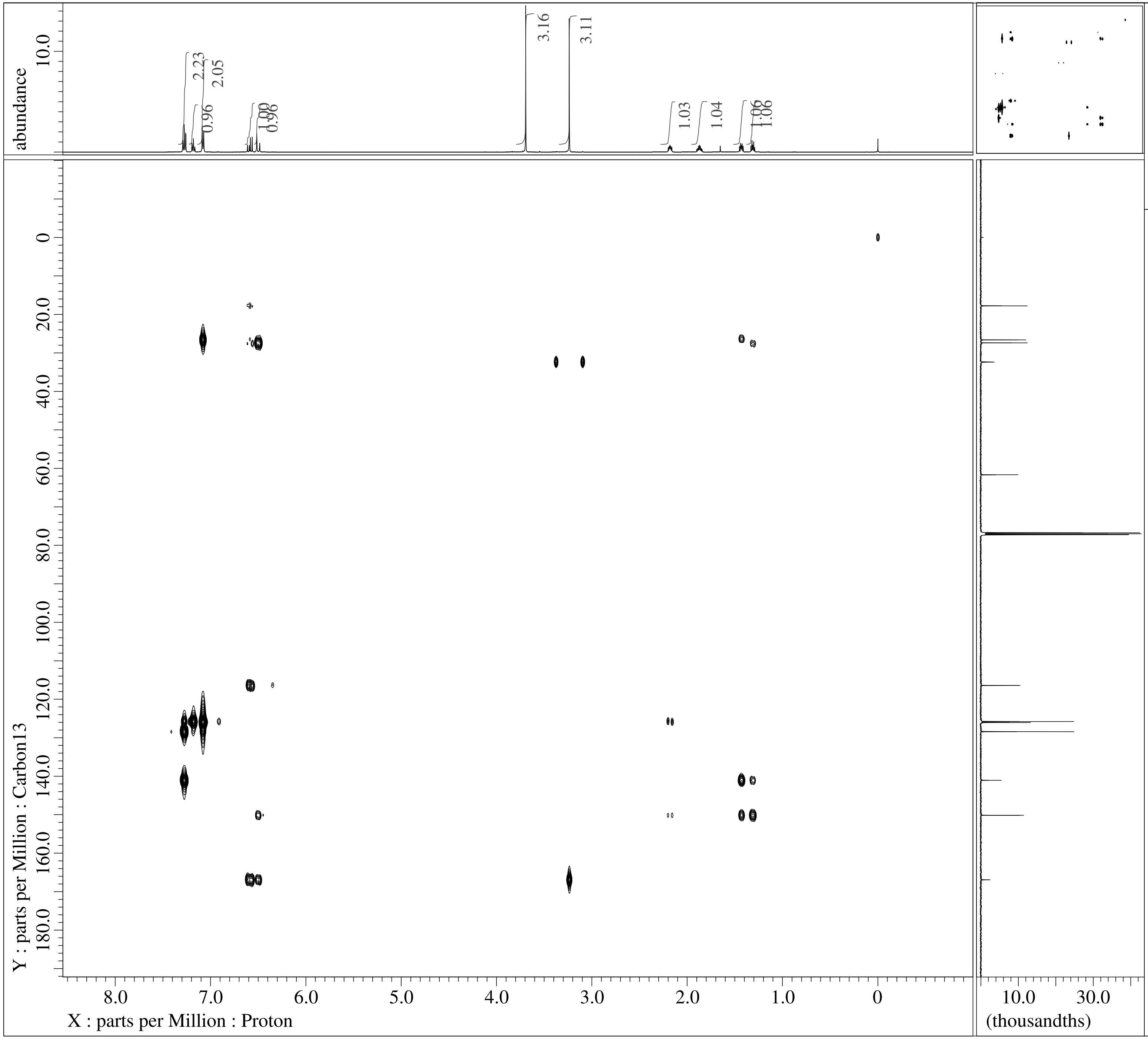


```
---- PROCESSING PARAMETERS ----
gauss( 5.0[Hz], 0.0[s] )
sinbell_auto
zerofill( 1 )
fft( 1, TRUE, TRUE )
ppm
[transpose]
sinbell4( -60, 160 )
trapezoid( 0[%], 5[%], 80[%], 100[%] )
zerofill( 2 )
fft( 1, TRUE, TRUE )
```

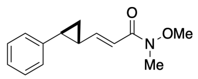

(*E*)-16r

```
Filename            = HT-1375-PTLC 3_13C_HMBC
Author              = delta
Experiment          = hmbc.jxp
Sample_Id           = HT-1375-PTLC 2_13C
Solvent             = CHLOROFORM-D
Creation_Time       = 24-APR-2023 23:49:31
Revision_Time       = 10-APR-2024 21:44:45
Current_Time        = 10-APR-2024 21:45:29

Comment             = gradient enhanced HMBC
Data_Format         = 2D REAL REAL
Dim_Size            = 1638, 512
Dim_Title           = Proton Carbon13
Dim_Units           = [ppm] [ppm]
Dimensions          = X Y
Site                = JNM-ECA500II
Spectrometer        = DELTA2_NMR

Field_Strength      = 11.7473579[T] (500[MHz]
X_Acq_Duration      = 0.33882112[s]
X_Domain            = 1H
X_Freq              = 500.15991521[MHz]
X_Offset            = 3.70593703[ppm]
X_Points            = 2048
X_Prescans          = 4
X_Resolution        = 2.95140988[Hz]
X_Sweep             = 6.04448743[kHz]
X_Sweep_Clipped     = 4.83558994[kHz]
Y_Domain            = 13C
Y_Freq              = 125.76529768[MHz]
Y_Offset            = 83.55115782[ppm]
Y_Points            = 256
Y_Prescans          = 0
Y_Resolution        = 106.84491247[Hz]
Y_Sweep             = 27.35229759[kHz]
Tri_Domain          = Proton
Tri_Freq            = 500.15991521[MHz]
Tri_Offset          = 5.0[ppm]
Clipped             = FALSE
Scans               = 32
Total_Scans         = 8192

Relaxation_Delay    = 1.5[s]
Recvr_Gain          = 50
Temp_Get            = 23.5[dC]
X_Acq_Time          = 0.33882112[s]
X_Atn               = 3.2[dB]
X_Gamma             = 42576375
X_Pulse             = 7.68[us]
```

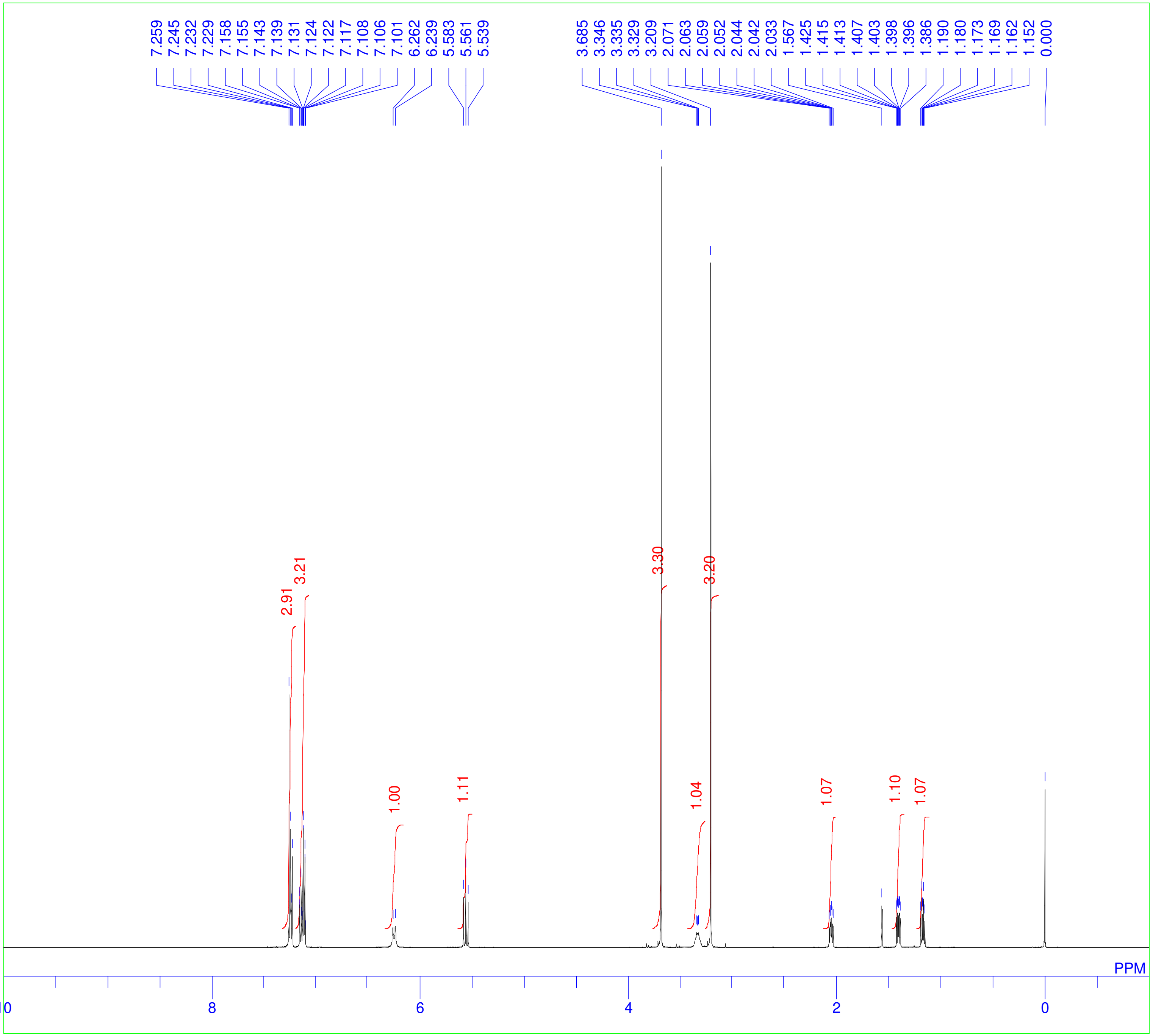

DFILE	(Z)-16r_1H.als
COMNT	
DATIM	2023-11-29 21:33:45
OBNUC	1H
EXMOD	proton.jxp
OBFRQ	500.16 MHz
OBSET	2.41 KHz
OBFIN	6.01 Hz
POINT	13107
FREQU	7507.51 Hz
SCANS	8
ACQTM	1.7459 sec
PD	5.0000 sec
PW1	3.80 usec
IRNUC	1H
CTEMP	23.6 c
SLVNT	CDCL3
EXREF	0.00 ppm
BF	0.30 Hz
RGAIN	40

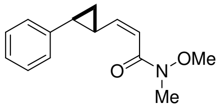

(*Z*)-16r

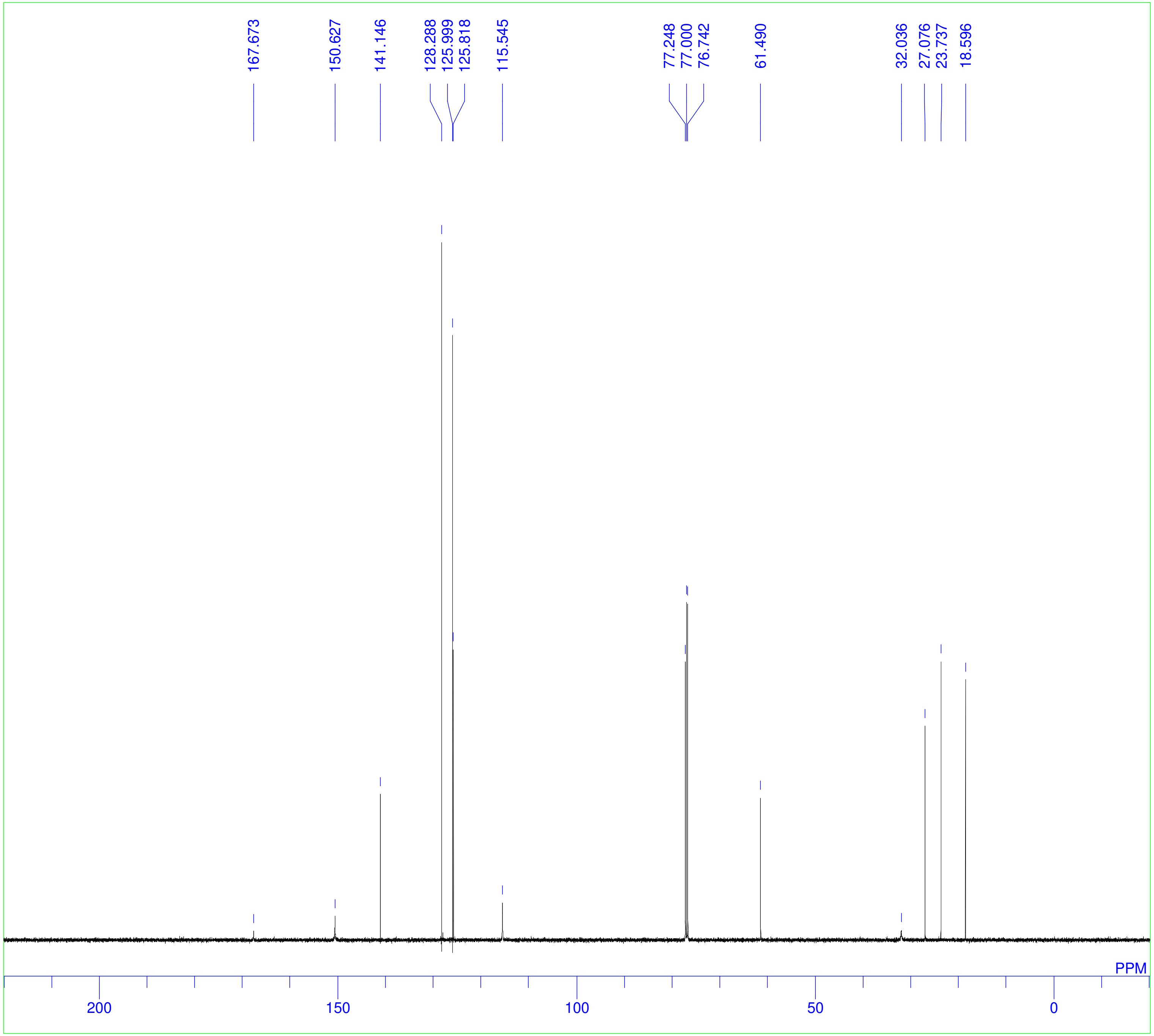

DFILE (Z)-16r_13C.als
COMNT
DATIM 2023-11-29 19:26:26
OBNUC 13C
EXMOD carbon.jxp
OBFRQ 125.77 MHz
OBSET 7.87 KHz
OBFIN 4.21 Hz
POINT 26214
FREQU 31446.54 Hz
SCANS 1024
ACQTM 0.8336 sec
PD 2.0000 sec
PW1 4.30 usec
IRNUC 1H
CTEMP 23.9 c
SLVNT CDCL3
EXREF 77.00 ppm
BF 0.30 Hz
RGAIN 30

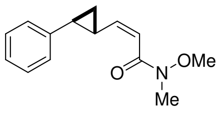

(*Z*)-16r

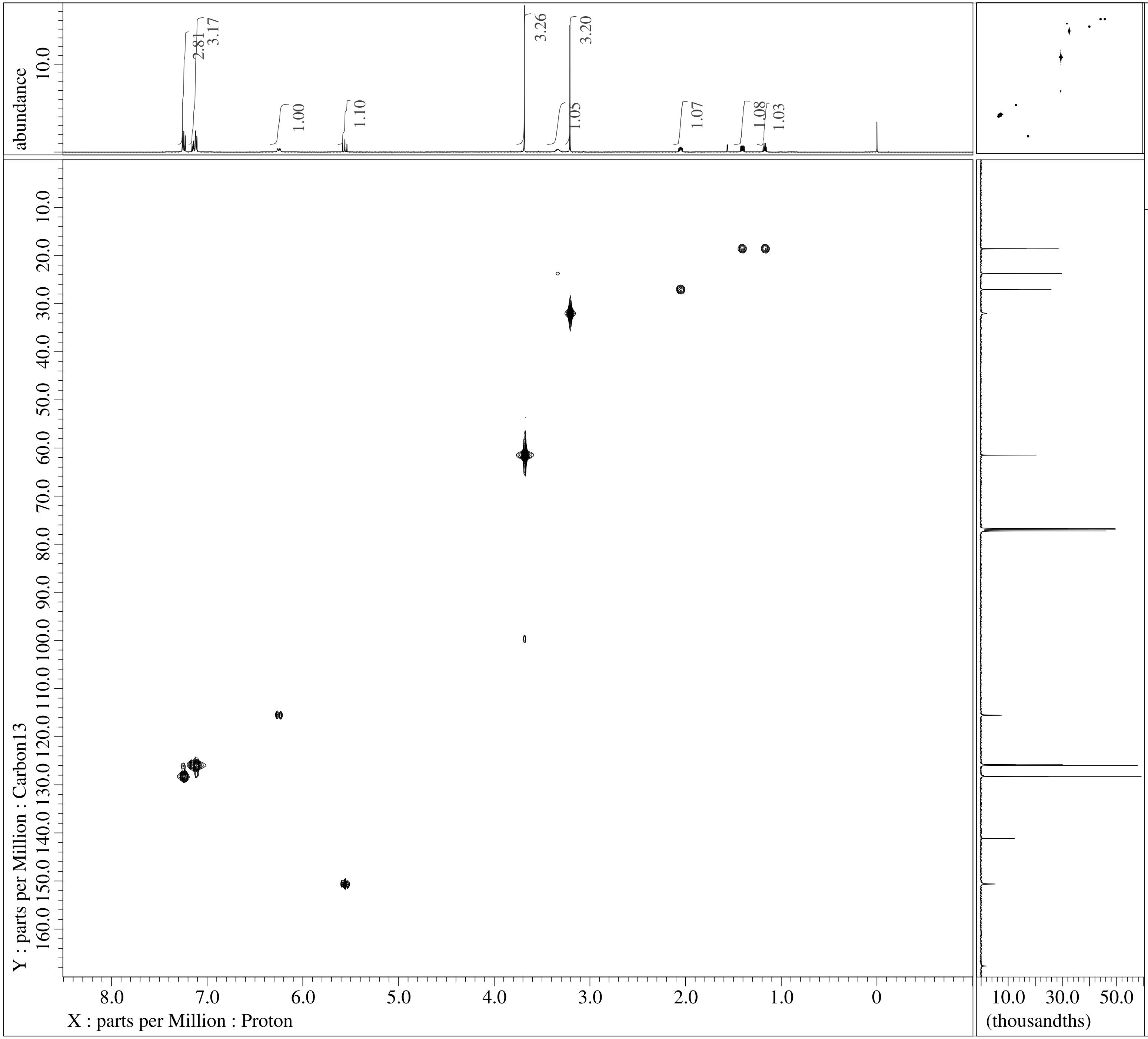


```
---- PROCESSING PARAMETERS ----
sinbell4( -60, 160 )
zerofill( 1 )
fft( 1, TRUE, TRUE )
ppm
[transpose]
sinbell4( -60, 160 )
zerofill( 2 )
fft( 1, TRUE, TRUE )
ppm
abs
```

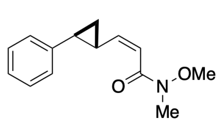

(*E*)-16r

```
Filename            = HT-1620-PTLC 1_HMQC_HMQ
Author              = delta
Experiment          = hmqc.jxp
Sample_Id           = HT-1620-PTLC 1_HMQC
Solvent             = CHLOROFORM-D
Creation_Time       = 30-NOV-2023 08:01:57
Revision_Time       = 10-APR-2024 21:47:54
Current_Time        = 10-APR-2024 21:48:35

Comment             = gradient enhanced HMQC
Data_Format         = 2D REAL REAL
Dim_Size            = 819, 512
Dim_Title           = Proton Carbon13
Dim_Units           = [ppm] [ppm]
Dimensions          = X Y
Site                = JNM-ECA500II
Spectrometer        = DELTA2_NMR

Field_Strength      = 11.7473579[T] (500[MHz]
X_Acq_Duration      = 0.17006592[s]
X_Domain            = 1H
X_Freq              = 500.15991521[MHz]
X_Offset            = 3.68761402[ppm]
X_Points            = 1024
X_Prescans          = 4
X_Resolution        = 5.88007286[Hz]
X_Sweep             = 6.02119461[kHz]
X_Sweep_Clipped     = 4.81695568[kHz]
Y_Domain            = 13C
Y_Freq              = 125.76529768[MHz]
Y_Offset            = 85[ppm]
Y_Points            = 256
Y_Prescans          = 0
Y_Resolution        = 83.60980308[Hz]
Y_Sweep             = 21.40410959[kHz]
Tri_Domain          = Proton
Tri_Freq            = 500.15991521[MHz]
Tri_Offset          = 5.0[ppm]
Clipped             = FALSE
Scans               = 8
Total_Scans         = 2048

Relaxation_Delay    = 1.5[s]
Recvr_Gain          = 50
Temp_Get            = 23.7[dC]
X_Acq_Time          = 0.17006592[s]
X_Atn               = 3.2[dB]
X_Gamma             = 42576375
X_Pulse             = 7.59[us]
```

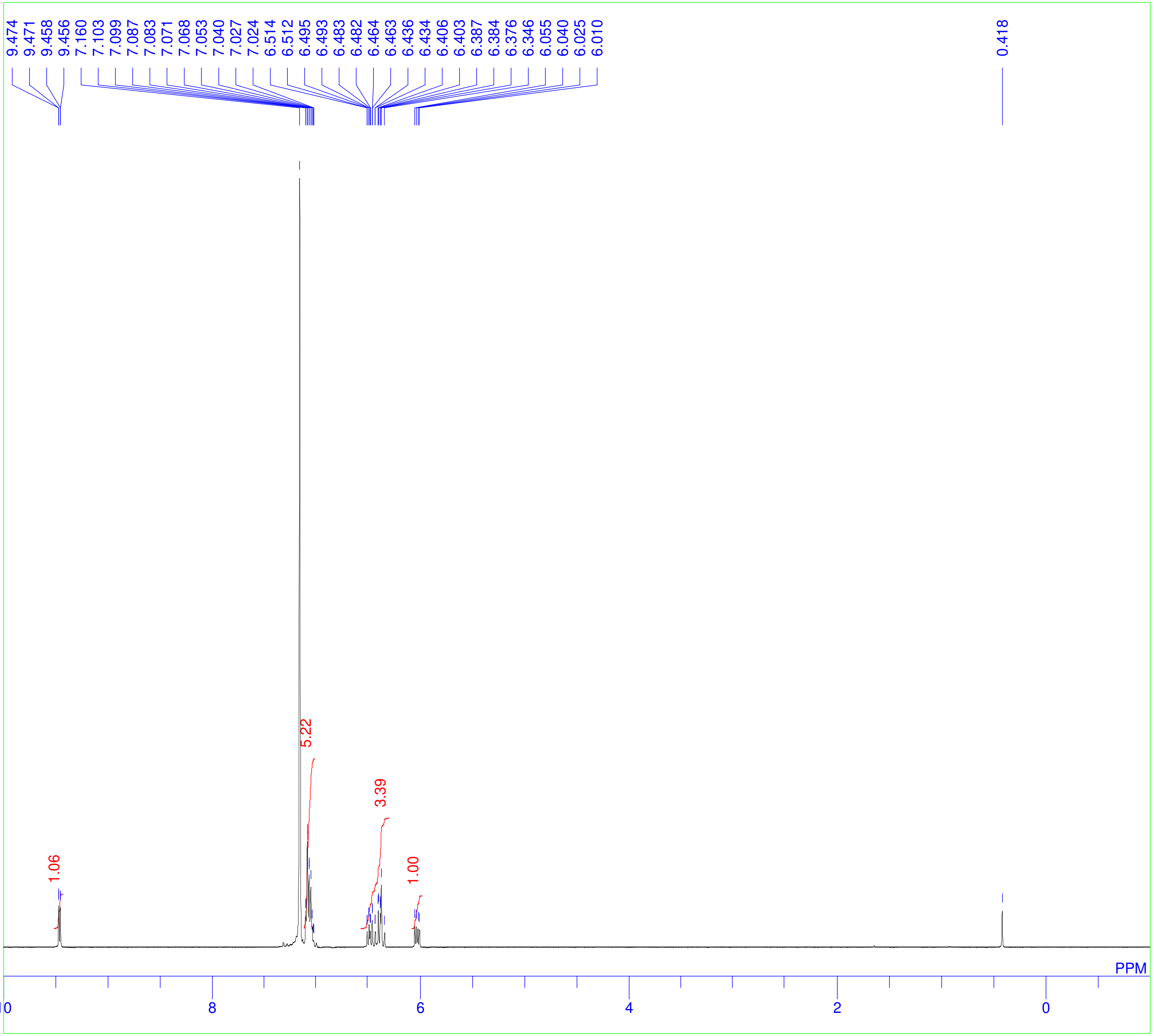

DFILE 17f_1H.als
COMNT
DATIM 2022-01-05 20:50:42
OBNUC 1H
EXMOD proton.jxp
OBFRQ 500.16 MHz
OBSET 2.41 KHz
OBFIN 6.01 Hz
POINT 13107
FREQU 7507.51 Hz
SCANS 8
ACQTM 1.7459 sec
PD 5.0000 sec
PW1 3.84 usec
IRNUC 1H
CTEMP 18.6 c
SLVNT C6D6
EXREF 7.16 ppm
BF 0.30 Hz
RGAIN 42

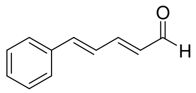

17f

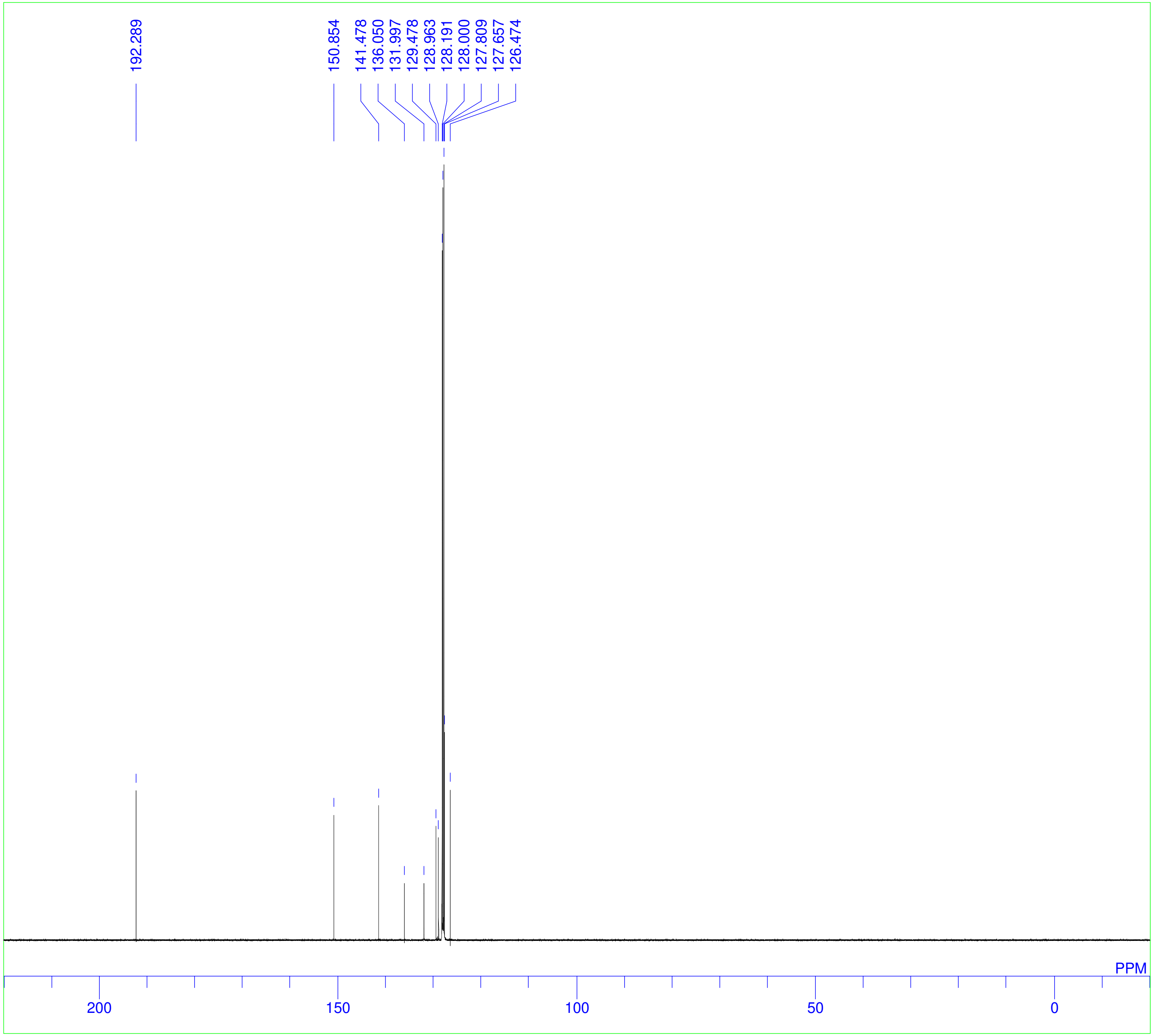

DFILE 17f_13C.als
COMNT
DATIM 2023-01-05 09:38:33
OBNUC 13C
EXMOD carbon.jxp
OBFRQ 125.77 MHz
OBSET 7.87 KHz
OBFIN 4.21 Hz
POINT 26214
FREQU 31446.54 Hz
SCANS 1024
ACQTM 0.8336 sec
PD 2.0000 sec
PW1 3.87 usec
IRNUC 1H
CTEMP 20.5 c
SLVNT C6D6
EXREF 128.00 ppm
BF 0.30 Hz
RGAIN 30

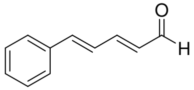

17f

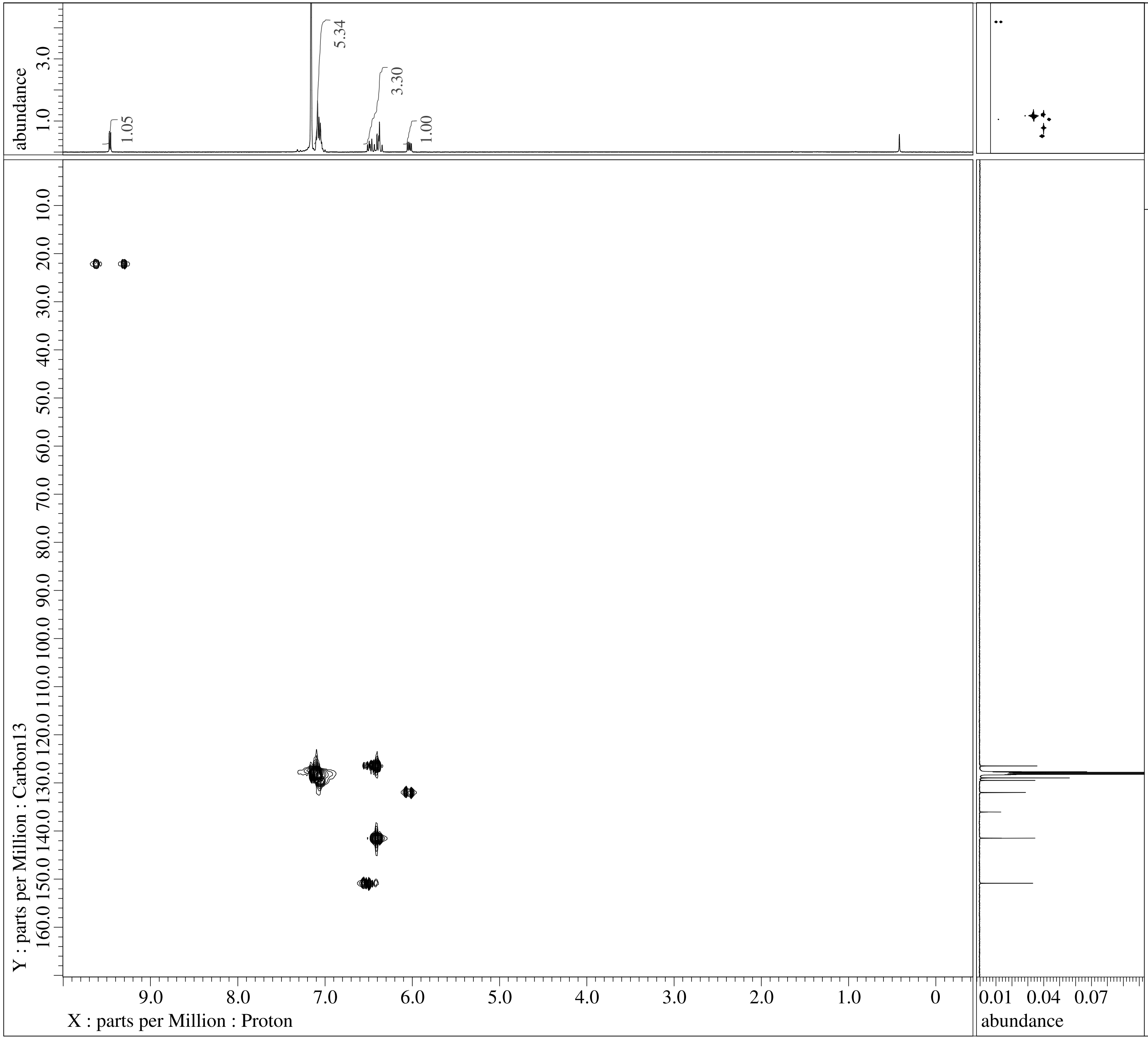


```
---- PROCESSING PARAMETERS ----
sinbell4( -60, 160 )
zerofill( 1 )
fft( 1, TRUE, TRUE )
ppm
[transpose]
sinbell4( -60, 160 )
zerofill( 2 )
fft( 1, TRUE, TRUE )
ppm
abs
```

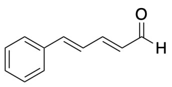

17f

```
Filename            = HT-775-TM-C6D6_HMQC,HMB
Author              = delta
Experiment          = hmqc.jxp
Sample_Id           = HT-775-TM-C6D6_HMQC,HMB
Solvent             = BENZENE-D6
Creation_Time       =  5-JAN-2024 17:32:08
Revision_Time       = 10-APR-2024 21:54:47
Current_Time        = 10-APR-2024 21:57:17

Comment             = gradient enhanced HMQC
Data_Format         = 2D REAL REAL
Dim_Size            = 819, 512
Dim_Title           = Proton Carbon13
Dim_Units           = [ppm] [ppm]
Dimensions          = X Y
Site                = JNM-ECA500II
Spectrometer        = DELTA2_NMR

Field_Strength      = 11.7473579[T] (500[MHz]
X_Acq_Duration      = 0.1437696[s]
X_Domain            = 1H
X_Freq              = 500.15991521[MHz]
X_Offset            = 5.19639982[ppm]
X_Points            = 1024
X_Prescans          = 4
X_Resolution        = 6.95557336[Hz]
X_Sweep             = 7.12250712[kHz]
X_Sweep_Clipped     = 5.6980057[kHz]
Y_Domain            = 13C
Y_Freq              = 125.76529768[MHz]
Y_Offset            = 85[ppm]
Y_Points            = 256
Y_Prescans          = 0
Y_Resolution        = 83.60980308[Hz]
Y_Sweep             = 21.40410959[kHz]
Tri_Domain          = Proton
Tri_Freq            = 500.15991521[MHz]
Tri_Offset          = 5.0[ppm]
Clipped             = FALSE
Scans               = 8
Total_Scans         = 2048

Relaxation_Delay    = 1.5[s]
Recvr_Gain          = 50
Temp_Get            = 22.9[dC]
X_Acq_Time          = 0.1437696[s]
X_Atn               = 3.2[dB]
X_Gamma             = 42576375
X_Pulse             = 7.59[us]
```

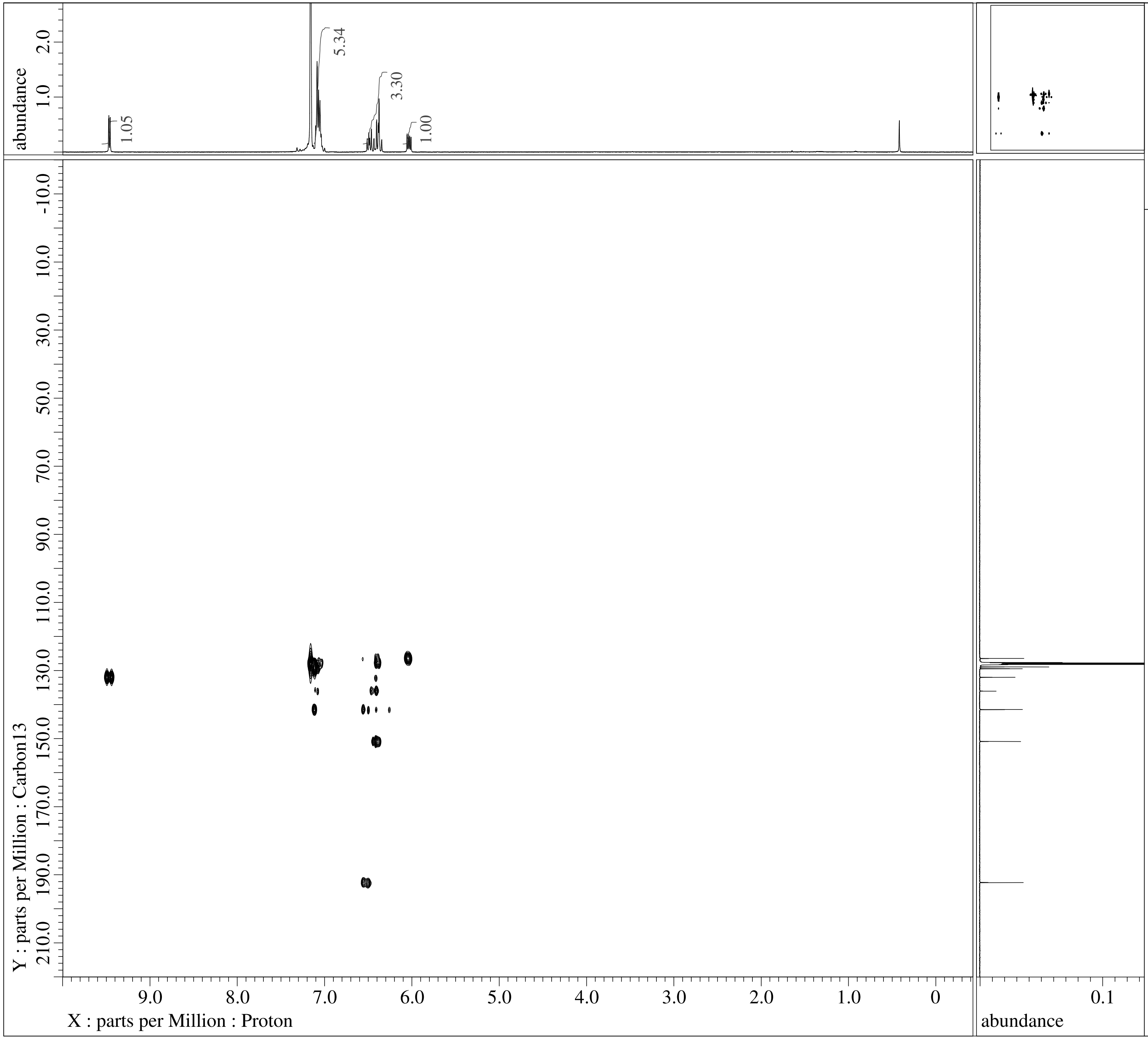


```
---- PROCESSING PARAMETERS ----
gauss( 5.0[Hz], 0.0[s] )
sinbell_auto
zerofill( 1 )
fft( 1, TRUE, TRUE )
ppm
[transpose]
sinbell4( -60, 160 )
trapezoid( 0[%], 5[%], 80[%], 100[%] )
zerofill( 2 )
fft( 1, TRUE, TRUE )
```

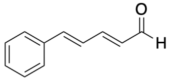

17f

```
Filename            = HT-775-TM-C6D6_HMQC,HMB
Author              = delta
Experiment          = hmbc.jxp
Sample_Id           = HT-775-TM-C6D6_HMQC,HMB
Solvent             = BENZENE-D6
Creation_Time       =  5-JAN-2024 18:29:18
Revision_Time       = 10-APR-2024 22:01:21
Current_Time        = 10-APR-2024 22:02:36

Comment             = gradient enhanced HMBC
Data_Format         = 2D REAL REAL
Dim_Size            = 1638, 512
Dim_Title           = Proton Carbon13
Dim_Units           = [ppm] [ppm]
Dimensions          = X Y
Site                = JNM-ECA500II
Spectrometer        = DELTA2_NMR

Field_Strength      = 11.7473579[T] (500[MHz]
X_Acq_Duration      = 0.2875392[s]
X_Domain            = 1H
X_Freq              = 500.15991521[MHz]
X_Offset            = 5.19639982[ppm]
X_Points            = 2048
X_Prescans          = 4
X_Resolution        = 3.47778668[Hz]
X_Sweep             = 7.12250712[kHz]
X_Sweep_Clipped     = 5.6980057[kHz]
Y_Domain            = 13C
Y_Freq              = 125.76529768[MHz]
Y_Offset            = 100[ppm]
Y_Points            = 256
Y_Prescans          = 0
Y_Resolution        = 122.99275819[Hz]
Y_Sweep             = 31.4861461[kHz]
Tri_Domain          = Proton
Tri_Freq            = 500.15991521[MHz]
Tri_Offset          = 5.0[ppm]
Clipped             = FALSE
Scans               = 8
Total_Scans         = 2048

Relaxation_Delay    = 1.5[s]
Recvr_Gain          = 50
Temp_Get            = 22.7[dC]
X_Acq_Time          = 0.2875392[s]
X_Atn               = 3.2[dB]
X_Gamma             = 42576375
X_Pulse             = 7.59[us]
```

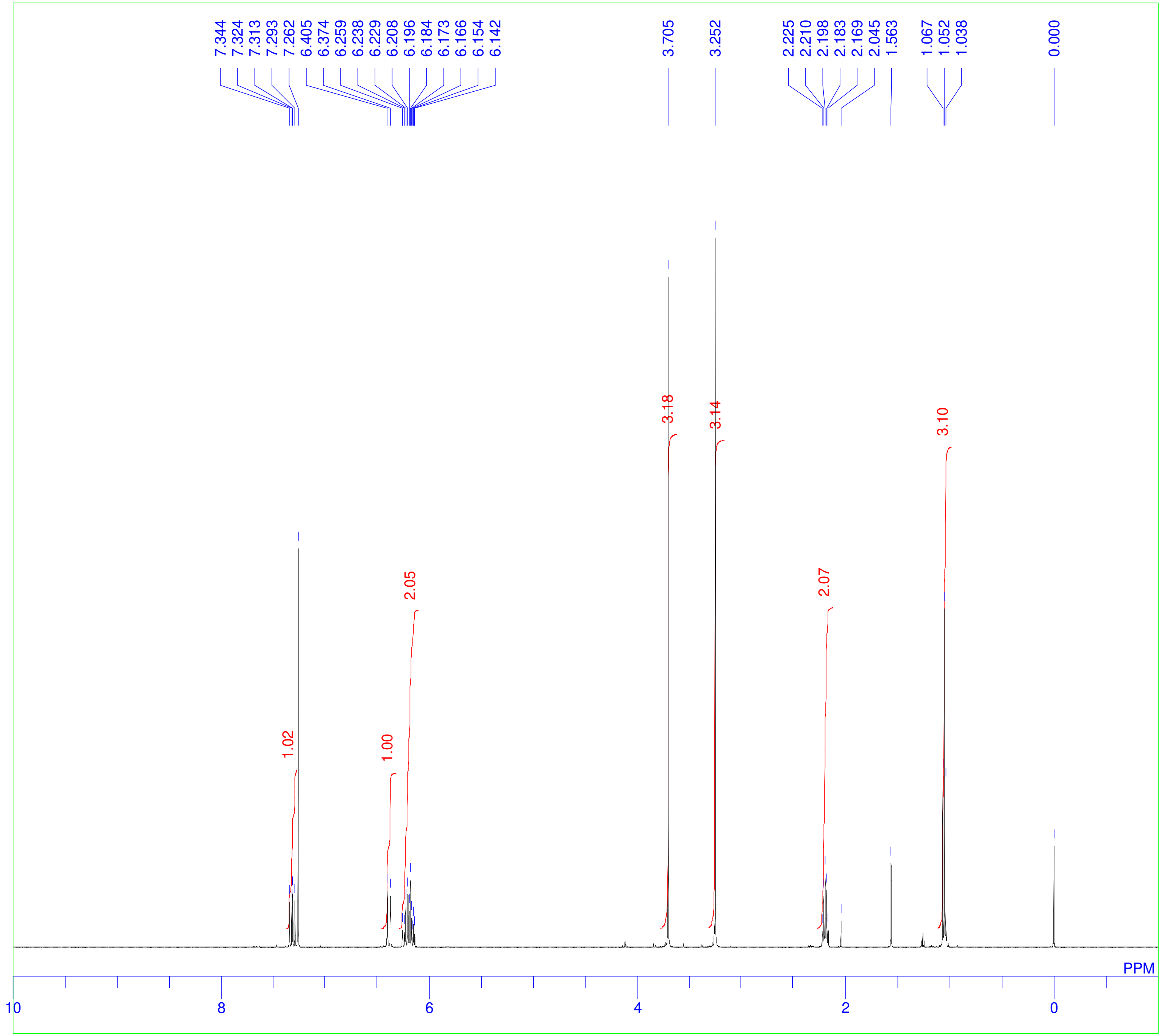

DFILE	(E)-18a_1H.als
COMNT	
DATIM	2023-04-13 13:34:42
OBNUC	1H
EXMOD	proton.jxp
OBFRQ	500.16 MHz
OBSET	2.41 KHz
OBFIN	6.01 Hz
POINT	13107
FREQU	7507.51 Hz
SCANS	8
ACQTM	1.7459 sec
PD	5.0000 sec
PW1	3.84 usec
IRNUC	1H
CTEMP	23.8 c
SLVNT	CDCL3
EXREF	0.00 ppm
BF	0.30 Hz
RGAIN	48

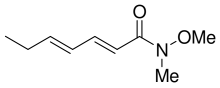

(*E*)-18a

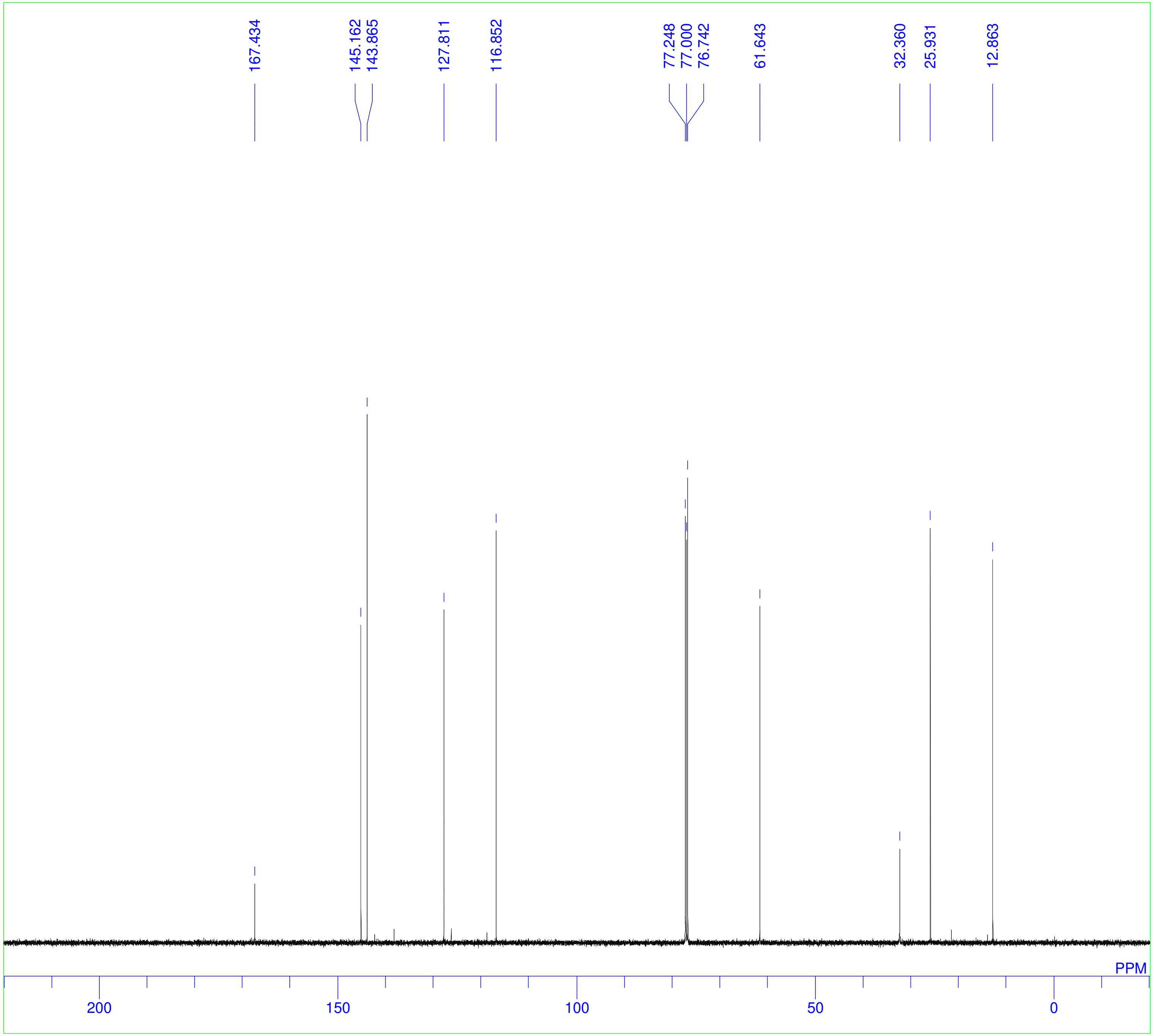

DFILE (E)-18a_13C.als
COMNT
DATIM 2023-04-17 14:04:53
OBNUC 13C
EXMOD carbon.jxp
OBFRQ 125.77 MHz
OBSET 7.87 KHz
OBFIN 4.21 Hz
POINT 26214
FREQU 31446.54 Hz
SCANS 1024
ACQTM 0.8336 sec
PD 2.0000 sec
PW1 3.87 usec
IRNUC 1H
CTEMP 24.1 c
SLVNT CDCL3
EXREF 77.00 ppm
BF 0.30 Hz
RGAIN 30

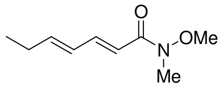

(*E*)-18a

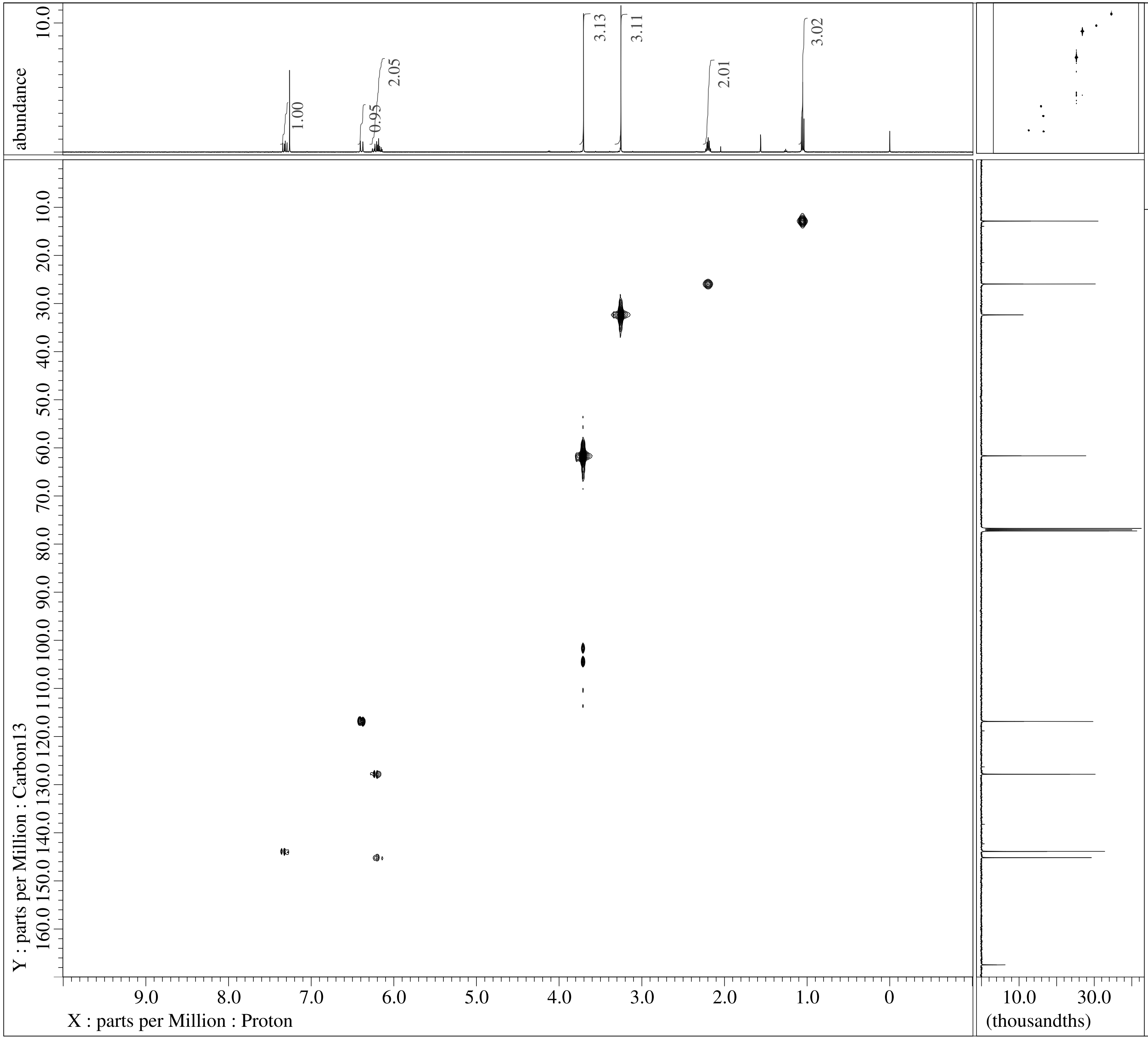


```
---- PROCESSING PARAMETERS ----
sinbell4( -60, 160 )
zerofill( 1 )
fft( 1, TRUE, TRUE )
ppm
[transpose]
sinbell4( -60, 160 )
zerofill( 2 )
fft( 1, TRUE, TRUE )
ppm
abs
```

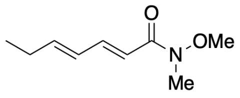

(*E*)-18a

```
Filename            = HT-1350-PTLC 2_HMQC_HMQ
Author              = delta
Experiment          = hmqc.jxp
Sample_Id           = HT-1350-PTLC 2_HMQC
Solvent             = CHLOROFORM-D
Creation_Time       =  4-MAY-2023 15:01:44
Revision_Time       = 10-APR-2024 22:07:13
Current_Time        = 10-APR-2024 22:07:56

Comment             = gradient enhanced HMQC
Data_Format         = 2D REAL REAL
Dim_Size            = 819, 512
Dim_Title           = Proton Carbon13
Dim_Units           = [ppm] [ppm]
Dimensions          = X Y
Site                = JNM-ECA500II
Spectrometer        = DELTA2_NMR

Field_Strength      = 11.7473579[T] (500[MHz]
X_Acq_Duration      = 0.12836864[s]
X_Domain            = 1H
X_Freq              = 500.15991521[MHz]
X_Offset            = 4.88376337[ppm]
X_Points            = 1024
X_Prescans          = 4
X_Resolution        = 7.79006461[Hz]
X_Sweep             = 7.97702616[kHz]
X_Sweep_Clipped     = 6.38162093[kHz]
Y_Domain            = 13C
Y_Freq              = 125.76529768[MHz]
Y_Offset            = 85[ppm]
Y_Points            = 256
Y_Prescans          = 0
Y_Resolution        = 83.60980308[Hz]
Y_Sweep             = 21.40410959[kHz]
Tri_Domain          = Proton
Tri_Freq            = 500.15991521[MHz]
Tri_Offset          = 5.0[ppm]
Clipped             = FALSE
Scans               = 8
Total_Scans         = 2048

Relaxation_Delay    = 1.5[s]
Recvr_Gain          = 50
Temp_Get            = 24[dC]
X_Acq_Time          = 0.12836864[s]
X_Atn               = 3.2[dB]
X_Gamma             = 42576375
X_Pulse             = 7.68[us]
```

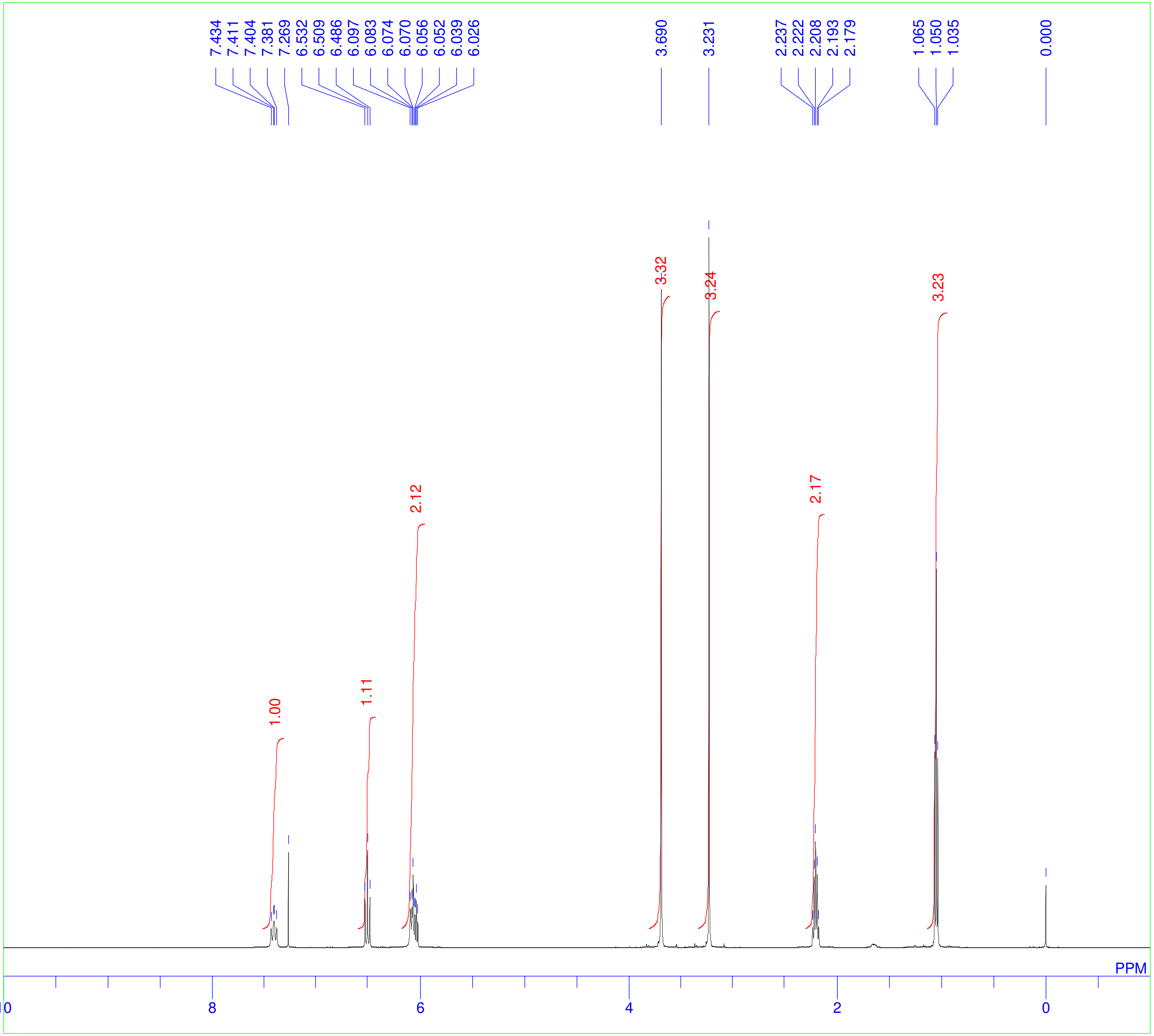

DFILE (Z)-18a_1H.als
COMNT
DATIM 2023-11-08 20:26:33
OBNUC 1H
EXMOD proton.jxp
OBFRQ 500.16 MHz
OBSET 2.41 KHz
OBFIN 6.01 Hz
POINT 13107
FREQU 7507.51 Hz
SCANS 8
ACQTM 1.7459 sec
PD 5.0000 sec
PW1 3.80 usec
IRNUC 1H
CTEMP 24.0 c
SLVNT CDCL3
EXREF 0.00 ppm
BF 0.30 Hz
RGAIN 32

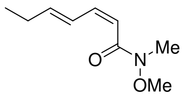

(*Z*)-18a

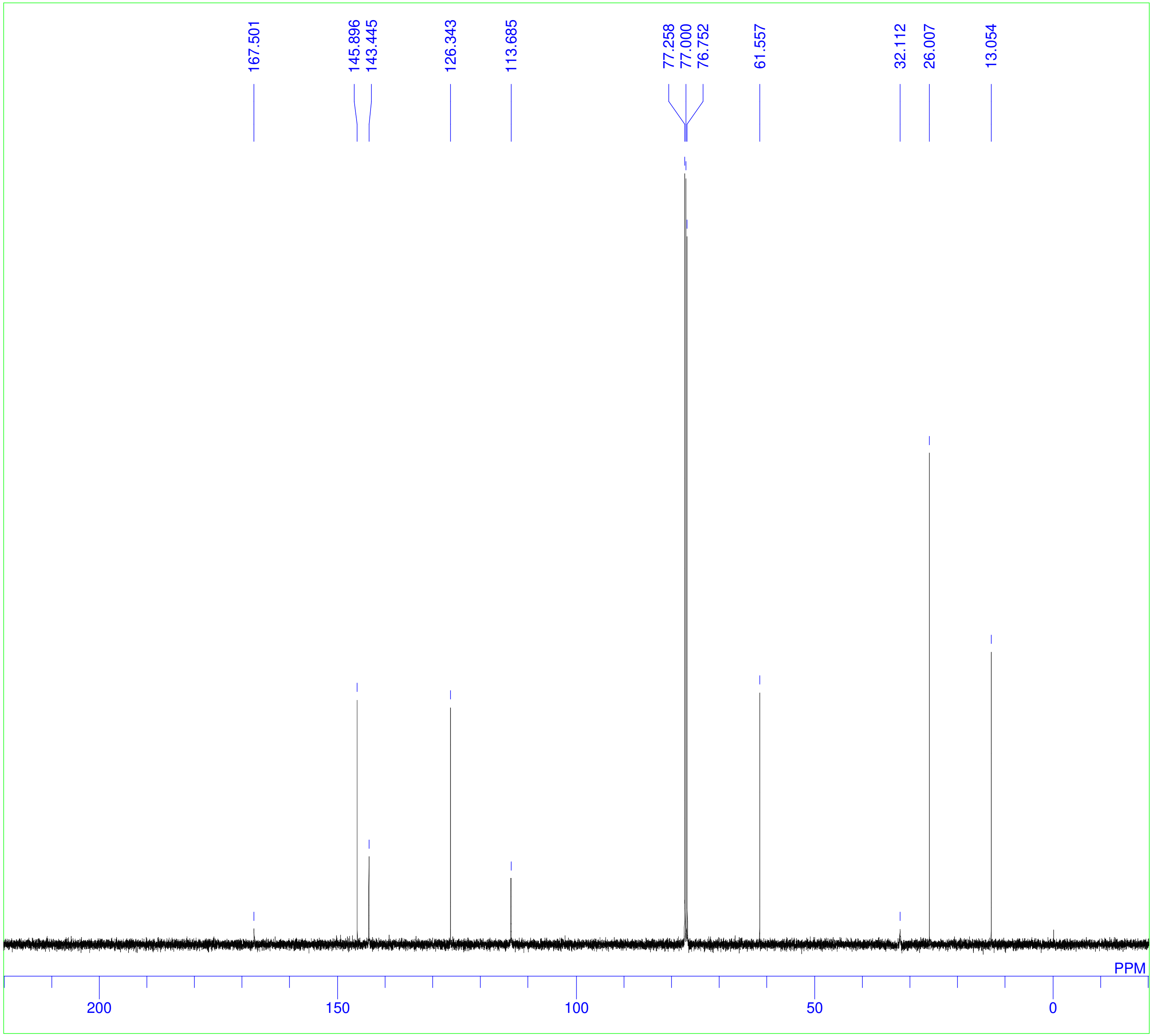

DFILE	(Z)-18a_13C.als
COMNT	
DATIM	2023-11-08 20:28:11
OBNUC	13C
EXMOD	carbon.jxp
OBFRQ	125.77 MHz
OBSET	7.87 KHz
OBFIN	4.21 Hz
POINT	26214
FREQU	31446.54 Hz
SCANS	1024
ACQTM	0.8336 sec
PD	2.0000 sec
PW1	4.30 usec
IRNUC	1H
CTEMP	24.3 c
SLVNT	CDCL3
EXREF	77.00 ppm
BF	0.30 Hz
RGAIN	30

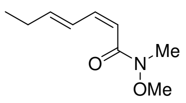

(*Z*)-18a

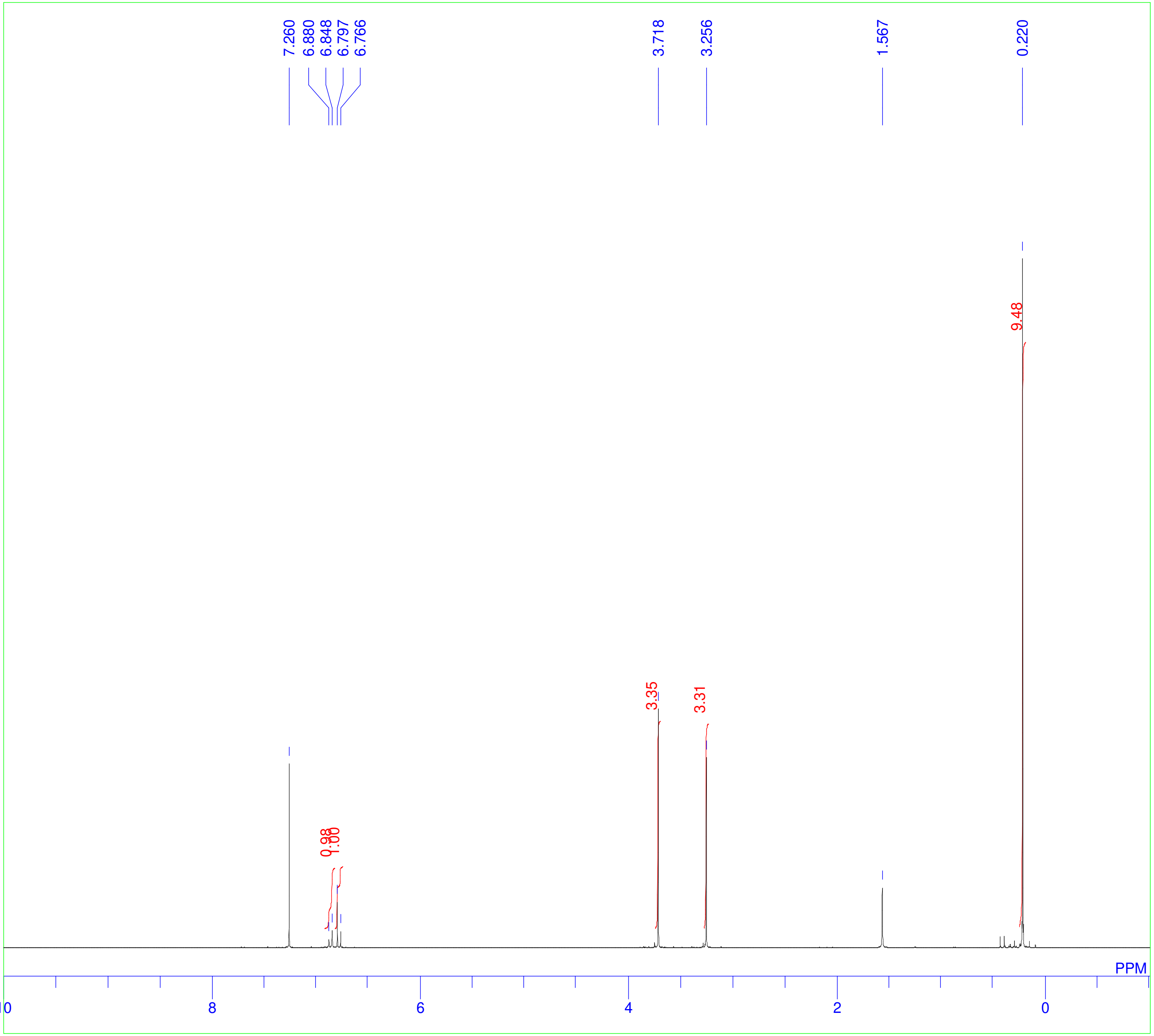

DFILE (E)-18c_1H.als
COMNT
DATIM 2022-01-12 16:06:05
OBNUC 1H
EXMOD proton.jxp
OBFRQ 500.16 MHz
OBSET 2.41 KHz
OBFIN 6.01 Hz
POINT 13107
FREQU 7507.51 Hz
SCANS 8
ACQTM 1.7459 sec
PD 5.0000 sec
PW1 3.84 usec
IRNUC 1H
CTEMP 19.5 c
SLVNT CDCL3
EXREF 7.26 ppm
BF 0.30 Hz
RGAIN 44

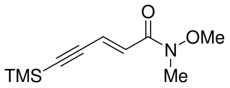

(*E*)-18c

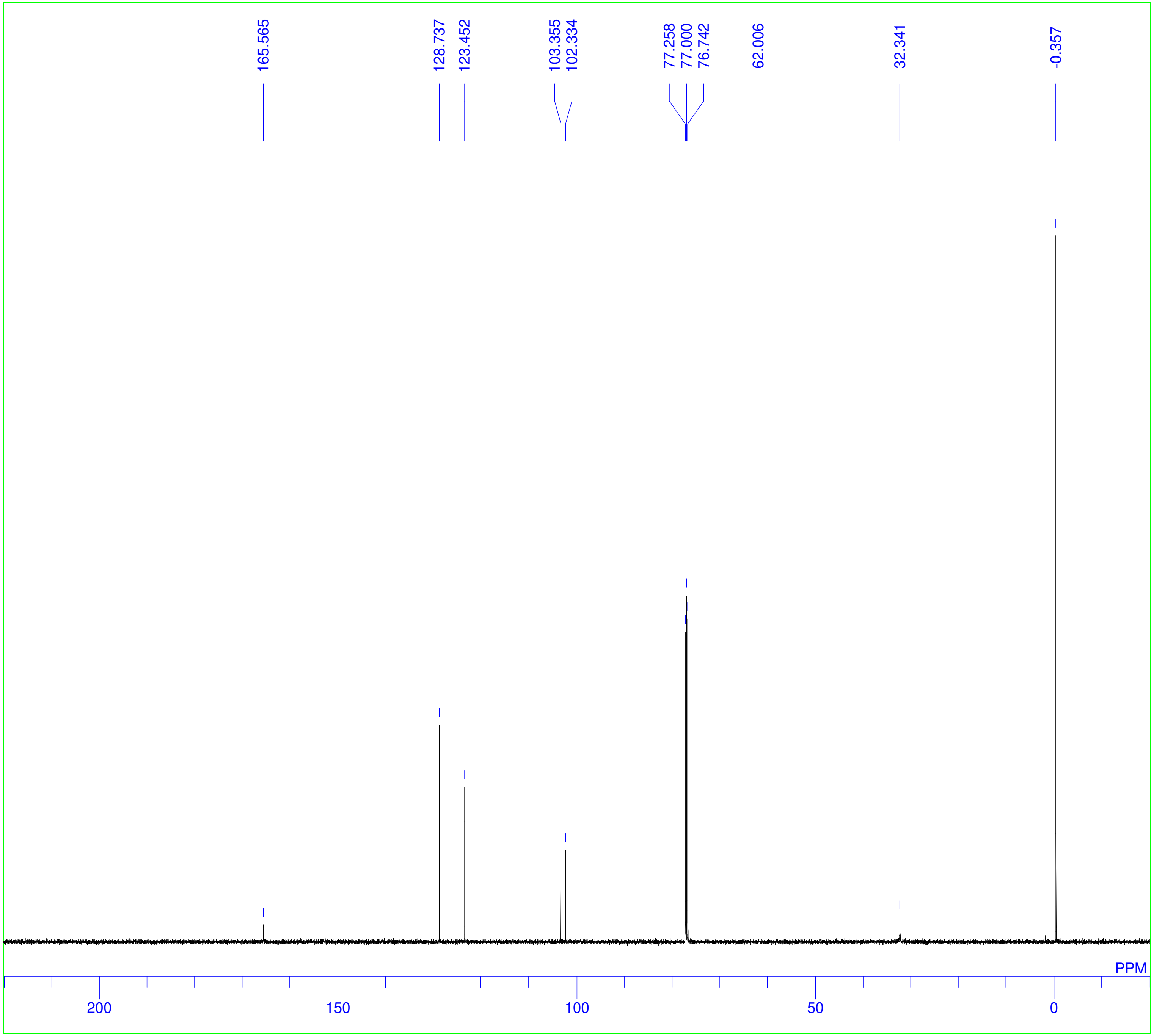

DFILE	(E)-18c_13C.als
COMNT	
DATIM	2023-01-06 08:06:10
OBNUC	13C
EXMOD	carbon.jxp
OBFRQ	125.77 MHz
OBSET	7.87 KHz
OBFIN	4.21 Hz
POINT	26214
FREQU	31446.54 Hz
SCANS	1024
ACQTM	0.8336 sec
PD	2.0000 sec
PW1	3.87 usec
IRNUC	1H
CTEMP	20.1 c
SLVNT	CDCL3
EXREF	77.00 ppm
BF	0.30 Hz
RGAIN	24

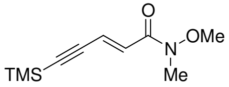

(*E*)-18c

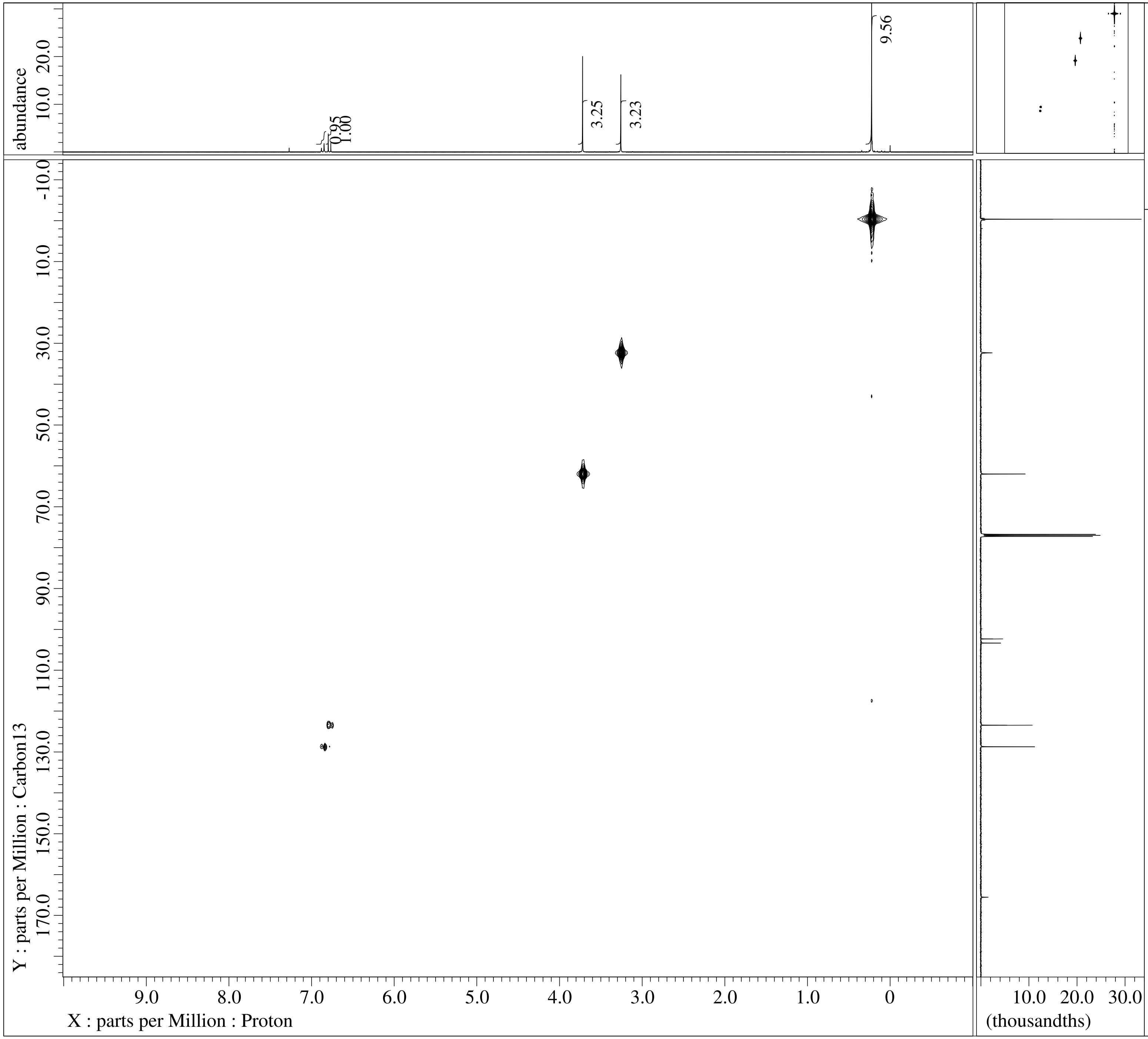


```
---- PROCESSING PARAMETERS ----
sinbell4( -60, 160 )
zerofill( 1 )
fft( 1, TRUE, TRUE )
ppm
[transpose]
sinbell4( -60, 160 )
zerofill( 2 )
fft( 1, TRUE, TRUE )
ppm
abs
```

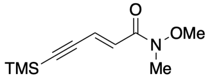

(*E*)-18c

```
Filename             = 4-p_HMQC_HMQC-2-5.jdf
Author               = delta
Experiment           = hmqc.jxp
Sample_Id            = (E)-TMS alkyne_HMQC
Solvent              = CHLOROFORM-D
Creation_Time        =  4-AUG-2024 15:11:13
Revision_Time        =  5-AUG-2024 16:39:13
Current_Time         =  5-AUG-2024 16:40:26

Comment              = gradient enhanced HMQC
Data_Format          = 2D REAL REAL
Dim_Size             = 819, 512
Dim_Title            = Proton Carbon13
Dim_Units            = [ppm] [ppm]
Dimensions           = X Y
Site                 = JNM-ECA500II
Spectrometer         = DELTA2_NMR

Field_Strength       = 11.7473579[T] (500[MHz]
X_Acq_Duration       = 0.10911744[s]
X_Domain             = 1H
X_Freq               = 500.15991521[MHz]
X_Offset             = 5.0[ppm]
X_Points             = 1024
X_Prescans           = 4
X_Resolution         = 9.16443788[Hz]
X_Sweep              = 9.38438438[kHz]
X_Sweep_Clipped      = 7.50750751[kHz]
Y_Domain             = 13C
Y_Freq               = 125.76529768[MHz]
Y_Offset             = 85[ppm]
Y_Points             = 256
Y_Prescans           = 0
Y_Resolution         = 98.4438004[Hz]
Y_Sweep              = 25.2016129[kHz]
Tri_Domain           = Proton
Tri_Freq             = 500.15991521[MHz]
Tri_Offset           = 5.0[ppm]
Clipped              = FALSE
Scans                = 4
Total_Scans          = 1024

Relaxation_Delay     = 1.5[s]
Recvr_Gain           = 50
Temp_Get             = 24.2[dC]
X_Acq_Time           = 0.10911744[s]
X_Atn                = 3.2[dB]
X_Gamma              = 42576375
X_Pulse              = 7.59[us]
```

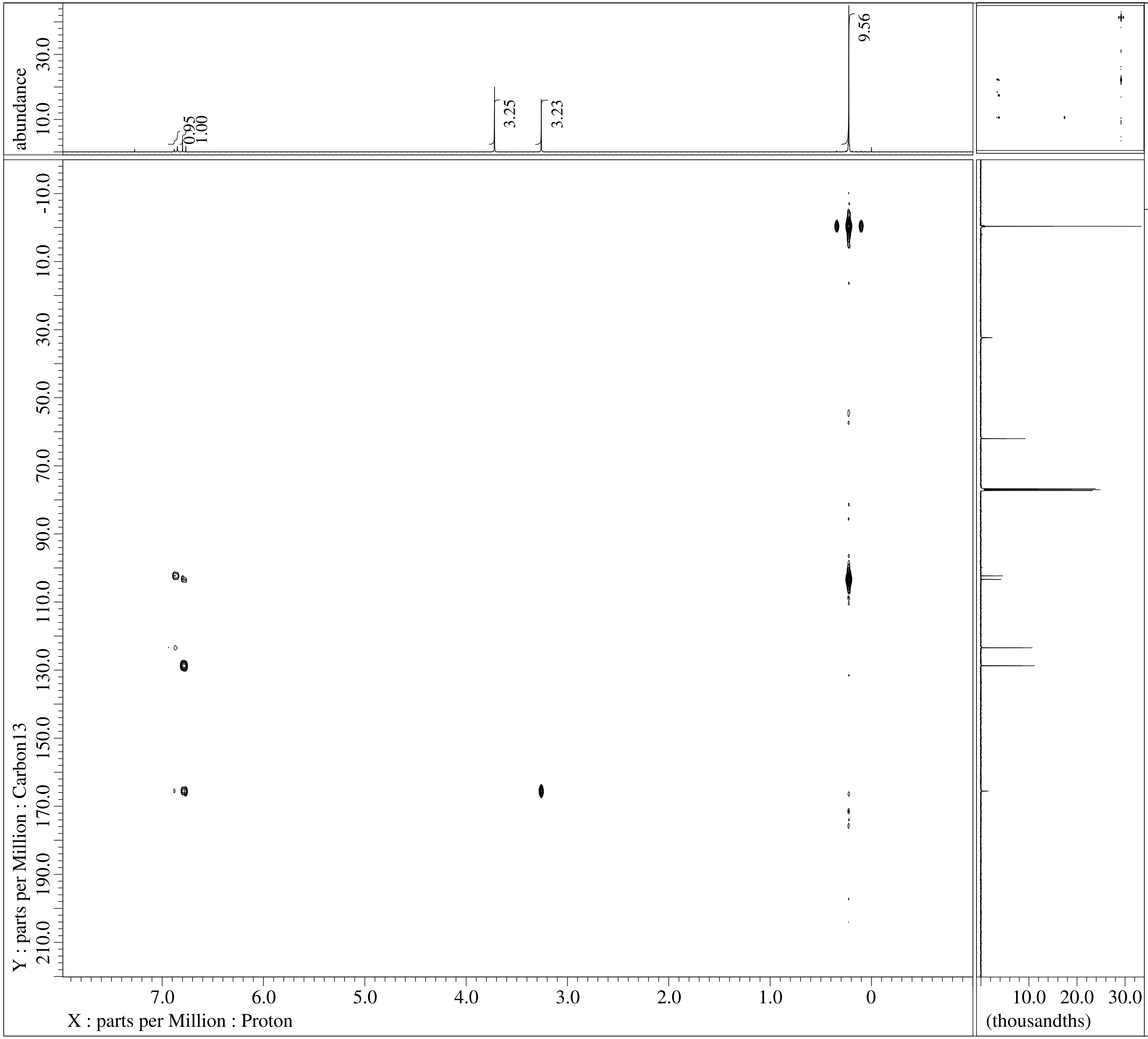


```
---- PROCESSING PARAMETERS ----
gauss( 5.0[Hz], 0.0[s] )
sinbell_auto
zerofill( 1 )
fft( 1, TRUE, TRUE )
ppm
[transpose]
sinbell4( -60, 160 )
trapezoid( 0[%], 5[%], 80[%], 100[%] )
zerofill( 2 )
fft( 1, TRUE, TRUE )
```

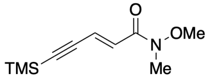

(*E*)-**18c**

```
Filename            = HT-4-p_HMQC,HMBC_HMBC-1
Author              = delta
Experiment          = hmbc.jxp
Sample_Id           = HT-4-p_HMQC,HMBC
Solvent             = CHLOROFORM-D
Creation_Time       =  9-JAN-2024 19:55:57
Revision_Time       = 10-APR-2024 22:23:06
Current_Time        = 10-APR-2024 22:24:03

Comment             = gradient enhanced HMBC
Data_Format         = 2D REAL REAL
Dim_Size            = 1638, 512
Dim_Title           = Proton Carbon13
Dim_Units           = [ppm] [ppm]
Dimensions          = X Y
Site                = JNM-ECA500II
Spectrometer        = DELTA2_NMR

Field_Strength      = 11.7473579[T] (500[MHz]
X_Acq_Duration      = 0.36306944[s]
X_Domain            = 1H
X_Freq              = 500.15991521[MHz]
X_Offset            = 3.44426147[ppm]
X_Points            = 2048
X_Prescans          = 4
X_Resolution        = 2.75429405[Hz]
X_Sweep             = 5.64079422[kHz]
X_Sweep_Clipped     = 4.51263538[kHz]
Y_Domain            = 13C
Y_Freq              = 125.76529768[MHz]
Y_Offset            = 100[ppm]
Y_Points            = 256
Y_Prescans          = 0
Y_Resolution        = 122.99275819[Hz]
Y_Sweep             = 31.4861461[kHz]
Tri_Domain          = Proton
Tri_Freq            = 500.15991521[MHz]
Tri_Offset          = 5.0[ppm]
Clipped             = FALSE
Scans               = 8
Total_Scans         = 2048

Relaxation_Delay    = 1.5[s]
Recvr_Gain          = 50
Temp_Get            = 21.8[dC]
X_Acq_Time          = 0.36306944[s]
X_Atn               = 3.2[dB]
X_Gamma             = 42576375
X_Pulse             = 7.59[us]
```

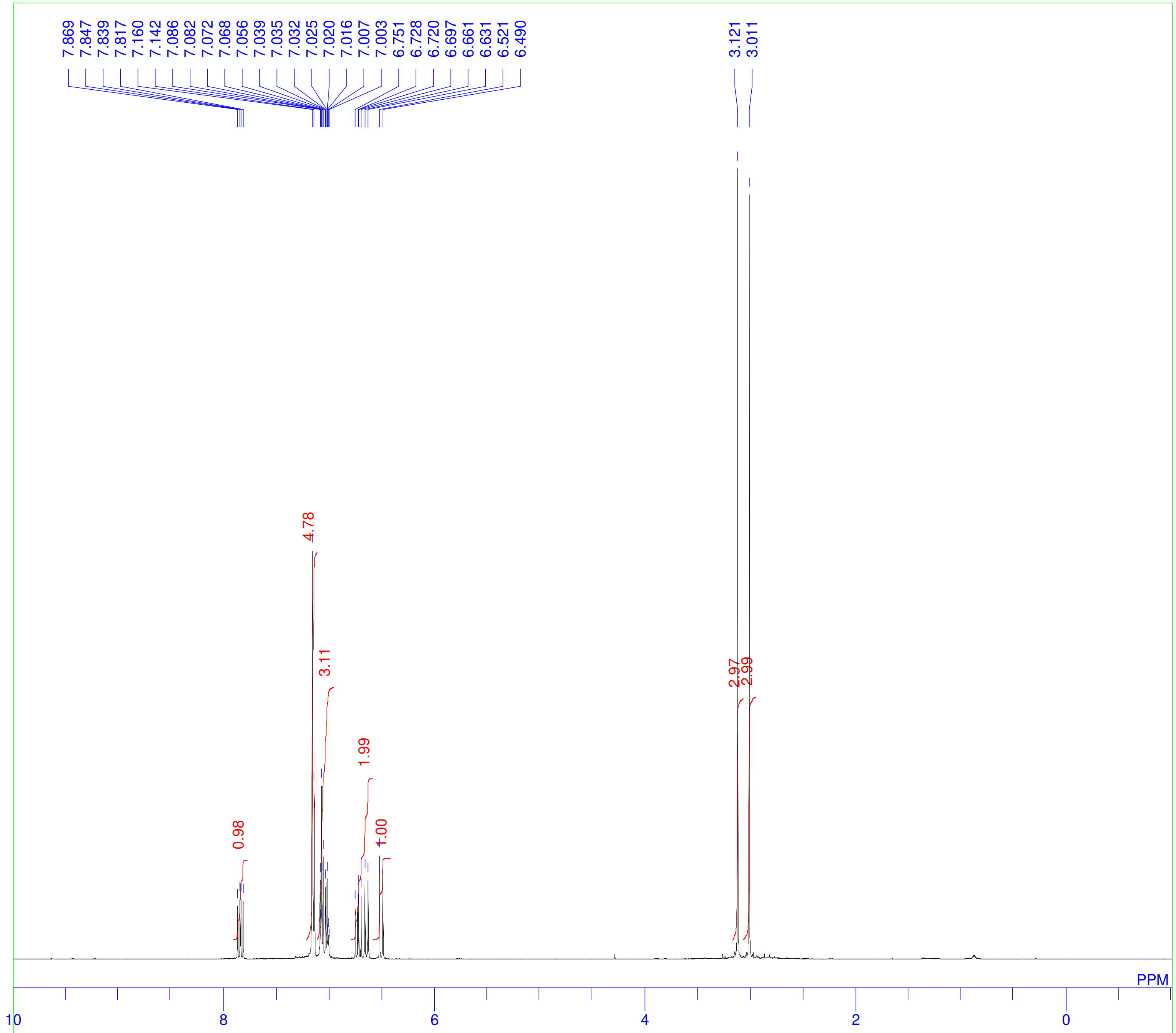

DFILE	(2E,4E)-18d_1H.als
COMNT	
DATIM	2023-01-05 12:33:05
OBNUC	1H
EXMOD	proton.jxp
OBFRQ	500.16 MHz
OBSET	2.41 KHz
OBFIN	6.01 Hz
POINT	13107
FREQU	7507.51 Hz
SCANS	8
ACQTM	1.7459 sec
PD	5.0000 sec
PW1	3.84 usec
IRNUC	1H
CTEMP	20.7 c
SLVNT	C6D6
EXREF	7.16 ppm
BF	0.30 Hz
RGAIN	32

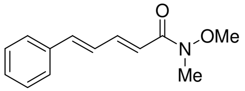

(2*E*,4*E*)-18d ((*E*)-18d)

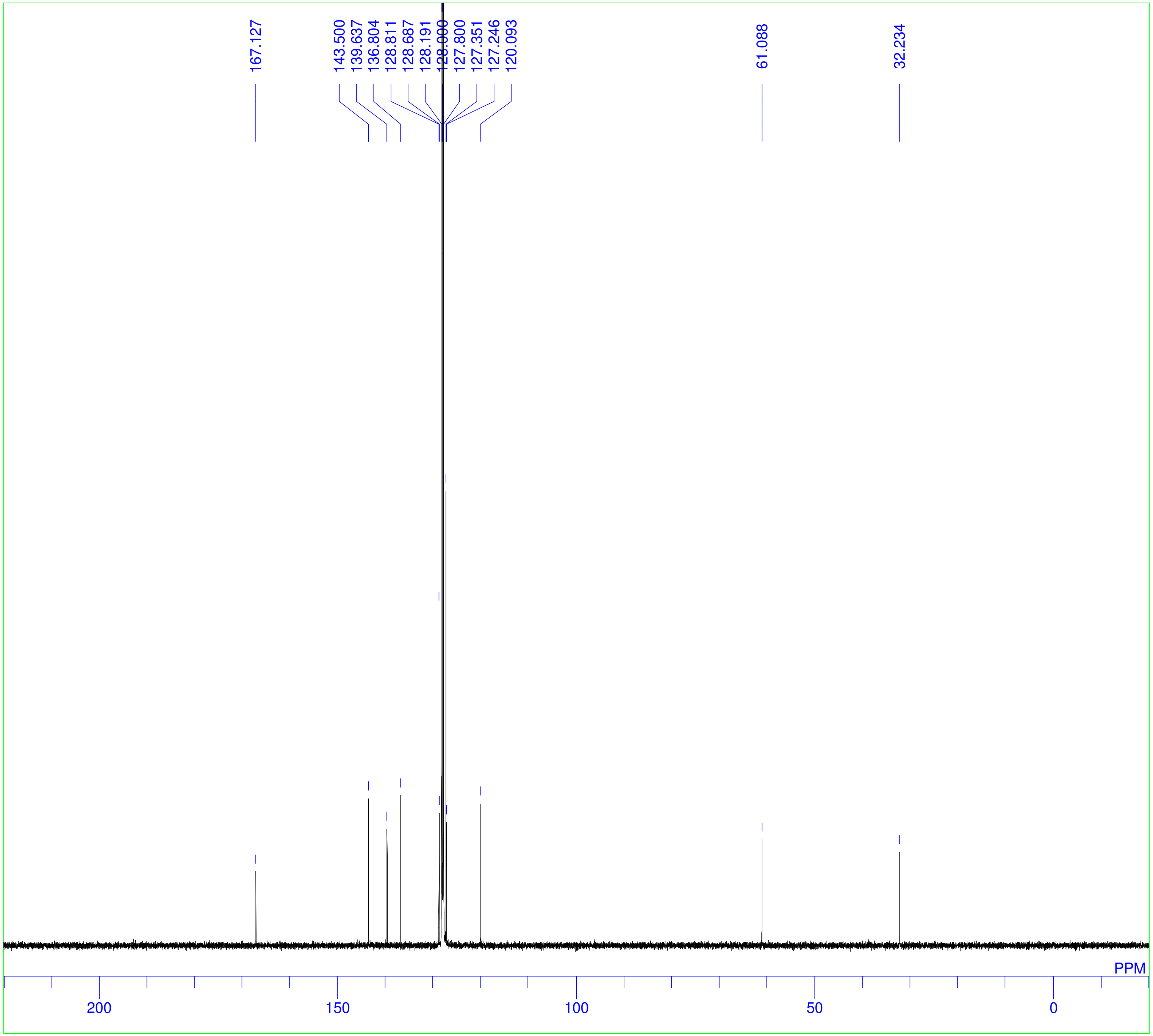

DFILE (2E,4E)-18d_13C.als
COMNT
DATIM 2023-01-05 12:35:31
OBNUC 13C
EXMOD carbon.jxp
OBFRQ 125.77 MHz
OBSET 7.87 KHz
OBFIN 4.21 Hz
POINT 26214
FREQU 31446.54 Hz
SCANS 1024
ACQTM 0.8336 sec
PD 2.0000 sec
PW1 3.87 usec
IRNUC 1H
CTEMP 20.8 c
SLVNT C6D6
EXREF 128.00 ppm
BF 0.30 Hz
RGAIN 24

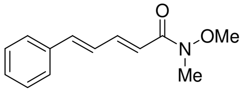

(2*E*,4*E*)-18d ((*E*)-18d)

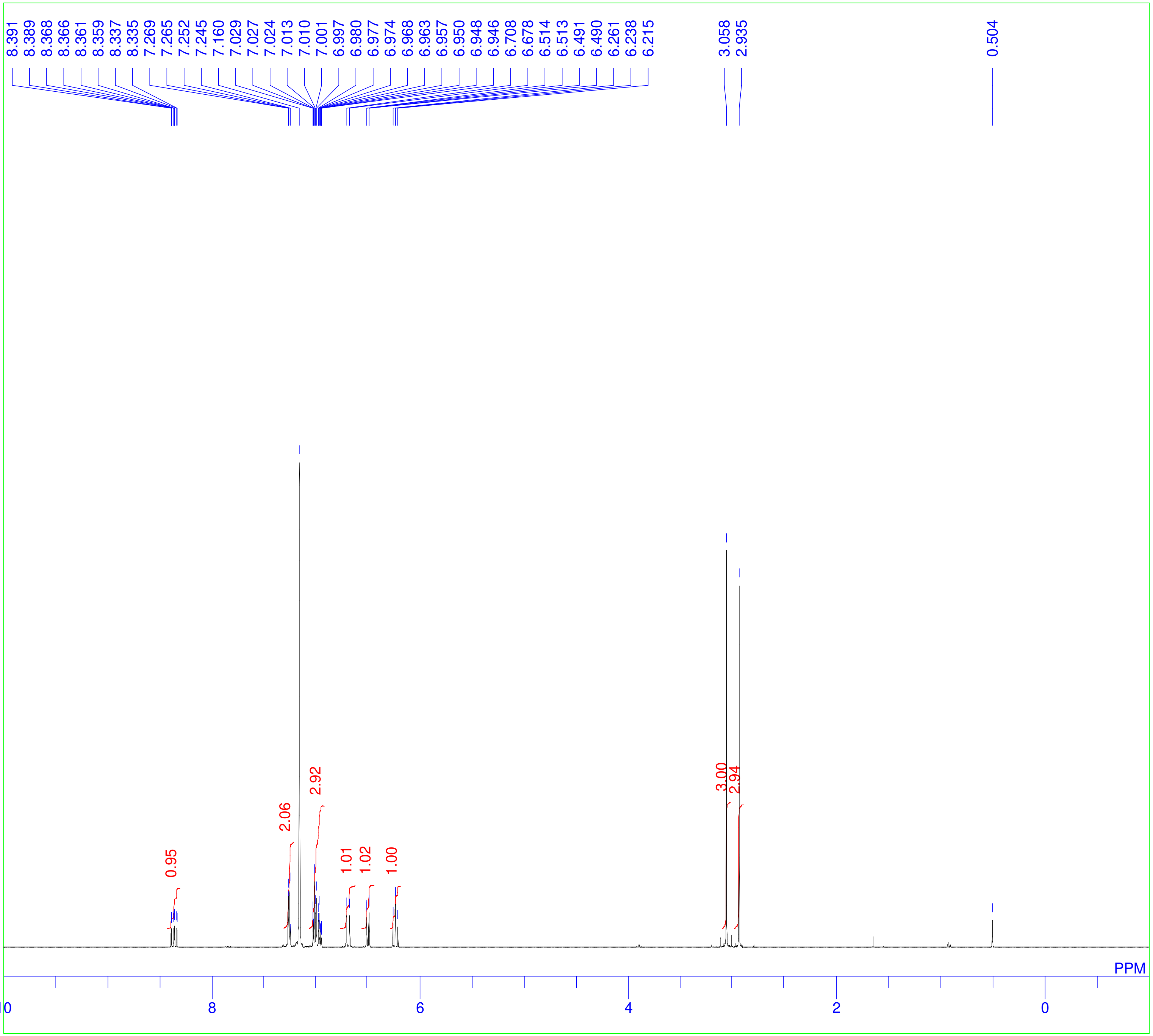

DFILE	(2E,4Z)-18d_1H.als
COMNT	
DATIM	2023-01-12 19:56:07
OBNUC	1H
EXMOD	proton.jxp
OBFRQ	500.16 MHz
OBSET	2.41 KHz
OBFIN	6.01 Hz
POINT	13107
FREQU	7507.51 Hz
SCANS	8
ACQTM	1.7459 sec
PD	5.0000 sec
PW1	3.84 usec
IRNUC	1H
CTEMP	22.5 c
SLVNT	C6D6
EXREF	7.16 ppm
BF	0.30 Hz
RGAIN	40

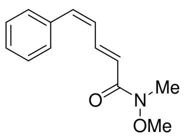

(2*E*,4*Z*)-18d

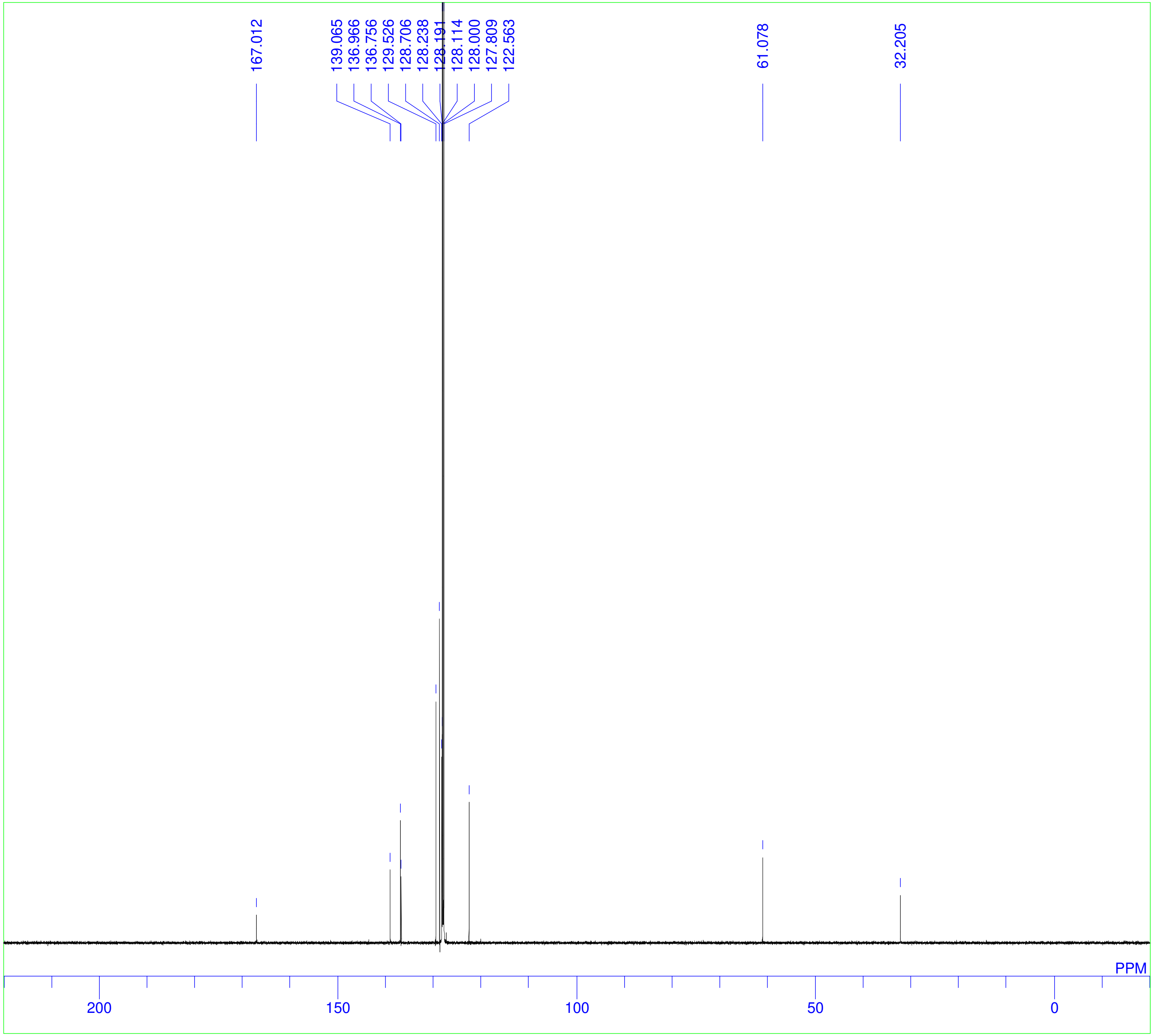

DFILE (2E,4Z)-18d_13C.als
COMNT
DATIM 2023-01-12 20:07:50
OBNUC 13C
EXMOD carbon.jxp
OBFRQ 125.77 MHz
OBSET 7.87 KHz
OBFIN 4.21 Hz
POINT 26214
FREQU 31446.54 Hz
SCANS 1024
ACQTM 0.8336 sec
PD 2.0000 sec
PW1 3.87 usec
IRNUC 1H
CTEMP 22.3 c
SLVNT C6D6
EXREF 128.00 ppm
BF 0.30 Hz
RGAIN 30

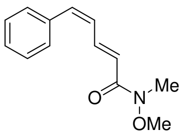

(2*E*,4*Z*)-18d

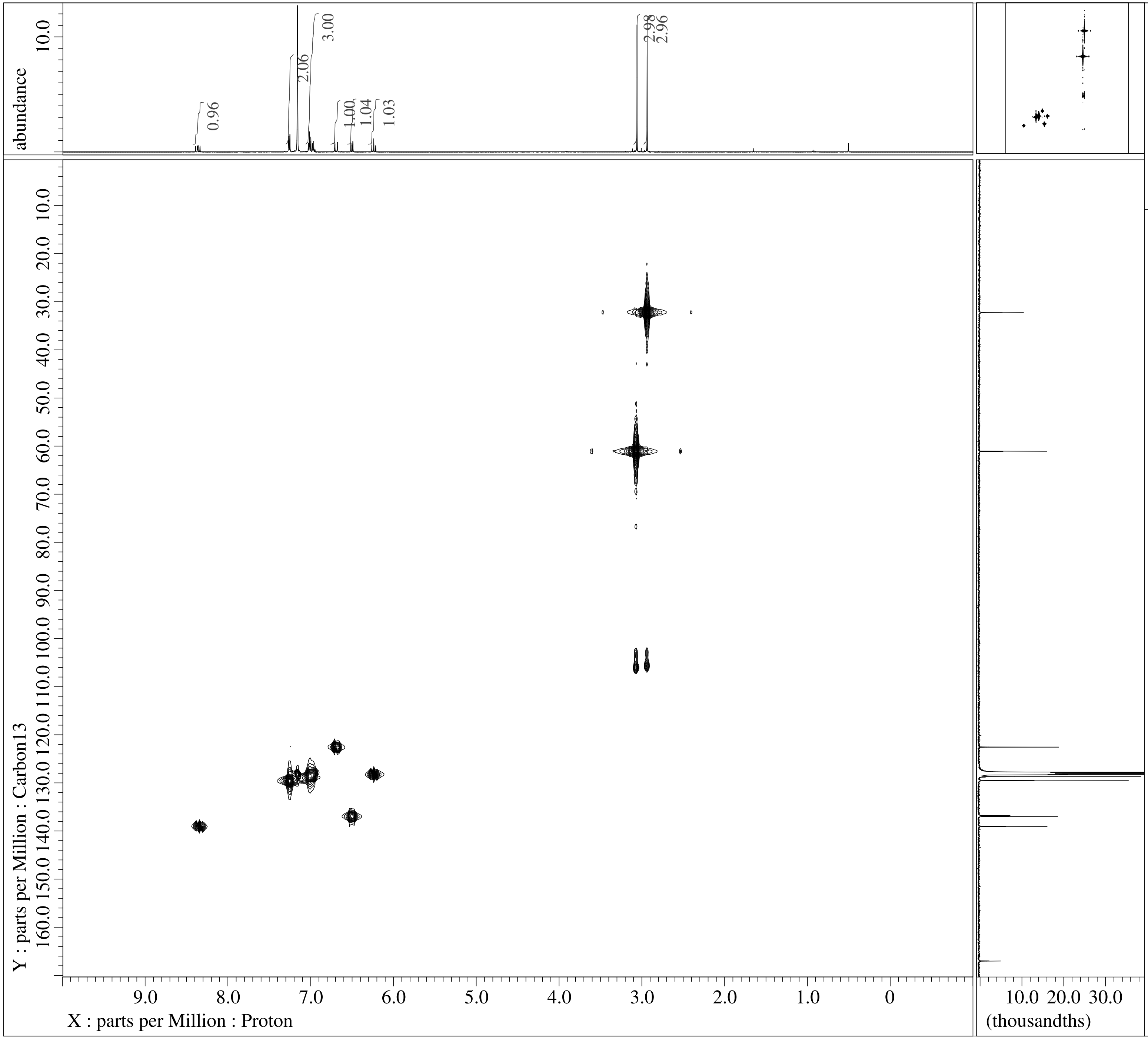


```
---- PROCESSING PARAMETERS ----
sinbell4( -60, 160 )
zerofill( 1 )
fft( 1, TRUE, TRUE )
ppm
[transpose]
sinbell4( -60, 160 )
zerofill( 2 )
fft( 1, TRUE, TRUE )
ppm
abs
```

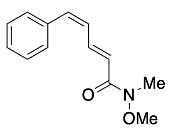

(2*E*,4*Z*)-18d

```
Filename            = HT-1241-PTLC 2 _HMQC_HM
Author              = delta
Experiment          = hmqc.jxp
Sample_Id           = HT-1241-PTLC 2
Solvent             = BENZENE-D6
Creation_Time       = 13-JAN-2023 11:13:28
Revision_Time       = 10-APR-2024 22:35:02
Current_Time        = 10-APR-2024 22:35:36

Comment             = gradient enhanced HMQC
Data_Format         = 2D REAL REAL
Dim_Size            = 819, 512
Dim_Title           = Proton Carbon13
Dim_Units           = [ppm] [ppm]
Dimensions          = X Y
Site                = JNM-ECA500II
Spectrometer        = DELTA2_NMR

Field_Strength      = 11.7473579[T] (500[MHz]
X_Acq_Duration      = 0.10911744[s]
X_Domain            = 1H
X_Freq              = 500.15991521[MHz]
X_Offset            = 5.0[ppm]
X_Points            = 1024
X_Prescans          = 4
X_Resolution        = 9.16443788[Hz]
X_Sweep             = 9.38438438[kHz]
X_Sweep_Clipped     = 7.50750751[kHz]
Y_Domain            = 13C
Y_Freq              = 125.76529768[MHz]
Y_Offset            = 85[ppm]
Y_Points            = 256
Y_Prescans          = 0
Y_Resolution        = 83.60980308[Hz]
Y_Sweep             = 21.40410959[kHz]
Tri_Domain          = Proton
Tri_Freq            = 500.15991521[MHz]
Tri_Offset          = 5.0[ppm]
Clipped             = FALSE
Scans               = 8
Total_Scans         = 2048

Relaxation_Delay    = 1.5[s]
Recvr_Gain          = 50
Temp_Get            = 21.6[dC]
X_Acq_Time          = 0.10911744[s]
X_Atn               = 3.2[dB]
X_Gamma             = 42576375
X_Pulse             = 7.68[us]
```

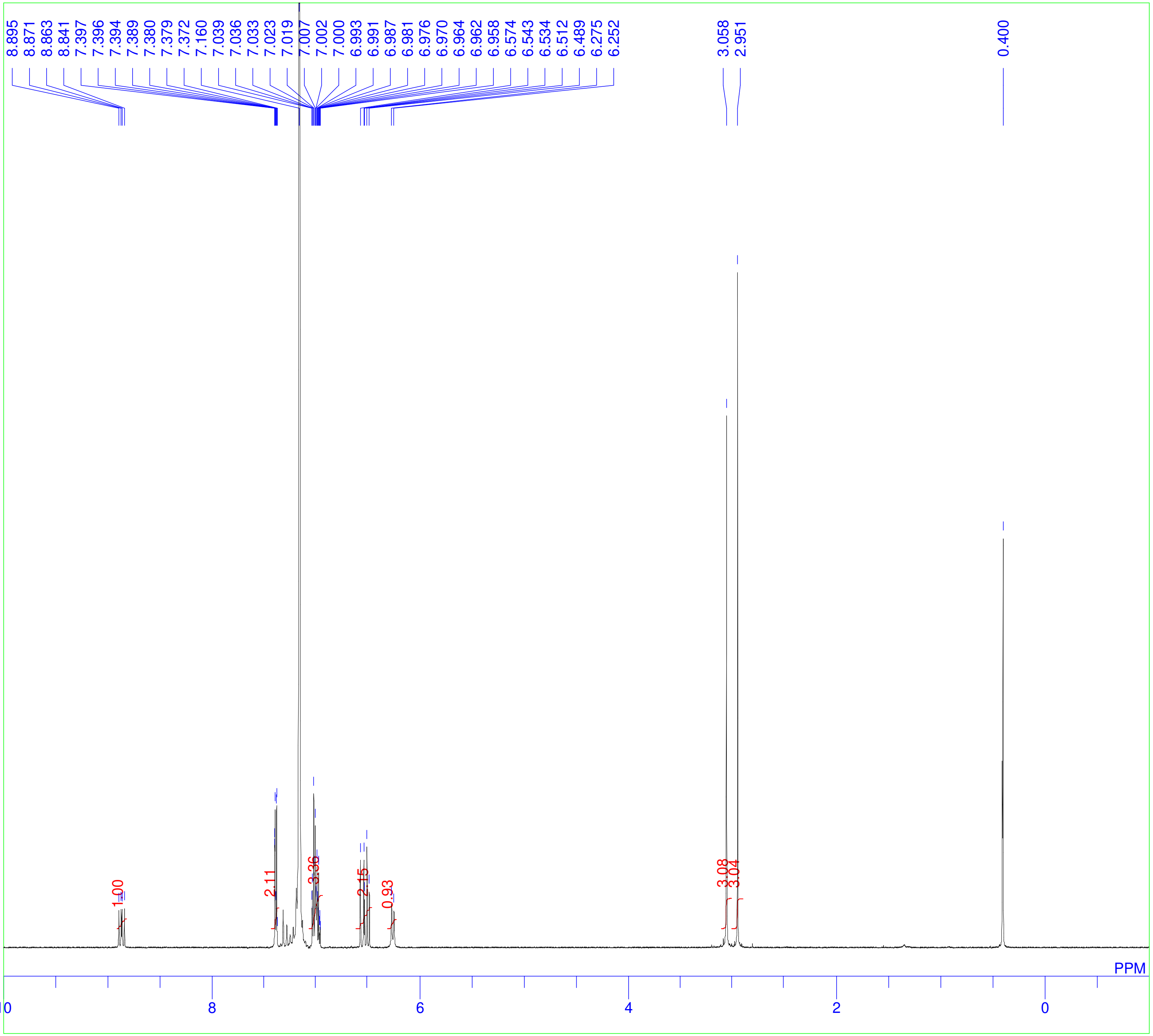

DFILE	(2Z,4E)-18d_1H.als
COMNT	
DATIM	2023-01-16 20:41:21
OBNUC	1H
EXMOD	proton.jxp
OBFRQ	500.16 MHz
OBSET	2.41 KHz
OBFIN	6.01 Hz
POINT	13107
FREQU	7507.51 Hz
SCANS	8
ACQTM	1.7459 sec
PD	5.0000 sec
PW1	3.84 usec
IRNUC	1H
CTEMP	22.0 c
SLVNT	C6D6
EXREF	7.16 ppm
BF	0.30 Hz
RGAIN	44

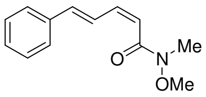

(2*Z*,4*E*)-18d ((*Z*)-18d)

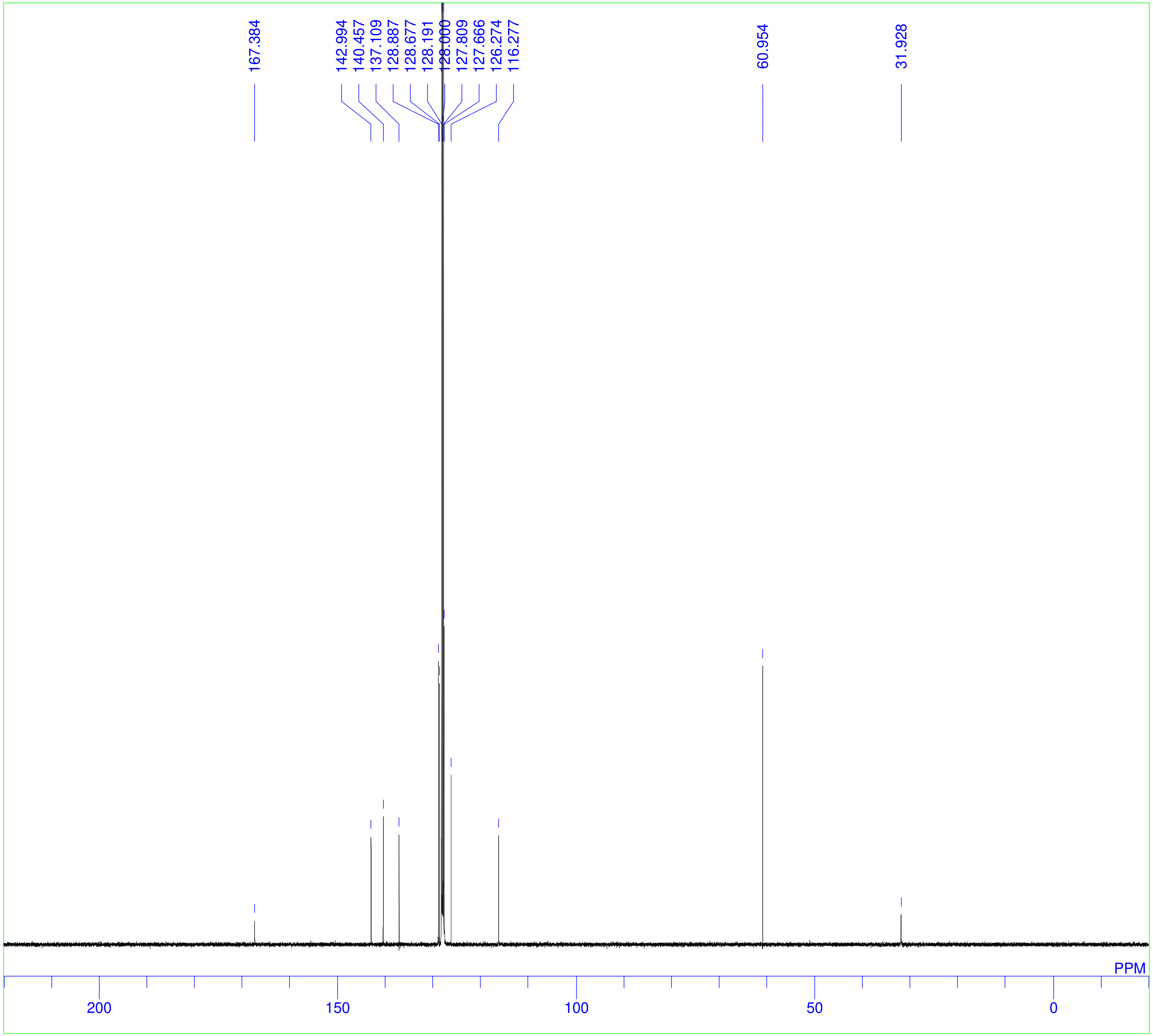

DFILE	(2Z,4E)-18d_13C.als
COMNT	
DATIM	2023-01-16 21:07:12
OBNUC	13C
EXMOD	carbon.jxp
OBFRQ	125.77 MHz
OBSET	7.87 KHz
OBFIN	4.21 Hz
POINT	26214
FREQU	31446.54 Hz
SCANS	1024
ACQTM	0.8336 sec
PD	2.0000 sec
PW1	3.87 usec
IRNUC	1H
CTEMP	21.9 c
SLVNT	C6D6
EXREF	128.00 ppm
BF	0.30 Hz
RGAIN	30

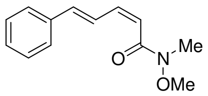

(2*Z*,4*E*)-18d ((*Z*)-18d)

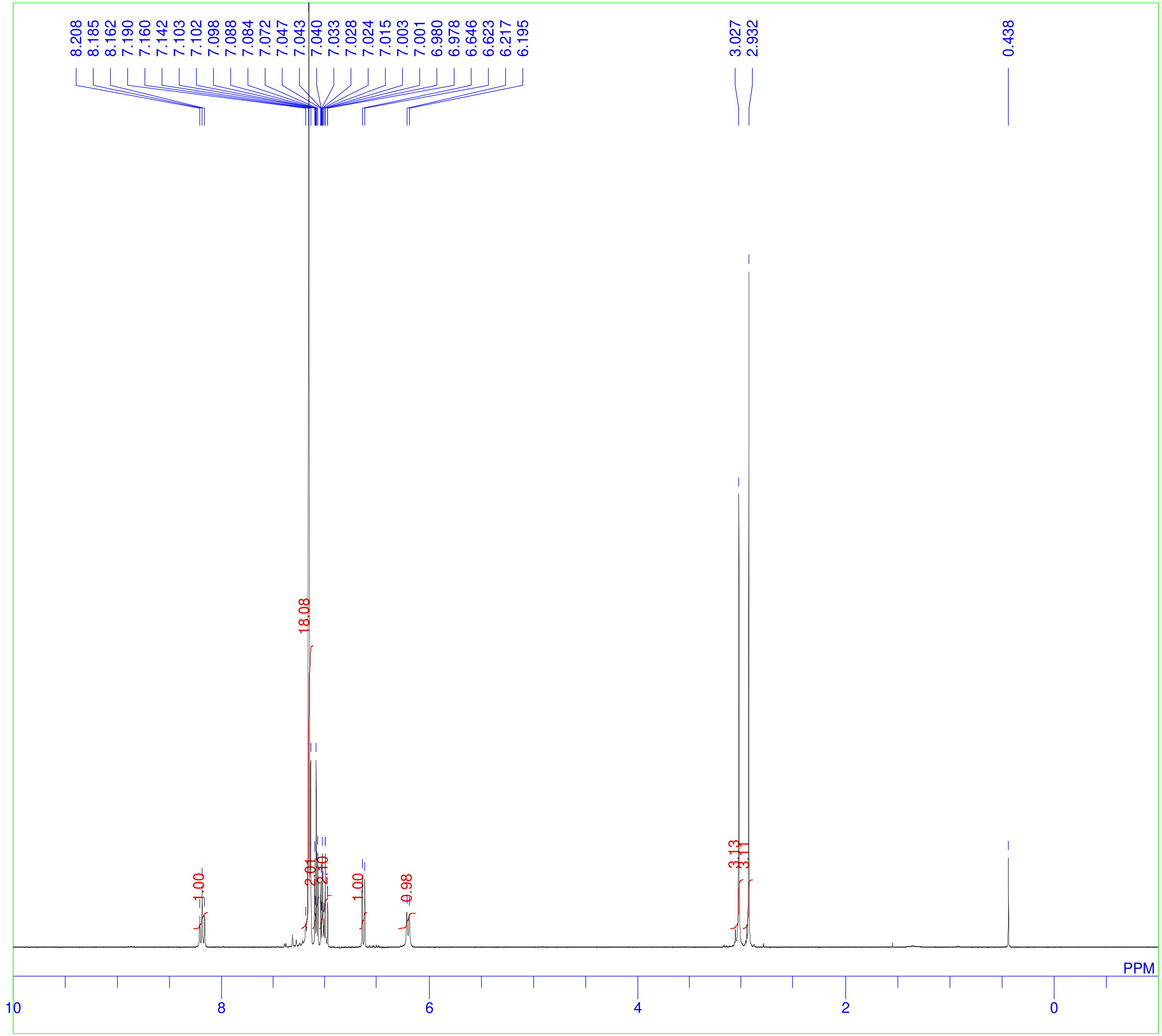

DFILE	(2Z,4Z)-18d_1H.als
COMNT	
DATIM	2023-01-12 20:01:49
OBNUC	1H
EXMOD	proton.jxp
OBFRQ	500.16 MHz
OBSET	2.41 KHz
OBFIN	6.01 Hz
POINT	13107
FREQU	7507.51 Hz
SCANS	8
ACQTM	1.7459 sec
PD	5.0000 sec
PW1	3.84 usec
IRNUC	1H
CTEMP	22.5 c
SLVNT	C6D6
EXREF	7.16 ppm
BF	0.30 Hz
RGAIN	42

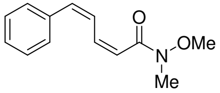

(2*Z*,4*Z*)-18d

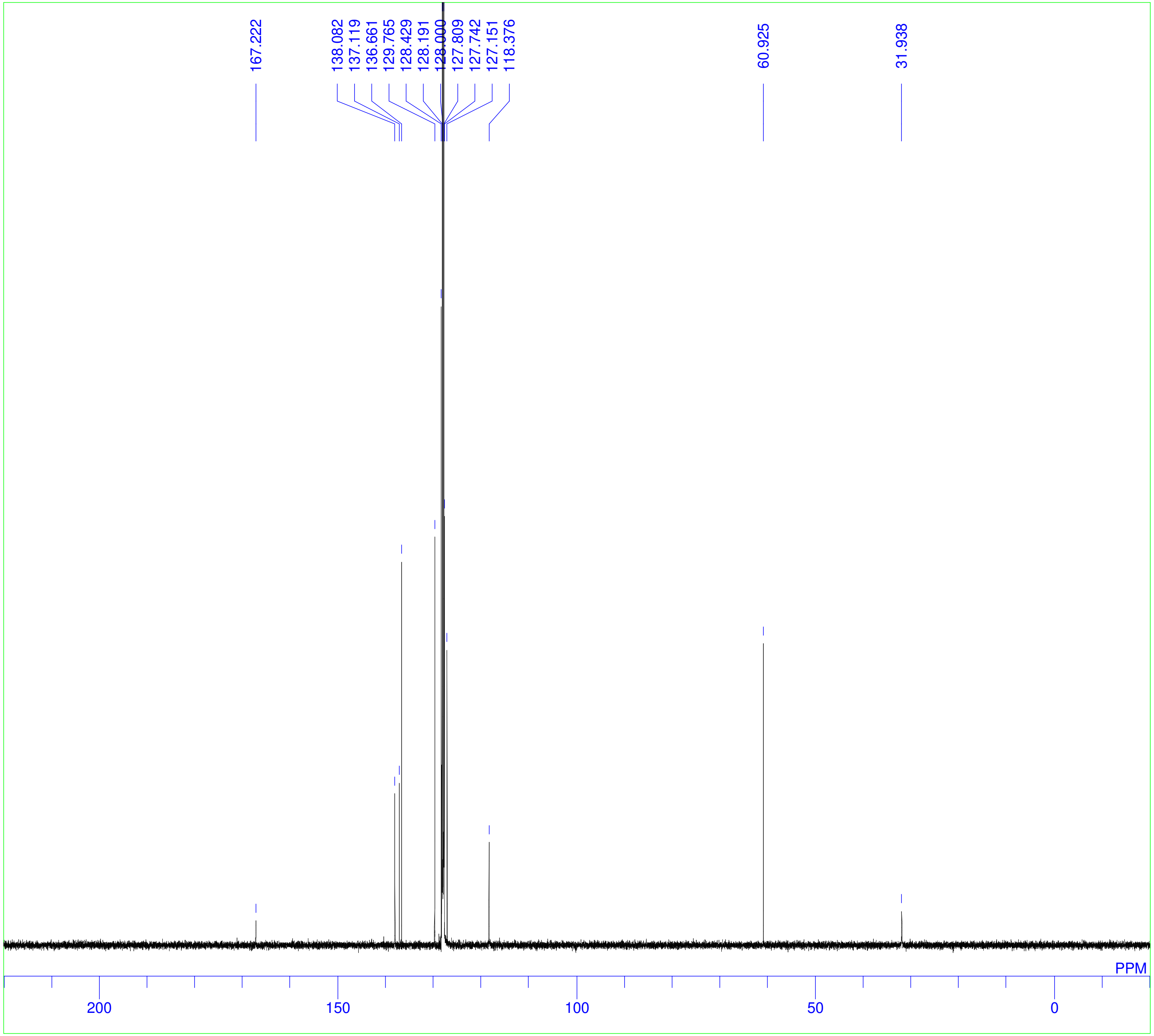

DFILE (2Z,4Z)-18d_13C.als
COMNT
DATIM 2023-01-12 21:01:22
OBNUC 13C
EXMOD carbon.jxp
OBFRQ 125.77 MHz
OBSET 7.87 KHz
OBFIN 4.21 Hz
POINT 26214
FREQU 31446.54 Hz
SCANS 1024
ACQTM 0.8336 sec
PD 2.0000 sec
PW1 3.87 usec
IRNUC 1H
CTEMP 22.2 c
SLVNT C6D6
EXREF 128.00 ppm
BF 0.30 Hz
RGAIN 26

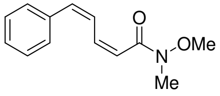

(2*Z*,4*Z*)-18d

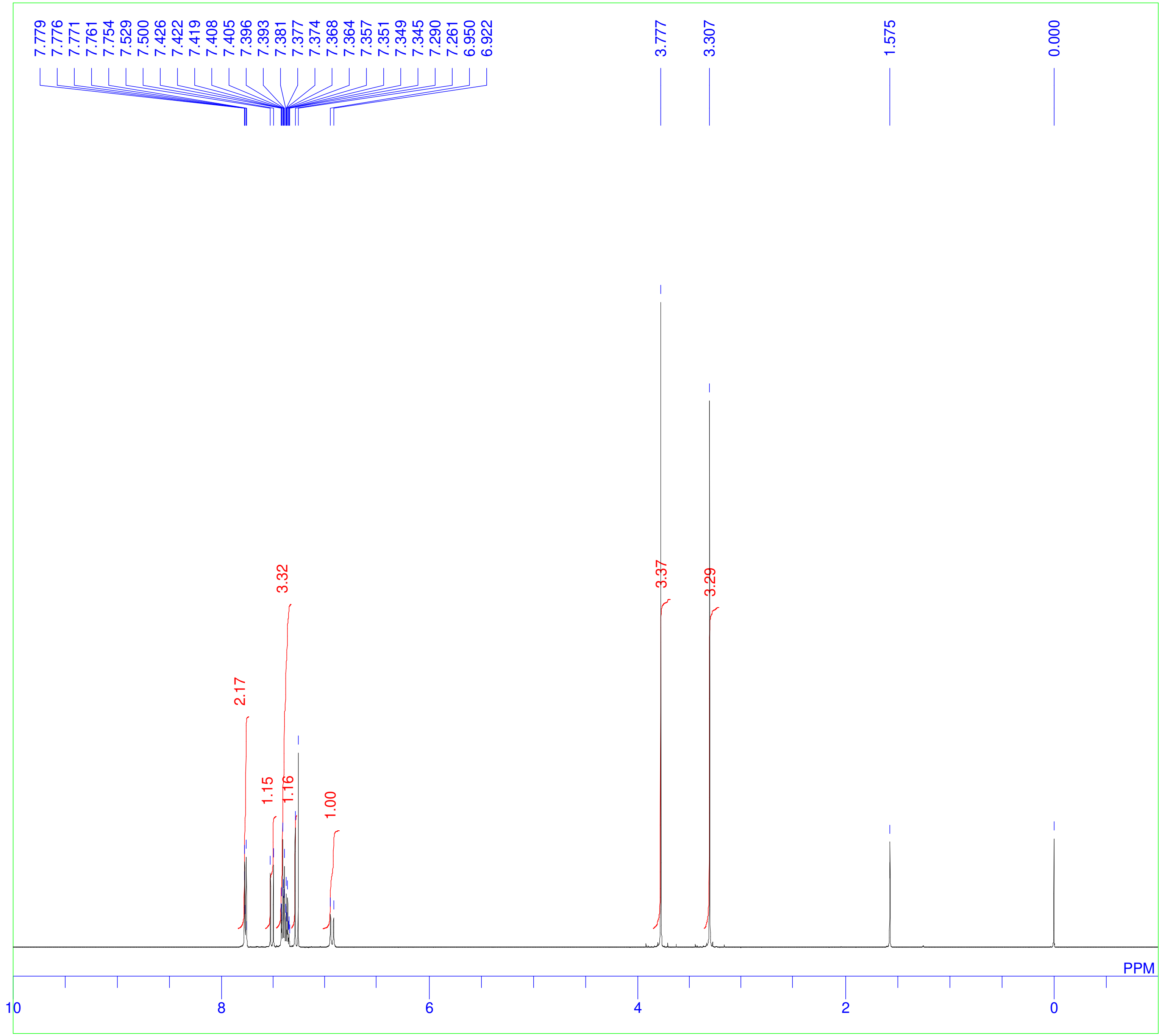

DFILE	(E)-18e_1H.als
COMNT	
DATIM	2023-09-13 21:21:24
OBNUC	1H
EXMOD	proton.jxp
OBFRQ	500.16 MHz
OBSET	2.41 KHz
OBFIN	6.01 Hz
POINT	13107
FREQU	7507.51 Hz
SCANS	8
ACQTM	1.7459 sec
PD	5.0000 sec
PW1	3.84 usec
IRNUC	1H
CTEMP	23.9 c
SLVNT	CDCL3
EXREF	0.00 ppm
BF	0.30 Hz
RGAIN	44

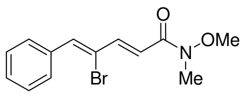

(*E*)-18e

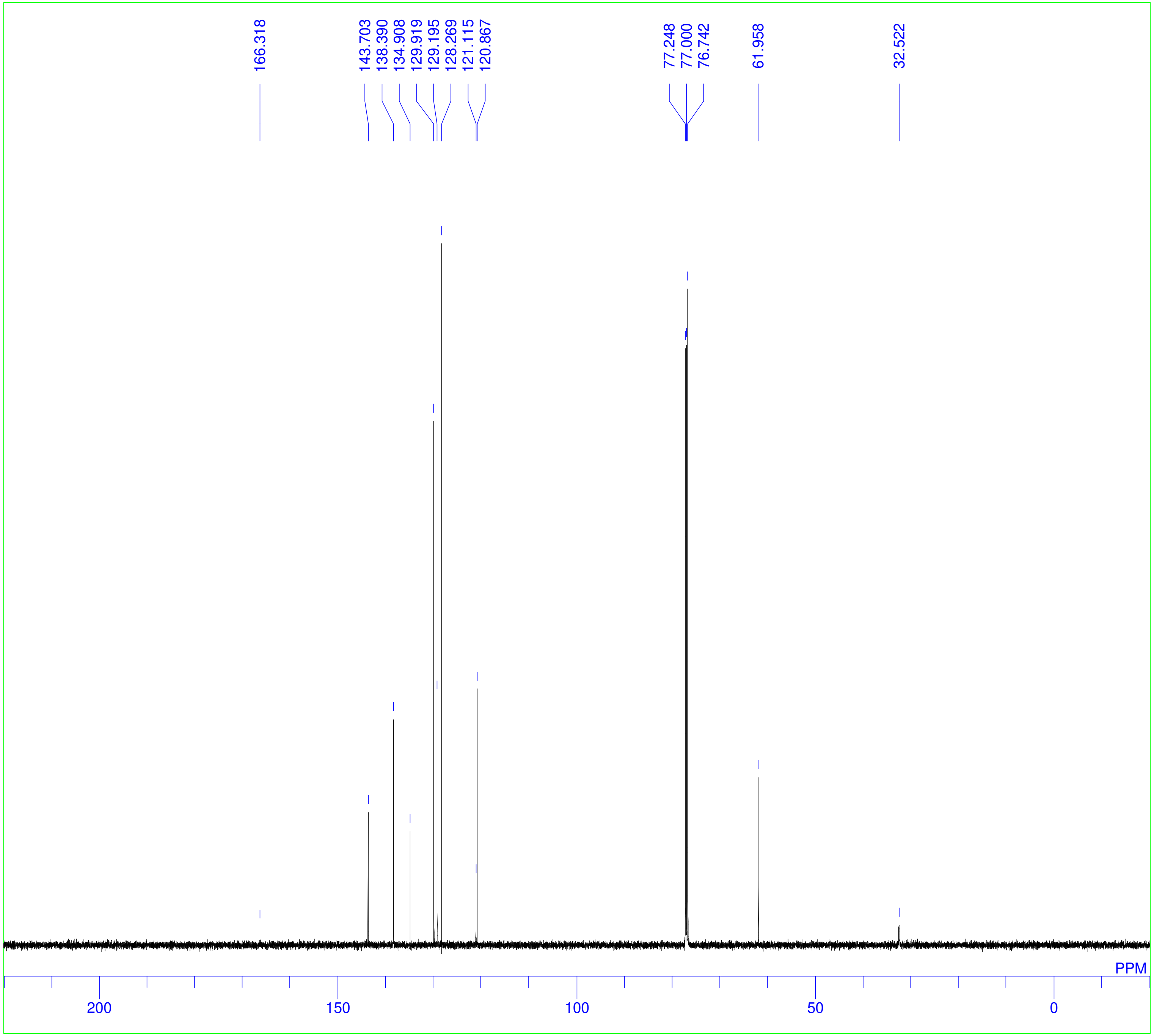

DFILE (E)-18e_13C.als
COMNT
DATIM 2023-09-16 17:59:53
OBNUC 13C
EXMOD carbon.jxp
OBFRQ 125.77 MHz
OBSET 7.87 KHz
OBFIN 4.21 Hz
POINT 26214
FREQU 31446.54 Hz
SCANS 1024
ACQTM 0.8336 sec
PD 2.0000 sec
PW1 3.87 usec
IRNUC 1H
CTEMP 24.4 c
SLVNT CDCL3
EXREF 77.00 ppm
BF 0.30 Hz
RGAIN 30

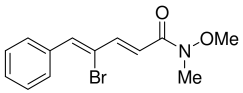

(*E*)-18e

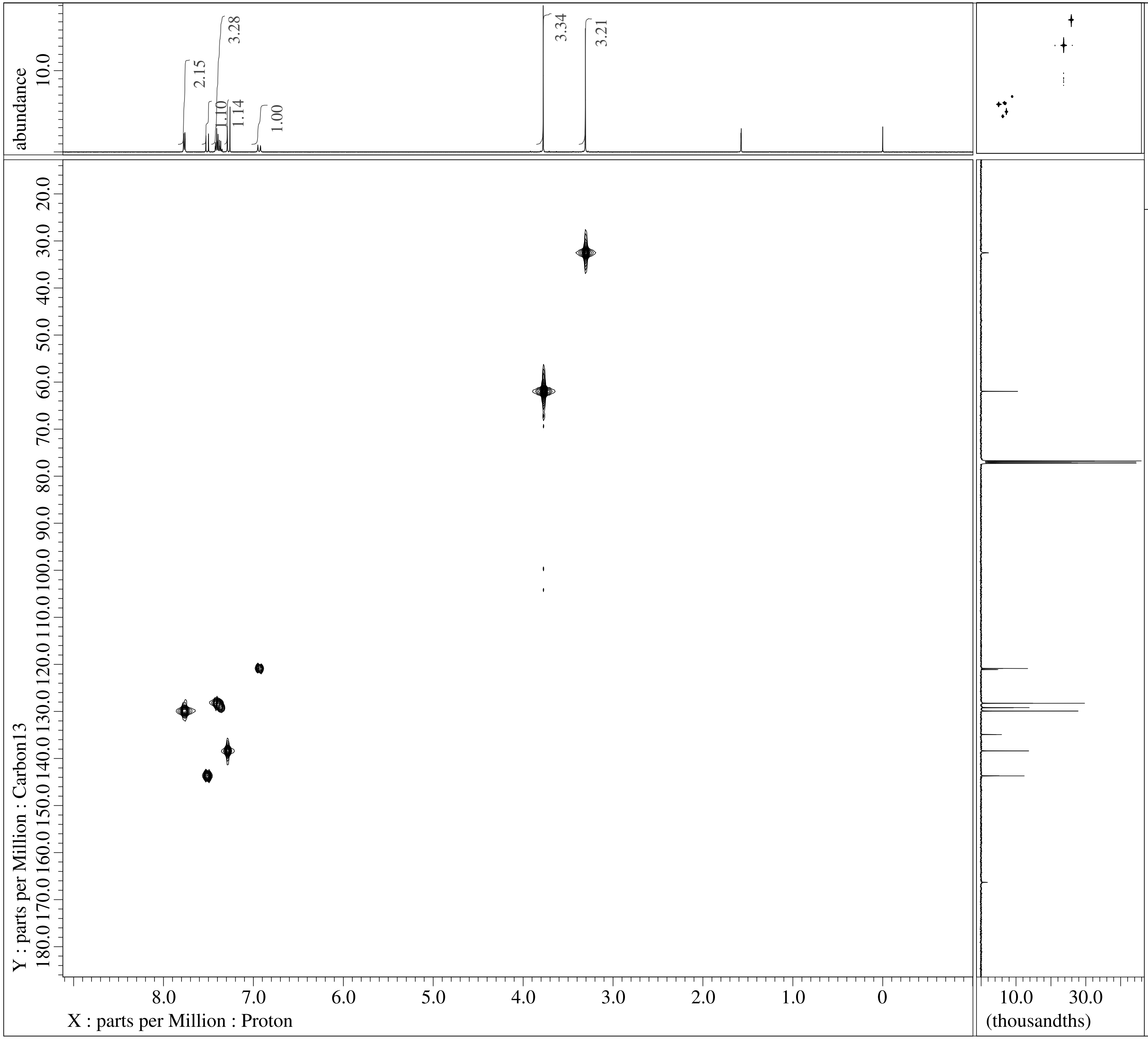


```
---- PROCESSING PARAMETERS ----
sinbell4( -60, 160 )
zerofill( 1 )
fft( 1, TRUE, TRUE )
ppm
[transpose]
sinbell4( -60, 160 )
zerofill( 2 )
fft( 1, TRUE, TRUE )
ppm
abs
```

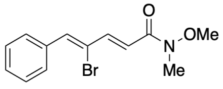

(E)-18e

```
Filename            = HT-1545,1546-TM_13C_HMQ
Author              = delta
Experiment          = hmqc.jxp
Sample_Id           = HT-1545,1546-TM_13C
Solvent             = CHLOROFORM-D
Creation_Time       = 16-SEP-2023 18:48:46
Revision_Time       = 10-APR-2024 22:41:50
Current_Time        = 10-APR-2024 22:42:14

Comment             = gradient enhanced HMQC
Data_Format         = 2D REAL REAL
Dim_Size            = 819, 512
Dim_Title           = Proton Carbon13
Dim_Units           = [ppm] [ppm]
Dimensions          = X Y
Site                = JNM-ECA500II
Spectrometer        = DELTA2_NMR

Field_Strength      = 11.7473579[T] (500[MHz]
X_Acq_Duration      = 0.15859712[s]
X_Domain            = 1H
X_Freq              = 500.15991521[MHz]
X_Offset            = 3.95043477[ppm]
X_Points            = 1024
X_Prescans          = 4
X_Resolution        = 6.30528474[Hz]
X_Sweep             = 6.45661157[kHz]
X_Sweep_Clipped     = 5.16528926[kHz]
Y_Domain            = 13C
Y_Freq              = 125.76529768[MHz]
Y_Offset            = 99.53739122[ppm]
Y_Points            = 256
Y_Prescans          = 0
Y_Resolution        = 85.51335377[Hz]
Y_Sweep             = 21.89141856[kHz]
Tri_Domain          = Proton
Tri_Freq            = 500.15991521[MHz]
Tri_Offset          = 5.0[ppm]
Clipped             = FALSE
Scans               = 8
Total_Scans         = 2048

Relaxation_Delay    = 1.5[s]
Recvr_Gain          = 50
Temp_Get            = 24[dC]
X_Acq_Time          = 0.15859712[s]
X_Atn               = 3.2[dB]
X_Gamma             = 42576375
X_Pulse             = 7.68[us]
```

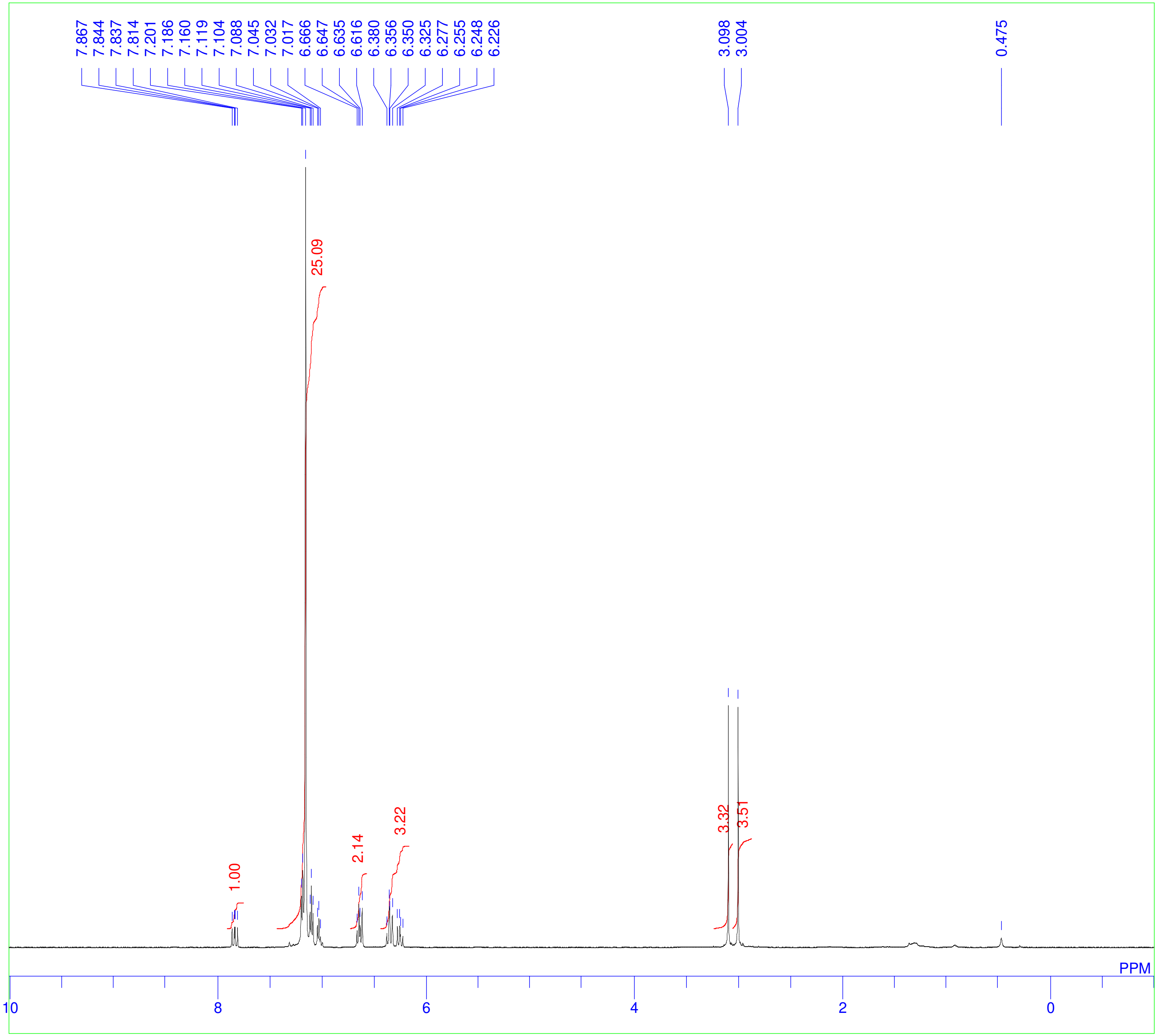

DFILE (E)-18f_1H.als
COMNT
DATIM 2023-01-10 16:49:14
OBNUC 1H
EXMOD proton.jxp
OBFRQ 500.16 MHz
OBSET 2.41 KHz
OBFIN 6.01 Hz
POINT 13107
FREQU 7507.51 Hz
SCANS 8
ACQTM 1.7459 sec
PD 5.0000 sec
PW1 3.84 usec
IRNUC 1H
CTEMP 21.7 c
SLVNT C6D6
EXREF 7.16 ppm
BF 0.30 Hz
RGAIN 42

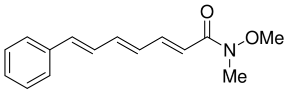

(*E*)-18f ((2*E*,4*E*,6*E*)-18f)

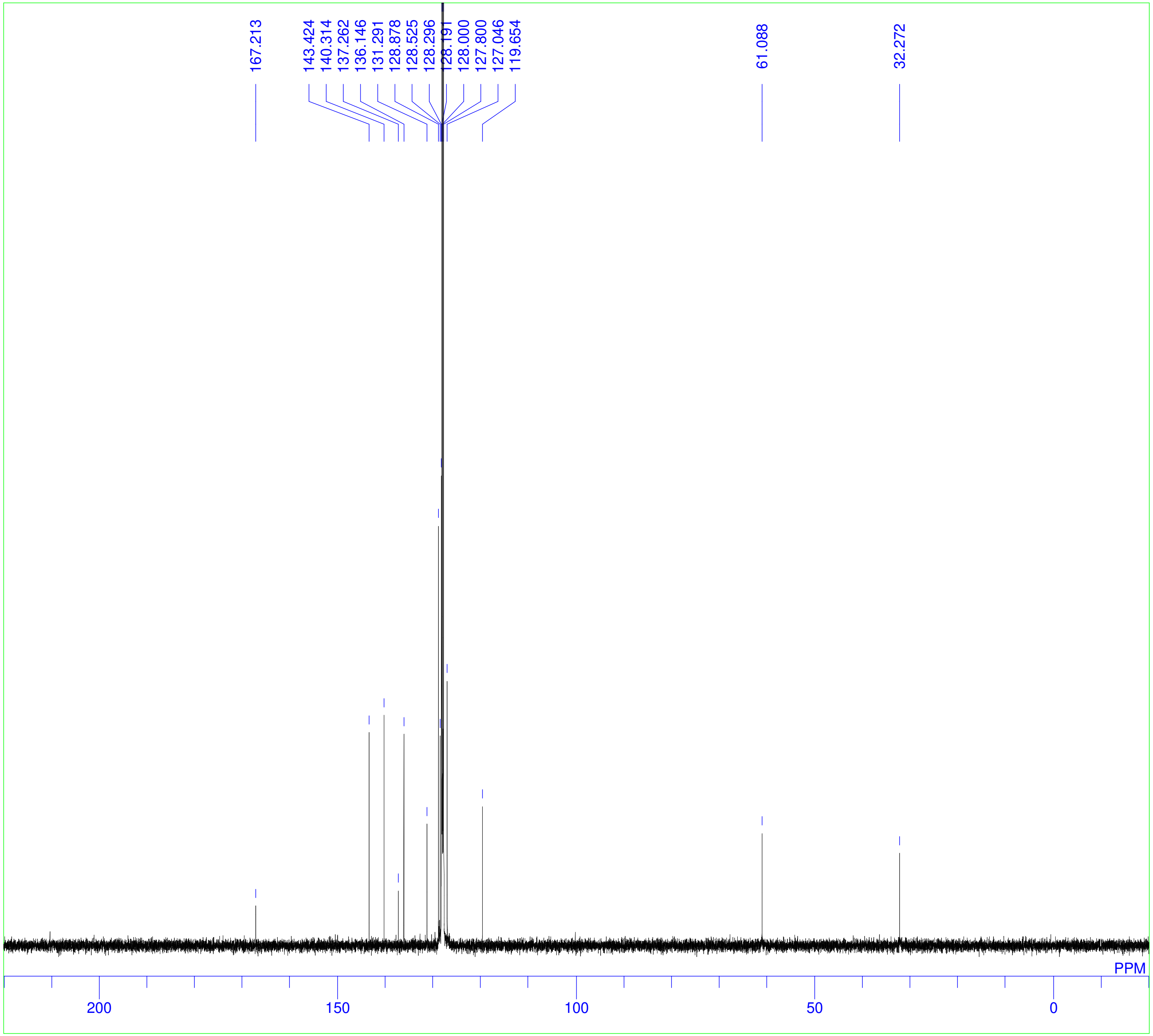

DFILE	(E)-18f_13C.als
COMNT	
DATIM	2023-01-09 10:18:53
OBNUC	13C
EXMOD	carbon.jxp
OBFRQ	125.77 MHz
OBSET	7.87 KHz
OBFIN	4.21 Hz
POINT	26214
FREQU	31446.54 Hz
SCANS	1024
ACQTM	0.8336 sec
PD	2.0000 sec
PW1	3.87 usec
IRNUC	1H
CTEMP	21.3 c
SLVNT	C6D6
EXREF	128.00 ppm
BF	0.30 Hz
RGAIN	26

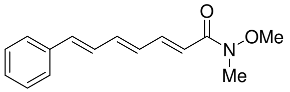

(*E*)-18f ((2*E*,4*E*,6*E*)-18f)

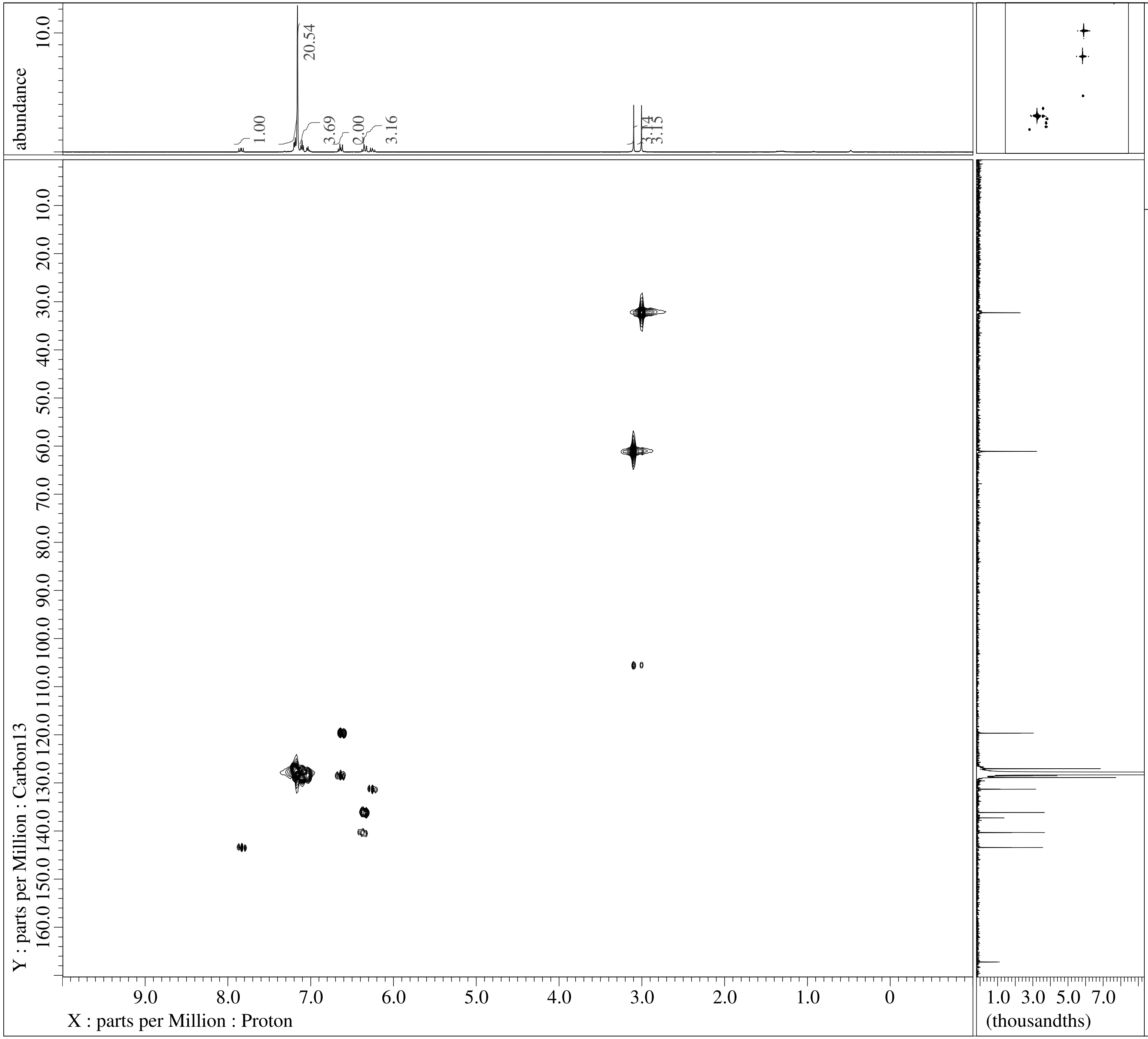


```
---- PROCESSING PARAMETERS ----
sinbell4( -60, 160 )
zerofill( 1 )
fft( 1, TRUE, TRUE )
ppm
[transpose]
sinbell4( -60, 160 )
zerofill( 2 )
fft( 1, TRUE, TRUE )
ppm
abs
```

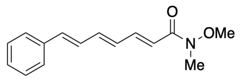

(*E*)-18f ((2*E*,4*E*,6*E*)-18f)

```
Filename            = dien-aldehyde-E_HMQC_HM
Author              = delta
Experiment          = hmqc.jxp
Sample_Id           = dien-aldehyde-E_HMQC
Solvent             = BENZENE-D6
Creation_Time       =  9-JAN-2023 12:18:35
Revision_Time       = 10-APR-2024 22:45:49
Current_Time        = 10-APR-2024 22:46:26

Comment             = gradient enhanced HMQC
Data_Format         = 2D REAL REAL
Dim_Size            = 819, 512
Dim_Title           = Proton Carbon13
Dim_Units           = [ppm] [ppm]
Dimensions          = X Y
Site                = JNM-ECA500II
Spectrometer        = DELTA2_NMR

Field_Strength      = 11.7473579[T] (500[MHz]
X_Acq_Duration      = 0.10911744[s]
X_Domain            = 1H
X_Freq              = 500.15991521[MHz]
X_Offset            = 5.0[ppm]
X_Points            = 1024
X_Prescans          = 4
X_Resolution        = 9.16443788[Hz]
X_Sweep             = 9.38438438[kHz]
X_Sweep_Clipped     = 7.50750751[kHz]
Y_Domain            = 13C
Y_Freq              = 125.76529768[MHz]
Y_Offset            = 85[ppm]
Y_Points            = 256
Y_Prescans          = 0
Y_Resolution        = 83.60980308[Hz]
Y_Sweep             = 21.40410959[kHz]
Tri_Domain          = Proton
Tri_Freq            = 500.15991521[MHz]
Tri_Offset          = 5.0[ppm]
Clipped             = FALSE
Scans               = 16
Total_Scans         = 4096

Relaxation_Delay    = 1.5[s]
Recvr_Gain          = 50
Temp_Get            = 21.8[dC]
X_Acq_Time          = 0.10911744[s]
X_Atn               = 3.2[dB]
X_Gamma             = 42576375
X_Pulse             = 7.68[us]
```

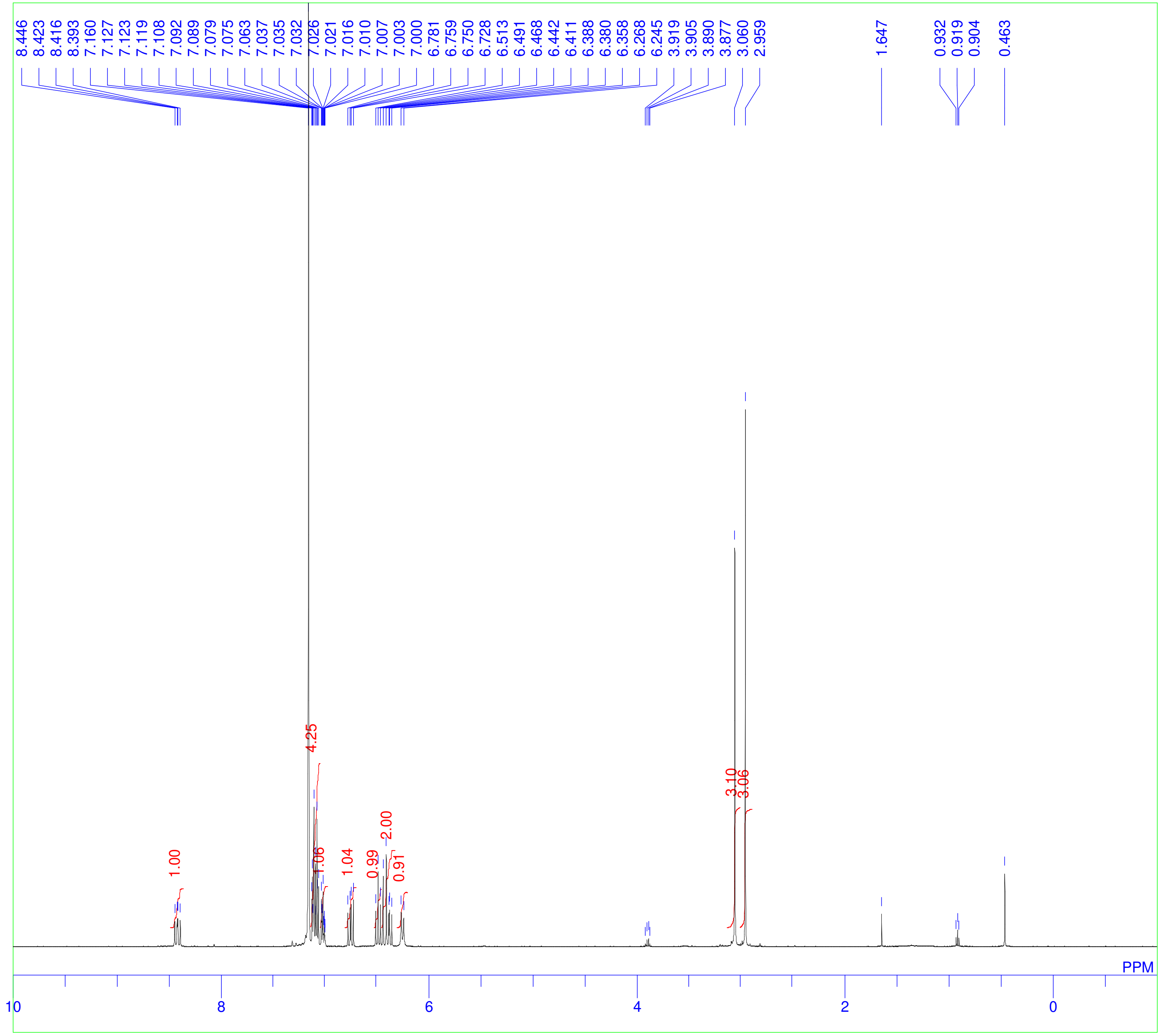

DFILE	(Z)-18f_1H.als
COMNT	
DATIM	2023-01-27 19:45:45
OBNUC	1H
EXMOD	proton.jxp
OBFRQ	500.16 MHz
OBSET	2.41 KHz
OBFIN	6.01 Hz
POINT	13107
FREQU	7507.51 Hz
SCANS	8
ACQTM	1.7459 sec
PD	5.0000 sec
PW1	3.84 usec
IRNUC	1H
CTEMP	19.6 c
SLVNT	C6D6
EXREF	7.16 ppm
BF	0.30 Hz
RGAIN	40

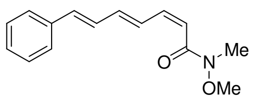

(*Z*)-18f ((2*Z*,4*E*,6*E*)-18f)

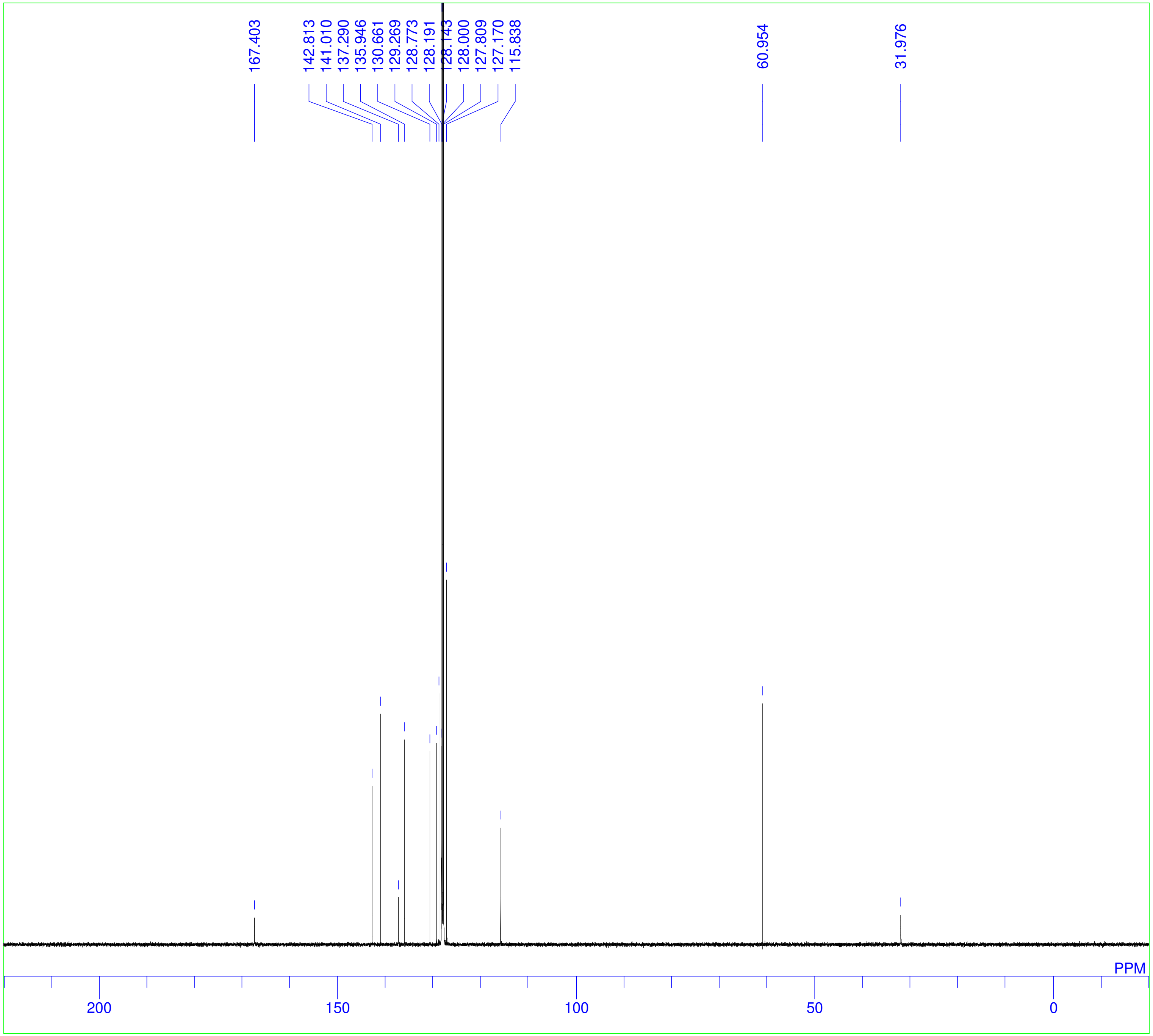

DFILE	(Z)-18f_13C.als
COMNT	
DATIM	2023-01-27 21:07:31
OBNUC	13C
EXMOD	carbon.jxp
OBFRQ	125.77 MHz
OBSET	7.87 KHz
OBFIN	4.21 Hz
POINT	26214
FREQU	31446.54 Hz
SCANS	1024
ACQTM	0.8336 sec
PD	2.0000 sec
PW1	3.87 usec
IRNUC	1H
CTEMP	19.9 c
SLVNT	C6D6
EXREF	128.00 ppm
BF	0.30 Hz
RGAIN	28

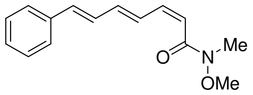

(*Z*)-18f ((2*Z*,4*E*,6*E*)-18f)

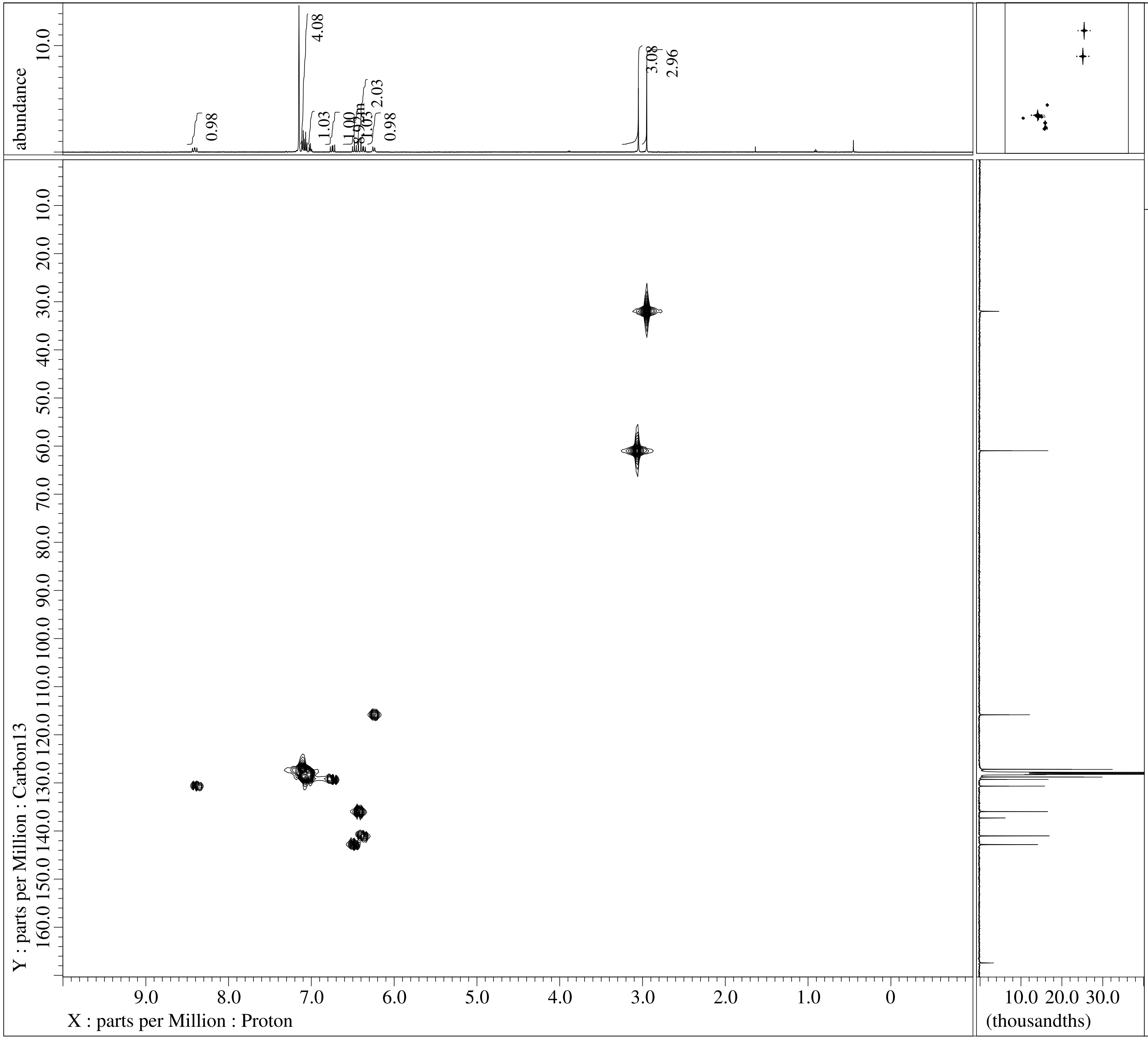


```
---- PROCESSING PARAMETERS ----
sinbell4( -60, 160 )
zerofill( 1 )
fft( 1, TRUE, TRUE )
ppm
[transpose]
sinbell4( -60, 160 )
zerofill( 2 )
fft( 1, TRUE, TRUE )
ppm
abs
```

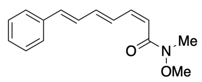

(*Z*)-18f ((2*Z*,4*E*,6*E*)-18f)

```
Filename            = HT-1258-PTLC 1_HMQC_HMQ
Author              = delta
Experiment          = hmqc.jxp
Sample_Id           = HT-1258-PTLC 1_HMQC
Solvent             = BENZENE-D6
Creation_Time       = 29-JAN-2023 18:36:04
Revision_Time       = 10-APR-2024 22:48:47
Current_Time        = 10-APR-2024 22:49:20

Comment             = gradient enhanced HMQC
Data_Format         = 2D REAL REAL
Dim_Size            = 819, 512
Dim_Title           = Proton Carbon13
Dim_Units           = [ppm] [ppm]
Dimensions          = X Y
Site                = JNM-ECA500II
Spectrometer        = DELTA2_NMR

Field_Strength      = 11.7473579[T] (500[MHz]
X_Acq_Duration      = 0.10911744[s]
X_Domain            = 1H
X_Freq              = 500.15991521[MHz]
X_Offset            = 5.0[ppm]
X_Points            = 1024
X_Prescans          = 4
X_Resolution        = 9.16443788[Hz]
X_Sweep             = 9.38438438[kHz]
X_Sweep_Clipped     = 7.50750751[kHz]
Y_Domain            = 13C
Y_Freq              = 125.76529768[MHz]
Y_Offset            = 85[ppm]
Y_Points            = 256
Y_Prescans          = 0
Y_Resolution        = 83.60980308[Hz]
Y_Sweep             = 21.40410959[kHz]
Tri_Domain          = Proton
Tri_Freq            = 500.15991521[MHz]
Tri_Offset          = 5.0[ppm]
Clipped             = FALSE
Scans               = 8
Total_Scans         = 2048

Relaxation_Delay    = 1.5[s]
Recvr_Gain          = 50
Temp_Get            = 18.8[dC]
X_Acq_Time          = 0.10911744[s]
X_Atn               = 3.2[dB]
X_Gamma             = 42576375
X_Pulse             = 7.68[us]
```

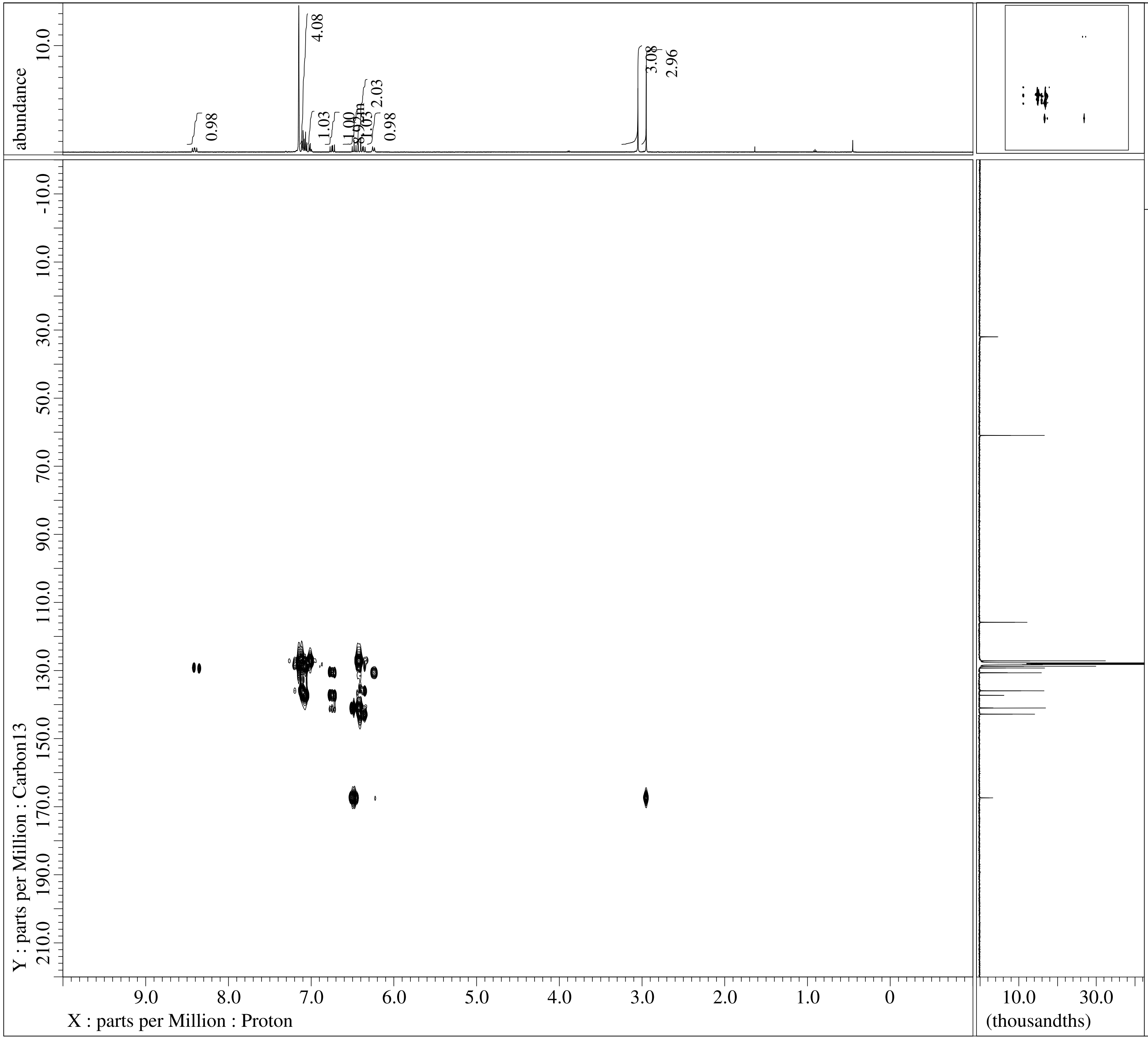

---- PROCESSING PARAMETERS ----
gauss(5.0[Hz], 0.0[s])
sinbell_auto
zerofill(1)
fft(1, TRUE, TRUE)
ppm
[transpose]
sinbell4(-60, 160)
trapezoid(0[%], 5[%], 80[%], 100[%])
zerofill(2)
fft(1, TRUE, TRUE)

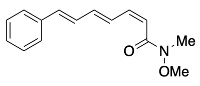

(*Z*)-18f ((2*Z*,4*E*,6*E*)-18f)

Filename = HT-1258-PTLC 1_HMBC_HMB
Author = delta
Experiment = hmbc.jxp
Sample_Id = HT-1258-PTLC 1_HMBC
Solvent = BENZENE-D6
Creation_Time = 30-JAN-2023 20:14:45
Revision_Time = 10-APR-2024 22:50:52
Current_Time = 10-APR-2024 22:51:37

Comment = gradient enhanced HMBC
Data_Format = 2D REAL REAL
Dim_Size = 1638, 512
Dim_Title = Proton Carbon13
Dim_Units = [ppm] [ppm]
Dimensions = X Y
Site = JNM-ECA500II
Spectrometer = DELTA2_NMR

Field_Strength = 11.7473579[T] (500[MHz]
X_Acq_Duration = 0.21823488[s]
X_Domain = 1H
X_Freq = 500.15991521[MHz]
X_Offset = 5.0[ppm]
X_Points = 2048
X_Prescans = 4
X_Resolution = 4.58221894[Hz]
X_Sweep = 9.38438438[kHz]
X_Sweep_Clipped = 7.50750751[kHz]
Y_Domain = 13C
Y_Freq = 125.76529768[MHz]
Y_Offset = 100[ppm]
Y_Points = 256
Y_Prescans = 0
Y_Resolution = 122.99275819[Hz]
Y_Sweep = 31.4861461[kHz]
Tri_Domain = Proton
Tri_Freq = 500.15991521[MHz]
Tri_Offset = 5.0[ppm]
Clipped = FALSE
Scans = 8
Total_Scans = 2048

Relaxation_Delay = 1.5[s]
Recvr_Gain = 50
Temp_Get = 19.4[dC]
X_Acq_Time = 0.21823488[s]
X_Atn = 3.2[dB]
X_Gamma = 42576375
X_Pulse = 7.68[us]

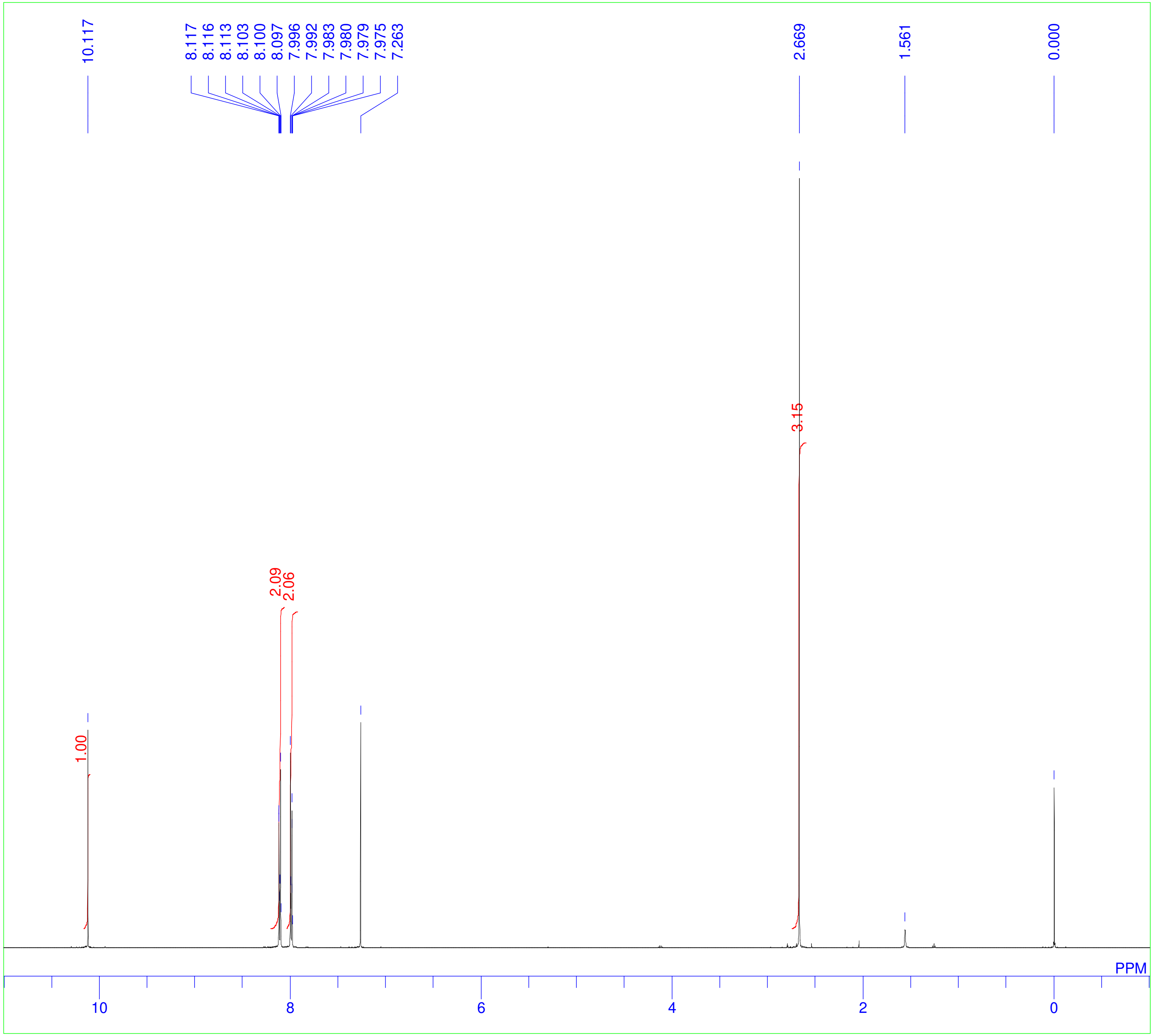

DFILE	19n_1H.als
COMNT	
DATIM	2023-04-10 17:31:13
OBNUC	1H
EXMOD	proton.jxp
OBFRQ	500.16 MHz
OBSET	2.41 KHz
OBFIN	6.01 Hz
POINT	13107
FREQU	7507.51 Hz
SCANS	8
ACQTM	1.7459 sec
PD	5.0000 sec
PW1	3.84 usec
IRNUC	1H
CTEMP	23.6 c
SLVNT	CDCL3
EXREF	0.00 ppm
BF	0.12 Hz
RGAIN	48

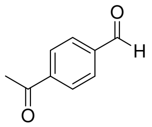

19n

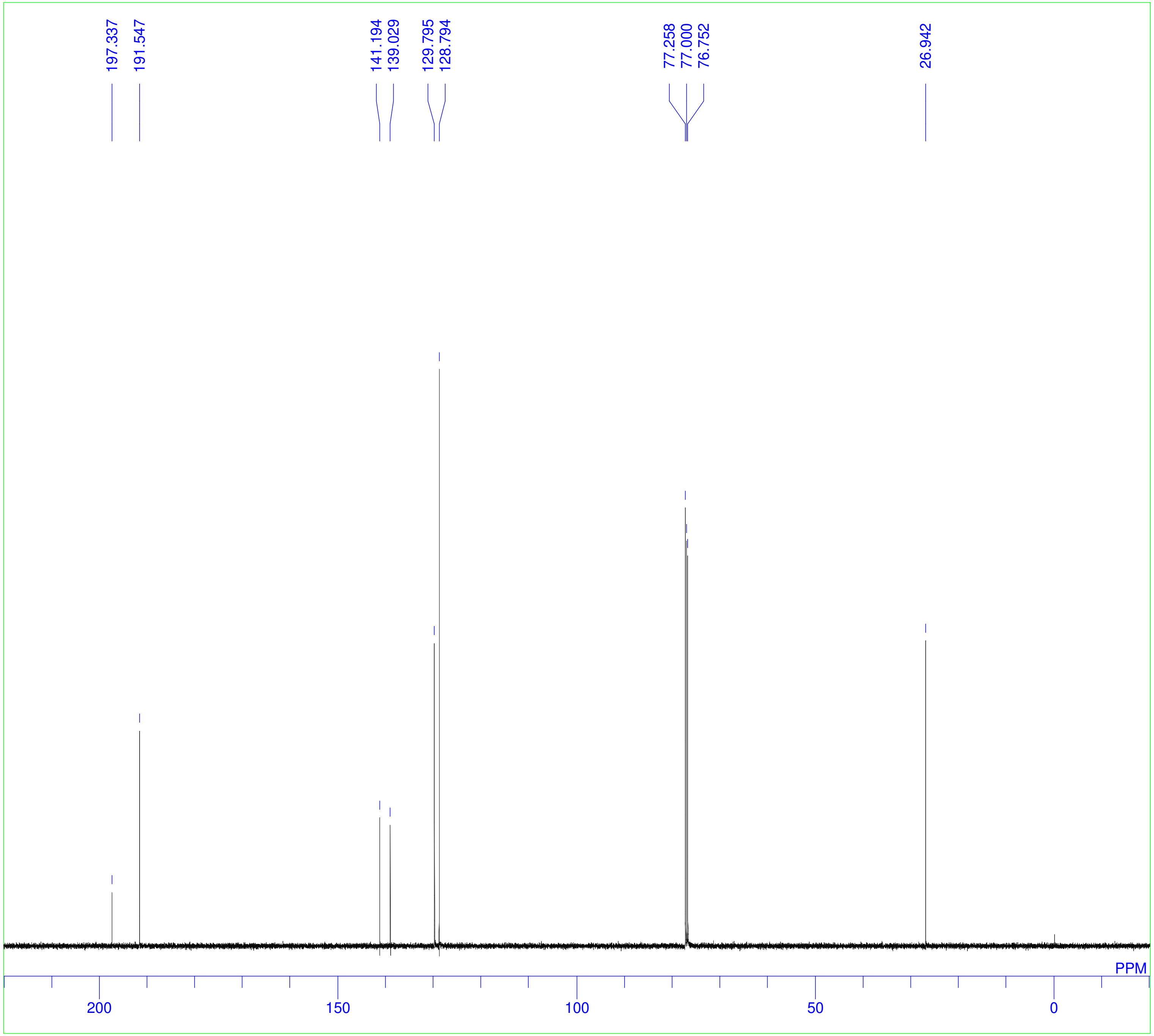

DFILE	19n_13C.als
COMNT	
DATIM	2023-04-17 20:57:49
OBNUC	13C
EXMOD	carbon.jxp
OBFRQ	125.77 MHz
OBSET	7.87 KHz
OBFIN	4.21 Hz
POINT	26214
FREQU	31446.54 Hz
SCANS	1024
ACQTM	0.8336 sec
PD	2.0000 sec
PW1	3.87 usec
IRNUC	1H
CTEMP	24.1 c
SLVNT	CDCL3
EXREF	77.00 ppm
BF	0.12 Hz
RGAIN	30

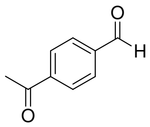

19n

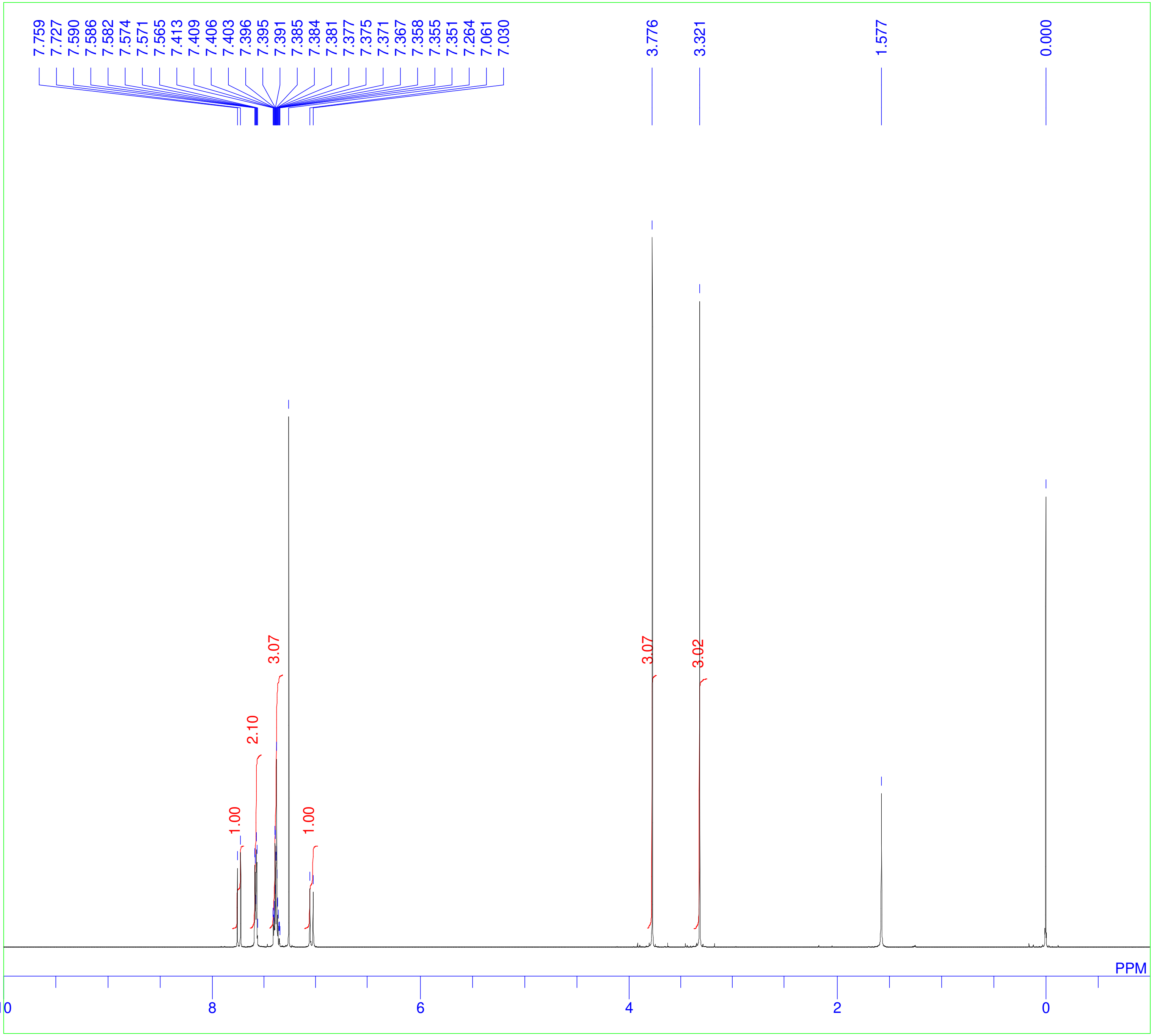

DFILE	(E)-20a_1H.als
COMNT	
DATIM	2022-01-02 13:40:06
OBNUC	1H
EXMOD	proton.jxp
OBFRQ	500.16 MHz
OBSET	2.41 KHz
OBFIN	6.01 Hz
POINT	13107
FREQU	7507.51 Hz
SCANS	8
ACQTM	1.7459 sec
PD	5.0000 sec
PW1	3.84 usec
IRNUC	1H
CTEMP	18.0 c
SLVNT	CDCL3
EXREF	0.00 ppm
BF	0.30 Hz
RGAIN	50

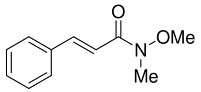

(*E*)-20a ((*E*)-5b)

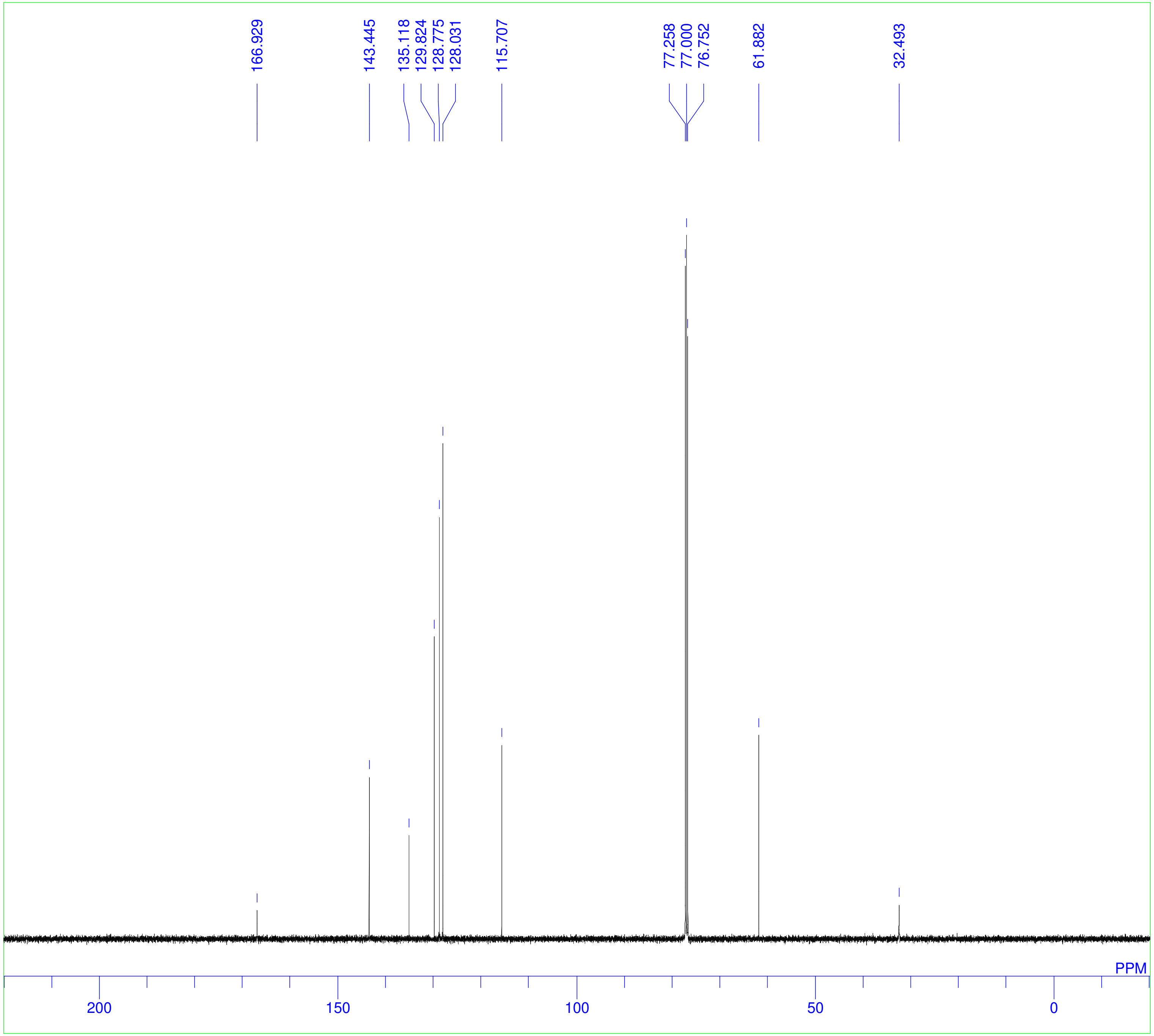

DFILE	(E)-20a_13C.als
COMNT	
DATIM	2022-02-07 19:04:54
OBNUC	13C
EXMOD	carbon.jxp
OBFRQ	125.77 MHz
OBSET	7.87 KHz
OBFIN	4.21 Hz
POINT	26214
FREQU	31446.54 Hz
SCANS	1792
ACQTM	0.8336 sec
PD	2.0000 sec
PW1	3.87 usec
IRNUC	1H
CTEMP	19.3 c
SLVNT	CDCL3
EXREF	77.00 ppm
BF	0.30 Hz
RGAIN	28

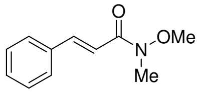

(*E*)-20a ((*E*)-5b)

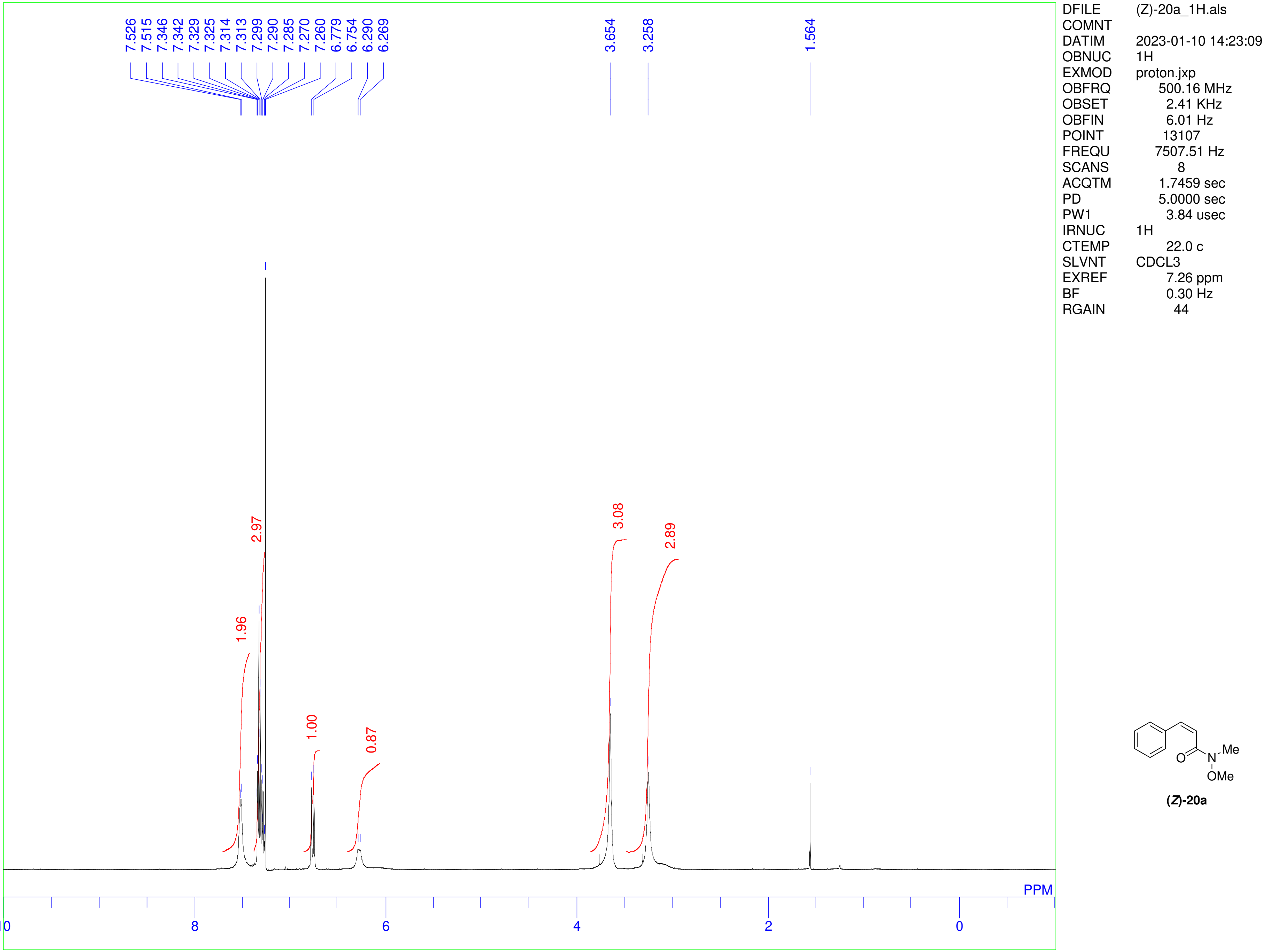
7.526
7.515
7.346
7.342
7.329
7.325
7.314
7.313
7.299
7.290
7.285
7.270
7.260
6.779
6.754
6.290
6.269
3.654
3.258
1.564
1.96
2.97
1.00
0.87
3.08
2.89
PPM
10
8
6
4
2
0
DFILE (Z)-20a_1H.als
COMNT
DATIM 2023-01-10 14:23:09
OBNUC 1H
EXMOD proton.jxp
OBFRQ 500.16 MHz
OBSET 2.41 KHz
OBFIN 6.01 Hz
POINT 13107
FREQU 7507.51 Hz
SCANS 8
ACQTM 1.7459 sec
PD 5.0000 sec
PW1 3.84 usec
IRNUC 1H
CTEMP 22.0 c
SLVNT CDCL3
EXREF 7.26 ppm
BF 0.30 Hz
RGAIN 44
O
N
Me
OMe

(*Z*)-20a

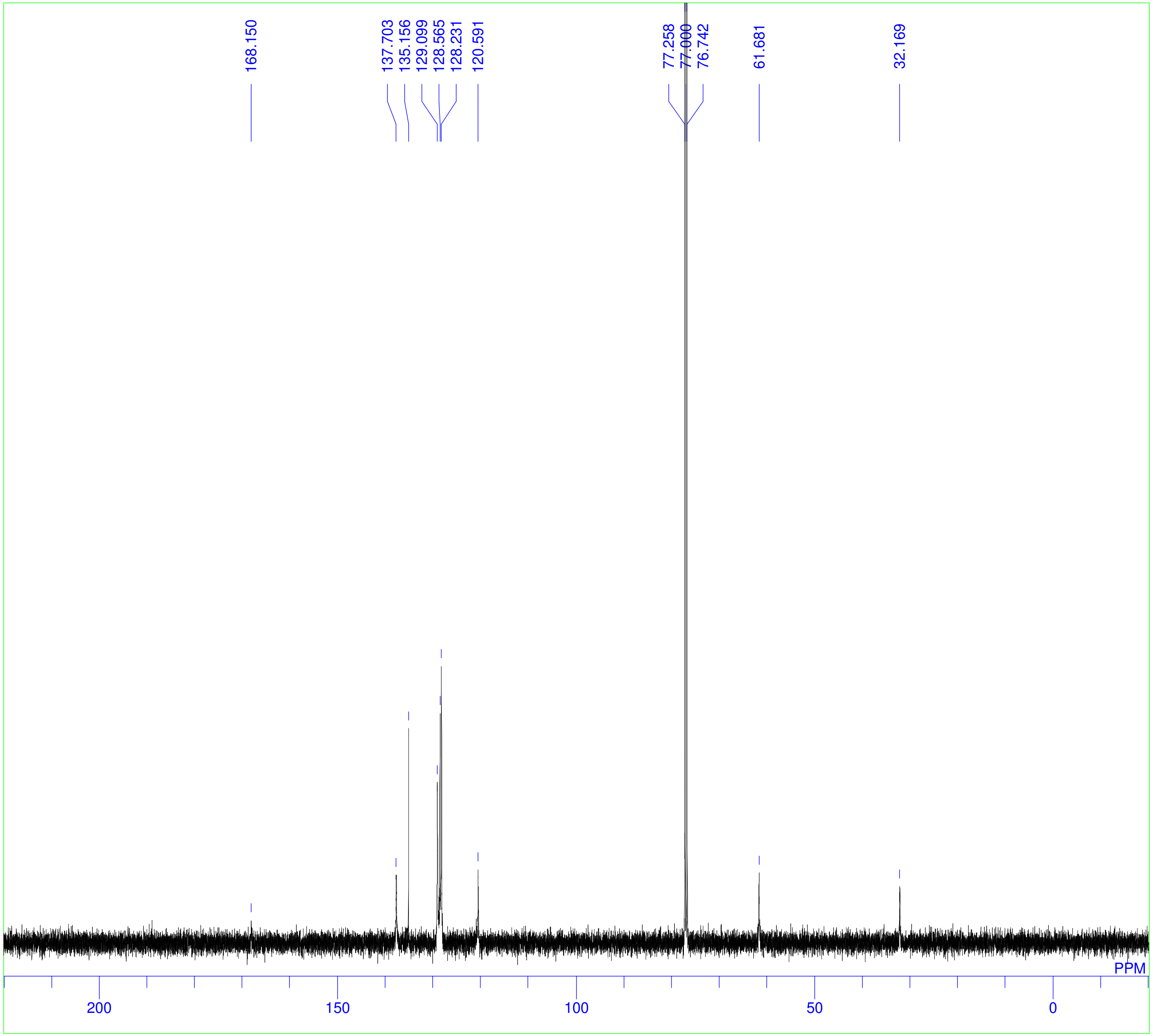

DFILE (Z)-20a_13C.als
COMNT
DATIM 2023-01-10 15:41:22
OBNUC 13C
EXMOD carbon.jxp
OBFRQ 125.77 MHz
OBSET 7.87 KHz
OBFIN 4.21 Hz
POINT 26214
FREQU 31446.54 Hz
SCANS 1024
ACQTM 0.8336 sec
PD 2.0000 sec
PW1 3.87 usec
IRNUC 1H
CTEMP 21.9 c
SLVNT CDCL3
EXREF 77.00 ppm
BF 0.30 Hz
RGAIN 24

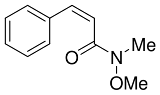

(*Z*)-20a

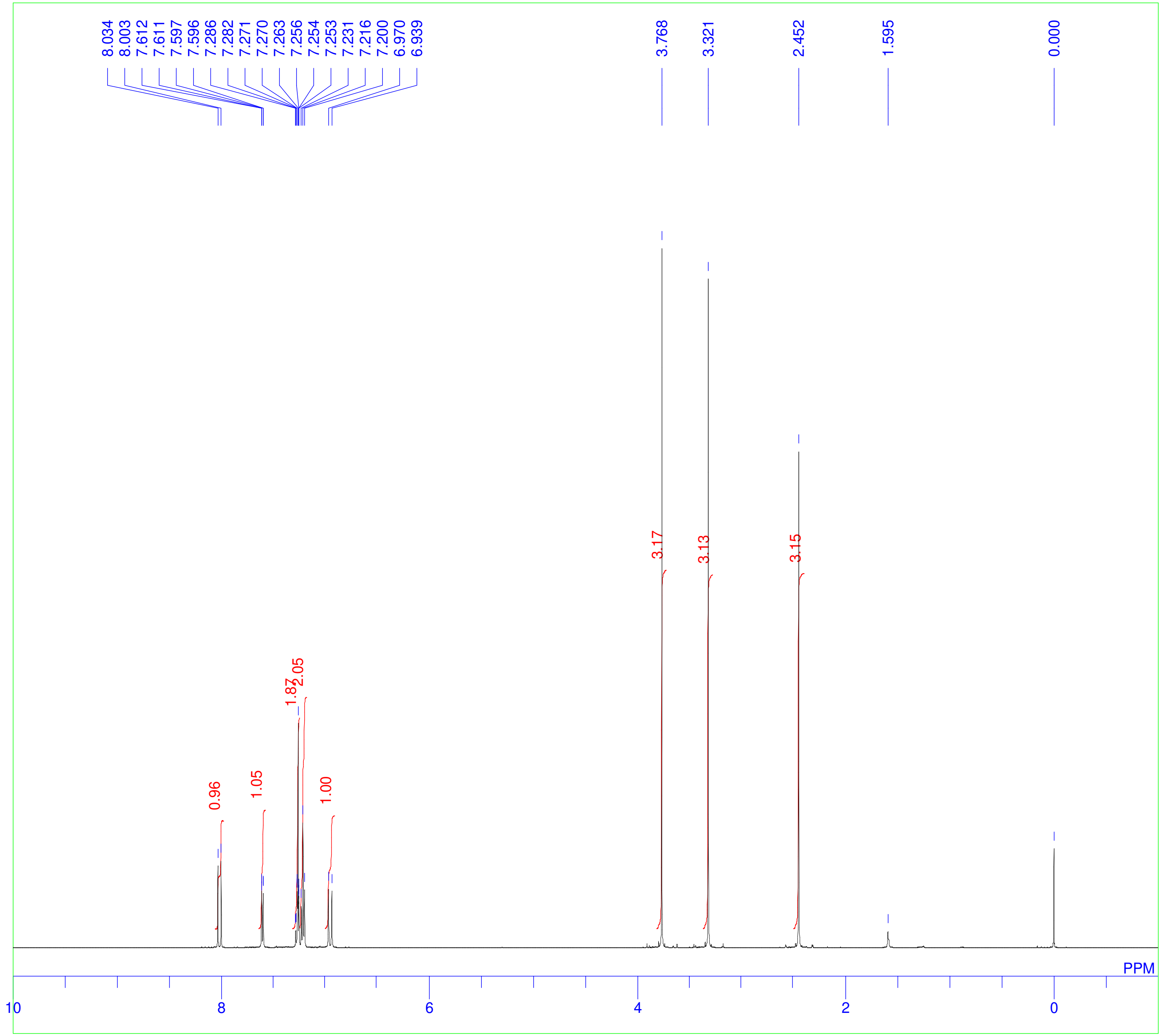

DFILE (E)-20b_1H.als
COMNT
DATIM 2022-01-09 17:00:53
OBNUC 1H
EXMOD proton.jxp
OBFRQ 500.16 MHz
OBSET 2.41 KHz
OBFIN 6.01 Hz
POINT 13107
FREQU 7507.51 Hz
SCANS 8
ACQTM 1.7459 sec
PD 5.0000 sec
PW1 3.84 usec
IRNUC 1H
CTEMP 18.8 c
SLVNT CDCL3
EXREF 0.00 ppm
BF 0.30 Hz
RGAIN 42

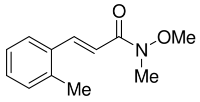

(*E*)-20b

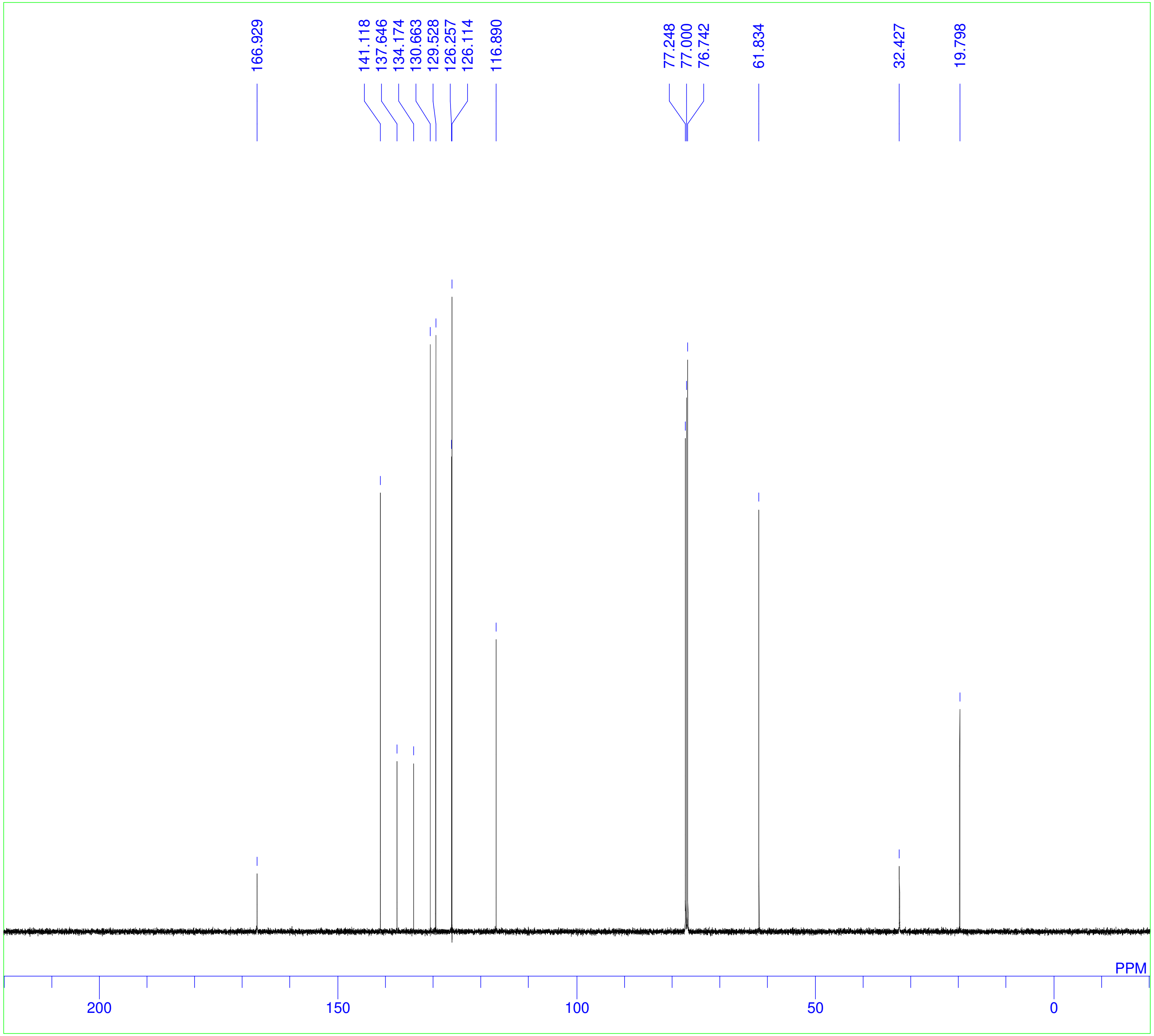

DFILE (E)-20b_13C.als
COMNT
DATIM 2023-01-06 10:40:24
OBNUC 13C
EXMOD carbon.jxp
OBFRQ 125.77 MHz
OBSET 7.87 KHz
OBFIN 4.21 Hz
POINT 26214
FREQU 31446.54 Hz
SCANS 1024
ACQTM 0.8336 sec
PD 2.0000 sec
PW1 3.87 usec
IRNUC 1H
CTEMP 20.3 c
SLVNT CDCL3
EXREF 77.00 ppm
BF 0.30 Hz
RGAIN 24

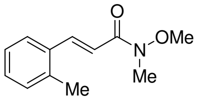

(*E*)-20b

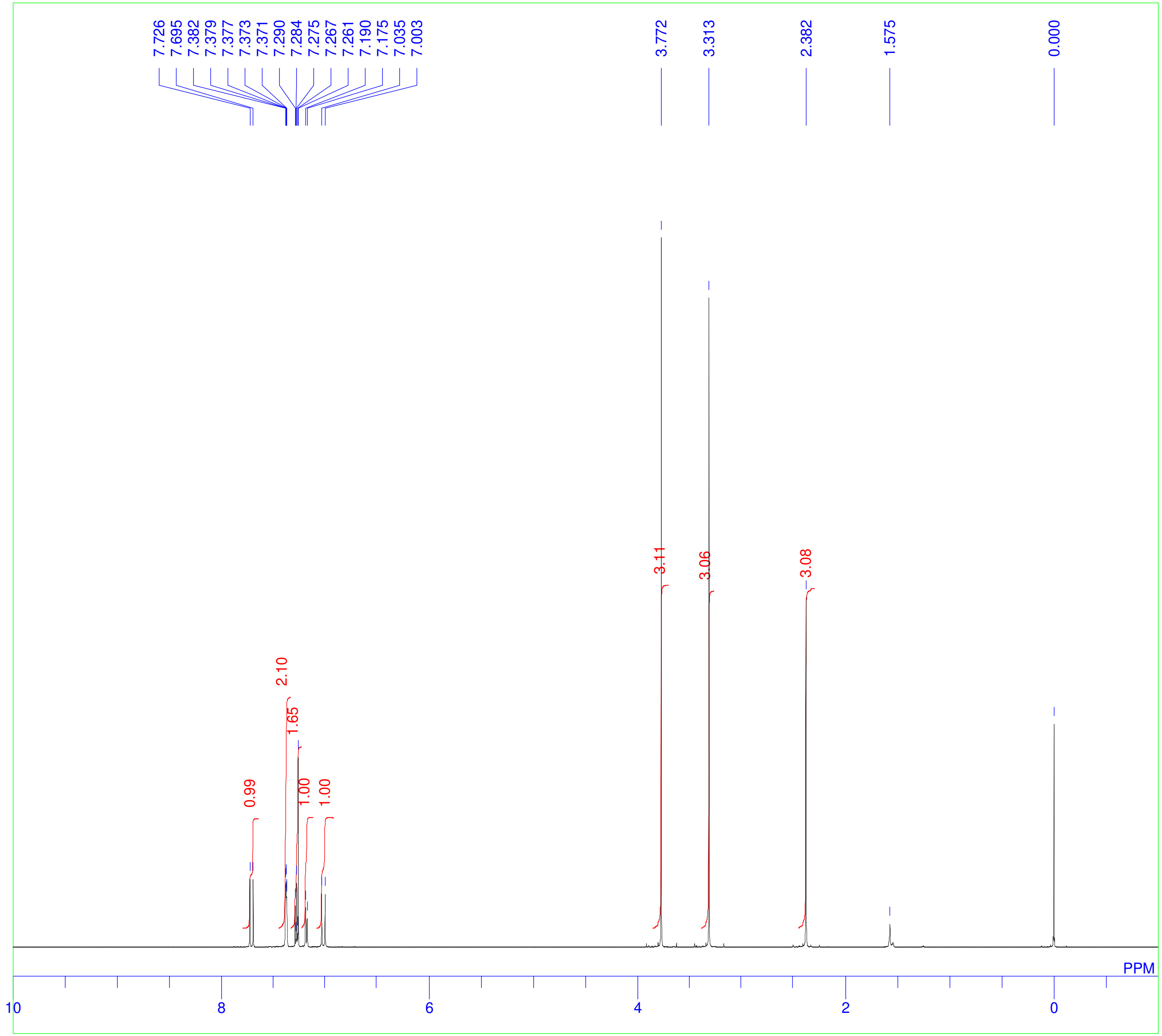

DFILE	(E)-20c_1H.als
COMNT	
DATIM	2023-12-15 08:59:05
OBNUC	1H
EXMOD	proton.jxp
OBFRQ	500.16 MHz
OBSET	2.41 KHz
OBFIN	6.01 Hz
POINT	13107
FREQU	7507.51 Hz
SCANS	8
ACQTM	1.7459 sec
PD	5.0000 sec
PW1	3.80 usec
IRNUC	1H
CTEMP	23.5 c
SLVNT	CDCL3
EXREF	0.00 ppm
BF	0.12 Hz
RGAIN	42

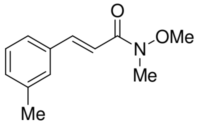

(*E*)-20c

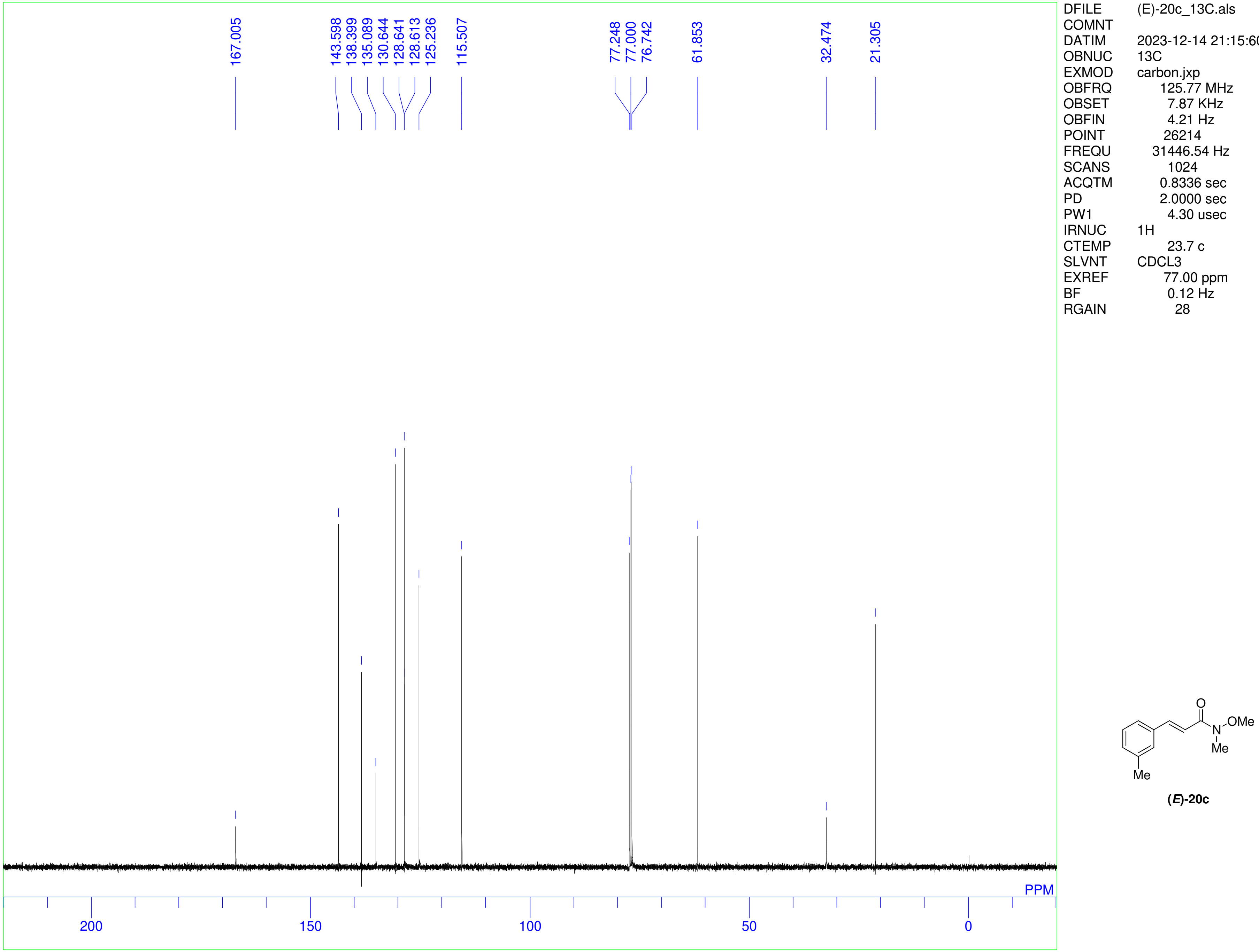

(*E*)-20c

DFILE	(E)-20c_13C.als
COMNT	
DATIM	2023-12-14 21:15:60
OBNUC	13C
EXMOD	carbon.jxp
OBFRQ	125.77 MHz
OBSET	7.87 KHz
OBFIN	4.21 Hz
POINT	26214
FREQU	31446.54 Hz
SCANS	1024
ACQTM	0.8336 sec
PD	2.0000 sec
PW1	4.30 usec
IRNUC	1H
CTEMP	23.7 c
SLVNT	CDCL3
EXREF	77.00 ppm
BF	0.12 Hz
RGAIN	28

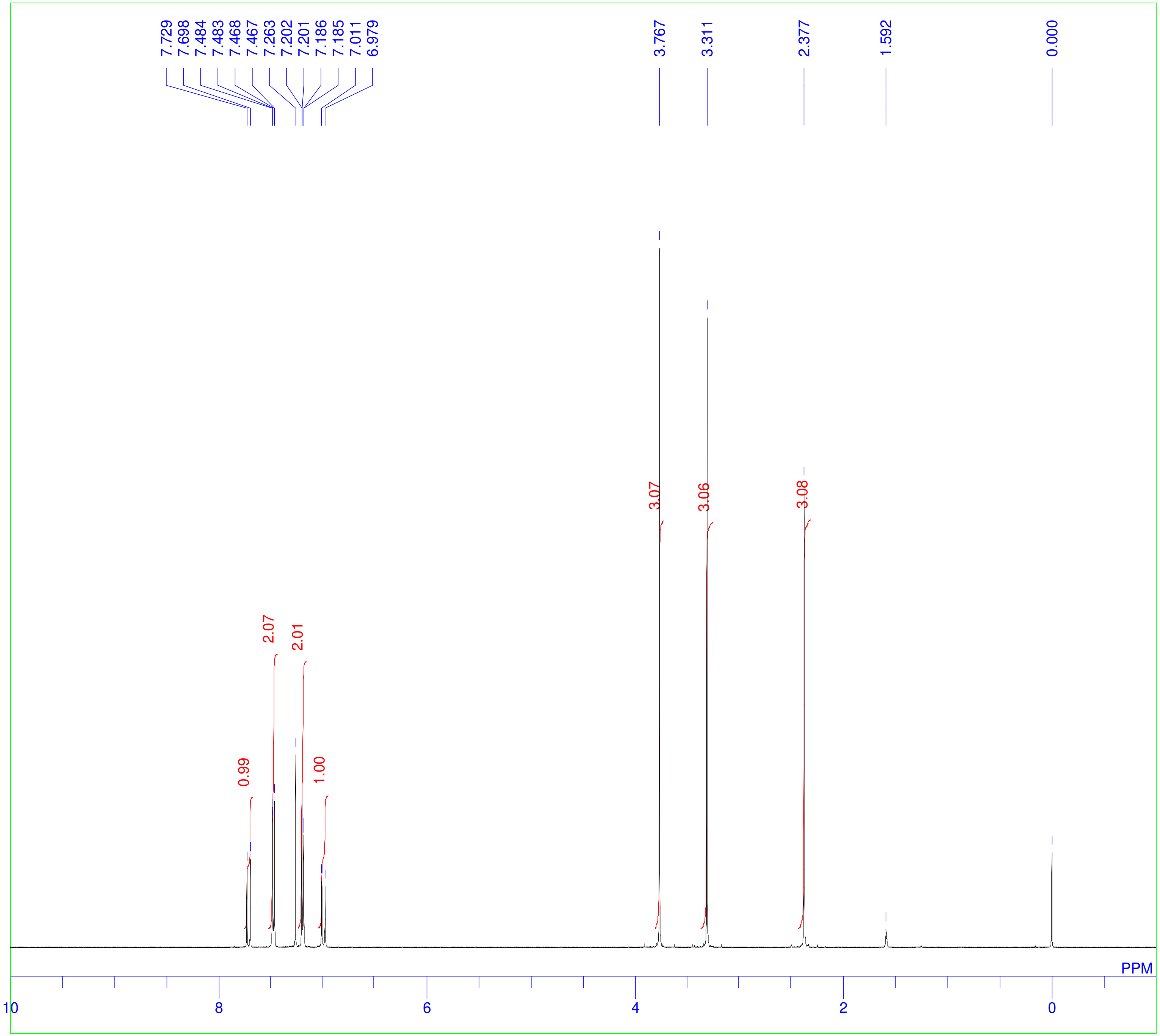

DFILE	(E)-20d_1H.als
COMNT	
DATIM	2022-01-09 17:07:17
OBNUC	1H
EXMOD	proton.jxp
OBFRQ	500.16 MHz
OBSET	2.41 KHz
OBFIN	6.01 Hz
POINT	13107
FREQU	7507.51 Hz
SCANS	8
ACQTM	1.7459 sec
PD	5.0000 sec
PW1	3.84 usec
IRNUC	1H
CTEMP	18.8 c
SLVNT	CDCL3
EXREF	0.00 ppm
BF	0.30 Hz
RGAIN	42

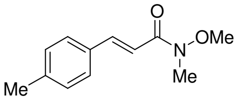

(*E*)-20d

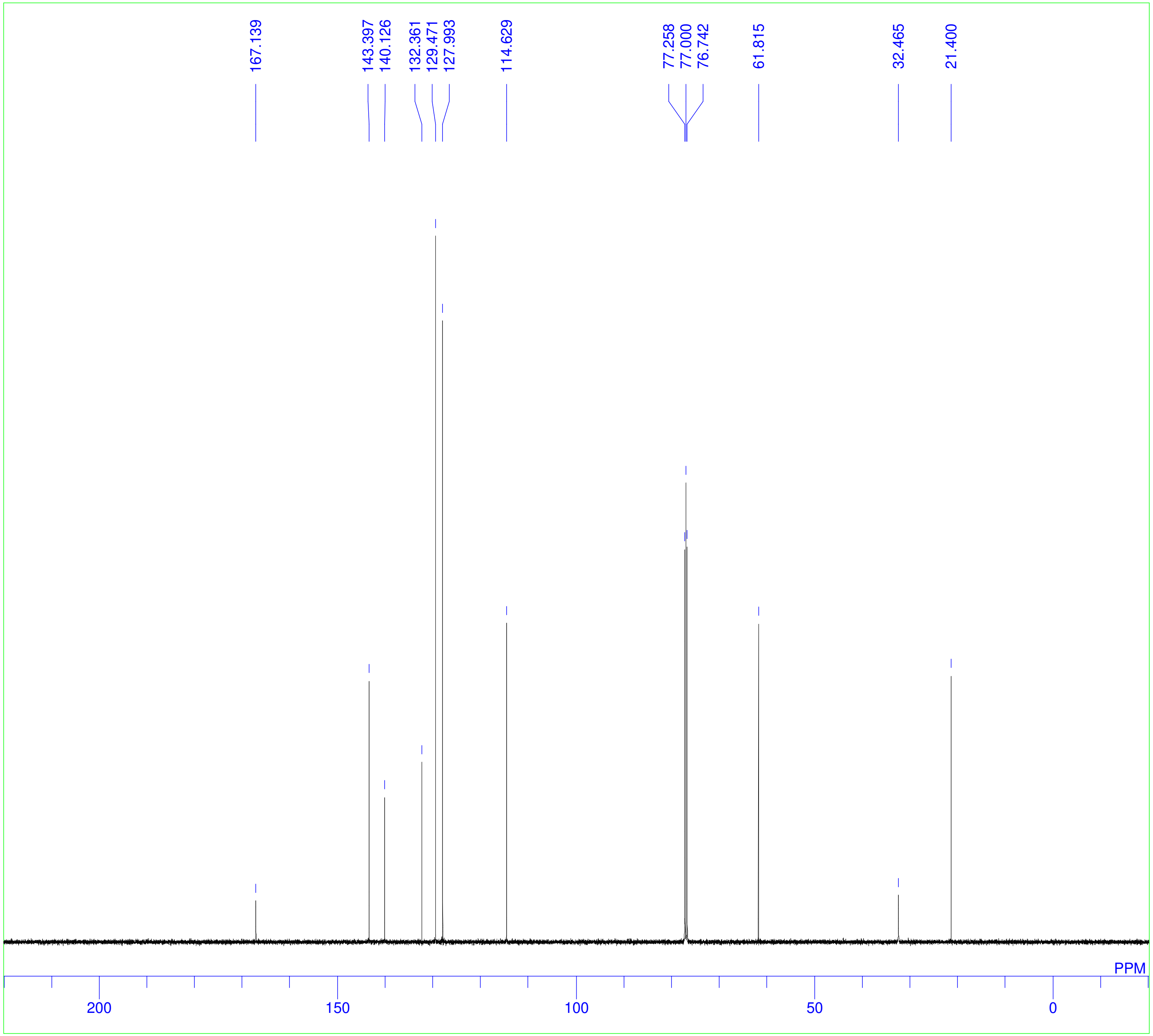

DFILE	(E)-20d_13C.als
COMNT	
DATIM	2023-01-07 09:35:53
OBNUC	13C
EXMOD	carbon.jxp
OBFRQ	125.77 MHz
OBSET	7.87 KHz
OBFIN	4.21 Hz
POINT	26214
FREQU	31446.54 Hz
SCANS	1024
ACQTM	0.8336 sec
PD	2.0000 sec
PW1	3.87 usec
IRNUC	1H
CTEMP	20.6 c
SLVNT	CDCL3
EXREF	77.00 ppm
BF	0.30 Hz
RGAIN	26

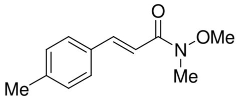

(*E*)-20d

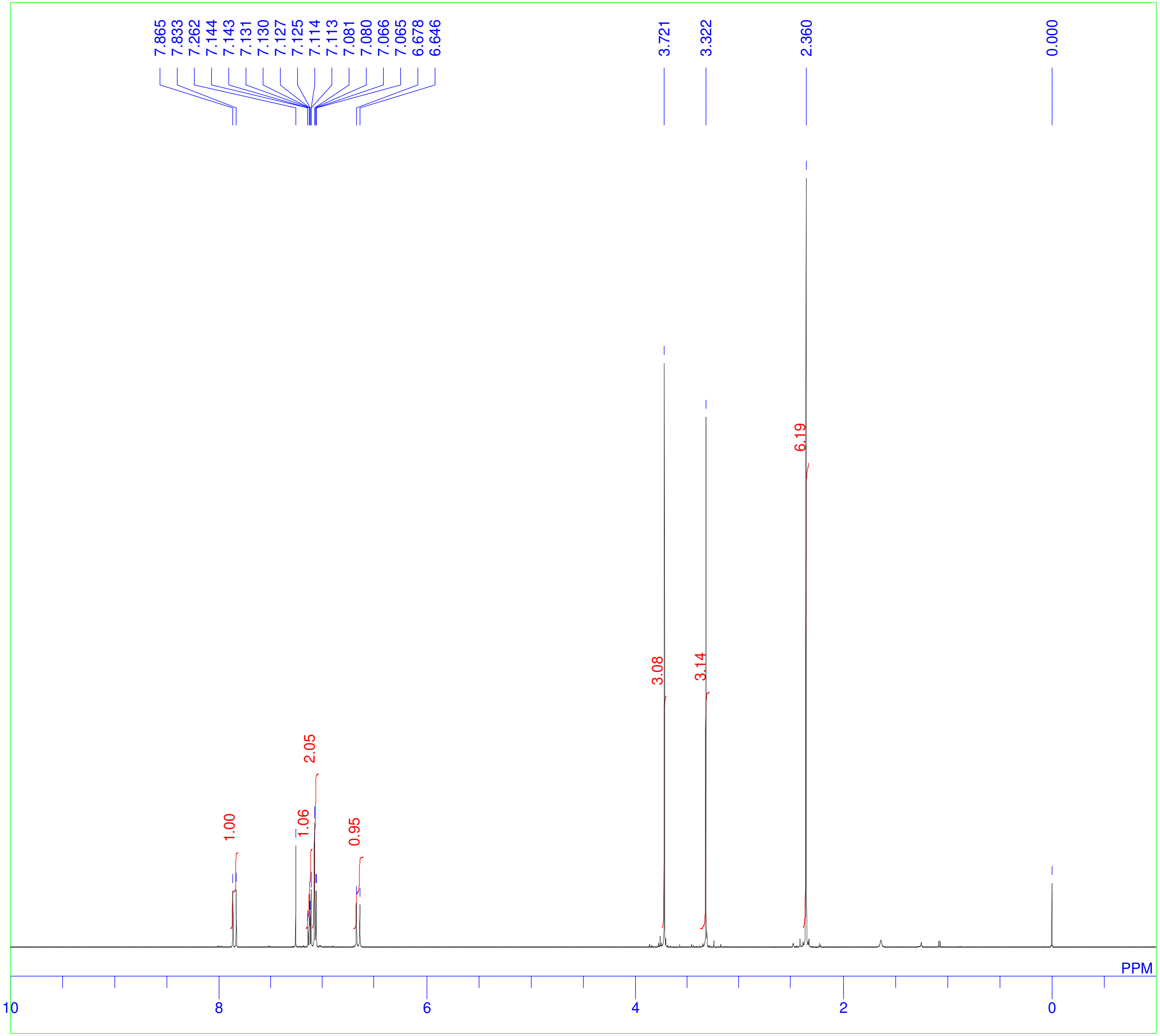

DFILE (E)-20e_1H.als
COMNT
DATIM 2022-02-12 19:39:40
OBNUC 1H
EXMOD proton.jxp
OBFRQ 500.16 MHz
OBSET 2.41 KHz
OBFIN 6.01 Hz
POINT 13107
FREQU 7507.51 Hz
SCANS 8
ACQTM 1.7459 sec
PD 5.0000 sec
PW1 3.84 usec
IRNUC 1H
CTEMP 18.6 c
SLVNT CDCL3
EXREF 0.00 ppm
BF 0.30 Hz
RGAIN 46

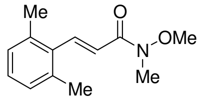

(*E*)-20e

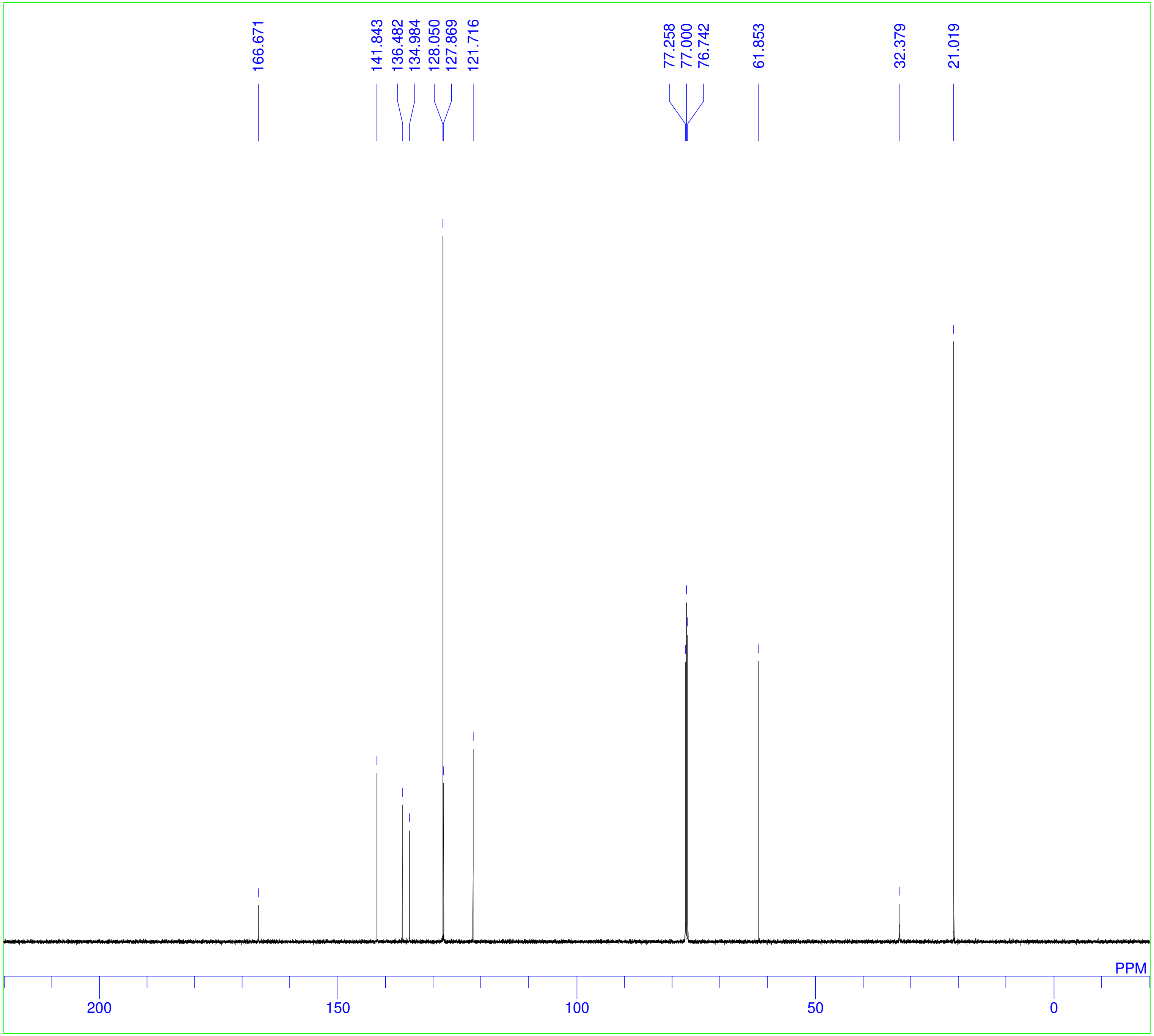

DFILE (E)-20e_13C.als
COMNT
DATIM 2022-03-04 12:11:54
OBNUC 13C
EXMOD carbon.jxp
OBFRQ 125.77 MHz
OBSET 7.87 KHz
OBFIN 4.21 Hz
POINT 26214
FREQU 31446.54 Hz
SCANS 1024
ACQTM 0.8336 sec
PD 2.0000 sec
PW1 3.87 usec
IRNUC 1H
CTEMP 20.7 c
SLVNT CDCL3
EXREF 77.00 ppm
BF 0.30 Hz
RGAIN 26

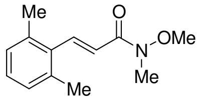

(*E*)-20e

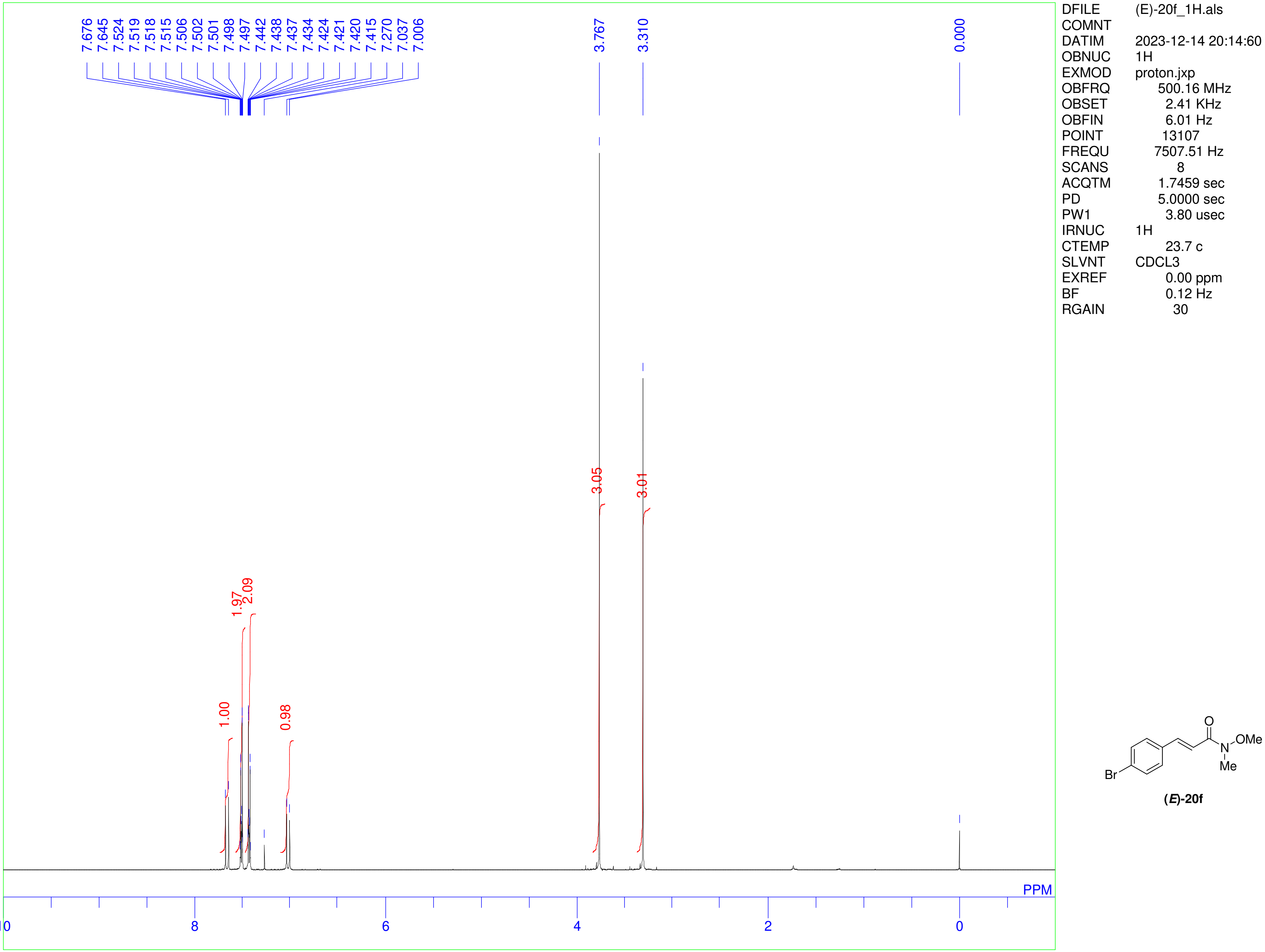
7.676
7.645
7.524
7.519
7.518
7.515
7.506
7.502
7.501
7.498
7.497
7.442
7.438
7.437
7.434
7.424
7.421
7.420
7.415
7.270
7.037
7.006
3.767
3.310
0.000
1.00
1.97
2.09
0.98
3.05
3.01
PPM
10
8
6
4
2
0
DFILE (E)-20f_1H.als
COMNT
DATIM 2023-12-14 20:14:60
OBNUC 1H
EXMOD proton.jxp
OBFRQ 500.16 MHz
OBSET 2.41 KHz
OBFIN 6.01 Hz
POINT 13107
FREQU 7507.51 Hz
SCANS 8
ACQTM 1.7459 sec
PD 5.0000 sec
PW1 3.80 usec
IRNUC 1H
CTEMP 23.7 c
SLVNT CDCL3
EXREF 0.00 ppm
BF 0.12 Hz
RGAIN 30
O
N
OMe
Me
Br

(*E*)-20f

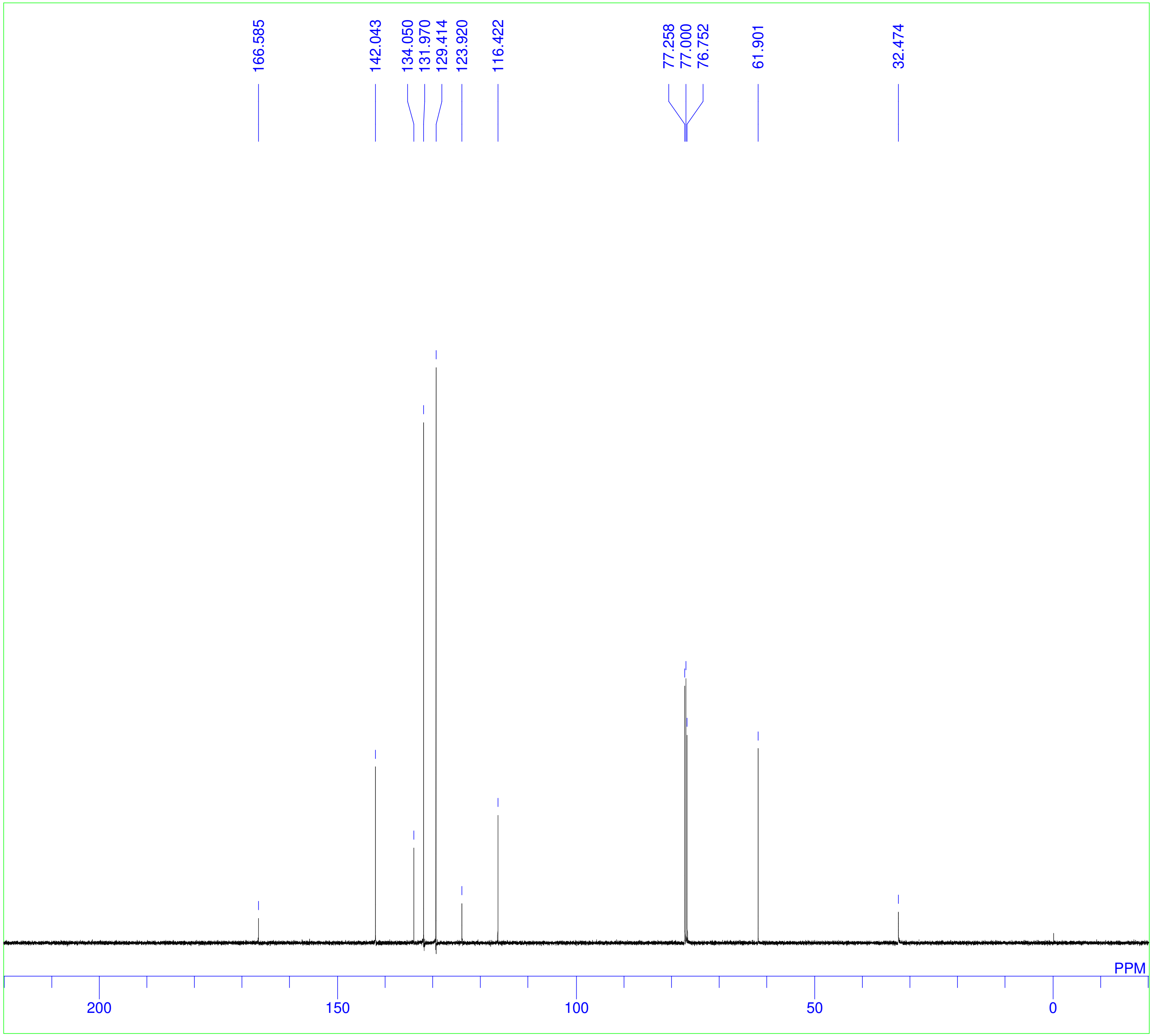

DFILE	(E)-20f_13C.als
COMNT	
DATIM	2023-12-14 20:16:37
OBNUC	13C
EXMOD	carbon.jxp
OBFRQ	125.77 MHz
OBSET	7.87 KHz
OBFIN	4.21 Hz
POINT	26214
FREQU	31446.54 Hz
SCANS	1024
ACQTM	0.8336 sec
PD	2.0000 sec
PW1	4.30 usec
IRNUC	1H
CTEMP	23.8 c
SLVNT	CDCL3
EXREF	77.00 ppm
BF	0.12 Hz
RGAIN	30

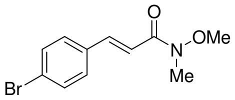

(*E*)-20f

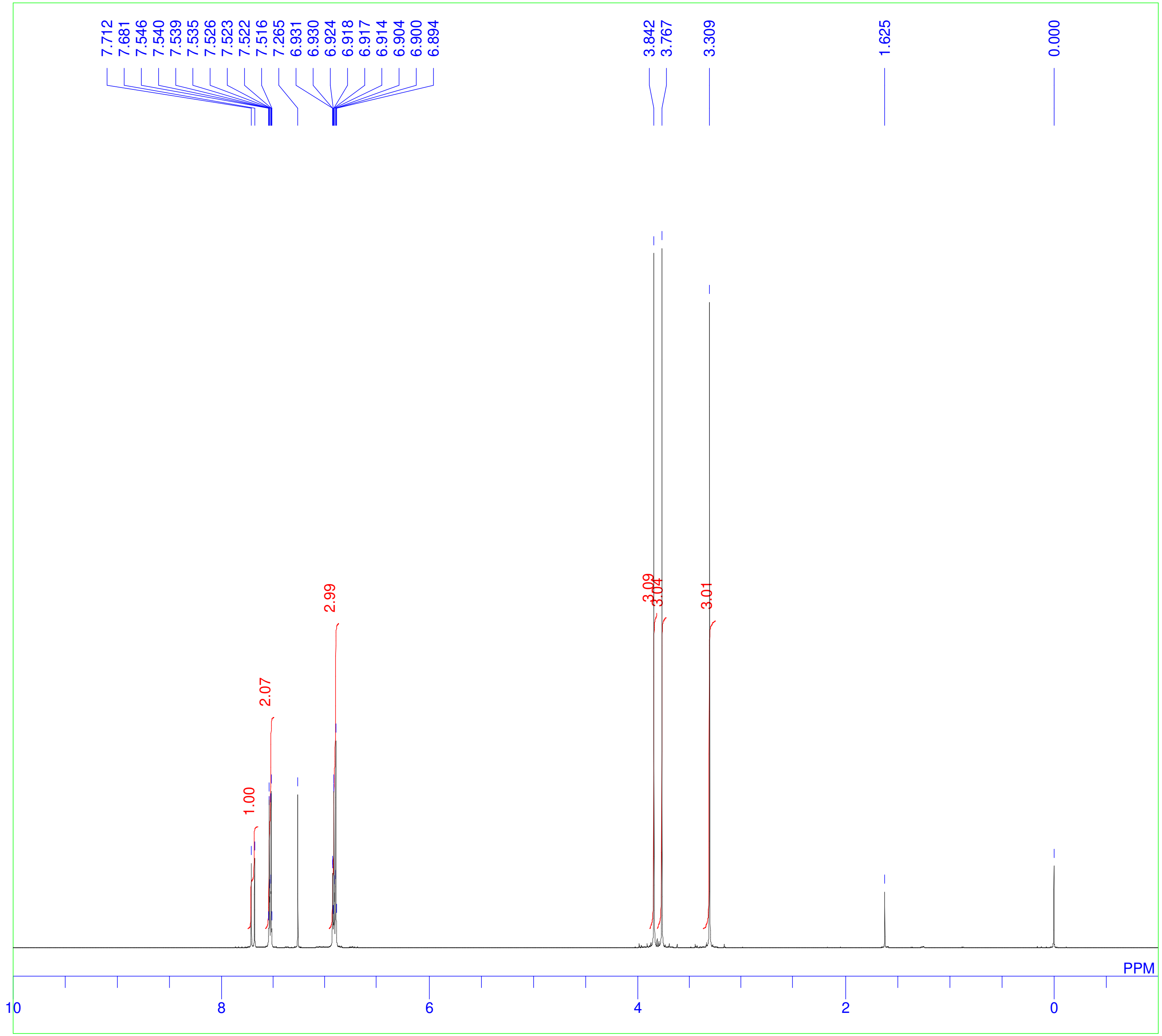

DFILE (E)-20g_1H.als
COMNT
DATIM 2022-01-08 17:17:26
OBNUC 1H
EXMOD proton.jxp
OBFRQ 500.16 MHz
OBSET 2.41 KHz
OBFIN 6.01 Hz
POINT 13107
FREQU 7507.51 Hz
SCANS 8
ACQTM 1.7459 sec
PD 5.0000 sec
PW1 3.84 usec
IRNUC 1H
CTEMP 18.5 c
SLVNT CDCL3
EXREF 0.00 ppm
BF 0.30 Hz
RGAIN 40

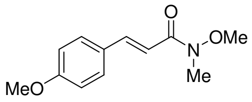

(*E*)-20g

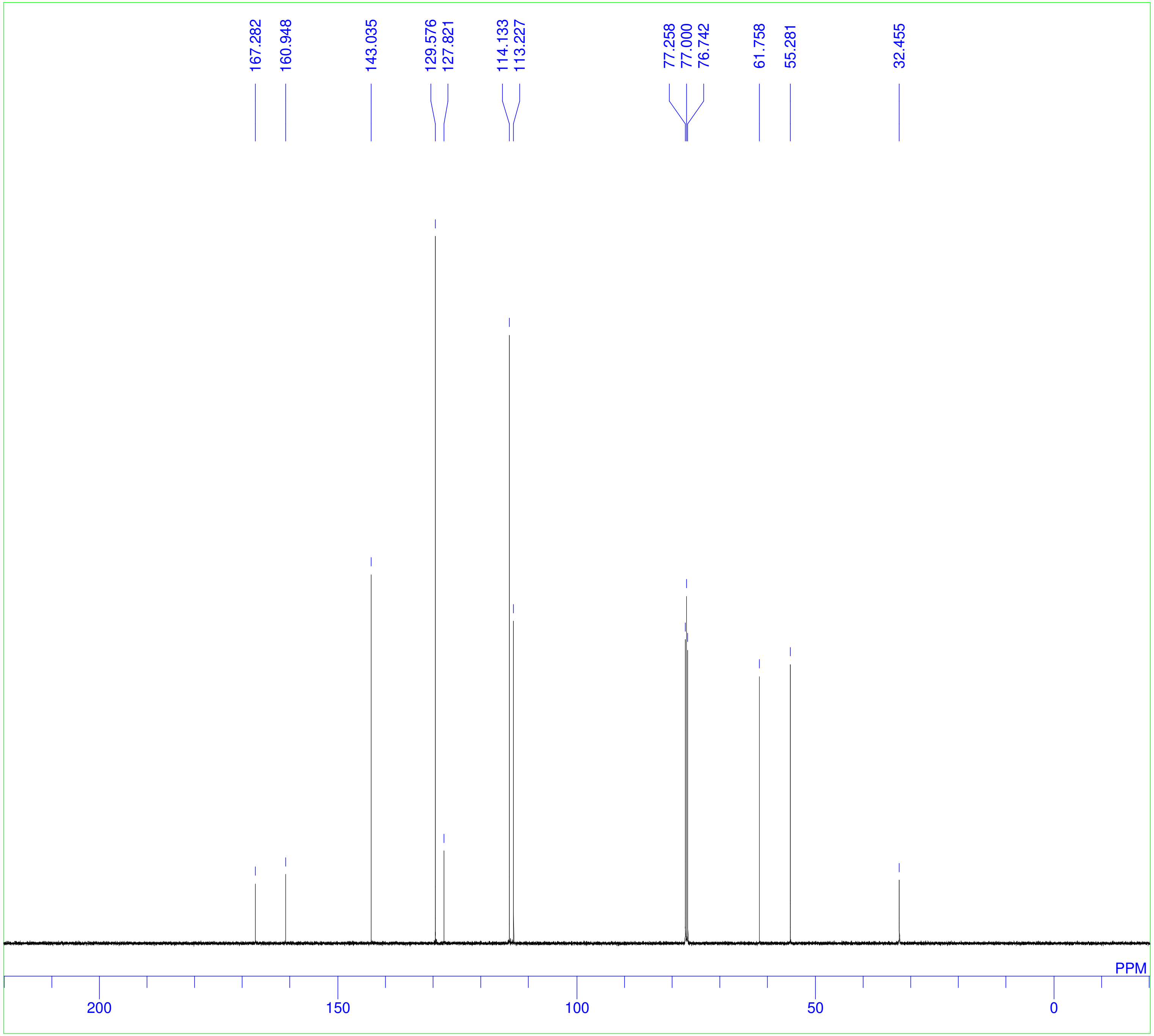

DFILE	(E)-20g_13C.als
COMNT	
DATIM	2023-01-06 15:16:48
OBNUC	13C
EXMOD	carbon.jxp
OBFRQ	125.77 MHz
OBSET	7.87 KHz
OBFIN	4.21 Hz
POINT	26214
FREQU	31446.54 Hz
SCANS	1024
ACQTM	0.8336 sec
PD	2.0000 sec
PW1	3.87 usec
IRNUC	1H
CTEMP	20.9 c
SLVNT	CDCL3
EXREF	77.00 ppm
BF	0.30 Hz
RGAIN	24

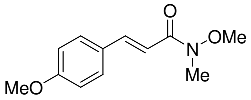

(*E*)-20g

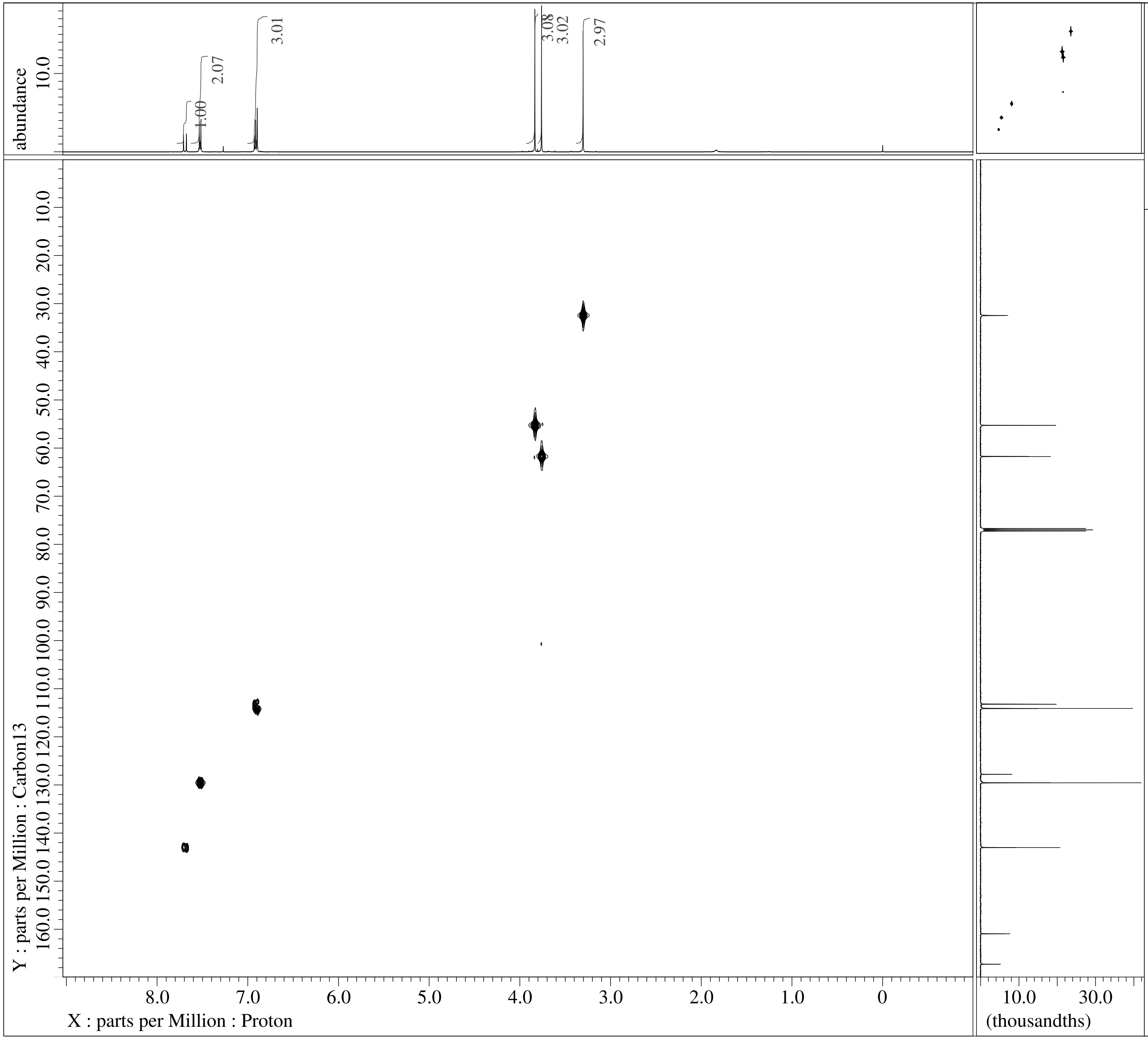


```
---- PROCESSING PARAMETERS ----
sinbell4( -60, 160 )
zerofill( 1 )
fft( 1, TRUE, TRUE )
ppm
[transpose]
sinbell4( -60, 160 )
zerofill( 2 )
fft( 1, TRUE, TRUE )
ppm
abs
```

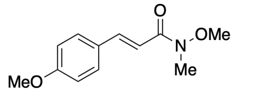

(*E*)-20g

```
Filename            = HT-4-v_HMQC_HMQC-1-5.jd
Author              = delta
Experiment          = hmqc.jxp
Sample_Id           = HT-4-v_HMQC
Solvent             = CHLOROFORM-D
Creation_Time       = 10-JAN-2024 11:01:38
Revision_Time       = 10-APR-2024 22:54:38
Current_Time        = 10-APR-2024 22:55:13

Comment             = gradient enhanced HMQC
Data_Format         = 2D REAL REAL
Dim_Size            = 819, 512
Dim_Title           = Proton Carbon13
Dim_Units           = [ppm] [ppm]
Dimensions          = X Y
Site                = JNM-ECA500II
Spectrometer        = DELTA2_NMR

Field_Strength      = 11.7473579[T] (500[MHz]
X_Acq_Duration      = 0.15998976[s]
X_Domain            = 1H
X_Freq              = 500.15991521[MHz]
X_Offset            = 3.90462723[ppm]
X_Points            = 1024
X_Prescans          = 4
X_Resolution        = 6.25040003[Hz]
X_Sweep             = 6.40040963[kHz]
X_Sweep_Clipped     = 5.1203277[kHz]
Y_Domain            = 13C
Y_Freq              = 125.76529768[MHz]
Y_Offset            = 85[ppm]
Y_Points            = 256
Y_Prescans          = 0
Y_Resolution        = 83.60980308[Hz]
Y_Sweep             = 21.40410959[kHz]
Tri_Domain          = Proton
Tri_Freq            = 500.15991521[MHz]
Tri_Offset          = 5.0[ppm]
Clipped             = FALSE
Scans               = 8
Total_Scans         = 2048

Relaxation_Delay    = 1.5[s]
Recvr_Gain          = 50
Temp_Get            = 21.3[dC]
X_Acq_Time          = 0.15998976[s]
X_Atn               = 3.2[dB]
X_Gamma             = 42576375
X_Pulse             = 7.59[us]
```

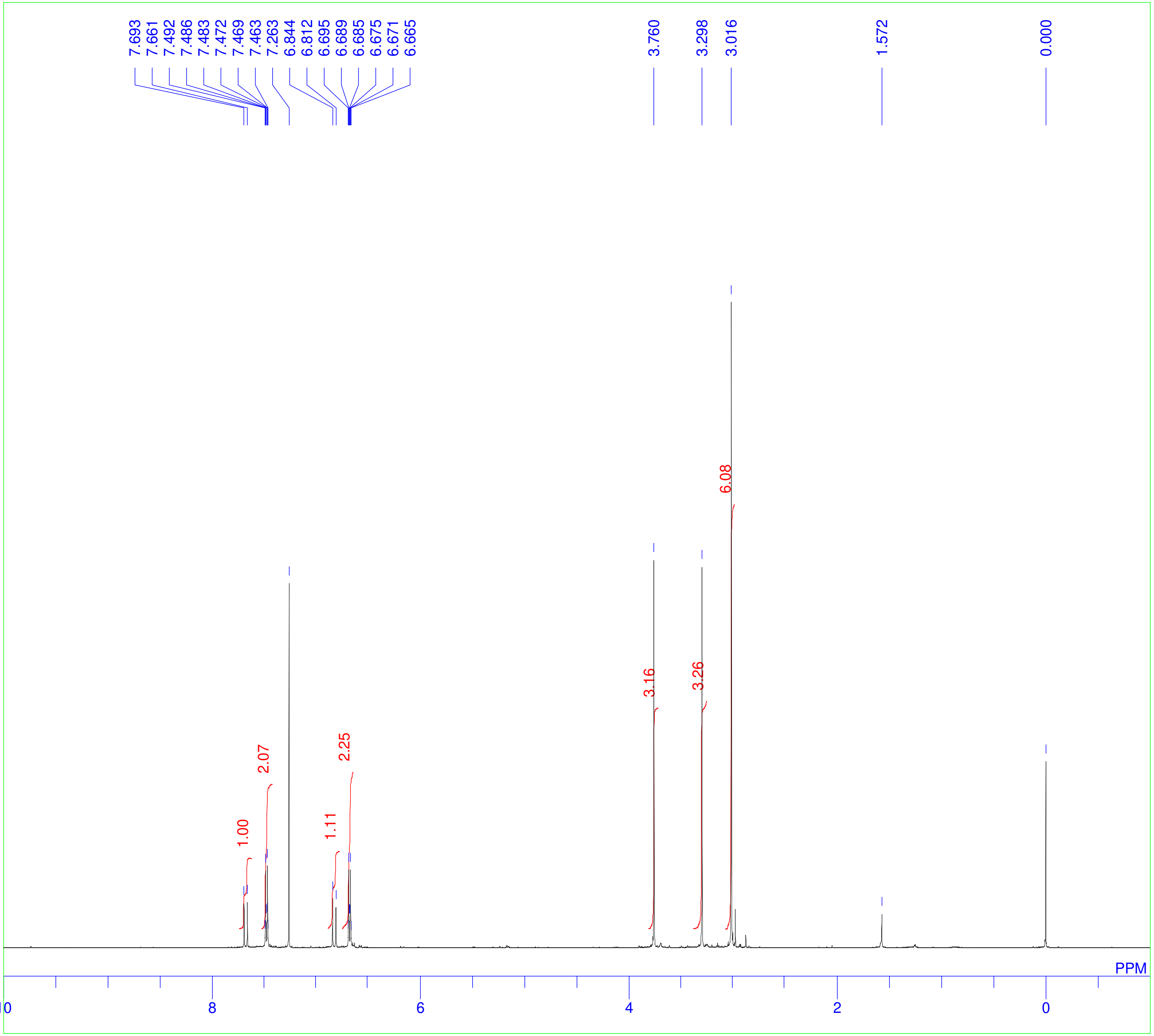

DFILE	(E)-20h_1H.als
COMNT	
DATIM	2023-01-29 19:51:59
OBNUC	1H
EXMOD	proton.jxp
OBFRQ	500.16 MHz
OBSET	2.41 KHz
OBFIN	6.01 Hz
POINT	13107
FREQU	7507.51 Hz
SCANS	8
ACQTM	1.7459 sec
PD	5.0000 sec
PW1	3.84 usec
IRNUC	1H
CTEMP	19.0 c
SLVNT	CDCL3
EXREF	0.00 ppm
BF	0.30 Hz
RGAIN	44

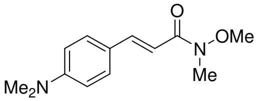

(*E*)-20h

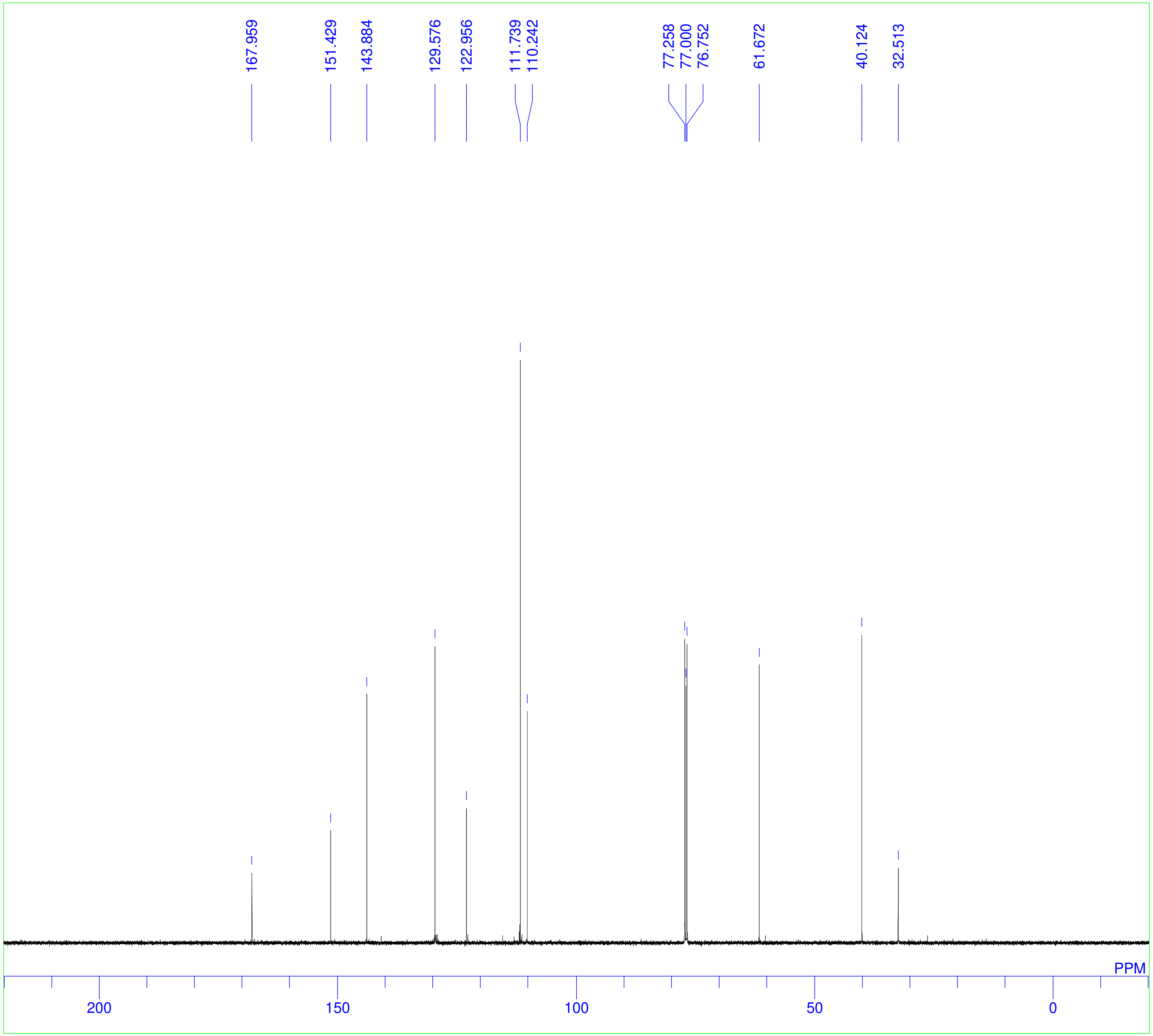

DFILE	(E)-20h_13C.als
COMNT	
DATIM	2023-01-29 20:00:47
OBNUC	13C
EXMOD	carbon.jxp
OBFRQ	125.77 MHz
OBSET	7.87 KHz
OBFIN	4.21 Hz
POINT	26214
FREQU	31446.54 Hz
SCANS	1024
ACQTM	0.8336 sec
PD	2.0000 sec
PW1	3.87 usec
IRNUC	1H
CTEMP	19.1 c
SLVNT	CDCL3
EXREF	77.00 ppm
BF	0.30 Hz
RGAIN	24

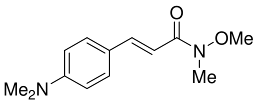

(*E*)-20h

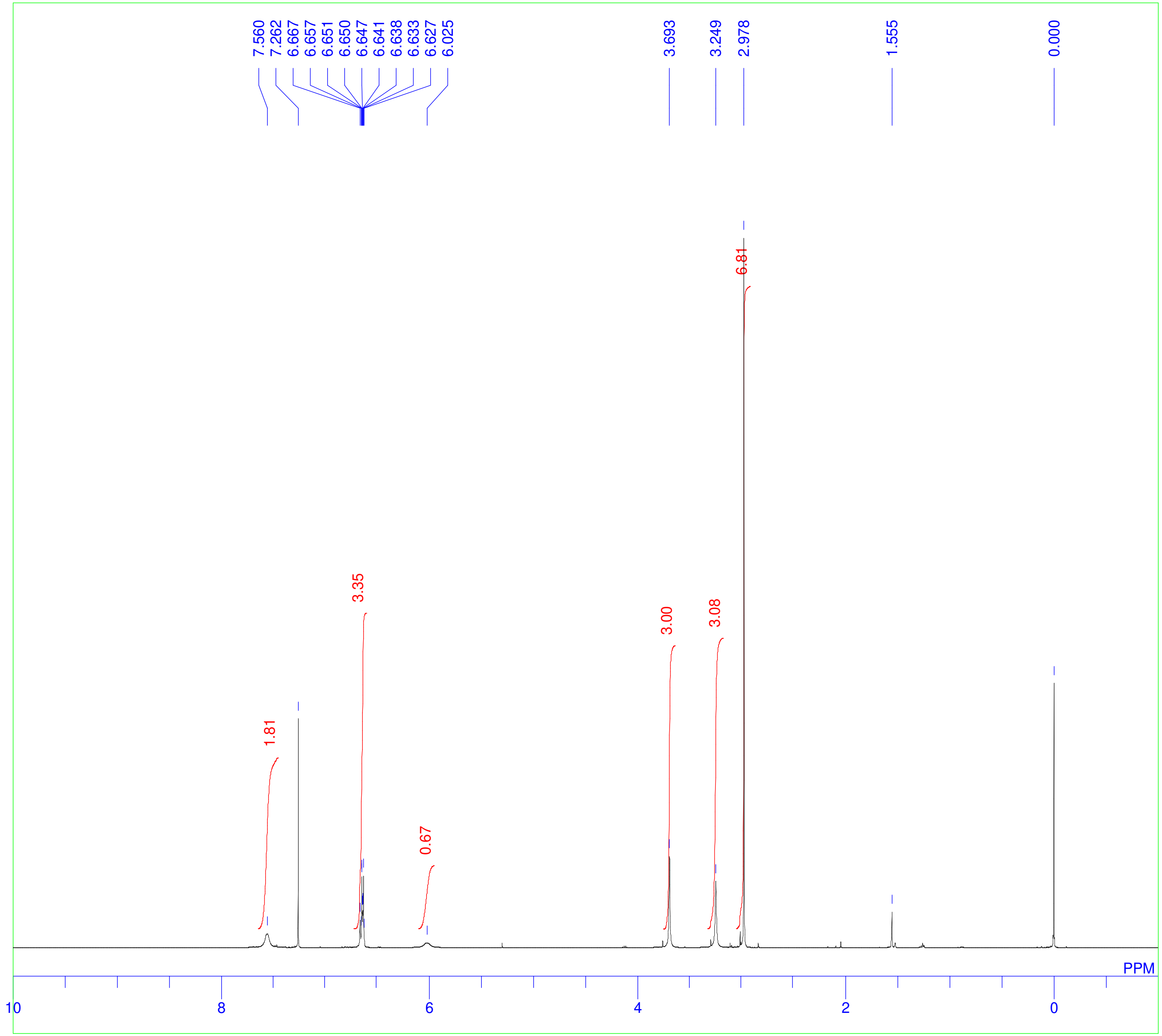

DFILE	(Z)-20h_1H.als
COMNT	
DATIM	2023-12-23 15:10:42
OBNUC	1H
EXMOD	proton.jxp
OBFRQ	500.16 MHz
OBSET	2.41 KHz
OBFIN	6.01 Hz
POINT	13107
FREQU	7507.51 Hz
SCANS	8
ACQTM	1.7459 sec
PD	5.0000 sec
PW1	3.80 usec
IRNUC	1H
CTEMP	22.1 c
SLVNT	CDCL3
EXREF	0.00 ppm
BF	0.30 Hz
RGAIN	48

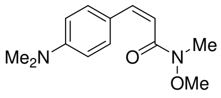

(*Z*)-20h

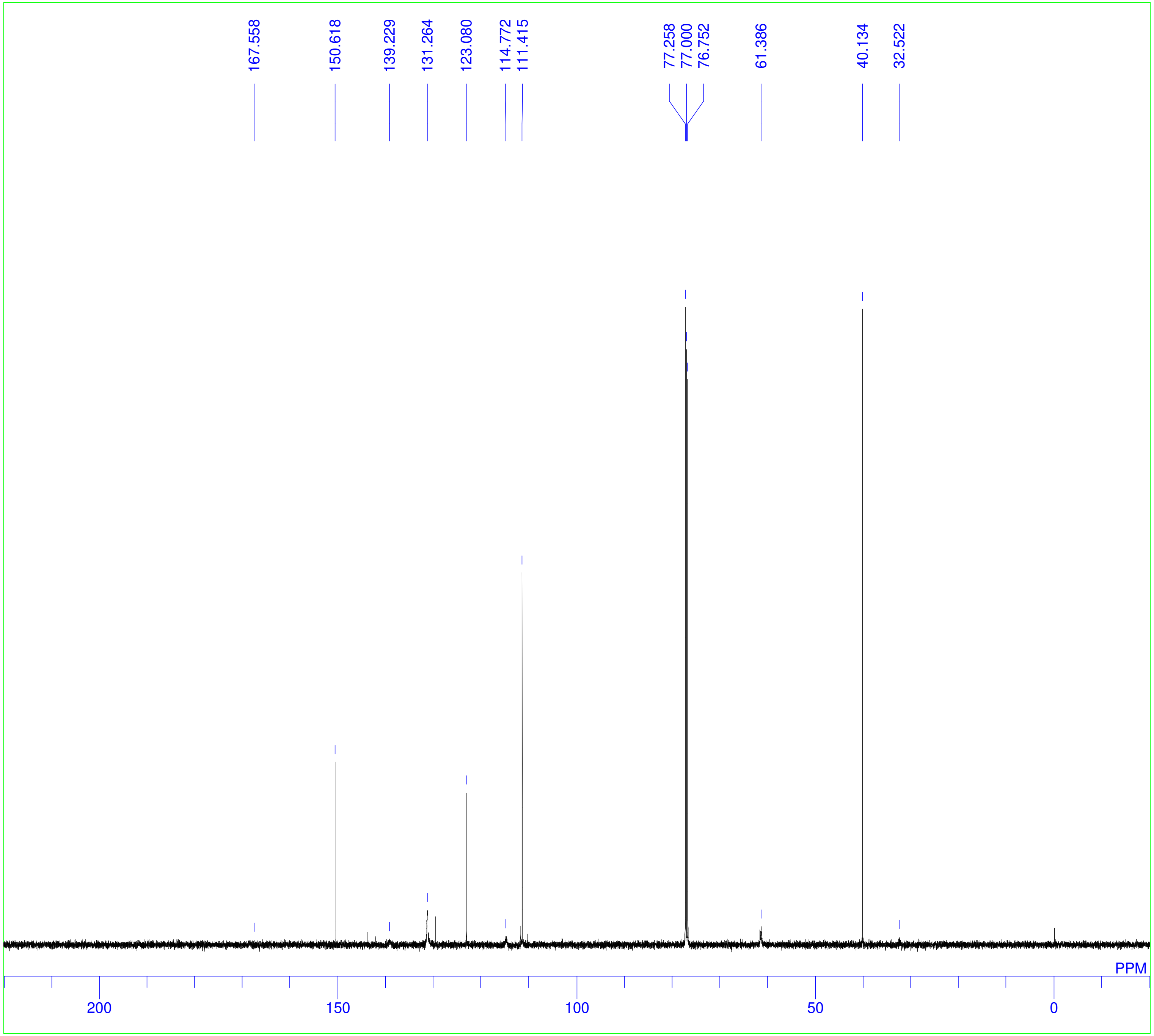

DFILE (Z)-20h_13C.als
COMNT
DATIM 2023-12-27 09:37:59
OBNUC 13C
EXMOD carbon.jxp
OBFRQ 125.77 MHz
OBSET 7.87 KHz
OBFIN 4.21 Hz
POINT 26214
FREQU 31446.54 Hz
SCANS 1024
ACQTM 0.8336 sec
PD 2.0000 sec
PW1 4.30 usec
IRNUC 1H
CTEMP 21.5 c
SLVNT CDCL3
EXREF 77.00 ppm
BF 0.30 Hz
RGAIN 30

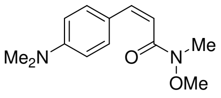

(*Z*)-20h

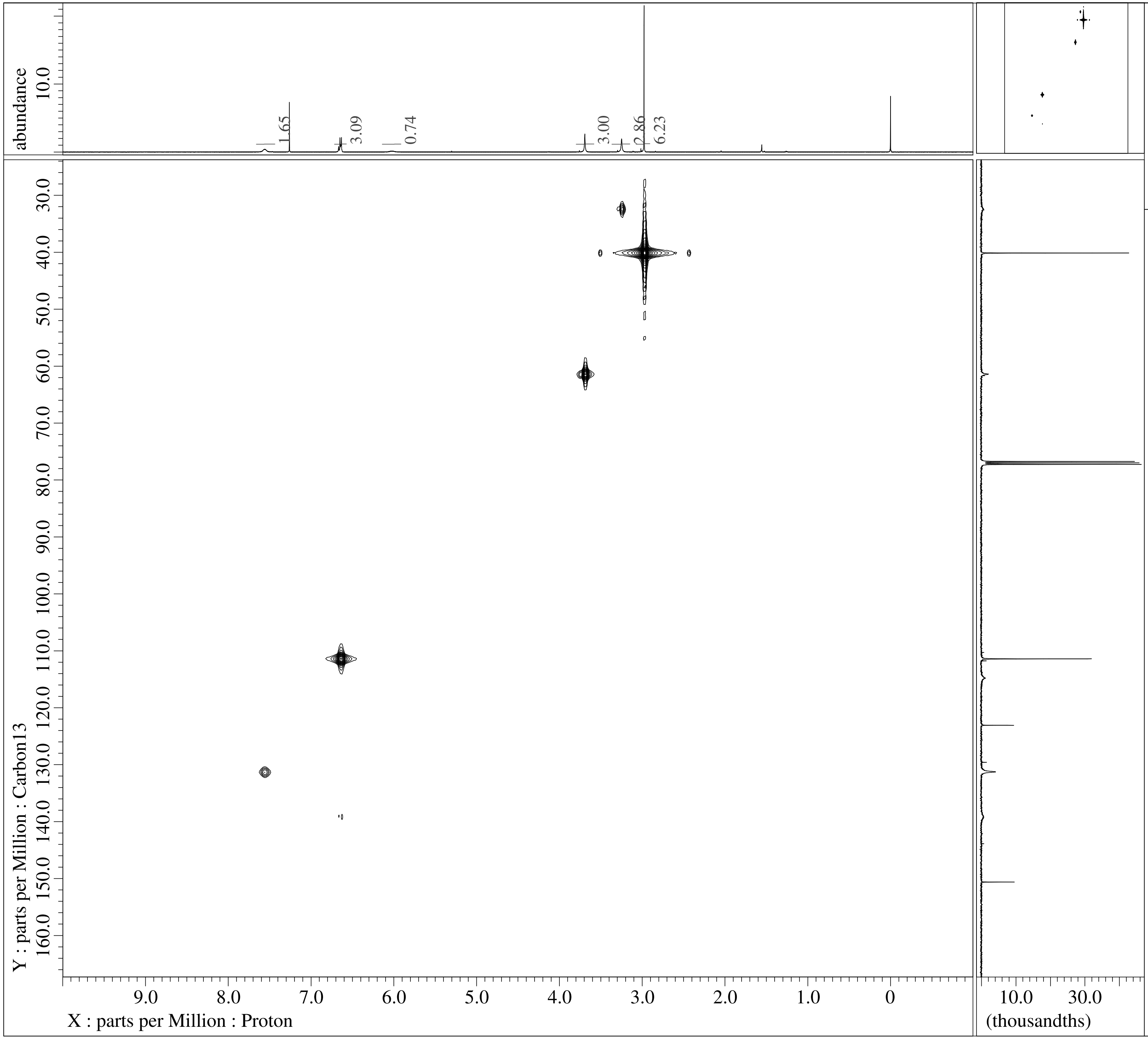


```
---- PROCESSING PARAMETERS ----
sinbell4( -60, 160 )
zerofill( 1 )
fft( 1, TRUE, TRUE )
ppm
[transpose]
sinbell4( -60, 160 )
zerofill( 2 )
fft( 1, TRUE, TRUE )
ppm
abs
```

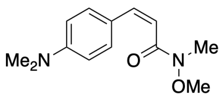

(Z)-20h

```
Filename            = HT-1701-TM_13C_HMQC-1-5
Author              = delta
Experiment          = hmqc.jxp
Sample_Id           = HT-1701-TM_13C
Solvent             = CHLOROFORM-D
Creation_Time       = 23-DEC-2023 21:32:32
Revision_Time       = 11-APR-2024 09:47:06
Current_Time        = 11-APR-2024 09:48:14

Comment             = gradient enhanced HMQC
Data_Format         = 2D REAL REAL
Dim_Size            = 819, 512
Dim_Title           = Proton Carbon13
Dim_Units           = [ppm] [ppm]
Dimensions          = X Y
Site                = JNM-ECA500II
Spectrometer        = DELTA2_NMR

Field_Strength      = 11.7473579[T] (500[MHz]
X_Acq_Duration      = 0.10911744[s]
X_Domain            = 1H
X_Freq              = 500.15991521[MHz]
X_Offset            = 5.0[ppm]
X_Points            = 1024
X_Prescans          = 4
X_Resolution        = 9.16443788[Hz]
X_Sweep             = 9.38438438[kHz]
X_Sweep_Clipped     = 7.50750751[kHz]
Y_Domain            = 13C
Y_Freq              = 125.76529768[MHz]
Y_Offset            = 95.52652538[ppm]
Y_Points            = 256
Y_Prescans          = 0
Y_Resolution        = 70.66298842[Hz]
Y_Sweep             = 18.08972504[kHz]
Tri_Domain          = Proton
Tri_Freq            = 500.15991521[MHz]
Tri_Offset          = 5.0[ppm]
Clipped             = FALSE
Scans               = 4
Total_Scans         = 1024

Relaxation_Delay    = 1.5[s]
Recvr_Gain          = 50
Temp_Get            = 22.1[dC]
X_Acq_Time          = 0.10911744[s]
X_Atn               = 3.2[dB]
X_Gamma             = 42576375
X_Pulse             = 7.59[us]
```

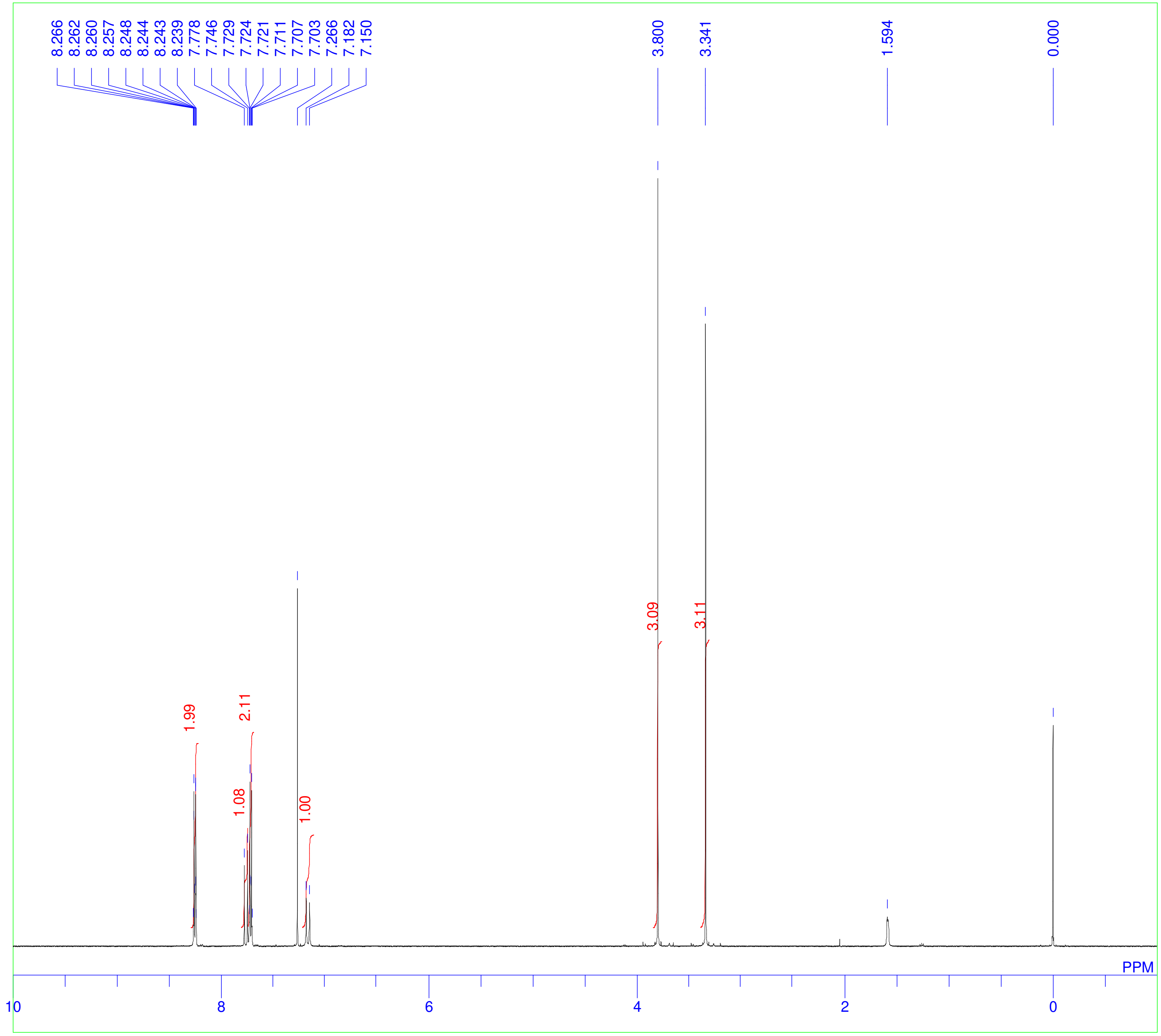

DFILE	(E)-20i_1H.als
COMNT	
DATIM	2022-02-13 11:35:23
OBNUC	1H
EXMOD	proton.jxp
OBFRQ	500.16 MHz
OBSET	2.41 KHz
OBFIN	6.01 Hz
POINT	13107
FREQU	7507.51 Hz
SCANS	8
ACQTM	1.7459 sec
PD	5.0000 sec
PW1	3.84 usec
IRNUC	1H
CTEMP	17.7 c
SLVNT	CDCL3
EXREF	0.00 ppm
BF	0.30 Hz
RGAIN	50

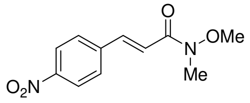

(*E*)-20i

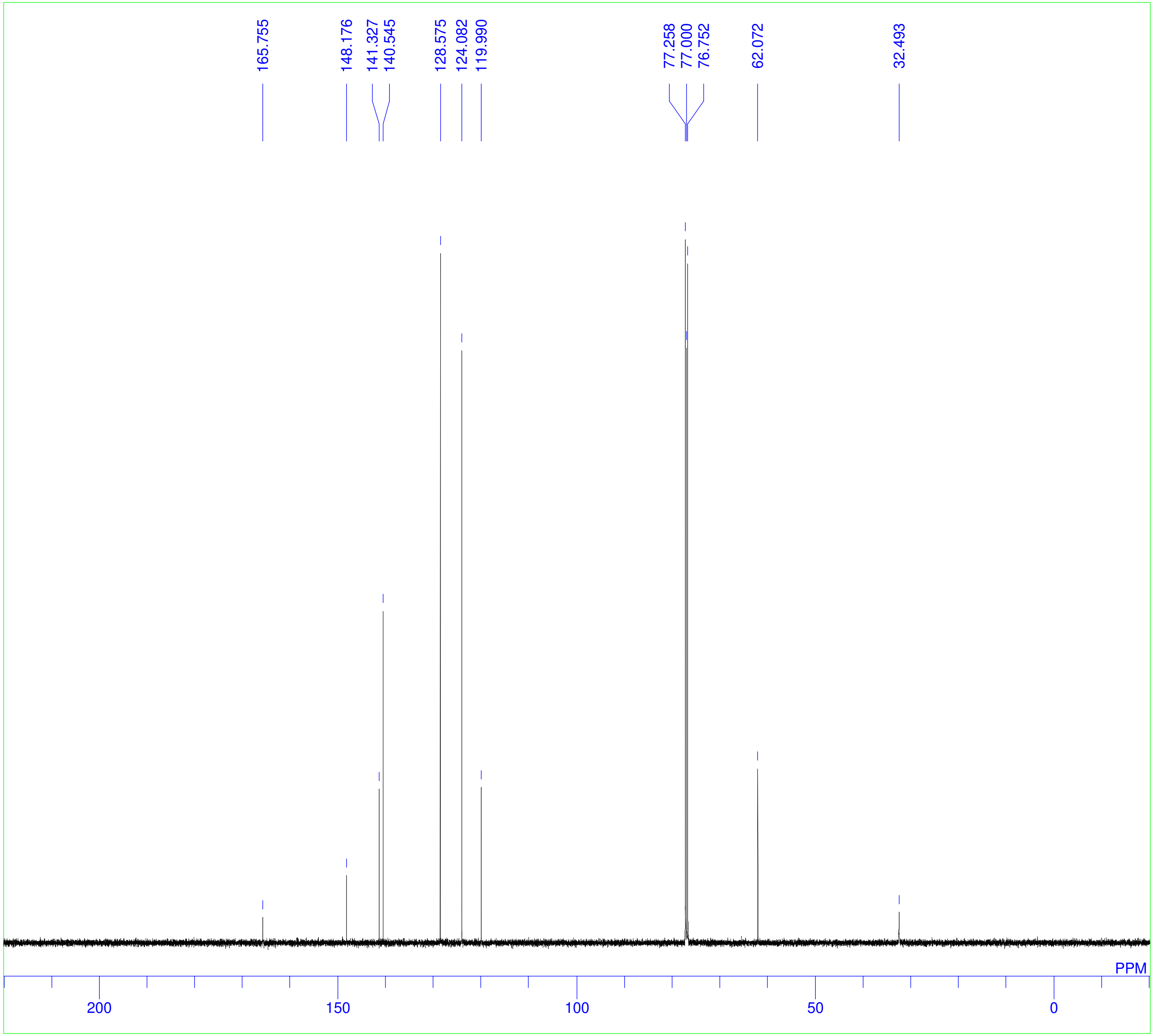

DFILE	(E)-20i_13C.als
COMNT	
DATIM	2023-01-07 10:43:58
OBNUC	13C
EXMOD	carbon.jxp
OBFRQ	125.77 MHz
OBSET	7.87 KHz
OBFIN	4.21 Hz
POINT	26214
FREQU	31446.54 Hz
SCANS	1024
ACQTM	0.8336 sec
PD	2.0000 sec
PW1	3.87 usec
IRNUC	1H
CTEMP	20.7 c
SLVNT	CDCL3
EXREF	77.00 ppm
BF	0.30 Hz
RGAIN	28

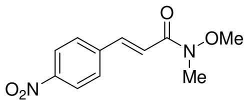

(*E*)-20i

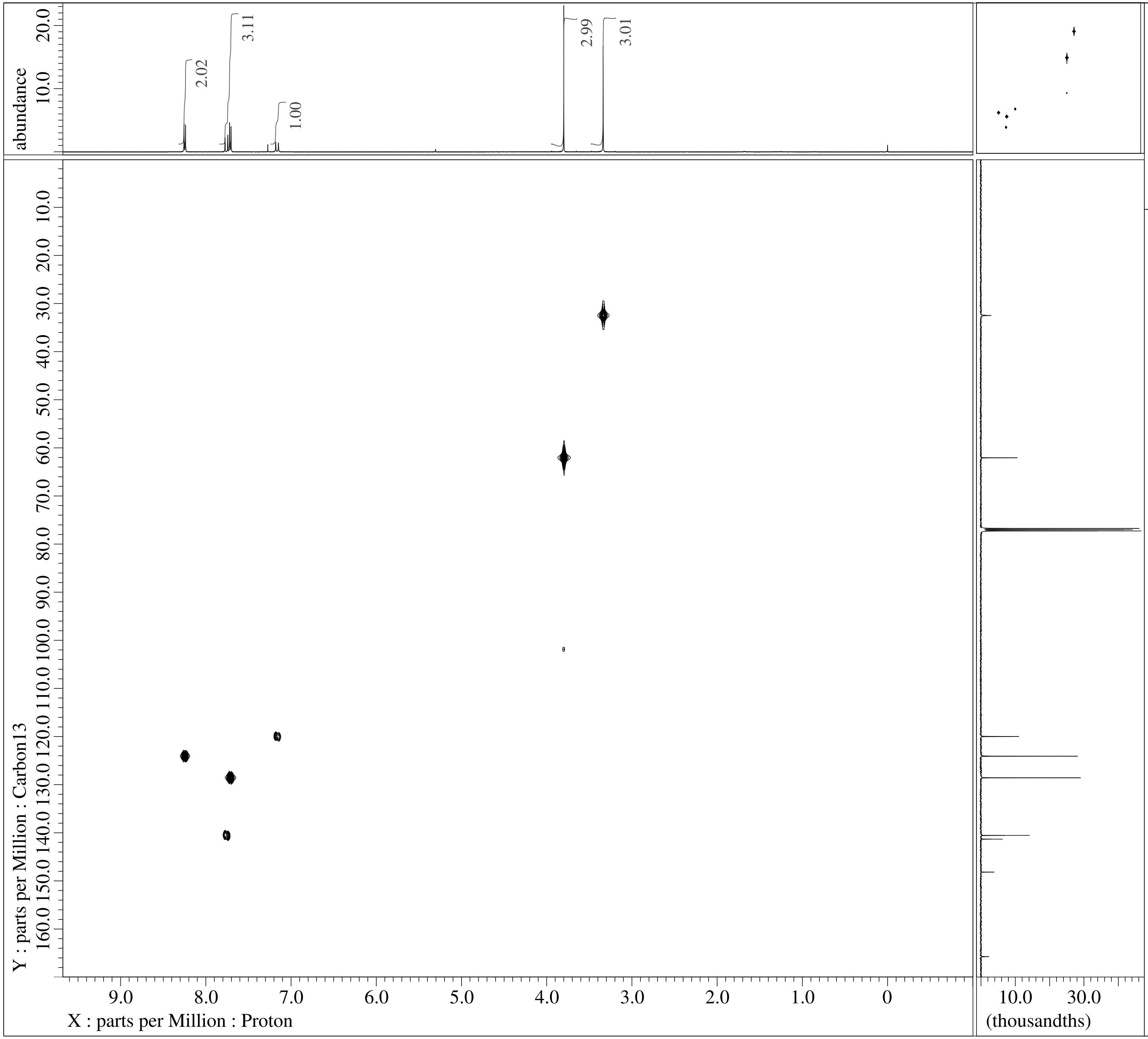


```
---- PROCESSING PARAMETERS ----
sinbell4( -60, 160 )
zerofill( 1 )
fft( 1, TRUE, TRUE )
ppm
[transpose]
sinbell4( -60, 160 )
zerofill( 2 )
fft( 1, TRUE, TRUE )
ppm
abs
```

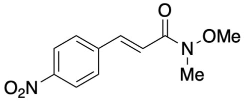

(*E*)-20i

```
Filename            = HT-4-w_HMQC_HMQC-1-5.jd
Author              = delta
Experiment          = hmqc.jxp
Sample_Id           = HT-4-w_HMQC
Solvent             = CHLOROFORM-D
Creation_Time       = 10-JAN-2024 18:35:01
Revision_Time       = 11-APR-2024 09:51:52
Current_Time        = 11-APR-2024 09:52:30

Comment             = gradient enhanced HMQC
Data_Format         = 2D REAL REAL
Dim_Size            = 819, 512
Dim_Title           = Proton Carbon13
Dim_Units           = [ppm] [ppm]
Dimensions          = X Y
Site                = JNM-ECA500II
Spectrometer        = DELTA2_NMR

Field_Strength      = 11.7473579[T] (500[MHz]
X_Acq_Duration      = 0.14958592[s]
X_Domain            = 1H
X_Freq              = 500.15991521[MHz]
X_Offset            = 4.1851984[ppm]
X_Points            = 1024
X_Prescans          = 4
X_Resolution        = 6.68512117[Hz]
X_Sweep             = 6.84556407[kHz]
X_Sweep_Clipped     = 5.47645126[kHz]
Y_Domain            = 13C
Y_Freq              = 125.76529768[MHz]
Y_Offset            = 85[ppm]
Y_Points            = 256
Y_Prescans          = 0
Y_Resolution        = 83.60980308[Hz]
Y_Sweep             = 21.40410959[kHz]
Tri_Domain          = Proton
Tri_Freq            = 500.15991521[MHz]
Tri_Offset          = 5.0[ppm]
Clipped             = FALSE
Scans               = 8
Total_Scans         = 2048

Relaxation_Delay    = 1.5[s]
Recvr_Gain          = 50
Temp_Get            = 22.2[dC]
X_Acq_Time          = 0.14958592[s]
X_Atn               = 3.2[dB]
X_Gamma             = 42576375
X_Pulse             = 7.59[us]
```

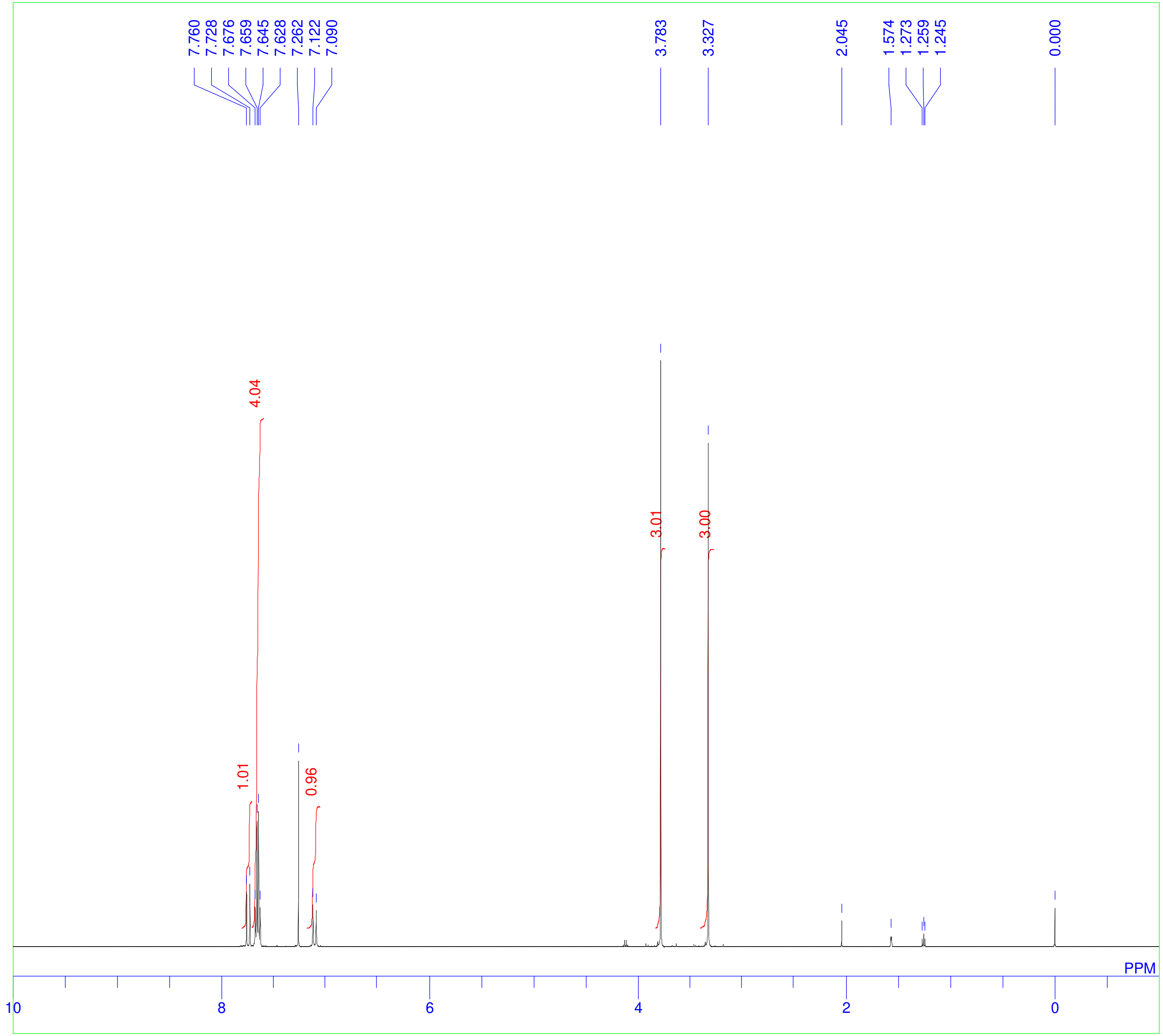

DFILE	(E)-20j_1H.als
COMNT	
DATIM	2023-04-13 13:22:48
OBNUC	1H
EXMOD	proton.jxp
OBFRQ	500.16 MHz
OBSET	2.41 KHz
OBFIN	6.01 Hz
POINT	13107
FREQU	7507.51 Hz
SCANS	8
ACQTM	1.7459 sec
PD	5.0000 sec
PW1	3.84 usec
IRNUC	1H
CTEMP	23.8 c
SLVNT	CDCL3
EXREF	0.00 ppm
BF	0.30 Hz
RGAIN	46

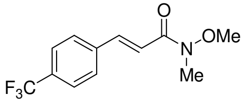

(***E***)**-20j**

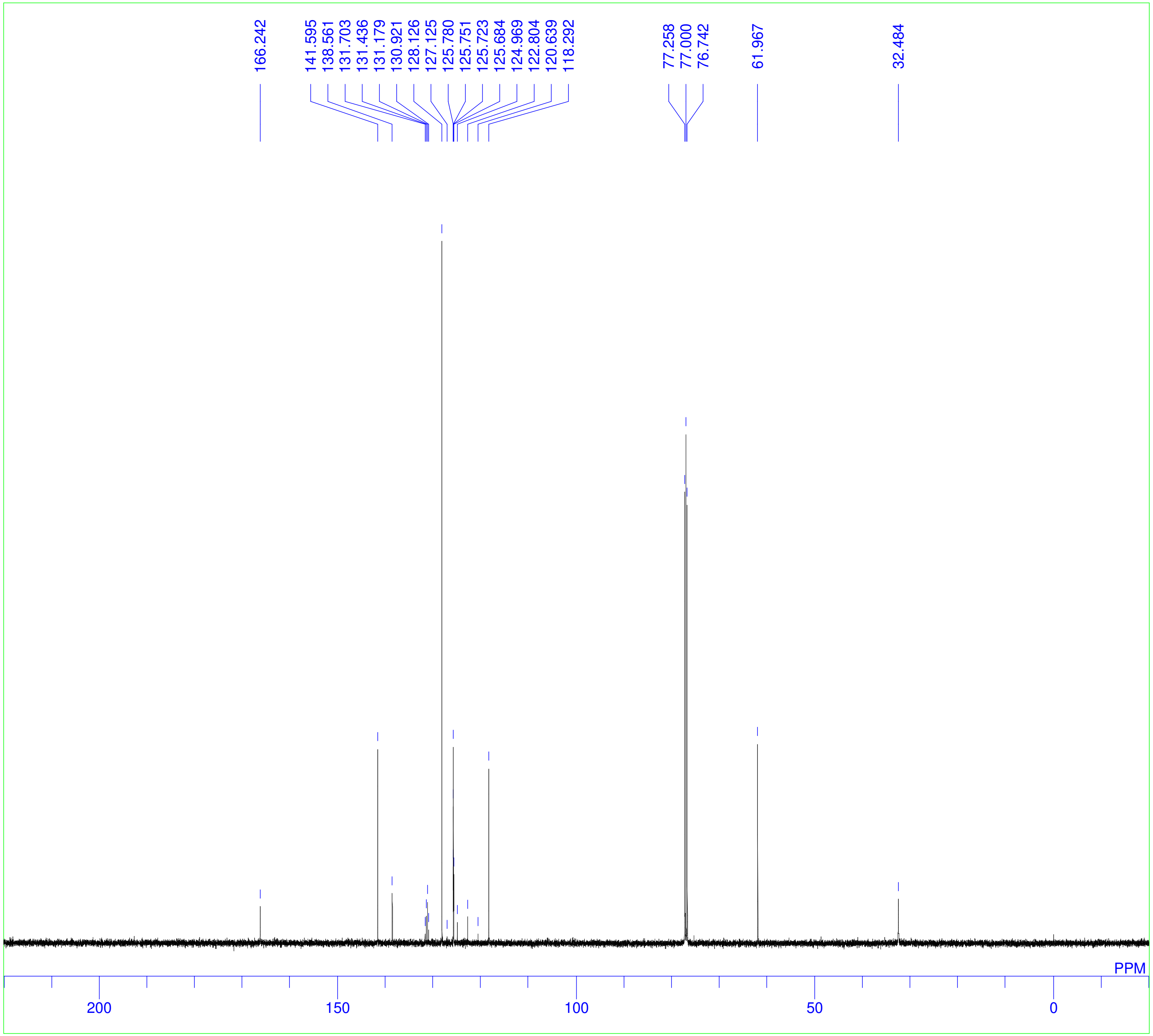

DFILE	(E)-20j_13C.als
COMNT	
DATIM	2023-04-14 07:05:37
OBNUC	13C
EXMOD	carbon.jxp
OBFRQ	125.77 MHz
OBSET	7.87 KHz
OBFIN	4.21 Hz
POINT	26214
FREQU	31446.54 Hz
SCANS	1024
ACQTM	0.8336 sec
PD	2.0000 sec
PW1	3.87 usec
IRNUC	1H
CTEMP	23.9 c
SLVNT	CDCL3
EXREF	77.00 ppm
BF	0.30 Hz
RGAIN	30

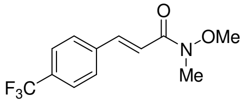

(*E*)-20j

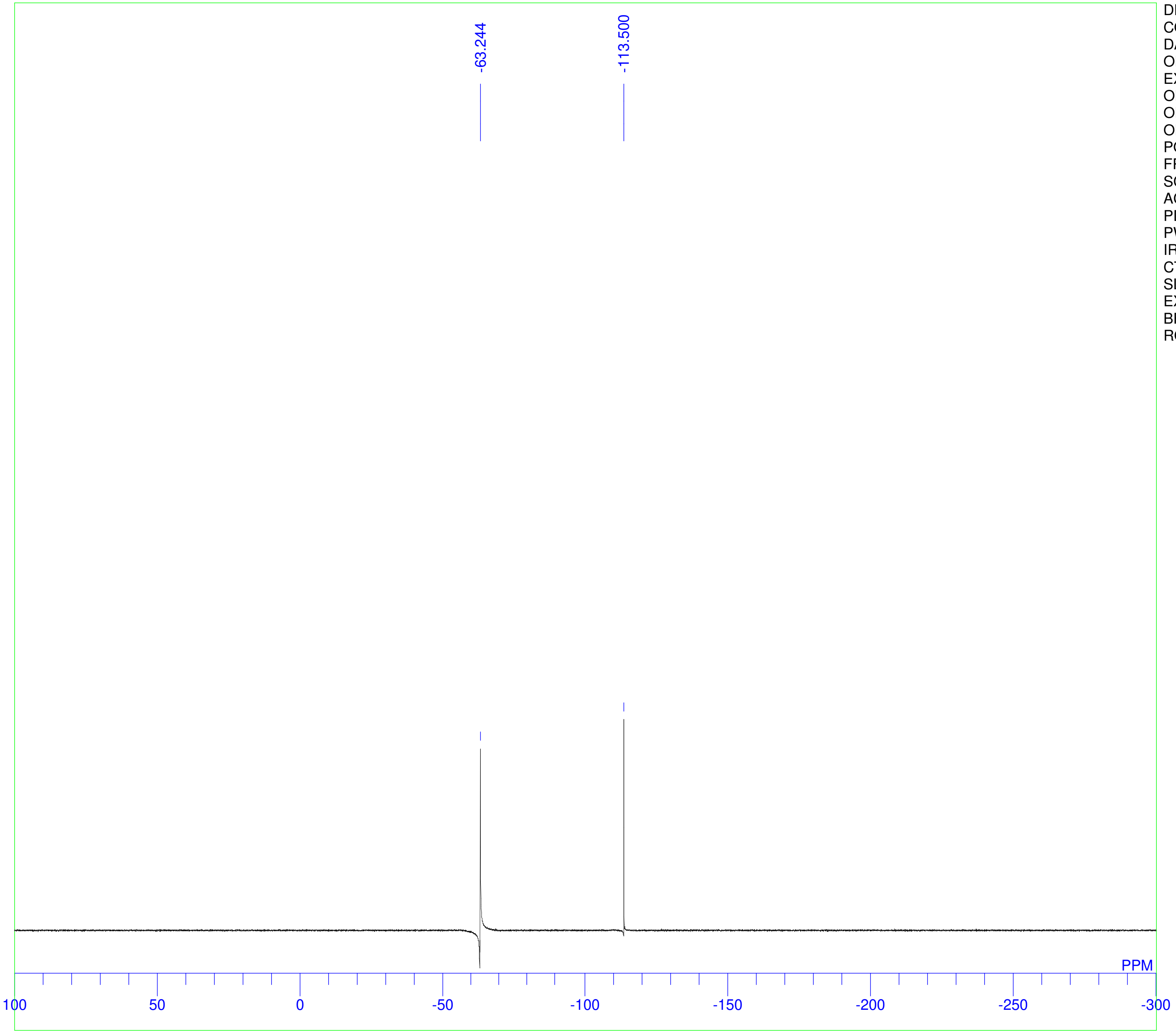

DFILE	(E)-20j_19F.als
COMNT	
DATIM	2024-01-12 18:45:25
OBNUC	19F
EXMOD	single_pulse.jxp
OBFRQ	470.62 MHz
OBSET	0.46 KHz
OBFIN	0.84 Hz
POINT	13107
FREQU	285714.28 Hz
SCANS	8
ACQTM	0.0459 sec
PD	5.0000 sec
PW1	4.25 usec
IRNUC	19F
CTEMP	22.4 c
SLVNT	CDCL3
EXREF	-113.50 ppm
BF	0.25 Hz
RGAIN	50

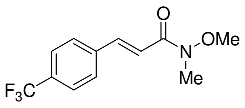

(*E*)-20j

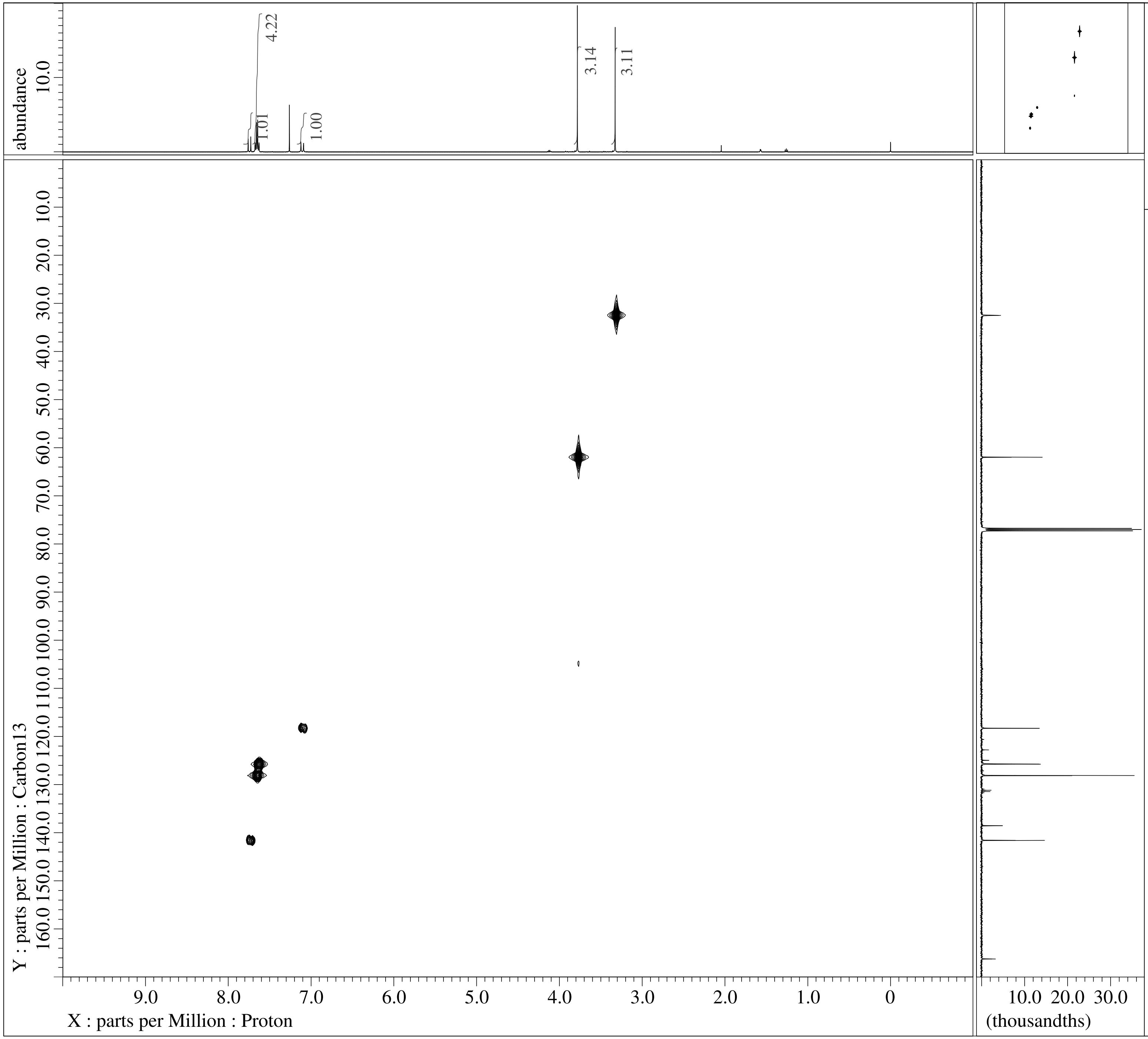


```
---- PROCESSING PARAMETERS ----
sinbell4( -60, 160 )
zerofill( 1 )
fft( 1, TRUE, TRUE )
ppm
[transpose]
sinbell4( -60, 160 )
zerofill( 2 )
fft( 1, TRUE, TRUE )
ppm
abs
```

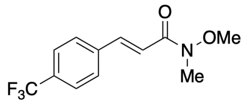

(*E*)-20j

```
Filename            = HT-1348,1349-TM_HMQC_HM
Author              = delta
Experiment          = hmqc.jxp
Sample_Id           = HT-1348,1349-TM_HMQC
Solvent             = CHLOROFORM-D
Creation_Time       = 12-JAN-2024 18:54:21
Revision_Time       = 11-APR-2024 09:58:29
Current_Time        = 11-APR-2024 09:59:03

Comment             = gradient enhanced HMQC
Data_Format         = 2D REAL REAL
Dim_Size            = 819, 512
Dim_Title           = Proton Carbon13
Dim_Units           = [ppm] [ppm]
Dimensions          = X Y
Site                = JNM-ECA500II
Spectrometer        = DELTA2_NMR

Field_Strength      = 11.7473579[T] (500[MHz]
X_Acq_Duration      = 0.10911744[s]
X_Domain            = 1H
X_Freq              = 500.15991521[MHz]
X_Offset            = 5.0[ppm]
X_Points            = 1024
X_Prescans          = 4
X_Resolution        = 9.16443788[Hz]
X_Sweep             = 9.38438438[kHz]
X_Sweep_Clipped     = 7.50750751[kHz]
Y_Domain            = 13C
Y_Freq              = 125.76529768[MHz]
Y_Offset            = 85[ppm]
Y_Points            = 256
Y_Prescans          = 0
Y_Resolution        = 83.60980308[Hz]
Y_Sweep             = 21.40410959[kHz]
Tri_Domain          = Proton
Tri_Freq            = 500.15991521[MHz]
Tri_Offset          = 5.0[ppm]
Clipped             = FALSE
Scans               = 16
Total_Scans         = 4096

Relaxation_Delay    = 1.5[s]
Recvr_Gain          = 50
Temp_Get            = 22.2[dC]
X_Acq_Time          = 0.10911744[s]
X_Atn               = 3.2[dB]
X_Gamma             = 42576375
X_Pulse             = 7.59[us]
```

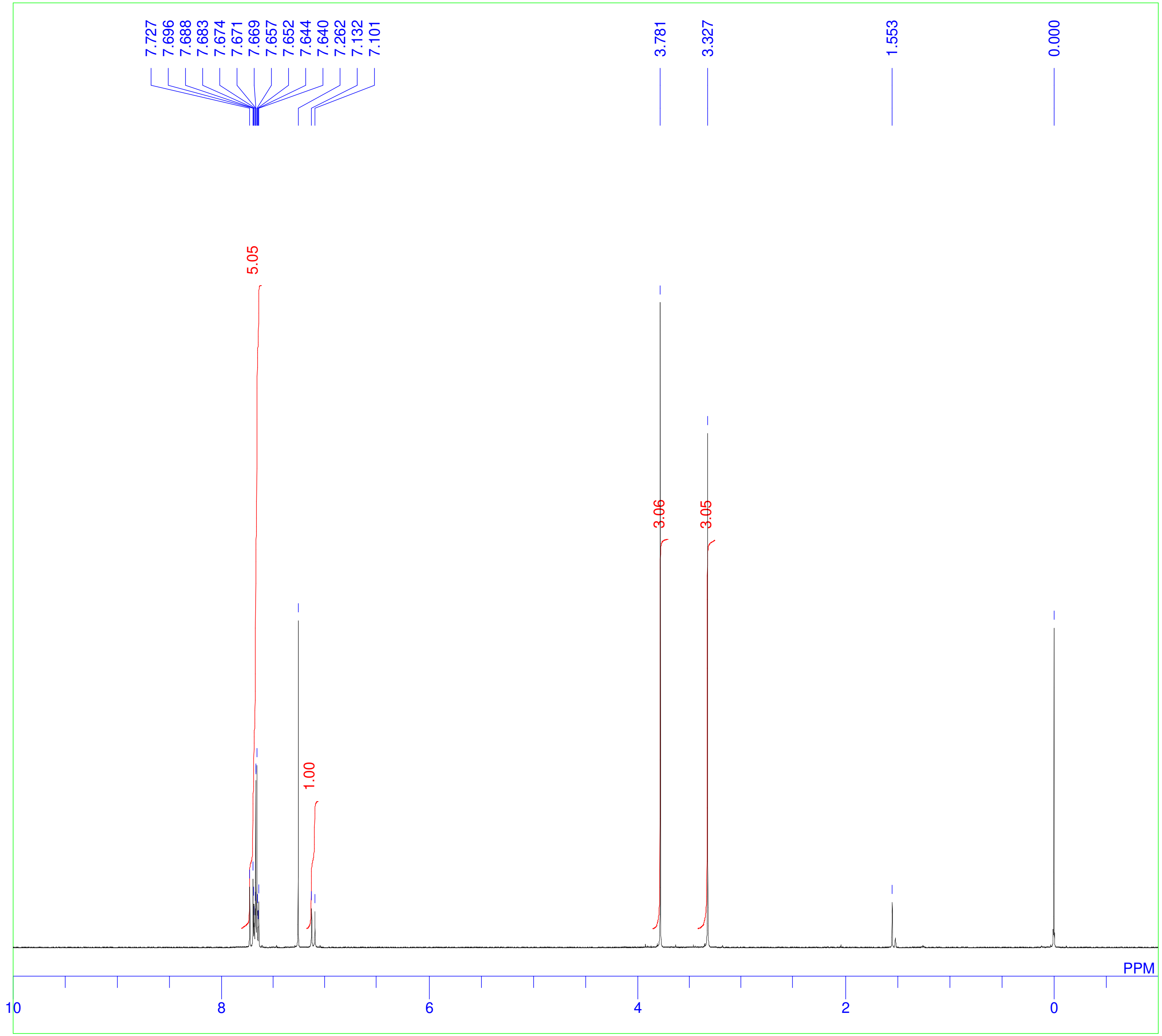

DFILE	(E)-20k_1H.als
COMNT	
DATIM	2023-12-20 08:40:22
OBNUC	1H
EXMOD	proton.jxp
OBFRQ	500.16 MHz
OBSET	2.41 KHz
OBFIN	6.01 Hz
POINT	13107
FREQU	7507.51 Hz
SCANS	8
ACQTM	1.7459 sec
PD	5.0000 sec
PW1	3.80 usec
IRNUC	1H
CTEMP	22.9 c
SLVNT	CDCL3
EXREF	0.00 ppm
BF	0.30 Hz
RGAIN	46

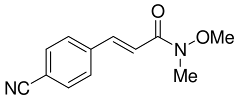

(*E*)-20k

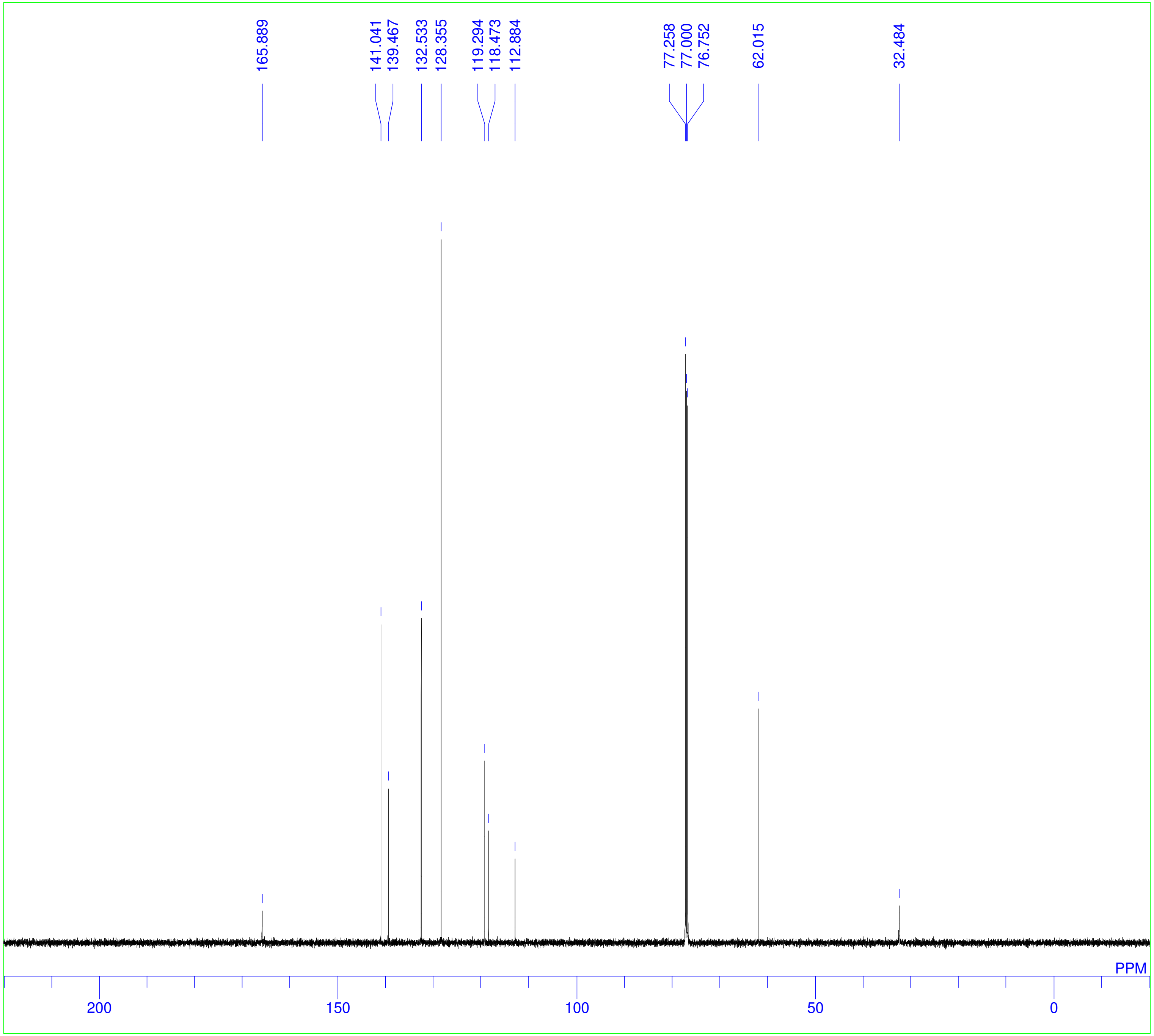

DFILE (E)-20k_13C.als
COMNT
DATIM 2023-12-20 20:46:04
OBNUC 13C
EXMOD carbon.jxp
OBFRQ 125.77 MHz
OBSET 7.87 KHz
OBFIN 4.21 Hz
POINT 26214
FREQU 31446.54 Hz
SCANS 1024
ACQTM 0.8336 sec
PD 2.0000 sec
PW1 4.30 usec
IRNUC 1H
CTEMP 23.5 c
SLVNT CDCL3
EXREF 77.00 ppm
BF 0.30 Hz
RGAIN 30

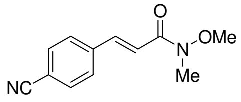

(*E*)-20k

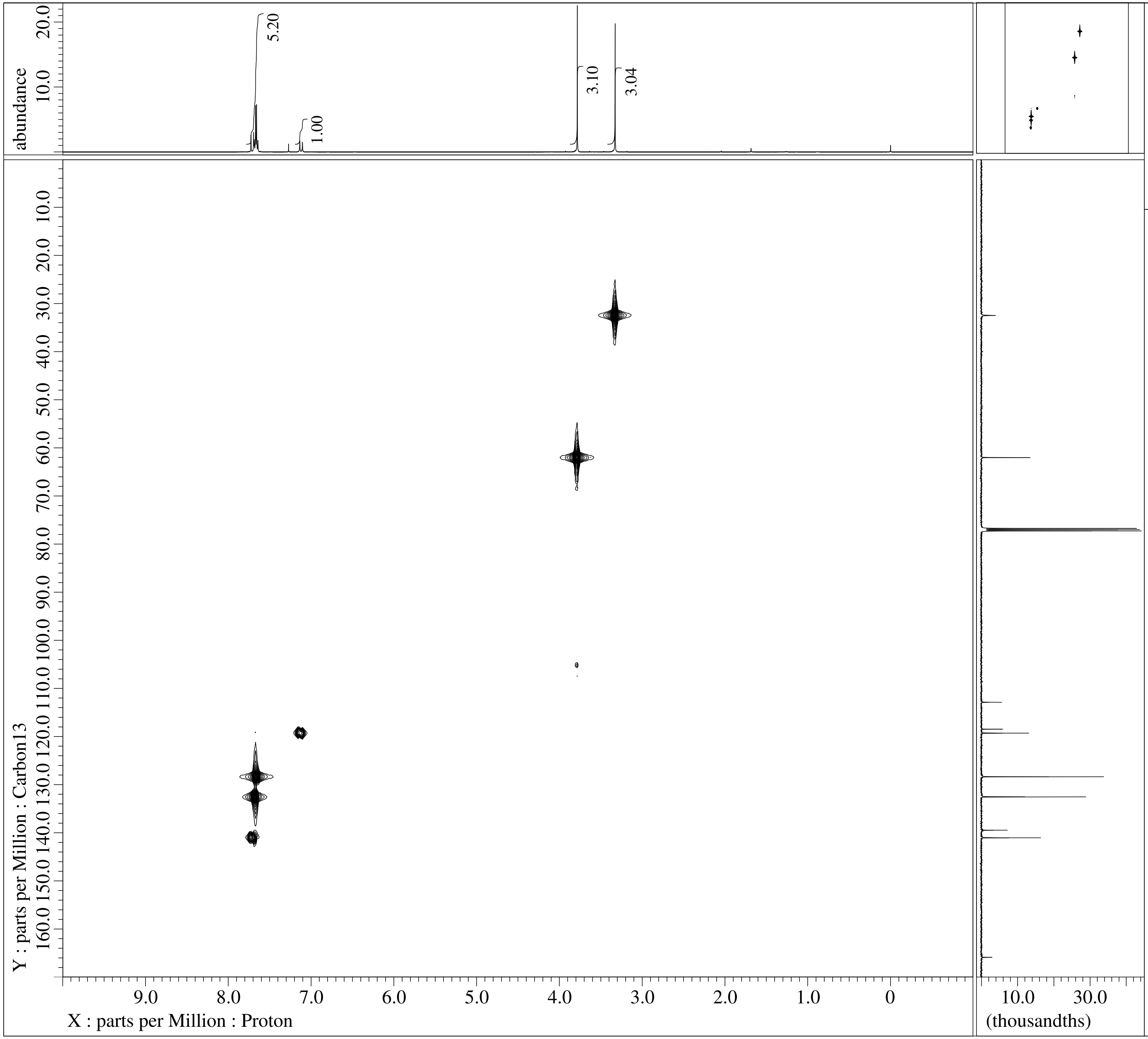


```
---- PROCESSING PARAMETERS ----
sinbell4( -60, 160 )
zerofill( 1 )
fft( 1, TRUE, TRUE )
ppm
[transpose]
sinbell4( -60, 160 )
zerofill( 2 )
fft( 1, TRUE, TRUE )
ppm
abs
```

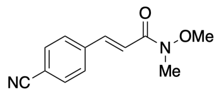

(*E*)-20k

```
Filename            = HT-1690,1691-TM_HMQC,HM
Author              = delta
Experiment          = hmqc.jxp
Sample_Id           = HT-1690,1691-TM_HMQC,HM
Solvent             = CHLOROFORM-D
Creation_Time       = 16-JAN-2024 20:02:49
Revision_Time       = 11-APR-2024 10:08:36
Current_Time        = 11-APR-2024 10:08:58

Comment             = gradient enhanced HMQC
Data_Format         = 2D REAL REAL
Dim_Size            = 819, 512
Dim_Title           = Proton Carbon13
Dim_Units           = [ppm] [ppm]
Dimensions          = X Y
Site                = JNM-ECA500II
Spectrometer        = DELTA2_NMR

Field_Strength      = 11.7473579[T] (500[MHz]
X_Acq_Duration      = 0.10911744[s]
X_Domain            = 1H
X_Freq              = 500.15991521[MHz]
X_Offset            = 5.0[ppm]
X_Points            = 1024
X_Prescans          = 4
X_Resolution        = 9.16443788[Hz]
X_Sweep             = 9.38438438[kHz]
X_Sweep_Clipped     = 7.50750751[kHz]
Y_Domain            = 13C
Y_Freq              = 125.76529768[MHz]
Y_Offset            = 85[ppm]
Y_Points            = 256
Y_Prescans          = 0
Y_Resolution        = 83.60980308[Hz]
Y_Sweep             = 21.40410959[kHz]
Tri_Domain          = Proton
Tri_Freq            = 500.15991521[MHz]
Tri_Offset          = 5.0[ppm]
Clipped             = FALSE
Scans               = 8
Total_Scans         = 2048

Relaxation_Delay    = 1.5[s]
Recvr_Gain          = 50
Temp_Get            = 20.4[dC]
X_Acq_Time          = 0.10911744[s]
X_Atn               = 3.2[dB]
X_Gamma             = 42576375
X_Pulse             = 7.59[us]
```

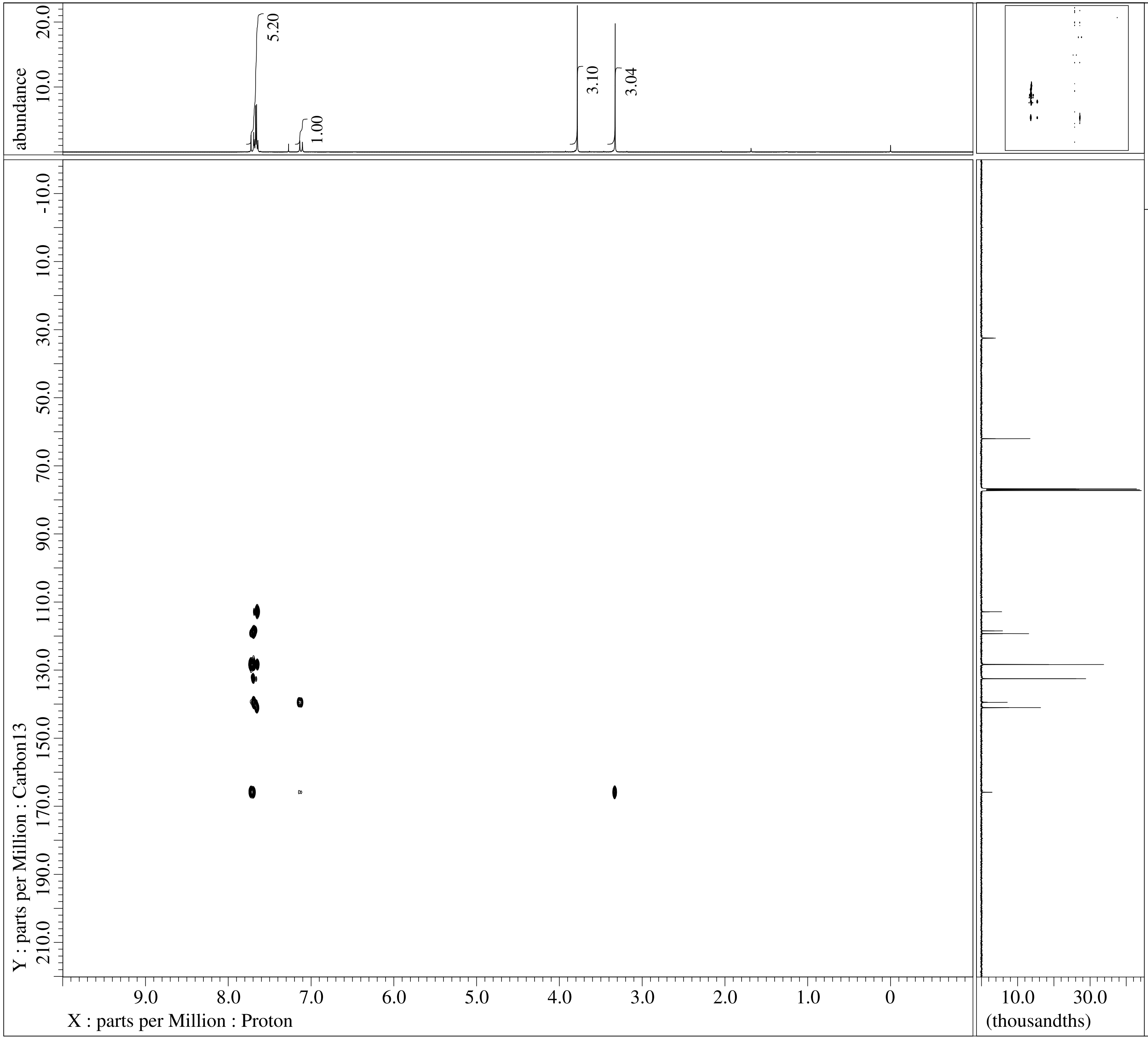

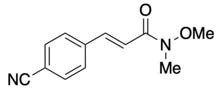

(*E*)-20k

```
---- PROCESSING PARAMETERS ----
gauss( 5.0[Hz], 0.0[s] )
sinbell_auto
zerofill( 1 )
fft( 1, TRUE, TRUE )
ppm
[transpose]
sinbell4( -60, 160 )
trapezoid( 0[%], 5[%], 80[%], 100[%] )
zerofill( 2 )
fft( 1, TRUE, TRUE )
```

```
Filename            = HT-1690,1691-TM_HMQC,HM
Author              = delta
Experiment          = hmbc.jxp
Sample_Id           = HT-1690,1691-TM_HMQC,HM
Solvent             = CHLOROFORM-D
Creation_Time       = 16-JAN-2024 20:58:49
Revision_Time       = 11-APR-2024 10:10:51
Current_Time        = 11-APR-2024 10:11:50

Comment             = gradient enhanced HMBC
Data_Format         = 2D REAL REAL
Dim_Size            = 1638, 512
Dim_Title           = Proton Carbon13
Dim_Units           = [ppm] [ppm]
Dimensions          = X Y
Site                = JNM-ECA500II
Spectrometer        = DELTA2_NMR

Field_Strength      = 11.7473579[T] (500[MHz]
X_Acq_Duration      = 0.21823488[s]
X_Domain            = 1H
X_Freq              = 500.15991521[MHz]
X_Offset            = 5.0[ppm]
X_Points            = 2048
X_Prescans          = 4
X_Resolution        = 4.58221894[Hz]
X_Sweep             = 9.38438438[kHz]
X_Sweep_Clipped     = 7.50750751[kHz]
Y_Domain            = 13C
Y_Freq              = 125.76529768[MHz]
Y_Offset            = 100[ppm]
Y_Points            = 256
Y_Prescans          = 0
Y_Resolution        = 122.99275819[Hz]
Y_Sweep             = 31.4861461[kHz]
Tri_Domain          = Proton
Tri_Freq            = 500.15991521[MHz]
Tri_Offset          = 5.0[ppm]
Clipped             = FALSE
Scans               = 8
Total_Scans         = 2048

Relaxation_Delay    = 1.5[s]
Recvr_Gain          = 50
Temp_Get            = 20.4[dC]
X_Acq_Time          = 0.21823488[s]
X_Atn               = 3.2[dB]
X_Gamma             = 42576375
X_Pulse             = 7.59[us]
```

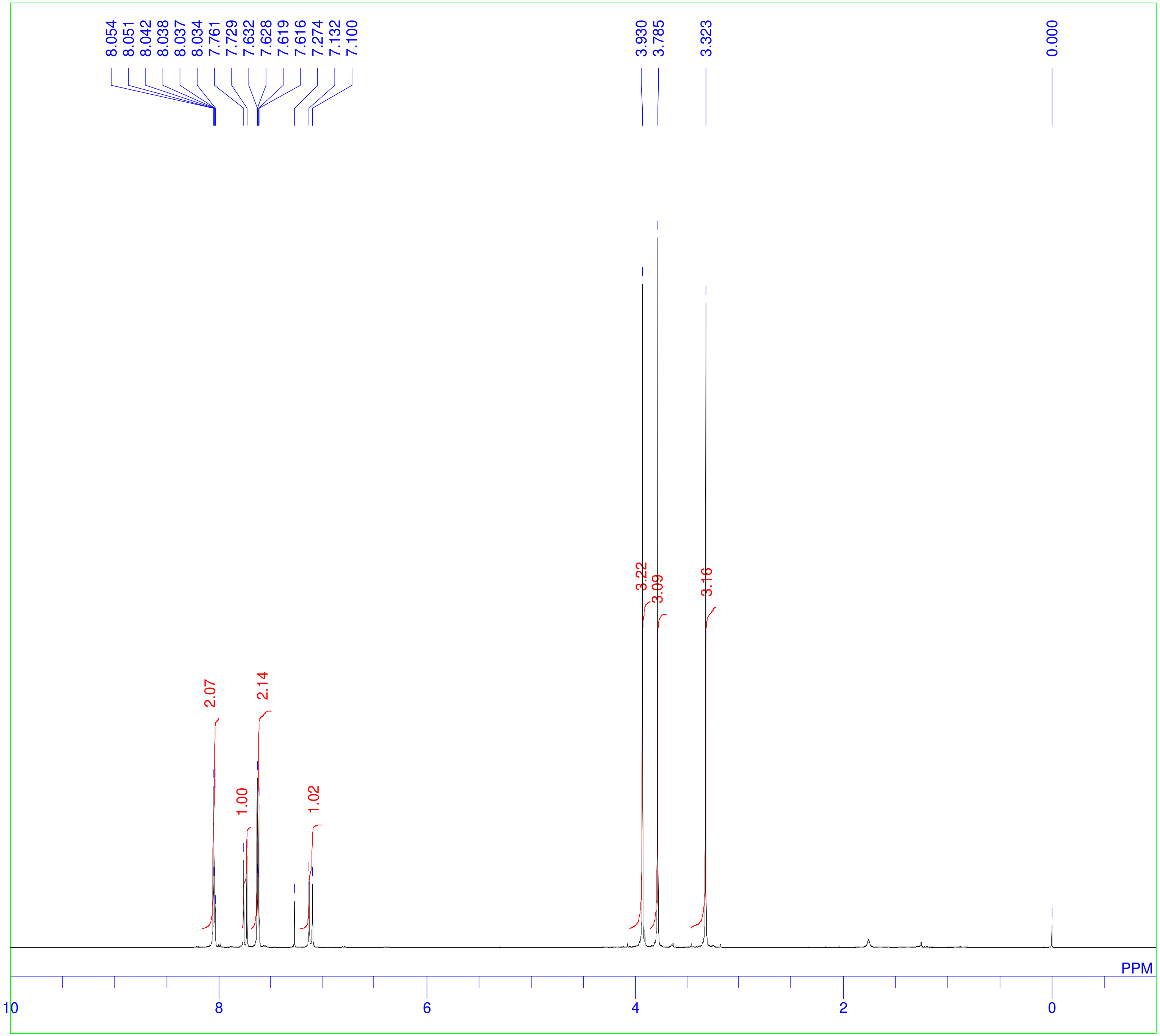

DFILE (E)-20l_1H.als
COMNT
DATIM 2023-04-13 21:49:35
OBNUC 1H
EXMOD proton.jxp
OBFRQ 500.16 MHz
OBSET 2.41 KHz
OBFIN 6.01 Hz
POINT 13107
FREQU 7507.51 Hz
SCANS 8
ACQTM 1.7459 sec
PD 5.0000 sec
PW1 3.84 usec
IRNUC 1H
CTEMP 24.2 c
SLVNT CDCL3
EXREF 0.00 ppm
BF 0.30 Hz
RGAIN 30

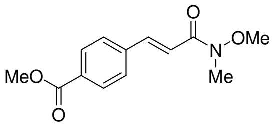

(*E*)-20l

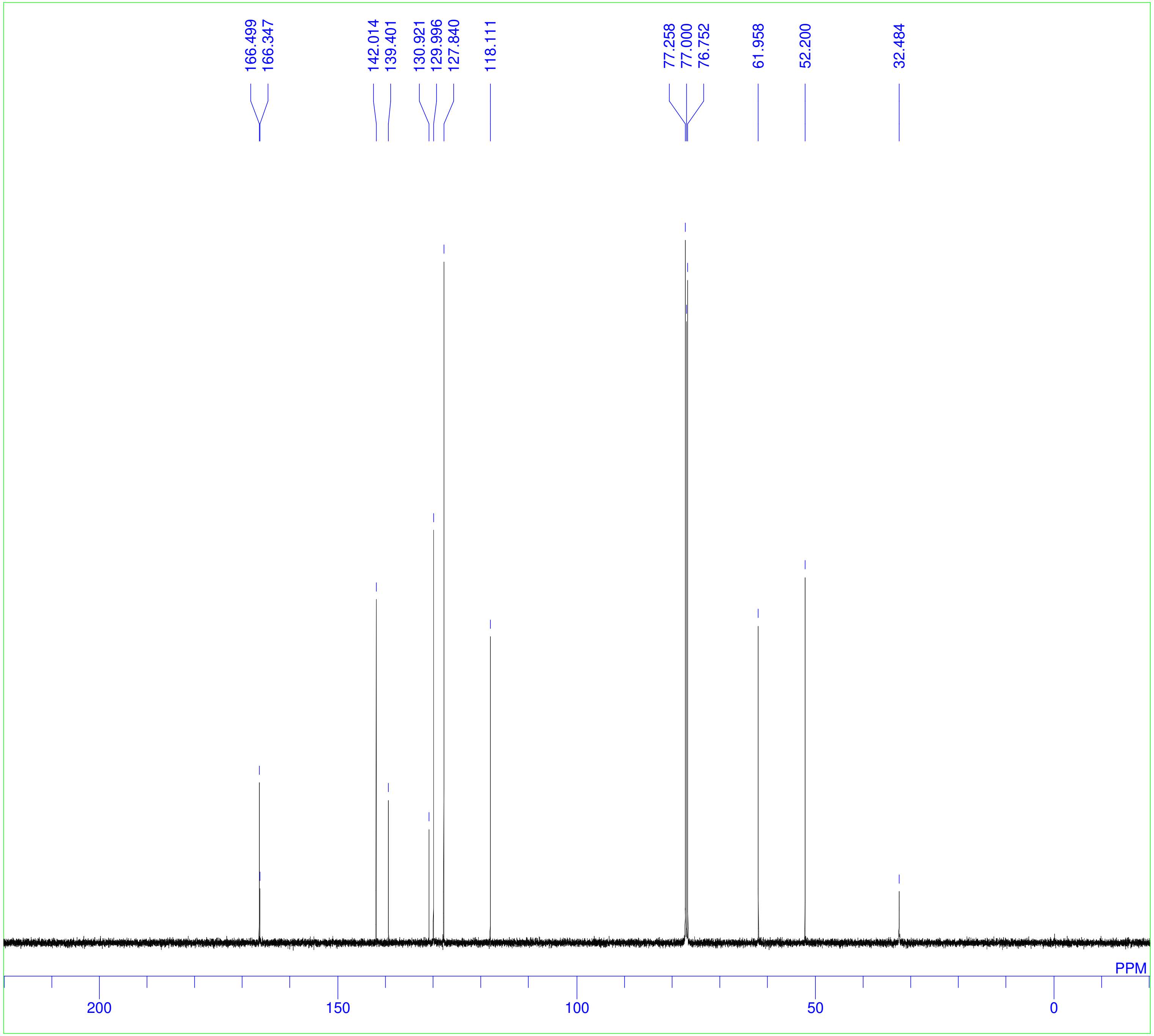

DFILE (E)-20l_13C.als
COMNT
DATIM 2023-04-13 21:00:18
OBNUC 13C
EXMOD carbon.jxp
OBFRQ 125.77 MHz
OBSET 7.87 KHz
OBFIN 4.21 Hz
POINT 26214
FREQU 31446.54 Hz
SCANS 1024
ACQTM 0.8336 sec
PD 2.0000 sec
PW1 3.87 usec
IRNUC 1H
CTEMP 24.2 c
SLVNT CDCL3
EXREF 77.00 ppm
BF 0.30 Hz
RGAIN 32

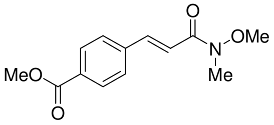

(*E*)-20l

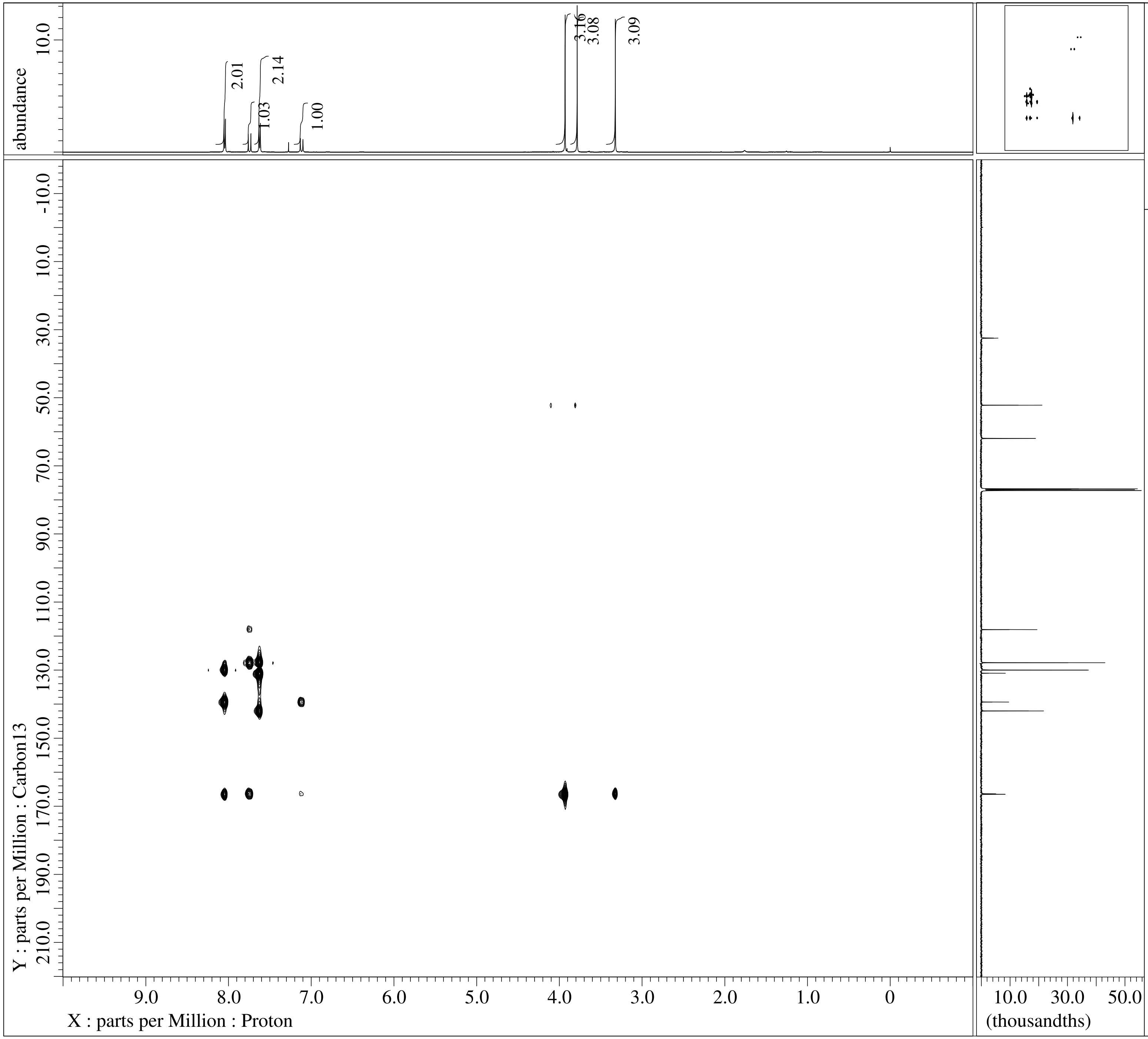


```
---- PROCESSING PARAMETERS ----
gauss( 5.0[Hz], 0.0[s] )
sinbell_auto
zerofill( 1 )
fft( 1, TRUE, TRUE )
ppm
[transpose]
sinbell4( -60, 160 )
trapezoid( 0[%], 5[%], 80[%], 100[%] )
zerofill( 2 )
fft( 1, TRUE, TRUE )
```

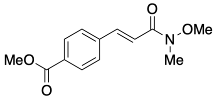

(E)-20l

```
Filename            = HT-1346,1347-TM_HMBC_HM
Author              = delta
Experiment          = hmbc.jxp
Sample_Id           = HT-1346,1347-TM_HMBC
Solvent             = CHLOROFORM-D
Creation_Time       = 12-JAN-2024 20:50:04
Revision_Time       = 11-APR-2024 10:13:33
Current_Time        = 11-APR-2024 10:15:48

Comment             = gradient enhanced HMBC
Data_Format         = 2D REAL REAL
Dim_Size            = 1638, 512
Dim_Title           = Proton Carbon13
Dim_Units           = [ppm] [ppm]
Dimensions          = X Y
Site                = JNM-ECA500II
Spectrometer        = DELTA2_NMR

Field_Strength      = 11.7473579[T] (500[MHz]
X_Acq_Duration      = 0.21823488[s]
X_Domain            = 1H
X_Freq              = 500.15991521[MHz]
X_Offset            = 5.0[ppm]
X_Points            = 2048
X_Prescans          = 4
X_Resolution        = 4.58221894[Hz]
X_Sweep             = 9.38438438[kHz]
X_Sweep_Clipped     = 7.50750751[kHz]
Y_Domain            = 13C
Y_Freq              = 125.76529768[MHz]
Y_Offset            = 100[ppm]
Y_Points            = 256
Y_Prescans          = 0
Y_Resolution        = 122.99275819[Hz]
Y_Sweep             = 31.4861461[kHz]
Tri_Domain          = Proton
Tri_Freq            = 500.15991521[MHz]
Tri_Offset          = 5.0[ppm]
Clipped             = FALSE
Scans               = 8
Total_Scans         = 2048

Relaxation_Delay    = 1.5[s]
Recvr_Gain          = 50
Temp_Get            = 22[dC]
X_Acq_Time          = 0.21823488[s]
X_Atn               = 3.2[dB]
X_Gamma             = 42576375
X_Pulse             = 7.59[us]
```

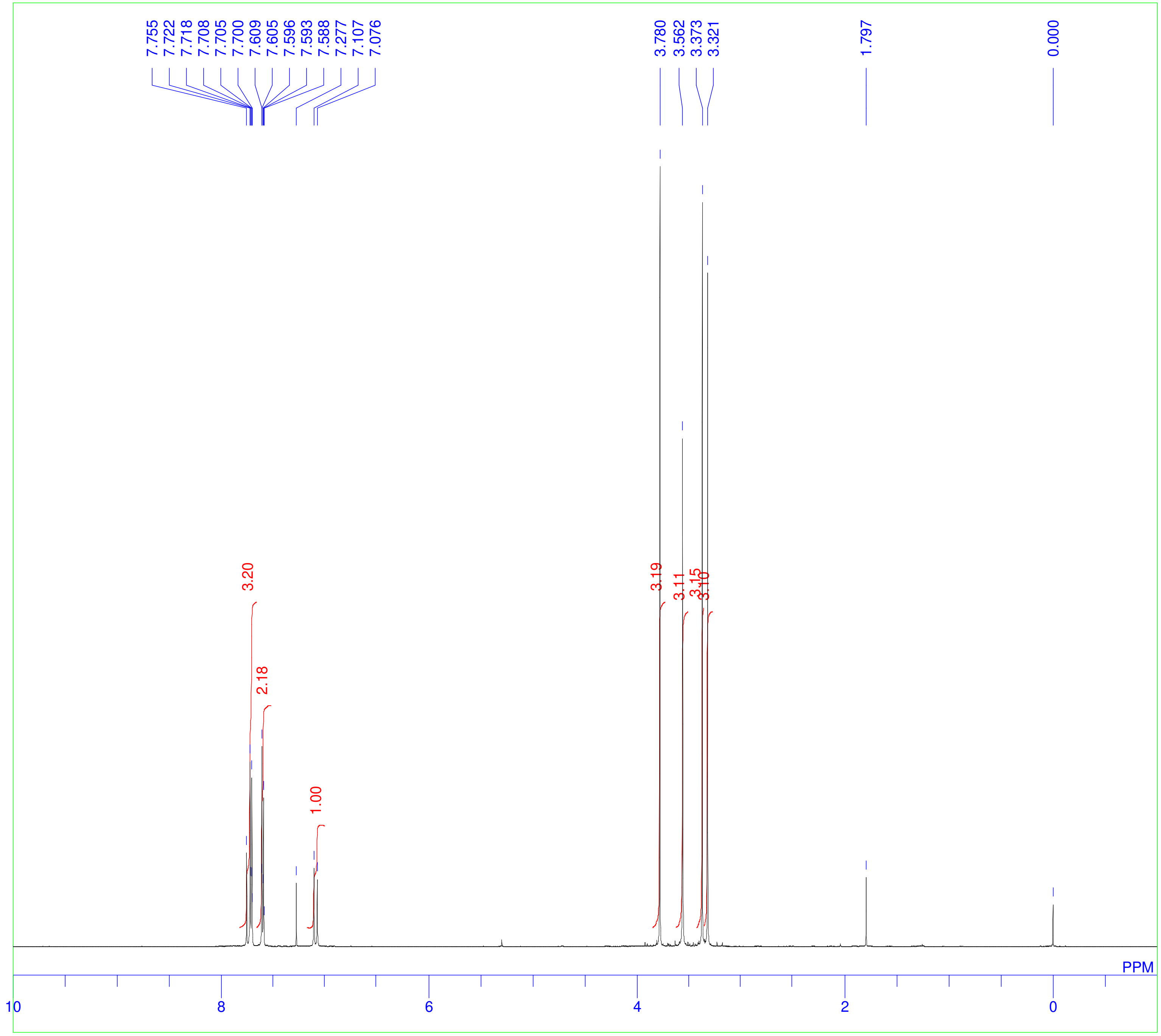

DFILE (E)-20m_1H.als
COMNT
DATIM 2023-08-08 21:47:46
OBNUC 1H
EXMOD proton.jxp
OBFRQ 500.16 MHz
OBSET 2.41 KHz
OBFIN 6.01 Hz
POINT 13107
FREQU 7507.51 Hz
SCANS 8
ACQTM 1.7459 sec
PD 5.0000 sec
PW1 3.84 usec
IRNUC 1H
CTEMP 24.2 c
SLVNT CDCL3
EXREF 0.00 ppm
BF 0.30 Hz
RGAIN 30

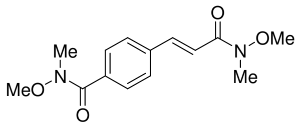

(*E*)-20m

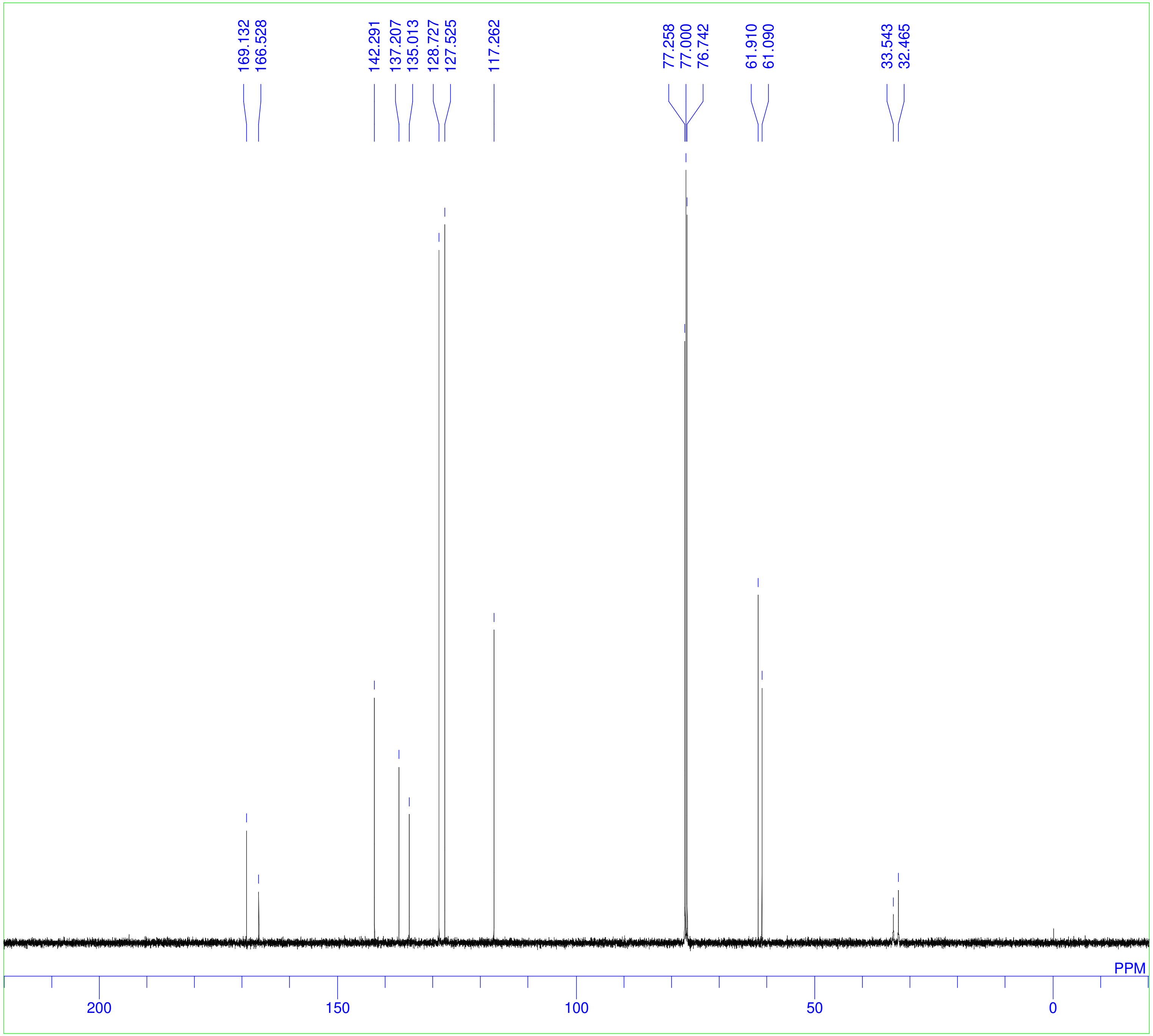

DFILE	(E)-20m_13C.als
COMNT	
DATIM	2023-08-08 20:58:29
OBNUC	13C
EXMOD	carbon.jxp
OBFRQ	125.77 MHz
OBSET	7.87 KHz
OBFIN	4.21 Hz
POINT	26214
FREQU	31446.54 Hz
SCANS	1024
ACQTM	0.8336 sec
PD	2.0000 sec
PW1	3.87 usec
IRNUC	1H
CTEMP	24.4 c
SLVNT	CDCL3
EXREF	77.00 ppm
BF	0.30 Hz
RGAIN	32

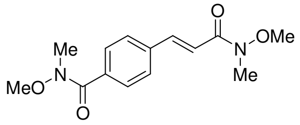

(*E*)-20m

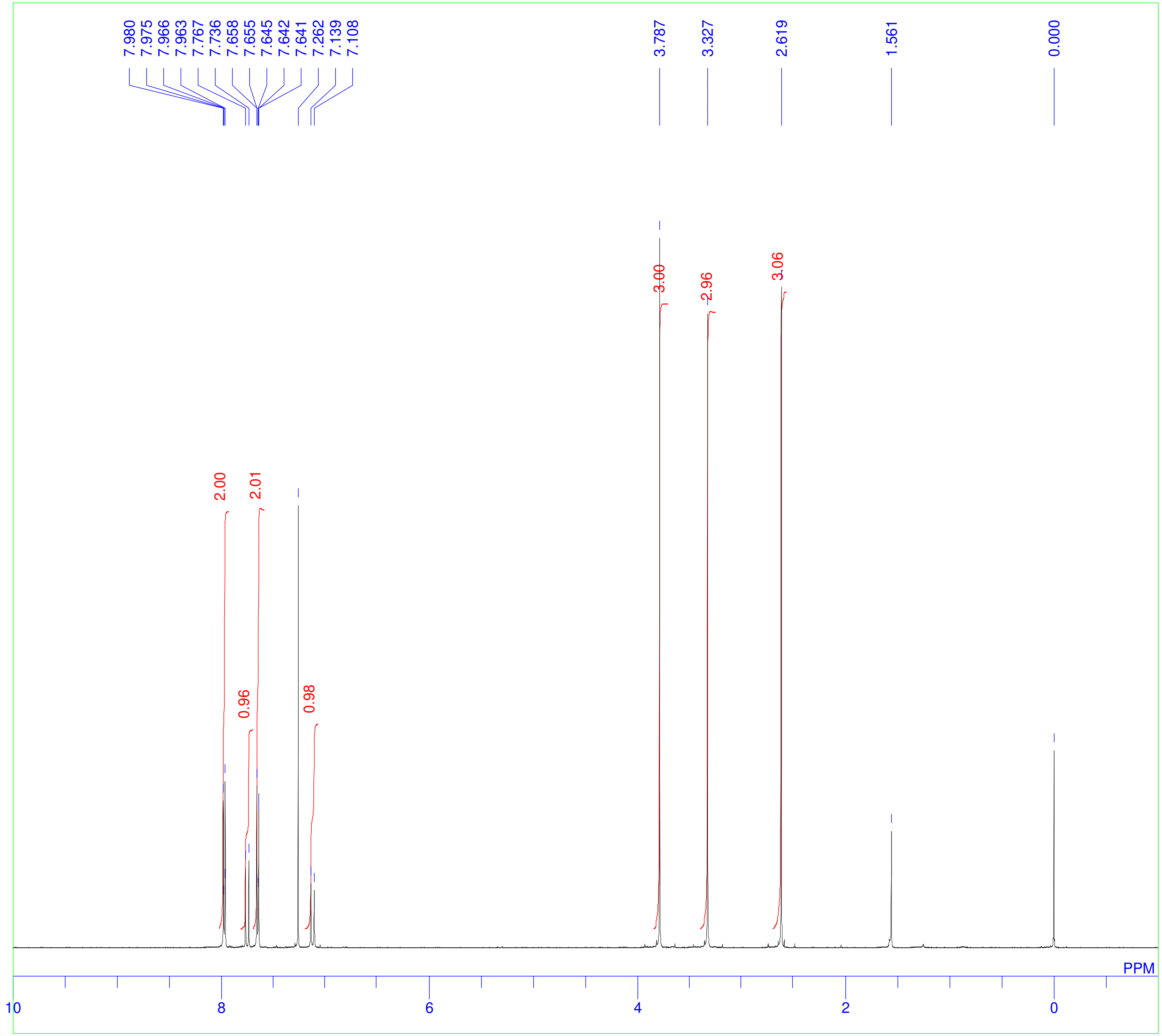

DFILE	(E)-20n_1H.als
COMNT	
DATIM	2023-04-12 13:17:35
OBNUC	1H
EXMOD	proton.jxp
OBFRQ	500.16 MHz
OBSET	2.41 KHz
OBFIN	6.01 Hz
POINT	13107
FREQU	7507.51 Hz
SCANS	8
ACQTM	1.7459 sec
PD	5.0000 sec
PW1	3.84 usec
IRNUC	1H
CTEMP	24.2 c
SLVNT	CDCL3
EXREF	0.00 ppm
BF	0.30 Hz
RGAIN	48

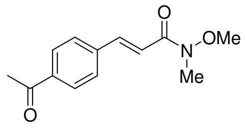

(*E*)-20n

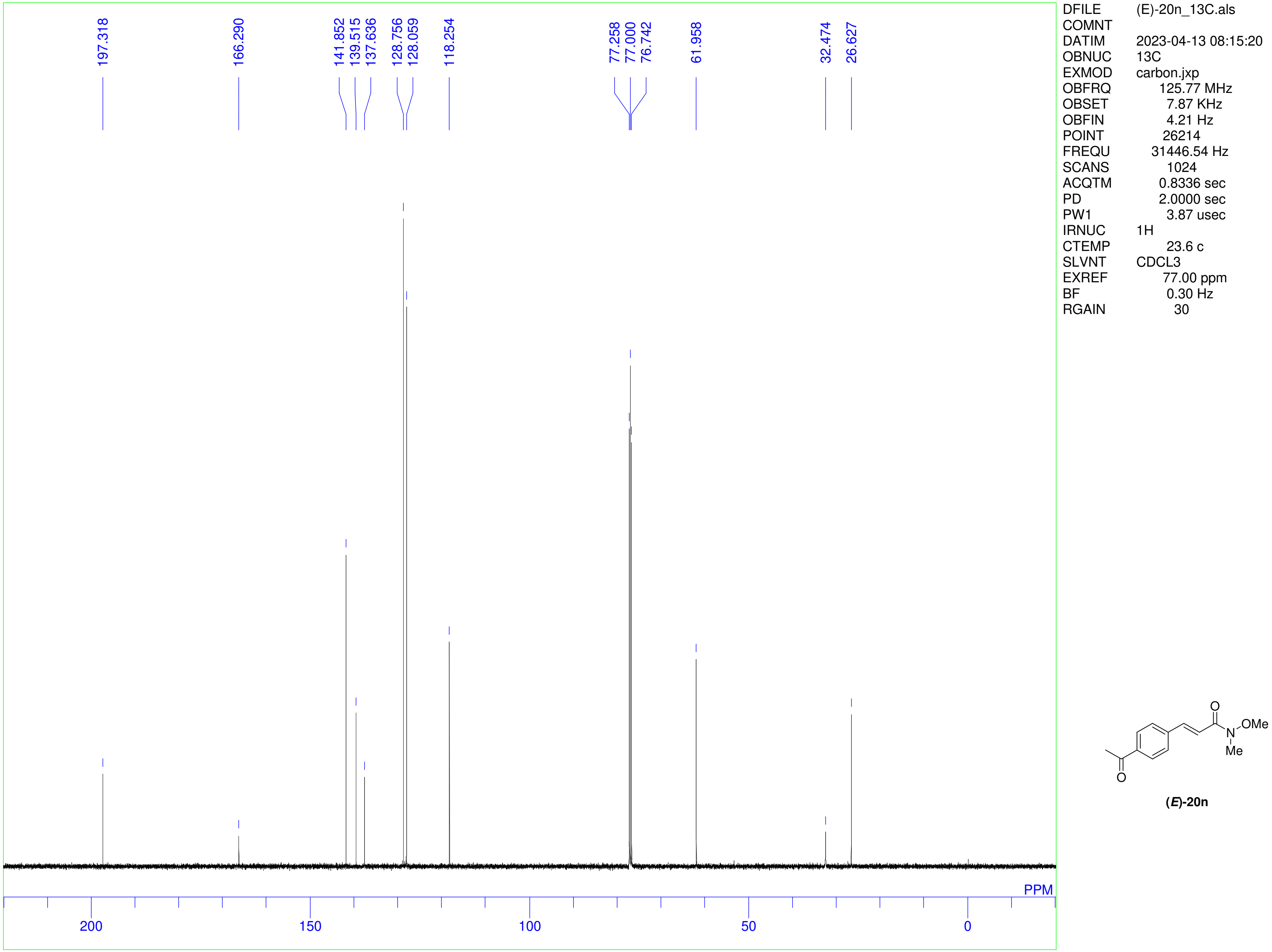
197.318
166.290
141.852
139.515
137.636
128.756
128.059
118.254
77.258
77.000
76.742
61.958
32.474
26.627
PPM
200
150
100
50
0
DFILE (E)-20n_13C.als
COMNT
DATIM 2023-04-13 08:15:20
OBNUC 13C
EXMOD carbon.jxp
OBFRQ 125.77 MHz
OBSET 7.87 KHz
OBFIN 4.21 Hz
POINT 26214
FREQU 31446.54 Hz
SCANS 1024
ACQTM 0.8336 sec
PD 2.0000 sec
PW1 3.87 usec
IRNUC 1H
CTEMP 23.6 c
SLVNT CDCL3
EXREF 77.00 ppm
BF 0.30 Hz
RGAIN 30
O
N
OMe
Me
O

(*E*)-20n

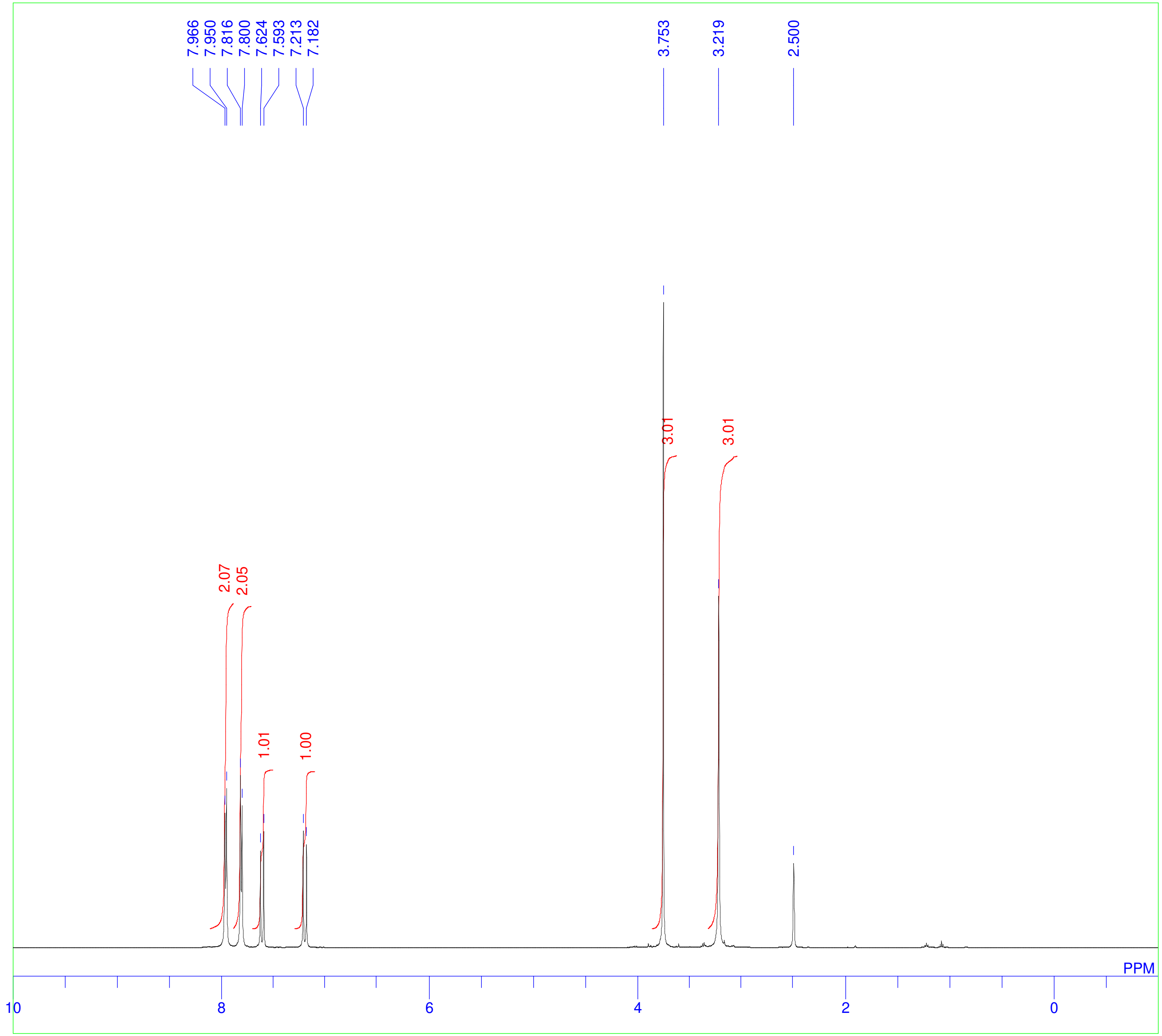

DFILE (E)-20o_1H.als
COMNT
DATIM 2024-01-16 22:10:45
OBNUC 1H
EXMOD proton.jxp
OBFRQ 500.16 MHz
OBSET 2.41 KHz
OBFIN 6.01 Hz
POINT 13107
FREQU 7507.51 Hz
SCANS 16
ACQTM 1.7459 sec
PD 5.0000 sec
PW1 3.80 usec
IRNUC 1H
CTEMP 20.4 c
SLVNT DMSO
EXREF 2.50 ppm
BF 0.30 Hz
RGAIN 30

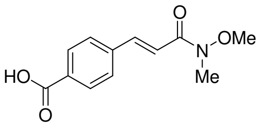

(*E*)-20o

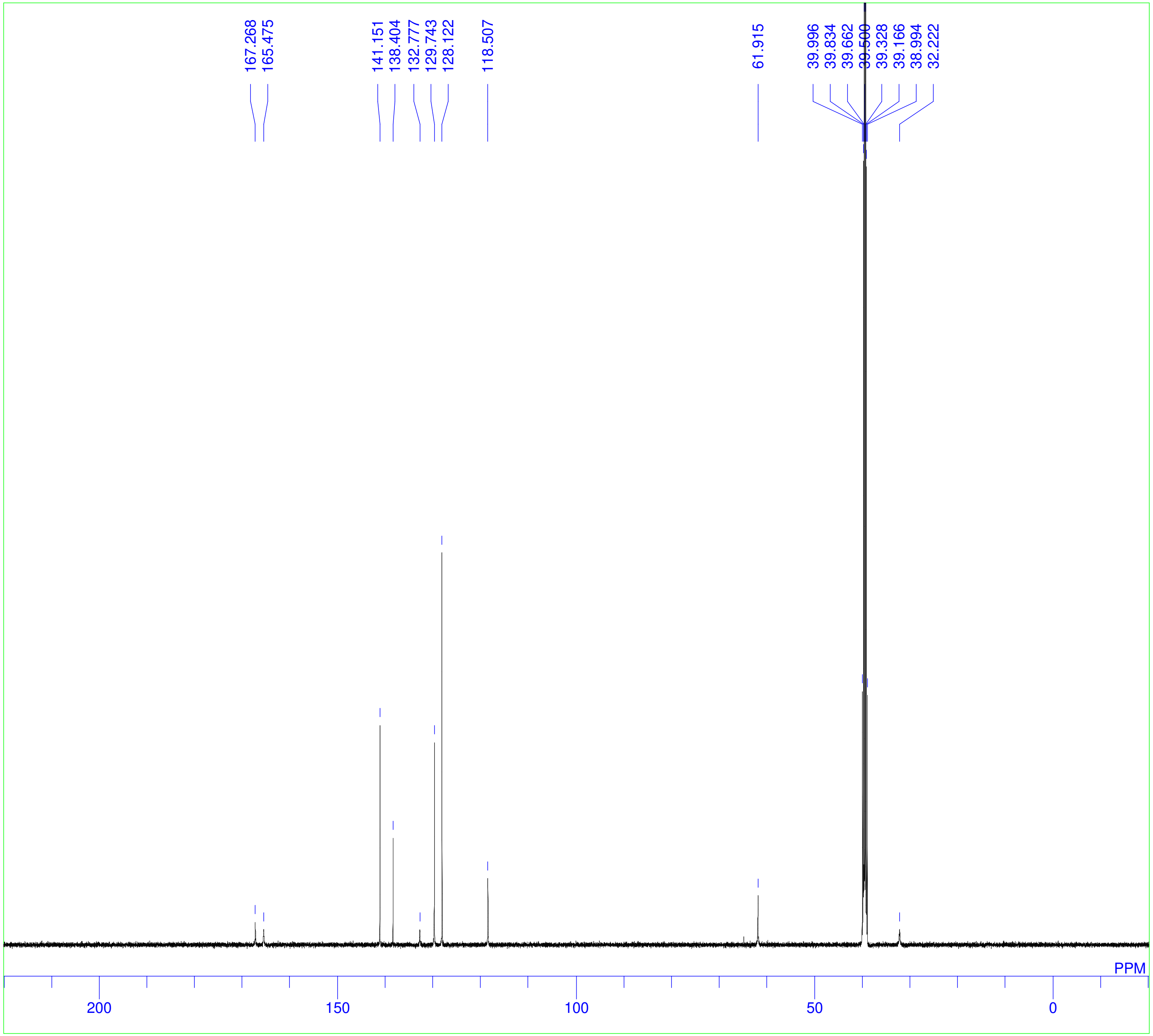

DFILE	(E)-20o_13C.als
COMNT	
DATIM	2024-01-16 22:14:04
OBNUC	13C
EXMOD	carbon.jxp
OBFRQ	125.77 MHz
OBSET	7.87 KHz
OBFIN	4.21 Hz
POINT	26214
FREQU	31446.54 Hz
SCANS	4096
ACQTM	0.8336 sec
PD	2.0000 sec
PW1	4.30 usec
IRNUC	1H
CTEMP	20.1 c
SLVNT	DMSO
EXREF	39.50 ppm
BF	0.30 Hz
RGAIN	26

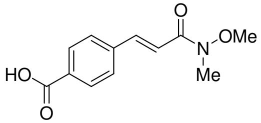

(*E*)-20o

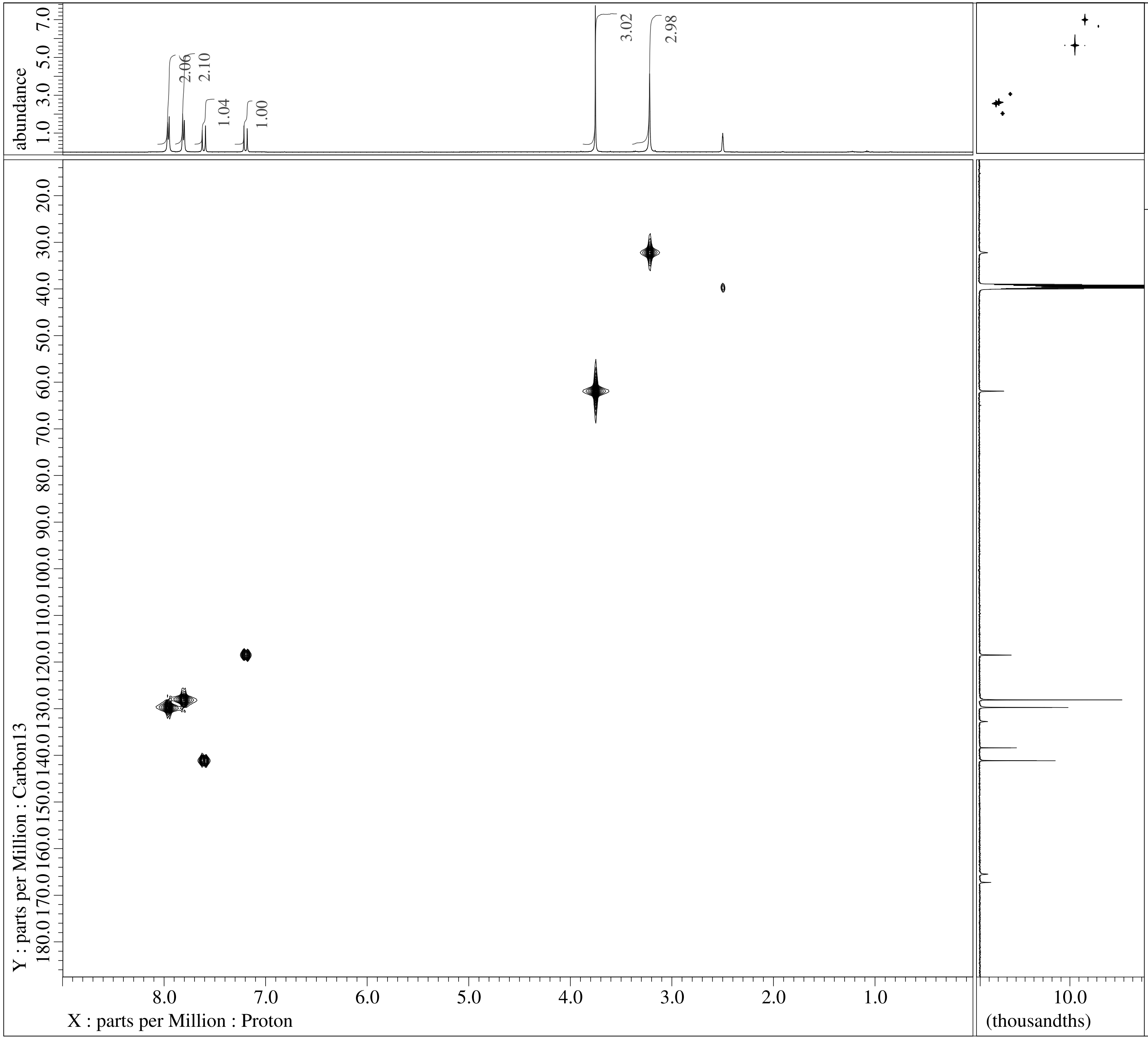


```
---- PROCESSING PARAMETERS ----
sinbell4( -60, 160 )
zerofill( 1 )
fft( 1, TRUE, TRUE )
ppm
[transpose]
sinbell4( -60, 160 )
zerofill( 2 )
fft( 1, TRUE, TRUE )
ppm
abs
```

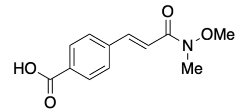

(*E*)-20o

```
Filename            = HT-1679-TM_DMSO-D6_HMQC
Author              = delta
Experiment          = hmqc.jxp
Sample_Id           = HT-1679-TM_DMSO-D6
Solvent             = DMSO-D6
Creation_Time       = 17-JAN-2024 01:28:01
Revision_Time       = 11-APR-2024 10:18:34
Current_Time        = 11-APR-2024 10:19:26

Comment             = gradient enhanced HMQC
Data_Format         = 2D REAL REAL
Dim_Size            = 819, 512
Dim_Title           = Proton Carbon13
Dim_Units           = [ppm] [ppm]
Dimensions          = X Y
Site                = JNM-ECA500II
Spectrometer        = DELTA2_NMR

Field_Strength      = 11.7473579[T] (500[MHz]
X_Acq_Duration      = 0.18251776[s]
X_Domain            = 1H
X_Freq              = 500.15991521[MHz]
X_Offset            = 4.47664887[ppm]
X_Points            = 1024
X_Prescans          = 4
X_Resolution        = 5.47891887[Hz]
X_Sweep             = 5.61041293[kHz]
X_Sweep_Clipped     = 4.48833034[kHz]
Y_Domain            = 13C
Y_Freq              = 125.76529768[MHz]
Y_Offset            = 100.25753479[ppm]
Y_Points            = 256
Y_Prescans          = 0
Y_Resolution        = 86.26877208[Hz]
Y_Sweep             = 22.08480565[kHz]
Tri_Domain          = Proton
Tri_Freq            = 500.15991521[MHz]
Tri_Offset          = 5.0[ppm]
Clipped             = FALSE
Scans               = 32
Total_Scans         = 8192

Relaxation_Delay    = 1.5[s]
Recvr_Gain          = 50
Temp_Get            = 19.8[dC]
X_Acq_Time          = 0.18251776[s]
X_Atn               = 3.2[dB]
X_Gamma             = 42576375
X_Pulse             = 7.59[us]
```

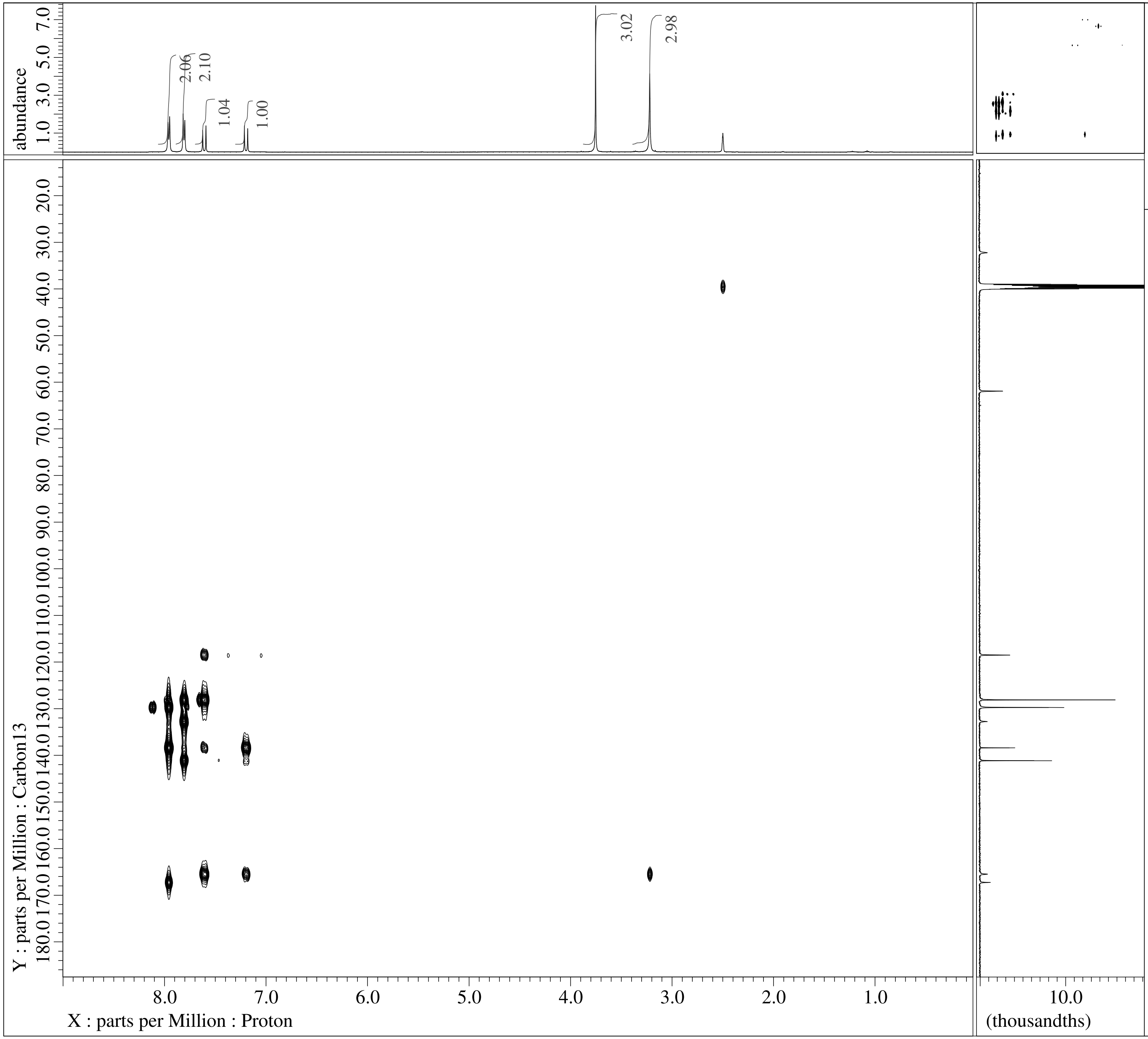

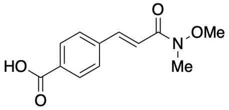
(E)-20o

```
---- PROCESSING PARAMETERS ----
gauss( 5.0[Hz], 0.0[s]
sinbell_auto
zerofill( 1 )
fft( 1, TRUE, TRUE )
ppm
[transpose]
sinbell4( -60, 160 )
trapezoid( 0[%], 5[%], 80[%], 100[%] )
zerofill( 2 )
fft( 1, TRUE, TRUE )
```

```
Filename            = HT-1679-TM_DMSO-D6_HMBC
Author              = delta
Experiment          = hmbc.jxp
Sample_Id           = HT-1679-TM_DMSO-D6
Solvent             = DMSO-D6
Creation_Time       = 17-JAN-2024 05:20:23
Revision_Time       = 11-APR-2024 10:20:55
Current_Time        = 11-APR-2024 10:21:30

Comment             = gradient enhanced HMBC
Data_Format         = 2D REAL REAL
Dim_Size            = 1638, 512
Dim_Title           = Proton Carbon13
Dim_Units           = [ppm] [ppm]
Dimensions          = X Y
Site                = JNM-ECA500II
Spectrometer        = DELTA2_NMR

Field_Strength      = 11.7473579[T] (500[MHz]
X_Acq_Duration      = 0.36503552[s]
X_Domain            = 1H
X_Freq              = 500.15991521[MHz]
X_Offset            = 4.47664887[ppm]
X_Points            = 2048
X_Prescans          = 4
X_Resolution        = 2.73945944[Hz]
X_Sweep             = 5.61041293[kHz]
X_Sweep_Clipped     = 4.48833034[kHz]
Y_Domain            = 13C
Y_Freq              = 125.76529768[MHz]
Y_Offset            = 100.25753479[ppm]
Y_Points            = 256
Y_Prescans          = 0
Y_Resolution        = 86.26877208[Hz]
Y_Sweep             = 22.08480565[kHz]
Tri_Domain          = Proton
Tri_Freq            = 500.15991521[MHz]
Tri_Offset          = 5.0[ppm]
Clipped             = FALSE
Scans               = 32
Total_Scans         = 8192

Relaxation_Delay    = 1.5[s]
Recvr_Gain          = 50
Temp_Get            = 19.3[dC]
X_Acq_Time          = 0.36503552[s]
X_Atn               = 3.2[dB]
X_Gamma             = 42576375
X_Pulse             = 7.59[us]
```

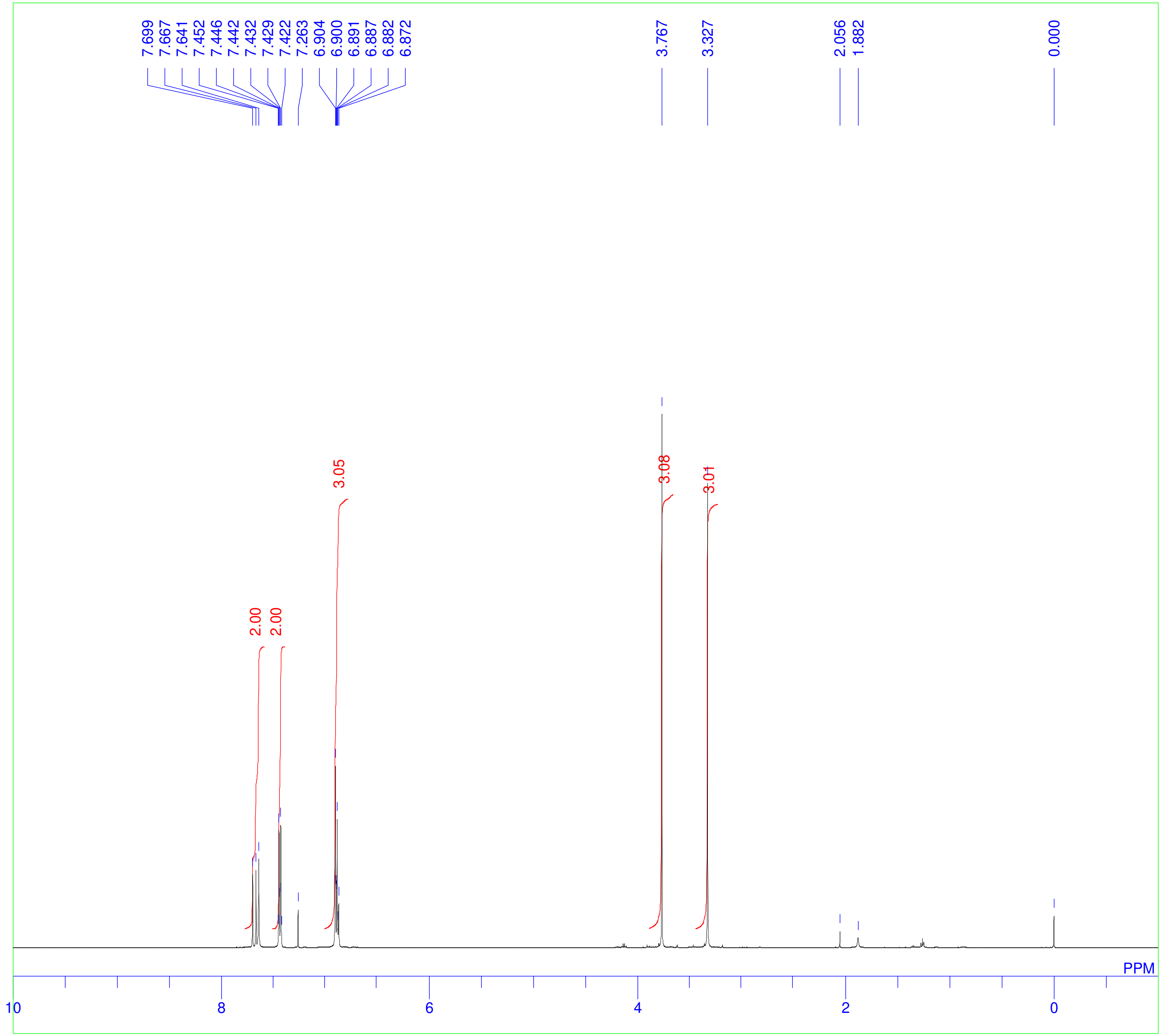

DFILE	(E)-20p_1H.als
COMNT	
DATIM	2023-07-28 18:24:02
OBNUC	1H
EXMOD	proton.jxp
OBFRQ	500.16 MHz
OBSET	2.41 KHz
OBFIN	6.01 Hz
POINT	13107
FREQU	7507.51 Hz
SCANS	8
ACQTM	1.7459 sec
PD	5.0000 sec
PW1	3.84 usec
IRNUC	1H
CTEMP	24.2 c
SLVNT	CDCL3
EXREF	0.00 ppm
BF	0.30 Hz
RGAIN	34

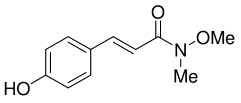

(*E*)-20p

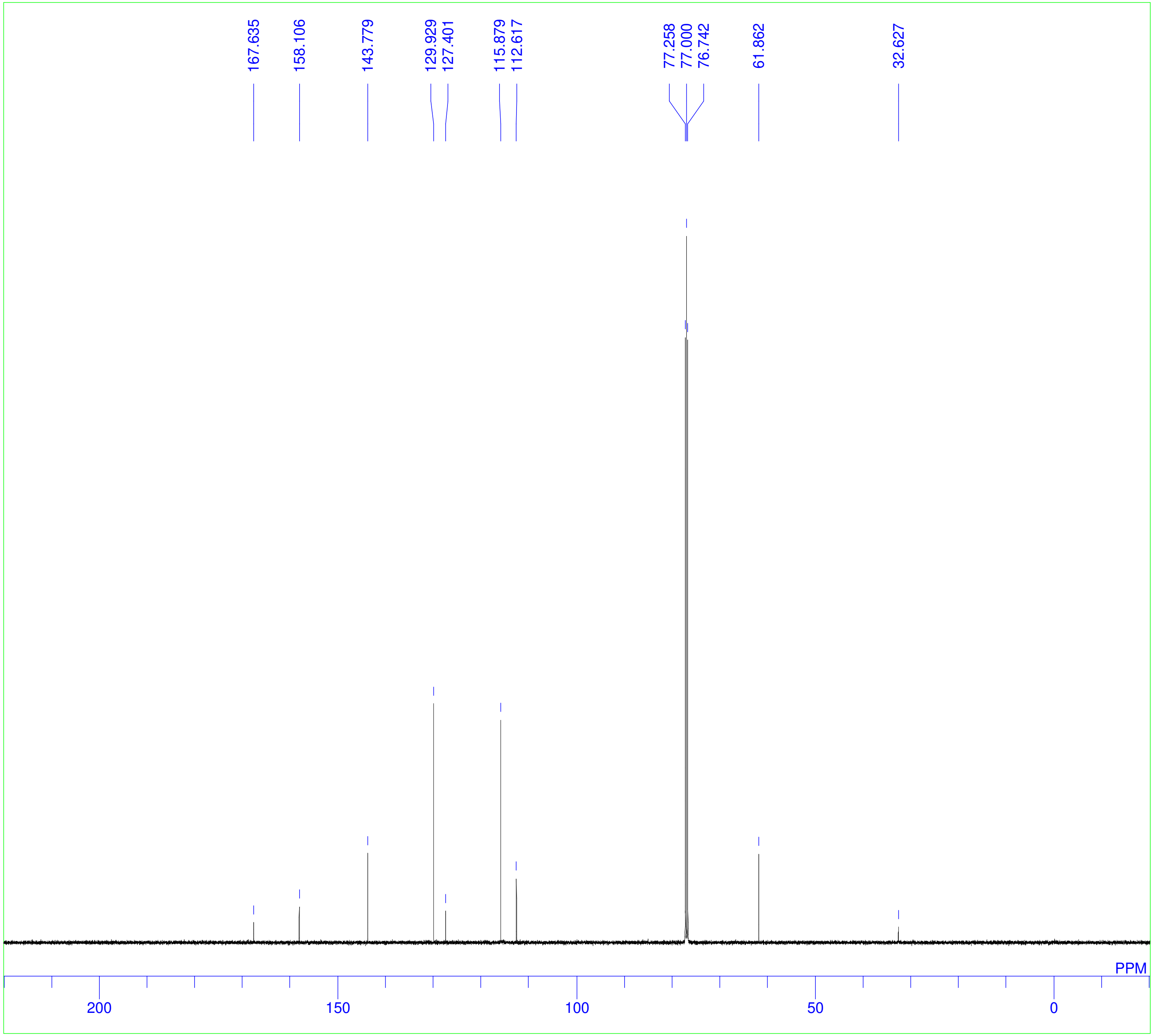

DFILE (E)-20p_13C.als
COMNT
DATIM 2023-05-31 22:06:49
OBNUC 13C
EXMOD carbon.jxp
OBFRQ 125.77 MHz
OBSET 7.87 KHz
OBFIN 4.21 Hz
POINT 26214
FREQU 31446.54 Hz
SCANS 4096
ACQTM 0.8336 sec
PD 2.0000 sec
PW1 3.87 usec
IRNUC 1H
CTEMP 24.1 c
SLVNT CDCL3
EXREF 77.00 ppm
BF 0.30 Hz
RGAIN 30

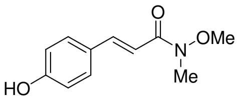

(*E*)-20p

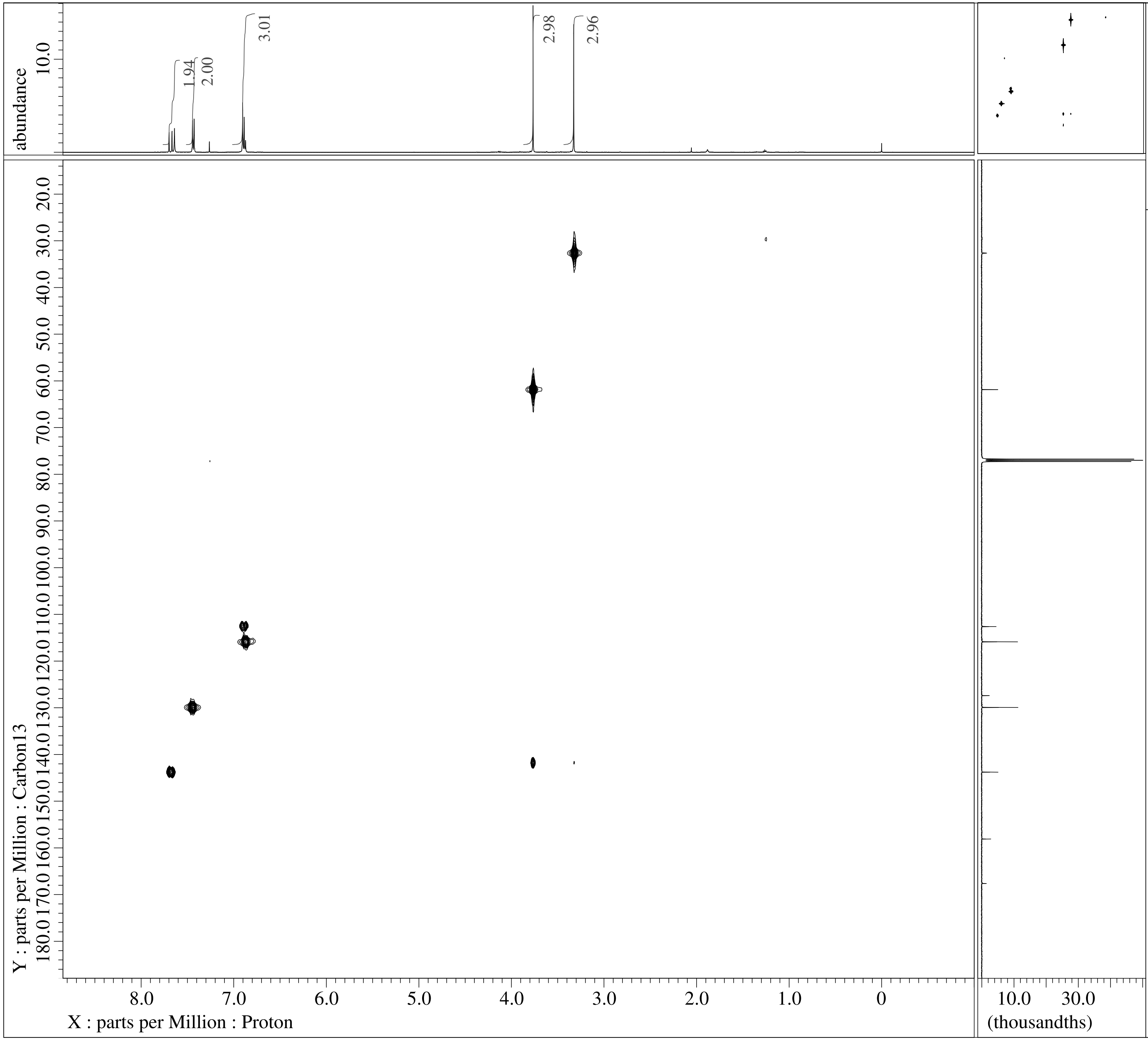


```
---- PROCESSING PARAMETERS ----
sinbell4( -60, 160 )
zerofill( 1 )
fft( 1, TRUE, TRUE )
ppm
[transpose]
sinbell4( -60, 160 )
zerofill( 2 )
fft( 1, TRUE, TRUE )
ppm
abs
```

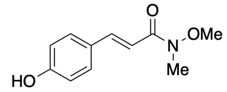

(*E*)-20p

```
Filename            = HT-1466-PTLC 2_13C_HMQC
Author              = delta
Experiment          = hmqc.jxp
Sample_Id           = HT-1466-PTLC 2_13C
Solvent             = CHLOROFORM-D
Creation_Time       =  1-JUN-2023 01:20:46
Revision_Time       = 11-APR-2024 10:24:42
Current_Time        = 11-APR-2024 10:25:45

Comment             = gradient enhanced HMQC
Data_Format         = 2D REAL REAL
Dim_Size            = 819, 512
Dim_Title           = Proton Carbon13
Dim_Units           = [ppm] [ppm]
Dimensions          = X Y
Site                = JNM-ECA500II
Spectrometer        = DELTA2_NMR

Field_Strength      = 11.7473579[T] (500[MHz]
X_Acq_Duration      = 0.16367616[s]
X_Domain            = 1H
X_Freq              = 500.15991521[MHz]
X_Offset            = 3.83419814[ppm]
X_Points            = 1024
X_Prescans          = 4
X_Resolution        = 6.10962525[Hz]
X_Sweep             = 6.25625626[kHz]
X_Sweep_Clipped     = 5.00500501[kHz]
Y_Domain            = 13C
Y_Freq              = 125.76529768[MHz]
Y_Offset            = 100.23368897[ppm]
Y_Points            = 256
Y_Prescans          = 0
Y_Resolution        = 86.26877208[Hz]
Y_Sweep             = 22.08480565[kHz]
Tri_Domain          = Proton
Tri_Freq            = 500.15991521[MHz]
Tri_Offset          = 5.0[ppm]
Clipped             = FALSE
Scans               = 32
Total_Scans         = 8192

Relaxation_Delay    = 1.5[s]
Recvr_Gain          = 50
Temp_Get            = 24.1[dC]
X_Acq_Time          = 0.16367616[s]
X_Atn               = 3.2[dB]
X_Gamma             = 42576375
X_Pulse             = 7.68[us]
```

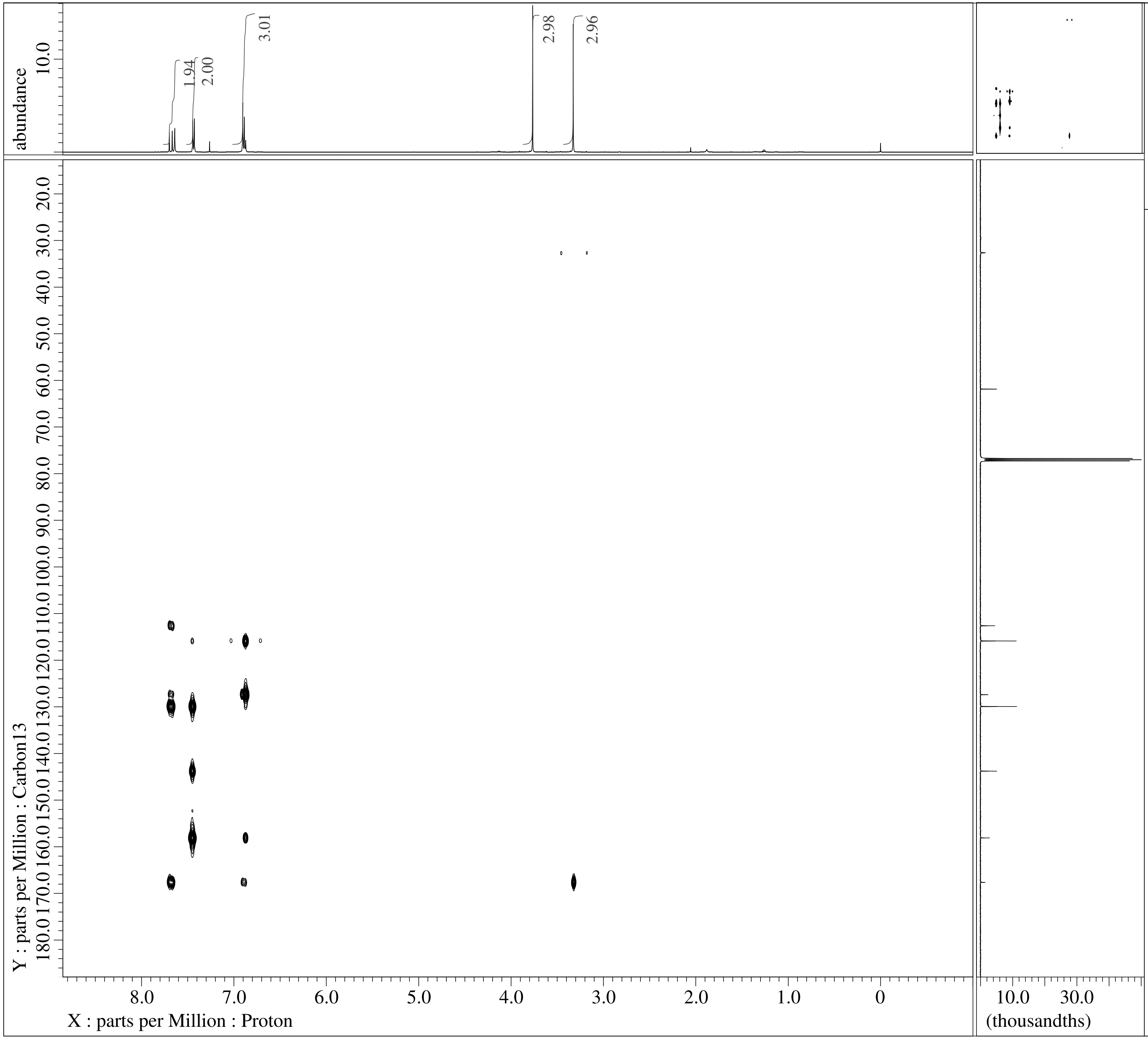


```
---- PROCESSING PARAMETERS ----
gauss( 5.0[Hz], 0.0[s] )
sinbell_auto
zerofill( 1 )
fft( 1, TRUE, TRUE )
ppm
[transpose]
sinbell4( -60, 160 )
trapezoid( 0[%], 5[%], 80[%], 100[%] )
zerofill( 2 )
fft( 1, TRUE, TRUE )
```

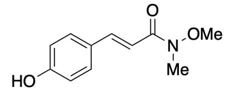

(E)-20p

```
Filename            = HT-1466-PTLC 2_13C_HMBC
Author              = delta
Experiment          = hmbc.jxp
Sample_Id           = HT-1466-PTLC 2_13C
Solvent             = CHLOROFORM-D
Creation_Time       =  1-JUN-2023 05:10:33
Revision_Time       = 11-APR-2024 10:27:21
Current_Time        = 11-APR-2024 10:27:51

Comment             = gradient enhanced HMBC
Data_Format         = 2D REAL REAL
Dim_Size            = 1638, 512
Dim_Title           = Proton Carbon13
Dim_Units           = [ppm] [ppm]
Dimensions          = X Y
Site                = JNM-ECA500II
Spectrometer        = DELTA2_NMR

Field_Strength      = 11.7473579[T] (500[MHz]
X_Acq_Duration      = 0.32735232[s]
X_Domain            = 1H
X_Freq              = 500.15991521[MHz]
X_Offset            = 3.83419814[ppm]
X_Points            = 2048
X_Prescans          = 4
X_Resolution        = 3.05481263[Hz]
X_Sweep             = 6.25625626[kHz]
X_Sweep_Clipped     = 5.00500501[kHz]
Y_Domain            = 13C
Y_Freq              = 125.76529768[MHz]
Y_Offset            = 100.23368897[ppm]
Y_Points            = 256
Y_Prescans          = 0
Y_Resolution        = 86.26877208[Hz]
Y_Sweep             = 22.08480565[kHz]
Tri_Domain          = Proton
Tri_Freq            = 500.15991521[MHz]
Tri_Offset          = 5.0[ppm]
Clipped             = FALSE
Scans               = 16
Total_Scans         = 4096

Relaxation_Delay    = 1.5[s]
Recvr_Gain          = 50
Temp_Get            = 23.7[dC]
X_Acq_Time          = 0.32735232[s]
X_Atn               = 3.2[dB]
X_Gamma             = 42576375
X_Pulse             = 7.68[us]
```

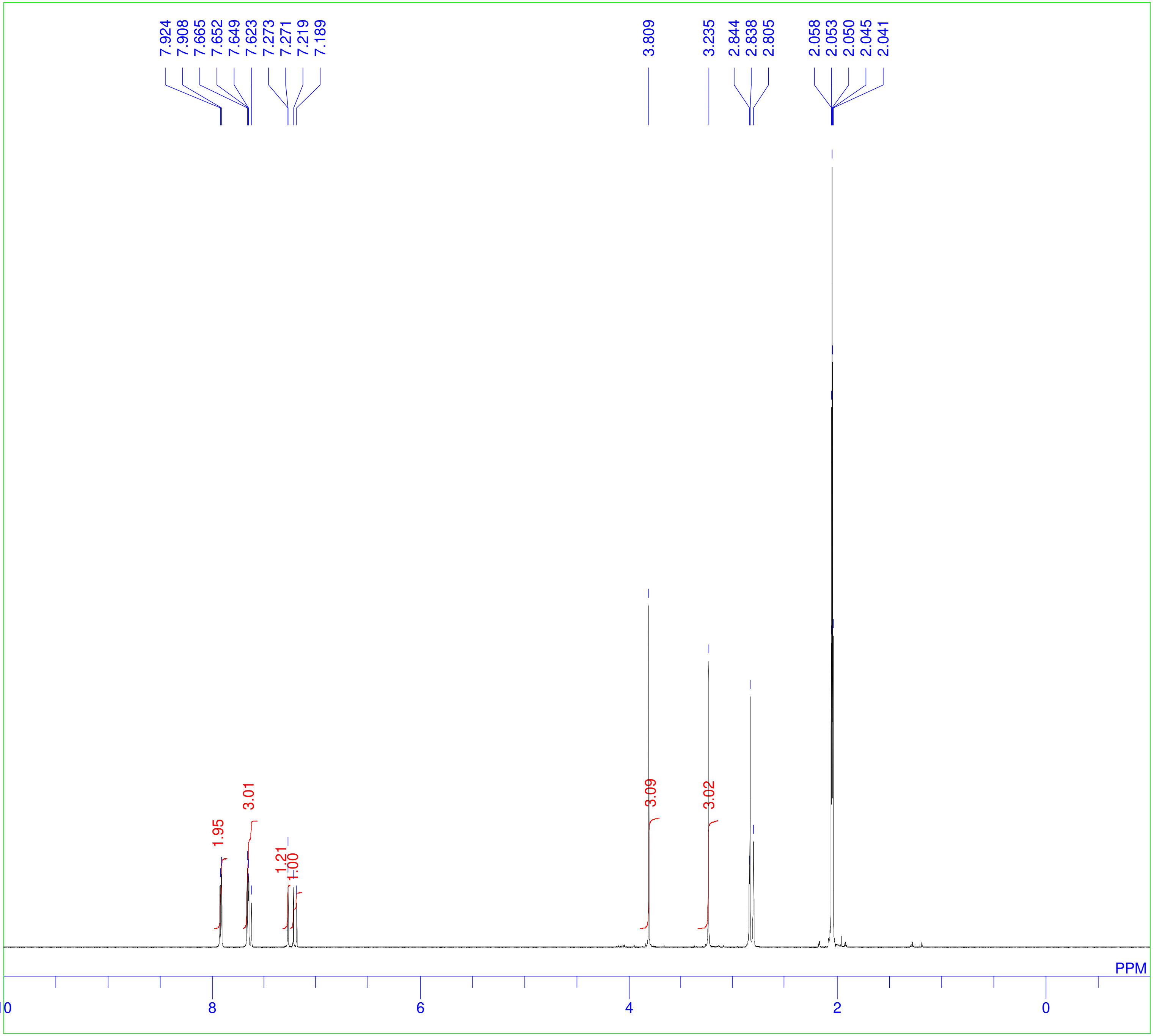

DFILE	(E)-20q_1H.als
COMNT	
DATIM	2024-02-08 18:14:47
OBNUC	1H
EXMOD	proton.jxp
OBFRQ	500.16 MHz
OBSET	2.41 KHz
OBFIN	6.01 Hz
POINT	13107
FREQU	7507.51 Hz
SCANS	8
ACQTM	1.7459 sec
PD	5.0000 sec
PW1	3.80 usec
IRNUC	1H
CTEMP	20.3 c
SLVNT	ACETN
EXREF	2.05 ppm
BF	0.30 Hz
RGAIN	48

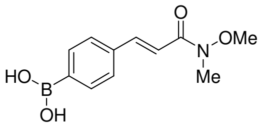

(*E*)-20q

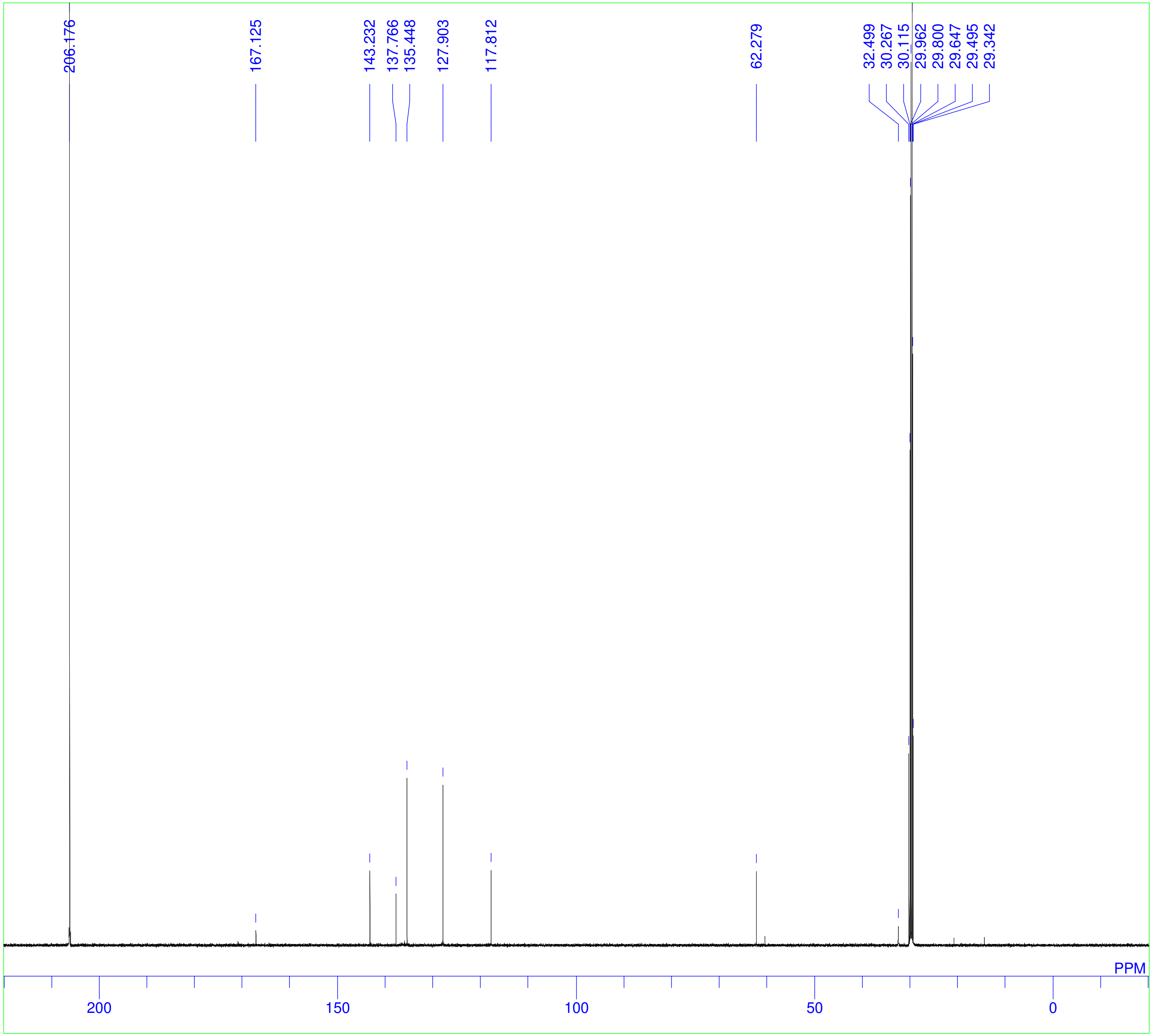

DFILE	(E)-20q_13C.als
COMNT	
DATIM	2024-02-08 18:59:30
OBNUC	13C
EXMOD	carbon.jxp
OBFRQ	125.77 MHz
OBSET	7.87 KHz
OBFIN	4.21 Hz
POINT	26214
FREQU	31446.54 Hz
SCANS	2048
ACQTM	0.8336 sec
PD	2.0000 sec
PW1	4.30 usec
IRNUC	1H
CTEMP	20.2 c
SLVNT	ACETN
EXREF	29.80 ppm
BF	0.30 Hz
RGAIN	24

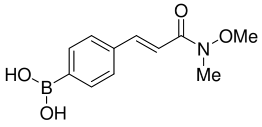

(*E*)-20q

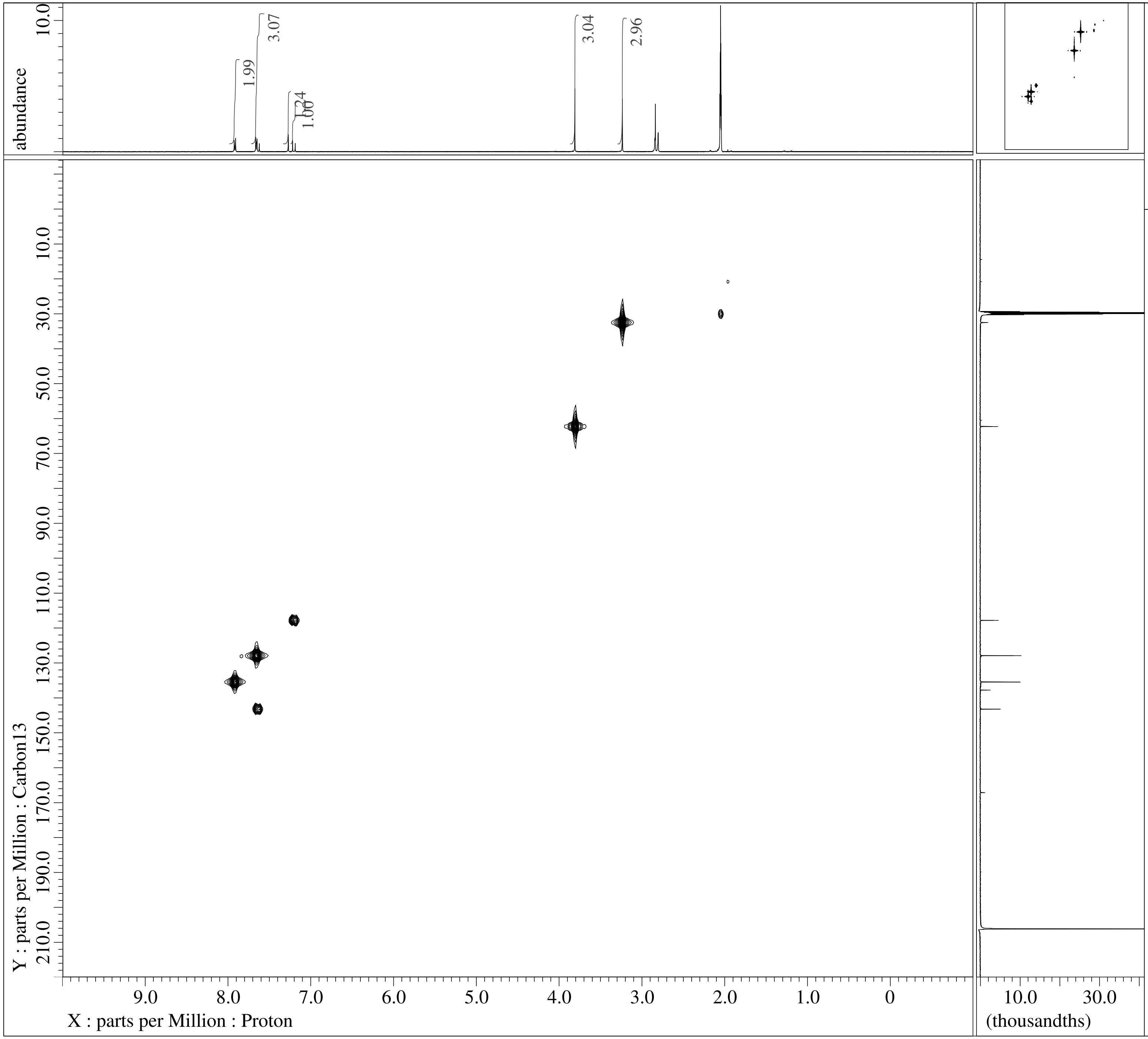


```
---- PROCESSING PARAMETERS ----
sinbell4( -60, 160 )
zerofill( 1 )
fft( 1, TRUE, TRUE )
ppm
[transpose]
sinbell4( -60, 160 )
zerofill( 2 )
fft( 1, TRUE, TRUE )
ppm
abs
```

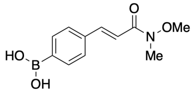

(*E*)-20q

```
Filename            = HT-1730-TM_13C_acetone-
Author              = delta
Experiment          = hmqc.jxp
Sample_Id           = HT-1730-TM_13C_acetone-
Solvent             = ACETONE-D6
Creation_Time       =  8-FEB-2024 20:36:42
Revision_Time       = 11-APR-2024 10:30:28
Current_Time        = 11-APR-2024 10:31:09

Comment             = gradient enhanced HMQC
Data_Format         = 2D REAL REAL
Dim_Size            = 819, 512
Dim_Title           = Proton Carbon13
Dim_Units           = [ppm] [ppm]
Dimensions          = X Y
Site                = JNM-ECA500II
Spectrometer        = DELTA2_NMR

Field_Strength      = 11.7473579[T] (500[MHz]
X_Acq_Duration      = 0.10911744[s]
X_Domain            = 1H
X_Freq              = 500.15991521[MHz]
X_Offset            = 5.0[ppm]
X_Points            = 1024
X_Prescans          = 4
X_Resolution        = 9.16443788[Hz]
X_Sweep             = 9.38438438[kHz]
X_Sweep_Clipped     = 7.50750751[kHz]
Y_Domain            = 13C
Y_Freq              = 125.76529768[MHz]
Y_Offset            = 104.95516005[ppm]
Y_Points            = 256
Y_Prescans          = 0
Y_Resolution        = 118.22790557[Hz]
Y_Sweep             = 30.26634383[kHz]
Tri_Domain          = Proton
Tri_Freq            = 500.15991521[MHz]
Tri_Offset          = 5.0[ppm]
Clipped             = FALSE
Scans               = 8
Total_Scans         = 2048

Relaxation_Delay    = 1.5[s]
Recvr_Gain          = 50
Temp_Get            = 20[dC]
X_Acq_Time          = 0.10911744[s]
X_Atn               = 3.2[dB]
X_Gamma             = 42576375
X_Pulse             = 7.59[us]
```

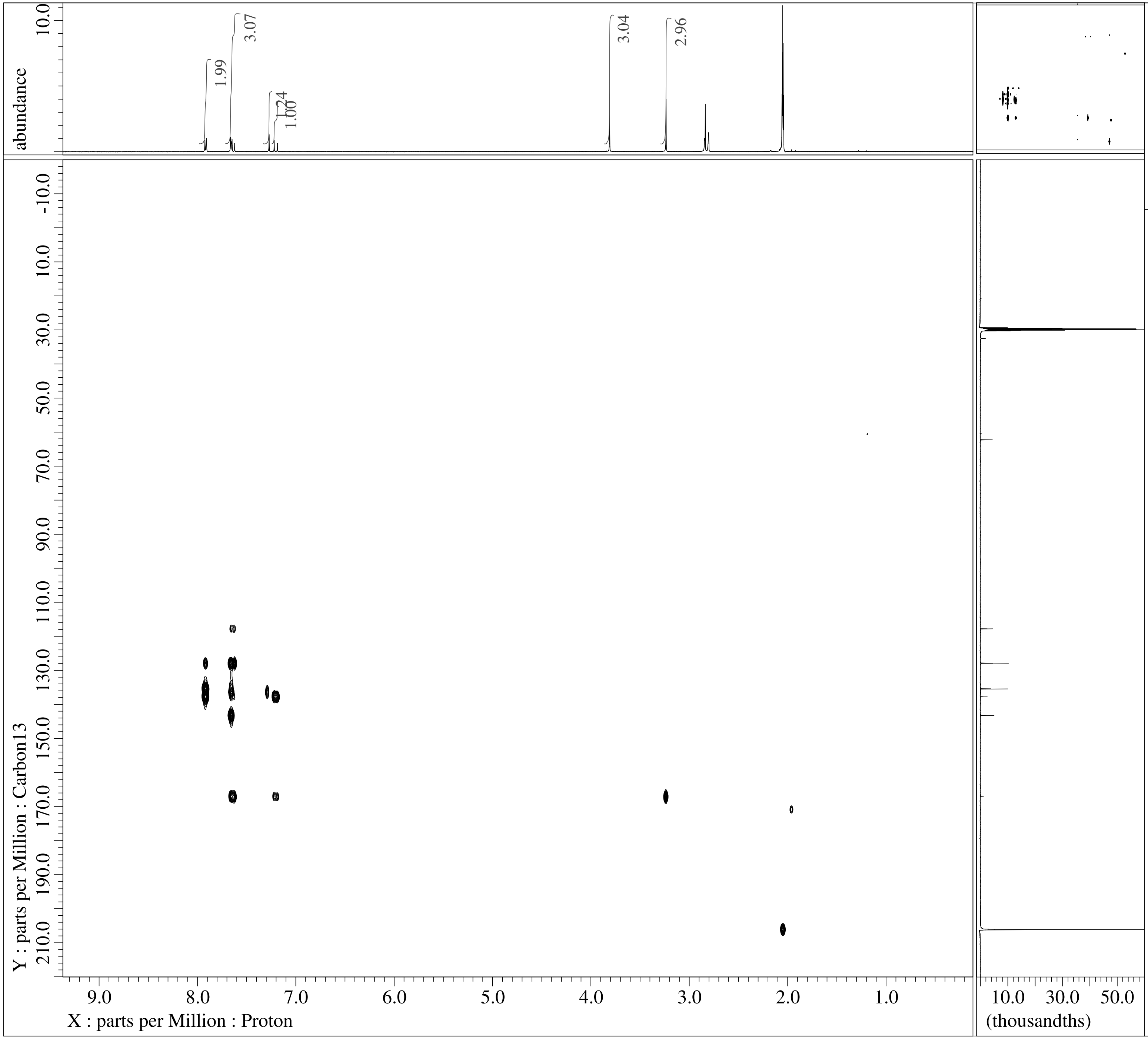


```
---- PROCESSING PARAMETERS ----
gauss( 5.0[Hz], 0.0[s] )
sinbell_auto
zerofill( 1 )
fft( 1, TRUE, TRUE )
ppm
[transpose]
sinbell4( -60, 160 )
trapezoid( 0[%], 5[%], 80[%], 100[%] )
zerofill( 2 )
fft( 1, TRUE, TRUE )
```

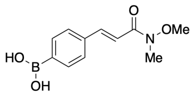

(*E*)-20q

```
Filename            = HT-1730-TM_HMBC_HMBC-1-
Author              = delta
Experiment          = hmbc.jxp
Sample_Id           = HT-1730-TM_HMBC
Solvent             = ACETONE-D6
Creation_Time       =  9-FEB-2024 11:39:20
Revision_Time       = 11-APR-2024 10:32:28
Current_Time        = 11-APR-2024 10:33:24

Comment             = gradient enhanced HMBC
Data_Format         = 2D REAL REAL
Dim_Size            = 1638, 512
Dim_Title           = Proton Carbon13
Dim_Units           = [ppm] [ppm]
Dimensions          = X Y
Site                = JNM-ECA500II
Spectrometer        = DELTA2_NMR

Field_Strength      = 11.7473579[T] (500[MHz]
X_Acq_Duration      = 0.35373056[s]
X_Domain            = 1H
X_Freq              = 500.15991521[MHz]
X_Offset            = 4.71026732[ppm]
X_Points            = 2048
X_Prescans          = 4
X_Resolution        = 2.82701048[Hz]
X_Sweep             = 5.78971746[kHz]
X_Sweep_Clipped     = 4.63177397[kHz]
Y_Domain            = 13C
Y_Freq              = 125.76529768[MHz]
Y_Offset            = 100[ppm]
Y_Points            = 256
Y_Prescans          = 0
Y_Resolution        = 122.99275819[Hz]
Y_Sweep             = 31.4861461[kHz]
Tri_Domain          = Proton
Tri_Freq            = 500.15991521[MHz]
Tri_Offset          = 5.0[ppm]
Clipped             = FALSE
Scans               = 8
Total_Scans         = 2048

Relaxation_Delay    = 1.5[s]
Recvr_Gain          = 50
Temp_Get            = 19.6[dC]
X_Acq_Time          = 0.35373056[s]
X_Atn               = 3.2[dB]
X_Gamma             = 42576375
X_Pulse             = 7.59[us]
```

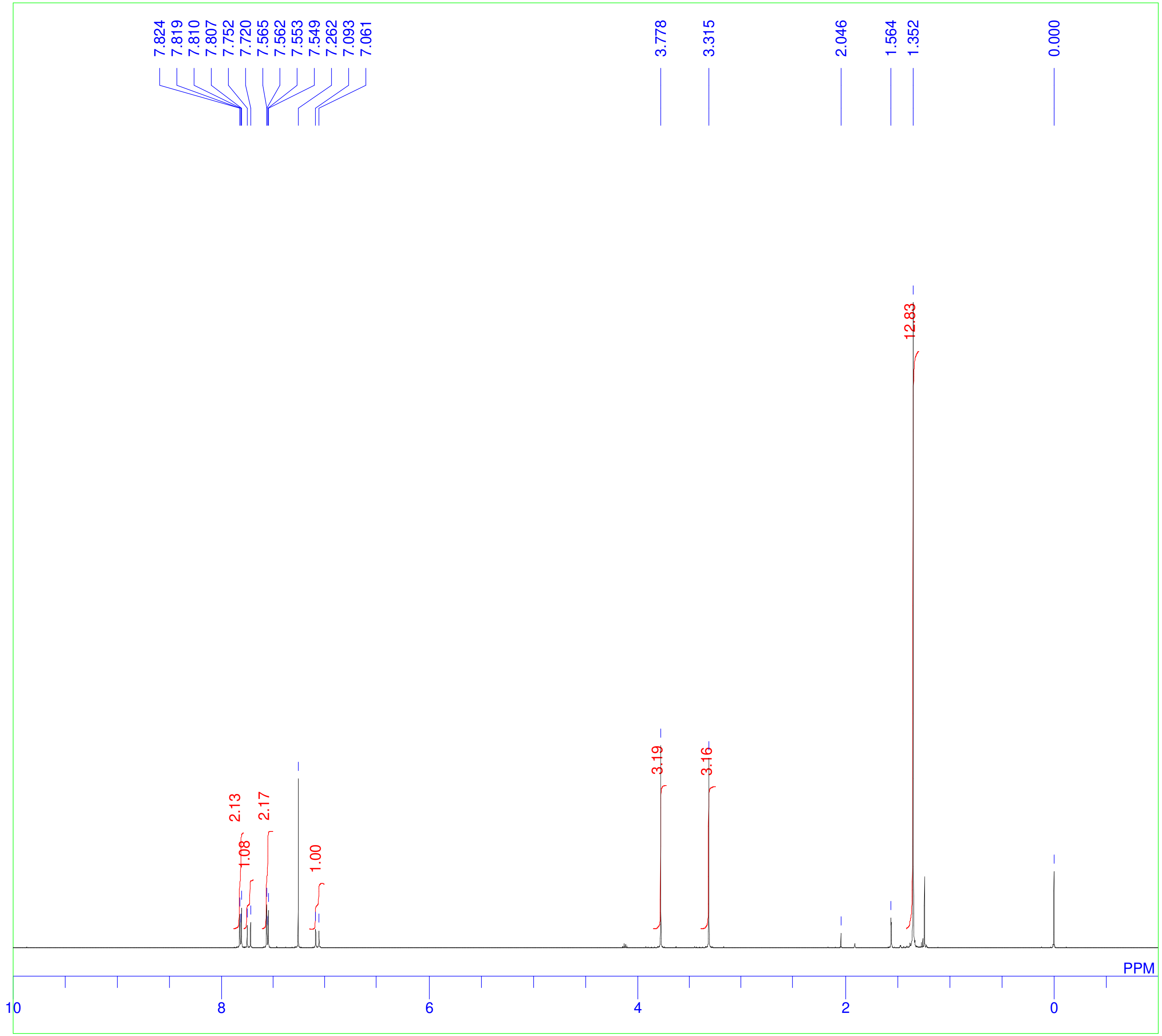

DFILE (E)-20r_1H.als
COMNT
DATIM 2023-12-08 16:47:55
OBNUC 1H
EXMOD proton.jxp
OBFRQ 500.16 MHz
OBSET 2.41 KHz
OBFIN 6.01 Hz
POINT 13107
FREQU 7507.51 Hz
SCANS 8
ACQTM 1.7459 sec
PD 5.0000 sec
PW1 3.80 usec
IRNUC 1H
CTEMP 23.8 c
SLVNT CDCL3
EXREF 0.00 ppm
BF 0.30 Hz
RGAIN 46

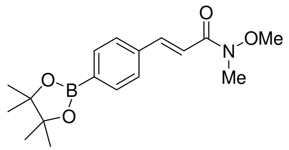

(*E*)-20r

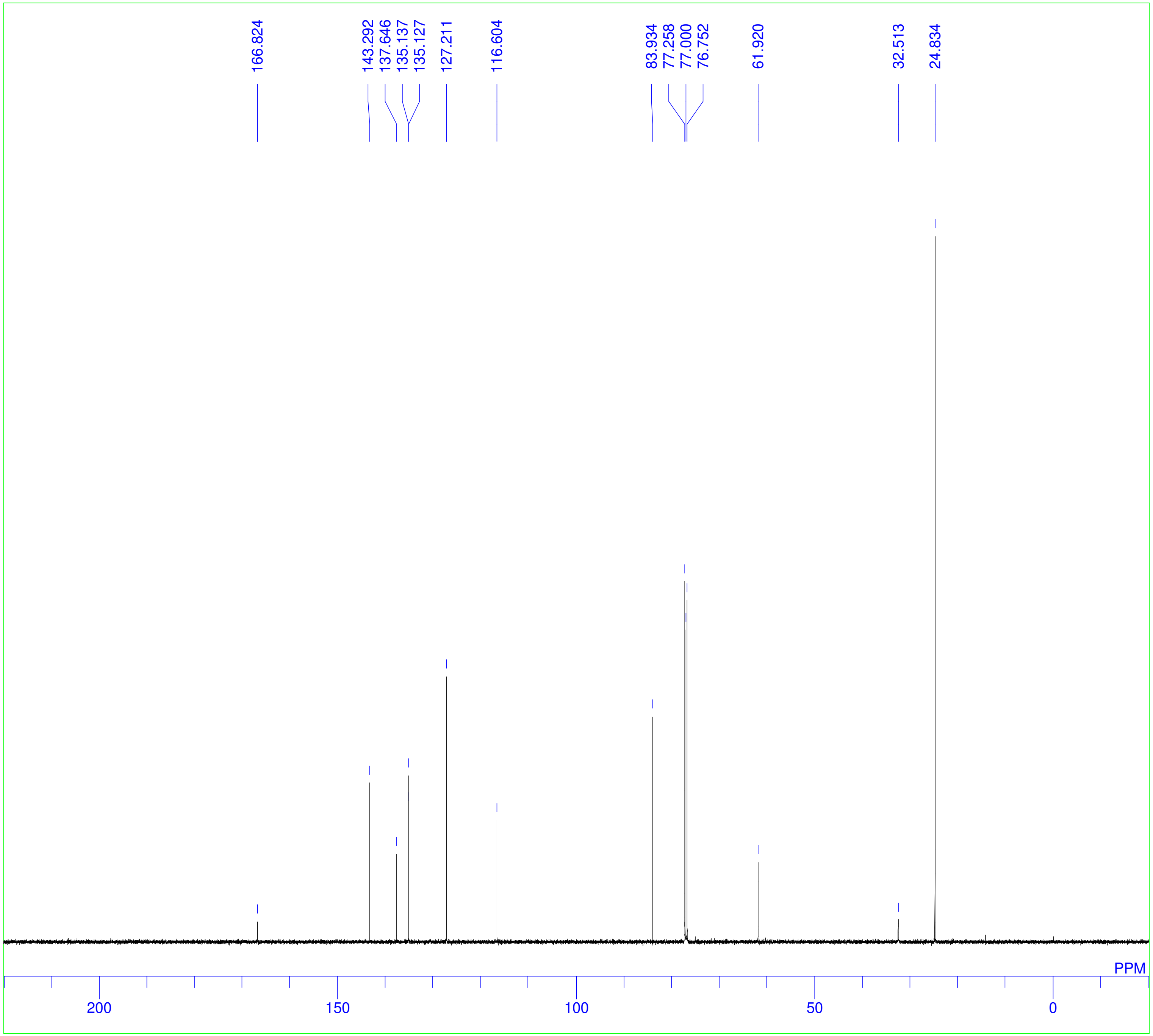
166.824
143.292
137.646
135.137
135.127
127.211
116.604
83.934
77.258
77.000
76.752
61.920
32.513
24.834
PPM
200
150
100
50
0
DFILE (E)-20r_13C.als
COMNT
DATIM 2023-12-08 16:54:52
OBNUC 13C
EXMOD carbon.jxp
OBFRQ 125.77 MHz
OBSET 7.87 KHz
OBFIN 4.21 Hz
POINT 26214
FREQU 31446.54 Hz
SCANS 1024
ACQTM 0.8336 sec
PD 2.0000 sec
PW1 4.30 usec
IRNUC 1H
CTEMP 24.0 c
SLVNT CDCL3
EXREF 77.00 ppm
BF 0.30 Hz
RGAIN 30

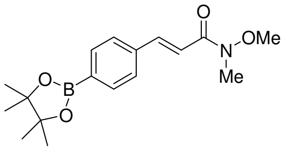
O
OMe
N
Me
O
B
O

(*E*)-20r

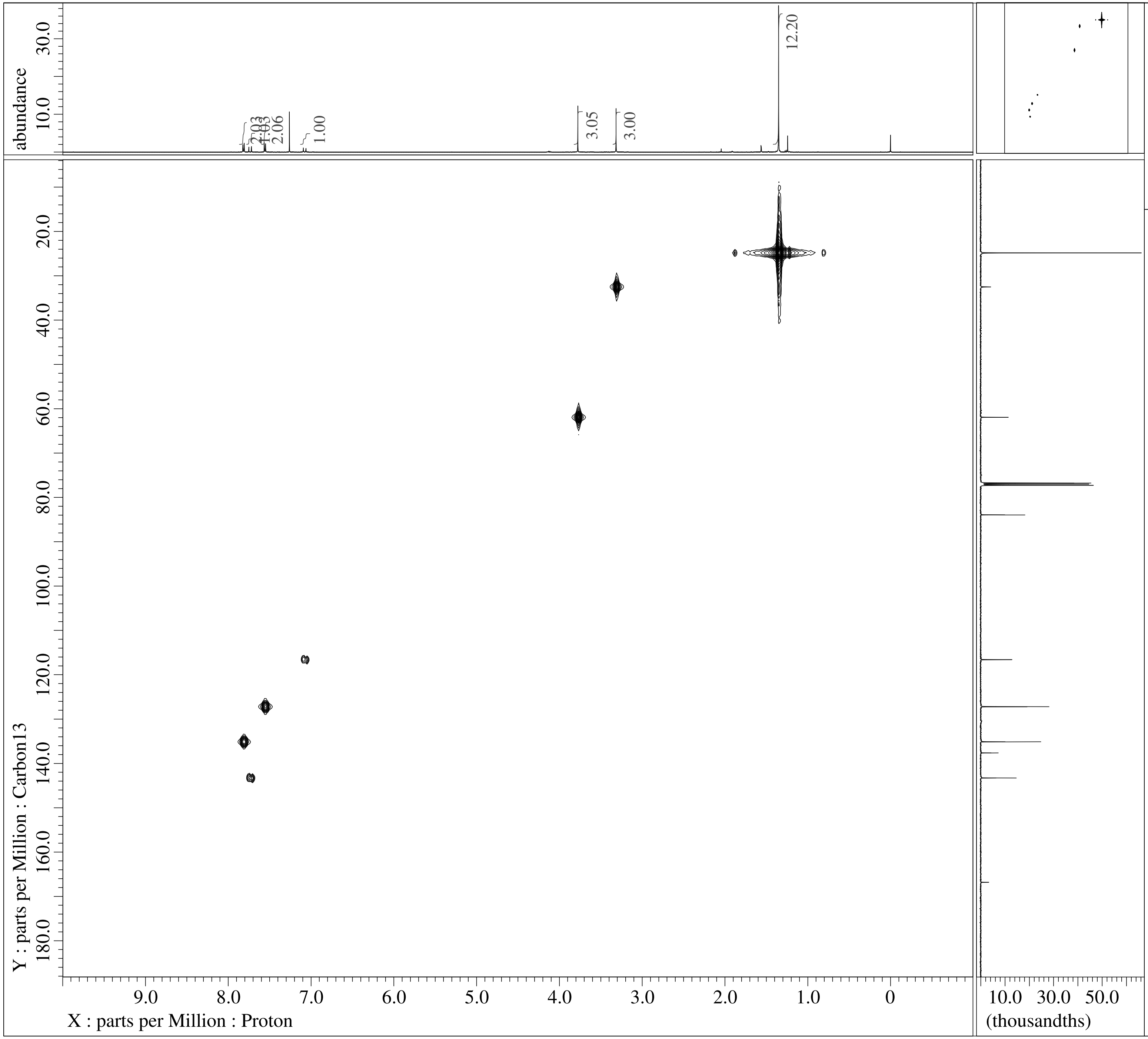


```
---- PROCESSING PARAMETERS ----
sinbell4( -60, 160 )
zerofill( 1 )
fft( 1, TRUE, TRUE )
ppm
[transpose]
sinbell4( -60, 160 )
zerofill( 2 )
fft( 1, TRUE, TRUE )
ppm
abs
```

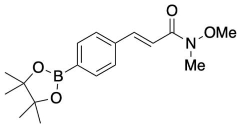

(*E*)-20r

```
Filename            = HT-1660-PTLC 1_13C_HMQC
Author              = delta
Experiment          = hmqc.jxp
Sample_Id           = HT-1660-PTLC 1_13C
Solvent             = CHLOROFORM-D
Creation_Time       =  8-DEC-2023 17:43:44
Revision_Time       = 11-APR-2024 10:36:06
Current_Time        = 11-APR-2024 10:36:31

Comment             = gradient enhanced HMQC
Data_Format         = 2D REAL REAL
Dim_Size            = 819, 512
Dim_Title           = Proton Carbon13
Dim_Units           = [ppm] [ppm]
Dimensions          = X Y
Site                = JNM-ECA500II
Spectrometer        = DELTA2_NMR

Field_Strength      = 11.7473579[T] (500[MHz]
X_Acq_Duration      = 0.10911744[s]
X_Domain            = 1H
X_Freq              = 500.15991521[MHz]
X_Offset            = 5.0[ppm]
X_Points            = 1024
X_Prescans          = 4
X_Resolution        = 9.16443788[Hz]
X_Sweep             = 9.38438438[kHz]
X_Sweep_Clipped     = 7.50750751[kHz]
Y_Domain            = 13C
Y_Freq              = 125.76529768[MHz]
Y_Offset            = 95.93190421[ppm]
Y_Points            = 256
Y_Prescans          = 0
Y_Resolution        = 90.75859665[Hz]
Y_Sweep             = 23.23420074[kHz]
Tri_Domain          = Proton
Tri_Freq            = 500.15991521[MHz]
Tri_Offset          = 5.0[ppm]
Clipped             = FALSE
Scans               = 4
Total_Scans         = 1024

Relaxation_Delay    = 1.5[s]
Recvr_Gain          = 50
Temp_Get            = 23.8[dC]
X_Acq_Time          = 0.10911744[s]
X_Atn               = 3.2[dB]
X_Gamma             = 42576375
X_Pulse             = 7.59[us]
```

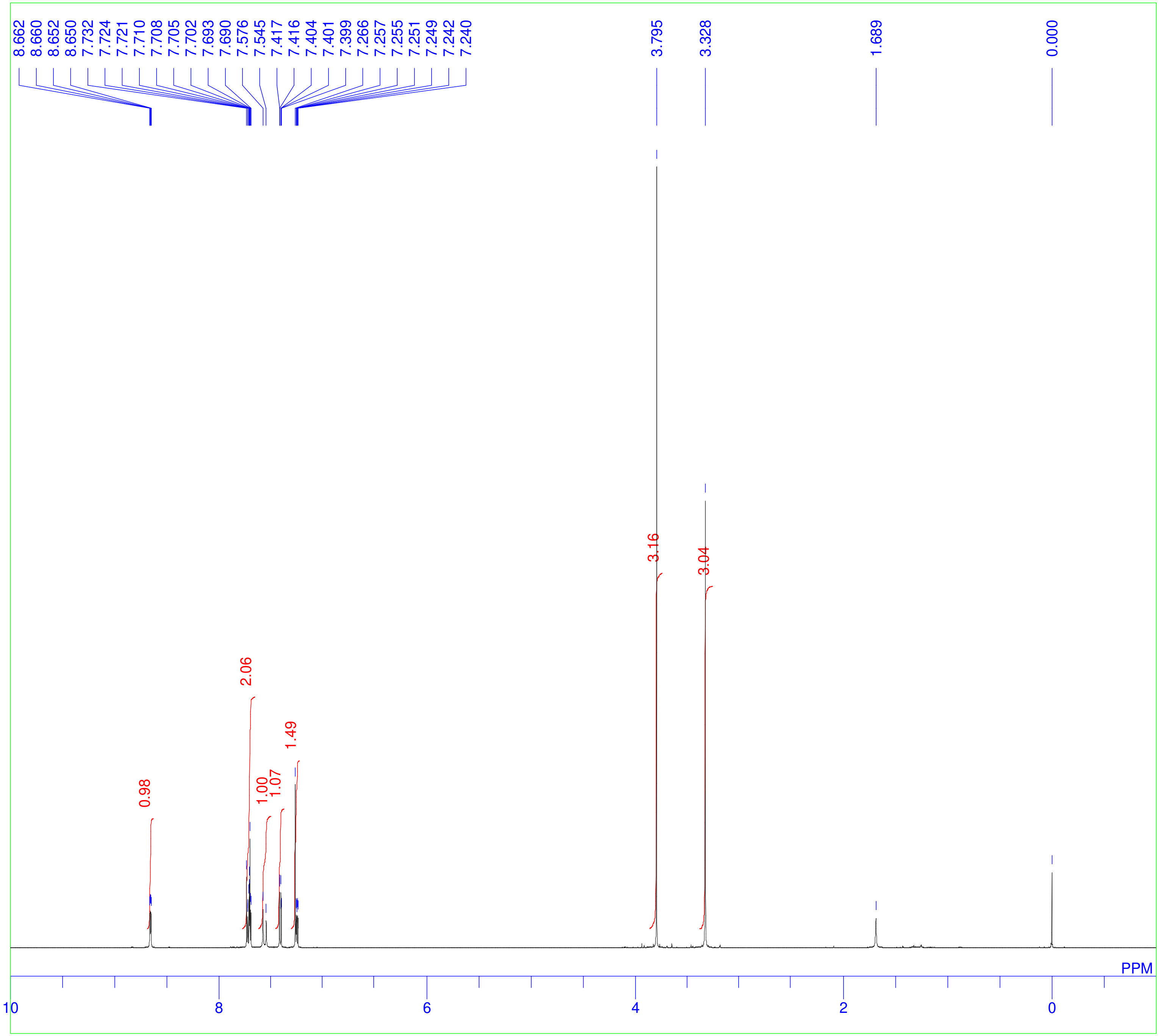

DFILE	(E)-20s_1H.als
COMNT	
DATIM	2023-12-06 21:44:60
OBNUC	1H
EXMOD	proton.jxp
OBFRQ	500.16 MHz
OBSET	2.41 KHz
OBFIN	6.01 Hz
POINT	13107
FREQU	7507.51 Hz
SCANS	8
ACQTM	1.7459 sec
PD	5.0000 sec
PW1	3.80 usec
IRNUC	1H
CTEMP	23.7 c
SLVNT	CDCL3
EXREF	0.00 ppm
BF	0.12 Hz
RGAIN	40

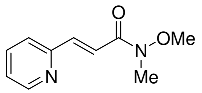

(*E*)-20s

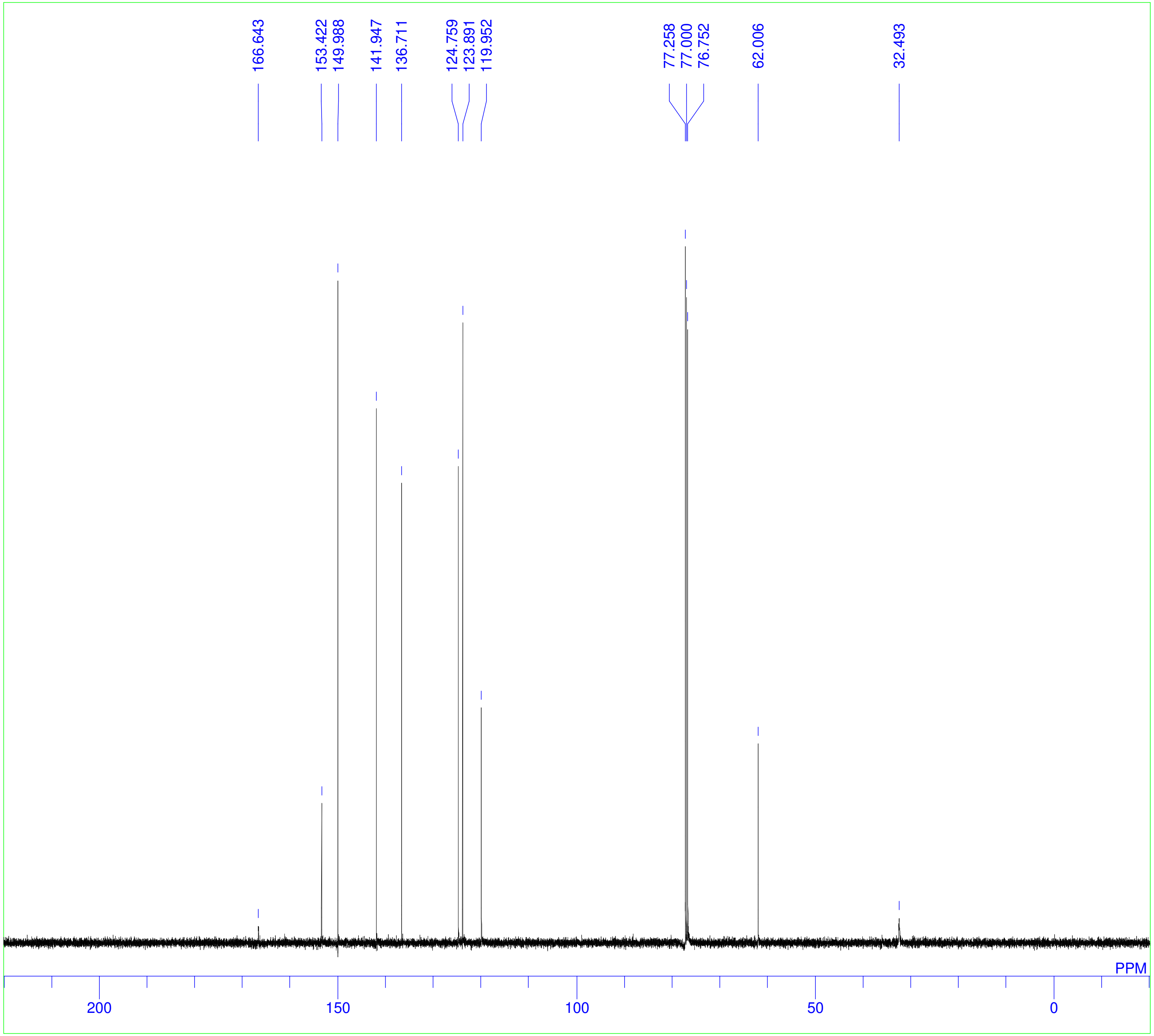

DFILE (E)-20s_13C.als
COMNT
DATIM 2023-12-07 20:05:28
OBNUC 13C
EXMOD carbon.jxp
OBFRQ 125.77 MHz
OBSET 7.87 KHz
OBFIN 4.21 Hz
POINT 26214
FREQU 31446.54 Hz
SCANS 1024
ACQTM 0.8336 sec
PD 2.0000 sec
PW1 4.30 usec
IRNUC 1H
CTEMP 23.8 c
SLVNT CDCL3
EXREF 77.00 ppm
BF 0.12 Hz
RGAIN 30

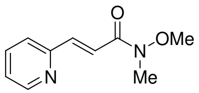

(*E*)-20s

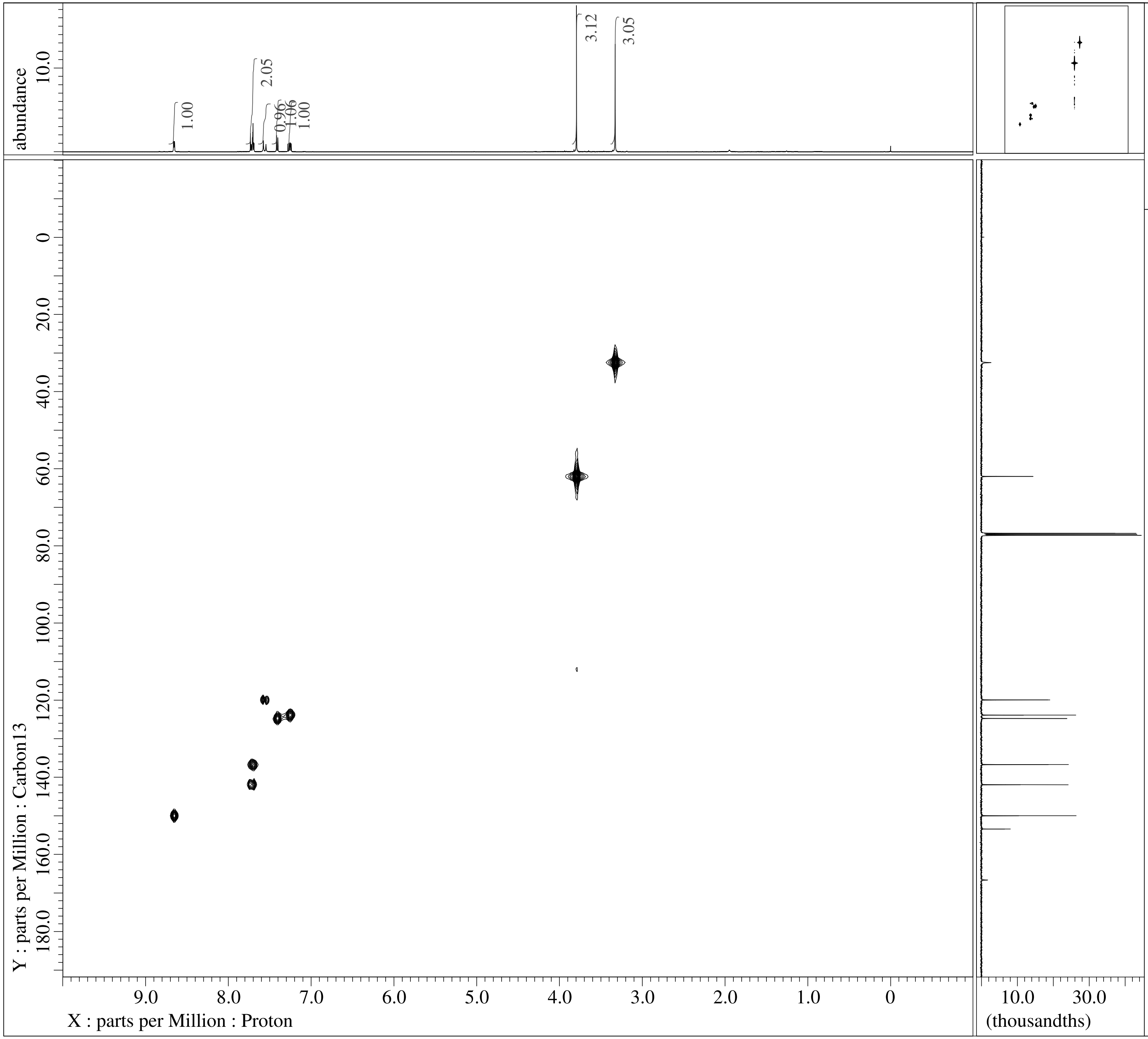


```
---- PROCESSING PARAMETERS ----
sinbell4( -60, 160 )
zerofill( 1 )
fft( 1, TRUE, TRUE )
ppm
[transpose]
sinbell4( -60, 160 )
zerofill( 2 )
fft( 1, TRUE, TRUE )
ppm
abs
```

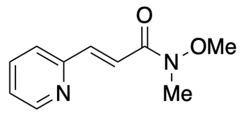

(*E*)-20s

```
Filename            = HT-1658-PTLC 2_13C_HMQC
Author              = delta
Experiment          = hmqc.jxp
Sample_Id           = HT-1658-PTLC 2_13C
Solvent             = CHLOROFORM-D
Creation_Time       =  7-DEC-2023 20:54:20
Revision_Time       = 11-APR-2024 10:46:02
Current_Time        = 11-APR-2024 10:47:46

Comment             = gradient enhanced HMQC
Data_Format         = 2D REAL REAL
Dim_Size            = 819, 512
Dim_Title           = Proton Carbon13
Dim_Units           = [ppm] [ppm]
Dimensions          = X Y
Site                = JNM-ECA500II
Spectrometer        = DELTA2_NMR

Field_Strength      = 11.7473579[T] (500[MHz]
X_Acq_Duration      = 0.10911744[s]
X_Domain            = 1H
X_Freq              = 500.15991521[MHz]
X_Offset            = 5.0[ppm]
X_Points            = 1024
X_Prescans          = 4
X_Resolution        = 9.16443788[Hz]
X_Sweep             = 9.38438438[kHz]
X_Sweep_Clipped     = 7.50750751[kHz]
Y_Domain            = 13C
Y_Freq              = 125.76529768[MHz]
Y_Offset            = 83.4128521[ppm]
Y_Points            = 256
Y_Prescans          = 0
Y_Resolution        = 106.61162664[Hz]
Y_Sweep             = 27.29257642[kHz]
Tri_Domain          = Proton
Tri_Freq            = 500.15991521[MHz]
Tri_Offset          = 5.0[ppm]
Clipped             = FALSE
Scans               = 8
Total_Scans         = 2048

Relaxation_Delay    = 1.5[s]
Recvr_Gain          = 50
Temp_Get            = 23.7[dC]
X_Acq_Time          = 0.10911744[s]
X_Atn               = 3.2[dB]
X_Gamma             = 42576375
X_Pulse             = 7.59[us]
```

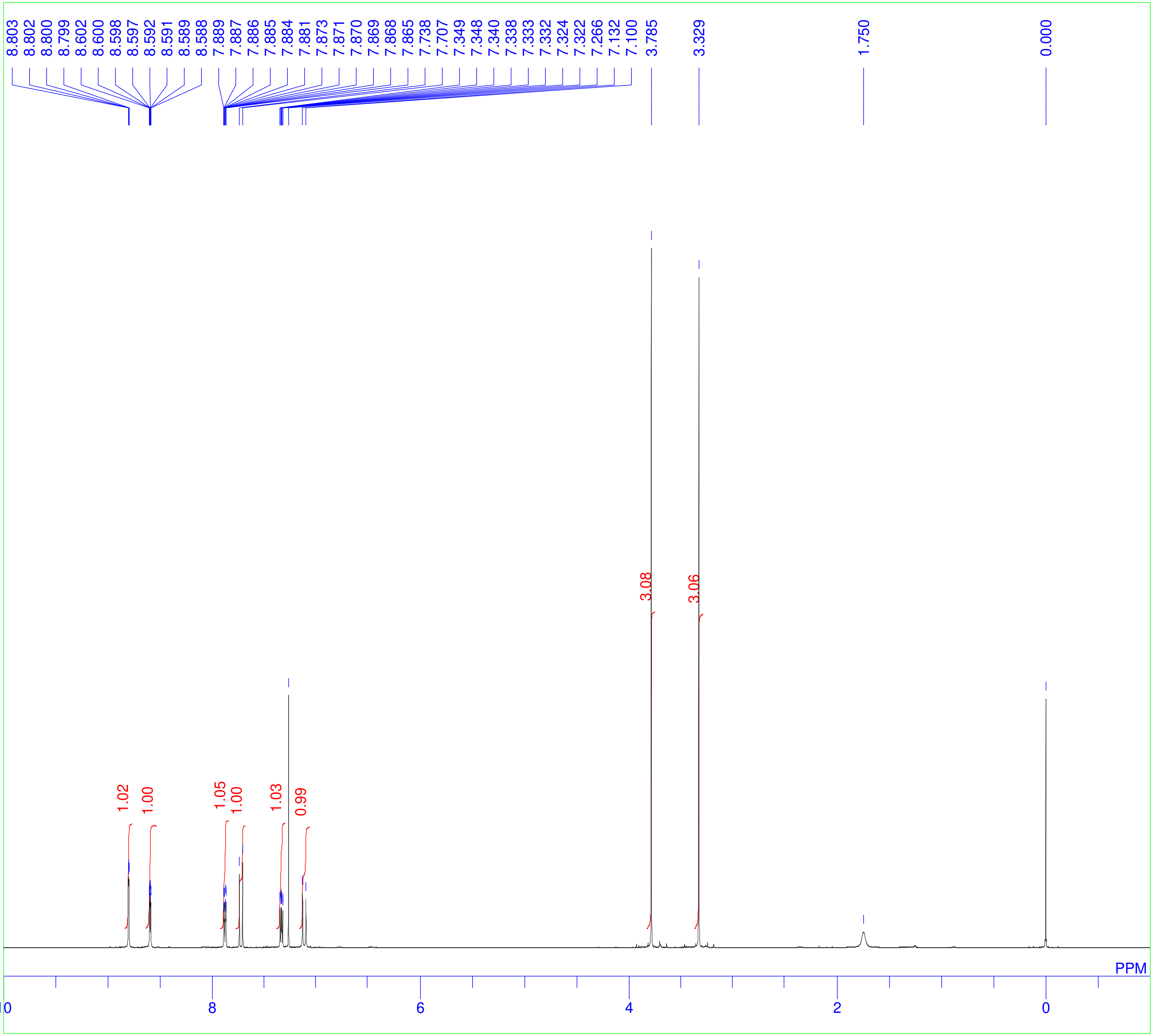

DFILE (E)-20t_1H.als
COMNT
DATIM 2022-01-26 20:14:19
OBNUC 1H
EXMOD proton.jxp
OBFRQ 500.16 MHz
OBSET 2.41 KHz
OBFIN 6.01 Hz
POINT 13107
FREQU 7507.51 Hz
SCANS 8
ACQTM 1.7459 sec
PD 5.0000 sec
PW1 3.84 usec
IRNUC 1H
CTEMP 19.8 c
SLVNT CDCL3
EXREF 0.00 ppm
BF 0.30 Hz
RGAIN 44

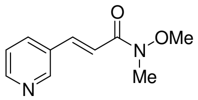

(*E*)-20t

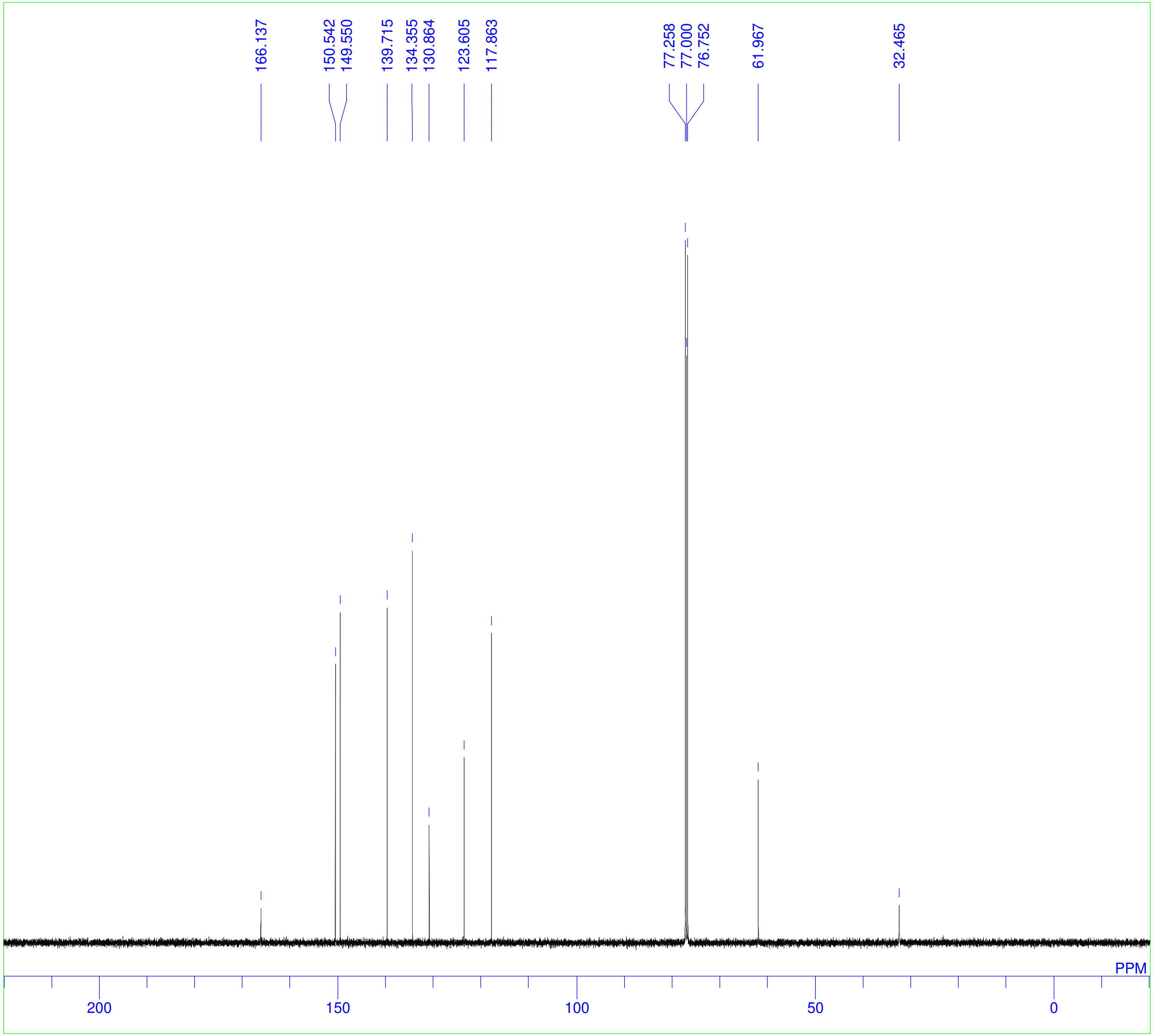

DFILE (E)-20t_13C.als
COMNT
DATIM 2023-01-09 11:17:41
OBNUC 13C
EXMOD carbon.jxp
OBFRQ 125.77 MHz
OBSET 7.87 KHz
OBFIN 4.21 Hz
POINT 26214
FREQU 31446.54 Hz
SCANS 1024
ACQTM 0.8336 sec
PD 2.0000 sec
PW1 3.87 usec
IRNUC 1H
CTEMP 21.6 c
SLVNT CDCL3
EXREF 77.00 ppm
BF 0.30 Hz
RGAIN 24

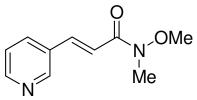

(*E*)-20t

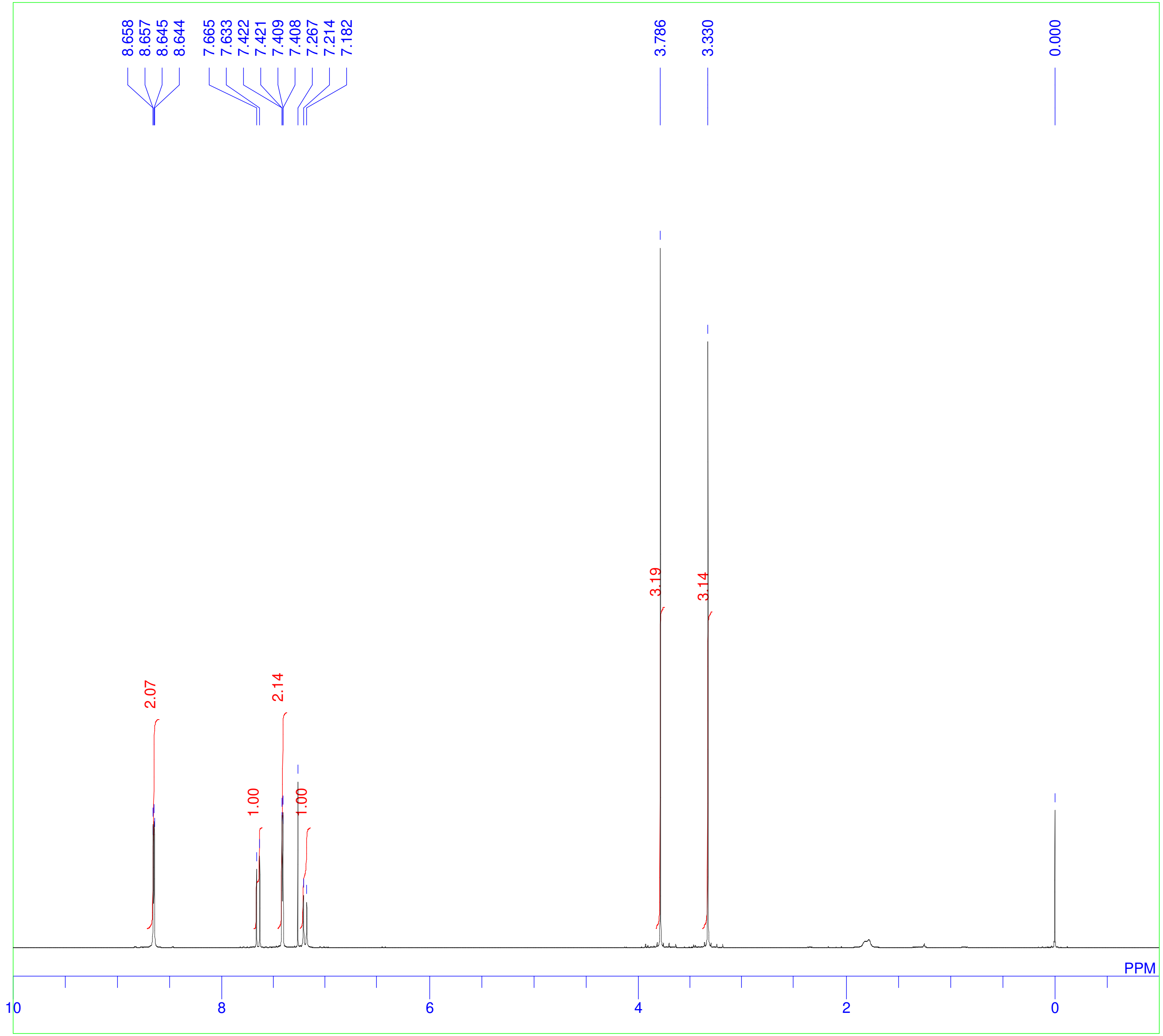

DFILE (E)-20u_1H.als
COMNT
DATIM 2022-01-26 20:20:39
OBNUC 1H
EXMOD proton.jxp
OBFRQ 500.16 MHz
OBSET 2.41 KHz
OBFIN 6.01 Hz
POINT 13107
FREQU 7507.51 Hz
SCANS 8
ACQTM 1.7459 sec
PD 5.0000 sec
PW1 3.84 usec
IRNUC 1H
CTEMP 19.8 c
SLVNT CDCL3
EXREF 0.00 ppm
BF 0.30 Hz
RGAIN 42

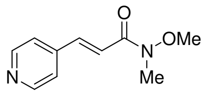

(*E*)-20u

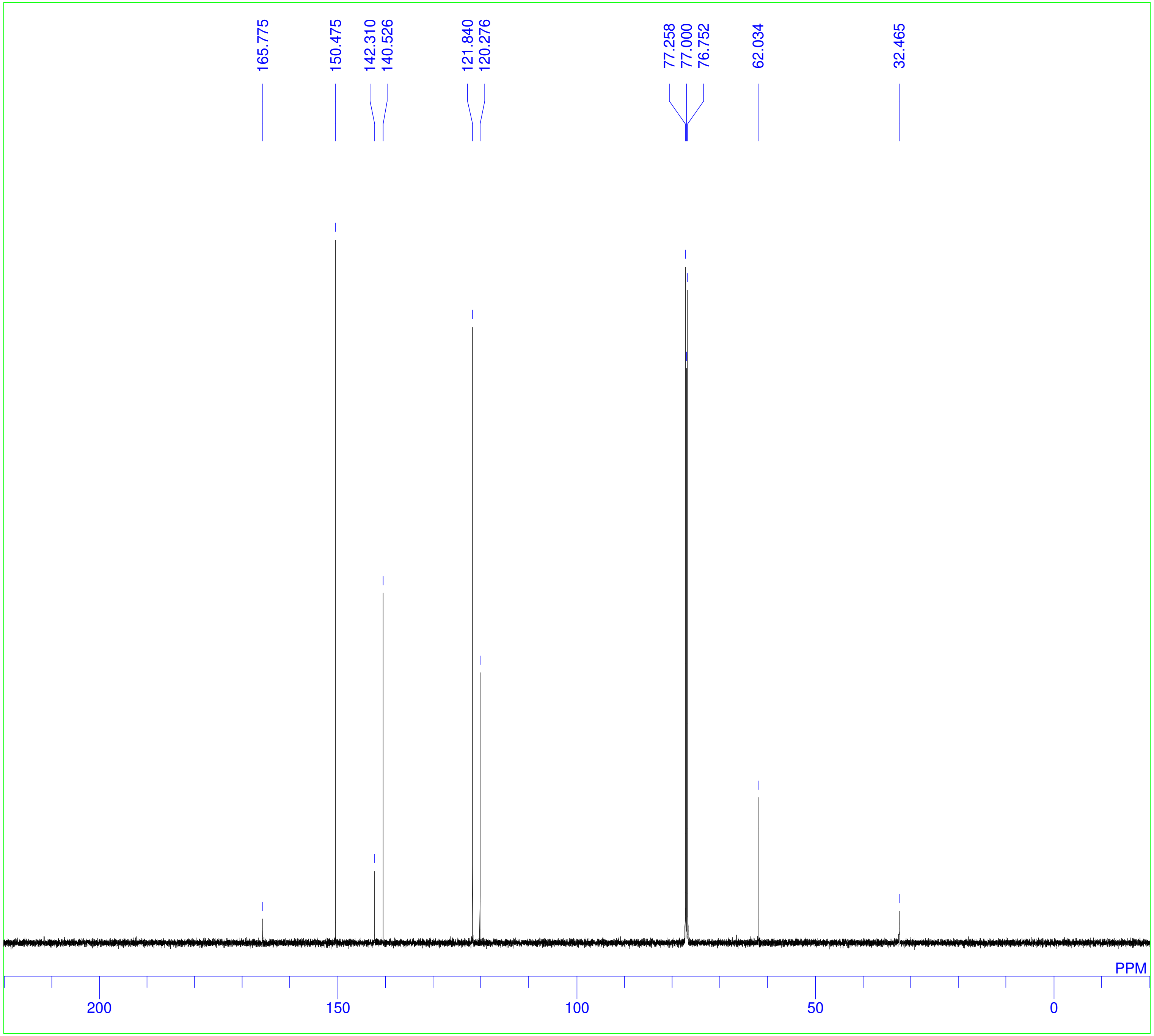

DFILE (E)-20u_13C.als
COMNT
DATIM 2023-01-09 14:22:54
OBNUC 13C
EXMOD carbon.jxp
OBFRQ 125.77 MHz
OBSET 7.87 KHz
OBFIN 4.21 Hz
POINT 26214
FREQU 31446.54 Hz
SCANS 1024
ACQTM 0.8336 sec
PD 2.0000 sec
PW1 3.87 usec
IRNUC 1H
CTEMP 22.1 c
SLVNT CDCL3
EXREF 77.00 ppm
BF 0.30 Hz
RGAIN 26

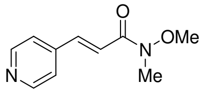

(*E*)-20u

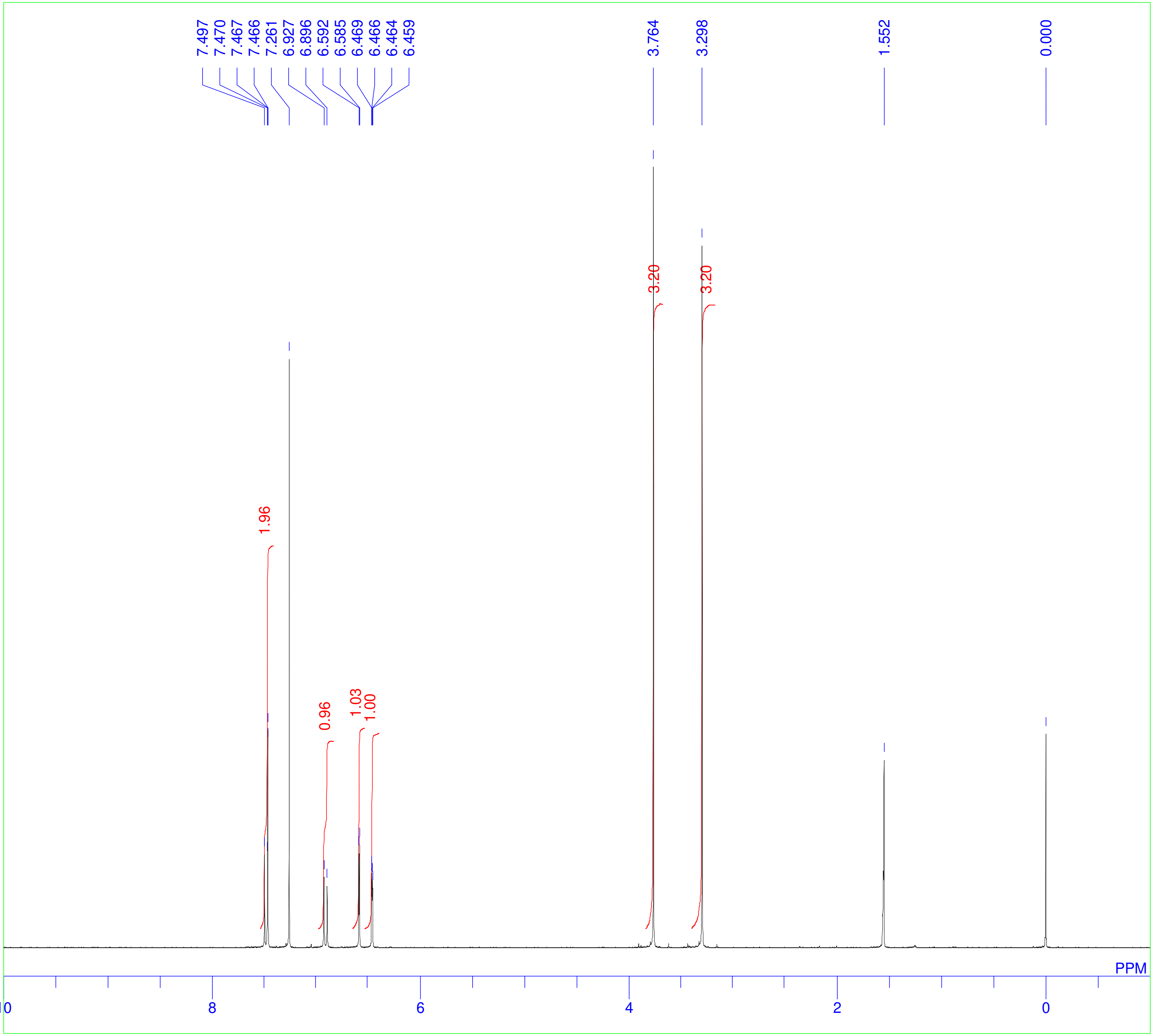

DFILE	(E)-20v_1H.als
COMNT	
DATIM	2023-04-19 17:18:31
OBNUC	1H
EXMOD	proton.jxp
OBFRQ	500.16 MHz
OBSET	2.41 KHz
OBFIN	6.01 Hz
POINT	13107
FREQU	7507.51 Hz
SCANS	8
ACQTM	1.7459 sec
PD	5.0000 sec
PW1	3.84 usec
IRNUC	1H
CTEMP	24.3 c
SLVNT	CDCL3
EXREF	0.00 ppm
BF	0.30 Hz
RGAIN	50

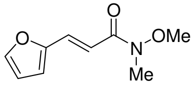

(*E*)-20v

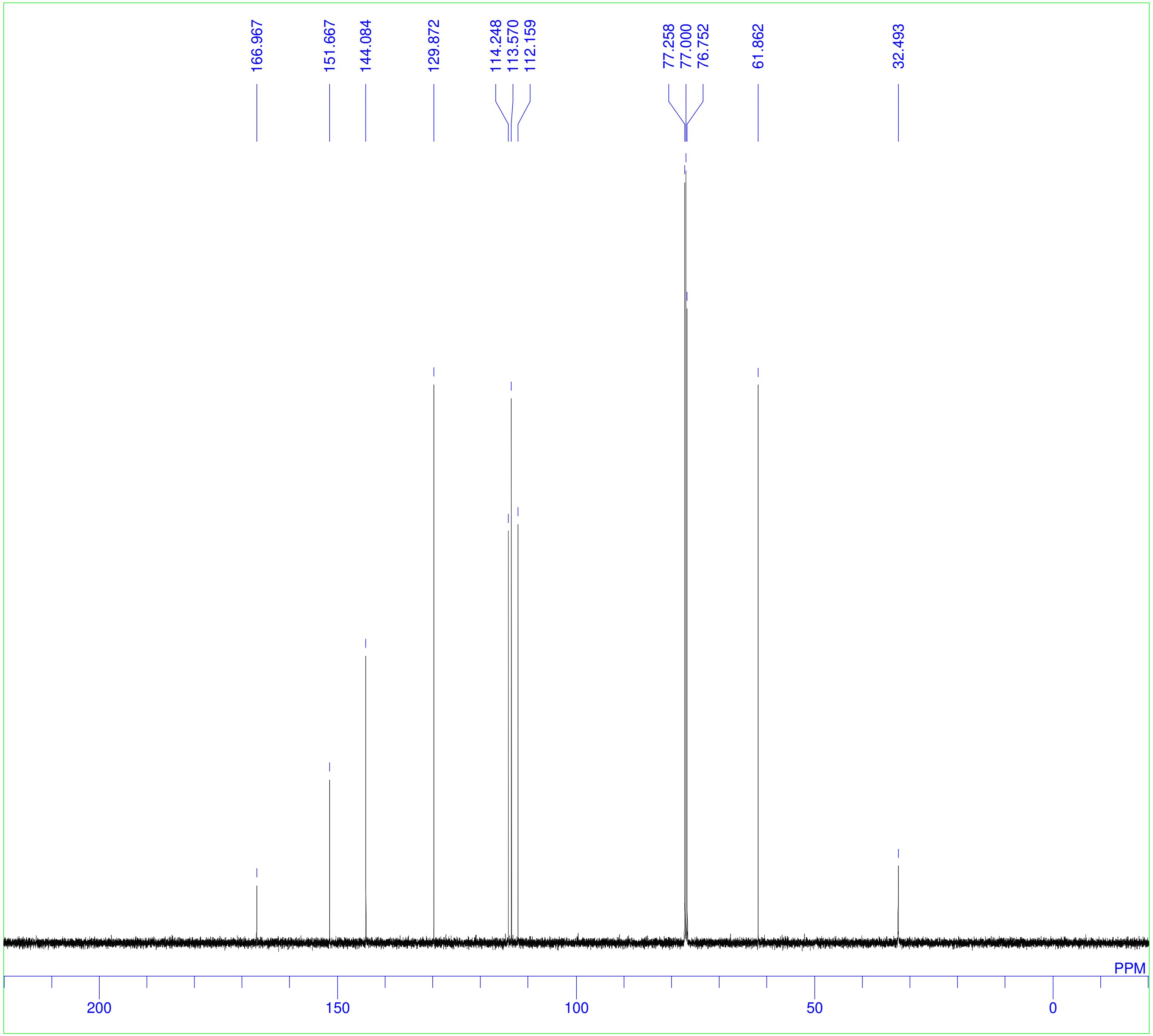

DFILE	(E)-20v_13C.als
COMNT	
DATIM	2023-04-19 19:46:46
OBNUC	13C
EXMOD	carbon.jxp
OBFRQ	125.77 MHz
OBSET	7.87 KHz
OBFIN	4.21 Hz
POINT	26214
FREQU	31446.54 Hz
SCANS	1024
ACQTM	0.8336 sec
PD	2.0000 sec
PW1	3.87 usec
IRNUC	1H
CTEMP	23.9 c
SLVNT	CDCL3
EXREF	77.00 ppm
BF	0.30 Hz
RGAIN	32

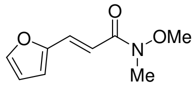

(*E*)-20v

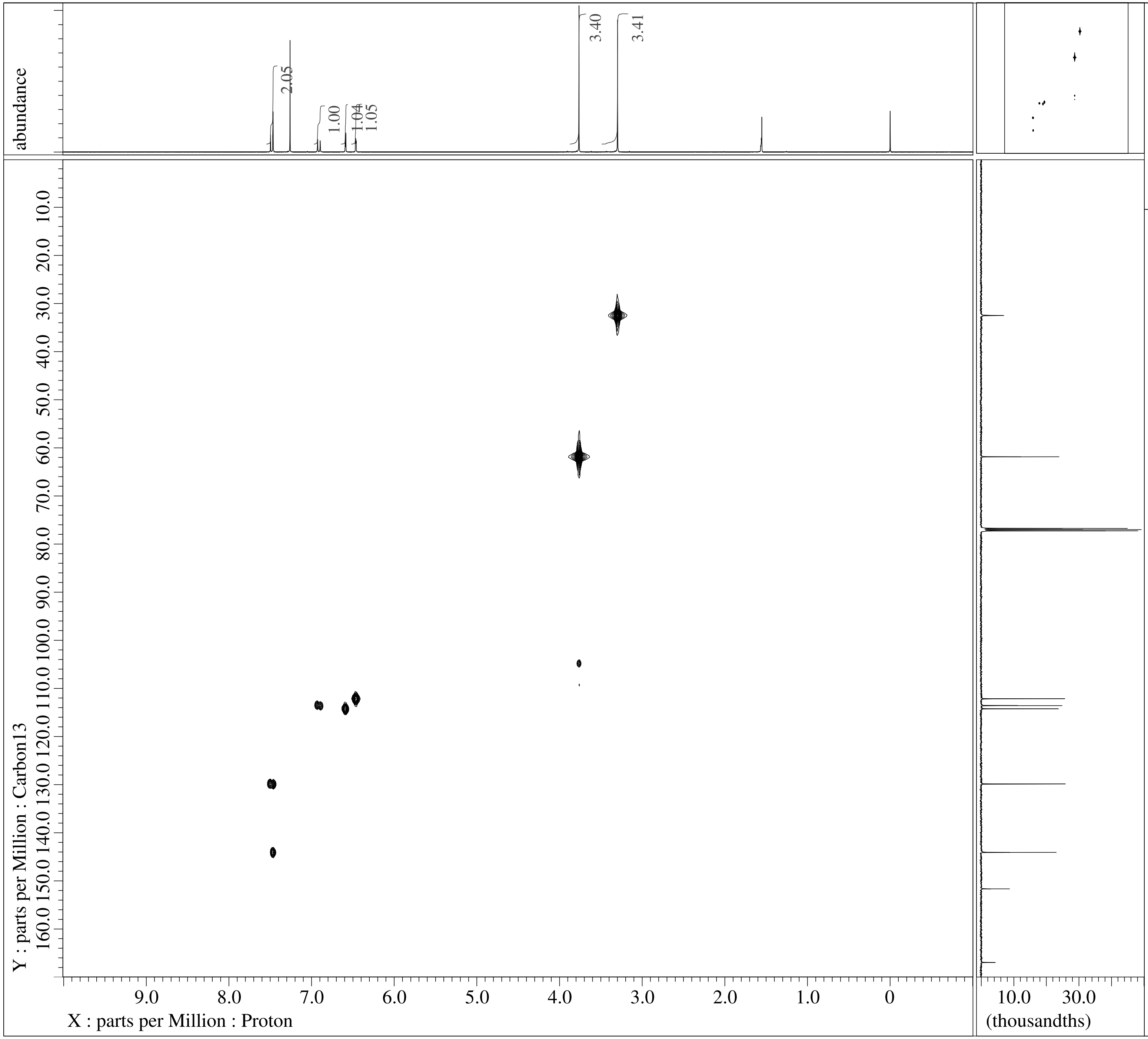


```
---- PROCESSING PARAMETERS ----
sinbell4( -60, 160 )
zerofill( 1 )
fft( 1, TRUE, TRUE )
ppm
[transpose]
sinbell4( -60, 160 )
zerofill( 2 )
fft( 1, TRUE, TRUE )
ppm
abs
```

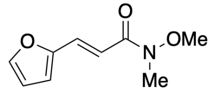

(E)-20v

```
Filename            = HT-1362-PTLC 1_HMBC,HMQ
Author              = delta
Experiment          = hmqc.jxp
Sample_Id           = HT-1362-PTLC 1_HMBC,HMQ
Solvent             = CHLOROFORM-D
Creation_Time       = 25-APR-2023 19:31:39
Revision_Time       = 11-APR-2024 10:50:41
Current_Time        = 11-APR-2024 10:51:10

Comment             = gradient enhanced HMQC
Data_Format         = 2D REAL REAL
Dim_Size            = 819, 512
Dim_Title           = Proton Carbon13
Dim_Units           = [ppm] [ppm]
Dimensions          = X Y
Site                = JNM-ECA500II
Spectrometer        = DELTA2_NMR

Field_Strength      = 11.7473579[T] (500[MHz]
X_Acq_Duration      = 0.10911744[s]
X_Domain            = 1H
X_Freq              = 500.15991521[MHz]
X_Offset            = 5.0[ppm]
X_Points            = 1024
X_Prescans          = 4
X_Resolution        = 9.16443788[Hz]
X_Sweep             = 9.38438438[kHz]
X_Sweep_Clipped     = 7.50750751[kHz]
Y_Domain            = 13C
Y_Freq              = 125.76529768[MHz]
Y_Offset            = 85[ppm]
Y_Points            = 256
Y_Prescans          = 0
Y_Resolution        = 83.60980308[Hz]
Y_Sweep             = 21.40410959[kHz]
Tri_Domain          = Proton
Tri_Freq            = 500.15991521[MHz]
Tri_Offset          = 5.0[ppm]
Clipped             = FALSE
Scans               = 8
Total_Scans         = 2048

Relaxation_Delay    = 1.5[s]
Recvr_Gain          = 50
Temp_Get            = 24[dC]
X_Acq_Time          = 0.10911744[s]
X_Atn               = 3.2[dB]
X_Gamma             = 42576375
X_Pulse             = 7.68[us]
```

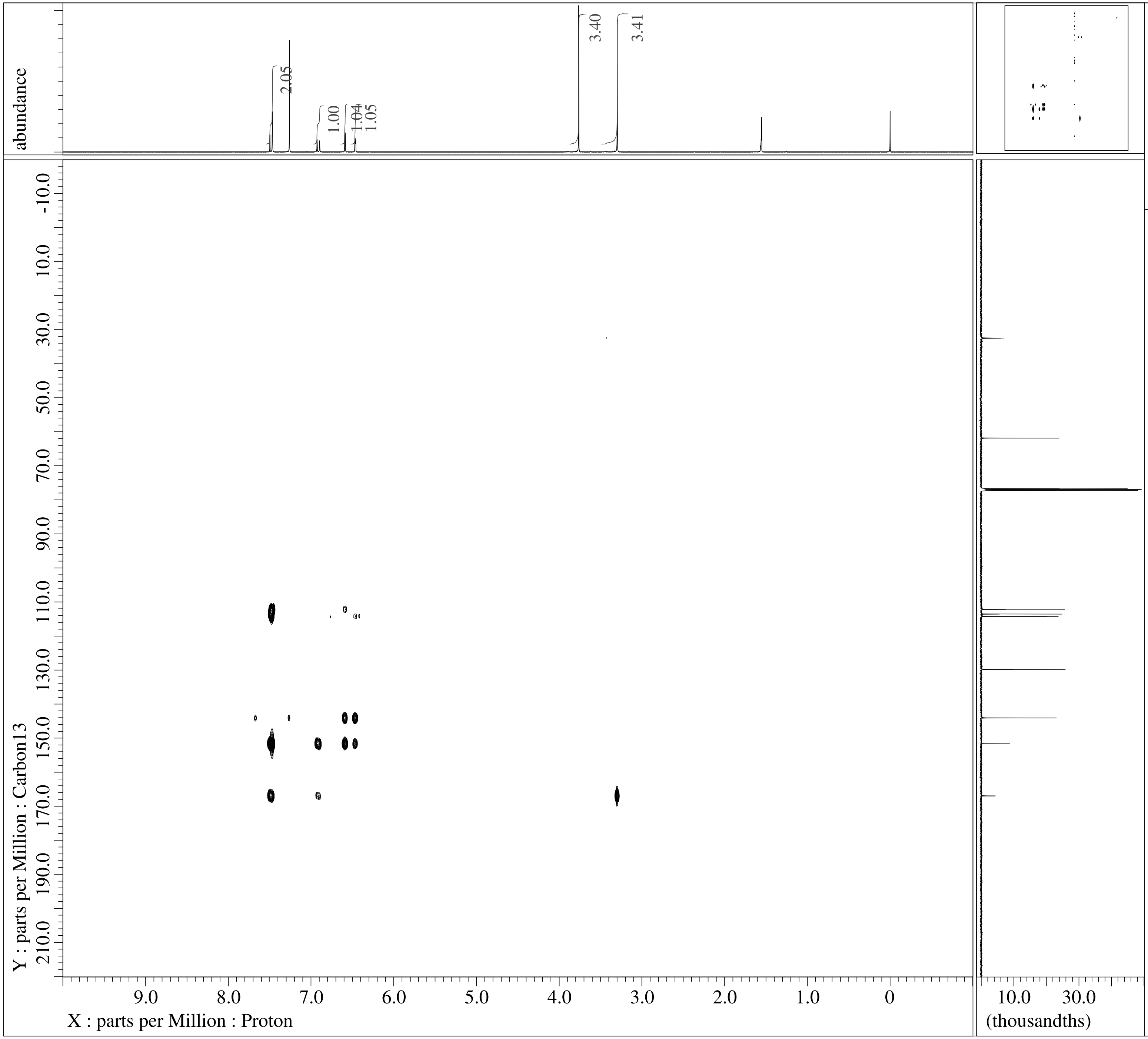

---- PROCESSING PARAMETERS ----
gauss(5.0[Hz], 0.0[s])
sinbell_auto
zerofill(1)
fft(1, TRUE, TRUE)
ppm
[transpose]
sinbell4(-60, 160)
trapezoid(0[%], 5[%], 80[%], 100[%])
zerofill(2)
fft(1, TRUE, TRUE)

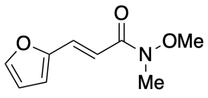

(E)-20v

Filename	= HT-1362-PTLC 1_HMBC,HMQ
Author	= delta
Experiment	= hmbc.jxp
Sample_Id	= HT-1362-PTLC 1_HMBC,HMQ
Solvent	= CHLOROFORM-D
Creation_Time	= 25-APR-2023 18:30:02
Revision_Time	= 11-APR-2024 10:52:53
Current_Time	= 11-APR-2024 10:53:30
Comment	= gradient enhanced HMBC
Data_Format	= 2D REAL REAL
Dim_Size	= 1638, 512
Dim_Title	= Proton Carbon13
Dim_Units	= [ppm] [ppm]
Dimensions	= X Y
Site	= JNM-ECA500II
Spectrometer	= DELTA2_NMR
Field_Strength	= 11.7473579[T] (500[MHz]
X_Acq_Duration	= 0.21823488[s]
X_Domain	= 1H
X_Freq	= 500.15991521[MHz]
X_Offset	= 5.0[ppm]
X_Points	= 2048
X_Prescans	= 4
X_Resolution	= 4.58221894[Hz]
X_Sweep	= 9.38438438[kHz]
X_Sweep_Clipped	= 7.50750751[kHz]
Y_Domain	= 13C
Y_Freq	= 125.76529768[MHz]
Y_Offset	= 100[ppm]
Y_Points	= 256
Y_Prescans	= 0
Y_Resolution	= 122.99275819[Hz]
Y_Sweep	= 31.4861461[kHz]
Tri_Domain	= Proton
Tri_Freq	= 500.15991521[MHz]
Tri_Offset	= 5.0[ppm]
Clipped	= FALSE
Scans	= 8
Total_Scans	= 2048
Relaxation_Delay	= 1.5[s]
Recvr_Gain	= 50
Temp_Get	= 23.9[dC]
X_Acq_Time	= 0.21823488[s]
X_Atn	= 3.2[dB]
X_Gamma	= 42576375
X_Pulse	= 7.68[us]

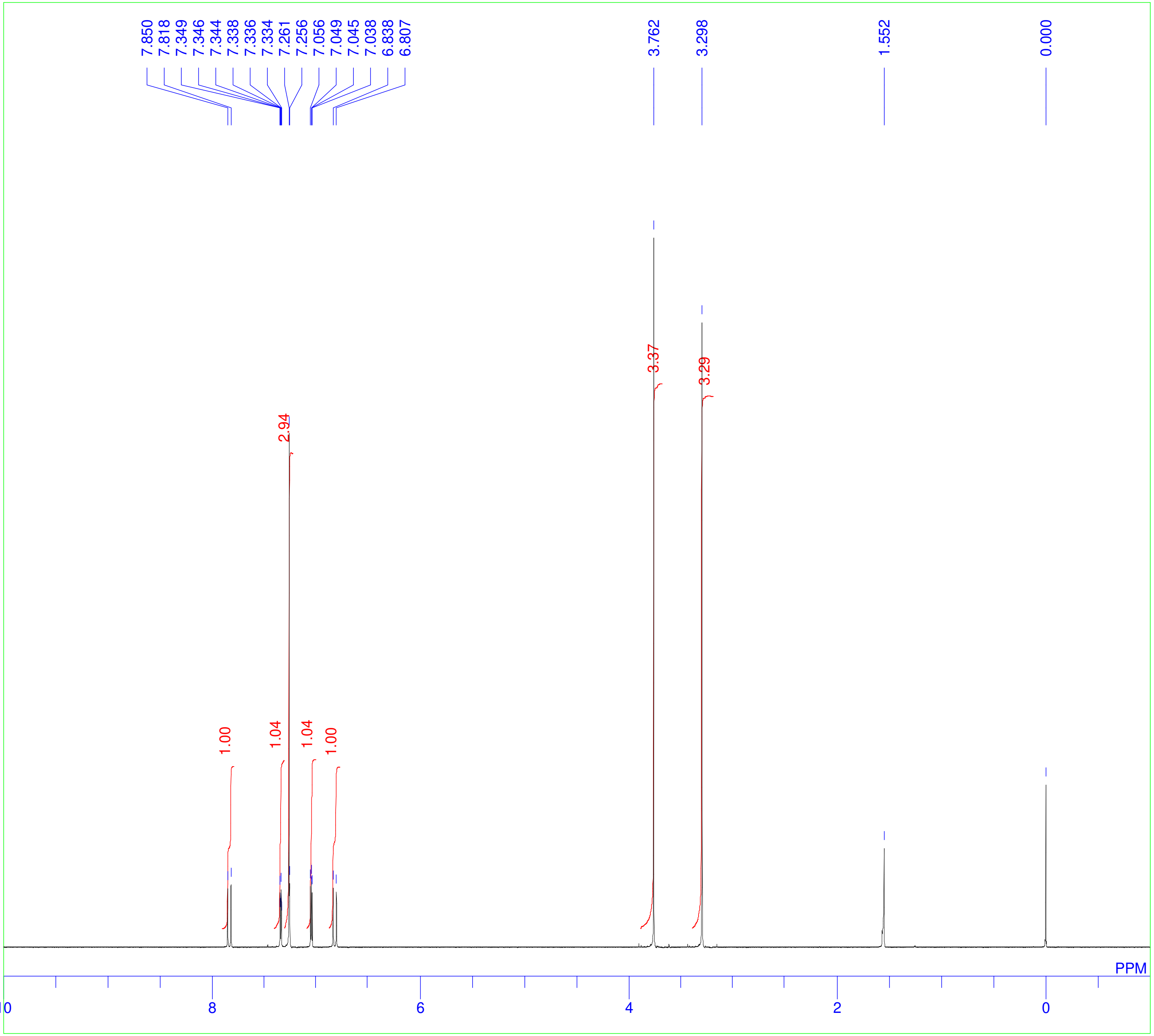

DFILE	(E)-20w_1H.als
COMNT	
DATIM	2023-04-19 17:31:10
OBNUC	1H
EXMOD	proton.jxp
OBFRQ	500.16 MHz
OBSET	2.41 KHz
OBFIN	6.01 Hz
POINT	13107
FREQU	7507.51 Hz
SCANS	8
ACQTM	1.7459 sec
PD	5.0000 sec
PW1	3.84 usec
IRNUC	1H
CTEMP	24.2 c
SLVNT	CDCL3
EXREF	0.00 ppm
BF	0.30 Hz
RGAIN	50

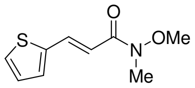

(*E*)-20w

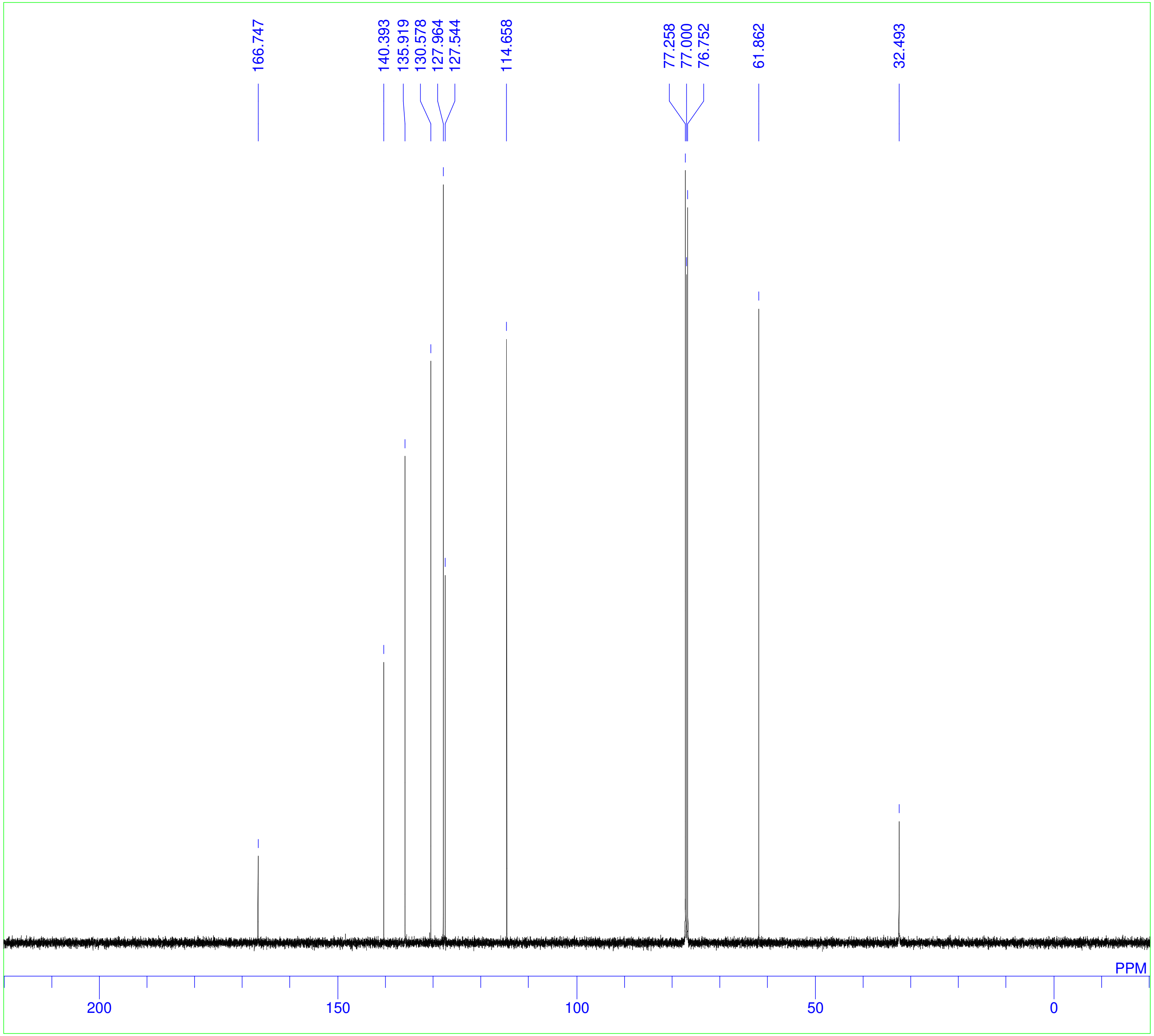

DFILE (E)-20w_13C.als
COMNT
DATIM 2023-04-19 20:50:51
OBNUC 13C
EXMOD carbon.jxp
OBFRQ 125.77 MHz
OBSET 7.87 KHz
OBFIN 4.21 Hz
POINT 26214
FREQU 31446.54 Hz
SCANS 1024
ACQTM 0.8336 sec
PD 2.0000 sec
PW1 3.87 usec
IRNUC 1H
CTEMP 24.0 c
SLVNT CDCL3
EXREF 77.00 ppm
BF 0.30 Hz
RGAIN 30

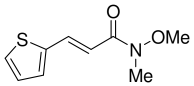

(*E*)-20w

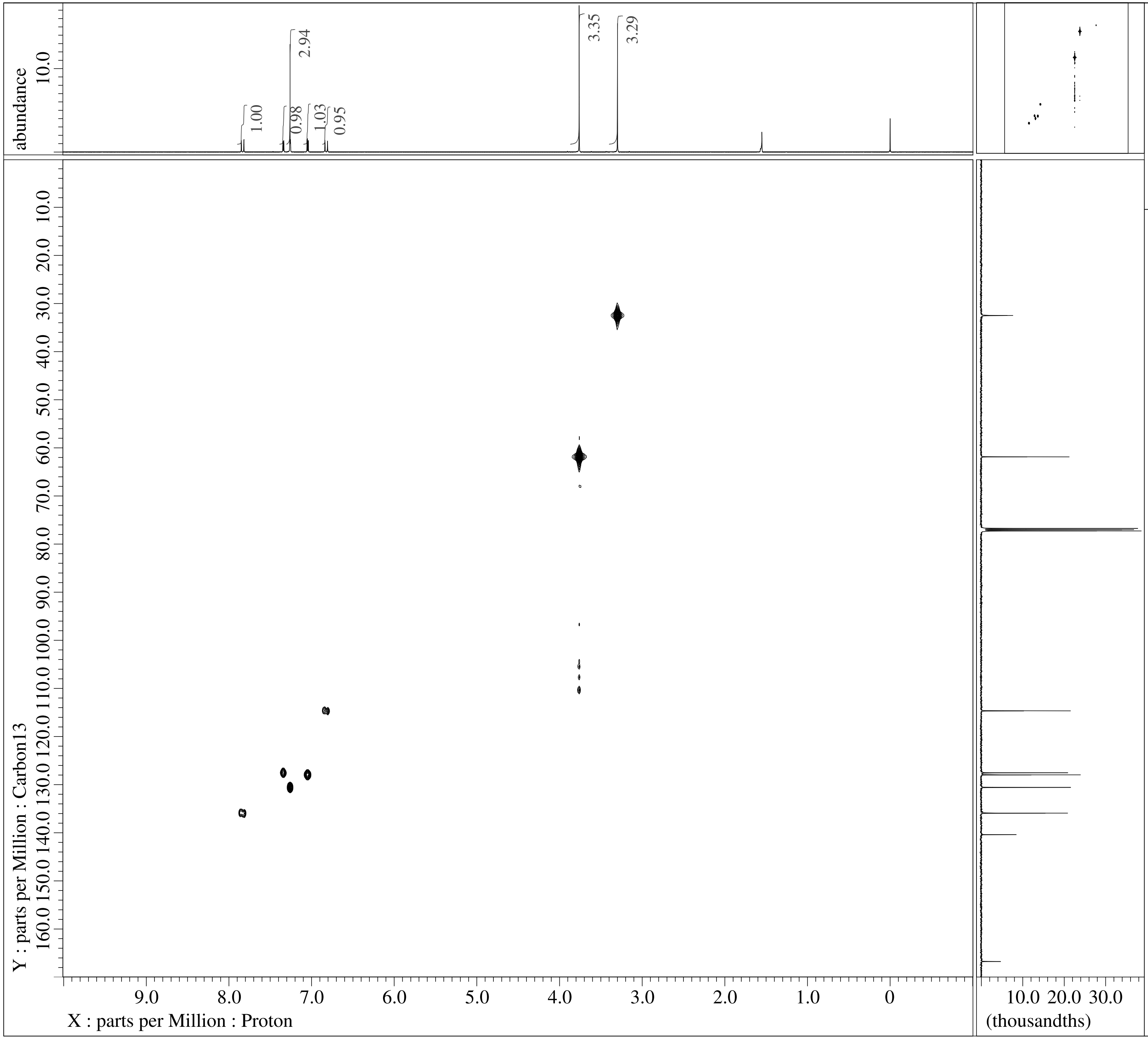


```
---- PROCESSING PARAMETERS ----
sinbell4( -60, 160 )
zerofill( 1 )
fft( 1, TRUE, TRUE )
ppm
[transpose]
sinbell4( -60, 160 )
zerofill( 2 )
fft( 1, TRUE, TRUE )
ppm
abs
```

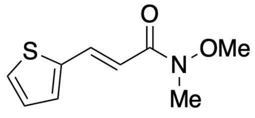

(*E*)-20w

```
Filename            = HT-1364-PTLC 1_HMQC_HMQ
Author              = delta
Experiment          = hmqc.jxp
Sample_Id           = HT-1364-PTLC 1_HMQC
Solvent             = CHLOROFORM-D
Creation_Time       = 25-APR-2023 20:44:07
Revision_Time       = 11-APR-2024 10:55:29
Current_Time        = 11-APR-2024 10:56:02

Comment             = gradient enhanced HMQC
Data_Format         = 2D REAL REAL
Dim_Size            = 819, 512
Dim_Title           = Proton Carbon13
Dim_Units           = [ppm] [ppm]
Dimensions          = X Y
Site                = JNM-ECA500II
Spectrometer        = DELTA2_NMR

Field_Strength      = 11.7473579[T] (500[MHz]
X_Acq_Duration      = 0.10911744[s]
X_Domain            = 1H
X_Freq              = 500.15991521[MHz]
X_Offset            = 5.0[ppm]
X_Points            = 1024
X_Prescans          = 4
X_Resolution        = 9.16443788[Hz]
X_Sweep             = 9.38438438[kHz]
X_Sweep_Clipped     = 7.50750751[kHz]
Y_Domain            = 13C
Y_Freq              = 125.76529768[MHz]
Y_Offset            = 85[ppm]
Y_Points            = 256
Y_Prescans          = 0
Y_Resolution        = 83.60980308[Hz]
Y_Sweep             = 21.40410959[kHz]
Tri_Domain          = Proton
Tri_Freq            = 500.15991521[MHz]
Tri_Offset          = 5.0[ppm]
Clipped             = FALSE
Scans               = 8
Total_Scans         = 2048

Relaxation_Delay    = 1.5[s]
Recvr_Gain          = 50
Temp_Get            = 24.1[dC]
X_Acq_Time          = 0.10911744[s]
X_Atn               = 3.2[dB]
X_Gamma             = 42576375
X_Pulse             = 7.68[us]
```

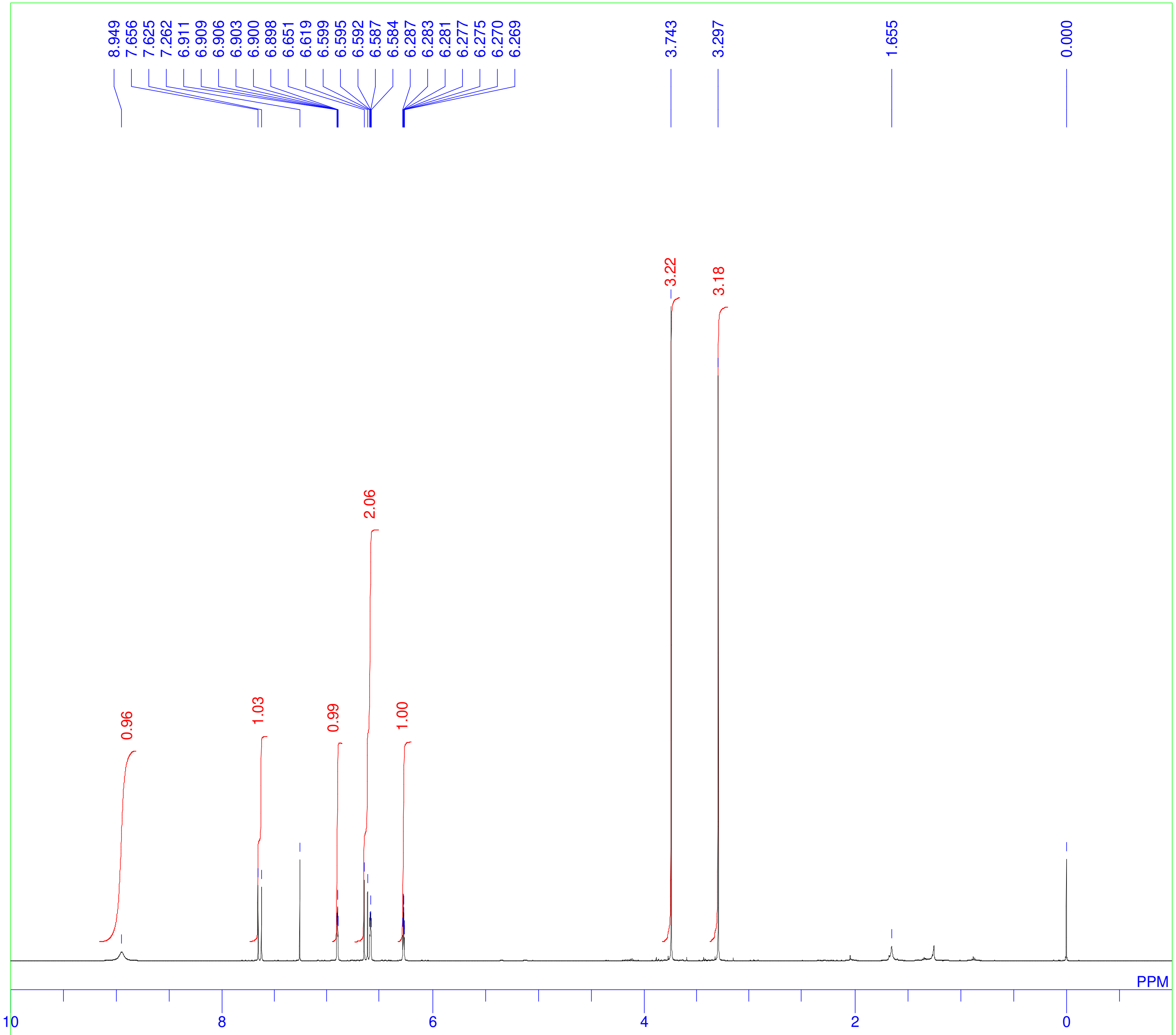

DFILE	(E)-20x_1H.als
COMNT	
DATIM	2023-06-15 13:31:07
OBNUC	1H
EXMOD	proton.jxp
OBFRQ	500.16 MHz
OBSET	2.41 KHz
OBFIN	6.01 Hz
POINT	13107
FREQU	7507.51 Hz
SCANS	8
ACQTM	1.7459 sec
PD	5.0000 sec
PW1	3.84 usec
IRNUC	1H
CTEMP	23.4 c
SLVNT	CDCL3
EXREF	0.00 ppm
BF	0.30 Hz
RGAIN	40

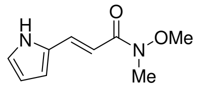

(*E*)-20x

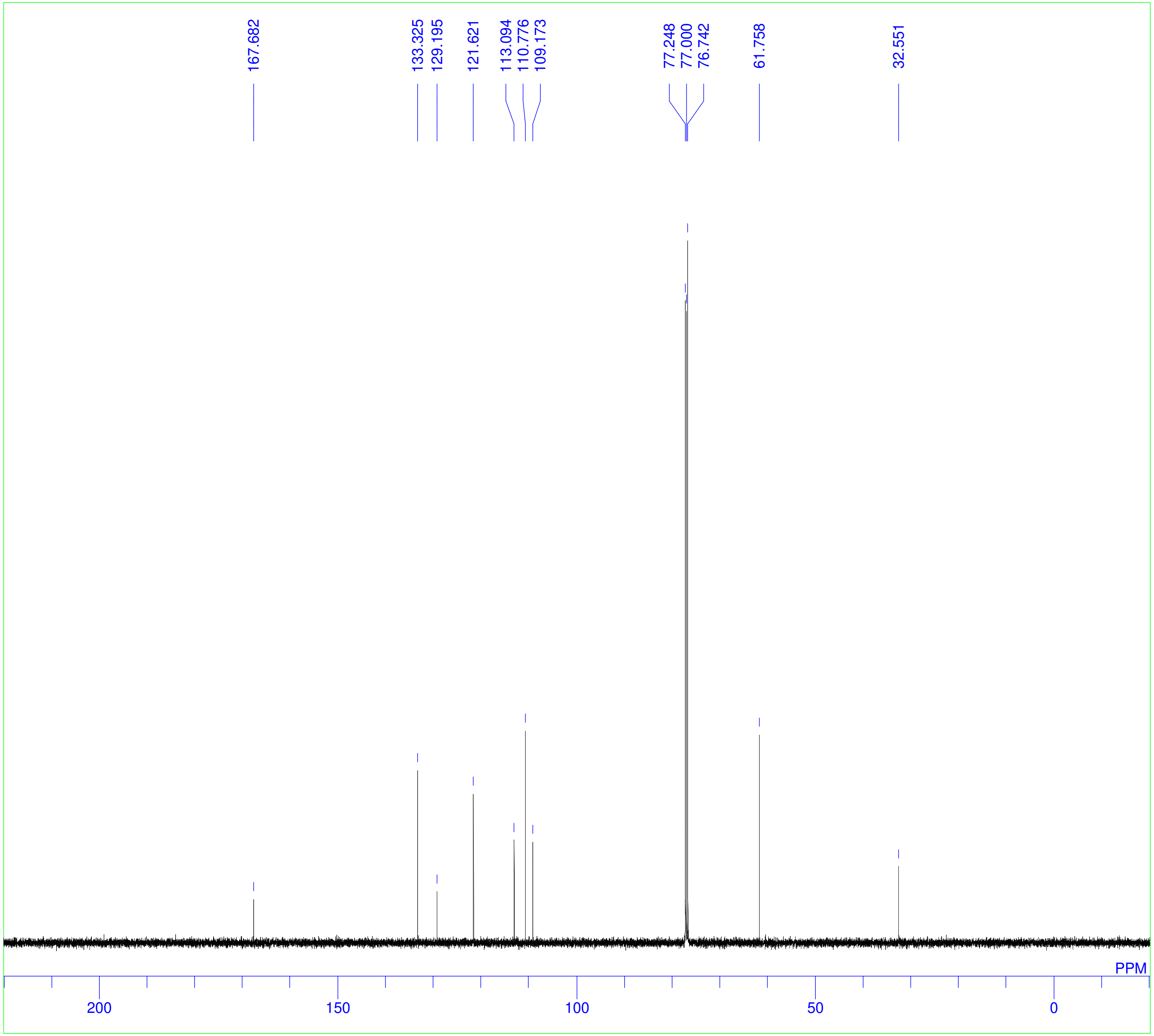

DFILE (E)-20x_13C.als
COMNT
DATIM 2023-04-23 18:08:36
OBNUC 13C
EXMOD carbon.jxp
OBFRQ 125.77 MHz
OBSET 7.87 KHz
OBFIN 4.21 Hz
POINT 26214
FREQU 31446.54 Hz
SCANS 1024
ACQTM 0.8336 sec
PD 2.0000 sec
PW1 3.87 usec
IRNUC 1H
CTEMP 24.1 c
SLVNT CDCL3
EXREF 77.00 ppm
BF 0.30 Hz
RGAIN 30

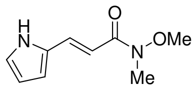

(*E*)-20x

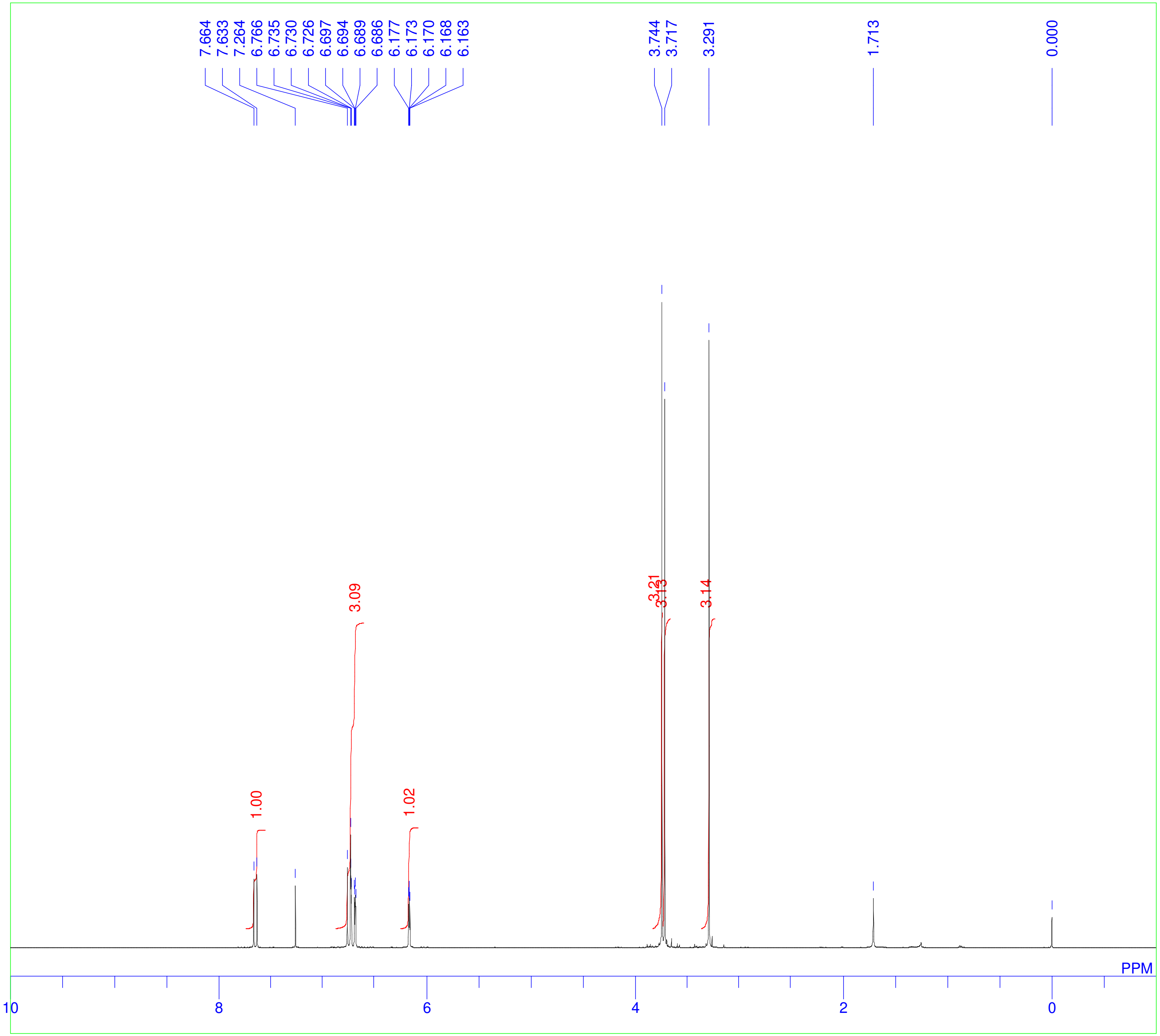

DFILE	(E)-20y_1H.als
COMNT	
DATIM	2023-05-05 16:27:28
OBNUC	1H
EXMOD	proton.jxp
OBFRQ	500.16 MHz
OBSET	2.41 KHz
OBFIN	6.01 Hz
POINT	13107
FREQU	7507.51 Hz
SCANS	8
ACQTM	1.7459 sec
PD	5.0000 sec
PW1	3.84 usec
IRNUC	1H
CTEMP	24.3 c
SLVNT	CDCL3
EXREF	0.00 ppm
BF	0.30 Hz
RGAIN	34

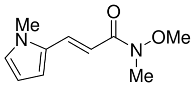

(*E*)-20y

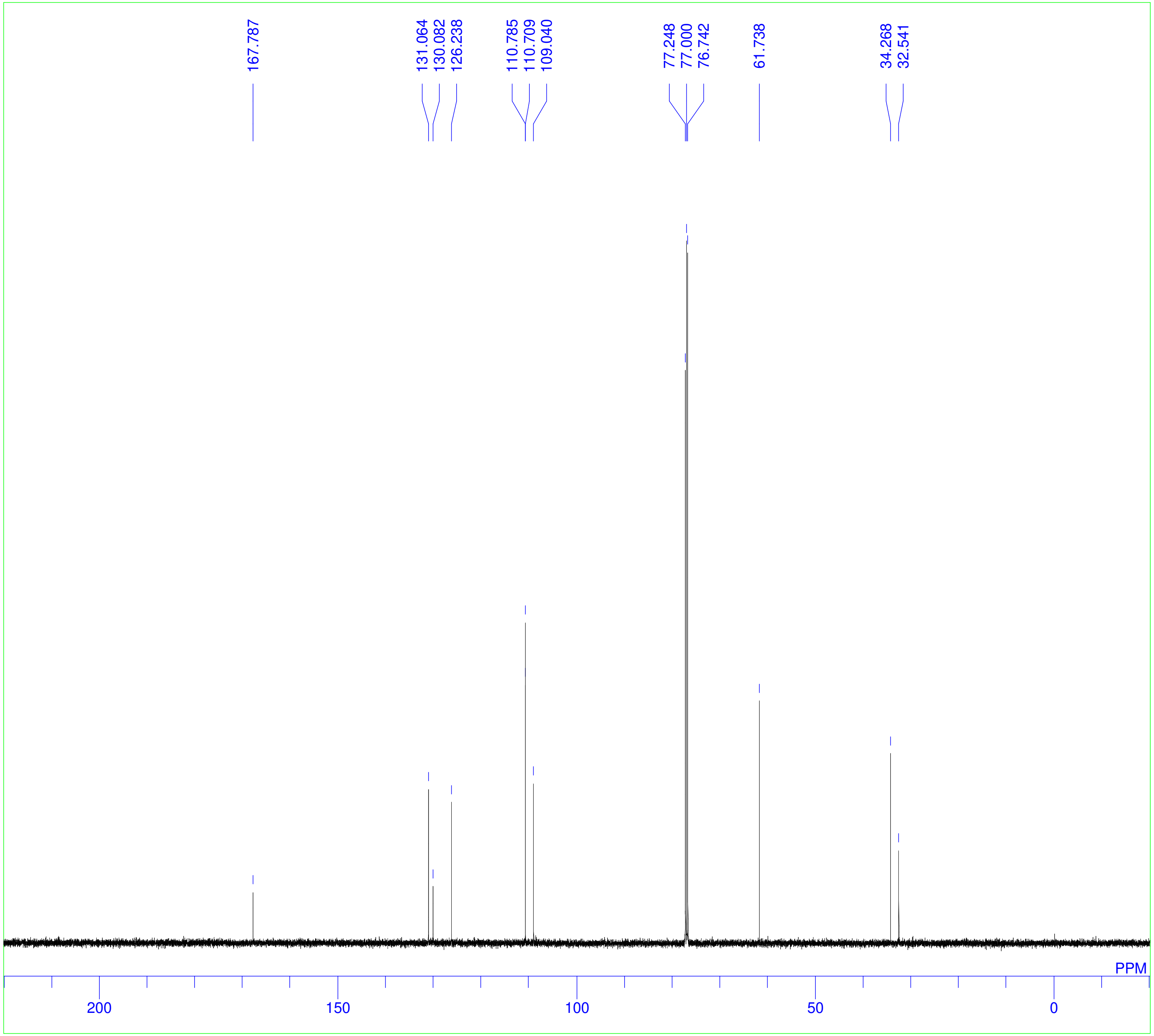

DFILE	(E)-20y_13C.als
COMNT	
DATIM	2023-05-05 16:29:17
OBNUC	13C
EXMOD	carbon.jxp
OBFRQ	125.77 MHz
OBSET	7.87 KHz
OBFIN	4.21 Hz
POINT	26214
FREQU	31446.54 Hz
SCANS	1024
ACQTM	0.8336 sec
PD	2.0000 sec
PW1	3.87 usec
IRNUC	1H
CTEMP	23.8 c
SLVNT	CDCL3
EXREF	77.00 ppm
BF	0.30 Hz
RGAIN	28

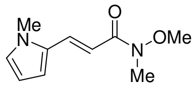

(*E*)-20y

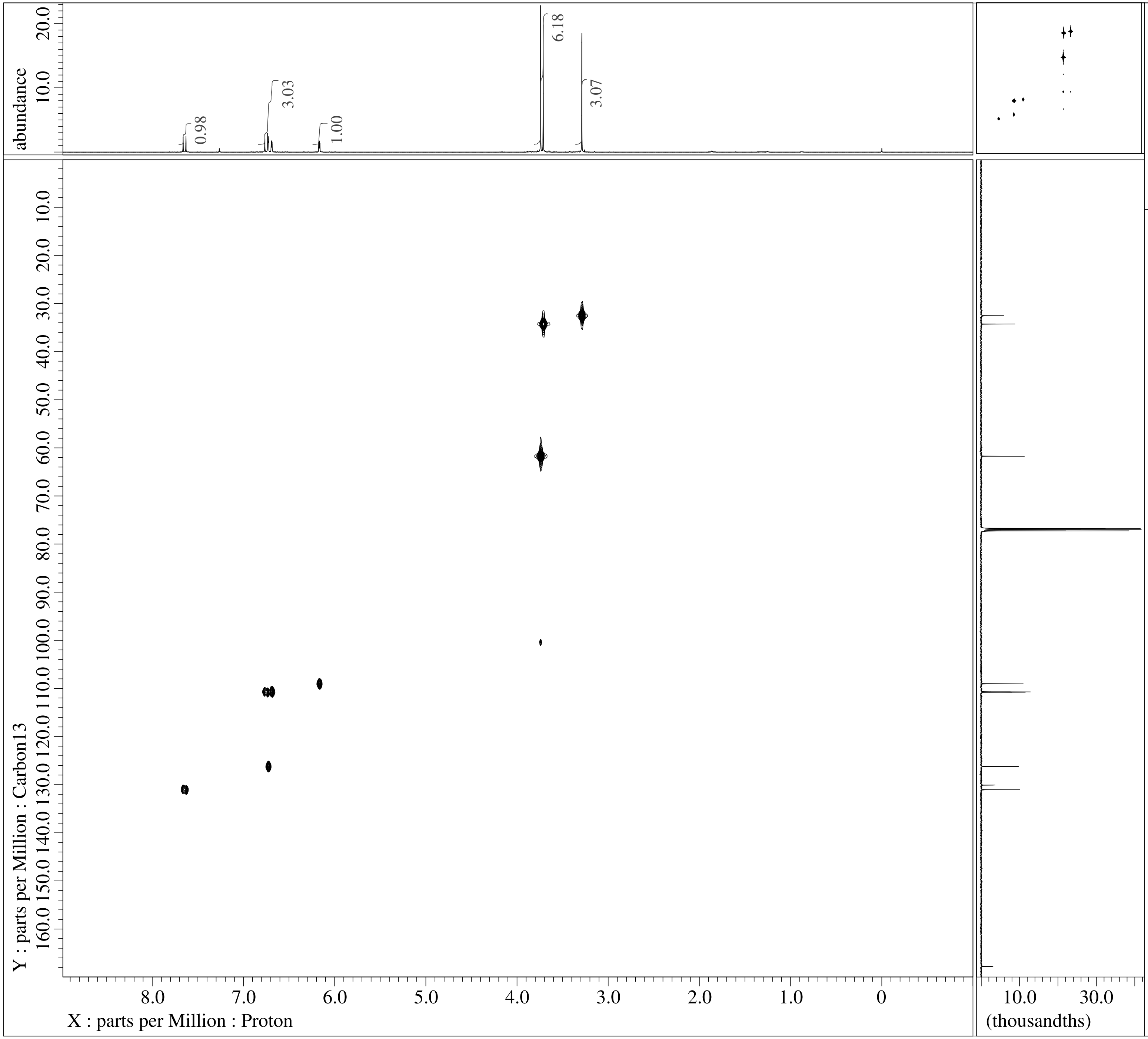


```
---- PROCESSING PARAMETERS ----
sinbell4( -60, 160 )
zerofill( 1 )
fft( 1, TRUE, TRUE )
ppm
[transpose]
sinbell4( -60, 160 )
zerofill( 2 )
fft( 1, TRUE, TRUE )
ppm
abs
```

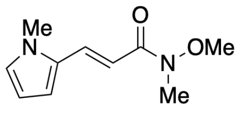

(*E*)-20y

```
Filename            = HT-1710-PTLC 1_HMQC-1-5
Author              = delta
Experiment          = hmqc.jxp
Sample_Id           = HT-1710-PTLC 1
Solvent             = CHLOROFORM-D
Creation_Time       = 18-JAN-2024 18:42:33
Revision_Time       = 11-APR-2024 11:00:07
Current_Time        = 11-APR-2024 11:01:32

Comment             = gradient enhanced HMQC
Data_Format         = 2D REAL REAL
Dim_Size            = 819, 512
Dim_Title           = Proton Carbon13
Dim_Units           = [ppm] [ppm]
Dimensions          = X Y
Site                = JNM-ECA500II
Spectrometer        = DELTA2_NMR

Field_Strength      = 11.7473579[T] (500[MHz]
X_Acq_Duration      = 0.16105472[s]
X_Domain            = 1H
X_Freq              = 500.15991521[MHz]
X_Offset            = 3.88458643[ppm]
X_Points            = 1024
X_Prescans          = 4
X_Resolution        = 6.20906981[Hz]
X_Sweep             = 6.35808749[kHz]
X_Sweep_Clipped     = 5.08646999[kHz]
Y_Domain            = 13C
Y_Freq              = 125.76529768[MHz]
Y_Offset            = 85[ppm]
Y_Points            = 256
Y_Prescans          = 0
Y_Resolution        = 83.60980308[Hz]
Y_Sweep             = 21.40410959[kHz]
Tri_Domain          = Proton
Tri_Freq            = 500.15991521[MHz]
Tri_Offset          = 5.0[ppm]
Clipped             = FALSE
Scans               = 8
Total_Scans         = 2048

Relaxation_Delay    = 1.5[s]
Recvr_Gain          = 50
Temp_Get            = 22.2[dC]
X_Acq_Time          = 0.16105472[s]
X_Atn               = 3.2[dB]
X_Gamma             = 42576375
X_Pulse             = 7.59[us]
```

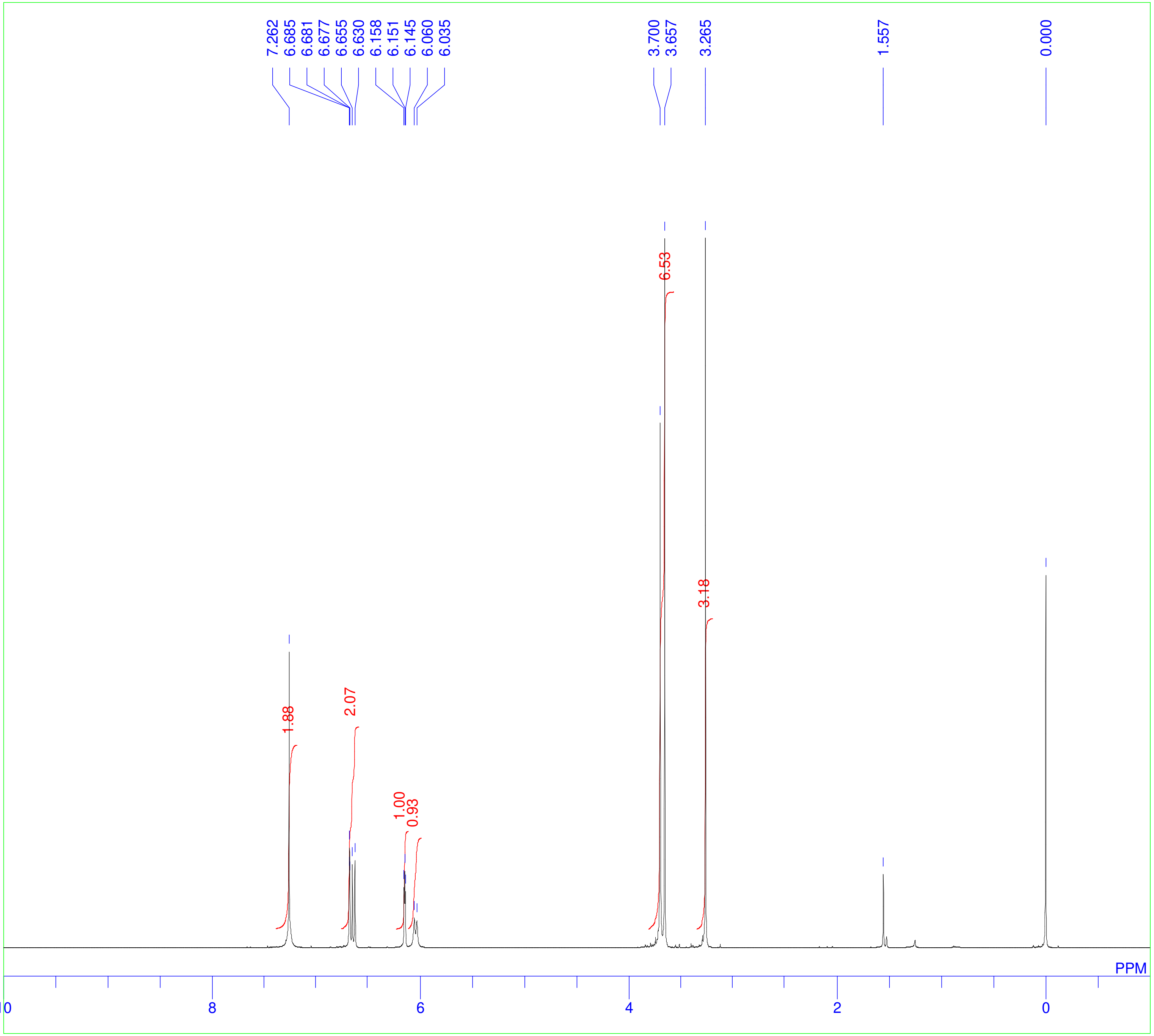

DFILE	(Z)-20y_1H.als
COMNT	
DATIM	2023-12-23 15:04:39
OBNUC	1H
EXMOD	proton.jxp
OBFRQ	500.16 MHz
OBSET	2.41 KHz
OBFIN	6.01 Hz
POINT	13107
FREQU	7507.51 Hz
SCANS	8
ACQTM	1.7459 sec
PD	5.0000 sec
PW1	3.80 usec
IRNUC	1H
CTEMP	22.1 c
SLVNT	CDCL3
EXREF	0.00 ppm
BF	1.00 Hz
RGAIN	48

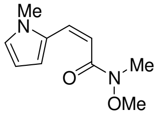

(*Z*)-20y

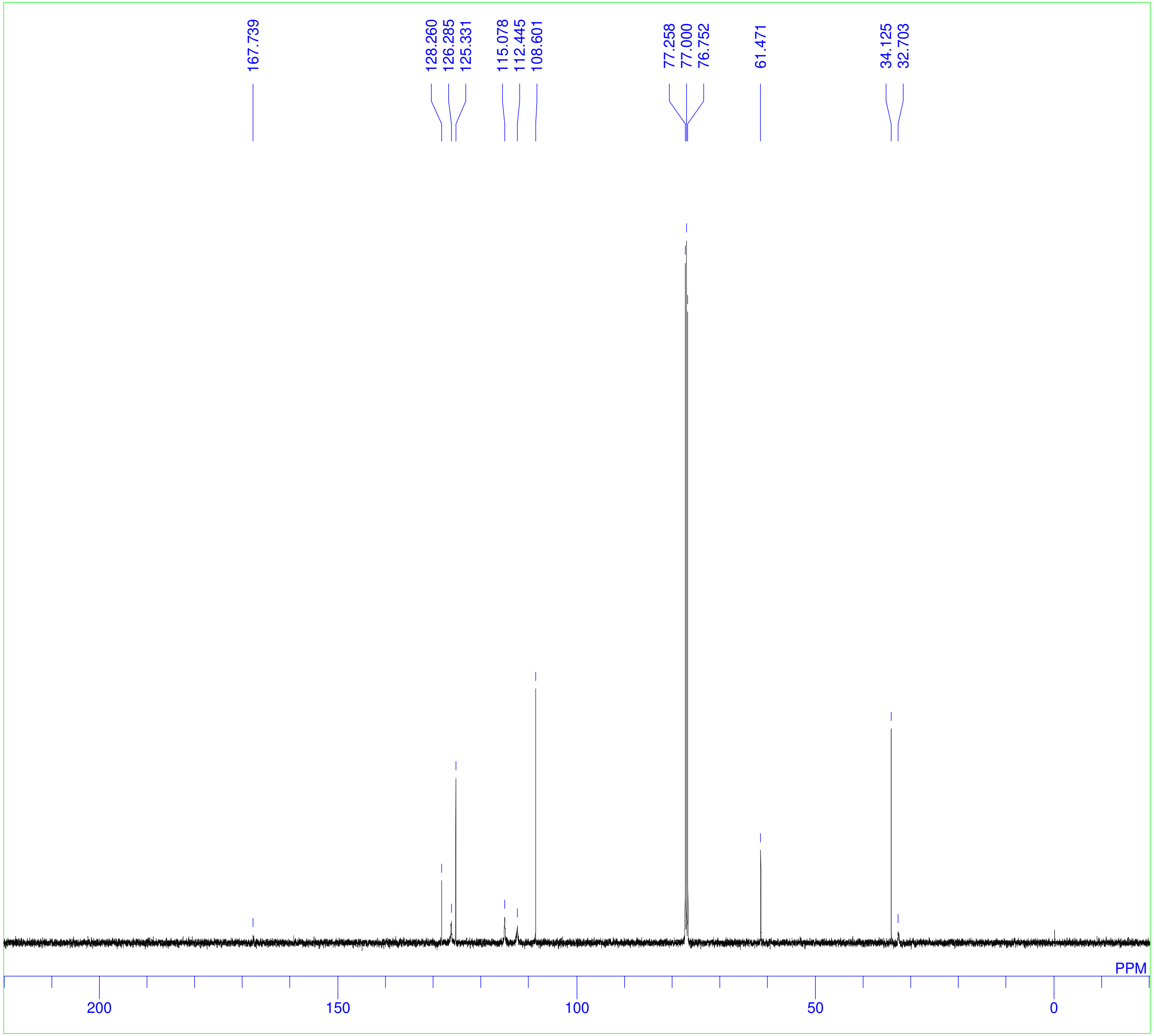

DFILE	(Z)-20y_13C.als
COMNT	
DATIM	2023-12-23 19:50:05
OBNUC	13C
EXMOD	carbon.jxp
OBFRQ	125.77 MHz
OBSET	7.87 KHz
OBFIN	4.21 Hz
POINT	26214
FREQU	31446.54 Hz
SCANS	1024
ACQTM	0.8336 sec
PD	2.0000 sec
PW1	4.30 usec
IRNUC	1H
CTEMP	22.4 c
SLVNT	CDCL3
EXREF	77.00 ppm
BF	1.00 Hz
RGAIN	28

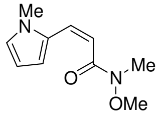

(*Z*)-20y

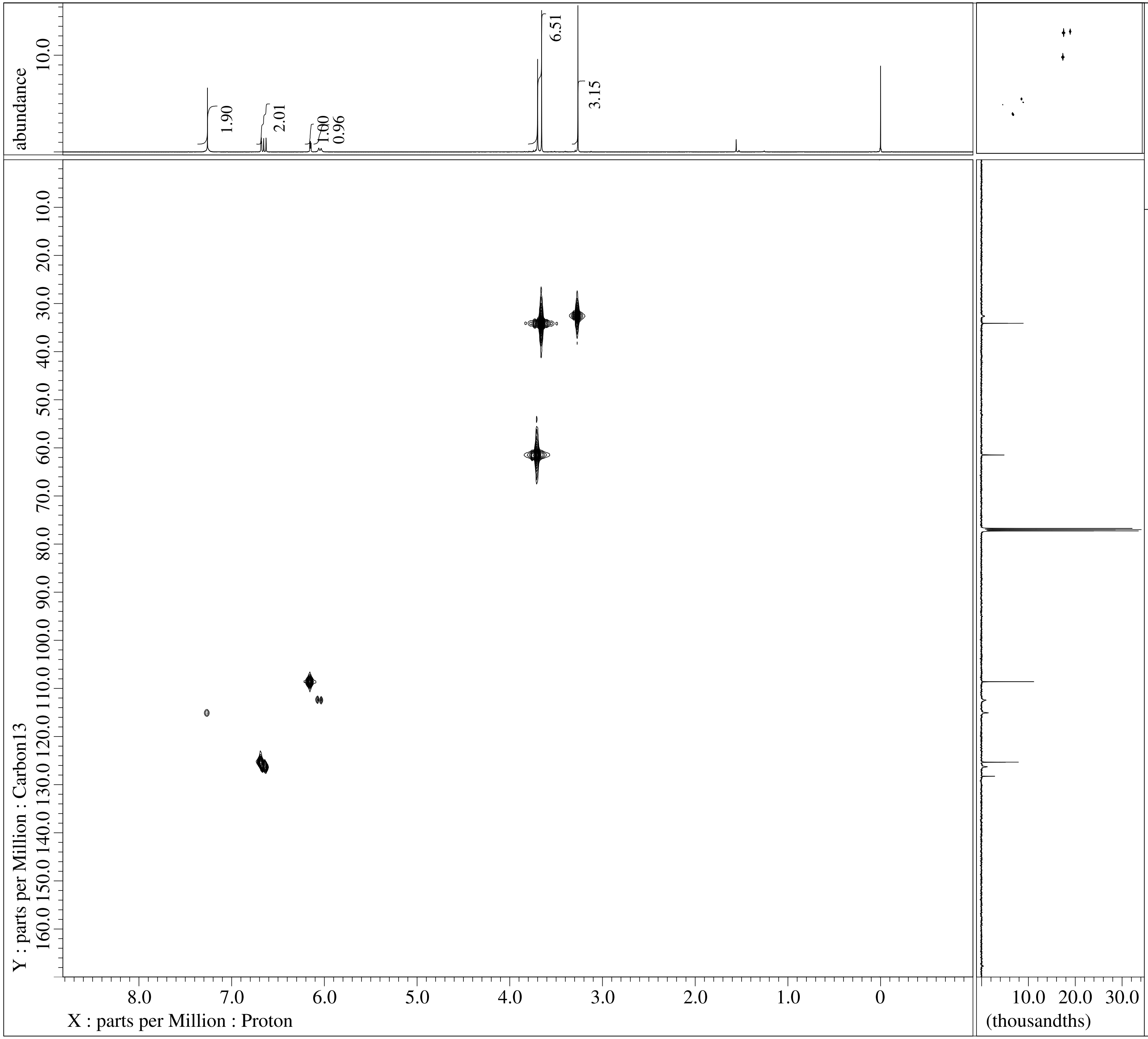

---- PROCESSING PARAMETERS ----
sinbell4(-60, 160)
zerofill(1)
fft(1, TRUE, TRUE)
ppm
[transpose]
sinbell4(-60, 160)
zerofill(2)
fft(1, TRUE, TRUE)
ppm
abs

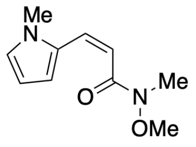

(*Z*)-20y

Filename	= HT-1700-TM_HMQC_HMQC-1-
Author	= delta
Experiment	= hmqc.jxp
Sample_Id	= HT-1700-TM_HMQC
Solvent	= CHLOROFORM-D
Creation_Time	= 15-JAN-2024 20:16:06
Revision_Time	= 11-APR-2024 11:14:13
Current_Time	= 11-APR-2024 11:15:01
Comment	= gradient enhanced HMQC
Data_Format	= 2D REAL REAL
Dim_Size	= 819, 512
Dim_Title	= Proton Carbon13
Dim_Units	= [ppm] [ppm]
Dimensions	= X Y
Site	= JNM-ECA500II
Spectrometer	= DELTA2_NMR
Field_Strength	= 11.7473579[T] (500[MHz]
X_Acq_Duration	= 0.16433152[s]
X_Domain	= 1H
X_Freq	= 500.15991521[MHz]
X_Offset	= 3.81530253[ppm]
X_Points	= 1024
X_Prescans	= 4
X_Resolution	= 6.08525985[Hz]
X_Sweep	= 6.23130608[kHz]
X_Sweep_Clipped	= 4.98504487[kHz]
Y_Domain	= 13C
Y_Freq	= 125.76529768[MHz]
Y_Offset	= 85[ppm]
Y_Points	= 256
Y_Prescans	= 0
Y_Resolution	= 83.60980308[Hz]
Y_Sweep	= 21.40410959[kHz]
Tri_Domain	= Proton
Tri_Freq	= 500.15991521[MHz]
Tri_Offset	= 5.0[ppm]
Clipped	= FALSE
Scans	= 8
Total_Scans	= 2048
Relaxation_Delay	= 1.5[s]
Recvr_Gain	= 50
Temp_Get	= 21.4[dC]
X_Acq_Time	= 0.16433152[s]
X_Atn	= 3.2[dB]
X_Gamma	= 42576375
X_Pulse	= 7.59[us]

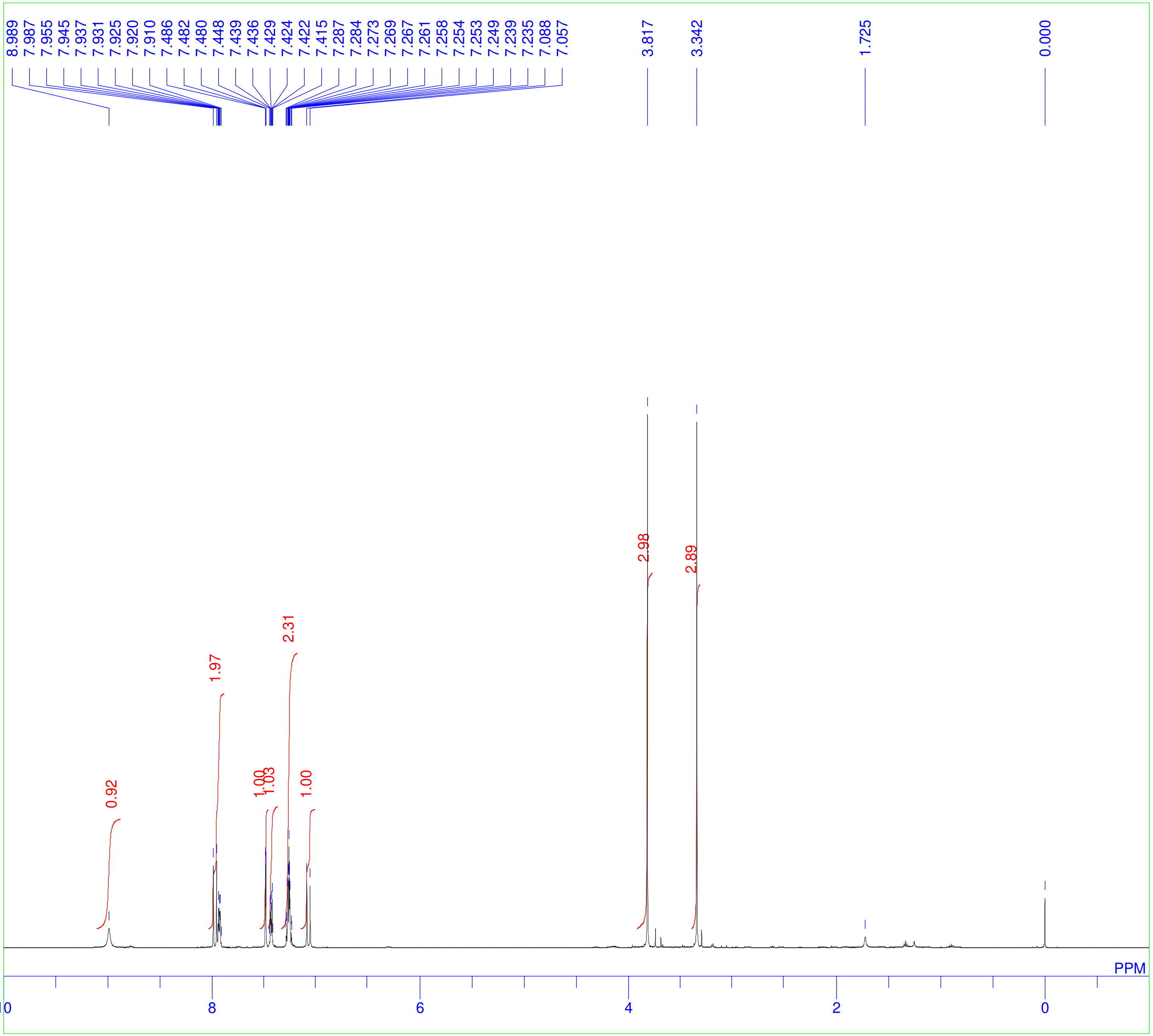

DFILE	(E)-20z_1H.als
COMNT	
DATIM	2023-12-21 20:48:01
OBNUC	1H
EXMOD	proton.jxp
OBFRQ	500.16 MHz
OBSET	2.41 KHz
OBFIN	6.01 Hz
POINT	13107
FREQU	7507.51 Hz
SCANS	8
ACQTM	1.7459 sec
PD	5.0000 sec
PW1	3.80 usec
IRNUC	1H
CTEMP	23.3 c
SLVNT	CDCL3
EXREF	0.00 ppm
BF	0.30 Hz
RGAIN	36

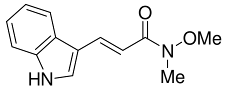

(*E*)-20z

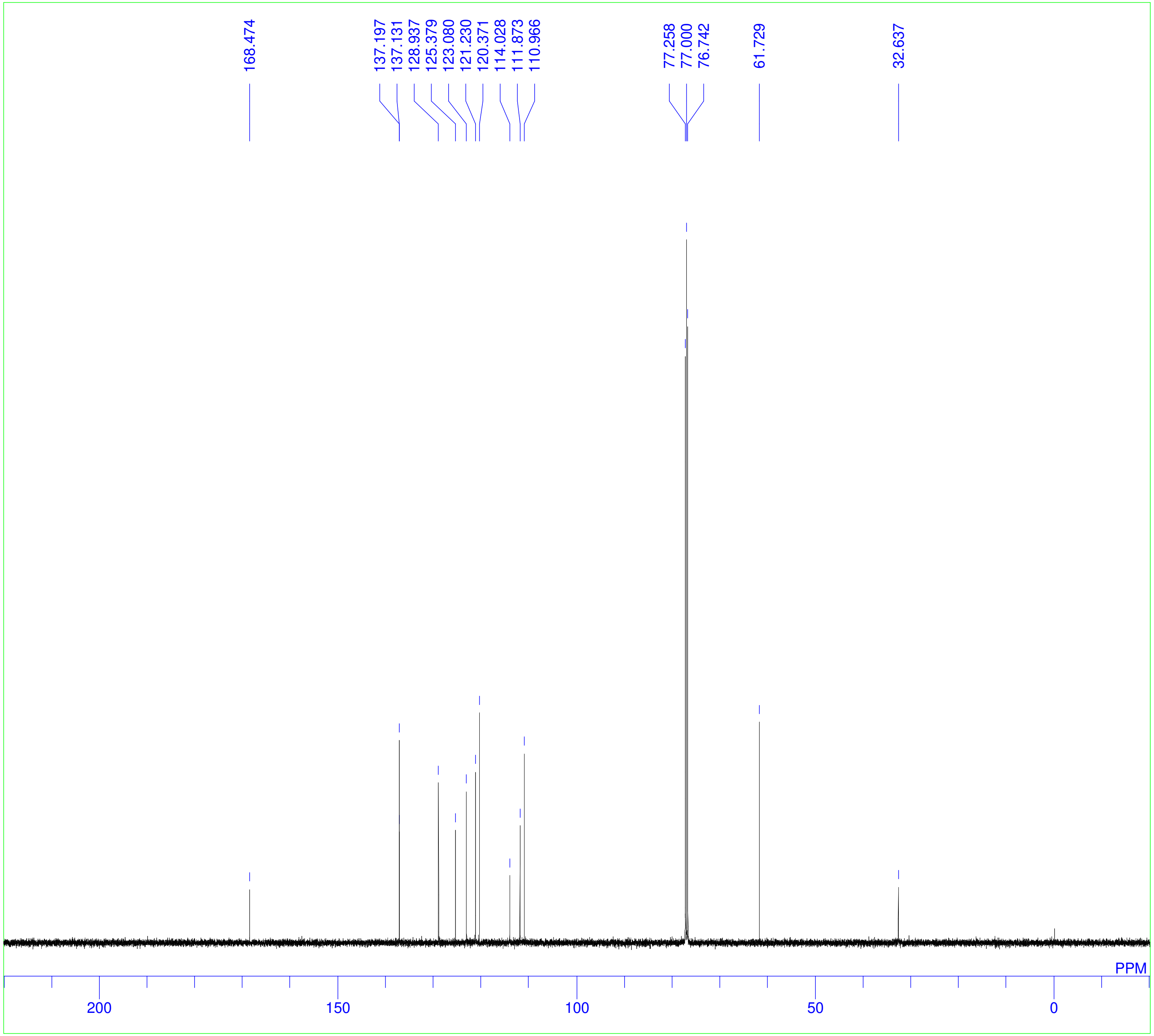

DFILE	(E)-20z_13C.als
COMNT	
DATIM	2023-12-21 20:49:45
OBNUC	13C
EXMOD	carbon.jxp
OBFRQ	125.77 MHz
OBSET	7.87 KHz
OBFIN	4.21 Hz
POINT	26214
FREQU	31446.54 Hz
SCANS	1024
ACQTM	0.8336 sec
PD	2.0000 sec
PW1	4.30 usec
IRNUC	1H
CTEMP	23.3 c
SLVNT	CDCL3
EXREF	77.00 ppm
BF	0.30 Hz
RGAIN	28

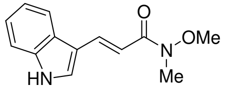

(*E*)-20z

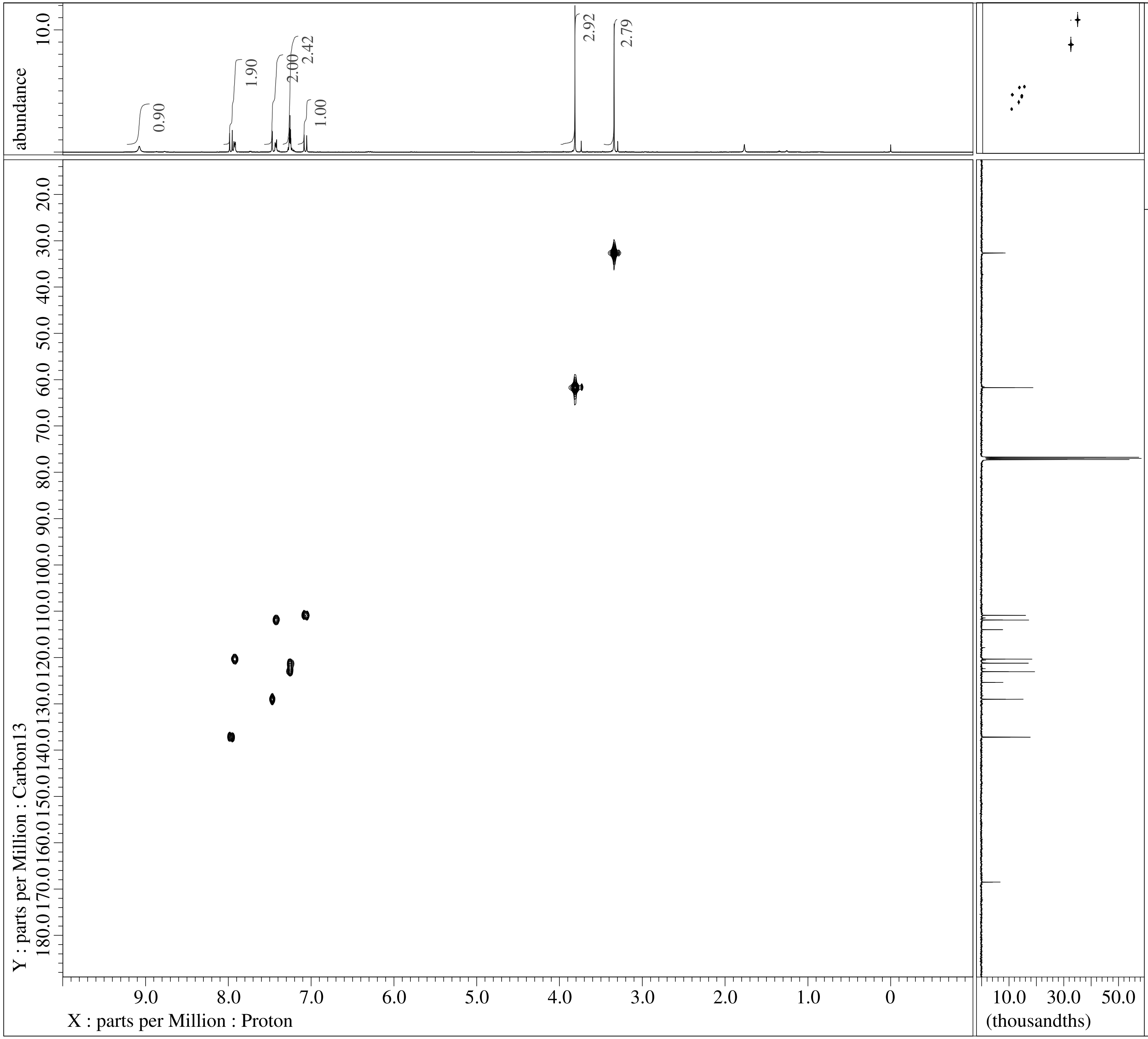


```
---- PROCESSING PARAMETERS ----
dc_balance( 0, FALSE )
sexp( 0.2[Hz], 0.0[s] )
trapezoid( 0[%], 0[%], 80[%], 100[%] )
zerofill( 1 )
fft( 1, TRUE, TRUE )
machinephase
ppm
```

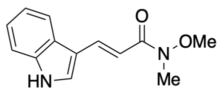

(*E*)-20z

```
Filename            = HT-1568-PTLC 3_13C_HMQC
Author              = delta
Experiment          = hmqc.jxp
Sample_Id           = HT-1568-PTLC 3_13C
Solvent             = CHLOROFORM-D
Creation_Time       =  3-OCT-2023 18:48:54
Revision_Time       = 11-APR-2024 11:19:19
Current_Time        = 11-APR-2024 11:20:12

Comment             = gradient enhanced HMQC
Data_Format         = 2D REAL REAL
Dim_Size            = 819, 512
Dim_Title           = Proton Carbon13
Dim_Units           = [ppm] [ppm]
Dimensions          = X Y
Site                = JNM-ECA500II
Spectrometer        = DELTA2_NMR

Field_Strength      = 11.7473579[T] (500[MHz]
X_Acq_Duration      = 0.13877248[s]
X_Domain            = 1H
X_Freq              = 500.15991521[MHz]
X_Offset            = 4.52990013[ppm]
X_Points            = 1024
X_Prescans          = 4
X_Resolution        = 7.2060397[Hz]
X_Sweep             = 7.37898465[kHz]
X_Sweep_Clipped     = 5.90318772[kHz]
Y_Domain            = 13C
Y_Freq              = 125.76529768[MHz]
Y_Offset            = 100.69629776[ppm]
Y_Points            = 256
Y_Prescans          = 0
Y_Resolution        = 86.8827847[Hz]
Y_Sweep             = 22.24199288[kHz]
Tri_Domain          = Proton
Tri_Freq            = 500.15991521[MHz]
Tri_Offset          = 5.0[ppm]
Clipped             = FALSE
Scans               = 8
Total_Scans         = 2048

Relaxation_Delay    = 1.5[s]
Recvr_Gain          = 50
Temp_Get            = 23.9[dC]
X_Acq_Time          = 0.13877248[s]
X_Atn               = 3.2[dB]
X_Gamma             = 42576375
X_Pulse             = 7.59[us]
```

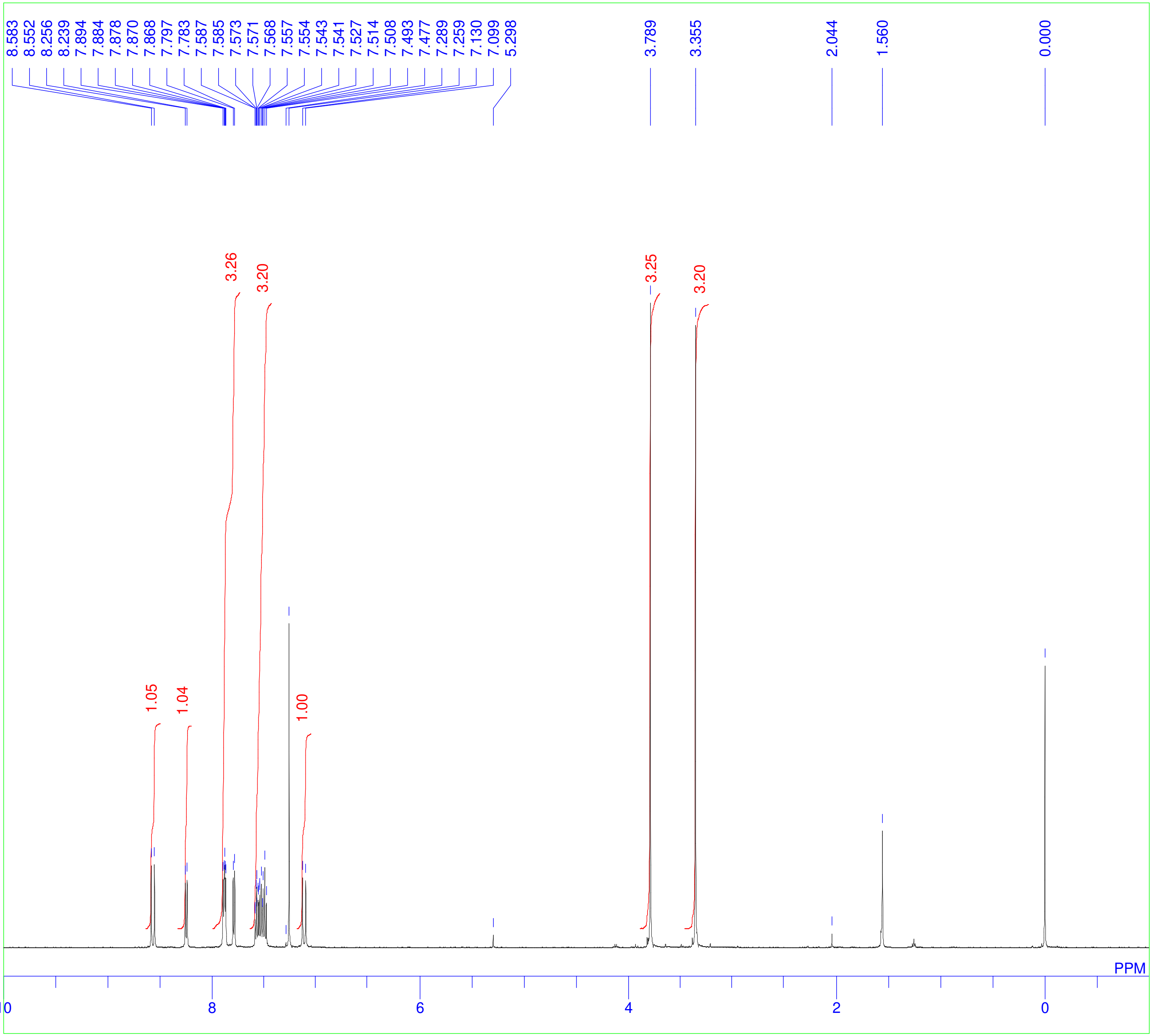

DFILE (E)-20aa_1H.als
COMNT
DATIM 2023-05-05 15:11:42
OBNUC 1H
EXMOD proton.jxp
OBFRQ 500.16 MHz
OBSET 2.41 KHz
OBFIN 6.01 Hz
POINT 13107
FREQU 7507.51 Hz
SCANS 8
ACQTM 1.7459 sec
PD 5.0000 sec
PW1 3.84 usec
IRNUC 1H
CTEMP 24.3 c
SLVNT CDCL3
EXREF 0.00 ppm
BF 1.00 Hz
RGAIN 48

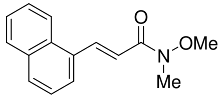

(*E*)-20aa

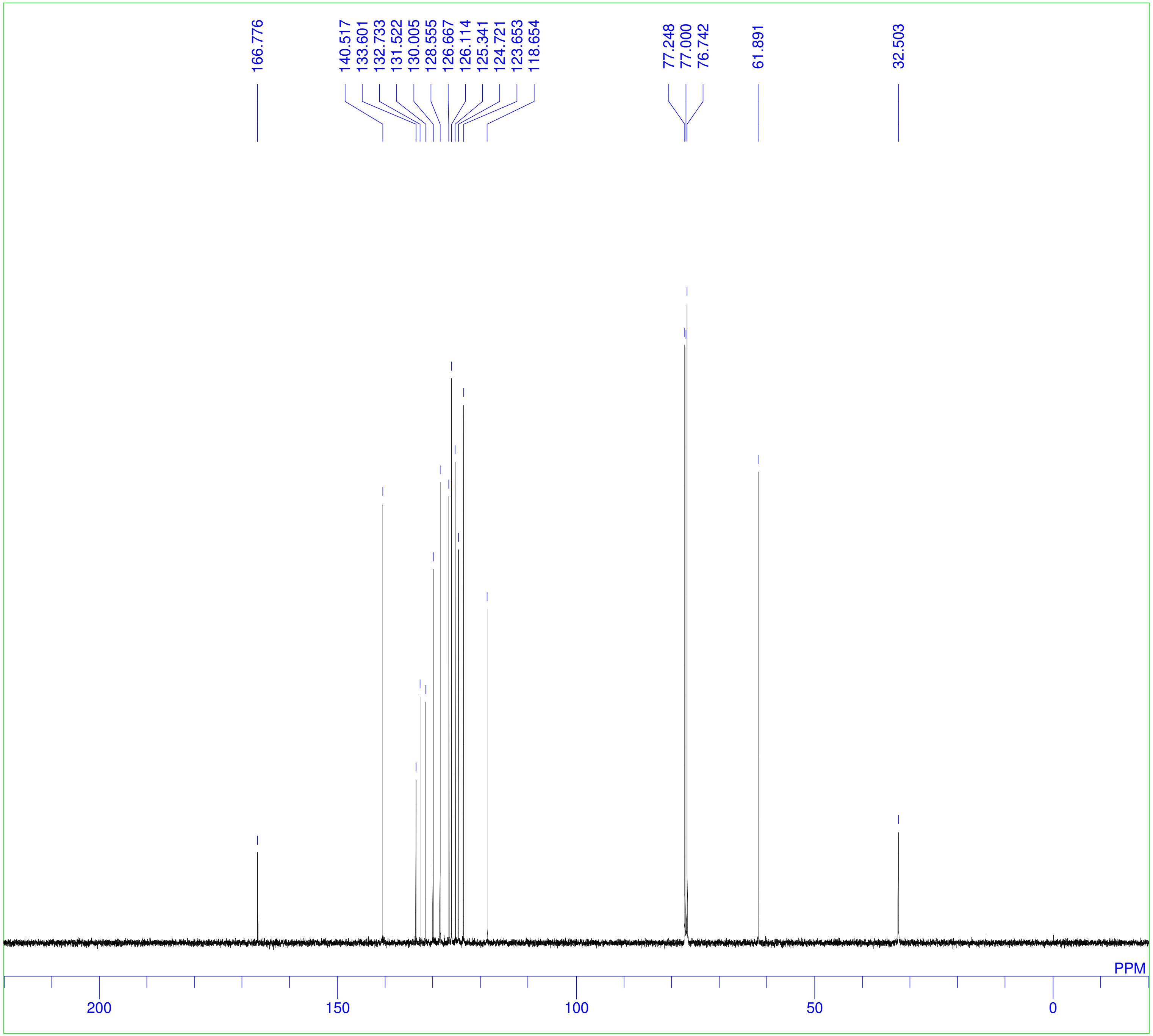

DFILE	(E)-20aa_13C.als
COMNT	
DATIM	2023-05-05 19:55:30
OBNUC	13C
EXMOD	carbon.jxp
OBFRQ	125.77 MHz
OBSET	7.87 KHz
OBFIN	4.21 Hz
POINT	26214
FREQU	31446.54 Hz
SCANS	1024
ACQTM	0.8336 sec
PD	2.0000 sec
PW1	3.87 usec
IRNUC	1H
CTEMP	23.9 c
SLVNT	CDCL3
EXREF	77.00 ppm
BF	1.00 Hz
RGAIN	32

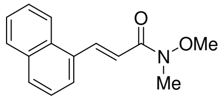

(*E*)-20aa

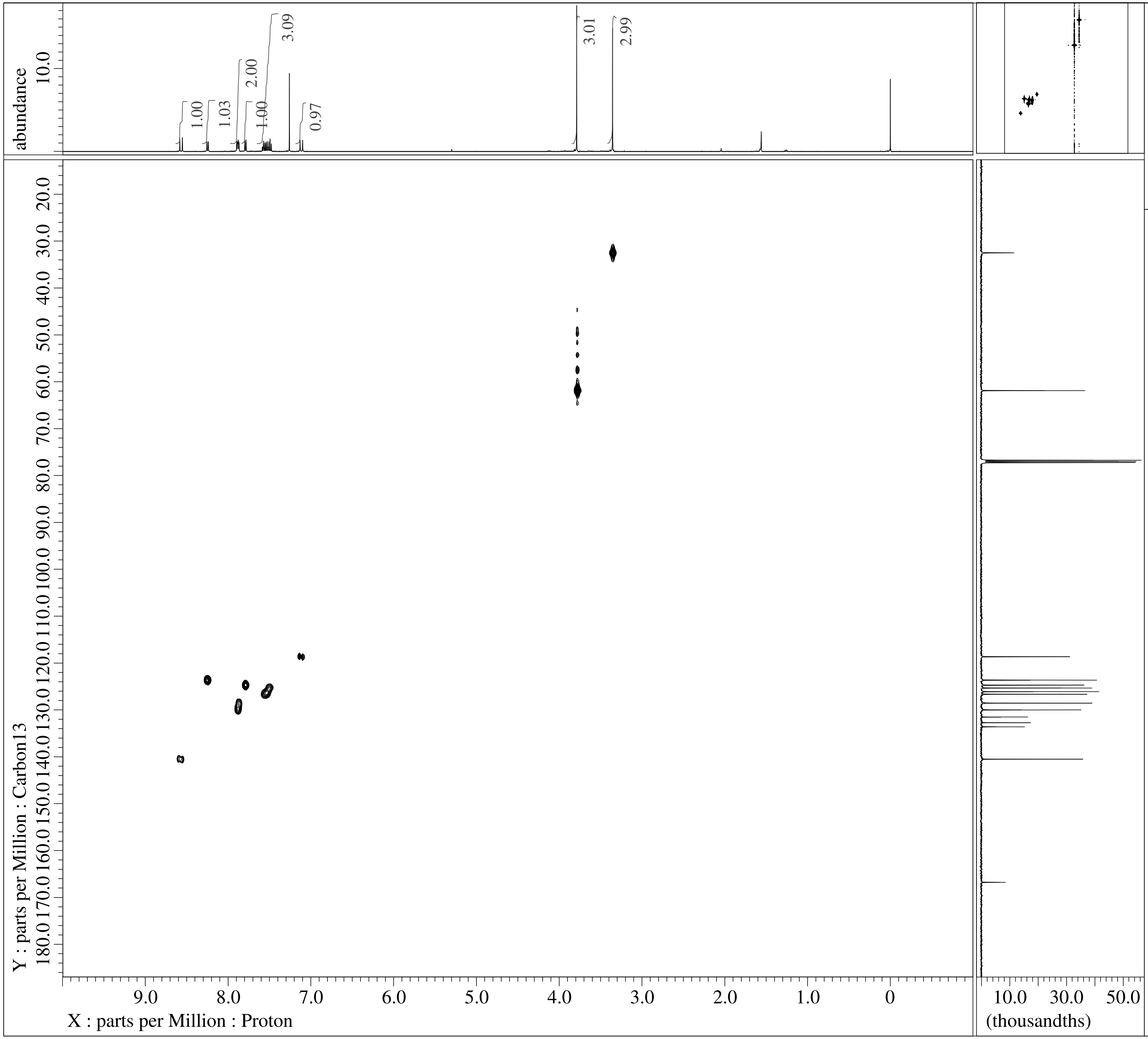

---- PROCESSING PARAMETERS ----
sinbell4(-60, 160)
zerofill(1)
fft(1, TRUE, TRUE)
ppm
[transpose]
sinbell4(-60, 160)
zerofill(2)
fft(1, TRUE, TRUE)
ppm
abs

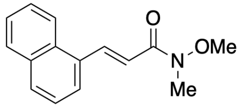

(*E*)-20aa

Filename	= HT-1392-PTLC 1_13C_HMQC
Author	= delta
Experiment	= hmqc.jxp
Sample_Id	= HT-1392-PTLC 1_13C
Solvent	= CHLOROFORM-D
Creation_Time	= 5-MAY-2023 20:44:21
Revision_Time	= 11-APR-2024 11:22:18
Current_Time	= 11-APR-2024 11:23:10
Comment	= gradient enhanced HMQC
Data_Format	= 2D REAL REAL
Dim_Size	= 819, 512
Dim_Title	= Proton Carbon13
Dim_Units	= [ppm] [ppm]
Dimensions	= X Y
Site	= JNM-ECA500II
Spectrometer	= DELTA2_NMR
Field_Strength	= 11.7473579[T] (500[MHz]
X_Acq_Duration	= 0.10911744[s]
X_Domain	= 1H
X_Freq	= 500.15991521[MHz]
X_Offset	= 5.0[ppm]
X_Points	= 1024
X_Prescans	= 4
X_Resolution	= 9.16443788[Hz]
X_Sweep	= 9.38438438[kHz]
X_Sweep_Clipped	= 7.50750751[kHz]
Y_Domain	= 13C
Y_Freq	= 125.76529768[MHz]
Y_Offset	= 99.78538768[ppm]
Y_Points	= 256
Y_Prescans	= 0
Y_Resolution	= 85.81392794[Hz]
Y_Sweep	= 21.96836555[kHz]
Tri_Domain	= Proton
Tri_Freq	= 500.15991521[MHz]
Tri_Offset	= 5.0[ppm]
Clipped	= FALSE
Scans	= 8
Total_Scans	= 2048
Relaxation_Delay	= 1.5[s]
Recvr_Gain	= 50
Temp_Get	= 23.9[dC]
X_Acq_Time	= 0.10911744[s]
X_Atn	= 3.2[dB]
X_Gamma	= 42576375
X_Pulse	= 7.68[us]

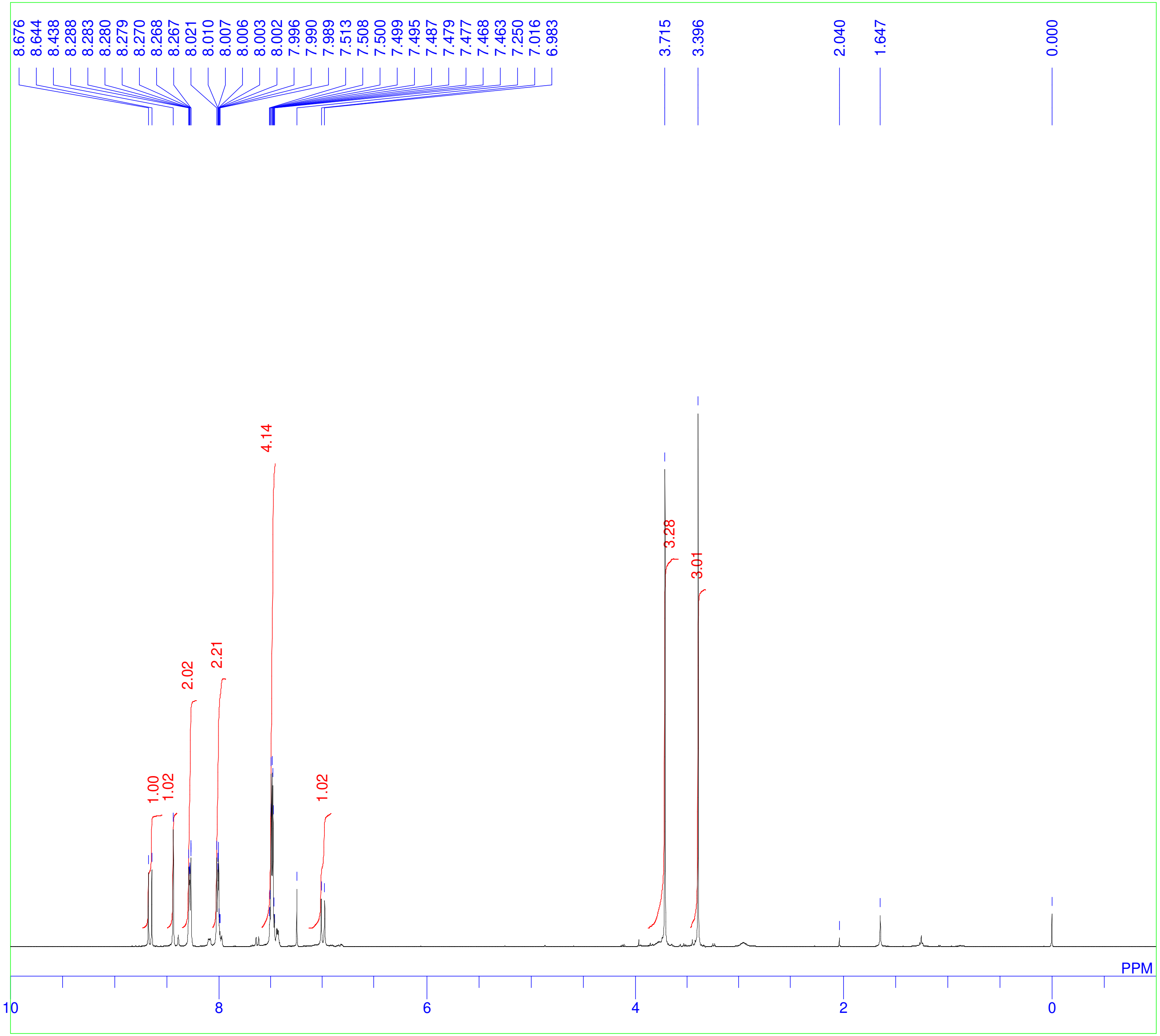

DFILE (E)-20ab_1H.als
COMNT
DATIM 2023-05-05 15:24:58
OBNUC 1H
EXMOD proton.jxp
OBFRQ 500.16 MHz
OBSET 2.41 KHz
OBFIN 6.01 Hz
POINT 13107
FREQU 7507.51 Hz
SCANS 8
ACQTM 1.7459 sec
PD 5.0000 sec
PW1 3.84 usec
IRNUC 1H
CTEMP 23.7 c
SLVNT CDCL3
EXREF 0.00 ppm
BF 1.00 Hz
RGAIN 34

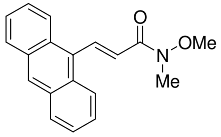

(*E*)-20ab

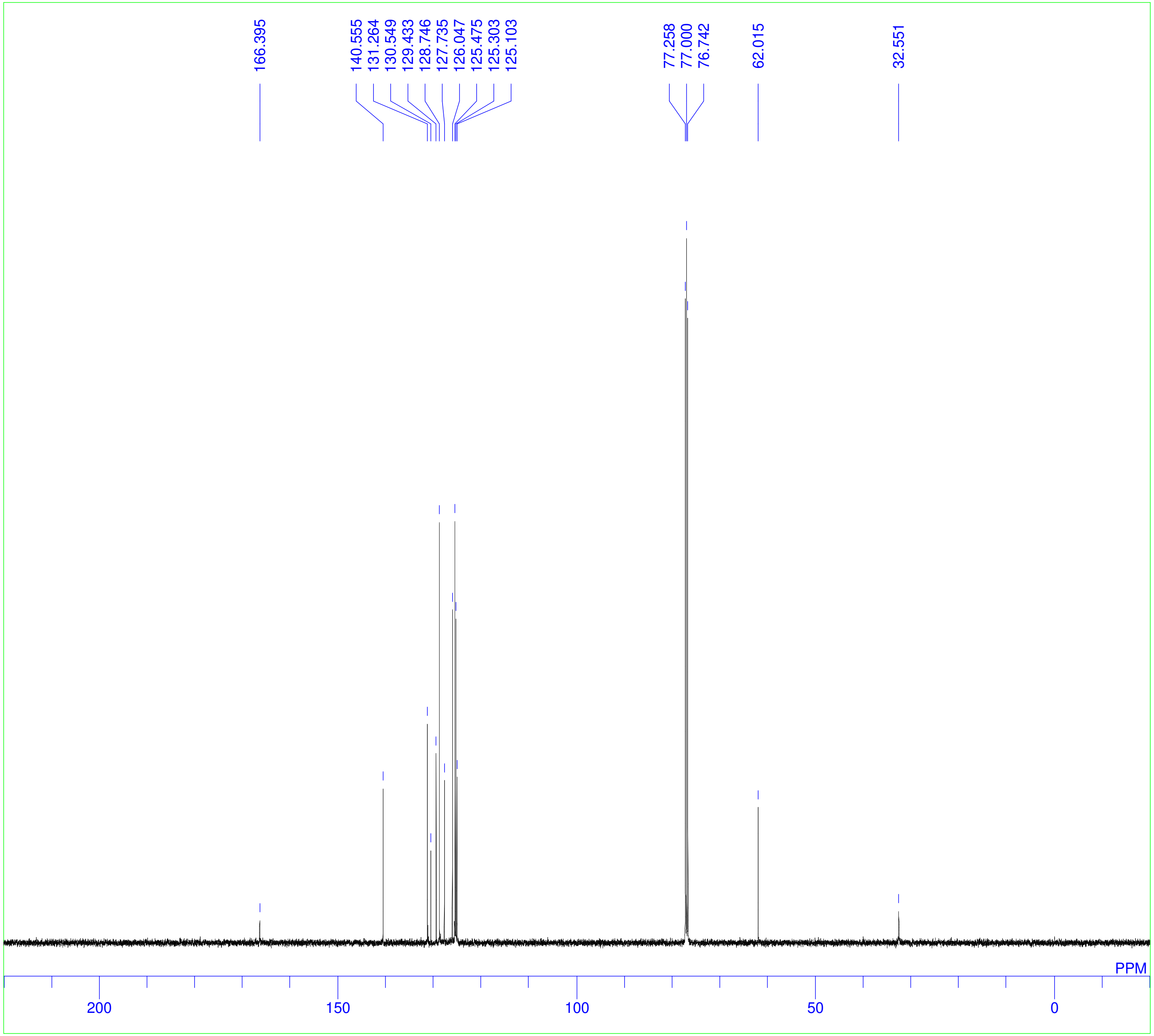

DFILE (E)-20ab_13C.als
COMNT
DATIM 2023-05-05 15:26:41
OBNUC 13C
EXMOD carbon.jxp
OBFRQ 125.77 MHz
OBSET 7.87 KHz
OBFIN 4.21 Hz
POINT 26214
FREQU 31446.54 Hz
SCANS 1024
ACQTM 0.8336 sec
PD 2.0000 sec
PW1 3.87 usec
IRNUC 1H
CTEMP 24.3 c
SLVNT CDCL3
EXREF 77.00 ppm
BF 1.00 Hz
RGAIN 28

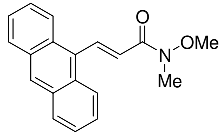

(*E*)-20ab

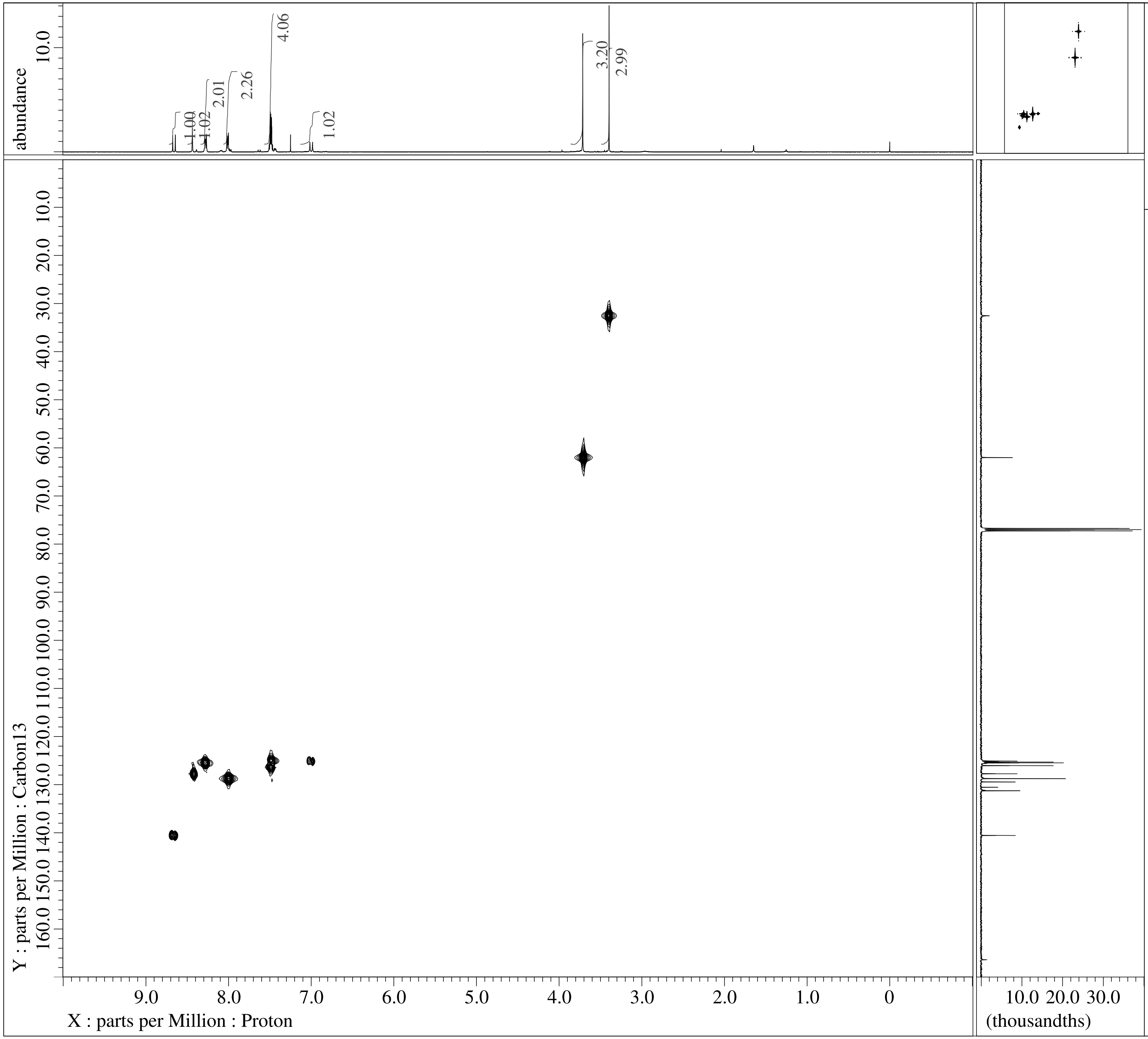

---- PROCESSING PARAMETERS ----
sinbell4(-60, 160)
zerofill(1)
fft(1, TRUE, TRUE)
ppm
[transpose]
sinbell4(-60, 160)
zerofill(2)
fft(1, TRUE, TRUE)
ppm
abs

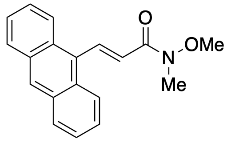

(*E*)-20ab

Filename	= HT-1708-PTLC 1_HMQC,HMB
Author	= delta
Experiment	= hmqc.jxp
Sample_Id	= HT-1708-PTLC 1_HMQC,HMB
Solvent	= CHLOROFORM-D
Creation_Time	= 13-JAN-2024 12:08:48
Revision_Time	= 11-APR-2024 11:28:16
Current_Time	= 11-APR-2024 11:28:58
Comment	= gradient enhanced HMQC
Data_Format	= 2D REAL REAL
Dim_Size	= 819, 512
Dim_Title	= Proton Carbon13
Dim_Units	= [ppm] [ppm]
Dimensions	= X Y
Site	= JNM-ECA500II
Spectrometer	= DELTA2_NMR
Field_Strength	= 11.7473579[T] (500[MHz]
X_Acq_Duration	= 0.10911744[s]
X_Domain	= 1H
X_Freq	= 500.15991521[MHz]
X_Offset	= 5.0[ppm]
X_Points	= 1024
X_Prescans	= 4
X_Resolution	= 9.16443788[Hz]
X_Sweep	= 9.38438438[kHz]
X_Sweep_Clipped	= 7.50750751[kHz]
Y_Domain	= 13C
Y_Freq	= 125.76529768[MHz]
Y_Offset	= 85[ppm]
Y_Points	= 256
Y_Prescans	= 0
Y_Resolution	= 83.60980308[Hz]
Y_Sweep	= 21.40410959[kHz]
Tri_Domain	= Proton
Tri_Freq	= 500.15991521[MHz]
Tri_Offset	= 5.0[ppm]
Clipped	= FALSE
Scans	= 4
Total_Scans	= 1024
Relaxation_Delay	= 1.5[s]
Recvr_Gain	= 50
Temp_Get	= 22[dC]
X_Acq_Time	= 0.10911744[s]
X_Atn	= 3.2[dB]
X_Gamma	= 42576375
X_Pulse	= 7.59[us]

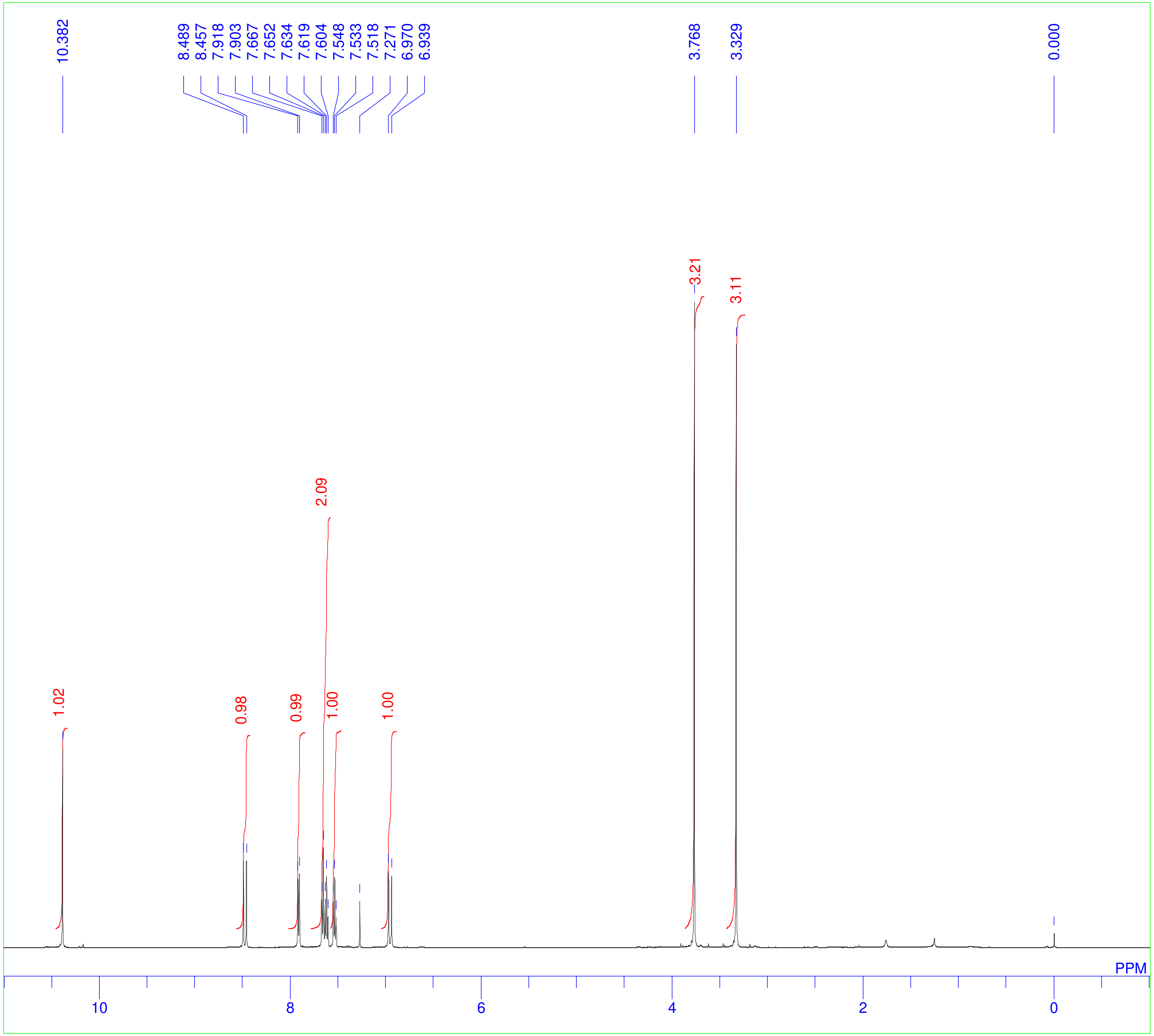

DFILE	(E)-20ac_1H.als
COMNT	
DATIM	2023-04-27 20:06:15
OBNUC	1H
EXMOD	proton.jxp
OBFRQ	500.16 MHz
OBSET	2.41 KHz
OBFIN	6.01 Hz
POINT	13107
FREQU	7507.51 Hz
SCANS	8
ACQTM	1.7459 sec
PD	5.0000 sec
PW1	3.84 usec
IRNUC	1H
CTEMP	24.2 c
SLVNT	CDCL3
EXREF	0.00 ppm
BF	1.00 Hz
RGAIN	38

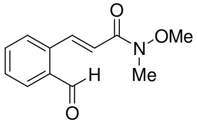

(*E*)-20ac

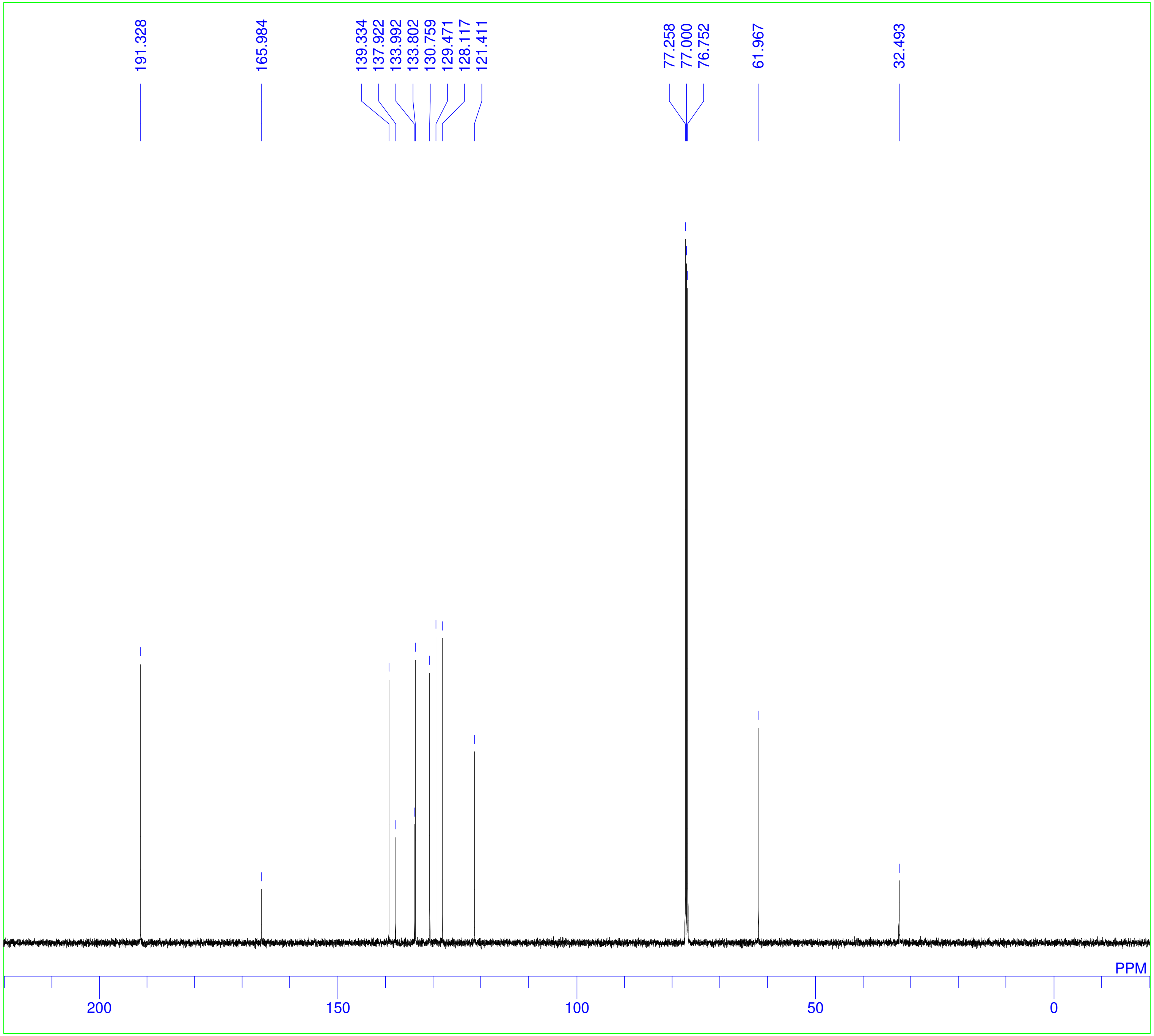

DFILE (E)-20ac_13C.als
COMNT
DATIM 2023-04-27 20:07:58
OBNUC 13C
EXMOD carbon.jxp
OBFRQ 125.77 MHz
OBSET 7.87 KHz
OBFIN 4.21 Hz
POINT 26214
FREQU 31446.54 Hz
SCANS 1024
ACQTM 0.8336 sec
PD 2.0000 sec
PW1 3.87 usec
IRNUC 1H
CTEMP 24.1 c
SLVNT CDCL3
EXREF 77.00 ppm
BF 1.00 Hz
RGAIN 34

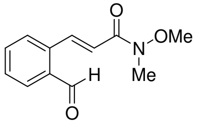

(*E*)-20ac

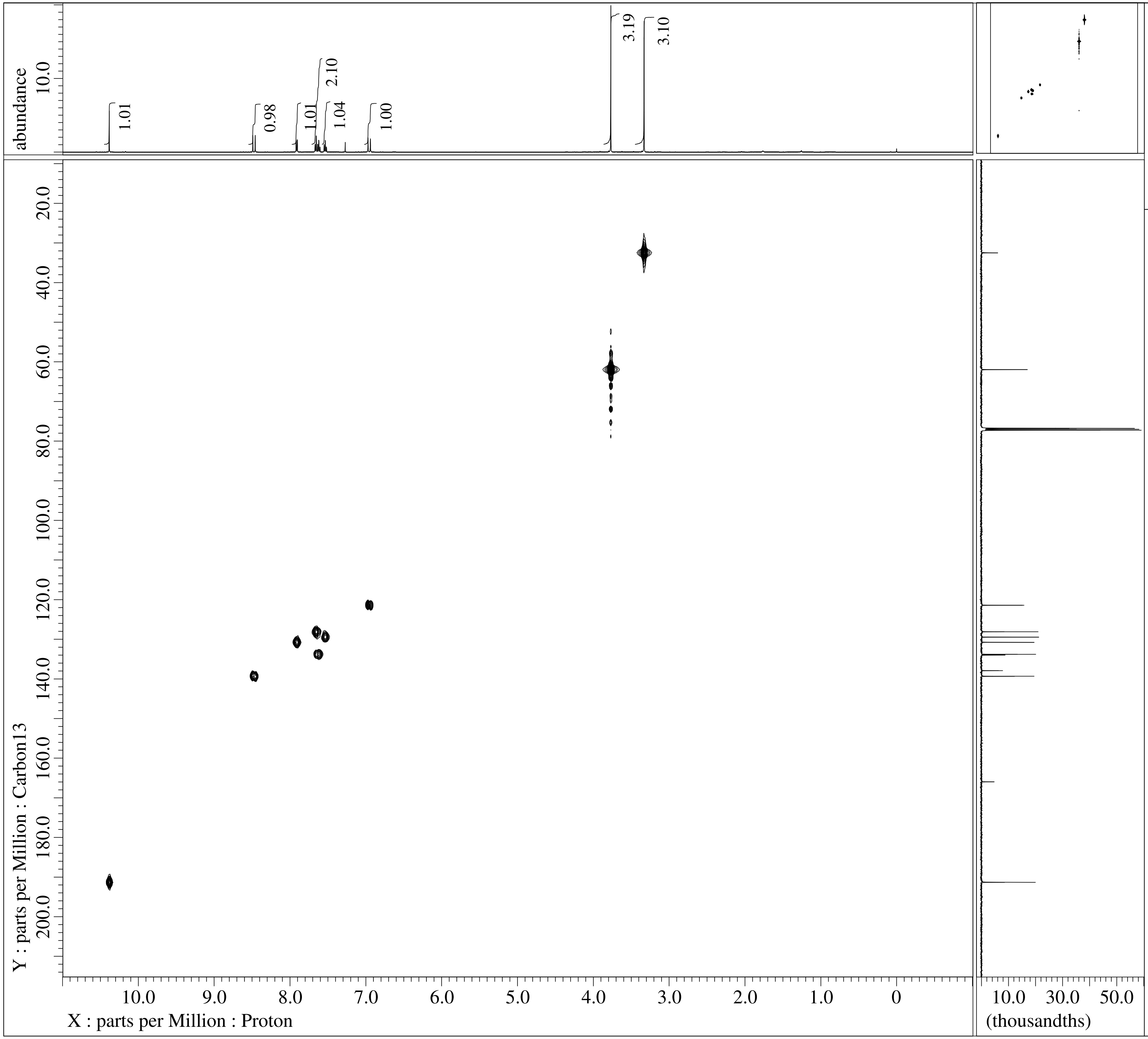


```
---- PROCESSING PARAMETERS ----
sinbell4( -60, 160 )
zerofill( 1 )
fft( 1, TRUE, TRUE )
ppm
[transpose]
sinbell4( -60, 160 )
zerofill( 2 )
fft( 1, TRUE, TRUE )
ppm
abs
```

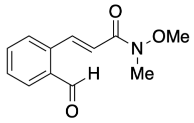

(*E*)-20ac

```
Filename            = HT-1382-PTLC 1_HMQC-1-5
Author              = delta
Experiment          = hmqc.jxp
Sample_Id           = HT-1382-PTLC 1
Solvent             = CHLOROFORM-D
Creation_Time       = 27-APR-2023 20:56:51
Revision_Time       = 11-APR-2024 11:31:24
Current_Time        = 11-APR-2024 11:32:08

Comment             = gradient enhanced HMQC
Data_Format         = 2D REAL REAL
Dim_Size            = 819, 512
Dim_Title           = Proton Carbon13
Dim_Units           = [ppm] [ppm]
Dimensions          = X Y
Site                = JNM-ECA500II
Spectrometer        = DELTA2_NMR

Field_Strength      = 11.7473579[T] (500[MHz]
X_Acq_Duration      = 0.11927552[s]
X_Domain            = 1H
X_Freq              = 500.15991521[MHz]
X_Offset            = 5.25480443[ppm]
X_Points            = 1024
X_Prescans          = 4
X_Resolution        = 8.38395003[Hz]
X_Sweep             = 8.58516484[kHz]
X_Sweep_Clipped     = 6.86813187[kHz]
Y_Domain            = 13C
Y_Freq              = 125.76529768[MHz]
Y_Offset            = 112.01829002[ppm]
Y_Points            = 256
Y_Prescans          = 0
Y_Resolution        = 101.51377339[Hz]
Y_Sweep             = 25.98752599[kHz]
Tri_Domain          = Proton
Tri_Freq            = 500.15991521[MHz]
Tri_Offset          = 5.0[ppm]
Clipped             = FALSE
Scans               = 8
Total_Scans         = 2048

Relaxation_Delay    = 1.5[s]
Recvr_Gain          = 50
Temp_Get            = 24.1[dC]
X_Acq_Time          = 0.11927552[s]
X_Atn               = 3.2[dB]
X_Gamma             = 42576375
X_Pulse             = 7.68[us]
```

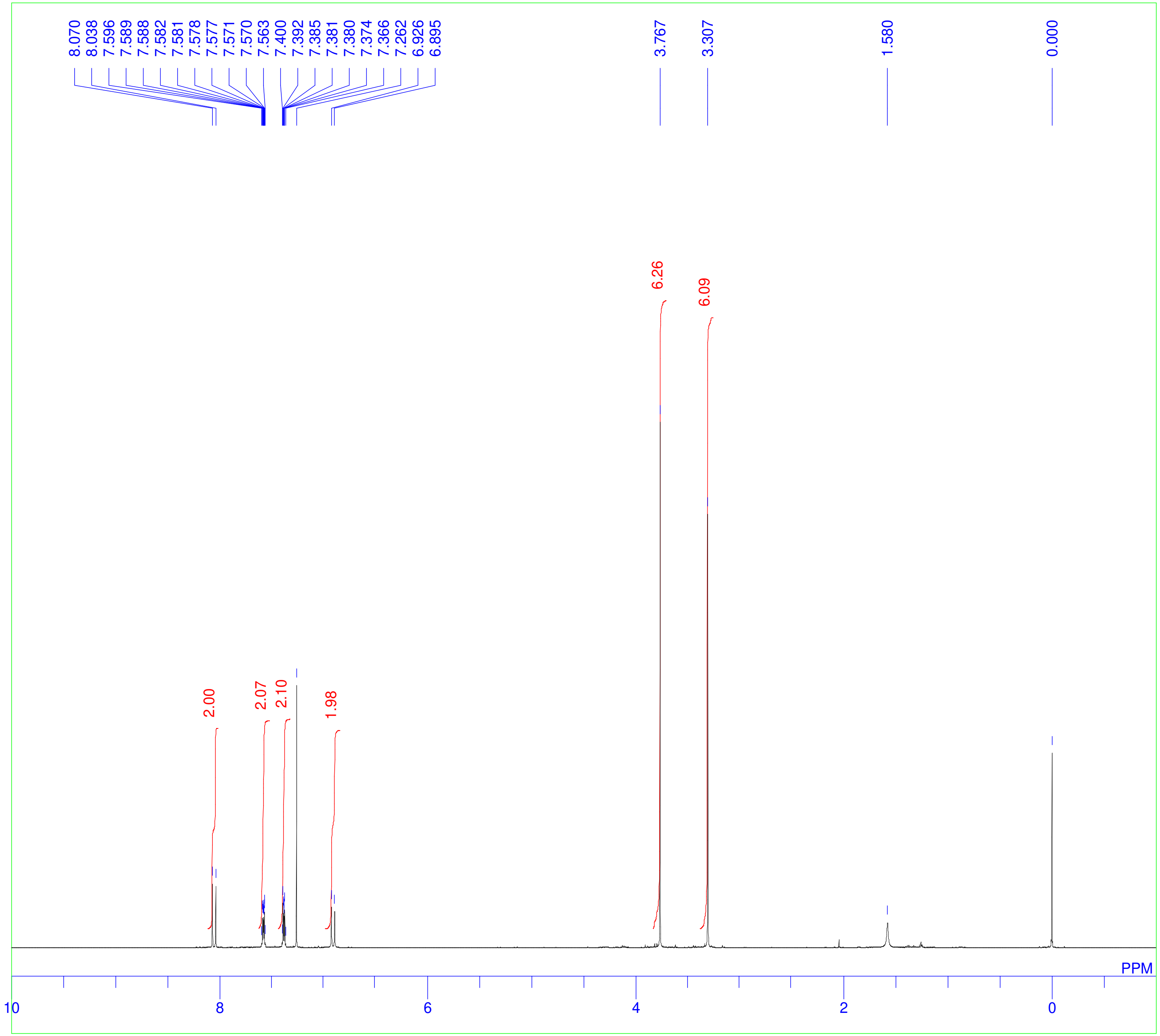

DFILE	(E,E)-20ac_1H.als
COMNT	
DATIM	2023-04-26 19:02:57
OBNUC	1H
EXMOD	proton.jxp
OBFRQ	500.16 MHz
OBSET	2.41 KHz
OBFIN	6.01 Hz
POINT	13107
FREQU	7507.51 Hz
SCANS	8
ACQTM	1.7459 sec
PD	5.0000 sec
PW1	3.84 usec
IRNUC	1H
CTEMP	23.6 c
SLVNT	CDCL3
EXREF	0.00 ppm
BF	0.30 Hz
RGAIN	48

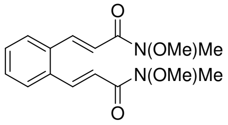

(*E*,*E*)-20ac

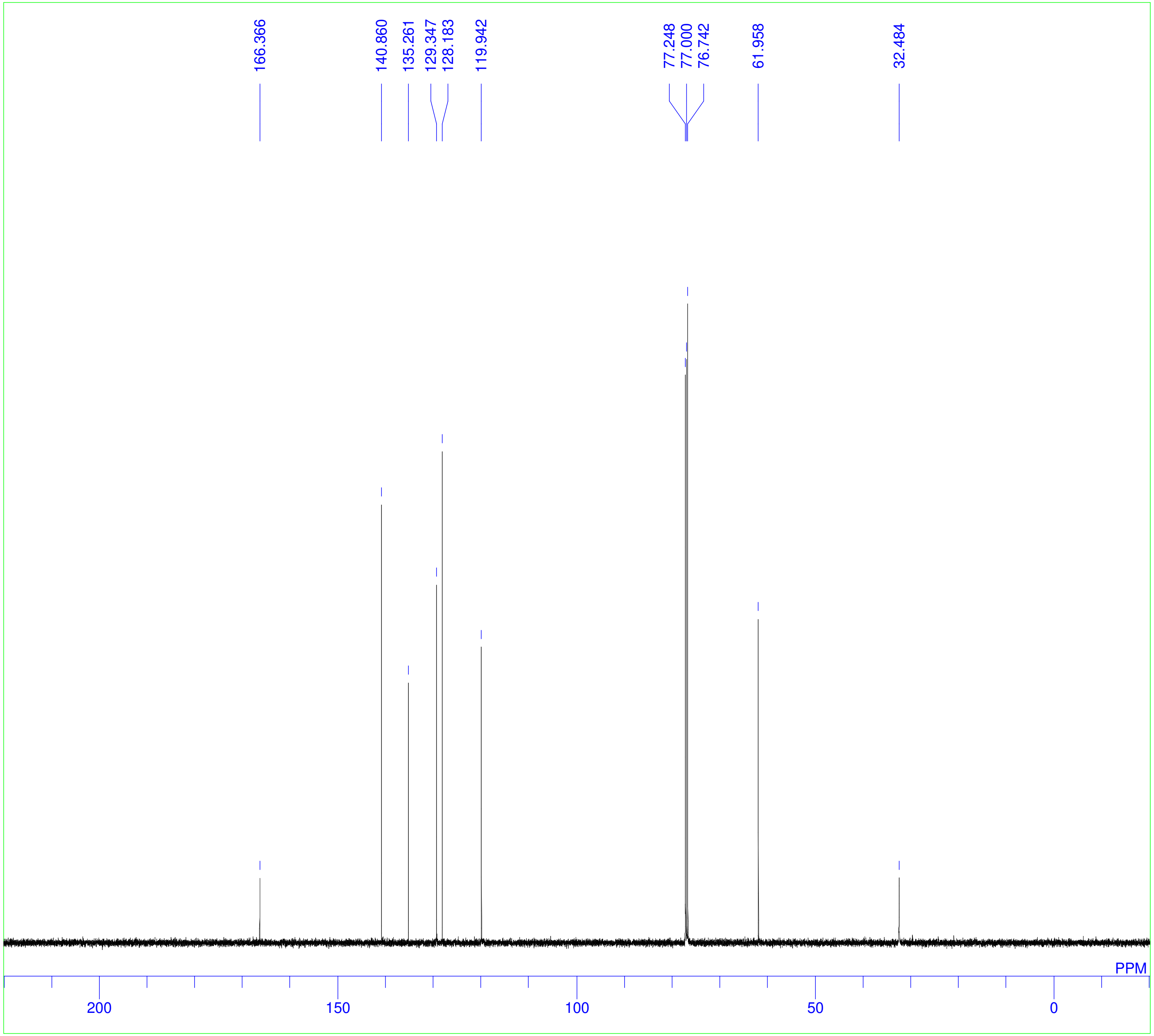

DFILE	(E,E)-20ac_13C.als
COMNT	
DATIM	2023-04-27 07:55:55
OBNUC	13C
EXMOD	carbon.jxp
OBFRQ	125.77 MHz
OBSET	7.87 KHz
OBFIN	4.21 Hz
POINT	26214
FREQU	31446.54 Hz
SCANS	1024
ACQTM	0.8336 sec
PD	2.0000 sec
PW1	3.87 usec
IRNUC	1H
CTEMP	23.8 c
SLVNT	CDCL3
EXREF	77.00 ppm
BF	0.30 Hz
RGAIN	30

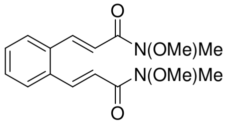

(*E*,*E*)-20ac

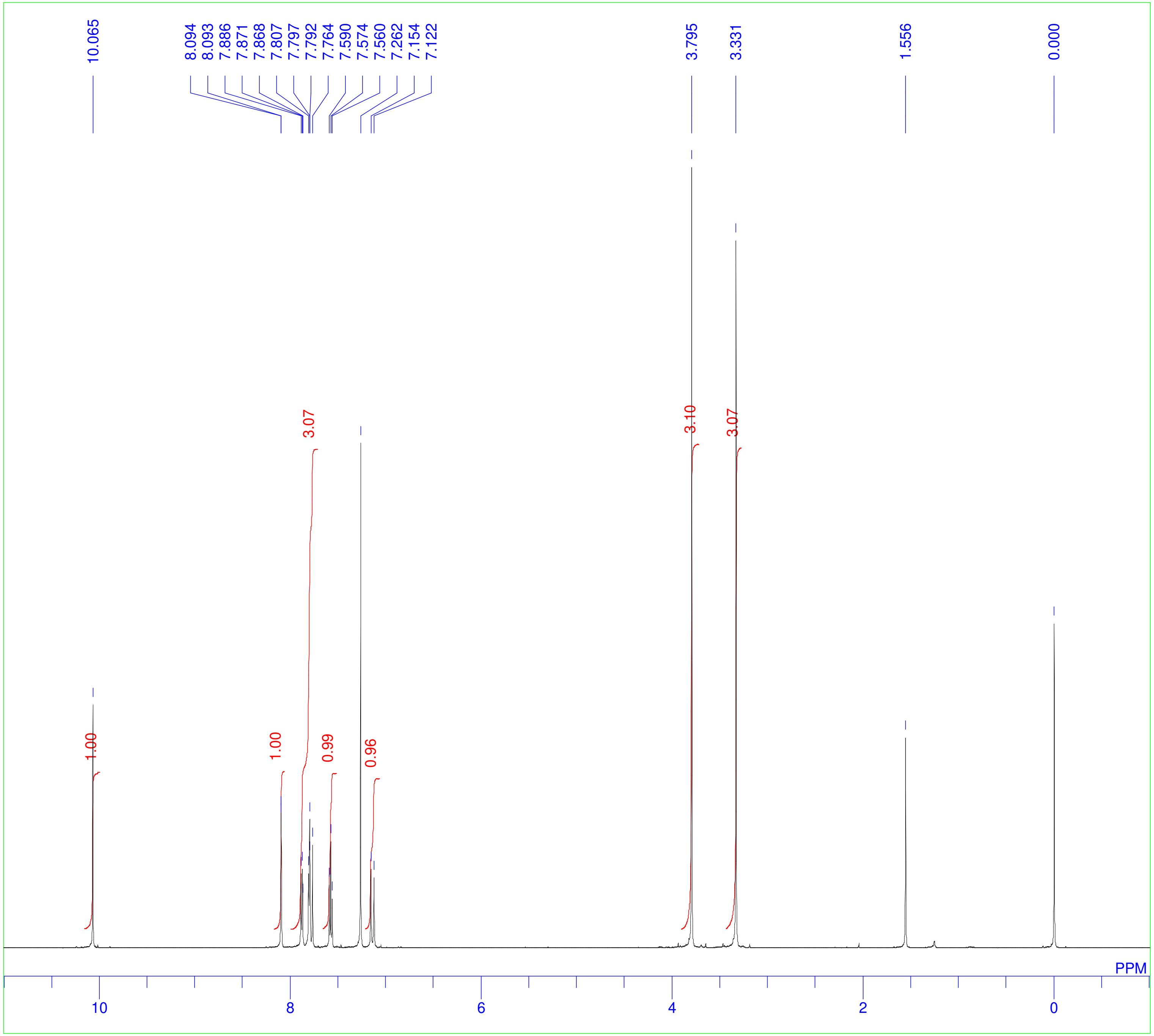

DFILE (E)-20ad_1H.als
COMNT
DATIM 2023-04-27 13:27:04
OBNUC 1H
EXMOD proton.jxp
OBFRQ 500.16 MHz
OBSET 2.41 KHz
OBFIN 6.01 Hz
POINT 13107
FREQU 7507.51 Hz
SCANS 8
ACQTM 1.7459 sec
PD 5.0000 sec
PW1 3.84 usec
IRNUC 1H
CTEMP 23.8 c
SLVNT CDCL3
EXREF 0.00 ppm
BF 1.00 Hz
RGAIN 50

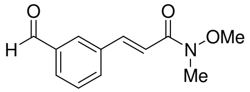

(*E*)-20ad

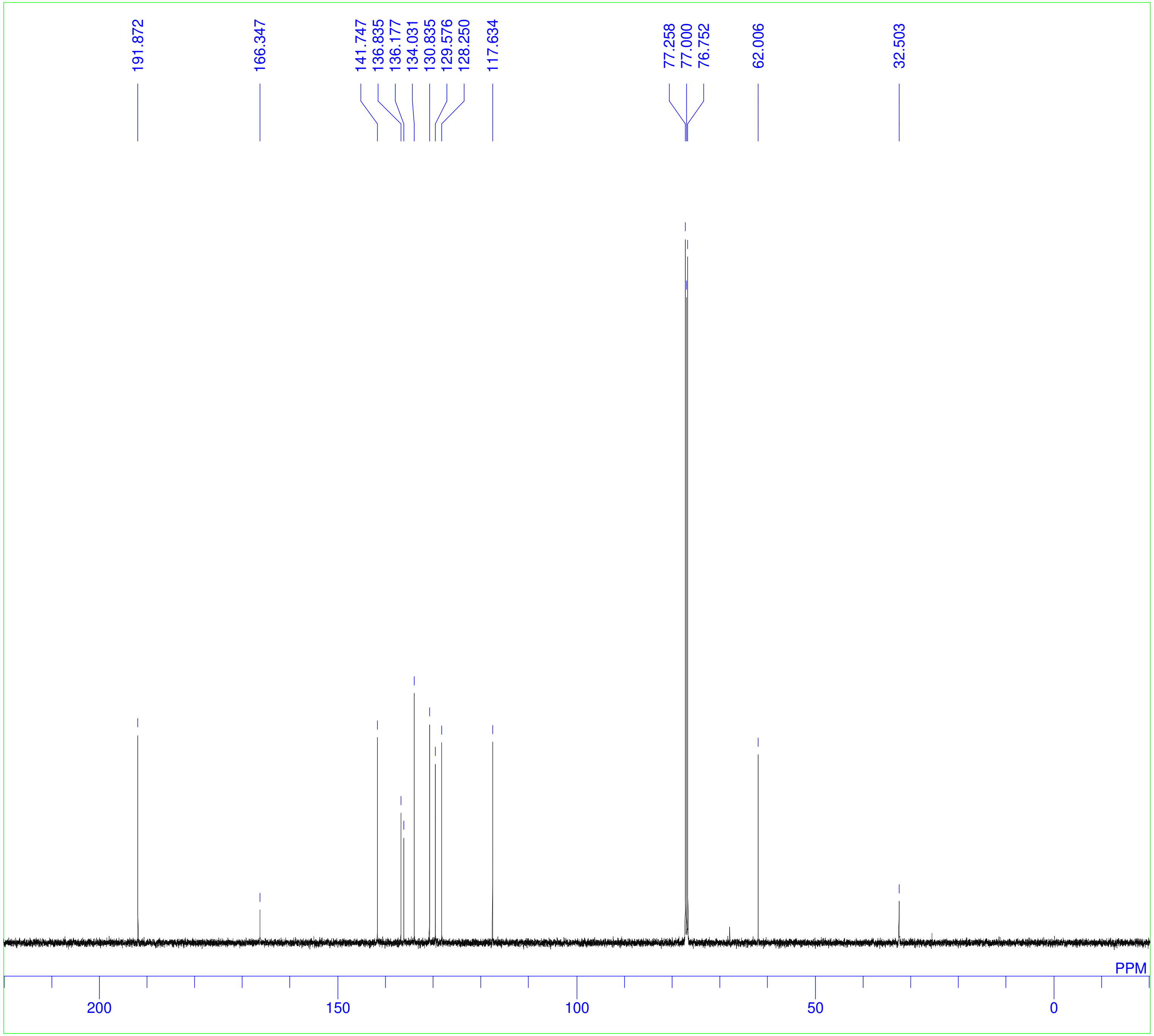

DFILE (E)-20ad_13C.als
COMNT
DATIM 2023-04-26 21:04:33
OBNUC 13C
EXMOD carbon.jxp
OBFRQ 125.77 MHz
OBSET 7.87 KHz
OBFIN 4.21 Hz
POINT 26214
FREQU 31446.54 Hz
SCANS 1024
ACQTM 0.8336 sec
PD 2.0000 sec
PW1 3.87 usec
IRNUC 1H
CTEMP 23.8 c
SLVNT CDCL3
EXREF 77.00 ppm
BF 1.00 Hz
RGAIN 32

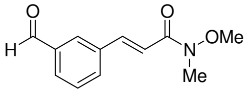

(*E*)-20ad

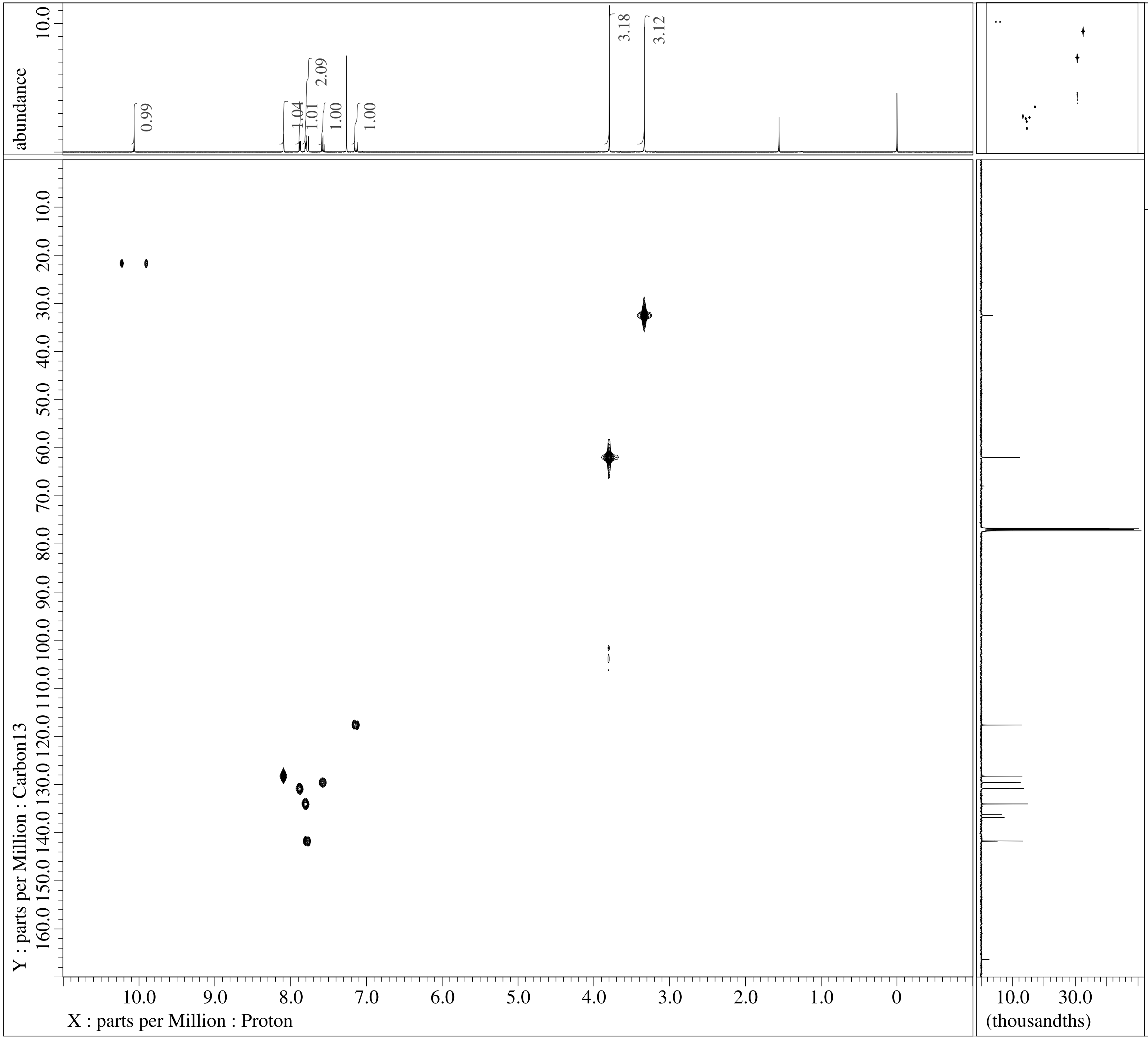


```
---- PROCESSING PARAMETERS ----
sinbell4( -60, 160 )
zerofill( 1 )
fft( 1, TRUE, TRUE )
ppm
[transpose]
sinbell4( -60, 160 )
zerofill( 2 )
fft( 1, TRUE, TRUE )
ppm
abs
```

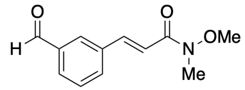

(*E*)-20ad

```
Filename             = HT-1383-PTLC 3_HMQC,HMB
Author               = delta
Experiment           = hmqc.jxp
Sample_Id            = HT-1383-PTLC 3_HMQC,HMB
Solvent              = CHLOROFORM-D
Creation_Time        = 30-MAY-2023 20:00:28
Revision_Time        = 11-APR-2024 11:34:50
Current_Time         = 11-APR-2024 11:35:23

Comment              = gradient enhanced HMQC
Data_Format          = 2D REAL REAL
Dim_Size             = 819, 512
Dim_Title            = Proton Carbon13
Dim_Units            = [ppm] [ppm]
Dimensions           = X Y
Site                 = JNM-ECA500II
Spectrometer         = DELTA2_NMR

Field_Strength       = 11.7473579[T] (500[MHz]
X_Acq_Duration       = 0.12304384[s]
X_Domain             = 1H
X_Freq               = 500.15991521[MHz]
X_Offset             = 5.09562324[ppm]
X_Points             = 1024
X_Prescans           = 4
X_Resolution         = 8.12718459[Hz]
X_Sweep              = 8.32223702[kHz]
X_Sweep_Clipped      = 6.65778961[kHz]
Y_Domain             = 13C
Y_Freq               = 125.76529768[MHz]
Y_Offset             = 85[ppm]
Y_Points             = 256
Y_Prescans           = 0
Y_Resolution         = 83.60980308[Hz]
Y_Sweep              = 21.40410959[kHz]
Tri_Domain           = Proton
Tri_Freq             = 500.15991521[MHz]
Tri_Offset           = 5.0[ppm]
Clipped              = FALSE
Scans                = 8
Total_Scans          = 2048

Relaxation_Delay     = 1.5[s]
Recvr_Gain           = 50
Temp_Get             = 23.7[dC]
X_Acq_Time           = 0.12304384[s]
X_Atn                = 3.2[dB]
X_Gamma              = 42576375
X_Pulse              = 7.68[us]
```

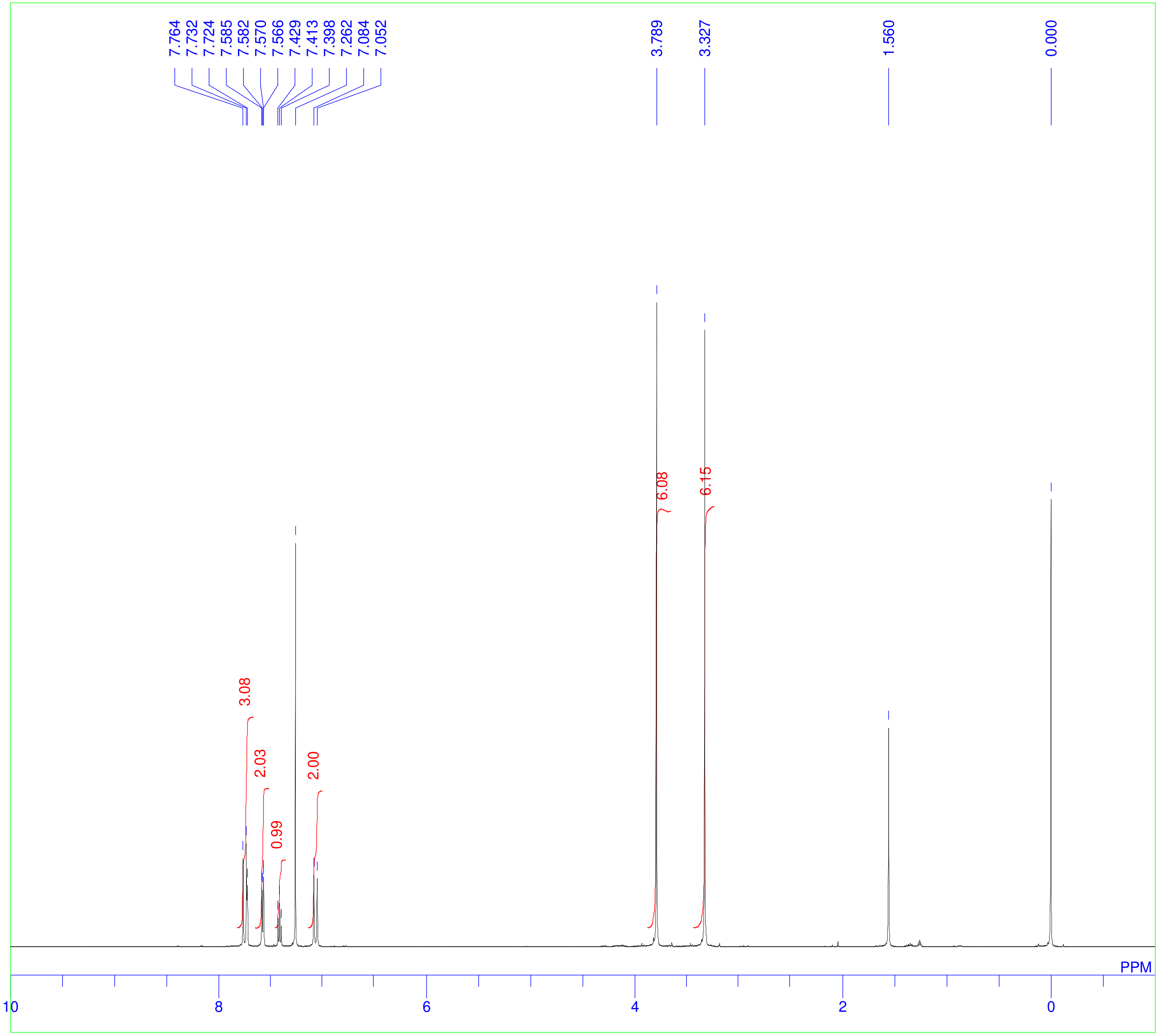

DFILE	(E,E)-20ad_1H.als
COMNT	
DATIM	2023-04-28 19:41:04
OBNUC	1H
EXMOD	proton.jxp
OBFRQ	500.16 MHz
OBSET	2.41 KHz
OBFIN	6.01 Hz
POINT	13107
FREQU	7507.51 Hz
SCANS	8
ACQTM	1.7459 sec
PD	5.0000 sec
PW1	3.84 usec
IRNUC	1H
CTEMP	23.7 c
SLVNT	CDCL3
EXREF	0.00 ppm
BF	1.00 Hz
RGAIN	48

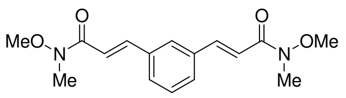

(*E*,*E*)-20ad

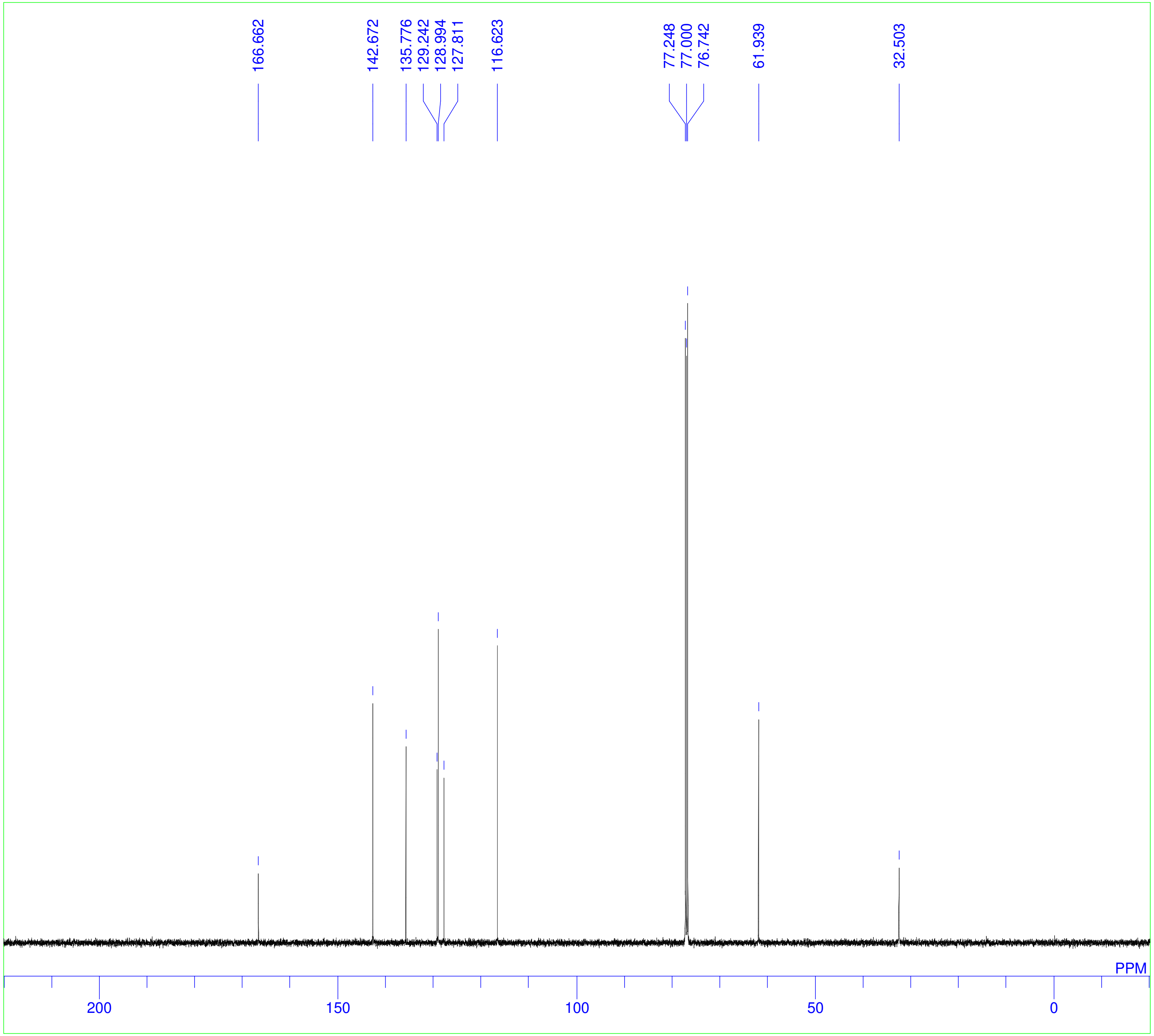

DFILE (E,E)-20ad_13C.als
COMNT
DATIM 2023-04-27 07:01:46
OBNUC 13C
EXMOD carbon.jxp
OBFRQ 125.77 MHz
OBSET 7.87 KHz
OBFIN 4.21 Hz
POINT 26214
FREQU 31446.54 Hz
SCANS 1024
ACQTM 0.8336 sec
PD 2.0000 sec
PW1 3.87 usec
IRNUC 1H
CTEMP 24.0 c
SLVNT CDCL3
EXREF 77.00 ppm
BF 1.00 Hz
RGAIN 28

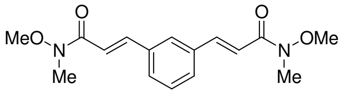

(*E*,*E*)-20ad

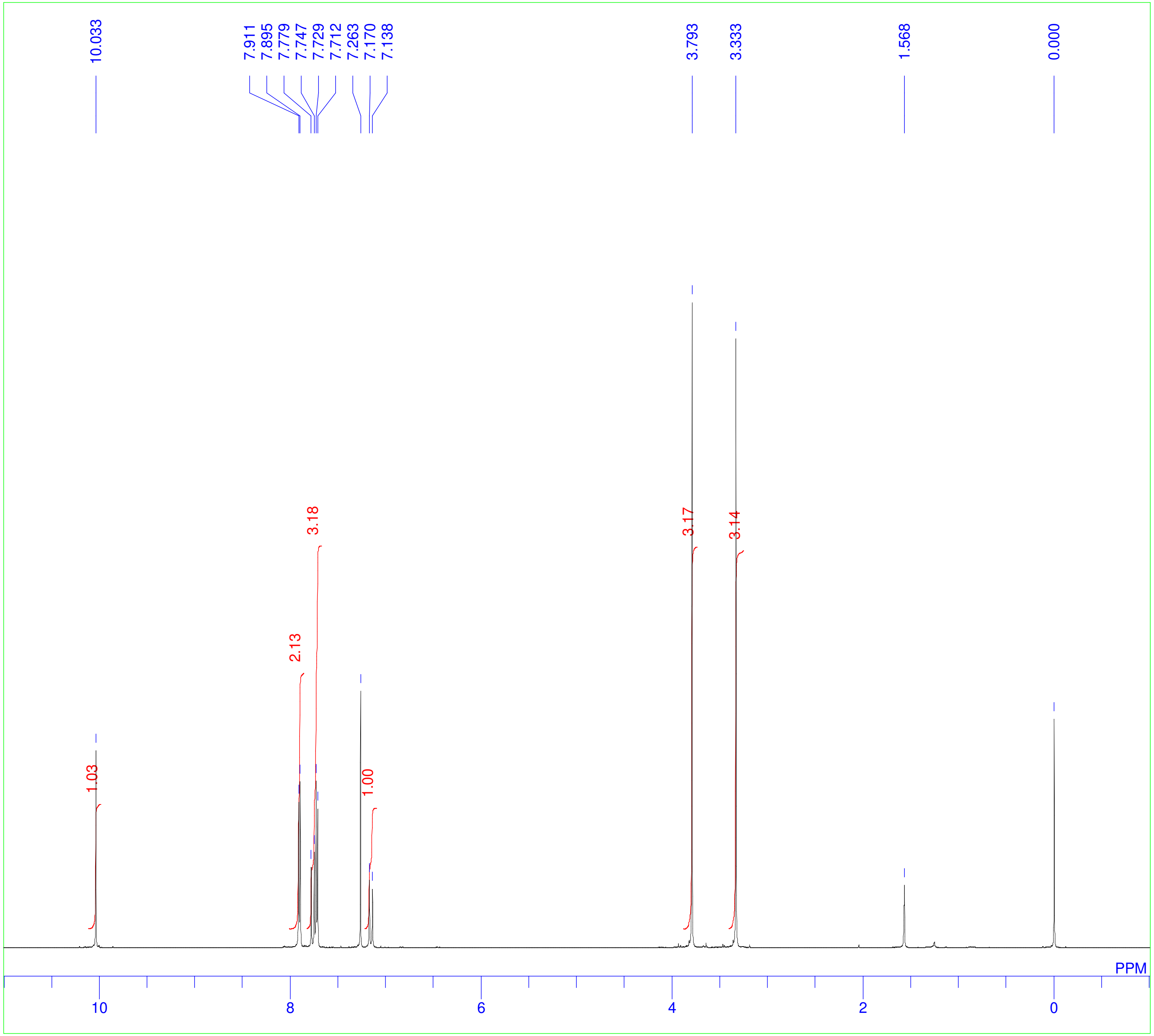

DFILE (E)-20ae_1H.als
COMNT
DATIM 2023-04-26 19:35:20
OBNUC 1H
EXMOD proton.jxp
OBFRQ 500.16 MHz
OBSET 2.41 KHz
OBFIN 6.01 Hz
POINT 13107
FREQU 7507.51 Hz
SCANS 8
ACQTM 1.7459 sec
PD 5.0000 sec
PW1 3.84 usec
IRNUC 1H
CTEMP 23.7 c
SLVNT CDCL3
EXREF 0.00 ppm
BF 1.00 Hz
RGAIN 48

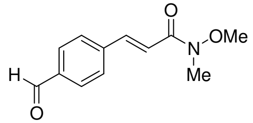

(*E*)-20ae

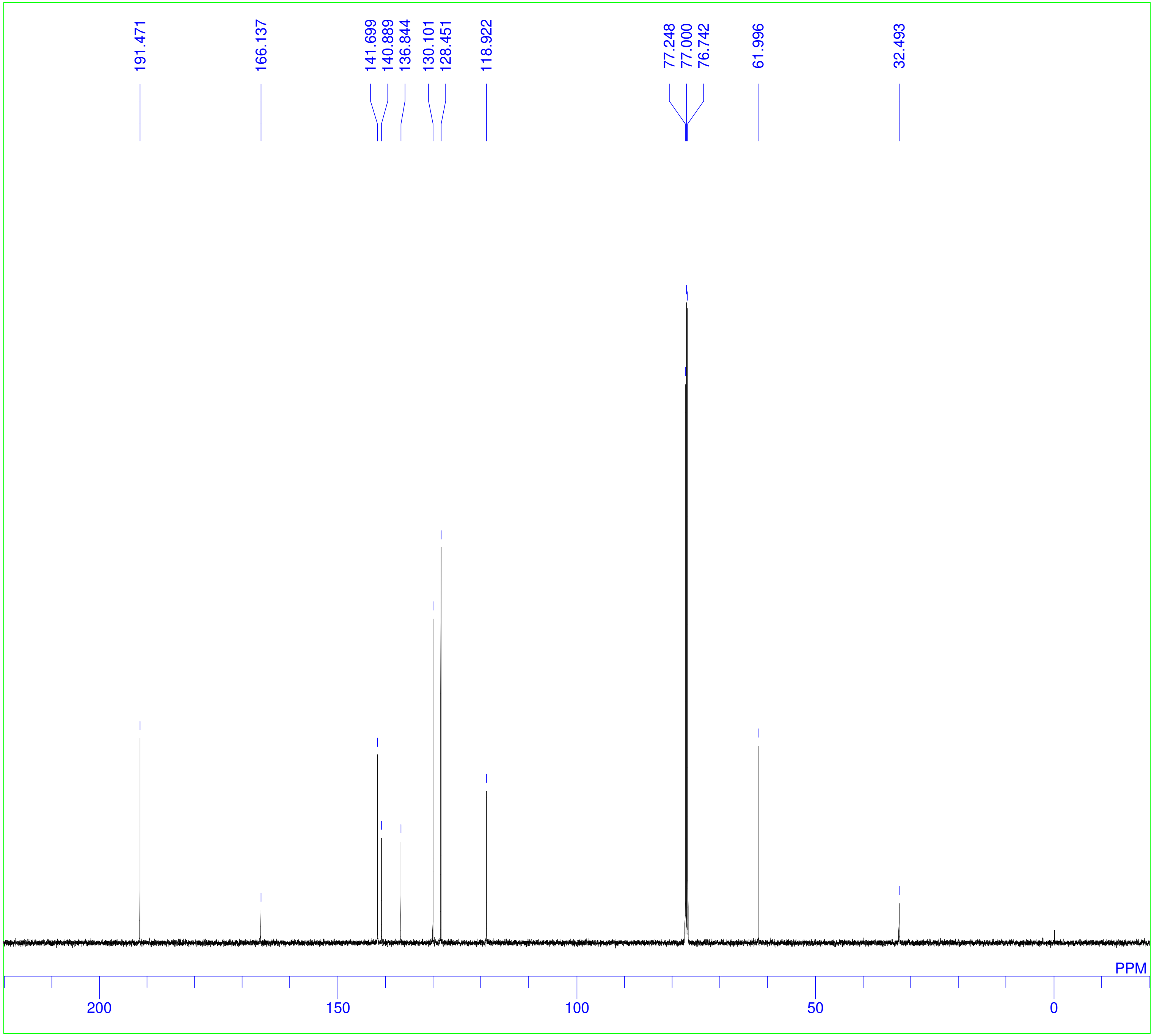

DFILE	(E)-20ae_13C.als
COMNT	
DATIM	2023-04-29 11:26:12
OBNUC	13C
EXMOD	carbon.jxp
OBFRQ	125.77 MHz
OBSET	7.87 KHz
OBFIN	4.21 Hz
POINT	26214
FREQU	31446.54 Hz
SCANS	1024
ACQTM	0.8336 sec
PD	2.0000 sec
PW1	3.87 usec
IRNUC	1H
CTEMP	24.3 c
SLVNT	CDCL3
EXREF	77.00 ppm
BF	1.00 Hz
RGAIN	34

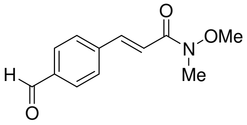

(*E*)-20ae

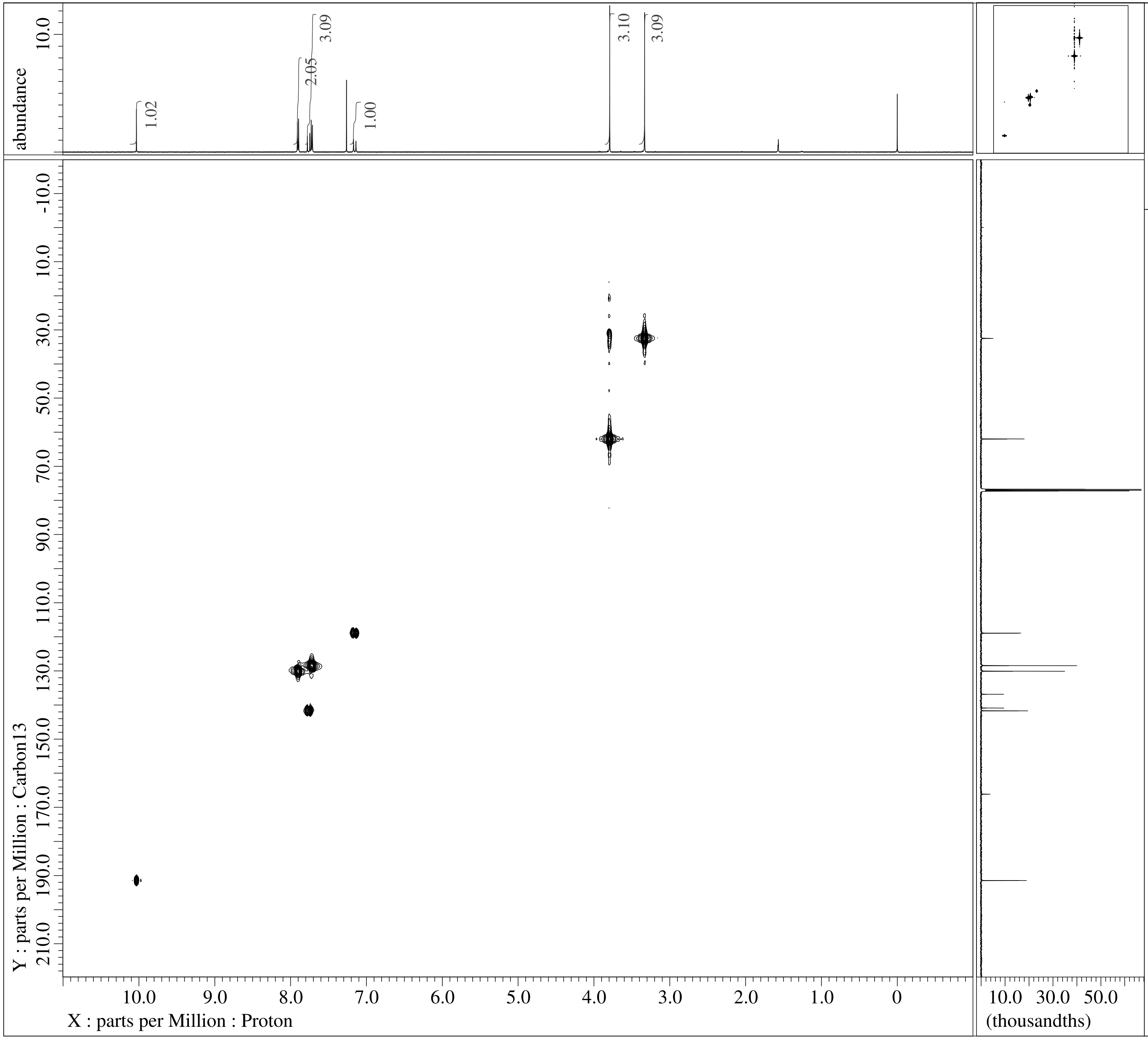


```
---- PROCESSING PARAMETERS ----
sinbell4( -60, 160 )
zerofill( 1 )
fft( 1, TRUE, TRUE )
ppm
[transpose]
sinbell4( -60, 160 )
zerofill( 2 )
fft( 1, TRUE, TRUE )
ppm
abs
```

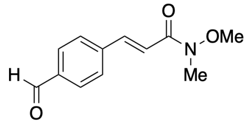

(*E*)-20ae

```
Filename            = HT-1380-PTLC 2_13C_HMQC
Author              = delta
Experiment          = hmqc.jxp
Sample_Id           = HT-1380-PTLC 2_13C
Solvent             = CHLOROFORM-D
Creation_Time       = 29-APR-2023 12:15:02
Revision_Time       = 11-APR-2024 11:55:50
Current_Time        = 11-APR-2024 11:56:52

Comment             = gradient enhanced HMQC
Data_Format         = 2D REAL REAL
Dim_Size            = 819, 512
Dim_Title           = Proton Carbon13
Dim_Units           = [ppm] [ppm]
Dimensions          = X Y
Site                = JNM-ECA500II
Spectrometer        = DELTA2_NMR

Field_Strength      = 11.7473579[T] (500[MHz]
X_Acq_Duration      = 0.10911744[s]
X_Domain            = 1H
X_Freq              = 500.15991521[MHz]
X_Offset            = 5.0[ppm]
X_Points            = 1024
X_Prescans          = 4
X_Resolution        = 9.16443788[Hz]
X_Sweep             = 9.38438438[kHz]
X_Sweep_Clipped     = 7.50750751[kHz]
Y_Domain            = 13C
Y_Freq              = 125.76529768[MHz]
Y_Offset            = 97.65357195[ppm]
Y_Points            = 256
Y_Prescans          = 0
Y_Resolution        = 120.5632716[Hz]
Y_Sweep             = 30.86419753[kHz]
Tri_Domain          = Proton
Tri_Freq            = 500.15991521[MHz]
Tri_Offset          = 5.0[ppm]
Clipped             = FALSE
Scans               = 8
Total_Scans         = 2048

Relaxation_Delay    = 1.5[s]
Recvr_Gain          = 50
Temp_Get            = 24.2[dC]
X_Acq_Time          = 0.10911744[s]
X_Atn               = 3.2[dB]
X_Gamma             = 42576375
X_Pulse             = 7.68[us]
```

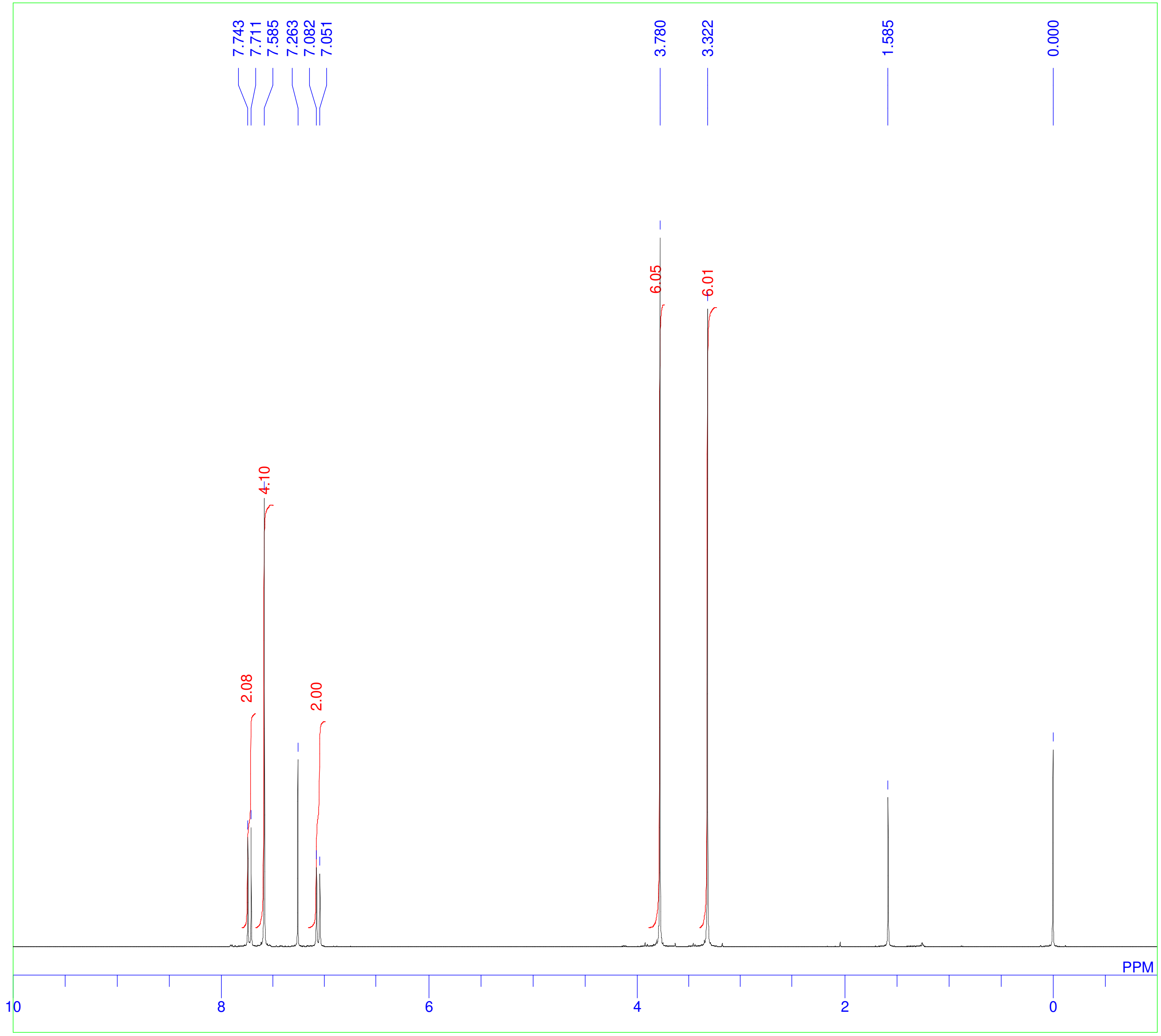

DFILE	(E,E)-20ae_1H.als
COMNT	
DATIM	2023-05-17 21:17:29
OBNUC	1H
EXMOD	proton.jxp
OBFRQ	500.16 MHz
OBSET	2.41 KHz
OBFIN	6.01 Hz
POINT	13107
FREQU	7507.51 Hz
SCANS	8
ACQTM	1.7459 sec
PD	5.0000 sec
PW1	3.84 usec
IRNUC	1H
CTEMP	23.9 c
SLVNT	CDCL3
EXREF	0.00 ppm
BF	1.00 Hz
RGAIN	44

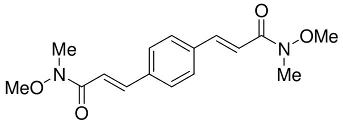

(*E*,*E*)-20ae

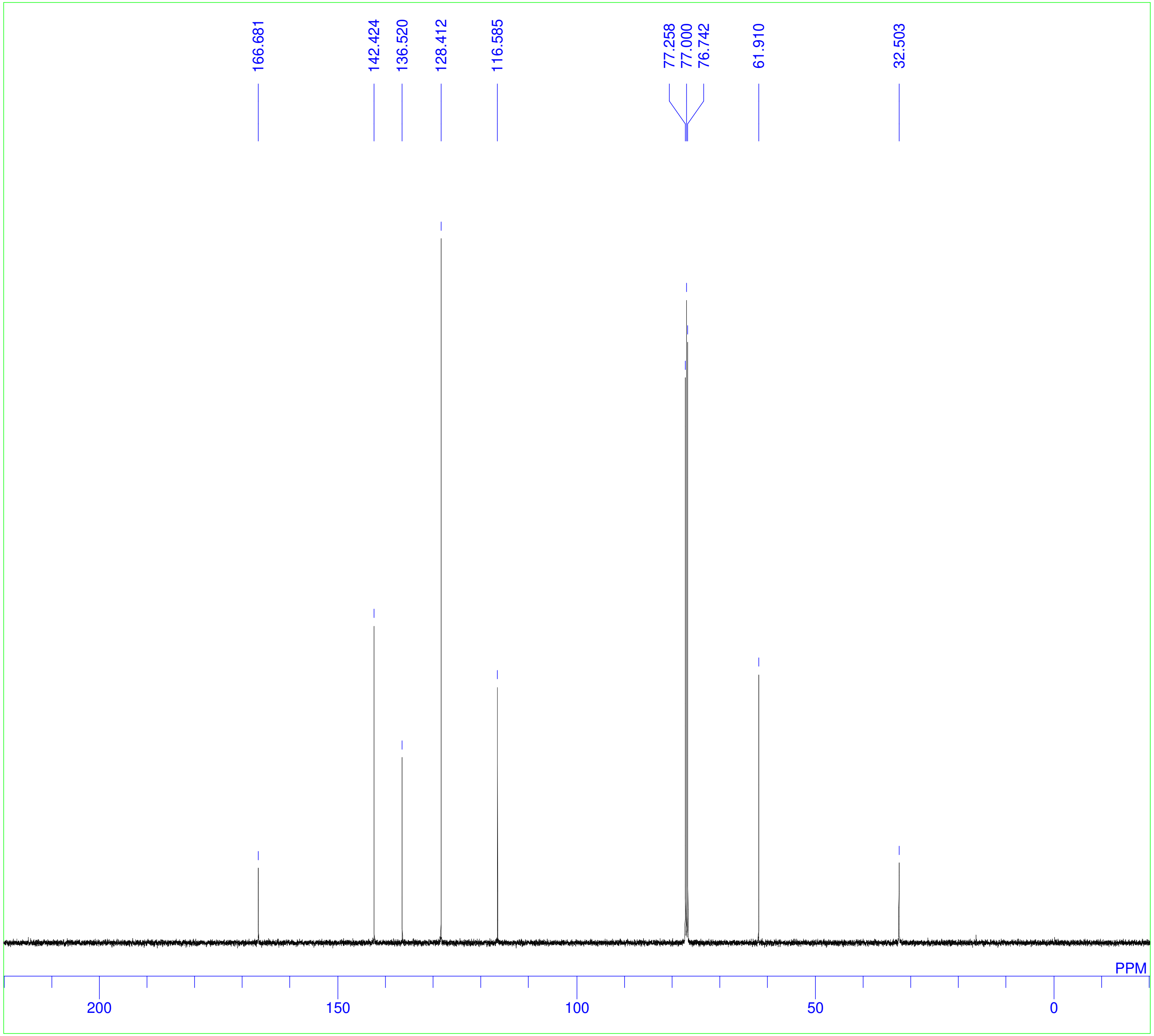

DFILE	(E,E)-20ae_13C.als
COMNT	
DATIM	2023-05-06 15:41:52
OBNUC	13C
EXMOD	carbon.jxp
OBFRQ	125.77 MHz
OBSET	7.87 KHz
OBFIN	4.21 Hz
POINT	26214
FREQU	31446.54 Hz
SCANS	1024
ACQTM	0.8336 sec
PD	2.0000 sec
PW1	3.87 usec
IRNUC	1H
CTEMP	24.1 c
SLVNT	CDCL3
EXREF	77.00 ppm
BF	1.00 Hz
RGAIN	30

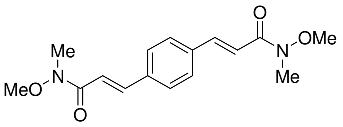

(*E*,*E*)-20ae

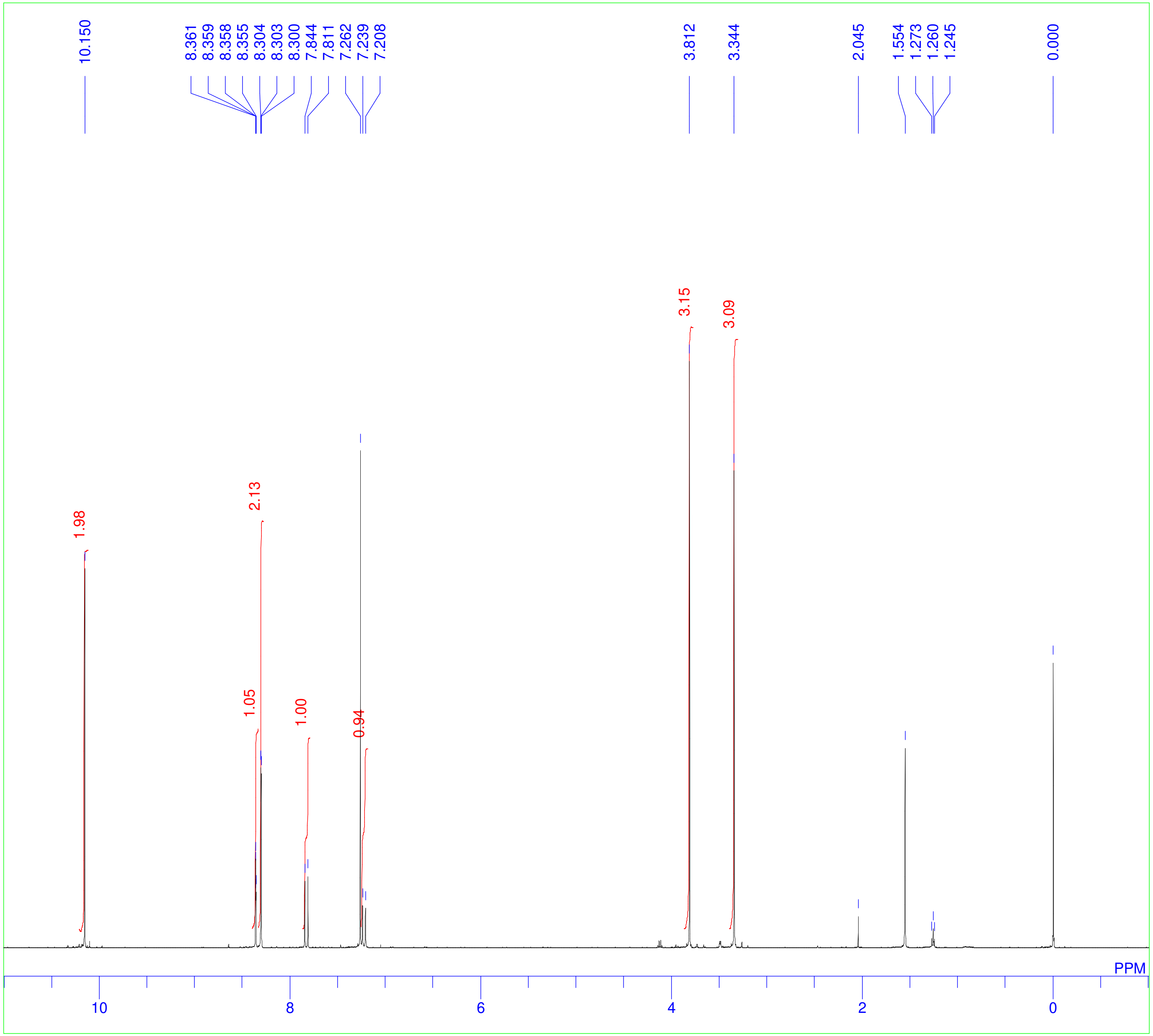

DFILE (E)-20af_1H.als
COMNT
DATIM 2023-05-31 13:31:16
OBNUC 1H
EXMOD proton.jxp
OBFRQ 500.16 MHz
OBSET 2.41 KHz
OBFIN 6.01 Hz
POINT 13107
FREQU 7507.51 Hz
SCANS 8
ACQTM 1.7459 sec
PD 5.0000 sec
PW1 3.84 usec
IRNUC 1H
CTEMP 24.2 c
SLVNT CDCL3
EXREF 0.00 ppm
BF 0.30 Hz
RGAIN 50

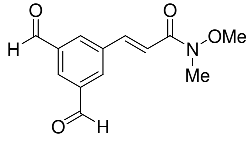

(*E*)-20af

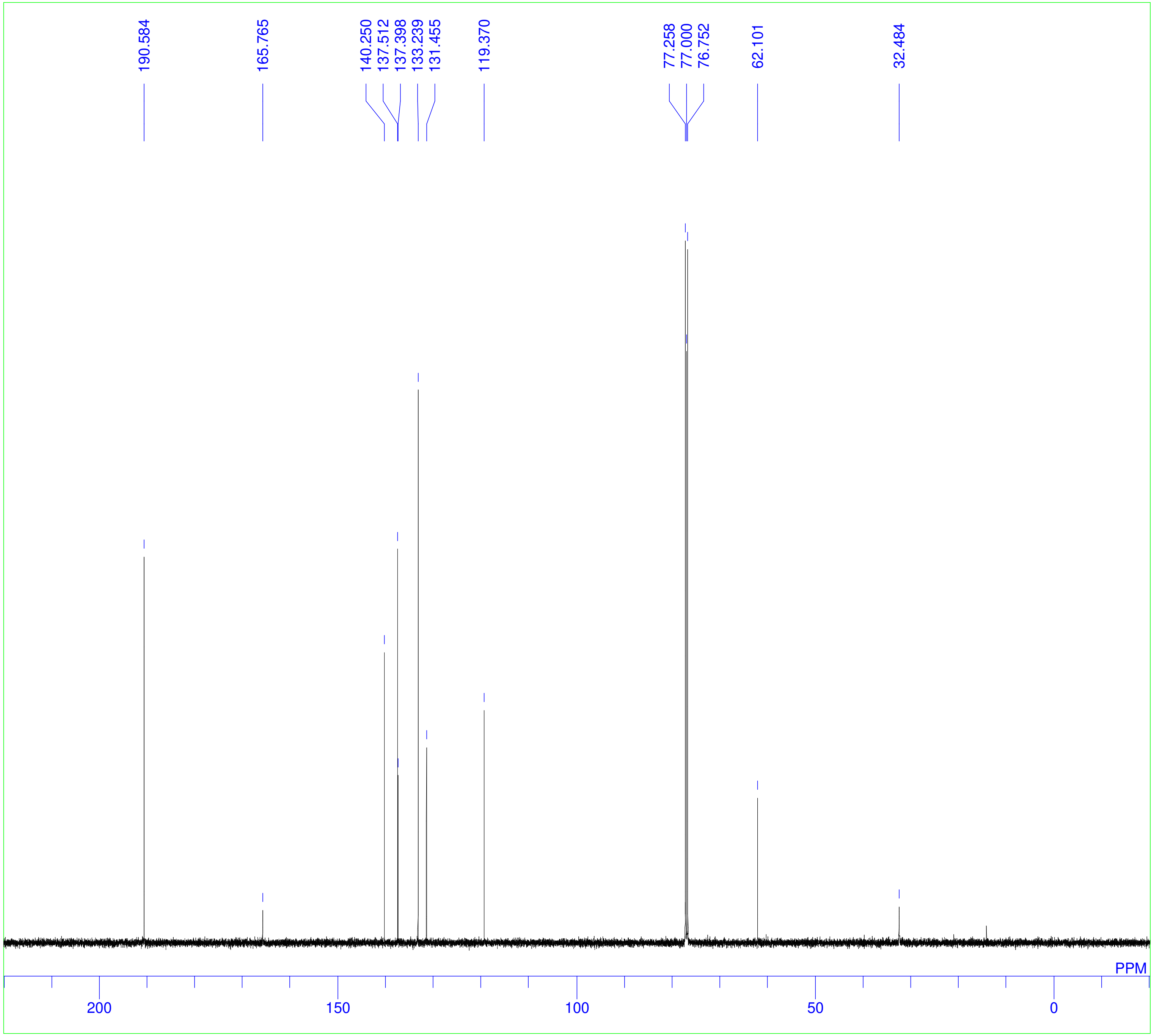

DFILE	(E)-20af_13C.als
COMNT	
DATIM	2023-06-05 14:31:45
OBNUC	13C
EXMOD	carbon.jxp
OBFRQ	125.77 MHz
OBSET	7.87 KHz
OBFIN	4.21 Hz
POINT	26214
FREQU	31446.54 Hz
SCANS	1024
ACQTM	0.8336 sec
PD	2.0000 sec
PW1	3.87 usec
IRNUC	1H
CTEMP	24.1 c
SLVNT	CDCL3
EXREF	77.00 ppm
BF	0.30 Hz
RGAIN	28

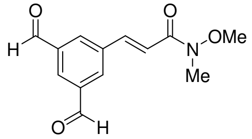

(*E*)-20af

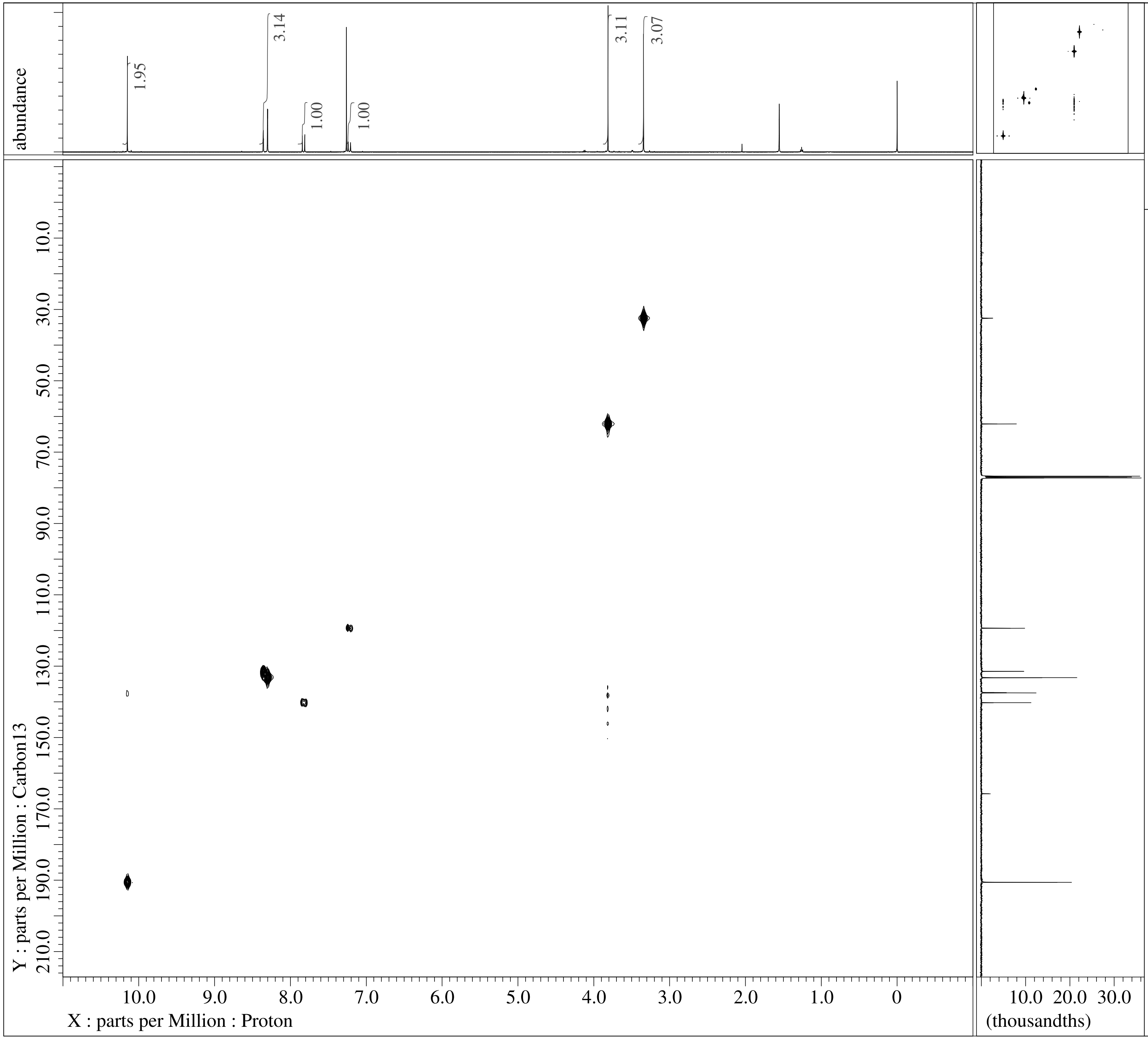


```
---- PROCESSING PARAMETERS ----
sinbell4( -60, 160 )
zerofill( 1 )
fft( 1, TRUE, TRUE )
ppm
[transpose]
sinbell4( -60, 160 )
zerofill( 2 )
fft( 1, TRUE, TRUE )
ppm
abs
```

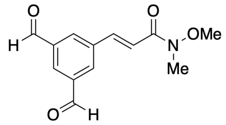

(*E*)-20af

```
Filename            = HT-1468-PTLC 2_13C_HMQC
Author              = delta
Experiment          = hmqc.jxp
Sample_Id           = HT-1468-PTLC 2_13C
Solvent             = CHLOROFORM-D
Creation_Time       =  5-JUN-2023 15:20:37
Revision_Time       = 11-APR-2024 11:59:37
Current_Time        = 11-APR-2024 12:00:16

Comment             = gradient enhanced HMQC
Data_Format         = 2D REAL REAL
Dim_Size            = 819, 512
Dim_Title           = Proton Carbon13
Dim_Units           = [ppm] [ppm]
Dimensions          = X Y
Site                = JNM-ECA500II
Spectrometer        = DELTA2_NMR

Field_Strength      = 11.7473579[T] (500[MHz]
X_Acq_Duration      = 0.10911744[s]
X_Domain            = 1H
X_Freq              = 500.15991521[MHz]
X_Offset            = 5.0[ppm]
X_Points            = 1024
X_Prescans          = 4
X_Resolution        = 9.16443788[Hz]
X_Sweep             = 9.38438438[kHz]
X_Sweep_Clipped     = 7.50750751[kHz]
Y_Domain            = 13C
Y_Freq              = 125.76529768[MHz]
Y_Offset            = 102.47996461[ppm]
Y_Points            = 256
Y_Prescans          = 0
Y_Resolution        = 112.76703233[Hz]
Y_Sweep             = 28.86836028[kHz]
Tri_Domain          = Proton
Tri_Freq            = 500.15991521[MHz]
Tri_Offset          = 5.0[ppm]
Clipped             = FALSE
Scans               = 6
Total_Scans         = 1536

Relaxation_Delay    = 1.5[s]
Recvr_Gain          = 50
Temp_Get            = 24.4[dC]
X_Acq_Time          = 0.10911744[s]
X_Atn               = 3.2[dB]
X_Gamma             = 42576375
X_Pulse             = 7.68[us]
```

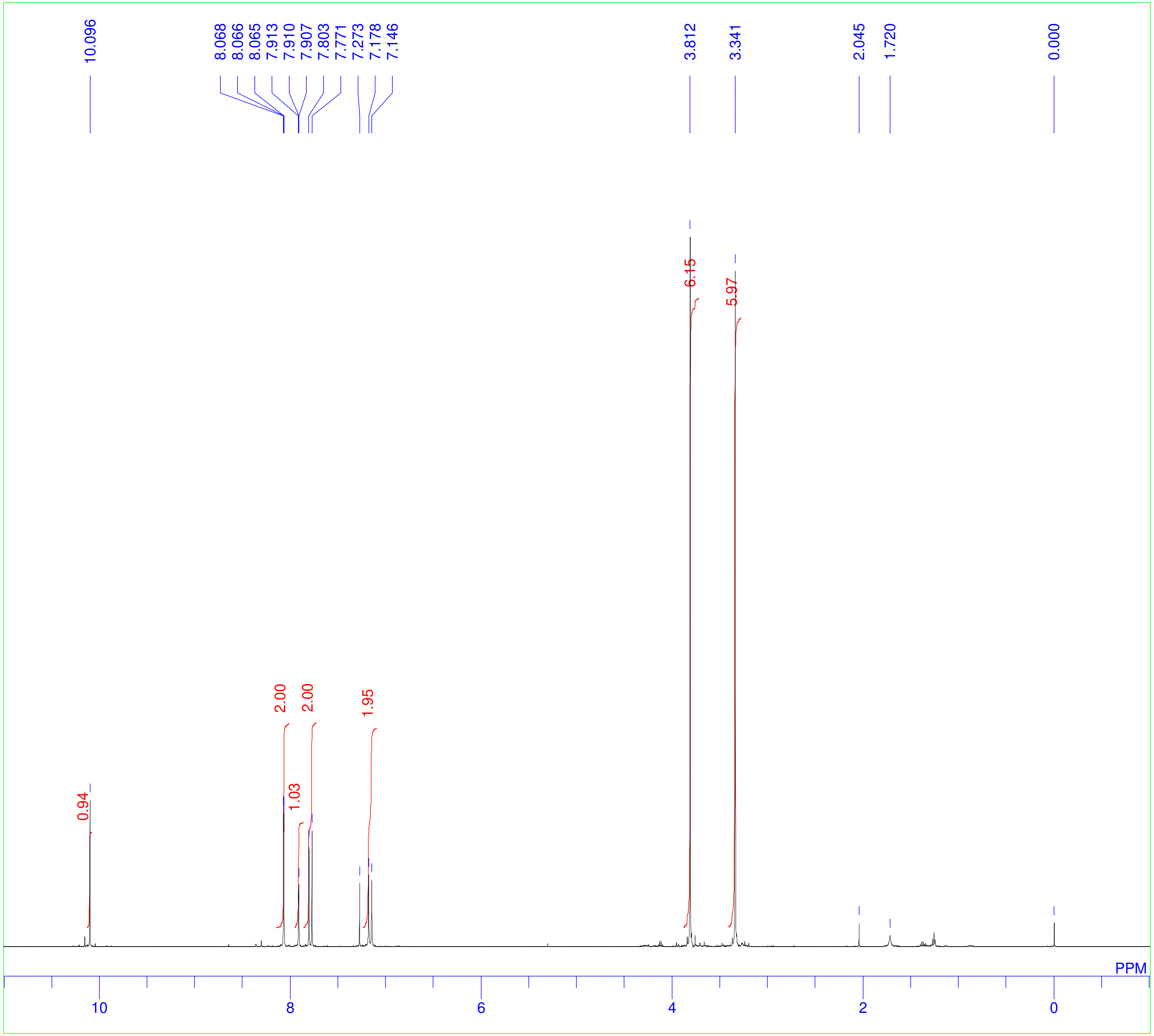

DFILE (E,E)-20af_1H.als
COMNT
DATIM 2023-06-05 21:46:31
OBNUC 1H
EXMOD proton.jxp
OBFRQ 500.16 MHz
OBSET 2.41 KHz
OBFIN 6.01 Hz
POINT 13107
FREQU 7507.51 Hz
SCANS 8
ACQTM 1.7459 sec
PD 5.0000 sec
PW1 3.84 usec
IRNUC 1H
CTEMP 24.1 c
SLVNT CDCL3
EXREF 0.00 ppm
BF 0.30 Hz
RGAIN 36

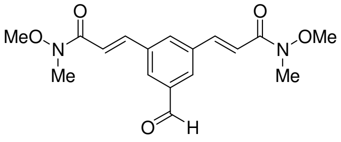

(*E*,*E*)-20af

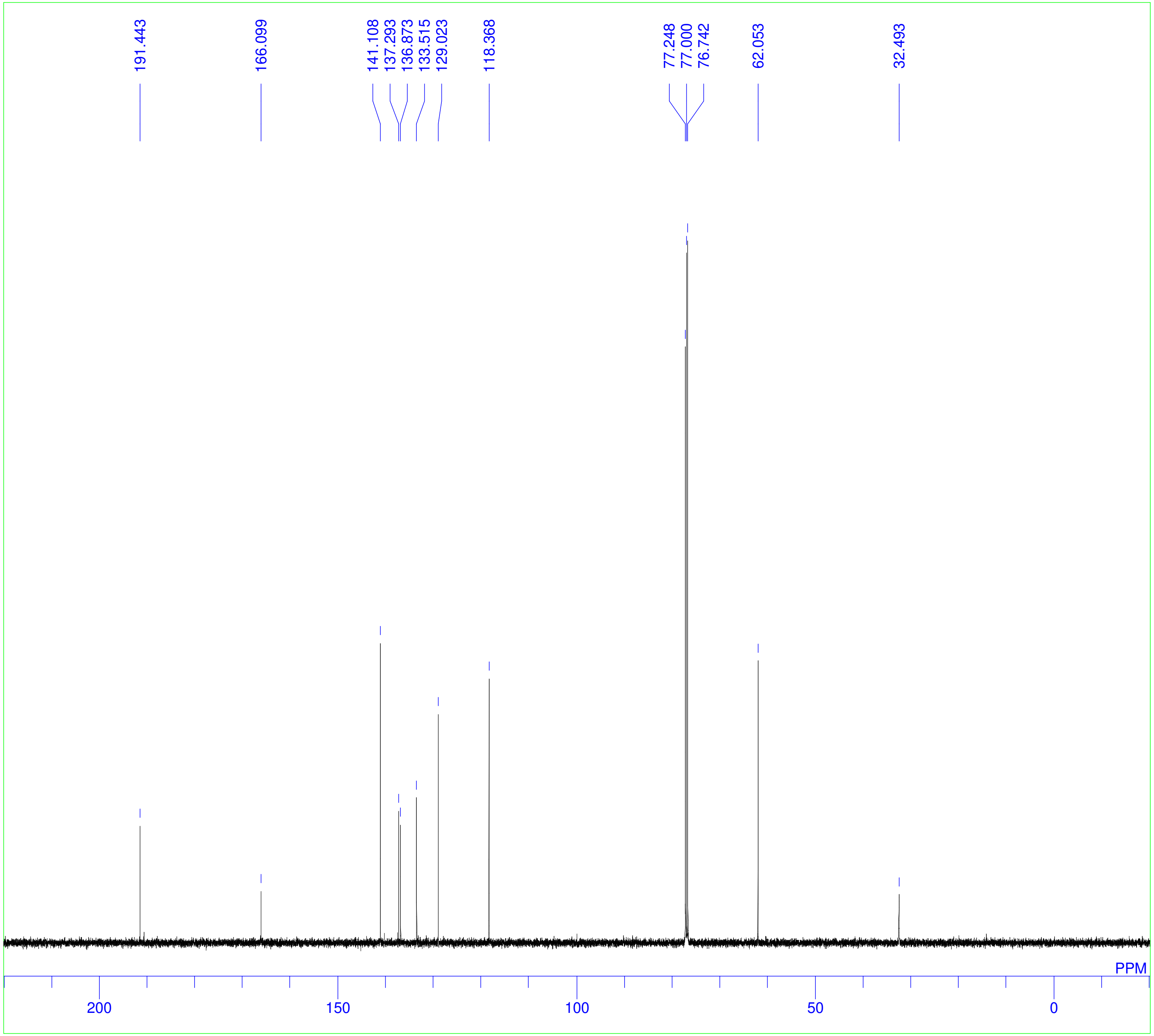

DFILE	(E,E)-20af_13C.als
COMNT	
DATIM	2023-06-05 20:01:01
OBNUC	13C
EXMOD	carbon.jxp
OBFRQ	125.77 MHz
OBSET	7.87 KHz
OBFIN	4.21 Hz
POINT	26214
FREQU	31446.54 Hz
SCANS	1024
ACQTM	0.8336 sec
PD	2.0000 sec
PW1	3.87 usec
IRNUC	1H
CTEMP	23.9 c
SLVNT	CDCL3
EXREF	77.00 ppm
BF	0.30 Hz
RGAIN	28

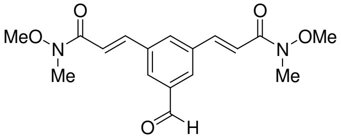

(*E*,*E*)-20af

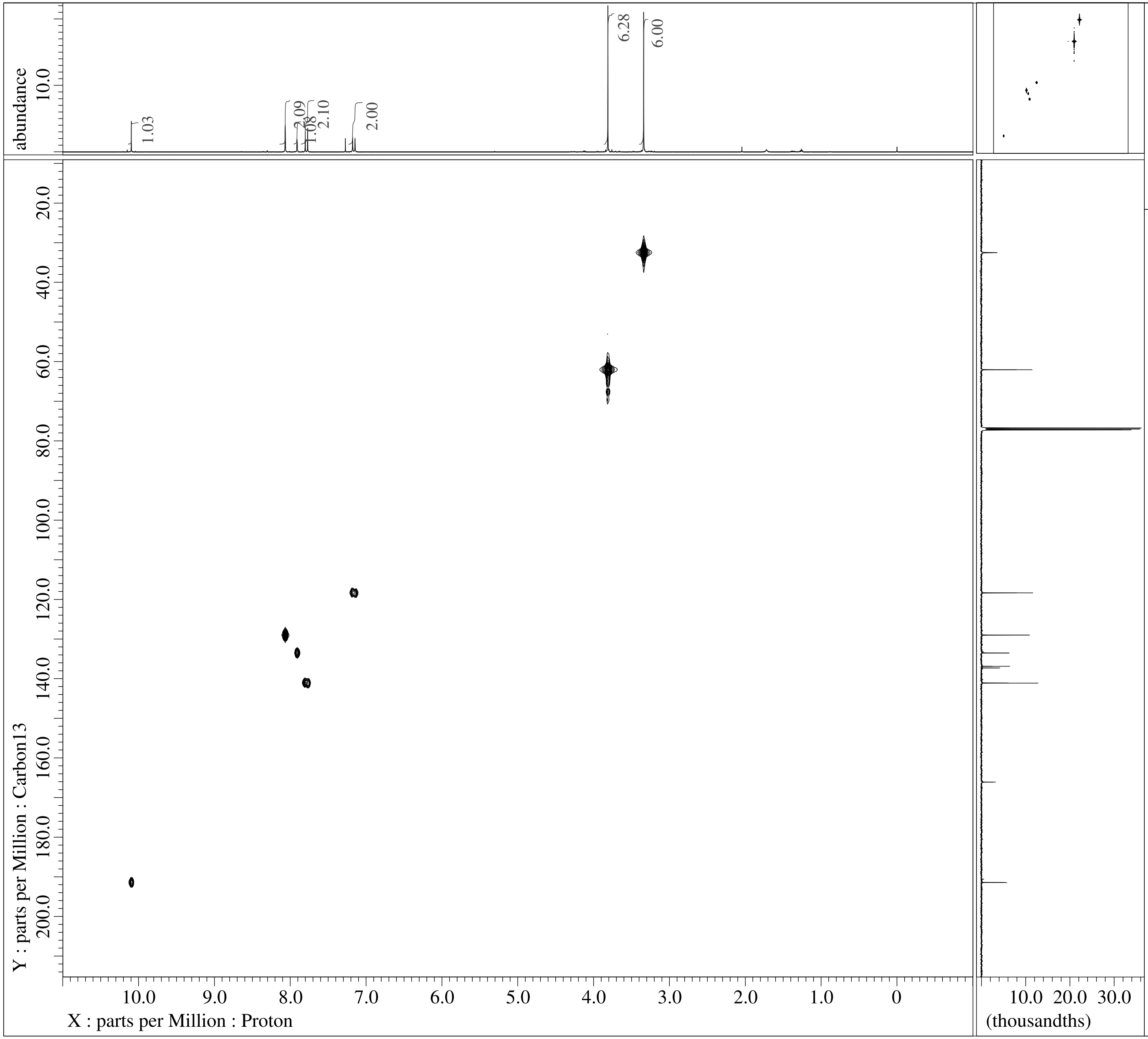


```
---- PROCESSING PARAMETERS ----
sinbell4( -60, 160 )
zerofill( 1 )
fft( 1, TRUE, TRUE )
ppm
[transpose]
sinbell4( -60, 160 )
zerofill( 2 )
fft( 1, TRUE, TRUE )
ppm
abs
```

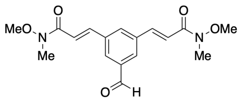

(*E*)-20af

```
Filename            = HT-1467-PTLC 3_13C_HMQC
Author              = delta
Experiment          = hmqc.jxp
Sample_Id           = HT-1467-PTLC 3_13C
Solvent             = CHLOROFORM-D
Creation_Time       =  5-JUN-2023 20:49:53
Revision_Time       = 11-APR-2024 12:01:38
Current_Time        = 11-APR-2024 12:02:03

Comment             = gradient enhanced HMQC
Data_Format         = 2D REAL REAL
Dim_Size            = 819, 512
Dim_Title           = Proton Carbon13
Dim_Units           = [ppm] [ppm]
Dimensions          = X Y
Site                = JNM-ECA500II
Spectrometer        = DELTA2_NMR

Field_Strength      = 11.7473579[T] (500[MHz]
X_Acq_Duration      = 0.10911744[s]
X_Domain            = 1H
X_Freq              = 500.15991521[MHz]
X_Offset            = 5.0[ppm]
X_Points            = 1024
X_Prescans          = 4
X_Resolution        = 9.16443788[Hz]
X_Sweep             = 9.38438438[kHz]
X_Sweep_Clipped     = 7.50750751[kHz]
Y_Domain            = 13C
Y_Freq              = 125.76529768[MHz]
Y_Offset            = 112.08028914[ppm]
Y_Points            = 256
Y_Prescans          = 0
Y_Resolution        = 101.51377339[Hz]
Y_Sweep             = 25.98752599[kHz]
Tri_Domain          = Proton
Tri_Freq            = 500.15991521[MHz]
Tri_Offset          = 5.0[ppm]
Clipped             = FALSE
Scans               = 8
Total_Scans         = 2048

Relaxation_Delay    = 1.5[s]
Recvr_Gain          = 50
Temp_Get            = 23.9[dC]
X_Acq_Time          = 0.10911744[s]
X_Atn               = 3.2[dB]
X_Gamma             = 42576375
X_Pulse             = 7.68[us]
```

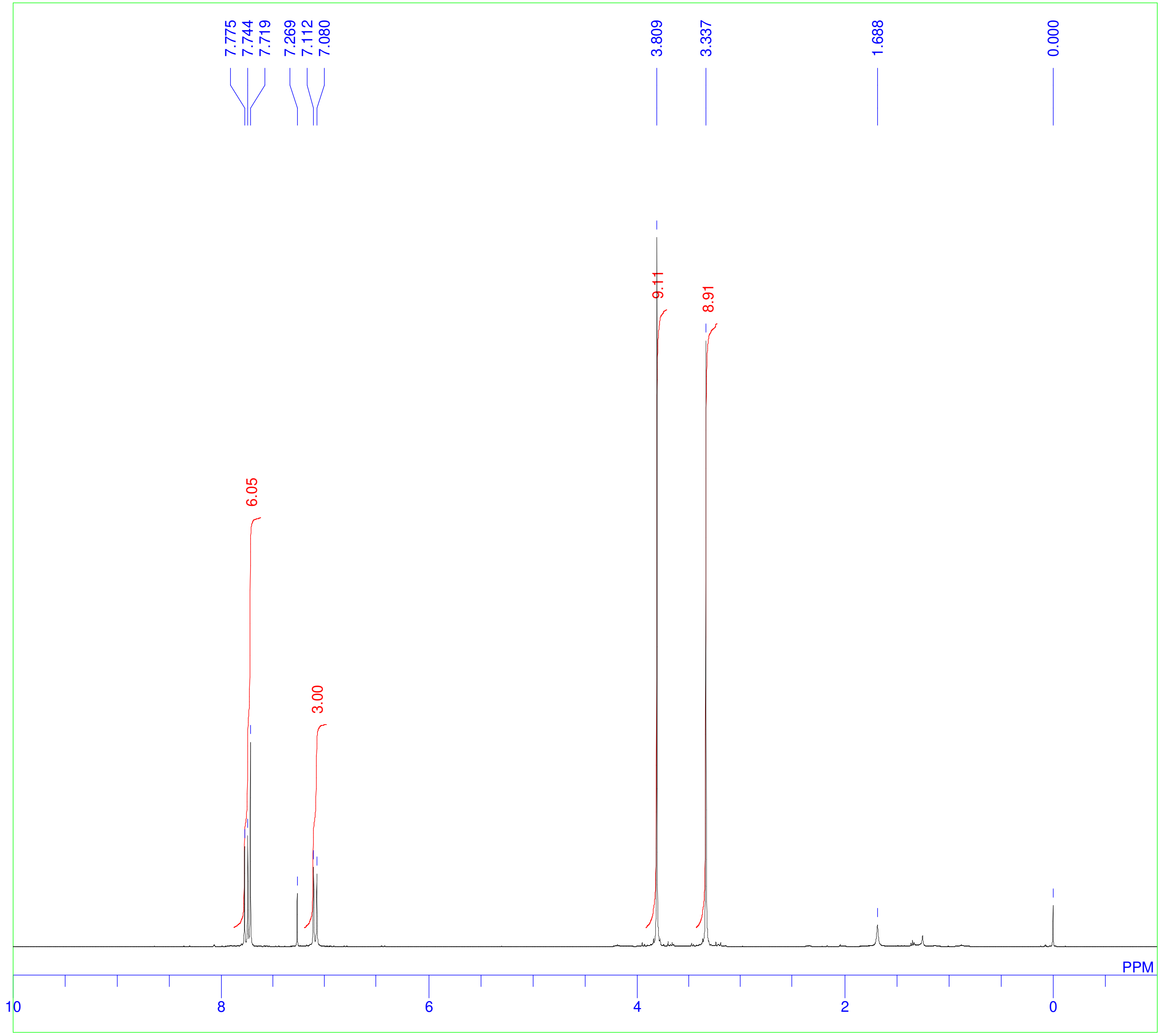

DFILE (E,E,E)-20af_1H.als
COMNT
DATIM 2023-06-01 22:42:51
OBNUC 1H
EXMOD proton.jxp
OBFRQ 500.16 MHz
OBSET 2.41 KHz
OBFIN 6.01 Hz
POINT 13107
FREQU 7507.51 Hz
SCANS 8
ACQTM 1.7459 sec
PD 5.0000 sec
PW1 3.84 usec
IRNUC 1H
CTEMP 23.8 c
SLVNT CDCL3
EXREF 0.00 ppm
BF 1.00 Hz
RGAIN 38

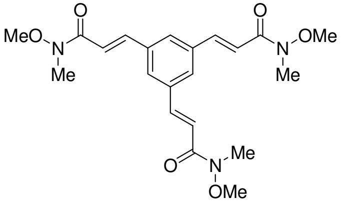

(*E*,*E*,*E*)-20af

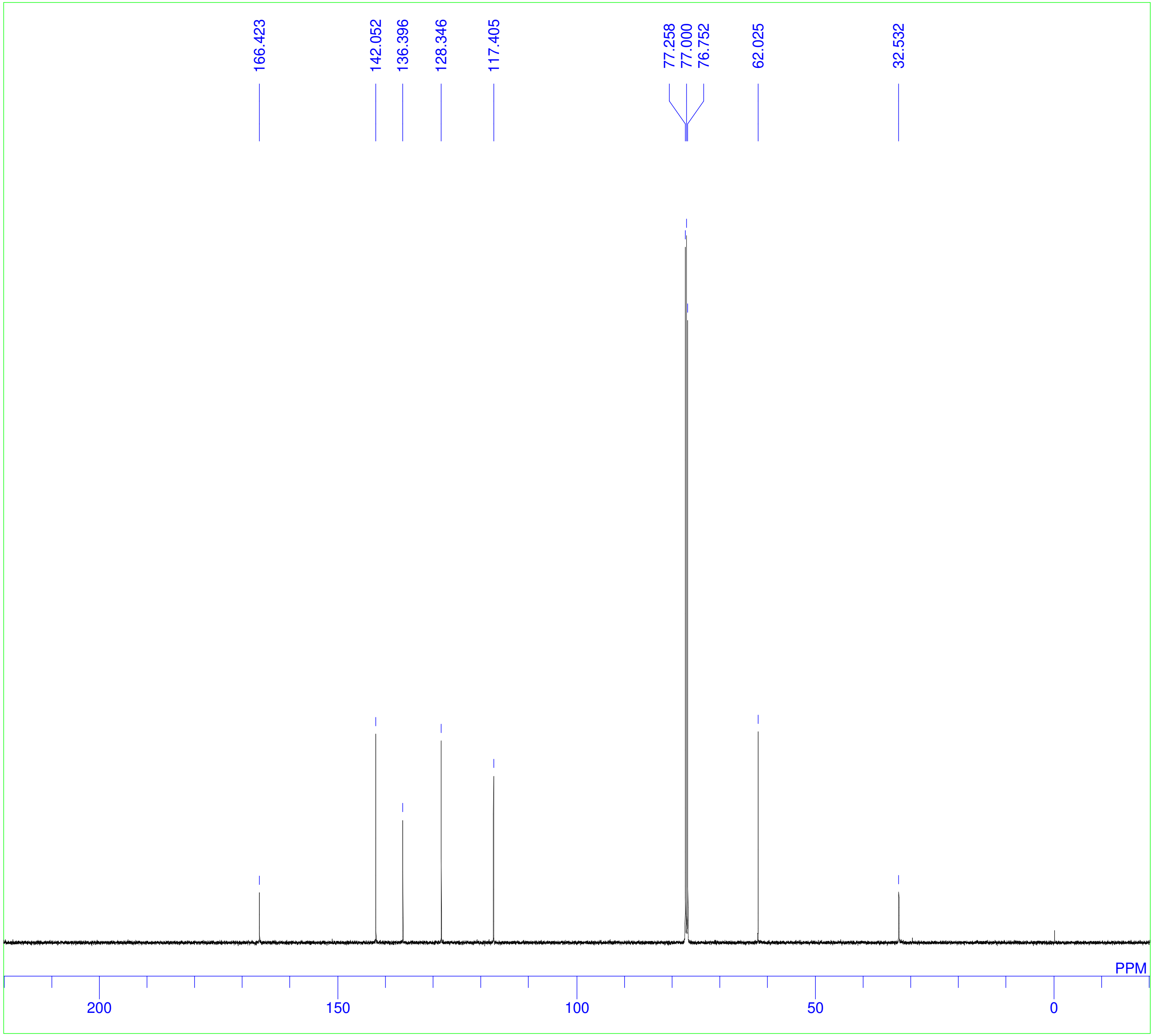

DFILE	(E,E,E)-20af_13C.als
COMNT	
DATIM	2023-06-01 22:44:27
OBNUC	13C
EXMOD	carbon.jxp
OBFRQ	125.77 MHz
OBSET	7.87 KHz
OBFIN	4.21 Hz
POINT	26214
FREQU	31446.54 Hz
SCANS	4096
ACQTM	0.8336 sec
PD	2.0000 sec
PW1	3.87 usec
IRNUC	1H
CTEMP	23.8 c
SLVNT	CDCL3
EXREF	77.00 ppm
BF	1.00 Hz
RGAIN	30

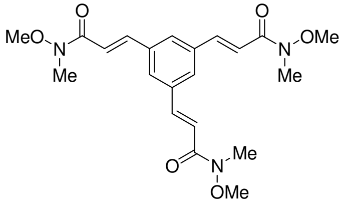

(*E*,*E*,*E*)-20af

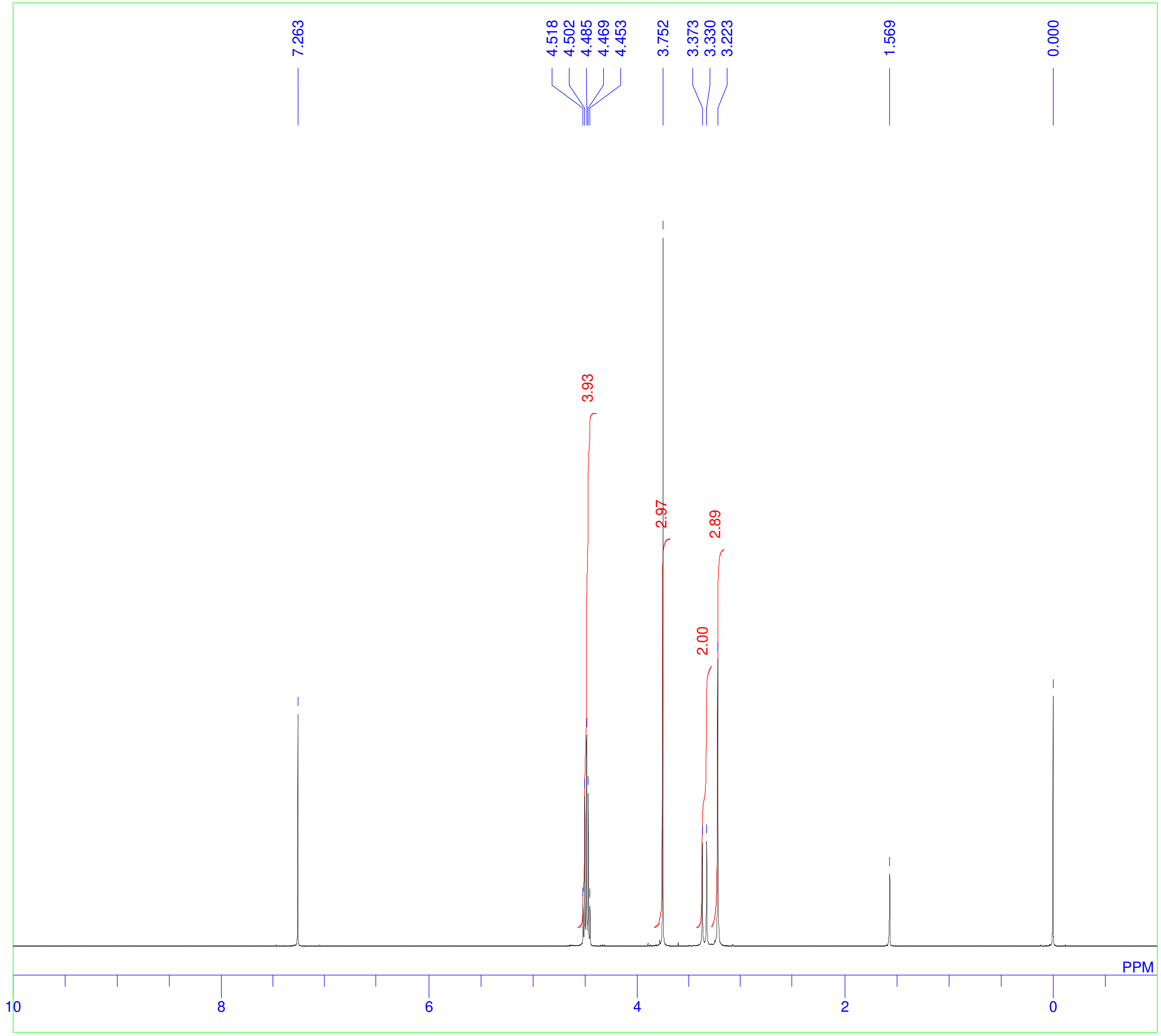

DFILE	21_1H.als
COMNT	
DATIM	2023-05-18 16:15:20
OBNUC	1H
EXMOD	proton.jxp
OBFRQ	500.16 MHz
OBSET	2.41 KHz
OBFIN	6.01 Hz
POINT	13107
FREQU	7507.51 Hz
SCANS	8
ACQTM	1.7459 sec
PD	5.0000 sec
PW1	3.84 usec
IRNUC	1H
CTEMP	24.0 c
SLVNT	CDCL3
EXREF	0.00 ppm
BF	0.30 Hz
RGAIN	46

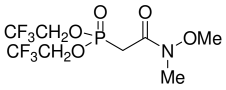

21

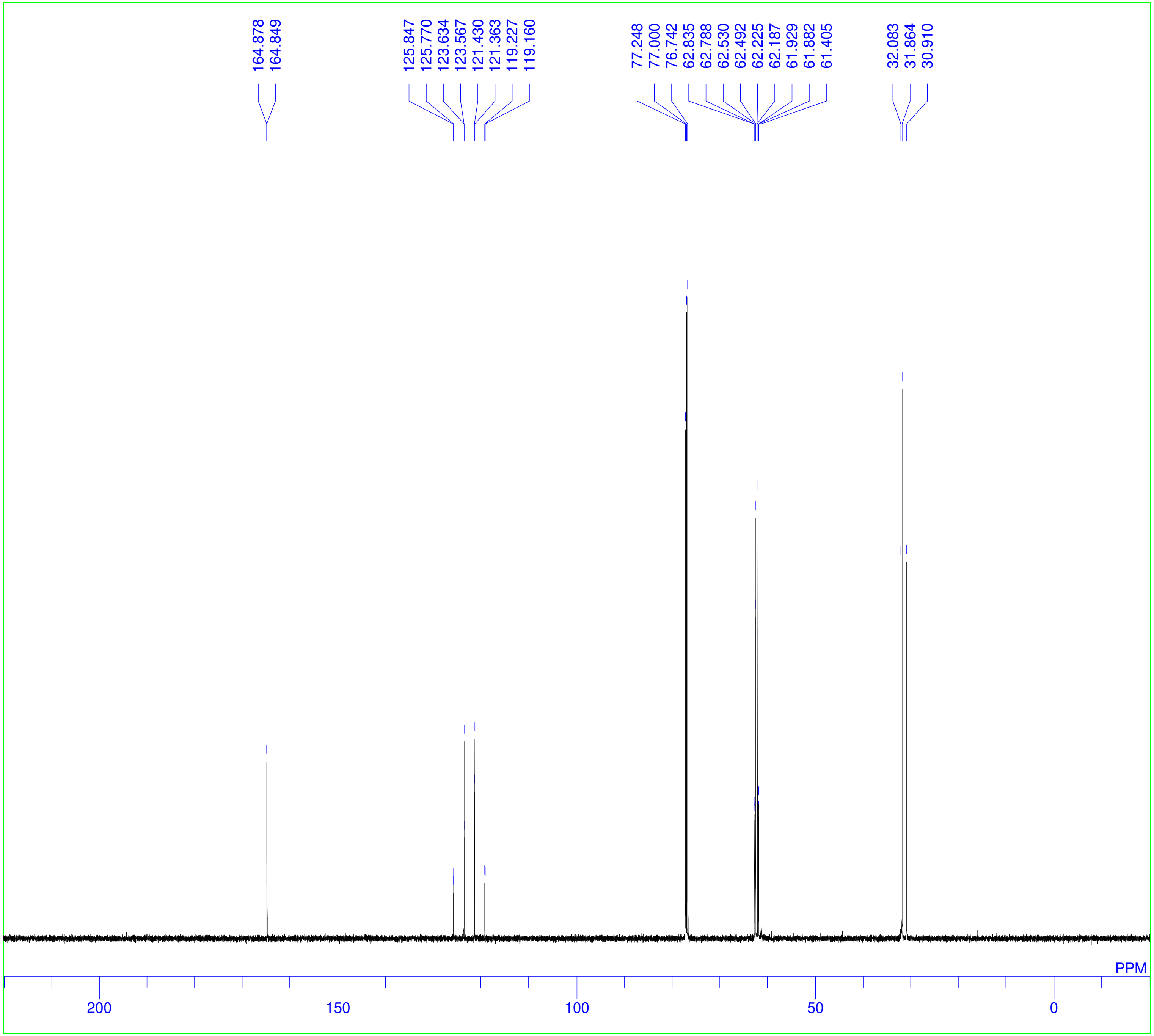

DFILE	21_13C.als
COMNT	
DATIM	2022-12-27 12:03:09
OBNUC	13C
EXMOD	carbon.jxp
OBFRQ	125.77 MHz
OBSET	7.87 KHz
OBFIN	4.21 Hz
POINT	26214
FREQU	31446.54 Hz
SCANS	1024
ACQTM	0.8336 sec
PD	2.0000 sec
PW1	3.87 usec
IRNUC	1H
CTEMP	21.7 c
SLVNT	CDCL3
EXREF	77.00 ppm
BF	0.30 Hz
RGAIN	26

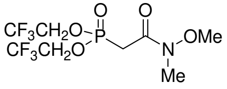

21

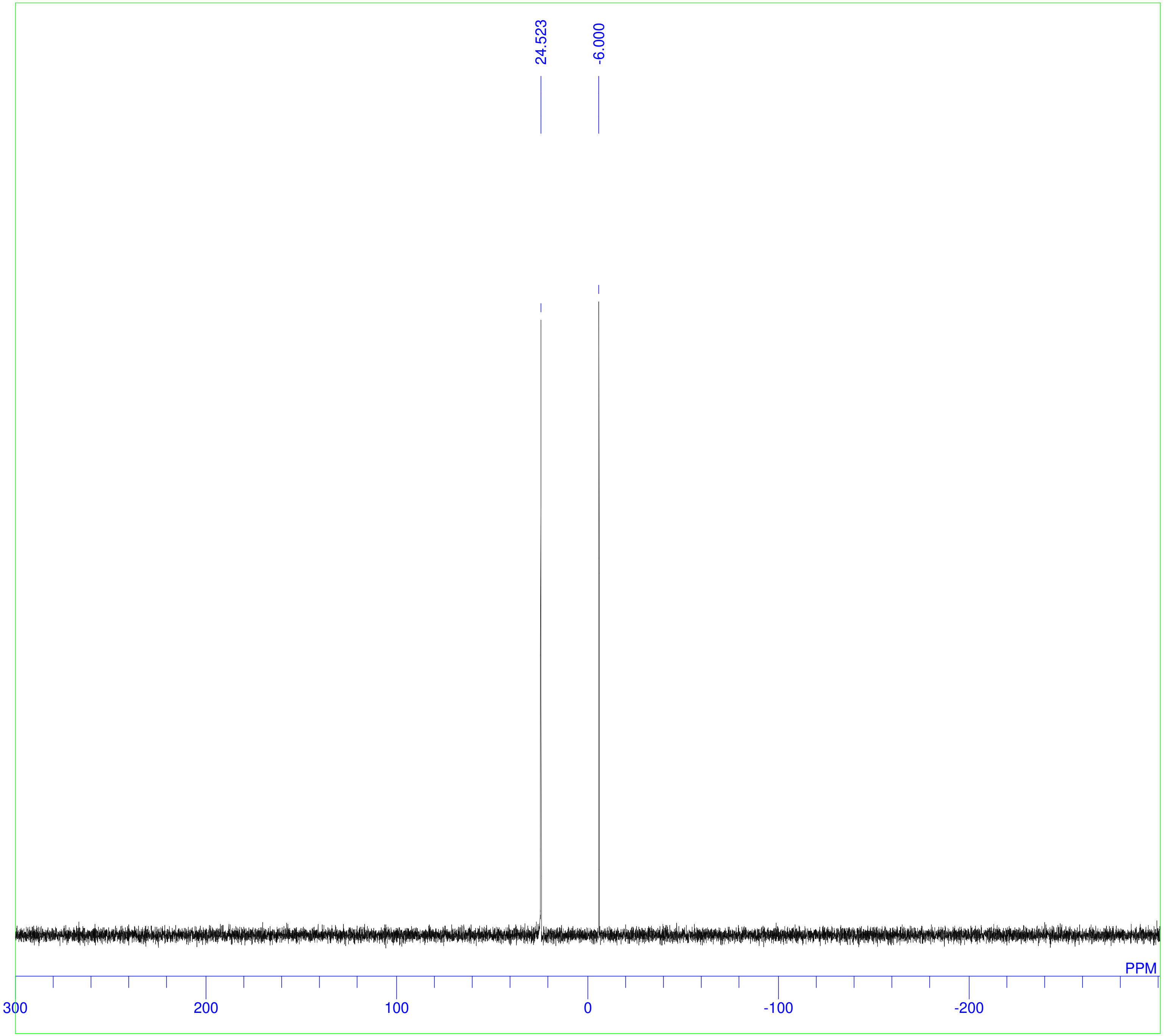

DFILE 21_31P.als
COMNT
DATIM 2023-11-05 14:40:16
OBNUC 31P
EXMOD single_pulse.jxp
OBFRQ 202.46 MHz
OBSET 8.31 KHz
OBFIN 0.75 Hz
POINT 13107
FREQU 142857.14 Hz
SCANS 64
ACQTM 0.0918 sec
PD 5.0000 sec
PW1 6.45 usec
IRNUC 31P
CTEMP 23.8 c
SLVNT CDCL3
EXREF -6.00 ppm
BF 0.30 Hz
RGAIN 50

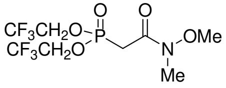

21

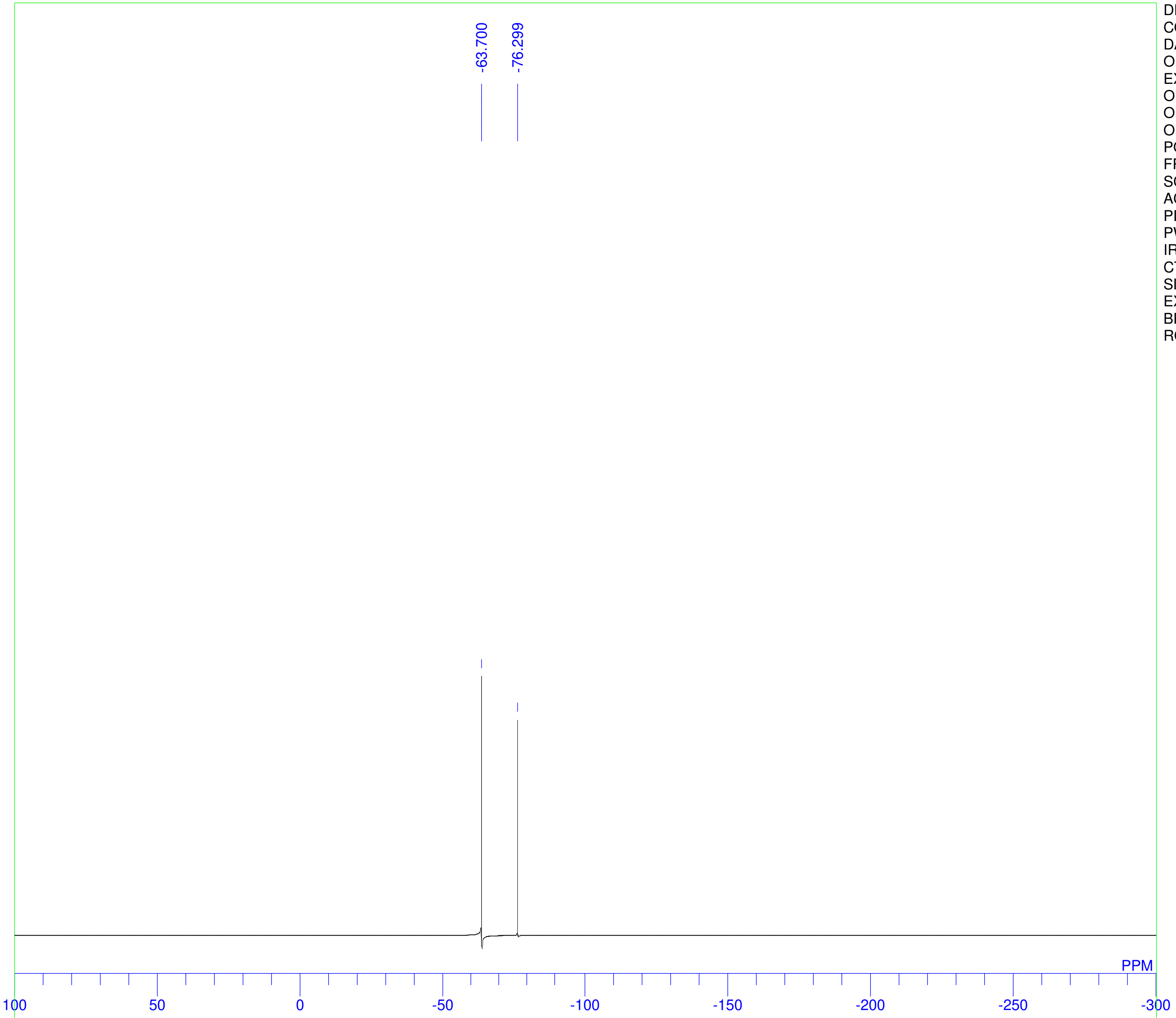

DFILE	21_19F.als
COMNT	
DATIM	2024-02-11 16:11:44
OBNUC	19F
EXMOD	single_pulse.jxp
OBFRQ	470.62 MHz
OBSET	0.46 KHz
OBFIN	0.84 Hz
POINT	13107
FREQU	285714.28 Hz
SCANS	8
ACQTM	0.0459 sec
PD	5.0000 sec
PW1	4.25 usec
IRNUC	19F
CTEMP	21.4 c
SLVNT	CDCL3
EXREF	-63.70 ppm
BF	0.30 Hz
RGAIN	50

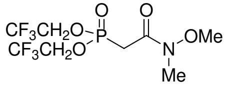

21

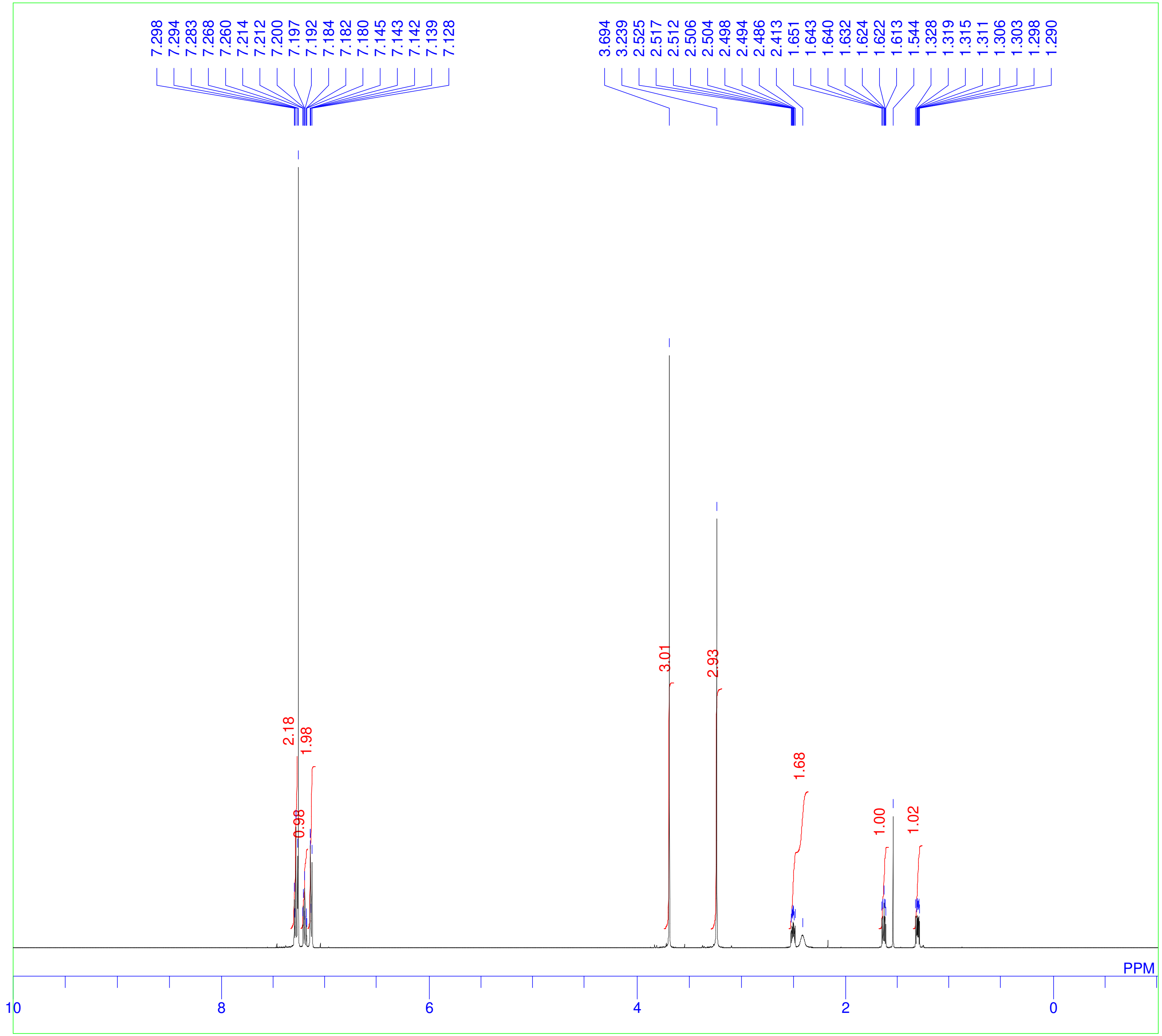

DFILE trans-22_1H.als
COMNT
DATIM 2022-12-15 21:34:49
OBNUC 1H
EXMOD proton.jxp
OBFRQ 500.16 MHz
OBSET 2.41 KHz
OBFIN 6.01 Hz
POINT 13107
FREQU 7507.51 Hz
SCANS 8
ACQTM 1.7459 sec
PD 5.0000 sec
PW1 3.84 usec
IRNUC 1H
CTEMP 23.4 c
SLVNT CDCL3
EXREF 7.26 ppm
BF 0.30 Hz
RGAIN 48

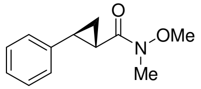

***trans*-22**

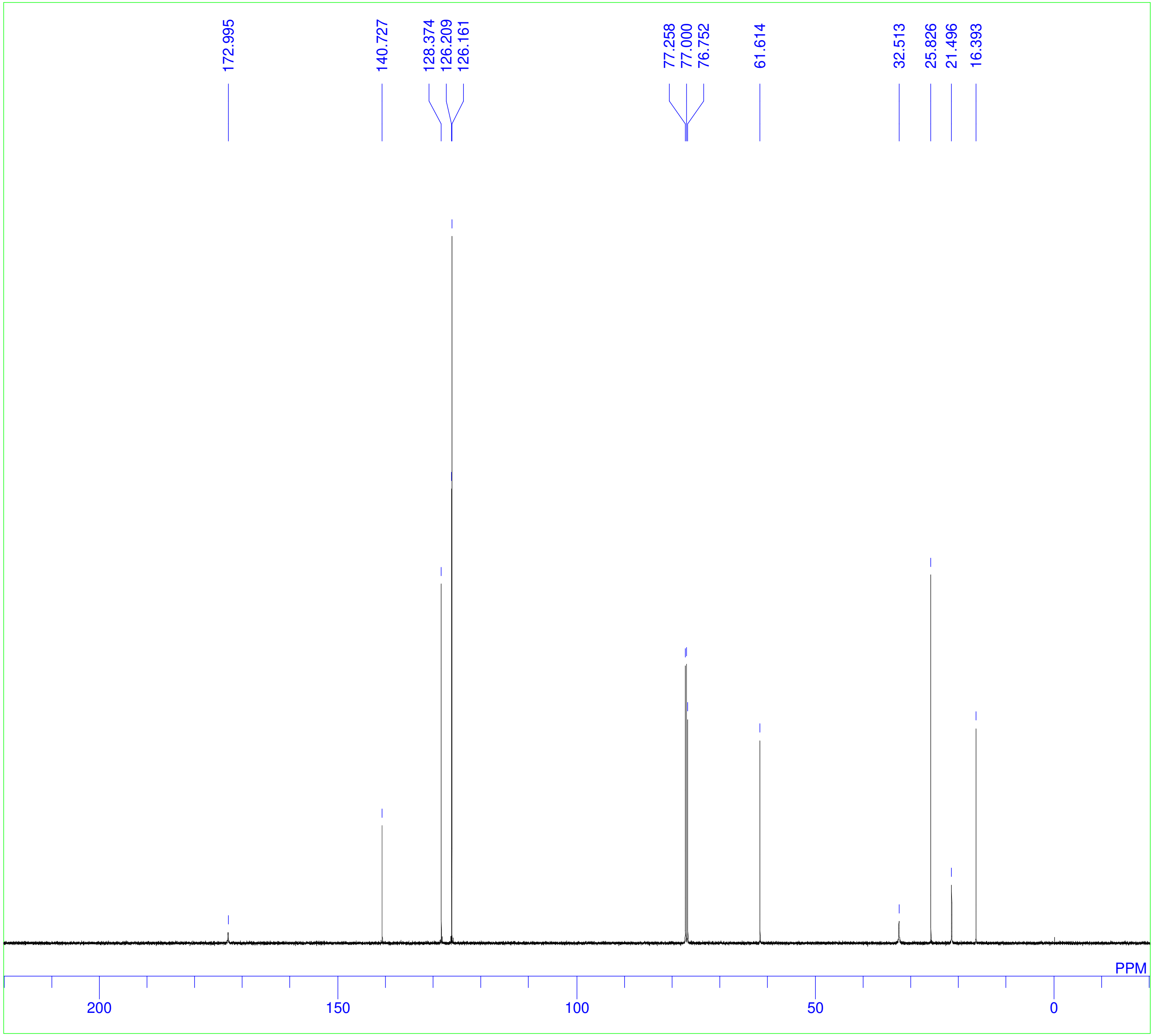

DFILE	trans-22_13C.als
COMNT	
DATIM	2023-04-22 08:26:30
OBNUC	13C
EXMOD	carbon.jxp
OBFRQ	125.77 MHz
OBSET	7.87 KHz
OBFIN	4.21 Hz
POINT	26214
FREQU	31446.54 Hz
SCANS	1024
ACQTM	0.8336 sec
PD	2.0000 sec
PW1	3.87 usec
IRNUC	1H
CTEMP	24.0 c
SLVNT	CDCL3
EXREF	77.00 ppm
BF	0.30 Hz
RGAIN	30

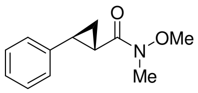

***trans*-22**

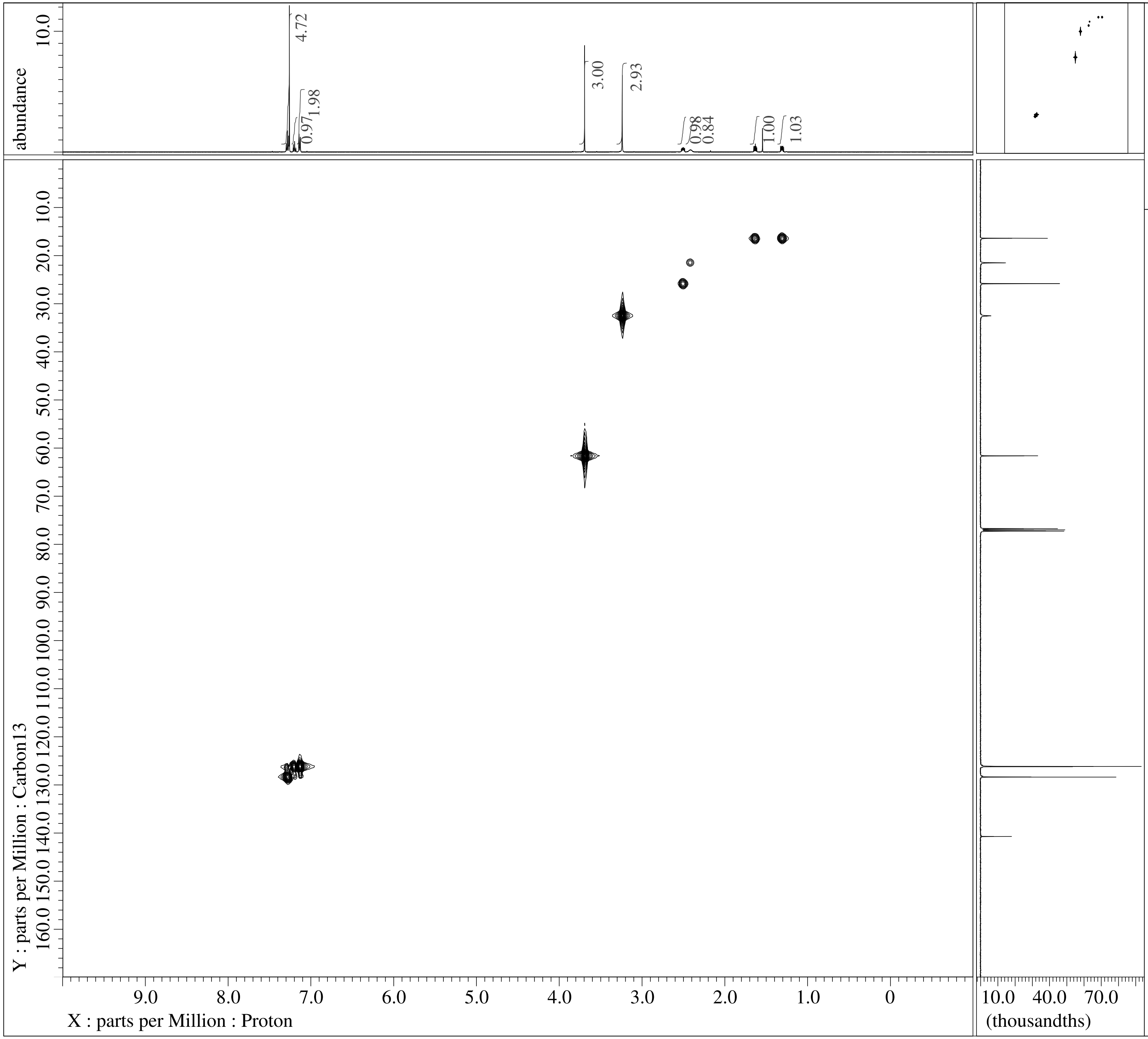


```
---- PROCESSING PARAMETERS ----
sinbell4( -60, 160 )
zerofill( 1 )
fft( 1, TRUE, TRUE )
ppm
[transpose]
sinbell4( -60, 160 )
zerofill( 2 )
fft( 1, TRUE, TRUE )
ppm
abs
```

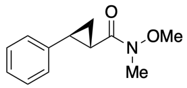

trans-**22**

```
Filename            = HT-1366-TM_HMQC,HMBC_HM
Author              = delta
Experiment          = hmqc.jxp
Sample_Id           = HT-1366-TM_HMQC,HMBC
Solvent             = CHLOROFORM-D
Creation_Time       = 11-JAN-2024 14:46:30
Revision_Time       = 11-APR-2024 12:04:52
Current_Time        = 11-APR-2024 12:05:25

Comment             = gradient enhanced HMQC
Data_Format         = 2D REAL REAL
Dim_Size            = 819, 512
Dim_Title           = Proton Carbon13
Dim_Units           = [ppm] [ppm]
Dimensions          = X Y
Site                = JNM-ECA500II
Spectrometer        = DELTA2_NMR

Field_Strength      = 11.7473579[T] (500[MHz]
X_Acq_Duration      = 0.10911744[s]
X_Domain            = 1H
X_Freq              = 500.15991521[MHz]
X_Offset            = 5.0[ppm]
X_Points            = 1024
X_Prescans          = 4
X_Resolution        = 9.16443788[Hz]
X_Sweep             = 9.38438438[kHz]
X_Sweep_Clipped     = 7.50750751[kHz]
Y_Domain            = 13C
Y_Freq              = 125.76529768[MHz]
Y_Offset            = 85[ppm]
Y_Points            = 256
Y_Prescans          = 0
Y_Resolution        = 83.60980308[Hz]
Y_Sweep             = 21.40410959[kHz]
Tri_Domain          = Proton
Tri_Freq            = 500.15991521[MHz]
Tri_Offset          = 5.0[ppm]
Clipped             = FALSE
Scans               = 4
Total_Scans         = 1024

Relaxation_Delay    = 1.5[s]
Recvr_Gain          = 50
Temp_Get            = 21.1[dC]
X_Acq_Time          = 0.10911744[s]
X_Atn               = 3.2[dB]
X_Gamma             = 42576375
X_Pulse             = 7.59[us]
```

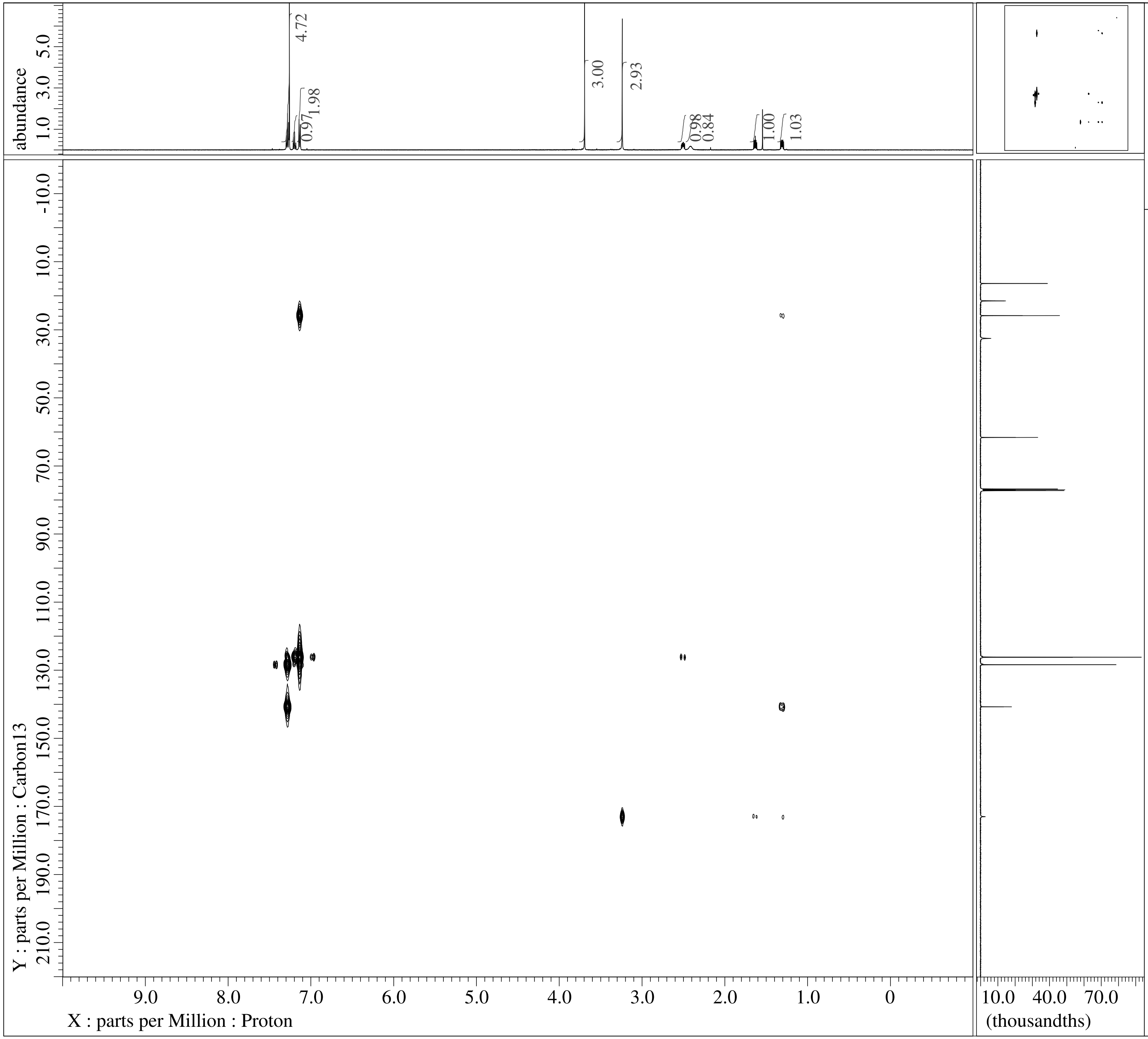


```
---- PROCESSING PARAMETERS ----
gauss( 5.0[Hz], 0.0[s] )
sinbell_auto
zerofill( 1 )
fft( 1, TRUE, TRUE )
ppm
[transpose]
sinbell4( -60, 160 )
trapezoid( 0[%], 5[%], 80[%], 100[%] )
zerofill( 2 )
fft( 1, TRUE, TRUE )
```

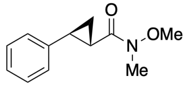

***trans*-22**

```
Filename            = HT-1366-TM_HMQC,HMBC_HM
Author              = delta
Experiment          = hmbc.jxp
Sample_Id           = HT-1366-TM_HMQC,HMBC
Solvent             = CHLOROFORM-D
Creation_Time       = 11-JAN-2024 15:14:45
Revision_Time       = 11-APR-2024 12:06:49
Current_Time        = 11-APR-2024 12:08:08

Comment             = gradient enhanced HMBC
Data_Format         = 2D REAL REAL
Dim_Size            = 1638, 512
Dim_Title           = Proton Carbon13
Dim_Units           = [ppm] [ppm]
Dimensions          = X Y
Site                = JNM-ECA500II
Spectrometer        = DELTA2_NMR

Field_Strength      = 11.7473579[T] (500[MHz]
X_Acq_Duration      = 0.21823488[s]
X_Domain            = 1H
X_Freq              = 500.15991521[MHz]
X_Offset            = 5.0[ppm]
X_Points            = 2048
X_Prescans          = 4
X_Resolution        = 4.58221894[Hz]
X_Sweep             = 9.38438438[kHz]
X_Sweep_Clipped     = 7.50750751[kHz]
Y_Domain            = 13C
Y_Freq              = 125.76529768[MHz]
Y_Offset            = 100[ppm]
Y_Points            = 256
Y_Prescans          = 0
Y_Resolution        = 122.99275819[Hz]
Y_Sweep             = 31.4861461[kHz]
Tri_Domain          = Proton
Tri_Freq            = 500.15991521[MHz]
Tri_Offset          = 5.0[ppm]
Clipped             = FALSE
Scans               = 4
Total_Scans         = 1024

Relaxation_Delay    = 1.5[s]
Recvr_Gain          = 50
Temp_Get            = 21.1[dC]
X_Acq_Time          = 0.21823488[s]
X_Atn               = 3.2[dB]
X_Gamma             = 42576375
X_Pulse             = 7.59[us]
```

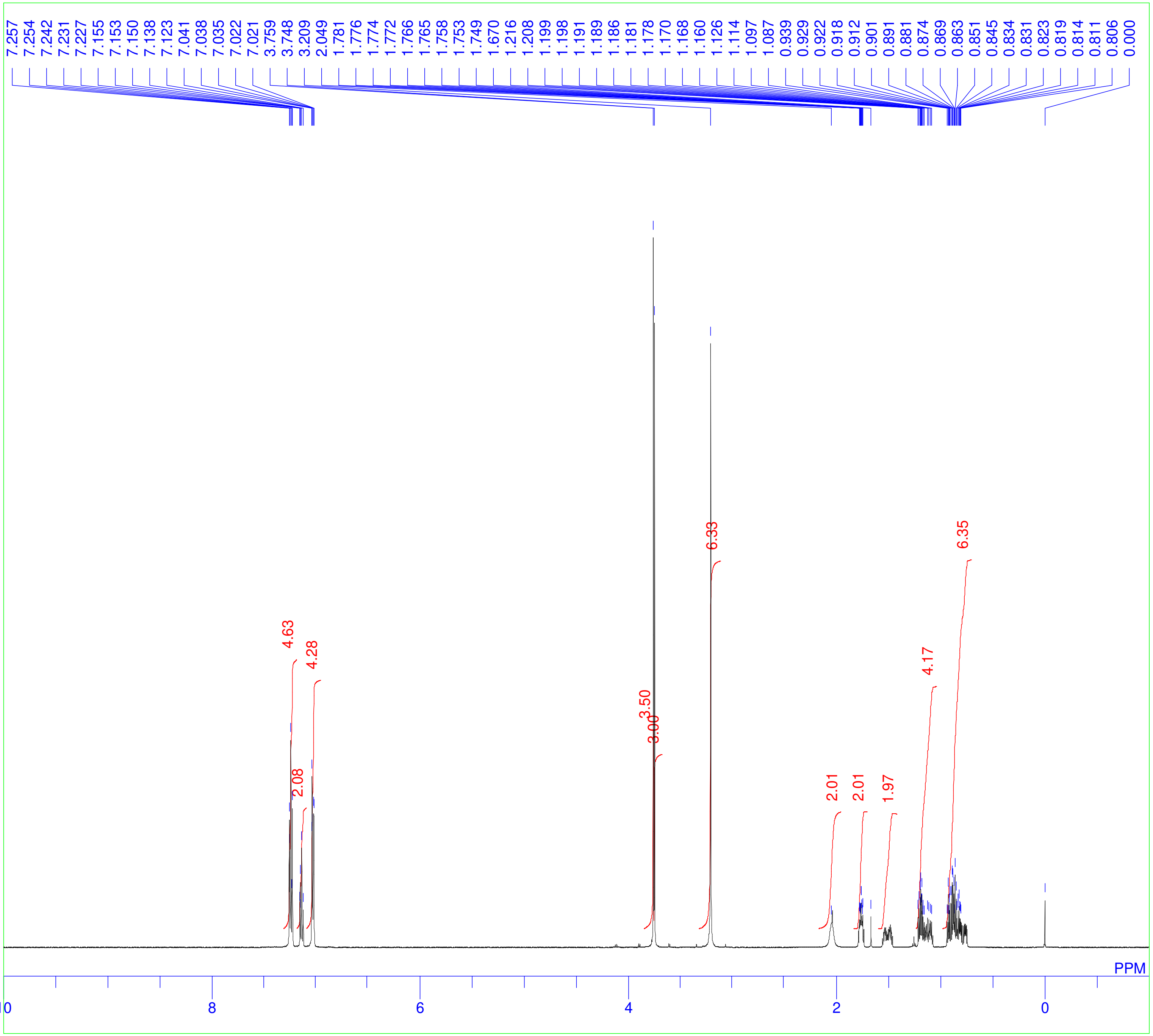

DFILE	23 (trans-syn-trans-23 + trans-anti-trans-23)_1H.als
COMNT	
DATIM	2023-11-30 20:15:35
OBNUC	1H
EXMOD	proton.jxp
OBFRQ	500.16 MHz
OBSET	2.41 KHz
OBFIN	6.01 Hz
POINT	13107
FREQU	7507.51 Hz
SCANS	8
ACQTM	1.7459 sec
PD	5.0000 sec
PW1	3.80 usec
IRNUC	1H
CTEMP	23.8 c
SLVNT	CDCL3
EXREF	0.00 ppm
BF	0.30 Hz
RGAIN	30

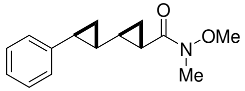

***trans-syn-trans*-23**

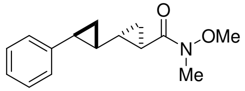

***trans-anti-trans*-23**

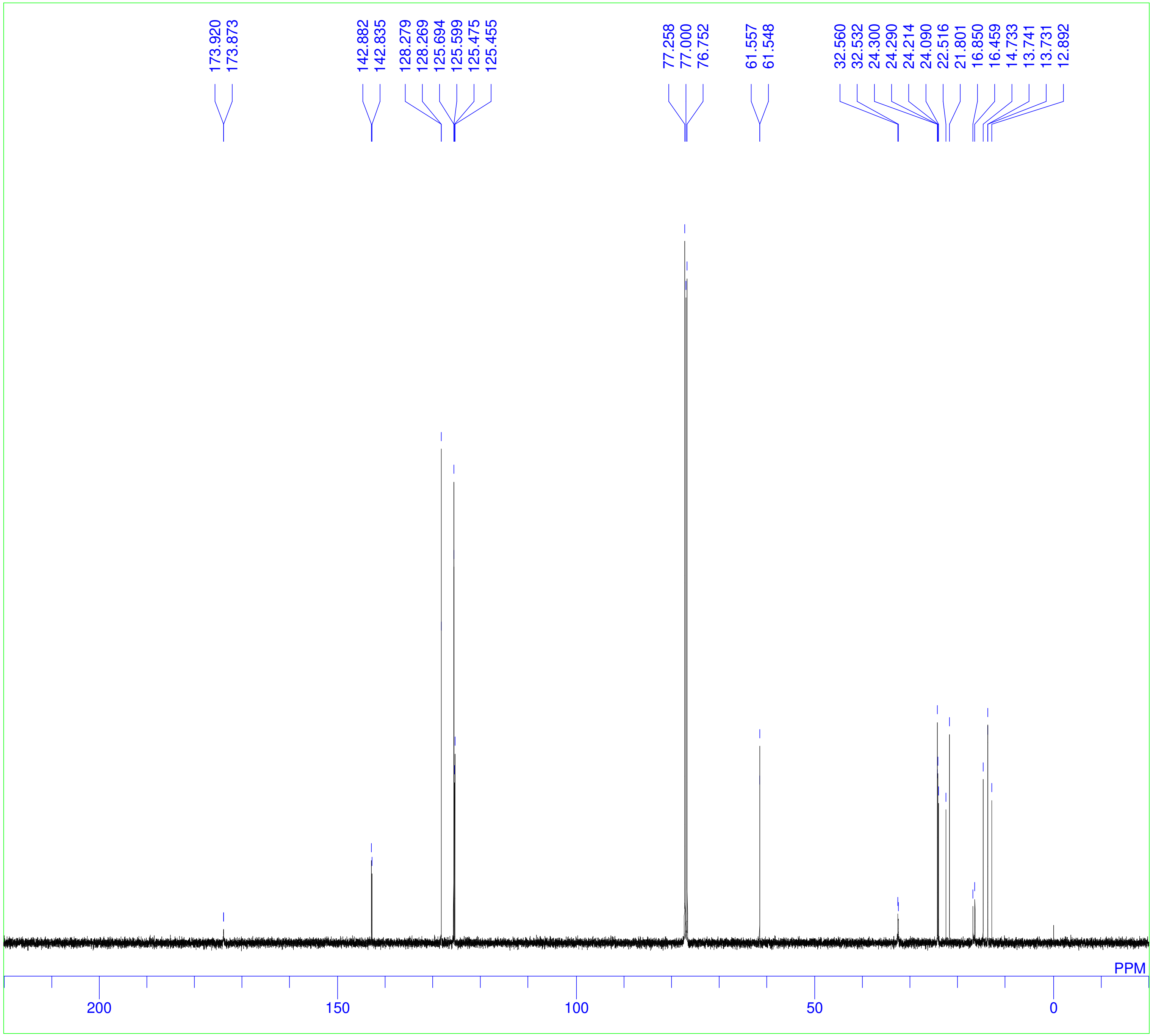

DFILE 23 (trans-syn-trans-23 + trans-anti-trans-23)_13C.als
COMNT
DATIM 2023-11-30 20:17:13
OBNUC 13C
EXMOD carbon.jxp
OBFRQ 125.77 MHz
OBSET 7.87 KHz
OBFIN 4.21 Hz
POINT 26214
FREQU 31446.54 Hz
SCANS 1024
ACQTM 0.8336 sec
PD 2.0000 sec
PW1 4.30 usec
IRNUC 1H
CTEMP 23.8 c
SLVNT CDCL3
EXREF 77.00 ppm
BF 0.30 Hz
RGAIN 30

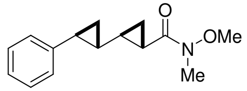

***trans-syn-trans*-23**

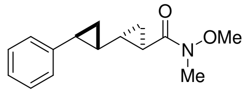

***trans-anti-trans*-23**

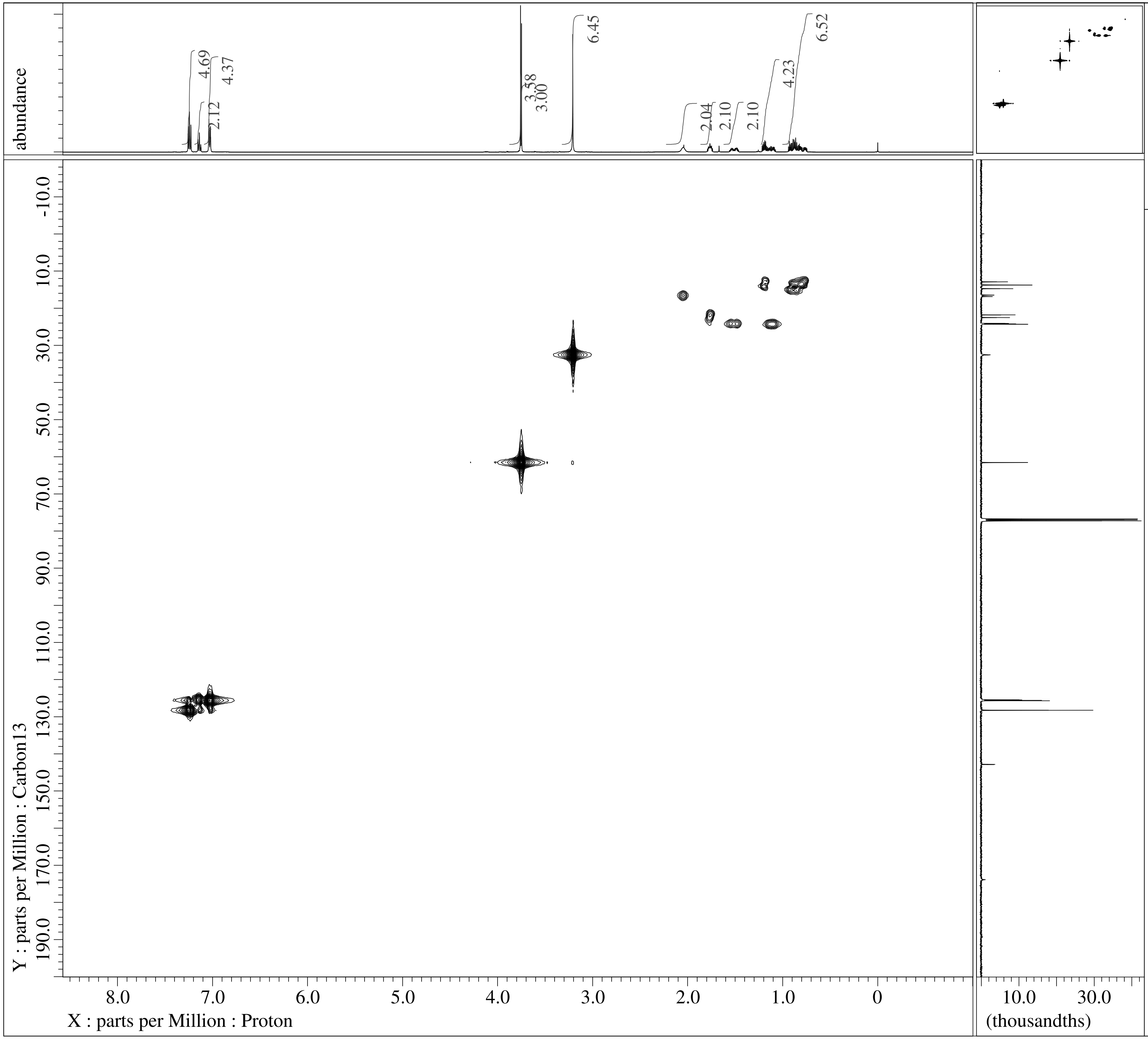


```
---- PROCESSING PARAMETERS ---
sinbell4( -60, 160 )
zerofill( 1 )
fft( 1, TRUE, TRUE )
ppm
[transpose]
sinbell4( -60, 160 )
zerofill( 2 )
fft( 1, TRUE, TRUE )
ppm
abs
```

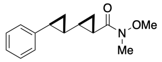

trans-syn-trans-23

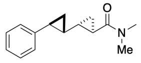

trans-anti-trans-23

```
Filename            = HT-1628-PTLC 1_HMQC-1-5
Author              = delta
Experiment          = hmqc.jxp
Sample_Id           = HT-1628-PTLC 1
Solvent             = CHLOROFORM-D
Creation_Time       = 30-NOV-2023 21:06:05
Revision_Time       = 11-APR-2024 12:09:47
Current_Time        = 11-APR-2024 12:10:20

Comment             = gradient enhanced HMQC
Data_Format         = 2D REAL REAL
Dim_Size            = 819, 512
Dim_Title           = Proton Carbon13
Dim_Units           = [ppm] [ppm]
Dimensions          = X Y
Site                = JNM-ECA500II
Spectrometer        = DELTA2_NMR

Field_Strength      = 11.7473579[T] (500[MHz]
X_Acq_Duration      = 0.16867328[s]
X_Domain            = 1H
X_Freq              = 500.15991521[MHz]
X_Offset            = 3.72082448[ppm]
X_Points            = 1024
X_Prescans          = 4
X_Resolution        = 5.9286213[Hz]
X_Sweep             = 6.07090821[kHz]
X_Sweep_Clipped     = 4.85672657[kHz]
Y_Domain            = 13C
Y_Freq              = 125.76529768[MHz]
Y_Offset            = 87.53340868[ppm]
Y_Points            = 256
Y_Prescans          = 0
Y_Resolution        = 110.72137188[Hz]
Y_Sweep             = 28.3446712[kHz]
Tri_Domain          = Proton
Tri_Freq            = 500.15991521[MHz]
Tri_Offset          = 5.0[ppm]
Clipped             = FALSE
Scans               = 4
Total_Scans         = 1024

Relaxation_Delay    = 1.5[s]
Recvr_Gain          = 50
Temp_Get            = 23.8[dC]
X_Acq_Time          = 0.16867328[s]
X_Atn               = 3.2[dB]
X_Gamma             = 42576375
X_Pulse             = 7.59[us]
```

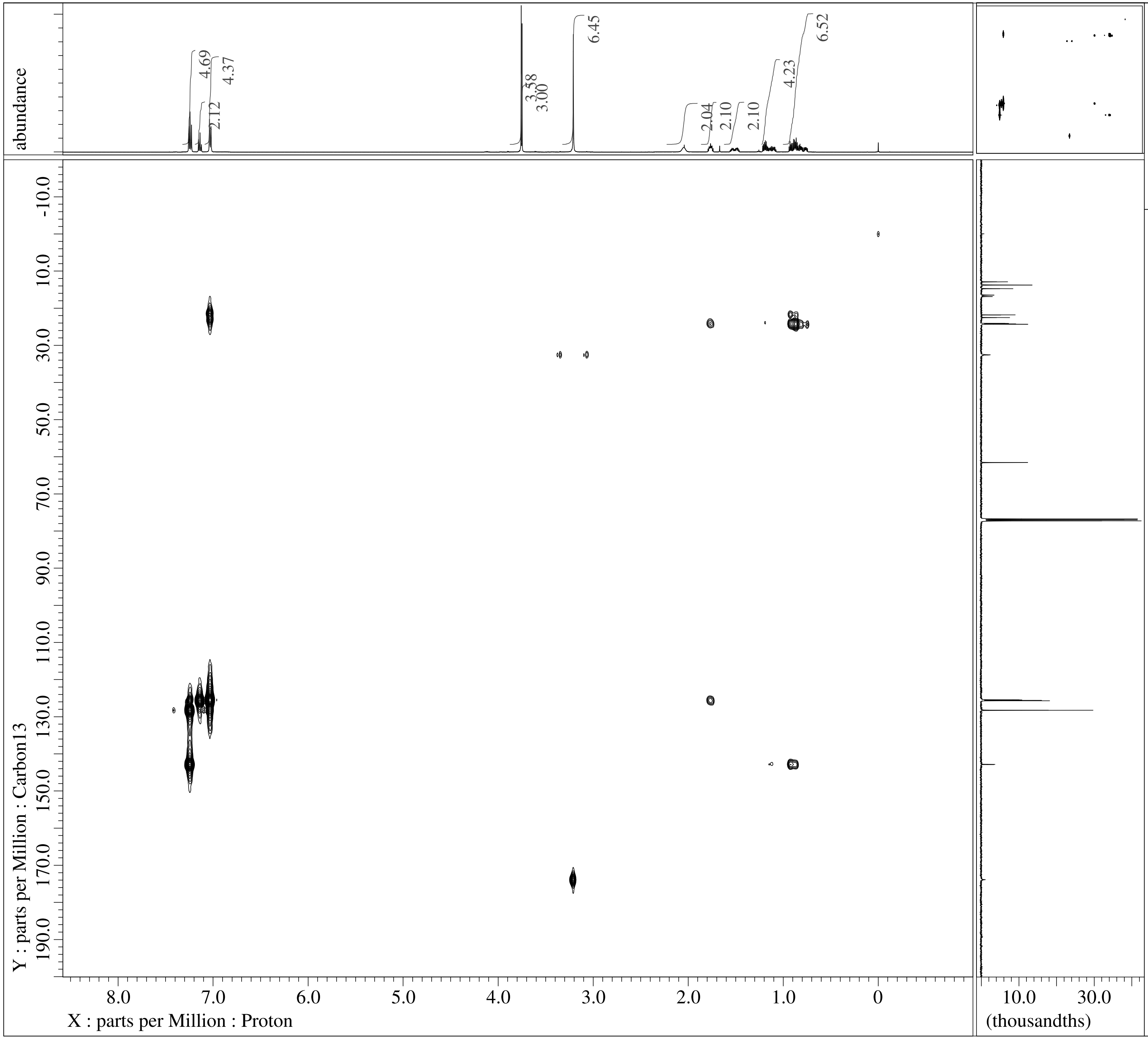


```
---- PROCESSING PARAMETERS ---
gauss( 5.0[Hz], 0.0[s] )
sinbell_auto
zerofill( 1 )
fft( 1, TRUE, TRUE )
ppm
[transpose]
sinbell4( -60, 160 )
trapezoid( 0[%], 5[%], 80[%], 100[%] )
zerofill( 2 )
fft( 1, TRUE, TRUE )
```

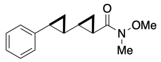

trans-syn-trans-23

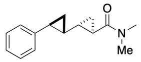

trans-anti-trans-23

```
Filename            = HT-1628-PTLC 1_HMBC-1-5
Author              = delta
Experiment          = hmbc.jxp
Sample_Id           = HT-1628-PTLC 1
Solvent             = CHLOROFORM-D
Creation_Time       = 30-NOV-2023 21:35:22
Revision_Time       = 11-APR-2024 12:11:42
Current_Time        = 11-APR-2024 12:12:31

Comment             = gradient enhanced HMBC
Data_Format         = 2D REAL REAL
Dim_Size            = 1638, 512
Dim_Title           = Proton Carbon13
Dim_Units           = [ppm] [ppm]
Dimensions          = X Y
Site                = JNM-ECA500II
Spectrometer        = DELTA2_NMR

Field_Strength      = 11.7473579[T] (500[MHz]
X_Acq_Duration      = 0.33734656[s]
X_Domain            = 1H
X_Freq              = 500.15991521[MHz]
X_Offset            = 3.72082448[ppm]
X_Points            = 2048
X_Prescans          = 4
X_Resolution        = 2.96431065[Hz]
X_Sweep             = 6.07090821[kHz]
X_Sweep_Clipped     = 4.85672657[kHz]
Y_Domain            = 13C
Y_Freq              = 125.76529768[MHz]
Y_Offset            = 87.53340868[ppm]
Y_Points            = 256
Y_Prescans          = 0
Y_Resolution        = 110.72137188[Hz]
Y_Sweep             = 28.3446712[kHz]
Tri_Domain          = Proton
Tri_Freq            = 500.15991521[MHz]
Tri_Offset          = 5.0[ppm]
Clipped             = FALSE
Scans               = 8
Total_Scans         = 2048

Relaxation_Delay    = 1.5[s]
Recvr_Gain          = 50
Temp_Get            = 23.6[dC]
X_Acq_Time          = 0.33734656[s]
X_Atn               = 3.2[dB]
X_Gamma             = 42576375
X_Pulse             = 7.59[us]
```

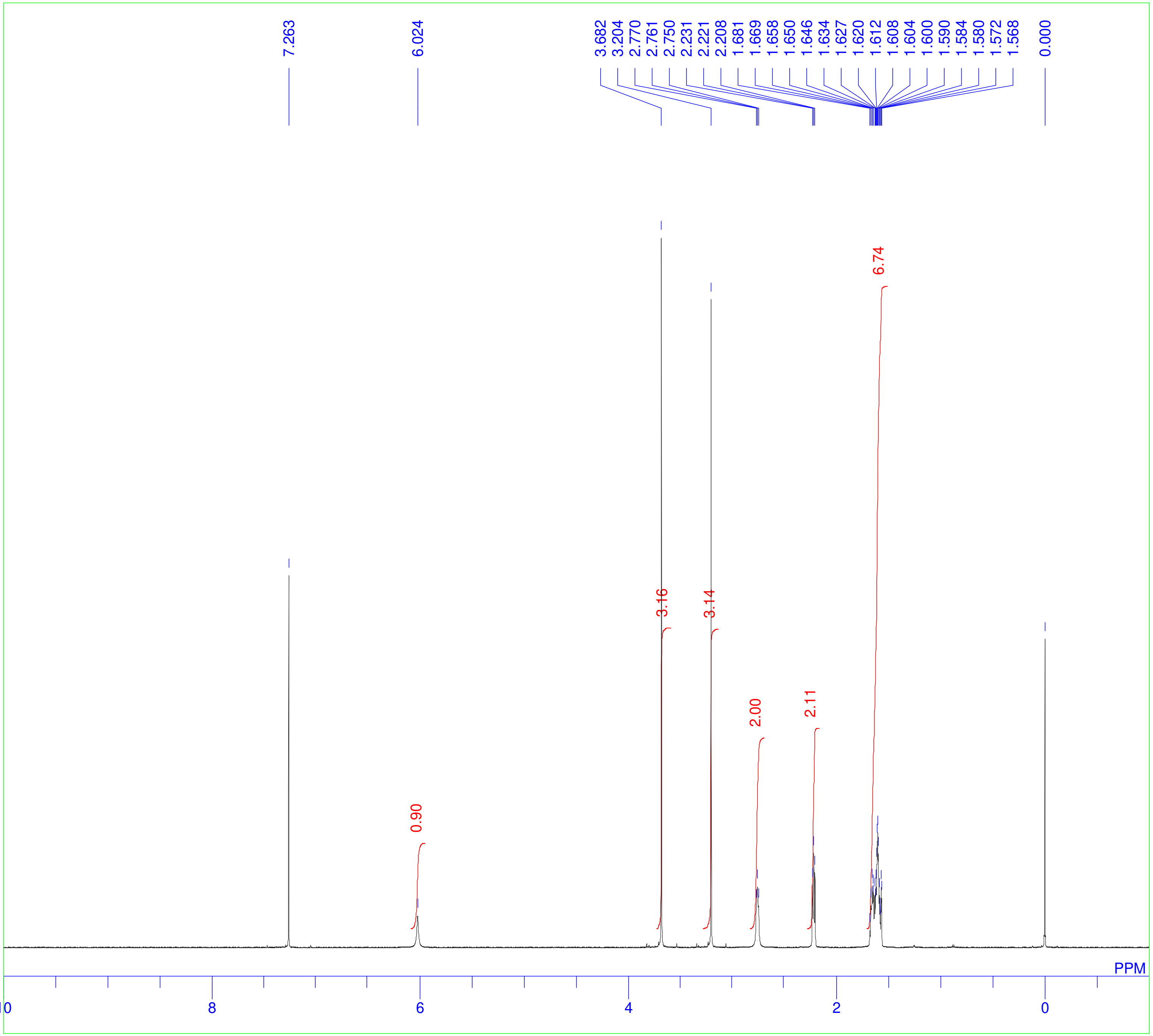

DFILE 25_1H.als
COMNT
DATIM 2023-02-25 13:49:02
OBNUC 1H
EXMOD proton.jxp
OBFRQ 500.16 MHz
OBSET 2.41 KHz
OBFIN 6.01 Hz
POINT 13107
FREQU 7507.51 Hz
SCANS 8
ACQTM 1.7459 sec
PD 5.0000 sec
PW1 3.84 usec
IRNUC 1H
CTEMP 22.0 c
SLVNT CDCL3
EXREF 0.00 ppm
BF 0.30 Hz
RGAIN 44

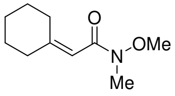

25

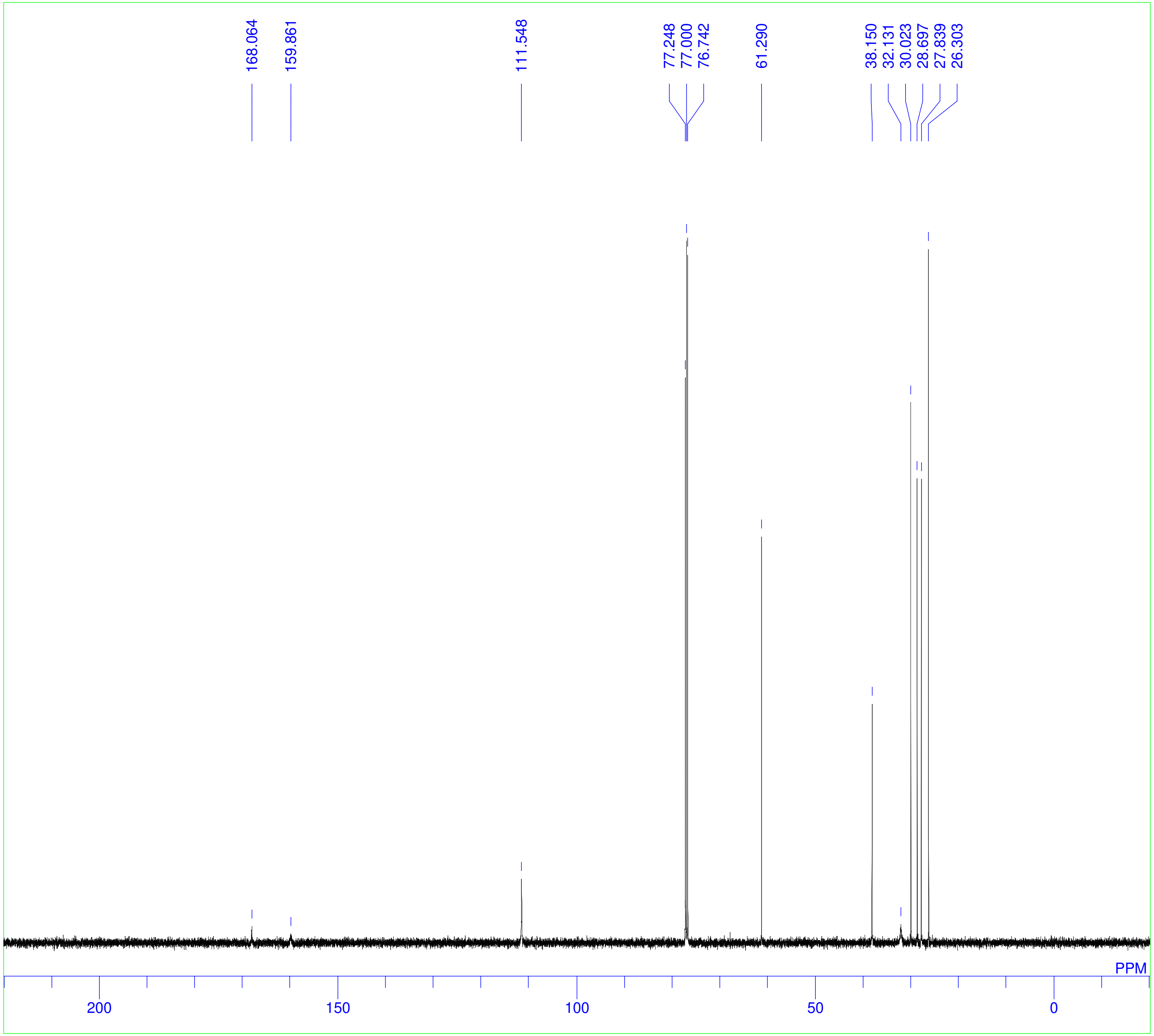

DFILE 25_13C.als
COMNT
DATIM 2023-02-26 16:58:08
OBNUC 13C
EXMOD carbon.jxp
OBFRQ 125.77 MHz
OBSET 7.87 KHz
OBFIN 4.21 Hz
POINT 26214
FREQU 31446.54 Hz
SCANS 1024
ACQTM 0.8336 sec
PD 2.0000 sec
PW1 3.87 usec
IRNUC 1H
CTEMP 21.0 c
SLVNT CDCL3
EXREF 77.00 ppm
BF 0.30 Hz
RGAIN 28

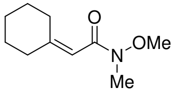

25

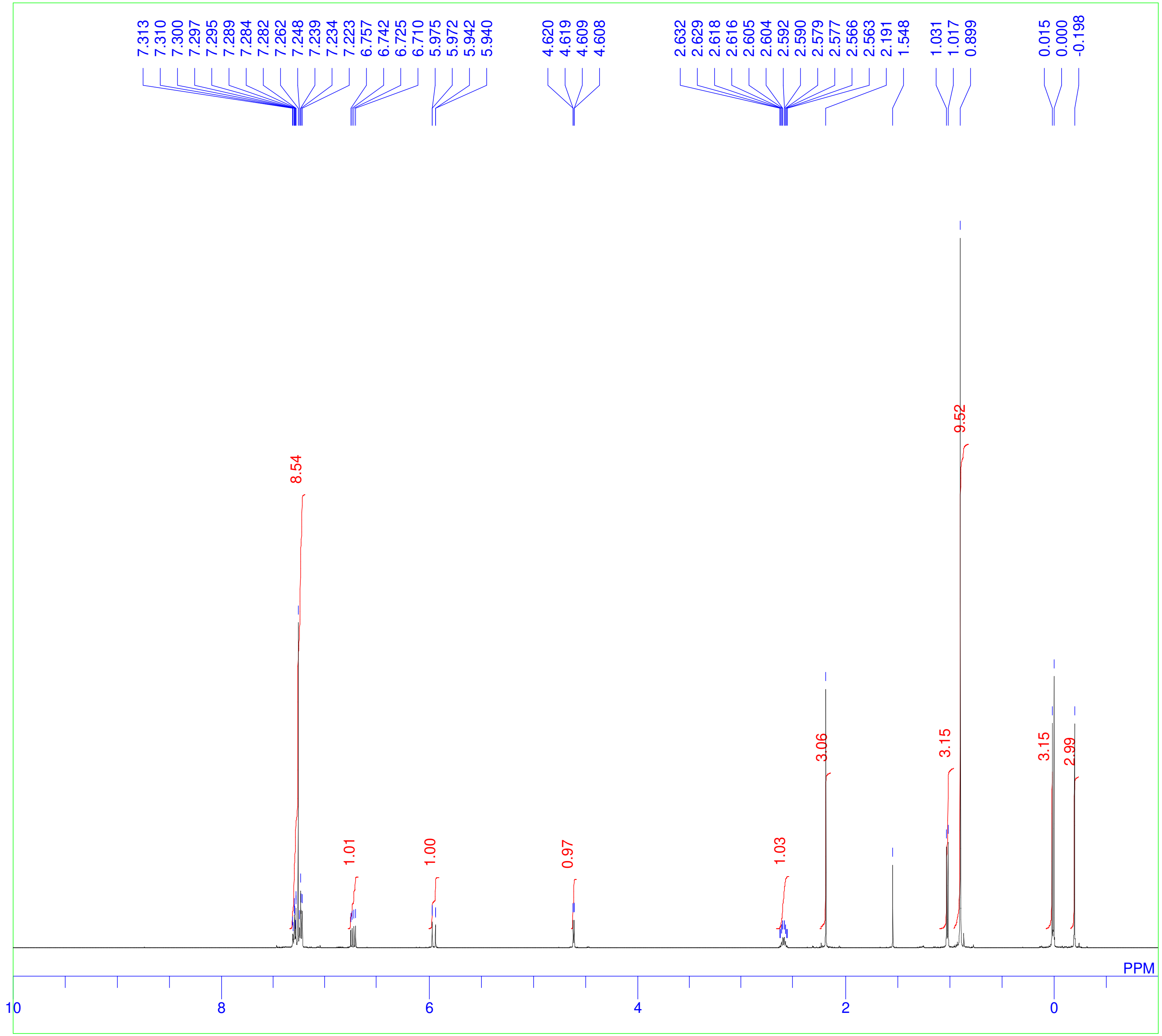

DFILE syn-26_1H.als
COMNT
DATIM 2023-02-26 16:44:53
OBNUC 1H
EXMOD proton.jxp
OBFRQ 500.16 MHz
OBSET 2.41 KHz
OBFIN 6.01 Hz
POINT 13107
FREQU 7507.51 Hz
SCANS 8
ACQTM 1.7459 sec
PD 5.0000 sec
PW1 3.84 usec
IRNUC 1H
CTEMP 21.0 c
SLVNT CDCL3
EXREF 0.00 ppm
BF 0.30 Hz
RGAIN 46

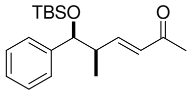

syn-**26**

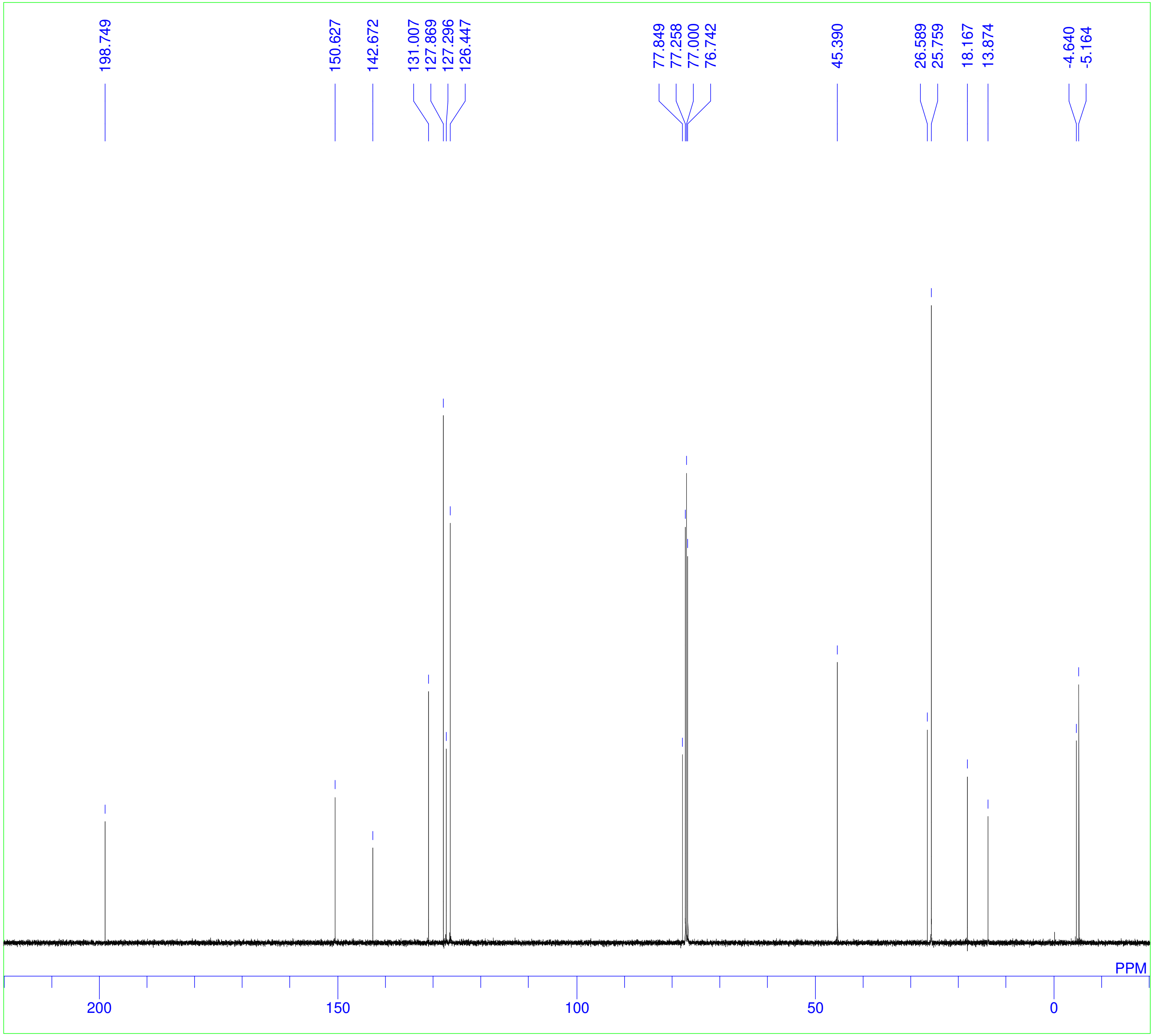

DFILE	syn-26_13C.als
COMNT	
DATIM	2023-02-26 17:53:18
OBNUC	13C
EXMOD	carbon.jxp
OBFRQ	125.77 MHz
OBSET	7.87 KHz
OBFIN	4.21 Hz
POINT	26214
FREQU	31446.54 Hz
SCANS	1024
ACQTM	0.8336 sec
PD	2.0000 sec
PW1	3.87 usec
IRNUC	1H
CTEMP	20.9 c
SLVNT	CDCL3
EXREF	77.00 ppm
BF	0.30 Hz
RGAIN	26

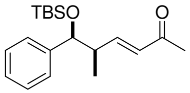

***syn*-26**

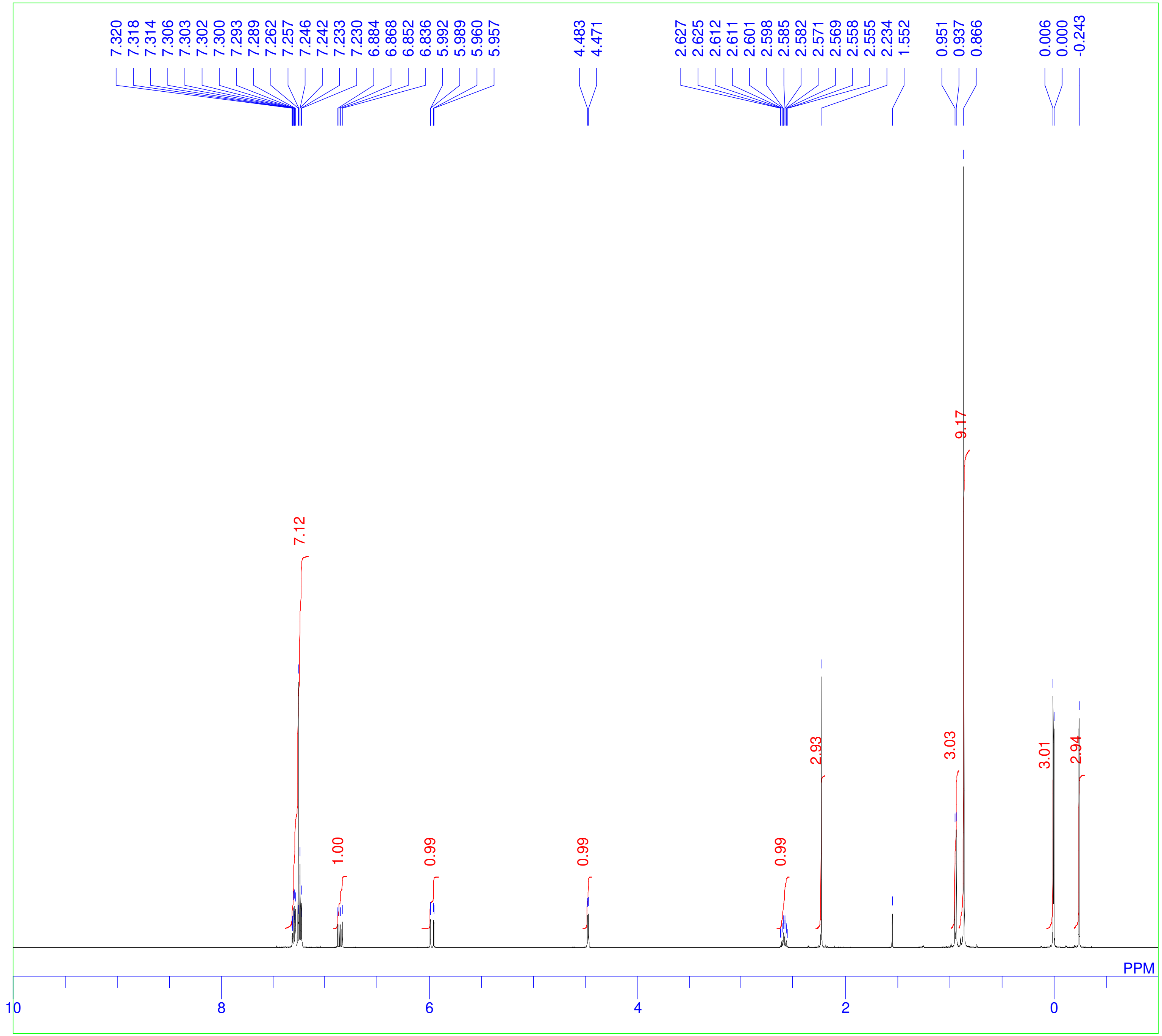

DFILE anti-26_1H.als
COMNT
DATIM 2023-02-26 16:51:58
OBNUC 1H
EXMOD proton.jxp
OBFRQ 500.16 MHz
OBSET 2.41 KHz
OBFIN 6.01 Hz
POINT 13107
FREQU 7507.51 Hz
SCANS 8
ACQTM 1.7459 sec
PD 5.0000 sec
PW1 3.84 usec
IRNUC 1H
CTEMP 21.0 c
SLVNT CDCL3
EXREF 0.00 ppm
BF 0.30 Hz
RGAIN 42

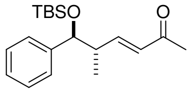

***anti*-26**

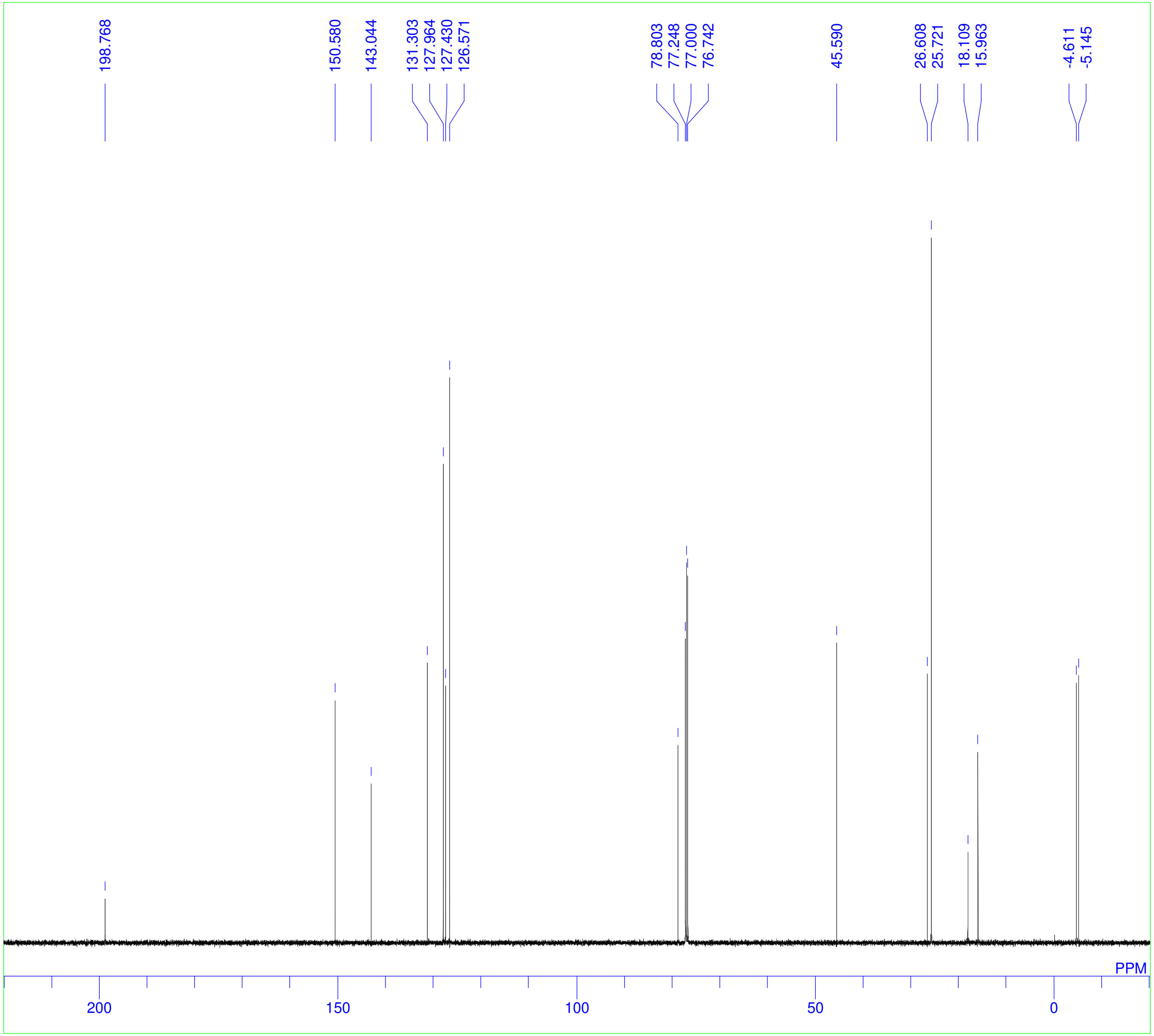

DFILE anti-26_13C.als
COMNT
DATIM 2023-02-26 18:47:13
OBNUC 13C
EXMOD carbon.jxp
OBFRQ 125.77 MHz
OBSET 7.87 KHz
OBFIN 4.21 Hz
POINT 26214
FREQU 31446.54 Hz
SCANS 1024
ACQTM 0.8336 sec
PD 2.0000 sec
PW1 3.87 usec
IRNUC 1H
CTEMP 20.8 c
SLVNT CDCL3
EXREF 77.00 ppm
BF 0.30 Hz
RGAIN 28

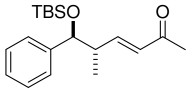

***anti*-26**

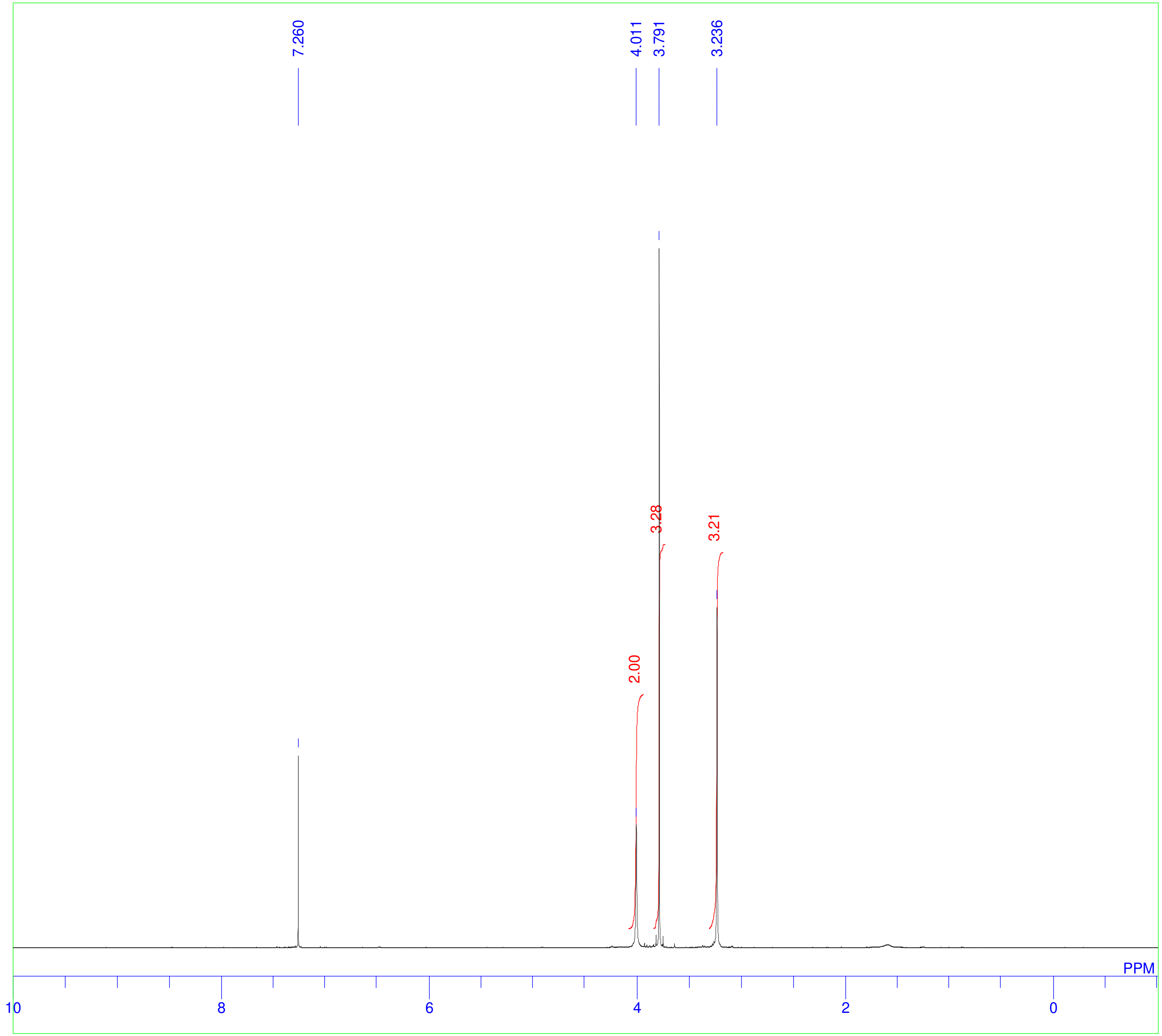

DFILE S1_1H.als
COMNT
DATIM 2021-11-30 21:40:02
OBNUC 1H
EXMOD proton.jxp
OBFRQ 500.16 MHz
OBSET 2.41 KHz
OBFIN 6.01 Hz
POINT 13107
FREQU 7507.51 Hz
SCANS 8
ACQTM 1.7459 sec
PD 5.0000 sec
PW1 3.84 usec
IRNUC 1H
CTEMP 23.1 c
SLVNT CDCL3
EXREF 7.26 ppm
BF 0.30 Hz
RGAIN 50

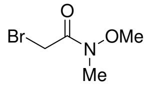

S1

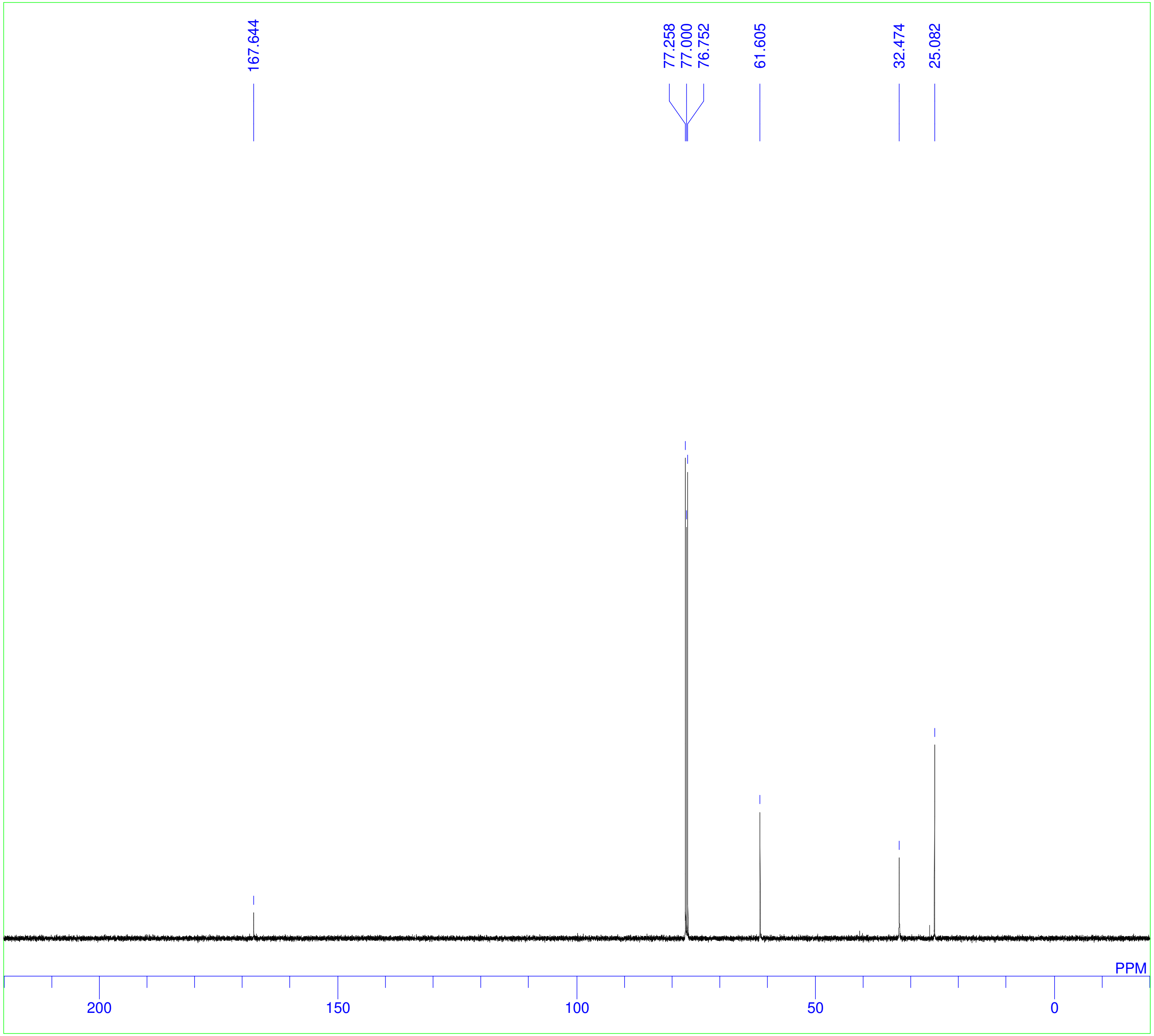

DFILE	S1_13C.als
COMNT	
DATIM	2022-02-27 14:47:31
OBNUC	13C
EXMOD	carbon.jxp
OBFRQ	125.77 MHz
OBSET	7.87 KHz
OBFIN	4.21 Hz
POINT	26214
FREQU	31446.54 Hz
SCANS	1024
ACQTM	0.8336 sec
PD	2.0000 sec
PW1	3.87 usec
IRNUC	1H
CTEMP	20.9 c
SLVNT	CDCL3
EXREF	77.00 ppm
BF	0.30 Hz
RGAIN	26

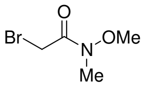

S1

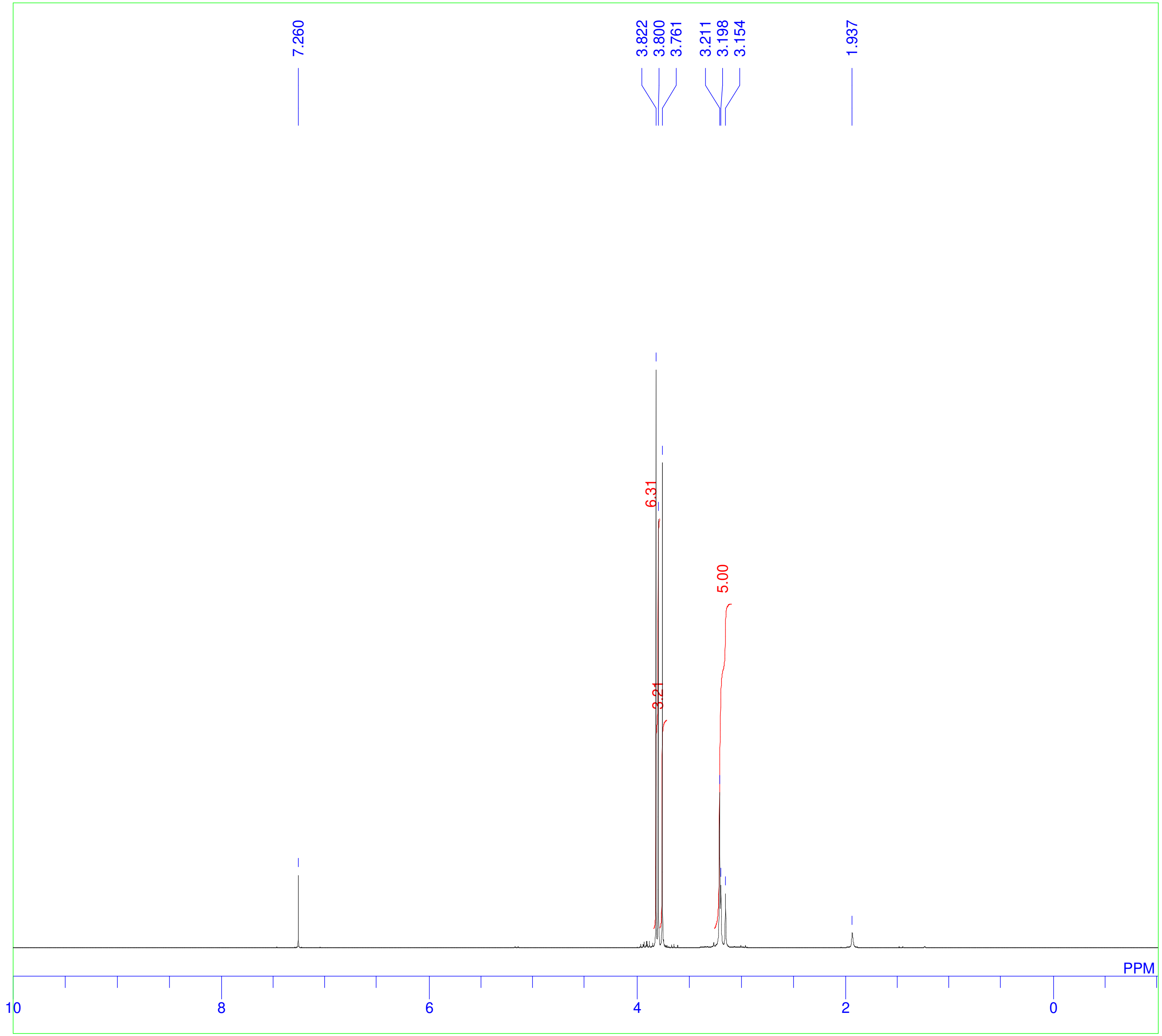

DFILE	S2_1H.als
COMNT	
DATIM	2021-12-13 08:06:50
OBNUC	1H
EXMOD	proton.jxp
OBFRQ	500.16 MHz
OBSET	2.41 KHz
OBFIN	6.01 Hz
POINT	13107
FREQU	7507.51 Hz
SCANS	8
ACQTM	1.7459 sec
PD	5.0000 sec
PW1	3.84 usec
IRNUC	1H
CTEMP	22.0 c
SLVNT	CDCL3
EXREF	7.26 ppm
BF	0.30 Hz
RGAIN	42

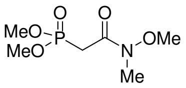

S2

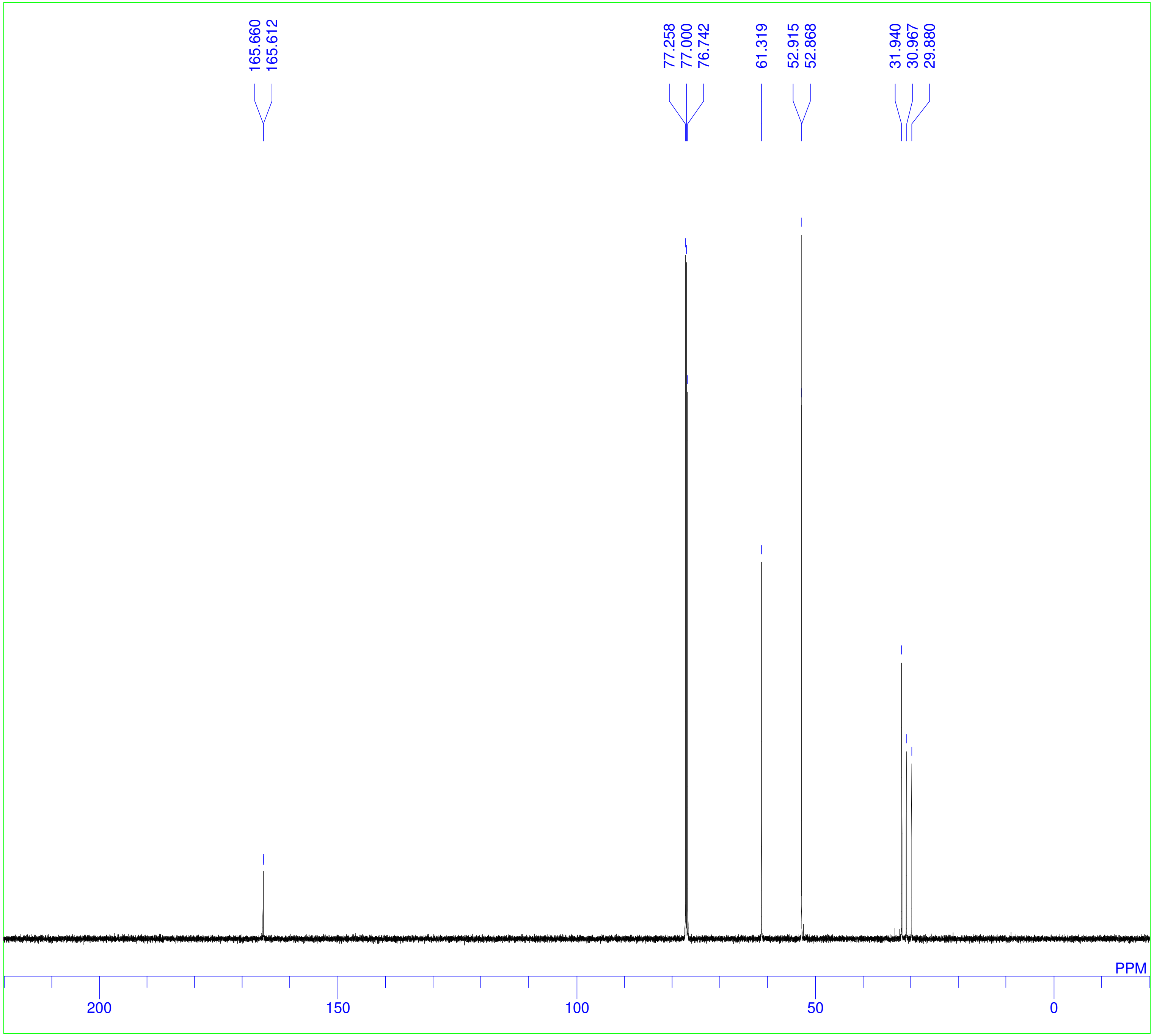

DFILE S2_13C.als
COMNT
DATIM 2022-12-22 13:05:52
OBNUC 13C
EXMOD carbon.jxp
OBFRQ 125.77 MHz
OBSET 7.87 KHz
OBFIN 4.21 Hz
POINT 26214
FREQU 31446.54 Hz
SCANS 1024
ACQTM 0.8336 sec
PD 2.0000 sec
PW1 3.87 usec
IRNUC 1H
CTEMP 22.8 c
SLVNT CDCL3
EXREF 77.00 ppm
BF 0.30 Hz
RGAIN 28

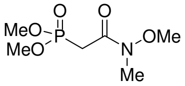

S2

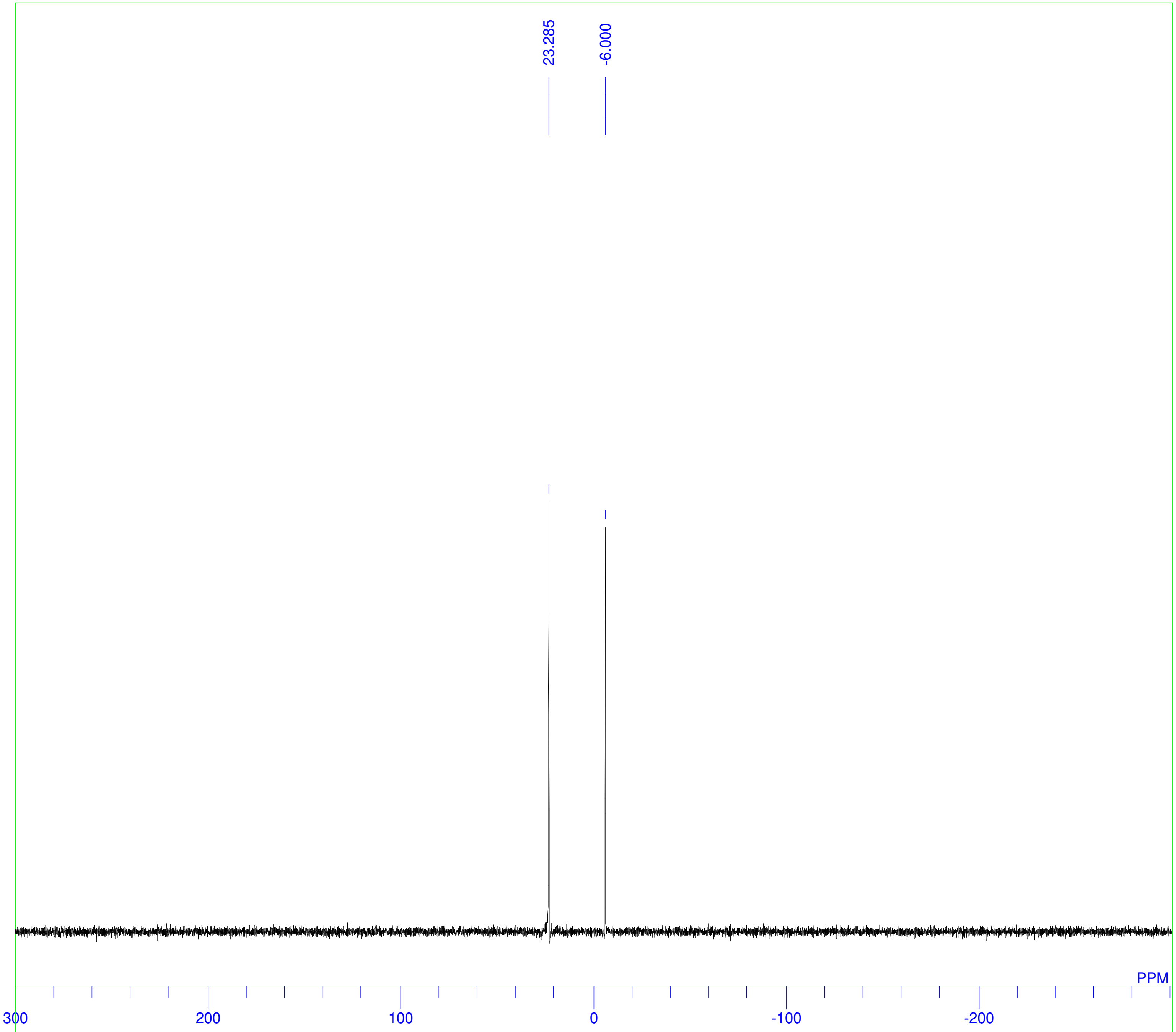

DFILE	S2_31P.als
COMNT	
DATIM	2023-04-03 14:15:15
OBNUC	31P
EXMOD	single_pulse.jxp
OBFRQ	202.46 MHz
OBSET	8.31 KHz
OBFIN	0.75 Hz
POINT	13107
FREQU	142857.14 Hz
SCANS	64
ACQTM	0.0918 sec
PD	5.0000 sec
PW1	6.45 usec
IRNUC	31P
CTEMP	23.4 c
SLVNT	CDCL3
EXREF	-6.00 ppm
BF	0.30 Hz
RGAIN	50

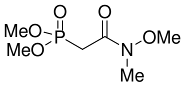

S2

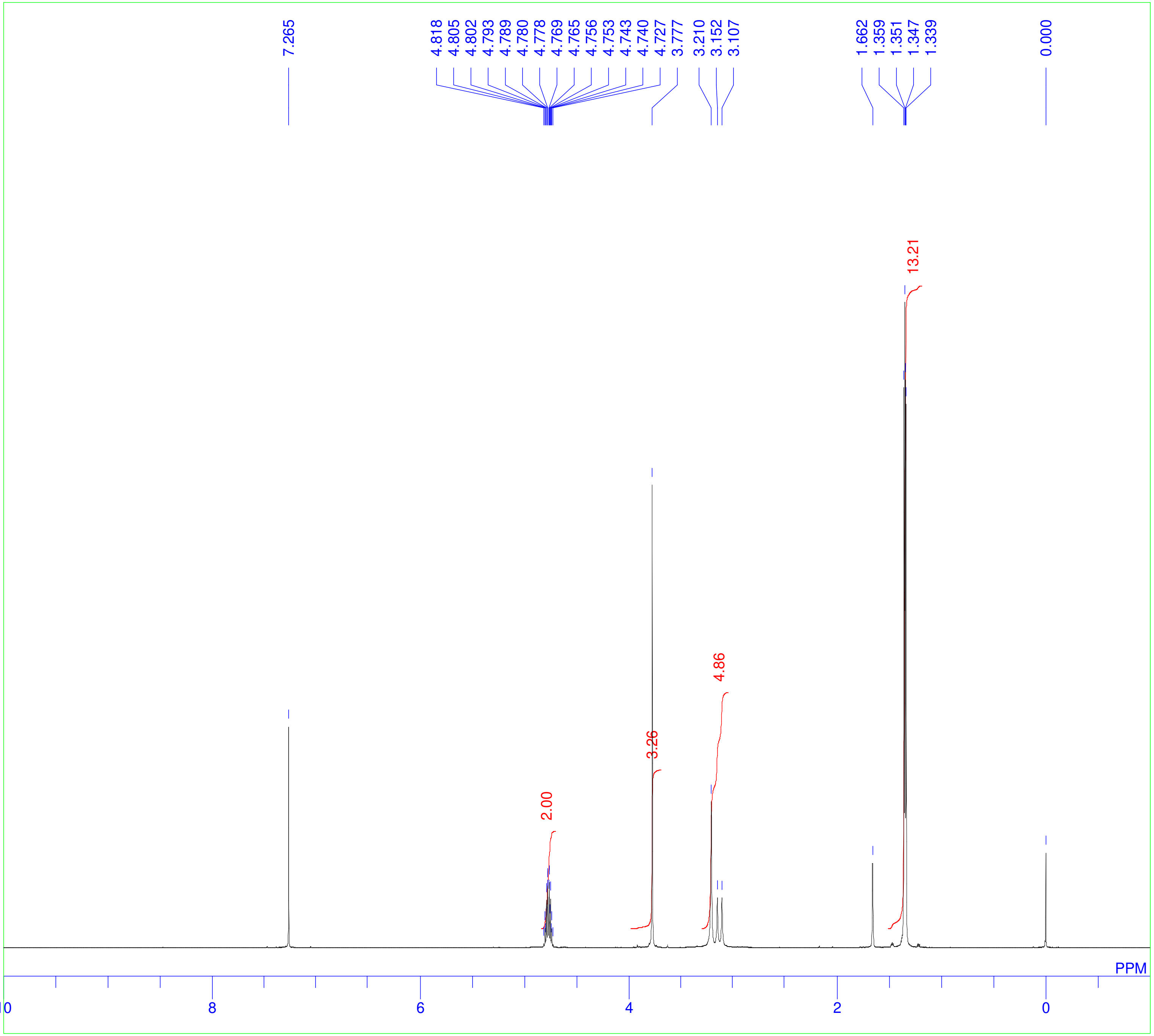

DFILE	S3_1H.als
COMNT	
DATIM	2023-11-05 13:54:49
OBNUC	1H
EXMOD	proton.jxp
OBFRQ	500.16 MHz
OBSET	2.41 KHz
OBFIN	6.01 Hz
POINT	13107
FREQU	7507.51 Hz
SCANS	8
ACQTM	1.7459 sec
PD	5.0000 sec
PW1	3.80 usec
IRNUC	1H
CTEMP	23.5 c
SLVNT	CDCL3
EXREF	0.00 ppm
BF	0.30 Hz
RGAIN	40

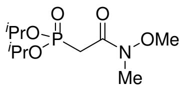

S3

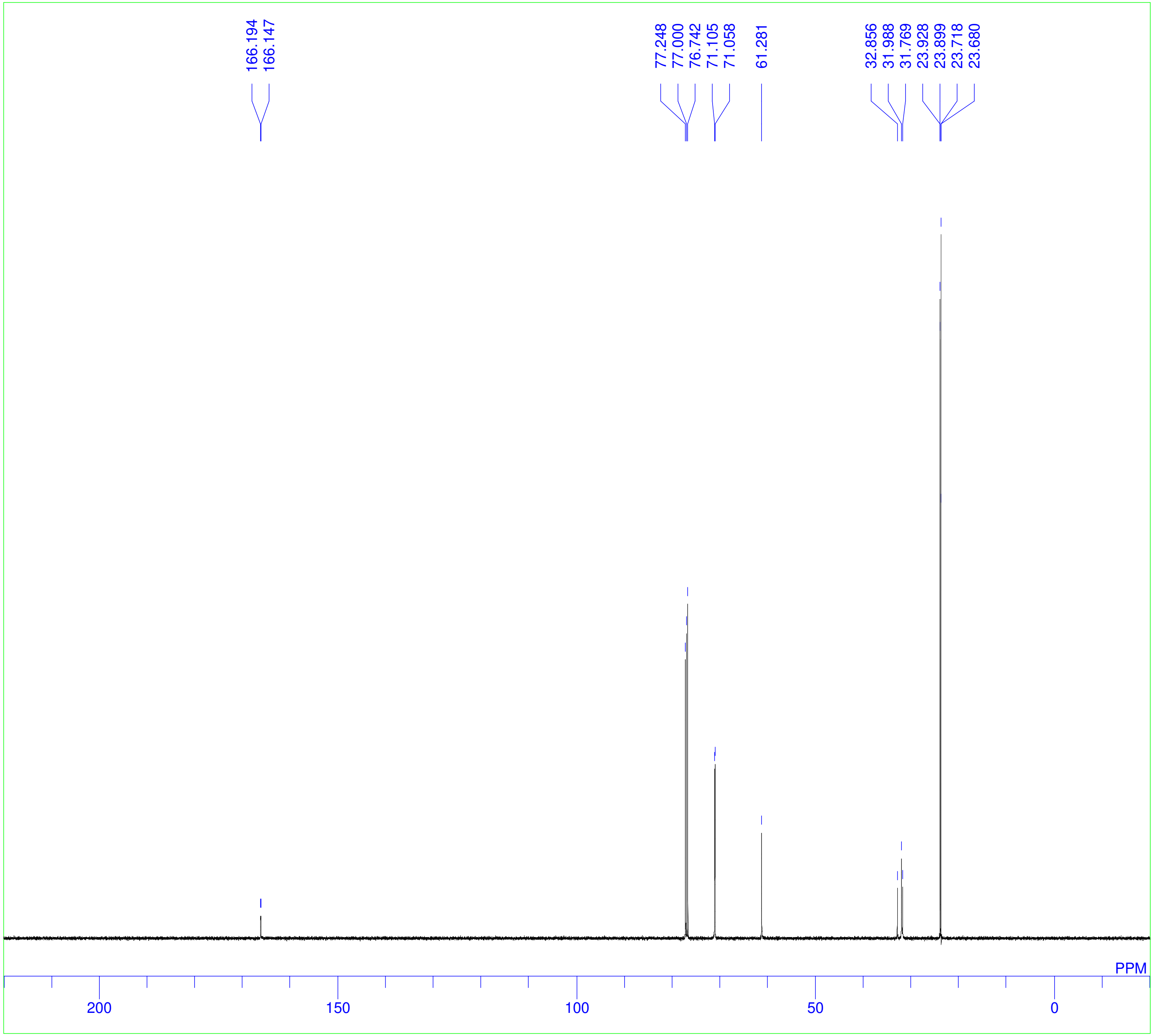

DFILE	S3_13C.als
COMNT	
DATIM	2022-12-25 14:27:24
OBNUC	13C
EXMOD	carbon.jxp
OBFRQ	125.77 MHz
OBSET	7.87 KHz
OBFIN	4.21 Hz
POINT	26214
FREQU	31446.54 Hz
SCANS	1024
ACQTM	0.8336 sec
PD	2.0000 sec
PW1	3.87 usec
IRNUC	1H
CTEMP	22.2 c
SLVNT	CDCL3
EXREF	77.00 ppm
BF	0.25 Hz
RGAIN	26

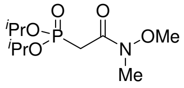

S3

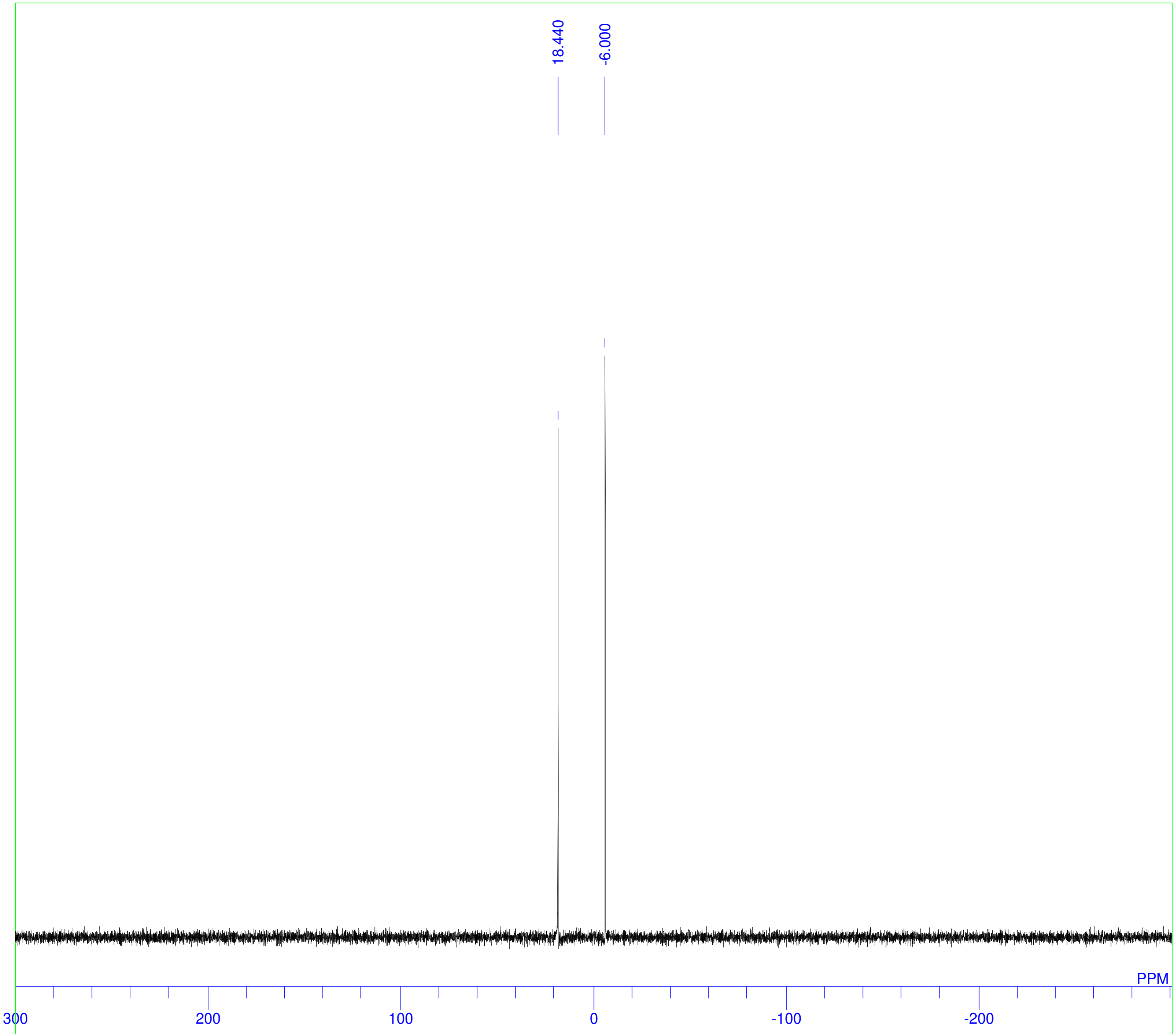

DFILE	S3_31P.als
COMNT	
DATIM	2023-04-03 14:26:03
OBNUC	31P
EXMOD	single_pulse.jxp
OBFRQ	202.46 MHz
OBSET	8.31 KHz
OBFIN	0.75 Hz
POINT	13107
FREQU	142857.14 Hz
SCANS	64
ACQTM	0.0918 sec
PD	5.0000 sec
PW1	6.45 usec
IRNUC	31P
CTEMP	23.8 c
SLVNT	CDCL3
EXREF	-6.00 ppm
BF	0.25 Hz
RGAIN	50

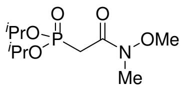

S3

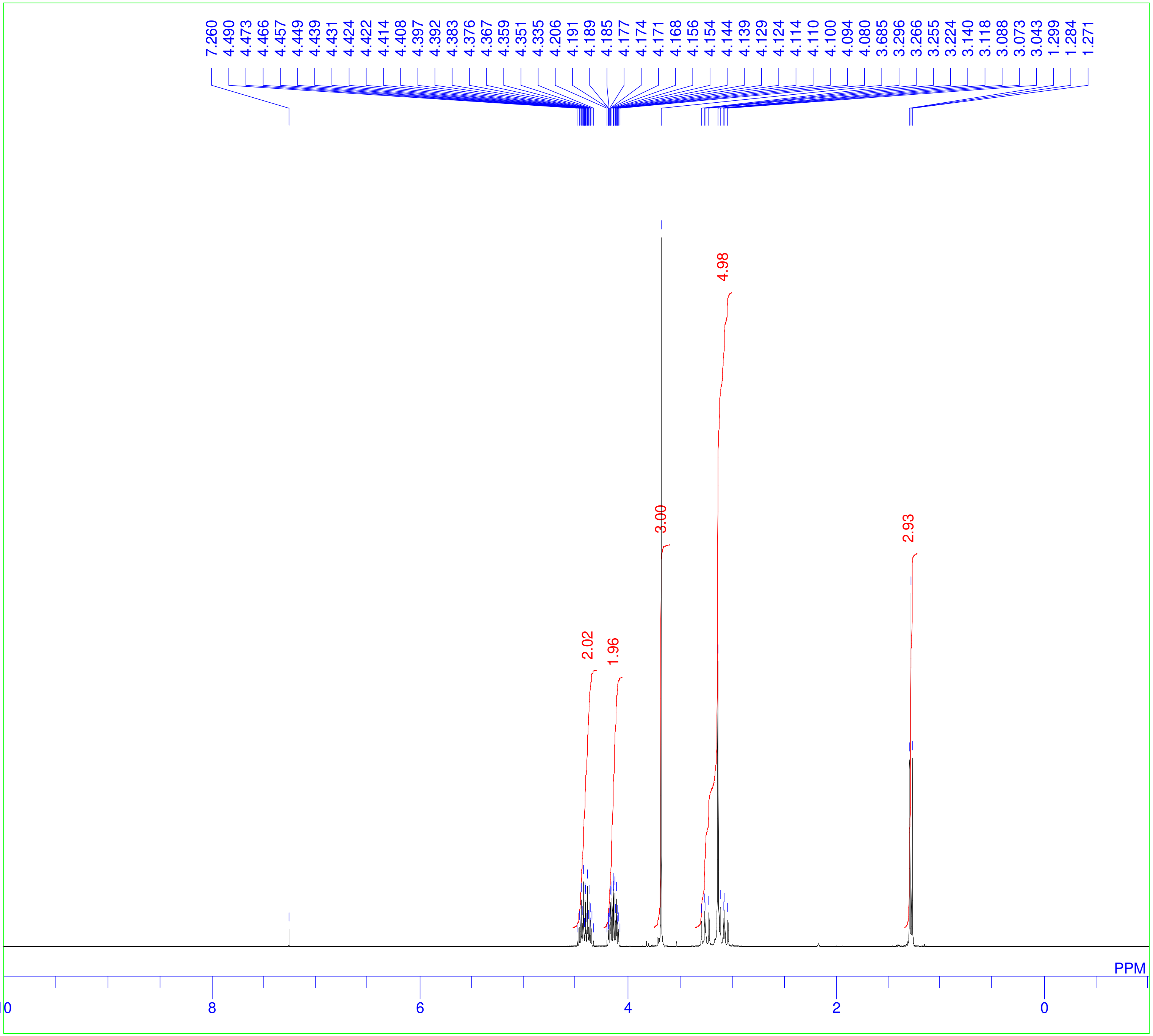

DFILE	S4_1H.als
COMNT	
DATIM	2022-12-27 16:49:17
OBNUC	1H
EXMOD	proton.jxp
OBFRQ	500.16 MHz
OBSET	2.41 KHz
OBFIN	6.01 Hz
POINT	13107
FREQU	7507.51 Hz
SCANS	8
ACQTM	1.7459 sec
PD	5.0000 sec
PW1	3.84 usec
IRNUC	1H
CTEMP	22.1 c
SLVNT	CDCL3
EXREF	7.26 ppm
BF	0.30 Hz
RGAIN	20

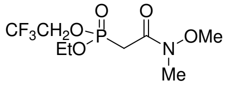

S4

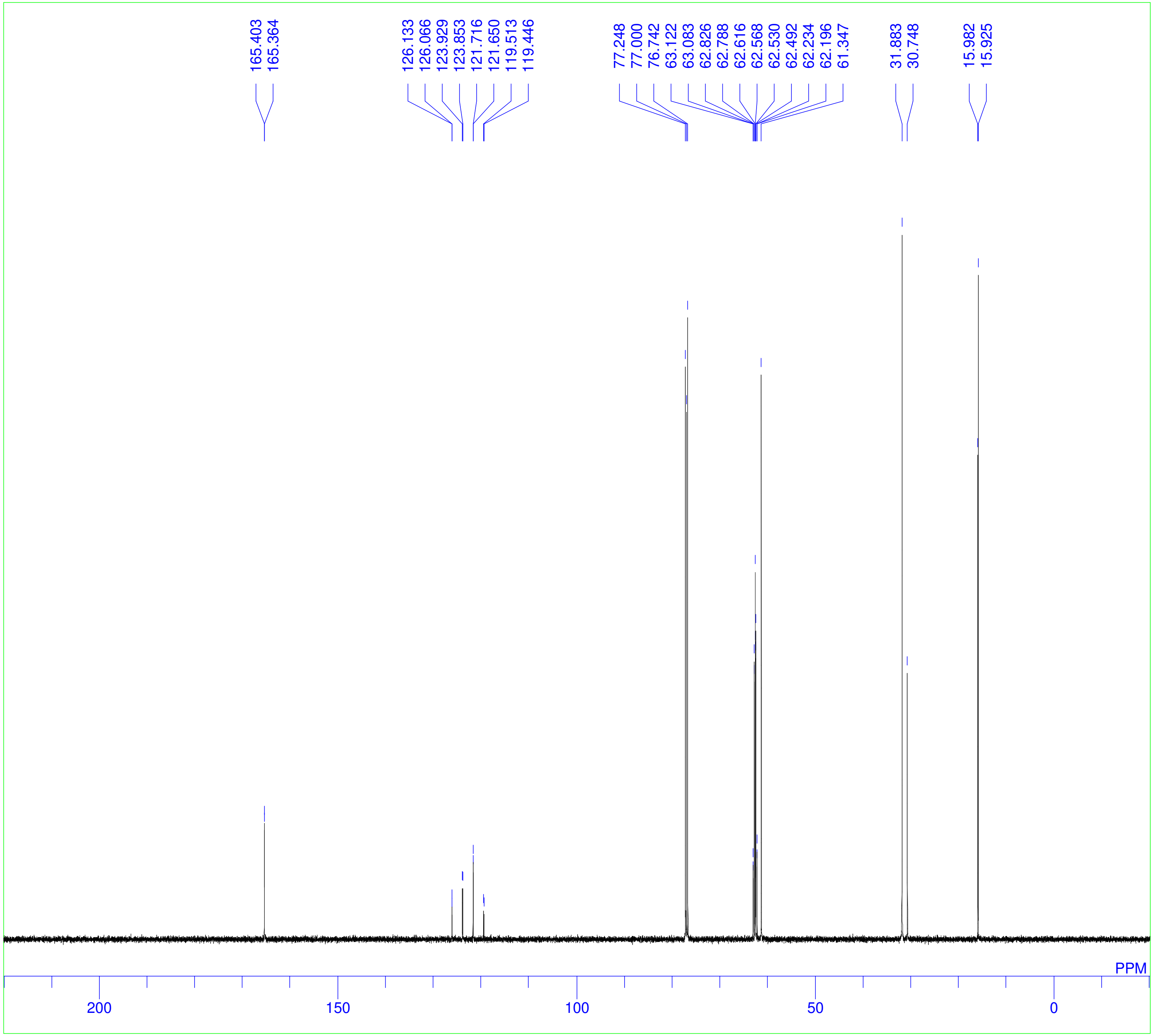

DFILE	S4_13C.als
COMNT	
DATIM	2022-12-27 15:59:34
OBNUC	13C
EXMOD	carbon.jxp
OBFRQ	125.77 MHz
OBSET	7.87 KHz
OBFIN	4.21 Hz
POINT	26214
FREQU	31446.54 Hz
SCANS	1024
ACQTM	0.8336 sec
PD	2.0000 sec
PW1	3.87 usec
IRNUC	1H
CTEMP	22.2 c
SLVNT	CDCL3
EXREF	77.00 ppm
BF	0.30 Hz
RGAIN	24

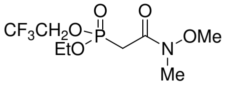

S4

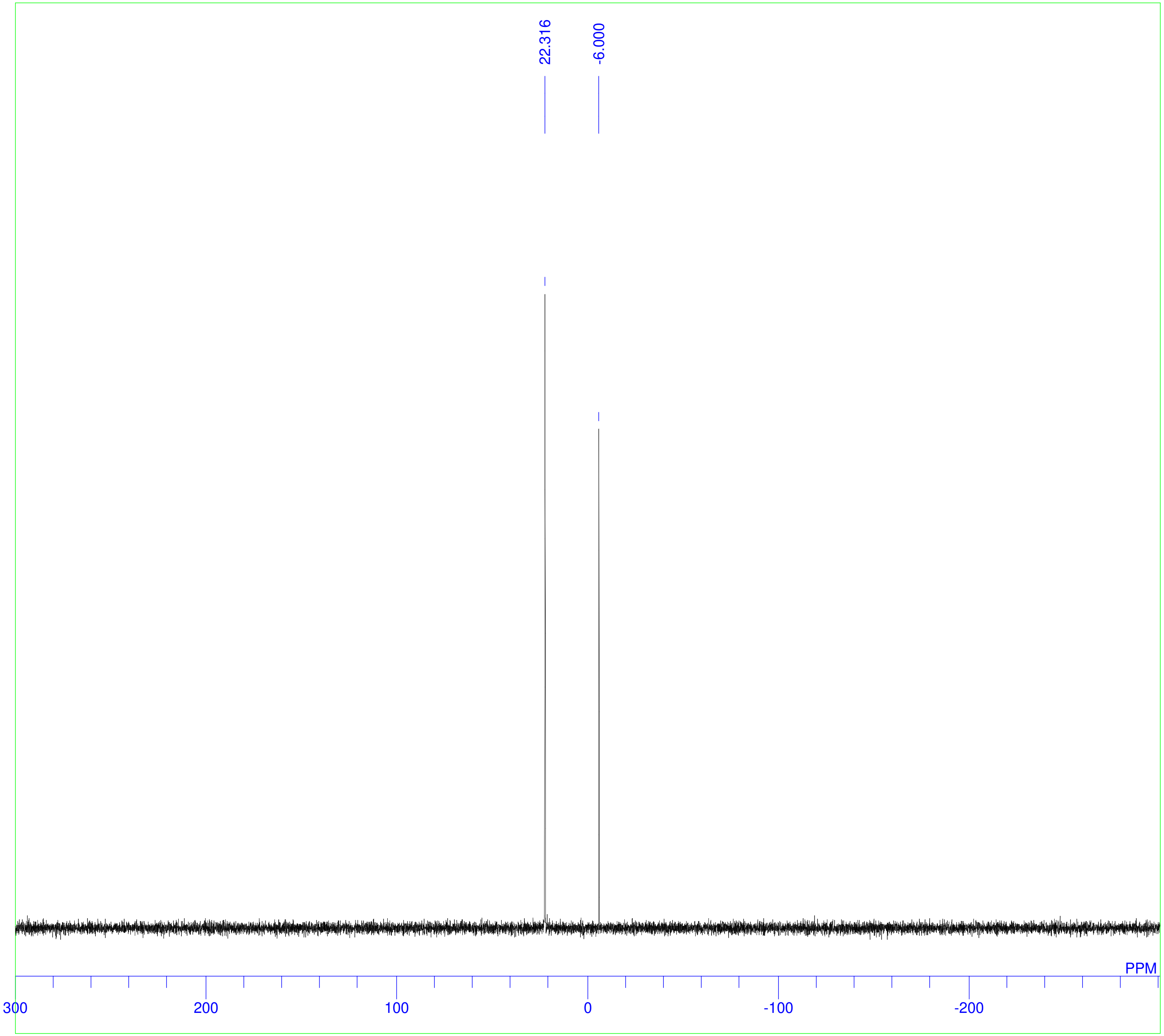

DFILE	S4_31P.als
COMNT	
DATIM	2023-12-28 19:28:29
OBNUC	31P
EXMOD	single_pulse.jxp
OBFRQ	202.46 MHz
OBSET	8.31 KHz
OBFIN	0.75 Hz
POINT	13107
FREQU	142857.14 Hz
SCANS	64
ACQTM	0.0918 sec
PD	5.0000 sec
PW1	6.45 usec
IRNUC	31P
CTEMP	22.1 c
SLVNT	CDCL3
EXREF	-6.00 ppm
BF	0.30 Hz
RGAIN	50

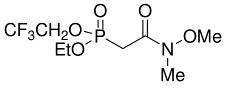

S4

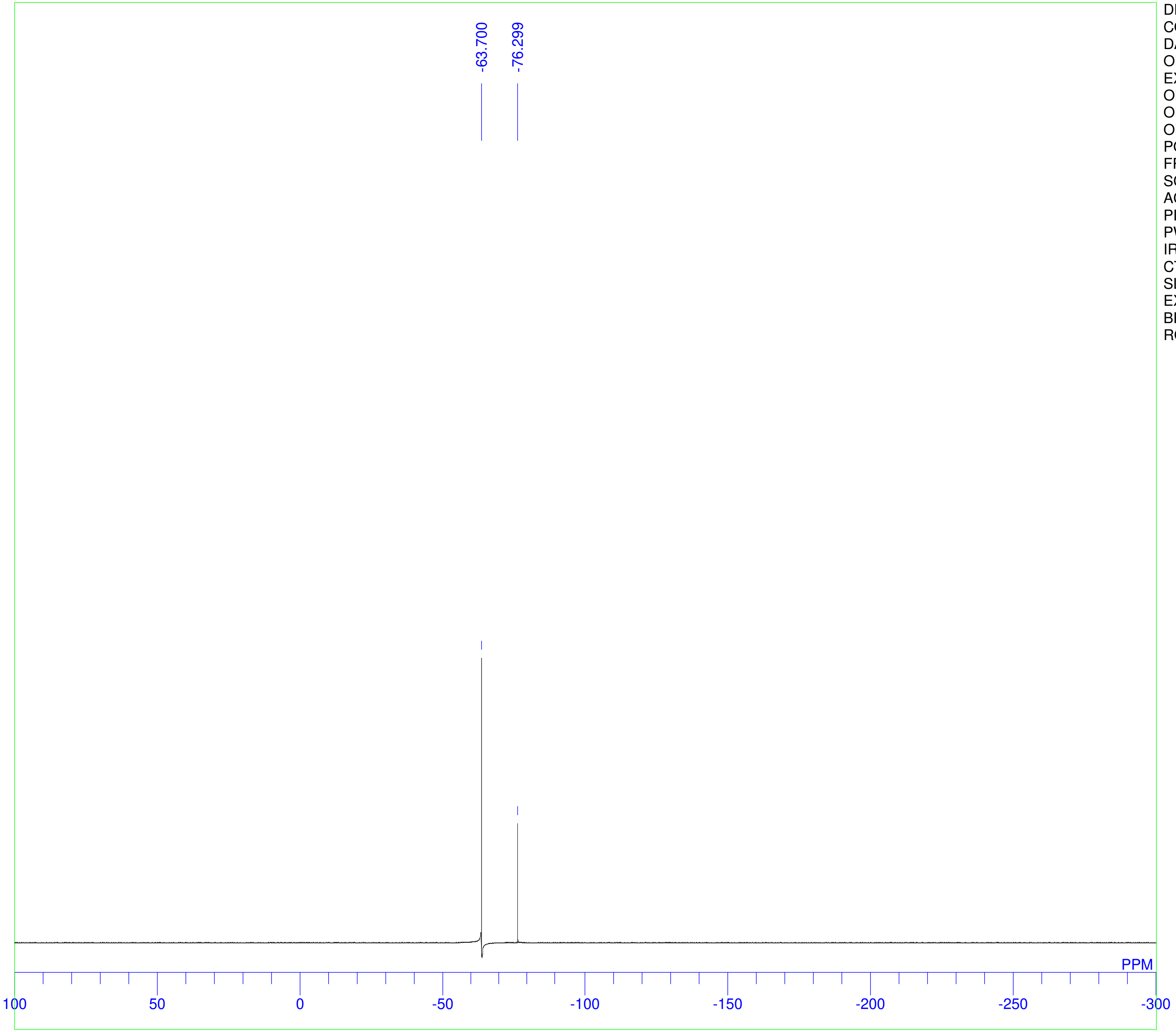

DFILE	S4_19F.als
COMNT	
DATIM	2024-02-11 15:56:25
OBNUC	19F
EXMOD	single_pulse.jxp
OBFRQ	470.62 MHz
OBSET	0.46 KHz
OBFIN	0.84 Hz
POINT	13107
FREQU	285714.28 Hz
SCANS	8
ACQTM	0.0459 sec
PD	5.0000 sec
PW1	4.25 usec
IRNUC	19F
CTEMP	21.4 c
SLVNT	CDCL3
EXREF	-63.70 ppm
BF	0.12 Hz
RGAIN	50

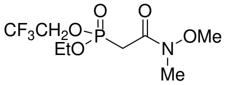

S4

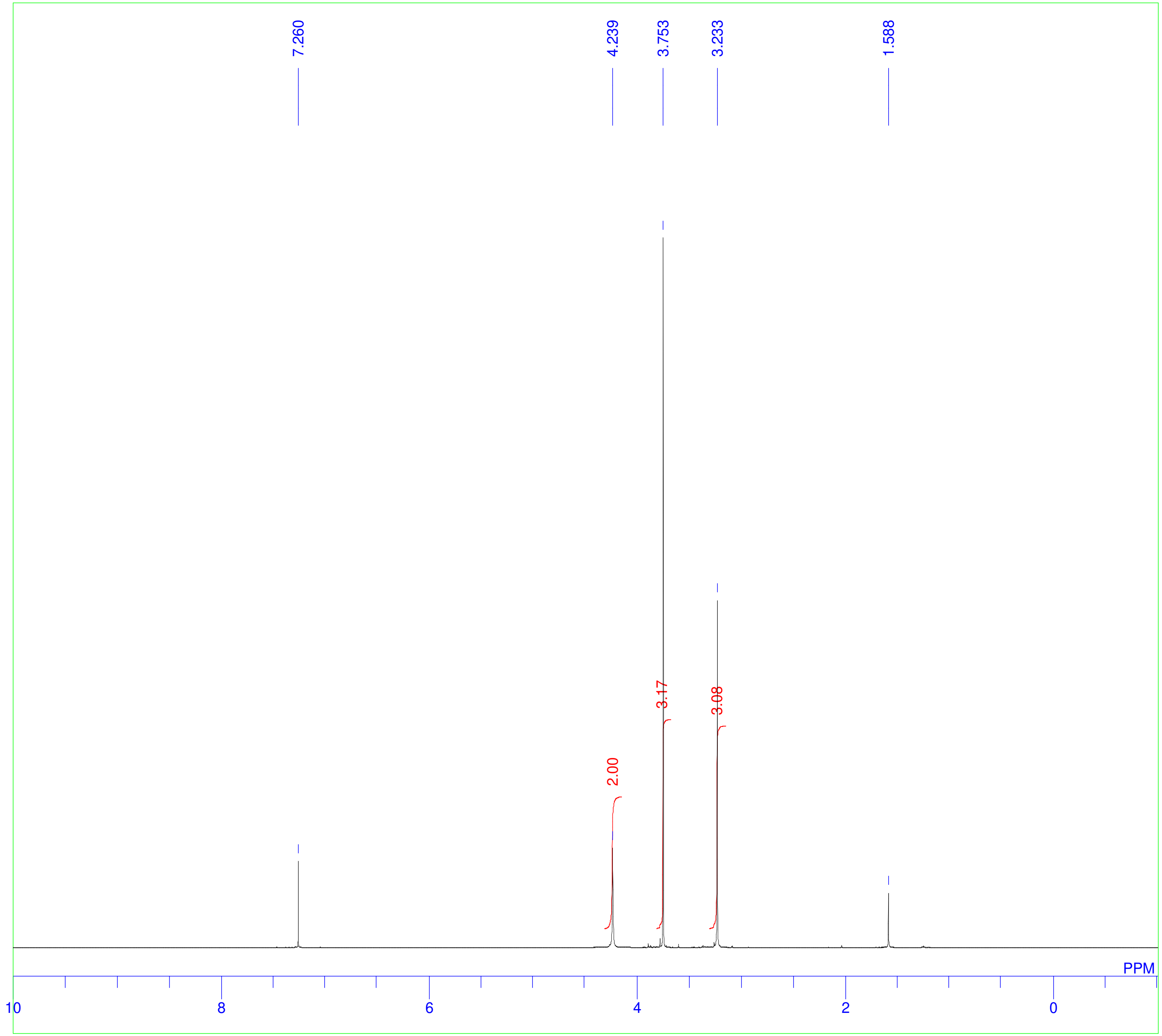

DFILE	S5_1H.als
COMNT	
DATIM	2022-08-10 18:25:12
OBNUC	1H
EXMOD	proton.jxp
OBFRQ	500.16 MHz
OBSET	2.41 KHz
OBFIN	6.01 Hz
POINT	13107
FREQU	7507.51 Hz
SCANS	8
ACQTM	1.7459 sec
PD	5.0000 sec
PW1	3.84 usec
IRNUC	1H
CTEMP	28.1 c
SLVNT	CDCL3
EXREF	7.26 ppm
BF	0.30 Hz
RGAIN	48

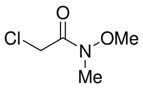

S5

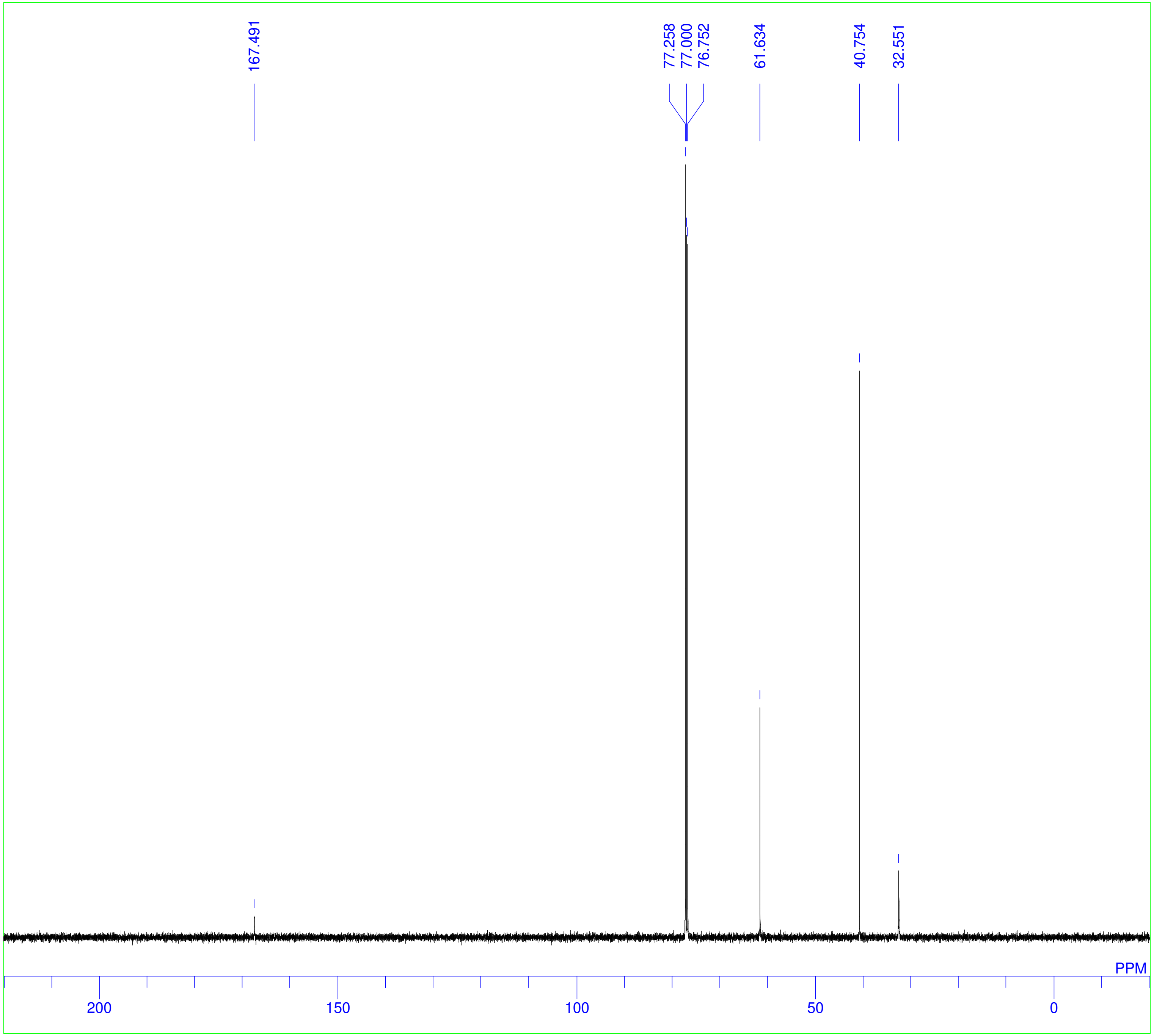

DFILE	S5_13C.als
COMNT	
DATIM	2022-12-29 15:18:07
OBNUC	13C
EXMOD	carbon.jxp
OBFRQ	125.77 MHz
OBSET	7.87 KHz
OBFIN	4.21 Hz
POINT	26214
FREQU	31446.54 Hz
SCANS	1024
ACQTM	0.8336 sec
PD	2.0000 sec
PW1	3.87 usec
IRNUC	1H
CTEMP	22.6 c
SLVNT	CDCL3
EXREF	77.00 ppm
BF	0.30 Hz
RGAIN	22

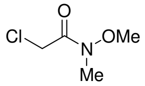

S5

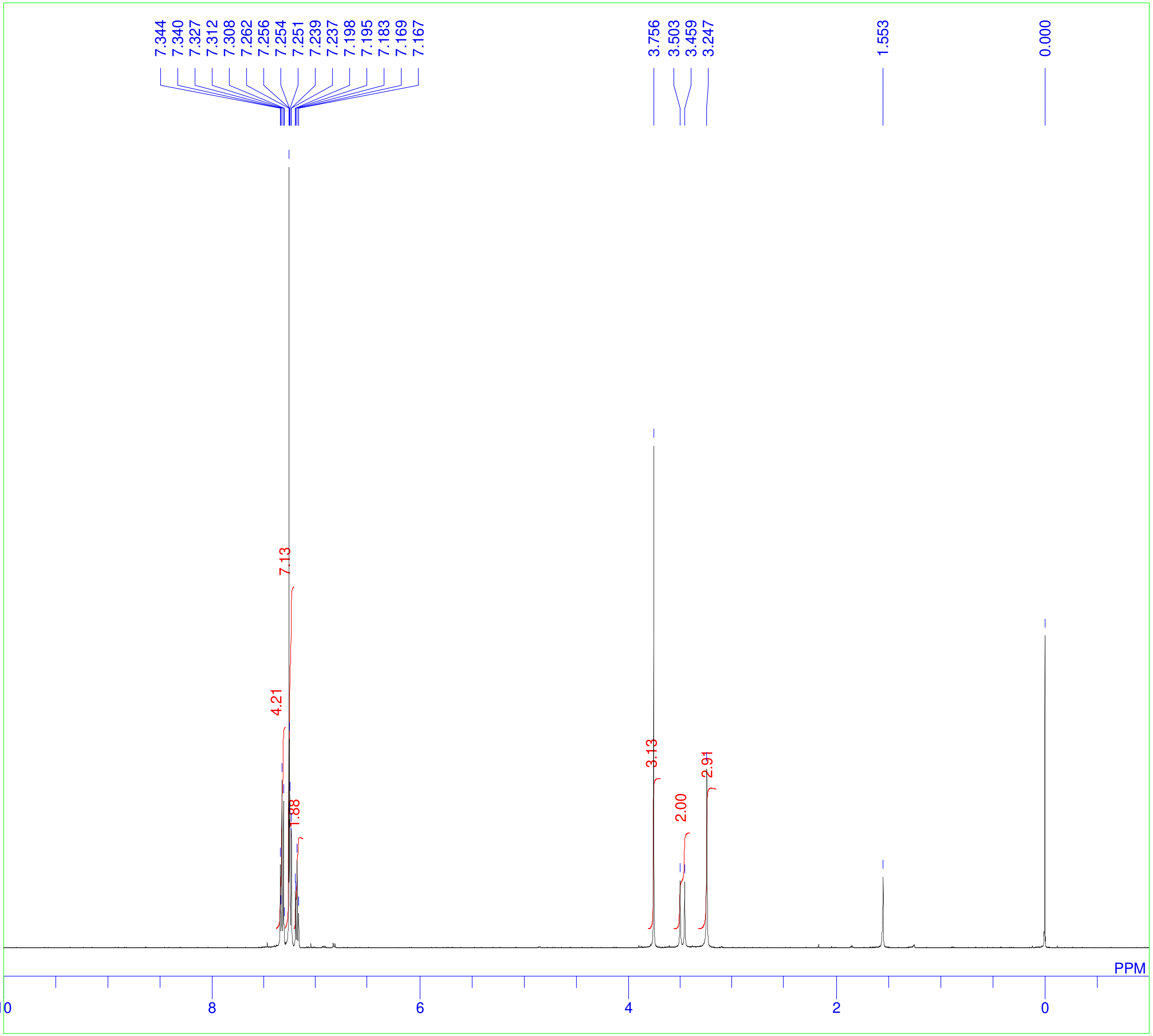

DFILE	S6_1H.als
COMNT	
DATIM	2023-02-23 18:31:03
OBNUC	1H
EXMOD	proton.jxp
OBFRQ	500.16 MHz
OBSET	2.41 KHz
OBFIN	6.01 Hz
POINT	13107
FREQU	7507.51 Hz
SCANS	8
ACQTM	1.7459 sec
PD	5.0000 sec
PW1	3.84 usec
IRNUC	1H
CTEMP	22.6 c
SLVNT	CDCL3
EXREF	0.00 ppm
BF	0.30 Hz
RGAIN	46

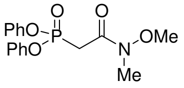

S6

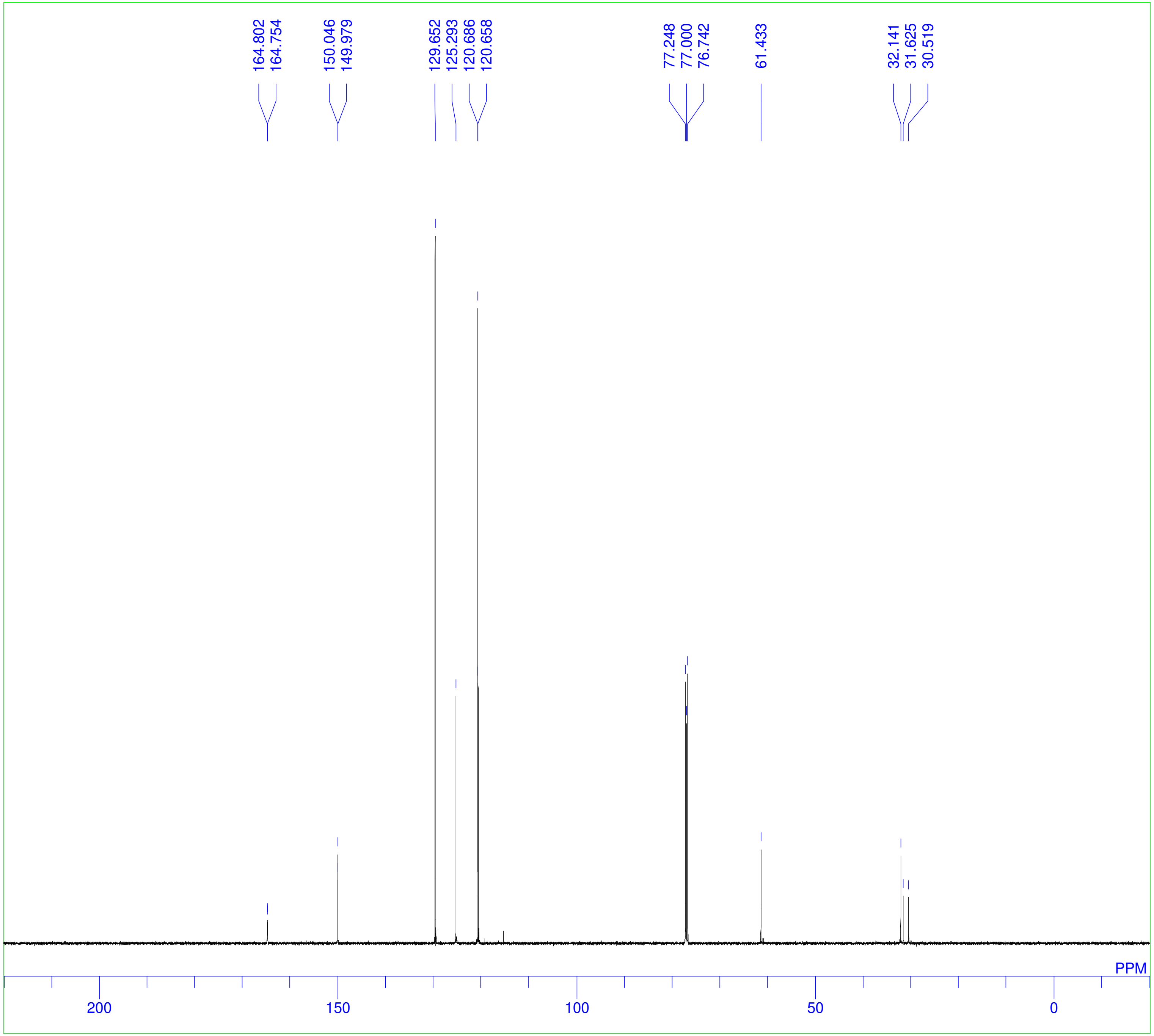

DFILE	S6_13C.als
COMNT	
DATIM	2022-12-26 13:52:18
OBNUC	13C
EXMOD	carbon.jxp
OBFRQ	125.77 MHz
OBSET	7.87 KHz
OBFIN	4.21 Hz
POINT	26214
FREQU	31446.54 Hz
SCANS	1024
ACQTM	0.8336 sec
PD	2.0000 sec
PW1	3.87 usec
IRNUC	1H
CTEMP	22.1 c
SLVNT	CDCL3
EXREF	77.00 ppm
BF	0.30 Hz
RGAIN	26

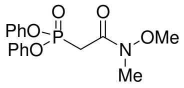

S6

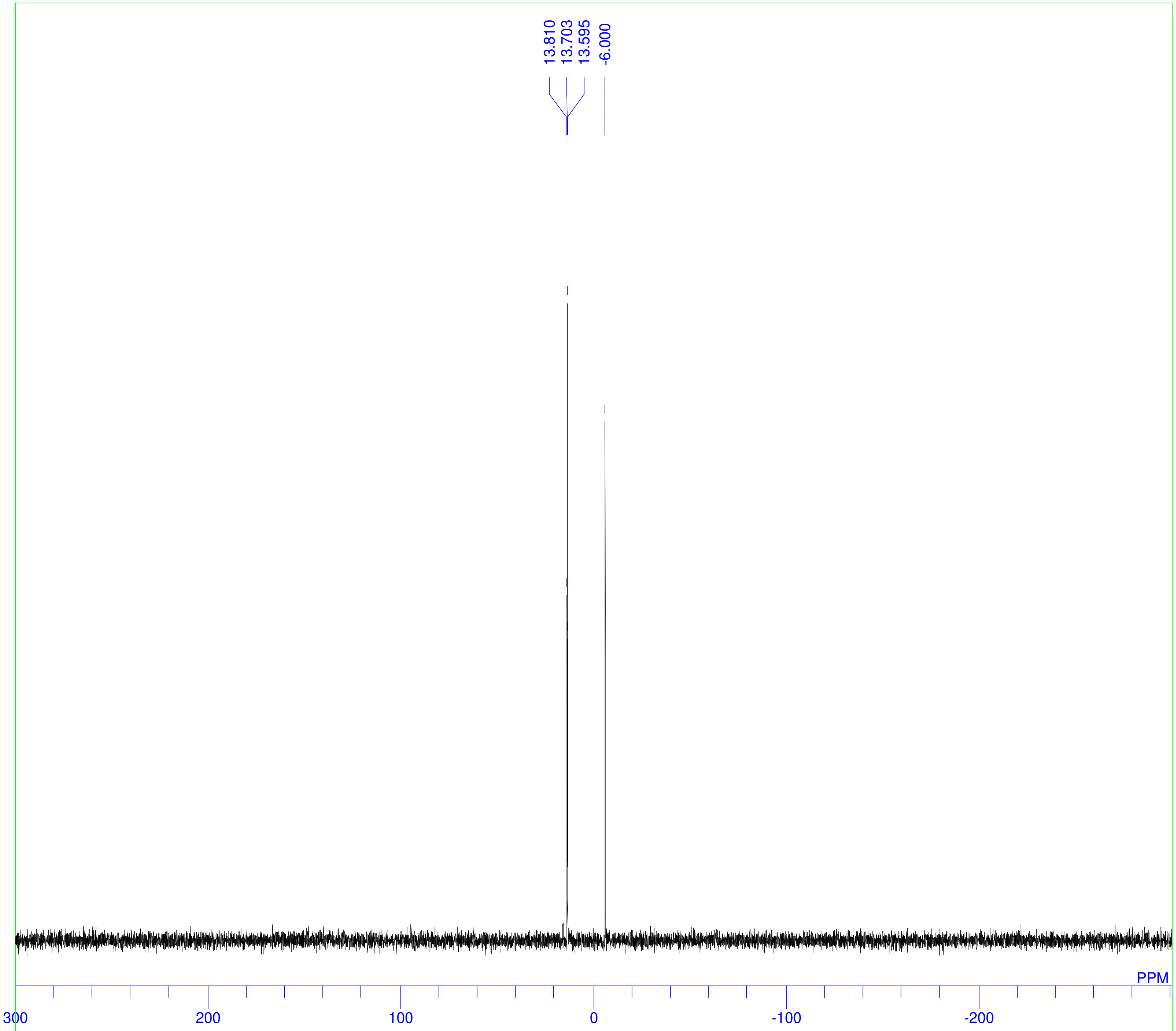

DFILE	S6_31P.als
COMNT	
DATIM	2023-11-05 14:22:12
OBNUC	31P
EXMOD	single_pulse.jxp
OBFRQ	202.46 MHz
OBSET	8.31 KHz
OBFIN	0.75 Hz
POINT	13107
FREQU	142857.14 Hz
SCANS	32
ACQTM	0.0918 sec
PD	5.0000 sec
PW1	6.45 usec
IRNUC	31P
CTEMP	23.5 c
SLVNT	CDCL3
EXREF	-6.00 ppm
BF	0.30 Hz
RGAIN	50

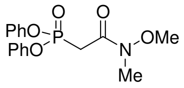

S6

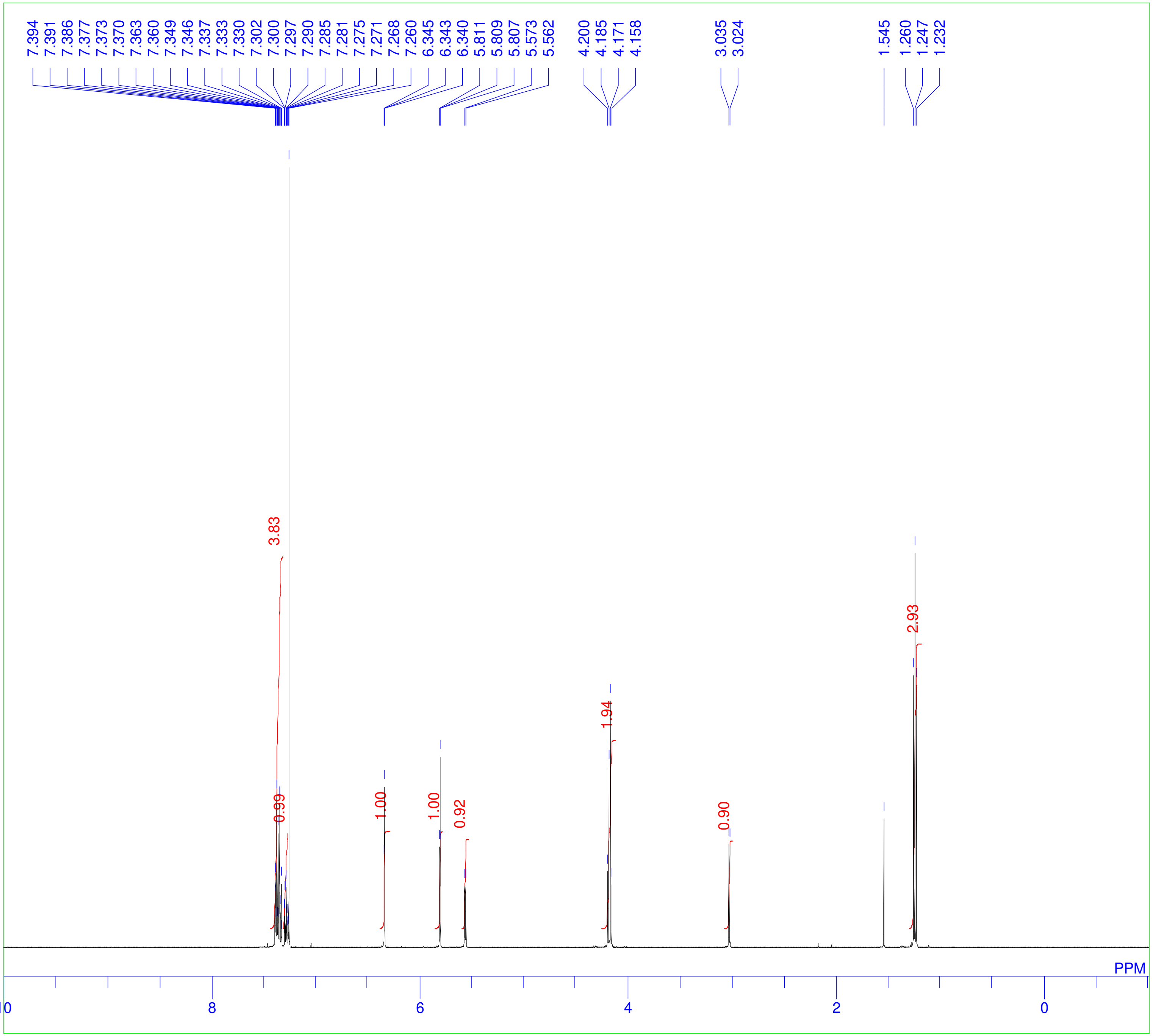

DFILE S7_1H.als
COMNT
DATIM 2023-01-12 21:56:48
OBNUC 1H
EXMOD proton.jxp
OBFRQ 500.16 MHz
OBSET 2.41 KHz
OBFIN 6.01 Hz
POINT 13107
FREQU 7507.51 Hz
SCANS 8
ACQTM 1.7459 sec
PD 5.0000 sec
PW1 3.84 usec
IRNUC 1H
CTEMP 22.1 c
SLVNT CDCL3
EXREF 7.26 ppm
BF 0.12 Hz
RGAIN 48

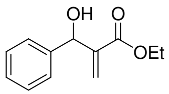

S7

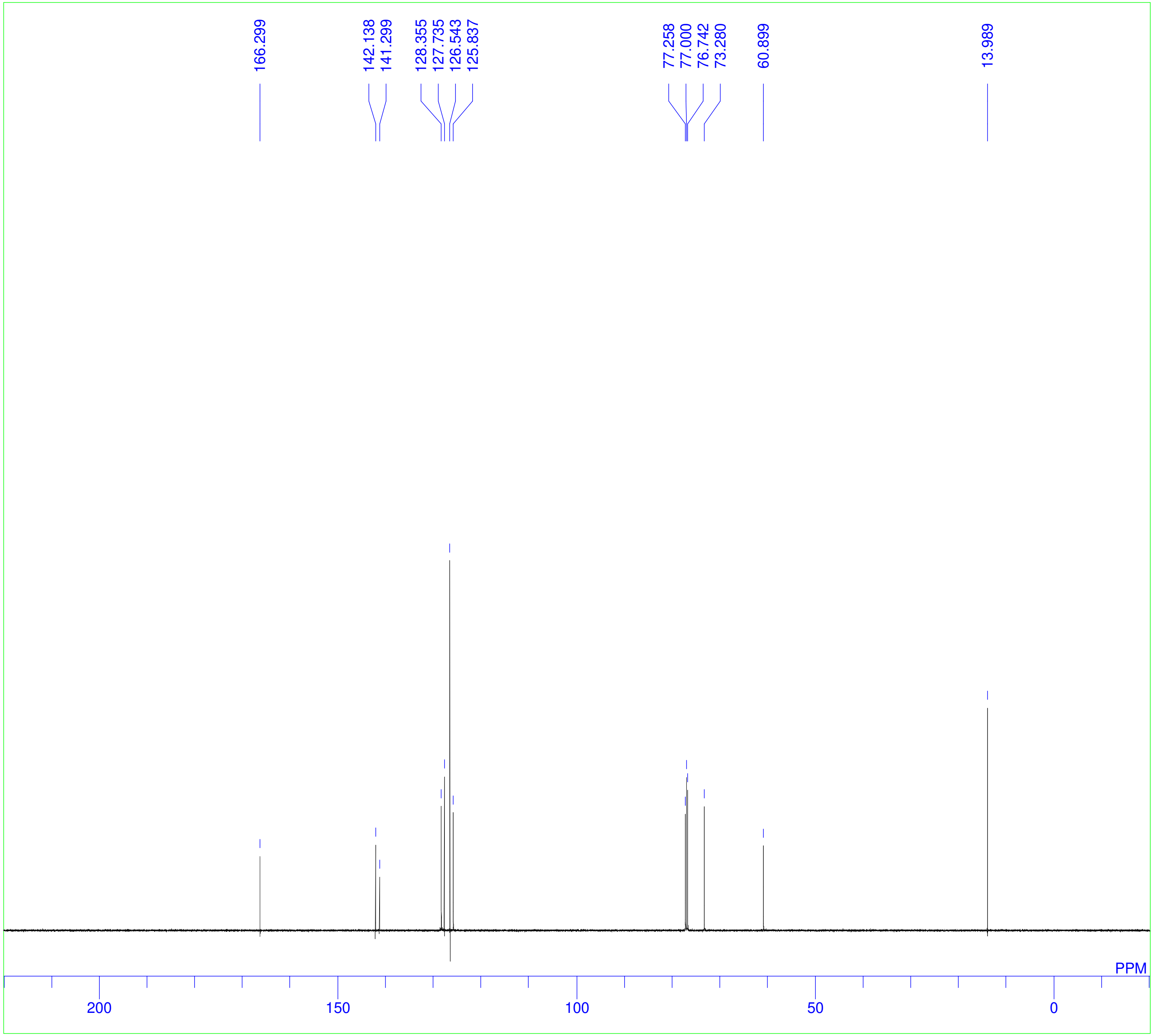

DFILE	S7_13C.als
COMNT	
DATIM	2023-01-11 20:49:24
OBNUC	13C
EXMOD	carbon.jxp
OBFRQ	125.77 MHz
OBSET	7.87 KHz
OBFIN	4.21 Hz
POINT	26214
FREQU	31446.54 Hz
SCANS	1024
ACQTM	0.8336 sec
PD	2.0000 sec
PW1	3.87 usec
IRNUC	1H
CTEMP	22.4 c
SLVNT	CDCL3
EXREF	77.00 ppm
BF	0.12 Hz
RGAIN	24

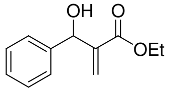

S7

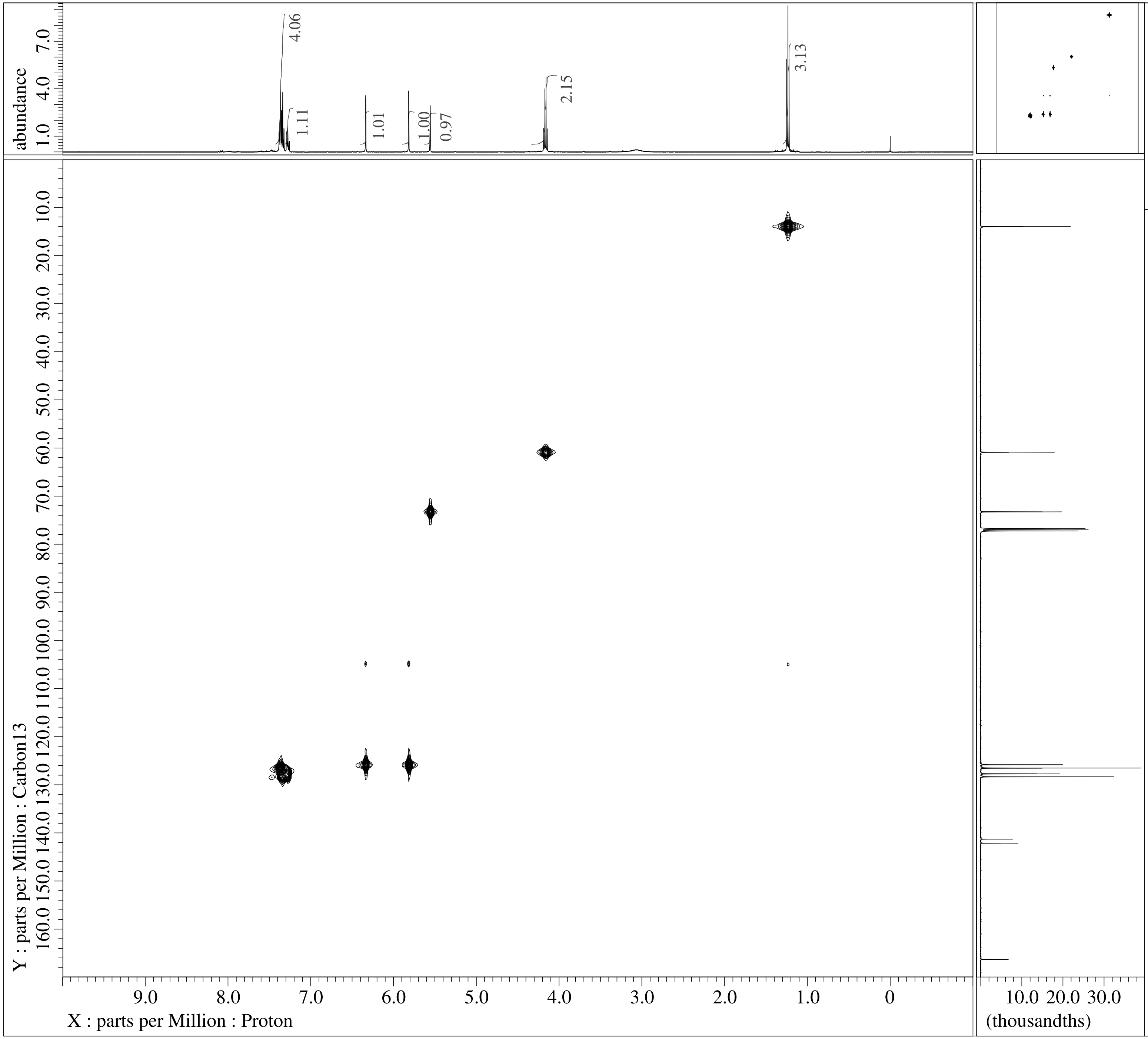


```
---- PROCESSING PARAMETERS ----
sinbell4( -60, 160 )
zerofill( 1 )
fft( 1, TRUE, TRUE )
ppm
[transpose]
sinbell4( -60, 160 )
zerofill( 2 )
fft( 1, TRUE, TRUE )
ppm
abs
```

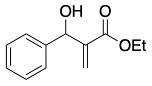

S7

```
Filename            = HT-1233-TM_HMQC_HMQC-1-
Author              = delta
Experiment          = hmqc.jxp
Sample_Id           = HT-1233-TM_HMQC
Solvent             = CHLOROFORM-D
Creation_Time       =  6-JAN-2024 10:03:44
Revision_Time       =  9-APR-2024 22:29:56
Current_Time        =  9-APR-2024 22:30:54

Comment             = gradient enhanced HMQC
Data_Format         = 2D REAL REAL
Dim_Size            = 819, 512
Dim_Title           = Proton Carbon13
Dim_Units           = [ppm] [ppm]
Dimensions          = X Y
Site                = JNM-ECA500II
Spectrometer        = DELTA2_NMR

Field_Strength      = 11.7473579[T] (500[MHz]
X_Acq_Duration      = 0.12582912[s]
X_Domain            = 1H
X_Freq              = 500.15991521[MHz]
X_Offset            = 4.99484665[ppm]
X_Points            = 1024
X_Prescans          = 4
X_Resolution        = 7.94728597[Hz]
X_Sweep             = 8.13802083[kHz]
X_Sweep_Clipped     = 6.51041667[kHz]
Y_Domain            = 13C
Y_Freq              = 125.76529768[MHz]
Y_Offset            = 85[ppm]
Y_Points            = 256
Y_Prescans          = 0
Y_Resolution        = 83.60980308[Hz]
Y_Sweep             = 21.40410959[kHz]
Tri_Domain          = Proton
Tri_Freq            = 500.15991521[MHz]
Tri_Offset          = 5.0[ppm]
Clipped             = FALSE
Scans               = 8
Total_Scans         = 2048

Relaxation_Delay    = 1.5[s]
Recvr_Gain          = 50
Temp_Get            = 22.4[dC]
X_Acq_Time          = 0.12582912[s]
X_Atn               = 3.2[dB]
X_Gamma             = 42576375
X_Pulse             = 7.59[us]
```

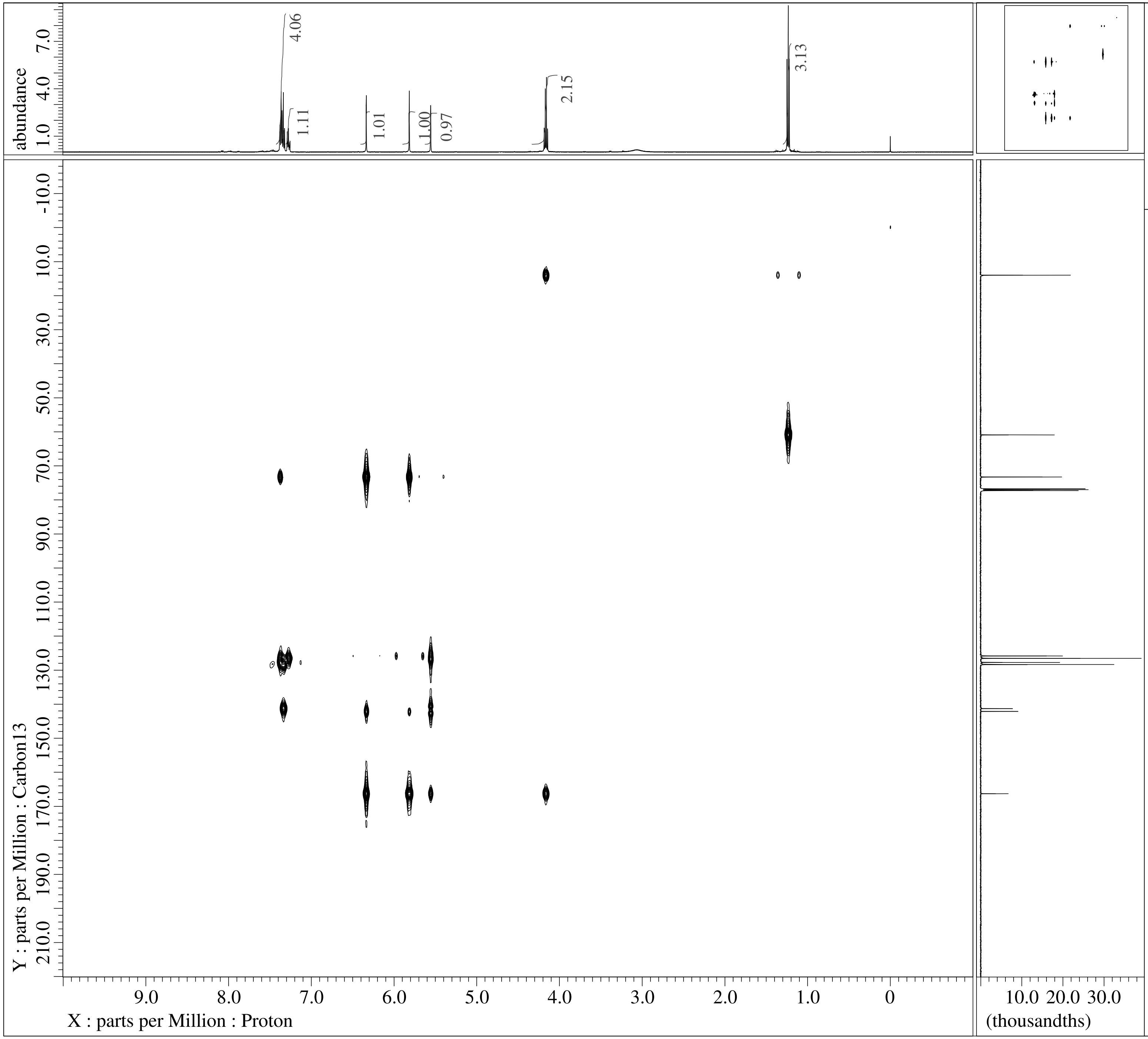


```
---- PROCESSING PARAMETERS ----
gauss( 5.0[Hz], 0.0[s] )
sinbell_auto
zerofill( 1 )
fft( 1, TRUE, TRUE )
ppm
[transpose]
sinbell4( -60, 160 )
trapezoid( 0[%], 5[%], 80[%], 100[%] )
zerofill( 2 )
fft( 1, TRUE, TRUE )
```

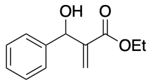

S7

```
Filename            = HT-1233-TM_HMBC_HMBC-1-
Author              = delta
Experiment          = hmbc.jxp
Sample_Id           = HT-1233-TM_HMBC
Solvent             = CHLOROFORM-D
Creation_Time       =  6-JAN-2024 12:06:10
Revision_Time       =  9-APR-2024 22:27:18
Current_Time        =  9-APR-2024 22:28:25

Comment             = gradient enhanced HMBC
Data_Format         = 2D REAL REAL
Dim_Size            = 1638, 512
Dim_Title           = Proton Carbon13
Dim_Units           = [ppm] [ppm]
Dimensions          = X Y
Site                = JNM-ECA500II
Spectrometer        = DELTA2_NMR

Field_Strength      = 11.7473579[T] (500[MHz]
X_Acq_Duration      = 0.21823488[s]
X_Domain            = 1H
X_Freq              = 500.15991521[MHz]
X_Offset            = 5.0[ppm]
X_Points            = 2048
X_Prescans          = 4
X_Resolution        = 4.58221894[Hz]
X_Sweep             = 9.38438438[kHz]
X_Sweep_Clipped     = 7.50750751[kHz]
Y_Domain            = 13C
Y_Freq              = 125.76529768[MHz]
Y_Offset            = 100[ppm]
Y_Points            = 256
Y_Prescans          = 0
Y_Resolution        = 122.99275819[Hz]
Y_Sweep             = 31.4861461[kHz]
Tri_Domain          = Proton
Tri_Freq            = 500.15991521[MHz]
Tri_Offset          = 5.0[ppm]
Clipped             = FALSE
Scans               = 8
Total_Scans         = 2048

Relaxation_Delay    = 1.5[s]
Recvr_Gain          = 50
Temp_Get            = 22.8[dC]
X_Acq_Time          = 0.21823488[s]
X_Atn               = 3.2[dB]
X_Gamma             = 42576375
X_Pulse             = 7.59[us]
```

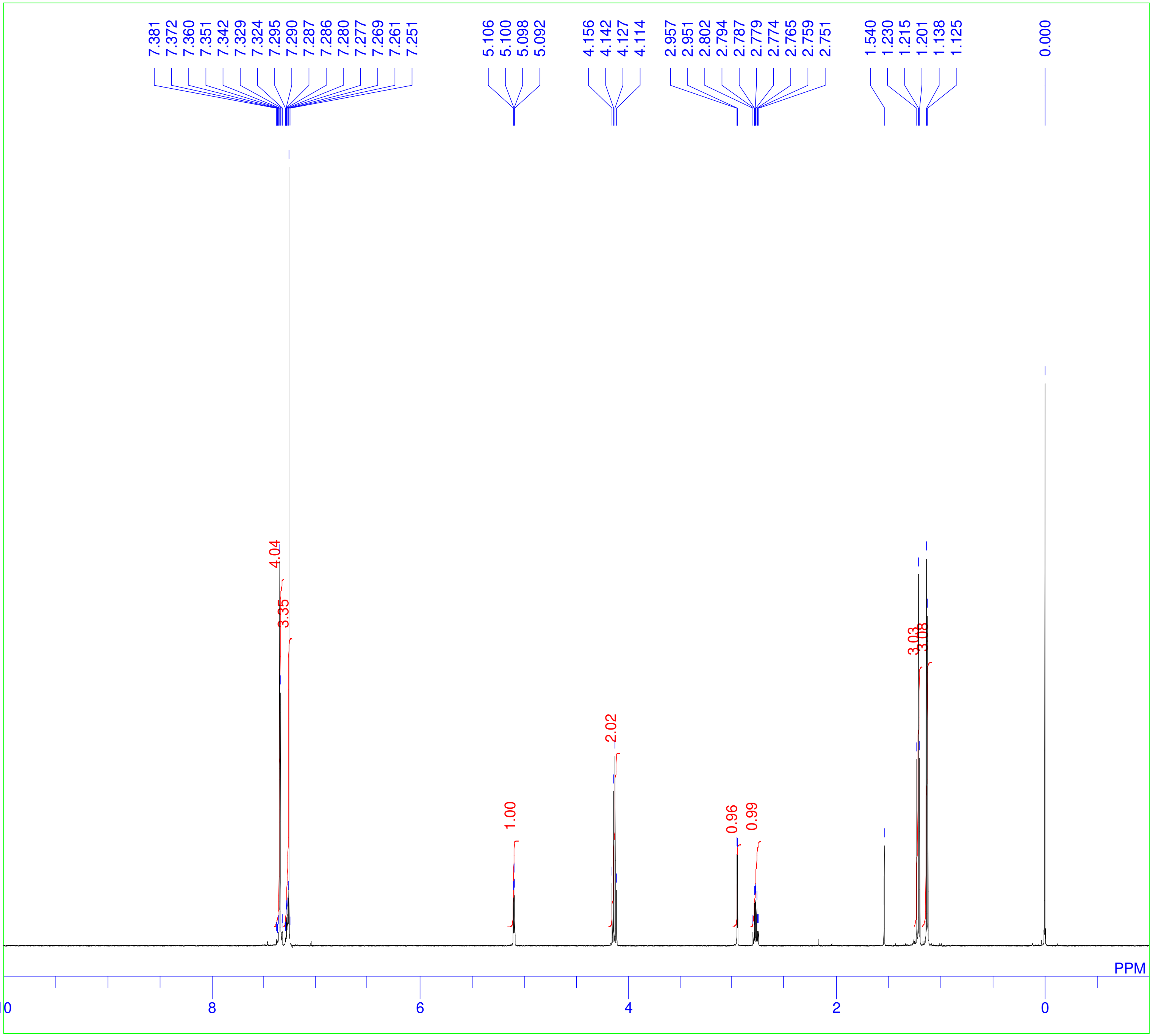

DFILE	syn-S8_1H.als
COMNT	
DATIM	2023-01-13 18:55:05
OBNUC	1H
EXMOD	proton.jxp
OBFRQ	500.16 MHz
OBSET	2.41 KHz
OBFIN	6.01 Hz
POINT	13107
FREQU	7507.51 Hz
SCANS	8
ACQTM	1.7459 sec
PD	5.0000 sec
PW1	3.84 usec
IRNUC	1H
CTEMP	22.9 c
SLVNT	CDCL3
EXREF	0.00 ppm
BF	0.12 Hz
RGAIN	48

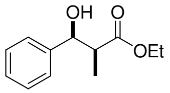

***syn*-S8**

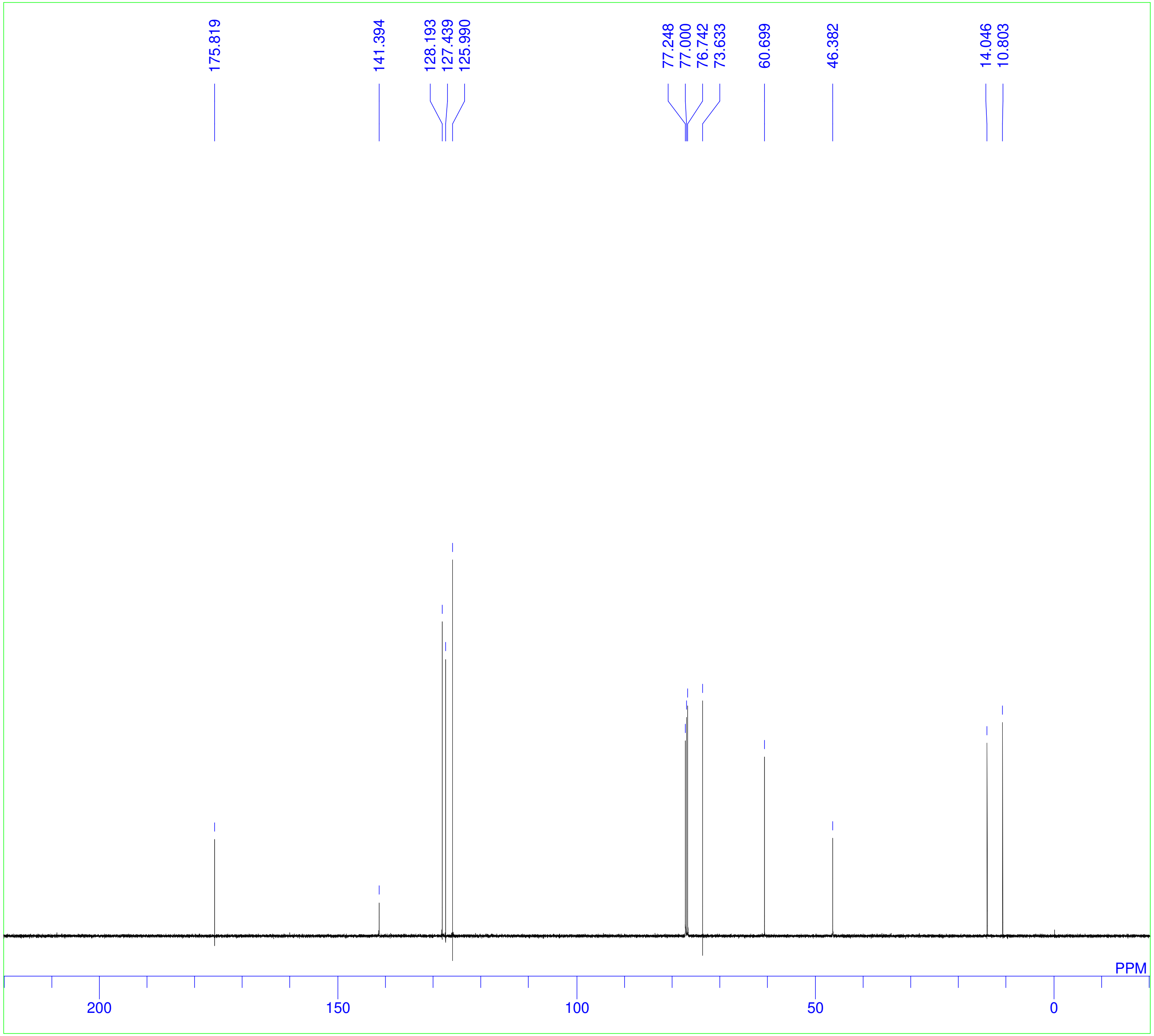

DFILE	syn-S8_13C.als
COMNT	
DATIM	2023-01-13 20:05:24
OBNUC	13C
EXMOD	carbon.jxp
OBFRQ	125.77 MHz
OBSET	7.87 KHz
OBFIN	4.21 Hz
POINT	26214
FREQU	31446.54 Hz
SCANS	1024
ACQTM	0.8336 sec
PD	2.0000 sec
PW1	3.87 usec
IRNUC	1H
CTEMP	23.0 c
SLVNT	CDCL3
EXREF	77.00 ppm
BF	0.12 Hz
RGAIN	28

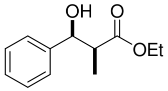

***syn*-S8**

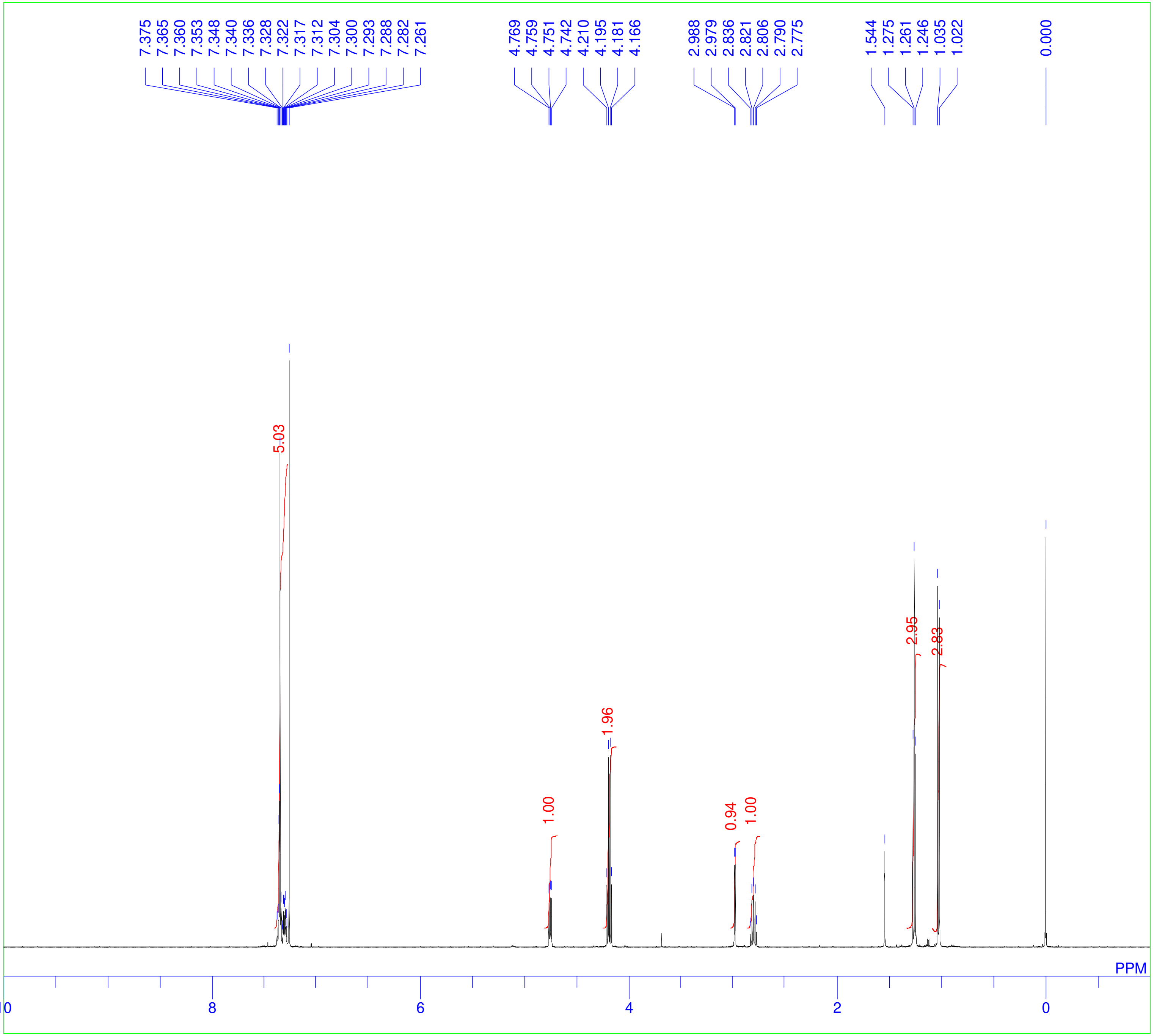

DFILE anti-S8_1H.als
COMNT
DATIM 2023-01-13 19:01:40
OBNUC 1H
EXMOD proton.jxp
OBFRQ 500.16 MHz
OBSET 2.41 KHz
OBFIN 6.01 Hz
POINT 13107
FREQU 7507.51 Hz
SCANS 8
ACQTM 1.7459 sec
PD 5.0000 sec
PW1 3.84 usec
IRNUC 1H
CTEMP 23.0 c
SLVNT CDCL3
EXREF 0.00 ppm
BF 0.12 Hz
RGAIN 46

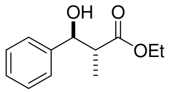

***anti*-S8**

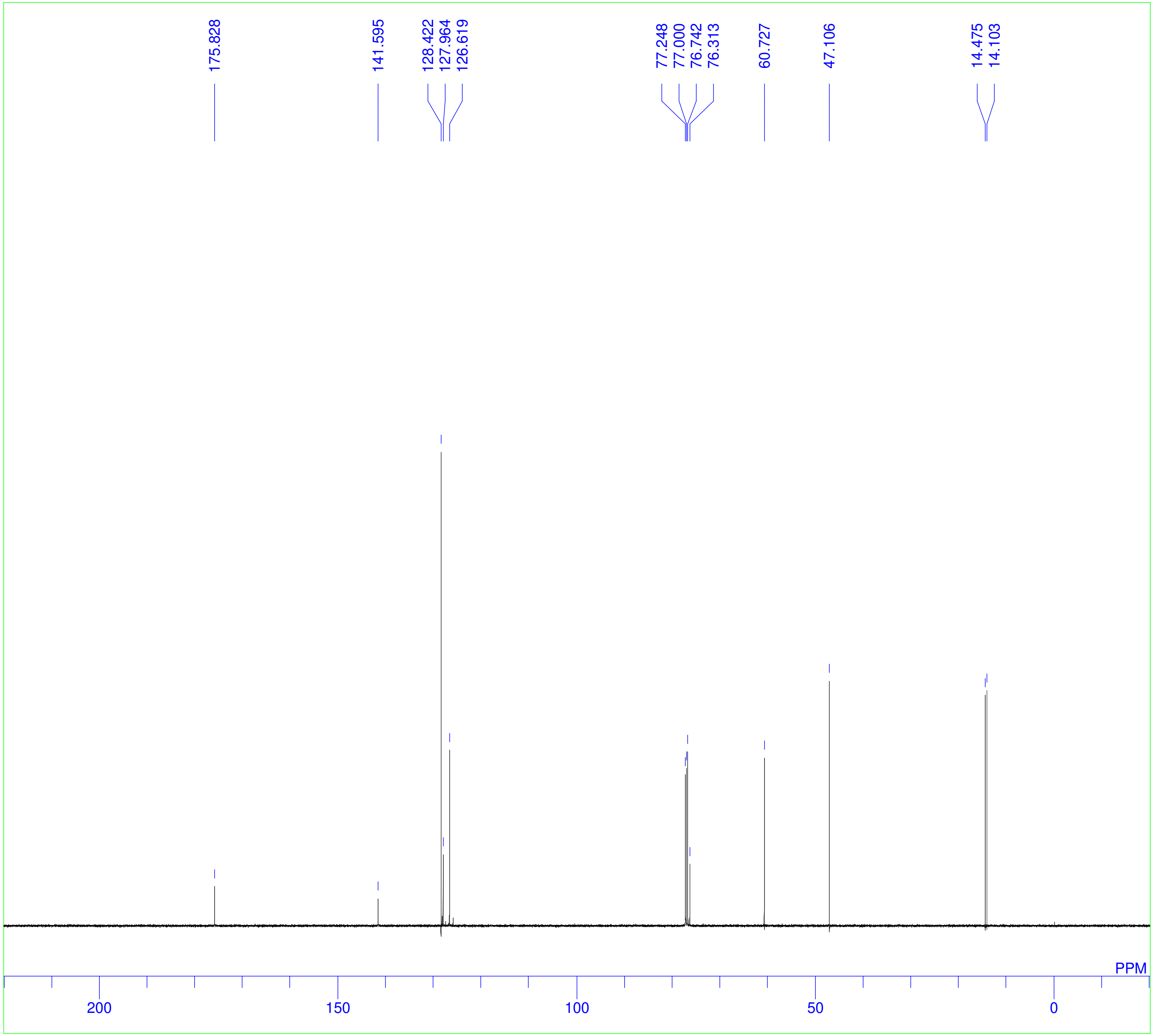

DFILE	anti-S8_13C.als
COMNT	
DATIM	2023-01-13 21:02:25
OBNUC	13C
EXMOD	carbon.jxp
OBFRQ	125.77 MHz
OBSET	7.87 KHz
OBFIN	4.21 Hz
POINT	26214
FREQU	31446.54 Hz
SCANS	1024
ACQTM	0.8336 sec
PD	2.0000 sec
PW1	3.87 usec
IRNUC	1H
CTEMP	23.0 c
SLVNT	CDCL3
EXREF	77.00 ppm
BF	0.12 Hz
RGAIN	28

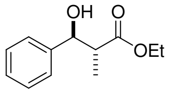

***anti*-S8**

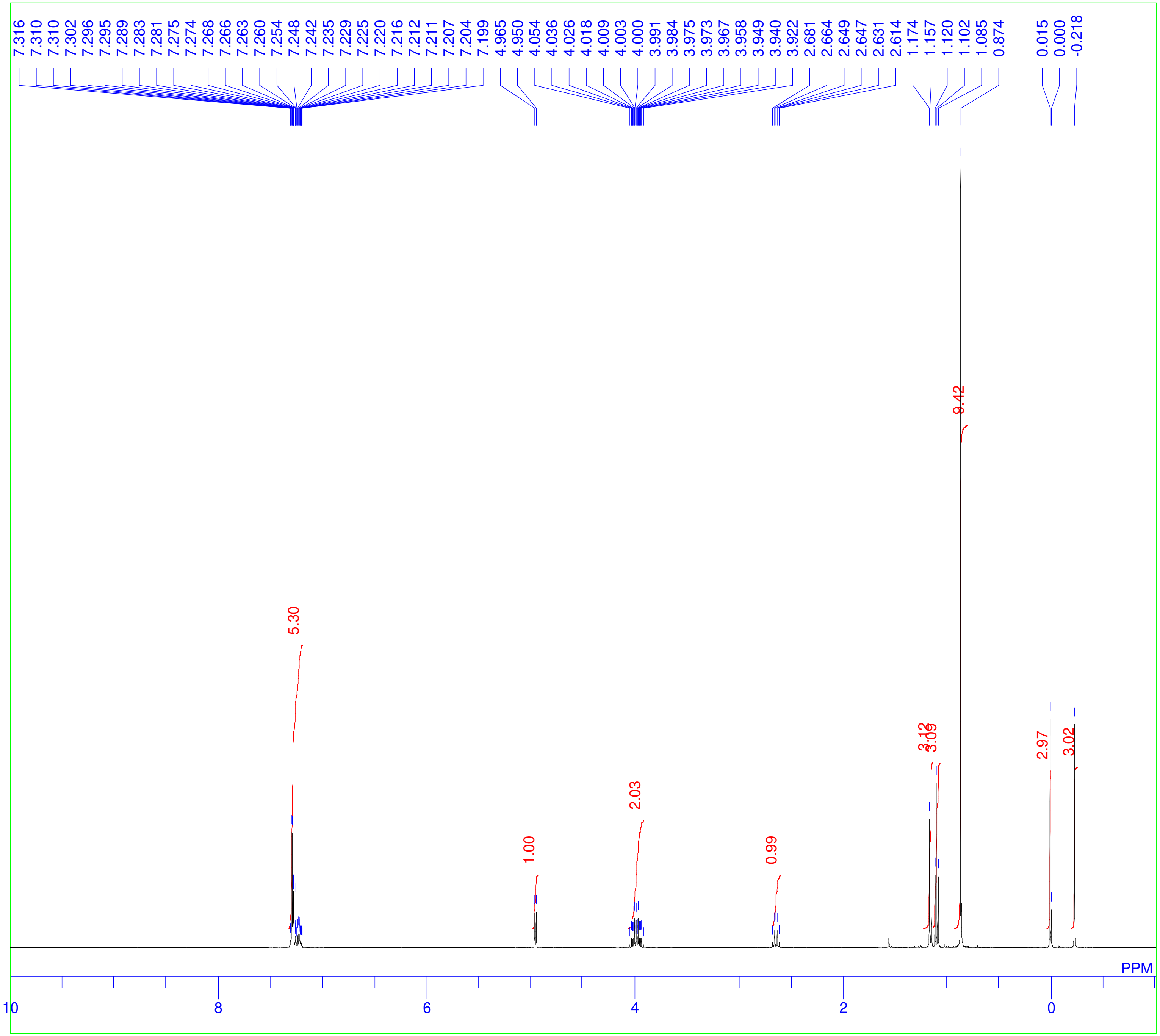

DFILE syn-S9_1H.als
COMNT
DATIM
OBNUC 1H
EXMOD
OBFRQ 400.18 MHz
OBSET 2.47 KHz
OBFIN 1.30 Hz
POINT 32768
FREQU 8223.68 Hz
SCANS 8
ACQTM 0.0000 sec
PD 0.0000 sec
PW1 10.00 usec
IRNUC
CTEMP 0.0 c
SLVNT
EXREF 7.26 ppm
BF 0.30 Hz
RGAIN 0

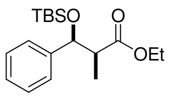

***syn*-S9**

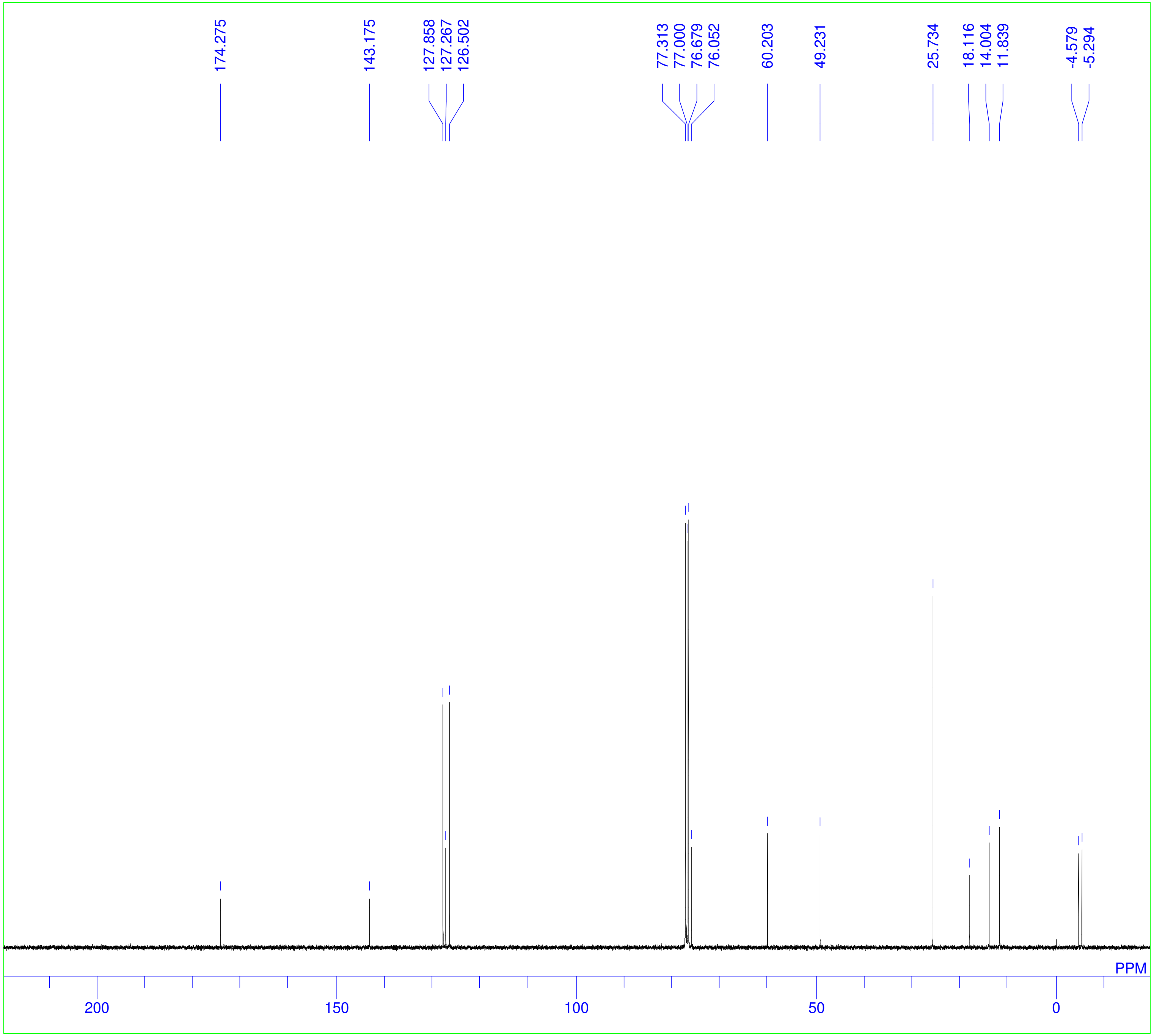

DFILE syn-S9_13C.als
COMNT
DATIM
OBNUC 13C
EXMOD
OBFRQ 100.63 MHz
OBSET 5.40 KHz
OBFIN 3.60 Hz
POINT 32768
FREQU 24038.46 Hz
SCANS 8
ACQTM 0.0000 sec
PD 0.0000 sec
PW1 10.00 usec
IRNUC
CTEMP 0.0 c
SLVNT
EXREF 77.00 ppm
BF 0.30 Hz
RGAIN 0

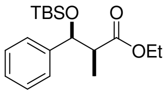

***syn*-S9**

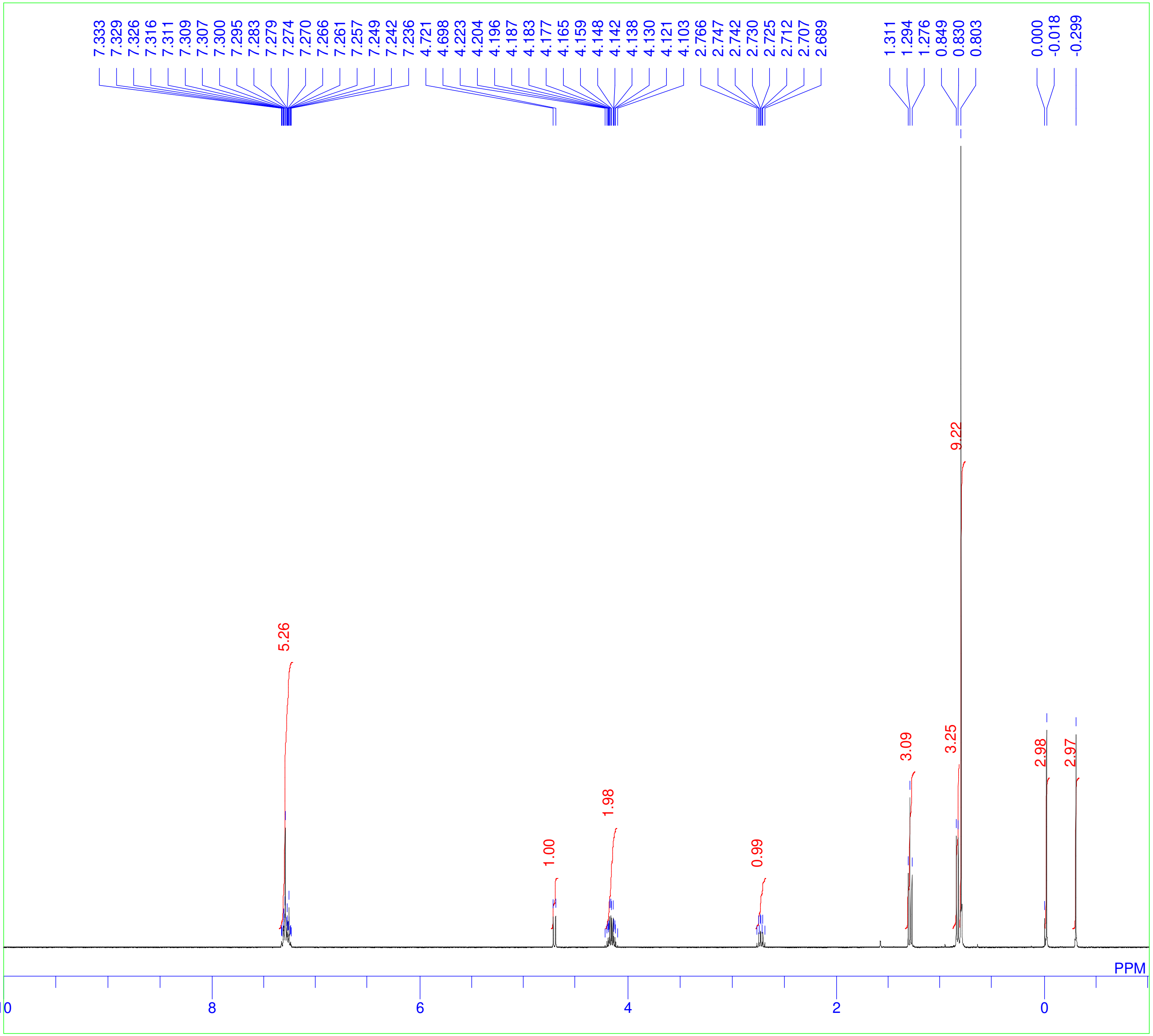
7.333
7.329
7.326
7.316
7.311
7.309
7.307
7.300
7.295
7.283
7.279
7.274
7.270
7.266
7.261
7.257
7.249
7.242
7.236
4.721
4.698
4.223
4.204
4.196
4.187
4.183
4.177
4.165
4.159
4.148
4.142
4.138
4.130
4.121
4.103
2.766
2.747
2.742
2.730
2.725
2.712
2.707
2.689
1.311
1.294
1.276
0.849
0.830
0.803
0.000
-0.018
-0.299
5.26
1.00
1.98
0.99
3.09
3.25
9.22
2.98
2.97
PPM
10
8
6
4
2
0
DFILE anti-S9_1H.als
COMNT
DATIM
OBNUC 1H
EXMOD
OBFRQ 400.18 MHz
OBSET 2.47 KHz
OBFIN 1.30 Hz
POINT 32768
FREQU 8223.68 Hz
SCANS 8
ACQTM 0.0000 sec
PD 0.0000 sec
PW1 10.00 usec
IRNUC
CTEMP 0.0 c
SLVNT
EXREF 0.00 ppm
BF 0.30 Hz
RGAIN 0

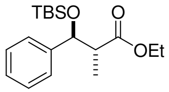
TBSO
O
OEt

***anti*-S9**

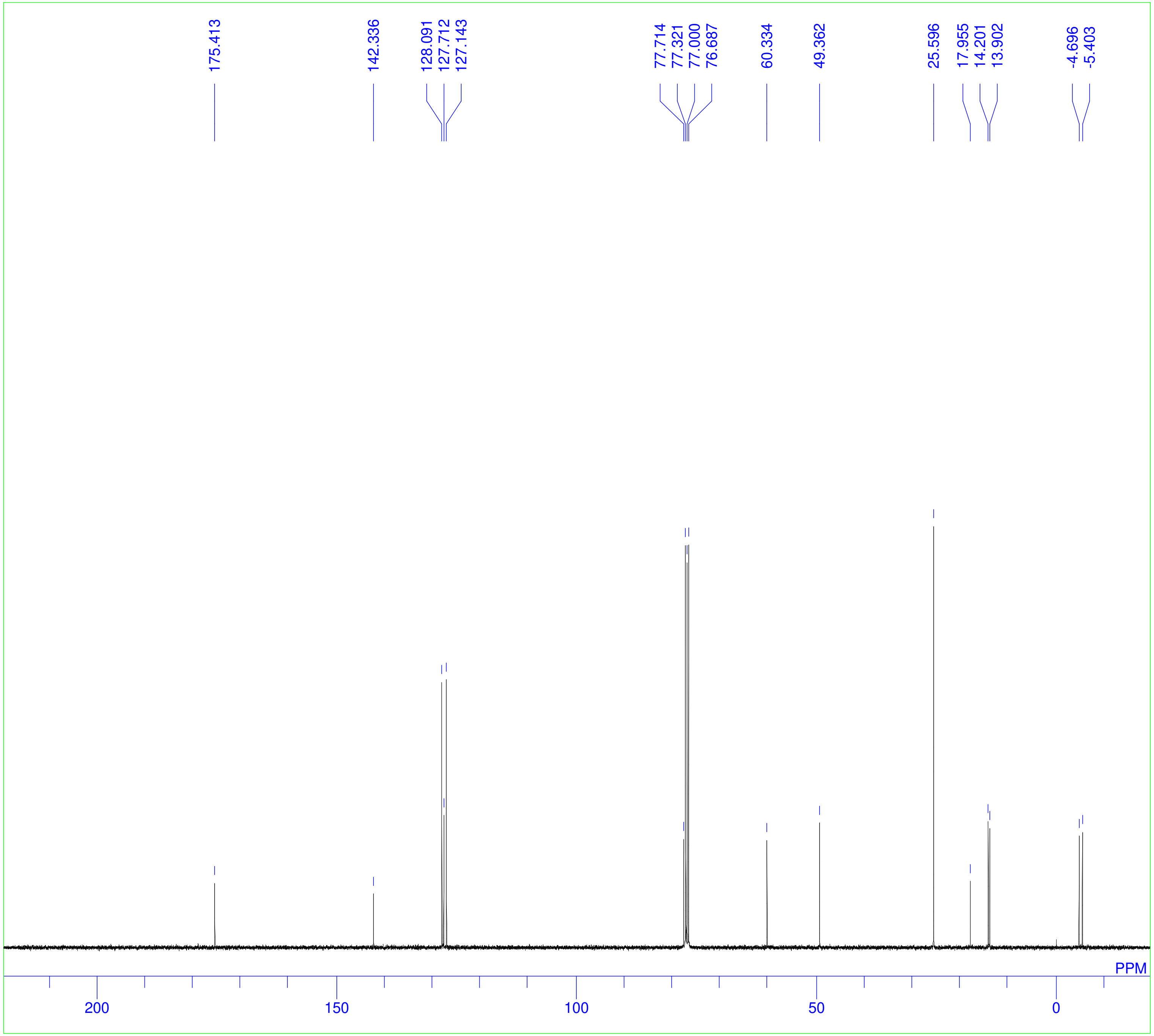

DFILE	anti-S9_13C.als
COMNT	
DATIM	
OBNUC	13C
EXMOD	
OBFRQ	100.63 MHz
OBSET	5.40 KHz
OBFIN	3.60 Hz
POINT	32768
FREQU	24038.46 Hz
SCANS	8
ACQTM	0.0000 sec
PD	0.0000 sec
PW1	10.00 usec
IRNUC	
CTEMP	0.0 c
SLVNT	
EXREF	77.00 ppm
BF	0.30 Hz
RGAIN	0

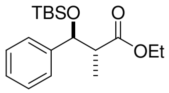

***anti*-S9**

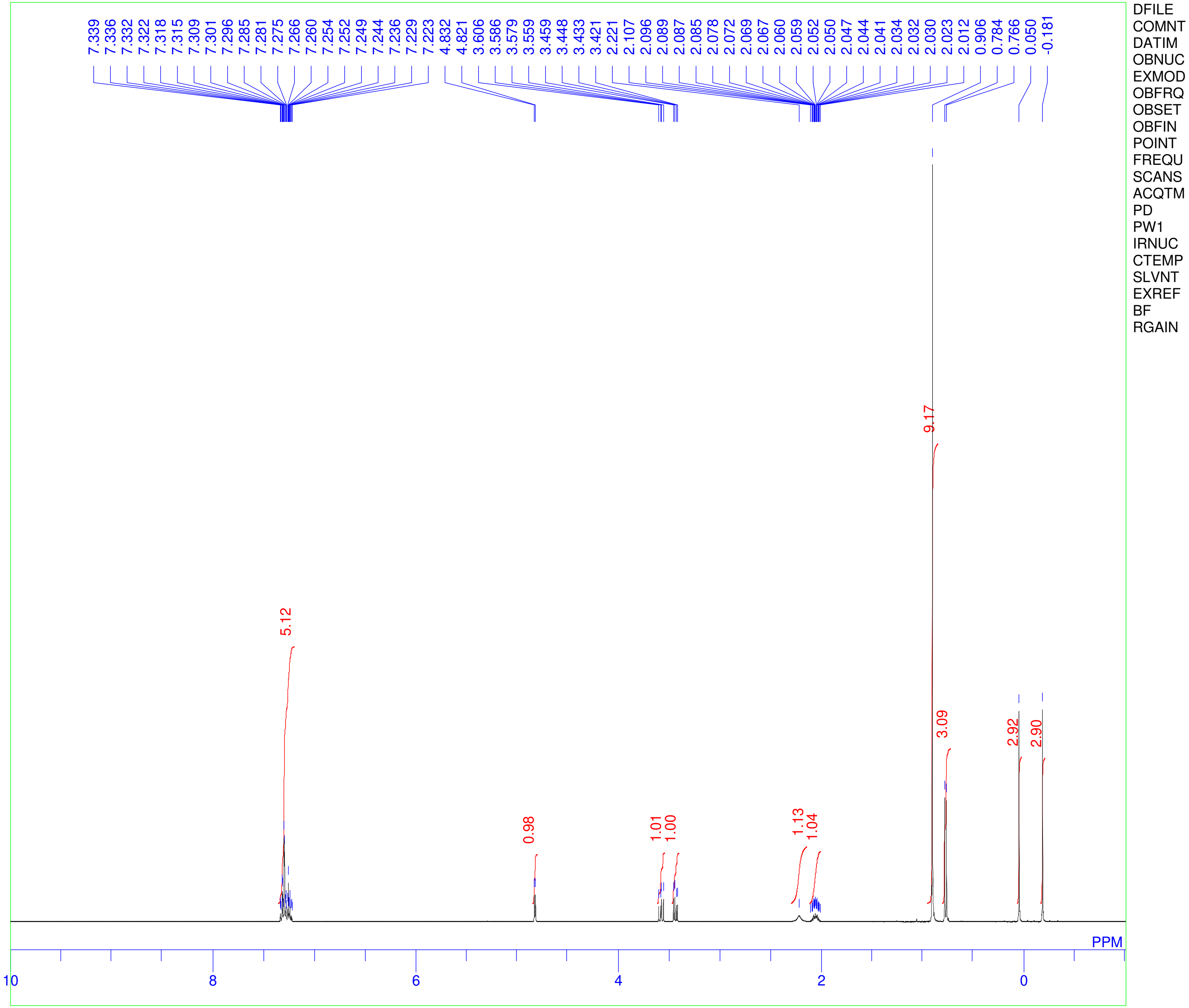

DFILE syn-S10_1H.als
COMNT
DATIM
OBNUC 1H
EXMOD
OBFRQ 400.18 MHz
OBSET 2.47 KHz
OBFIN 1.30 Hz
POINT 32768
FREQU 8223.68 Hz
SCANS 8
ACQTM 0.0000 sec
PD 0.0000 sec
PW1 10.00 usec
IRNUC
CTEMP 0.0 c
SLVNT
EXREF 7.26 ppm
BF 0.30 Hz
RGAIN 0

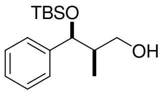

***syn*-S10**

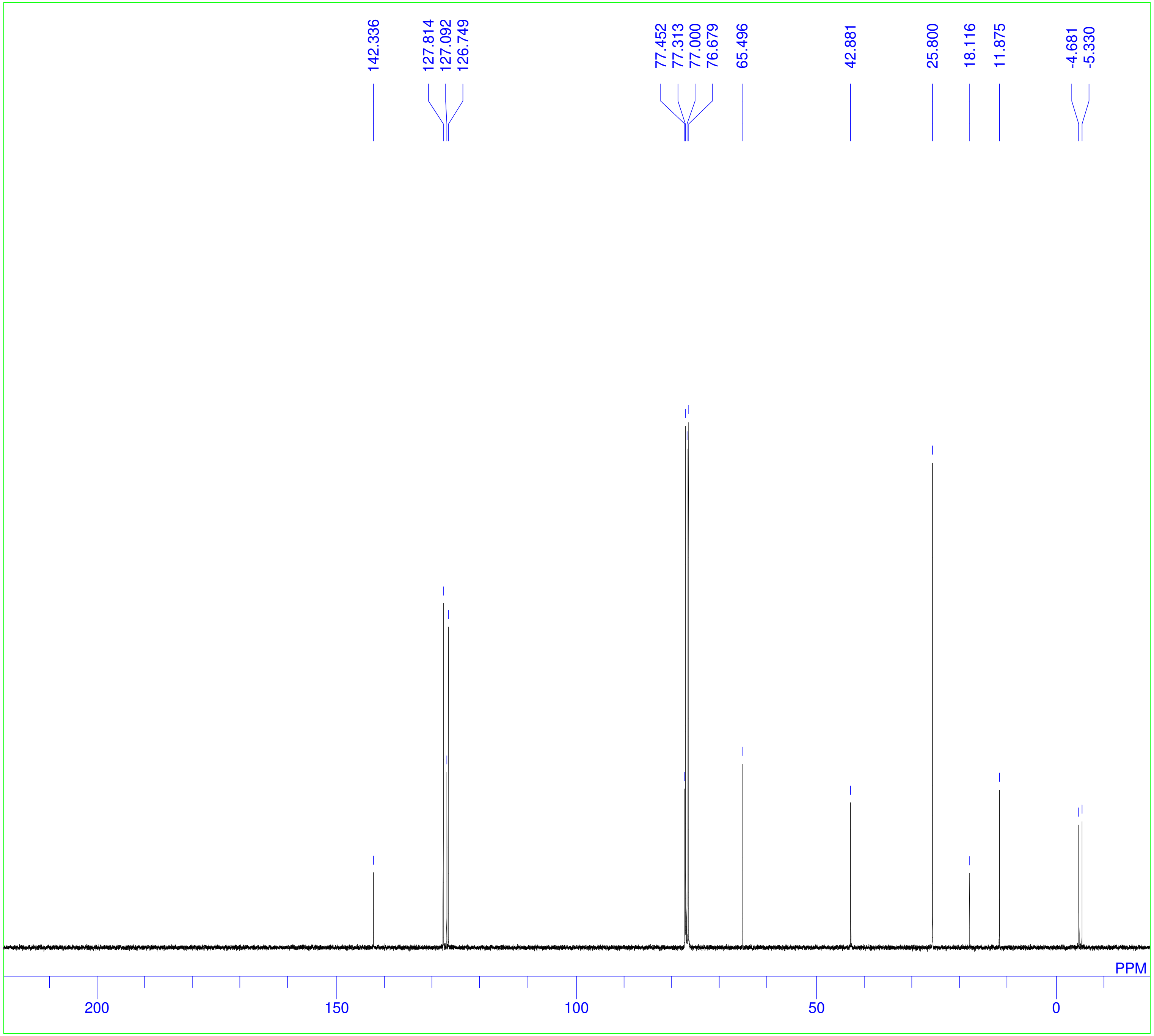

DFILE	syn-S10_13C.als
COMNT	
DATIM	
OBNUC	13C
EXMOD	
OBFRQ	100.63 MHz
OBSET	5.40 KHz
OBFIN	3.60 Hz
POINT	32768
FREQU	24038.46 Hz
SCANS	8
ACQTM	0.0000 sec
PD	0.0000 sec
PW1	10.00 usec
IRNUC	
CTEMP	0.0 c
SLVNT	
EXREF	77.00 ppm
BF	0.30 Hz
RGAIN	0

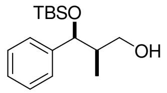

***syn*-S10**

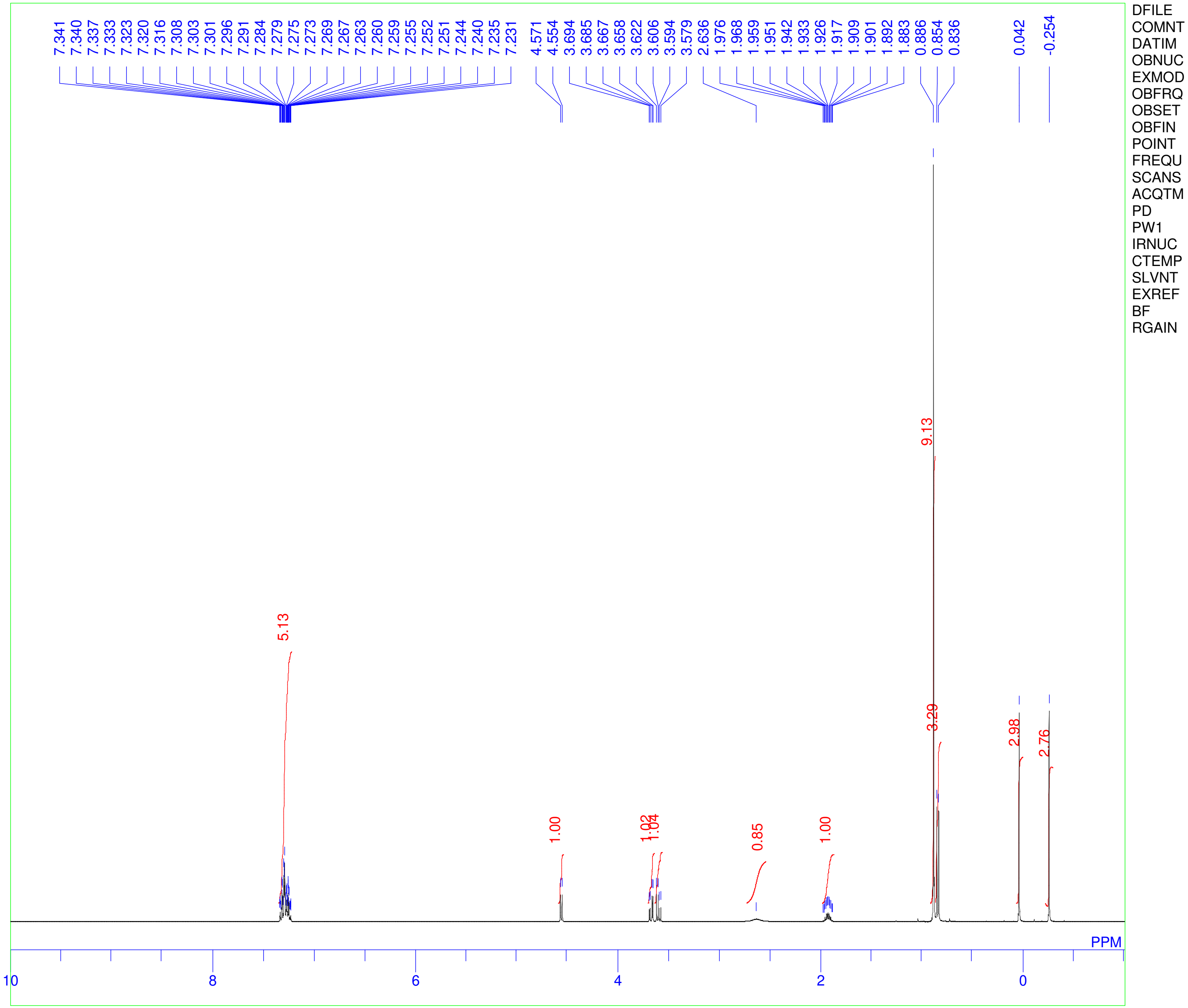

DFILE anti-S10_1H.als
COMNT
DATIM
OBNUC 1H
EXMOD
OBFRQ 400.18 MHz
OBSET 2.47 KHz
OBFIN 1.30 Hz
POINT 32768
FREQU 8223.68 Hz
SCANS 8
ACQTM 0.0000 sec
PD 0.0000 sec
PW1 10.00 usec
IRNUC
CTEMP 0.0 c
SLVNT
EXREF 7.26 ppm
BF 0.30 Hz
RGAIN 0

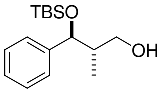

***anti*-S10**

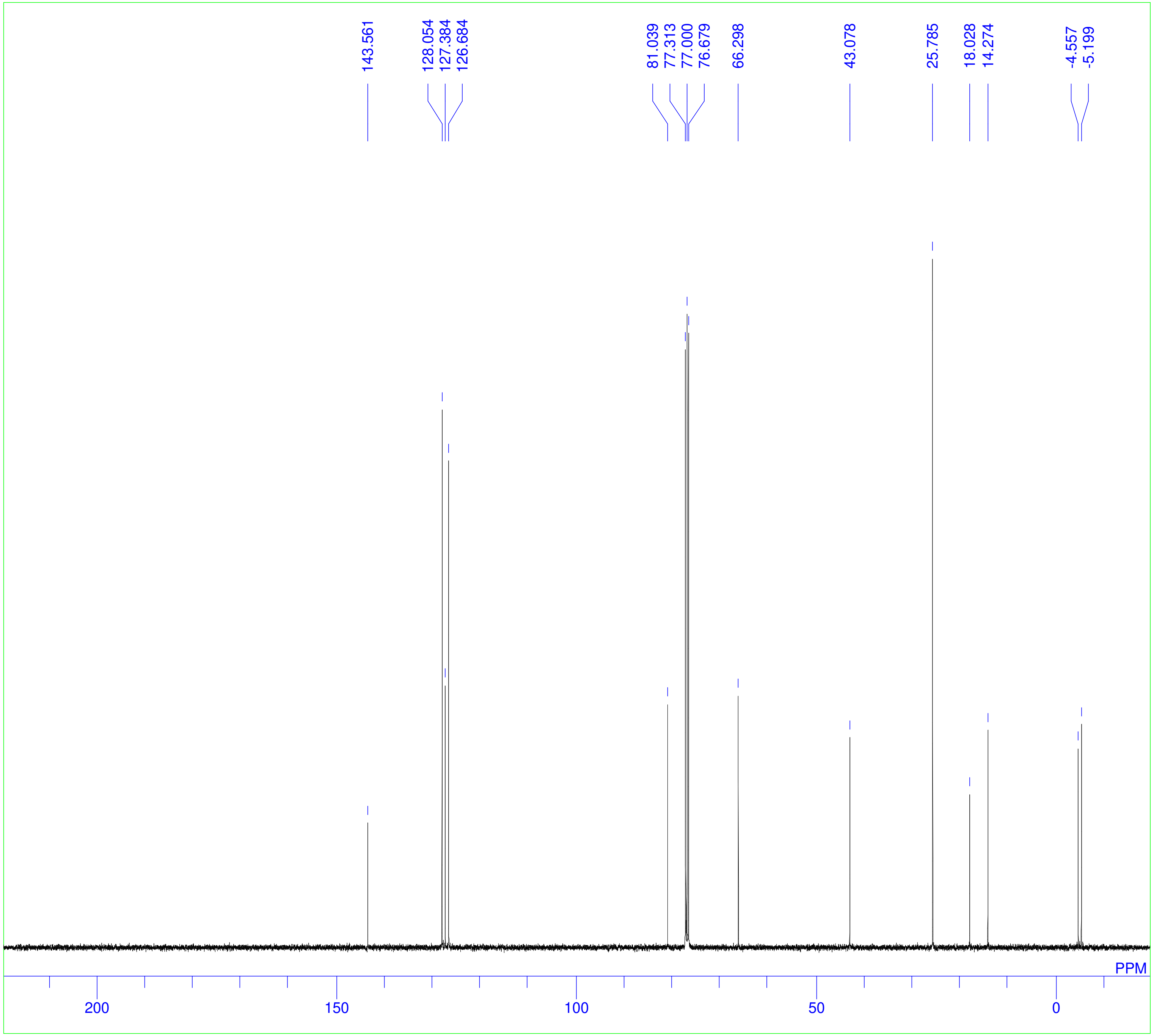

DFILE anti-S10_13C.als
COMNT
DATIM
OBNUC 13C
EXMOD
OBFRQ 100.63 MHz
OBSET 5.40 KHz
OBFIN 3.60 Hz
POINT 32768
FREQU 24038.46 Hz
SCANS 8
ACQTM 0.0000 sec
PD 0.0000 sec
PW1 10.00 usec
IRNUC
CTEMP 0.0 c
SLVNT
EXREF 77.00 ppm
BF 0.30 Hz
RGAIN 0

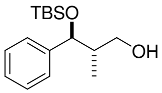

***anti*-S10**

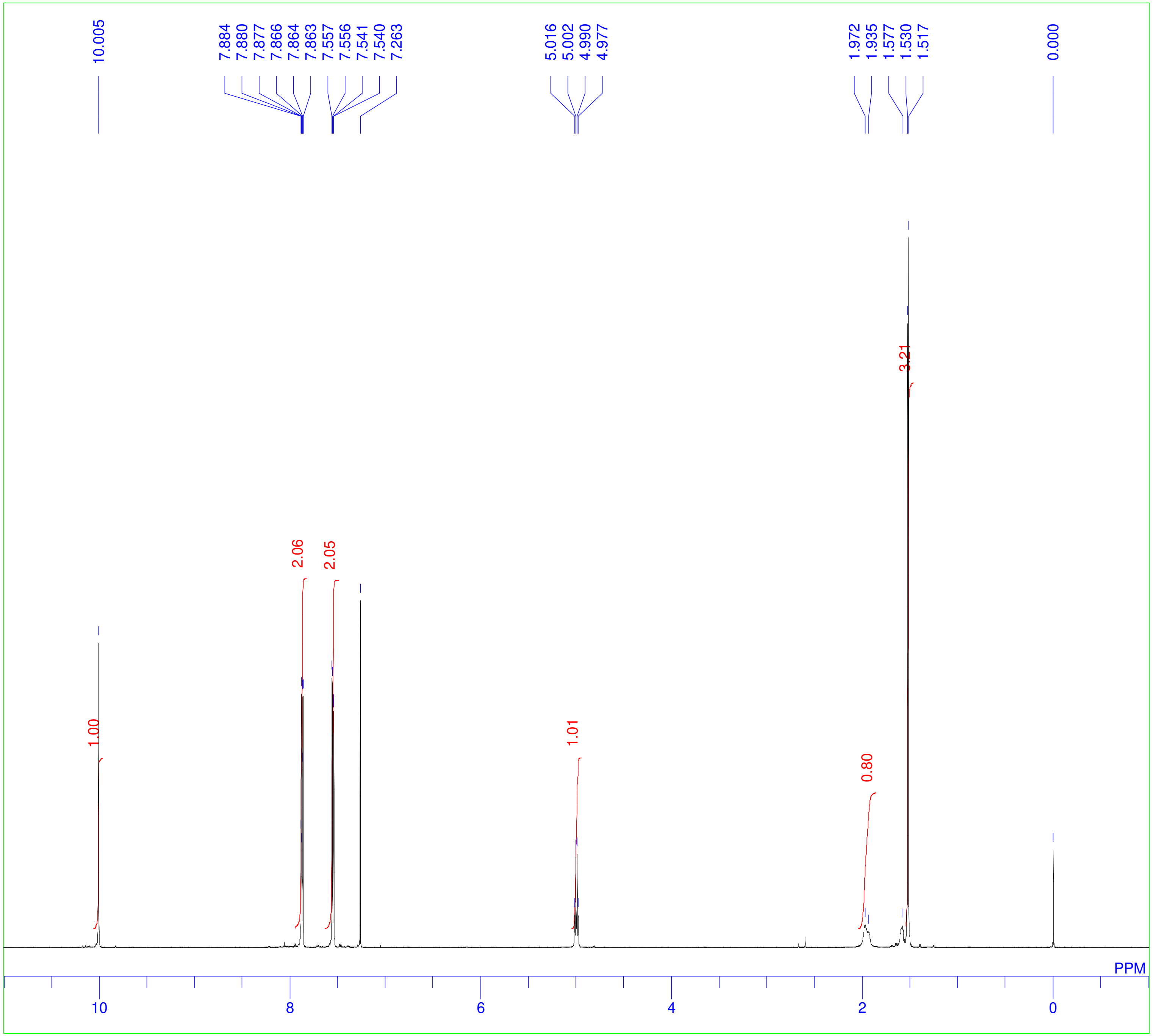

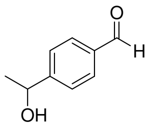

S11

DFILE	S11_1H.als
COMNT	
DATIM	2023-04-07 07:30:45
OBNUC	1H
EXMOD	proton.jxp
OBFRQ	500.16 MHz
OBSET	2.41 KHz
OBFIN	6.01 Hz
POINT	13107
FREQU	7507.51 Hz
SCANS	8
ACQTM	1.7459 sec
PD	5.0000 sec
PW1	3.84 usec
IRNUC	1H
CTEMP	23.6 c
SLVNT	CDCL3
EXREF	0.00 ppm
BF	0.30 Hz
RGAIN	44

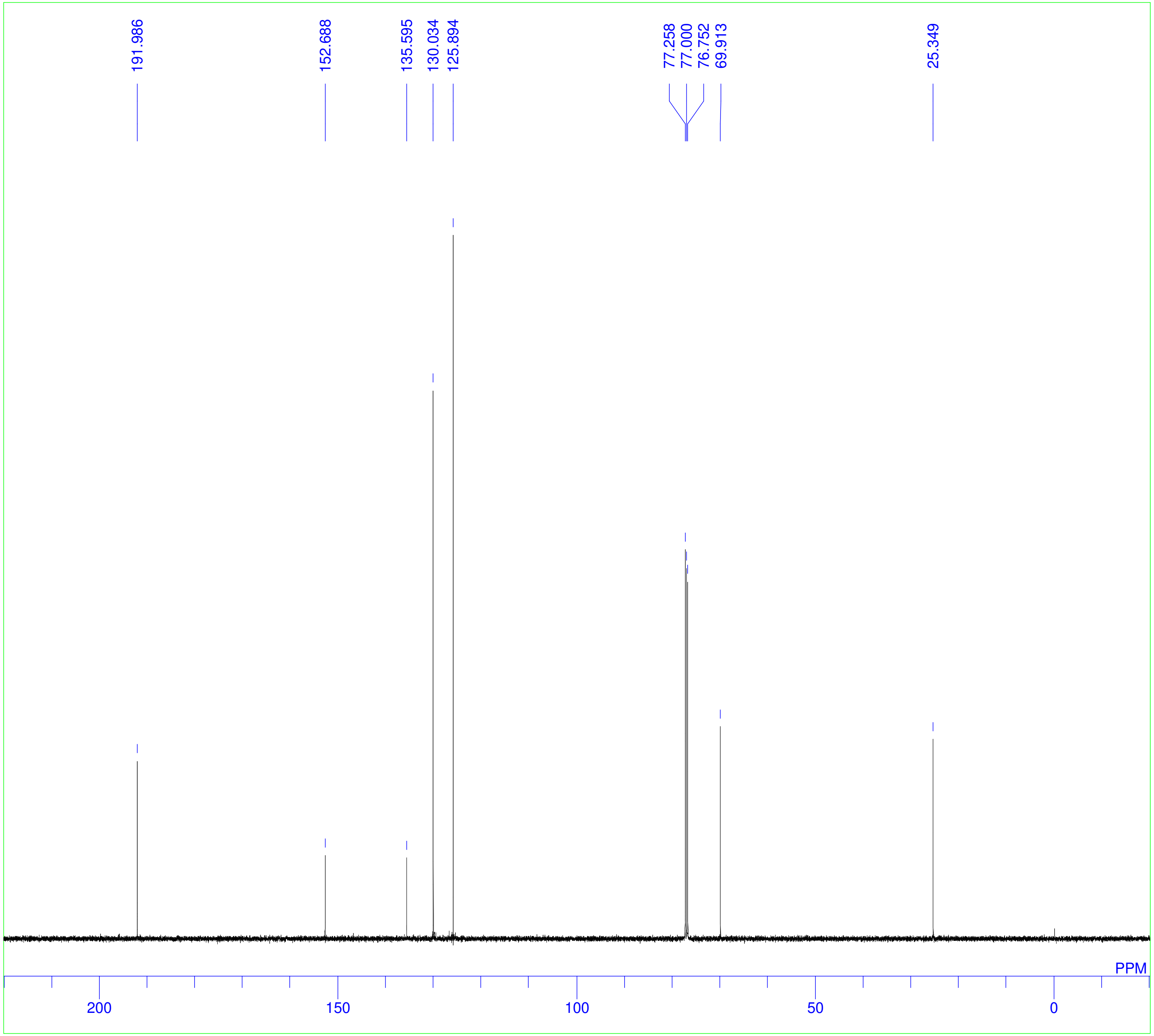

DFILE S11_13C.als
COMNT
DATIM 2023-04-20 18:30:49
OBNUC 13C
EXMOD carbon.jxp
OBFRQ 125.77 MHz
OBSET 7.87 KHz
OBFIN 4.21 Hz
POINT 26214
FREQU 31446.54 Hz
SCANS 1024
ACQTM 0.8336 sec
PD 2.0000 sec
PW1 3.87 usec
IRNUC 1H
CTEMP 24.1 c
SLVNT CDCL3
EXREF 77.00 ppm
BF 0.30 Hz
RGAIN 30

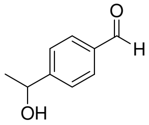

S11

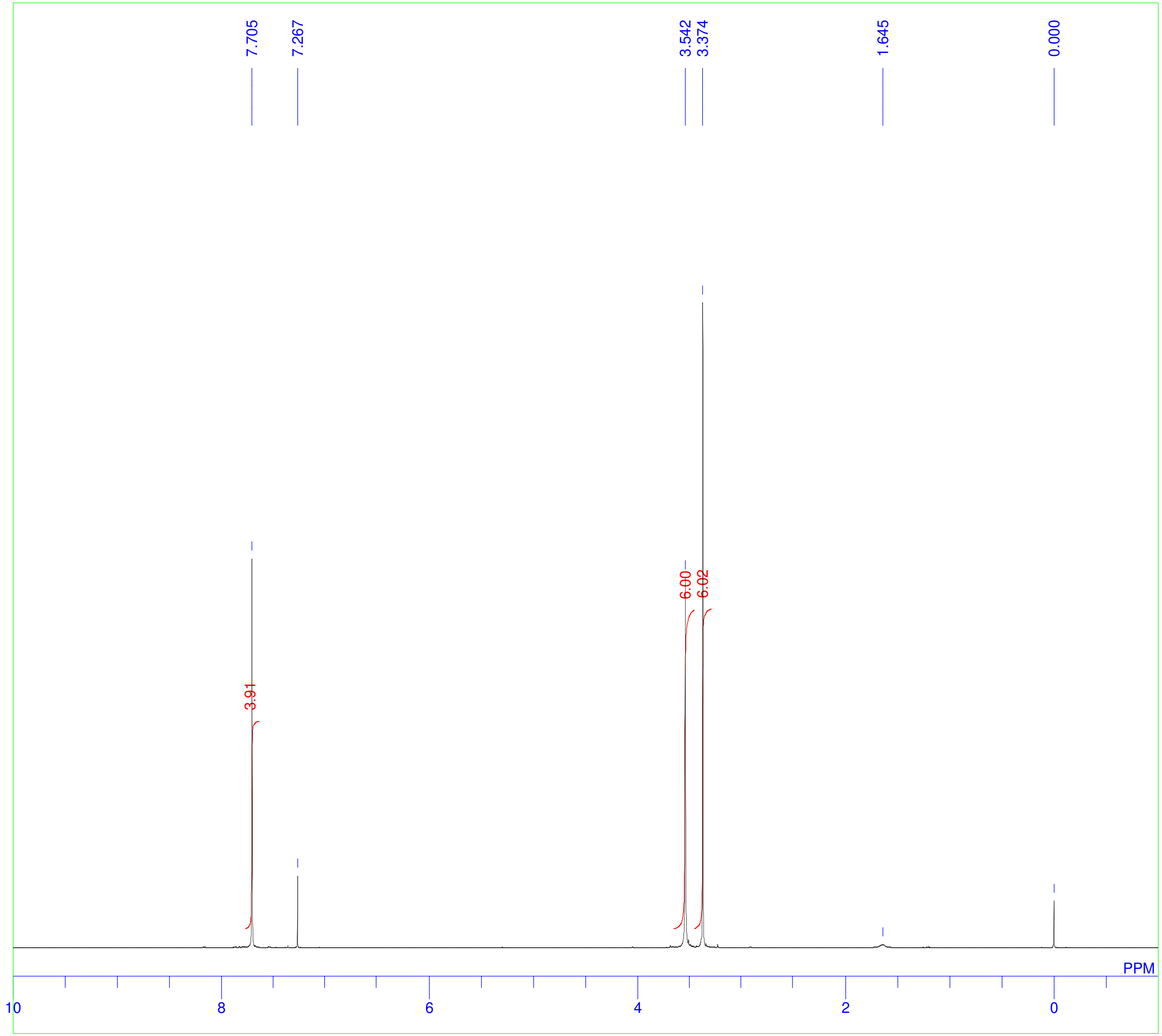

DFILE S12_1H.als
COMNT
DATIM 2023-05-25 21:50:07
OBNUC 1H
EXMOD proton.jxp
OBFRQ 500.16 MHz
OBSET 2.41 KHz
OBFIN 6.01 Hz
POINT 13107
FREQU 7507.51 Hz
SCANS 8
ACQTM 1.7459 sec
PD 5.0000 sec
PW1 3.84 usec
IRNUC 1H
CTEMP 24.4 c
SLVNT CDCL3
EXREF 0.00 ppm
BF 0.30 Hz
RGAIN 38

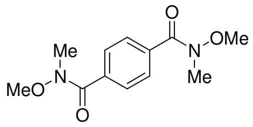

S12

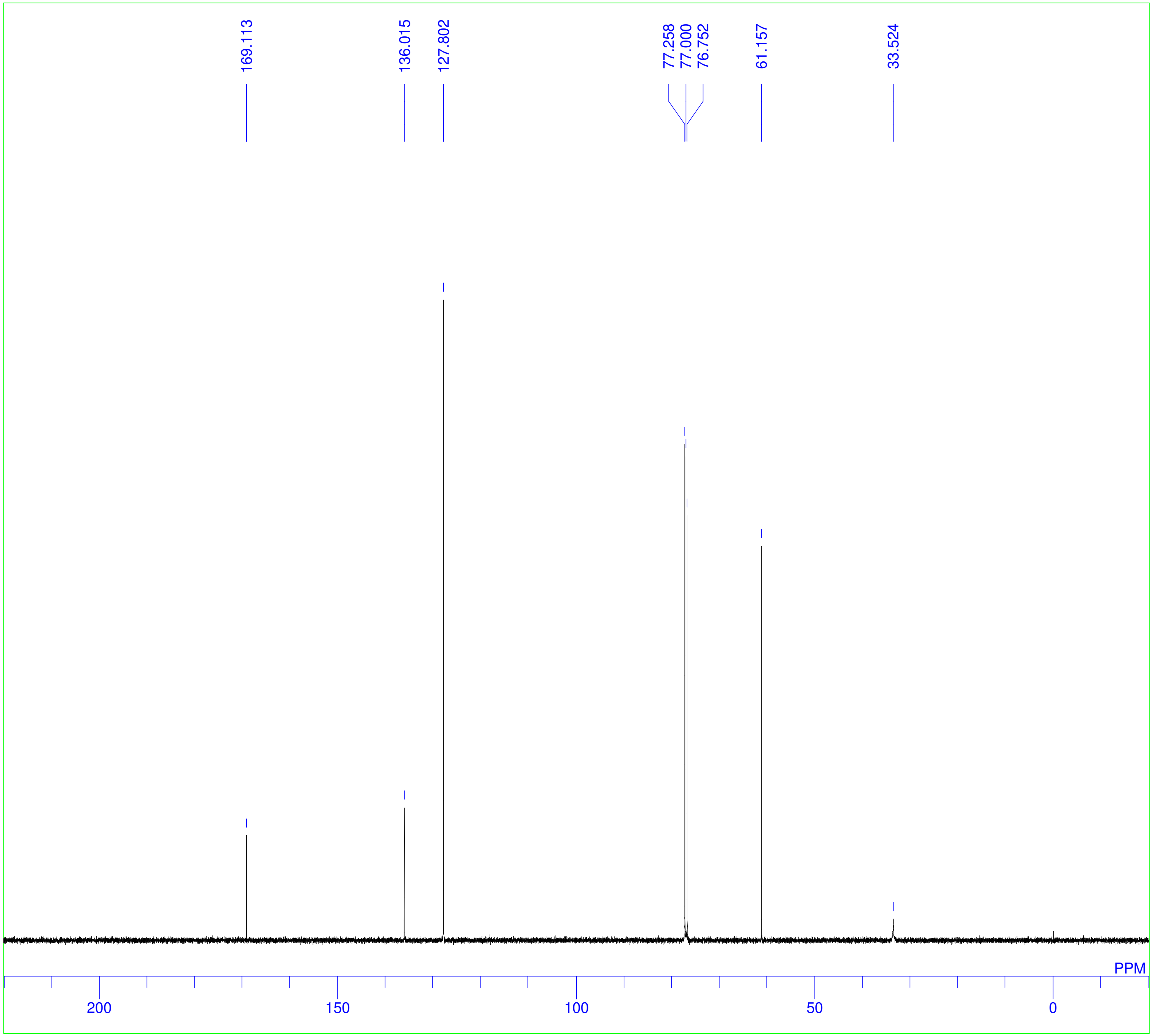

DFILE S12_13C.als
COMNT
DATIM 2023-05-27 15:01:12
OBNUC 13C
EXMOD carbon.jxp
OBFRQ 125.77 MHz
OBSET 7.87 KHz
OBFIN 4.21 Hz
POINT 26214
FREQU 31446.54 Hz
SCANS 1024
ACQTM 0.8336 sec
PD 2.0000 sec
PW1 3.87 usec
IRNUC 1H
CTEMP 24.2 c
SLVNT CDCL3
EXREF 77.00 ppm
BF 0.30 Hz
RGAIN 30

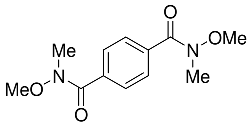

S12
